# Spatial heterogeneity in the temperature–hand, foot, and mouth disease association among children: A multicounty time-series study in western China

**DOI:** 10.1371/journal.pntd.0013801

**Published:** 2026-01-02

**Authors:** Jie Sun, Guanghai Yao, Jing Gu, Hui Tang, Yueqian Wu, Yikun Chang, Jinwei Chen, Wangjian Zhang, Zhicheng Du, Yuantao Hao

**Affiliations:** 1 Department of Medical Statistics, School of Public Health & Center for Health Information Research & Sun Yat-sen Global Health Institute, Sun Yat-sen University, Guangzhou, Guangdong, China; 2 Institute for the Control of Infectious Diseases, Guizhou Center for Disease Control and Prevention, Guiyang, Guizhou, China; 3 Guangzhou Joint Research Center for Disease Surveillance and Risk Assessment, Sun Yat-sen University & Guangzhou Center for Disease Control and Prevention, Guangzhou, Guangdong, China; 4 Peking University Center for Public Health and Epidemic Preparedness & Response, Peking University, Beijing, China; 5 Department of Epidemiology & Biostatistics, School of Public Health, Peking University, Beijing, China; 6 Key Laboratory of Epidemiology of Major Diseases (Peking University), Ministry of Education, Peking University, Beijing, China; Public Health Agency of Canada, CANADA

## Abstract

While meteorological and socioeconomic factors are well-documented modifiers of spatial heterogeneity in temperature–hand, foot, and mouth disease (HFMD) associations, substantial unexplained heterogeneity remains. This study investigates underexplored environmental modifiers—including extreme temperature events (heat waves and cold spells), air pollution, and normalized difference vegetation index (NDVI)—by analyzing 484,928 HFMD cases among children under six years old in western China (2013–2019) using distributed lag nonlinear models and meta-regression. We found that cold spells (accounted for 3.84% of the spatial heterogeneity attributable above the baseline level), PM_2.5_ (3.06%), heat waves (2.72%), PM_10_ (2.08%), NDVI (1.57%) and O_3_ (0.78%) were statistically significant modifiers of spatial heterogeneity in the temperature-HFMD associations. Further analysis of PM_2.5_ components identified nitrate (1.78%) and ammonium (1.58%) as additional modifiers. Among these, cold spells, heat waves, PM_2.5_, and NDVI were the primary contributors. Specifically, the relative risk (*RR*) of HFMD at the 95th temperature percentile reached 3.17 (95% CI: 2.39–4.19) in frequent heat waves regions and 3.05 (2.35–3.95) in high-PM_2.5_ areas. Conversely, regions with low cold-spell frequency also exhibited increased temperature-related risk (*RR* = 3.13, 2.40–4.07) at the same temperature, as did low NDVI regions (*RR* = 2.16, 1.79–2.59). Spatial cluster analysis further revealed that the central and northeastern regions exhibited higher temperature-related HFMD risks compared to the southwestern region. These pronounced spatial modification effects challenge the generalizability of single-site study and highlight the importance of region-specific public health strategies that integrate early warning systems for extreme temperatures, air pollution mitigation, and locally adapted greening interventions.

## Author summary

Hand, foot, and mouth disease (HFMD) is a common illness among young children, especially in Asia. Although temperature is known to influence the risk of HFMD, the relationship between temperature and disease varies from place to place. In this study, we analyzed over 480,000 HFMD cases in children under six years old across 88 counties in western China. We explored how regional environmental conditions—such as heat waves, cold spells, air pollution, and green space—might explain these differences. We found that areas with more heat waves or higher levels of air pollution had greater HFMD risk during hot weather. In contrast, regions with more green space or frequent cold spells showed weaker temperature effects. We also used clustering methods to group regions with similar environmental patterns and found that some areas were more vulnerable to temperature-related HFMD than others. These results show that local environmental conditions play an important role in shaping disease risk and suggest that region-specific prevention strategies may help reduce HFMD in children.

## Introduction

Hand, foot and mouth disease (HFMD) is a highly prevalent infectious disease of childhood caused by enteroviruses and characterized primarily by fever, oral ulcers and vesicular rashes [1]. Although most cases are mild and self-limiting, severe complications such as encephalitis and cardiopulmonary failure can occur [2]. Since the late 1990s, recurrent large-scale outbreaks have caused a significant burden of disease throughout the Asia-Pacific region [3,4]. Across eight high-burden Asia-Pacific countries and regions, including mainland China, HFMD contributes to an estimated 96,900 age-adjusted DALYs annually [5]. In mainland China, HFMD has been notifiable since 2008, with over 22 million cases and 3,500 deaths reported between 2008 and 2019 [6,7]. In the absence of targeted antiviral treatment [8], strengthening prevention remains a public health priority. Understanding the factors that influence HFMD transmission is therefore essential for effective control strategies.

Ambient temperature is an important environmental determinant of HFMD risk and has been extensively studied. A systematic review and meta-analysis of 11 studies from five countries confirmed a significant positive association between temperature and HFMD incidence [3]. However, substantial variation in effect estimates was observed both between and within countries, as reported in previous studies [9–11]. These inconsistencies arise from heterogeneity in population demographics, exposure definitions, statistical models, and study designs. In addition to methodological factors, recent research has increasingly focused on the role of meteorological and socioeconomic variables in shaping the spatial heterogeneity of temperature-HFMD associations [11–13]. For example, a multicenter study of 143 Chinese cities found that factors such as temperature, relative humidity, atmospheric pressure, sunshine duration, and GDP per capita explained only 0.01% to 2.0% of the observed spatial variation [11]. Although these factors partially account for the heterogeneity, they remain insufficient to fully explain regional disparities, suggesting the presence of additional, unmeasured environmental modifiers.

Regional temperature significantly influences the spatial heterogeneity of HFMD-environment relationships [11–13]. Extreme temperature events (ETEs), including heat waves and cold spells, have gained increasing attention due to their severe health impacts. While the exact biological mechanisms remain unclear, extreme temperatures disrupt thermoregulation and trigger systemic stress responses such as cardiovascular dysfunction, fluid imbalance, and inflammation [14]. These responses can severely compromise human health. Due to immature thermoregulatory and immune systems, children are more susceptible to extreme temperatures, increasing their risk of HFMD [15,16]. Moreover, while extreme temperatures may accelerate viral inactivation, they can also promote indoor crowding, which increases the risk of HFMD transmission [17]. However, the role of extreme temperatures in driving HFMD spatial heterogeneity remains unclear. In addition to temperature, air pollution may modify the temperature–HFMD relationship. Accumulating evidence links pollutants such as PM_2.5_, PM_10_, and O_3_ to increased HFMD risk [18,19]. The small particle size and large surface area of PM_2.5_ and PM_10_ enable them to absorb toxic substances and penetrate the alveoli, inducing oxidative stress and inflammatory responses that impair antiviral defenses [20,21]. Additionally, these particles may act as carriers for enteric viruses, increasing airborne exposure and facilitating HFMD transmission [22,23]. O_3_, a potent oxidizing agent, can damage respiratory mucosal barriers and impair host immunity, further increasing susceptibility to HFMD, although it can also inactivate viruses [24,25]. Despite these insights, the influence of air pollution on HFMD spatial heterogeneity has received limited attention. Given its ubiquity and potential synergistic effects with temperature, air pollution may contribute to regional variations in the temperature-HFMD relationship.

In addition, traditional economic factors, such as population density and GDP per capita, have been shown to be closely associated with HFMD [11,12]. Nighttime light index and Normalized Difference Vegetation Index (NDVI) are modern indicators that correlate strongly with these economic factors. Nighttime light not only serves as a proxy for economic activity but also reflects urbanization levels, energy consumption, and human activity intensity [26]. NDVI, an indicator of regional green space, may influence HFMD risk by modifying behavioral exposures and physiological recovery [27]. While these indicators offer novel insights, their specific roles as modifiers of the temperature-HFMD relationship remains limited, especially in high-burden regions such as Guizhou Province that face a distinct scarcity of epidemiological evidence regarding multiple environmental exposures [28,29].

To address these gaps, this study introduces ETEs, PM_2.5_, PM_10_, O_3_, nighttime light, and NDVI to broaden the scope of regional heterogeneity research. We applied a Distributed Lag Nonlinear Model (DLNM) combined with meta-regression to analyze the HFMD time-series data from 88 counties in Guizhou Province in western China. The aim was to quantify the modifying effects of these environmental factors on the temperature-HFMD relationship. Additionally, spatial clustering analysis based on these environmental modifiers was performed to identify distinct regional patterns in temperature-HFMD associations.

## Materials and methods

### Ethics statement

The HFMD data used in this study was obtained from the Guizhou Provincial Center for Disease Control and Prevention. The dataset did not contain any personally identifiable information, such as names, identification numbers, or contact details. No individual-level interventions were conducted, and there was no direct contact with participants. This study was conducted at the county level using spatially aggregated data and did not involve any individual-level analysis or operations. In accordance with established ethical guidelines and practices for public health surveillance research, formal ethical approval and informed consent were not required.

### Study area and population

This study analyzed 484,928 HFMD cases among children under six years old in Guizhou Province, China, from 2013 to 2019, using data from the China Disease Prevention and Control Information System. Guizhou, located in western China (103°36′–109°35′E, 24°37′–29°13′N), covers 176,200 km^2^, with 88 counties and a population of 38.56 million as of November 1, 2020. Daily case counts were aggregated based on the first date of symptom onset for each county to support spatio-temporal analysis.

### Map data

The Guizhou Province base map was sourced from the National Platform for Common GeoSpatial Information Services (Tianditu platform; https://cloudcenter.tianditu.gov.cn/administrativeDivision) in Chinese, under the Map Review Approval Number: GS (2024) 0650.

### Meteorological data

Meteorological data were sourced from the China Meteorological Data Sharing Service (http://data.cma.cn/site/index.html), including daily measurements of temperature, air pressure, precipitation, relative humidity, wind speed, and sunshine duration from 31 national surface meteorological stations in Guizhou Province. Spatial interpolation of station data to 88 county-level administrative units across the province was performed using the nearest neighbor matching method to ensure geographical accuracy.

Following previous research practices, this study extends the analysis by treating ETEs as regional environmental factors [11,12]. Specifically, heat waves and cold spells were examined [30,31], with heatwave days defined as consecutive days (*D* ≥ 3 days) with daily maximum temperature (*T*_max_) above the 95th percentile threshold (*T*_max_ ≥ 95th percentile), whereas cold spell days were identified as sustained periods (*D* ≥ 3 days) with daily minimum temperature (*T*_min_) below the 5th percentile threshold (*T*_min_ ≤ 5th percentile). Compared to singular temperature metrics, ETEs provide a multidimensional characterization of event intensity, frequency, and duration [14]. Binary indicators (1 for event days, 0 otherwise) were used to quantify ETE occurrences, with cumulative counts of heatwave and cold spell days subsequently analyzed at the county level [32].

### Other County-level data

Air pollution data were derived from the China Air Pollutants Dataset (CHAP) (https://weijing-rs.github.io/product.html), which provides daily PM_2.5_ and PM_10_ concentrations at 1 km resolution, and O_3_ concentrations at 10 km resolution. These data were generated using the Spatial-Temporal Extremely Random Tree model, which has been validated for accuracy and reliability [33–35]. In addition, data on selected PM_2.5_ components—including ammonium (${NH}_{4}^{+}$ ), nitrate (${NO}_{3}^{-}$), black carbon (BC), and organic matter (OM)—were obtained from the TAP dataset (http://tapdata.org.cn/?page_id=129), provided at 10 km resolution. These constituents, although not exhaustive, represent key components relevant to health and environmental studies.

Environmental indicators of urbanization, including nighttime light and Normalized Difference Vegetation Index (NDVI), were used to represent human activity and green space, respectively [36,37]. Nighttime light data were obtained from VIIRS on board the Suomi NPP satellite, provided by the Earth Observation Group (EOG), with annual mean illuminance (1 km × 1 km resolution) after quality control to remove interference (e.g., auroras, biomass burning). NDVI data representing vegetation cover was obtained from the NASA MODIS13Q1 dataset, with values ranging from -1 to 1 and updated every 16 days at 1 km resolution. Both indicators were aggregated into county-level annual averages using GIS tools in R.

Finally, county-level socio-economic data (population density and GDP per capita) were obtained from the Guizhou Provincial Statistical Yearbook and included as covariates to account for spatial heterogeneity in development and demographics.

### Study design and statistical analysis

The analysis consisted of three stages. First, county-level temperature-HFMD relationships were estimated. Second, a meta-regression analysis was performed to identify environmental factors contributing to spatial heterogeneity. Finally, cluster analysis was applied to group counties with similar heterogeneous characteristics to further explore their grouping patterns.

### County-level nonlinear and lagged analysis

We utilized quasi-Poisson regression combined with DLNMs [9]; this framework utilized a two-dimensional exposure-lag-response model to characterise the relationship between temperature and HFMD, using a cross-basis function within the DLNM framework [38]. The equation represents the applied specific model for temperature exposure:


$$Y_{t}=\text{Quasi}-\text{Poisson}(u_{t})$$

$$\begin{array}{cc} \text{Log}[E(Y_{t})]=\; & cb(temperature)+ns(ralative\;humidity,df=\text{3})+ns(windspeed,df=\text{3})\\ & \;\;+ns(sunshine\;duration,df=\text{3})+ns(airpressure,df=\text{3})+ns(doy,df=\text{8}*\text{7})\\ & \;\;+dow+holidays \end{array}$$

where *Y*_*t*_ is the daily count of HFMD cases on day t, following a quasi-Poisson distribution to account for overdispersion; *u*_*t*_ is the mean of the quasi-Poisson distribution, representing the expected number of HFMD cases on day t. To capture the non-linear exposure-response and lag-response associations, natural cubic splines (ns) with 3 degrees of freedom (*df*) were applied to both dimensions. The degrees of freedom were selected based on the model fit evaluated by the Quasi Akaike Information Criterion (QAIC) [39], as detailed in Supplementary Materials. To thoroughly investigate the lag structure of the temperature effect, a maximum lag of 14 days was chosen, taking into account the incubation and duration of HFMD [11,12]. To account for long-term trends and seasonal variations, natural cubic splines with 8 *df* per year were applied to calendar time. Moving averages of meteorological variables (relative humidity, wind speed, sunshine duration, air pressure) over the same lag period were incorporated to control for potential time-varying confounders. In addition, day of the week (*dow*) and holiday indicators (including school vacations and national public holidays) were included to further adjust for temporal patterns.

### Meta-regression analysis of spatial heterogeneity

In the second stage, a meta-regression approach was applied to pool county-specific temperature-HFMD associations, aiming to identify environmental factors driving spatial heterogeneity [40]. Each environmental factor was first assessed individually for its contribution to heterogeneity. Factors showing significant associations were subsequently entered into a multivariate meta-regression model using a stepwise procedure. As population density and GDP per capita are well-documented determinants of infectious disease dynamics [41], these were treated as fixed covariates throughout the model selection process. To mitigate multicollinearity, we screened regional variables by excluding variable pairs with a correlation coefficient greater than 0.8 [42]. In addition to this pairwise analysis, we specifically evaluated the four key fixed covariates—nighttime light, NDVI, population density, and GDP per capita—using the Variance Inflation Factor (VIF), applying a conservative threshold of 2.5 to identify potential issues [43]. The final set of predictors was determined based on model fit assessed by the Akaike Information Criterion (AIC) and significance tested using the multivariate likelihood ratio (LR) test. Residual heterogeneity was evaluated using the *I*^2^ statistic and Cochran’s Q test [39].

### Spatial clustering analysis

Based on the identified environmental drivers of heterogeneity, hierarchical cluster analysis was performed to group counties into distinct clusters with similar heterogeneity profiles. Subsequent cluster-specific analyses were conducted to identify homogeneous regions with similar environmental and socioeconomic profiles. First, variables were selected based on prior heterogeneity analysis and multicollinearity assessment. Following Z-score standardization of all continuous variables, model-based clustering was conducted using the *Mclust* package in R, which evaluates 14 distinct covariance parameterizations ranging from spherical to general (ellipsoidal) covariance structures. The optimal number of clusters and the corresponding covariance model were determined by maximizing the Bayesian Information Criterion (BIC). After model-based clustering, hierarchical cluster analysis was performed to group counties into distinct clusters with similar heterogeneity profiles. This clustering approach provided additional insights into spatially clustered patterns of vulnerability. For comprehensive details regarding the methodology and steps involved, refer to Supporting Information S1 Text.

### Sensitivity analysis

To assess the robustness of our findings, sensitivity analyses were conducted by varying the *df* for the exposure-response, lag-response, and meteorological covariates (relative humidity, wind speed, sunshine duration, and air pressure) from 3 to 5. Furthermore, to evaluate the influence of heat wave days and cold spell days definitions, we applied alternative definitions: heat wave was defined as daily maximum temperature (*T*_max_, °C) ≥ 95th percentile for at least 2 consecutive days, while cold spell was defined as daily minimum temperature (*T*_min_, °C) ≤ 5th percentile for at least 2 consecutive days. These alternative definitions were incorporated into the meta-regression framework.

All analyses were conducted in R 4.2.1, using “data.table” and “dplyr” for data processing, “dlnm” for distributed lag non-linear modeling, “mvmeta” for meta-analysis, “sp” and “sf” for spatial analysis, “raster” for raster data, and “ggplot2” for visualization.

## Results

### Descriptive characteristics of HFMD cases and environmental exposures

**Fig 1:**
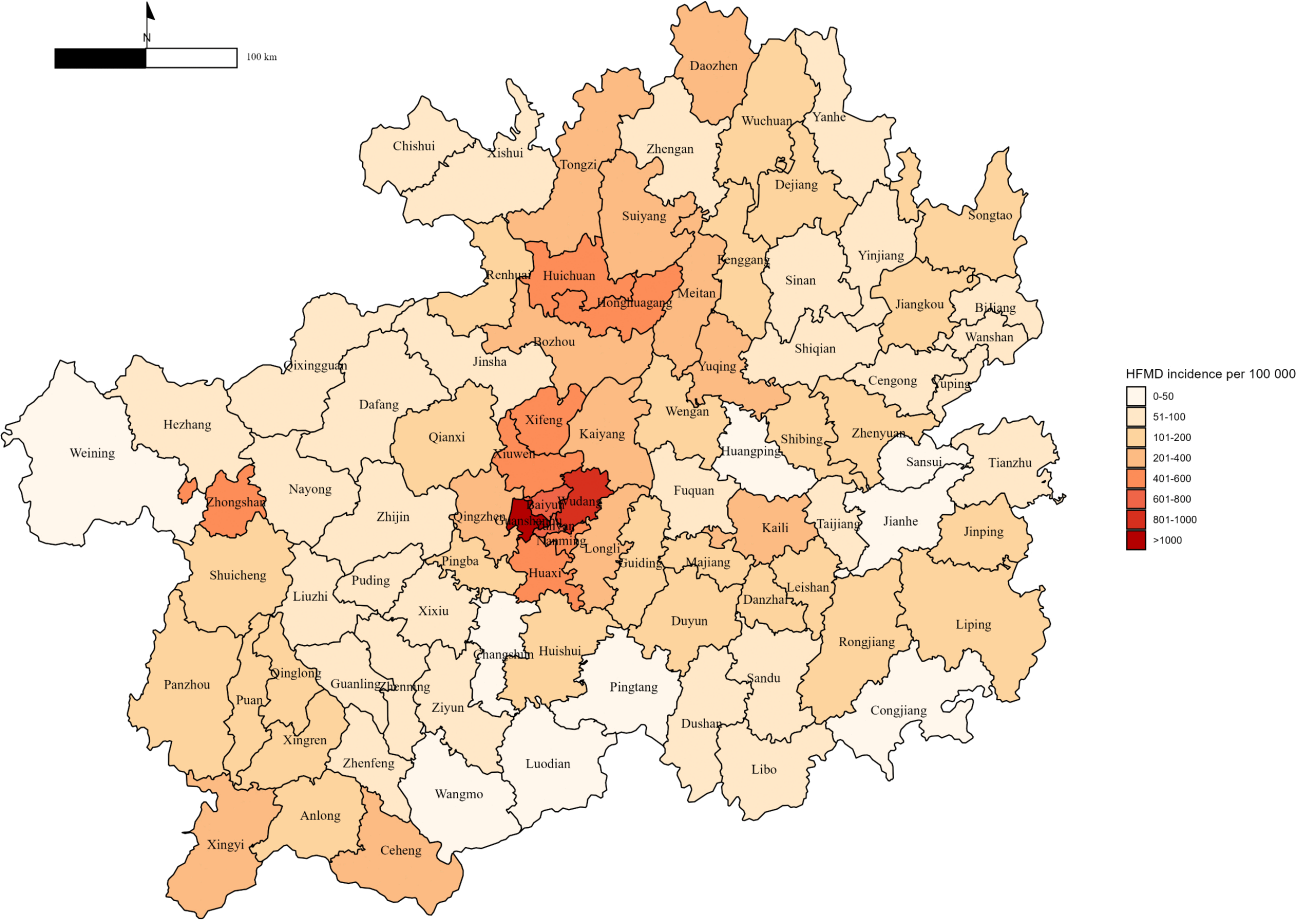
Spatial distribution of county-level HFMD incidence in Guizhou Province from 2013 to 2019. The base layers of the map were obtained from the National Platform for Common GeoSpatial Information Services via the Tianditu platform (in Chinese) (https://cloudcenter.tianditu.gov.cn/administrativeDivision).

Table 1 and Fig 1 present the descriptive statistics of HFMD cases and environmental factors across 88 counties in Guizhou Province from 2013 to 2019, highlighting spatial variation. A total of 484,928 HFMD cases were reported among children under six years old, with a median of 2,846 cases per county (range: 326–44,096). Population density varied considerably, ranging from 53 to 10,575 persons per km^2^, while GDP per capita ranged from CNY 16,251 to CNY 116,523. Air pollution levels also varied considerably, with median concentrations of 34.12 µg/m^3^ for PM_2.5_, 54.17 µg/m^3^ for PM_10_, and 75.52 µg/m^3^ for O_3_. In addition, important PM_2.5_ components such as ${NO}_{3}^{-}$, ${NH}_{4}^{+}$, BC, and OM showed substantial differences across counties (Table 1). Indicators of environmental urbanization were also highly variable, with a median nighttime light intensity of 52.30 nW/cm^2^/sr and a median NDVI of 0.54 (Table 1). A full list of the 88 counties included in the study is provided in the Supporting Information (S1 Table). In addition, the temporal variation of six environmental variables across Guizhou Province from 2013 to 2019 is presented in the Supporting Information (S1 Fig).

**Table 1:** Characteristics of 88 counties in Guizhou Province, 2013-2019.

Variables^a^	Min	25^th^	Median	75^th^	Max
HFMD Case					
Annual number for each county (person)	326	1228	2846	6613	44096
Demographic factors					
Population density (people per km^2^)	53	119	176	277	10575
Economic factors					
GDP per capita (CNY)	16251	23936	27678	39612	116523
ETEs factors					
Number of heat wave days (Heat waves)	13	27	39	46	57
Number of cold spell days (cold spells)	37	44	49	53	65
Air pollution factors^a^					
PM_2.5_ (µg/m^3^)	29.12	32.32	34.12	36.66	44.68
PM_10_ (µg/m^3^)	47.35	50.91	54.17	57.98	68.79
O_3_ (µg/m^3^)	71.01	73.80	75.52	77.68	83.43
PM_2.5_ components factors					
${NO}_{3}^{-}$ (µg/m^3^)	4.23	5.57	6.59	7.74	10.18
BC (µg/m^3^)	1.63	2.06	2.28	2.76	3.74
OM (µg/m^3^)	7.10	8.85	10.37	11.77	15.97
${NH}_{4}^{+}$ (µg/m^3^)	3.61	4.42	5.07	5.83	7.77
Environmental urbanization indicators					
Nighttime light (nW/cm^2^/sr)	41.96	48.76	52.30	58.33	66.59
NDVI	0.37	0.52	0.54	0.56	0.61

^a^The data from 2013 to 2019 was processed to calculate the mean values for each factor across the 88 counties in Guizhou Province. Quartiles were then used to summarize these characteristics, describing their central tendency and dispersion.

### Nonlinear temperature-HFMD association and spatial heterogeneity

**Fig 2:**
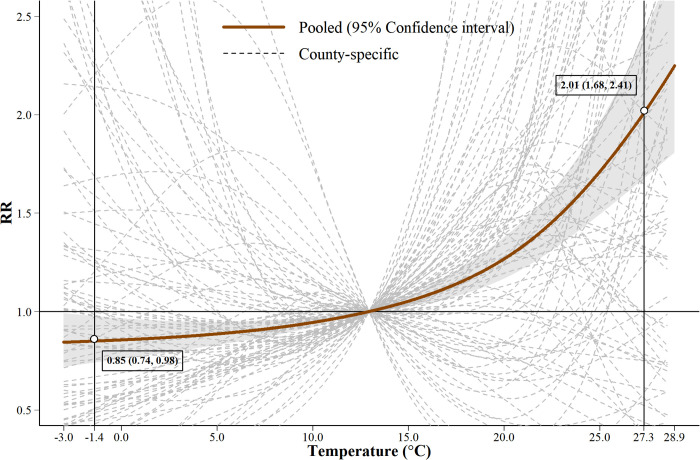
County-level and overall estimates of temperature-HFMD associations. Note: 1) All estimates are referenced to the median temperature (12.9 °C). 2) The two vertical black lines represent the 5th (1.4 °C) and 95th (27.3 °C) percentiles of the temperature distribution. 3) The gray band represents the 95% confidence interval for the pooled effect estimate. 4) The gray dashed lines represent county-specific estimates of temperature-HFMD associations.

As shown in Fig 2, the pooled exposure-response curve between temperature and HFMD exhibits a characteristic J-shaped pattern. Compared to the median temperature, the relative risk (*RR*) of HFMD at the 5th percentile temperature (-1.4 °C) was 0.85 (95% confidence interval [CI]: 0.74, 0.98), whereas at the 95th percentile temperature (27.3 °C), the *RR* increased sharply to 2.01 (95% CI: 1.68, 2.41). The dashed lines indicate the county-specific estimates, showing considerable variation in the temperature-HFMD associations across the 88 counties, suggesting the presence of spatial heterogeneity. Lag-response relationships are shown in Supporting Information (S2 Fig).

### Environmental determinants of spatial heterogeneity in the temperature–HFMD association

Table 2 presents the multivariate meta-regression results quantifying the spatial heterogeneity in the temperature-HFMD associations. The intercept-only model indicated moderate heterogeneity (*I*^2^ = 50.08%). Six environmental variables were identified as significant contributors to spatial heterogeneity in the temperature-HFMD association. These included cold spells (*p* < 0.001, Δ*I*^2^ = 3.84%, representing the proportion of spatial heterogeneity above the baseline level), PM_2.5_ (*p* = 0.001, Δ*I*^2^ = 3.06%), heat waves (*p* = 0.001, Δ*I*^2^ = 2.72%), PM_10_ (*p* = 0.020, Δ*I*^2^ = 2.08%), NDVI (*p* = 0.025, Δ*I*^2^ = 1.57%), and O_3_ (*p* = 0.041, Δ*I*^2^ = 0.78%). Notably, the concentration difference between PM_10_ and PM_2.5_ was not statistically significant (*p* = 0.416), suggesting limited relevance in explaining spatial heterogeneity. Given the strong correlation between PM_2.5_ and PM_10_ (*r* = 0.97) (S3 Fig), the observed PM_10_ effect may largely reflect the contribution of PM_2.5_. Taken together, cold spells, heat waves, PM_2.5_, and NDVI emerged as the main contributors to spatial heterogeneity. In addition, further analysis of four PM_2.5_ components identified ${NO}_{3}^{-}$ (*p* = 0.026, Δ*I*^2^ = 1.78%) and ${NH}_{4}^{+}$ (*p* = 0.028, Δ*I*^2^ = 1.58%) as additional modifying factors. To identify the optimal combination of factors explaining spatial heterogeneity, we employed a stepwise approach to build multivariate meta-regression models (Table 2). Although a full model including all significant modifiers was tested, it did not improve explanatory power, resulting in a slightly lower Δ*I*^2^ of 5.07%. In contrast, the optimal model, selected on the basis of both the Akaike Information Criterion (AIC) and the proportion of heterogeneity explained, included four variables: population density, GDP per capita, cold spells, and PM_2.5_. These variables together accounted for 6.20% of the spatial heterogeneity. The Wald test confirmed the statistical significance of these factors (*p*-values < 0.001), highlighting their key role in shaping regional variation in temperature-related HFMD risk.

**Table 2:** Quantification of spatial heterogeneity using multivariate meta-regression models.

Meta-predictors	Wald test	Model fits			Cochran Q test^a^			Heterogeneity (%)	
	(*p*)	LogLik	AIC	BIC	*Q*	*df*	*p*	*I* ^2^	Δ*I*^2b^
**Intercept-only model**									
Intercept only	–	–410.60	839.20	871.28	522.86	261	<0.001	50.08	–
**Single meta-predictor models**									
**ETEs**									
Heat waves	0.001	-413.71	851.43	894.06	490.15	258	<0.001	47.36	2.72
Cold spells	<0.001	-409.75	843.50	886.14	479.91	258	<0.001	46.24	3.84
**Air pollution factors**									
PM_2.5_	0.001	-411.55	847.10	889.74	486.93	258	<0.001	47.02	3.06
PM_10_	0.020	-414.98	853.96	896.60	496.17	258	<0.001	48.00	2.08
PM_10_ -PM_2.5_	0.416	-415.14	854.27	896.91	512.15	258	<0.001	49.62	0.46
O_3_	0.041	-413.19	850.38	893.02	508.87	258	<0.001	49.30	0.78
**PM**_**2.5**_ **components factors**									
${NO}_{3}^{-}$	0.026	-411.79	847.58	890.21	499.03	258	<0.001	48.30	1.78
BC	0.187	-410.30	844.60	887.24	510.07	258	<0.001	49.42	0.66
OM	0.180	-414.63	853.25	895.89	507.55	258	<0.001	49.17	0.91
${NH}_{4}^{+}$	0.028	-410.59	845.18	887.81	500.95	258	<0.001	48.50	1.58
**Environmental urbanization indicators**									
Nighttime light	0.279	-418.18	860.37	903.00	513.92	258	<0.001	49.8	0.28
NDVI	0.025	-401.11	826.21	868.85	501.04	258	<0.001	48.51	1.57
**Multiple meta-predictors model**									
**Three factors meta-predictors model (Optimal model)**									
Population density + GDP per capita + cold spells	<0.001	-467.39	970.77	1034.30	465.39	252	<0.001	45.85	5.52
**Four factors meta-predictors model (Optimal model**									
Population density + GDP per capita + Cold spells + PM_2.5_	<0.001	-472.02	986.04	1059.90	454.12	249	<0.001	45.17	6.20
**Five factors meta-predictors model (Optimal model)**									
Population density + GDP per capita + Cold spells + PM_2.5_ + NDVI	<0.001	-462.81	973.61	1057.74	449.46	246	<0.001	45.27	6.10
**Six factors meta-predictors model (Optimal model)**									
Population density + GDP per capita + Heat waves + Cold spells + PM_2.5_ + NDVI	0.001	-472.1	998.20	1092.51	447.85	243	<0.001	45.74	5.63
**Seven factors meta-predictors model (Optimal model)**									
Population density + GDP per capita + Heat waves + Cold spells + PM_2.5_ + O_3_ + NDVI	0.002	-477.73	1015.46	1119.88	446.89	240	<0.001	46.3	5.07

^a^The Cochran Q test was utilized to assess residual heterogeneity.

^b^Δ*I*^2^ represents the difference in *I*^2^ between the meta-regression model including a given predictor and the intercept-only model. This value reflects the proportion of spatial heterogeneity explained by that predictor.

In addition, this phase of the analysis primarily focuses on the correlations between nighttime light, NDVI, and key socioeconomic indicators, including population density and GDP per capita. The correlation matrix (S3 Fig) revealed that, among the four variables, NDVI and population density exhibited the highest correlation (*r* = -0.62), which remains below the |0.8| threshold commonly used to indicate strong correlation. Consistent with this, VIF values for all four variables were below 2.5 (S7 Table), indicating low multicollinearity.

### Modifying effects of regional factors on temperature-related HFMD risk

**Fig 3:**
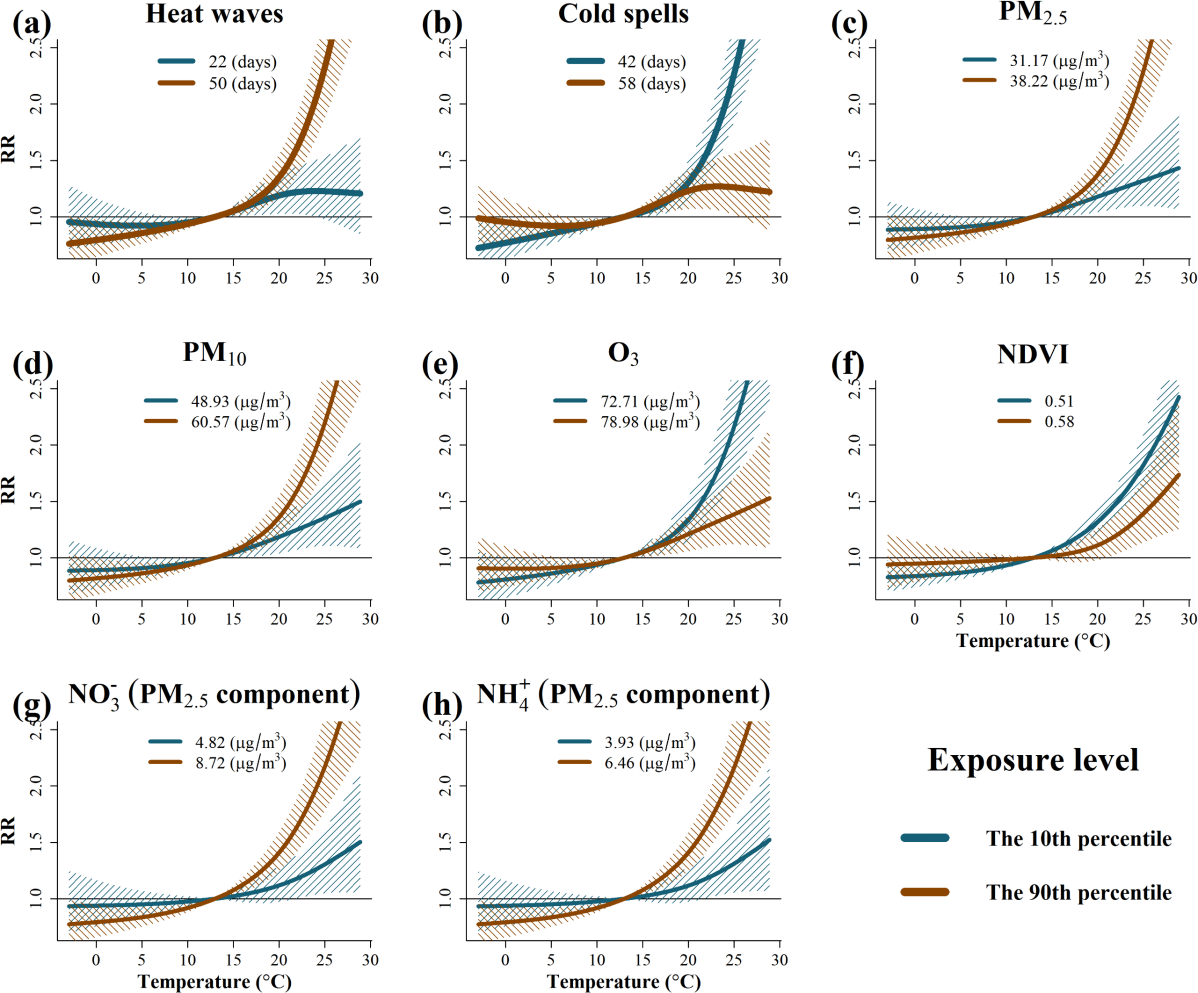
Pooled temperature-HFMD associations stratified by eight environmental modifiers contributing to spatial heterogeneity, Guizhou Province, 2013–2019. Notes: 1) The curves represent the temperature-HFMD associations at the 10th (blue line) and 90th (red line) percentiles of each spatial environmental factor. 2) The reference temperature is 12.9 °C. 3) Shaded areas indicate 95% confidence intervals.

Fig 3 illustrates the modifying effects of eight regional factors—heat waves, cold spells, PM_2.5_, PM_10_, O_3_, NDVI, and two PM_2.5_ components (${NO}_{3}^{-}$ and ${NH}_{4}^{+}$)—on the temperature-HFMD association. These modifiers showed relatively stable effects at low temperatures but either amplified or suppressed HFMD risk at higher temperatures. For example, regions with more frequent heat waves (the 90th percentile) exhibited a substantially higher HFMD risk under high-temperature conditions. At the 95th percentile of temperature in these regions, the *RR* of HFMD was 3.17 (95% CI: 2.39-4.19), whereas in regions with infrequent heat waves (the 10th percentile), the *RR* was 1.23 (95% CI: 1.03-1.63) (Fig 3a; S2 Table). A similar modifying effect was observed for PM_2.5_. At the 95th percentile of temperature in PM_2.5_ high-exposure regions (the 90th percentile), the *RR* of HFMD was 3.05 (95% CI: 2.35-3.95), compared with 1.39 (95% CI: 1.10-1.77) in regions with lower PM_2.5_ concentrations (the 10th percentile) (Fig 3c; S2 Table). Comparable modification patterns in temperature-HFMD relationship were also identified for PM_10_ and two PM_2.5_ components (Fig 3d, 3g, and 3h; S2 Table). Conversely, cold spells demonstrated a protective modifying effect on the temperature-HFMD relationship. Under the same high temperature condition (the 95th percentile), regions with more frequent cold spells (the 90th percentile) had an *RR* of HFMD of 1.27 (95% CI: 1.07-1.63), compared to 3.13 (95% CI: 2.40-4.07) in regions with fewer cold spells (the 10th percentile) (Fig 3b; S2 Table). A similar inverse pattern was also evident for NDVI and O_3_. For NDVI, at the 95th percentile of temperature in regions with low NDVI (the 10th percentile) showed a higher HFMD *RR* of 2.16 (1.79-2.59) compared to 1.59 (1.21-2.08) in regions with higher NDVI (the 90th percentile) (Fig 3f). Details of results for O_3_ and the lag-response relationships of these environmental modifiers are provided in the Supporting Information (S2 Table and S4 Fig).

### Cluster analysis of regional heterogeneity in temperature–HFMD associations

**Fig 4:**
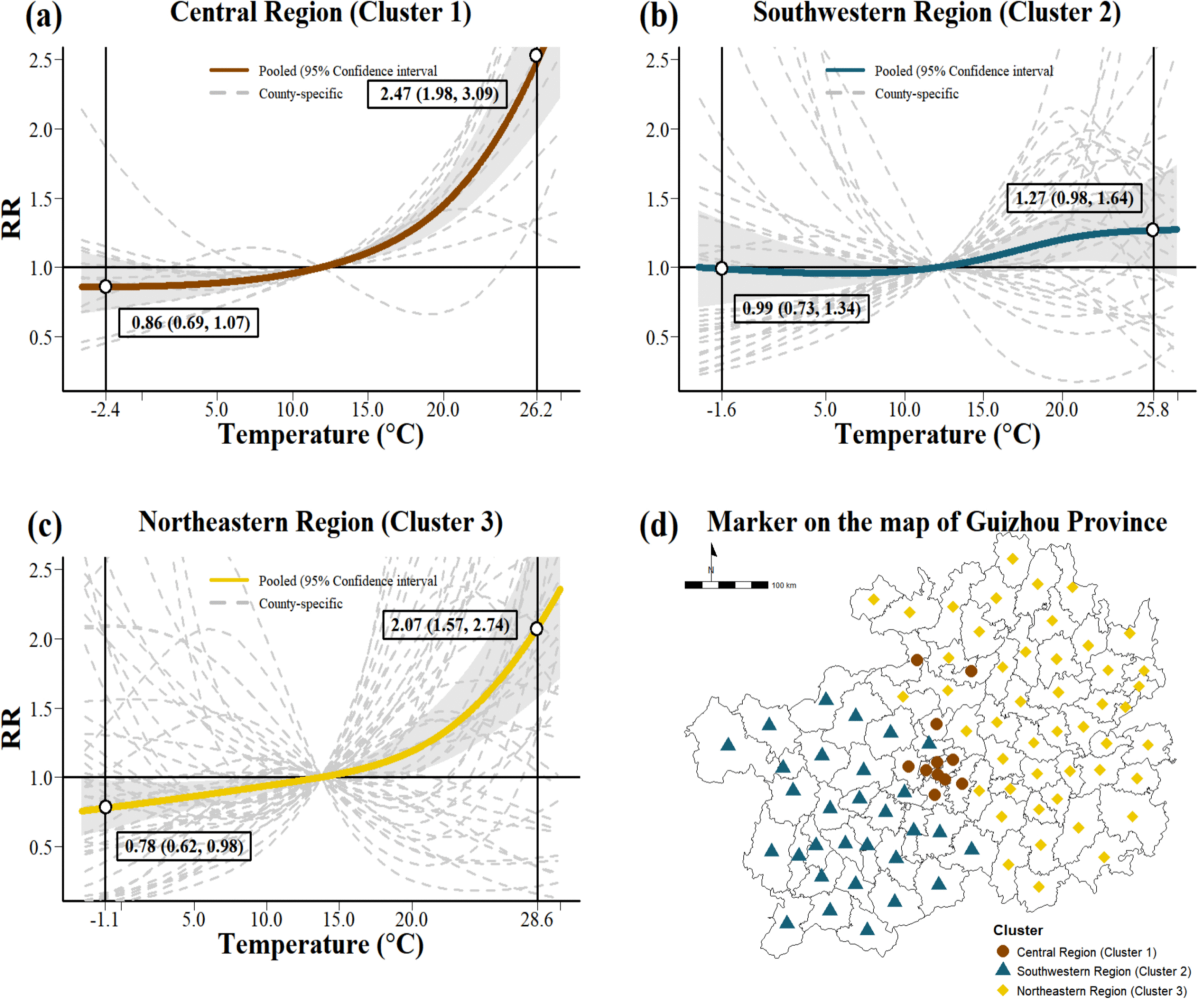
Temperature-HFMD associations in three spatial clusters. The base layers of the map were obtained from the National Platform for Common GeoSpatial Information Services via the Tianditu platform (in Chinese) (https://cloudcenter.tianditu.gov.cn/administrativeDivision). Notes: Gray shaded areas indicate 95% confidence intervals, and gray dashed lines represent county-level estimates.

Our study identified 3 distinct clusters among the 88 counties using 14 clustering algorithms, with the optimal classification determined by the Bayesian Information Criterion (S5 Fig). Fig 4 illustrates the temperature-HFMD associations across clusters, highlighting substantial regional heterogeneity. Analysis of variance (ANOVA) results (S4 Table, S6 Fig) consistently yielded *p*-values < 0.05, indicating significant differences in environmental characteristics between the clusters. In the central region (Cluster 1; Fig 4a), HFMD risk increased sharply with increasing temperature, reaching a *RR* of 2.47 (1.98-3.09) at the 95th percentile temperature (26.2 °C). A similar upward trend was observed in the northeastern region (Cluster 3; Fig 4c), with a peak *RR* of 2.07 (1.57-2.74) at the 95th percentile (28.6 °C). By contrast, the south-western region (Cluster 2; Fig 4b) showed an S-shaped exposure-response relationship with no clear monotonic pattern. At the 95th percentile (25.8 °C), the *RR* was 1.27 (0.98-1.64), indicating a nonsignificant association. A full list of the 88 counties classified into the three clusters is provided in Supporting Information (S3 Table).

### Sensitivity analyses

Sensitivity analyses confirmed the robustness of the model, with QAIC values ranging from 6587.50 to 6605.00 across different *df* configurations. The optimal model used 3 *df* for exposure-response, lag-response and meteorological covariates (S5 Table). In addition, alternative definitions of heat wave days and cold spell days were used. The heterogeneity analysis based on alternative definitions remained statistically significant, supporting the robustness of the primary findings despite small variations in heterogeneity (S6 Table).

## Discussion

Our study identified eight statistically significant effect modifiers of the temperature–HFMD association: heat waves, cold spells, PM_2.5_, PM_10_, NDVI, O_3_, and two PM_2.5_ components (${NO}_{3}^{-}$ and ${NH}_{4}^{+}$). To the best of our knowledge, evidence on these spatial effect modifiers has not been previously reported in the literature. Among them, cold spells, heat waves, PM_2.5_, and NDVI were the main contributors to spatial heterogeneity. Specifically, heat waves and PM_2.5_ positively modified the association, increasing the risk of HFMD under high temperature conditions. In contrast, cold spells, NDVI, and O₃ negatively modified the association, attenuating the temperature-related risk. Further spatial cluster analysis revealed three distinct regional patterns of the temperature-HFMD association, reinforcing the spatially modifying role of these environmental factors across regions.

In studies of spatial heterogeneity in infectious diseases like HFMD, which are influenced by multiple complex factors, the proportion of heterogeneity explained by each individual factor is typically limited. For instance, key meteorological variables such as temperature, rainfall, and relative humidity usually explain 1% to 3% of the spatial variation in disease incidence, even after considering socioeconomic factors [11,12,28]. However, this relatively modest explanatory power does not diminish the value of these factors, as they can still provide meaningful insights when statistically significant. In contrast, this study focuses on ETEs (cold spells and heat waves), which are expected to have a greater potential impact on human health, particularly on children, compared to average temperature. The explanatory power we found for these extreme events (3.82% for cold spells, 2.72% for heat waves) is, respectively, higher than or close to the upper limit of the range typically reported for conventional meteorological variables (e.g., 1–3%), which aligns with theoretical expectations [11]. Furthermore, the inclusion of air pollutants (PM_2.5_: 3.06%, PM_10_: 2.08%) and NDVI (1.57%) significantly increased the explanatory power, surpassing the explanatory values of many conventional meteorological variables in previous studies. Even though O_3_ (0.78%) showed lower explanatory power, it remained statistically significant. In this study, the four factors—cold spells, PM_2.5_, population density, and GDP per capita—collectively explain 6.2% of the heterogeneity. This cumulative explanatory power not only significantly exceeds the level of single factors in previous studies but also reflects the combined effect of multiple significant factors. Specifically, the individual explanatory power of cold spells (3.82%) and PM_2.5_ (3.06%) both surpass the typical range of 1%-3% for conventional meteorological variables reported in prior research. Population density and GDP per capita, meanwhile, are well-recognized key factors influencing regional heterogeneity in existing studies. Thus, these four factors can be identified as the main contributors to the spatial heterogeneity of HFMD.

Building on the discussion of the combined effects of multiple factors driving spatial heterogeneity in HFMD, this study reveals that heat waves and cold spells independently explain the spatial heterogeneity in temperature-related HFMD risk, significantly surpassing the explanatory power of average temperature [11,12]. This underscores the critical role of ETEs as key drivers of regional heterogeneity in the temperature-HFMD association. The stronger contribution of ETEs can be mechanistically supported by children’s unique biological and behavioral vulnerabilities, particularly regarding heat waves. Biologically, extreme heat accelerates thermoregulation (e.g., sweating and skin perfusion), leading to dehydration and increased cardiac output [14]. When thermoregulation fails, core body temperature rises, triggering systemic inflammation, oxidative stress, endothelial dysfunction and direct cytotoxicity, all of which can impair immune responses and increase susceptibility to viral infections [44,45]. These effects are of particular concern in children, whose thermoregulatory systems and immune defenses are still developing. Behaviorally, heatwaves may alter caregiving routines and increase children’s exposure to communal environments such as swimming pools or other public facilities [17], thereby increasing opportunities for person-to-person contact and exposure to enterovirus-contaminated water [46,47]. In regions with frequent cold spells, the absence of an increased risk of HFMD under high temperature conditions may be due to behavioral adaptation. Residents in these regions are more sensitive to temperature changes and tend to adopt precautionary behaviors during heat events, such as reducing outdoor activity and adopting protective measures. These adaptive behaviors are likely to contribute to the reduced risk of HFMD observed during periods of high temperature in these regions.

In addition to temperature-related factors, ambient air pollution may further modify the temperature-HFMD association and contribute to regional disparities. Our results underscore the important role of air pollution—particularly PM_2.5_, PM_10_, O_3_, and two PM_2.5_ components (${NO}_{3}^{-}$ and ${NH}_{4}^{+}$)—in shaping the spatial heterogeneity of this association. Specifically, the strength of the temperature-HFMD relationship varied with regional PM_2.5_ levels, with markedly higher HFMD risk under high-temperature conditions observed in areas with elevated PM_2.5_ concentrations. Although direct evidence on PM_2.5_ as a modifier of the temperature-HFMD association remains limited, several studies have reported significant associations between PM_2.5_ exposure and HFMD incidence. For example, a study conducted in Shijiazhuang [48], a city with severe PM_2.5_ pollution, found an increased risk of HFMD when PM_2.5_ concentrations ranged from 76 to 200 μg/m^3^, whereas a protective effect was observed at extremely high concentrations. These results are not directly comparable with our findings because of the much lower PM_2.5_ concentrations in our study area (29.12-44.68 μg/m^3^). In contrast, a study conducted in Guilin [25], a geographically adjacent area, reported a positive association between PM_2.5_ and HFMD, with an estimated risk ratio of 1.01 (95% CI: 0.70-1.46), partially supporting the plausibility of our findings. Two plausible mechanisms may explain this relationship: first, enteroviruses may adsorb to particulate matter, increasing the atmospheric stability and long-range transmission potential of these viruses [49]; second, PM_2.5_ may impair host defenses by inducing oxidative stress, promoting inflammation and even causing genetic damage, thereby increasing susceptibility to infection [50,51]. Although synergistic effects between PM_2.5_ and high temperatures on HFMD have not been directly confirmed, previous studies have shown that co-exposure to high PM_2.5_ levels and elevated temperatures can exacerbate health risks [52], which indirectly supports our findings. Moreover, the observed modification of the temperature-HFMD association by PM_2.5_ suggests a potential synergistic effect with high temperature. To test this hypothesis, future studies could explicitly model the interaction between PM_2.5_ and heat waves. Beyond association, analytical approaches such as Structural Equation Modeling could be applied to elucidate the causal pathways linking temperature, air pollution, and HFMD risk. In contrast, O_3_ accounted for only 0.78% of the observed heterogeneity, suggesting a relatively limited role in modifying the temperature-HFMD relationship at the regional scale. However, unlike PM_2.5_, the modifying effect of O_3_ showed an inverse pattern - regions with lower O_3_ levels exhibited a stronger association between high temperatures and HFMD risk. This finding is consistent with experimental studies demonstrating that higher O_3_ concentrations can reduce viral survival and replication [53], as well as epidemiological evidence showing a negative association between ambient O_3_ levels and HFMD incidence [25]. Although the modifying effect of O_3_ as a regional factor appears to be weak, these results highlight the need for further mechanistic and epidemiological studies to explore the potential interactions between O_3_ and temperature in shaping enterovirus transmission under real-world environmental conditions.

To further explore the contribution of PM_2.5_ to spatial heterogeneity in the temperature-HFMD relationship, we examined the effects of four specific PM_2.5_ components, among which ${NO}_{3}^{-}$ and ${NH}_{4}^{+}$ emerged as key contributors. Both are secondary inorganic aerosols commonly derived from atmospheric reactions involving NO_x_ and ammonia, which originate from traffic emissions, agricultural activities, and industrial processes. Their potential mechanisms are similar to those of total PM_2.5_, including increased oxidative stress, immune dysfunction, and their role as carriers of enteric viruses [54,55]. However, these two components explained only part of the observed heterogeneity, suggesting that other PM_2.5_ constituents—such as organic carbon, elemental carbon, and trace metals—may further contribute. Future studies should characterize these components and quantify their individual and synergistic effects on the temperature-HFMD association.

In addition to ETEs and air pollutants, our study included broader environmental indicators of urbanization, extending previous research on spatial heterogeneity. While earlier studies mainly emphasized socio-economic factors such as per capita GDP, we introduced two novel indicators: NDVI, which reflects green space coverage, and nighttime light, which captures urban economic activity. Among them, NDVI emerged as a statistically significant modifier of the temperature-HFMD associational novel finding in our study. Consistent with cold spells and O₃, NDVI showed a negative modifying effect. Temperature-related HFMD risk was more pronounced in regions with low NDVI, whereas the association was attenuated in greener areas, suggesting a potential buffering role of vegetation against heat-related HFMD risk. Although NDVI has not previously been examined as a modifier in this context, our finding is consistent with previous evidence linking greater green space to reduced HFMD incidence [56]. Moreover, this protective role of vegetation appears to extend beyond HFMD. For instance, a multi-country study across 49 low- and middle-income countries reported that greater greenspace access was associated with lower prevalence of acute respiratory infections, fever, and diarrhea among children under five [57]. Similarly, a nationwide analysis of 266 Chinese cities found a negative association between NDVI and COVID-19 incidence [58], suggesting a general protective role of vegetation against infectious diseases. Mechanistically, this effect may operate through multiple pathways relevant to HFMD transmission. Environmentally, vegetation can moderate local microclimates via evapotranspiration, which may reduce enterovirus stability and human exposure [59]. Concurrently, green space attenuates air pollutants (e.g., PM_2.5_, O_3_), limiting exposure to virus-laden aerosols and mitigating pollutant-induced inflammation that could facilitate infection [60]. Behaviorally, access to greenspace may encourage outdoor activity in less crowded, better-ventilated settings, reducing close-contact transmission, while also enhancing immune and psychological resilience [61]. Future studies could explore the causal pathways through which NDVI influences HFMD transmission, or its potential interactions with temperature and air pollutants in modulating disease risk.

This study has several important strengths that enhance the validity of its findings. Most importantly, it incorporates complete population-level data covering all reported HFMD cases in Guizhou Province (n ≈ 500,000), providing substantial statistical power and minimizing selection bias. Furthermore, it advances current knowledge by systematically examining spatial variation in the temperature-HFMD association across different environmental contexts, providing novel insights into potential effect modification by ecological and pollution-related factors. Nevertheless, several methodological limitations need to be considered. First, as an ecological time-series study, the results are inherently limited in inferring individual-level causality. However, the large sample size combined with sensitivity analyses supports the robustness of the results. Second, the use of area-level environmental data to estimate exposure may lead to non-differential misclassification, which tends to attenuate effect estimates towards the null. Third, due to the lack of surveillance data on enterovirus subtypes (e.g., EV71, CA16) associated with HFMD, this study did not analyze the spatial heterogeneity of these viral subtypes. Different subtypes may vary in sensitivity to temperature and other environmental conditions, which could influence the observed heterogeneity in the temperature-HFMD relationship. This limitation is common in current HFMD spatial heterogeneity research, so caution is needed when extrapolating these findings to regions with distinct viral subtype distributions. Additionally, the study period was necessarily limited to 2013–2019, both because of the availability of standardized air pollution data and to minimize potential confounding from behavioral changes, health care disruptions and environmental changes during the COVID-19 pandemic. Although China’s HFMD surveillance system may underreport mild cases, such bias is unlikely to significantly affect internal comparisons, as time-series methods rely on relative variation within consistent reporting systems and adjust for seasonality and secular trends. Notably, the degree of heterogeneity observed in our study is consistent with previous findings [11,12] and supports the robustness of our estimates. However, the limited explanatory power of the measured modifiers highlights the complex, multifactorial nature of environmental health interactions. Future research should examine additional contributors, such as PM_2.5_ metal constituents, behavioral adaptation, regional access to health care, and viral strain variability.

## Conclusion

This study identified eight environmental modifiers contributing to the spatial heterogeneity in the association between temperature and HFMD, with heat waves, cold spells, PM_2.5_, and NDVI being the primary contributors. These findings provide new evidence on how environmental factors drive spatial variation in temperature–HFMD associations and deepen the understanding of environmental determinants of disease risk. The results demonstrate that temperature-related HFMD risks are substantially modified by regional environmental contexts, challenging the generalizability of single-site study. These insights also underscore the need for regional, tailored prevention strategies, including context-adapted greening interventions, to mitigate HFMD and other environmentally mediated infectious diseases.

## Supporting information

S1 Data Weekly meteorological DLNM dataset. (PDF)

S2 Data Multicounty aggregated dataset for meta-analysis. (PDF)

S1 Table List of 88 counties in Guizhou province included in the study. (DOCX)

S2 Table Relative risk analysis of heterogeneous environmental factors in the temperature-HFMD associations. (DOCX)

S3 Table A list of 88 counties in Guizhou Province divided into three clustering types. (DOCX)

S4 Table Statistical description of the three clusters and ANOVA. (DOCX)

S5 Table Sensitivity analysis comparing different definitions of heat wave and cold spell in meta-analysis. (DOCX)

S6 Table Sensitivity analysis of differently defined heat and cold spell in meta-analysis. (DOCX)

S7 Table Multicollinearity diagnostics based on Variance Inflation Factor (VIF). (DOCX)

S1 Fig Time series plot of six meteorological factors in Guizhou Province. (TIF)

S2 Fig Lag plots of county-level and overall estimates of temperature-HFMD associations. (TIF)

S3 Fig Correlation matrix of regional environmental factors. (TIF)

S4 Fig Lag plots of environmental modifiers contributing to spatial heterogeneity in temperature-HFMD associations. (TIF)

S5 Fig Model-based clustering plots. (TIF)

S6 Fig Lags of three clustered areas in the temperature–HFMD associations. (TIF)

S1 Text Details of the clustering analysis. (DOCX)
