# Supplementary material for: Spatial heterogeneity in the temperature–hand, foot, and mouth disease association among children: A multicounty time-series study in western China
Source: PLoS Negl Trop Dis. 2026 Jan 2;20(1):e0013801. doi: 10.1371/journal.pntd.0013801 (PMC12758769; doi:10.1371/journal.pntd.0013801)
Supplement: S1 Data — (PDF) [file pntd.0013801.s001.pdf]

seqcode	year	week	tmean	humidity	pressure	sunshine	windspeed
31	2013	1	-0.4833	79.8333	851.3202	1.6500	1.0310
79	2013	1	1.6333	79.9048	993.3607	0.9512	0.7631
51	2013	1	-0.6167	87.7024	951.0750	1.4798	1.6464
14	2013	1	0.2167	86.0595	905.1821	1.5071	2.4452
67	2013	1	-0.2500	79.5357	910.8321	1.5619	3.2298
42	2013	1	-0.4667	84.3333	881.6500	1.4214	2.6357
50	2013	1	0.8167	78.5595	911.1190	1.3929	1.7381
43	2013	1	-0.4667	84.3333	881.6500	1.4214	2.6357
85	2013	1	1.7333	86.9167	920.0369	0.9286	1.5190
25	2013	1	3.6500	82.5833	990.5369	1.3690	1.1940
69	2013	1	1.2333	74.1905	951.7667	2.0226	1.4440
57	2013	1	0.5333	88.8929	902.6952	0.7500	1.7274
9	2013	1	0.1500	81.6310	859.6786	1.7452	2.6595
72	2013	1	1.5833	75.3452	884.4048	1.9774	2.6095
26	2013	1	2.8333	79.3690	872.9631	1.9452	2.0488
7	2013	1	1.6500	77.7143	864.5000	2.3321	1.9917
83	2013	1	5.9667	73.7857	954.5893	1.4976	1.0321
76	2013	1	0.0667	81.5238	931.3060	1.6000	2.0214
36	2013	1	0.1333	84.4167	939.6667	1.5119	1.6190
81	2013	1	-0.6167	87.7024	951.0750	1.4798	1.6464
15	2013	1	1.7000	72.6667	944.3405	1.6429	0.9417
32	2013	1	-0.4667	84.3333	881.6500	1.4214	2.6357
73	2013	1	2.5167	80.1190	975.5774	1.5964	0.8310
71	2013	1	0.1333	84.4167	939.6667	1.5119	1.6190
41	2013	1	-0.0167	83.2500	881.2536	1.3071	1.2071
10	2013	1	0.5000	79.4405	977.9179	1.7000	1.4905
23	2013	1	-0.4000	74.8571	776.5929	4.8190	2.5881
27	2013	1	1.6500	77.7143	864.5000	2.3321	1.9917
60	2013	1	-0.6167	87.7024	951.0750	1.4798	1.6464
53	2013	1	0.1500	81.6310	859.6786	1.7452	2.6595
66	2013	1	0.2167	86.0595	905.1821	1.5071	2.4452
59	2013	1	0.5333	88.8929	902.6952	0.7500	1.7274
61	2013	1	2.5167	80.1190	975.5774	1.5964	0.8310
84	2013	1	2.5167	80.1190	975.5774	1.5964	0.8310
38	2013	1	0.5333	88.8929	902.6952	0.7500	1.7274
87	2013	1	1.7833	72.7262	907.4214	1.2714	2.7083
34	2013	1	0.5333	88.8929	902.6952	0.7500	1.7274
29	2013	1	1.2333	74.1905	951.7667	2.0226	1.4440
5	2013	1	0.1000	80.5833	836.1976	2.8214	2.0607
8	2013	1	0.1500	81.6310	859.6786	1.7452	2.6595
12	2013	1	0.1000	80.5833	836.1976	2.8214	2.0607
13	2013	1	5.9667	73.7857	954.5893	1.4976	1.0321
18	2013	1	0.9000	80.6429	978.1476	1.1048	1.1524
33	2013	1	0.8167	78.5595	911.1190	1.3929	1.7381
56	2013	1	3.6500	82.5833	990.5369	1.3690	1.1940
77	2013	1	1.7000	72.6667	944.3405	1.6429	0.9417
54	2013	1	0.1000	80.5833	836.1976	2.8214	2.0607
21	2013	1	0.8167	78.5595	911.1190	1.3929	1.7381
68	2013	1	1.6333	79.9048	993.3607	0.9512	0.7631

74	2013	1	2.5167	80.1190	975.5774	1.5964	0.8310
88	2013	1	-0.4667	84.3333	881.6500	1.4214	2.6357
16	2013	1	0.0667	81.5238	931.3060	1.6000	2.0214
30	2013	1	0.2167	86.0595	905.1821	1.5071	2.4452
6	2013	1	1.6333	79.9048	993.3607	0.9512	0.7631
49	2013	1	1.2333	74.1905	951.7667	2.0226	1.4440
22	2013	1	-0.4667	84.3333	881.6500	1.4214	2.6357
45	2013	1	2.1667	69.8571	820.7250	4.1548	2.4738
58	2013	1	1.2333	74.1905	951.7667	2.0226	1.4440
37	2013	1	1.6333	79.9048	993.3607	0.9512	0.7631
17	2013	1	-0.2500	79.5357	910.8321	1.5619	3.2298
55	2013	1	1.5833	75.3452	884.4048	1.9774	2.6095
46	2013	1	0.0667	81.5238	931.3060	1.6000	2.0214
86	2013	1	1.2333	77.2262	872.9238	1.5464	1.4393
2	2013	1	1.2333	77.2262	872.9238	1.5464	1.4393
4	2013	1	0.8167	78.5595	911.1190	1.3929	1.7381
47	2013	1	6.4667	70.8571	970.4607	1.5417	0.6560
82	2013	1	-0.4667	84.3333	881.6500	1.4214	2.6357
19	2013	1	4.7333	71.6071	972.0167	1.6845	1.3214
20	2013	1	0.1500	81.6310	859.6786	1.7452	2.6595
80	2013	1	-0.4667	84.3333	881.6500	1.4214	2.6357
3	2013	1	5.9667	73.7857	954.5893	1.4976	1.0321
52	2013	1	-0.2500	79.5357	910.8321	1.5619	3.2298
70	2013	1	1.7333	86.9167	920.0369	0.9286	1.5190
64	2013	1	-0.4000	74.8571	776.5929	4.8190	2.5881
48	2013	1	1.7000	72.6667	944.3405	1.6429	0.9417
65	2013	1	-0.2500	79.5357	910.8321	1.5619	3.2298
44	2013	1	1.7333	86.9167	920.0369	0.9286	1.5190
75	2013	1	-0.4000	74.8571	776.5929	4.8190	2.5881
40	2013	1	0.1667	88.3929	957.6738	1.6000	1.9750
11	2013	1	1.5833	75.3452	884.4048	1.9774	2.6095
35	2013	1	-0.6167	87.7024	951.0750	1.4798	1.6464
78	2013	1	1.7833	72.7262	907.4214	1.2714	2.7083
28	2013	1	0.1333	84.4167	939.6667	1.5119	1.6190
39	2013	1	-0.2500	79.5357	910.8321	1.5619	3.2298
24	2013	1	1.2333	74.1905	951.7667	2.0226	1.4440
63	2013	1	0.1667	88.3929	957.6738	1.6000	1.9750
62	2013	1	-0.0167	83.2500	881.2536	1.3071	1.2071
1	2013	1	-0.4667	84.3333	881.6500	1.4214	2.6357
31	2013	2	0.6000	76.8469	851.1306	1.3673	1.0061
79	2013	2	5.3286	75.2857	993.1173	1.0551	0.7480
51	2013	2	3.3571	82.1429	950.9694	1.1929	1.3684
14	2013	2	2.9143	85.7755	905.1857	1.0327	2.1939
67	2013	2	2.8429	79.3061	910.8571	0.7694	2.8357
42	2013	2	1.2571	84.7143	881.6102	0.8265	2.4776
50	2013	2	2.1429	77.6939	910.9612	0.9622	1.6194
43	2013	2	1.2571	84.7143	881.6102	0.8265	2.4776
85	2013	2	2.8857	85.1531	920.2173	0.7949	1.3286
25	2013	2	6.0429	82.7143	990.7439	0.8796	1.0949
69	2013	2	4.5857	73.3163	951.8214	1.4867	1.1949

57	2013	2	2.3571	86.9694	902.8276	0.8959	1.7000
9	2013	2	1.1714	82.2959	859.5867	1.1837	2.5276
72	2013	2	2.5714	76.6327	884.4378	1.1929	2.5296
26	2013	2	2.8857	83.6429	873.0398	1.1163	1.6571
7	2013	2	2.4000	81.9898	864.4847	1.4245	1.7582
83	2013	2	6.7714	76.7041	955.2143	0.5490	0.9255
76	2013	2	3.0429	79.1020	931.4122	1.2969	1.7500
36	2013	2	3.5143	79.3776	939.7071	0.7949	1.5000
81	2013	2	3.3571	82.1429	950.9694	1.1929	1.3684
15	2013	2	3.7000	71.0918	944.4327	1.4531	0.8163
32	2013	2	1.2571	84.7143	881.6102	0.8265	2.4776
73	2013	2	5.7429	78.2653	975.5929	1.3684	0.7704
71	2013	2	3.5143	79.3776	939.7071	0.7949	1.5000
41	2013	2	1.9143	82.0510	881.3194	1.0765	1.1327
10	2013	2	4.2286	77.0000	977.8592	1.2316	1.1551
23	2013	2	-1.1571	77.3265	776.0429	3.9276	2.4112
27	2013	2	2.4000	81.9898	864.4847	1.4245	1.7582
60	2013	2	3.3571	82.1429	950.9694	1.1929	1.3684
53	2013	2	1.1714	82.2959	859.5867	1.1837	2.5276
66	2013	2	2.9143	85.7755	905.1857	1.0327	2.1939
59	2013	2	2.3571	86.9694	902.8276	0.8959	1.7000
61	2013	2	5.7429	78.2653	975.5929	1.3684	0.7704
84	2013	2	5.7429	78.2653	975.5929	1.3684	0.7704
38	2013	2	2.3571	86.9694	902.8276	0.8959	1.7000
87	2013	2	3.3143	73.9184	907.5388	0.8204	2.5520
34	2013	2	2.3571	86.9694	902.8276	0.8959	1.7000
29	2013	2	4.5857	73.3163	951.8214	1.4867	1.1949
5	2013	2	0.5286	83.9286	836.0071	1.9592	1.7061
8	2013	2	1.1714	82.2959	859.5867	1.1837	2.5276
12	2013	2	0.5286	83.9286	836.0071	1.9592	1.7061
13	2013	2	6.7714	76.7041	955.2143	0.5490	0.9255
18	2013	2	4.1714	77.1939	977.8694	1.1357	0.9378
33	2013	2	2.1429	77.6939	910.9612	0.9622	1.6194
56	2013	2	6.0429	82.7143	990.7439	0.8796	1.0949
77	2013	2	3.7000	71.0918	944.4327	1.4531	0.8163
54	2013	2	0.5286	83.9286	836.0071	1.9592	1.7061
21	2013	2	2.1429	77.6939	910.9612	0.9622	1.6194
68	2013	2	5.3286	75.2857	993.1173	1.0551	0.7480
74	2013	2	5.7429	78.2653	975.5929	1.3684	0.7704
88	2013	2	1.2571	84.7143	881.6102	0.8265	2.4776
16	2013	2	3.0429	79.1020	931.4122	1.2969	1.7500
30	2013	2	2.9143	85.7755	905.1857	1.0327	2.1939
6	2013	2	5.3286	75.2857	993.1173	1.0551	0.7480
49	2013	2	4.5857	73.3163	951.8214	1.4867	1.1949
22	2013	2	1.2571	84.7143	881.6102	0.8265	2.4776
45	2013	2	1.2571	81.8469	820.4306	2.2939	1.7051
58	2013	2	4.5857	73.3163	951.8214	1.4867	1.1949
37	2013	2	5.3286	75.2857	993.1173	1.0551	0.7480
17	2013	2	2.8429	79.3061	910.8571	0.7694	2.8357
55	2013	2	2.5714	76.6327	884.4378	1.1929	2.5296

46	2013	2	3.0429	79.1020	931.4122	1.2969	1.7500
86	2013	2	2.3571	77.5816	872.9429	1.1582	1.2092
2	2013	2	2.3571	77.5816	872.9429	1.1582	1.2092
4	2013	2	2.1429	77.6939	910.9612	0.9622	1.6194
47	2013	2	7.4571	72.3673	970.9622	0.8367	0.5898
82	2013	2	1.2571	84.7143	881.6102	0.8265	2.4776
19	2013	2	6.6857	68.0510	972.2276	1.0816	1.2786
20	2013	2	1.1714	82.2959	859.5867	1.1837	2.5276
80	2013	2	1.2571	84.7143	881.6102	0.8265	2.4776
3	2013	2	6.7714	76.7041	955.2143	0.5490	0.9255
52	2013	2	2.8429	79.3061	910.8571	0.7694	2.8357
70	2013	2	2.8857	85.1531	920.2173	0.7949	1.3286
64	2013	2	-1.1571	77.3265	776.0429	3.9276	2.4112
48	2013	2	3.7000	71.0918	944.4327	1.4531	0.8163
65	2013	2	2.8429	79.3061	910.8571	0.7694	2.8357
44	2013	2	2.8857	85.1531	920.2173	0.7949	1.3286
75	2013	2	-1.1571	77.3265	776.0429	3.9276	2.4112
40	2013	2	3.4143	87.4184	957.6898	0.9939	1.6745
11	2013	2	2.5714	76.6327	884.4378	1.1929	2.5296
35	2013	2	3.3571	82.1429	950.9694	1.1929	1.3684
78	2013	2	3.3143	73.9184	907.5388	0.8204	2.5520
28	2013	2	3.5143	79.3776	939.7071	0.7949	1.5000
39	2013	2	2.8429	79.3061	910.8571	0.7694	2.8357
24	2013	2	4.5857	73.3163	951.8214	1.4867	1.1949
63	2013	2	3.4143	87.4184	957.6898	0.9939	1.6745
62	2013	2	1.9143	82.0510	881.3194	1.0765	1.1327
1	2013	2	1.2571	84.7143	881.6102	0.8265	2.4776
31	2013	3	4.6143	82.4490	851.0908	0.9776	0.8888
79	2013	3	7.7286	72.1122	991.7714	2.3714	0.7194
51	2013	3	6.2143	76.1939	950.1133	2.2153	1.1806
14	2013	3	6.3714	86.2653	905.0418	1.3847	1.9041
67	2013	3	6.5000	77.7653	910.5878	0.8449	2.6000
42	2013	3	5.3571	87.0612	881.5551	0.8122	2.2092
50	2013	3	5.5286	81.9388	910.5561	0.7235	1.4847
43	2013	3	5.3571	87.0612	881.5551	0.8122	2.2092
85	2013	3	6.0000	89.3776	919.8653	0.6245	1.0990
25	2013	3	9.6000	85.4184	989.8867	1.0122	0.7816
69	2013	3	7.8714	71.3980	951.0582	1.5745	1.0439
57	2013	3	5.8429	89.8367	902.5990	0.6592	1.3827
9	2013	3	4.8571	88.5510	859.7102	0.5786	2.2480
72	2013	3	6.3571	82.7755	884.4724	0.9929	2.2796
26	2013	3	6.6143	93.0510	873.2265	0.6102	1.3551
7	2013	3	6.2571	90.8367	864.6092	0.7704	1.6143
83	2013	3	10.2000	83.2857	955.0204	0.6867	0.6949
76	2013	3	6.1143	78.4286	930.8980	1.4163	1.5357
36	2013	3	7.5857	75.2041	939.0735	1.3337	1.3622
81	2013	3	6.2143	76.1939	950.1133	2.2153	1.1806
15	2013	3	6.7143	75.2959	943.8827	0.7990	0.5265
32	2013	3	5.3571	87.0612	881.5551	0.8122	2.2092
73	2013	3	8.4571	77.2245	974.6429	1.6051	0.6551

71	2013	3	7.5857	75.2041	939.0735	1.3337	1.3622
41	2013	3	5.5571	85.8469	881.2439	0.8704	1.0571
10	2013	3	7.1857	75.2347	976.7388	1.9490	0.8786
23	2013	3	3.1714	85.2449	776.4929	3.2439	2.3255
27	2013	3	6.2571	90.8367	864.6092	0.7704	1.6143
60	2013	3	6.2143	76.1939	950.1133	2.2153	1.1806
53	2013	3	4.8571	88.5510	859.7102	0.5786	2.2480
66	2013	3	6.3714	86.2653	905.0418	1.3847	1.9041
59	2013	3	5.8429	89.8367	902.5990	0.6592	1.3827
61	2013	3	8.4571	77.2245	974.6429	1.6051	0.6551
84	2013	3	8.4571	77.2245	974.6429	1.6051	0.6551
38	2013	3	5.8429	89.8367	902.5990	0.6592	1.3827
87	2013	3	7.0857	78.0000	907.4439	0.9531	2.2643
34	2013	3	5.8429	89.8367	902.5990	0.6592	1.3827
29	2013	3	7.8714	71.3980	951.0582	1.5745	1.0439
5	2013	3	4.5571	93.7959	836.2082	1.3255	1.6286
8	2013	3	4.8571	88.5510	859.7102	0.5786	2.2480
12	2013	3	4.5571	93.7959	836.2082	1.3255	1.6286
13	2013	3	10.2000	83.2857	955.0204	0.6867	0.6949
18	2013	3	6.8000	75.7653	976.7694	2.0531	0.8224
33	2013	3	5.5286	81.9388	910.5561	0.7235	1.4847
56	2013	3	9.6000	85.4184	989.8867	1.0122	0.7816
77	2013	3	6.7143	75.2959	943.8827	0.7990	0.5265
54	2013	3	4.5571	93.7959	836.2082	1.3255	1.6286
21	2013	3	5.5286	81.9388	910.5561	0.7235	1.4847
68	2013	3	7.7286	72.1122	991.7714	2.3714	0.7194
74	2013	3	8.4571	77.2245	974.6429	1.6051	0.6551
88	2013	3	5.3571	87.0612	881.5551	0.8122	2.2092
16	2013	3	6.1143	78.4286	930.8980	1.4163	1.5357
30	2013	3	6.3714	86.2653	905.0418	1.3847	1.9041
6	2013	3	7.7286	72.1122	991.7714	2.3714	0.7194
49	2013	3	7.8714	71.3980	951.0582	1.5745	1.0439
22	2013	3	5.3571	87.0612	881.5551	0.8122	2.2092
45	2013	3	6.9000	90.7449	820.7673	1.9592	1.4357
58	2013	3	7.8714	71.3980	951.0582	1.5745	1.0439
37	2013	3	7.7286	72.1122	991.7714	2.3714	0.7194
17	2013	3	6.5000	77.7653	910.5878	0.8449	2.6000
55	2013	3	6.3571	82.7755	884.4724	0.9929	2.2796
46	2013	3	6.1143	78.4286	930.8980	1.4163	1.5357
86	2013	3	6.1429	82.8878	872.9469	1.0357	1.0306
2	2013	3	6.1429	82.8878	872.9469	1.0357	1.0306
4	2013	3	5.5286	81.9388	910.5561	0.7235	1.4847
47	2013	3	10.7429	79.8673	970.6602	0.7255	0.5276
82	2013	3	5.3571	87.0612	881.5551	0.8122	2.2092
19	2013	3	9.3143	71.6020	971.7796	1.0786	1.1245
20	2013	3	4.8571	88.5510	859.7102	0.5786	2.2480
80	2013	3	5.3571	87.0612	881.5551	0.8122	2.2092
3	2013	3	10.2000	83.2857	955.0204	0.6867	0.6949
52	2013	3	6.5000	77.7653	910.5878	0.8449	2.6000
70	2013	3	6.0000	89.3776	919.8653	0.6245	1.0990

64	2013	3	3.1714	85.2449	776.4929	3.2439	2.3255
48	2013	3	6.7143	75.2959	943.8827	0.7990	0.5265
65	2013	3	6.5000	77.7653	910.5878	0.8449	2.6000
44	2013	3	6.0000	89.3776	919.8653	0.6245	1.0990
75	2013	3	3.1714	85.2449	776.4929	3.2439	2.3255
40	2013	3	7.5571	85.0612	956.8755	1.5500	1.3724
11	2013	3	6.3571	82.7755	884.4724	0.9929	2.2796
35	2013	3	6.2143	76.1939	950.1133	2.2153	1.1806
78	2013	3	7.0857	78.0000	907.4439	0.9531	2.2643
28	2013	3	7.5857	75.2041	939.0735	1.3337	1.3622
39	2013	3	6.5000	77.7653	910.5878	0.8449	2.6000
24	2013	3	7.8714	71.3980	951.0582	1.5745	1.0439
63	2013	3	7.5571	85.0612	956.8755	1.5500	1.3724
62	2013	3	5.5571	85.8469	881.2439	0.8704	1.0571
1	2013	3	5.3571	87.0612	881.5551	0.8122	2.2092
31	2013	4	4.6571	80.7857	851.2112	1.4796	0.8398
79	2013	4	7.3000	76.6531	990.9551	1.9857	0.7449
51	2013	4	5.3714	80.5918	949.5276	1.7337	1.3602
14	2013	4	6.6857	89.4286	904.9041	1.2337	2.0908
67	2013	4	6.2857	80.3367	910.2796	0.8020	3.0010
42	2013	4	5.6857	85.5612	881.4980	0.8296	2.3265
50	2013	4	6.4000	80.7857	910.1418	0.6245	1.7316
43	2013	4	5.6857	85.5612	881.4980	0.8296	2.3265
85	2013	4	7.1857	87.4286	919.3122	0.5694	1.2990
25	2013	4	9.7714	86.8367	988.8122	0.8173	0.7296
69	2013	4	6.6286	73.5816	950.3551	1.2031	1.1929
57	2013	4	5.9429	89.3265	902.2010	0.5633	1.5347
9	2013	4	5.8571	88.0612	859.8847	0.5071	2.2959
72	2013	4	7.8857	82.3878	884.3980	1.0673	2.3469
26	2013	4	9.5571	91.8776	873.2714	1.2184	1.6490
7	2013	4	8.0000	89.5816	864.7071	0.9337	1.6980
83	2013	4	11.9714	83.5000	954.0969	0.7908	0.5684
76	2013	4	6.1714	79.7143	930.3429	1.2204	1.6418
36	2013	4	6.2000	78.6224	938.4633	1.1592	1.3837
81	2013	4	5.3714	80.5918	949.5276	1.7337	1.3602
15	2013	4	7.4857	77.6939	943.1908	0.8520	0.4653
32	2013	4	5.6857	85.5612	881.4980	0.8296	2.3265
73	2013	4	7.9571	80.0714	973.7908	1.2020	0.6429
71	2013	4	6.2000	78.6224	938.4633	1.1592	1.3837
41	2013	4	5.9571	84.0102	881.0806	0.9102	1.2633
10	2013	4	6.2286	78.9592	975.9306	1.5296	0.9020
23	2013	4	3.9143	77.0510	777.5724	4.5857	2.3857
27	2013	4	8.0000	89.5816	864.7071	0.9337	1.6980
60	2013	4	5.3714	80.5918	949.5276	1.7337	1.3602
53	2013	4	5.8571	88.0612	859.8847	0.5071	2.2959
66	2013	4	6.6857	89.4286	904.9041	1.2337	2.0908
59	2013	4	5.9429	89.3265	902.2010	0.5633	1.5347
61	2013	4	7.9571	80.0714	973.7908	1.2020	0.6429
84	2013	4	7.9571	80.0714	973.7908	1.2020	0.6429
38	2013	4	5.9429	89.3265	902.2010	0.5633	1.5347

87	2013	4	7.7857	76.9388	907.1061	0.7622	2.2827
34	2013	4	5.9429	89.3265	902.2010	0.5633	1.5347
29	2013	4	6.6286	73.5816	950.3551	1.2031	1.1929
5	2013	4	5.7429	89.6633	836.5929	1.6429	1.8816
8	2013	4	5.8571	88.0612	859.8847	0.5071	2.2959
12	2013	4	5.7429	89.6633	836.5929	1.6429	1.8816
13	2013	4	11.9714	83.5000	954.0969	0.7908	0.5684
18	2013	4	6.3857	80.3061	976.1194	1.8204	0.9316
33	2013	4	6.4000	80.7857	910.1418	0.6245	1.7316
56	2013	4	9.7714	86.8367	988.8122	0.8173	0.7296
77	2013	4	7.4857	77.6939	943.1908	0.8520	0.4653
54	2013	4	5.7429	89.6633	836.5929	1.6429	1.8816
21	2013	4	6.4000	80.7857	910.1418	0.6245	1.7316
68	2013	4	7.3000	76.6531	990.9551	1.9857	0.7449
74	2013	4	7.9571	80.0714	973.7908	1.2020	0.6429
88	2013	4	5.6857	85.5612	881.4980	0.8296	2.3265
16	2013	4	6.1714	79.7143	930.3429	1.2204	1.6418
30	2013	4	6.6857	89.4286	904.9041	1.2337	2.0908
6	2013	4	7.3000	76.6531	990.9551	1.9857	0.7449
49	2013	4	6.6286	73.5816	950.3551	1.2031	1.1929
22	2013	4	5.6857	85.5612	881.4980	0.8296	2.3265
45	2013	4	8.9143	76.6837	821.3480	4.2306	2.0255
58	2013	4	6.6286	73.5816	950.3551	1.2031	1.1929
37	2013	4	7.3000	76.6531	990.9551	1.9857	0.7449
17	2013	4	6.2857	80.3367	910.2796	0.8020	3.0010
55	2013	4	7.8857	82.3878	884.3980	1.0673	2.3469
46	2013	4	6.1714	79.7143	930.3429	1.2204	1.6418
86	2013	4	6.3714	81.8980	872.9439	0.9571	1.2327
2	2013	4	6.3714	81.8980	872.9439	0.9571	1.2327
4	2013	4	6.4000	80.7857	910.1418	0.6245	1.7316
47	2013	4	12.6286	80.1531	969.6000	0.8020	0.5510
82	2013	4	5.6857	85.5612	881.4980	0.8296	2.3265
19	2013	4	10.5000	76.9388	971.0551	0.9418	0.9520
20	2013	4	5.8571	88.0612	859.8847	0.5071	2.2959
80	2013	4	5.6857	85.5612	881.4980	0.8296	2.3265
3	2013	4	11.9714	83.5000	954.0969	0.7908	0.5684
52	2013	4	6.2857	80.3367	910.2796	0.8020	3.0010
70	2013	4	7.1857	87.4286	919.3122	0.5694	1.2990
64	2013	4	3.9143	77.0510	777.5724	4.5857	2.3857
48	2013	4	7.4857	77.6939	943.1908	0.8520	0.4653
65	2013	4	6.2857	80.3367	910.2796	0.8020	3.0010
44	2013	4	7.1857	87.4286	919.3122	0.5694	1.2990
75	2013	4	3.9143	77.0510	777.5724	4.5857	2.3857
40	2013	4	5.8000	87.5408	956.2041	1.3653	1.7041
11	2013	4	7.8857	82.3878	884.3980	1.0673	2.3469
35	2013	4	5.3714	80.5918	949.5276	1.7337	1.3602
78	2013	4	7.7857	76.9388	907.1061	0.7622	2.2827
28	2013	4	6.2000	78.6224	938.4633	1.1592	1.3837
39	2013	4	6.2857	80.3367	910.2796	0.8020	3.0010
24	2013	4	6.6286	73.5816	950.3551	1.2031	1.1929

63	2013	4	5.8000	87.5408	956.2041	1.3653	1.7041
62	2013	4	5.9571	84.0102	881.0806	0.9102	1.2633
1	2013	4	5.6857	85.5612	881.4980	0.8296	2.3265
31	2013	5	11.0571	73.8878	850.7296	2.4010	0.9367
79	2013	5	10.0714	80.3571	989.8224	1.3867	0.6990
51	2013	5	11.1000	82.1735	948.5184	1.7051	1.3673
14	2013	5	13.7714	88.1735	904.4684	1.4684	2.4684
67	2013	5	14.6571	77.6735	909.5867	1.1214	3.5347
42	2013	5	13.5286	79.0306	880.9041	1.8663	2.2592
50	2013	5	11.5571	75.8776	909.1051	0.9480	1.8133
43	2013	5	13.5286	79.0306	880.9041	1.8663	2.2592
85	2013	5	13.5857	79.6122	918.0398	1.3469	1.5153
25	2013	5	15.7429	84.8469	987.5459	1.2551	0.8235
69	2013	5	13.3857	73.6531	949.2500	1.7429	1.1827
57	2013	5	13.8000	82.8571	901.2439	1.1286	1.8102
9	2013	5	12.8000	81.8878	859.5245	1.5367	2.5429
72	2013	5	13.9571	76.0000	883.8622	2.2929	2.5653
26	2013	5	14.2000	82.7449	872.8888	2.9602	2.0000
7	2013	5	13.7714	80.6531	864.3908	2.3265	1.8622
83	2013	5	17.6286	76.2143	952.9041	1.8378	0.7929
76	2013	5	11.0000	79.3673	929.3776	1.5245	1.7316
36	2013	5	14.7714	76.9694	937.4520	1.9592	1.4378
81	2013	5	11.1000	82.1735	948.5184	1.7051	1.3673
15	2013	5	10.5571	76.6122	942.1133	1.2837	0.5643
32	2013	5	13.5286	79.0306	880.9041	1.8663	2.2592
73	2013	5	11.8714	82.1531	972.6612	1.3418	0.7112
71	2013	5	14.7714	76.9694	937.4520	1.9592	1.4378
41	2013	5	13.0714	79.1633	880.3378	1.1857	1.3367
10	2013	5	12.4571	78.5612	974.7306	1.2520	1.0061
23	2013	5	9.4429	65.1327	777.8173	6.2847	2.4500
27	2013	5	13.7714	80.6531	864.3908	2.3265	1.8622
60	2013	5	11.1000	82.1735	948.5184	1.7051	1.3673
53	2013	5	12.8000	81.8878	859.5245	1.5367	2.5429
66	2013	5	13.7714	88.1735	904.4684	1.4684	2.4684
59	2013	5	13.8000	82.8571	901.2439	1.1286	1.8102
61	2013	5	11.8714	82.1531	972.6612	1.3418	0.7112
84	2013	5	11.8714	82.1531	972.6612	1.3418	0.7112
38	2013	5	13.8000	82.8571	901.2439	1.1286	1.8102
87	2013	5	14.9429	71.0000	906.2898	1.0296	2.2837
34	2013	5	13.8000	82.8571	901.2439	1.1286	1.8102
29	2013	5	13.3857	73.6531	949.2500	1.7429	1.1827
5	2013	5	12.0571	78.4388	836.4724	3.2755	2.1704
8	2013	5	12.8000	81.8878	859.5245	1.5367	2.5429
12	2013	5	12.0571	78.4388	836.4724	3.2755	2.1704
13	2013	5	17.6286	76.2143	952.9041	1.8378	0.7929
18	2013	5	8.7286	83.3673	975.0990	1.4520	0.8684
33	2013	5	11.5571	75.8776	909.1051	0.9480	1.8133
56	2013	5	15.7429	84.8469	987.5459	1.2551	0.8235
77	2013	5	10.5571	76.6122	942.1133	1.2837	0.5643
54	2013	5	12.0571	78.4388	836.4724	3.2755	2.1704

21	2013	5	11.5571	75.8776	909.1051	0.9480	1.8133
68	2013	5	10.0714	80.3571	989.8224	1.3867	0.6990
74	2013	5	11.8714	82.1531	972.6612	1.3418	0.7112
88	2013	5	13.5286	79.0306	880.9041	1.8663	2.2592
16	2013	5	11.0000	79.3673	929.3776	1.5245	1.7316
30	2013	5	13.7714	88.1735	904.4684	1.4684	2.4684
6	2013	5	10.0714	80.3571	989.8224	1.3867	0.6990
49	2013	5	13.3857	73.6531	949.2500	1.7429	1.1827
22	2013	5	13.5286	79.0306	880.9041	1.8663	2.2592
45	2013	5	13.0000	65.4694	821.3908	5.8408	2.4480
58	2013	5	13.3857	73.6531	949.2500	1.7429	1.1827
37	2013	5	10.0714	80.3571	989.8224	1.3867	0.6990
17	2013	5	14.6571	77.6735	909.5867	1.1214	3.5347
55	2013	5	13.9571	76.0000	883.8622	2.2929	2.5653
46	2013	5	11.0000	79.3673	929.3776	1.5245	1.7316
86	2013	5	13.5143	76.1429	872.3561	1.3755	1.3184
2	2013	5	13.5143	76.1429	872.3561	1.3755	1.3184
4	2013	5	11.5571	75.8776	909.1051	0.9480	1.8133
47	2013	5	17.4000	73.2653	968.2786	1.6878	0.6286
82	2013	5	13.5286	79.0306	880.9041	1.8663	2.2592
19	2013	5	14.9143	77.2347	970.1224	1.3020	0.9020
20	2013	5	12.8000	81.8878	859.5245	1.5367	2.5429
80	2013	5	13.5286	79.0306	880.9041	1.8663	2.2592
3	2013	5	17.6286	76.2143	952.9041	1.8378	0.7929
52	2013	5	14.6571	77.6735	909.5867	1.1214	3.5347
70	2013	5	13.5857	79.6122	918.0398	1.3469	1.5153
64	2013	5	9.4429	65.1327	777.8173	6.2847	2.4500
48	2013	5	10.5571	76.6122	942.1133	1.2837	0.5643
65	2013	5	14.6571	77.6735	909.5867	1.1214	3.5347
44	2013	5	13.5857	79.6122	918.0398	1.3469	1.5153
75	2013	5	9.4429	65.1327	777.8173	6.2847	2.4500
40	2013	5	14.2857	87.2245	955.2378	1.4449	1.9582
11	2013	5	13.9571	76.0000	883.8622	2.2929	2.5653
35	2013	5	11.1000	82.1735	948.5184	1.7051	1.3673
78	2013	5	14.9429	71.0000	906.2898	1.0296	2.2837
28	2013	5	14.7714	76.9694	937.4520	1.9592	1.4378
39	2013	5	14.6571	77.6735	909.5867	1.1214	3.5347
24	2013	5	13.3857	73.6531	949.2500	1.7429	1.1827
63	2013	5	14.2857	87.2245	955.2378	1.4449	1.9582
62	2013	5	13.0714	79.1633	880.3378	1.1857	1.3367
1	2013	5	13.5286	79.0306	880.9041	1.8663	2.2592
31	2013	6	8.7429	65.6327	848.8500	4.1469	1.2398
79	2013	6	6.1714	84.6837	987.4969	1.2214	0.7898
51	2013	6	4.6857	84.4490	946.1776	2.1735	1.6184
14	2013	6	10.0000	84.4490	902.7010	2.9102	3.2633
67	2013	6	8.7286	72.2653	907.4255	2.5816	4.4673
42	2013	6	9.1286	71.7449	878.9531	4.5327	2.4663
50	2013	6	8.7000	73.8163	906.5112	2.5367	2.0235
43	2013	6	9.1286	71.7449	878.9531	4.5327	2.4663
85	2013	6	9.2429	75.7857	915.1735	2.9122	1.8673

25	2013	6	12.3714	80.6122	984.6112	2.8867	1.3286
69	2013	6	9.1571	69.9898	946.4041	3.2235	1.6071
57	2013	6	9.2571	75.1327	898.7092	3.1490	2.1867
9	2013	6	9.9571	72.0714	857.9245	4.3388	2.9531
72	2013	6	11.6429	68.1429	882.0939	5.0031	2.6898
26	2013	6	13.6000	66.9286	871.4735	5.5480	2.2347
7	2013	6	12.0857	67.2857	862.9816	5.4000	2.3520
83	2013	6	15.6000	68.8061	950.4439	4.7357	1.0633
76	2013	6	6.8143	79.8265	926.7653	2.7000	2.2204
36	2013	6	8.1429	73.4592	934.9082	3.7265	1.8745
81	2013	6	4.6857	84.4490	946.1776	2.1735	1.6184
15	2013	6	8.9857	75.4796	939.3571	2.3847	0.9245
32	2013	6	9.1286	71.7449	878.9531	4.5327	2.4663
73	2013	6	8.7857	81.1531	969.8643	2.2102	0.8561
71	2013	6	8.1429	73.4592	934.9082	3.7265	1.8745
41	2013	6	9.4143	71.2959	878.1378	3.0837	1.6612
10	2013	6	5.8143	78.6122	972.2163	1.6439	1.3643
23	2013	6	8.2143	54.2959	777.1908	7.3653	2.7102
27	2013	6	12.0857	67.2857	862.9816	5.4000	2.3520
60	2013	6	4.6857	84.4490	946.1776	2.1735	1.6184
53	2013	6	9.9571	72.0714	857.9245	4.3388	2.9531
66	2013	6	10.0000	84.4490	902.7010	2.9102	3.2633
59	2013	6	9.2571	75.1327	898.7092	3.1490	2.1867
61	2013	6	8.7857	81.1531	969.8643	2.2102	0.8561
84	2013	6	8.7857	81.1531	969.8643	2.2102	0.8561
38	2013	6	9.2571	75.1327	898.7092	3.1490	2.1867
87	2013	6	11.4143	64.6224	904.0857	3.5847	2.5255
34	2013	6	9.2571	75.1327	898.7092	3.1490	2.1867
29	2013	6	9.1571	69.9898	946.4041	3.2235	1.6071
5	2013	6	10.3429	62.3265	835.3316	6.0908	2.8398
8	2013	6	9.9571	72.0714	857.9245	4.3388	2.9531
12	2013	6	10.3429	62.3265	835.3316	6.0908	2.8398
13	2013	6	15.6000	68.8061	950.4439	4.7357	1.0633
18	2013	6	5.8571	86.7653	972.8786	1.0276	1.0092
33	2013	6	8.7000	73.8163	906.5112	2.5367	2.0235
56	2013	6	12.3714	80.6122	984.6112	2.8867	1.3286
77	2013	6	8.9857	75.4796	939.3571	2.3847	0.9245
54	2013	6	10.3429	62.3265	835.3316	6.0908	2.8398
21	2013	6	8.7000	73.8163	906.5112	2.5367	2.0235
68	2013	6	6.1714	84.6837	987.4969	1.2214	0.7898
74	2013	6	8.7857	81.1531	969.8643	2.2102	0.8561
88	2013	6	9.1286	71.7449	878.9531	4.5327	2.4663
16	2013	6	6.8143	79.8265	926.7653	2.7000	2.2204
30	2013	6	10.0000	84.4490	902.7010	2.9102	3.2633
6	2013	6	6.1714	84.6837	987.4969	1.2214	0.7898
49	2013	6	9.1571	69.9898	946.4041	3.2235	1.6071
22	2013	6	9.1286	71.7449	878.9531	4.5327	2.4663
45	2013	6	12.7143	56.2143	820.5918	7.0582	2.8867
58	2013	6	9.1571	69.9898	946.4041	3.2235	1.6071
37	2013	6	6.1714	84.6837	987.4969	1.2214	0.7898

17	2013	6	8.7286	72.2653	907.4255	2.5816	4.4673
55	2013	6	11.6429	68.1429	882.0939	5.0031	2.6898
46	2013	6	6.8143	79.8265	926.7653	2.7000	2.2204
86	2013	6	10.1571	66.6837	870.3214	3.5500	1.4929
2	2013	6	10.1571	66.6837	870.3214	3.5500	1.4929
4	2013	6	8.7000	73.8163	906.5112	2.5367	2.0235
47	2013	6	15.7714	70.0204	965.5367	3.9153	0.7806
82	2013	6	9.1286	71.7449	878.9531	4.5327	2.4663
19	2013	6	13.5143	76.9796	967.5745	2.4561	1.2633
20	2013	6	9.9571	72.0714	857.9245	4.3388	2.9531
80	2013	6	9.1286	71.7449	878.9531	4.5327	2.4663
3	2013	6	15.6000	68.8061	950.4439	4.7357	1.0633
52	2013	6	8.7286	72.2653	907.4255	2.5816	4.4673
70	2013	6	9.2429	75.7857	915.1735	2.9122	1.8673
64	2013	6	8.2143	54.2959	777.1908	7.3653	2.7102
48	2013	6	8.9857	75.4796	939.3571	2.3847	0.9245
65	2013	6	8.7286	72.2653	907.4255	2.5816	4.4673
44	2013	6	9.2429	75.7857	915.1735	2.9122	1.8673
75	2013	6	8.2143	54.2959	777.1908	7.3653	2.7102
40	2013	6	7.4000	85.3571	952.8898	2.3929	2.3296
11	2013	6	11.6429	68.1429	882.0939	5.0031	2.6898
35	2013	6	4.6857	84.4490	946.1776	2.1735	1.6184
78	2013	6	11.4143	64.6224	904.0857	3.5847	2.5255
28	2013	6	8.1429	73.4592	934.9082	3.7265	1.8745
39	2013	6	8.7286	72.2653	907.4255	2.5816	4.4673
24	2013	6	9.1571	69.9898	946.4041	3.2235	1.6071
63	2013	6	7.4000	85.3571	952.8898	2.3929	2.3296
62	2013	6	9.4143	71.2959	878.1378	3.0837	1.6612
1	2013	6	9.1286	71.7449	878.9531	4.5327	2.4663
31	2013	7	5.0571	72.3571	848.3724	3.2429	1.2408
79	2013	7	6.7857	84.5918	988.5378	0.6418	0.9847
51	2013	7	4.4714	89.2857	946.8439	0.8643	1.7480
14	2013	7	5.6429	89.6429	902.2561	1.9459	3.0684
67	2013	7	5.1857	80.9898	907.3531	1.8000	4.0929
42	2013	7	4.8000	82.1735	878.5959	3.2184	2.4000
50	2013	7	5.9000	79.3265	906.5796	1.9245	2.1469
43	2013	7	4.8000	82.1735	878.5959	3.2184	2.4000
85	2013	7	6.2714	84.4898	915.4143	1.9184	1.9051
25	2013	7	8.6571	81.4490	985.2367	2.0929	1.6980
69	2013	7	6.3429	72.4898	946.9888	1.7990	1.8612
57	2013	7	5.3429	84.0510	898.7510	2.3378	2.1194
9	2013	7	5.7857	79.5000	857.2673	3.3000	2.9663
72	2013	7	7.1143	75.3673	881.4929	3.5204	2.8020
26	2013	7	9.7286	72.7449	870.5653	4.7112	2.1827
7	2013	7	10.0714	73.0510	862.0847	5.3265	2.5245
83	2013	7	12.4143	70.0816	950.0133	3.7643	1.1214
76	2013	7	5.2286	84.7245	927.1173	1.5765	2.4582
36	2013	7	5.3000	79.4796	935.3500	2.1224	2.0551
81	2013	7	4.4714	89.2857	946.8439	0.8643	1.7480
15	2013	7	7.3857	73.7449	939.6163	1.6918	1.0990

32	2013	7	4.8000	82.1735	878.5959	3.2184	2.4000
73	2013	7	7.6143	80.0000	970.5490	1.7235	0.8939
71	2013	7	5.3000	79.4796	935.3500	2.1224	2.0551
41	2013	7	5.1429	79.4286	877.9143	2.5888	1.7112
10	2013	7	5.6143	83.0102	973.2194	0.6439	1.5724
23	2013	7	8.9571	56.0612	776.3673	7.2867	3.1133
27	2013	7	10.0714	73.0510	862.0847	5.3265	2.5245
60	2013	7	4.4714	89.2857	946.8439	0.8643	1.7480
53	2013	7	5.7857	79.5000	857.2673	3.3000	2.9663
66	2013	7	5.6429	89.6429	902.2561	1.9459	3.0684
59	2013	7	5.3429	84.0510	898.7510	2.3378	2.1194
61	2013	7	7.6143	80.0000	970.5490	1.7235	0.8939
84	2013	7	7.6143	80.0000	970.5490	1.7235	0.8939
38	2013	7	5.3429	84.0510	898.7510	2.3378	2.1194
87	2013	7	7.1857	71.4286	903.8082	2.9041	2.8071
34	2013	7	5.3429	84.0510	898.7510	2.3378	2.1194
29	2013	7	6.3429	72.4898	946.9888	1.7990	1.8612
5	2013	7	9.1429	70.2347	834.4459	5.4561	2.8561
8	2013	7	5.7857	79.5000	857.2673	3.3000	2.9663
12	2013	7	9.1429	70.2347	834.4459	5.4561	2.8561
13	2013	7	12.4143	70.0816	950.0133	3.7643	1.1214
18	2013	7	6.2429	86.2857	973.7041	0.4031	1.3265
33	2013	7	5.9000	79.3265	906.5796	1.9245	2.1469
56	2013	7	8.6571	81.4490	985.2367	2.0929	1.6980
77	2013	7	7.3857	73.7449	939.6163	1.6918	1.0990
54	2013	7	9.1429	70.2347	834.4459	5.4561	2.8561
21	2013	7	5.9000	79.3265	906.5796	1.9245	2.1469
68	2013	7	6.7857	84.5918	988.5378	0.6418	0.9847
74	2013	7	7.6143	80.0000	970.5490	1.7235	0.8939
88	2013	7	4.8000	82.1735	878.5959	3.2184	2.4000
16	2013	7	5.2286	84.7245	927.1173	1.5765	2.4582
30	2013	7	5.6429	89.6429	902.2561	1.9459	3.0684
6	2013	7	6.7857	84.5918	988.5378	0.6418	0.9847
49	2013	7	6.3429	72.4898	946.9888	1.7990	1.8612
22	2013	7	4.8000	82.1735	878.5959	3.2184	2.4000
45	2013	7	14.0000	55.6633	819.5582	6.9245	3.1867
58	2013	7	6.3429	72.4898	946.9888	1.7990	1.8612
37	2013	7	6.7857	84.5918	988.5378	0.6418	0.9847
17	2013	7	5.1857	80.9898	907.3531	1.8000	4.0929
55	2013	7	7.1143	75.3673	881.4929	3.5204	2.8020
46	2013	7	5.2286	84.7245	927.1173	1.5765	2.4582
86	2013	7	6.9286	73.2857	869.8337	3.0816	1.6051
2	2013	7	6.9286	73.2857	869.8337	3.0816	1.6051
4	2013	7	5.9000	79.3265	906.5796	1.9245	2.1469
47	2013	7	11.4429	73.0102	965.4786	2.9929	0.8520
82	2013	7	4.8000	82.1735	878.5959	3.2184	2.4000
19	2013	7	9.0571	77.1939	967.5418	1.5908	1.5102
20	2013	7	5.7857	79.5000	857.2673	3.3000	2.9663
80	2013	7	4.8000	82.1735	878.5959	3.2184	2.4000
3	2013	7	12.4143	70.0816	950.0133	3.7643	1.1214

52	2013	7	5.1857	80.9898	907.3531	1.8000	4.0929
70	2013	7	6.2714	84.4898	915.4143	1.9184	1.9051
64	2013	7	8.9571	56.0612	776.3673	7.2867	3.1133
48	2013	7	7.3857	73.7449	939.6163	1.6918	1.0990
65	2013	7	5.1857	80.9898	907.3531	1.8000	4.0929
44	2013	7	6.2714	84.4898	915.4143	1.9184	1.9051
75	2013	7	8.9571	56.0612	776.3673	7.2867	3.1133
40	2013	7	5.3286	90.8878	953.4235	1.3745	2.3990
11	2013	7	7.1143	75.3673	881.4929	3.5204	2.8020
35	2013	7	4.4714	89.2857	946.8439	0.8643	1.7480
78	2013	7	7.1857	71.4286	903.8082	2.9041	2.8071
28	2013	7	5.3000	79.4796	935.3500	2.1224	2.0551
39	2013	7	5.1857	80.9898	907.3531	1.8000	4.0929
24	2013	7	6.3429	72.4898	946.9888	1.7990	1.8612
63	2013	7	5.3286	90.8878	953.4235	1.3745	2.3990
62	2013	7	5.1429	79.4286	877.9143	2.5888	1.7112
1	2013	7	4.8000	82.1735	878.5959	3.2184	2.4000
31	2013	8	5.3429	83.1735	848.9276	1.7837	0.9908
79	2013	8	7.9571	82.2653	989.5633	0.9663	0.9122
51	2013	8	5.7429	88.3061	947.9194	0.5112	1.2173
14	2013	8	7.6143	94.3776	902.6286	0.4878	2.4255
67	2013	8	7.0000	87.8061	908.1224	0.3327	3.4153
42	2013	8	6.7143	91.9184	879.1469	0.7622	1.8082
50	2013	8	6.8429	82.3776	907.7878	0.4480	1.8398
43	2013	8	6.7143	91.9184	879.1469	0.7622	1.8082
85	2013	8	7.2571	90.7449	916.8796	0.3714	1.4796
25	2013	8	10.1000	86.0714	986.6571	0.7010	1.3194
69	2013	8	8.0714	76.3980	948.4745	0.2357	1.4122
57	2013	8	6.0143	93.9694	899.9245	0.3224	1.7939
9	2013	8	7.5857	88.7653	857.4888	1.2663	2.7255
72	2013	8	9.1143	84.0816	881.8092	1.5745	2.7163
26	2013	8	11.9714	83.8163	870.4969	3.0796	2.2122
7	2013	8	10.9143	81.8673	862.0316	3.8082	2.5092
83	2013	8	13.9286	73.7551	950.7153	1.4510	1.0245
76	2013	8	6.6286	84.7755	928.4061	0.3796	2.0908
36	2013	8	6.6429	84.8265	936.5429	0.2592	1.7388
81	2013	8	5.7429	88.3061	947.9194	0.5112	1.2173
15	2013	8	8.4429	71.9694	940.9776	0.6245	0.8765
32	2013	8	6.7143	91.9184	879.1469	0.7622	1.8082
73	2013	8	9.5429	80.4490	971.9398	1.0959	0.8500
71	2013	8	6.6429	84.8265	936.5429	0.2592	1.7388
41	2013	8	6.2429	90.7347	878.7418	1.0571	1.3480
10	2013	8	6.4000	83.9286	974.4673	0.3673	1.3704
23	2013	8	8.3429	62.0816	776.0082	6.6694	3.3092
27	2013	8	10.9143	81.8673	862.0316	3.8082	2.5092
60	2013	8	5.7429	88.3061	947.9194	0.5112	1.2173
53	2013	8	7.5857	88.7653	857.4888	1.2663	2.7255
66	2013	8	7.6143	94.3776	902.6286	0.4878	2.4255
59	2013	8	6.0143	93.9694	899.9245	0.3224	1.7939
61	2013	8	9.5429	80.4490	971.9398	1.0959	0.8500

84	2013	8	9.5429	80.4490	971.9398	1.0959	0.8500
38	2013	8	6.0143	93.9694	899.9245	0.3224	1.7939
87	2013	8	9.1714	79.5612	904.5612	0.7418	2.6816
34	2013	8	6.0143	93.9694	899.9245	0.3224	1.7939
29	2013	8	8.0714	76.3980	948.4745	0.2357	1.4122
5	2013	8	8.8286	79.1837	834.3020	3.6520	2.8735
8	2013	8	7.5857	88.7653	857.4888	1.2663	2.7255
12	2013	8	8.8286	79.1837	834.3020	3.6520	2.8735
13	2013	8	13.9286	73.7551	950.7153	1.4510	1.0245
18	2013	8	7.7143	81.5816	974.6316	1.0133	1.2735
33	2013	8	6.8429	82.3776	907.7878	0.4480	1.8398
56	2013	8	10.1000	86.0714	986.6571	0.7010	1.3194
77	2013	8	8.4429	71.9694	940.9776	0.6245	0.8765
54	2013	8	8.8286	79.1837	834.3020	3.6520	2.8735
21	2013	8	6.8429	82.3776	907.7878	0.4480	1.8398
68	2013	8	7.9571	82.2653	989.5633	0.9663	0.9122
74	2013	8	9.5429	80.4490	971.9398	1.0959	0.8500
88	2013	8	6.7143	91.9184	879.1469	0.7622	1.8082
16	2013	8	6.6286	84.7755	928.4061	0.3796	2.0908
30	2013	8	7.6143	94.3776	902.6286	0.4878	2.4255
6	2013	8	7.9571	82.2653	989.5633	0.9663	0.9122
49	2013	8	8.0714	76.3980	948.4745	0.2357	1.4122
22	2013	8	6.7143	91.9184	879.1469	0.7622	1.8082
45	2013	8	12.1143	63.5918	819.2663	5.6459	3.3245
58	2013	8	8.0714	76.3980	948.4745	0.2357	1.4122
37	2013	8	7.9571	82.2653	989.5633	0.9663	0.9122
17	2013	8	7.0000	87.8061	908.1224	0.3327	3.4153
55	2013	8	9.1143	84.0816	881.8092	1.5745	2.7163
46	2013	8	6.6286	84.7755	928.4061	0.3796	2.0908
86	2013	8	8.0286	82.7857	870.3959	1.9469	1.4969
2	2013	8	8.0286	82.7857	870.3959	1.9469	1.4969
4	2013	8	6.8429	82.3776	907.7878	0.4480	1.8398
47	2013	8	13.4143	77.5816	966.5878	0.8837	0.6561
82	2013	8	6.7143	91.9184	879.1469	0.7622	1.8082
19	2013	8	10.7286	79.3776	968.5480	0.4510	1.2418
20	2013	8	7.5857	88.7653	857.4888	1.2663	2.7255
80	2013	8	6.7143	91.9184	879.1469	0.7622	1.8082
3	2013	8	13.9286	73.7551	950.7153	1.4510	1.0245
52	2013	8	7.0000	87.8061	908.1224	0.3327	3.4153
70	2013	8	7.2571	90.7449	916.8796	0.3714	1.4796
64	2013	8	8.3429	62.0816	776.0082	6.6694	3.3092
48	2013	8	8.4429	71.9694	940.9776	0.6245	0.8765
65	2013	8	7.0000	87.8061	908.1224	0.3327	3.4153
44	2013	8	7.2571	90.7449	916.8796	0.3714	1.4796
75	2013	8	8.3429	62.0816	776.0082	6.6694	3.3092
40	2013	8	5.9571	94.9694	954.4316	0.6806	1.9388
11	2013	8	9.1143	84.0816	881.8092	1.5745	2.7163
35	2013	8	5.7429	88.3061	947.9194	0.5112	1.2173
78	2013	8	9.1714	79.5612	904.5612	0.7418	2.6816
28	2013	8	6.6429	84.8265	936.5429	0.2592	1.7388

39	2013	8	7.0000	87.8061	908.1224	0.3327	3.4153
24	2013	8	8.0714	76.3980	948.4745	0.2357	1.4122
63	2013	8	5.9571	94.9694	954.4316	0.6806	1.9388
62	2013	8	6.2429	90.7347	878.7418	1.0571	1.3480
1	2013	8	6.7143	91.9184	879.1469	0.7622	1.8082
31	2013	9	9.5571	82.0918	849.2459	2.3918	0.9286
79	2013	9	10.7857	81.7245	988.2214	1.0071	0.7980
51	2013	9	9.6857	84.8469	947.0122	1.2490	1.2122
14	2013	9	11.5571	94.1429	902.4510	0.8429	2.5143
67	2013	9	10.7143	86.1327	907.7643	0.6694	3.4633
42	2013	9	10.6571	89.7755	879.0704	1.0612	1.8194
50	2013	9	10.0286	83.2449	907.5388	0.4061	1.5622
43	2013	9	10.6571	89.7755	879.0704	1.0612	1.8194
85	2013	9	10.5143	90.6837	916.5786	0.4398	1.3816
25	2013	9	13.8286	86.7857	985.4990	1.2633	1.0469
69	2013	9	11.7714	75.8265	947.5765	0.6531	1.1969
57	2013	9	10.0714	93.2143	899.6847	0.4582	1.6571
9	2013	9	11.9286	84.5408	857.6316	2.3449	2.7796
72	2013	9	13.9714	79.5408	881.7378	2.9582	2.7265
26	2013	9	16.7143	76.6224	870.5163	4.7612	2.4745
7	2013	9	15.2286	76.7551	862.1878	4.5398	2.5500
83	2013	9	19.6571	71.0612	949.9990	3.3612	1.0378
76	2013	9	9.8143	84.1531	927.7735	0.4102	1.7408
36	2013	9	11.0571	83.5204	935.7735	0.7480	1.5388
81	2013	9	9.6857	84.8469	947.0122	1.2490	1.2122
15	2013	9	11.3286	73.3367	940.3520	0.4602	0.6735
32	2013	9	10.6571	89.7755	879.0704	1.0612	1.8194
73	2013	9	12.1857	80.2551	970.7939	0.9255	0.8082
71	2013	9	11.0571	83.5204	935.7735	0.7480	1.5388
41	2013	9	10.4429	88.5816	878.7194	1.5204	1.2827
10	2013	9	10.3286	82.2959	973.2724	1.1235	1.2102
23	2013	9	9.9000	60.6429	776.5980	7.4490	3.0010
27	2013	9	15.2286	76.7551	862.1878	4.5398	2.5500
60	2013	9	9.6857	84.8469	947.0122	1.2490	1.2122
53	2013	9	11.9286	84.5408	857.6316	2.3449	2.7796
66	2013	9	11.5571	94.1429	902.4510	0.8429	2.5143
59	2013	9	10.0714	93.2143	899.6847	0.4582	1.6571
61	2013	9	12.1857	80.2551	970.7939	0.9255	0.8082
84	2013	9	12.1857	80.2551	970.7939	0.9255	0.8082
38	2013	9	10.0714	93.2143	899.6847	0.4582	1.6571
87	2013	9	13.5714	76.8469	904.2520	1.1541	2.5510
34	2013	9	10.0714	93.2143	899.6847	0.4582	1.6571
29	2013	9	11.7714	75.8265	947.5765	0.6531	1.1969
5	2013	9	12.4143	74.9796	834.6245	4.3673	2.8755
8	2013	9	11.9286	84.5408	857.6316	2.3449	2.7796
12	2013	9	12.4143	74.9796	834.6245	4.3673	2.8755
13	2013	9	19.6571	71.0612	949.9990	3.3612	1.0378
18	2013	9	10.3143	80.9184	973.4551	1.2490	1.0337
33	2013	9	10.0286	83.2449	907.5388	0.4061	1.5622
56	2013	9	13.8286	86.7857	985.4990	1.2633	1.0469

77	2013	9	11.3286	73.3367	940.3520	0.4602	0.6735
54	2013	9	12.4143	74.9796	834.6245	4.3673	2.8755
21	2013	9	10.0286	83.2449	907.5388	0.4061	1.5622
68	2013	9	10.7857	81.7245	988.2214	1.0071	0.7980
74	2013	9	12.1857	80.2551	970.7939	0.9255	0.8082
88	2013	9	10.6571	89.7755	879.0704	1.0612	1.8194
16	2013	9	9.8143	84.1531	927.7735	0.4102	1.7408
30	2013	9	11.5571	94.1429	902.4510	0.8429	2.5143
6	2013	9	10.7857	81.7245	988.2214	1.0071	0.7980
49	2013	9	11.7714	75.8265	947.5765	0.6531	1.1969
22	2013	9	10.6571	89.7755	879.0704	1.0612	1.8194
45	2013	9	14.5429	63.5816	819.7102	6.5041	3.1041
58	2013	9	11.7714	75.8265	947.5765	0.6531	1.1969
37	2013	9	10.7857	81.7245	988.2214	1.0071	0.7980
17	2013	9	10.7143	86.1327	907.7643	0.6694	3.4633
55	2013	9	13.9714	79.5408	881.7378	2.9582	2.7265
46	2013	9	9.8143	84.1531	927.7735	0.4102	1.7408
86	2013	9	12.3571	79.7755	870.4888	2.4633	1.3612
2	2013	9	12.3571	79.7755	870.4888	2.4633	1.3612
4	2013	9	10.0286	83.2449	907.5388	0.4061	1.5622
47	2013	9	18.7286	74.6429	965.6673	1.6173	0.6847
82	2013	9	10.6571	89.7755	879.0704	1.0612	1.8194
19	2013	9	14.8571	80.0612	967.7173	1.0684	1.1541
20	2013	9	11.9286	84.5408	857.6316	2.3449	2.7796
80	2013	9	10.6571	89.7755	879.0704	1.0612	1.8194
3	2013	9	19.6571	71.0612	949.9990	3.3612	1.0378
52	2013	9	10.7143	86.1327	907.7643	0.6694	3.4633
70	2013	9	10.5143	90.6837	916.5786	0.4398	1.3816
64	2013	9	9.9000	60.6429	776.5980	7.4490	3.0010
48	2013	9	11.3286	73.3367	940.3520	0.4602	0.6735
65	2013	9	10.7143	86.1327	907.7643	0.6694	3.4633
44	2013	9	10.5143	90.6837	916.5786	0.4398	1.3816
75	2013	9	9.9000	60.6429	776.5980	7.4490	3.0010
40	2013	9	10.5857	92.7347	953.5133	1.3184	1.7929
11	2013	9	13.9714	79.5408	881.7378	2.9582	2.7265
35	2013	9	9.6857	84.8469	947.0122	1.2490	1.2122
78	2013	9	13.5714	76.8469	904.2520	1.1541	2.5510
28	2013	9	11.0571	83.5204	935.7735	0.7480	1.5388
39	2013	9	10.7143	86.1327	907.7643	0.6694	3.4633
24	2013	9	11.7714	75.8265	947.5765	0.6531	1.1969
63	2013	9	10.5857	92.7347	953.5133	1.3184	1.7929
62	2013	9	10.4429	88.5816	878.7194	1.5204	1.2827
1	2013	9	10.6571	89.7755	879.0704	1.0612	1.8194
31	2013	10	13.8571	72.2245	851.1847	3.2673	1.1265
79	2013	10	16.1429	73.3776	988.2990	2.7469	0.8010
51	2013	10	13.7571	74.4490	947.6857	3.9939	1.4612
14	2013	10	14.8714	82.5306	903.9204	2.6082	2.3673
67	2013	10	15.4571	74.4796	909.1204	2.7724	3.0592
42	2013	10	15.1000	77.2551	880.7449	3.0582	2.3694
50	2013	10	14.9857	76.3673	908.8837	2.2531	1.6653

43	2013	10	15.1000	77.2551	880.7449	3.0582	2.3694
85	2013	10	16.5857	82.3469	917.7061	2.2337	1.5704
25	2013	10	16.9429	78.8265	986.2347	3.6276	1.1459
69	2013	10	15.2857	67.7551	948.3520	3.4010	1.1929
57	2013	10	16.1286	81.3776	901.0571	2.4888	1.5582
9	2013	10	14.7571	72.9490	859.4827	3.7776	2.6908
72	2013	10	15.2286	67.6837	883.4204	4.8837	2.6939
26	2013	10	16.0714	65.7449	872.3194	5.8980	2.4847
7	2013	10	14.8143	68.7959	864.1051	5.1898	2.3082
83	2013	10	18.8571	62.4898	951.2347	5.5224	1.0959
76	2013	10	14.9143	76.6327	928.7551	2.8520	1.6041
36	2013	10	15.4429	72.9082	936.7388	3.3806	1.5245
81	2013	10	13.7571	74.4490	947.6857	3.9939	1.4612
15	2013	10	15.2000	68.3265	941.2898	2.5439	0.7051
32	2013	10	15.1000	77.2551	880.7449	3.0582	2.3694
73	2013	10	16.1857	73.4388	971.3173	3.1194	0.7143
71	2013	10	15.4429	72.9082	936.7388	3.3806	1.5245
41	2013	10	14.6429	78.9388	880.3806	3.4612	1.3000
10	2013	10	15.1143	72.3061	973.6020	3.4520	1.2224
23	2013	10	10.6143	63.9694	778.8531	7.5500	2.7143
27	2013	10	14.8143	68.7959	864.1051	5.1898	2.3082
60	2013	10	13.7571	74.4490	947.6857	3.9939	1.4612
53	2013	10	14.7571	72.9490	859.4827	3.7776	2.6908
66	2013	10	14.8714	82.5306	903.9204	2.6082	2.3673
59	2013	10	16.1286	81.3776	901.0571	2.4888	1.5582
61	2013	10	16.1857	73.4388	971.3173	3.1194	0.7143
84	2013	10	16.1857	73.4388	971.3173	3.1194	0.7143
38	2013	10	16.1286	81.3776	901.0571	2.4888	1.5582
87	2013	10	15.8429	66.8265	905.6980	3.1724	2.4898
34	2013	10	16.1286	81.3776	901.0571	2.4888	1.5582
29	2013	10	15.2857	67.7551	948.3520	3.4010	1.1929
5	2013	10	12.8714	71.4898	836.6633	5.2194	2.2418
8	2013	10	14.7571	72.9490	859.4827	3.7776	2.6908
12	2013	10	12.8714	71.4898	836.6633	5.2194	2.2418
13	2013	10	18.8571	62.4898	951.2347	5.5224	1.0959
18	2013	10	14.8857	74.2245	973.6480	3.0541	0.9684
33	2013	10	14.9857	76.3673	908.8837	2.2531	1.6653
56	2013	10	16.9429	78.8265	986.2347	3.6276	1.1459
77	2013	10	15.2000	68.3265	941.2898	2.5439	0.7051
54	2013	10	12.8714	71.4898	836.6633	5.2194	2.2418
21	2013	10	14.9857	76.3673	908.8837	2.2531	1.6653
68	2013	10	16.1429	73.3776	988.2990	2.7469	0.8010
74	2013	10	16.1857	73.4388	971.3173	3.1194	0.7143
88	2013	10	15.1000	77.2551	880.7449	3.0582	2.3694
16	2013	10	14.9143	76.6327	928.7551	2.8520	1.6041
30	2013	10	14.8714	82.5306	903.9204	2.6082	2.3673
6	2013	10	16.1429	73.3776	988.2990	2.7469	0.8010
49	2013	10	15.2857	67.7551	948.3520	3.4010	1.1929
22	2013	10	15.1000	77.2551	880.7449	3.0582	2.3694
45	2013	10	13.6286	64.0204	821.7408	6.7531	2.4480

58	2013	10	15.2857	67.7551	948.3520	3.4010	1.1929
37	2013	10	16.1429	73.3776	988.2990	2.7469	0.8010
17	2013	10	15.4571	74.4796	909.1204	2.7724	3.0592
55	2013	10	15.2286	67.6837	883.4204	4.8837	2.6939
46	2013	10	14.9143	76.6327	928.7551	2.8520	1.6041
86	2013	10	15.5143	70.7245	872.2714	3.8582	1.3378
2	2013	10	15.5143	70.7245	872.2714	3.8582	1.3378
4	2013	10	14.9857	76.3673	908.8837	2.2531	1.6653
47	2013	10	18.9714	64.1837	966.6878	3.4959	0.8367
82	2013	10	15.1000	77.2551	880.7449	3.0582	2.3694
19	2013	10	16.9143	72.3163	968.5969	3.4571	1.2388
20	2013	10	14.7571	72.9490	859.4827	3.7776	2.6908
80	2013	10	15.1000	77.2551	880.7449	3.0582	2.3694
3	2013	10	18.8571	62.4898	951.2347	5.5224	1.0959
52	2013	10	15.4571	74.4796	909.1204	2.7724	3.0592
70	2013	10	16.5857	82.3469	917.7061	2.2337	1.5704
64	2013	10	10.6143	63.9694	778.8531	7.5500	2.7143
48	2013	10	15.2000	68.3265	941.2898	2.5439	0.7051
65	2013	10	15.4571	74.4796	909.1204	2.7724	3.0592
44	2013	10	16.5857	82.3469	917.7061	2.2337	1.5704
75	2013	10	10.6143	63.9694	778.8531	7.5500	2.7143
40	2013	10	15.6143	80.4592	954.2439	3.7061	1.7735
11	2013	10	15.2286	67.6837	883.4204	4.8837	2.6939
35	2013	10	13.7571	74.4490	947.6857	3.9939	1.4612
78	2013	10	15.8429	66.8265	905.6980	3.1724	2.4898
28	2013	10	15.4429	72.9082	936.7388	3.3806	1.5245
39	2013	10	15.4571	74.4796	909.1204	2.7724	3.0592
24	2013	10	15.2857	67.7551	948.3520	3.4010	1.1929
63	2013	10	15.6143	80.4592	954.2439	3.7061	1.7735
62	2013	10	14.6429	78.9388	880.3806	3.4612	1.3000
1	2013	10	15.1000	77.2551	880.7449	3.0582	2.3694
31	2013	11	14.6000	62.6939	849.6500	4.5347	1.3786
79	2013	11	13.8000	66.4694	985.7337	4.0429	0.9092
51	2013	11	11.7000	71.1122	945.6643	5.1888	1.4837
14	2013	11	14.9714	78.1122	902.6582	3.3765	2.8306
67	2013	11	14.8857	68.1020	907.4337	3.8592	3.4786
42	2013	11	15.3143	68.0816	879.3857	4.7888	3.1347
50	2013	11	14.0714	68.1837	906.7765	4.0776	2.2163
43	2013	11	15.3143	68.0816	879.3857	4.7888	3.1347
85	2013	11	15.6429	72.1531	915.1449	4.6245	1.8969
25	2013	11	17.3429	74.0816	983.9531	4.5112	1.4480
69	2013	11	14.2857	63.9184	946.1612	4.8439	1.5051
57	2013	11	14.6143	71.7143	899.0510	3.7112	2.0306
9	2013	11	15.4571	66.3265	858.4235	4.7020	2.8173
72	2013	11	17.1000	62.6327	882.2643	5.7480	2.7643
26	2013	11	18.1857	61.3673	871.5388	6.3724	2.6102
7	2013	11	17.6286	64.2143	863.2429	5.8459	2.4561
83	2013	11	22.0571	59.2857	949.6908	6.1643	1.1316
76	2013	11	12.7714	69.8367	926.6776	4.3602	1.9643
36	2013	11	14.6143	67.5306	934.7510	4.9704	1.7398

81	2013	11	11.7000	71.1122	945.6643	5.1888	1.4837
15	2013	11	14.8286	61.0102	938.9765	4.1592	1.1551
32	2013	11	15.3143	68.0816	879.3857	4.7888	3.1347
73	2013	11	14.8286	67.0408	968.8939	4.6071	0.7643
71	2013	11	14.6143	67.5306	934.7510	4.9704	1.7398
41	2013	11	15.2857	71.2959	878.7071	5.2000	1.6102
10	2013	11	12.8429	66.8776	971.2388	4.0745	1.3541
23	2013	11	12.8429	62.2449	778.5031	7.4041	2.7449
27	2013	11	17.6286	64.2143	863.2429	5.8459	2.4561
60	2013	11	11.7000	71.1122	945.6643	5.1888	1.4837
53	2013	11	15.4571	66.3265	858.4235	4.7020	2.8173
66	2013	11	14.9714	78.1122	902.6582	3.3765	2.8306
59	2013	11	14.6143	71.7143	899.0510	3.7112	2.0306
61	2013	11	14.8286	67.0408	968.8939	4.6071	0.7643
84	2013	11	14.8286	67.0408	968.8939	4.6071	0.7643
38	2013	11	14.6143	71.7143	899.0510	3.7112	2.0306
87	2013	11	17.5286	61.5816	904.1898	4.8847	2.3867
34	2013	11	14.6143	71.7143	899.0510	3.7112	2.0306
29	2013	11	14.2857	63.9184	946.1612	4.8439	1.5051
5	2013	11	15.7143	64.3061	835.9643	5.8429	2.4245
8	2013	11	15.4571	66.3265	858.4235	4.7020	2.8173
12	2013	11	15.7143	64.3061	835.9643	5.8429	2.4245
13	2013	11	22.0571	59.2857	949.6908	6.1643	1.1316
18	2013	11	13.0286	66.4796	971.2847	4.1939	1.1796
33	2013	11	14.0714	68.1837	906.7765	4.0776	2.2163
56	2013	11	17.3429	74.0816	983.9531	4.5112	1.4480
77	2013	11	14.8286	61.0102	938.9765	4.1592	1.1551
54	2013	11	15.7143	64.3061	835.9643	5.8429	2.4245
21	2013	11	14.0714	68.1837	906.7765	4.0776	2.2163
68	2013	11	13.8000	66.4694	985.7337	4.0429	0.9092
74	2013	11	14.8286	67.0408	968.8939	4.6071	0.7643
88	2013	11	15.3143	68.0816	879.3857	4.7888	3.1347
16	2013	11	12.7714	69.8367	926.6776	4.3602	1.9643
30	2013	11	14.9714	78.1122	902.6582	3.3765	2.8306
6	2013	11	13.8000	66.4694	985.7337	4.0429	0.9092
49	2013	11	14.2857	63.9184	946.1612	4.8439	1.5051
22	2013	11	15.3143	68.0816	879.3857	4.7888	3.1347
45	2013	11	17.2857	60.8571	821.3133	6.4561	2.5582
58	2013	11	14.2857	63.9184	946.1612	4.8439	1.5051
37	2013	11	13.8000	66.4694	985.7337	4.0429	0.9092
17	2013	11	14.8857	68.1020	907.4337	3.8592	3.4786
55	2013	11	17.1000	62.6327	882.2643	5.7480	2.7643
46	2013	11	12.7714	69.8367	926.6776	4.3602	1.9643
86	2013	11	16.8000	63.4796	870.8439	5.3878	1.5796
2	2013	11	16.8000	63.4796	870.8439	5.3878	1.5796
4	2013	11	14.0714	68.1837	906.7765	4.0776	2.2163
47	2013	11	21.4857	61.3673	964.8133	4.3449	1.0112
82	2013	11	15.3143	68.0816	879.3857	4.7888	3.1347
19	2013	11	18.7286	67.9592	966.7847	3.8765	1.2959
20	2013	11	15.4571	66.3265	858.4235	4.7020	2.8173

80	2013	11	15.3143	68.0816	879.3857	4.7888	3.1347
3	2013	11	22.0571	59.2857	949.6908	6.1643	1.1316
52	2013	11	14.8857	68.1020	907.4337	3.8592	3.4786
70	2013	11	15.6429	72.1531	915.1449	4.6245	1.8969
64	2013	11	12.8429	62.2449	778.5031	7.4041	2.7449
48	2013	11	14.8286	61.0102	938.9765	4.1592	1.1551
65	2013	11	14.8857	68.1020	907.4337	3.8592	3.4786
44	2013	11	15.6429	72.1531	915.1449	4.6245	1.8969
75	2013	11	12.8429	62.2449	778.5031	7.4041	2.7449
40	2013	11	14.8143	73.5612	952.2704	4.9286	2.0531
11	2013	11	17.1000	62.6327	882.2643	5.7480	2.7643
35	2013	11	11.7000	71.1122	945.6643	5.1888	1.4837
78	2013	11	17.5286	61.5816	904.1898	4.8847	2.3867
28	2013	11	14.6143	67.5306	934.7510	4.9704	1.7398
39	2013	11	14.8857	68.1020	907.4337	3.8592	3.4786
24	2013	11	14.2857	63.9184	946.1612	4.8439	1.5051
63	2013	11	14.8143	73.5612	952.2704	4.9286	2.0531
62	2013	11	15.2857	71.2959	878.7071	5.2000	1.6102
1	2013	11	15.3143	68.0816	879.3857	4.7888	3.1347
31	2013	12	14.2286	66.0204	846.6163	4.6194	1.3439
79	2013	12	16.5286	74.3061	982.0102	2.6122	0.9276
51	2013	12	15.9286	80.1837	942.0082	3.7337	1.5163
14	2013	12	17.2286	87.3776	899.2939	2.7704	3.0857
67	2013	12	16.9571	75.8367	903.9092	2.4714	3.4296
42	2013	12	16.0857	72.5816	876.0969	3.9490	3.0347
50	2013	12	16.4286	70.3980	903.2235	3.6592	2.3286
43	2013	12	16.0857	72.5816	876.0969	3.9490	3.0347
85	2013	12	17.2429	74.3571	911.5337	4.4827	1.9776
25	2013	12	19.7286	82.0408	979.7622	2.8000	1.3602
69	2013	12	17.6000	70.1939	942.3122	3.5337	1.7306
57	2013	12	16.2143	78.5816	895.5888	2.8816	2.1122
9	2013	12	16.1143	71.4490	855.3143	4.6306	3.0622
72	2013	12	17.6286	68.2041	878.9398	5.1561	2.8143
26	2013	12	19.6000	63.7755	868.3204	6.6082	2.7163
7	2013	12	17.5000	66.5102	860.0918	5.6867	2.5480
83	2013	12	22.3571	63.3367	945.6980	5.6704	1.2582
76	2013	12	16.1286	74.3673	923.0602	3.2847	2.1051
36	2013	12	17.5571	75.6122	930.9755	3.9704	1.7122
81	2013	12	15.9286	80.1837	942.0082	3.7337	1.5163
15	2013	12	17.0286	63.3673	935.2571	3.6276	1.3439
32	2013	12	16.0857	72.5816	876.0969	3.9490	3.0347
73	2013	12	17.4143	72.7551	965.0153	3.5878	0.8510
71	2013	12	17.5571	75.6122	930.9755	3.9704	1.7122
41	2013	12	15.2857	74.9898	875.4184	4.5163	1.6949
10	2013	12	16.1571	75.6122	967.4449	2.1653	1.4459
23	2013	12	12.3143	60.0816	776.0469	7.3316	2.6714
27	2013	12	17.5000	66.5102	860.0918	5.6867	2.5480
60	2013	12	15.9286	80.1837	942.0082	3.7337	1.5163
53	2013	12	16.1143	71.4490	855.3143	4.6306	3.0622
66	2013	12	17.2286	87.3776	899.2939	2.7704	3.0857

59	2013	12	16.2143	78.5816	895.5888	2.8816	2.1122
61	2013	12	17.4143	72.7551	965.0153	3.5878	0.8510
84	2013	12	17.4143	72.7551	965.0153	3.5878	0.8510
38	2013	12	16.2143	78.5816	895.5888	2.8816	2.1122
87	2013	12	18.2429	65.4490	900.6398	4.3847	2.3020
34	2013	12	16.2143	78.5816	895.5888	2.8816	2.1122
29	2013	12	17.6000	70.1939	942.3122	3.5337	1.7306
5	2013	12	16.0571	63.2143	832.9949	6.1888	2.6663
8	2013	12	16.1143	71.4490	855.3143	4.6306	3.0622
12	2013	12	16.0571	63.2143	832.9949	6.1888	2.6663
13	2013	12	22.3571	63.3367	945.6980	5.6704	1.2582
18	2013	12	15.9286	72.6735	967.7296	2.9153	1.2980
33	2013	12	16.4286	70.3980	903.2235	3.6592	2.3286
56	2013	12	19.7286	82.0408	979.7622	2.8000	1.3602
77	2013	12	17.0286	63.3673	935.2571	3.6276	1.3439
54	2013	12	16.0571	63.2143	832.9949	6.1888	2.6663
21	2013	12	16.4286	70.3980	903.2235	3.6592	2.3286
68	2013	12	16.5286	74.3061	982.0102	2.6122	0.9276
74	2013	12	17.4143	72.7551	965.0153	3.5878	0.8510
88	2013	12	16.0857	72.5816	876.0969	3.9490	3.0347
16	2013	12	16.1286	74.3673	923.0602	3.2847	2.1051
30	2013	12	17.2286	87.3776	899.2939	2.7704	3.0857
6	2013	12	16.5286	74.3061	982.0102	2.6122	0.9276
49	2013	12	17.6000	70.1939	942.3122	3.5337	1.7306
22	2013	12	16.0857	72.5816	876.0969	3.9490	3.0347
45	2013	12	16.6429	54.5408	818.4898	7.1510	2.9071
58	2013	12	17.6000	70.1939	942.3122	3.5337	1.7306
37	2013	12	16.5286	74.3061	982.0102	2.6122	0.9276
17	2013	12	16.9571	75.8367	903.9092	2.4714	3.4296
55	2013	12	17.6286	68.2041	878.9398	5.1561	2.8143
46	2013	12	16.1286	74.3673	923.0602	3.2847	2.1051
86	2013	12	16.7429	65.5918	867.6153	4.9357	1.5673
2	2013	12	16.7429	65.5918	867.6153	4.9357	1.5673
4	2013	12	16.4286	70.3980	903.2235	3.6592	2.3286
47	2013	12	22.1429	68.1122	960.7061	3.8714	1.1245
82	2013	12	16.0857	72.5816	876.0969	3.9490	3.0347
19	2013	12	20.8000	74.0000	962.8582	1.8745	1.3010
20	2013	12	16.1143	71.4490	855.3143	4.6306	3.0622
80	2013	12	16.0857	72.5816	876.0969	3.9490	3.0347
3	2013	12	22.3571	63.3367	945.6980	5.6704	1.2582
52	2013	12	16.9571	75.8367	903.9092	2.4714	3.4296
70	2013	12	17.2429	74.3571	911.5337	4.4827	1.9776
64	2013	12	12.3143	60.0816	776.0469	7.3316	2.6714
48	2013	12	17.0286	63.3673	935.2571	3.6276	1.3439
65	2013	12	16.9571	75.8367	903.9092	2.4714	3.4296
44	2013	12	17.2429	74.3571	911.5337	4.4827	1.9776
75	2013	12	12.3143	60.0816	776.0469	7.3316	2.6714
40	2013	12	17.2143	82.3571	948.4561	3.1173	2.1898
11	2013	12	17.6286	68.2041	878.9398	5.1561	2.8143
35	2013	12	15.9286	80.1837	942.0082	3.7337	1.5163

78	2013	12	18.2429	65.4490	900.6398	4.3847	2.3020
28	2013	12	17.5571	75.6122	930.9755	3.9704	1.7122
39	2013	12	16.9571	75.8367	903.9092	2.4714	3.4296
24	2013	12	17.6000	70.1939	942.3122	3.5337	1.7306
63	2013	12	17.2143	82.3571	948.4561	3.1173	2.1898
62	2013	12	15.2857	74.9898	875.4184	4.5163	1.6949
1	2013	12	16.0857	72.5816	876.0969	3.9490	3.0347
31	2013	13	12.9571	69.8776	845.9959	4.0847	1.2469
79	2013	13	14.7000	82.3163	980.8878	2.0061	0.9153
51	2013	13	13.1571	84.0918	940.8143	3.2643	1.5857
14	2013	13	13.9000	89.0816	897.8980	3.3673	2.8684
67	2013	13	13.8143	78.2959	902.8510	1.9122	3.0327
42	2013	13	13.4429	76.5102	875.0827	3.4194	2.5959
50	2013	13	13.7571	72.5510	902.4194	3.4898	2.1071
43	2013	13	13.4429	76.5102	875.0827	3.4194	2.5959
85	2013	13	14.9143	77.9490	910.9306	3.7939	2.0337
25	2013	13	17.4429	84.5000	978.2255	3.0735	1.1867
69	2013	13	14.3571	74.0204	941.2531	3.0622	1.6888
57	2013	13	13.7429	83.6224	894.7276	3.0255	1.7388
9	2013	13	13.9143	73.0408	854.2541	4.8551	2.9765
72	2013	13	15.4714	69.6531	877.7031	5.6255	2.6806
26	2013	13	16.8857	62.7551	866.9531	6.5296	2.5663
7	2013	13	15.7429	67.6224	858.8469	4.7949	2.3235
83	2013	13	20.7714	62.4694	944.1092	5.1143	1.2929
76	2013	13	13.5429	78.2551	922.0816	3.1980	1.9980
36	2013	13	13.9429	80.0408	929.8500	3.7133	1.6612
81	2013	13	13.1571	84.0918	940.8143	3.2643	1.5857
15	2013	13	14.6286	68.2245	934.3122	3.7000	1.2959
32	2013	13	13.4429	76.5102	875.0827	3.4194	2.5959
73	2013	13	15.1000	79.8776	963.9724	3.3908	0.8949
71	2013	13	13.9429	80.0408	929.8500	3.7133	1.6612
41	2013	13	14.0714	78.2143	874.5857	4.3071	1.5133
10	2013	13	13.7857	80.5510	966.2418	1.9194	1.5337
23	2013	13	9.5571	62.6837	775.1214	6.5449	2.7837
27	2013	13	15.7429	67.6224	858.8469	4.7949	2.3235
60	2013	13	13.1571	84.0918	940.8143	3.2643	1.5857
53	2013	13	13.9143	73.0408	854.2541	4.8551	2.9765
66	2013	13	13.9000	89.0816	897.8980	3.3673	2.8684
59	2013	13	13.7429	83.6224	894.7276	3.0255	1.7388
61	2013	13	15.1000	79.8776	963.9724	3.3908	0.8949
84	2013	13	15.1000	79.8776	963.9724	3.3908	0.8949
38	2013	13	13.7429	83.6224	894.7276	3.0255	1.7388
87	2013	13	15.5286	67.2551	899.4622	3.4276	2.4031
34	2013	13	13.7429	83.6224	894.7276	3.0255	1.7388
29	2013	13	14.3571	74.0204	941.2531	3.0622	1.6888
5	2013	13	13.8286	61.8061	831.8582	5.9082	2.5000
8	2013	13	13.9143	73.0408	854.2541	4.8551	2.9765
12	2013	13	13.8286	61.8061	831.8582	5.9082	2.5000
13	2013	13	20.7714	62.4694	944.1092	5.1143	1.2929
18	2013	13	13.4714	80.6633	966.6571	2.3031	1.2867

33	2013	13	13.7571	72.5510	902.4194	3.4898	2.1071
56	2013	13	17.4429	84.5000	978.2255	3.0735	1.1867
77	2013	13	14.6286	68.2245	934.3122	3.7000	1.2959
54	2013	13	13.8286	61.8061	831.8582	5.9082	2.5000
21	2013	13	13.7571	72.5510	902.4194	3.4898	2.1071
68	2013	13	14.7000	82.3163	980.8878	2.0061	0.9153
74	2013	13	15.1000	79.8776	963.9724	3.3908	0.8949
88	2013	13	13.4429	76.5102	875.0827	3.4194	2.5959
16	2013	13	13.5429	78.2551	922.0816	3.1980	1.9980
30	2013	13	13.9000	89.0816	897.8980	3.3673	2.8684
6	2013	13	14.7000	82.3163	980.8878	2.0061	0.9153
49	2013	13	14.3571	74.0204	941.2531	3.0622	1.6888
22	2013	13	13.4429	76.5102	875.0827	3.4194	2.5959
45	2013	13	13.6571	55.0714	817.3010	6.9531	2.5857
58	2013	13	14.3571	74.0204	941.2531	3.0622	1.6888
37	2013	13	14.7000	82.3163	980.8878	2.0061	0.9153
17	2013	13	13.8143	78.2959	902.8510	1.9122	3.0327
55	2013	13	15.4714	69.6531	877.7031	5.6255	2.6806
46	2013	13	13.5429	78.2551	922.0816	3.1980	1.9980
86	2013	13	14.7714	68.2653	866.7286	4.5714	1.4714
2	2013	13	14.7714	68.2653	866.7286	4.5714	1.4714
4	2013	13	13.7571	72.5510	902.4194	3.4898	2.1071
47	2013	13	20.7571	69.3367	959.1061	4.3020	1.0204
82	2013	13	13.4429	76.5102	875.0827	3.4194	2.5959
19	2013	13	18.3286	74.5408	961.0918	2.3786	1.3755
20	2013	13	13.9143	73.0408	854.2541	4.8551	2.9765
80	2013	13	13.4429	76.5102	875.0827	3.4194	2.5959
3	2013	13	20.7714	62.4694	944.1092	5.1143	1.2929
52	2013	13	13.8143	78.2959	902.8510	1.9122	3.0327
70	2013	13	14.9143	77.9490	910.9306	3.7939	2.0337
64	2013	13	9.5571	62.6837	775.1214	6.5449	2.7837
48	2013	13	14.6286	68.2245	934.3122	3.7000	1.2959
65	2013	13	13.8143	78.2959	902.8510	1.9122	3.0327
44	2013	13	14.9143	77.9490	910.9306	3.7939	2.0337
75	2013	13	9.5571	62.6837	775.1214	6.5449	2.7837
40	2013	13	13.8143	87.9082	947.0694	2.5388	2.0612
11	2013	13	15.4714	69.6531	877.7031	5.6255	2.6806
35	2013	13	13.1571	84.0918	940.8143	3.2643	1.5857
78	2013	13	15.5286	67.2551	899.4622	3.4276	2.4031
28	2013	13	13.9429	80.0408	929.8500	3.7133	1.6612
39	2013	13	13.8143	78.2959	902.8510	1.9122	3.0327
24	2013	13	14.3571	74.0204	941.2531	3.0622	1.6888
63	2013	13	13.8143	87.9082	947.0694	2.5388	2.0612
62	2013	13	14.0714	78.2143	874.5857	4.3071	1.5133
1	2013	13	13.4429	76.5102	875.0827	3.4194	2.5959
31	2013	14	12.1000	73.1531	846.2653	3.2888	1.1633
79	2013	14	14.9714	86.3061	981.0082	1.4847	0.8133
51	2013	14	12.9857	86.8980	941.1347	2.0214	1.4122
14	2013	14	13.5286	91.0000	898.0755	2.3959	2.4276
67	2013	14	12.9571	80.8265	903.2510	1.1204	2.8173

42	2013	14	12.4429	81.9592	875.3990	2.6990	2.6296
50	2013	14	12.6000	77.3776	902.9194	2.4898	1.8306
43	2013	14	12.4429	81.9592	875.3990	2.6990	2.6296
85	2013	14	13.5429	82.2449	911.4367	2.5847	1.9235
25	2013	14	17.6143	84.8878	978.4694	2.8245	1.1102
69	2013	14	13.7714	78.3265	941.8551	1.9459	1.4827
57	2013	14	12.6857	86.5408	895.1224	2.0694	1.7653
9	2013	14	13.5143	75.3367	854.5112	3.7418	2.7653
72	2013	14	14.6143	72.8061	877.9653	4.7194	2.5520
26	2013	14	17.6571	66.7857	867.0143	5.6500	2.4898
7	2013	14	15.6714	71.8878	858.9755	3.2847	1.9847
83	2013	14	19.9571	64.4694	944.5082	3.8612	1.1653
76	2013	14	13.0857	82.3163	922.5622	2.2949	1.8469
36	2013	14	13.7000	83.7755	930.2990	2.6153	1.5357
81	2013	14	12.9857	86.8980	941.1347	2.0214	1.4122
15	2013	14	13.9714	75.1327	934.8429	2.4286	1.0071
32	2013	14	12.4429	81.9592	875.3990	2.6990	2.6296
73	2013	14	15.3000	84.7143	964.4510	2.4378	0.6582
71	2013	14	13.7000	83.7755	930.2990	2.6153	1.5357
41	2013	14	13.1143	80.8571	874.9582	3.5327	1.4806
10	2013	14	14.0429	82.0918	966.4643	1.7235	1.3296
23	2013	14	9.7286	71.4490	775.1765	5.1214	2.7469
27	2013	14	15.6714	71.8878	858.9755	3.2847	1.9847
60	2013	14	12.9857	86.8980	941.1347	2.0214	1.4122
53	2013	14	13.5143	75.3367	854.5112	3.7418	2.7653
66	2013	14	13.5286	91.0000	898.0755	2.3959	2.4276
59	2013	14	12.6857	86.5408	895.1224	2.0694	1.7653
61	2013	14	15.3000	84.7143	964.4510	2.4378	0.6582
84	2013	14	15.3000	84.7143	964.4510	2.4378	0.6582
38	2013	14	12.6857	86.5408	895.1224	2.0694	1.7653
87	2013	14	15.1286	71.4388	899.8388	1.9490	2.3102
34	2013	14	12.6857	86.5408	895.1224	2.0694	1.7653
29	2013	14	13.7714	78.3265	941.8551	1.9459	1.4827
5	2013	14	12.9571	69.6122	832.0194	4.5531	2.3776
8	2013	14	13.5143	75.3367	854.5112	3.7418	2.7653
12	2013	14	12.9571	69.6122	832.0194	4.5531	2.3776
13	2013	14	19.9571	64.4694	944.5082	3.8612	1.1653
18	2013	14	14.2286	85.5204	966.8337	1.5898	1.0939
33	2013	14	12.6000	77.3776	902.9194	2.4898	1.8306
56	2013	14	17.6143	84.8878	978.4694	2.8245	1.1102
77	2013	14	13.9714	75.1327	934.8429	2.4286	1.0071
54	2013	14	12.9571	69.6122	832.0194	4.5531	2.3776
21	2013	14	12.6000	77.3776	902.9194	2.4898	1.8306
68	2013	14	14.9714	86.3061	981.0082	1.4847	0.8133
74	2013	14	15.3000	84.7143	964.4510	2.4378	0.6582
88	2013	14	12.4429	81.9592	875.3990	2.6990	2.6296
16	2013	14	13.0857	82.3163	922.5622	2.2949	1.8469
30	2013	14	13.5286	91.0000	898.0755	2.3959	2.4276
6	2013	14	14.9714	86.3061	981.0082	1.4847	0.8133
49	2013	14	13.7714	78.3265	941.8551	1.9459	1.4827

22	2013	14	12.4429	81.9592	875.3990	2.6990	2.6296
45	2013	14	14.6714	67.5306	817.2408	5.4816	2.0224
58	2013	14	13.7714	78.3265	941.8551	1.9459	1.4827
37	2013	14	14.9714	86.3061	981.0082	1.4847	0.8133
17	2013	14	12.9571	80.8265	903.2510	1.1204	2.8173
55	2013	14	14.6143	72.8061	877.9653	4.7194	2.5520
46	2013	14	13.0857	82.3163	922.5622	2.2949	1.8469
86	2013	14	14.3286	71.3469	867.0765	3.6939	1.3704
2	2013	14	14.3286	71.3469	867.0765	3.6939	1.3704
4	2013	14	12.6000	77.3776	902.9194	2.4898	1.8306
47	2013	14	19.4714	71.1020	959.5755	3.3878	0.8276
82	2013	14	12.4429	81.9592	875.3990	2.6990	2.6296
19	2013	14	17.7429	74.8878	961.2316	2.4153	1.2541
20	2013	14	13.5143	75.3367	854.5112	3.7418	2.7653
80	2013	14	12.4429	81.9592	875.3990	2.6990	2.6296
3	2013	14	19.9571	64.4694	944.5082	3.8612	1.1653
52	2013	14	12.9571	80.8265	903.2510	1.1204	2.8173
70	2013	14	13.5429	82.2449	911.4367	2.5847	1.9235
64	2013	14	9.7286	71.4490	775.1765	5.1214	2.7469
48	2013	14	13.9714	75.1327	934.8429	2.4286	1.0071
65	2013	14	12.9571	80.8265	903.2510	1.1204	2.8173
44	2013	14	13.5429	82.2449	911.4367	2.5847	1.9235
75	2013	14	9.7286	71.4490	775.1765	5.1214	2.7469
40	2013	14	13.8714	90.6429	947.2673	2.1378	1.7510
11	2013	14	14.6143	72.8061	877.9653	4.7194	2.5520
35	2013	14	12.9857	86.8980	941.1347	2.0214	1.4122
78	2013	14	15.1286	71.4388	899.8388	1.9490	2.3102
28	2013	14	13.7000	83.7755	930.2990	2.6153	1.5357
39	2013	14	12.9571	80.8265	903.2510	1.1204	2.8173
24	2013	14	13.7714	78.3265	941.8551	1.9459	1.4827
63	2013	14	13.8714	90.6429	947.2673	2.1378	1.7510
62	2013	14	13.1143	80.8571	874.9582	3.5327	1.4806
1	2013	14	12.4429	81.9592	875.3990	2.6990	2.6296
31	2013	15	10.1286	78.9184	847.6041	2.3847	1.0867
79	2013	15	15.0000	86.0918	982.3694	0.8408	0.7163
51	2013	15	12.7571	86.0612	942.5684	1.2439	1.1806
14	2013	15	12.1143	91.2347	899.3847	0.9827	2.1051
67	2013	15	11.8286	82.2755	904.6776	0.2122	2.9031
42	2013	15	10.6571	85.2245	876.7582	1.6694	2.7980
50	2013	15	12.0429	81.4490	904.6286	1.1857	1.6143
43	2013	15	10.6571	85.2245	876.7582	1.6694	2.7980
85	2013	15	12.3714	86.9490	913.3296	1.2276	1.6143
25	2013	15	16.2143	83.2143	980.1255	2.2520	1.1286
69	2013	15	12.9571	80.8061	943.5908	0.9520	1.1449
57	2013	15	11.2000	89.2959	896.8541	0.6745	1.8296
9	2013	15	10.8286	78.7653	855.7602	2.4316	2.9020
72	2013	15	12.2571	76.3163	879.3561	2.7500	2.3367
26	2013	15	13.3857	71.0714	868.1980	4.1582	2.4194
7	2013	15	12.2714	75.7041	860.1898	2.1827	1.7010
83	2013	15	17.1143	68.9082	946.4500	2.5112	1.0173

76	2013	15	12.3429	84.6327	924.1704	1.1704	1.6388
36	2013	15	12.5571	83.7449	931.8939	1.4122	1.3643
81	2013	15	12.7571	86.0612	942.5684	1.2439	1.1806
15	2013	15	13.6429	77.1531	936.5776	1.7133	0.7867
32	2013	15	10.6571	85.2245	876.7582	1.6694	2.7980
73	2013	15	14.8000	85.5510	966.0694	1.4459	0.5898
71	2013	15	12.5571	83.7449	931.8939	1.4122	1.3643
41	2013	15	11.0000	83.8980	876.5276	2.0796	1.4163
10	2013	15	14.0000	79.4286	967.8602	1.5765	1.1929
23	2013	15	7.2143	80.1327	775.9173	3.7878	2.4724
27	2013	15	12.2714	75.7041	860.1898	2.1827	1.7010
60	2013	15	12.7571	86.0612	942.5684	1.2439	1.1806
53	2013	15	10.8286	78.7653	855.7602	2.4316	2.9020
66	2013	15	12.1143	91.2347	899.3847	0.9827	2.1051
59	2013	15	11.2000	89.2959	896.8541	0.6745	1.8296
61	2013	15	14.8000	85.5510	966.0694	1.4459	0.5898
84	2013	15	14.8000	85.5510	966.0694	1.4459	0.5898
38	2013	15	11.2000	89.2959	896.8541	0.6745	1.8296
87	2013	15	12.9429	74.5612	901.3888	1.0163	2.2327
34	2013	15	11.2000	89.2959	896.8541	0.6745	1.8296
29	2013	15	12.9571	80.8061	943.5908	0.9520	1.1449
5	2013	15	9.7857	78.5510	833.0969	3.3071	2.2857
8	2013	15	10.8286	78.7653	855.7602	2.4316	2.9020
12	2013	15	9.7857	78.5510	833.0969	3.3071	2.2857
13	2013	15	17.1143	68.9082	946.4500	2.5112	1.0173
18	2013	15	14.9571	84.0408	968.1102	1.2582	0.9592
33	2013	15	12.0429	81.4490	904.6286	1.1857	1.6143
56	2013	15	16.2143	83.2143	980.1255	2.2520	1.1286
77	2013	15	13.6429	77.1531	936.5776	1.7133	0.7867
54	2013	15	9.7857	78.5510	833.0969	3.3071	2.2857
21	2013	15	12.0429	81.4490	904.6286	1.1857	1.6143
68	2013	15	15.0000	86.0918	982.3694	0.8408	0.7163
74	2013	15	14.8000	85.5510	966.0694	1.4459	0.5898
88	2013	15	10.6571	85.2245	876.7582	1.6694	2.7980
16	2013	15	12.3429	84.6327	924.1704	1.1704	1.6388
30	2013	15	12.1143	91.2347	899.3847	0.9827	2.1051
6	2013	15	15.0000	86.0918	982.3694	0.8408	0.7163
49	2013	15	12.9571	80.8061	943.5908	0.9520	1.1449
22	2013	15	10.6571	85.2245	876.7582	1.6694	2.7980
45	2013	15	10.4571	76.1735	817.9918	4.1745	1.9592
58	2013	15	12.9571	80.8061	943.5908	0.9520	1.1449
37	2013	15	15.0000	86.0918	982.3694	0.8408	0.7163
17	2013	15	11.8286	82.2755	904.6776	0.2122	2.9031
55	2013	15	12.2571	76.3163	879.3561	2.7500	2.3367
46	2013	15	12.3429	84.6327	924.1704	1.1704	1.6388
86	2013	15	11.6714	74.6939	868.5153	2.7163	1.2592
2	2013	15	11.6714	74.6939	868.5153	2.7163	1.2592
4	2013	15	12.0429	81.4490	904.6286	1.1857	1.6143
47	2013	15	17.2000	73.0102	961.6398	2.4776	0.8357
82	2013	15	10.6571	85.2245	876.7582	1.6694	2.7980

19	2013	15	16.2857	74.0102	962.9000	1.7173	1.1867
20	2013	15	10.8286	78.7653	855.7602	2.4316	2.9020
80	2013	15	10.6571	85.2245	876.7582	1.6694	2.7980
3	2013	15	17.1143	68.9082	946.4500	2.5112	1.0173
52	2013	15	11.8286	82.2755	904.6776	0.2122	2.9031
70	2013	15	12.3714	86.9490	913.3296	1.2276	1.6143
64	2013	15	7.2143	80.1327	775.9173	3.7878	2.4724
48	2013	15	13.6429	77.1531	936.5776	1.7133	0.7867
65	2013	15	11.8286	82.2755	904.6776	0.2122	2.9031
44	2013	15	12.3714	86.9490	913.3296	1.2276	1.6143
75	2013	15	7.2143	80.1327	775.9173	3.7878	2.4724
40	2013	15	13.6000	87.4082	948.6459	1.9082	1.7102
11	2013	15	12.2571	76.3163	879.3561	2.7500	2.3367
35	2013	15	12.7571	86.0612	942.5684	1.2439	1.1806
78	2013	15	12.9429	74.5612	901.3888	1.0163	2.2327
28	2013	15	12.5571	83.7449	931.8939	1.4122	1.3643
39	2013	15	11.8286	82.2755	904.6776	0.2122	2.9031
24	2013	15	12.9571	80.8061	943.5908	0.9520	1.1449
63	2013	15	13.6000	87.4082	948.6459	1.9082	1.7102
62	2013	15	11.0000	83.8980	876.5276	2.0796	1.4163
1	2013	15	10.6571	85.2245	876.7582	1.6694	2.7980
31	2013	16	17.9429	74.1837	847.4918	3.9796	1.0347
79	2013	16	18.1714	80.5102	981.2857	1.6622	0.8520
51	2013	16	16.3429	79.6633	941.8837	3.2378	1.2469
14	2013	16	18.4857	85.9796	899.2980	2.2653	2.3449
67	2013	16	17.9429	75.4490	904.3520	1.7561	3.2571
42	2013	16	18.1429	78.9082	876.6347	3.1878	2.5827
50	2013	16	18.5429	77.1735	904.1306	3.3541	1.7367
43	2013	16	18.1429	78.9082	876.6347	3.1878	2.5827
85	2013	16	20.4286	80.6837	912.7786	3.5459	1.6796
25	2013	16	21.5571	78.2245	979.6500	3.7878	1.2990
69	2013	16	17.7429	76.0816	942.9082	3.2633	1.2633
57	2013	16	18.2714	83.9592	896.5153	1.7827	1.7867
9	2013	16	18.6286	73.8367	855.8020	3.7551	2.8031
72	2013	16	19.6429	72.4184	879.4480	4.0949	1.9429
26	2013	16	22.3714	68.5000	868.6673	4.7439	2.1143
7	2013	16	20.5000	72.8878	860.5194	3.5796	1.7347
83	2013	16	23.4000	69.8367	946.6204	3.5582	0.9592
76	2013	16	17.7143	78.9796	923.5520	3.0255	1.6765
36	2013	16	18.1286	77.2755	931.4153	3.3592	1.3806
81	2013	16	16.3429	79.6633	941.8837	3.2378	1.2469
15	2013	16	18.6857	72.6122	935.8684	3.3418	0.9653
32	2013	16	18.1429	78.9082	876.6347	3.1878	2.5827
73	2013	16	19.1286	80.3673	965.1337	3.0173	0.7633
71	2013	16	18.1286	77.2755	931.4153	3.3592	1.3806
41	2013	16	17.9714	79.1531	876.3092	3.6949	1.3041
10	2013	16	17.6000	72.9082	967.0051	3.2214	1.2163
23	2013	16	15.9286	73.9082	776.3020	4.6827	2.2622
27	2013	16	20.5000	72.8878	860.5194	3.5796	1.7347
60	2013	16	16.3429	79.6633	941.8837	3.2378	1.2469

53	2013	16	18.6286	73.8367	855.8020	3.7551	2.8031
66	2013	16	18.4857	85.9796	899.2980	2.2653	2.3449
59	2013	16	18.2714	83.9592	896.5153	1.7827	1.7867
61	2013	16	19.1286	80.3673	965.1337	3.0173	0.7633
84	2013	16	19.1286	80.3673	965.1337	3.0173	0.7633
38	2013	16	18.2714	83.9592	896.5153	1.7827	1.7867
87	2013	16	19.7000	71.0000	901.2990	2.6398	2.0306
34	2013	16	18.2714	83.9592	896.5153	1.7827	1.7867
29	2013	16	17.7429	76.0816	942.9082	3.2633	1.2633
5	2013	16	18.9429	72.7143	833.4388	4.2122	2.3010
8	2013	16	18.6286	73.8367	855.8020	3.7551	2.8031
12	2013	16	18.9429	72.7143	833.4388	4.2122	2.3010
13	2013	16	23.4000	69.8367	946.6204	3.5582	0.9592
18	2013	16	17.1429	76.5000	967.0439	2.4469	1.0602
33	2013	16	18.5429	77.1735	904.1306	3.3541	1.7367
56	2013	16	21.5571	78.2245	979.6500	3.7878	1.2990
77	2013	16	18.6857	72.6122	935.8684	3.3418	0.9653
54	2013	16	18.9429	72.7143	833.4388	4.2122	2.3010
21	2013	16	18.5429	77.1735	904.1306	3.3541	1.7367
68	2013	16	18.1714	80.5102	981.2857	1.6622	0.8520
74	2013	16	19.1286	80.3673	965.1337	3.0173	0.7633
88	2013	16	18.1429	78.9082	876.6347	3.1878	2.5827
16	2013	16	17.7143	78.9796	923.5520	3.0255	1.6765
30	2013	16	18.4857	85.9796	899.2980	2.2653	2.3449
6	2013	16	18.1714	80.5102	981.2857	1.6622	0.8520
49	2013	16	17.7429	76.0816	942.9082	3.2633	1.2633
22	2013	16	18.1429	78.9082	876.6347	3.1878	2.5827
45	2013	16	19.7429	72.9286	818.4929	4.6469	2.0061
58	2013	16	17.7429	76.0816	942.9082	3.2633	1.2633
37	2013	16	18.1714	80.5102	981.2857	1.6622	0.8520
17	2013	16	17.9429	75.4490	904.3520	1.7561	3.2571
55	2013	16	19.6429	72.4184	879.4480	4.0949	1.9429
46	2013	16	17.7143	78.9796	923.5520	3.0255	1.6765
86	2013	16	18.9714	71.8265	868.4194	4.1347	1.3418
2	2013	16	18.9714	71.8265	868.4194	4.1347	1.3418
4	2013	16	18.5429	77.1735	904.1306	3.3541	1.7367
47	2013	16	23.6429	73.3061	961.5551	3.5490	0.8786
82	2013	16	18.1429	78.9082	876.6347	3.1878	2.5827
19	2013	16	21.3714	71.8265	962.7980	3.1418	1.3704
20	2013	16	18.6286	73.8367	855.8020	3.7551	2.8031
80	2013	16	18.1429	78.9082	876.6347	3.1878	2.5827
3	2013	16	23.4000	69.8367	946.6204	3.5582	0.9592
52	2013	16	17.9429	75.4490	904.3520	1.7561	3.2571
70	2013	16	20.4286	80.6837	912.7786	3.5459	1.6796
64	2013	16	15.9286	73.9082	776.3020	4.6827	2.2622
48	2013	16	18.6857	72.6122	935.8684	3.3418	0.9653
65	2013	16	17.9429	75.4490	904.3520	1.7561	3.2571
44	2013	16	20.4286	80.6837	912.7786	3.5459	1.6796
75	2013	16	15.9286	73.9082	776.3020	4.6827	2.2622
40	2013	16	18.7143	79.7959	948.1908	3.6357	1.8918

11	2013	16	19.6429	72.4184	879.4480	4.0949	1.9429
35	2013	16	16.3429	79.6633	941.8837	3.2378	1.2469
78	2013	16	19.7000	71.0000	901.2990	2.6398	2.0306
28	2013	16	18.1286	77.2755	931.4153	3.3592	1.3806
39	2013	16	17.9429	75.4490	904.3520	1.7561	3.2571
24	2013	16	17.7429	76.0816	942.9082	3.2633	1.2633
63	2013	16	18.7143	79.7959	948.1908	3.6357	1.8918
62	2013	16	17.9714	79.1531	876.3092	3.6949	1.3041
1	2013	16	18.1429	78.9082	876.6347	3.1878	2.5827
31	2013	17	16.8429	69.5510	846.0367	5.2827	1.1092
79	2013	17	18.1143	80.6429	979.8786	2.2367	0.9622
51	2013	17	15.8143	82.4184	940.4571	3.6969	1.5082
14	2013	17	16.8571	87.7245	897.8888	2.9133	2.7204
67	2013	17	16.4857	76.1735	902.8704	2.7194	2.8663
42	2013	17	16.8857	77.9796	875.1224	4.1133	2.5255
50	2013	17	17.7286	75.3571	902.2143	4.4112	1.8684
43	2013	17	16.8857	77.9796	875.1224	4.1133	2.5255
85	2013	17	18.9571	76.1122	910.5990	4.7939	1.8724
25	2013	17	19.5429	79.6531	977.9296	3.8051	1.3612
69	2013	17	17.1000	74.6224	941.1745	4.2439	1.5776
57	2013	17	16.3000	83.1633	894.7286	2.4929	1.7959
9	2013	17	18.1571	71.0102	854.4551	5.0031	2.8735
72	2013	17	19.9143	70.0204	877.8163	5.9316	2.4133
26	2013	17	22.2714	64.6837	867.1755	7.1561	2.2980
7	2013	17	20.9857	66.5714	859.0684	5.9306	2.3071
83	2013	17	24.6571	68.1020	944.1418	4.9673	1.0816
76	2013	17	16.7714	77.8163	921.8816	3.7847	1.8929
36	2013	17	16.6714	77.8061	929.7990	4.3051	1.5265
81	2013	17	15.8143	82.4184	940.4571	3.6969	1.5082
15	2013	17	18.7714	70.9184	933.8908	3.7827	1.1398
32	2013	17	16.8857	77.9796	875.1224	4.1133	2.5255
73	2013	17	18.4571	79.9592	963.4051	3.7571	0.8235
71	2013	17	16.6714	77.8061	929.7990	4.3051	1.5265
41	2013	17	17.6571	76.5918	874.6418	4.9449	1.4878
10	2013	17	16.5000	76.4388	965.6214	3.2704	1.2592
23	2013	17	15.8429	62.0510	775.8878	7.2061	2.4684
27	2013	17	20.9857	66.5714	859.0684	5.9306	2.3071
60	2013	17	15.8143	82.4184	940.4571	3.6969	1.5082
53	2013	17	18.1571	71.0102	854.4551	5.0031	2.8735
66	2013	17	16.8571	87.7245	897.8888	2.9133	2.7204
59	2013	17	16.3000	83.1633	894.7286	2.4929	1.7959
61	2013	17	18.4571	79.9592	963.4051	3.7571	0.8235
84	2013	17	18.4571	79.9592	963.4051	3.7571	0.8235
38	2013	17	16.3000	83.1633	894.7286	2.4929	1.7959
87	2013	17	19.6500	69.4082	899.6898	4.0449	1.8367
34	2013	17	16.3000	83.1633	894.7286	2.4929	1.7959
29	2013	17	17.1000	74.6224	941.1745	4.2439	1.5776
5	2013	17	19.0000	62.9082	832.3255	6.4000	2.8133
8	2013	17	18.1571	71.0102	854.4551	5.0031	2.8735
12	2013	17	19.0000	62.9082	832.3255	6.4000	2.8133

13	2013	17	24.6571	68.1020	944.1418	4.9673	1.0816
18	2013	17	17.4429	77.3367	965.6786	2.8224	1.1551
33	2013	17	17.7286	75.3571	902.2143	4.4112	1.8684
56	2013	17	19.5429	79.6531	977.9296	3.8051	1.3612
77	2013	17	18.7714	70.9184	933.8908	3.7827	1.1398
54	2013	17	19.0000	62.9082	832.3255	6.4000	2.8133
21	2013	17	17.7286	75.3571	902.2143	4.4112	1.8684
68	2013	17	18.1143	80.6429	979.8786	2.2367	0.9622
74	2013	17	18.4571	79.9592	963.4051	3.7571	0.8235
88	2013	17	16.8857	77.9796	875.1224	4.1133	2.5255
16	2013	17	16.7714	77.8163	921.8816	3.7847	1.8929
30	2013	17	16.8571	87.7245	897.8888	2.9133	2.7204
6	2013	17	18.1143	80.6429	979.8786	2.2367	0.9622
49	2013	17	17.1000	74.6224	941.1745	4.2439	1.5776
22	2013	17	16.8857	77.9796	875.1224	4.1133	2.5255
45	2013	17	20.2286	59.1837	817.7367	7.3827	2.4653
58	2013	17	17.1000	74.6224	941.1745	4.2439	1.5776
37	2013	17	18.1143	80.6429	979.8786	2.2367	0.9622
17	2013	17	16.4857	76.1735	902.8704	2.7194	2.8663
55	2013	17	19.9143	70.0204	877.8163	5.9316	2.4133
46	2013	17	16.7714	77.8163	921.8816	3.7847	1.8929
86	2013	17	19.2000	68.4694	866.8327	4.8714	1.4357
2	2013	17	19.2000	68.4694	866.8327	4.8714	1.4357
4	2013	17	17.7286	75.3571	902.2143	4.4112	1.8684
47	2013	17	23.8143	72.7959	959.1643	4.3408	0.9306
82	2013	17	16.8857	77.9796	875.1224	4.1133	2.5255
19	2013	17	19.5286	76.7245	961.1469	3.5765	1.3143
20	2013	17	18.1571	71.0102	854.4551	5.0031	2.8735
80	2013	17	16.8857	77.9796	875.1224	4.1133	2.5255
3	2013	17	24.6571	68.1020	944.1418	4.9673	1.0816
52	2013	17	16.4857	76.1735	902.8704	2.7194	2.8663
70	2013	17	18.9571	76.1122	910.5990	4.7939	1.8724
64	2013	17	15.8429	62.0510	775.8878	7.2061	2.4684
48	2013	17	18.7714	70.9184	933.8908	3.7827	1.1398
65	2013	17	16.4857	76.1735	902.8704	2.7194	2.8663
44	2013	17	18.9571	76.1122	910.5990	4.7939	1.8724
75	2013	17	15.8429	62.0510	775.8878	7.2061	2.4684
40	2013	17	16.6143	82.5000	946.8643	3.8878	2.0663
11	2013	17	19.9143	70.0204	877.8163	5.9316	2.4133
35	2013	17	15.8143	82.4184	940.4571	3.6969	1.5082
78	2013	17	19.6500	69.4082	899.6898	4.0449	1.8367
28	2013	17	16.6714	77.8061	929.7990	4.3051	1.5265
39	2013	17	16.4857	76.1735	902.8704	2.7194	2.8663
24	2013	17	17.1000	74.6224	941.1745	4.2439	1.5776
63	2013	17	16.6143	82.5000	946.8643	3.8878	2.0663
62	2013	17	17.6571	76.5918	874.6418	4.9449	1.4878
1	2013	17	16.8857	77.9796	875.1224	4.1133	2.5255
31	2013	18	15.3000	75.2857	846.2102	3.4337	1.1796
79	2013	18	19.8143	81.3571	979.6480	2.8143	0.9408
51	2013	18	17.3000	85.6327	940.3173	2.6082	1.3500

14	2013	18	16.7143	93.0102	897.8133	1.8510	2.6031
67	2013	18	16.5714	81.9796	902.8602	1.5724	1.9143
42	2013	18	16.2857	82.8571	875.1173	2.4857	2.5745
50	2013	18	17.4000	77.9490	902.1276	2.4255	1.9020
43	2013	18	16.2857	82.8571	875.1173	2.4857	2.5745
85	2013	18	17.6286	81.4184	910.5561	2.9612	1.7449
25	2013	18	20.2286	85.2755	977.6408	2.2439	1.2224
69	2013	18	18.4000	76.8163	940.9694	2.7316	1.4173
57	2013	18	17.0571	87.5918	894.7112	1.6000	1.7102
9	2013	18	16.2143	75.9592	854.4439	3.6296	3.1500
72	2013	18	17.5857	73.2245	877.6418	4.1929	2.8020
26	2013	18	18.1571	72.5816	866.8551	6.3051	2.3867
7	2013	18	17.2000	70.6020	858.8735	4.9633	2.4449
83	2013	18	21.7429	69.5408	943.5918	3.9776	1.1684
76	2013	18	17.5857	80.1429	921.7357	2.3163	1.8082
36	2013	18	17.4857	81.9184	929.6857	2.7163	1.4929
81	2013	18	17.3000	85.6327	940.3173	2.6082	1.3500
15	2013	18	18.4143	72.4286	933.7020	2.8786	0.9857
32	2013	18	16.2857	82.8571	875.1173	2.4857	2.5745
73	2013	18	19.5714	81.2347	963.1429	3.2122	0.8694
71	2013	18	17.4857	81.9184	929.6857	2.7163	1.4929
41	2013	18	16.6714	80.8776	874.6622	3.1653	1.5571
10	2013	18	18.2143	80.3980	965.4582	2.3020	1.3082
23	2013	18	11.7286	68.6837	776.0398	6.2316	2.7418
27	2013	18	17.2000	70.6020	858.8735	4.9633	2.4449
60	2013	18	17.3000	85.6327	940.3173	2.6082	1.3500
53	2013	18	16.2143	75.9592	854.4439	3.6296	3.1500
66	2013	18	16.7143	93.0102	897.8133	1.8510	2.6031
59	2013	18	17.0571	87.5918	894.7112	1.6000	1.7102
61	2013	18	19.5714	81.2347	963.1429	3.2122	0.8694
84	2013	18	19.5714	81.2347	963.1429	3.2122	0.8694
38	2013	18	17.0571	87.5918	894.7112	1.6000	1.7102
87	2013	18	18.3000	71.6327	899.5429	2.8939	1.8235
34	2013	18	17.0571	87.5918	894.7112	1.6000	1.7102
29	2013	18	18.4000	76.8163	940.9694	2.7316	1.4173
5	2013	18	15.2857	69.3367	832.2347	5.4224	2.7000
8	2013	18	16.2143	75.9592	854.4439	3.6296	3.1500
12	2013	18	15.2857	69.3367	832.2347	5.4224	2.7000
13	2013	18	21.7429	69.5408	943.5918	3.9776	1.1684
18	2013	18	18.8143	78.8367	965.5551	2.9082	1.1031
33	2013	18	17.4000	77.9490	902.1276	2.4255	1.9020
56	2013	18	20.2286	85.2755	977.6408	2.2439	1.2224
77	2013	18	18.4143	72.4286	933.7020	2.8786	0.9857
54	2013	18	15.2857	69.3367	832.2347	5.4224	2.7000
21	2013	18	17.4000	77.9490	902.1276	2.4255	1.9020
68	2013	18	19.8143	81.3571	979.6480	2.8143	0.9408
74	2013	18	19.5714	81.2347	963.1429	3.2122	0.8694
88	2013	18	16.2857	82.8571	875.1173	2.4857	2.5745
16	2013	18	17.5857	80.1429	921.7357	2.3163	1.8082
30	2013	18	16.7143	93.0102	897.8133	1.8510	2.6031

6	2013	18	19.8143	81.3571	979.6480	2.8143	0.9408
49	2013	18	18.4000	76.8163	940.9694	2.7316	1.4173
22	2013	18	16.2857	82.8571	875.1173	2.4857	2.5745
45	2013	18	15.5143	63.5000	817.5939	6.7245	2.4888
58	2013	18	18.4000	76.8163	940.9694	2.7316	1.4173
37	2013	18	19.8143	81.3571	979.6480	2.8143	0.9408
17	2013	18	16.5714	81.9796	902.8602	1.5724	1.9143
55	2013	18	17.5857	73.2245	877.6418	4.1929	2.8020
46	2013	18	17.5857	80.1429	921.7357	2.3163	1.8082
86	2013	18	17.3000	72.0204	866.8398	2.9316	1.3296
2	2013	18	17.3000	72.0204	866.8398	2.9316	1.3296
4	2013	18	17.4000	77.9490	902.1276	2.4255	1.9020
47	2013	18	21.3429	75.9184	958.7296	3.3878	0.9459
82	2013	18	16.2857	82.8571	875.1173	2.4857	2.5745
19	2013	18	20.4286	80.4388	960.7500	2.3163	1.0347
20	2013	18	16.2143	75.9592	854.4439	3.6296	3.1500
80	2013	18	16.2857	82.8571	875.1173	2.4857	2.5745
3	2013	18	21.7429	69.5408	943.5918	3.9776	1.1684
52	2013	18	16.5714	81.9796	902.8602	1.5724	1.9143
70	2013	18	17.6286	81.4184	910.5561	2.9612	1.7449
64	2013	18	11.7286	68.6837	776.0398	6.2316	2.7418
48	2013	18	18.4143	72.4286	933.7020	2.8786	0.9857
65	2013	18	16.5714	81.9796	902.8602	1.5724	1.9143
44	2013	18	17.6286	81.4184	910.5561	2.9612	1.7449
75	2013	18	11.7286	68.6837	776.0398	6.2316	2.7418
40	2013	18	17.8714	87.5306	946.6949	2.4235	1.8786
11	2013	18	17.5857	73.2245	877.6418	4.1929	2.8020
35	2013	18	17.3000	85.6327	940.3173	2.6082	1.3500
78	2013	18	18.3000	71.6327	899.5429	2.8939	1.8235
28	2013	18	17.4857	81.9184	929.6857	2.7163	1.4929
39	2013	18	16.5714	81.9796	902.8602	1.5724	1.9143
24	2013	18	18.4000	76.8163	940.9694	2.7316	1.4173
63	2013	18	17.8714	87.5306	946.6949	2.4235	1.8786
62	2013	18	16.6714	80.8776	874.6622	3.1653	1.5571
1	2013	18	16.2857	82.8571	875.1173	2.4857	2.5745
31	2013	19	16.0143	79.0510	846.9102	1.9704	1.1337
79	2013	19	21.7429	83.4286	978.7000	2.2306	0.7929
51	2013	19	19.5143	85.4388	939.9061	2.2296	1.0949
14	2013	19	18.9143	93.6633	898.1490	1.5459	2.3429
67	2013	19	18.4857	82.9286	902.9939	1.0653	1.9235
42	2013	19	17.1571	84.3061	875.6367	1.9806	2.4724
50	2013	19	18.4143	80.3265	902.5755	1.5439	1.9694
43	2013	19	17.1571	84.3061	875.6367	1.9806	2.4724
85	2013	19	18.7714	85.6122	911.0908	2.4429	1.7061
25	2013	19	22.8286	88.1224	977.3357	2.3908	1.0133
69	2013	19	19.9000	77.3163	940.6786	1.9306	1.1245
57	2013	19	17.8286	87.8469	895.0918	1.4929	1.6347
9	2013	19	17.1286	79.5000	855.0622	2.7286	2.9061
72	2013	19	18.4286	77.1531	878.3449	3.0990	2.4031
26	2013	19	19.5857	83.9082	867.7041	4.2214	2.0684

7	2013	19	18.5143	79.1939	859.6745	3.0194	1.7551
83	2013	19	22.2714	74.9694	944.5990	3.4714	1.0888
76	2013	19	19.0000	81.6429	921.7592	1.6388	1.5724
36	2013	19	19.3857	83.6429	929.6296	2.0347	1.2296
81	2013	19	19.5143	85.4388	939.9061	2.2296	1.0949
15	2013	19	19.6286	77.1531	933.8969	2.0408	0.7031
32	2013	19	17.1571	84.3061	875.6367	1.9806	2.4724
73	2013	19	21.2286	82.8980	962.7245	2.7765	0.7102
71	2013	19	19.3857	83.6429	929.6296	2.0347	1.2296
41	2013	19	17.6000	83.1735	875.2061	2.2214	1.3745
10	2013	19	20.2714	81.4694	964.7755	1.7929	1.2031
23	2013	19	12.4429	79.8571	776.5571	3.6051	2.5184
27	2013	19	18.5143	79.1939	859.6745	3.0194	1.7551
60	2013	19	19.5143	85.4388	939.9061	2.2296	1.0949
53	2013	19	17.1286	79.5000	855.0622	2.7286	2.9061
66	2013	19	18.9143	93.6633	898.1490	1.5459	2.3429
59	2013	19	17.8286	87.8469	895.0918	1.4929	1.6347
61	2013	19	21.2286	82.8980	962.7245	2.7765	0.7102
84	2013	19	21.2286	82.8980	962.7245	2.7765	0.7102
38	2013	19	17.8286	87.8469	895.0918	1.4929	1.6347
87	2013	19	19.1571	74.0306	899.9622	1.8765	1.6878
34	2013	19	17.8286	87.8469	895.0918	1.4929	1.6347
29	2013	19	19.9000	77.3163	940.6786	1.9306	1.1245
5	2013	19	16.4714	79.1939	832.9235	3.1735	2.0714
8	2013	19	17.1286	79.5000	855.0622	2.7286	2.9061
12	2013	19	16.4714	79.1939	832.9235	3.1735	2.0714
13	2013	19	22.2714	74.9694	944.5990	3.4714	1.0888
18	2013	19	20.2429	81.3980	964.8469	2.2663	0.9602
33	2013	19	18.4143	80.3265	902.5755	1.5439	1.9694
56	2013	19	22.8286	88.1224	977.3357	2.3908	1.0133
77	2013	19	19.6286	77.1531	933.8969	2.0408	0.7031
54	2013	19	16.4714	79.1939	832.9235	3.1735	2.0714
21	2013	19	18.4143	80.3265	902.5755	1.5439	1.9694
68	2013	19	21.7429	83.4286	978.7000	2.2306	0.7929
74	2013	19	21.2286	82.8980	962.7245	2.7765	0.7102
88	2013	19	17.1571	84.3061	875.6367	1.9806	2.4724
16	2013	19	19.0000	81.6429	921.7592	1.6388	1.5724
30	2013	19	18.9143	93.6633	898.1490	1.5459	2.3429
6	2013	19	21.7429	83.4286	978.7000	2.2306	0.7929
49	2013	19	19.9000	77.3163	940.6786	1.9306	1.1245
22	2013	19	17.1571	84.3061	875.6367	1.9806	2.4724
45	2013	19	16.5000	77.8980	818.1653	3.7398	2.0714
58	2013	19	19.9000	77.3163	940.6786	1.9306	1.1245
37	2013	19	21.7429	83.4286	978.7000	2.2306	0.7929
17	2013	19	18.4857	82.9286	902.9939	1.0653	1.9235
55	2013	19	18.4286	77.1531	878.3449	3.0990	2.4031
46	2013	19	19.0000	81.6429	921.7592	1.6388	1.5724
86	2013	19	17.6143	76.0000	867.5010	2.2888	1.2510
2	2013	19	17.6143	76.0000	867.5010	2.2888	1.2510
4	2013	19	18.4143	80.3265	902.5755	1.5439	1.9694

47	2013	19	22.9857	80.6531	959.4112	3.0469	0.8235
82	2013	19	17.1571	84.3061	875.6367	1.9806	2.4724
19	2013	19	22.5429	79.7245	960.7490	2.1480	0.8837
20	2013	19	17.1286	79.5000	855.0622	2.7286	2.9061
80	2013	19	17.1571	84.3061	875.6367	1.9806	2.4724
3	2013	19	22.2714	74.9694	944.5990	3.4714	1.0888
52	2013	19	18.4857	82.9286	902.9939	1.0653	1.9235
70	2013	19	18.7714	85.6122	911.0908	2.4429	1.7061
64	2013	19	12.4429	79.8571	776.5571	3.6051	2.5184
48	2013	19	19.6286	77.1531	933.8969	2.0408	0.7031
65	2013	19	18.4857	82.9286	902.9939	1.0653	1.9235
44	2013	19	18.7714	85.6122	911.0908	2.4429	1.7061
75	2013	19	12.4429	79.8571	776.5571	3.6051	2.5184
40	2013	19	20.6000	88.6327	946.3041	2.0724	1.6092
11	2013	19	18.4286	77.1531	878.3449	3.0990	2.4031
35	2013	19	19.5143	85.4388	939.9061	2.2296	1.0949
78	2013	19	19.1571	74.0306	899.9622	1.8765	1.6878
28	2013	19	19.3857	83.6429	929.6296	2.0347	1.2296
39	2013	19	18.4857	82.9286	902.9939	1.0653	1.9235
24	2013	19	19.9000	77.3163	940.6786	1.9306	1.1245
63	2013	19	20.6000	88.6327	946.3041	2.0724	1.6092
62	2013	19	17.6000	83.1735	875.2061	2.2214	1.3745
1	2013	19	17.1571	84.3061	875.6367	1.9806	2.4724
31	2013	20	18.2429	76.1531	845.7082	3.0071	1.0878
79	2013	20	21.7857	83.0612	976.8265	2.2133	0.7776
51	2013	20	19.8286	83.6837	938.1806	3.0561	1.2133
14	2013	20	20.5429	90.0204	896.5878	2.7724	2.2980
67	2013	20	19.7429	80.2245	901.4224	2.2806	2.6847
42	2013	20	19.5143	81.6020	874.2673	3.4122	2.4694
50	2013	20	19.5571	77.7755	901.1480	3.2224	2.0398
43	2013	20	19.5143	81.6020	874.2673	3.4122	2.4694
85	2013	20	20.7714	81.9796	909.5837	3.6112	1.7316
25	2013	20	23.6429	84.3469	975.3133	4.0776	1.1561
69	2013	20	20.9714	74.4898	938.9041	2.6316	1.3010
57	2013	20	20.2000	84.2653	893.6224	2.9112	1.7806
9	2013	20	20.2714	76.1224	853.7918	4.4133	2.6051
72	2013	20	21.5857	76.3163	876.9714	4.6102	2.2143
26	2013	20	23.9857	80.2755	866.4276	5.4571	2.1316
7	2013	20	22.1571	76.3980	858.3898	3.9939	1.3520
83	2013	20	26.2143	73.8061	942.9235	4.5388	1.1000
76	2013	20	19.8000	80.2551	920.2367	2.9102	1.7408
36	2013	20	20.4429	81.8673	927.9684	3.4408	1.2990
81	2013	20	19.8286	83.6837	938.1806	3.0561	1.2133
15	2013	20	20.5571	76.0102	932.3255	2.5153	0.7418
32	2013	20	19.5143	81.6020	874.2673	3.4122	2.4694
73	2013	20	21.4143	82.0918	960.9776	3.2235	0.7265
71	2013	20	20.4429	81.8673	927.9684	3.4408	1.2990
41	2013	20	19.4714	81.5204	873.8786	3.6092	1.3041
10	2013	20	20.6857	79.6633	962.8765	2.7673	1.2031
23	2013	20	17.1143	74.6327	775.7071	4.5837	2.2224

27	2013	20	22.1571	76.3980	858.3898	3.9939	1.3520
60	2013	20	19.8286	83.6837	938.1806	3.0561	1.2133
53	2013	20	20.2714	76.1224	853.7918	4.4133	2.6051
66	2013	20	20.5429	90.0204	896.5878	2.7724	2.2980
59	2013	20	20.2000	84.2653	893.6224	2.9112	1.7806
61	2013	20	21.4143	82.0918	960.9776	3.2235	0.7265
84	2013	20	21.4143	82.0918	960.9776	3.2235	0.7265
38	2013	20	20.2000	84.2653	893.6224	2.9112	1.7806
87	2013	20	21.9571	72.3776	898.4490	3.2827	1.8673
34	2013	20	20.2000	84.2653	893.6224	2.9112	1.7806
29	2013	20	20.9714	74.4898	938.9041	2.6316	1.3010
5	2013	20	20.3429	74.9184	831.7949	4.3408	1.9214
8	2013	20	20.2714	76.1224	853.7918	4.4133	2.6051
12	2013	20	20.3429	74.9184	831.7949	4.3408	1.9214
13	2013	20	26.2143	73.8061	942.9235	4.5388	1.1000
18	2013	20	20.4714	82.1633	963.0449	2.6990	1.0378
33	2013	20	19.5571	77.7755	901.1480	3.2224	2.0398
56	2013	20	23.6429	84.3469	975.3133	4.0776	1.1561
77	2013	20	20.5571	76.0102	932.3255	2.5153	0.7418
54	2013	20	20.3429	74.9184	831.7949	4.3408	1.9214
21	2013	20	19.5571	77.7755	901.1480	3.2224	2.0398
68	2013	20	21.7857	83.0612	976.8265	2.2133	0.7776
74	2013	20	21.4143	82.0918	960.9776	3.2235	0.7265
88	2013	20	19.5143	81.6020	874.2673	3.4122	2.4694
16	2013	20	19.8000	80.2551	920.2367	2.9102	1.7408
30	2013	20	20.5429	90.0204	896.5878	2.7724	2.2980
6	2013	20	21.7857	83.0612	976.8265	2.2133	0.7776
49	2013	20	20.9714	74.4898	938.9041	2.6316	1.3010
22	2013	20	19.5143	81.6020	874.2673	3.4122	2.4694
45	2013	20	21.6429	71.7959	817.1480	5.0806	2.1357
58	2013	20	20.9714	74.4898	938.9041	2.6316	1.3010
37	2013	20	21.7857	83.0612	976.8265	2.2133	0.7776
17	2013	20	19.7429	80.2245	901.4224	2.2806	2.6847
55	2013	20	21.5857	76.3163	876.9714	4.6102	2.2143
46	2013	20	19.8000	80.2551	920.2367	2.9102	1.7408
86	2013	20	20.6143	73.1837	866.2173	3.8918	1.2908
2	2013	20	20.6143	73.1837	866.2173	3.8918	1.2908
4	2013	20	19.5571	77.7755	901.1480	3.2224	2.0398
47	2013	20	25.8143	77.7551	957.5551	4.1143	0.8490
82	2013	20	19.5143	81.6020	874.2673	3.4122	2.4694
19	2013	20	24.3857	75.4286	958.7898	3.7041	1.0582
20	2013	20	20.2714	76.1224	853.7918	4.4133	2.6051
80	2013	20	19.5143	81.6020	874.2673	3.4122	2.4694
3	2013	20	26.2143	73.8061	942.9235	4.5388	1.1000
52	2013	20	19.7429	80.2245	901.4224	2.2806	2.6847
70	2013	20	20.7714	81.9796	909.5837	3.6112	1.7316
64	2013	20	17.1143	74.6327	775.7071	4.5837	2.2224
48	2013	20	20.5571	76.0102	932.3255	2.5153	0.7418
65	2013	20	19.7429	80.2245	901.4224	2.2806	2.6847
44	2013	20	20.7714	81.9796	909.5837	3.6112	1.7316

75	2013	20	17.1143	74.6327	775.7071	4.5837	2.2224
40	2013	20	20.8143	85.2449	944.4857	3.7949	1.7510
11	2013	20	21.5857	76.3163	876.9714	4.6102	2.2143
35	2013	20	19.8286	83.6837	938.1806	3.0561	1.2133
78	2013	20	21.9571	72.3776	898.4490	3.2827	1.8673
28	2013	20	20.4429	81.8673	927.9684	3.4408	1.2990
39	2013	20	19.7429	80.2245	901.4224	2.2806	2.6847
24	2013	20	20.9714	74.4898	938.9041	2.6316	1.3010
63	2013	20	20.8143	85.2449	944.4857	3.7949	1.7510
62	2013	20	19.4714	81.5204	873.8786	3.6092	1.3041
1	2013	20	19.5143	81.6020	874.2673	3.4122	2.4694
31	2013	21	20.1286	73.2959	844.7031	4.6918	1.0755
79	2013	21	24.8714	79.5510	974.7827	3.3908	0.8857
51	2013	21	22.4857	80.5408	936.4531	4.5827	1.1306
14	2013	21	22.4143	86.7143	895.1235	3.8918	2.0429
67	2013	21	22.7429	75.8367	900.0173	2.8316	2.6480
42	2013	21	21.7143	76.7653	873.0112	4.4429	2.4133
50	2013	21	22.4429	74.6020	899.6592	4.9898	1.8102
43	2013	21	21.7143	76.7653	873.0112	4.4429	2.4133
85	2013	21	23.1429	77.8878	908.0633	4.7867	1.5510
25	2013	21	25.2429	82.4796	973.4153	4.6327	1.2816
69	2013	21	23.1714	71.8980	937.1898	3.9490	1.3765
57	2013	21	22.4143	80.7245	892.2398	4.0949	1.7633
9	2013	21	21.4000	72.5306	852.7051	5.3561	2.3663
72	2013	21	21.9857	74.1735	875.6408	4.8663	2.1582
26	2013	21	22.3857	77.2755	865.2020	6.7112	2.0980
7	2013	21	21.7000	74.7653	857.2602	4.3439	1.2520
83	2013	21	25.4857	73.1633	941.1092	5.2480	1.0837
76	2013	21	22.7571	76.7755	918.6000	4.7796	1.6908
36	2013	21	23.1571	79.0510	926.3367	5.0255	1.4418
81	2013	21	22.4857	80.5408	936.4531	4.5827	1.1306
15	2013	21	23.2571	71.7653	930.5531	4.1265	0.7122
32	2013	21	21.7143	76.7653	873.0112	4.4429	2.4133
73	2013	21	24.5429	79.3878	959.0735	4.1204	0.8173
71	2013	21	23.1571	79.0510	926.3367	5.0255	1.4418
41	2013	21	22.0429	77.8878	872.6714	5.2582	1.3286
10	2013	21	23.7714	75.2041	960.9153	4.2867	1.1806
23	2013	21	17.2143	71.2041	775.1633	5.9173	2.0939
27	2013	21	21.7000	74.7653	857.2602	4.3439	1.2520
60	2013	21	22.4857	80.5408	936.4531	4.5827	1.1306
53	2013	21	21.4000	72.5306	852.7051	5.3561	2.3663
66	2013	21	22.4143	86.7143	895.1235	3.8918	2.0429
59	2013	21	22.4143	80.7245	892.2398	4.0949	1.7633
61	2013	21	24.5429	79.3878	959.0735	4.1204	0.8173
84	2013	21	24.5429	79.3878	959.0735	4.1204	0.8173
38	2013	21	22.4143	80.7245	892.2398	4.0949	1.7633
87	2013	21	23.1286	69.7857	896.9867	4.1245	2.0653
34	2013	21	22.4143	80.7245	892.2398	4.0949	1.7633
29	2013	21	23.1714	71.8980	937.1898	3.9490	1.3765
5	2013	21	20.1000	73.4796	830.9133	5.3031	1.8469

8	2013	21	21.4000	72.5306	852.7051	5.3561	2.3663
12	2013	21	20.1000	73.4796	830.9133	5.3031	1.8469
13	2013	21	25.4857	73.1633	941.1092	5.2480	1.0837
18	2013	21	23.7429	79.0612	961.0061	3.8480	1.0837
33	2013	21	22.4429	74.6020	899.6592	4.9898	1.8102
56	2013	21	25.2429	82.4796	973.4153	4.6327	1.2816
77	2013	21	23.2571	71.7653	930.5531	4.1265	0.7122
54	2013	21	20.1000	73.4796	830.9133	5.3031	1.8469
21	2013	21	22.4429	74.6020	899.6592	4.9898	1.8102
68	2013	21	24.8714	79.5510	974.7827	3.3908	0.8857
74	2013	21	24.5429	79.3878	959.0735	4.1204	0.8173
88	2013	21	21.7143	76.7653	873.0112	4.4429	2.4133
16	2013	21	22.7571	76.7755	918.6000	4.7796	1.6908
30	2013	21	22.4143	86.7143	895.1235	3.8918	2.0429
6	2013	21	24.8714	79.5510	974.7827	3.3908	0.8857
49	2013	21	23.1714	71.8980	937.1898	3.9490	1.3765
22	2013	21	21.7143	76.7653	873.0112	4.4429	2.4133
45	2013	21	19.7857	69.7143	816.3296	6.5184	2.0296
58	2013	21	23.1714	71.8980	937.1898	3.9490	1.3765
37	2013	21	24.8714	79.5510	974.7827	3.3908	0.8857
17	2013	21	22.7429	75.8367	900.0173	2.8316	2.6480
55	2013	21	21.9857	74.1735	875.6408	4.8663	2.1582
46	2013	21	22.7571	76.7755	918.6000	4.7796	1.6908
86	2013	21	22.2000	71.2347	865.2306	5.0449	1.1888
2	2013	21	22.2000	71.2347	865.2306	5.0449	1.1888
4	2013	21	22.4429	74.6020	899.6592	4.9898	1.8102
47	2013	21	26.2714	76.8163	955.5980	3.5684	0.7673
82	2013	21	21.7143	76.7653	873.0112	4.4429	2.4133
19	2013	21	25.2857	73.4286	956.8827	4.2112	1.1561
20	2013	21	21.4000	72.5306	852.7051	5.3561	2.3663
80	2013	21	21.7143	76.7653	873.0112	4.4429	2.4133
3	2013	21	25.4857	73.1633	941.1092	5.2480	1.0837
52	2013	21	22.7429	75.8367	900.0173	2.8316	2.6480
70	2013	21	23.1429	77.8878	908.0633	4.7867	1.5510
64	2013	21	17.2143	71.2041	775.1633	5.9173	2.0939
48	2013	21	23.2571	71.7653	930.5531	4.1265	0.7122
65	2013	21	22.7429	75.8367	900.0173	2.8316	2.6480
44	2013	21	23.1429	77.8878	908.0633	4.7867	1.5510
75	2013	21	17.2143	71.2041	775.1633	5.9173	2.0939
40	2013	21	23.6143	82.0714	942.7520	5.1347	1.6694
11	2013	21	21.9857	74.1735	875.6408	4.8663	2.1582
35	2013	21	22.4857	80.5408	936.4531	4.5827	1.1306
78	2013	21	23.1286	69.7857	896.9867	4.1245	2.0653
28	2013	21	23.1571	79.0510	926.3367	5.0255	1.4418
39	2013	21	22.7429	75.8367	900.0173	2.8316	2.6480
24	2013	21	23.1714	71.8980	937.1898	3.9490	1.3765
63	2013	21	23.6143	82.0714	942.7520	5.1347	1.6694
62	2013	21	22.0429	77.8878	872.6714	5.2582	1.3286
1	2013	21	21.7143	76.7653	873.0112	4.4429	2.4133
31	2013	22	18.0857	73.1633	844.7776	5.4959	1.2112

79	2013	22	23.6143	79.3265	974.2235	3.9173	0.9949
51	2013	22	21.6286	80.2653	936.1571	5.2143	1.1918
14	2013	22	20.6143	87.3265	895.2398	4.3082	2.4571
67	2013	22	20.3000	74.9694	899.9602	3.2837	2.7837
42	2013	22	19.4000	76.1429	873.0816	4.7888	2.4776
50	2013	22	20.4286	74.5918	899.4755	5.3092	1.9908
43	2013	22	19.4000	76.1429	873.0816	4.7888	2.4776
85	2013	22	21.1286	76.8367	907.8102	5.3735	1.7714
25	2013	22	24.4857	83.1837	973.1265	4.7837	1.2827
69	2013	22	22.0571	72.3878	936.8316	4.3163	1.2867
57	2013	22	20.2714	78.9286	892.1418	3.6204	1.8480
9	2013	22	19.3143	73.8367	852.9112	5.3837	2.4184
72	2013	22	20.1714	74.8163	875.7796	5.1204	2.2449
26	2013	22	21.6286	83.0204	865.5439	6.9724	2.2357
7	2013	22	20.9286	75.6531	857.5418	4.8500	1.6847
83	2013	22	24.6857	75.5306	941.2398	6.1704	1.0837
76	2013	22	21.0429	76.8367	918.2867	5.2082	1.5173
36	2013	22	21.8143	78.0510	926.0724	5.5398	1.4673
81	2013	22	21.6286	80.2653	936.1571	5.2143	1.1918
15	2013	22	22.2143	70.9592	930.1653	4.4163	0.7449
32	2013	22	19.4000	76.1429	873.0816	4.7888	2.4776
73	2013	22	22.9571	78.8265	958.5245	4.4071	0.8214
71	2013	22	21.8143	78.0510	926.0724	5.5398	1.4673
41	2013	22	20.0571	75.4592	872.6429	5.9010	1.5459
10	2013	22	22.5286	74.6735	960.4388	4.2724	1.1990
23	2013	22	14.9143	73.5510	775.5684	5.7898	2.2531
27	2013	22	20.9286	75.6531	857.5418	4.8500	1.6847
60	2013	22	21.6286	80.2653	936.1571	5.2143	1.1918
53	2013	22	19.3143	73.8367	852.9112	5.3837	2.4184
66	2013	22	20.6143	87.3265	895.2398	4.3082	2.4571
59	2013	22	20.2714	78.9286	892.1418	3.6204	1.8480
61	2013	22	22.9571	78.8265	958.5245	4.4071	0.8214
84	2013	22	22.9571	78.8265	958.5245	4.4071	0.8214
38	2013	22	20.2714	78.9286	892.1418	3.6204	1.8480
87	2013	22	21.3143	70.3878	897.0296	3.9010	2.0112
34	2013	22	20.2714	78.9286	892.1418	3.6204	1.8480
29	2013	22	22.0571	72.3878	936.8316	4.3163	1.2867
5	2013	22	18.7000	75.3571	831.2214	5.8500	1.8459
8	2013	22	19.3143	73.8367	852.9112	5.3837	2.4184
12	2013	22	18.7000	75.3571	831.2214	5.8500	1.8459
13	2013	22	24.6857	75.5306	941.2398	6.1704	1.0837
18	2013	22	22.7000	77.6020	960.4724	4.0520	0.9582
33	2013	22	20.4286	74.5918	899.4755	5.3092	1.9908
56	2013	22	24.4857	83.1837	973.1265	4.7837	1.2827
77	2013	22	22.2143	70.9592	930.1653	4.4163	0.7449
54	2013	22	18.7000	75.3571	831.2214	5.8500	1.8459
21	2013	22	20.4286	74.5918	899.4755	5.3092	1.9908
68	2013	22	23.6143	79.3265	974.2235	3.9173	0.9949
74	2013	22	22.9571	78.8265	958.5245	4.4071	0.8214
88	2013	22	19.4000	76.1429	873.0816	4.7888	2.4776

16	2013	22	21.0429	76.8367	918.2867	5.2082	1.5173
30	2013	22	20.6143	87.3265	895.2398	4.3082	2.4571
6	2013	22	23.6143	79.3265	974.2235	3.9173	0.9949
49	2013	22	22.0571	72.3878	936.8316	4.3163	1.2867
22	2013	22	19.4000	76.1429	873.0816	4.7888	2.4776
45	2013	22	19.3429	75.0204	816.6918	6.5878	2.0224
58	2013	22	22.0571	72.3878	936.8316	4.3163	1.2867
37	2013	22	23.6143	79.3265	974.2235	3.9173	0.9949
17	2013	22	20.3000	74.9694	899.9602	3.2837	2.7837
55	2013	22	20.1714	74.8163	875.7796	5.1204	2.2449
46	2013	22	21.0429	76.8367	918.2867	5.2082	1.5173
86	2013	22	19.9000	72.2449	865.2959	5.5367	1.3061
2	2013	22	19.9000	72.2449	865.2959	5.5367	1.3061
4	2013	22	20.4286	74.5918	899.4755	5.3092	1.9908
47	2013	22	24.7429	78.7449	955.5327	3.3061	0.7010
82	2013	22	19.4000	76.1429	873.0816	4.7888	2.4776
19	2013	22	23.7857	75.5510	956.8898	4.1133	1.1255
20	2013	22	19.3143	73.8367	852.9112	5.3837	2.4184
80	2013	22	19.4000	76.1429	873.0816	4.7888	2.4776
3	2013	22	24.6857	75.5306	941.2398	6.1704	1.0837
52	2013	22	20.3000	74.9694	899.9602	3.2837	2.7837
70	2013	22	21.1286	76.8367	907.8102	5.3735	1.7714
64	2013	22	14.9143	73.5510	775.5684	5.7898	2.2531
48	2013	22	22.2143	70.9592	930.1653	4.4163	0.7449
65	2013	22	20.3000	74.9694	899.9602	3.2837	2.7837
44	2013	22	21.1286	76.8367	907.8102	5.3735	1.7714
75	2013	22	14.9143	73.5510	775.5684	5.7898	2.2531
40	2013	22	22.5000	81.7041	942.5296	5.5224	1.7439
11	2013	22	20.1714	74.8163	875.7796	5.1204	2.2449
35	2013	22	21.6286	80.2653	936.1571	5.2143	1.1918
78	2013	22	21.3143	70.3878	897.0296	3.9010	2.0112
28	2013	22	21.8143	78.0510	926.0724	5.5398	1.4673
39	2013	22	20.3000	74.9694	899.9602	3.2837	2.7837
24	2013	22	22.0571	72.3878	936.8316	4.3163	1.2867
63	2013	22	22.5000	81.7041	942.5296	5.5224	1.7439
62	2013	22	20.0571	75.4592	872.6429	5.9010	1.5459
1	2013	22	19.4000	76.1429	873.0816	4.7888	2.4776
31	2013	23	20.1429	74.5714	845.2816	4.5163	1.2000
79	2013	23	25.0286	79.6429	974.6990	3.8622	0.9796
51	2013	23	23.5714	80.2347	936.6551	4.8602	1.1776
14	2013	23	22.0571	91.2755	895.9622	3.5357	2.5714
67	2013	23	22.1571	79.2959	900.6071	2.8816	2.9969
42	2013	23	21.4571	79.9796	873.6827	4.5041	2.2735
50	2013	23	22.2571	74.5306	899.9755	4.9357	2.1633
43	2013	23	21.4571	79.9796	873.6827	4.5041	2.2735
85	2013	23	23.4429	75.9796	908.2276	5.0571	1.7878
25	2013	23	25.5143	85.8878	973.8133	3.7582	1.2388
69	2013	23	24.2143	72.3061	937.3571	4.0908	1.2224
57	2013	23	22.4714	80.2143	892.6724	3.1020	1.7592
9	2013	23	21.1000	76.5000	853.5235	4.5735	2.2878

72	2013	23	22.1286	77.2551	876.4867	4.5643	2.1020
26	2013	23	22.1143	87.6224	866.3306	5.3653	2.1898
7	2013	23	21.9857	77.7347	858.2571	4.5429	1.8796
83	2013	23	26.2000	76.6837	942.1469	5.7582	1.0286
76	2013	23	22.9571	76.9898	918.7878	4.6112	1.4837
36	2013	23	23.7000	79.5510	926.6745	4.5949	1.3439
81	2013	23	23.5714	80.2347	936.6551	4.8602	1.1776
15	2013	23	23.6143	69.5918	930.6051	4.1745	0.8143
32	2013	23	21.4571	79.9796	873.6827	4.5041	2.2735
73	2013	23	24.7571	79.8265	959.0000	4.2878	0.8388
71	2013	23	23.7000	79.5510	926.6745	4.5949	1.3439
41	2013	23	21.8714	76.5408	873.1816	4.6327	1.5520
10	2013	23	24.3857	76.0102	960.9112	3.5245	1.2286
23	2013	23	16.9714	76.5918	776.1653	4.7194	2.3143
27	2013	23	21.9857	77.7347	858.2571	4.5429	1.8796
60	2013	23	23.5714	80.2347	936.6551	4.8602	1.1776
53	2013	23	21.1000	76.5000	853.5235	4.5735	2.2878
66	2013	23	22.0571	91.2755	895.9622	3.5357	2.5714
59	2013	23	22.4714	80.2143	892.6724	3.1020	1.7592
61	2013	23	24.7571	79.8265	959.0000	4.2878	0.8388
84	2013	23	24.7571	79.8265	959.0000	4.2878	0.8388
38	2013	23	22.4714	80.2143	892.6724	3.1020	1.7592
87	2013	23	23.4000	73.4286	897.7316	3.6531	1.7898
34	2013	23	22.4714	80.2143	892.6724	3.1020	1.7592
29	2013	23	24.2143	72.3061	937.3571	4.0908	1.2224
5	2013	23	20.5000	78.3469	831.7612	4.8153	1.6663
8	2013	23	21.1000	76.5000	853.5235	4.5735	2.2878
12	2013	23	20.5000	78.3469	831.7612	4.8153	1.6663
13	2013	23	26.2000	76.6837	942.1469	5.7582	1.0286
18	2013	23	23.7857	77.5918	960.9980	3.6908	0.6092
33	2013	23	22.2571	74.5306	899.9755	4.9357	2.1633
56	2013	23	25.5143	85.8878	973.8133	3.7582	1.2388
77	2013	23	23.6143	69.5918	930.6051	4.1745	0.8143
54	2013	23	20.5000	78.3469	831.7612	4.8153	1.6663
21	2013	23	22.2571	74.5306	899.9755	4.9357	2.1633
68	2013	23	25.0286	79.6429	974.6990	3.8622	0.9796
74	2013	23	24.7571	79.8265	959.0000	4.2878	0.8388
88	2013	23	21.4571	79.9796	873.6827	4.5041	2.2735
16	2013	23	22.9571	76.9898	918.7878	4.6112	1.4837
30	2013	23	22.0571	91.2755	895.9622	3.5357	2.5714
6	2013	23	25.0286	79.6429	974.6990	3.8622	0.9796
49	2013	23	24.2143	72.3061	937.3571	4.0908	1.2224
22	2013	23	21.4571	79.9796	873.6827	4.5041	2.2735
45	2013	23	20.1000	79.3469	817.3469	5.1388	1.7449
58	2013	23	24.2143	72.3061	937.3571	4.0908	1.2224
37	2013	23	25.0286	79.6429	974.6990	3.8622	0.9796
17	2013	23	22.1571	79.2959	900.6071	2.8816	2.9969
55	2013	23	22.1286	77.2551	876.4867	4.5643	2.1020
46	2013	23	22.9571	76.9898	918.7878	4.6112	1.4837
86	2013	23	21.7571	73.8571	865.7133	4.5704	1.2867

2	2013	23	21.7571	73.8571	865.7133	4.5704	1.2867
4	2013	23	22.2571	74.5306	899.9755	4.9357	2.1633
47	2013	23	26.0857	82.1531	956.4235	3.3929	0.6939
82	2013	23	21.4571	79.9796	873.6827	4.5041	2.2735
19	2013	23	25.5143	78.8673	957.7418	3.3969	0.9469
20	2013	23	21.1000	76.5000	853.5235	4.5735	2.2878
80	2013	23	21.4571	79.9796	873.6827	4.5041	2.2735
3	2013	23	26.2000	76.6837	942.1469	5.7582	1.0286
52	2013	23	22.1571	79.2959	900.6071	2.8816	2.9969
70	2013	23	23.4429	75.9796	908.2276	5.0571	1.7878
64	2013	23	16.9714	76.5918	776.1653	4.7194	2.3143
48	2013	23	23.6143	69.5918	930.6051	4.1745	0.8143
65	2013	23	22.1571	79.2959	900.6071	2.8816	2.9969
44	2013	23	23.4429	75.9796	908.2276	5.0571	1.7878
75	2013	23	16.9714	76.5918	776.1653	4.7194	2.3143
40	2013	23	23.9429	84.0612	943.1490	4.8551	1.8071
11	2013	23	22.1286	77.2551	876.4867	4.5643	2.1020
35	2013	23	23.5714	80.2347	936.6551	4.8602	1.1776
78	2013	23	23.4000	73.4286	897.7316	3.6531	1.7898
28	2013	23	23.7000	79.5510	926.6745	4.5949	1.3439
39	2013	23	22.1571	79.2959	900.6071	2.8816	2.9969
24	2013	23	24.2143	72.3061	937.3571	4.0908	1.2224
63	2013	23	23.9429	84.0612	943.1490	4.8551	1.8071
62	2013	23	21.8714	76.5408	873.1816	4.6327	1.5520
1	2013	23	21.4571	79.9796	873.6827	4.5041	2.2735
31	2013	24	20.8857	73.5204	845.3633	4.5694	1.3684
79	2013	24	24.1000	80.4388	975.1551	3.9622	0.9561
51	2013	24	21.3000	80.8163	936.9184	4.5510	1.1316
14	2013	24	20.7857	91.0408	895.8949	3.6173	2.2643
67	2013	24	20.7143	80.1939	900.6622	2.8286	2.7837
42	2013	24	21.1857	79.9184	873.6959	5.1000	2.3714
50	2013	24	22.4286	74.9694	900.1449	5.2286	2.1235
43	2013	24	21.1857	79.9184	873.6959	5.1000	2.3714
85	2013	24	23.9143	76.2755	908.3367	5.2724	1.6969
25	2013	24	23.4571	87.1429	973.9796	3.2214	1.1449
69	2013	24	22.6571	71.3163	937.6122	4.5000	1.3571
57	2013	24	21.8571	82.7959	892.7459	4.3918	1.6847
9	2013	24	20.9571	74.0408	853.5122	5.0163	2.2061
72	2013	24	21.1286	74.9490	876.4694	4.7755	2.0531
26	2013	24	21.6714	83.7449	866.3347	4.8888	1.8867
7	2013	24	20.6571	76.1327	858.2827	4.6918	1.7418
83	2013	24	24.5857	75.1429	942.1408	5.8204	0.9255
76	2013	24	22.7429	76.4796	919.0276	4.6255	1.5602
36	2013	24	22.2571	80.8061	926.8490	4.3378	1.2714
81	2013	24	21.3000	80.8163	936.9184	4.5510	1.1316
15	2013	24	23.7714	68.9592	930.8490	4.8551	0.8888
32	2013	24	21.1857	79.9184	873.6959	5.1000	2.3714
73	2013	24	24.5000	79.0102	959.3337	4.6949	0.9418
71	2013	24	22.2571	80.8061	926.8490	4.3378	1.2714
41	2013	24	21.9286	77.2347	873.2602	4.5459	1.5327

10	2013	24	22.3714	77.1122	961.2418	3.6990	1.2816
23	2013	24	17.0286	77.2653	776.2378	5.0520	2.5347
27	2013	24	20.6571	76.1327	858.2827	4.6918	1.7418
60	2013	24	21.3000	80.8163	936.9184	4.5510	1.1316
53	2013	24	20.9571	74.0408	853.5122	5.0163	2.2061
66	2013	24	20.7857	91.0408	895.8949	3.6173	2.2643
59	2013	24	21.8571	82.7959	892.7459	4.3918	1.6847
61	2013	24	24.5000	79.0102	959.3337	4.6949	0.9418
84	2013	24	24.5000	79.0102	959.3337	4.6949	0.9418
38	2013	24	21.8571	82.7959	892.7459	4.3918	1.6847
87	2013	24	22.1429	72.9592	897.7398	4.5949	1.8010
34	2013	24	21.8571	82.7959	892.7459	4.3918	1.6847
29	2013	24	22.6571	71.3163	937.6122	4.5000	1.3571
5	2013	24	19.7857	76.8980	831.7276	4.4959	1.6704
8	2013	24	20.9571	74.0408	853.5122	5.0163	2.2061
12	2013	24	19.7857	76.8980	831.7276	4.4959	1.6704
13	2013	24	24.5857	75.1429	942.1408	5.8204	0.9255
18	2013	24	23.3429	78.2245	961.4724	3.5224	0.6622
33	2013	24	22.4286	74.9694	900.1449	5.2286	2.1235
56	2013	24	23.4571	87.1429	973.9796	3.2214	1.1449
77	2013	24	23.7714	68.9592	930.8490	4.8551	0.8888
54	2013	24	19.7857	76.8980	831.7276	4.4959	1.6704
21	2013	24	22.4286	74.9694	900.1449	5.2286	2.1235
68	2013	24	24.1000	80.4388	975.1551	3.9622	0.9561
74	2013	24	24.5000	79.0102	959.3337	4.6949	0.9418
88	2013	24	21.1857	79.9184	873.6959	5.1000	2.3714
16	2013	24	22.7429	76.4796	919.0276	4.6255	1.5602
30	2013	24	20.7857	91.0408	895.8949	3.6173	2.2643
6	2013	24	24.1000	80.4388	975.1551	3.9622	0.9561
49	2013	24	22.6571	71.3163	937.6122	4.5000	1.3571
22	2013	24	21.1857	79.9184	873.6959	5.1000	2.3714
45	2013	24	19.4571	76.3367	817.4102	4.9367	1.7510
58	2013	24	22.6571	71.3163	937.6122	4.5000	1.3571
37	2013	24	24.1000	80.4388	975.1551	3.9622	0.9561
17	2013	24	20.7143	80.1939	900.6622	2.8286	2.7837
55	2013	24	21.1286	74.9490	876.4694	4.7755	2.0531
46	2013	24	22.7429	76.4796	919.0276	4.6255	1.5602
86	2013	24	21.4429	72.6429	865.7622	4.8755	1.3235
2	2013	24	21.4429	72.6429	865.7622	4.8755	1.3235
4	2013	24	22.4286	74.9694	900.1449	5.2286	2.1235
47	2013	24	24.7000	82.2041	956.4755	4.2092	0.6337
82	2013	24	21.1857	79.9184	873.6959	5.1000	2.3714
19	2013	24	23.2857	79.6224	957.6949	3.5041	0.7480
20	2013	24	20.9571	74.0408	853.5122	5.0163	2.2061
80	2013	24	21.1857	79.9184	873.6959	5.1000	2.3714
3	2013	24	24.5857	75.1429	942.1408	5.8204	0.9255
52	2013	24	20.7143	80.1939	900.6622	2.8286	2.7837
70	2013	24	23.9143	76.2755	908.3367	5.2724	1.6969
64	2013	24	17.0286	77.2653	776.2378	5.0520	2.5347
48	2013	24	23.7714	68.9592	930.8490	4.8551	0.8888

65	2013	24	20.7143	80.1939	900.6622	2.8286	2.7837
44	2013	24	23.9143	76.2755	908.3367	5.2724	1.6969
75	2013	24	17.0286	77.2653	776.2378	5.0520	2.5347
40	2013	24	21.8429	85.8980	943.2724	4.3286	1.6704
11	2013	24	21.1286	74.9490	876.4694	4.7755	2.0531
35	2013	24	21.3000	80.8163	936.9184	4.5510	1.1316
78	2013	24	22.1429	72.9592	897.7398	4.5949	1.8010
28	2013	24	22.2571	80.8061	926.8490	4.3378	1.2714
39	2013	24	20.7143	80.1939	900.6622	2.8286	2.7837
24	2013	24	22.6571	71.3163	937.6122	4.5000	1.3571
63	2013	24	21.8429	85.8980	943.2724	4.3286	1.6704
62	2013	24	21.9286	77.2347	873.2602	4.5459	1.5327
1	2013	24	21.1857	79.9184	873.6959	5.1000	2.3714
31	2013	25	24.9429	67.3980	843.4235	7.4133	1.5459
79	2013	25	30.0857	77.4082	973.0633	6.3582	1.1816
51	2013	25	27.1000	78.2347	935.1959	6.5888	1.2020
14	2013	25	25.2286	87.3163	894.4612	5.9827	2.3418
67	2013	25	25.7571	74.4490	899.0786	5.2469	3.0867
42	2013	25	24.9714	73.0000	872.1439	7.5959	2.7867
50	2013	25	27.4143	70.1020	898.1224	8.5490	2.5663
43	2013	25	24.9714	73.0000	872.1439	7.5959	2.7867
85	2013	25	28.3857	70.1429	906.0194	8.1714	2.4255
25	2013	25	29.1571	82.6327	972.0490	5.5214	1.3102
69	2013	25	28.1857	67.7959	935.6765	7.0837	1.6194
57	2013	25	27.0000	75.8980	890.8112	7.6857	2.1429
9	2013	25	24.2000	68.1122	852.0704	8.1929	2.5888
72	2013	25	24.8143	70.0000	874.9704	7.2184	1.9990
26	2013	25	24.4429	76.2347	864.8908	7.9194	1.9694
7	2013	25	24.3714	71.1429	856.8388	6.9806	1.8327
83	2013	25	29.1000	69.3571	940.2602	8.3000	1.0092
76	2013	25	27.7857	71.2755	917.0857	7.8694	1.7827
36	2013	25	27.6714	75.0000	925.0633	6.8673	1.4633
81	2013	25	27.1000	78.2347	935.1959	6.5888	1.2020
15	2013	25	29.0571	64.3469	928.6949	7.3622	1.0796
32	2013	25	24.9714	73.0000	872.1439	7.5959	2.7867
73	2013	25	29.8571	71.9490	957.1133	7.2204	1.1500
71	2013	25	27.6714	75.0000	925.0633	6.8673	1.4633
41	2013	25	26.0000	72.1327	871.3969	7.6592	2.0133
10	2013	25	28.0857	73.5102	959.3429	6.4337	1.4745
23	2013	25	20.2286	73.0918	774.9735	7.8255	2.7429
27	2013	25	24.3714	71.1429	856.8388	6.9806	1.8327
60	2013	25	27.1000	78.2347	935.1959	6.5888	1.2020
53	2013	25	24.2000	68.1122	852.0704	8.1929	2.5888
66	2013	25	25.2286	87.3163	894.4612	5.9827	2.3418
59	2013	25	27.0000	75.8980	890.8112	7.6857	2.1429
61	2013	25	29.8571	71.9490	957.1133	7.2204	1.1500
84	2013	25	29.8571	71.9490	957.1133	7.2204	1.1500
38	2013	25	27.0000	75.8980	890.8112	7.6857	2.1429
87	2013	25	26.7286	66.6020	896.0469	7.5184	1.8531
34	2013	25	27.0000	75.8980	890.8112	7.6857	2.1429

29	2013	25	28.1857	67.7959	935.6765	7.0837	1.6194
5	2013	25	22.4429	73.2143	830.4796	7.0939	1.9020
8	2013	25	24.2000	68.1122	852.0704	8.1929	2.5888
12	2013	25	22.4429	73.2143	830.4796	7.0939	1.9020
13	2013	25	29.1000	69.3571	940.2602	8.3000	1.0092
18	2013	25	28.9857	73.3673	959.4082	6.4286	1.2184
33	2013	25	27.4143	70.1020	898.1224	8.5490	2.5663
56	2013	25	29.1571	82.6327	972.0490	5.5214	1.3102
77	2013	25	29.0571	64.3469	928.6949	7.3622	1.0796
54	2013	25	22.4429	73.2143	830.4796	7.0939	1.9020
21	2013	25	27.4143	70.1020	898.1224	8.5490	2.5663
68	2013	25	30.0857	77.4082	973.0633	6.3582	1.1816
74	2013	25	29.8571	71.9490	957.1133	7.2204	1.1500
88	2013	25	24.9714	73.0000	872.1439	7.5959	2.7867
16	2013	25	27.7857	71.2755	917.0857	7.8694	1.7827
30	2013	25	25.2286	87.3163	894.4612	5.9827	2.3418
6	2013	25	30.0857	77.4082	973.0633	6.3582	1.1816
49	2013	25	28.1857	67.7959	935.6765	7.0837	1.6194
22	2013	25	24.9714	73.0000	872.1439	7.5959	2.7867
45	2013	25	22.4857	71.2755	816.1214	7.2459	1.7520
58	2013	25	28.1857	67.7959	935.6765	7.0837	1.6194
37	2013	25	30.0857	77.4082	973.0633	6.3582	1.1816
17	2013	25	25.7571	74.4490	899.0786	5.2469	3.0867
55	2013	25	24.8143	70.0000	874.9704	7.2184	1.9990
46	2013	25	27.7857	71.2755	917.0857	7.8694	1.7827
86	2013	25	25.2000	67.0816	863.9520	7.8867	1.7327
2	2013	25	25.2000	67.0816	863.9520	7.8867	1.7327
4	2013	25	27.4143	70.1020	898.1224	8.5490	2.5663
47	2013	25	29.4571	76.1122	954.5367	6.6724	0.6612
82	2013	25	24.9714	73.0000	872.1439	7.5959	2.7867
19	2013	25	28.0286	77.4694	955.8949	5.0857	0.7357
20	2013	25	24.2000	68.1122	852.0704	8.1929	2.5888
80	2013	25	24.9714	73.0000	872.1439	7.5959	2.7867
3	2013	25	29.1000	69.3571	940.2602	8.3000	1.0092
52	2013	25	25.7571	74.4490	899.0786	5.2469	3.0867
70	2013	25	28.3857	70.1429	906.0194	8.1714	2.4255
64	2013	25	20.2286	73.0918	774.9735	7.8255	2.7429
48	2013	25	29.0571	64.3469	928.6949	7.3622	1.0796
65	2013	25	25.7571	74.4490	899.0786	5.2469	3.0867
44	2013	25	28.3857	70.1429	906.0194	8.1714	2.4255
75	2013	25	20.2286	73.0918	774.9735	7.8255	2.7429
40	2013	25	27.8429	80.4184	941.5378	6.5837	1.7020
11	2013	25	24.8143	70.0000	874.9704	7.2184	1.9990
35	2013	25	27.1000	78.2347	935.1959	6.5888	1.2020
78	2013	25	26.7286	66.6020	896.0469	7.5184	1.8531
28	2013	25	27.6714	75.0000	925.0633	6.8673	1.4633
39	2013	25	25.7571	74.4490	899.0786	5.2469	3.0867
24	2013	25	28.1857	67.7959	935.6765	7.0837	1.6194
63	2013	25	27.8429	80.4184	941.5378	6.5837	1.7020
62	2013	25	26.0000	72.1327	871.3969	7.6592	2.0133

1	2013	25	24.9714	73.0000	872.1439	7.5959	2.7867
31	2013	26	22.0857	68.3061	842.3857	7.0786	1.3020
79	2013	26	27.7000	78.1020	970.7031	6.5724	1.2490
51	2013	26	25.9714	77.9286	933.3459	6.9163	0.9888
14	2013	26	23.3857	88.6633	893.2622	5.9490	2.3112
67	2013	26	23.7286	74.1224	897.7786	4.2378	3.0041
42	2013	26	23.0286	75.1633	870.9969	6.4051	2.4133
50	2013	26	25.0286	70.1939	896.6122	8.5020	2.4357
43	2013	26	23.0286	75.1633	870.9969	6.4051	2.4133
85	2013	26	25.6571	70.7347	904.5224	8.2031	2.5449
25	2013	26	27.7286	80.5204	970.1602	6.0337	1.4980
69	2013	26	25.9000	69.1429	933.8704	6.7347	1.3663
57	2013	26	24.4286	75.7959	889.4378	7.0082	2.0000
9	2013	26	22.4143	71.7755	851.0827	7.2561	2.4418
72	2013	26	22.9571	73.7551	873.8622	6.0959	1.6296
26	2013	26	22.6857	77.4796	863.8704	7.5418	1.9959
7	2013	26	22.3571	75.8163	855.8663	5.7837	1.5327
83	2013	26	26.5143	70.7551	938.8347	7.6969	1.1010
76	2013	26	25.7571	71.9796	915.3745	8.6133	1.6990
36	2013	26	25.6714	73.3061	923.4469	6.5969	1.3847
81	2013	26	25.9714	77.9286	933.3459	6.9163	0.9888
15	2013	26	26.8429	64.8673	926.8122	7.4173	1.1133
32	2013	26	23.0286	75.1633	870.9969	6.4051	2.4133
73	2013	26	28.0429	71.8061	955.0051	6.9551	1.2684
71	2013	26	25.6714	73.3061	923.4469	6.5969	1.3847
41	2013	26	24.1143	72.9082	870.1847	7.0755	2.1143
10	2013	26	26.6714	72.7551	957.2704	6.7531	1.5082
23	2013	26	18.5429	75.0816	774.3337	7.3520	2.5102
27	2013	26	22.3571	75.8163	855.8663	5.7837	1.5327
60	2013	26	25.9714	77.9286	933.3459	6.9163	0.9888
53	2013	26	22.4143	71.7755	851.0827	7.2561	2.4418
66	2013	26	23.3857	88.6633	893.2622	5.9490	2.3112
59	2013	26	24.4286	75.7959	889.4378	7.0082	2.0000
61	2013	26	28.0429	71.8061	955.0051	6.9551	1.2684
84	2013	26	28.0429	71.8061	955.0051	6.9551	1.2684
38	2013	26	24.4286	75.7959	889.4378	7.0082	2.0000
87	2013	26	24.4286	68.3878	894.7827	6.1837	1.6510
34	2013	26	24.4286	75.7959	889.4378	7.0082	2.0000
29	2013	26	25.9000	69.1429	933.8704	6.7347	1.3663
5	2013	26	21.3143	79.0000	829.7184	6.1551	1.7786
8	2013	26	22.4143	71.7755	851.0827	7.2561	2.4418
12	2013	26	21.3143	79.0000	829.7184	6.1551	1.7786
13	2013	26	26.5143	70.7551	938.8347	7.6969	1.1010
18	2013	26	26.7571	73.7041	957.1776	6.1827	1.3071
33	2013	26	25.0286	70.1939	896.6122	8.5020	2.4357
56	2013	26	27.7286	80.5204	970.1602	6.0337	1.4980
77	2013	26	26.8429	64.8673	926.8122	7.4173	1.1133
54	2013	26	21.3143	79.0000	829.7184	6.1551	1.7786
21	2013	26	25.0286	70.1939	896.6122	8.5020	2.4357
68	2013	26	27.7000	78.1020	970.7031	6.5724	1.2490

74	2013	26	28.0429	71.8061	955.0051	6.9551	1.2684
88	2013	26	23.0286	75.1633	870.9969	6.4051	2.4133
16	2013	26	25.7571	71.9796	915.3745	8.6133	1.6990
30	2013	26	23.3857	88.6633	893.2622	5.9490	2.3112
6	2013	26	27.7000	78.1020	970.7031	6.5724	1.2490
49	2013	26	25.9000	69.1429	933.8704	6.7347	1.3663
22	2013	26	23.0286	75.1633	870.9969	6.4051	2.4133
45	2013	26	21.3429	76.7959	815.2898	6.1714	1.3255
58	2013	26	25.9000	69.1429	933.8704	6.7347	1.3663
37	2013	26	27.7000	78.1020	970.7031	6.5724	1.2490
17	2013	26	23.7286	74.1224	897.7786	4.2378	3.0041
55	2013	26	22.9571	73.7551	873.8622	6.0959	1.6296
46	2013	26	25.7571	71.9796	915.3745	8.6133	1.6990
86	2013	26	23.6286	69.5918	862.6959	7.6561	1.6531
2	2013	26	23.6286	69.5918	862.6959	7.6561	1.6531
4	2013	26	25.0286	70.1939	896.6122	8.5020	2.4357
47	2013	26	27.3286	76.4694	952.9755	6.4439	0.6745
82	2013	26	23.0286	75.1633	870.9969	6.4051	2.4133
19	2013	26	26.4143	77.8061	954.3398	4.6663	0.7765
20	2013	26	22.4143	71.7755	851.0827	7.2561	2.4418
80	2013	26	23.0286	75.1633	870.9969	6.4051	2.4133
3	2013	26	26.5143	70.7551	938.8347	7.6969	1.1010
52	2013	26	23.7286	74.1224	897.7786	4.2378	3.0041
70	2013	26	25.6571	70.7347	904.5224	8.2031	2.5449
64	2013	26	18.5429	75.0816	774.3337	7.3520	2.5102
48	2013	26	26.8429	64.8673	926.8122	7.4173	1.1133
65	2013	26	23.7286	74.1224	897.7786	4.2378	3.0041
44	2013	26	25.6571	70.7347	904.5224	8.2031	2.5449
75	2013	26	18.5429	75.0816	774.3337	7.3520	2.5102
40	2013	26	26.4571	77.9184	939.8990	7.1102	1.6898
11	2013	26	22.9571	73.7551	873.8622	6.0959	1.6296
35	2013	26	25.9714	77.9286	933.3459	6.9163	0.9888
78	2013	26	24.4286	68.3878	894.7827	6.1837	1.6510
28	2013	26	25.6714	73.3061	923.4469	6.5969	1.3847
39	2013	26	23.7286	74.1224	897.7786	4.2378	3.0041
24	2013	26	25.9000	69.1429	933.8704	6.7347	1.3663
63	2013	26	26.4571	77.9184	939.8990	7.1102	1.6898
62	2013	26	24.1143	72.9082	870.1847	7.0755	2.1143
1	2013	26	23.0286	75.1633	870.9969	6.4051	2.4133
31	2013	27	24.0571	71.9694	841.8827	5.2653	1.2561
79	2013	27	29.8143	78.8878	970.0602	4.9143	1.1622
51	2013	27	27.6571	77.3469	932.8204	6.0439	1.1796
14	2013	27	24.0286	89.1837	892.9847	4.1959	2.8265
67	2013	27	24.3857	78.7959	897.3622	1.6786	3.3041
42	2013	27	23.7714	80.0306	870.5857	3.7469	2.4776
50	2013	27	26.4857	72.7653	895.9347	5.1214	2.5776
43	2013	27	23.7714	80.0306	870.5857	3.7469	2.4776
85	2013	27	27.8571	72.2551	903.8633	5.8837	2.6133
25	2013	27	27.9000	81.1531	969.9153	4.8520	1.5173
69	2013	27	27.7286	70.3469	933.2806	3.8020	1.5347

57	2013	27	26.0429	79.3163	888.9071	4.7520	2.4418
9	2013	27	22.4714	78.0102	850.5939	4.5694	2.6969
72	2013	27	23.7571	78.5510	873.5561	3.9061	2.0837
26	2013	27	22.8429	83.1122	863.5388	5.2990	2.3367
7	2013	27	23.1000	80.6122	855.5898	3.2143	1.9071
83	2013	27	27.5714	75.7245	938.6684	4.7724	1.0776
76	2013	27	27.0429	74.5408	914.7204	5.5673	1.6582
36	2013	27	27.2143	74.0612	923.0173	4.8990	1.5918
81	2013	27	27.6571	77.3469	932.8204	6.0439	1.1796
15	2013	27	28.0714	66.0714	925.9959	4.7102	1.0918
32	2013	27	23.7714	80.0306	870.5857	3.7469	2.4776
73	2013	27	29.3286	72.5204	954.2796	5.1439	1.5520
71	2013	27	27.2143	74.0612	923.0173	4.8990	1.5918
41	2013	27	25.3000	74.9796	869.6204	4.0000	2.2786
10	2013	27	28.5571	74.2347	956.9520	4.6673	1.5092
23	2013	27	19.1571	79.7551	773.9000	4.5888	2.5980
27	2013	27	23.1000	80.6122	855.5898	3.2143	1.9071
60	2013	27	27.6571	77.3469	932.8204	6.0439	1.1796
53	2013	27	22.4714	78.0102	850.5939	4.5694	2.6969
66	2013	27	24.0286	89.1837	892.9847	4.1959	2.8265
59	2013	27	26.0429	79.3163	888.9071	4.7520	2.4418
61	2013	27	29.3286	72.5204	954.2796	5.1439	1.5520
84	2013	27	29.3286	72.5204	954.2796	5.1439	1.5520
38	2013	27	26.0429	79.3163	888.9071	4.7520	2.4418
87	2013	27	25.6143	74.0714	894.4878	2.9531	1.9051
34	2013	27	26.0429	79.3163	888.9071	4.7520	2.4418
29	2013	27	27.7286	70.3469	933.2806	3.8020	1.5347
5	2013	27	21.8143	81.8265	829.3163	4.1694	1.9000
8	2013	27	22.4714	78.0102	850.5939	4.5694	2.6969
12	2013	27	21.8143	81.8265	829.3163	4.1694	1.9000
13	2013	27	27.5714	75.7245	938.6684	4.7724	1.0776
18	2013	27	28.2143	75.5102	956.5071	4.1429	1.5510
33	2013	27	26.4857	72.7653	895.9347	5.1214	2.5776
56	2013	27	27.9000	81.1531	969.9153	4.8520	1.5173
77	2013	27	28.0714	66.0714	925.9959	4.7102	1.0918
54	2013	27	21.8143	81.8265	829.3163	4.1694	1.9000
21	2013	27	26.4857	72.7653	895.9347	5.1214	2.5776
68	2013	27	29.8143	78.8878	970.0602	4.9143	1.1622
74	2013	27	29.3286	72.5204	954.2796	5.1439	1.5520
88	2013	27	23.7714	80.0306	870.5857	3.7469	2.4776
16	2013	27	27.0429	74.5408	914.7204	5.5673	1.6582
30	2013	27	24.0286	89.1837	892.9847	4.1959	2.8265
6	2013	27	29.8143	78.8878	970.0602	4.9143	1.1622
49	2013	27	27.7286	70.3469	933.2806	3.8020	1.5347
22	2013	27	23.7714	80.0306	870.5857	3.7469	2.4776
45	2013	27	20.9429	80.6735	814.8939	3.3500	1.9684
58	2013	27	27.7286	70.3469	933.2806	3.8020	1.5347
37	2013	27	29.8143	78.8878	970.0602	4.9143	1.1622
17	2013	27	24.3857	78.7959	897.3622	1.6786	3.3041
55	2013	27	23.7571	78.5510	873.5561	3.9061	2.0837

46	2013	27	27.0429	74.5408	914.7204	5.5673	1.6582
86	2013	27	24.0500	73.7551	862.6551	4.8561	1.6633
2	2013	27	24.0500	73.7551	862.6551	4.8561	1.6633
4	2013	27	26.4857	72.7653	895.9347	5.1214	2.5776
47	2013	27	28.0000	79.6327	952.6418	4.3622	0.6755
82	2013	27	23.7714	80.0306	870.5857	3.7469	2.4776
19	2013	27	27.2286	79.6429	954.1592	3.8561	0.9061
20	2013	27	22.4714	78.0102	850.5939	4.5694	2.6969
80	2013	27	23.7714	80.0306	870.5857	3.7469	2.4776
3	2013	27	27.5714	75.7245	938.6684	4.7724	1.0776
52	2013	27	24.3857	78.7959	897.3622	1.6786	3.3041
70	2013	27	27.8571	72.2551	903.8633	5.8837	2.6133
64	2013	27	19.1571	79.7551	773.9000	4.5888	2.5980
48	2013	27	28.0714	66.0714	925.9959	4.7102	1.0918
65	2013	27	24.3857	78.7959	897.3622	1.6786	3.3041
44	2013	27	27.8571	72.2551	903.8633	5.8837	2.6133
75	2013	27	19.1571	79.7551	773.9000	4.5888	2.5980
40	2013	27	27.1143	78.6429	939.6806	6.1122	2.1561
11	2013	27	23.7571	78.5510	873.5561	3.9061	2.0837
35	2013	27	27.6571	77.3469	932.8204	6.0439	1.1796
78	2013	27	25.6143	74.0714	894.4878	2.9531	1.9051
28	2013	27	27.2143	74.0612	923.0173	4.8990	1.5918
39	2013	27	24.3857	78.7959	897.3622	1.6786	3.3041
24	2013	27	27.7286	70.3469	933.2806	3.8020	1.5347
63	2013	27	27.1143	78.6429	939.6806	6.1122	2.1561
62	2013	27	25.3000	74.9796	869.6204	4.0000	2.2786
1	2013	27	23.7714	80.0306	870.5857	3.7469	2.4776
31	2013	28	24.2857	69.2551	841.2520	5.3551	1.4357
79	2013	28	30.1571	73.5102	970.3184	4.6541	1.2959
51	2013	28	27.5857	70.0306	933.2469	7.3020	1.9235
14	2013	28	23.7857	85.5408	893.7153	3.8347	3.5235
67	2013	28	24.3714	80.5408	897.7735	1.5551	4.3082
42	2013	28	23.7857	77.8776	870.7816	4.0204	3.3163
50	2013	28	26.8143	68.7755	895.5337	4.7724	3.1357
43	2013	28	23.7857	77.8776	870.7816	4.0204	3.3163
85	2013	28	28.4571	64.0918	903.2194	6.6469	3.3173
25	2013	28	28.5571	77.4184	970.9622	4.8959	1.6837
69	2013	28	27.5000	65.3469	933.4806	3.0510	2.1745
57	2013	28	26.0429	74.2653	888.8000	5.3510	3.6306
9	2013	28	22.3571	80.1837	850.6449	4.1878	3.5071
72	2013	28	23.4714	78.4184	873.9908	3.5347	2.9429
26	2013	28	22.7429	85.6837	863.8041	4.4918	2.7776
7	2013	28	22.7143	79.4694	855.9286	2.2194	2.4939
83	2013	28	27.7857	73.8163	939.4959	4.3510	1.1633
76	2013	28	27.0571	71.1327	914.6378	4.2949	1.8949
36	2013	28	26.8857	69.5714	923.4806	5.4929	2.3265
81	2013	28	27.5857	70.0306	933.2469	7.3020	1.9235
15	2013	28	28.4286	61.8980	925.6673	3.6082	1.1337
32	2013	28	23.7857	77.8776	870.7816	4.0204	3.3163
73	2013	28	29.9000	67.7245	954.2980	5.2112	1.8724

71	2013	28	26.8857	69.5714	923.4806	5.4929	2.3265
41	2013	28	24.9429	71.3367	869.3531	3.6204	2.9980
10	2013	28	28.2000	71.1429	957.7520	5.2224	1.7786
23	2013	28	18.8000	81.2041	773.5276	3.3969	2.8878
27	2013	28	22.7143	79.4694	855.9286	2.2194	2.4939
60	2013	28	27.5857	70.0306	933.2469	7.3020	1.9235
53	2013	28	22.3571	80.1837	850.6449	4.1878	3.5071
66	2013	28	23.7857	85.5408	893.7153	3.8347	3.5235
59	2013	28	26.0429	74.2653	888.8000	5.3510	3.6306
61	2013	28	29.9000	67.7245	954.2980	5.2112	1.8724
84	2013	28	29.9000	67.7245	954.2980	5.2112	1.8724
38	2013	28	26.0429	74.2653	888.8000	5.3510	3.6306
87	2013	28	25.1429	73.8469	894.9612	2.6031	2.5327
34	2013	28	26.0429	74.2653	888.8000	5.3510	3.6306
29	2013	28	27.5000	65.3469	933.4806	3.0510	2.1745
5	2013	28	21.4857	79.8878	829.3143	3.5306	2.2663
8	2013	28	22.3571	80.1837	850.6449	4.1878	3.5071
12	2013	28	21.4857	79.8878	829.3143	3.5306	2.2663
13	2013	28	27.7857	73.8163	939.4959	4.3510	1.1633
18	2013	28	28.7571	72.4694	956.5929	4.2041	2.0031
33	2013	28	26.8143	68.7755	895.5337	4.7724	3.1357
56	2013	28	28.5571	77.4184	970.9622	4.8959	1.6837
77	2013	28	28.4286	61.8980	925.6673	3.6082	1.1337
54	2013	28	21.4857	79.8878	829.3143	3.5306	2.2663
21	2013	28	26.8143	68.7755	895.5337	4.7724	3.1357
68	2013	28	30.1571	73.5102	970.3184	4.6541	1.2959
74	2013	28	29.9000	67.7245	954.2980	5.2112	1.8724
88	2013	28	23.7857	77.8776	870.7816	4.0204	3.3163
16	2013	28	27.0571	71.1327	914.6378	4.2949	1.8949
30	2013	28	23.7857	85.5408	893.7153	3.8347	3.5235
6	2013	28	30.1571	73.5102	970.3184	4.6541	1.2959
49	2013	28	27.5000	65.3469	933.4806	3.0510	2.1745
22	2013	28	23.7857	77.8776	870.7816	4.0204	3.3163
45	2013	28	20.7429	79.3061	814.8776	1.8418	2.7306
58	2013	28	27.5000	65.3469	933.4806	3.0510	2.1745
37	2013	28	30.1571	73.5102	970.3184	4.6541	1.2959
17	2013	28	24.3714	80.5408	897.7735	1.5551	4.3082
55	2013	28	23.4714	78.4184	873.9908	3.5347	2.9429
46	2013	28	27.0571	71.1327	914.6378	4.2949	1.8949
86	2013	28	23.4571	73.6633	862.5449	3.1112	2.0980
2	2013	28	23.4571	73.6633	862.5449	3.1112	2.0980
4	2013	28	26.8143	68.7755	895.5337	4.7724	3.1357
47	2013	28	27.9143	78.1020	953.3000	3.7959	0.7214
82	2013	28	23.7857	77.8776	870.7816	4.0204	3.3163
19	2013	28	26.9429	78.9082	955.2112	3.9276	1.0857
20	2013	28	22.3571	80.1837	850.6449	4.1878	3.5071
80	2013	28	23.7857	77.8776	870.7816	4.0204	3.3163
3	2013	28	27.7857	73.8163	939.4959	4.3510	1.1633
52	2013	28	24.3714	80.5408	897.7735	1.5551	4.3082
70	2013	28	28.4571	64.0918	903.2194	6.6469	3.3173

64	2013	28	18.8000	81.2041	773.5276	3.3969	2.8878
48	2013	28	28.4286	61.8980	925.6673	3.6082	1.1337
65	2013	28	24.3714	80.5408	897.7735	1.5551	4.3082
44	2013	28	28.4571	64.0918	903.2194	6.6469	3.3173
75	2013	28	18.8000	81.2041	773.5276	3.3969	2.8878
40	2013	28	26.7571	76.9388	940.5847	6.7010	2.9204
11	2013	28	23.4714	78.4184	873.9908	3.5347	2.9429
35	2013	28	27.5857	70.0306	933.2469	7.3020	1.9235
78	2013	28	25.1429	73.8469	894.9612	2.6031	2.5327
28	2013	28	26.8857	69.5714	923.4806	5.4929	2.3265
39	2013	28	24.3714	80.5408	897.7735	1.5551	4.3082
24	2013	28	27.5000	65.3469	933.4806	3.0510	2.1745
63	2013	28	26.7571	76.9388	940.5847	6.7010	2.9204
62	2013	28	24.9429	71.3367	869.3531	3.6204	2.9980
1	2013	28	23.7857	77.8776	870.7816	4.0204	3.3163
31	2013	29	23.6857	67.5102	842.0714	5.3112	1.4480
79	2013	29	30.3000	70.3571	970.7102	6.0459	1.4163
51	2013	29	27.3857	66.8980	933.7541	8.5949	2.0408
14	2013	29	23.5000	82.5816	894.2816	4.9908	3.1929
67	2013	29	24.2714	79.0102	898.3643	3.3255	4.1245
42	2013	29	23.9714	74.9898	871.4673	5.3408	3.3010
50	2013	29	27.3429	65.2755	896.2939	6.1980	2.9102
43	2013	29	23.9714	74.9898	871.4673	5.3408	3.3010
85	2013	29	28.3000	59.4184	903.9939	7.8980	3.3061
25	2013	29	28.2143	73.3265	971.4602	6.2245	1.7194
69	2013	29	27.8429	62.1020	934.0806	4.6245	2.1153
57	2013	29	26.5000	69.9694	889.5684	6.2367	3.4031
9	2013	29	22.5000	79.5816	851.2806	5.3031	3.2847
72	2013	29	23.6429	78.2551	874.5745	4.7388	2.6224
26	2013	29	22.9857	84.6327	864.2673	5.4235	2.4735
7	2013	29	22.8143	79.1633	856.5184	2.8776	2.0449
83	2013	29	27.4286	71.5102	940.0000	5.8939	1.1857
76	2013	29	27.6429	68.0306	915.3153	5.4500	1.7214
36	2013	29	27.1000	67.5408	924.0653	7.1724	2.3408
81	2013	29	27.3857	66.8980	933.7541	8.5949	2.0408
15	2013	29	29.1857	59.1020	926.4010	4.9296	1.2204
32	2013	29	23.9714	74.9898	871.4673	5.3408	3.3010
73	2013	29	30.3286	65.2857	954.8684	6.3643	1.7827
71	2013	29	27.1000	67.5408	924.0653	7.1724	2.3408
41	2013	29	25.2143	68.9796	870.1296	4.8908	2.8929
10	2013	29	28.1714	69.8878	958.2378	7.3929	1.7204
23	2013	29	19.1714	81.5510	774.2571	4.3276	2.7745
27	2013	29	22.8143	79.1633	856.5184	2.8776	2.0449
60	2013	29	27.3857	66.8980	933.7541	8.5949	2.0408
53	2013	29	22.5000	79.5816	851.2806	5.3031	3.2847
66	2013	29	23.5000	82.5816	894.2816	4.9908	3.1929
59	2013	29	26.5000	69.9694	889.5684	6.2367	3.4031
61	2013	29	30.3286	65.2857	954.8684	6.3643	1.7827
84	2013	29	30.3286	65.2857	954.8684	6.3643	1.7827
38	2013	29	26.5000	69.9694	889.5684	6.2367	3.4031

87	2013	29	25.3143	72.9898	895.6184	4.3571	2.4633
34	2013	29	26.5000	69.9694	889.5684	6.2367	3.4031
29	2013	29	27.8429	62.1020	934.0806	4.6245	2.1153
5	2013	29	21.7857	79.6122	829.9092	4.4469	1.9408
8	2013	29	22.5000	79.5816	851.2806	5.3031	3.2847
12	2013	29	21.7857	79.6122	829.9092	4.4469	1.9408
13	2013	29	27.4286	71.5102	940.0000	5.8939	1.1857
18	2013	29	29.2286	71.1020	956.9939	5.8673	1.8061
33	2013	29	27.3429	65.2755	896.2939	6.1980	2.9102
56	2013	29	28.2143	73.3265	971.4602	6.2245	1.7194
77	2013	29	29.1857	59.1020	926.4010	4.9296	1.2204
54	2013	29	21.7857	79.6122	829.9092	4.4469	1.9408
21	2013	29	27.3429	65.2755	896.2939	6.1980	2.9102
68	2013	29	30.3000	70.3571	970.7102	6.0459	1.4163
74	2013	29	30.3286	65.2857	954.8684	6.3643	1.7827
88	2013	29	23.9714	74.9898	871.4673	5.3408	3.3010
16	2013	29	27.6429	68.0306	915.3153	5.4500	1.7214
30	2013	29	23.5000	82.5816	894.2816	4.9908	3.1929
6	2013	29	30.3000	70.3571	970.7102	6.0459	1.4163
49	2013	29	27.8429	62.1020	934.0806	4.6245	2.1153
22	2013	29	23.9714	74.9898	871.4673	5.3408	3.3010
45	2013	29	21.1286	79.1837	815.4500	2.9592	2.3633
58	2013	29	27.8429	62.1020	934.0806	4.6245	2.1153
37	2013	29	30.3000	70.3571	970.7102	6.0459	1.4163
17	2013	29	24.2714	79.0102	898.3643	3.3255	4.1245
55	2013	29	23.6429	78.2551	874.5745	4.7388	2.6224
46	2013	29	27.6429	68.0306	915.3153	5.4500	1.7214
86	2013	29	24.2571	72.3571	862.7673	4.1378	1.9520
2	2013	29	24.2571	72.3571	862.7673	4.1378	1.9520
4	2013	29	27.3429	65.2755	896.2939	6.1980	2.9102
47	2013	29	26.8143	76.7755	953.7878	4.6622	0.7429
82	2013	29	23.9714	74.9898	871.4673	5.3408	3.3010
19	2013	29	26.8143	76.9082	955.6990	4.8520	0.9878
20	2013	29	22.5000	79.5816	851.2806	5.3031	3.2847
80	2013	29	23.9714	74.9898	871.4673	5.3408	3.3010
3	2013	29	27.4286	71.5102	940.0000	5.8939	1.1857
52	2013	29	24.2714	79.0102	898.3643	3.3255	4.1245
70	2013	29	28.3000	59.4184	903.9939	7.8980	3.3061
64	2013	29	19.1714	81.5510	774.2571	4.3276	2.7745
48	2013	29	29.1857	59.1020	926.4010	4.9296	1.2204
65	2013	29	24.2714	79.0102	898.3643	3.3255	4.1245
44	2013	29	28.3000	59.4184	903.9939	7.8980	3.3061
75	2013	29	19.1714	81.5510	774.2571	4.3276	2.7745
40	2013	29	27.1429	75.2041	941.0408	7.8449	2.8694
11	2013	29	23.6429	78.2551	874.5745	4.7388	2.6224
35	2013	29	27.3857	66.8980	933.7541	8.5949	2.0408
78	2013	29	25.3143	72.9898	895.6184	4.3571	2.4633
28	2013	29	27.1000	67.5408	924.0653	7.1724	2.3408
39	2013	29	24.2714	79.0102	898.3643	3.3255	4.1245
24	2013	29	27.8429	62.1020	934.0806	4.6245	2.1153

63	2013	29	27.1429	75.2041	941.0408	7.8449	2.8694
62	2013	29	25.2143	68.9796	870.1296	4.8908	2.8929
1	2013	29	23.9714	74.9898	871.4673	5.3408	3.3010
31	2013	30	25.0571	67.2653	842.7020	5.9133	1.4551
79	2013	30	31.6429	66.8980	971.0051	7.9898	1.4408
51	2013	30	27.8143	66.8673	934.1296	8.8827	1.8612
14	2013	30	23.6571	83.3163	894.5235	5.7367	2.7388
67	2013	30	23.9286	80.0102	898.7510	3.6541	3.7520
42	2013	30	24.4571	73.1939	871.9214	5.8969	3.0745
50	2013	30	27.9286	62.5714	896.8398	6.8653	2.9429
43	2013	30	24.4571	73.1939	871.9214	5.8969	3.0745
85	2013	30	29.3000	57.2551	904.5908	7.9031	3.2092
25	2013	30	28.5143	73.8163	971.6459	6.6122	1.3776
69	2013	30	27.9143	60.6735	934.5418	5.7061	2.0714
57	2013	30	26.7714	66.9694	890.0949	6.6571	3.0816
9	2013	30	22.9143	77.7143	851.6918	7.0398	3.0969
72	2013	30	24.0286	77.4490	874.9082	6.4122	2.2020
26	2013	30	23.0286	82.5612	864.5031	7.0061	2.3653
7	2013	30	23.0857	78.3469	856.8153	4.4949	1.7163
83	2013	30	27.2571	73.3673	940.1622	6.4408	1.0929
76	2013	30	28.0429	64.6531	915.8357	6.8224	1.6357
36	2013	30	26.8286	68.1224	924.4816	7.3469	1.9939
81	2013	30	27.8143	66.8673	934.1296	8.8827	1.8612
15	2013	30	29.9000	54.6224	926.8959	5.9439	1.3673
32	2013	30	24.4571	73.1939	871.9214	5.8969	3.0745
73	2013	30	30.9857	62.0204	955.2939	6.8337	1.7704
71	2013	30	26.8286	68.1224	924.4816	7.3469	1.9939
41	2013	30	26.0714	66.3878	870.7061	6.4806	2.7296
10	2013	30	28.3714	70.0816	958.4888	8.6173	1.5071
23	2013	30	19.5571	80.2755	774.7745	5.5878	2.6969
27	2013	30	23.0857	78.3469	856.8153	4.4949	1.7163
60	2013	30	27.8143	66.8673	934.1296	8.8827	1.8612
53	2013	30	22.9143	77.7143	851.6918	7.0398	3.0969
66	2013	30	23.6571	83.3163	894.5235	5.7367	2.7388
59	2013	30	26.7714	66.9694	890.0949	6.6571	3.0816
61	2013	30	30.9857	62.0204	955.2939	6.8337	1.7704
84	2013	30	30.9857	62.0204	955.2939	6.8337	1.7704
38	2013	30	26.7714	66.9694	890.0949	6.6571	3.0816
87	2013	30	25.8714	73.1429	896.0133	5.4133	2.0214
34	2013	30	26.7714	66.9694	890.0949	6.6571	3.0816
29	2013	30	27.9143	60.6735	934.5418	5.7061	2.0714
5	2013	30	21.9714	79.3571	830.2806	6.7510	1.8265
8	2013	30	22.9143	77.7143	851.6918	7.0398	3.0969
12	2013	30	21.9714	79.3571	830.2806	6.7510	1.8265
13	2013	30	27.2571	73.3673	940.1622	6.4408	1.0929
18	2013	30	30.3429	66.8163	957.2755	7.6500	1.8153
33	2013	30	27.9286	62.5714	896.8398	6.8653	2.9429
56	2013	30	28.5143	73.8163	971.6459	6.6122	1.3776
77	2013	30	29.9000	54.6224	926.8959	5.9439	1.3673
54	2013	30	21.9714	79.3571	830.2806	6.7510	1.8265

21	2013	30	27.9286	62.5714	896.8398	6.8653	2.9429
68	2013	30	31.6429	66.8980	971.0051	7.9898	1.4408
74	2013	30	30.9857	62.0204	955.2939	6.8337	1.7704
88	2013	30	24.4571	73.1939	871.9214	5.8969	3.0745
16	2013	30	28.0429	64.6531	915.8357	6.8224	1.6357
30	2013	30	23.6571	83.3163	894.5235	5.7367	2.7388
6	2013	30	31.6429	66.8980	971.0051	7.9898	1.4408
49	2013	30	27.9143	60.6735	934.5418	5.7061	2.0714
22	2013	30	24.4571	73.1939	871.9214	5.8969	3.0745
45	2013	30	21.0286	79.3061	815.7694	4.9102	2.0551
58	2013	30	27.9143	60.6735	934.5418	5.7061	2.0714
37	2013	30	31.6429	66.8980	971.0051	7.9898	1.4408
17	2013	30	23.9286	80.0102	898.7510	3.6541	3.7520
55	2013	30	24.0286	77.4490	874.9082	6.4122	2.2020
46	2013	30	28.0429	64.6531	915.8357	6.8224	1.6357
86	2013	30	24.9429	69.4490	863.2551	5.7500	1.7643
2	2013	30	24.9429	69.4490	863.2551	5.7500	1.7643
4	2013	30	27.9286	62.5714	896.8398	6.8653	2.9429
47	2013	30	27.3857	79.5408	954.1173	4.9694	0.6816
82	2013	30	24.4571	73.1939	871.9214	5.8969	3.0745
19	2013	30	26.5143	78.4796	955.8255	5.0306	0.7357
20	2013	30	22.9143	77.7143	851.6918	7.0398	3.0969
80	2013	30	24.4571	73.1939	871.9214	5.8969	3.0745
3	2013	30	27.2571	73.3673	940.1622	6.4408	1.0929
52	2013	30	23.9286	80.0102	898.7510	3.6541	3.7520
70	2013	30	29.3000	57.2551	904.5908	7.9031	3.2092
64	2013	30	19.5571	80.2755	774.7745	5.5878	2.6969
48	2013	30	29.9000	54.6224	926.8959	5.9439	1.3673
65	2013	30	23.9286	80.0102	898.7510	3.6541	3.7520
44	2013	30	29.3000	57.2551	904.5908	7.9031	3.2092
75	2013	30	19.5571	80.2755	774.7745	5.5878	2.6969
40	2013	30	27.5714	73.0000	941.1378	8.4378	2.3337
11	2013	30	24.0286	77.4490	874.9082	6.4122	2.2020
35	2013	30	27.8143	66.8673	934.1296	8.8827	1.8612
78	2013	30	25.8714	73.1429	896.0133	5.4133	2.0214
28	2013	30	26.8286	68.1224	924.4816	7.3469	1.9939
39	2013	30	23.9286	80.0102	898.7510	3.6541	3.7520
24	2013	30	27.9143	60.6735	934.5418	5.7061	2.0714
63	2013	30	27.5714	73.0000	941.1378	8.4378	2.3337
62	2013	30	26.0714	66.3878	870.7061	6.4806	2.7296
1	2013	30	24.4571	73.1939	871.9214	5.8969	3.0745
31	2013	31	22.8286	67.1224	843.2490	6.4204	1.3847
79	2013	31	30.9714	62.2959	971.2786	8.2592	1.5265
51	2013	31	27.6000	65.7653	934.5041	8.6959	1.7980
14	2013	31	23.2571	83.9694	894.8316	5.6745	2.6010
67	2013	31	23.9000	81.7143	899.1673	2.9418	3.2959
42	2013	31	23.4000	72.4694	872.3480	5.3071	2.8663
50	2013	31	26.3714	61.8061	897.3388	7.3459	2.7765
43	2013	31	23.4000	72.4694	872.3480	5.3071	2.8663
85	2013	31	27.1857	56.9388	905.1378	7.8837	3.0429

25	2013	31	28.0571	74.6327	971.9520	6.1173	1.1939
69	2013	31	26.8714	61.1122	935.0061	5.8041	2.0500
57	2013	31	25.4714	66.3265	890.5735	6.2571	2.9306
9	2013	31	21.8857	77.0714	852.1235	7.1510	3.0867
72	2013	31	22.7714	76.9898	875.2878	6.5990	2.2847
26	2013	31	21.9429	83.3469	864.9020	6.5673	2.2694
7	2013	31	21.7571	79.2449	857.2061	5.0316	1.4449
83	2013	31	27.1286	72.8980	940.4694	6.5949	1.0918
76	2013	31	27.0714	63.4592	916.2735	7.5827	1.8418
36	2013	31	27.0429	69.0714	924.9031	6.6296	1.8480
81	2013	31	27.6000	65.7653	934.5041	8.6959	1.7980
15	2013	31	28.2714	52.5612	927.3459	6.3398	1.3663
32	2013	31	23.4000	72.4694	872.3480	5.3071	2.8663
73	2013	31	30.0714	59.5612	955.6816	6.8704	1.8571
71	2013	31	27.0429	69.0714	924.9031	6.6296	1.8480
41	2013	31	24.5714	65.0408	871.2153	7.3051	2.7714
10	2013	31	28.3571	69.6327	958.7837	8.7704	1.5704
23	2013	31	18.9286	78.7347	775.2990	6.2184	2.5673
27	2013	31	21.7571	79.2449	857.2061	5.0316	1.4449
60	2013	31	27.6000	65.7653	934.5041	8.6959	1.7980
53	2013	31	21.8857	77.0714	852.1235	7.1510	3.0867
66	2013	31	23.2571	83.9694	894.8316	5.6745	2.6010
59	2013	31	25.4714	66.3265	890.5735	6.2571	2.9306
61	2013	31	30.0714	59.5612	955.6816	6.8704	1.8571
84	2013	31	30.0714	59.5612	955.6816	6.8704	1.8571
38	2013	31	25.4714	66.3265	890.5735	6.2571	2.9306
87	2013	31	24.7286	72.6735	896.4143	5.5388	1.9612
34	2013	31	25.4714	66.3265	890.5735	6.2571	2.9306
29	2013	31	26.8714	61.1122	935.0061	5.8041	2.0500
5	2013	31	20.5429	80.6837	830.7061	6.6459	1.9000
8	2013	31	21.8857	77.0714	852.1235	7.1510	3.0867
12	2013	31	20.5429	80.6837	830.7061	6.6459	1.9000
13	2013	31	27.1286	72.8980	940.4694	6.5949	1.0918
18	2013	31	29.7857	61.9592	957.5102	8.1051	1.9459
33	2013	31	26.3714	61.8061	897.3388	7.3459	2.7765
56	2013	31	28.0571	74.6327	971.9520	6.1173	1.1939
77	2013	31	28.2714	52.5612	927.3459	6.3398	1.3663
54	2013	31	20.5429	80.6837	830.7061	6.6459	1.9000
21	2013	31	26.3714	61.8061	897.3388	7.3459	2.7765
68	2013	31	30.9714	62.2959	971.2786	8.2592	1.5265
74	2013	31	30.0714	59.5612	955.6816	6.8704	1.8571
88	2013	31	23.4000	72.4694	872.3480	5.3071	2.8663
16	2013	31	27.0714	63.4592	916.2735	7.5827	1.8418
30	2013	31	23.2571	83.9694	894.8316	5.6745	2.6010
6	2013	31	30.9714	62.2959	971.2786	8.2592	1.5265
49	2013	31	26.8714	61.1122	935.0061	5.8041	2.0500
22	2013	31	23.4000	72.4694	872.3480	5.3071	2.8663
45	2013	31	19.6857	80.8673	816.1969	5.1388	2.0837
58	2013	31	26.8714	61.1122	935.0061	5.8041	2.0500
37	2013	31	30.9714	62.2959	971.2786	8.2592	1.5265

17	2013	31	23.9000	81.7143	899.1673	2.9418	3.2959
55	2013	31	22.7714	76.9898	875.2878	6.5990	2.2847
46	2013	31	27.0714	63.4592	916.2735	7.5827	1.8418
86	2013	31	23.5429	68.1939	863.7378	5.7673	1.7929
2	2013	31	23.5429	68.1939	863.7378	5.7673	1.7929
4	2013	31	26.3714	61.8061	897.3388	7.3459	2.7765
47	2013	31	26.8143	80.5102	954.5214	5.3551	0.6561
82	2013	31	23.4000	72.4694	872.3480	5.3071	2.8663
19	2013	31	26.6857	79.5510	956.1357	5.2408	0.8878
20	2013	31	21.8857	77.0714	852.1235	7.1510	3.0867
80	2013	31	23.4000	72.4694	872.3480	5.3071	2.8663
3	2013	31	27.1286	72.8980	940.4694	6.5949	1.0918
52	2013	31	23.9000	81.7143	899.1673	2.9418	3.2959
70	2013	31	27.1857	56.9388	905.1378	7.8837	3.0429
64	2013	31	18.9286	78.7347	775.2990	6.2184	2.5673
48	2013	31	28.2714	52.5612	927.3459	6.3398	1.3663
65	2013	31	23.9000	81.7143	899.1673	2.9418	3.2959
44	2013	31	27.1857	56.9388	905.1378	7.8837	3.0429
75	2013	31	18.9286	78.7347	775.2990	6.2184	2.5673
40	2013	31	27.4000	69.3367	941.1949	8.3133	1.9561
11	2013	31	22.7714	76.9898	875.2878	6.5990	2.2847
35	2013	31	27.6000	65.7653	934.5041	8.6959	1.7980
78	2013	31	24.7286	72.6735	896.4143	5.5388	1.9612
28	2013	31	27.0429	69.0714	924.9031	6.6296	1.8480
39	2013	31	23.9000	81.7143	899.1673	2.9418	3.2959
24	2013	31	26.8714	61.1122	935.0061	5.8041	2.0500
63	2013	31	27.4000	69.3367	941.1949	8.3133	1.9561
62	2013	31	24.5714	65.0408	871.2153	7.3051	2.7714
1	2013	31	23.4000	72.4694	872.3480	5.3071	2.8663
31	2013	32	24.3857	67.3673	844.3316	6.7153	1.3663
79	2013	32	32.0571	61.0102	972.3265	7.8969	1.4908
51	2013	32	28.4429	63.9286	935.5490	9.0592	1.7122
14	2013	32	23.9571	82.1939	895.9000	6.0153	2.6173
67	2013	32	24.8714	79.0510	900.2357	3.1153	3.3418
42	2013	32	24.2143	73.2245	873.3888	5.4918	2.7714
50	2013	32	27.5429	63.9694	898.4704	7.8949	2.4347
43	2013	32	24.2143	73.2245	873.3888	5.4918	2.7714
85	2013	32	29.1714	58.8061	906.2959	7.8643	2.9694
25	2013	32	28.9571	73.3061	973.0510	6.5816	1.3265
69	2013	32	28.1714	62.0816	936.0990	6.3724	1.8459
57	2013	32	26.9286	67.3163	891.6469	6.4357	2.8102
9	2013	32	22.4143	77.2551	853.1633	6.5102	3.0296
72	2013	32	23.5571	77.8776	876.3459	6.3837	2.4898
26	2013	32	22.2714	84.2755	865.9796	6.3867	2.0827
7	2013	32	22.5286	80.3367	858.2673	5.4724	1.4122
83	2013	32	26.7429	71.4694	941.5776	6.7806	1.0694
76	2013	32	28.3714	63.8061	917.3296	8.1357	1.9969
36	2013	32	28.0143	66.5918	925.9408	7.8020	1.9755
81	2013	32	28.4429	63.9286	935.5490	9.0592	1.7122
15	2013	32	29.8143	54.3265	928.4622	7.2959	1.3786

32	2013	32	24.2143	73.2245	873.3888	5.4918	2.7714
73	2013	32	31.8429	58.9592	956.7837	7.8939	1.9184
71	2013	32	28.0143	66.5918	925.9408	7.8020	1.9755
41	2013	32	25.2143	66.7449	872.2776	7.3633	2.6622
10	2013	32	29.3286	68.1020	959.8490	8.8439	1.5755
23	2013	32	18.8429	76.6327	776.3194	6.8316	2.5378
27	2013	32	22.5286	80.3367	858.2673	5.4724	1.4122
60	2013	32	28.4429	63.9286	935.5490	9.0592	1.7122
53	2013	32	22.4143	77.2551	853.1633	6.5102	3.0296
66	2013	32	23.9571	82.1939	895.9000	6.0153	2.6173
59	2013	32	26.9286	67.3163	891.6469	6.4357	2.8102
61	2013	32	31.8429	58.9592	956.7837	7.8939	1.9184
84	2013	32	31.8429	58.9592	956.7837	7.8939	1.9184
38	2013	32	26.9286	67.3163	891.6469	6.4357	2.8102
87	2013	32	25.5714	73.3163	897.4745	5.9918	2.0092
34	2013	32	26.9286	67.3163	891.6469	6.4357	2.8102
29	2013	32	28.1714	62.0816	936.0990	6.3724	1.8459
5	2013	32	21.1714	81.5612	831.7541	6.1796	1.8214
8	2013	32	22.4143	77.2551	853.1633	6.5102	3.0296
12	2013	32	21.1714	81.5612	831.7541	6.1796	1.8214
13	2013	32	26.7429	71.4694	941.5776	6.7806	1.0694
18	2013	32	30.9571	60.6327	958.5520	8.5816	1.9255
33	2013	32	27.5429	63.9694	898.4704	7.8949	2.4347
56	2013	32	28.9571	73.3061	973.0510	6.5816	1.3265
77	2013	32	29.8143	54.3265	928.4622	7.2959	1.3786
54	2013	32	21.1714	81.5612	831.7541	6.1796	1.8214
21	2013	32	27.5429	63.9694	898.4704	7.8949	2.4347
68	2013	32	32.0571	61.0102	972.3265	7.8969	1.4908
74	2013	32	31.8429	58.9592	956.7837	7.8939	1.9184
88	2013	32	24.2143	73.2245	873.3888	5.4918	2.7714
16	2013	32	28.3714	63.8061	917.3296	8.1357	1.9969
30	2013	32	23.9571	82.1939	895.9000	6.0153	2.6173
6	2013	32	32.0571	61.0102	972.3265	7.8969	1.4908
49	2013	32	28.1714	62.0816	936.0990	6.3724	1.8459
22	2013	32	24.2143	73.2245	873.3888	5.4918	2.7714
45	2013	32	20.4857	80.8776	817.2439	5.6561	2.2173
58	2013	32	28.1714	62.0816	936.0990	6.3724	1.8459
37	2013	32	32.0571	61.0102	972.3265	7.8969	1.4908
17	2013	32	24.8714	79.0510	900.2357	3.1153	3.3418
55	2013	32	23.5571	77.8776	876.3459	6.3837	2.4898
46	2013	32	28.3714	63.8061	917.3296	8.1357	1.9969
86	2013	32	24.4429	68.5102	864.8337	6.3071	1.9306
2	2013	32	24.4429	68.5102	864.8337	6.3071	1.9306
4	2013	32	27.5429	63.9694	898.4704	7.8949	2.4347
47	2013	32	27.2857	78.2857	955.5765	5.8031	0.7041
82	2013	32	24.2143	73.2245	873.3888	5.4918	2.7714
19	2013	32	26.8286	77.1633	957.2265	6.1806	1.1337
20	2013	32	22.4143	77.2551	853.1633	6.5102	3.0296
80	2013	32	24.2143	73.2245	873.3888	5.4918	2.7714
3	2013	32	26.7429	71.4694	941.5776	6.7806	1.0694

52	2013	32	24.8714	79.0510	900.2357	3.1153	3.3418
70	2013	32	29.1714	58.8061	906.2959	7.8643	2.9694
64	2013	32	18.8429	76.6327	776.3194	6.8316	2.5378
48	2013	32	29.8143	54.3265	928.4622	7.2959	1.3786
65	2013	32	24.8714	79.0510	900.2357	3.1153	3.3418
44	2013	32	29.1714	58.8061	906.2959	7.8643	2.9694
75	2013	32	18.8429	76.6327	776.3194	6.8316	2.5378
40	2013	32	27.9571	67.1735	942.1224	8.0071	1.9878
11	2013	32	23.5571	77.8776	876.3459	6.3837	2.4898
35	2013	32	28.4429	63.9286	935.5490	9.0592	1.7122
78	2013	32	25.5714	73.3163	897.4745	5.9918	2.0092
28	2013	32	28.0143	66.5918	925.9408	7.8020	1.9755
39	2013	32	24.8714	79.0510	900.2357	3.1153	3.3418
24	2013	32	28.1714	62.0816	936.0990	6.3724	1.8459
63	2013	32	27.9571	67.1735	942.1224	8.0071	1.9878
62	2013	32	25.2143	66.7449	872.2776	7.3633	2.6622
1	2013	32	24.2143	73.2245	873.3888	5.4918	2.7714
31	2013	33	22.1571	66.8367	845.1061	6.8092	1.4235
79	2013	33	29.0571	61.4286	972.9204	7.9367	1.3898
51	2013	33	25.1143	64.8163	936.0582	8.7673	1.6531
14	2013	33	24.1714	78.3061	896.3520	6.3878	2.5347
67	2013	33	24.0714	75.0102	900.6990	3.9316	3.6663
42	2013	33	23.3857	71.8265	873.9878	6.2153	2.8388
50	2013	33	25.5571	64.2551	899.2000	8.3367	2.4347
43	2013	33	23.3857	71.8265	873.9878	6.2153	2.8388
85	2013	33	25.9286	60.0000	907.1000	7.6939	3.0633
25	2013	33	27.3429	71.1633	973.3837	7.1031	1.3959
69	2013	33	25.9143	60.8571	936.6418	6.7490	1.8276
57	2013	33	24.9429	65.9796	892.3031	6.9541	2.8714
9	2013	33	22.3857	75.4796	853.8306	6.5112	2.8806
72	2013	33	23.4429	76.1327	877.0082	6.3031	2.4602
26	2013	33	23.1714	82.9592	866.7765	6.2173	1.9735
7	2013	33	22.5429	78.1020	859.0480	5.6490	1.3878
83	2013	33	26.2000	74.2245	942.3796	6.0296	1.0235
76	2013	33	26.7857	62.3673	917.9490	8.8173	1.9929
36	2013	33	25.6286	64.5510	926.4235	8.0878	2.0143
81	2013	33	25.1143	64.8163	936.0582	8.7673	1.6531
15	2013	33	27.6857	55.8878	929.1959	7.9959	1.5276
32	2013	33	23.3857	71.8265	873.9878	6.2153	2.8388
73	2013	33	28.9143	58.8571	957.4398	8.3061	1.9439
71	2013	33	25.6286	64.5510	926.4235	8.0878	2.0143
41	2013	33	24.0857	66.8980	873.0041	7.5867	2.5684
10	2013	33	26.7714	66.7347	960.2806	8.6571	1.5857
23	2013	33	17.2714	77.1735	777.0357	5.6255	2.5133
27	2013	33	22.5429	78.1020	859.0480	5.6490	1.3878
60	2013	33	25.1143	64.8163	936.0582	8.7673	1.6531
53	2013	33	22.3857	75.4796	853.8306	6.5112	2.8806
66	2013	33	24.1714	78.3061	896.3520	6.3878	2.5347
59	2013	33	24.9429	65.9796	892.3031	6.9541	2.8714
61	2013	33	28.9143	58.8571	957.4398	8.3061	1.9439

84	2013	33	28.9143	58.8571	957.4398	8.3061	1.9439
38	2013	33	24.9429	65.9796	892.3031	6.9541	2.8714
87	2013	33	24.2833	73.1224	898.3296	6.3378	2.0806
34	2013	33	24.9429	65.9796	892.3031	6.9541	2.8714
29	2013	33	25.9143	60.8571	936.6418	6.7490	1.8276
5	2013	33	20.5429	80.6531	832.5276	5.9204	1.6918
8	2013	33	22.3857	75.4796	853.8306	6.5112	2.8806
12	2013	33	20.5429	80.6531	832.5276	5.9204	1.6918
13	2013	33	26.2000	74.2245	942.3796	6.0296	1.0235
18	2013	33	27.6286	61.9898	959.2102	8.0031	1.8051
33	2013	33	25.5571	64.2551	899.2000	8.3367	2.4347
56	2013	33	27.3429	71.1633	973.3837	7.1031	1.3959
77	2013	33	27.6857	55.8878	929.1959	7.9959	1.5276
54	2013	33	20.5429	80.6531	832.5276	5.9204	1.6918
21	2013	33	25.5571	64.2551	899.2000	8.3367	2.4347
68	2013	33	29.0571	61.4286	972.9204	7.9367	1.3898
74	2013	33	28.9143	58.8571	957.4398	8.3061	1.9439
88	2013	33	23.3857	71.8265	873.9878	6.2153	2.8388
16	2013	33	26.7857	62.3673	917.9490	8.8173	1.9929
30	2013	33	24.1714	78.3061	896.3520	6.3878	2.5347
6	2013	33	29.0571	61.4286	972.9204	7.9367	1.3898
49	2013	33	25.9143	60.8571	936.6418	6.7490	1.8276
22	2013	33	23.3857	71.8265	873.9878	6.2153	2.8388
45	2013	33	19.6714	79.1122	818.0520	5.3776	2.1286
58	2013	33	25.9143	60.8571	936.6418	6.7490	1.8276
37	2013	33	29.0571	61.4286	972.9204	7.9367	1.3898
17	2013	33	24.0714	75.0102	900.6990	3.9316	3.6663
55	2013	33	23.4429	76.1327	877.0082	6.3031	2.4602
46	2013	33	26.7857	62.3673	917.9490	8.8173	1.9929
86	2013	33	23.6000	66.9184	865.5786	6.9857	1.9633
2	2013	33	23.6000	66.9184	865.5786	6.9857	1.9633
4	2013	33	25.5571	64.2551	899.2000	8.3367	2.4347
47	2013	33	26.5286	77.6939	956.1602	5.3551	0.8255
82	2013	33	23.3857	71.8265	873.9878	6.2153	2.8388
19	2013	33	26.4286	76.2551	957.5939	6.1592	1.0735
20	2013	33	22.3857	75.4796	853.8306	6.5112	2.8806
80	2013	33	23.3857	71.8265	873.9878	6.2153	2.8388
3	2013	33	26.2000	74.2245	942.3796	6.0296	1.0235
52	2013	33	24.0714	75.0102	900.6990	3.9316	3.6663
70	2013	33	25.9286	60.0000	907.1000	7.6939	3.0633
64	2013	33	17.2714	77.1735	777.0357	5.6255	2.5133
48	2013	33	27.6857	55.8878	929.1959	7.9959	1.5276
65	2013	33	24.0714	75.0102	900.6990	3.9316	3.6663
44	2013	33	25.9286	60.0000	907.1000	7.6939	3.0633
75	2013	33	17.2714	77.1735	777.0357	5.6255	2.5133
40	2013	33	26.2429	66.2959	942.4163	7.6786	1.9816
11	2013	33	23.4429	76.1327	877.0082	6.3031	2.4602
35	2013	33	25.1143	64.8163	936.0582	8.7673	1.6531
78	2013	33	24.2833	73.1224	898.3296	6.3378	2.0806
28	2013	33	25.6286	64.5510	926.4235	8.0878	2.0143

39	2013	33	24.0714	75.0102	900.6990	3.9316	3.6663
24	2013	33	25.9143	60.8571	936.6418	6.7490	1.8276
63	2013	33	26.2429	66.2959	942.4163	7.6786	1.9816
62	2013	33	24.0857	66.8980	873.0041	7.5867	2.5684
1	2013	33	23.3857	71.8265	873.9878	6.2153	2.8388
31	2013	34	21.0714	72.5204	845.0459	5.2214	1.2357
79	2013	34	26.2143	72.8878	971.7847	5.1418	1.1990
51	2013	34	24.0143	76.8571	934.5122	4.9408	1.4520
14	2013	34	22.3286	80.4286	894.1408	4.3898	2.1245
67	2013	34	22.3286	80.0204	898.9010	3.0980	3.3347
42	2013	34	21.8143	75.8469	872.7031	4.6878	2.6112
50	2013	34	24.3286	70.3469	898.8367	6.7398	2.0969
43	2013	34	21.8143	75.8469	872.7031	4.6878	2.6112
85	2013	34	24.1714	69.6020	906.9847	5.5908	2.2847
25	2013	34	25.9143	78.9490	971.0347	4.3010	1.1184
69	2013	34	24.6857	68.5816	935.3918	4.6357	1.4071
57	2013	34	22.3714	75.8673	891.5398	4.5755	2.2510
9	2013	34	20.9857	77.1224	852.6276	5.6765	2.5980
72	2013	34	22.7143	76.3061	875.3827	4.9969	2.7204
26	2013	34	22.3286	80.6837	865.3408	4.6173	1.6031
7	2013	34	21.6000	78.3469	857.7214	4.2827	0.9214
83	2013	34	25.1571	80.2347	940.4786	4.0969	0.9316
76	2013	34	24.9714	67.7041	917.0980	7.3112	1.6908
36	2013	34	23.9143	75.5612	924.7541	4.6214	1.5000
81	2013	34	24.0143	76.8571	934.5122	4.9408	1.4520
15	2013	34	26.8714	60.3571	928.7939	7.1173	1.4286
32	2013	34	21.8143	75.8469	872.7031	4.6878	2.6112
73	2013	34	27.3000	65.9592	956.5255	6.1633	1.5459
71	2013	34	23.9143	75.5612	924.7541	4.6214	1.5000
41	2013	34	22.3000	73.1020	872.4061	6.1316	1.8469
10	2013	34	24.9857	76.9184	958.4918	4.7276	1.3714
23	2013	34	17.2286	80.2653	776.7480	4.4204	2.4378
27	2013	34	21.6000	78.3469	857.7214	4.2827	0.9214
60	2013	34	24.0143	76.8571	934.5122	4.9408	1.4520
53	2013	34	20.9857	77.1224	852.6276	5.6765	2.5980
66	2013	34	22.3286	80.4286	894.1408	4.3898	2.1245
59	2013	34	22.3714	75.8673	891.5398	4.5755	2.2510
61	2013	34	27.3000	65.9592	956.5255	6.1633	1.5459
84	2013	34	27.3000	65.9592	956.5255	6.1633	1.5459
38	2013	34	22.3714	75.8673	891.5398	4.5755	2.2510
87	2013	34	22.8429	77.2653	897.0939	4.4959	2.3408
34	2013	34	22.3714	75.8673	891.5398	4.5755	2.2510
29	2013	34	24.6857	68.5816	935.3918	4.6357	1.4071
5	2013	34	20.0286	81.5918	831.5163	5.0571	1.4908
8	2013	34	20.9857	77.1224	852.6276	5.6765	2.5980
12	2013	34	20.0286	81.5918	831.5163	5.0571	1.4908
13	2013	34	25.1571	80.2347	940.4786	4.0969	0.9316
18	2013	34	25.2571	72.8571	958.3041	4.7898	1.3592
33	2013	34	24.3286	70.3469	898.8367	6.7398	2.0969
56	2013	34	25.9143	78.9490	971.0347	4.3010	1.1184

77	2013	34	26.8714	60.3571	928.7939	7.1173	1.4286
54	2013	34	20.0286	81.5918	831.5163	5.0571	1.4908
21	2013	34	24.3286	70.3469	898.8367	6.7398	2.0969
68	2013	34	26.2143	72.8878	971.7847	5.1418	1.1990
74	2013	34	27.3000	65.9592	956.5255	6.1633	1.5459
88	2013	34	21.8143	75.8469	872.7031	4.6878	2.6112
16	2013	34	24.9714	67.7041	917.0980	7.3112	1.6908
30	2013	34	22.3286	80.4286	894.1408	4.3898	2.1245
6	2013	34	26.2143	72.8878	971.7847	5.1418	1.1990
49	2013	34	24.6857	68.5816	935.3918	4.6357	1.4071
22	2013	34	21.8143	75.8469	872.7031	4.6878	2.6112
45	2013	34	19.4571	79.7755	817.1439	4.5816	1.9163
58	2013	34	24.6857	68.5816	935.3918	4.6357	1.4071
37	2013	34	26.2143	72.8878	971.7847	5.1418	1.1990
17	2013	34	22.3286	80.0204	898.9010	3.0980	3.3347
55	2013	34	22.7143	76.3061	875.3827	4.9969	2.7204
46	2013	34	24.9714	67.7041	917.0980	7.3112	1.6908
86	2013	34	22.1571	70.6735	864.7653	5.6735	1.5153
2	2013	34	22.1571	70.6735	864.7653	5.6735	1.5153
4	2013	34	24.3286	70.3469	898.8367	6.7398	2.0969
47	2013	34	25.5143	81.7959	954.0867	3.6571	0.7745
82	2013	34	21.8143	75.8469	872.7031	4.6878	2.6112
19	2013	34	25.4857	79.4184	955.0133	4.0184	0.8276
20	2013	34	20.9857	77.1224	852.6276	5.6765	2.5980
80	2013	34	21.8143	75.8469	872.7031	4.6878	2.6112
3	2013	34	25.1571	80.2347	940.4786	4.0969	0.9316
52	2013	34	22.3286	80.0204	898.9010	3.0980	3.3347
70	2013	34	24.1714	69.6020	906.9847	5.5908	2.2847
64	2013	34	17.2286	80.2653	776.7480	4.4204	2.4378
48	2013	34	26.8714	60.3571	928.7939	7.1173	1.4286
65	2013	34	22.3286	80.0204	898.9010	3.0980	3.3347
44	2013	34	24.1714	69.6020	906.9847	5.5908	2.2847
75	2013	34	17.2286	80.2653	776.7480	4.4204	2.4378
40	2013	34	23.9000	76.7551	940.1122	4.3847	1.5939
11	2013	34	22.7143	76.3061	875.3827	4.9969	2.7204
35	2013	34	24.0143	76.8571	934.5122	4.9408	1.4520
78	2013	34	22.8429	77.2653	897.0939	4.4959	2.3408
28	2013	34	23.9143	75.5612	924.7541	4.6214	1.5000
39	2013	34	22.3286	80.0204	898.9010	3.0980	3.3347
24	2013	34	24.6857	68.5816	935.3918	4.6357	1.4071
63	2013	34	23.9000	76.7551	940.1122	4.3847	1.5939
62	2013	34	22.3000	73.1020	872.4061	6.1316	1.8469
1	2013	34	21.8143	75.8469	872.7031	4.6878	2.6112
31	2013	35	21.3000	78.0918	844.9388	3.9520	1.2041
79	2013	35	28.0143	82.6531	971.7469	3.4429	1.0398
51	2013	35	24.5286	84.3367	934.1857	3.4806	1.3082
14	2013	35	23.0143	85.6020	893.6010	3.2939	1.9041
67	2013	35	23.2429	85.5408	898.4408	2.2041	3.1092
42	2013	35	22.5429	81.6224	872.3143	3.7449	2.4908
50	2013	35	23.9571	76.3163	898.6969	4.9796	1.9867

43	2013	35	22.5429	81.6224	872.3143	3.7449	2.4908
85	2013	35	25.1286	76.2245	906.9480	4.2082	1.7500
25	2013	35	27.0286	84.5000	970.6378	3.3908	1.0184
69	2013	35	25.7000	74.5918	935.0847	3.6286	1.1092
57	2013	35	23.1286	84.2143	891.3224	3.1806	1.8776
9	2013	35	21.7714	81.2041	852.1776	4.9378	2.5918
72	2013	35	22.9429	79.5204	874.8561	4.0847	2.5857
26	2013	35	22.6429	80.8367	864.7143	4.0816	1.6041
7	2013	35	22.4714	80.1327	857.1378	3.1827	0.8592
83	2013	35	26.6000	83.2143	939.8724	3.8582	0.8806
76	2013	35	24.9429	73.7959	916.8602	5.4816	1.7194
36	2013	35	25.1857	81.7449	924.3235	3.9031	1.3459
81	2013	35	24.5286	84.3367	934.1857	3.4806	1.3082
15	2013	35	25.9571	64.7755	928.6643	5.4082	1.2286
32	2013	35	22.5429	81.6224	872.3143	3.7449	2.4908
73	2013	35	27.2857	73.3469	956.3969	4.6704	1.2531
71	2013	35	25.1857	81.7449	924.3235	3.9031	1.3459
41	2013	35	22.5429	79.3469	872.1541	4.6112	1.3551
10	2013	35	25.8571	85.5408	958.2357	2.8398	1.1673
23	2013	35	17.2571	81.5408	776.4786	4.6796	2.4582
27	2013	35	22.4714	80.1327	857.1378	3.1827	0.8592
60	2013	35	24.5286	84.3367	934.1857	3.4806	1.3082
53	2013	35	21.7714	81.2041	852.1776	4.9378	2.5918
66	2013	35	23.0143	85.6020	893.6010	3.2939	1.9041
59	2013	35	23.1286	84.2143	891.3224	3.1806	1.8776
61	2013	35	27.2857	73.3469	956.3969	4.6704	1.2531
84	2013	35	27.2857	73.3469	956.3969	4.6704	1.2531
38	2013	35	23.1286	84.2143	891.3224	3.1806	1.8776
87	2013	35	24.2286	82.1327	896.4051	3.0429	2.1816
34	2013	35	23.1286	84.2143	891.3224	3.1806	1.8776
29	2013	35	25.7000	74.5918	935.0847	3.6286	1.1092
5	2013	35	21.0000	82.4490	830.9847	4.9776	1.5163
8	2013	35	21.7714	81.2041	852.1776	4.9378	2.5918
12	2013	35	21.0000	82.4490	830.9847	4.9776	1.5163
13	2013	35	26.6000	83.2143	939.8724	3.8582	0.8806
18	2013	35	26.5286	81.5408	958.3051	3.1735	1.2214
33	2013	35	23.9571	76.3163	898.6969	4.9796	1.9867
56	2013	35	27.0286	84.5000	970.6378	3.3908	1.0184
77	2013	35	25.9571	64.7755	928.6643	5.4082	1.2286
54	2013	35	21.0000	82.4490	830.9847	4.9776	1.5163
21	2013	35	23.9571	76.3163	898.6969	4.9796	1.9867
68	2013	35	28.0143	82.6531	971.7469	3.4429	1.0398
74	2013	35	27.2857	73.3469	956.3969	4.6704	1.2531
88	2013	35	22.5429	81.6224	872.3143	3.7449	2.4908
16	2013	35	24.9429	73.7959	916.8602	5.4816	1.7194
30	2013	35	23.0143	85.6020	893.6010	3.2939	1.9041
6	2013	35	28.0143	82.6531	971.7469	3.4429	1.0398
49	2013	35	25.7000	74.5918	935.0847	3.6286	1.1092
22	2013	35	22.5429	81.6224	872.3143	3.7449	2.4908
45	2013	35	20.1000	80.2653	816.6102	4.8755	1.9480

58	2013	35	25.7000	74.5918	935.0847	3.6286	1.1092
37	2013	35	28.0143	82.6531	971.7469	3.4429	1.0398
17	2013	35	23.2429	85.5408	898.4408	2.2041	3.1092
55	2013	35	22.9429	79.5204	874.8561	4.0847	2.5857
46	2013	35	24.9429	73.7959	916.8602	5.4816	1.7194
86	2013	35	22.7000	77.0408	864.1633	4.2143	1.3633
2	2013	35	22.7000	77.0408	864.1633	4.2143	1.3633
4	2013	35	23.9571	76.3163	898.6969	4.9796	1.9867
47	2013	35	26.6286	85.7551	953.5551	3.1888	0.6153
82	2013	35	22.5429	81.6224	872.3143	3.7449	2.4908
19	2013	35	26.4429	82.0000	954.4816	3.2245	0.7959
20	2013	35	21.7714	81.2041	852.1776	4.9378	2.5918
80	2013	35	22.5429	81.6224	872.3143	3.7449	2.4908
3	2013	35	26.6000	83.2143	939.8724	3.8582	0.8806
52	2013	35	23.2429	85.5408	898.4408	2.2041	3.1092
70	2013	35	25.1286	76.2245	906.9480	4.2082	1.7500
64	2013	35	17.2571	81.5408	776.4786	4.6796	2.4582
48	2013	35	25.9571	64.7755	928.6643	5.4082	1.2286
65	2013	35	23.2429	85.5408	898.4408	2.2041	3.1092
44	2013	35	25.1286	76.2245	906.9480	4.2082	1.7500
75	2013	35	17.2571	81.5408	776.4786	4.6796	2.4582
40	2013	35	25.7286	84.6633	939.7571	3.0541	1.4520
11	2013	35	22.9429	79.5204	874.8561	4.0847	2.5857
35	2013	35	24.5286	84.3367	934.1857	3.4806	1.3082
78	2013	35	24.2286	82.1327	896.4051	3.0429	2.1816
28	2013	35	25.1857	81.7449	924.3235	3.9031	1.3459
39	2013	35	23.2429	85.5408	898.4408	2.2041	3.1092
24	2013	35	25.7000	74.5918	935.0847	3.6286	1.1092
63	2013	35	25.7286	84.6633	939.7571	3.0541	1.4520
62	2013	35	22.5429	79.3469	872.1541	4.6112	1.3551
1	2013	35	22.5429	81.6224	872.3143	3.7449	2.4908
31	2013	36	16.7143	79.2551	846.8490	2.4816	1.1439
79	2013	36	20.9571	80.8776	975.9796	3.0622	1.0143
51	2013	36	18.8143	83.3265	937.9112	3.2969	1.3214
14	2013	36	18.1143	88.0510	896.8153	3.0857	1.8622
67	2013	36	17.7143	87.7551	901.6337	2.1796	2.7388
42	2013	36	17.5857	83.0612	874.9684	2.8837	2.4673
50	2013	36	18.2286	79.7551	901.4255	2.9653	1.9469
43	2013	36	17.5857	83.0612	874.9684	2.8837	2.4673
85	2013	36	18.8000	77.7653	909.6827	2.8714	1.6408
25	2013	36	21.5857	83.1429	974.7888	3.6265	1.0939
69	2013	36	19.7857	73.3571	938.6265	3.0133	1.1551
57	2013	36	17.9857	84.5408	894.1031	2.6633	1.6918
9	2013	36	17.0000	82.5510	854.5459	3.3204	2.4888
72	2013	36	18.6000	81.5612	877.5684	2.9531	1.9908
26	2013	36	18.6571	84.7653	867.1694	3.7163	1.6357
7	2013	36	18.1571	82.1429	859.5010	2.2918	0.9143
83	2013	36	22.3429	81.9184	943.0888	4.1643	0.9184
76	2013	36	18.6000	77.7041	920.1929	3.4704	1.8551
36	2013	36	18.9714	81.2551	927.8367	4.0051	1.4082

81	2013	36	18.8143	83.3265	937.9112	3.2969	1.3214
15	2013	36	19.1286	72.2245	932.0551	2.8337	0.9939
32	2013	36	17.5857	83.0612	874.9684	2.8837	2.4673
73	2013	36	20.8286	77.8673	960.3286	3.1531	1.1429
71	2013	36	18.9714	81.2551	927.8367	4.0051	1.4082
41	2013	36	17.8857	81.3367	874.5459	2.9990	1.2837
10	2013	36	20.1571	85.0510	962.3184	2.9612	1.0878
23	2013	36	13.1000	83.6429	777.7929	3.1092	2.2214
27	2013	36	18.1571	82.1429	859.5010	2.2918	0.9143
60	2013	36	18.8143	83.3265	937.9112	3.2969	1.3214
53	2013	36	17.0000	82.5510	854.5459	3.3204	2.4888
66	2013	36	18.1143	88.0510	896.8153	3.0857	1.8622
59	2013	36	17.9857	84.5408	894.1031	2.6633	1.6918
61	2013	36	20.8286	77.8673	960.3286	3.1531	1.1429
84	2013	36	20.8286	77.8673	960.3286	3.1531	1.1429
38	2013	36	17.9857	84.5408	894.1031	2.6633	1.6918
87	2013	36	19.5143	81.6020	898.9602	2.8786	1.9898
34	2013	36	17.9857	84.5408	894.1031	2.6633	1.6918
29	2013	36	19.7857	73.3571	938.6265	3.0133	1.1551
5	2013	36	16.5857	84.0510	832.9939	3.9276	1.4786
8	2013	36	17.0000	82.5510	854.5459	3.3204	2.4888
12	2013	36	16.5857	84.0510	832.9939	3.9276	1.4786
13	2013	36	22.3429	81.9184	943.0888	4.1643	0.9184
18	2013	36	19.5571	81.2143	962.3327	2.7163	1.2296
33	2013	36	18.2286	79.7551	901.4255	2.9653	1.9469
56	2013	36	21.5857	83.1429	974.7888	3.6265	1.0939
77	2013	36	19.1286	72.2245	932.0551	2.8337	0.9939
54	2013	36	16.5857	84.0510	832.9939	3.9276	1.4786
21	2013	36	18.2286	79.7551	901.4255	2.9653	1.9469
68	2013	36	20.9571	80.8776	975.9796	3.0622	1.0143
74	2013	36	20.8286	77.8673	960.3286	3.1531	1.1429
88	2013	36	17.5857	83.0612	874.9684	2.8837	2.4673
16	2013	36	18.6000	77.7041	920.1929	3.4704	1.8551
30	2013	36	18.1143	88.0510	896.8153	3.0857	1.8622
6	2013	36	20.9571	80.8776	975.9796	3.0622	1.0143
49	2013	36	19.7857	73.3571	938.6265	3.0133	1.1551
22	2013	36	17.5857	83.0612	874.9684	2.8837	2.4673
45	2013	36	16.2714	82.4490	818.4520	3.8031	1.7765
58	2013	36	19.7857	73.3571	938.6265	3.0133	1.1551
37	2013	36	20.9571	80.8776	975.9796	3.0622	1.0143
17	2013	36	17.7143	87.7551	901.6337	2.1796	2.7388
55	2013	36	18.6000	81.5612	877.5684	2.9531	1.9908
46	2013	36	18.6000	77.7041	920.1929	3.4704	1.8551
86	2013	36	18.2714	79.5102	866.2367	2.9735	1.4306
2	2013	36	18.2714	79.5102	866.2367	2.9735	1.4306
4	2013	36	18.2286	79.7551	901.4255	2.9653	1.9469
47	2013	36	22.3143	85.5306	957.0643	3.3755	0.5673
82	2013	36	17.5857	83.0612	874.9684	2.8837	2.4673
19	2013	36	22.0000	81.0612	958.3194	3.1878	0.9306
20	2013	36	17.0000	82.5510	854.5459	3.3204	2.4888

80	2013	36	17.5857	83.0612	874.9684	2.8837	2.4673
3	2013	36	22.3429	81.9184	943.0888	4.1643	0.9184
52	2013	36	17.7143	87.7551	901.6337	2.1796	2.7388
70	2013	36	18.8000	77.7653	909.6827	2.8714	1.6408
64	2013	36	13.1000	83.6429	777.7929	3.1092	2.2214
48	2013	36	19.1286	72.2245	932.0551	2.8337	0.9939
65	2013	36	17.7143	87.7551	901.6337	2.1796	2.7388
44	2013	36	18.8000	77.7653	909.6827	2.8714	1.6408
75	2013	36	13.1000	83.6429	777.7929	3.1092	2.2214
40	2013	36	19.3714	82.9796	943.6939	3.3643	1.6327
11	2013	36	18.6000	81.5612	877.5684	2.9531	1.9908
35	2013	36	18.8143	83.3265	937.9112	3.2969	1.3214
78	2013	36	19.5143	81.6020	898.9602	2.8786	1.9898
28	2013	36	18.9714	81.2551	927.8367	4.0051	1.4082
39	2013	36	17.7143	87.7551	901.6337	2.1796	2.7388
24	2013	36	19.7857	73.3571	938.6265	3.0133	1.1551
63	2013	36	19.3714	82.9796	943.6939	3.3643	1.6327
62	2013	36	17.8857	81.3367	874.5459	2.9990	1.2837
1	2013	36	17.5857	83.0612	874.9684	2.8837	2.4673
31	2013	37	19.6000	80.7653	849.5214	2.1857	0.9531
79	2013	37	25.8714	81.6837	980.2061	2.6643	0.9776
51	2013	37	24.2000	82.1735	941.6888	3.0643	1.3653
14	2013	37	22.5143	87.6939	900.1163	2.8378	1.7837
67	2013	37	23.0143	87.4694	904.9714	2.5255	2.5163
42	2013	37	22.1429	83.4898	877.9888	2.6163	2.4388
50	2013	37	21.4571	84.0102	904.8224	1.6122	1.8112
43	2013	37	22.1429	83.4898	877.9888	2.6163	2.4388
85	2013	37	22.2429	82.0204	913.2500	1.5337	1.7163
25	2013	37	26.1286	84.1122	978.8878	3.1837	1.0051
69	2013	37	24.4000	74.5000	942.3929	2.7551	1.2265
57	2013	37	22.3571	84.4490	897.3531	2.3949	1.6541
9	2013	37	21.4286	82.4490	857.3449	2.7347	2.4102
72	2013	37	21.9714	82.1633	880.6480	3.4867	1.9143
26	2013	37	22.0000	85.7245	870.1541	4.0041	1.7429
7	2013	37	21.4000	82.9184	862.4041	2.2765	1.1051
83	2013	37	25.5571	81.7143	946.7551	4.1857	0.9163
76	2013	37	23.8143	80.2857	923.8143	2.8592	1.6327
36	2013	37	24.4429	81.8980	931.4663	3.7051	1.3316
81	2013	37	24.2000	82.1735	941.6888	3.0643	1.3653
15	2013	37	22.9286	79.3776	935.9439	1.0622	0.8173
32	2013	37	22.1429	83.4898	877.9888	2.6163	2.4388
73	2013	37	25.9143	80.0204	964.3500	2.4571	0.9980
71	2013	37	24.4429	81.8980	931.4663	3.7051	1.3316
41	2013	37	21.6857	81.7143	877.5041	2.7531	1.2939
10	2013	37	24.8143	84.5000	966.3765	2.6337	0.9765
23	2013	37	16.4429	85.3061	779.7214	1.8939	2.1224
27	2013	37	21.4000	82.9184	862.4041	2.2765	1.1051
60	2013	37	24.2000	82.1735	941.6888	3.0643	1.3653
53	2013	37	21.4286	82.4490	857.3449	2.7347	2.4102
66	2013	37	22.5143	87.6939	900.1163	2.8378	1.7837

59	2013	37	22.3571	84.4490	897.3531	2.3949	1.6541
61	2013	37	25.9143	80.0204	964.3500	2.4571	0.9980
84	2013	37	25.9143	80.0204	964.3500	2.4571	0.9980
38	2013	37	22.3571	84.4490	897.3531	2.3949	1.6541
87	2013	37	23.5429	80.6939	902.1857	3.7133	2.0357
34	2013	37	22.3571	84.4490	897.3531	2.3949	1.6541
29	2013	37	24.4000	74.5000	942.3929	2.7551	1.2265
5	2013	37	19.6571	84.7143	835.5939	3.2673	1.5265
8	2013	37	21.4286	82.4490	857.3449	2.7347	2.4102
12	2013	37	19.6571	84.7143	835.5939	3.2673	1.5265
13	2013	37	25.5571	81.7143	946.7551	4.1857	0.9163
18	2013	37	24.3429	83.0714	966.4020	2.1490	1.0418
33	2013	37	21.4571	84.0102	904.8224	1.6122	1.8112
56	2013	37	26.1286	84.1122	978.8878	3.1837	1.0051
77	2013	37	22.9286	79.3776	935.9439	1.0622	0.8173
54	2013	37	19.6571	84.7143	835.5939	3.2673	1.5265
21	2013	37	21.4571	84.0102	904.8224	1.6122	1.8112
68	2013	37	25.8714	81.6837	980.2061	2.6643	0.9776
74	2013	37	25.9143	80.0204	964.3500	2.4571	0.9980
88	2013	37	22.1429	83.4898	877.9888	2.6163	2.4388
16	2013	37	23.8143	80.2857	923.8143	2.8592	1.6327
30	2013	37	22.5143	87.6939	900.1163	2.8378	1.7837
6	2013	37	25.8714	81.6837	980.2061	2.6643	0.9776
49	2013	37	24.4000	74.5000	942.3929	2.7551	1.2265
22	2013	37	22.1429	83.4898	877.9888	2.6163	2.4388
45	2013	37	19.0000	83.7959	820.9010	3.3582	1.7459
58	2013	37	24.4000	74.5000	942.3929	2.7551	1.2265
37	2013	37	25.8714	81.6837	980.2061	2.6643	0.9776
17	2013	37	23.0143	87.4694	904.9714	2.5255	2.5163
55	2013	37	21.9714	82.1633	880.6480	3.4867	1.9143
46	2013	37	23.8143	80.2857	923.8143	2.8592	1.6327
86	2013	37	21.7286	79.7653	869.0694	2.8316	1.4643
2	2013	37	21.7286	79.7653	869.0694	2.8316	1.4643
4	2013	37	21.4571	84.0102	904.8224	1.6122	1.8112
47	2013	37	25.1857	85.1020	960.8500	3.4255	0.4969
82	2013	37	22.1429	83.4898	877.9888	2.6163	2.4388
19	2013	37	25.6571	79.4490	962.1561	3.2245	0.9857
20	2013	37	21.4286	82.4490	857.3449	2.7347	2.4102
80	2013	37	22.1429	83.4898	877.9888	2.6163	2.4388
3	2013	37	25.5571	81.7143	946.7551	4.1857	0.9163
52	2013	37	23.0143	87.4694	904.9714	2.5255	2.5163
70	2013	37	22.2429	82.0204	913.2500	1.5337	1.7163
64	2013	37	16.4429	85.3061	779.7214	1.8939	2.1224
48	2013	37	22.9286	79.3776	935.9439	1.0622	0.8173
65	2013	37	23.0143	87.4694	904.9714	2.5255	2.5163
44	2013	37	22.2429	82.0204	913.2500	1.5337	1.7163
75	2013	37	16.4429	85.3061	779.7214	1.8939	2.1224
40	2013	37	24.7571	83.1429	947.5061	3.4500	1.6510
11	2013	37	21.9714	82.1633	880.6480	3.4867	1.9143
35	2013	37	24.2000	82.1735	941.6888	3.0643	1.3653

78	2013	37	23.5429	80.6939	902.1857	3.7133	2.0357
28	2013	37	24.4429	81.8980	931.4663	3.7051	1.3316
39	2013	37	23.0143	87.4694	904.9714	2.5255	2.5163
24	2013	37	24.4000	74.5000	942.3929	2.7551	1.2265
63	2013	37	24.7571	83.1429	947.5061	3.4500	1.6510
62	2013	37	21.6857	81.7143	877.5041	2.7531	1.2939
1	2013	37	22.1429	83.4898	877.9888	2.6163	2.4388
31	2013	38	21.7857	75.3061	849.1020	4.9531	1.0347
79	2013	38	26.9000	78.8776	979.2408	5.5531	1.0163
51	2013	38	23.4857	77.1837	941.0490	5.7878	1.1663
14	2013	38	22.1571	83.2857	900.0000	5.1480	1.5520
67	2013	38	22.5714	80.2653	904.6663	4.4806	2.2316
42	2013	38	22.5000	77.0306	877.8082	4.7622	2.2847
50	2013	38	24.1857	76.1429	904.2643	4.3786	2.0276
43	2013	38	22.5000	77.0306	877.8082	4.7622	2.2847
85	2013	38	26.0571	71.9898	912.4776	4.2276	2.1673
25	2013	38	26.0429	81.0612	978.2755	5.4388	1.1163
69	2013	38	24.2714	70.7041	941.7286	6.0827	1.2857
57	2013	38	23.0857	76.1020	896.8520	4.7276	1.6990
9	2013	38	21.6000	77.3061	857.2704	5.3582	2.1969
72	2013	38	21.7429	81.1531	880.6214	6.3469	1.3531
26	2013	38	21.6000	82.8980	870.2194	6.0071	1.8990
7	2013	38	20.9143	80.5306	862.4418	4.2102	1.4194
83	2013	38	25.6429	78.2551	946.5408	6.4990	0.9276
76	2013	38	24.2571	74.2143	923.1378	6.5469	1.4561
36	2013	38	23.7143	76.6939	930.9224	6.8347	1.3082
81	2013	38	23.4857	77.1837	941.0490	5.7878	1.1663
15	2013	38	25.4143	71.9694	935.1061	3.5541	0.9735
32	2013	38	22.5000	77.0306	877.8082	4.7622	2.2847
73	2013	38	26.9000	73.7551	963.3796	5.9531	0.9531
71	2013	38	23.7143	76.6939	930.9224	6.8347	1.3082
41	2013	38	22.7571	75.1224	877.1367	5.5612	1.4347
10	2013	38	24.6857	79.5000	965.6398	5.6204	1.0847
23	2013	38	17.7143	80.2245	779.6837	4.8888	2.1684
27	2013	38	20.9143	80.5306	862.4418	4.2102	1.4194
60	2013	38	23.4857	77.1837	941.0490	5.7878	1.1663
53	2013	38	21.6000	77.3061	857.2704	5.3582	2.1969
66	2013	38	22.1571	83.2857	900.0000	5.1480	1.5520
59	2013	38	23.0857	76.1020	896.8520	4.7276	1.6990
61	2013	38	26.9000	73.7551	963.3796	5.9531	0.9531
84	2013	38	26.9000	73.7551	963.3796	5.9531	0.9531
38	2013	38	23.0857	76.1020	896.8520	4.7276	1.6990
87	2013	38	23.8143	76.0102	901.9786	6.2265	1.6173
34	2013	38	23.0857	76.1020	896.8520	4.7276	1.6990
29	2013	38	24.2714	70.7041	941.7286	6.0827	1.2857
5	2013	38	19.6429	81.7959	835.6582	5.1633	1.4408
8	2013	38	21.6000	77.3061	857.2704	5.3582	2.1969
12	2013	38	19.6429	81.7959	835.6582	5.1633	1.4408
13	2013	38	25.6429	78.2551	946.5408	6.4990	0.9276
18	2013	38	25.3000	80.6939	965.5000	5.0847	0.9051

33	2013	38	24.1857	76.1429	904.2643	4.3786	2.0276
56	2013	38	26.0429	81.0612	978.2755	5.4388	1.1163
77	2013	38	25.4143	71.9694	935.1061	3.5541	0.9735
54	2013	38	19.6429	81.7959	835.6582	5.1633	1.4408
21	2013	38	24.1857	76.1429	904.2643	4.3786	2.0276
68	2013	38	26.9000	78.8776	979.2408	5.5531	1.0163
74	2013	38	26.9000	73.7551	963.3796	5.9531	0.9531
88	2013	38	22.5000	77.0306	877.8082	4.7622	2.2847
16	2013	38	24.2571	74.2143	923.1378	6.5469	1.4561
30	2013	38	22.1571	83.2857	900.0000	5.1480	1.5520
6	2013	38	26.9000	78.8776	979.2408	5.5531	1.0163
49	2013	38	24.2714	70.7041	941.7286	6.0827	1.2857
22	2013	38	22.5000	77.0306	877.8082	4.7622	2.2847
45	2013	38	18.9571	80.1020	821.0245	5.3173	1.6959
58	2013	38	24.2714	70.7041	941.7286	6.0827	1.2857
37	2013	38	26.9000	78.8776	979.2408	5.5531	1.0163
17	2013	38	22.5714	80.2653	904.6663	4.4806	2.2316
55	2013	38	21.7429	81.1531	880.6214	6.3469	1.3531
46	2013	38	24.2571	74.2143	923.1378	6.5469	1.4561
86	2013	38	22.3571	75.0510	868.7673	5.5765	1.5143
2	2013	38	22.3571	75.0510	868.7673	5.5765	1.5143
4	2013	38	24.1857	76.1429	904.2643	4.3786	2.0276
47	2013	38	25.7429	83.0510	960.4990	5.6929	0.5122
82	2013	38	22.5000	77.0306	877.8082	4.7622	2.2847
19	2013	38	25.3857	77.8163	961.8194	5.4663	0.9388
20	2013	38	21.6000	77.3061	857.2704	5.3582	2.1969
80	2013	38	22.5000	77.0306	877.8082	4.7622	2.2847
3	2013	38	25.6429	78.2551	946.5408	6.4990	0.9276
52	2013	38	22.5714	80.2653	904.6663	4.4806	2.2316
70	2013	38	26.0571	71.9898	912.4776	4.2276	2.1673
64	2013	38	17.7143	80.2245	779.6837	4.8888	2.1684
48	2013	38	25.4143	71.9694	935.1061	3.5541	0.9735
65	2013	38	22.5714	80.2653	904.6663	4.4806	2.2316
44	2013	38	26.0571	71.9898	912.4776	4.2276	2.1673
75	2013	38	17.7143	80.2245	779.6837	4.8888	2.1684
40	2013	38	24.2714	77.7449	946.9755	6.2612	1.4051
11	2013	38	21.7429	81.1531	880.6214	6.3469	1.3531
35	2013	38	23.4857	77.1837	941.0490	5.7878	1.1663
78	2013	38	23.8143	76.0102	901.9786	6.2265	1.6173
28	2013	38	23.7143	76.6939	930.9224	6.8347	1.3082
39	2013	38	22.5714	80.2653	904.6663	4.4806	2.2316
24	2013	38	24.2714	70.7041	941.7286	6.0827	1.2857
63	2013	38	24.2714	77.7449	946.9755	6.2612	1.4051
62	2013	38	22.7571	75.1224	877.1367	5.5612	1.4347
1	2013	38	22.5000	77.0306	877.8082	4.7622	2.2847
31	2013	39	15.8143	72.8061	848.4510	5.2745	1.1704
79	2013	39	18.6143	78.2347	978.5561	5.1439	1.0235
51	2013	39	16.4286	77.2551	940.1551	4.9480	1.2622
14	2013	39	16.2286	81.4388	898.6082	5.0622	1.8622
67	2013	39	15.8143	79.9490	903.6000	3.7929	2.9143

42	2013	39	15.8429	75.5204	876.8571	4.5286	2.5949
50	2013	39	17.9857	71.1633	903.5694	5.2459	2.0612
43	2013	39	15.8429	75.5204	876.8571	4.5286	2.5949
85	2013	39	18.5429	66.5612	911.6571	4.9969	2.0020
25	2013	39	19.5571	79.1939	976.9112	5.0602	1.2500
69	2013	39	17.8571	70.4796	940.9990	5.8316	1.2755
57	2013	39	16.1143	76.7143	896.0724	3.9551	1.6092
9	2013	39	16.1000	75.0510	856.2827	5.1806	2.4755
72	2013	39	17.4714	78.6122	879.4327	5.6847	1.5786
26	2013	39	18.8714	79.1735	869.0296	5.6286	1.8071
7	2013	39	17.7714	77.7653	861.3378	4.0867	1.5633
83	2013	39	21.0571	76.4898	945.1143	5.7673	1.0071
76	2013	39	17.8286	72.5714	922.4602	6.8092	1.5143
36	2013	39	16.4714	77.3673	930.0092	5.9143	1.4245
81	2013	39	16.4286	77.2551	940.1551	4.9480	1.2622
15	2013	39	19.3429	67.1531	934.4245	4.4714	1.1041
32	2013	39	15.8429	75.5204	876.8571	4.5286	2.5949
73	2013	39	19.8714	70.9898	962.7520	6.0031	1.0806
71	2013	39	16.4714	77.3673	930.0092	5.9143	1.4245
41	2013	39	16.7571	73.4388	876.4245	5.2214	1.4082
10	2013	39	17.2143	79.4286	964.6112	5.2061	1.2480
23	2013	39	12.9857	77.1020	779.1439	6.1286	2.3102
27	2013	39	17.7714	77.7653	861.3378	4.0867	1.5633
60	2013	39	16.4286	77.2551	940.1551	4.9480	1.2622
53	2013	39	16.1000	75.0510	856.2827	5.1806	2.4755
66	2013	39	16.2286	81.4388	898.6082	5.0622	1.8622
59	2013	39	16.1143	76.7143	896.0724	3.9551	1.6092
61	2013	39	19.8714	70.9898	962.7520	6.0031	1.0806
84	2013	39	19.8714	70.9898	962.7520	6.0031	1.0806
38	2013	39	16.1143	76.7143	896.0724	3.9551	1.6092
87	2013	39	17.3429	74.9694	900.9041	5.5378	2.0214
34	2013	39	16.1143	76.7143	896.0724	3.9551	1.6092
29	2013	39	17.8571	70.4796	940.9990	5.8316	1.2755
5	2013	39	16.0714	80.0612	834.7367	5.4276	1.3469
8	2013	39	16.1000	75.0510	856.2827	5.1806	2.4755
12	2013	39	16.0714	80.0612	834.7367	5.4276	1.3469
13	2013	39	21.0571	76.4898	945.1143	5.7673	1.0071
18	2013	39	18.3429	78.2347	964.8551	5.2531	1.1735
33	2013	39	17.9857	71.1633	903.5694	5.2459	2.0612
56	2013	39	19.5571	79.1939	976.9112	5.0602	1.2500
77	2013	39	19.3429	67.1531	934.4245	4.4714	1.1041
54	2013	39	16.0714	80.0612	834.7367	5.4276	1.3469
21	2013	39	17.9857	71.1633	903.5694	5.2459	2.0612
68	2013	39	18.6143	78.2347	978.5561	5.1439	1.0235
74	2013	39	19.8714	70.9898	962.7520	6.0031	1.0806
88	2013	39	15.8429	75.5204	876.8571	4.5286	2.5949
16	2013	39	17.8286	72.5714	922.4602	6.8092	1.5143
30	2013	39	16.2286	81.4388	898.6082	5.0622	1.8622
6	2013	39	18.6143	78.2347	978.5561	5.1439	1.0235
49	2013	39	17.8571	70.4796	940.9990	5.8316	1.2755

22	2013	39	15.8429	75.5204	876.8571	4.5286	2.5949
45	2013	39	16.1857	76.7449	820.1367	5.5429	1.7612
58	2013	39	17.8571	70.4796	940.9990	5.8316	1.2755
37	2013	39	18.6143	78.2347	978.5561	5.1439	1.0235
17	2013	39	15.8143	79.9490	903.6000	3.7929	2.9143
55	2013	39	17.4714	78.6122	879.4327	5.6847	1.5786
46	2013	39	17.8286	72.5714	922.4602	6.8092	1.5143
86	2013	39	16.6286	74.8367	868.0051	5.6286	1.4224
2	2013	39	16.6286	74.8367	868.0051	5.6286	1.4224
4	2013	39	17.9857	71.1633	903.5694	5.2459	2.0612
47	2013	39	20.3571	82.2959	959.1286	5.2898	0.6327
82	2013	39	15.8429	75.5204	876.8571	4.5286	2.5949
19	2013	39	19.8571	76.4694	960.2469	5.3378	0.9765
20	2013	39	16.1000	75.0510	856.2827	5.1806	2.4755
80	2013	39	15.8429	75.5204	876.8571	4.5286	2.5949
3	2013	39	21.0571	76.4898	945.1143	5.7673	1.0071
52	2013	39	15.8143	79.9490	903.6000	3.7929	2.9143
70	2013	39	18.5429	66.5612	911.6571	4.9969	2.0020
64	2013	39	12.9857	77.1020	779.1439	6.1286	2.3102
48	2013	39	19.3429	67.1531	934.4245	4.4714	1.1041
65	2013	39	15.8143	79.9490	903.6000	3.7929	2.9143
44	2013	39	18.5429	66.5612	911.6571	4.9969	2.0020
75	2013	39	12.9857	77.1020	779.1439	6.1286	2.3102
40	2013	39	16.3571	78.3673	945.6388	5.3806	1.4929
11	2013	39	17.4714	78.6122	879.4327	5.6847	1.5786
35	2013	39	16.4286	77.2551	940.1551	4.9480	1.2622
78	2013	39	17.3429	74.9694	900.9041	5.5378	2.0214
28	2013	39	16.4714	77.3673	930.0092	5.9143	1.4245
39	2013	39	15.8143	79.9490	903.6000	3.7929	2.9143
24	2013	39	17.8571	70.4796	940.9990	5.8316	1.2755
63	2013	39	16.3571	78.3673	945.6388	5.3806	1.4929
62	2013	39	16.7571	73.4388	876.4245	5.2214	1.4082
1	2013	39	15.8429	75.5204	876.8571	4.5286	2.5949
31	2013	40	14.9571	75.0306	850.8704	4.2337	1.0245
79	2013	40	21.0429	81.7959	982.0082	3.3276	0.8888
51	2013	40	18.6143	79.5918	943.0704	3.6673	1.3857
14	2013	40	18.2143	78.6531	900.6949	4.0551	2.0796
67	2013	40	17.8571	79.2755	905.9847	3.1592	3.3622
42	2013	40	16.7429	76.3980	879.0602	3.8898	2.6745
50	2013	40	17.5571	73.6837	906.4041	4.9143	1.8571
43	2013	40	16.7429	76.3980	879.0602	3.8898	2.6745
85	2013	40	18.3857	72.1020	914.7827	4.2765	1.4551
25	2013	40	22.0000	77.8980	980.0612	4.1163	1.2469
69	2013	40	18.4143	73.2857	944.0827	4.0276	1.0847
57	2013	40	16.4000	82.0816	898.7735	3.4592	1.5112
9	2013	40	16.1000	73.7551	858.2531	3.9020	2.4959
72	2013	40	17.7286	75.1429	881.4541	4.1980	1.9082
26	2013	40	17.8000	76.4898	870.8816	4.4724	1.6439
7	2013	40	16.8857	75.5000	863.2082	3.3092	1.5031
83	2013	40	21.3286	76.9082	947.7959	4.0010	0.9418

76	2013	40	18.0571	75.6327	925.3510	5.4184	1.4286
36	2013	40	18.8571	78.4184	932.8102	4.0918	1.5582
81	2013	40	18.6143	79.5918	943.0704	3.6673	1.3857
15	2013	40	19.2571	69.1429	937.5388	4.1418	1.0061
32	2013	40	16.7429	76.3980	879.0602	3.8898	2.6745
73	2013	40	20.8000	74.3878	966.1684	4.3296	1.0806
71	2013	40	18.8571	78.4184	932.8102	4.0918	1.5582
41	2013	40	16.2286	75.4388	878.9439	4.0980	1.2347
10	2013	40	19.8000	81.9694	967.8000	3.6204	1.1694
23	2013	40	11.6857	77.2857	780.6878	4.7592	2.0776
27	2013	40	16.8857	75.5000	863.2082	3.3092	1.5031
60	2013	40	18.6143	79.5918	943.0704	3.6673	1.3857
53	2013	40	16.1000	73.7551	858.2531	3.9020	2.4959
66	2013	40	18.2143	78.6531	900.6949	4.0551	2.0796
59	2013	40	16.4000	82.0816	898.7735	3.4592	1.5112
61	2013	40	20.8000	74.3878	966.1684	4.3296	1.0806
84	2013	40	20.8000	74.3878	966.1684	4.3296	1.0806
38	2013	40	16.4000	82.0816	898.7735	3.4592	1.5112
87	2013	40	18.5286	75.3571	903.2959	4.1929	2.3500
34	2013	40	16.4000	82.0816	898.7735	3.4592	1.5112
29	2013	40	18.4143	73.2857	944.0827	4.0276	1.0847
5	2013	40	15.0714	78.5102	836.4857	4.3306	1.4643
8	2013	40	16.1000	73.7551	858.2531	3.9020	2.4959
12	2013	40	15.0714	78.5102	836.4857	4.3306	1.4643
13	2013	40	21.3286	76.9082	947.7959	4.0010	0.9418
18	2013	40	19.6714	79.8776	968.0612	4.0082	1.1582
33	2013	40	17.5571	73.6837	906.4041	4.9143	1.8571
56	2013	40	22.0000	77.8980	980.0612	4.1163	1.2469
77	2013	40	19.2571	69.1429	937.5388	4.1418	1.0061
54	2013	40	15.0714	78.5102	836.4857	4.3306	1.4643
21	2013	40	17.5571	73.6837	906.4041	4.9143	1.8571
68	2013	40	21.0429	81.7959	982.0082	3.3276	0.8888
74	2013	40	20.8000	74.3878	966.1684	4.3296	1.0806
88	2013	40	16.7429	76.3980	879.0602	3.8898	2.6745
16	2013	40	18.0571	75.6327	925.3510	5.4184	1.4286
30	2013	40	18.2143	78.6531	900.6949	4.0551	2.0796
6	2013	40	21.0429	81.7959	982.0082	3.3276	0.8888
49	2013	40	18.4143	73.2857	944.0827	4.0276	1.0847
22	2013	40	16.7429	76.3980	879.0602	3.8898	2.6745
45	2013	40	14.6571	75.7857	821.7449	4.7408	1.6878
58	2013	40	18.4143	73.2857	944.0827	4.0276	1.0847
37	2013	40	21.0429	81.7959	982.0082	3.3276	0.8888
17	2013	40	17.8571	79.2755	905.9847	3.1592	3.3622
55	2013	40	17.7286	75.1429	881.4541	4.1980	1.9082
46	2013	40	18.0571	75.6327	925.3510	5.4184	1.4286
86	2013	40	16.2000	77.2857	870.3878	4.2214	1.2653
2	2013	40	16.2000	77.2857	870.3878	4.2214	1.2653
4	2013	40	17.5571	73.6837	906.4041	4.9143	1.8571
47	2013	40	21.7286	80.1327	961.9949	3.5969	0.6520
82	2013	40	16.7429	76.3980	879.0602	3.8898	2.6745

19	2013	40	21.8286	74.0408	962.9755	4.4429	1.0633
20	2013	40	16.1000	73.7551	858.2531	3.9020	2.4959
80	2013	40	16.7429	76.3980	879.0602	3.8898	2.6745
3	2013	40	21.3286	76.9082	947.7959	4.0010	0.9418
52	2013	40	17.8571	79.2755	905.9847	3.1592	3.3622
70	2013	40	18.3857	72.1020	914.7827	4.2765	1.4551
64	2013	40	11.6857	77.2857	780.6878	4.7592	2.0776
48	2013	40	19.2571	69.1429	937.5388	4.1418	1.0061
65	2013	40	17.8571	79.2755	905.9847	3.1592	3.3622
44	2013	40	18.3857	72.1020	914.7827	4.2765	1.4551
75	2013	40	11.6857	77.2857	780.6878	4.7592	2.0776
40	2013	40	19.4000	80.9184	948.4439	3.8551	1.5173
11	2013	40	17.7286	75.1429	881.4541	4.1980	1.9082
35	2013	40	18.6143	79.5918	943.0704	3.6673	1.3857
78	2013	40	18.5286	75.3571	903.2959	4.1929	2.3500
28	2013	40	18.8571	78.4184	932.8102	4.0918	1.5582
39	2013	40	17.8571	79.2755	905.9847	3.1592	3.3622
24	2013	40	18.4143	73.2857	944.0827	4.0276	1.0847
63	2013	40	19.4000	80.9184	948.4439	3.8551	1.5173
62	2013	40	16.2286	75.4388	878.9439	4.0980	1.2347
1	2013	40	16.7429	76.3980	879.0602	3.8898	2.6745
31	2013	41	15.9143	75.7449	853.0673	4.4306	0.7908
79	2013	41	20.7429	78.6327	984.4531	4.8347	0.8541
51	2013	41	17.4571	75.7551	945.5449	5.6816	1.1878
14	2013	41	18.3143	70.5816	903.1367	5.7051	1.6663
67	2013	41	18.3000	71.5918	908.3439	5.0745	3.1673
42	2013	41	18.3000	71.9898	881.3357	5.1224	2.4041
50	2013	41	19.0286	73.3878	908.8041	6.0000	1.6153
43	2013	41	18.3000	71.9898	881.3357	5.1224	2.4041
85	2013	41	20.7714	73.3367	917.2908	5.3051	1.2000
25	2013	41	20.4286	73.6531	982.9194	5.4000	1.2571
69	2013	41	18.4571	72.0510	946.6480	5.0071	0.9306
57	2013	41	17.3571	80.8571	901.1735	5.2755	1.4102
9	2013	41	17.8143	69.8980	860.4724	4.7929	2.0500
72	2013	41	17.7000	71.3980	883.8204	5.1663	1.9051
26	2013	41	18.0000	75.7245	873.2071	5.2612	1.4551
7	2013	41	16.5429	74.7551	865.4888	3.9602	1.3031
83	2013	41	20.4143	75.8265	950.6204	4.7082	0.8551
76	2013	41	18.8429	74.3673	927.7194	6.4735	1.2806
36	2013	41	18.1714	73.3061	935.2745	5.9357	1.5041
81	2013	41	17.4571	75.7551	945.5449	5.6816	1.1878
15	2013	41	20.3143	68.9286	939.9980	5.6704	0.8776
32	2013	41	18.3000	71.9898	881.3357	5.1224	2.4041
73	2013	41	20.9571	73.4388	968.7184	5.6908	0.9806
71	2013	41	18.1714	73.3061	935.2745	5.9357	1.5041
41	2013	41	17.1571	75.8469	881.2582	5.1398	1.0551
10	2013	41	19.0286	75.7653	970.3918	5.4633	1.0582
23	2013	41	13.1286	75.7857	782.4204	4.6490	1.7806
27	2013	41	16.5429	74.7551	865.4888	3.9602	1.3031
60	2013	41	17.4571	75.7551	945.5449	5.6816	1.1878

53	2013	41	17.8143	69.8980	860.4724	4.7929	2.0500
66	2013	41	18.3143	70.5816	903.1367	5.7051	1.6663
59	2013	41	17.3571	80.8571	901.1735	5.2755	1.4102
61	2013	41	20.9571	73.4388	968.7184	5.6908	0.9806
84	2013	41	20.9571	73.4388	968.7184	5.6908	0.9806
38	2013	41	17.3571	80.8571	901.1735	5.2755	1.4102
87	2013	41	18.1000	71.7857	905.7602	5.1898	2.1224
34	2013	41	17.3571	80.8571	901.1735	5.2755	1.4102
29	2013	41	18.4571	72.0510	946.6480	5.0071	0.9306
5	2013	41	15.6286	76.3980	838.5837	4.9245	1.4622
8	2013	41	17.8143	69.8980	860.4724	4.7929	2.0500
12	2013	41	15.6286	76.3980	838.5837	4.9245	1.4622
13	2013	41	20.4143	75.8265	950.6204	4.7082	0.8551
18	2013	41	18.7857	77.6531	970.3724	5.5704	0.9296
33	2013	41	19.0286	73.3878	908.8041	6.0000	1.6153
56	2013	41	20.4286	73.6531	982.9194	5.4000	1.2571
77	2013	41	20.3143	68.9286	939.9980	5.6704	0.8776
54	2013	41	15.6286	76.3980	838.5837	4.9245	1.4622
21	2013	41	19.0286	73.3878	908.8041	6.0000	1.6153
68	2013	41	20.7429	78.6327	984.4531	4.8347	0.8541
74	2013	41	20.9571	73.4388	968.7184	5.6908	0.9806
88	2013	41	18.3000	71.9898	881.3357	5.1224	2.4041
16	2013	41	18.8429	74.3673	927.7194	6.4735	1.2806
30	2013	41	18.3143	70.5816	903.1367	5.7051	1.6663
6	2013	41	20.7429	78.6327	984.4531	4.8347	0.8541
49	2013	41	18.4571	72.0510	946.6480	5.0071	0.9306
22	2013	41	18.3000	71.9898	881.3357	5.1224	2.4041
45	2013	41	14.4571	75.9592	823.7724	5.2000	1.4112
58	2013	41	18.4571	72.0510	946.6480	5.0071	0.9306
37	2013	41	20.7429	78.6327	984.4531	4.8347	0.8541
17	2013	41	18.3000	71.5918	908.3439	5.0745	3.1673
55	2013	41	17.7000	71.3980	883.8204	5.1663	1.9051
46	2013	41	18.8429	74.3673	927.7194	6.4735	1.2806
86	2013	41	16.9143	75.7551	872.6796	4.7286	1.1622
2	2013	41	16.9143	75.7551	872.6796	4.7286	1.1622
4	2013	41	19.0286	73.3878	908.8041	6.0000	1.6153
47	2013	41	20.2571	76.8673	964.7949	4.5765	0.5735
82	2013	41	18.3000	71.9898	881.3357	5.1224	2.4041
19	2013	41	19.8571	70.1327	965.7929	5.9755	1.0847
20	2013	41	17.8143	69.8980	860.4724	4.7929	2.0500
80	2013	41	18.3000	71.9898	881.3357	5.1224	2.4041
3	2013	41	20.4143	75.8265	950.6204	4.7082	0.8551
52	2013	41	18.3000	71.5918	908.3439	5.0745	3.1673
70	2013	41	20.7714	73.3367	917.2908	5.3051	1.2000
64	2013	41	13.1286	75.7857	782.4204	4.6490	1.7806
48	2013	41	20.3143	68.9286	939.9980	5.6704	0.8776
65	2013	41	18.3000	71.5918	908.3439	5.0745	3.1673
44	2013	41	20.7714	73.3367	917.2908	5.3051	1.2000
75	2013	41	13.1286	75.7857	782.4204	4.6490	1.7806
40	2013	41	19.0143	72.5714	951.0571	5.6439	1.4031

11	2013	41	17.7000	71.3980	883.8204	5.1663	1.9051
35	2013	41	17.4571	75.7551	945.5449	5.6816	1.1878
78	2013	41	18.1000	71.7857	905.7602	5.1898	2.1224
28	2013	41	18.1714	73.3061	935.2745	5.9357	1.5041
39	2013	41	18.3000	71.5918	908.3439	5.0745	3.1673
24	2013	41	18.4571	72.0510	946.6480	5.0071	0.9306
63	2013	41	19.0143	72.5714	951.0571	5.6439	1.4031
62	2013	41	17.1571	75.8469	881.2582	5.1398	1.0551
1	2013	41	18.3000	71.9898	881.3357	5.1224	2.4041
31	2013	42	12.0143	75.3571	853.3061	4.4796	0.8418
79	2013	42	16.9857	73.1020	985.3122	5.5633	0.9765
51	2013	42	14.6286	73.3776	946.2643	6.0653	1.2143
14	2013	42	14.2000	69.6224	903.7459	5.7612	1.2908
67	2013	42	13.7857	70.5408	908.9041	5.6990	2.8878
42	2013	42	13.1143	70.0714	881.7510	5.6337	2.3020
50	2013	42	14.1714	72.9184	909.1827	5.9612	1.6061
43	2013	42	13.1143	70.0714	881.7510	5.6337	2.3020
85	2013	42	14.7714	71.5204	917.6102	5.4755	1.3469
25	2013	42	17.7286	73.0918	983.7173	5.3541	1.3429
69	2013	42	15.7143	69.7653	947.2469	5.0153	1.1357
57	2013	42	13.3286	79.3878	901.5571	5.5980	1.4643
9	2013	42	12.8857	68.8673	860.8378	5.2622	2.0010
72	2013	42	14.3143	71.7143	884.3122	5.9929	1.8194
26	2013	42	15.4143	74.7143	873.6663	6.1327	1.4327
7	2013	42	14.3000	74.3265	865.9184	4.2143	1.2653
83	2013	42	18.7143	73.9490	951.1816	5.0316	0.9704
76	2013	42	14.3714	72.5204	928.2653	6.3480	1.3939
36	2013	42	14.8571	72.0510	935.8929	6.3316	1.4571
81	2013	42	14.6286	73.3776	946.2643	6.0653	1.2143
15	2013	42	15.2286	69.4592	940.5418	5.7306	0.9061
32	2013	42	13.1143	70.0714	881.7510	5.6337	2.3020
73	2013	42	17.0286	69.9388	969.3878	5.9684	1.0327
71	2013	42	14.8571	72.0510	935.8929	6.3316	1.4571
41	2013	42	13.0429	75.9082	881.5694	5.5347	1.0500
10	2013	42	15.7714	71.9388	971.1765	6.0153	1.1378
23	2013	42	8.8000	75.4898	782.5367	4.7806	1.9255
27	2013	42	14.3000	74.3265	865.9184	4.2143	1.2653
60	2013	42	14.6286	73.3776	946.2643	6.0653	1.2143
53	2013	42	12.8857	68.8673	860.8378	5.2622	2.0010
66	2013	42	14.2000	69.6224	903.7459	5.7612	1.2908
59	2013	42	13.3286	79.3878	901.5571	5.5980	1.4643
61	2013	42	17.0286	69.9388	969.3878	5.9684	1.0327
84	2013	42	17.0286	69.9388	969.3878	5.9684	1.0327
38	2013	42	13.3286	79.3878	901.5571	5.5980	1.4643
87	2013	42	15.0714	71.0918	906.2612	5.8082	1.9663
34	2013	42	13.3286	79.3878	901.5571	5.5980	1.4643
29	2013	42	15.7143	69.7653	947.2469	5.0153	1.1357
5	2013	42	12.4000	75.7143	838.9255	5.6082	1.4969
8	2013	42	12.8857	68.8673	860.8378	5.2622	2.0010
12	2013	42	12.4000	75.7143	838.9255	5.6082	1.4969

13	2013	42	18.7143	73.9490	951.1816	5.0316	0.9704
18	2013	42	15.6571	73.8469	971.2602	5.6765	1.1102
33	2013	42	14.1714	72.9184	909.1827	5.9612	1.6061
56	2013	42	17.7286	73.0918	983.7173	5.3541	1.3429
77	2013	42	15.2286	69.4592	940.5418	5.7306	0.9061
54	2013	42	12.4000	75.7143	838.9255	5.6082	1.4969
21	2013	42	14.1714	72.9184	909.1827	5.9612	1.6061
68	2013	42	16.9857	73.1020	985.3122	5.5633	0.9765
74	2013	42	17.0286	69.9388	969.3878	5.9684	1.0327
88	2013	42	13.1143	70.0714	881.7510	5.6337	2.3020
16	2013	42	14.3714	72.5204	928.2653	6.3480	1.3939
30	2013	42	14.2000	69.6224	903.7459	5.7612	1.2908
6	2013	42	16.9857	73.1020	985.3122	5.5633	0.9765
49	2013	42	15.7143	69.7653	947.2469	5.0153	1.1357
22	2013	42	13.1143	70.0714	881.7510	5.6337	2.3020
45	2013	42	12.6286	76.1837	824.0735	5.5500	1.3908
58	2013	42	15.7143	69.7653	947.2469	5.0153	1.1357
37	2013	42	16.9857	73.1020	985.3122	5.5633	0.9765
17	2013	42	13.7857	70.5408	908.9041	5.6990	2.8878
55	2013	42	14.3143	71.7143	884.3122	5.9929	1.8194
46	2013	42	14.3714	72.5204	928.2653	6.3480	1.3939
86	2013	42	13.4857	74.6020	873.0010	4.8745	1.1622
2	2013	42	13.4857	74.6020	873.0010	4.8745	1.1622
4	2013	42	14.1714	72.9184	909.1827	5.9612	1.6061
47	2013	42	18.4714	77.0816	965.4286	5.1061	0.5041
82	2013	42	13.1143	70.0714	881.7510	5.6337	2.3020
19	2013	42	18.0143	69.7959	966.5204	6.3735	1.0827
20	2013	42	12.8857	68.8673	860.8378	5.2622	2.0010
80	2013	42	13.1143	70.0714	881.7510	5.6337	2.3020
3	2013	42	18.7143	73.9490	951.1816	5.0316	0.9704
52	2013	42	13.7857	70.5408	908.9041	5.6990	2.8878
70	2013	42	14.7714	71.5204	917.6102	5.4755	1.3469
64	2013	42	8.8000	75.4898	782.5367	4.7806	1.9255
48	2013	42	15.2286	69.4592	940.5418	5.7306	0.9061
65	2013	42	13.7857	70.5408	908.9041	5.6990	2.8878
44	2013	42	14.7714	71.5204	917.6102	5.4755	1.3469
75	2013	42	8.8000	75.4898	782.5367	4.7806	1.9255
40	2013	42	14.8571	69.7245	951.7929	5.6561	1.5806
11	2013	42	14.3143	71.7143	884.3122	5.9929	1.8194
35	2013	42	14.6286	73.3776	946.2643	6.0653	1.2143
78	2013	42	15.0714	71.0918	906.2612	5.8082	1.9663
28	2013	42	14.8571	72.0510	935.8929	6.3316	1.4571
39	2013	42	13.7857	70.5408	908.9041	5.6990	2.8878
24	2013	42	15.7143	69.7653	947.2469	5.0153	1.1357
63	2013	42	14.8571	69.7245	951.7929	5.6561	1.5806
62	2013	42	13.0429	75.9082	881.5694	5.5347	1.0500
1	2013	42	13.1143	70.0714	881.7510	5.6337	2.3020
31	2013	43	11.4429	77.5816	854.1388	2.8918	0.8520
79	2013	43	16.4000	76.8265	988.1449	3.2286	0.8959
51	2013	43	13.6143	79.3571	948.5296	3.3316	1.1000

14	2013	43	13.3571	80.2653	905.4092	3.0898	1.2918
67	2013	43	13.2000	80.5612	910.6439	3.3755	2.3204
42	2013	43	12.6429	78.8776	883.0490	3.2531	2.0643
50	2013	43	13.7857	78.7041	910.6786	3.3745	1.5490
43	2013	43	12.6429	78.8776	883.0490	3.2531	2.0643
85	2013	43	14.6429	78.2245	919.2163	2.9704	1.2663
25	2013	43	16.5143	81.1224	986.4980	2.9561	1.0694
69	2013	43	14.6000	72.7857	949.3296	2.7153	1.0857
57	2013	43	12.6571	84.7347	903.0418	3.1541	1.3153
9	2013	43	12.0571	76.9082	861.8663	3.2255	1.9092
72	2013	43	12.8571	79.6020	885.6765	3.6153	1.5561
26	2013	43	13.1857	81.7857	874.8714	3.8010	1.3418
7	2013	43	12.1714	80.4286	867.0255	2.5327	1.2296
83	2013	43	16.7429	79.0204	953.2837	3.0378	0.9031
76	2013	43	13.9857	77.1429	930.2092	3.5541	1.4184
36	2013	43	13.9714	79.6837	937.9378	3.4643	1.3867
81	2013	43	13.6143	79.3571	948.5296	3.3316	1.1000
15	2013	43	15.3429	74.0612	942.5122	3.1622	0.7255
32	2013	43	12.6429	78.8776	883.0490	3.2531	2.0643
73	2013	43	16.3857	74.7347	971.9378	3.2551	0.9255
71	2013	43	13.9714	79.6837	937.9378	3.4643	1.3867
41	2013	43	12.2714	81.2245	882.7765	3.2765	1.0010
10	2013	43	15.2429	79.0102	973.8122	3.4867	1.0082
23	2013	43	8.0286	81.9490	782.5827	3.1184	1.9551
27	2013	43	12.1714	80.4286	867.0255	2.5327	1.2296
60	2013	43	13.6143	79.3571	948.5296	3.3316	1.1000
53	2013	43	12.0571	76.9082	861.8663	3.2255	1.9092
66	2013	43	13.3571	80.2653	905.4092	3.0898	1.2918
59	2013	43	12.6571	84.7347	903.0418	3.1541	1.3153
61	2013	43	16.3857	74.7347	971.9378	3.2551	0.9255
84	2013	43	16.3857	74.7347	971.9378	3.2551	0.9255
38	2013	43	12.6571	84.7347	903.0418	3.1541	1.3153
87	2013	43	13.7429	78.4388	907.8224	3.4388	1.7184
34	2013	43	12.6571	84.7347	903.0418	3.1541	1.3153
29	2013	43	14.6000	72.7857	949.3296	2.7153	1.0857
5	2013	43	10.7143	83.7347	839.6959	3.5061	1.4316
8	2013	43	12.0571	76.9082	861.8663	3.2255	1.9092
12	2013	43	10.7143	83.7347	839.6959	3.5061	1.4316
13	2013	43	16.7429	79.0204	953.2837	3.0378	0.9031
18	2013	43	14.8286	77.7449	973.9704	3.1031	1.0133
33	2013	43	13.7857	78.7041	910.6786	3.3745	1.5490
56	2013	43	16.5143	81.1224	986.4980	2.9561	1.0694
77	2013	43	15.3429	74.0612	942.5122	3.1622	0.7255
54	2013	43	10.7143	83.7347	839.6959	3.5061	1.4316
21	2013	43	13.7857	78.7041	910.6786	3.3745	1.5490
68	2013	43	16.4000	76.8265	988.1449	3.2286	0.8959
74	2013	43	16.3857	74.7347	971.9378	3.2551	0.9255
88	2013	43	12.6429	78.8776	883.0490	3.2531	2.0643
16	2013	43	13.9857	77.1429	930.2092	3.5541	1.4184
30	2013	43	13.3571	80.2653	905.4092	3.0898	1.2918

6	2013	43	16.4000	76.8265	988.1449	3.2286	0.8959
49	2013	43	14.6000	72.7857	949.3296	2.7153	1.0857
22	2013	43	12.6429	78.8776	883.0490	3.2531	2.0643
45	2013	43	10.3857	82.8878	824.6184	3.4214	1.2194
58	2013	43	14.6000	72.7857	949.3296	2.7153	1.0857
37	2013	43	16.4000	76.8265	988.1449	3.2286	0.8959
17	2013	43	13.2000	80.5612	910.6439	3.3755	2.3204
55	2013	43	12.8571	79.6020	885.6765	3.6153	1.5561
46	2013	43	13.9857	77.1429	930.2092	3.5541	1.4184
86	2013	43	12.5000	79.7857	874.0653	2.9571	1.1265
2	2013	43	12.5000	79.7857	874.0653	2.9571	1.1265
4	2013	43	13.7857	78.7041	910.6786	3.3745	1.5490
47	2013	43	17.1714	81.6633	967.7551	2.8398	0.4582
82	2013	43	12.6429	78.8776	883.0490	3.2531	2.0643
19	2013	43	16.3571	75.3265	969.0204	3.5571	0.9796
20	2013	43	12.0571	76.9082	861.8663	3.2255	1.9092
80	2013	43	12.6429	78.8776	883.0490	3.2531	2.0643
3	2013	43	16.7429	79.0204	953.2837	3.0378	0.9031
52	2013	43	13.2000	80.5612	910.6439	3.3755	2.3204
70	2013	43	14.6429	78.2245	919.2163	2.9704	1.2663
64	2013	43	8.0286	81.9490	782.5827	3.1184	1.9551
48	2013	43	15.3429	74.0612	942.5122	3.1622	0.7255
65	2013	43	13.2000	80.5612	910.6439	3.3755	2.3204
44	2013	43	14.6429	78.2245	919.2163	2.9704	1.2663
75	2013	43	8.0286	81.9490	782.5827	3.1184	1.9551
40	2013	43	14.3857	80.0816	954.2367	3.0959	1.6112
11	2013	43	12.8571	79.6020	885.6765	3.6153	1.5561
35	2013	43	13.6143	79.3571	948.5296	3.3316	1.1000
78	2013	43	13.7429	78.4388	907.8224	3.4388	1.7184
28	2013	43	13.9714	79.6837	937.9378	3.4643	1.3867
39	2013	43	13.2000	80.5612	910.6439	3.3755	2.3204
24	2013	43	14.6000	72.7857	949.3296	2.7153	1.0857
63	2013	43	14.3857	80.0816	954.2367	3.0959	1.6112
62	2013	43	12.2714	81.2245	882.7765	3.2765	1.0010
1	2013	43	12.6429	78.8776	883.0490	3.2531	2.0643
31	2013	44	11.2714	82.0408	853.7102	1.4459	0.7082
79	2013	44	15.4857	83.9388	988.9388	1.5541	0.7459
51	2013	44	13.7143	85.7245	949.0204	1.6755	0.8592
14	2013	44	14.3714	84.8878	905.7367	1.9122	1.7643
67	2013	44	13.4429	86.7041	910.8969	1.8929	2.3439
42	2013	44	13.0429	85.3061	883.0020	1.4755	2.0061
50	2013	44	13.4714	82.8061	910.6929	1.3071	1.4551
43	2013	44	13.0429	85.3061	883.0020	1.4755	2.0061
85	2013	44	13.7286	84.4592	919.3306	0.9000	1.2806
25	2013	44	17.3857	86.6224	987.3571	1.7704	0.7449
69	2013	44	14.9143	78.0918	949.7204	1.3857	0.8306
57	2013	44	13.3429	89.0510	903.0296	1.1643	1.2265
9	2013	44	13.1429	83.1531	861.7082	2.1612	2.1173
72	2013	44	14.8714	83.7755	885.7316	1.9255	1.5888
26	2013	44	15.8714	86.8571	874.8765	2.5867	1.4908

7	2013	44	15.4857	84.3469	866.8918	1.7592	1.4459
83	2013	44	19.3714	83.5612	953.8245	2.1061	0.7010
76	2013	44	13.4571	82.6224	930.4898	1.7000	1.3337
36	2013	44	14.2571	85.8776	938.3265	1.7408	1.1102
81	2013	44	13.7143	85.7245	949.0204	1.6755	0.8592
15	2013	44	14.4000	78.1122	942.8378	1.5571	0.5010
32	2013	44	13.0429	85.3061	883.0020	1.4755	2.0061
73	2013	44	15.6571	81.6837	972.5878	1.5306	0.7633
71	2013	44	14.2571	85.8776	938.3265	1.7408	1.1102
41	2013	44	13.0714	85.9388	882.6010	1.3776	0.9684
10	2013	44	15.0286	85.5000	974.5133	1.8551	0.8214
23	2013	44	9.9857	85.5000	781.7480	2.5224	1.9194
27	2013	44	15.4857	84.3469	866.8918	1.7592	1.4459
60	2013	44	13.7143	85.7245	949.0204	1.6755	0.8592
53	2013	44	13.1429	83.1531	861.7082	2.1612	2.1173
66	2013	44	14.3714	84.8878	905.7367	1.9122	1.7643
59	2013	44	13.3429	89.0510	903.0296	1.1643	1.2265
61	2013	44	15.6571	81.6837	972.5878	1.5306	0.7633
84	2013	44	15.6571	81.6837	972.5878	1.5306	0.7633
38	2013	44	13.3429	89.0510	903.0296	1.1643	1.2265
87	2013	44	15.6286	83.9388	907.9898	1.8061	1.5663
34	2013	44	13.3429	89.0510	903.0296	1.1643	1.2265
29	2013	44	14.9143	78.0918	949.7204	1.3857	0.8306
5	2013	44	13.5143	87.8367	839.3163	2.6418	1.3163
8	2013	44	13.1429	83.1531	861.7082	2.1612	2.1173
12	2013	44	13.5143	87.8367	839.3163	2.6418	1.3163
13	2013	44	19.3714	83.5612	953.8245	2.1061	0.7010
18	2013	44	14.6286	83.3061	974.6357	1.6214	0.7367
33	2013	44	13.4714	82.8061	910.6929	1.3071	1.4551
56	2013	44	17.3857	86.6224	987.3571	1.7704	0.7449
77	2013	44	14.4000	78.1122	942.8378	1.5571	0.5010
54	2013	44	13.5143	87.8367	839.3163	2.6418	1.3163
21	2013	44	13.4714	82.8061	910.6929	1.3071	1.4551
68	2013	44	15.4857	83.9388	988.9388	1.5541	0.7459
74	2013	44	15.6571	81.6837	972.5878	1.5306	0.7633
88	2013	44	13.0429	85.3061	883.0020	1.4755	2.0061
16	2013	44	13.4571	82.6224	930.4898	1.7000	1.3337
30	2013	44	14.3714	84.8878	905.7367	1.9122	1.7643
6	2013	44	15.4857	83.9388	988.9388	1.5541	0.7459
49	2013	44	14.9143	78.0918	949.7204	1.3857	0.8306
22	2013	44	13.0429	85.3061	883.0020	1.4755	2.0061
45	2013	44	13.8714	84.3571	824.1429	2.8306	1.4429
58	2013	44	14.9143	78.0918	949.7204	1.3857	0.8306
37	2013	44	15.4857	83.9388	988.9388	1.5541	0.7459
17	2013	44	13.4429	86.7041	910.8969	1.8929	2.3439
55	2013	44	14.8714	83.7755	885.7316	1.9255	1.5888
46	2013	44	13.4571	82.6224	930.4898	1.7000	1.3337
86	2013	44	13.8000	83.1735	873.8531	1.6378	1.2112
2	2013	44	13.8000	83.1735	873.8531	1.6378	1.2112
4	2013	44	13.4714	82.8061	910.6929	1.3071	1.4551

47	2013	44	18.8000	85.0510	968.4071	1.4929	0.4633
82	2013	44	13.0429	85.3061	883.0020	1.4755	2.0061
19	2013	44	17.8857	80.1633	969.8673	1.9276	0.8286
20	2013	44	13.1429	83.1531	861.7082	2.1612	2.1173
80	2013	44	13.0429	85.3061	883.0020	1.4755	2.0061
3	2013	44	19.3714	83.5612	953.8245	2.1061	0.7010
52	2013	44	13.4429	86.7041	910.8969	1.8929	2.3439
70	2013	44	13.7286	84.4592	919.3306	0.9000	1.2806
64	2013	44	9.9857	85.5000	781.7480	2.5224	1.9194
48	2013	44	14.4000	78.1122	942.8378	1.5571	0.5010
65	2013	44	13.4429	86.7041	910.8969	1.8929	2.3439
44	2013	44	13.7286	84.4592	919.3306	0.9000	1.2806
75	2013	44	9.9857	85.5000	781.7480	2.5224	1.9194
40	2013	44	14.7143	85.2653	954.8837	2.0929	1.3918
11	2013	44	14.8714	83.7755	885.7316	1.9255	1.5888
35	2013	44	13.7143	85.7245	949.0204	1.6755	0.8592
78	2013	44	15.6286	83.9388	907.9898	1.8061	1.5663
28	2013	44	14.2571	85.8776	938.3265	1.7408	1.1102
39	2013	44	13.4429	86.7041	910.8969	1.8929	2.3439
24	2013	44	14.9143	78.0918	949.7204	1.3857	0.8306
63	2013	44	14.7143	85.2653	954.8837	2.0929	1.3918
62	2013	44	13.0714	85.9388	882.6010	1.3776	0.9684
1	2013	44	13.0429	85.3061	883.0020	1.4755	2.0061
31	2013	45	13.5857	84.2347	852.6765	1.8684	0.8408
79	2013	45	18.8000	84.9694	988.1378	1.7184	0.6755
51	2013	45	16.6000	84.7959	948.2378	2.0041	1.0806
14	2013	45	16.6286	84.1531	905.1520	2.4806	2.0082
67	2013	45	17.3571	85.0714	910.1633	1.8173	2.5959
42	2013	45	16.0429	84.3673	882.1776	1.7031	2.3102
50	2013	45	16.0429	81.1327	909.5827	1.3357	1.5776
43	2013	45	16.0429	84.3673	882.1776	1.7031	2.3102
85	2013	45	16.0714	83.2347	918.2908	1.1867	1.4663
25	2013	45	19.3143	84.5612	986.4459	2.0092	0.8224
69	2013	45	18.0000	77.1020	948.7531	1.6255	0.9296
57	2013	45	16.7429	86.3571	902.0010	1.2520	1.6173
9	2013	45	15.3143	83.5816	860.9194	2.7092	2.5786
72	2013	45	16.9857	82.3776	884.9306	2.7816	1.8184
26	2013	45	17.5000	82.3878	873.9714	4.8765	1.8837
7	2013	45	16.8429	80.5714	865.9694	3.7459	1.9622
83	2013	45	20.2571	78.7551	952.7071	2.8092	0.6888
76	2013	45	16.9143	83.4286	929.5694	1.6061	1.3622
36	2013	45	17.9714	83.4796	937.4704	2.2143	1.2122
81	2013	45	16.6000	84.7959	948.2378	2.0041	1.0806
15	2013	45	17.0571	78.7857	941.8908	1.3122	0.5378
32	2013	45	16.0429	84.3673	882.1776	1.7031	2.3102
73	2013	45	18.8571	82.6429	971.5898	1.5847	0.6878
71	2013	45	17.9714	83.4796	937.4704	2.2143	1.2122
41	2013	45	15.6714	85.0306	881.5908	1.8786	1.2235
10	2013	45	17.3286	86.5918	973.7592	2.0520	0.7929
23	2013	45	12.1714	81.7449	780.9204	4.8459	2.1643

27	2013	45	16.8429	80.5714	865.9694	3.7459	1.9622
60	2013	45	16.6000	84.7959	948.2378	2.0041	1.0806
53	2013	45	15.3143	83.5816	860.9194	2.7092	2.5786
66	2013	45	16.6286	84.1531	905.1520	2.4806	2.0082
59	2013	45	16.7429	86.3571	902.0010	1.2520	1.6173
61	2013	45	18.8571	82.6429	971.5898	1.5847	0.6878
84	2013	45	18.8571	82.6429	971.5898	1.5847	0.6878
38	2013	45	16.7429	86.3571	902.0010	1.2520	1.6173
87	2013	45	17.8571	81.5510	907.1306	2.4469	1.5714
34	2013	45	16.7429	86.3571	902.0010	1.2520	1.6173
29	2013	45	18.0000	77.1020	948.7531	1.6255	0.9296
5	2013	45	15.3000	84.0204	838.4367	4.7847	1.7561
8	2013	45	15.3143	83.5816	860.9194	2.7092	2.5786
12	2013	45	15.3000	84.0204	838.4367	4.7847	1.7561
13	2013	45	20.2571	78.7551	952.7071	2.8092	0.6888
18	2013	45	17.6143	83.6939	973.8633	1.6010	0.6143
33	2013	45	16.0429	81.1327	909.5827	1.3357	1.5776
56	2013	45	19.3143	84.5612	986.4459	2.0092	0.8224
77	2013	45	17.0571	78.7857	941.8908	1.3122	0.5378
54	2013	45	15.3000	84.0204	838.4367	4.7847	1.7561
21	2013	45	16.0429	81.1327	909.5827	1.3357	1.5776
68	2013	45	18.8000	84.9694	988.1378	1.7184	0.6755
74	2013	45	18.8571	82.6429	971.5898	1.5847	0.6878
88	2013	45	16.0429	84.3673	882.1776	1.7031	2.3102
16	2013	45	16.9143	83.4286	929.5694	1.6061	1.3622
30	2013	45	16.6286	84.1531	905.1520	2.4806	2.0082
6	2013	45	18.8000	84.9694	988.1378	1.7184	0.6755
49	2013	45	18.0000	77.1020	948.7531	1.6255	0.9296
22	2013	45	16.0429	84.3673	882.1776	1.7031	2.3102
45	2013	45	15.3429	77.0918	823.2857	5.6786	2.0469
58	2013	45	18.0000	77.1020	948.7531	1.6255	0.9296
37	2013	45	18.8000	84.9694	988.1378	1.7184	0.6755
17	2013	45	17.3571	85.0714	910.1633	1.8173	2.5959
55	2013	45	16.9857	82.3776	884.9306	2.7816	1.8184
46	2013	45	16.9143	83.4286	929.5694	1.6061	1.3622
86	2013	45	16.2857	82.6122	872.9010	2.1531	1.3653
2	2013	45	16.2857	82.6122	872.9010	2.1531	1.3653
4	2013	45	16.0429	81.1327	909.5827	1.3357	1.5776
47	2013	45	19.7714	81.9082	967.4071	2.4378	0.5327
82	2013	45	16.0429	84.3673	882.1776	1.7031	2.3102
19	2013	45	19.4714	78.3571	969.0327	2.7418	0.9429
20	2013	45	15.3143	83.5816	860.9194	2.7092	2.5786
80	2013	45	16.0429	84.3673	882.1776	1.7031	2.3102
3	2013	45	20.2571	78.7551	952.7071	2.8092	0.6888
52	2013	45	17.3571	85.0714	910.1633	1.8173	2.5959
70	2013	45	16.0714	83.2347	918.2908	1.1867	1.4663
64	2013	45	12.1714	81.7449	780.9204	4.8459	2.1643
48	2013	45	17.0571	78.7857	941.8908	1.3122	0.5378
65	2013	45	17.3571	85.0714	910.1633	1.8173	2.5959
44	2013	45	16.0714	83.2347	918.2908	1.1867	1.4663

75	2013	45	12.1714	81.7449	780.9204	4.8459	2.1643
40	2013	45	17.6714	83.6020	954.2276	2.7653	1.4582
11	2013	45	16.9857	82.3776	884.9306	2.7816	1.8184
35	2013	45	16.6000	84.7959	948.2378	2.0041	1.0806
78	2013	45	17.8571	81.5510	907.1306	2.4469	1.5714
28	2013	45	17.9714	83.4796	937.4704	2.2143	1.2122
39	2013	45	17.3571	85.0714	910.1633	1.8173	2.5959
24	2013	45	18.0000	77.1020	948.7531	1.6255	0.9296
63	2013	45	17.6714	83.6020	954.2276	2.7653	1.4582
62	2013	45	15.6714	85.0306	881.5908	1.8786	1.2235
1	2013	45	16.0429	84.3673	882.1776	1.7031	2.3102
31	2013	46	9.1143	84.0612	852.5153	2.1827	1.0184
79	2013	46	13.1000	82.5408	988.1878	1.8673	0.7378
51	2013	46	10.5571	82.8163	948.1490	2.3347	1.4622
14	2013	46	11.3143	84.2245	904.7929	2.7367	2.1112
67	2013	46	10.8286	83.6531	909.9204	1.6133	3.1367
42	2013	46	10.2714	84.6429	881.9153	2.0235	2.6378
50	2013	46	11.7429	80.8776	909.3031	1.4602	1.7827
43	2013	46	10.2714	84.6429	881.9153	2.0235	2.6378
85	2013	46	11.6714	84.1224	918.2214	1.5551	1.3837
25	2013	46	13.7714	83.1837	986.2755	1.8684	1.0041
69	2013	46	12.3429	74.1020	948.5776	2.0898	1.3214
57	2013	46	10.8571	83.3163	901.7092	1.5449	1.9908
9	2013	46	10.2000	85.1224	860.6143	2.8531	2.7041
72	2013	46	12.0857	81.5204	884.5786	3.1122	2.2602
26	2013	46	12.5429	82.9082	873.5673	4.8000	1.9429
7	2013	46	11.7000	82.0102	865.6551	4.0694	2.0296
83	2013	46	16.7429	76.5000	952.2184	2.4653	0.8459
76	2013	46	11.3286	82.8571	929.3776	1.2857	1.4980
36	2013	46	11.2143	80.4592	937.3276	2.6643	1.6194
81	2013	46	10.5571	82.8163	948.1490	2.3347	1.4622
15	2013	46	12.5714	78.0816	941.6980	1.0051	0.7673
32	2013	46	10.2714	84.6429	881.9153	2.0235	2.6378
73	2013	46	13.5000	81.4592	971.4235	1.5735	0.8061
71	2013	46	11.2143	80.4592	937.3276	2.6643	1.6194
41	2013	46	10.6429	84.0000	881.3694	2.6510	1.5367
10	2013	46	11.5143	85.8367	973.7367	2.1561	1.0112
23	2013	46	7.8571	81.0612	780.7184	5.5133	2.2561
27	2013	46	11.7000	82.0102	865.6551	4.0694	2.0296
60	2013	46	10.5571	82.8163	948.1490	2.3347	1.4622
53	2013	46	10.2000	85.1224	860.6143	2.8531	2.7041
66	2013	46	11.3143	84.2245	904.7929	2.7367	2.1112
59	2013	46	10.8571	83.3163	901.7092	1.5449	1.9908
61	2013	46	13.5000	81.4592	971.4235	1.5735	0.8061
84	2013	46	13.5000	81.4592	971.4235	1.5735	0.8061
38	2013	46	10.8571	83.3163	901.7092	1.5449	1.9908
87	2013	46	12.9429	79.1224	906.7806	2.8929	2.0582
34	2013	46	10.8571	83.3163	901.7092	1.5449	1.9908
29	2013	46	12.3429	74.1020	948.5776	2.0898	1.3214
5	2013	46	9.6714	85.2551	838.1551	4.5796	1.9908

8	2013	46	10.2000	85.1224	860.6143	2.8531	2.7041
12	2013	46	9.6714	85.2551	838.1551	4.5796	1.9908
13	2013	46	16.7429	76.5000	952.2184	2.4653	0.8459
18	2013	46	11.8857	82.6939	973.8816	1.6602	0.8316
33	2013	46	11.7429	80.8776	909.3031	1.4602	1.7827
56	2013	46	13.7714	83.1837	986.2755	1.8684	1.0041
77	2013	46	12.5714	78.0816	941.6980	1.0051	0.7673
54	2013	46	9.6714	85.2551	838.1551	4.5796	1.9908
21	2013	46	11.7429	80.8776	909.3031	1.4602	1.7827
68	2013	46	13.1000	82.5408	988.1878	1.8673	0.7378
74	2013	46	13.5000	81.4592	971.4235	1.5735	0.8061
88	2013	46	10.2714	84.6429	881.9153	2.0235	2.6378
16	2013	46	11.3286	82.8571	929.3776	1.2857	1.4980
30	2013	46	11.3143	84.2245	904.7929	2.7367	2.1112
6	2013	46	13.1000	82.5408	988.1878	1.8673	0.7378
49	2013	46	12.3429	74.1020	948.5776	2.0898	1.3214
22	2013	46	10.2714	84.6429	881.9153	2.0235	2.6378
45	2013	46	10.3143	78.7857	823.0041	5.3929	2.0898
58	2013	46	12.3429	74.1020	948.5776	2.0898	1.3214
37	2013	46	13.1000	82.5408	988.1878	1.8673	0.7378
17	2013	46	10.8286	83.6531	909.9204	1.6133	3.1367
55	2013	46	12.0857	81.5204	884.5786	3.1122	2.2602
46	2013	46	11.3286	82.8571	929.3776	1.2857	1.4980
86	2013	46	11.1571	83.1122	872.6398	2.4755	1.4306
2	2013	46	11.1571	83.1122	872.6398	2.4755	1.4306
4	2013	46	11.7429	80.8776	909.3031	1.4602	1.7827
47	2013	46	16.4429	79.5408	966.9633	2.7378	0.6786
82	2013	46	10.2714	84.6429	881.9153	2.0235	2.6378
19	2013	46	14.4714	76.2245	968.6592	2.6602	1.2286
20	2013	46	10.2000	85.1224	860.6143	2.8531	2.7041
80	2013	46	10.2714	84.6429	881.9153	2.0235	2.6378
3	2013	46	16.7429	76.5000	952.2184	2.4653	0.8459
52	2013	46	10.8286	83.6531	909.9204	1.6133	3.1367
70	2013	46	11.6714	84.1224	918.2214	1.5551	1.3837
64	2013	46	7.8571	81.0612	780.7184	5.5133	2.2561
48	2013	46	12.5714	78.0816	941.6980	1.0051	0.7673
65	2013	46	10.8286	83.6531	909.9204	1.6133	3.1367
44	2013	46	11.6714	84.1224	918.2214	1.5551	1.3837
75	2013	46	7.8571	81.0612	780.7184	5.5133	2.2561
40	2013	46	10.8286	83.2653	954.0929	3.0051	2.0000
11	2013	46	12.0857	81.5204	884.5786	3.1122	2.2602
35	2013	46	10.5571	82.8163	948.1490	2.3347	1.4622
78	2013	46	12.9429	79.1224	906.7806	2.8929	2.0582
28	2013	46	11.2143	80.4592	937.3276	2.6643	1.6194
39	2013	46	10.8286	83.6531	909.9204	1.6133	3.1367
24	2013	46	12.3429	74.1020	948.5776	2.0898	1.3214
63	2013	46	10.8286	83.2653	954.0929	3.0051	2.0000
62	2013	46	10.6429	84.0000	881.3694	2.6510	1.5367
1	2013	46	10.2714	84.6429	881.9153	2.0235	2.6378
31	2013	47	10.5429	84.3878	852.5857	1.3500	0.9602

79	2013	47	14.2571	80.0918	988.5439	2.5765	0.8061
51	2013	47	12.7429	81.6429	948.3582	2.5571	1.2388
14	2013	47	12.2286	83.0306	904.9357	1.8704	1.8704
67	2013	47	12.5143	82.7449	910.0582	0.9816	2.7500
42	2013	47	11.6143	84.4388	882.0071	1.5459	2.3020
50	2013	47	12.5857	80.9796	909.5561	1.4000	1.8082
43	2013	47	11.6143	84.4388	882.0071	1.5459	2.3020
85	2013	47	13.0000	85.2449	918.5398	1.0694	1.2633
25	2013	47	15.7571	83.8980	986.8786	1.6204	0.8796
69	2013	47	14.2000	73.2449	948.9071	2.4082	1.3173
57	2013	47	12.6286	83.4490	901.8837	1.2316	1.6561
9	2013	47	10.6714	86.2245	860.6510	1.8816	2.2684
72	2013	47	11.9143	82.9694	884.7204	1.8133	1.9480
26	2013	47	12.3857	89.4184	873.7520	2.2388	1.6918
7	2013	47	12.0286	87.2449	865.8184	2.2561	1.7663
83	2013	47	16.8286	77.6633	952.8235	1.5571	0.8490
76	2013	47	12.7571	81.8673	929.6122	1.1929	1.4510
36	2013	47	13.7000	80.3061	937.5908	2.5286	1.3714
81	2013	47	12.7429	81.6429	948.3582	2.5571	1.2388
15	2013	47	13.3000	79.1735	942.0071	0.9602	0.7786
32	2013	47	11.6143	84.4388	882.0071	1.5459	2.3020
73	2013	47	14.9571	80.8469	971.7969	1.8612	0.8316
71	2013	47	13.7000	80.3061	937.5908	2.5286	1.3714
41	2013	47	12.0571	84.3163	881.5622	1.8755	1.4755
10	2013	47	13.7000	82.7449	974.0612	2.5500	1.0929
23	2013	47	7.2857	85.2551	780.4480	3.7786	2.1245
27	2013	47	12.0286	87.2449	865.8184	2.2561	1.7663
60	2013	47	12.7429	81.6429	948.3582	2.5571	1.2388
53	2013	47	10.6714	86.2245	860.6510	1.8816	2.2684
66	2013	47	12.2286	83.0306	904.9357	1.8704	1.8704
59	2013	47	12.6286	83.4490	901.8837	1.2316	1.6561
61	2013	47	14.9571	80.8469	971.7969	1.8612	0.8316
84	2013	47	14.9571	80.8469	971.7969	1.8612	0.8316
38	2013	47	12.6286	83.4490	901.8837	1.2316	1.6561
87	2013	47	13.2429	80.0714	907.0276	1.7918	1.9490
34	2013	47	12.6286	83.4490	901.8837	1.2316	1.6561
29	2013	47	14.2000	73.2449	948.9071	2.4082	1.3173
5	2013	47	10.2000	90.6735	838.1561	2.3969	1.6724
8	2013	47	10.6714	86.2245	860.6510	1.8816	2.2684
12	2013	47	10.2000	90.6735	838.1561	2.3969	1.6724
13	2013	47	16.8286	77.6633	952.8235	1.5571	0.8490
18	2013	47	12.7571	82.0612	974.1480	2.4561	0.8990
33	2013	47	12.5857	80.9796	909.5561	1.4000	1.8082
56	2013	47	15.7571	83.8980	986.8786	1.6204	0.8796
77	2013	47	13.3000	79.1735	942.0071	0.9602	0.7786
54	2013	47	10.2000	90.6735	838.1561	2.3969	1.6724
21	2013	47	12.5857	80.9796	909.5561	1.4000	1.8082
68	2013	47	14.2571	80.0918	988.5439	2.5765	0.8061
74	2013	47	14.9571	80.8469	971.7969	1.8612	0.8316
88	2013	47	11.6143	84.4388	882.0071	1.5459	2.3020

16	2013	47	12.7571	81.8673	929.6122	1.1929	1.4510
30	2013	47	12.2286	83.0306	904.9357	1.8704	1.8704
6	2013	47	14.2571	80.0918	988.5439	2.5765	0.8061
49	2013	47	14.2000	73.2449	948.9071	2.4082	1.3173
22	2013	47	11.6143	84.4388	882.0071	1.5459	2.3020
45	2013	47	10.9143	85.8061	822.9163	2.8265	1.7918
58	2013	47	14.2000	73.2449	948.9071	2.4082	1.3173
37	2013	47	14.2571	80.0918	988.5439	2.5765	0.8061
17	2013	47	12.5143	82.7449	910.0582	0.9816	2.7500
55	2013	47	11.9143	82.9694	884.7204	1.8133	1.9480
46	2013	47	12.7571	81.8673	929.6122	1.1929	1.4510
86	2013	47	11.9714	84.0000	872.7929	1.6612	1.3612
2	2013	47	11.9714	84.0000	872.7929	1.6612	1.3612
4	2013	47	12.5857	80.9796	909.5561	1.4000	1.8082
47	2013	47	16.4000	80.3163	967.5592	1.4714	0.6255
82	2013	47	11.6143	84.4388	882.0071	1.5459	2.3020
19	2013	47	15.6714	77.3673	969.2541	1.9347	1.0020
20	2013	47	10.6714	86.2245	860.6510	1.8816	2.2684
80	2013	47	11.6143	84.4388	882.0071	1.5459	2.3020
3	2013	47	16.8286	77.6633	952.8235	1.5571	0.8490
52	2013	47	12.5143	82.7449	910.0582	0.9816	2.7500
70	2013	47	13.0000	85.2449	918.5398	1.0694	1.2633
64	2013	47	7.2857	85.2551	780.4480	3.7786	2.1245
48	2013	47	13.3000	79.1735	942.0071	0.9602	0.7786
65	2013	47	12.5143	82.7449	910.0582	0.9816	2.7500
44	2013	47	13.0000	85.2449	918.5398	1.0694	1.2633
75	2013	47	7.2857	85.2551	780.4480	3.7786	2.1245
40	2013	47	13.8571	81.4694	954.3520	2.8469	1.7316
11	2013	47	11.9143	82.9694	884.7204	1.8133	1.9480
35	2013	47	12.7429	81.6429	948.3582	2.5571	1.2388
78	2013	47	13.2429	80.0714	907.0276	1.7918	1.9490
28	2013	47	13.7000	80.3061	937.5908	2.5286	1.3714
39	2013	47	12.5143	82.7449	910.0582	0.9816	2.7500
24	2013	47	14.2000	73.2449	948.9071	2.4082	1.3173
63	2013	47	13.8571	81.4694	954.3520	2.8469	1.7316
62	2013	47	12.0571	84.3163	881.5622	1.8755	1.4755
1	2013	47	11.6143	84.4388	882.0071	1.5459	2.3020
31	2013	48	6.7571	80.8571	852.6316	2.1429	1.0276
79	2013	48	11.2571	80.4184	988.9663	2.3265	0.7663
51	2013	48	8.9571	81.3673	948.6520	2.4643	1.0153
14	2013	48	9.2429	79.8776	905.2602	1.7296	1.8133
67	2013	48	9.3000	79.1531	910.2602	0.9969	2.4878
42	2013	48	8.6429	79.0816	882.2061	1.7724	2.0959
50	2013	48	8.6571	77.0918	910.0143	2.1918	1.7408
43	2013	48	8.6429	79.0816	882.2061	1.7724	2.0959
85	2013	48	9.2857	82.2959	918.9867	1.7847	1.5092
25	2013	48	12.4429	82.5918	987.4796	1.5908	0.7643
69	2013	48	10.2143	72.1327	949.3541	2.3357	1.0694
57	2013	48	8.7857	80.5918	902.2020	2.0092	1.5122
9	2013	48	7.7286	81.5102	860.7500	2.0980	2.2255

72	2013	48	9.4429	79.4286	884.9867	1.8867	1.8347
26	2013	48	10.1286	84.9082	873.9143	2.0694	1.7153
7	2013	48	9.1429	82.5204	865.9122	2.6592	1.8929
83	2013	48	14.5000	72.2449	953.5643	2.0000	0.8224
76	2013	48	8.7429	79.5918	930.0316	1.8908	1.2898
36	2013	48	10.1143	77.6020	937.8612	2.2551	1.0694
81	2013	48	8.9571	81.3673	948.6520	2.4643	1.0153
15	2013	48	8.7857	78.2041	942.6878	1.2867	0.7459
32	2013	48	8.6429	79.0816	882.2061	1.7724	2.0959
73	2013	48	11.0000	79.7959	972.4112	1.8286	0.7939
71	2013	48	10.1143	77.6020	937.8612	2.2551	1.0694
41	2013	48	8.6429	79.2347	881.7653	2.4673	1.5724
10	2013	48	9.8714	81.1327	974.4878	2.2949	1.0398
23	2013	48	3.5571	84.1020	780.0143	3.6265	2.3561
27	2013	48	9.1429	82.5204	865.9122	2.6592	1.8929
60	2013	48	8.9571	81.3673	948.6520	2.4643	1.0153
53	2013	48	7.7286	81.5102	860.7500	2.0980	2.2255
66	2013	48	9.2429	79.8776	905.2602	1.7296	1.8133
59	2013	48	8.7857	80.5918	902.2020	2.0092	1.5122
61	2013	48	11.0000	79.7959	972.4112	1.8286	0.7939
84	2013	48	11.0000	79.7959	972.4112	1.8286	0.7939
38	2013	48	8.7857	80.5918	902.2020	2.0092	1.5122
87	2013	48	10.0286	78.2857	907.4214	1.2847	1.6469
34	2013	48	8.7857	80.5918	902.2020	2.0092	1.5122
29	2013	48	10.2143	72.1327	949.3541	2.3357	1.0694
5	2013	48	6.7857	87.7653	838.0612	2.8571	1.6602
8	2013	48	7.7286	81.5102	860.7500	2.0980	2.2255
12	2013	48	6.7857	87.7653	838.0612	2.8571	1.6602
13	2013	48	14.5000	72.2449	953.5643	2.0000	0.8224
18	2013	48	9.5429	81.6837	974.5102	2.4816	0.7755
33	2013	48	8.6571	77.0918	910.0143	2.1918	1.7408
56	2013	48	12.4429	82.5918	987.4796	1.5908	0.7643
77	2013	48	8.7857	78.2041	942.6878	1.2867	0.7459
54	2013	48	6.7857	87.7653	838.0612	2.8571	1.6602
21	2013	48	8.6571	77.0918	910.0143	2.1918	1.7408
68	2013	48	11.2571	80.4184	988.9663	2.3265	0.7663
74	2013	48	11.0000	79.7959	972.4112	1.8286	0.7939
88	2013	48	8.6429	79.0816	882.2061	1.7724	2.0959
16	2013	48	8.7429	79.5918	930.0316	1.8908	1.2898
30	2013	48	9.2429	79.8776	905.2602	1.7296	1.8133
6	2013	48	11.2571	80.4184	988.9663	2.3265	0.7663
49	2013	48	10.2143	72.1327	949.3541	2.3357	1.0694
22	2013	48	8.6429	79.0816	882.2061	1.7724	2.0959
45	2013	48	7.2571	81.0816	822.6602	3.5510	1.9653
58	2013	48	10.2143	72.1327	949.3541	2.3357	1.0694
37	2013	48	11.2571	80.4184	988.9663	2.3265	0.7663
17	2013	48	9.3000	79.1531	910.2602	0.9969	2.4878
55	2013	48	9.4429	79.4286	884.9867	1.8867	1.8347
46	2013	48	8.7429	79.5918	930.0316	1.8908	1.2898
86	2013	48	8.7429	80.0918	872.9673	2.1204	1.3480

2	2013	48	8.7429	80.0918	872.9673	2.1204	1.3480
4	2013	48	8.6571	77.0918	910.0143	2.1918	1.7408
47	2013	48	13.9857	76.0102	968.3918	1.3418	0.5816
82	2013	48	8.6429	79.0816	882.2061	1.7724	2.0959
19	2013	48	13.2000	75.0408	969.9806	2.2316	0.7633
20	2013	48	7.7286	81.5102	860.7500	2.0980	2.2255
80	2013	48	8.6429	79.0816	882.2061	1.7724	2.0959
3	2013	48	14.5000	72.2449	953.5643	2.0000	0.8224
52	2013	48	9.3000	79.1531	910.2602	0.9969	2.4878
70	2013	48	9.2857	82.2959	918.9867	1.7847	1.5092
64	2013	48	3.5571	84.1020	780.0143	3.6265	2.3561
48	2013	48	8.7857	78.2041	942.6878	1.2867	0.7459
65	2013	48	9.3000	79.1531	910.2602	0.9969	2.4878
44	2013	48	9.2857	82.2959	918.9867	1.7847	1.5092
75	2013	48	3.5571	84.1020	780.0143	3.6265	2.3561
40	2013	48	9.8000	78.6735	954.7173	2.7837	1.3071
11	2013	48	9.4429	79.4286	884.9867	1.8867	1.8347
35	2013	48	8.9571	81.3673	948.6520	2.4643	1.0153
78	2013	48	10.0286	78.2857	907.4214	1.2847	1.6469
28	2013	48	10.1143	77.6020	937.8612	2.2551	1.0694
39	2013	48	9.3000	79.1531	910.2602	0.9969	2.4878
24	2013	48	10.2143	72.1327	949.3541	2.3357	1.0694
63	2013	48	9.8000	78.6735	954.7173	2.7837	1.3071
62	2013	48	8.6429	79.2347	881.7653	2.4673	1.5724
1	2013	48	8.6429	79.0816	882.2061	1.7724	2.0959
31	2013	49	7.8571	75.8776	852.6694	3.4041	1.0490
79	2013	49	11.2857	79.7551	989.2714	2.8490	0.7602
51	2013	49	9.8571	80.1122	948.8347	3.4980	0.9796
14	2013	49	11.2857	76.3265	905.3857	3.1173	1.7122
67	2013	49	11.6286	72.2449	910.4020	2.3939	2.8082
42	2013	49	10.3857	73.4490	882.3918	2.9041	2.1612
50	2013	49	10.2286	74.6020	910.1990	2.6745	1.6347
43	2013	49	10.3857	73.4490	882.3918	2.9041	2.1612
85	2013	49	11.4000	79.6122	919.0694	2.8459	1.6071
25	2013	49	13.1714	79.9898	987.7398	2.9153	0.8847
69	2013	49	12.1143	69.7857	949.5684	3.1776	0.9980
57	2013	49	11.2000	77.0408	902.3531	3.1684	1.6673
9	2013	49	9.5857	75.1327	860.8816	3.0724	2.2061
72	2013	49	10.4714	72.6327	885.2224	3.1990	1.9337
26	2013	49	11.7714	76.2653	874.2306	3.3673	1.7153
7	2013	49	10.8714	75.6735	866.0622	3.2949	1.7245
83	2013	49	14.6429	67.9184	953.9071	3.4827	0.7235
76	2013	49	11.0286	75.8367	929.8571	3.4694	1.1684
36	2013	49	11.6571	74.1327	938.0153	3.3357	1.1184
81	2013	49	9.8571	80.1122	948.8347	3.4980	0.9796
15	2013	49	9.9143	76.5408	943.0010	2.0755	0.7010
32	2013	49	10.3857	73.4490	882.3918	2.9041	2.1612
73	2013	49	12.2714	78.1224	972.7214	2.6776	0.7949
71	2013	49	11.6571	74.1327	938.0153	3.3357	1.1184
41	2013	49	9.4857	76.0408	881.8898	3.4408	1.4755

10	2013	49	10.2286	77.8061	974.7031	2.9398	1.0439
23	2013	49	6.5571	79.4694	779.9673	4.6276	2.2990
27	2013	49	10.8714	75.6735	866.0622	3.2949	1.7245
60	2013	49	9.8571	80.1122	948.8347	3.4980	0.9796
53	2013	49	9.5857	75.1327	860.8816	3.0724	2.2061
66	2013	49	11.2857	76.3265	905.3857	3.1173	1.7122
59	2013	49	11.2000	77.0408	902.3531	3.1684	1.6673
61	2013	49	12.2714	78.1224	972.7214	2.6776	0.7949
84	2013	49	12.2714	78.1224	972.7214	2.6776	0.7949
38	2013	49	11.2000	77.0408	902.3531	3.1684	1.6673
87	2013	49	11.0000	74.1939	907.6888	2.3102	1.7143
34	2013	49	11.2000	77.0408	902.3531	3.1684	1.6673
29	2013	49	12.1143	69.7857	949.5684	3.1776	0.9980
5	2013	49	9.6000	80.8265	838.2286	3.6684	1.5939
8	2013	49	9.5857	75.1327	860.8816	3.0724	2.2061
12	2013	49	9.6000	80.8265	838.2286	3.6684	1.5939
13	2013	49	14.6429	67.9184	953.9071	3.4827	0.7235
18	2013	49	9.6571	78.5000	974.7480	3.4469	0.8031
33	2013	49	10.2286	74.6020	910.1990	2.6745	1.6347
56	2013	49	13.1714	79.9898	987.7398	2.9153	0.8847
77	2013	49	9.9143	76.5408	943.0010	2.0755	0.7010
54	2013	49	9.6000	80.8265	838.2286	3.6684	1.5939
21	2013	49	10.2286	74.6020	910.1990	2.6745	1.6347
68	2013	49	11.2857	79.7551	989.2714	2.8490	0.7602
74	2013	49	12.2714	78.1224	972.7214	2.6776	0.7949
88	2013	49	10.3857	73.4490	882.3918	2.9041	2.1612
16	2013	49	11.0286	75.8367	929.8571	3.4694	1.1684
30	2013	49	11.2857	76.3265	905.3857	3.1173	1.7122
6	2013	49	11.2857	79.7551	989.2714	2.8490	0.7602
49	2013	49	12.1143	69.7857	949.5684	3.1776	0.9980
22	2013	49	10.3857	73.4490	882.3918	2.9041	2.1612
45	2013	49	9.9714	77.8980	822.8908	4.4214	1.9286
58	2013	49	12.1143	69.7857	949.5684	3.1776	0.9980
37	2013	49	11.2857	79.7551	989.2714	2.8490	0.7602
17	2013	49	11.6286	72.2449	910.4020	2.3939	2.8082
55	2013	49	10.4714	72.6327	885.2224	3.1990	1.9337
46	2013	49	11.0286	75.8367	929.8571	3.4694	1.1684
86	2013	49	10.0714	75.0612	873.0796	3.0020	1.3990
2	2013	49	10.0714	75.0612	873.0796	3.0020	1.3990
4	2013	49	10.2286	74.6020	910.1990	2.6745	1.6347
47	2013	49	14.4000	72.8163	968.8929	2.6112	0.5490
82	2013	49	10.3857	73.4490	882.3918	2.9041	2.1612
19	2013	49	13.7571	71.0714	970.2122	3.9684	0.8102
20	2013	49	9.5857	75.1327	860.8816	3.0724	2.2061
80	2013	49	10.3857	73.4490	882.3918	2.9041	2.1612
3	2013	49	14.6429	67.9184	953.9071	3.4827	0.7235
52	2013	49	11.6286	72.2449	910.4020	2.3939	2.8082
70	2013	49	11.4000	79.6122	919.0694	2.8459	1.6071
64	2013	49	6.5571	79.4694	779.9673	4.6276	2.2990
48	2013	49	9.9143	76.5408	943.0010	2.0755	0.7010

65	2013	49	11.6286	72.2449	910.4020	2.3939	2.8082
44	2013	49	11.4000	79.6122	919.0694	2.8459	1.6071
75	2013	49	6.5571	79.4694	779.9673	4.6276	2.2990
40	2013	49	10.8571	73.1735	954.8949	4.0235	1.3041
11	2013	49	10.4714	72.6327	885.2224	3.1990	1.9337
35	2013	49	9.8571	80.1122	948.8347	3.4980	0.9796
78	2013	49	11.0000	74.1939	907.6888	2.3102	1.7143
28	2013	49	11.6571	74.1327	938.0153	3.3357	1.1184
39	2013	49	11.6286	72.2449	910.4020	2.3939	2.8082
24	2013	49	12.1143	69.7857	949.5684	3.1776	0.9980
63	2013	49	10.8571	73.1735	954.8949	4.0235	1.3041
62	2013	49	9.4857	76.0408	881.8898	3.4408	1.4755
1	2013	49	10.3857	73.4490	882.3918	2.9041	2.1612
31	2013	50	4.0286	77.6735	851.8490	2.0429	1.0510
79	2013	50	9.2857	75.5204	988.8490	2.8908	0.7765
51	2013	50	6.9000	79.5204	948.2235	3.1418	1.0367
14	2013	50	6.9857	82.0000	904.6041	2.2633	1.5816
67	2013	50	6.2857	72.6224	909.7745	1.8245	2.7745
42	2013	50	5.5857	77.9694	881.6480	1.8500	2.2357
50	2013	50	6.7143	77.4796	909.3418	1.3898	1.4480
43	2013	50	5.5857	77.9694	881.6480	1.8500	2.2357
85	2013	50	7.3286	79.4592	918.0806	1.8459	1.5714
25	2013	50	11.1429	79.0102	987.0643	3.0561	0.9898
69	2013	50	8.4143	68.4082	948.9235	2.7796	0.9643
57	2013	50	6.1714	80.1224	901.6398	1.7531	1.6071
9	2013	50	5.4714	77.6633	860.0378	1.6806	2.1582
72	2013	50	7.0000	73.1429	884.4561	2.4990	1.9429
26	2013	50	7.9143	79.7449	873.5469	2.9031	1.6388
7	2013	50	7.1571	77.6939	865.1602	2.0480	1.5173
83	2013	50	12.4571	71.9286	953.1224	2.8847	0.6051
76	2013	50	7.1000	74.7959	928.6776	2.9449	1.1398
36	2013	50	7.3714	74.4490	937.4235	2.8163	1.1510
81	2013	50	6.9000	79.5204	948.2235	3.1418	1.0367
15	2013	50	8.1000	76.7653	942.1643	1.5418	0.5878
32	2013	50	5.5857	77.9694	881.6480	1.8500	2.2357
73	2013	50	9.7143	76.4184	972.1153	2.6306	0.7357
71	2013	50	7.3714	74.4490	937.4235	2.8163	1.1510
41	2013	50	6.0429	80.4490	881.1459	1.8684	1.2071
10	2013	50	8.1286	73.7245	974.0857	2.6724	1.0235
23	2013	50	1.2857	80.6531	779.0265	3.9765	2.1480
27	2013	50	7.1571	77.6939	865.1602	2.0480	1.5173
60	2013	50	6.9000	79.5204	948.2235	3.1418	1.0367
53	2013	50	5.4714	77.6633	860.0378	1.6806	2.1582
66	2013	50	6.9857	82.0000	904.6041	2.2633	1.5816
59	2013	50	6.1714	80.1224	901.6398	1.7531	1.6071
61	2013	50	9.7143	76.4184	972.1153	2.6306	0.7357
84	2013	50	9.7143	76.4184	972.1153	2.6306	0.7357
38	2013	50	6.1714	80.1224	901.6398	1.7531	1.6071
87	2013	50	7.8286	75.4592	906.9929	1.8163	1.9133
34	2013	50	6.1714	80.1224	901.6398	1.7531	1.6071

29	2013	50	8.4143	68.4082	948.9235	2.7796	0.9643
5	2013	50	5.1143	81.1633	837.4051	2.4816	1.6663
8	2013	50	5.4714	77.6633	860.0378	1.6806	2.1582
12	2013	50	5.1143	81.1633	837.4051	2.4816	1.6663
13	2013	50	12.4571	71.9286	953.1224	2.8847	0.6051
18	2013	50	8.2857	74.4388	974.3214	3.1694	0.8418
33	2013	50	6.7143	77.4796	909.3418	1.3898	1.4480
56	2013	50	11.1429	79.0102	987.0643	3.0561	0.9898
77	2013	50	8.1000	76.7653	942.1643	1.5418	0.5878
54	2013	50	5.1143	81.1633	837.4051	2.4816	1.6663
21	2013	50	6.7143	77.4796	909.3418	1.3898	1.4480
68	2013	50	9.2857	75.5204	988.8490	2.8908	0.7765
74	2013	50	9.7143	76.4184	972.1153	2.6306	0.7357
88	2013	50	5.5857	77.9694	881.6480	1.8500	2.2357
16	2013	50	7.1000	74.7959	928.6776	2.9449	1.1398
30	2013	50	6.9857	82.0000	904.6041	2.2633	1.5816
6	2013	50	9.2857	75.5204	988.8490	2.8908	0.7765
49	2013	50	8.4143	68.4082	948.9235	2.7796	0.9643
22	2013	50	5.5857	77.9694	881.6480	1.8500	2.2357
45	2013	50	5.6429	82.4286	822.1520	3.1102	1.9316
58	2013	50	8.4143	68.4082	948.9235	2.7796	0.9643
37	2013	50	9.2857	75.5204	988.8490	2.8908	0.7765
17	2013	50	6.2857	72.6224	909.7745	1.8245	2.7745
55	2013	50	7.0000	73.1429	884.4561	2.4990	1.9429
46	2013	50	7.1000	74.7959	928.6776	2.9449	1.1398
86	2013	50	6.3857	76.4898	872.2378	1.7449	1.4255
2	2013	50	6.3857	76.4898	872.2378	1.7449	1.4255
4	2013	50	6.7143	77.4796	909.3418	1.3898	1.4480
47	2013	50	12.4714	76.3367	968.2980	1.9337	0.4735
82	2013	50	5.5857	77.9694	881.6480	1.8500	2.2357
19	2013	50	11.4000	69.6122	969.4918	3.6173	0.9561
20	2013	50	5.4714	77.6633	860.0378	1.6806	2.1582
80	2013	50	5.5857	77.9694	881.6480	1.8500	2.2357
3	2013	50	12.4571	71.9286	953.1224	2.8847	0.6051
52	2013	50	6.2857	72.6224	909.7745	1.8245	2.7745
70	2013	50	7.3286	79.4592	918.0806	1.8459	1.5714
64	2013	50	1.2857	80.6531	779.0265	3.9765	2.1480
48	2013	50	8.1000	76.7653	942.1643	1.5418	0.5878
65	2013	50	6.2857	72.6224	909.7745	1.8245	2.7745
44	2013	50	7.3286	79.4592	918.0806	1.8459	1.5714
75	2013	50	1.2857	80.6531	779.0265	3.9765	2.1480
40	2013	50	7.5857	71.2143	954.2837	3.6173	1.4082
11	2013	50	7.0000	73.1429	884.4561	2.4990	1.9429
35	2013	50	6.9000	79.5204	948.2235	3.1418	1.0367
78	2013	50	7.8286	75.4592	906.9929	1.8163	1.9133
28	2013	50	7.3714	74.4490	937.4235	2.8163	1.1510
39	2013	50	6.2857	72.6224	909.7745	1.8245	2.7745
24	2013	50	8.4143	68.4082	948.9235	2.7796	0.9643
63	2013	50	7.5857	71.2143	954.2837	3.6173	1.4082
62	2013	50	6.0429	80.4490	881.1459	1.8684	1.2071

1	2013	50	5.5857	77.9694	881.6480	1.8500	2.2357
31	2013	51	0.6857	79.2755	851.8082	1.5684	1.0561
79	2013	51	5.4429	76.1939	990.4776	1.5143	0.8082
51	2013	51	2.4143	84.4286	949.3224	1.2612	1.3112
14	2013	51	1.5571	89.2857	904.7163	1.2939	1.9041
67	2013	51	1.6000	78.2041	910.2755	0.7276	3.0827
42	2013	51	0.9571	83.5714	881.6224	1.1694	2.4714
50	2013	51	2.5000	79.7551	909.9245	1.2306	1.3980
43	2013	51	0.9571	83.5714	881.6224	1.1694	2.4714
85	2013	51	3.3000	80.8673	918.7500	1.4204	1.6010
25	2013	51	5.5857	81.7653	988.2908	1.9541	1.0551
69	2013	51	3.9429	70.7449	950.0939	1.2255	1.0857
57	2013	51	1.2857	85.5510	902.1878	0.4480	1.5765
9	2013	51	1.1571	81.4184	859.7418	1.5735	2.3673
72	2013	51	2.9857	74.7347	884.3347	2.3776	2.0786
26	2013	51	4.0857	85.1837	873.2704	2.6622	1.5704
7	2013	51	2.7286	81.4796	864.8439	2.1245	1.4745
83	2013	51	7.4286	78.3163	953.7082	1.9245	0.6520
76	2013	51	2.6571	78.2449	929.5112	1.1184	1.1704
36	2013	51	2.5429	77.9388	938.4214	0.8612	1.3143
81	2013	51	2.4143	84.4286	949.3224	1.2612	1.3112
15	2013	51	4.5429	74.7857	943.0694	1.0724	0.7480
32	2013	51	0.9571	83.5714	881.6224	1.1694	2.4714
73	2013	51	5.9143	76.2959	973.5347	1.3837	0.8194
71	2013	51	2.5429	77.9388	938.4214	0.8612	1.3143
41	2013	51	1.1286	84.0204	881.4041	1.3469	1.0888
10	2013	51	3.3000	76.2551	975.4429	1.1031	1.1122
23	2013	51	-1.5857	80.1429	778.1133	3.5194	2.1673
27	2013	51	2.7286	81.4796	864.8439	2.1245	1.4745
60	2013	51	2.4143	84.4286	949.3224	1.2612	1.3112
53	2013	51	1.1571	81.4184	859.7418	1.5735	2.3673
66	2013	51	1.5571	89.2857	904.7163	1.2939	1.9041
59	2013	51	1.2857	85.5510	902.1878	0.4480	1.5765
61	2013	51	5.9143	76.2959	973.5347	1.3837	0.8194
84	2013	51	5.9143	76.2959	973.5347	1.3837	0.8194
38	2013	51	1.2857	85.5510	902.1878	0.4480	1.5765
87	2013	51	2.8857	77.2653	907.2214	1.5092	2.4327
34	2013	51	1.2857	85.5510	902.1878	0.4480	1.5765
29	2013	51	3.9429	70.7449	950.0939	1.2255	1.0857
5	2013	51	1.2571	81.7347	836.9153	2.4102	1.7276
8	2013	51	1.1571	81.4184	859.7418	1.5735	2.3673
12	2013	51	1.2571	81.7347	836.9153	2.4102	1.7276
13	2013	51	7.4286	78.3163	953.7082	1.9245	0.6520
18	2013	51	4.3714	76.2857	975.7990	1.3939	1.0020
33	2013	51	2.5000	79.7551	909.9245	1.2306	1.3980
56	2013	51	5.5857	81.7653	988.2908	1.9541	1.0551
77	2013	51	4.5429	74.7857	943.0694	1.0724	0.7480
54	2013	51	1.2571	81.7347	836.9153	2.4102	1.7276
21	2013	51	2.5000	79.7551	909.9245	1.2306	1.3980
68	2013	51	5.4429	76.1939	990.4776	1.5143	0.8082

74	2013	51	5.9143	76.2959	973.5347	1.3837	0.8194
88	2013	51	0.9571	83.5714	881.6224	1.1694	2.4714
16	2013	51	2.6571	78.2449	929.5112	1.1184	1.1704
30	2013	51	1.5571	89.2857	904.7163	1.2939	1.9041
6	2013	51	5.4429	76.1939	990.4776	1.5143	0.8082
49	2013	51	3.9429	70.7449	950.0939	1.2255	1.0857
22	2013	51	0.9571	83.5714	881.6224	1.1694	2.4714
45	2013	51	0.9714	83.4898	821.5122	2.8163	1.8908
58	2013	51	3.9429	70.7449	950.0939	1.2255	1.0857
37	2013	51	5.4429	76.1939	990.4776	1.5143	0.8082
17	2013	51	1.6000	78.2041	910.2755	0.7276	3.0827
55	2013	51	2.9857	74.7347	884.3347	2.3776	2.0786
46	2013	51	2.6571	78.2449	929.5112	1.1184	1.1704
86	2013	51	2.2714	79.4082	872.2643	1.4837	1.4204
2	2013	51	2.2714	79.4082	872.2643	1.4837	1.4204
4	2013	51	2.5000	79.7551	909.9245	1.2306	1.3980
47	2013	51	6.6000	81.6633	969.1765	1.4796	0.5306
82	2013	51	0.9571	83.5714	881.6224	1.1694	2.4714
19	2013	51	5.4286	71.6020	970.3327	2.2500	1.1082
20	2013	51	1.1571	81.4184	859.7418	1.5735	2.3673
80	2013	51	0.9571	83.5714	881.6224	1.1694	2.4714
3	2013	51	7.4286	78.3163	953.7082	1.9245	0.6520
52	2013	51	1.6000	78.2041	910.2755	0.7276	3.0827
70	2013	51	3.3000	80.8673	918.7500	1.4204	1.6010
64	2013	51	-1.5857	80.1429	778.1133	3.5194	2.1673
48	2013	51	4.5429	74.7857	943.0694	1.0724	0.7480
65	2013	51	1.6000	78.2041	910.2755	0.7276	3.0827
44	2013	51	3.3000	80.8673	918.7500	1.4204	1.6010
75	2013	51	-1.5857	80.1429	778.1133	3.5194	2.1673
40	2013	51	2.2000	78.2449	955.2531	1.6745	1.6510
11	2013	51	2.9857	74.7347	884.3347	2.3776	2.0786
35	2013	51	2.4143	84.4286	949.3224	1.2612	1.3112
78	2013	51	2.8857	77.2653	907.2214	1.5092	2.4327
28	2013	51	2.5429	77.9388	938.4214	0.8612	1.3143
39	2013	51	1.6000	78.2041	910.2755	0.7276	3.0827
24	2013	51	3.9429	70.7449	950.0939	1.2255	1.0857
63	2013	51	2.2000	78.2449	955.2531	1.6745	1.6510
62	2013	51	1.1286	84.0204	881.4041	1.3469	1.0888
1	2013	51	0.9571	83.5714	881.6224	1.1694	2.4714
31	2013	52	0.5857	82.2347	853.7418	1.3929	0.8857
79	2013	52	6.8714	72.1735	994.3092	1.2867	0.7306
51	2013	52	4.3571	81.3776	952.6316	0.7020	1.3214
14	2013	52	3.9286	80.8673	907.3163	1.6816	1.7214
67	2013	52	3.6000	70.3469	913.0939	0.6367	3.2500
42	2013	52	2.0000	78.4184	883.9316	1.1857	2.3480
50	2013	52	3.4000	75.7041	912.8602	1.3582	1.3704
43	2013	52	2.0000	78.4184	883.9316	1.1857	2.3480
85	2013	52	3.6143	79.9592	921.8316	1.2500	1.2408
25	2013	52	7.7857	79.3061	992.0755	2.1163	1.0194
69	2013	52	5.5571	68.2959	953.5673	0.6235	0.9714

57	2013	52	2.7714	83.6020	905.0531	0.2500	1.2949
9	2013	52	1.6857	79.1224	861.8000	1.7429	2.1724
72	2013	52	3.9286	68.5306	886.6786	2.2786	1.9347
26	2013	52	4.4143	81.9796	875.3041	2.3776	1.4133
7	2013	52	3.4000	79.9082	866.9378	2.0071	1.2296
83	2013	52	9.4571	75.5918	956.9214	1.6286	0.5898
76	2013	52	4.5143	72.9490	932.7184	0.3714	1.0378
36	2013	52	4.5857	72.5816	941.6704	0.3653	1.3092
81	2013	52	4.3571	81.3776	952.6316	0.7020	1.3214
15	2013	52	5.2429	69.3469	946.4133	0.9520	0.6949
32	2013	52	2.0000	78.4184	883.9316	1.1857	2.3480
73	2013	52	7.6429	70.5612	977.2112	0.7388	0.7296
71	2013	52	4.5857	72.5816	941.6704	0.3653	1.3092
41	2013	52	1.9286	83.0000	883.9153	1.1571	0.9439
10	2013	52	5.1714	73.6837	979.0959	0.7286	1.1102
23	2013	52	-2.2714	83.5918	779.0449	2.4255	1.8663
27	2013	52	3.4000	79.9082	866.9378	2.0071	1.2296
60	2013	52	4.3571	81.3776	952.6316	0.7020	1.3214
53	2013	52	1.6857	79.1224	861.8000	1.7429	2.1724
66	2013	52	3.9286	80.8673	907.3163	1.6816	1.7214
59	2013	52	2.7714	83.6020	905.0531	0.2500	1.2949
61	2013	52	7.6429	70.5612	977.2112	0.7388	0.7296
84	2013	52	7.6429	70.5612	977.2112	0.7388	0.7296
38	2013	52	2.7714	83.6020	905.0531	0.2500	1.2949
87	2013	52	4.6000	71.0306	909.9296	1.4929	2.3827
34	2013	52	2.7714	83.6020	905.0531	0.2500	1.2949
29	2013	52	5.5571	68.2959	953.5673	0.6235	0.9714
5	2013	52	0.6571	84.0000	838.6959	2.1112	1.5735
8	2013	52	1.6857	79.1224	861.8000	1.7429	2.1724
12	2013	52	0.6571	84.0000	838.6959	2.1112	1.5735
13	2013	52	9.4571	75.5918	956.9214	1.6286	0.5898
18	2013	52	5.9714	73.7653	979.4020	1.0020	0.9459
33	2013	52	3.4000	75.7041	912.8602	1.3582	1.3704
56	2013	52	7.7857	79.3061	992.0755	2.1163	1.0194
77	2013	52	5.2429	69.3469	946.4133	0.9520	0.6949
54	2013	52	0.6571	84.0000	838.6959	2.1112	1.5735
21	2013	52	3.4000	75.7041	912.8602	1.3582	1.3704
68	2013	52	6.8714	72.1735	994.3092	1.2867	0.7306
74	2013	52	7.6429	70.5612	977.2112	0.7388	0.7296
88	2013	52	2.0000	78.4184	883.9316	1.1857	2.3480
16	2013	52	4.5143	72.9490	932.7184	0.3714	1.0378
30	2013	52	3.9286	80.8673	907.3163	1.6816	1.7214
6	2013	52	6.8714	72.1735	994.3092	1.2867	0.7306
49	2013	52	5.5571	68.2959	953.5673	0.6235	0.9714
22	2013	52	2.0000	78.4184	883.9316	1.1857	2.3480
45	2013	52	0.8571	87.8061	823.0847	2.2673	1.4173
58	2013	52	5.5571	68.2959	953.5673	0.6235	0.9714
37	2013	52	6.8714	72.1735	994.3092	1.2867	0.7306
17	2013	52	3.6000	70.3469	913.0939	0.6367	3.2500
55	2013	52	3.9286	68.5306	886.6786	2.2786	1.9347

46	2013	52	4.5143	72.9490	932.7184	0.3714	1.0378
86	2013	52	2.5000	79.2245	874.5704	1.3929	1.2633
2	2013	52	2.5000	79.2245	874.5704	1.3929	1.2633
4	2013	52	3.4000	75.7041	912.8602	1.3582	1.3704
47	2013	52	9.3000	74.6837	972.6908	1.8224	0.5051
82	2013	52	2.0000	78.4184	883.9316	1.1857	2.3480
19	2013	52	8.0429	67.8061	973.7327	3.2327	1.0041
20	2013	52	1.6857	79.1224	861.8000	1.7429	2.1724
80	2013	52	2.0000	78.4184	883.9316	1.1857	2.3480
3	2013	52	9.4571	75.5918	956.9214	1.6286	0.5898
52	2013	52	3.6000	70.3469	913.0939	0.6367	3.2500
70	2013	52	3.6143	79.9592	921.8316	1.2500	1.2408
64	2013	52	-2.2714	83.5918	779.0449	2.4255	1.8663
48	2013	52	5.2429	69.3469	946.4133	0.9520	0.6949
65	2013	52	3.6000	70.3469	913.0939	0.6367	3.2500
44	2013	52	3.6143	79.9592	921.8316	1.2500	1.2408
75	2013	52	-2.2714	83.5918	779.0449	2.4255	1.8663
40	2013	52	4.5429	75.8265	958.5449	1.8306	1.3857
11	2013	52	3.9286	68.5306	886.6786	2.2786	1.9347
35	2013	52	4.3571	81.3776	952.6316	0.7020	1.3214
78	2013	52	4.6000	71.0306	909.9296	1.4929	2.3827
28	2013	52	4.5857	72.5816	941.6704	0.3653	1.3092
39	2013	52	3.6000	70.3469	913.0939	0.6367	3.2500
24	2013	52	5.5571	68.2959	953.5673	0.6235	0.9714
63	2013	52	4.5429	75.8265	958.5449	1.8306	1.3857
62	2013	52	1.9286	83.0000	883.9153	1.1571	0.9439
1	2013	52	2.0000	78.4184	883.9316	1.1857	2.3480
31	2014	1	4.1429	82.3776	853.3765	1.0806	0.9031
79	2014	1	9.1143	64.0510	993.3847	2.1133	0.6510
51	2014	1	7.2286	71.1735	951.8673	1.7520	1.0980
14	2014	1	7.7714	66.2959	907.0633	2.2173	1.4918
67	2014	1	7.8571	57.0306	912.5888	1.3918	2.9633
42	2014	1	6.8286	67.3265	883.6061	1.1163	2.1633
50	2014	1	5.2143	69.7653	912.5122	1.4959	1.3265
43	2014	1	6.8286	67.3265	883.6061	1.1163	2.1633
85	2014	1	5.8143	75.8367	921.5735	1.0582	1.0357
25	2014	1	9.0286	71.1327	991.5122	2.9816	0.9480
69	2014	1	7.6571	61.1939	952.8959	1.6102	0.8582
57	2014	1	5.6857	74.5816	903.4153	1.0337	1.2184
9	2014	1	5.8429	74.6327	861.5469	0.9541	2.0582
72	2014	1	7.6286	62.9796	886.4602	1.8531	1.8071
26	2014	1	8.3143	79.9082	875.0265	1.4357	1.4765
7	2014	1	7.5000	75.7857	866.7020	1.4255	1.1827
83	2014	1	11.7429	67.6531	956.6071	1.8663	0.6276
76	2014	1	6.8286	61.3673	932.2051	1.7663	1.1265
36	2014	1	7.8286	61.3980	941.0010	1.7653	1.1888
81	2014	1	7.2286	71.1735	951.8673	1.7520	1.0980
15	2014	1	6.0714	65.2449	945.9755	1.3204	0.7102
32	2014	1	6.8286	67.3265	883.6061	1.1163	2.1633
73	2014	1	9.0571	63.1224	976.4571	1.5459	0.6878

71	2014	1	7.8286	61.3980	941.0010	1.7653	1.1888
41	2014	1	5.7000	77.3265	883.5143	1.0031	0.8990
10	2014	1	8.2429	64.8980	978.2888	1.7602	1.0643
23	2014	1	2.7143	87.0714	778.6480	1.6898	1.7255
27	2014	1	7.5000	75.7857	866.7020	1.4255	1.1827
60	2014	1	7.2286	71.1735	951.8673	1.7520	1.0980
53	2014	1	5.8429	74.6327	861.5469	0.9541	2.0582
66	2014	1	7.7714	66.2959	907.0633	2.2173	1.4918
59	2014	1	5.6857	74.5816	903.4153	1.0337	1.2184
61	2014	1	9.0571	63.1224	976.4571	1.5459	0.6878
84	2014	1	9.0571	63.1224	976.4571	1.5459	0.6878
38	2014	1	5.6857	74.5816	903.4153	1.0337	1.2184
87	2014	1	7.7429	63.6633	909.6133	1.3714	1.9633
34	2014	1	5.6857	74.5816	903.4153	1.0337	1.2184
29	2014	1	7.6571	61.1939	952.8959	1.6102	0.8582
5	2014	1	5.6000	86.2653	838.4316	1.5092	1.5255
8	2014	1	5.8429	74.6327	861.5469	0.9541	2.0582
12	2014	1	5.6000	86.2653	838.4316	1.5092	1.5255
13	2014	1	11.7429	67.6531	956.6071	1.8663	0.6276
18	2014	1	7.7429	65.8469	978.5061	1.9306	0.8194
33	2014	1	5.2143	69.7653	912.5122	1.4959	1.3265
56	2014	1	9.0286	71.1327	991.5122	2.9816	0.9480
77	2014	1	6.0714	65.2449	945.9755	1.3204	0.7102
54	2014	1	5.6000	86.2653	838.4316	1.5092	1.5255
21	2014	1	5.2143	69.7653	912.5122	1.4959	1.3265
68	2014	1	9.1143	64.0510	993.3847	2.1133	0.6510
74	2014	1	9.0571	63.1224	976.4571	1.5459	0.6878
88	2014	1	6.8286	67.3265	883.6061	1.1163	2.1633
16	2014	1	6.8286	61.3673	932.2051	1.7663	1.1265
30	2014	1	7.7714	66.2959	907.0633	2.2173	1.4918
6	2014	1	9.1143	64.0510	993.3847	2.1133	0.6510
49	2014	1	7.6571	61.1939	952.8959	1.6102	0.8582
22	2014	1	6.8286	67.3265	883.6061	1.1163	2.1633
45	2014	1	6.3000	88.8265	822.7827	1.6592	1.3745
58	2014	1	7.6571	61.1939	952.8959	1.6102	0.8582
37	2014	1	9.1143	64.0510	993.3847	2.1133	0.6510
17	2014	1	7.8571	57.0306	912.5888	1.3918	2.9633
55	2014	1	7.6286	62.9796	886.4602	1.8531	1.8071
46	2014	1	6.8286	61.3673	932.2051	1.7663	1.1265
86	2014	1	6.8000	76.5816	874.2245	1.1102	1.1500
2	2014	1	6.8000	76.5816	874.2245	1.1102	1.1500
4	2014	1	5.2143	69.7653	912.5122	1.4959	1.3265
47	2014	1	11.3857	63.8571	972.2082	1.7286	0.4673
82	2014	1	6.8286	67.3265	883.6061	1.1163	2.1633
19	2014	1	9.6143	59.3980	973.4020	4.0469	0.8398
20	2014	1	5.8429	74.6327	861.5469	0.9541	2.0582
80	2014	1	6.8286	67.3265	883.6061	1.1163	2.1633
3	2014	1	11.7429	67.6531	956.6071	1.8663	0.6276
52	2014	1	7.8571	57.0306	912.5888	1.3918	2.9633
70	2014	1	5.8143	75.8367	921.5735	1.0582	1.0357

64	2014	1	2.7143	87.0714	778.6480	1.6898	1.7255
48	2014	1	6.0714	65.2449	945.9755	1.3204	0.7102
65	2014	1	7.8571	57.0306	912.5888	1.3918	2.9633
44	2014	1	5.8143	75.8367	921.5735	1.0582	1.0357
75	2014	1	2.7143	87.0714	778.6480	1.6898	1.7255
40	2014	1	7.8571	63.2653	957.9878	3.1612	1.1959
11	2014	1	7.6286	62.9796	886.4602	1.8531	1.8071
35	2014	1	7.2286	71.1735	951.8673	1.7520	1.0980
78	2014	1	7.7429	63.6633	909.6133	1.3714	1.9633
28	2014	1	7.8286	61.3980	941.0010	1.7653	1.1888
39	2014	1	7.8571	57.0306	912.5888	1.3918	2.9633
24	2014	1	7.6571	61.1939	952.8959	1.6102	0.8582
63	2014	1	7.8571	63.2653	957.9878	3.1612	1.1959
62	2014	1	5.7000	77.3265	883.5143	1.0031	0.8990
1	2014	1	6.8286	67.3265	883.6061	1.1163	2.1633
31	2014	2	1.8286	75.4490	851.0939	1.8143	1.0316
79	2014	2	6.3143	70.1633	989.8857	2.1714	0.6990
51	2014	2	3.9286	72.1020	948.6847	2.1286	1.1857
14	2014	2	5.4857	67.7143	904.1990	2.3551	2.1684
67	2014	2	3.9000	64.8367	909.5082	1.7357	2.8255
42	2014	2	3.0286	69.7347	880.9276	1.4357	2.2878
50	2014	2	3.8571	74.0408	909.6531	1.5429	1.2806
43	2014	2	3.0286	69.7347	880.9276	1.4357	2.2878
85	2014	2	4.2286	77.9388	918.8531	1.2571	1.0602
25	2014	2	8.6571	73.0510	987.9969	2.5398	0.8398
69	2014	2	5.4000	67.8673	949.5949	2.1571	1.2602
57	2014	2	2.5000	76.8163	896.5857	1.4643	1.4714
9	2014	2	3.4571	75.6020	858.9898	1.0786	2.3255
72	2014	2	5.7571	67.9388	883.6112	2.2429	2.1357
26	2014	2	7.5286	78.6837	872.2745	2.8796	2.0765
7	2014	2	5.9429	76.3571	864.1092	2.4286	1.5153
83	2014	2	11.1000	67.8469	953.1735	2.7214	0.7490
76	2014	2	4.0429	67.5918	929.5173	2.5786	1.5500
36	2014	2	4.4000	67.1020	937.7663	2.3347	1.3480
81	2014	2	3.9286	72.1020	948.6847	2.1286	1.1857
15	2014	2	5.5143	68.8776	942.8663	1.7000	0.8408
32	2014	2	3.0286	69.7347	880.9276	1.4357	2.2878
73	2014	2	7.0857	68.1633	973.0551	2.0786	0.9061
71	2014	2	4.4000	67.1020	937.7663	2.3347	1.3480
41	2014	2	3.0571	76.8469	880.7704	1.2643	0.9643
10	2014	2	5.3571	70.1224	974.9398	2.1980	0.9357
23	2014	2	0.0000	80.4694	776.8745	3.9582	2.1214
27	2014	2	5.9429	76.3571	864.1092	2.4286	1.5153
60	2014	2	3.9286	72.1020	948.6847	2.1286	1.1857
53	2014	2	3.4571	75.6020	858.9898	1.0786	2.3255
66	2014	2	5.4857	67.7143	904.1990	2.3551	2.1684
59	2014	2	2.5000	76.8163	896.5857	1.4643	1.4714
61	2014	2	7.0857	68.1633	973.0551	2.0786	0.9061
84	2014	2	7.0857	68.1633	973.0551	2.0786	0.9061
38	2014	2	2.5000	76.8163	896.5857	1.4643	1.4714

87	2014	2	6.2286	67.0408	906.5796	1.4714	2.2888
34	2014	2	2.5000	76.8163	896.5857	1.4643	1.4714
29	2014	2	5.4000	67.8673	949.5949	2.1571	1.2602
5	2014	2	4.1857	83.1939	835.9296	2.8592	1.7949
8	2014	2	3.4571	75.6020	858.9898	1.0786	2.3255
12	2014	2	4.1857	83.1939	835.9296	2.8592	1.7949
13	2014	2	11.1000	67.8469	953.1735	2.7214	0.7490
18	2014	2	5.4286	71.1020	975.1000	2.2296	0.8184
33	2014	2	3.8571	74.0408	909.6531	1.5429	1.2806
56	2014	2	8.6571	73.0510	987.9969	2.5398	0.8398
77	2014	2	5.5143	68.8776	942.8663	1.7000	0.8408
54	2014	2	4.1857	83.1939	835.9296	2.8592	1.7949
21	2014	2	3.8571	74.0408	909.6531	1.5429	1.2806
68	2014	2	6.3143	70.1633	989.8857	2.1714	0.6990
74	2014	2	7.0857	68.1633	973.0551	2.0786	0.9061
88	2014	2	3.0286	69.7347	880.9276	1.4357	2.2878
16	2014	2	4.0429	67.5918	929.5173	2.5786	1.5500
30	2014	2	5.4857	67.7143	904.1990	2.3551	2.1684
6	2014	2	6.3143	70.1633	989.8857	2.1714	0.6990
49	2014	2	5.4000	67.8673	949.5949	2.1571	1.2602
22	2014	2	3.0286	69.7347	880.9276	1.4357	2.2878
45	2014	2	6.1714	77.0510	820.3612	3.5418	2.0776
58	2014	2	5.4000	67.8673	949.5949	2.1571	1.2602
37	2014	2	6.3143	70.1633	989.8857	2.1714	0.6990
17	2014	2	3.9000	64.8367	909.5082	1.7357	2.8255
55	2014	2	5.7571	67.9388	883.6112	2.2429	2.1357
46	2014	2	4.0429	67.5918	929.5173	2.5786	1.5500
86	2014	2	3.7286	76.3265	871.6214	1.4000	1.2224
2	2014	2	3.7286	76.3265	871.6214	1.4000	1.2224
4	2014	2	3.8571	74.0408	909.6531	1.5429	1.2806
47	2014	2	11.0571	65.7959	968.4051	1.6786	0.6235
82	2014	2	3.0286	69.7347	880.9276	1.4357	2.2878
19	2014	2	9.5714	63.8673	970.1918	3.1653	1.0408
20	2014	2	3.4571	75.6020	858.9898	1.0786	2.3255
80	2014	2	3.0286	69.7347	880.9276	1.4357	2.2878
3	2014	2	11.1000	67.8469	953.1735	2.7214	0.7490
52	2014	2	3.9000	64.8367	909.5082	1.7357	2.8255
70	2014	2	4.2286	77.9388	918.8531	1.2571	1.0602
64	2014	2	0.0000	80.4694	776.8745	3.9582	2.1214
48	2014	2	5.5143	68.8776	942.8663	1.7000	0.8408
65	2014	2	3.9000	64.8367	909.5082	1.7357	2.8255
44	2014	2	4.2286	77.9388	918.8531	1.2571	1.0602
75	2014	2	0.0000	80.4694	776.8745	3.9582	2.1214
40	2014	2	4.8714	67.1224	954.9010	2.7918	1.4959
11	2014	2	5.7571	67.9388	883.6112	2.2429	2.1357
35	2014	2	3.9286	72.1020	948.6847	2.1286	1.1857
78	2014	2	6.2286	67.0408	906.5796	1.4714	2.2888
28	2014	2	4.4000	67.1020	937.7663	2.3347	1.3480
39	2014	2	3.9000	64.8367	909.5082	1.7357	2.8255
24	2014	2	5.4000	67.8673	949.5949	2.1571	1.2602

63	2014	2	4.8714	67.1224	954.9010	2.7918	1.4959
62	2014	2	3.0571	76.8469	880.7704	1.2643	0.9643
1	2014	2	3.0286	69.7347	880.9276	1.4357	2.2878
31	2014	3	3.3429	76.6122	852.0663	2.1163	0.9643
79	2014	3	7.2571	75.7347	991.9571	1.8480	0.6969
51	2014	3	4.2571	77.7755	950.5449	2.0959	1.3071
14	2014	3	5.2857	76.2347	905.5245	2.3102	2.3286
67	2014	3	5.2143	78.1837	910.9204	1.6418	2.8102
42	2014	3	3.8857	81.0306	882.1816	1.3480	2.3133
50	2014	3	4.7286	79.7041	910.8347	0.9082	1.3092
43	2014	3	3.8857	81.0306	882.1816	1.3480	2.3133
85	2014	3	5.4429	81.6531	920.0918	1.2490	1.1724
25	2014	3	7.5714	78.2551	989.9031	1.8898	0.8429
69	2014	3	4.8857	78.9796	951.3092	1.4429	1.3551
57	2014	3	3.6000	85.9592	895.0071	0.8735	1.5031
9	2014	3	4.0571	80.9490	860.1071	1.4561	2.3306
72	2014	3	5.2571	76.2347	884.7612	1.7878	2.2704
26	2014	3	6.3143	79.6122	873.3429	3.3929	2.2245
7	2014	3	5.2857	80.3163	865.2224	2.5276	1.7490
83	2014	3	8.9000	72.1531	954.6163	2.3020	0.7653
76	2014	3	4.0143	79.7755	931.3939	1.5429	1.5959
36	2014	3	4.9143	78.0612	939.4306	2.0541	1.4582
81	2014	3	4.2571	77.7755	950.5449	2.0959	1.3071
15	2014	3	6.0571	71.8776	944.2418	1.2990	0.8980
32	2014	3	3.8857	81.0306	882.1816	1.3480	2.3133
73	2014	3	6.9143	75.7551	974.8959	1.5827	1.0449
71	2014	3	4.9143	78.0612	939.4306	2.0541	1.4582
41	2014	3	4.0571	81.5510	881.8612	1.6398	1.1449
10	2014	3	5.9143	78.0714	976.9612	2.1857	0.9490
23	2014	3	1.3714	80.3265	777.8000	4.8551	2.3020
27	2014	3	5.2857	80.3163	865.2224	2.5276	1.7490
60	2014	3	4.2571	77.7755	950.5449	2.0959	1.3071
53	2014	3	4.0571	80.9490	860.1071	1.4561	2.3306
66	2014	3	5.2857	76.2347	905.5245	2.3102	2.3286
59	2014	3	3.6000	85.9592	895.0071	0.8735	1.5031
61	2014	3	6.9143	75.7551	974.8959	1.5827	1.0449
84	2014	3	6.9143	75.7551	974.8959	1.5827	1.0449
38	2014	3	3.6000	85.9592	895.0071	0.8735	1.5031
87	2014	3	5.2571	74.1224	907.8735	1.3622	2.4582
34	2014	3	3.6000	85.9592	895.0071	0.8735	1.5031
29	2014	3	4.8857	78.9796	951.3092	1.4429	1.3551
5	2014	3	3.5000	84.5816	836.8786	3.1143	1.9510
8	2014	3	4.0571	80.9490	860.1071	1.4561	2.3306
12	2014	3	3.5000	84.5816	836.8786	3.1143	1.9510
13	2014	3	8.9000	72.1531	954.6163	2.3020	0.7653
18	2014	3	5.6429	77.1327	977.0663	2.0918	0.8408
33	2014	3	4.7286	79.7041	910.8347	0.9082	1.3092
56	2014	3	7.5714	78.2551	989.9031	1.8898	0.8429
77	2014	3	6.0571	71.8776	944.2418	1.2990	0.8980
54	2014	3	3.5000	84.5816	836.8786	3.1143	1.9510

21	2014	3	4.7286	79.7041	910.8347	0.9082	1.3092
68	2014	3	7.2571	75.7347	991.9571	1.8480	0.6969
74	2014	3	6.9143	75.7551	974.8959	1.5827	1.0449
88	2014	3	3.8857	81.0306	882.1816	1.3480	2.3133
16	2014	3	4.0143	79.7755	931.3939	1.5429	1.5959
30	2014	3	5.2857	76.2347	905.5245	2.3102	2.3286
6	2014	3	7.2571	75.7347	991.9571	1.8480	0.6969
49	2014	3	4.8857	78.9796	951.3092	1.4429	1.3551
22	2014	3	3.8857	81.0306	882.1816	1.3480	2.3133
45	2014	3	4.5286	74.5612	821.1918	4.0816	2.2949
58	2014	3	4.8857	78.9796	951.3092	1.4429	1.3551
37	2014	3	7.2571	75.7347	991.9571	1.8480	0.6969
17	2014	3	5.2143	78.1837	910.9204	1.6418	2.8102
55	2014	3	5.2571	76.2347	884.7612	1.7878	2.2704
46	2014	3	4.0143	79.7755	931.3939	1.5429	1.5959
86	2014	3	4.8429	79.5306	872.6694	1.6888	1.3398
2	2014	3	4.8429	79.5306	872.6694	1.6888	1.3398
4	2014	3	4.7286	79.7041	910.8347	0.9082	1.3092
47	2014	3	8.6000	71.3980	969.8714	1.5337	0.7122
82	2014	3	3.8857	81.0306	882.1816	1.3480	2.3133
19	2014	3	7.5143	72.6122	971.9500	2.1918	1.2806
20	2014	3	4.0571	80.9490	860.1071	1.4561	2.3306
80	2014	3	3.8857	81.0306	882.1816	1.3480	2.3133
3	2014	3	8.9000	72.1531	954.6163	2.3020	0.7653
52	2014	3	5.2143	78.1837	910.9204	1.6418	2.8102
70	2014	3	5.4429	81.6531	920.0918	1.2490	1.1724
64	2014	3	1.3714	80.3265	777.8000	4.8551	2.3020
48	2014	3	6.0571	71.8776	944.2418	1.2990	0.8980
65	2014	3	5.2143	78.1837	910.9204	1.6418	2.8102
44	2014	3	5.4429	81.6531	920.0918	1.2490	1.1724
75	2014	3	1.3714	80.3265	777.8000	4.8551	2.3020
40	2014	3	5.5143	78.4388	956.7337	1.8684	1.7224
11	2014	3	5.2571	76.2347	884.7612	1.7878	2.2704
35	2014	3	4.2571	77.7755	950.5449	2.0959	1.3071
78	2014	3	5.2571	74.1224	907.8735	1.3622	2.4582
28	2014	3	4.9143	78.0612	939.4306	2.0541	1.4582
39	2014	3	5.2143	78.1837	910.9204	1.6418	2.8102
24	2014	3	4.8857	78.9796	951.3092	1.4429	1.3551
63	2014	3	5.5143	78.4388	956.7337	1.8684	1.7224
62	2014	3	4.0571	81.5510	881.8612	1.6398	1.1449
1	2014	3	3.8857	81.0306	882.1816	1.3480	2.3133
31	2014	4	5.9286	74.7551	854.2153	3.6041	0.9112
79	2014	4	9.6429	70.1939	994.1449	4.0694	0.6622
51	2014	4	7.6714	72.9286	952.8469	4.7990	0.9724
14	2014	4	7.4143	69.9286	908.2214	4.3204	1.9622
67	2014	4	8.0857	70.8469	913.4571	4.0010	3.0143
42	2014	4	7.8000	73.4490	884.6837	3.6010	2.2949
50	2014	4	8.1857	71.0000	912.9612	3.3245	1.4459
43	2014	4	7.8000	73.4490	884.6837	3.6010	2.2949
85	2014	4	10.3429	71.3673	922.0480	3.5765	1.5541

25	2014	4	8.9571	76.4796	992.7255	3.4061	0.8888
69	2014	4	9.1429	73.6735	953.6429	3.7949	1.1796
57	2014	4	8.6143	75.5714	897.1745	3.3224	1.7153
9	2014	4	7.1714	72.3673	862.7122	4.4102	2.1296
72	2014	4	7.7429	70.7551	887.5551	4.0010	1.9327
26	2014	4	8.4714	75.8776	876.2459	4.2459	1.8969
7	2014	4	6.9000	75.2755	868.0102	4.0153	1.6102
83	2014	4	10.4286	69.6122	957.8643	3.5837	0.7898
76	2014	4	7.9429	73.4388	933.6490	3.6510	1.3653
36	2014	4	8.8571	72.8776	941.8561	4.3378	1.4082
81	2014	4	7.6714	72.9286	952.8469	4.7990	0.9724
15	2014	4	8.9143	64.3367	946.3510	3.2969	0.9061
32	2014	4	7.8000	73.4490	884.6837	3.6010	2.2949
73	2014	4	10.4000	71.6939	977.1092	3.7745	0.8949
71	2014	4	8.8571	72.8776	941.8561	4.3378	1.4082
41	2014	4	7.0429	75.2347	884.1500	4.2000	1.1827
10	2014	4	8.5429	71.2347	979.3051	4.5490	0.9582
23	2014	4	4.6143	76.9898	780.3633	5.3551	2.2347
27	2014	4	6.9000	75.2755	868.0102	4.0153	1.6102
60	2014	4	7.6714	72.9286	952.8469	4.7990	0.9724
53	2014	4	7.1714	72.3673	862.7122	4.4102	2.1296
66	2014	4	7.4143	69.9286	908.2214	4.3204	1.9622
59	2014	4	8.6143	75.5714	897.1745	3.3224	1.7153
61	2014	4	10.4000	71.6939	977.1092	3.7745	0.8949
84	2014	4	10.4000	71.6939	977.1092	3.7745	0.8949
38	2014	4	8.6143	75.5714	897.1745	3.3224	1.7153
87	2014	4	8.0286	70.3980	910.5898	3.6071	2.1571
34	2014	4	8.6143	75.5714	897.1745	3.3224	1.7153
29	2014	4	9.1429	73.6735	953.6429	3.7949	1.1796
5	2014	4	6.2143	78.6531	839.5857	4.2918	1.8204
8	2014	4	7.1714	72.3673	862.7122	4.4102	2.1296
12	2014	4	6.2143	78.6531	839.5857	4.2918	1.8204
13	2014	4	10.4286	69.6122	957.8643	3.5837	0.7898
18	2014	4	8.2143	71.1020	979.2214	4.4082	0.8020
33	2014	4	8.1857	71.0000	912.9612	3.3245	1.4459
56	2014	4	8.9571	76.4796	992.7255	3.4061	0.8888
77	2014	4	8.9143	64.3367	946.3510	3.2969	0.9061
54	2014	4	6.2143	78.6531	839.5857	4.2918	1.8204
21	2014	4	8.1857	71.0000	912.9612	3.3245	1.4459
68	2014	4	9.6429	70.1939	994.1449	4.0694	0.6622
74	2014	4	10.4000	71.6939	977.1092	3.7745	0.8949
88	2014	4	7.8000	73.4490	884.6837	3.6010	2.2949
16	2014	4	7.9429	73.4388	933.6490	3.6510	1.3653
30	2014	4	7.4143	69.9286	908.2214	4.3204	1.9622
6	2014	4	9.6429	70.1939	994.1449	4.0694	0.6622
49	2014	4	9.1429	73.6735	953.6429	3.7949	1.1796
22	2014	4	7.8000	73.4490	884.6837	3.6010	2.2949
45	2014	4	7.2286	74.0612	823.8816	4.7959	2.0347
58	2014	4	9.1429	73.6735	953.6429	3.7949	1.1796
37	2014	4	9.6429	70.1939	994.1449	4.0694	0.6622

17	2014	4	8.0857	70.8469	913.4571	4.0010	3.0143
55	2014	4	7.7429	70.7551	887.5551	4.0010	1.9327
46	2014	4	7.9429	73.4388	933.6490	3.6510	1.3653
86	2014	4	7.7857	71.7245	875.0673	3.8724	1.5337
2	2014	4	7.7857	71.7245	875.0673	3.8724	1.5337
4	2014	4	8.1857	71.0000	912.9612	3.3245	1.4459
47	2014	4	10.5714	70.6020	973.0776	3.3469	0.6429
82	2014	4	7.8000	73.4490	884.6837	3.6010	2.2949
19	2014	4	9.2143	72.3469	974.9306	3.6061	1.0847
20	2014	4	7.1714	72.3673	862.7122	4.4102	2.1296
80	2014	4	7.8000	73.4490	884.6837	3.6010	2.2949
3	2014	4	10.4286	69.6122	957.8643	3.5837	0.7898
52	2014	4	8.0857	70.8469	913.4571	4.0010	3.0143
70	2014	4	10.3429	71.3673	922.0480	3.5765	1.5541
64	2014	4	4.6143	76.9898	780.3633	5.3551	2.2347
48	2014	4	8.9143	64.3367	946.3510	3.2969	0.9061
65	2014	4	8.0857	70.8469	913.4571	4.0010	3.0143
44	2014	4	10.3429	71.3673	922.0480	3.5765	1.5541
75	2014	4	4.6143	76.9898	780.3633	5.3551	2.2347
40	2014	4	8.2000	71.2755	959.2367	3.8745	1.5724
11	2014	4	7.7429	70.7551	887.5551	4.0010	1.9327
35	2014	4	7.6714	72.9286	952.8469	4.7990	0.9724
78	2014	4	8.0286	70.3980	910.5898	3.6071	2.1571
28	2014	4	8.8571	72.8776	941.8561	4.3378	1.4082
39	2014	4	8.0857	70.8469	913.4571	4.0010	3.0143
24	2014	4	9.1429	73.6735	953.6429	3.7949	1.1796
63	2014	4	8.2000	71.2755	959.2367	3.8745	1.5724
62	2014	4	7.0429	75.2347	884.1500	4.2000	1.1827
1	2014	4	7.8000	73.4490	884.6837	3.6010	2.2949
31	2014	5	10.2000	69.5204	851.5908	4.5296	0.9969
79	2014	5	12.4286	68.4490	989.6622	4.0867	0.6561
51	2014	5	11.8143	65.6633	948.9051	5.2418	1.0143
14	2014	5	12.6857	70.6837	905.4296	4.2561	2.2541
67	2014	5	12.2714	68.2143	910.3194	4.5878	3.0857
42	2014	5	11.5714	68.1939	881.8276	4.6561	2.2663
50	2014	5	10.6857	64.2245	909.4612	5.1602	1.5408
43	2014	5	11.5714	68.1939	881.8276	4.6561	2.2663
85	2014	5	11.7143	62.1224	918.3112	4.9643	1.7959
25	2014	5	15.1143	75.8265	988.6153	3.7010	0.8827
69	2014	5	12.7571	65.3163	949.6020	4.7490	1.2776
57	2014	5	11.5143	65.2449	893.9092	5.0765	2.0143
9	2014	5	11.6714	68.3265	860.2133	5.5755	2.2245
72	2014	5	12.8714	69.0510	884.8071	5.5020	1.7337
26	2014	5	14.0143	71.3571	873.6408	6.0173	2.2133
7	2014	5	13.1000	71.5612	865.4847	5.8449	1.6061
83	2014	5	16.9714	67.1735	954.1898	5.2653	0.7571
76	2014	5	10.3571	66.2347	929.8980	4.9112	1.3969
36	2014	5	13.0571	65.5204	938.1520	4.7561	1.4398
81	2014	5	11.8143	65.6633	948.9051	5.2418	1.0143
15	2014	5	10.0571	60.6122	942.5082	3.7704	0.7806

32	2014	5	11.5714	68.1939	881.8276	4.6561	2.2663
73	2014	5	11.8286	67.8469	972.8102	4.2765	0.7500
71	2014	5	13.0571	65.5204	938.1520	4.7561	1.4398
41	2014	5	10.9571	70.6735	881.1163	5.1765	1.2449
10	2014	5	13.0286	67.4694	975.1327	4.7520	0.9439
23	2014	5	9.5714	66.8878	779.0112	6.8163	2.4061
27	2014	5	13.1000	71.5612	865.4847	5.8449	1.6061
60	2014	5	11.8143	65.6633	948.9051	5.2418	1.0143
53	2014	5	11.6714	68.3265	860.2133	5.5755	2.2245
66	2014	5	12.6857	70.6837	905.4296	4.2561	2.2541
59	2014	5	11.5143	65.2449	893.9092	5.0765	2.0143
61	2014	5	11.8286	67.8469	972.8102	4.2765	0.7500
84	2014	5	11.8286	67.8469	972.8102	4.2765	0.7500
38	2014	5	11.5143	65.2449	893.9092	5.0765	2.0143
87	2014	5	13.1000	69.7449	907.4480	4.4357	1.8418
34	2014	5	11.5143	65.2449	893.9092	5.0765	2.0143
29	2014	5	12.7571	65.3163	949.6020	4.7490	1.2776
5	2014	5	11.8857	71.8163	837.3898	6.1990	1.9388
8	2014	5	11.6714	68.3265	860.2133	5.5755	2.2245
12	2014	5	11.8857	71.8163	837.3898	6.1990	1.9388
13	2014	5	16.9714	67.1735	954.1898	5.2653	0.7571
18	2014	5	10.5571	68.5408	974.9510	3.9653	0.7357
33	2014	5	10.6857	64.2245	909.4612	5.1602	1.5408
56	2014	5	15.1143	75.8265	988.6153	3.7010	0.8827
77	2014	5	10.0571	60.6122	942.5082	3.7704	0.7806
54	2014	5	11.8857	71.8163	837.3898	6.1990	1.9388
21	2014	5	10.6857	64.2245	909.4612	5.1602	1.5408
68	2014	5	12.4286	68.4490	989.6622	4.0867	0.6561
74	2014	5	11.8286	67.8469	972.8102	4.2765	0.7500
88	2014	5	11.5714	68.1939	881.8276	4.6561	2.2663
16	2014	5	10.3571	66.2347	929.8980	4.9112	1.3969
30	2014	5	12.6857	70.6837	905.4296	4.2561	2.2541
6	2014	5	12.4286	68.4490	989.6622	4.0867	0.6561
49	2014	5	12.7571	65.3163	949.6020	4.7490	1.2776
22	2014	5	11.5714	68.1939	881.8276	4.6561	2.2663
45	2014	5	12.9429	65.4082	821.9694	6.7673	2.2133
58	2014	5	12.7571	65.3163	949.6020	4.7490	1.2776
37	2014	5	12.4286	68.4490	989.6622	4.0867	0.6561
17	2014	5	12.2714	68.2143	910.3194	4.5878	3.0857
55	2014	5	12.8714	69.0510	884.8071	5.5020	1.7337
46	2014	5	10.3571	66.2347	929.8980	4.9112	1.3969
86	2014	5	12.2714	66.8163	872.2745	4.5908	1.6020
2	2014	5	12.2714	66.8163	872.2745	4.5908	1.6020
4	2014	5	10.6857	64.2245	909.4612	5.1602	1.5408
47	2014	5	16.1857	70.5408	969.2347	4.0143	0.6306
82	2014	5	11.5714	68.1939	881.8276	4.6561	2.2663
19	2014	5	15.6143	73.4082	971.3357	3.6092	0.8459
20	2014	5	11.6714	68.3265	860.2133	5.5755	2.2245
80	2014	5	11.5714	68.1939	881.8276	4.6561	2.2663
3	2014	5	16.9714	67.1735	954.1898	5.2653	0.7571

52	2014	5	12.2714	68.2143	910.3194	4.5878	3.0857
70	2014	5	11.7143	62.1224	918.3112	4.9643	1.7959
64	2014	5	9.5714	66.8878	779.0112	6.8163	2.4061
48	2014	5	10.0571	60.6122	942.5082	3.7704	0.7806
65	2014	5	12.2714	68.2143	910.3194	4.5878	3.0857
44	2014	5	11.7143	62.1224	918.3112	4.9643	1.7959
75	2014	5	9.5714	66.8878	779.0112	6.8163	2.4061
40	2014	5	13.7714	65.8469	955.5837	4.3643	1.4776
11	2014	5	12.8714	69.0510	884.8071	5.5020	1.7337
35	2014	5	11.8143	65.6633	948.9051	5.2418	1.0143
78	2014	5	13.1000	69.7449	907.4480	4.4357	1.8418
28	2014	5	13.0571	65.5204	938.1520	4.7561	1.4398
39	2014	5	12.2714	68.2143	910.3194	4.5878	3.0857
24	2014	5	12.7571	65.3163	949.6020	4.7490	1.2776
63	2014	5	13.7714	65.8469	955.5837	4.3643	1.4776
62	2014	5	10.9571	70.6735	881.1163	5.1765	1.2449
1	2014	5	11.5714	68.1939	881.8276	4.6561	2.2663
31	2014	6	6.6429	72.9694	846.7306	4.2684	1.0918
79	2014	6	5.2429	76.5102	984.3490	1.6806	0.7704
51	2014	6	3.4857	71.9898	943.6806	2.9653	1.5133
14	2014	6	8.0286	80.2959	900.1633	2.7265	2.5612
67	2014	6	4.9857	78.8673	905.0939	2.7735	2.9327
42	2014	6	5.5857	78.2857	876.6878	2.9796	2.5316
50	2014	6	5.0429	73.3673	904.2612	3.2612	1.6949
43	2014	6	5.5857	78.2857	876.6878	2.9796	2.5316
85	2014	6	6.4857	71.3061	912.9816	3.2061	1.6255
25	2014	6	9.5143	76.3776	982.5214	2.7847	1.2990
69	2014	6	5.2857	72.0204	944.1990	2.2265	1.4908
57	2014	6	4.2857	76.1429	888.7500	3.6398	1.9949
9	2014	6	8.1000	75.7755	855.2265	4.5418	2.6296
72	2014	6	11.1286	73.5510	879.4520	4.9204	1.9122
26	2014	6	13.8714	70.4898	868.4776	6.6500	2.6510
7	2014	6	12.4571	71.6224	860.4143	6.1969	1.9765
83	2014	6	15.5429	68.1429	947.6643	5.6367	0.8908
76	2014	6	3.6857	76.1735	924.6969	2.5918	1.5796
36	2014	6	4.6286	71.4898	932.7653	2.8408	1.6735
81	2014	6	3.4857	71.9898	943.6806	2.9653	1.5133
15	2014	6	5.4143	71.5102	937.2929	1.5102	0.9592
32	2014	6	5.5857	78.2857	876.6878	2.9796	2.5316
73	2014	6	6.5429	75.9082	967.4235	1.7755	0.8592
71	2014	6	4.6286	71.4898	932.7653	2.8408	1.6735
41	2014	6	5.4000	77.5102	875.9755	3.5949	1.4020
10	2014	6	4.8571	74.8571	969.7378	3.0653	1.4245
23	2014	6	8.2000	56.1327	775.0980	7.9612	2.5449
27	2014	6	12.4571	71.6224	860.4143	6.1969	1.9765
60	2014	6	3.4857	71.9898	943.6806	2.9653	1.5133
53	2014	6	8.1000	75.7755	855.2265	4.5418	2.6296
66	2014	6	8.0286	80.2959	900.1633	2.7265	2.5612
59	2014	6	4.2857	76.1429	888.7500	3.6398	1.9949
61	2014	6	6.5429	75.9082	967.4235	1.7755	0.8592

84	2014	6	6.5429	75.9082	967.4235	1.7755	0.8592
38	2014	6	4.2857	76.1429	888.7500	3.6398	1.9949
87	2014	6	9.4429	75.2653	901.8673	3.2745	1.9765
34	2014	6	4.2857	76.1429	888.7500	3.6398	1.9949
29	2014	6	5.2857	72.0204	944.1990	2.2265	1.4908
5	2014	6	11.1429	68.7449	832.7010	6.9000	2.3551
8	2014	6	8.1000	75.7755	855.2265	4.5418	2.6296
12	2014	6	11.1429	68.7449	832.7010	6.9000	2.3551
13	2014	6	15.5429	68.1429	947.6643	5.6367	0.8908
18	2014	6	4.4286	77.5714	969.8459	1.3347	0.9888
33	2014	6	5.0429	73.3673	904.2612	3.2612	1.6949
56	2014	6	9.5143	76.3776	982.5214	2.7847	1.2990
77	2014	6	5.4143	71.5102	937.2929	1.5102	0.9592
54	2014	6	11.1429	68.7449	832.7010	6.9000	2.3551
21	2014	6	5.0429	73.3673	904.2612	3.2612	1.6949
68	2014	6	5.2429	76.5102	984.3490	1.6806	0.7704
74	2014	6	6.5429	75.9082	967.4235	1.7755	0.8592
88	2014	6	5.5857	78.2857	876.6878	2.9796	2.5316
16	2014	6	3.6857	76.1735	924.6969	2.5918	1.5796
30	2014	6	8.0286	80.2959	900.1633	2.7265	2.5612
6	2014	6	5.2429	76.5102	984.3490	1.6806	0.7704
49	2014	6	5.2857	72.0204	944.1990	2.2265	1.4908
22	2014	6	5.5857	78.2857	876.6878	2.9796	2.5316
45	2014	6	12.0286	56.4286	817.5806	7.9806	2.4827
58	2014	6	5.2857	72.0204	944.1990	2.2265	1.4908
37	2014	6	5.2429	76.5102	984.3490	1.6806	0.7704
17	2014	6	4.9857	78.8673	905.0939	2.7735	2.9327
55	2014	6	11.1286	73.5510	879.4520	4.9204	1.9122
46	2014	6	3.6857	76.1735	924.6969	2.5918	1.5796
86	2014	6	7.8000	73.8776	867.1245	3.6622	1.6408
2	2014	6	7.8000	73.8776	867.1245	3.6622	1.6408
4	2014	6	5.0429	73.3673	904.2612	3.2612	1.6949
47	2014	6	14.6143	73.2245	962.5929	3.5622	0.6582
82	2014	6	5.5857	78.2857	876.6878	2.9796	2.5316
19	2014	6	12.3857	75.0000	965.1133	2.3173	1.0653
20	2014	6	8.1000	75.7755	855.2265	4.5418	2.6296
80	2014	6	5.5857	78.2857	876.6878	2.9796	2.5316
3	2014	6	15.5429	68.1429	947.6643	5.6367	0.8908
52	2014	6	4.9857	78.8673	905.0939	2.7735	2.9327
70	2014	6	6.4857	71.3061	912.9816	3.2061	1.6255
64	2014	6	8.2000	56.1327	775.0980	7.9612	2.5449
48	2014	6	5.4143	71.5102	937.2929	1.5102	0.9592
65	2014	6	4.9857	78.8673	905.0939	2.7735	2.9327
44	2014	6	6.4857	71.3061	912.9816	3.2061	1.6255
75	2014	6	8.2000	56.1327	775.0980	7.9612	2.5449
40	2014	6	4.5429	74.0612	950.2429	3.0255	1.7755
11	2014	6	11.1286	73.5510	879.4520	4.9204	1.9122
35	2014	6	3.4857	71.9898	943.6806	2.9653	1.5133
78	2014	6	9.4429	75.2653	901.8673	3.2745	1.9765
28	2014	6	4.6286	71.4898	932.7653	2.8408	1.6735

39	2014	6	4.9857	78.8673	905.0939	2.7735	2.9327
24	2014	6	5.2857	72.0204	944.1990	2.2265	1.4908
63	2014	6	4.5429	74.0612	950.2429	3.0255	1.7755
62	2014	6	5.4000	77.5102	875.9755	3.5949	1.4020
1	2014	6	5.5857	78.2857	876.6878	2.9796	2.5316
31	2014	7	-1.0143	79.3469	845.5316	2.8010	1.1255
79	2014	7	2.4714	77.4286	986.4878	0.6551	1.0612
51	2014	7	-0.5714	80.1735	944.7255	1.1255	1.8112
14	2014	7	-0.7286	79.6633	899.3490	1.8408	2.6143
67	2014	7	-1.2286	82.1531	904.8429	1.2316	2.8306
42	2014	7	-1.5714	83.3571	875.8735	1.4357	2.9500
50	2014	7	0.6286	76.6224	904.2561	0.9837	2.1041
43	2014	7	-1.5714	83.3571	875.8735	1.4357	2.9500
85	2014	7	1.5286	76.9082	913.1276	1.1724	1.5796
25	2014	7	3.9000	69.6939	983.5653	1.5296	1.9520
69	2014	7	1.4714	74.4796	945.1306	0.6347	1.7582
57	2014	7	-1.0714	85.6429	888.4480	1.6347	1.7949
9	2014	7	-1.4571	80.6735	853.8265	3.1255	3.1673
72	2014	7	0.4857	72.7143	878.2459	3.4051	2.5286
26	2014	7	2.1143	71.1531	866.9867	4.9184	2.7276
7	2014	7	1.0286	72.5816	858.8908	4.6398	2.4480
83	2014	7	6.2714	63.7959	947.2235	3.6490	1.2031
76	2014	7	0.2857	80.7653	925.2510	0.7990	1.7286
36	2014	7	0.0857	76.2449	933.3204	1.2837	1.9459
81	2014	7	-0.5714	80.1735	944.7255	1.1255	1.8112
15	2014	7	2.4571	70.2143	937.9480	0.7153	1.2939
32	2014	7	-1.5714	83.3571	875.8735	1.4357	2.9500
73	2014	7	3.3429	75.5306	968.7908	0.4959	1.0796
71	2014	7	0.0857	76.2449	933.3204	1.2837	1.9459
41	2014	7	-0.8429	83.0714	875.3480	1.7296	1.7224
10	2014	7	0.7143	79.8776	971.2643	1.4673	1.8102
23	2014	7	-2.0571	63.8571	772.5796	5.9082	2.5735
27	2014	7	1.0286	72.5816	858.8908	4.6398	2.4480
60	2014	7	-0.5714	80.1735	944.7255	1.1255	1.8112
53	2014	7	-1.4571	80.6735	853.8265	3.1255	3.1673
66	2014	7	-0.7286	79.6633	899.3490	1.8408	2.6143
59	2014	7	-1.0714	85.6429	888.4480	1.6347	1.7949
61	2014	7	3.3429	75.5306	968.7908	0.4959	1.0796
84	2014	7	3.3429	75.5306	968.7908	0.4959	1.0796
38	2014	7	-1.0714	85.6429	888.4480	1.6347	1.7949
87	2014	7	1.3714	72.9490	901.2020	2.2010	2.8459
34	2014	7	-1.0714	85.6429	888.4480	1.6347	1.7949
29	2014	7	1.4714	74.4796	945.1306	0.6347	1.7582
5	2014	7	-1.3857	72.9694	830.8888	4.9612	2.4918
8	2014	7	-1.4571	80.6735	853.8265	3.1255	3.1673
12	2014	7	-1.3857	72.9694	830.8888	4.9612	2.4918
13	2014	7	6.2714	63.7959	947.2235	3.6490	1.2031
18	2014	7	1.5429	78.8571	971.6398	0.4316	1.4490
33	2014	7	0.6286	76.6224	904.2561	0.9837	2.1041
56	2014	7	3.9000	69.6939	983.5653	1.5296	1.9520

77	2014	7	2.4571	70.2143	937.9480	0.7153	1.2939
54	2014	7	-1.3857	72.9694	830.8888	4.9612	2.4918
21	2014	7	0.6286	76.6224	904.2561	0.9837	2.1041
68	2014	7	2.4714	77.4286	986.4878	0.6551	1.0612
74	2014	7	3.3429	75.5306	968.7908	0.4959	1.0796
88	2014	7	-1.5714	83.3571	875.8735	1.4357	2.9500
16	2014	7	0.2857	80.7653	925.2510	0.7990	1.7286
30	2014	7	-0.7286	79.6633	899.3490	1.8408	2.6143
6	2014	7	2.4714	77.4286	986.4878	0.6551	1.0612
49	2014	7	1.4714	74.4796	945.1306	0.6347	1.7582
22	2014	7	-1.5714	83.3571	875.8735	1.4357	2.9500
45	2014	7	0.7714	62.4082	815.5408	6.1490	2.6847
58	2014	7	1.4714	74.4796	945.1306	0.6347	1.7582
37	2014	7	2.4714	77.4286	986.4878	0.6551	1.0612
17	2014	7	-1.2286	82.1531	904.8429	1.2316	2.8306
55	2014	7	0.4857	72.7143	878.2459	3.4051	2.5286
46	2014	7	0.2857	80.7653	925.2510	0.7990	1.7286
86	2014	7	0.0286	80.5510	866.1173	2.3500	1.4633
2	2014	7	0.0286	80.5510	866.1173	2.3500	1.4633
4	2014	7	0.6286	76.6224	904.2561	0.9837	2.1041
47	2014	7	5.8286	66.0204	962.5837	2.6520	0.8990
82	2014	7	-1.5714	83.3571	875.8735	1.4357	2.9500
19	2014	7	4.0857	67.9490	965.2245	1.3490	1.7714
20	2014	7	-1.4571	80.6735	853.8265	3.1255	3.1673
80	2014	7	-1.5714	83.3571	875.8735	1.4357	2.9500
3	2014	7	6.2714	63.7959	947.2235	3.6490	1.2031
52	2014	7	-1.2286	82.1531	904.8429	1.2316	2.8306
70	2014	7	1.5286	76.9082	913.1276	1.1724	1.5796
64	2014	7	-2.0571	63.8571	772.5796	5.9082	2.5735
48	2014	7	2.4571	70.2143	937.9480	0.7153	1.2939
65	2014	7	-1.2286	82.1531	904.8429	1.2316	2.8306
44	2014	7	1.5286	76.9082	913.1276	1.1724	1.5796
75	2014	7	-2.0571	63.8571	772.5796	5.9082	2.5735
40	2014	7	-0.3143	81.2959	950.9673	1.4867	2.1367
11	2014	7	0.4857	72.7143	878.2459	3.4051	2.5286
35	2014	7	-0.5714	80.1735	944.7255	1.1255	1.8112
78	2014	7	1.3714	72.9490	901.2020	2.2010	2.8459
28	2014	7	0.0857	76.2449	933.3204	1.2837	1.9459
39	2014	7	-1.2286	82.1531	904.8429	1.2316	2.8306
24	2014	7	1.4714	74.4796	945.1306	0.6347	1.7582
63	2014	7	-0.3143	81.2959	950.9673	1.4867	2.1367
62	2014	7	-0.8429	83.0714	875.3480	1.7296	1.7224
1	2014	7	-1.5714	83.3571	875.8735	1.4357	2.9500
31	2014	8	3.4000	83.8265	848.4469	1.2735	0.9898
79	2014	8	5.6286	75.7857	991.3265	0.7622	1.0031
51	2014	8	3.7000	82.3980	949.0449	0.6459	1.5276
14	2014	8	4.1571	80.6224	902.8184	1.1714	2.3153
67	2014	8	3.7000	82.5408	908.5398	0.4316	2.6643
42	2014	8	4.1286	85.0204	879.1031	0.8898	2.6653
50	2014	8	4.9286	73.9184	907.9765	0.3163	2.0755

43	2014	8	4.1286	85.0204	879.1031	0.8898	2.6653
85	2014	8	5.5286	76.0408	917.0929	0.5296	1.4857
25	2014	8	7.2857	70.1633	988.6806	0.9867	1.5663
69	2014	8	4.9143	74.0204	949.5388	0.3490	1.4082
57	2014	8	4.2857	85.8980	891.9796	0.7398	1.4714
9	2014	8	4.3857	85.3673	856.6694	1.3551	2.7071
72	2014	8	5.5571	77.5510	881.5112	1.3867	2.5378
26	2014	8	6.4714	81.4286	869.8847	2.2806	2.1367
7	2014	8	5.6143	81.9082	861.7092	2.2633	2.0633
83	2014	8	9.8000	66.5510	951.9276	1.2255	1.1429
76	2014	8	4.2857	77.5102	929.2969	0.5857	1.5929
36	2014	8	4.2857	77.6224	937.5908	0.6173	1.7041
81	2014	8	3.7000	82.3980	949.0449	0.6459	1.5276
15	2014	8	5.8000	63.8571	941.7296	0.8265	1.0878
32	2014	8	4.1286	85.0204	879.1031	0.8898	2.6653
73	2014	8	6.6000	72.1531	973.4704	0.4837	1.0429
71	2014	8	4.2857	77.6224	937.5908	0.6173	1.7041
41	2014	8	5.0429	83.8163	878.6949	0.7255	1.6837
10	2014	8	5.0000	82.4286	975.9633	0.8337	1.3582
23	2014	8	2.0286	83.0612	773.8633	3.3286	2.3143
27	2014	8	5.6143	81.9082	861.7092	2.2633	2.0633
60	2014	8	3.7000	82.3980	949.0449	0.6459	1.5276
53	2014	8	4.3857	85.3673	856.6694	1.3551	2.7071
66	2014	8	4.1571	80.6224	902.8184	1.1714	2.3153
59	2014	8	4.2857	85.8980	891.9796	0.7398	1.4714
61	2014	8	6.6000	72.1531	973.4704	0.4837	1.0429
84	2014	8	6.6000	72.1531	973.4704	0.4837	1.0429
38	2014	8	4.2857	85.8980	891.9796	0.7398	1.4714
87	2014	8	6.4143	73.8163	904.9000	1.2020	2.7367
34	2014	8	4.2857	85.8980	891.9796	0.7398	1.4714
29	2014	8	4.9143	74.0204	949.5388	0.3490	1.4082
5	2014	8	4.0143	88.4694	833.2286	2.0031	1.8765
8	2014	8	4.3857	85.3673	856.6694	1.3551	2.7071
12	2014	8	4.0143	88.4694	833.2286	2.0031	1.8765
13	2014	8	9.8000	66.5510	951.9276	1.2255	1.1429
18	2014	8	5.2714	75.7551	976.1582	0.6245	1.4153
33	2014	8	4.9286	73.9184	907.9765	0.3163	2.0755
56	2014	8	7.2857	70.1633	988.6806	0.9867	1.5663
77	2014	8	5.8000	63.8571	941.7296	0.8265	1.0878
54	2014	8	4.0143	88.4694	833.2286	2.0031	1.8765
21	2014	8	4.9286	73.9184	907.9765	0.3163	2.0755
68	2014	8	5.6286	75.7857	991.3265	0.7622	1.0031
74	2014	8	6.6000	72.1531	973.4704	0.4837	1.0429
88	2014	8	4.1286	85.0204	879.1031	0.8898	2.6653
16	2014	8	4.2857	77.5102	929.2969	0.5857	1.5929
30	2014	8	4.1571	80.6224	902.8184	1.1714	2.3153
6	2014	8	5.6286	75.7857	991.3265	0.7622	1.0031
49	2014	8	4.9143	74.0204	949.5388	0.3490	1.4082
22	2014	8	4.1286	85.0204	879.1031	0.8898	2.6653
45	2014	8	5.2000	80.2653	817.4041	3.3398	2.3704

58	2014	8	4.9143	74.0204	949.5388	0.3490	1.4082
37	2014	8	5.6286	75.7857	991.3265	0.7622	1.0031
17	2014	8	3.7000	82.5408	908.5398	0.4316	2.6643
55	2014	8	5.5571	77.5510	881.5112	1.3867	2.5378
46	2014	8	4.2857	77.5102	929.2969	0.5857	1.5929
86	2014	8	5.3286	84.5510	869.2867	1.0929	1.1378
2	2014	8	5.3286	84.5510	869.2867	1.0929	1.1378
4	2014	8	4.9286	73.9184	907.9765	0.3163	2.0755
47	2014	8	9.3143	66.7449	967.7194	1.2214	0.8949
82	2014	8	4.1286	85.0204	879.1031	0.8898	2.6653
19	2014	8	7.9143	66.7755	970.0908	0.8418	1.7918
20	2014	8	4.3857	85.3673	856.6694	1.3551	2.7071
80	2014	8	4.1286	85.0204	879.1031	0.8898	2.6653
3	2014	8	9.8000	66.5510	951.9276	1.2255	1.1429
52	2014	8	3.7000	82.5408	908.5398	0.4316	2.6643
70	2014	8	5.5286	76.0408	917.0929	0.5296	1.4857
64	2014	8	2.0286	83.0612	773.8633	3.3286	2.3143
48	2014	8	5.8000	63.8571	941.7296	0.8265	1.0878
65	2014	8	3.7000	82.5408	908.5398	0.4316	2.6643
44	2014	8	5.5286	76.0408	917.0929	0.5296	1.4857
75	2014	8	2.0286	83.0612	773.8633	3.3286	2.3143
40	2014	8	4.6000	84.3980	955.2592	0.5520	1.9520
11	2014	8	5.5571	77.5510	881.5112	1.3867	2.5378
35	2014	8	3.7000	82.3980	949.0449	0.6459	1.5276
78	2014	8	6.4143	73.8163	904.9000	1.2020	2.7367
28	2014	8	4.2857	77.6224	937.5908	0.6173	1.7041
39	2014	8	3.7000	82.5408	908.5398	0.4316	2.6643
24	2014	8	4.9143	74.0204	949.5388	0.3490	1.4082
63	2014	8	4.6000	84.3980	955.2592	0.5520	1.9520
62	2014	8	5.0429	83.8163	878.6949	0.7255	1.6837
1	2014	8	4.1286	85.0204	879.1031	0.8898	2.6653
31	2014	9	6.3286	83.8878	849.9990	1.5357	0.8724
79	2014	9	8.9857	85.0612	990.6378	0.7306	0.6398
51	2014	9	6.9000	87.0714	949.1133	0.8041	1.2429
14	2014	9	8.0714	87.7449	903.8143	1.1102	2.1041
67	2014	9	6.4429	87.8571	909.2857	0.4929	2.4684
42	2014	9	6.4571	88.2449	880.2082	1.1786	2.3041
50	2014	9	7.8429	77.9286	908.7122	0.6980	1.8408
43	2014	9	6.4571	88.2449	880.2082	1.1786	2.3041
85	2014	9	8.6286	80.5714	917.7786	0.9969	1.2582
25	2014	9	10.4000	83.4898	988.5653	1.1694	0.7459
69	2014	9	8.3857	83.2551	949.6663	0.3653	0.9643
57	2014	9	6.2857	86.8980	892.8714	0.9673	1.3827
9	2014	9	6.9714	87.0306	858.1602	1.1929	2.1388
72	2014	9	9.5143	83.8265	882.7765	1.4143	2.1602
26	2014	9	13.5143	86.4694	871.2653	2.7122	1.8888
7	2014	9	11.3286	86.2449	863.1673	2.2204	1.6337
83	2014	9	15.0571	76.2347	952.5837	1.4061	0.9500
76	2014	9	7.3000	82.0612	929.6898	0.7041	1.5245
36	2014	9	7.2571	84.6327	937.8786	0.6082	1.3163

81	2014	9	6.9000	87.0714	949.1133	0.8041	1.2429
15	2014	9	9.1857	71.1327	941.6306	0.6102	0.7643
32	2014	9	6.4571	88.2449	880.2082	1.1786	2.3041
73	2014	9	9.8143	80.0306	973.3306	0.5051	0.8061
71	2014	9	7.2571	84.6327	937.8786	0.6082	1.3163
41	2014	9	7.0286	83.3673	879.7888	1.1153	1.4663
10	2014	9	8.1143	88.4286	975.6327	1.0816	0.8694
23	2014	9	7.4429	82.2551	775.9857	5.2459	2.0694
27	2014	9	11.3286	86.2449	863.1673	2.2204	1.6337
60	2014	9	6.9000	87.0714	949.1133	0.8041	1.2429
53	2014	9	6.9714	87.0306	858.1602	1.1929	2.1388
66	2014	9	8.0714	87.7449	903.8143	1.1102	2.1041
59	2014	9	6.2857	86.8980	892.8714	0.9673	1.3827
61	2014	9	9.8143	80.0306	973.3306	0.5051	0.8061
84	2014	9	9.8143	80.0306	973.3306	0.5051	0.8061
38	2014	9	6.2857	86.8980	892.8714	0.9673	1.3827
87	2014	9	9.2571	81.2143	905.8388	1.2561	2.1398
34	2014	9	6.2857	86.8980	892.8714	0.9673	1.3827
29	2014	9	8.3857	83.2551	949.6663	0.3653	0.9643
5	2014	9	8.8286	91.6429	834.9469	2.3582	1.7020
8	2014	9	6.9714	87.0306	858.1602	1.1929	2.1388
12	2014	9	8.8286	91.6429	834.9469	2.3582	1.7020
13	2014	9	15.0571	76.2347	952.5837	1.4061	0.9500
18	2014	9	8.4000	83.4592	975.6939	0.6949	1.0398
33	2014	9	7.8429	77.9286	908.7122	0.6980	1.8408
56	2014	9	10.4000	83.4898	988.5653	1.1694	0.7459
77	2014	9	9.1857	71.1327	941.6306	0.6102	0.7643
54	2014	9	8.8286	91.6429	834.9469	2.3582	1.7020
21	2014	9	7.8429	77.9286	908.7122	0.6980	1.8408
68	2014	9	8.9857	85.0612	990.6378	0.7306	0.6398
74	2014	9	9.8143	80.0306	973.3306	0.5051	0.8061
88	2014	9	6.4571	88.2449	880.2082	1.1786	2.3041
16	2014	9	7.3000	82.0612	929.6898	0.7041	1.5245
30	2014	9	8.0714	87.7449	903.8143	1.1102	2.1041
6	2014	9	8.9857	85.0612	990.6378	0.7306	0.6398
49	2014	9	8.3857	83.2551	949.6663	0.3653	0.9643
22	2014	9	6.4571	88.2449	880.2082	1.1786	2.3041
45	2014	9	13.0429	80.3265	819.1908	4.7847	2.2122
58	2014	9	8.3857	83.2551	949.6663	0.3653	0.9643
37	2014	9	8.9857	85.0612	990.6378	0.7306	0.6398
17	2014	9	6.4429	87.8571	909.2857	0.4929	2.4684
55	2014	9	9.5143	83.8265	882.7765	1.4143	2.1602
46	2014	9	7.3000	82.0612	929.6898	0.7041	1.5245
86	2014	9	7.7286	83.8980	870.6265	1.1449	1.1122
2	2014	9	7.7286	83.8980	870.6265	1.1449	1.1122
4	2014	9	7.8429	77.9286	908.7122	0.6980	1.8408
47	2014	9	14.0857	78.7653	968.1286	1.1071	0.6398
82	2014	9	6.4571	88.2449	880.2082	1.1786	2.3041
19	2014	9	11.6714	78.0306	970.4490	1.1286	1.2306
20	2014	9	6.9714	87.0306	858.1602	1.1929	2.1388

80	2014	9	6.4571	88.2449	880.2082	1.1786	2.3041
3	2014	9	15.0571	76.2347	952.5837	1.4061	0.9500
52	2014	9	6.4429	87.8571	909.2857	0.4929	2.4684
70	2014	9	8.6286	80.5714	917.7786	0.9969	1.2582
64	2014	9	7.4429	82.2551	775.9857	5.2459	2.0694
48	2014	9	9.1857	71.1327	941.6306	0.6102	0.7643
65	2014	9	6.4429	87.8571	909.2857	0.4929	2.4684
44	2014	9	8.6286	80.5714	917.7786	0.9969	1.2582
75	2014	9	7.4429	82.2551	775.9857	5.2459	2.0694
40	2014	9	7.2571	88.1633	955.3694	0.8102	1.4918
11	2014	9	9.5143	83.8265	882.7765	1.4143	2.1602
35	2014	9	6.9000	87.0714	949.1133	0.8041	1.2429
78	2014	9	9.2571	81.2143	905.8388	1.2561	2.1398
28	2014	9	7.2571	84.6327	937.8786	0.6082	1.3163
39	2014	9	6.4429	87.8571	909.2857	0.4929	2.4684
24	2014	9	8.3857	83.2551	949.6663	0.3653	0.9643
63	2014	9	7.2571	88.1633	955.3694	0.8102	1.4918
62	2014	9	7.0286	83.3673	879.7888	1.1153	1.4663
1	2014	9	6.4571	88.2449	880.2082	1.1786	2.3041
31	2014	10	5.5571	86.2347	849.3235	0.8816	0.8602
79	2014	10	9.0286	87.8776	988.6173	0.2765	0.6388
51	2014	10	6.9286	89.2653	947.4469	0.5153	1.4092
14	2014	10	6.5571	92.6531	902.4745	0.7286	1.8582
67	2014	10	5.9429	92.8061	908.0010	0.1796	2.4857
42	2014	10	5.5143	92.5306	879.2582	0.4980	2.4653
50	2014	10	7.2000	81.5714	907.5265	0.4367	1.6255
43	2014	10	5.5143	92.5306	879.2582	0.4980	2.4653
85	2014	10	7.8857	84.5510	916.5582	0.5837	1.1786
25	2014	10	10.3571	88.6429	986.4173	0.6837	0.6776
69	2014	10	8.2571	86.1735	947.9827	0.3184	1.0867
57	2014	10	5.6143	90.6939	891.8541	0.3867	1.4102
9	2014	10	6.1000	90.5204	857.3449	0.4143	2.4102
72	2014	10	7.8429	87.7041	881.6173	0.8684	2.2204
26	2014	10	10.3429	85.3265	869.9888	3.2776	2.2541
7	2014	10	9.5714	86.1020	862.0847	2.4173	1.9704
83	2014	10	13.6714	77.0408	950.4061	1.5694	1.0398
76	2014	10	6.9000	85.3061	928.2439	0.3102	1.6010
36	2014	10	7.1429	88.4898	936.2990	0.4490	1.3663
81	2014	10	6.9286	89.2653	947.4469	0.5153	1.4092
15	2014	10	8.7000	73.4184	940.3908	0.1918	0.8602
32	2014	10	5.5143	92.5306	879.2582	0.4980	2.4653
73	2014	10	9.6714	82.3571	971.4153	0.2286	0.7959
71	2014	10	7.1429	88.4898	936.2990	0.4490	1.3663
41	2014	10	6.0429	87.8673	878.8949	0.6367	1.3898
10	2014	10	7.8143	91.4796	973.7184	0.4347	0.8888
23	2014	10	8.4000	74.9184	775.8031	7.5378	2.3816
27	2014	10	9.5714	86.1020	862.0847	2.4173	1.9704
60	2014	10	6.9286	89.2653	947.4469	0.5153	1.4092
53	2014	10	6.1000	90.5204	857.3449	0.4143	2.4102
66	2014	10	6.5571	92.6531	902.4745	0.7286	1.8582

59	2014	10	5.6143	90.6939	891.8541	0.3867	1.4102
61	2014	10	9.6714	82.3571	971.4153	0.2286	0.7959
84	2014	10	9.6714	82.3571	971.4153	0.2286	0.7959
38	2014	10	5.6143	90.6939	891.8541	0.3867	1.4102
87	2014	10	8.2143	85.5000	904.5735	0.6735	2.2745
34	2014	10	5.6143	90.6939	891.8541	0.3867	1.4102
29	2014	10	8.2571	86.1735	947.9827	0.3184	1.0867
5	2014	10	7.3571	91.4184	834.1867	2.8735	2.1531
8	2014	10	6.1000	90.5204	857.3449	0.4143	2.4102
12	2014	10	7.3571	91.4184	834.1867	2.8735	2.1531
13	2014	10	13.6714	77.0408	950.4061	1.5694	1.0398
18	2014	10	8.0571	87.4184	973.8418	0.2786	1.0010
33	2014	10	7.2000	81.5714	907.5265	0.4367	1.6255
56	2014	10	10.3571	88.6429	986.4173	0.6837	0.6776
77	2014	10	8.7000	73.4184	940.3908	0.1918	0.8602
54	2014	10	7.3571	91.4184	834.1867	2.8735	2.1531
21	2014	10	7.2000	81.5714	907.5265	0.4367	1.6255
68	2014	10	9.0286	87.8776	988.6173	0.2765	0.6388
74	2014	10	9.6714	82.3571	971.4153	0.2286	0.7959
88	2014	10	5.5143	92.5306	879.2582	0.4980	2.4653
16	2014	10	6.9000	85.3061	928.2439	0.3102	1.6010
30	2014	10	6.5571	92.6531	902.4745	0.7286	1.8582
6	2014	10	9.0286	87.8776	988.6173	0.2765	0.6388
49	2014	10	8.2571	86.1735	947.9827	0.3184	1.0867
22	2014	10	5.5143	92.5306	879.2582	0.4980	2.4653
45	2014	10	12.7571	67.7755	818.4898	7.0745	3.0541
58	2014	10	8.2571	86.1735	947.9827	0.3184	1.0867
37	2014	10	9.0286	87.8776	988.6173	0.2765	0.6388
17	2014	10	5.9429	92.8061	908.0010	0.1796	2.4857
55	2014	10	7.8429	87.7041	881.6173	0.8684	2.2204
46	2014	10	6.9000	85.3061	928.2439	0.3102	1.6010
86	2014	10	7.0857	86.4184	869.8286	0.6867	1.1276
2	2014	10	7.0857	86.4184	869.8286	0.6867	1.1276
4	2014	10	7.2000	81.5714	907.5265	0.4367	1.6255
47	2014	10	12.7286	80.0204	965.8714	0.8265	0.6714
82	2014	10	5.5143	92.5306	879.2582	0.4980	2.4653
19	2014	10	10.9714	82.9592	968.3051	0.8837	1.2061
20	2014	10	6.1000	90.5204	857.3449	0.4143	2.4102
80	2014	10	5.5143	92.5306	879.2582	0.4980	2.4653
3	2014	10	13.6714	77.0408	950.4061	1.5694	1.0398
52	2014	10	5.9429	92.8061	908.0010	0.1796	2.4857
70	2014	10	7.8857	84.5510	916.5582	0.5837	1.1786
64	2014	10	8.4000	74.9184	775.8031	7.5378	2.3816
48	2014	10	8.7000	73.4184	940.3908	0.1918	0.8602
65	2014	10	5.9429	92.8061	908.0010	0.1796	2.4857
44	2014	10	7.8857	84.5510	916.5582	0.5837	1.1786
75	2014	10	8.4000	74.9184	775.8031	7.5378	2.3816
40	2014	10	6.8571	93.0612	953.6051	0.5949	1.5347
11	2014	10	7.8429	87.7041	881.6173	0.8684	2.2204
35	2014	10	6.9286	89.2653	947.4469	0.5153	1.4092

78	2014	10	8.2143	85.5000	904.5735	0.6735	2.2745
28	2014	10	7.1429	88.4898	936.2990	0.4490	1.3663
39	2014	10	5.9429	92.8061	908.0010	0.1796	2.4857
24	2014	10	8.2571	86.1735	947.9827	0.3184	1.0867
63	2014	10	6.8571	93.0612	953.6051	0.5949	1.5347
62	2014	10	6.0429	87.8673	878.8949	0.6367	1.3898
1	2014	10	5.5143	92.5306	879.2582	0.4980	2.4653
31	2014	11	9.0714	85.8878	849.5010	0.6571	0.9551
79	2014	11	12.3857	81.4898	988.1490	0.8031	0.7765
51	2014	11	11.1000	83.4592	947.0878	0.9867	1.5367
14	2014	11	10.2000	90.3163	902.4806	0.9092	1.7786
67	2014	11	10.3714	89.9796	907.9459	0.3724	2.7265
42	2014	11	9.5429	91.0306	879.3867	0.3714	2.5459
50	2014	11	10.9429	79.1327	907.5041	0.7929	1.5337
43	2014	11	9.5429	91.0306	879.3867	0.3714	2.5459
85	2014	11	11.9429	81.0204	916.4908	1.2194	1.4337
25	2014	11	13.9571	83.0102	985.9878	0.5612	0.9112
69	2014	11	12.4429	80.1020	947.6796	1.1980	1.3541
57	2014	11	10.2571	87.8367	891.9163	0.7429	1.3867
9	2014	11	8.9286	90.3980	857.5112	0.3816	2.5184
72	2014	11	10.5143	86.4796	881.7776	0.8796	2.3082
26	2014	11	11.8286	85.2653	870.1908	2.5755	2.4276
7	2014	11	11.5429	83.6122	862.2724	2.4480	2.2122
83	2014	11	15.0143	73.0306	950.4755	1.5112	1.0827
76	2014	11	10.9571	81.1633	928.0714	0.7122	1.5102
36	2014	11	11.3000	84.7551	936.0541	0.7867	1.5020
81	2014	11	11.1000	83.4592	947.0878	0.9867	1.5367
15	2014	11	11.5857	69.9082	940.5867	0.5541	1.0408
32	2014	11	9.5429	91.0306	879.3867	0.3714	2.5459
73	2014	11	12.7000	76.6122	970.9796	0.9153	0.8673
71	2014	11	11.3000	84.7551	936.0541	0.7867	1.5020
41	2014	11	10.3714	86.9796	879.0265	0.4612	1.3031
10	2014	11	12.0571	86.7959	973.3031	0.7357	1.0551
23	2014	11	8.0000	69.8980	776.1857	7.9265	2.8582
27	2014	11	11.5429	83.6122	862.2724	2.4480	2.2122
60	2014	11	11.1000	83.4592	947.0878	0.9867	1.5367
53	2014	11	8.9286	90.3980	857.5112	0.3816	2.5184
66	2014	11	10.2000	90.3163	902.4806	0.9092	1.7786
59	2014	11	10.2571	87.8367	891.9163	0.7429	1.3867
61	2014	11	12.7000	76.6122	970.9796	0.9153	0.8673
84	2014	11	12.7000	76.6122	970.9796	0.9153	0.8673
38	2014	11	10.2571	87.8367	891.9163	0.7429	1.3867
87	2014	11	11.3000	83.8776	904.6520	0.1561	2.2408
34	2014	11	10.2571	87.8367	891.9163	0.7429	1.3867
29	2014	11	12.4429	80.1020	947.6796	1.1980	1.3541
5	2014	11	9.9143	88.9082	834.4316	2.5378	2.5112
8	2014	11	8.9286	90.3980	857.5112	0.3816	2.5184
12	2014	11	9.9143	88.9082	834.4316	2.5378	2.5112
13	2014	11	15.0143	73.0306	950.4755	1.5112	1.0827
18	2014	11	11.0143	81.3673	973.5092	0.8857	1.1031

33	2014	11	10.9429	79.1327	907.5041	0.7929	1.5337
56	2014	11	13.9571	83.0102	985.9878	0.5612	0.9112
77	2014	11	11.5857	69.9082	940.5867	0.5541	1.0408
54	2014	11	9.9143	88.9082	834.4316	2.5378	2.5112
21	2014	11	10.9429	79.1327	907.5041	0.7929	1.5337
68	2014	11	12.3857	81.4898	988.1490	0.8031	0.7765
74	2014	11	12.7000	76.6122	970.9796	0.9153	0.8673
88	2014	11	9.5429	91.0306	879.3867	0.3714	2.5459
16	2014	11	10.9571	81.1633	928.0714	0.7122	1.5102
30	2014	11	10.2000	90.3163	902.4806	0.9092	1.7786
6	2014	11	12.3857	81.4898	988.1490	0.8031	0.7765
49	2014	11	12.4429	80.1020	947.6796	1.1980	1.3541
22	2014	11	9.5429	91.0306	879.3867	0.3714	2.5459
45	2014	11	11.7143	64.0204	818.7418	6.5653	3.4510
58	2014	11	12.4429	80.1020	947.6796	1.1980	1.3541
37	2014	11	12.3857	81.4898	988.1490	0.8031	0.7765
17	2014	11	10.3714	89.9796	907.9459	0.3724	2.7265
55	2014	11	10.5143	86.4796	881.7776	0.8796	2.3082
46	2014	11	10.9571	81.1633	928.0714	0.7122	1.5102
86	2014	11	10.4714	85.8061	869.9786	0.8776	1.2235
2	2014	11	10.4714	85.8061	869.9786	0.8776	1.2235
4	2014	11	10.9429	79.1327	907.5041	0.7929	1.5337
47	2014	11	15.0000	74.5612	965.8694	0.6735	0.7827
82	2014	11	9.5429	91.0306	879.3867	0.3714	2.5459
19	2014	11	13.5857	79.4286	968.1306	0.8337	1.3194
20	2014	11	8.9286	90.3980	857.5112	0.3816	2.5184
80	2014	11	9.5429	91.0306	879.3867	0.3714	2.5459
3	2014	11	15.0143	73.0306	950.4755	1.5112	1.0827
52	2014	11	10.3714	89.9796	907.9459	0.3724	2.7265
70	2014	11	11.9429	81.0204	916.4908	1.2194	1.4337
64	2014	11	8.0000	69.8980	776.1857	7.9265	2.8582
48	2014	11	11.5857	69.9082	940.5867	0.5541	1.0408
65	2014	11	10.3714	89.9796	907.9459	0.3724	2.7265
44	2014	11	11.9429	81.0204	916.4908	1.2194	1.4337
75	2014	11	8.0000	69.8980	776.1857	7.9265	2.8582
40	2014	11	11.4286	90.4184	953.2908	0.6765	1.5735
11	2014	11	10.5143	86.4796	881.7776	0.8796	2.3082
35	2014	11	11.1000	83.4592	947.0878	0.9867	1.5367
78	2014	11	11.3000	83.8776	904.6520	0.1561	2.2408
28	2014	11	11.3000	84.7551	936.0541	0.7867	1.5020
39	2014	11	10.3714	89.9796	907.9459	0.3724	2.7265
24	2014	11	12.4429	80.1020	947.6796	1.1980	1.3541
63	2014	11	11.4286	90.4184	953.2908	0.6765	1.5735
62	2014	11	10.3714	86.9796	879.0265	0.4612	1.3031
1	2014	11	9.5429	91.0306	879.3867	0.3714	2.5459
31	2014	12	10.7143	78.3980	850.0663	2.7531	1.1153
79	2014	12	14.4714	78.9796	987.1265	1.5622	0.7582
51	2014	12	13.3857	76.7551	946.5214	2.5163	1.4286
14	2014	12	11.8429	87.2347	903.0357	1.8112	2.3184
67	2014	12	12.3714	84.0612	908.1102	1.6143	3.0602

42	2014	12	12.0571	84.6837	879.7571	1.9531	2.5806
50	2014	12	13.4714	74.2959	907.3408	2.8531	1.6643
43	2014	12	12.0571	84.6837	879.7571	1.9531	2.5806
85	2014	12	14.2714	74.7857	916.1316	3.1122	1.6296
25	2014	12	16.5143	77.8571	985.4592	1.4296	1.0071
69	2014	12	14.4286	75.0000	947.1806	2.3173	1.5000
57	2014	12	12.6000	79.0102	891.9388	2.4837	1.6684
9	2014	12	11.7429	85.3163	858.1714	2.1908	2.5337
72	2014	12	12.9000	82.3367	882.3918	2.4112	2.2102
26	2014	12	14.4714	80.9592	871.1684	2.9551	2.5122
7	2014	12	13.7714	77.9490	863.1357	3.6276	2.2367
83	2014	12	17.6286	71.7653	950.9541	2.2173	0.9643
76	2014	12	13.3143	75.8980	927.7184	2.2959	1.4602
36	2014	12	13.9286	78.5000	935.7561	2.1969	1.6531
81	2014	12	13.3857	76.7551	946.5214	2.5163	1.4286
15	2014	12	14.1000	68.5612	940.1612	1.6898	1.1439
32	2014	12	12.0571	84.6837	879.7571	1.9531	2.5806
73	2014	12	15.4429	73.4490	970.1969	2.1622	0.9000
71	2014	12	13.9286	78.5000	935.7561	2.1969	1.6531
41	2014	12	11.9714	80.2755	879.2173	2.4194	1.3806
10	2014	12	14.4286	80.2755	972.5163	1.9388	1.2316
23	2014	12	9.2857	67.0306	777.7051	7.1806	2.6724
27	2014	12	13.7714	77.9490	863.1357	3.6276	2.2367
60	2014	12	13.3857	76.7551	946.5214	2.5163	1.4286
53	2014	12	11.7429	85.3163	858.1714	2.1908	2.5337
66	2014	12	11.8429	87.2347	903.0357	1.8112	2.3184
59	2014	12	12.6000	79.0102	891.9388	2.4837	1.6684
61	2014	12	15.4429	73.4490	970.1969	2.1622	0.9000
84	2014	12	15.4429	73.4490	970.1969	2.1622	0.9000
38	2014	12	12.6000	79.0102	891.9388	2.4837	1.6684
87	2014	12	13.7714	80.3469	904.9704	1.4122	2.1337
34	2014	12	12.6000	79.0102	891.9388	2.4837	1.6684
29	2014	12	14.4286	75.0000	947.1806	2.3173	1.5000
5	2014	12	12.2286	79.6122	835.4327	3.5520	2.4949
8	2014	12	11.7429	85.3163	858.1714	2.1908	2.5337
12	2014	12	12.2286	79.6122	835.4327	3.5520	2.4949
13	2014	12	17.6286	71.7653	950.9541	2.2173	0.9643
18	2014	12	13.6000	77.2143	972.6296	1.8214	1.0347
33	2014	12	13.4714	74.2959	907.3408	2.8531	1.6643
56	2014	12	16.5143	77.8571	985.4592	1.4296	1.0071
77	2014	12	14.1000	68.5612	940.1612	1.6898	1.1439
54	2014	12	12.2286	79.6122	835.4327	3.5520	2.4949
21	2014	12	13.4714	74.2959	907.3408	2.8531	1.6643
68	2014	12	14.4714	78.9796	987.1265	1.5622	0.7582
74	2014	12	15.4429	73.4490	970.1969	2.1622	0.9000
88	2014	12	12.0571	84.6837	879.7571	1.9531	2.5806
16	2014	12	13.3143	75.8980	927.7184	2.2959	1.4602
30	2014	12	11.8429	87.2347	903.0357	1.8112	2.3184
6	2014	12	14.4714	78.9796	987.1265	1.5622	0.7582
49	2014	12	14.4286	75.0000	947.1806	2.3173	1.5000

22	2014	12	12.0571	84.6837	879.7571	1.9531	2.5806
45	2014	12	13.2286	64.5102	820.0735	5.7622	2.7673
58	2014	12	14.4286	75.0000	947.1806	2.3173	1.5000
37	2014	12	14.4714	78.9796	987.1265	1.5622	0.7582
17	2014	12	12.3714	84.0612	908.1102	1.6143	3.0602
55	2014	12	12.9000	82.3367	882.3918	2.4112	2.2102
46	2014	12	13.3143	75.8980	927.7184	2.2959	1.4602
86	2014	12	13.4571	78.3367	870.2663	2.9827	1.4122
2	2014	12	13.4571	78.3367	870.2663	2.9827	1.4122
4	2014	12	13.4714	74.2959	907.3408	2.8531	1.6643
47	2014	12	16.9286	72.7245	966.0776	1.5745	0.7714
82	2014	12	12.0571	84.6837	879.7571	1.9531	2.5806
19	2014	12	15.1714	78.5408	968.3306	0.7010	1.2327
20	2014	12	11.7429	85.3163	858.1714	2.1908	2.5337
80	2014	12	12.0571	84.6837	879.7571	1.9531	2.5806
3	2014	12	17.6286	71.7653	950.9541	2.2173	0.9643
52	2014	12	12.3714	84.0612	908.1102	1.6143	3.0602
70	2014	12	14.2714	74.7857	916.1316	3.1122	1.6296
64	2014	12	9.2857	67.0306	777.7051	7.1806	2.6724
48	2014	12	14.1000	68.5612	940.1612	1.6898	1.1439
65	2014	12	12.3714	84.0612	908.1102	1.6143	3.0602
44	2014	12	14.2714	74.7857	916.1316	3.1122	1.6296
75	2014	12	9.2857	67.0306	777.7051	7.1806	2.6724
40	2014	12	14.1143	83.9184	953.0061	1.5959	1.7316
11	2014	12	12.9000	82.3367	882.3918	2.4112	2.2102
35	2014	12	13.3857	76.7551	946.5214	2.5163	1.4286
78	2014	12	13.7714	80.3469	904.9704	1.4122	2.1337
28	2014	12	13.9286	78.5000	935.7561	2.1969	1.6531
39	2014	12	12.3714	84.0612	908.1102	1.6143	3.0602
24	2014	12	14.4286	75.0000	947.1806	2.3173	1.5000
63	2014	12	14.1143	83.9184	953.0061	1.5959	1.7316
62	2014	12	11.9714	80.2755	879.2173	2.4194	1.3806
1	2014	12	12.0571	84.6837	879.7571	1.9531	2.5806
31	2014	13	13.2143	77.9388	849.2776	3.8561	1.1643
79	2014	13	17.0714	81.8776	984.9020	1.1755	0.7122
51	2014	13	15.5000	79.9694	944.7816	2.4224	1.3061
14	2014	13	16.8000	87.2653	901.9010	1.9224	2.7347
67	2014	13	16.4286	83.6122	906.7398	2.1031	3.2163
42	2014	13	15.0714	83.1020	878.6041	2.7337	2.6255
50	2014	13	14.2000	75.9592	906.1000	2.5837	1.5408
43	2014	13	15.0714	83.1020	878.6041	2.7337	2.6255
85	2014	13	14.7286	78.4286	914.9020	2.7010	1.5245
25	2014	13	19.9429	78.0918	983.4214	2.5806	1.0163
69	2014	13	16.2857	78.1429	945.4582	1.8235	1.3612
57	2014	13	13.5286	80.2347	890.7694	2.4347	1.9827
9	2014	13	15.4429	82.5714	857.2082	3.7367	2.4918
72	2014	13	17.4857	80.5816	881.2143	3.1990	2.1327
26	2014	13	18.7714	76.9184	870.1990	4.7163	2.6163
7	2014	13	17.7429	75.3776	862.1694	5.2051	2.2510
83	2014	13	21.6714	72.2959	949.3551	3.5255	0.9837

76	2014	13	14.6143	77.9082	926.2010	2.1541	1.5245
36	2014	13	16.3714	78.7653	934.1255	2.6949	1.5490
81	2014	13	15.5000	79.9694	944.7816	2.4224	1.3061
15	2014	13	15.6000	69.5612	938.5929	1.4439	1.0745
32	2014	13	15.0714	83.1020	878.6041	2.7337	2.6255
73	2014	13	16.1286	76.4898	968.3224	1.6837	0.8735
71	2014	13	16.3714	78.7653	934.1255	2.6949	1.5490
41	2014	13	14.5857	81.2857	878.1153	3.2520	1.4673
10	2014	13	16.9143	82.0510	970.4878	1.9429	1.1490
23	2014	13	11.8429	70.8367	777.3510	6.0092	2.3469
27	2014	13	17.7429	75.3776	862.1694	5.2051	2.2510
60	2014	13	15.5000	79.9694	944.7816	2.4224	1.3061
53	2014	13	15.4429	82.5714	857.2082	3.7367	2.4918
66	2014	13	16.8000	87.2653	901.9010	1.9224	2.7347
59	2014	13	13.5286	80.2347	890.7694	2.4347	1.9827
61	2014	13	16.1286	76.4898	968.3224	1.6837	0.8735
84	2014	13	16.1286	76.4898	968.3224	1.6837	0.8735
38	2014	13	13.5286	80.2347	890.7694	2.4347	1.9827
87	2014	13	17.7857	78.9694	903.6224	2.6571	2.1602
34	2014	13	13.5286	80.2347	890.7694	2.4347	1.9827
29	2014	13	16.2857	78.1429	945.4582	1.8235	1.3612
5	2014	13	15.3000	77.3776	834.6173	5.2061	2.2367
8	2014	13	15.4429	82.5714	857.2082	3.7367	2.4918
12	2014	13	15.3000	77.3776	834.6173	5.2061	2.2367
13	2014	13	21.6714	72.2959	949.3551	3.5255	0.9837
18	2014	13	16.2000	80.0306	970.4867	1.6673	0.8857
33	2014	13	14.2000	75.9592	906.1000	2.5837	1.5408
56	2014	13	19.9429	78.0918	983.4214	2.5806	1.0163
77	2014	13	15.6000	69.5612	938.5929	1.4439	1.0745
54	2014	13	15.3000	77.3776	834.6173	5.2061	2.2367
21	2014	13	14.2000	75.9592	906.1000	2.5837	1.5408
68	2014	13	17.0714	81.8776	984.9020	1.1755	0.7122
74	2014	13	16.1286	76.4898	968.3224	1.6837	0.8735
88	2014	13	15.0714	83.1020	878.6041	2.7337	2.6255
16	2014	13	14.6143	77.9082	926.2010	2.1541	1.5245
30	2014	13	16.8000	87.2653	901.9010	1.9224	2.7347
6	2014	13	17.0714	81.8776	984.9020	1.1755	0.7122
49	2014	13	16.2857	78.1429	945.4582	1.8235	1.3612
22	2014	13	15.0714	83.1020	878.6041	2.7337	2.6255
45	2014	13	16.2714	63.7347	819.4765	6.0990	2.2990
58	2014	13	16.2857	78.1429	945.4582	1.8235	1.3612
37	2014	13	17.0714	81.8776	984.9020	1.1755	0.7122
17	2014	13	16.4286	83.6122	906.7398	2.1031	3.2163
55	2014	13	17.4857	80.5816	881.2143	3.1990	2.1327
46	2014	13	14.6143	77.9082	926.2010	2.1541	1.5245
86	2014	13	15.9857	75.9898	869.1633	4.1041	1.4418
2	2014	13	15.9857	75.9898	869.1633	4.1041	1.4418
4	2014	13	14.2000	75.9592	906.1000	2.5837	1.5408
47	2014	13	21.5286	74.9898	964.2888	2.7714	0.6694
82	2014	13	15.0714	83.1020	878.6041	2.7337	2.6255

19	2014	13	20.0000	81.0816	966.6755	0.7510	1.1337
20	2014	13	15.4429	82.5714	857.2082	3.7367	2.4918
80	2014	13	15.0714	83.1020	878.6041	2.7337	2.6255
3	2014	13	21.6714	72.2959	949.3551	3.5255	0.9837
52	2014	13	16.4286	83.6122	906.7398	2.1031	3.2163
70	2014	13	14.7286	78.4286	914.9020	2.7010	1.5245
64	2014	13	11.8429	70.8367	777.3510	6.0092	2.3469
48	2014	13	15.6000	69.5612	938.5929	1.4439	1.0745
65	2014	13	16.4286	83.6122	906.7398	2.1031	3.2163
44	2014	13	14.7286	78.4286	914.9020	2.7010	1.5245
75	2014	13	11.8429	70.8367	777.3510	6.0092	2.3469
40	2014	13	17.9857	83.3367	951.2765	2.4418	1.7908
11	2014	13	17.4857	80.5816	881.2143	3.1990	2.1327
35	2014	13	15.5000	79.9694	944.7816	2.4224	1.3061
78	2014	13	17.7857	78.9694	903.6224	2.6571	2.1602
28	2014	13	16.3714	78.7653	934.1255	2.6949	1.5490
39	2014	13	16.4286	83.6122	906.7398	2.1031	3.2163
24	2014	13	16.2857	78.1429	945.4582	1.8235	1.3612
63	2014	13	17.9857	83.3367	951.2765	2.4418	1.7908
62	2014	13	14.5857	81.2857	878.1153	3.2520	1.4673
1	2014	13	15.0714	83.1020	878.6041	2.7337	2.6255
31	2014	14	11.4143	82.0714	847.9163	2.9939	1.0806
79	2014	14	16.8857	83.3878	982.0827	1.0337	0.6827
51	2014	14	14.8857	82.7143	942.4388	1.6480	1.3306
14	2014	14	14.6714	85.8571	899.4949	1.8714	2.5827
67	2014	14	14.2714	83.7245	904.4837	1.5306	2.8286
42	2014	14	12.9143	84.9592	876.7541	2.2888	2.5520
50	2014	14	13.2857	81.4082	904.3939	1.0990	1.3245
43	2014	14	12.9143	84.9592	876.7541	2.2888	2.5520
85	2014	14	14.0286	84.3367	913.2265	1.6398	1.4908
25	2014	14	17.5857	81.3878	980.3561	2.8092	0.9357
69	2014	14	15.5571	82.0408	943.1673	1.5204	1.2857
57	2014	14	12.3143	86.4592	889.0673	1.5133	2.1612
9	2014	14	12.2286	83.0714	855.4878	3.7694	2.4286
72	2014	14	14.2857	80.1735	879.1622	2.8755	2.3378
26	2014	14	15.6429	75.5816	868.1622	5.5551	2.4143
7	2014	14	14.2714	76.8878	860.3276	5.1276	2.1745
83	2014	14	19.3571	73.7959	946.5622	4.5735	1.0265
76	2014	14	13.9857	82.2449	924.2265	1.3347	1.4704
36	2014	14	15.3143	81.2755	931.7571	2.2745	1.4010
81	2014	14	14.8857	82.7143	942.4388	1.6480	1.3306
15	2014	14	14.6286	74.5408	936.5918	0.8837	0.8224
32	2014	14	12.9143	84.9592	876.7541	2.2888	2.5520
73	2014	14	16.0571	81.8265	965.9827	1.0816	0.7541
71	2014	14	15.3143	81.2755	931.7571	2.2745	1.4010
41	2014	14	12.9286	84.0204	876.4867	2.0806	1.4367
10	2014	14	16.0429	83.8367	967.7265	1.7929	0.9541
23	2014	14	8.0286	78.6633	776.1082	4.5898	2.4541
27	2014	14	14.2714	76.8878	860.3276	5.1276	2.1745
60	2014	14	14.8857	82.7143	942.4388	1.6480	1.3306

53	2014	14	12.2286	83.0714	855.4878	3.7694	2.4286
66	2014	14	14.6714	85.8571	899.4949	1.8714	2.5827
59	2014	14	12.3143	86.4592	889.0673	1.5133	2.1612
61	2014	14	16.0571	81.8265	965.9827	1.0816	0.7541
84	2014	14	16.0571	81.8265	965.9827	1.0816	0.7541
38	2014	14	12.3143	86.4592	889.0673	1.5133	2.1612
87	2014	14	15.1143	79.8571	901.4765	2.7357	2.0796
34	2014	14	12.3143	86.4592	889.0673	1.5133	2.1612
29	2014	14	15.5571	82.0408	943.1673	1.5204	1.2857
5	2014	14	11.8429	82.7857	833.0214	4.6959	2.1071
8	2014	14	12.2286	83.0714	855.4878	3.7694	2.4286
12	2014	14	11.8429	82.7857	833.0214	4.6959	2.1071
13	2014	14	19.3571	73.7959	946.5622	4.5735	1.0265
18	2014	14	15.4143	82.7347	967.8765	1.5724	0.7929
33	2014	14	13.2857	81.4082	904.3939	1.0990	1.3245
56	2014	14	17.5857	81.3878	980.3561	2.8092	0.9357
77	2014	14	14.6286	74.5408	936.5918	0.8837	0.8224
54	2014	14	11.8429	82.7857	833.0214	4.6959	2.1071
21	2014	14	13.2857	81.4082	904.3939	1.0990	1.3245
68	2014	14	16.8857	83.3878	982.0827	1.0337	0.6827
74	2014	14	16.0571	81.8265	965.9827	1.0816	0.7541
88	2014	14	12.9143	84.9592	876.7541	2.2888	2.5520
16	2014	14	13.9857	82.2449	924.2265	1.3347	1.4704
30	2014	14	14.6714	85.8571	899.4949	1.8714	2.5827
6	2014	14	16.8857	83.3878	982.0827	1.0337	0.6827
49	2014	14	15.5571	82.0408	943.1673	1.5204	1.2857
22	2014	14	12.9143	84.9592	876.7541	2.2888	2.5520
45	2014	14	12.3429	69.7653	817.9408	5.4296	2.0418
58	2014	14	15.5571	82.0408	943.1673	1.5204	1.2857
37	2014	14	16.8857	83.3878	982.0827	1.0337	0.6827
17	2014	14	14.2714	83.7245	904.4837	1.5306	2.8286
55	2014	14	14.2857	80.1735	879.1622	2.8755	2.3378
46	2014	14	13.9857	82.2449	924.2265	1.3347	1.4704
86	2014	14	13.2286	78.5306	867.6031	3.0531	1.2990
2	2014	14	13.2286	78.5306	867.6031	3.0531	1.2990
4	2014	14	13.2857	81.4082	904.3939	1.0990	1.3245
47	2014	14	18.9143	78.3980	961.2510	3.6010	0.6276
82	2014	14	12.9143	84.9592	876.7541	2.2888	2.5520
19	2014	14	18.2286	80.9286	963.4786	1.4357	1.1276
20	2014	14	12.2286	83.0714	855.4878	3.7694	2.4286
80	2014	14	12.9143	84.9592	876.7541	2.2888	2.5520
3	2014	14	19.3571	73.7959	946.5622	4.5735	1.0265
52	2014	14	14.2714	83.7245	904.4837	1.5306	2.8286
70	2014	14	14.0286	84.3367	913.2265	1.6398	1.4908
64	2014	14	8.0286	78.6633	776.1082	4.5898	2.4541
48	2014	14	14.6286	74.5408	936.5918	0.8837	0.8224
65	2014	14	14.2714	83.7245	904.4837	1.5306	2.8286
44	2014	14	14.0286	84.3367	913.2265	1.6398	1.4908
75	2014	14	8.0286	78.6633	776.1082	4.5898	2.4541
40	2014	14	15.2571	85.0204	948.5316	2.6276	1.4347

11	2014	14	14.2857	80.1735	879.1622	2.8755	2.3378
35	2014	14	14.8857	82.7143	942.4388	1.6480	1.3306
78	2014	14	15.1143	79.8571	901.4765	2.7357	2.0796
28	2014	14	15.3143	81.2755	931.7571	2.2745	1.4010
39	2014	14	14.2714	83.7245	904.4837	1.5306	2.8286
24	2014	14	15.5571	82.0408	943.1673	1.5204	1.2857
63	2014	14	15.2571	85.0204	948.5316	2.6276	1.4347
62	2014	14	12.9286	84.0204	876.4867	2.0806	1.4367
1	2014	14	12.9143	84.9592	876.7541	2.2888	2.5520
31	2014	15	15.8714	82.2245	847.8633	2.2714	0.9929
79	2014	15	18.5429	84.2857	981.3806	1.0918	0.7337
51	2014	15	17.0429	83.5816	941.9602	1.7551	1.3724
14	2014	15	17.4571	87.0510	899.1520	1.8163	2.3051
67	2014	15	16.9000	85.8163	904.2235	0.8755	2.3561
42	2014	15	17.1857	86.2449	876.6520	2.0418	2.4071
50	2014	15	16.9571	83.5408	904.0041	1.0633	1.2918
43	2014	15	17.1857	86.2449	876.6520	2.0418	2.4071
85	2014	15	17.3000	84.4388	912.7684	1.6041	1.4092
25	2014	15	20.1857	85.0918	979.7235	2.0531	0.7673
69	2014	15	18.2286	84.2347	942.6735	1.7408	1.2378
57	2014	15	16.7571	86.7143	888.7816	1.5847	2.2255
9	2014	15	17.2714	84.5306	855.5224	2.9541	2.3929
72	2014	15	19.0000	81.7245	879.0612	2.3745	2.2735
26	2014	15	20.2857	77.0408	868.0418	5.1173	2.1378
7	2014	15	19.2857	79.5918	860.3224	4.3755	2.1918
83	2014	15	24.0571	74.5816	945.9010	4.7816	1.0122
76	2014	15	17.4571	83.6735	923.7520	1.5357	1.3878
36	2014	15	17.9143	83.2041	931.3296	2.1816	1.4459
81	2014	15	17.0429	83.5816	941.9602	1.7551	1.3724
15	2014	15	17.9857	79.1633	936.0378	1.1918	0.6969
32	2014	15	17.1857	86.2449	876.6520	2.0418	2.4071
73	2014	15	18.5714	85.2245	965.3327	1.2847	0.6561
71	2014	15	17.9143	83.2041	931.3296	2.1816	1.4459
41	2014	15	17.3429	83.6429	876.3265	1.6265	1.3286
10	2014	15	17.8143	84.8163	967.1306	1.8551	0.9163
23	2014	15	14.7714	80.7143	776.5184	4.4429	2.5531
27	2014	15	19.2857	79.5918	860.3224	4.3755	2.1918
60	2014	15	17.0429	83.5816	941.9602	1.7551	1.3724
53	2014	15	17.2714	84.5306	855.5224	2.9541	2.3929
66	2014	15	17.4571	87.0510	899.1520	1.8163	2.3051
59	2014	15	16.7571	86.7143	888.7816	1.5847	2.2255
61	2014	15	18.5714	85.2245	965.3327	1.2847	0.6561
84	2014	15	18.5714	85.2245	965.3327	1.2847	0.6561
38	2014	15	16.7571	86.7143	888.7816	1.5847	2.2255
87	2014	15	19.8714	82.0612	901.2347	2.1071	1.7888
34	2014	15	16.7571	86.7143	888.7816	1.5847	2.2255
29	2014	15	18.2286	84.2347	942.6735	1.7408	1.2378
5	2014	15	17.4714	84.9490	833.2031	3.6265	2.0806
8	2014	15	17.2714	84.5306	855.5224	2.9541	2.3929
12	2014	15	17.4714	84.9490	833.2031	3.6265	2.0806

13	2014	15	24.0571	74.5816	945.9010	4.7816	1.0122
18	2014	15	17.5000	84.7551	967.3041	1.5949	0.8071
33	2014	15	16.9571	83.5408	904.0041	1.0633	1.2918
56	2014	15	20.1857	85.0918	979.7235	2.0531	0.7673
77	2014	15	17.9857	79.1633	936.0378	1.1918	0.6969
54	2014	15	17.4714	84.9490	833.2031	3.6265	2.0806
21	2014	15	16.9571	83.5408	904.0041	1.0633	1.2918
68	2014	15	18.5429	84.2857	981.3806	1.0918	0.7337
74	2014	15	18.5714	85.2245	965.3327	1.2847	0.6561
88	2014	15	17.1857	86.2449	876.6520	2.0418	2.4071
16	2014	15	17.4571	83.6735	923.7520	1.5357	1.3878
30	2014	15	17.4571	87.0510	899.1520	1.8163	2.3051
6	2014	15	18.5429	84.2857	981.3806	1.0918	0.7337
49	2014	15	18.2286	84.2347	942.6735	1.7408	1.2378
22	2014	15	17.1857	86.2449	876.6520	2.0418	2.4071
45	2014	15	18.3857	74.1939	818.1459	5.0296	1.8755
58	2014	15	18.2286	84.2347	942.6735	1.7408	1.2378
37	2014	15	18.5429	84.2857	981.3806	1.0918	0.7337
17	2014	15	16.9000	85.8163	904.2235	0.8755	2.3561
55	2014	15	19.0000	81.7245	879.0612	2.3745	2.2735
46	2014	15	17.4571	83.6735	923.7520	1.5357	1.3878
86	2014	15	17.9571	80.2551	867.6153	2.3265	1.2031
2	2014	15	17.9571	80.2551	867.6153	2.3265	1.2031
4	2014	15	16.9571	83.5408	904.0041	1.0633	1.2918
47	2014	15	22.8286	80.5714	960.5204	3.1694	0.5765
82	2014	15	17.1857	86.2449	876.6520	2.0418	2.4071
19	2014	15	20.5143	82.7041	962.6663	1.4480	1.1194
20	2014	15	17.2714	84.5306	855.5224	2.9541	2.3929
80	2014	15	17.1857	86.2449	876.6520	2.0418	2.4071
3	2014	15	24.0571	74.5816	945.9010	4.7816	1.0122
52	2014	15	16.9000	85.8163	904.2235	0.8755	2.3561
70	2014	15	17.3000	84.4388	912.7684	1.6041	1.4092
64	2014	15	14.7714	80.7143	776.5184	4.4429	2.5531
48	2014	15	17.9857	79.1633	936.0378	1.1918	0.6969
65	2014	15	16.9000	85.8163	904.2235	0.8755	2.3561
44	2014	15	17.3000	84.4388	912.7684	1.6041	1.4092
75	2014	15	14.7714	80.7143	776.5184	4.4429	2.5531
40	2014	15	17.4429	87.3367	948.0061	2.2633	1.3816
11	2014	15	19.0000	81.7245	879.0612	2.3745	2.2735
35	2014	15	17.0429	83.5816	941.9602	1.7551	1.3724
78	2014	15	19.8714	82.0612	901.2347	2.1071	1.7888
28	2014	15	17.9143	83.2041	931.3296	2.1816	1.4459
39	2014	15	16.9000	85.8163	904.2235	0.8755	2.3561
24	2014	15	18.2286	84.2347	942.6735	1.7408	1.2378
63	2014	15	17.4429	87.3367	948.0061	2.2633	1.3816
62	2014	15	17.3429	83.6429	876.3265	1.6265	1.3286
1	2014	15	17.1857	86.2449	876.6520	2.0418	2.4071
31	2014	16	20.1286	78.3061	846.7092	3.6663	1.0214
79	2014	16	18.9857	85.5714	980.7296	0.7571	0.8316
51	2014	16	19.0714	83.8878	941.2020	2.5122	1.7908

14	2014	16	20.2857	89.2245	898.9306	1.6378	3.1000
67	2014	16	20.0286	88.0102	903.6582	1.1449	2.9439
42	2014	16	21.5429	82.8980	875.8143	4.0214	2.8694
50	2014	16	20.2429	81.1837	902.5939	2.2582	1.9408
43	2014	16	21.5429	82.8980	875.8143	4.0214	2.8694
85	2014	16	22.0857	80.7959	911.0949	2.7990	1.5796
25	2014	16	23.4429	82.9388	979.1347	1.7133	1.0592
69	2014	16	21.6286	82.2755	941.4643	1.9398	1.6214
57	2014	16	20.9857	80.4388	887.4561	3.5582	2.8337
9	2014	16	21.9000	80.1327	854.8959	4.9837	2.9041
72	2014	16	23.1429	79.2551	878.5173	4.2214	2.3092
26	2014	16	24.0429	75.4184	867.7418	6.5704	2.5224
7	2014	16	23.2714	75.8061	859.9143	6.4520	2.8153
83	2014	16	27.6571	70.6531	944.9510	5.7194	1.1643
76	2014	16	19.8000	82.8673	922.4408	2.5276	1.7102
36	2014	16	21.4857	81.4184	930.4755	3.2316	1.7439
81	2014	16	19.0714	83.8878	941.2020	2.5122	1.7908
15	2014	16	20.1571	80.5612	934.6847	1.5602	0.8347
32	2014	16	21.5429	82.8980	875.8143	4.0214	2.8694
73	2014	16	20.9143	83.7347	964.0367	1.6602	0.7939
71	2014	16	21.4857	81.4184	930.4755	3.2316	1.7439
41	2014	16	21.4000	79.4592	875.0867	3.3837	1.5102
10	2014	16	21.2000	85.2551	966.4816	1.8714	1.1265
23	2014	16	18.9286	65.3469	776.4480	7.5500	2.5969
27	2014	16	23.2714	75.8061	859.9143	6.4520	2.8153
60	2014	16	19.0714	83.8878	941.2020	2.5122	1.7908
53	2014	16	21.9000	80.1327	854.8959	4.9837	2.9041
66	2014	16	20.2857	89.2245	898.9306	1.6378	3.1000
59	2014	16	20.9857	80.4388	887.4561	3.5582	2.8337
61	2014	16	20.9143	83.7347	964.0367	1.6602	0.7939
84	2014	16	20.9143	83.7347	964.0367	1.6602	0.7939
38	2014	16	20.9857	80.4388	887.4561	3.5582	2.8337
87	2014	16	24.0143	78.3163	900.2827	3.1357	1.9296
34	2014	16	20.9857	80.4388	887.4561	3.5582	2.8337
29	2014	16	21.6286	82.2755	941.4643	1.9398	1.6214
5	2014	16	22.7000	72.8367	832.8602	6.4031	2.5571
8	2014	16	21.9000	80.1327	854.8959	4.9837	2.9041
12	2014	16	22.7000	72.8367	832.8602	6.4031	2.5571
13	2014	16	27.6571	70.6531	944.9510	5.7194	1.1643
18	2014	16	17.0143	87.5306	966.7949	0.7173	0.9112
33	2014	16	20.2429	81.1837	902.5939	2.2582	1.9408
56	2014	16	23.4429	82.9388	979.1347	1.7133	1.0592
77	2014	16	20.1571	80.5612	934.6847	1.5602	0.8347
54	2014	16	22.7000	72.8367	832.8602	6.4031	2.5571
21	2014	16	20.2429	81.1837	902.5939	2.2582	1.9408
68	2014	16	18.9857	85.5714	980.7296	0.7571	0.8316
74	2014	16	20.9143	83.7347	964.0367	1.6602	0.7939
88	2014	16	21.5429	82.8980	875.8143	4.0214	2.8694
16	2014	16	19.8000	82.8673	922.4408	2.5276	1.7102
30	2014	16	20.2857	89.2245	898.9306	1.6378	3.1000

6	2014	16	18.9857	85.5714	980.7296	0.7571	0.8316
49	2014	16	21.6286	82.2755	941.4643	1.9398	1.6214
22	2014	16	21.5429	82.8980	875.8143	4.0214	2.8694
45	2014	16	22.8286	61.4490	818.0602	7.6949	2.4490
58	2014	16	21.6286	82.2755	941.4643	1.9398	1.6214
37	2014	16	18.9857	85.5714	980.7296	0.7571	0.8316
17	2014	16	20.0286	88.0102	903.6582	1.1449	2.9439
55	2014	16	23.1429	79.2551	878.5173	4.2214	2.3092
46	2014	16	19.8000	82.8673	922.4408	2.5276	1.7102
86	2014	16	22.4857	76.3980	866.5378	4.3367	1.4969
2	2014	16	22.4857	76.3980	866.5378	4.3367	1.4969
4	2014	16	20.2429	81.1837	902.5939	2.2582	1.9408
47	2014	16	26.6000	77.9898	959.5061	3.2755	0.5867
82	2014	16	21.5429	82.8980	875.8143	4.0214	2.8694
19	2014	16	22.9143	84.8265	962.2969	0.6633	1.2153
20	2014	16	21.9000	80.1327	854.8959	4.9837	2.9041
80	2014	16	21.5429	82.8980	875.8143	4.0214	2.8694
3	2014	16	27.6571	70.6531	944.9510	5.7194	1.1643
52	2014	16	20.0286	88.0102	903.6582	1.1449	2.9439
70	2014	16	22.0857	80.7959	911.0949	2.7990	1.5796
64	2014	16	18.9286	65.3469	776.4480	7.5500	2.5969
48	2014	16	20.1571	80.5612	934.6847	1.5602	0.8347
65	2014	16	20.0286	88.0102	903.6582	1.1449	2.9439
44	2014	16	22.0857	80.7959	911.0949	2.7990	1.5796
75	2014	16	18.9286	65.3469	776.4480	7.5500	2.5969
40	2014	16	21.1429	86.3367	947.6878	2.4878	1.8143
11	2014	16	23.1429	79.2551	878.5173	4.2214	2.3092
35	2014	16	19.0714	83.8878	941.2020	2.5122	1.7908
78	2014	16	24.0143	78.3163	900.2827	3.1357	1.9296
28	2014	16	21.4857	81.4184	930.4755	3.2316	1.7439
39	2014	16	20.0286	88.0102	903.6582	1.1449	2.9439
24	2014	16	21.6286	82.2755	941.4643	1.9398	1.6214
63	2014	16	21.1429	86.3367	947.6878	2.4878	1.8143
62	2014	16	21.4000	79.4592	875.0867	3.3837	1.5102
1	2014	16	21.5429	82.8980	875.8143	4.0214	2.8694
31	2014	17	14.1429	76.4388	845.5541	3.9194	1.1327
79	2014	17	16.9143	85.5102	979.6837	0.7082	0.9541
51	2014	17	14.6286	83.7857	940.0235	2.4449	2.0429
14	2014	17	15.2714	88.5816	897.6347	1.4857	3.4918
67	2014	17	14.2714	87.5816	902.3776	1.3061	3.4908
42	2014	17	14.7286	80.2245	874.4878	4.6163	3.2755
50	2014	17	15.1714	79.4898	901.2112	2.8561	2.2949
43	2014	17	14.7286	80.2245	874.4878	4.6163	3.2755
85	2014	17	15.9143	77.6429	909.6102	3.1031	1.7786
25	2014	17	18.2429	79.4082	977.6480	1.6908	1.4031
69	2014	17	16.2143	79.2245	940.0786	1.9490	1.8929
57	2014	17	14.0286	78.2143	886.0980	3.9194	3.0520
9	2014	17	15.6571	75.9694	853.6153	6.0694	3.5704
72	2014	17	17.3143	76.0816	877.1204	4.7398	2.7153
26	2014	17	19.7857	70.6020	866.3102	8.0684	2.8092

7	2014	17	18.6571	71.0510	858.5490	7.6031	3.1092
83	2014	17	22.3143	67.7449	943.0561	5.7786	1.3112
76	2014	17	15.2714	82.2755	921.0704	2.7184	1.9633
36	2014	17	15.1857	78.9694	929.1918	3.6122	2.0531
81	2014	17	14.6286	83.7857	940.0235	2.4449	2.0429
15	2014	17	16.5429	80.1327	933.2776	1.7122	1.0643
32	2014	17	14.7286	80.2245	874.4878	4.6163	3.2755
73	2014	17	17.2571	81.2449	962.6867	1.6092	1.0133
71	2014	17	15.1857	78.9694	929.1918	3.6122	2.0531
41	2014	17	15.2000	77.0408	873.7612	3.8541	1.6408
10	2014	17	15.8857	83.9592	965.2490	1.6265	1.3439
23	2014	17	13.0000	57.5204	775.7041	9.5122	2.7551
27	2014	17	18.6571	71.0510	858.5490	7.6031	3.1092
60	2014	17	14.6286	83.7857	940.0235	2.4449	2.0429
53	2014	17	15.6571	75.9694	853.6153	6.0694	3.5704
66	2014	17	15.2714	88.5816	897.6347	1.4857	3.4918
59	2014	17	14.0286	78.2143	886.0980	3.9194	3.0520
61	2014	17	17.2571	81.2449	962.6867	1.6092	1.0133
84	2014	17	17.2571	81.2449	962.6867	1.6092	1.0133
38	2014	17	14.0286	78.2143	886.0980	3.9194	3.0520
87	2014	17	17.7571	74.1531	898.7571	3.3735	2.4888
34	2014	17	14.0286	78.2143	886.0980	3.9194	3.0520
29	2014	17	16.2143	79.2245	940.0786	1.9490	1.8929
5	2014	17	16.1000	65.0918	831.6663	7.9255	2.8735
8	2014	17	15.6571	75.9694	853.6153	6.0694	3.5704
12	2014	17	16.1000	65.0918	831.6663	7.9255	2.8735
13	2014	17	22.3143	67.7449	943.0561	5.7786	1.3112
18	2014	17	16.0714	87.6327	965.8408	0.0663	1.0194
33	2014	17	15.1714	79.4898	901.2112	2.8561	2.2949
56	2014	17	18.2429	79.4082	977.6480	1.6908	1.4031
77	2014	17	16.5429	80.1327	933.2776	1.7122	1.0643
54	2014	17	16.1000	65.0918	831.6663	7.9255	2.8735
21	2014	17	15.1714	79.4898	901.2112	2.8561	2.2949
68	2014	17	16.9143	85.5102	979.6837	0.7082	0.9541
74	2014	17	17.2571	81.2449	962.6867	1.6092	1.0133
88	2014	17	14.7286	80.2245	874.4878	4.6163	3.2755
16	2014	17	15.2714	82.2755	921.0704	2.7184	1.9633
30	2014	17	15.2714	88.5816	897.6347	1.4857	3.4918
6	2014	17	16.9143	85.5102	979.6837	0.7082	0.9541
49	2014	17	16.2143	79.2245	940.0786	1.9490	1.8929
22	2014	17	14.7286	80.2245	874.4878	4.6163	3.2755
45	2014	17	18.0857	49.5204	817.0173	9.3786	3.0469
58	2014	17	16.2143	79.2245	940.0786	1.9490	1.8929
37	2014	17	16.9143	85.5102	979.6837	0.7082	0.9541
17	2014	17	14.2714	87.5816	902.3776	1.3061	3.4908
55	2014	17	17.3143	76.0816	877.1204	4.7398	2.7153
46	2014	17	15.2714	82.2755	921.0704	2.7184	1.9633
86	2014	17	16.4143	72.0204	865.1673	5.2010	1.7337
2	2014	17	16.4143	72.0204	865.1673	5.2010	1.7337
4	2014	17	15.1714	79.4898	901.2112	2.8561	2.2949

47	2014	17	21.0571	75.4490	957.6469	3.3857	0.6092
82	2014	17	14.7286	80.2245	874.4878	4.6163	3.2755
19	2014	17	18.7714	83.3061	960.7735	0.2959	1.3490
20	2014	17	15.6571	75.9694	853.6153	6.0694	3.5704
80	2014	17	14.7286	80.2245	874.4878	4.6163	3.2755
3	2014	17	22.3143	67.7449	943.0561	5.7786	1.3112
52	2014	17	14.2714	87.5816	902.3776	1.3061	3.4908
70	2014	17	15.9143	77.6429	909.6102	3.1031	1.7786
64	2014	17	13.0000	57.5204	775.7041	9.5122	2.7551
48	2014	17	16.5429	80.1327	933.2776	1.7122	1.0643
65	2014	17	14.2714	87.5816	902.3776	1.3061	3.4908
44	2014	17	15.9143	77.6429	909.6102	3.1031	1.7786
75	2014	17	13.0000	57.5204	775.7041	9.5122	2.7551
40	2014	17	14.8286	85.8265	946.5122	2.3969	2.1704
11	2014	17	17.3143	76.0816	877.1204	4.7398	2.7153
35	2014	17	14.6286	83.7857	940.0235	2.4449	2.0429
78	2014	17	17.7571	74.1531	898.7571	3.3735	2.4888
28	2014	17	15.1857	78.9694	929.1918	3.6122	2.0531
39	2014	17	14.2714	87.5816	902.3776	1.3061	3.4908
24	2014	17	16.2143	79.2245	940.0786	1.9490	1.8929
63	2014	17	14.8286	85.8265	946.5122	2.3969	2.1704
62	2014	17	15.2000	77.0408	873.7612	3.8541	1.6408
1	2014	17	14.7286	80.2245	874.4878	4.6163	3.2755
31	2014	18	13.1571	80.1327	848.2173	2.4520	1.1316
79	2014	18	18.1000	84.0000	981.9480	1.4878	0.8959
51	2014	18	15.9857	83.1429	942.3816	2.0898	1.5449
14	2014	18	16.0143	87.1429	899.4347	1.7133	2.2867
67	2014	18	15.6714	86.3265	904.5541	1.4837	2.9571
42	2014	18	15.1714	81.7857	876.8684	2.7449	2.8031
50	2014	18	15.3857	78.8571	904.1469	2.7031	1.8449
43	2014	18	15.1714	81.7857	876.8684	2.7449	2.8031
85	2014	18	16.1571	78.4898	912.8643	2.6051	1.5194
25	2014	18	19.1286	81.5306	979.9724	1.6684	1.1684
69	2014	18	16.7143	80.3980	942.8633	2.0367	1.3796
57	2014	18	14.7571	80.7143	888.9612	2.4265	2.2112
9	2014	18	14.7857	79.2551	855.7857	3.7612	3.0214
72	2014	18	16.2571	79.1020	879.2857	2.6449	2.4949
26	2014	18	16.8857	76.7857	868.2347	5.4908	2.2908
7	2014	18	15.9143	75.9796	860.5469	4.7827	2.4235
83	2014	18	20.3571	73.3061	945.7276	3.8071	1.1980
76	2014	18	15.8143	81.2857	923.8388	2.2337	1.6276
36	2014	18	16.4714	80.0816	931.6643	2.4878	1.7235
81	2014	18	15.9857	83.1429	942.3816	2.0898	1.5449
15	2014	18	16.1571	79.6122	936.0673	2.0837	0.9776
32	2014	18	15.1714	81.7857	876.8684	2.7449	2.8031
73	2014	18	17.6571	81.4082	965.4388	1.5959	1.0296
71	2014	18	16.4714	80.0816	931.6643	2.4878	1.7235
41	2014	18	15.0143	80.2653	876.5500	2.4041	1.3214
10	2014	18	17.2857	83.5102	967.6010	1.7816	1.1827
23	2014	18	10.5429	71.2755	777.3041	6.5541	2.7051

27	2014	18	15.9143	75.9796	860.5469	4.7827	2.4235
60	2014	18	15.9857	83.1429	942.3816	2.0898	1.5449
53	2014	18	14.7857	79.2551	855.7857	3.7612	3.0214
66	2014	18	16.0143	87.1429	899.4347	1.7133	2.2867
59	2014	18	14.7571	80.7143	888.9612	2.4265	2.2112
61	2014	18	17.6571	81.4082	965.4388	1.5959	1.0296
84	2014	18	17.6571	81.4082	965.4388	1.5959	1.0296
38	2014	18	14.7571	80.7143	888.9612	2.4265	2.2112
87	2014	18	16.7571	77.4082	901.2582	2.0847	2.2582
34	2014	18	14.7571	80.7143	888.9612	2.4265	2.2112
29	2014	18	16.7143	80.3980	942.8633	2.0367	1.3796
5	2014	18	14.1714	76.7041	833.5918	4.8418	2.3153
8	2014	18	14.7857	79.2551	855.7857	3.7612	3.0214
12	2014	18	14.1714	76.7041	833.5918	4.8418	2.3153
13	2014	18	20.3571	73.3061	945.7276	3.8071	1.1980
18	2014	18	16.8429	84.3776	967.9143	1.2163	1.0306
33	2014	18	15.3857	78.8571	904.1469	2.7031	1.8449
56	2014	18	19.1286	81.5306	979.9724	1.6684	1.1684
77	2014	18	16.1571	79.6122	936.0673	2.0837	0.9776
54	2014	18	14.1714	76.7041	833.5918	4.8418	2.3153
21	2014	18	15.3857	78.8571	904.1469	2.7031	1.8449
68	2014	18	18.1000	84.0000	981.9480	1.4878	0.8959
74	2014	18	17.6571	81.4082	965.4388	1.5959	1.0296
88	2014	18	15.1714	81.7857	876.8684	2.7449	2.8031
16	2014	18	15.8143	81.2857	923.8388	2.2337	1.6276
30	2014	18	16.0143	87.1429	899.4347	1.7133	2.2867
6	2014	18	18.1000	84.0000	981.9480	1.4878	0.8959
49	2014	18	16.7143	80.3980	942.8633	2.0367	1.3796
22	2014	18	15.1714	81.7857	876.8684	2.7449	2.8031
45	2014	18	14.1571	65.5102	818.6367	5.9418	2.4337
58	2014	18	16.7143	80.3980	942.8633	2.0367	1.3796
37	2014	18	18.1000	84.0000	981.9480	1.4878	0.8959
17	2014	18	15.6714	86.3265	904.5541	1.4837	2.9571
55	2014	18	16.2571	79.1020	879.2857	2.6449	2.4949
46	2014	18	15.8143	81.2857	923.8388	2.2337	1.6276
86	2014	18	15.3000	75.3367	867.7724	3.0939	1.4306
2	2014	18	15.3000	75.3367	867.7724	3.0939	1.4306
4	2014	18	15.3857	78.8571	904.1469	2.7031	1.8449
47	2014	18	19.9286	79.2143	960.4469	2.2663	0.5306
82	2014	18	15.1714	81.7857	876.8684	2.7449	2.8031
19	2014	18	19.6429	80.3878	962.8031	1.0653	1.2551
20	2014	18	14.7857	79.2551	855.7857	3.7612	3.0214
80	2014	18	15.1714	81.7857	876.8684	2.7449	2.8031
3	2014	18	20.3571	73.3061	945.7276	3.8071	1.1980
52	2014	18	15.6714	86.3265	904.5541	1.4837	2.9571
70	2014	18	16.1571	78.4898	912.8643	2.6051	1.5194
64	2014	18	10.5429	71.2755	777.3041	6.5541	2.7051
48	2014	18	16.1571	79.6122	936.0673	2.0837	0.9776
65	2014	18	15.6714	86.3265	904.5541	1.4837	2.9571
44	2014	18	16.1571	78.4898	912.8643	2.6051	1.5194

75	2014	18	10.5429	71.2755	777.3041	6.5541	2.7051
40	2014	18	16.8143	86.7755	948.4888	1.9704	1.7520
11	2014	18	16.2571	79.1020	879.2857	2.6449	2.4949
35	2014	18	15.9857	83.1429	942.3816	2.0898	1.5449
78	2014	18	16.7571	77.4082	901.2582	2.0847	2.2582
28	2014	18	16.4714	80.0816	931.6643	2.4878	1.7235
39	2014	18	15.6714	86.3265	904.5541	1.4837	2.9571
24	2014	18	16.7143	80.3980	942.8633	2.0367	1.3796
63	2014	18	16.8143	86.7755	948.4888	1.9704	1.7520
62	2014	18	15.0143	80.2653	876.5500	2.4041	1.3214
1	2014	18	15.1714	81.7857	876.8684	2.7449	2.8031
31	2014	19	14.9571	81.2755	849.6796	2.0612	1.0531
79	2014	19	19.8857	80.3367	982.6571	2.8806	0.7990
51	2014	19	17.8429	78.2551	943.3765	3.0490	1.3357
14	2014	19	17.2143	83.2755	900.7194	2.4163	1.7531
67	2014	19	17.1857	82.0204	905.7724	2.3449	2.7357
42	2014	19	16.3000	80.3776	878.3143	2.1510	2.4245
50	2014	19	16.8143	76.6837	905.6388	3.1816	1.5714
43	2014	19	16.3000	80.3776	878.3143	2.1510	2.4245
85	2014	19	17.4571	78.7653	914.4816	3.2000	1.3908
25	2014	19	21.0000	81.5000	981.0888	2.3755	0.9582
69	2014	19	18.5143	79.2857	944.1561	2.8337	1.2163
57	2014	19	15.8429	79.7755	890.4673	2.2714	1.9816
9	2014	19	15.9857	79.5408	857.2388	2.9398	2.4194
72	2014	19	17.5143	78.8061	880.8612	2.5898	2.0041
26	2014	19	19.6429	79.1531	869.9143	3.7143	2.0204
7	2014	19	18.5857	77.9796	862.1531	3.4520	1.9520
83	2014	19	22.2571	73.8061	947.8531	3.7224	1.0724
76	2014	19	17.5571	76.9694	925.1582	2.7592	1.3745
36	2014	19	17.8429	78.8163	932.8969	2.5786	1.3480
81	2014	19	17.8429	78.2551	943.3765	3.0490	1.3357
15	2014	19	18.6286	78.0204	937.4796	3.0061	0.7816
32	2014	19	16.3000	80.3776	878.3143	2.1510	2.4245
73	2014	19	19.0571	79.1224	966.5867	3.0061	0.9694
71	2014	19	17.8429	78.8163	932.8969	2.5786	1.3480
41	2014	19	16.4571	79.8571	878.0776	1.8694	1.1204
10	2014	19	18.7857	79.8980	968.5276	2.9173	1.1010
23	2014	19	12.1714	81.0204	778.4582	3.3714	2.4633
27	2014	19	18.5857	77.9796	862.1531	3.4520	1.9520
60	2014	19	17.8429	78.2551	943.3765	3.0490	1.3357
53	2014	19	15.9857	79.5408	857.2388	2.9398	2.4194
66	2014	19	17.2143	83.2755	900.7194	2.4163	1.7531
59	2014	19	15.8429	79.7755	890.4673	2.2714	1.9816
61	2014	19	19.0571	79.1224	966.5867	3.0061	0.9694
84	2014	19	19.0571	79.1224	966.5867	3.0061	0.9694
38	2014	19	15.8429	79.7755	890.4673	2.2714	1.9816
87	2014	19	18.3286	77.8673	902.8959	2.5714	1.9000
34	2014	19	15.8429	79.7755	890.4673	2.2714	1.9816
29	2014	19	18.5143	79.2857	944.1561	2.8337	1.2163
5	2014	19	16.2714	83.1224	835.0520	3.5031	1.9102

8	2014	19	15.9857	79.5408	857.2388	2.9398	2.4194
12	2014	19	16.2714	83.1224	835.0520	3.5031	1.9102
13	2014	19	22.2571	73.8061	947.8531	3.7224	1.0724
18	2014	19	18.7714	79.9490	968.5357	3.1337	1.0510
33	2014	19	16.8143	76.6837	905.6388	3.1816	1.5714
56	2014	19	21.0000	81.5000	981.0888	2.3755	0.9582
77	2014	19	18.6286	78.0204	937.4796	3.0061	0.7816
54	2014	19	16.2714	83.1224	835.0520	3.5031	1.9102
21	2014	19	16.8143	76.6837	905.6388	3.1816	1.5714
68	2014	19	19.8857	80.3367	982.6571	2.8806	0.7990
74	2014	19	19.0571	79.1224	966.5867	3.0061	0.9694
88	2014	19	16.3000	80.3776	878.3143	2.1510	2.4245
16	2014	19	17.5571	76.9694	925.1582	2.7592	1.3745
30	2014	19	17.2143	83.2755	900.7194	2.4163	1.7531
6	2014	19	19.8857	80.3367	982.6571	2.8806	0.7990
49	2014	19	18.5143	79.2857	944.1561	2.8337	1.2163
22	2014	19	16.3000	80.3776	878.3143	2.1510	2.4245
45	2014	19	16.6571	78.3163	819.9980	3.3265	1.9020
58	2014	19	18.5143	79.2857	944.1561	2.8337	1.2163
37	2014	19	19.8857	80.3367	982.6571	2.8806	0.7990
17	2014	19	17.1857	82.0204	905.7724	2.3449	2.7357
55	2014	19	17.5143	78.8061	880.8612	2.5898	2.0041
46	2014	19	17.5571	76.9694	925.1582	2.7592	1.3745
86	2014	19	17.0857	77.1531	869.3102	2.1316	1.2561
2	2014	19	17.0857	77.1531	869.3102	2.1316	1.2561
4	2014	19	16.8143	76.6837	905.6388	3.1816	1.5714
47	2014	19	21.8857	79.1327	962.4265	2.8827	0.5051
82	2014	19	16.3000	80.3776	878.3143	2.1510	2.4245
19	2014	19	20.7000	77.7245	964.2051	2.0724	1.1000
20	2014	19	15.9857	79.5408	857.2388	2.9398	2.4194
80	2014	19	16.3000	80.3776	878.3143	2.1510	2.4245
3	2014	19	22.2571	73.8061	947.8531	3.7224	1.0724
52	2014	19	17.1857	82.0204	905.7724	2.3449	2.7357
70	2014	19	17.4571	78.7653	914.4816	3.2000	1.3908
64	2014	19	12.1714	81.0204	778.4582	3.3714	2.4633
48	2014	19	18.6286	78.0204	937.4796	3.0061	0.7816
65	2014	19	17.1857	82.0204	905.7724	2.3449	2.7357
44	2014	19	17.4571	78.7653	914.4816	3.2000	1.3908
75	2014	19	12.1714	81.0204	778.4582	3.3714	2.4633
40	2014	19	19.0000	82.4286	949.3837	2.8857	1.2653
11	2014	19	17.5143	78.8061	880.8612	2.5898	2.0041
35	2014	19	17.8429	78.2551	943.3765	3.0490	1.3357
78	2014	19	18.3286	77.8673	902.8959	2.5714	1.9000
28	2014	19	17.8429	78.8163	932.8969	2.5786	1.3480
39	2014	19	17.1857	82.0204	905.7724	2.3449	2.7357
24	2014	19	18.5143	79.2857	944.1561	2.8337	1.2163
63	2014	19	19.0000	82.4286	949.3837	2.8857	1.2653
62	2014	19	16.4571	79.8571	878.0776	1.8694	1.1204
1	2014	19	16.3000	80.3776	878.3143	2.1510	2.4245
31	2014	20	15.8571	80.6837	846.6327	1.4439	1.0306

79	2014	20	20.5000	81.1531	978.4878	2.3694	0.7694
51	2014	20	19.0714	80.4490	939.4704	2.4551	1.3939
14	2014	20	18.7714	83.9898	897.3490	2.1816	2.1878
67	2014	20	18.4714	83.5714	902.2653	1.6816	2.5847
42	2014	20	17.7429	82.2041	875.0663	1.6633	2.3776
50	2014	20	17.3000	79.2857	902.0827	1.9633	1.4510
43	2014	20	17.7429	82.2041	875.0663	1.6633	2.3776
85	2014	20	18.1714	80.2959	910.8745	2.7439	1.4337
25	2014	20	22.4000	80.7653	976.7633	2.2296	1.0071
69	2014	20	20.2571	80.6224	940.2173	2.2959	1.4306
57	2014	20	16.8714	82.6122	887.0551	1.2969	2.0878
9	2014	20	18.0571	79.3367	854.1388	3.4500	2.6204
72	2014	20	19.9429	77.5102	877.5327	3.1031	2.1653
26	2014	20	23.6429	71.2041	866.5408	5.7653	2.4173
7	2014	20	22.0000	72.6837	858.8531	4.7480	2.2143
83	2014	20	26.0714	68.8878	943.6980	5.3673	1.0949
76	2014	20	18.3857	78.8878	921.4133	1.9071	1.3796
36	2014	20	19.3143	81.4490	929.1286	2.2582	1.2429
81	2014	20	19.0714	80.4490	939.4704	2.4551	1.3939
15	2014	20	19.1571	77.5408	933.7082	2.3010	0.7582
32	2014	20	17.7429	82.2041	875.0663	1.6633	2.3776
73	2014	20	20.1571	81.2143	962.5214	2.3622	0.8327
71	2014	20	19.3143	81.4490	929.1286	2.2582	1.2429
41	2014	20	17.8571	80.3265	874.7408	1.7500	1.1704
10	2014	20	19.9286	82.5408	964.4306	2.1929	1.1296
23	2014	20	15.5857	79.2245	775.9602	4.7173	2.4980
27	2014	20	22.0000	72.6837	858.8531	4.7480	2.2143
60	2014	20	19.0714	80.4490	939.4704	2.4551	1.3939
53	2014	20	18.0571	79.3367	854.1388	3.4500	2.6204
66	2014	20	18.7714	83.9898	897.3490	2.1816	2.1878
59	2014	20	16.8714	82.6122	887.0551	1.2969	2.0878
61	2014	20	20.1571	81.2143	962.5214	2.3622	0.8327
84	2014	20	20.1571	81.2143	962.5214	2.3622	0.8327
38	2014	20	16.8714	82.6122	887.0551	1.2969	2.0878
87	2014	20	20.3000	77.5102	899.4224	2.7561	2.1265
34	2014	20	16.8714	82.6122	887.0551	1.2969	2.0878
29	2014	20	20.2571	80.6224	940.2173	2.2959	1.4306
5	2014	20	19.5714	77.6735	832.0082	5.3367	2.0592
8	2014	20	18.0571	79.3367	854.1388	3.4500	2.6204
12	2014	20	19.5714	77.6735	832.0082	5.3367	2.0592
13	2014	20	26.0714	68.8878	943.6980	5.3673	1.0949
18	2014	20	19.5714	80.0918	964.4622	2.9469	1.0071
33	2014	20	17.3000	79.2857	902.0827	1.9633	1.4510
56	2014	20	22.4000	80.7653	976.7633	2.2296	1.0071
77	2014	20	19.1571	77.5408	933.7082	2.3010	0.7582
54	2014	20	19.5714	77.6735	832.0082	5.3367	2.0592
21	2014	20	17.3000	79.2857	902.0827	1.9633	1.4510
68	2014	20	20.5000	81.1531	978.4878	2.3694	0.7694
74	2014	20	20.1571	81.2143	962.5214	2.3622	0.8327
88	2014	20	17.7429	82.2041	875.0663	1.6633	2.3776

16	2014	20	18.3857	78.8878	921.4133	1.9071	1.3796
30	2014	20	18.7714	83.9898	897.3490	2.1816	2.1878
6	2014	20	20.5000	81.1531	978.4878	2.3694	0.7694
49	2014	20	20.2571	80.6224	940.2173	2.2959	1.4306
22	2014	20	17.7429	82.2041	875.0663	1.6633	2.3776
45	2014	20	21.3000	69.4082	817.1296	5.1735	2.3071
58	2014	20	20.2571	80.6224	940.2173	2.2959	1.4306
37	2014	20	20.5000	81.1531	978.4878	2.3694	0.7694
17	2014	20	18.4714	83.5714	902.2653	1.6816	2.5847
55	2014	20	19.9429	77.5102	877.5327	3.1031	2.1653
46	2014	20	18.3857	78.8878	921.4133	1.9071	1.3796
86	2014	20	18.7857	76.3265	866.0602	2.3714	1.3633
2	2014	20	18.7857	76.3265	866.0602	2.3714	1.3633
4	2014	20	17.3000	79.2857	902.0827	1.9633	1.4510
47	2014	20	24.5714	74.6020	958.2418	3.5643	0.5694
82	2014	20	17.7429	82.2041	875.0663	1.6633	2.3776
19	2014	20	22.9857	78.9388	960.2122	1.7255	1.1745
20	2014	20	18.0571	79.3367	854.1388	3.4500	2.6204
80	2014	20	17.7429	82.2041	875.0663	1.6633	2.3776
3	2014	20	26.0714	68.8878	943.6980	5.3673	1.0949
52	2014	20	18.4714	83.5714	902.2653	1.6816	2.5847
70	2014	20	18.1714	80.2959	910.8745	2.7439	1.4337
64	2014	20	15.5857	79.2245	775.9602	4.7173	2.4980
48	2014	20	19.1571	77.5408	933.7082	2.3010	0.7582
65	2014	20	18.4714	83.5714	902.2653	1.6816	2.5847
44	2014	20	18.1714	80.2959	910.8745	2.7439	1.4337
75	2014	20	15.5857	79.2245	775.9602	4.7173	2.4980
40	2014	20	19.3571	84.0000	945.4908	2.7418	1.2684
11	2014	20	19.9429	77.5102	877.5327	3.1031	2.1653
35	2014	20	19.0714	80.4490	939.4704	2.4551	1.3939
78	2014	20	20.3000	77.5102	899.4224	2.7561	2.1265
28	2014	20	19.3143	81.4490	929.1286	2.2582	1.2429
39	2014	20	18.4714	83.5714	902.2653	1.6816	2.5847
24	2014	20	20.2571	80.6224	940.2173	2.2959	1.4306
63	2014	20	19.3571	84.0000	945.4908	2.7418	1.2684
62	2014	20	17.8571	80.3265	874.7408	1.7500	1.1704
1	2014	20	17.7429	82.2041	875.0663	1.6633	2.3776
31	2014	21	18.3000	80.6020	844.5469	1.4092	1.0816
79	2014	21	21.7143	84.0102	975.5673	1.2020	0.7694
51	2014	21	20.1714	84.7347	936.7786	1.4163	1.3735
14	2014	21	21.4714	87.8367	894.7490	1.7673	2.2694
67	2014	21	20.2429	88.2653	899.7408	0.7102	2.5827
42	2014	21	20.3286	85.7143	872.6990	1.5592	2.4939
50	2014	21	19.6143	83.4388	899.5867	0.8459	1.4551
43	2014	21	20.3286	85.7143	872.6990	1.5592	2.4939
85	2014	21	20.4143	81.6327	908.3204	2.6276	1.3245
25	2014	21	23.6714	82.7245	973.6796	1.5980	1.0061
69	2014	21	21.2714	83.7347	937.4153	1.1469	1.4010
57	2014	21	19.2571	85.2755	884.6224	0.7765	1.9245
9	2014	21	20.6429	80.6735	851.8939	3.7633	2.8408

72	2014	21	22.3429	79.3061	875.0051	2.8990	2.5704
26	2014	21	25.5000	68.8469	863.7582	7.7133	2.7071
7	2014	21	23.4857	71.4694	856.3133	5.7092	2.6755
83	2014	21	27.2286	68.7755	940.2541	5.9827	1.2163
76	2014	21	20.0286	84.1837	918.7786	0.9714	1.3990
36	2014	21	20.5571	85.0306	926.4347	1.5520	1.3000
81	2014	21	20.1714	84.7347	936.7786	1.4163	1.3735
15	2014	21	20.6571	80.7755	930.9959	1.2112	0.7622
32	2014	21	20.3286	85.7143	872.6990	1.5592	2.4939
73	2014	21	21.8429	85.0510	959.5449	0.8867	0.7153
71	2014	21	20.5571	85.0306	926.4347	1.5520	1.3000
41	2014	21	20.0714	81.8980	872.3837	2.1929	1.2714
10	2014	21	21.0143	87.5204	961.6316	0.8714	0.9633
23	2014	21	18.0143	74.5408	774.3020	7.2408	2.6745
27	2014	21	23.4857	71.4694	856.3133	5.7092	2.6755
60	2014	21	20.1714	84.7347	936.7786	1.4163	1.3735
53	2014	21	20.6429	80.6735	851.8939	3.7633	2.8408
66	2014	21	21.4714	87.8367	894.7490	1.7673	2.2694
59	2014	21	19.2571	85.2755	884.6224	0.7765	1.9245
61	2014	21	21.8429	85.0510	959.5449	0.8867	0.7153
84	2014	21	21.8429	85.0510	959.5449	0.8867	0.7153
38	2014	21	19.2571	85.2755	884.6224	0.7765	1.9245
87	2014	21	22.7571	79.4898	896.7571	1.9286	2.2735
34	2014	21	19.2571	85.2755	884.6224	0.7765	1.9245
29	2014	21	21.2714	83.7347	937.4153	1.1469	1.4010
5	2014	21	22.0143	74.7041	829.7694	6.9714	2.3633
8	2014	21	20.6429	80.6735	851.8939	3.7633	2.8408
12	2014	21	22.0143	74.7041	829.7694	6.9714	2.3633
13	2014	21	27.2286	68.7755	940.2541	5.9827	1.2163
18	2014	21	21.1857	82.7755	961.6112	1.4622	0.9735
33	2014	21	19.6143	83.4388	899.5867	0.8459	1.4551
56	2014	21	23.6714	82.7245	973.6796	1.5980	1.0061
77	2014	21	20.6571	80.7755	930.9959	1.2112	0.7622
54	2014	21	22.0143	74.7041	829.7694	6.9714	2.3633
21	2014	21	19.6143	83.4388	899.5867	0.8459	1.4551
68	2014	21	21.7143	84.0102	975.5673	1.2020	0.7694
74	2014	21	21.8429	85.0510	959.5449	0.8867	0.7153
88	2014	21	20.3286	85.7143	872.6990	1.5592	2.4939
16	2014	21	20.0286	84.1837	918.7786	0.9714	1.3990
30	2014	21	21.4714	87.8367	894.7490	1.7673	2.2694
6	2014	21	21.7143	84.0102	975.5673	1.2020	0.7694
49	2014	21	21.2714	83.7347	937.4153	1.1469	1.4010
22	2014	21	20.3286	85.7143	872.6990	1.5592	2.4939
45	2014	21	23.2857	63.4898	814.9653	7.2714	2.5112
58	2014	21	21.2714	83.7347	937.4153	1.1469	1.4010
37	2014	21	21.7143	84.0102	975.5673	1.2020	0.7694
17	2014	21	20.2429	88.2653	899.7408	0.7102	2.5827
55	2014	21	22.3429	79.3061	875.0051	2.8990	2.5704
46	2014	21	20.0286	84.1837	918.7786	0.9714	1.3990
86	2014	21	20.7857	76.6735	863.7888	3.1224	1.3469

2	2014	21	20.7857	76.6735	863.7888	3.1224	1.3469
4	2014	21	19.6143	83.4388	899.5867	0.8459	1.4551
47	2014	21	26.1000	75.4490	954.8449	2.8224	0.6673
82	2014	21	20.3286	85.7143	872.6990	1.5592	2.4939
19	2014	21	24.5143	80.5306	957.0163	1.2867	1.3102
20	2014	21	20.6429	80.6735	851.8939	3.7633	2.8408
80	2014	21	20.3286	85.7143	872.6990	1.5592	2.4939
3	2014	21	27.2286	68.7755	940.2541	5.9827	1.2163
52	2014	21	20.2429	88.2653	899.7408	0.7102	2.5827
70	2014	21	20.4143	81.6327	908.3204	2.6276	1.3245
64	2014	21	18.0143	74.5408	774.3020	7.2408	2.6745
48	2014	21	20.6571	80.7755	930.9959	1.2112	0.7622
65	2014	21	20.2429	88.2653	899.7408	0.7102	2.5827
44	2014	21	20.4143	81.6327	908.3204	2.6276	1.3245
75	2014	21	18.0143	74.5408	774.3020	7.2408	2.6745
40	2014	21	21.2571	89.2653	942.8071	1.6071	1.4735
11	2014	21	22.3429	79.3061	875.0051	2.8990	2.5704
35	2014	21	20.1714	84.7347	936.7786	1.4163	1.3735
78	2014	21	22.7571	79.4898	896.7571	1.9286	2.2735
28	2014	21	20.5571	85.0306	926.4347	1.5520	1.3000
39	2014	21	20.2429	88.2653	899.7408	0.7102	2.5827
24	2014	21	21.2714	83.7347	937.4153	1.1469	1.4010
63	2014	21	21.2571	89.2653	942.8071	1.6071	1.4735
62	2014	21	20.0714	81.8980	872.3837	2.1929	1.2714
1	2014	21	20.3286	85.7143	872.6990	1.5592	2.4939
31	2014	22	19.3429	78.3878	846.0276	1.2112	1.0714
79	2014	22	24.5714	85.9796	976.2439	1.2082	0.7378
51	2014	22	23.1143	85.3367	937.7520	1.2551	1.2316
14	2014	22	21.6000	88.3265	895.9245	1.5276	2.0765
67	2014	22	21.5571	88.3673	900.8898	0.4480	2.6357
42	2014	22	19.5714	85.6122	873.9867	1.3071	2.3296
50	2014	22	19.7571	84.3469	900.8684	0.7908	1.4602
43	2014	22	19.5714	85.6122	873.9867	1.3071	2.3296
85	2014	22	20.6429	82.1224	909.6224	2.2622	1.2041
25	2014	22	25.5143	86.0102	974.5755	1.6133	0.8714
69	2014	22	22.4000	86.3776	938.4592	0.7418	1.1520
57	2014	22	19.2000	84.5000	885.9163	0.8316	1.7531
9	2014	22	19.1429	80.5510	853.3133	2.8082	2.4255
72	2014	22	20.9714	80.1122	876.3622	2.0398	2.3388
26	2014	22	21.1714	75.4490	865.2612	6.1755	2.3500
7	2014	22	20.6571	76.6429	857.8469	4.4541	2.4306
83	2014	22	25.0000	74.2041	941.6071	4.3510	1.2071
76	2014	22	20.8000	86.5816	919.8929	1.1296	1.4908
36	2014	22	22.8857	86.0612	927.5153	1.2184	1.1724
81	2014	22	23.1143	85.3367	937.7520	1.2551	1.2316
15	2014	22	21.0714	84.0714	932.1082	1.0224	0.6816
32	2014	22	19.5714	85.6122	873.9867	1.3071	2.3296
73	2014	22	22.7857	86.6122	960.4653	0.9571	0.6592
71	2014	22	22.8857	86.0612	927.5153	1.2184	1.1724
41	2014	22	20.3143	80.8469	873.7653	1.5980	1.2622

10	2014	22	24.4143	87.5102	962.4490	1.0571	0.7847
23	2014	22	16.1143	75.3469	775.9531	5.9306	2.5398
27	2014	22	20.6571	76.6429	857.8469	4.4541	2.4306
60	2014	22	23.1143	85.3367	937.7520	1.2551	1.2316
53	2014	22	19.1429	80.5510	853.3133	2.8082	2.4255
66	2014	22	21.6000	88.3265	895.9245	1.5276	2.0765
59	2014	22	19.2000	84.5000	885.9163	0.8316	1.7531
61	2014	22	22.7857	86.6122	960.4653	0.9571	0.6592
84	2014	22	22.7857	86.6122	960.4653	0.9571	0.6592
38	2014	22	19.2000	84.5000	885.9163	0.8316	1.7531
87	2014	22	21.5286	79.9490	898.0071	1.1133	2.2398
34	2014	22	19.2000	84.5000	885.9163	0.8316	1.7531
29	2014	22	22.4000	86.3776	938.4592	0.7418	1.1520
5	2014	22	19.0429	78.2653	831.3133	5.5153	2.1378
8	2014	22	19.1429	80.5510	853.3133	2.8082	2.4255
12	2014	22	19.0429	78.2653	831.3133	5.5153	2.1378
13	2014	22	25.0000	74.2041	941.6071	4.3510	1.2071
18	2014	22	22.5857	84.5306	962.4418	0.6224	0.8918
33	2014	22	19.7571	84.3469	900.8684	0.7908	1.4602
56	2014	22	25.5143	86.0102	974.5755	1.6133	0.8714
77	2014	22	21.0714	84.0714	932.1082	1.0224	0.6816
54	2014	22	19.0429	78.2653	831.3133	5.5153	2.1378
21	2014	22	19.7571	84.3469	900.8684	0.7908	1.4602
68	2014	22	24.5714	85.9796	976.2439	1.2082	0.7378
74	2014	22	22.7857	86.6122	960.4653	0.9571	0.6592
88	2014	22	19.5714	85.6122	873.9867	1.3071	2.3296
16	2014	22	20.8000	86.5816	919.8929	1.1296	1.4908
30	2014	22	21.6000	88.3265	895.9245	1.5276	2.0765
6	2014	22	24.5714	85.9796	976.2439	1.2082	0.7378
49	2014	22	22.4000	86.3776	938.4592	0.7418	1.1520
22	2014	22	19.5714	85.6122	873.9867	1.3071	2.3296
45	2014	22	19.0571	70.3673	816.5092	5.7765	2.1031
58	2014	22	22.4000	86.3776	938.4592	0.7418	1.1520
37	2014	22	24.5714	85.9796	976.2439	1.2082	0.7378
17	2014	22	21.5571	88.3673	900.8898	0.4480	2.6357
55	2014	22	20.9714	80.1122	876.3622	2.0398	2.3388
46	2014	22	20.8000	86.5816	919.8929	1.1296	1.4908
86	2014	22	20.5143	77.2449	865.2704	2.4490	1.1643
2	2014	22	20.5143	77.2449	865.2704	2.4490	1.1643
4	2014	22	19.7571	84.3469	900.8684	0.7908	1.4602
47	2014	22	25.2143	79.5714	956.0061	2.0337	0.7459
82	2014	22	19.5714	85.6122	873.9867	1.3071	2.3296
19	2014	22	25.5429	82.6224	958.0347	1.3031	1.1204
20	2014	22	19.1429	80.5510	853.3133	2.8082	2.4255
80	2014	22	19.5714	85.6122	873.9867	1.3071	2.3296
3	2014	22	25.0000	74.2041	941.6071	4.3510	1.2071
52	2014	22	21.5571	88.3673	900.8898	0.4480	2.6357
70	2014	22	20.6429	82.1224	909.6224	2.2622	1.2041
64	2014	22	16.1143	75.3469	775.9531	5.9306	2.5398
48	2014	22	21.0714	84.0714	932.1082	1.0224	0.6816

65	2014	22	21.5571	88.3673	900.8898	0.4480	2.6357
44	2014	22	20.6429	82.1224	909.6224	2.2622	1.2041
75	2014	22	16.1143	75.3469	775.9531	5.9306	2.5398
40	2014	22	23.9429	89.5306	943.7286	1.5551	1.4204
11	2014	22	20.9714	80.1122	876.3622	2.0398	2.3388
35	2014	22	23.1143	85.3367	937.7520	1.2551	1.2316
78	2014	22	21.5286	79.9490	898.0071	1.1133	2.2398
28	2014	22	22.8857	86.0612	927.5153	1.2184	1.1724
39	2014	22	21.5571	88.3673	900.8898	0.4480	2.6357
24	2014	22	22.4000	86.3776	938.4592	0.7418	1.1520
63	2014	22	23.9429	89.5306	943.7286	1.5551	1.4204
62	2014	22	20.3143	80.8469	873.7653	1.5980	1.2622
1	2014	22	19.5714	85.6122	873.9867	1.3071	2.3296
31	2014	23	20.5857	75.6531	845.3041	1.4643	0.9765
79	2014	23	23.2143	87.6531	974.8327	1.3031	0.6347
51	2014	23	21.5571	84.5510	936.6694	1.4969	1.1418
14	2014	23	22.1857	87.3367	895.3520	1.3449	2.1235
67	2014	23	20.9714	87.3980	900.1827	0.6520	2.8133
42	2014	23	20.6571	86.1020	873.3878	1.0357	2.1000
50	2014	23	20.7000	85.0306	899.8878	1.1357	1.4510
43	2014	23	20.6571	86.1020	873.3878	1.0357	2.1000
85	2014	23	21.8714	82.1327	908.4898	1.6184	1.3010
25	2014	23	25.1286	86.1837	973.3316	2.0551	0.8367
69	2014	23	22.2857	87.4898	937.3816	0.8459	1.2031
57	2014	23	19.9000	84.2041	885.1031	0.9990	2.0316
9	2014	23	20.9286	81.4388	852.8796	2.4153	2.2327
72	2014	23	22.5000	79.9490	875.9071	1.8378	2.0153
26	2014	23	22.9857	80.9388	865.2622	4.7602	2.0459
7	2014	23	22.4429	79.9694	857.6776	3.6214	2.0214
83	2014	23	26.2429	77.2959	941.1122	3.7939	1.1337
76	2014	23	20.9571	87.8469	918.9694	1.4347	1.5143
36	2014	23	22.3000	84.1939	926.5296	1.4571	1.1663
81	2014	23	21.5571	84.5510	936.6694	1.4969	1.1418
15	2014	23	21.8000	85.5612	931.0663	1.3306	0.5255
32	2014	23	20.6571	86.1020	873.3878	1.0357	2.1000
73	2014	23	22.7857	88.4286	959.3347	1.2459	0.5622
71	2014	23	22.3000	84.1939	926.5296	1.4571	1.1663
41	2014	23	20.8857	81.0408	873.0265	1.5500	1.3235
10	2014	23	22.7143	85.6327	961.1194	1.8724	0.8867
23	2014	23	18.0429	75.9286	775.9408	4.6490	2.2745
27	2014	23	22.4429	79.9694	857.6776	3.6214	2.0214
60	2014	23	21.5571	84.5510	936.6694	1.4969	1.1418
53	2014	23	20.9286	81.4388	852.8796	2.4153	2.2327
66	2014	23	22.1857	87.3367	895.3520	1.3449	2.1235
59	2014	23	19.9000	84.2041	885.1031	0.9990	2.0316
61	2014	23	22.7857	88.4286	959.3347	1.2459	0.5622
84	2014	23	22.7857	88.4286	959.3347	1.2459	0.5622
38	2014	23	19.9000	84.2041	885.1031	0.9990	2.0316
87	2014	23	23.2143	80.5816	897.3480	1.0143	1.9571
34	2014	23	19.9000	84.2041	885.1031	0.9990	2.0316

29	2014	23	22.2857	87.4898	937.3816	0.8459	1.2031
5	2014	23	20.8000	80.5000	831.1796	4.3929	1.7092
8	2014	23	20.9286	81.4388	852.8796	2.4153	2.2327
12	2014	23	20.8000	80.5000	831.1796	4.3929	1.7092
13	2014	23	26.2429	77.2959	941.1122	3.7939	1.1337
18	2014	23	22.4286	85.8878	961.2704	0.4755	0.7888
33	2014	23	20.7000	85.0306	899.8878	1.1357	1.4510
56	2014	23	25.1286	86.1837	973.3316	2.0551	0.8367
77	2014	23	21.8000	85.5612	931.0663	1.3306	0.5255
54	2014	23	20.8000	80.5000	831.1796	4.3929	1.7092
21	2014	23	20.7000	85.0306	899.8878	1.1357	1.4510
68	2014	23	23.2143	87.6531	974.8327	1.3031	0.6347
74	2014	23	22.7857	88.4286	959.3347	1.2459	0.5622
88	2014	23	20.6571	86.1020	873.3878	1.0357	2.1000
16	2014	23	20.9571	87.8469	918.9694	1.4347	1.5143
30	2014	23	22.1857	87.3367	895.3520	1.3449	2.1235
6	2014	23	23.2143	87.6531	974.8327	1.3031	0.6347
49	2014	23	22.2857	87.4898	937.3816	0.8459	1.2031
22	2014	23	20.6571	86.1020	873.3878	1.0357	2.1000
45	2014	23	20.4571	76.5000	816.5122	4.6531	1.8347
58	2014	23	22.2857	87.4898	937.3816	0.8459	1.2031
37	2014	23	23.2143	87.6531	974.8327	1.3031	0.6347
17	2014	23	20.9714	87.3980	900.1827	0.6520	2.8133
55	2014	23	22.5000	79.9490	875.9071	1.8378	2.0153
46	2014	23	20.9571	87.8469	918.9694	1.4347	1.5143
86	2014	23	21.6857	77.9082	864.6173	1.9857	1.1704
2	2014	23	21.6857	77.9082	864.6173	1.9857	1.1704
4	2014	23	20.7000	85.0306	899.8878	1.1357	1.4510
47	2014	23	26.4429	80.5714	955.1378	2.1398	0.7296
82	2014	23	20.6571	86.1020	873.3878	1.0357	2.1000
19	2014	23	25.6143	83.8061	957.0867	1.5153	1.0204
20	2014	23	20.9286	81.4388	852.8796	2.4153	2.2327
80	2014	23	20.6571	86.1020	873.3878	1.0357	2.1000
3	2014	23	26.2429	77.2959	941.1122	3.7939	1.1337
52	2014	23	20.9714	87.3980	900.1827	0.6520	2.8133
70	2014	23	21.8714	82.1327	908.4898	1.6184	1.3010
64	2014	23	18.0429	75.9286	775.9408	4.6490	2.2745
48	2014	23	21.8000	85.5612	931.0663	1.3306	0.5255
65	2014	23	20.9714	87.3980	900.1827	0.6520	2.8133
44	2014	23	21.8714	82.1327	908.4898	1.6184	1.3010
75	2014	23	18.0429	75.9286	775.9408	4.6490	2.2745
40	2014	23	22.3857	88.2245	942.6276	2.3561	1.4776
11	2014	23	22.5000	79.9490	875.9071	1.8378	2.0153
35	2014	23	21.5571	84.5510	936.6694	1.4969	1.1418
78	2014	23	23.2143	80.5816	897.3480	1.0143	1.9571
28	2014	23	22.3000	84.1939	926.5296	1.4571	1.1663
39	2014	23	20.9714	87.3980	900.1827	0.6520	2.8133
24	2014	23	22.2857	87.4898	937.3816	0.8459	1.2031
63	2014	23	22.3857	88.2245	942.6276	2.3561	1.4776
62	2014	23	20.8857	81.0408	873.0265	1.5500	1.3235

1	2014	23	20.6571	86.1020	873.3878	1.0357	2.1000
31	2014	24	17.2143	81.1327	844.1133	1.0510	0.8776
79	2014	24	25.3429	88.1837	973.1990	0.8622	0.6337
51	2014	24	23.1571	84.8061	935.1041	1.1020	1.0847
14	2014	24	22.5429	86.3469	893.8327	0.7520	1.8929
67	2014	24	21.7714	89.3061	898.7388	0.6153	2.8653
42	2014	24	20.4857	88.7755	872.0898	0.6286	2.0357
50	2014	24	19.7857	88.0816	898.5163	0.7367	1.2908
43	2014	24	20.4857	88.7755	872.0898	0.6286	2.0357
85	2014	24	19.6000	86.4898	907.1245	0.8633	1.2551
25	2014	24	26.1286	84.6020	971.5449	1.5490	0.8898
69	2014	24	23.9000	87.6633	935.8602	0.6847	1.2398
57	2014	24	19.3143	88.2143	883.7969	0.6449	2.1582
9	2014	24	19.5429	84.1531	851.6378	1.6378	2.3551
72	2014	24	20.9857	82.6327	874.6235	1.2908	1.9969
26	2014	24	20.7857	84.3673	864.1857	3.3990	1.8469
7	2014	24	20.1857	83.2653	856.5500	2.4398	1.7531
83	2014	24	24.7714	80.1429	939.7898	3.2184	1.0224
76	2014	24	22.3571	87.8776	917.5857	0.8796	1.2857
36	2014	24	23.2571	84.3265	924.9816	1.2520	1.1745
81	2014	24	23.1571	84.8061	935.1041	1.1020	1.0847
15	2014	24	22.2571	86.5306	929.6265	1.1510	0.4888
32	2014	24	20.4857	88.7755	872.0898	0.6286	2.0357
73	2014	24	24.2857	88.3163	957.7643	1.2122	0.5316
71	2014	24	23.2571	84.3265	924.9816	1.2520	1.1745
41	2014	24	19.9000	85.8367	871.7704	1.2796	1.1204
10	2014	24	24.2714	85.5306	959.4429	1.4520	0.9327
23	2014	24	14.7429	82.2245	774.9704	2.7082	2.1582
27	2014	24	20.1857	83.2653	856.5500	2.4398	1.7531
60	2014	24	23.1571	84.8061	935.1041	1.1020	1.0847
53	2014	24	19.5429	84.1531	851.6378	1.6378	2.3551
66	2014	24	22.5429	86.3469	893.8327	0.7520	1.8929
59	2014	24	19.3143	88.2143	883.7969	0.6449	2.1582
61	2014	24	24.2857	88.3163	957.7643	1.2122	0.5316
84	2014	24	24.2857	88.3163	957.7643	1.2122	0.5316
38	2014	24	19.3143	88.2143	883.7969	0.6449	2.1582
87	2014	24	22.2000	83.7959	895.9622	0.6776	1.6592
34	2014	24	19.3143	88.2143	883.7969	0.6449	2.1582
29	2014	24	23.9000	87.6633	935.8602	0.6847	1.2398
5	2014	24	18.4857	84.3367	830.1316	3.0643	1.3776
8	2014	24	19.5429	84.1531	851.6378	1.6378	2.3551
12	2014	24	18.4857	84.3367	830.1316	3.0643	1.3776
13	2014	24	24.7714	80.1429	939.7898	3.2184	1.0224
18	2014	24	24.2571	86.1633	959.6929	0.6367	0.7612
33	2014	24	19.7857	88.0816	898.5163	0.7367	1.2908
56	2014	24	26.1286	84.6020	971.5449	1.5490	0.8898
77	2014	24	22.2571	86.5306	929.6265	1.1510	0.4888
54	2014	24	18.4857	84.3367	830.1316	3.0643	1.3776
21	2014	24	19.7857	88.0816	898.5163	0.7367	1.2908
68	2014	24	25.3429	88.1837	973.1990	0.8622	0.6337

74	2014	24	24.2857	88.3163	957.7643	1.2122	0.5316
88	2014	24	20.4857	88.7755	872.0898	0.6286	2.0357
16	2014	24	22.3571	87.8776	917.5857	0.8796	1.2857
30	2014	24	22.5429	86.3469	893.8327	0.7520	1.8929
6	2014	24	25.3429	88.1837	973.1990	0.8622	0.6337
49	2014	24	23.9000	87.6633	935.8602	0.6847	1.2398
22	2014	24	20.4857	88.7755	872.0898	0.6286	2.0357
45	2014	24	17.8857	83.4490	815.5337	2.8867	1.5520
58	2014	24	23.9000	87.6633	935.8602	0.6847	1.2398
37	2014	24	25.3429	88.1837	973.1990	0.8622	0.6337
17	2014	24	21.7714	89.3061	898.7388	0.6153	2.8653
55	2014	24	20.9857	82.6327	874.6235	1.2908	1.9969
46	2014	24	22.3571	87.8776	917.5857	0.8796	1.2857
86	2014	24	20.1857	81.1224	863.3602	1.5286	1.2306
2	2014	24	20.1857	81.1224	863.3602	1.5286	1.2306
4	2014	24	19.7857	88.0816	898.5163	0.7367	1.2908
47	2014	24	25.4429	82.8673	953.6092	1.6888	0.5908
82	2014	24	20.4857	88.7755	872.0898	0.6286	2.0357
19	2014	24	26.4429	82.2449	955.2663	1.3092	1.0643
20	2014	24	19.5429	84.1531	851.6378	1.6378	2.3551
80	2014	24	20.4857	88.7755	872.0898	0.6286	2.0357
3	2014	24	24.7714	80.1429	939.7898	3.2184	1.0224
52	2014	24	21.7714	89.3061	898.7388	0.6153	2.8653
70	2014	24	19.6000	86.4898	907.1245	0.8633	1.2551
64	2014	24	14.7429	82.2245	774.9704	2.7082	2.1582
48	2014	24	22.2571	86.5306	929.6265	1.1510	0.4888
65	2014	24	21.7714	89.3061	898.7388	0.6153	2.8653
44	2014	24	19.6000	86.4898	907.1245	0.8633	1.2551
75	2014	24	14.7429	82.2245	774.9704	2.7082	2.1582
40	2014	24	23.9857	87.2857	940.9735	2.3918	1.4449
11	2014	24	20.9857	82.6327	874.6235	1.2908	1.9969
35	2014	24	23.1571	84.8061	935.1041	1.1020	1.0847
78	2014	24	22.2000	83.7959	895.9622	0.6776	1.6592
28	2014	24	23.2571	84.3265	924.9816	1.2520	1.1745
39	2014	24	21.7714	89.3061	898.7388	0.6153	2.8653
24	2014	24	23.9000	87.6633	935.8602	0.6847	1.2398
63	2014	24	23.9857	87.2857	940.9735	2.3918	1.4449
62	2014	24	19.9000	85.8367	871.7704	1.2796	1.1204
1	2014	24	20.4857	88.7755	872.0898	0.6286	2.0357
31	2014	25	19.7286	84.2551	844.4408	0.5276	0.8949
79	2014	25	24.6143	86.5612	972.5704	1.2694	0.7684
51	2014	25	23.0429	83.8367	934.6500	1.7041	1.0735
14	2014	25	22.3857	83.4898	893.6582	1.5878	1.8122
67	2014	25	21.8143	88.1531	898.5306	1.0684	2.6980
42	2014	25	20.9143	87.1939	872.1296	0.5490	1.9918
50	2014	25	21.1000	88.2143	898.6582	0.5847	1.3255
43	2014	25	20.9143	87.1939	872.1296	0.5490	1.9918
85	2014	25	21.9857	87.5306	907.4673	0.5684	1.2163
25	2014	25	26.2714	83.0816	971.0704	2.0082	0.9867
69	2014	25	23.4714	85.2755	935.5500	1.4663	1.1735

57	2014	25	20.7000	87.0612	883.9378	1.0000	2.0153
9	2014	25	20.6000	82.9388	851.7418	2.0633	2.1969
72	2014	25	21.6571	83.7245	874.7429	1.5347	1.8020
26	2014	25	22.6857	83.8061	864.3500	3.0551	1.7112
7	2014	25	22.3143	83.0918	856.7000	2.5571	1.5867
83	2014	25	25.6429	81.0204	939.9980	2.7184	0.8827
76	2014	25	22.1571	85.2857	917.3480	0.8571	1.2133
36	2014	25	23.2143	83.1531	924.6929	2.1510	1.1265
81	2014	25	23.0429	83.8367	934.6500	1.7041	1.0735
15	2014	25	22.8143	84.0306	929.4653	1.1745	0.6184
32	2014	25	20.9143	87.1939	872.1296	0.5490	1.9918
73	2014	25	24.0714	86.3469	957.2929	1.5653	0.5959
71	2014	25	23.2143	83.1531	924.6929	2.1510	1.1265
41	2014	25	21.2857	85.8673	871.9531	0.8867	0.8694
10	2014	25	23.9429	85.5510	958.9061	1.2214	0.8867
23	2014	25	16.4714	85.0306	775.1051	0.9888	2.2408
27	2014	25	22.3143	83.0918	856.7000	2.5571	1.5867
60	2014	25	23.0429	83.8367	934.6500	1.7041	1.0735
53	2014	25	20.6000	82.9388	851.7418	2.0633	2.1969
66	2014	25	22.3857	83.4898	893.6582	1.5878	1.8122
59	2014	25	20.7000	87.0612	883.9378	1.0000	2.0153
61	2014	25	24.0714	86.3469	957.2929	1.5653	0.5959
84	2014	25	24.0714	86.3469	957.2929	1.5653	0.5959
38	2014	25	20.7000	87.0612	883.9378	1.0000	2.0153
87	2014	25	22.9714	82.8673	896.0214	1.4286	1.7531
34	2014	25	20.7000	87.0612	883.9378	1.0000	2.0153
29	2014	25	23.4714	85.2755	935.5500	1.4663	1.1735
5	2014	25	20.2143	85.2347	830.2837	2.4959	1.2337
8	2014	25	20.6000	82.9388	851.7418	2.0633	2.1969
12	2014	25	20.2143	85.2347	830.2837	2.4959	1.2337
13	2014	25	25.6429	81.0204	939.9980	2.7184	0.8827
18	2014	25	23.9429	83.6735	959.0520	0.9786	0.8857
33	2014	25	21.1000	88.2143	898.6582	0.5847	1.3255
56	2014	25	26.2714	83.0816	971.0704	2.0082	0.9867
77	2014	25	22.8143	84.0306	929.4653	1.1745	0.6184
54	2014	25	20.2143	85.2347	830.2837	2.4959	1.2337
21	2014	25	21.1000	88.2143	898.6582	0.5847	1.3255
68	2014	25	24.6143	86.5612	972.5704	1.2694	0.7684
74	2014	25	24.0714	86.3469	957.2929	1.5653	0.5959
88	2014	25	20.9143	87.1939	872.1296	0.5490	1.9918
16	2014	25	22.1571	85.2857	917.3480	0.8571	1.2133
30	2014	25	22.3857	83.4898	893.6582	1.5878	1.8122
6	2014	25	24.6143	86.5612	972.5704	1.2694	0.7684
49	2014	25	23.4714	85.2755	935.5500	1.4663	1.1735
22	2014	25	20.9143	87.1939	872.1296	0.5490	1.9918
45	2014	25	19.9286	84.9184	815.7173	1.6031	1.3724
58	2014	25	23.4714	85.2755	935.5500	1.4663	1.1735
37	2014	25	24.6143	86.5612	972.5704	1.2694	0.7684
17	2014	25	21.8143	88.1531	898.5306	1.0684	2.6980
55	2014	25	21.6571	83.7245	874.7429	1.5347	1.8020

46	2014	25	22.1571	85.2857	917.3480	0.8571	1.2133
86	2014	25	21.6143	80.9184	863.5653	1.2327	1.2622
2	2014	25	21.6143	80.9184	863.5653	1.2327	1.2622
4	2014	25	21.1000	88.2143	898.6582	0.5847	1.3255
47	2014	25	25.8143	83.3265	953.7204	2.0908	0.4673
82	2014	25	20.9143	87.1939	872.1296	0.5490	1.9918
19	2014	25	25.8571	79.9898	955.0184	2.1102	1.0306
20	2014	25	20.6000	82.9388	851.7418	2.0633	2.1969
80	2014	25	20.9143	87.1939	872.1296	0.5490	1.9918
3	2014	25	25.6429	81.0204	939.9980	2.7184	0.8827
52	2014	25	21.8143	88.1531	898.5306	1.0684	2.6980
70	2014	25	21.9857	87.5306	907.4673	0.5684	1.2163
64	2014	25	16.4714	85.0306	775.1051	0.9888	2.2408
48	2014	25	22.8143	84.0306	929.4653	1.1745	0.6184
65	2014	25	21.8143	88.1531	898.5306	1.0684	2.6980
44	2014	25	21.9857	87.5306	907.4673	0.5684	1.2163
75	2014	25	16.4714	85.0306	775.1051	0.9888	2.2408
40	2014	25	23.8143	84.5000	940.4918	3.2347	1.2327
11	2014	25	21.6571	83.7245	874.7429	1.5347	1.8020
35	2014	25	23.0429	83.8367	934.6500	1.7041	1.0735
78	2014	25	22.9714	82.8673	896.0214	1.4286	1.7531
28	2014	25	23.2143	83.1531	924.6929	2.1510	1.1265
39	2014	25	21.8143	88.1531	898.5306	1.0684	2.6980
24	2014	25	23.4714	85.2755	935.5500	1.4663	1.1735
63	2014	25	23.8143	84.5000	940.4918	3.2347	1.2327
62	2014	25	21.2857	85.8673	871.9531	0.8867	0.8694
1	2014	25	20.9143	87.1939	872.1296	0.5490	1.9918
31	2014	26	19.4429	81.7551	844.0510	0.6571	0.9827
79	2014	26	24.7571	87.6531	972.2163	1.3704	0.7327
51	2014	26	23.1714	84.7551	934.3357	2.1316	1.2061
14	2014	26	22.4429	84.2449	893.5582	2.3153	2.0531
67	2014	26	22.3714	87.5714	898.2816	1.3122	2.7429
42	2014	26	21.4000	85.6939	871.8867	0.6816	2.0827
50	2014	26	20.9857	87.6633	898.2082	0.7214	1.3806
43	2014	26	21.4000	85.6939	871.8867	0.6816	2.0827
85	2014	26	21.7714	84.3265	906.9071	0.8510	1.2949
25	2014	26	25.7857	83.9286	970.9031	2.2980	1.0000
69	2014	26	23.9571	85.3878	935.1429	1.7337	1.1816
57	2014	26	21.0429	83.9898	883.5459	1.0561	2.0418
9	2014	26	20.4857	81.9184	851.5071	2.7806	2.2316
72	2014	26	22.3000	82.7551	874.5622	2.0969	1.7357
26	2014	26	22.1571	82.8980	864.1500	3.8827	1.7429
7	2014	26	21.6286	80.7755	856.4724	3.3347	1.7378
83	2014	26	26.5286	77.8469	939.7214	3.8051	0.9173
76	2014	26	22.5429	85.3163	916.8867	1.0490	1.3337
36	2014	26	23.8143	82.3571	924.3765	2.7643	1.2663
81	2014	26	23.1714	84.7551	934.3357	2.1316	1.2061
15	2014	26	22.5286	83.7245	928.9888	1.0898	0.6163
32	2014	26	21.4000	85.6939	871.8867	0.6816	2.0827
73	2014	26	24.5286	86.0510	956.8265	1.4327	0.6684

71	2014	26	23.8143	82.3571	924.3765	2.7643	1.2663
41	2014	26	21.3000	83.3980	871.5510	0.9867	1.0765
10	2014	26	24.0000	87.3776	958.6286	1.3194	0.8929
23	2014	26	16.5143	83.1327	774.9041	1.6235	2.3592
27	2014	26	21.6286	80.7755	856.4724	3.3347	1.7378
60	2014	26	23.1714	84.7551	934.3357	2.1316	1.2061
53	2014	26	20.4857	81.9184	851.5071	2.7806	2.2316
66	2014	26	22.4429	84.2449	893.5582	2.3153	2.0531
59	2014	26	21.0429	83.9898	883.5459	1.0561	2.0418
61	2014	26	24.5286	86.0510	956.8265	1.4327	0.6684
84	2014	26	24.5286	86.0510	956.8265	1.4327	0.6684
38	2014	26	21.0429	83.9898	883.5459	1.0561	2.0418
87	2014	26	23.2286	80.9286	895.7837	1.8806	1.8745
34	2014	26	21.0429	83.9898	883.5459	1.0561	2.0418
29	2014	26	23.9571	85.3878	935.1429	1.7337	1.1816
5	2014	26	20.0143	83.9388	830.0602	3.1898	1.4041
8	2014	26	20.4857	81.9184	851.5071	2.7806	2.2316
12	2014	26	20.0143	83.9388	830.0602	3.1898	1.4041
13	2014	26	26.5286	77.8469	939.7214	3.8051	0.9173
18	2014	26	23.8429	85.3776	958.6622	0.9173	0.9010
33	2014	26	20.9857	87.6633	898.2082	0.7214	1.3806
56	2014	26	25.7857	83.9286	970.9031	2.2980	1.0000
77	2014	26	22.5286	83.7245	928.9888	1.0898	0.6163
54	2014	26	20.0143	83.9388	830.0602	3.1898	1.4041
21	2014	26	20.9857	87.6633	898.2082	0.7214	1.3806
68	2014	26	24.7571	87.6531	972.2163	1.3704	0.7327
74	2014	26	24.5286	86.0510	956.8265	1.4327	0.6684
88	2014	26	21.4000	85.6939	871.8867	0.6816	2.0827
16	2014	26	22.5429	85.3163	916.8867	1.0490	1.3337
30	2014	26	22.4429	84.2449	893.5582	2.3153	2.0531
6	2014	26	24.7571	87.6531	972.2163	1.3704	0.7327
49	2014	26	23.9571	85.3878	935.1429	1.7337	1.1816
22	2014	26	21.4000	85.6939	871.8867	0.6816	2.0827
45	2014	26	20.0857	81.8673	815.5388	2.2510	1.5429
58	2014	26	23.9571	85.3878	935.1429	1.7337	1.1816
37	2014	26	24.7571	87.6531	972.2163	1.3704	0.7327
17	2014	26	22.3714	87.5714	898.2816	1.3122	2.7429
55	2014	26	22.3000	82.7551	874.5622	2.0969	1.7357
46	2014	26	22.5429	85.3163	916.8867	1.0490	1.3337
86	2014	26	21.7286	78.7653	863.1898	1.5031	1.3092
2	2014	26	21.7286	78.7653	863.1898	1.5031	1.3092
4	2014	26	20.9857	87.6633	898.2082	0.7214	1.3806
47	2014	26	26.2143	82.0204	953.5276	3.1888	0.3878
82	2014	26	21.4000	85.6939	871.8867	0.6816	2.0827
19	2014	26	25.8571	80.5612	954.9684	2.2959	1.0184
20	2014	26	20.4857	81.9184	851.5071	2.7806	2.2316
80	2014	26	21.4000	85.6939	871.8867	0.6816	2.0827
3	2014	26	26.5286	77.8469	939.7214	3.8051	0.9173
52	2014	26	22.3714	87.5714	898.2816	1.3122	2.7429
70	2014	26	21.7714	84.3265	906.9071	0.8510	1.2949

64	2014	26	16.5143	83.1327	774.9041	1.6235	2.3592
48	2014	26	22.5286	83.7245	928.9888	1.0898	0.6163
65	2014	26	22.3714	87.5714	898.2816	1.3122	2.7429
44	2014	26	21.7714	84.3265	906.9071	0.8510	1.2949
75	2014	26	16.5143	83.1327	774.9041	1.6235	2.3592
40	2014	26	23.8286	87.0306	940.2959	3.1980	1.2786
11	2014	26	22.3000	82.7551	874.5622	2.0969	1.7357
35	2014	26	23.1714	84.7551	934.3357	2.1316	1.2061
78	2014	26	23.2286	80.9286	895.7837	1.8806	1.8745
28	2014	26	23.8143	82.3571	924.3765	2.7643	1.2663
39	2014	26	22.3714	87.5714	898.2816	1.3122	2.7429
24	2014	26	23.9571	85.3878	935.1429	1.7337	1.1816
63	2014	26	23.8286	87.0306	940.2959	3.1980	1.2786
62	2014	26	21.3000	83.3980	871.5510	0.9867	1.0765
1	2014	26	21.4000	85.6939	871.8867	0.6816	2.0827
31	2014	27	19.7429	83.6429	844.7765	0.5694	0.8908
79	2014	27	26.1571	90.0714	973.0357	1.0449	0.6929
51	2014	27	24.6714	87.4490	935.0939	1.5643	1.2592
14	2014	27	22.8857	89.1122	894.2735	1.6663	1.8561
67	2014	27	23.3143	90.5714	898.9622	0.7214	2.3092
42	2014	27	21.6429	89.1224	872.5490	0.7163	2.0449
50	2014	27	21.6714	90.0612	898.9010	0.3776	1.1010
43	2014	27	21.6429	89.1224	872.5490	0.7163	2.0449
85	2014	27	22.4714	86.0816	907.5969	0.7235	1.1500
25	2014	27	27.0571	86.4592	971.7163	2.2704	0.9806
69	2014	27	24.5000	89.1429	935.9092	1.4827	1.1163
57	2014	27	21.2429	86.1224	884.2469	0.4184	1.8633
9	2014	27	21.1571	85.8673	852.1520	1.9684	2.0694
72	2014	27	22.5857	84.7857	875.1255	1.6408	1.5245
26	2014	27	22.6571	86.5306	864.6765	3.7408	1.5796
7	2014	27	22.1000	84.7245	857.0153	2.4245	1.6224
83	2014	27	26.8429	80.0102	940.1694	4.9449	0.9908
76	2014	27	23.2286	88.6224	917.6276	0.6449	1.2643
36	2014	27	24.7143	85.6429	925.0827	2.3429	1.2133
81	2014	27	24.6714	87.4490	935.0939	1.5643	1.2592
15	2014	27	24.0000	86.0102	929.6378	0.4041	0.5122
32	2014	27	21.6429	89.1224	872.5490	0.7163	2.0449
73	2014	27	25.4286	88.0000	957.6112	0.7571	0.6847
71	2014	27	24.7143	85.6429	925.0827	2.3429	1.2133
41	2014	27	21.4286	86.1020	872.2469	0.6439	1.1092
10	2014	27	25.8143	89.4592	959.3898	1.2939	0.9235
23	2014	27	16.8000	87.2755	775.5755	1.7347	2.1398
27	2014	27	22.1000	84.7245	857.0153	2.4245	1.6224
60	2014	27	24.6714	87.4490	935.0939	1.5643	1.2592
53	2014	27	21.1571	85.8673	852.1520	1.9684	2.0694
66	2014	27	22.8857	89.1122	894.2735	1.6663	1.8561
59	2014	27	21.2429	86.1224	884.2469	0.4184	1.8633
61	2014	27	25.4286	88.0000	957.6112	0.7571	0.6847
84	2014	27	25.4286	88.0000	957.6112	0.7571	0.6847
38	2014	27	21.2429	86.1224	884.2469	0.4184	1.8633

87	2014	27	23.5571	85.1939	896.3990	1.2020	1.5704
34	2014	27	21.2429	86.1224	884.2469	0.4184	1.8633
29	2014	27	24.5000	89.1429	935.9092	1.4827	1.1163
5	2014	27	20.4143	87.7959	830.6286	2.7153	1.4061
8	2014	27	21.1571	85.8673	852.1520	1.9684	2.0694
12	2014	27	20.4143	87.7959	830.6286	2.7153	1.4061
13	2014	27	26.8429	80.0102	940.1694	4.9449	0.9908
18	2014	27	24.6286	89.5306	959.4918	0.8327	0.8306
33	2014	27	21.6714	90.0612	898.9010	0.3776	1.1010
56	2014	27	27.0571	86.4592	971.7163	2.2704	0.9806
77	2014	27	24.0000	86.0102	929.6378	0.4041	0.5122
54	2014	27	20.4143	87.7959	830.6286	2.7153	1.4061
21	2014	27	21.6714	90.0612	898.9010	0.3776	1.1010
68	2014	27	26.1571	90.0714	973.0357	1.0449	0.6929
74	2014	27	25.4286	88.0000	957.6112	0.7571	0.6847
88	2014	27	21.6429	89.1224	872.5490	0.7163	2.0449
16	2014	27	23.2286	88.6224	917.6276	0.6449	1.2643
30	2014	27	22.8857	89.1122	894.2735	1.6663	1.8561
6	2014	27	26.1571	90.0714	973.0357	1.0449	0.6929
49	2014	27	24.5000	89.1429	935.9092	1.4827	1.1163
22	2014	27	21.6429	89.1224	872.5490	0.7163	2.0449
45	2014	27	20.1429	85.2245	816.0878	1.9082	1.3214
58	2014	27	24.5000	89.1429	935.9092	1.4827	1.1163
37	2014	27	26.1571	90.0714	973.0357	1.0449	0.6929
17	2014	27	23.3143	90.5714	898.9622	0.7214	2.3092
55	2014	27	22.5857	84.7857	875.1255	1.6408	1.5245
46	2014	27	23.2286	88.6224	917.6276	0.6449	1.2643
86	2014	27	21.6714	82.0204	863.8857	1.1837	1.1469
2	2014	27	21.6714	82.0204	863.8857	1.1837	1.1469
4	2014	27	21.6714	90.0612	898.9010	0.3776	1.1010
47	2014	27	27.1286	84.3367	954.0908	3.2827	0.3857
82	2014	27	21.6429	89.1224	872.5490	0.7163	2.0449
19	2014	27	26.6429	84.5204	955.6878	1.9663	0.9551
20	2014	27	21.1571	85.8673	852.1520	1.9684	2.0694
80	2014	27	21.6429	89.1224	872.5490	0.7163	2.0449
3	2014	27	26.8429	80.0102	940.1694	4.9449	0.9908
52	2014	27	23.3143	90.5714	898.9622	0.7214	2.3092
70	2014	27	22.4714	86.0816	907.5969	0.7235	1.1500
64	2014	27	16.8000	87.2755	775.5755	1.7347	2.1398
48	2014	27	24.0000	86.0102	929.6378	0.4041	0.5122
65	2014	27	23.3143	90.5714	898.9622	0.7214	2.3092
44	2014	27	22.4714	86.0816	907.5969	0.7235	1.1500
75	2014	27	16.8000	87.2755	775.5755	1.7347	2.1398
40	2014	27	25.4714	89.2551	941.0857	3.1224	1.4347
11	2014	27	22.5857	84.7857	875.1255	1.6408	1.5245
35	2014	27	24.6714	87.4490	935.0939	1.5643	1.2592
78	2014	27	23.5571	85.1939	896.3990	1.2020	1.5704
28	2014	27	24.7143	85.6429	925.0827	2.3429	1.2133
39	2014	27	23.3143	90.5714	898.9622	0.7214	2.3092
24	2014	27	24.5000	89.1429	935.9092	1.4827	1.1163

63	2014	27	25.4714	89.2551	941.0857	3.1224	1.4347
62	2014	27	21.4286	86.1020	872.2469	0.6439	1.1092
1	2014	27	21.6429	89.1224	872.5490	0.7163	2.0449
31	2014	28	22.7429	83.2857	844.5449	2.8827	0.9133
79	2014	28	28.7429	85.5612	972.3684	3.8847	0.9408
51	2014	28	26.8000	83.9694	934.6929	3.6673	1.3449
14	2014	28	25.0286	88.3265	894.2245	3.3469	1.7857
67	2014	28	25.5714	87.6224	898.7724	2.3439	1.8918
42	2014	28	24.7143	86.2449	872.4173	3.3857	1.8980
50	2014	28	25.3429	85.1939	898.4806	2.9694	1.2520
43	2014	28	24.7143	86.2449	872.4173	3.3857	1.8980
85	2014	28	26.1286	81.1224	907.0327	3.4194	1.2837
25	2014	28	28.9571	83.8061	971.3020	4.6357	1.1214
69	2014	28	27.0429	86.0000	935.4786	4.0133	1.1020
57	2014	28	24.4286	82.8571	883.9337	3.0663	1.8500
9	2014	28	23.0571	84.2245	852.1673	4.3051	1.8908
72	2014	28	24.3857	84.2959	875.0990	3.4969	1.2418
26	2014	28	23.7000	85.9898	864.6837	5.2316	1.4724
7	2014	28	24.0857	84.3061	857.0306	4.4857	1.5347
83	2014	28	27.9429	79.5918	939.9857	7.1133	0.9633
76	2014	28	26.6714	84.4592	917.2327	3.1796	1.2796
36	2014	28	27.2571	81.9898	924.7439	5.1347	1.3490
81	2014	28	26.8000	83.9694	934.6929	3.6673	1.3449
15	2014	28	27.2000	80.3878	928.9663	2.4898	0.7122
32	2014	28	24.7143	86.2449	872.4173	3.3857	1.8980
73	2014	28	28.8286	83.1531	956.9735	3.5337	0.8337
71	2014	28	27.2571	81.9898	924.7439	5.1347	1.3490
41	2014	28	24.6571	84.2653	872.0204	3.0071	1.1714
10	2014	28	27.1286	86.7653	958.8959	3.5265	0.9490
23	2014	28	19.2143	86.3265	775.7061	3.1429	2.0143
27	2014	28	24.0857	84.3061	857.0306	4.4857	1.5347
60	2014	28	26.8000	83.9694	934.6929	3.6673	1.3449
53	2014	28	23.0571	84.2245	852.1673	4.3051	1.8908
66	2014	28	25.0286	88.3265	894.2245	3.3469	1.7857
59	2014	28	24.4286	82.8571	883.9337	3.0663	1.8500
61	2014	28	28.8286	83.1531	956.9735	3.5337	0.8337
84	2014	28	28.8286	83.1531	956.9735	3.5337	0.8337
38	2014	28	24.4286	82.8571	883.9337	3.0663	1.8500
87	2014	28	26.2429	83.6531	896.2265	3.1959	1.2500
34	2014	28	24.4286	82.8571	883.9337	3.0663	1.8500
29	2014	28	27.0429	86.0000	935.4786	4.0133	1.1020
5	2014	28	22.1143	86.5816	830.6918	4.5571	1.3622
8	2014	28	23.0571	84.2245	852.1673	4.3051	1.8908
12	2014	28	22.1143	86.5816	830.6918	4.5571	1.3622
13	2014	28	27.9429	79.5918	939.9857	7.1133	0.9633
18	2014	28	27.6143	85.4184	958.8857	3.3000	0.8878
33	2014	28	25.3429	85.1939	898.4806	2.9694	1.2520
56	2014	28	28.9571	83.8061	971.3020	4.6357	1.1214
77	2014	28	27.2000	80.3878	928.9663	2.4898	0.7122
54	2014	28	22.1143	86.5816	830.6918	4.5571	1.3622

21	2014	28	25.3429	85.1939	898.4806	2.9694	1.2520
68	2014	28	28.7429	85.5612	972.3684	3.8847	0.9408
74	2014	28	28.8286	83.1531	956.9735	3.5337	0.8337
88	2014	28	24.7143	86.2449	872.4173	3.3857	1.8980
16	2014	28	26.6714	84.4592	917.2327	3.1796	1.2796
30	2014	28	25.0286	88.3265	894.2245	3.3469	1.7857
6	2014	28	28.7429	85.5612	972.3684	3.8847	0.9408
49	2014	28	27.0429	86.0000	935.4786	4.0133	1.1020
22	2014	28	24.7143	86.2449	872.4173	3.3857	1.8980
45	2014	28	21.3143	84.9286	816.1969	3.0786	1.0745
58	2014	28	27.0429	86.0000	935.4786	4.0133	1.1020
37	2014	28	28.7429	85.5612	972.3684	3.8847	0.9408
17	2014	28	25.5714	87.6224	898.7724	2.3439	1.8918
55	2014	28	24.3857	84.2959	875.0990	3.4969	1.2418
46	2014	28	26.6714	84.4592	917.2327	3.1796	1.2796
86	2014	28	24.9286	80.5918	863.6959	3.6786	0.8765
2	2014	28	24.9286	80.5918	863.6959	3.6786	0.8765
4	2014	28	25.3429	85.1939	898.4806	2.9694	1.2520
47	2014	28	28.3714	83.2959	953.8194	5.1265	0.4367
82	2014	28	24.7143	86.2449	872.4173	3.3857	1.8980
19	2014	28	28.0857	85.0408	955.4602	4.5673	0.9694
20	2014	28	23.0571	84.2245	852.1673	4.3051	1.8908
80	2014	28	24.7143	86.2449	872.4173	3.3857	1.8980
3	2014	28	27.9429	79.5918	939.9857	7.1133	0.9633
52	2014	28	25.5714	87.6224	898.7724	2.3439	1.8918
70	2014	28	26.1286	81.1224	907.0327	3.4194	1.2837
64	2014	28	19.2143	86.3265	775.7061	3.1429	2.0143
48	2014	28	27.2000	80.3878	928.9663	2.4898	0.7122
65	2014	28	25.5714	87.6224	898.7724	2.3439	1.8918
44	2014	28	26.1286	81.1224	907.0327	3.4194	1.2837
75	2014	28	19.2143	86.3265	775.7061	3.1429	2.0143
40	2014	28	27.1000	85.0000	940.7929	4.8439	1.4347
11	2014	28	24.3857	84.2959	875.0990	3.4969	1.2418
35	2014	28	26.8000	83.9694	934.6929	3.6673	1.3449
78	2014	28	26.2429	83.6531	896.2265	3.1959	1.2500
28	2014	28	27.2571	81.9898	924.7439	5.1347	1.3490
39	2014	28	25.5714	87.6224	898.7724	2.3439	1.8918
24	2014	28	27.0429	86.0000	935.4786	4.0133	1.1020
63	2014	28	27.1000	85.0000	940.7929	4.8439	1.4347
62	2014	28	24.6571	84.2653	872.0204	3.0071	1.1714
1	2014	28	24.7143	86.2449	872.4173	3.3857	1.8980
31	2014	29	19.8714	83.8061	844.4357	3.4102	0.9571
79	2014	29	24.9000	85.7245	972.5153	4.6255	1.0041
51	2014	29	23.8429	83.1837	934.8469	4.2990	1.4388
14	2014	29	22.8857	85.5714	894.4888	4.0918	2.1245
67	2014	29	22.4857	85.8776	898.9755	2.8367	2.4061
42	2014	29	21.2714	83.8061	872.5153	3.9694	2.0316
50	2014	29	22.3714	83.0612	898.3847	4.1520	1.3724
43	2014	29	21.2714	83.8061	872.5153	3.9694	2.0316
85	2014	29	22.8429	80.4898	906.8194	4.4500	1.5622

25	2014	29	26.7429	82.4592	971.5724	5.2520	1.0571
69	2014	29	23.1000	85.8980	935.6020	4.1316	1.1378
57	2014	29	20.9714	82.1735	884.0020	4.0255	2.0112
9	2014	29	20.6429	82.2143	852.2735	4.8102	2.2143
72	2014	29	22.0000	82.7143	875.3439	4.0020	1.5939
26	2014	29	21.7286	85.1939	864.9184	5.2571	1.6265
7	2014	29	21.2000	81.4490	857.2357	5.4735	1.7816
83	2014	29	25.9571	78.7041	940.3082	6.5184	0.8776
76	2014	29	22.3571	82.9286	917.3122	4.0296	1.3806
36	2014	29	23.8571	80.0510	924.8959	5.9245	1.4663
81	2014	29	23.8429	83.1837	934.8469	4.2990	1.4388
15	2014	29	23.8143	78.7143	928.9510	3.2602	0.9184
32	2014	29	21.2714	83.8061	872.5153	3.9694	2.0316
73	2014	29	23.9571	82.8878	957.0510	4.1418	0.8643
71	2014	29	23.8571	80.0510	924.8959	5.9245	1.4663
41	2014	29	21.2000	83.8571	871.9612	3.5204	1.2194
10	2014	29	25.2286	86.2245	959.0990	4.0439	0.9245
23	2014	29	16.5286	83.8980	775.8122	3.5031	2.0592
27	2014	29	21.2000	81.4490	857.2357	5.4735	1.7816
60	2014	29	23.8429	83.1837	934.8469	4.2990	1.4388
53	2014	29	20.6429	82.2143	852.2735	4.8102	2.2143
66	2014	29	22.8857	85.5714	894.4888	4.0918	2.1245
59	2014	29	20.9714	82.1735	884.0020	4.0255	2.0112
61	2014	29	23.9571	82.8878	957.0510	4.1418	0.8643
84	2014	29	23.9571	82.8878	957.0510	4.1418	0.8643
38	2014	29	20.9714	82.1735	884.0020	4.0255	2.0112
87	2014	29	22.9143	80.9898	896.4061	3.9031	1.4520
34	2014	29	20.9714	82.1735	884.0020	4.0255	2.0112
29	2014	29	23.1000	85.8980	935.6020	4.1316	1.1378
5	2014	29	19.5000	84.9184	830.8520	4.7408	1.3929
8	2014	29	20.6429	82.2143	852.2735	4.8102	2.2143
12	2014	29	19.5000	84.9184	830.8520	4.7408	1.3929
13	2014	29	25.9571	78.7041	940.3082	6.5184	0.8776
18	2014	29	23.7429	86.0918	959.0051	3.8612	0.7867
33	2014	29	22.3714	83.0612	898.3847	4.1520	1.3724
56	2014	29	26.7429	82.4592	971.5724	5.2520	1.0571
77	2014	29	23.8143	78.7143	928.9510	3.2602	0.9184
54	2014	29	19.5000	84.9184	830.8520	4.7408	1.3929
21	2014	29	22.3714	83.0612	898.3847	4.1520	1.3724
68	2014	29	24.9000	85.7245	972.5153	4.6255	1.0041
74	2014	29	23.9571	82.8878	957.0510	4.1418	0.8643
88	2014	29	21.2714	83.8061	872.5153	3.9694	2.0316
16	2014	29	22.3571	82.9286	917.3122	4.0296	1.3806
30	2014	29	22.8857	85.5714	894.4888	4.0918	2.1245
6	2014	29	24.9000	85.7245	972.5153	4.6255	1.0041
49	2014	29	23.1000	85.8980	935.6020	4.1316	1.1378
22	2014	29	21.2714	83.8061	872.5153	3.9694	2.0316
45	2014	29	19.3286	83.5816	816.4000	3.2041	1.2133
58	2014	29	23.1000	85.8980	935.6020	4.1316	1.1378
37	2014	29	24.9000	85.7245	972.5153	4.6255	1.0041

17	2014	29	22.4857	85.8776	898.9755	2.8367	2.4061
55	2014	29	22.0000	82.7143	875.3439	4.0020	1.5939
46	2014	29	22.3571	82.9286	917.3122	4.0296	1.3806
86	2014	29	21.1143	78.8571	863.6255	4.3612	0.7163
2	2014	29	21.1143	78.8571	863.6255	4.3612	0.7163
4	2014	29	22.3714	83.0612	898.3847	4.1520	1.3724
47	2014	29	25.5286	83.2653	954.1214	5.7908	0.4816
82	2014	29	21.2714	83.8061	872.5153	3.9694	2.0316
19	2014	29	26.4143	83.1429	955.7908	5.4194	1.1633
20	2014	29	20.6429	82.2143	852.2735	4.8102	2.2143
80	2014	29	21.2714	83.8061	872.5153	3.9694	2.0316
3	2014	29	25.9571	78.7041	940.3082	6.5184	0.8776
52	2014	29	22.4857	85.8776	898.9755	2.8367	2.4061
70	2014	29	22.8429	80.4898	906.8194	4.4500	1.5622
64	2014	29	16.5286	83.8980	775.8122	3.5031	2.0592
48	2014	29	23.8143	78.7143	928.9510	3.2602	0.9184
65	2014	29	22.4857	85.8776	898.9755	2.8367	2.4061
44	2014	29	22.8429	80.4898	906.8194	4.4500	1.5622
75	2014	29	16.5286	83.8980	775.8122	3.5031	2.0592
40	2014	29	25.8429	82.4694	941.0306	4.7867	1.5061
11	2014	29	22.0000	82.7143	875.3439	4.0020	1.5939
35	2014	29	23.8429	83.1837	934.8469	4.2990	1.4388
78	2014	29	22.9143	80.9898	896.4061	3.9031	1.4520
28	2014	29	23.8571	80.0510	924.8959	5.9245	1.4663
39	2014	29	22.4857	85.8776	898.9755	2.8367	2.4061
24	2014	29	23.1000	85.8980	935.6020	4.1316	1.1378
63	2014	29	25.8429	82.4694	941.0306	4.7867	1.5061
62	2014	29	21.2000	83.8571	871.9612	3.5204	1.2194
1	2014	29	21.2714	83.8061	872.5153	3.9694	2.0316
31	2014	30	22.4286	84.8673	845.6347	3.6082	0.8765
79	2014	30	28.7286	87.2857	973.7653	4.1704	0.9745
51	2014	30	25.9714	84.2347	936.0765	4.2000	1.3653
14	2014	30	23.4714	85.4286	895.5816	4.2245	2.2082
67	2014	30	23.9571	87.6429	900.2102	2.5735	2.3439
42	2014	30	23.3429	86.2755	873.6980	2.9194	2.0224
50	2014	30	25.7714	81.7857	899.6092	5.1347	1.3898
43	2014	30	23.3429	86.2755	873.6980	2.9194	2.0224
85	2014	30	26.5857	80.1122	908.0878	5.2255	1.5429
25	2014	30	27.7714	82.8265	972.8337	5.0480	0.9735
69	2014	30	26.0286	88.0612	936.9449	3.5245	1.2082
57	2014	30	24.3857	81.7449	885.5143	4.3245	1.9602
9	2014	30	22.2714	84.4694	853.3408	3.6612	2.1133
72	2014	30	22.7571	84.8980	876.4255	3.1255	1.8041
26	2014	30	22.2429	87.3469	865.9163	4.0439	1.6112
7	2014	30	21.6000	84.8673	858.2561	4.0786	1.8429
83	2014	30	26.7429	80.1327	941.4898	4.8265	0.9276
76	2014	30	26.4857	83.3673	918.5898	4.7010	1.3643
36	2014	30	25.0857	83.2653	926.1724	4.4735	1.2255
81	2014	30	25.9714	84.2347	936.0765	4.2000	1.3653
15	2014	30	27.6714	78.9184	930.2398	4.1673	0.8296

32	2014	30	23.3429	86.2755	873.6980	2.9194	2.0224
73	2014	30	28.1143	84.7755	958.4153	3.9622	0.8071
71	2014	30	25.0857	83.2653	926.1724	4.4735	1.2255
41	2014	30	24.0143	85.4898	873.2082	2.7827	1.2265
10	2014	30	27.1143	85.1939	960.3031	4.2031	1.0959
23	2014	30	18.2571	84.1224	776.8337	3.3653	1.8888
27	2014	30	21.6000	84.8673	858.2561	4.0786	1.8429
60	2014	30	25.9714	84.2347	936.0765	4.2000	1.3653
53	2014	30	22.2714	84.4694	853.3408	3.6612	2.1133
66	2014	30	23.4714	85.4286	895.5816	4.2245	2.2082
59	2014	30	24.3857	81.7449	885.5143	4.3245	1.9602
61	2014	30	28.1143	84.7755	958.4153	3.9622	0.8071
84	2014	30	28.1143	84.7755	958.4153	3.9622	0.8071
38	2014	30	24.3857	81.7449	885.5143	4.3245	1.9602
87	2014	30	23.9714	84.1531	897.6408	3.2051	1.4622
34	2014	30	24.3857	81.7449	885.5143	4.3245	1.9602
29	2014	30	26.0286	88.0612	936.9449	3.5245	1.2082
5	2014	30	20.5571	88.2347	831.8571	3.2888	1.5255
8	2014	30	22.2714	84.4694	853.3408	3.6612	2.1133
12	2014	30	20.5571	88.2347	831.8571	3.2888	1.5255
13	2014	30	26.7429	80.1327	941.4898	4.8265	0.9276
18	2014	30	27.7429	87.1224	960.2857	3.5071	0.8061
33	2014	30	25.7714	81.7857	899.6092	5.1347	1.3898
56	2014	30	27.7714	82.8265	972.8337	5.0480	0.9735
77	2014	30	27.6714	78.9184	930.2398	4.1673	0.8296
54	2014	30	20.5571	88.2347	831.8571	3.2888	1.5255
21	2014	30	25.7714	81.7857	899.6092	5.1347	1.3898
68	2014	30	28.7286	87.2857	973.7653	4.1704	0.9745
74	2014	30	28.1143	84.7755	958.4153	3.9622	0.8071
88	2014	30	23.3429	86.2755	873.6980	2.9194	2.0224
16	2014	30	26.4857	83.3673	918.5898	4.7010	1.3643
30	2014	30	23.4714	85.4286	895.5816	4.2245	2.2082
6	2014	30	28.7286	87.2857	973.7653	4.1704	0.9745
49	2014	30	26.0286	88.0612	936.9449	3.5245	1.2082
22	2014	30	23.3429	86.2755	873.6980	2.9194	2.0224
45	2014	30	19.6857	86.1429	817.3724	2.3500	1.2622
58	2014	30	26.0286	88.0612	936.9449	3.5245	1.2082
37	2014	30	28.7286	87.2857	973.7653	4.1704	0.9745
17	2014	30	23.9571	87.6429	900.2102	2.5735	2.3439
55	2014	30	22.7571	84.8980	876.4255	3.1255	1.8041
46	2014	30	26.4857	83.3673	918.5898	4.7010	1.3643
86	2014	30	23.3571	82.1633	864.8847	3.7469	0.7153
2	2014	30	23.3571	82.1633	864.8847	3.7469	0.7153
4	2014	30	25.7714	81.7857	899.6092	5.1347	1.3898
47	2014	30	26.8286	85.8367	955.4306	4.4582	0.4439
82	2014	30	23.3429	86.2755	873.6980	2.9194	2.0224
19	2014	30	26.5857	83.1633	956.9806	4.7857	1.1908
20	2014	30	22.2714	84.4694	853.3408	3.6612	2.1133
80	2014	30	23.3429	86.2755	873.6980	2.9194	2.0224
3	2014	30	26.7429	80.1327	941.4898	4.8265	0.9276

52	2014	30	23.9571	87.6429	900.2102	2.5735	2.3439
70	2014	30	26.5857	80.1122	908.0878	5.2255	1.5429
64	2014	30	18.2571	84.1224	776.8337	3.3653	1.8888
48	2014	30	27.6714	78.9184	930.2398	4.1673	0.8296
65	2014	30	23.9571	87.6429	900.2102	2.5735	2.3439
44	2014	30	26.5857	80.1122	908.0878	5.2255	1.5429
75	2014	30	18.2571	84.1224	776.8337	3.3653	1.8888
40	2014	30	26.3571	81.5000	942.1459	5.3582	1.4929
11	2014	30	22.7571	84.8980	876.4255	3.1255	1.8041
35	2014	30	25.9714	84.2347	936.0765	4.2000	1.3653
78	2014	30	23.9714	84.1531	897.6408	3.2051	1.4622
28	2014	30	25.0857	83.2653	926.1724	4.4735	1.2255
39	2014	30	23.9571	87.6429	900.2102	2.5735	2.3439
24	2014	30	26.0286	88.0612	936.9449	3.5245	1.2082
63	2014	30	26.3571	81.5000	942.1459	5.3582	1.4929
62	2014	30	24.0143	85.4898	873.2082	2.7827	1.2265
1	2014	30	23.3429	86.2755	873.6980	2.9194	2.0224
31	2014	31	23.4429	77.8673	845.5643	7.0643	0.9765
79	2014	31	29.7571	81.3776	973.4194	6.3694	1.1194
51	2014	31	26.3571	81.3878	936.0357	6.5071	1.2398
14	2014	31	24.2143	85.0918	895.7061	5.6847	1.8878
67	2014	31	24.7857	85.5714	900.3122	4.6112	1.9704
42	2014	31	24.1143	83.3163	873.8153	4.2061	1.9571
50	2014	31	26.3857	73.8980	899.3939	8.2898	1.5745
43	2014	31	24.1143	83.3163	873.8153	4.2061	1.9571
85	2014	31	27.4000	70.1122	907.7469	8.5418	1.5898
25	2014	31	27.8429	80.6020	972.8847	6.1296	1.1378
69	2014	31	27.2429	83.1020	936.7969	6.6612	1.2980
57	2014	31	25.4143	73.3980	885.3296	7.3704	2.3643
9	2014	31	22.8429	80.7857	853.5112	6.1418	1.8857
72	2014	31	23.6714	83.4286	876.5765	5.1694	1.5224
26	2014	31	23.1571	85.6224	866.1949	5.4622	1.5796
7	2014	31	22.8714	85.1429	858.5276	4.9163	1.5704
83	2014	31	27.6571	77.5000	941.6449	7.1765	1.0184
76	2014	31	26.6143	77.1224	918.3490	8.0214	1.4398
36	2014	31	26.8571	79.9898	926.2245	6.3357	1.2429
81	2014	31	26.3571	81.3878	936.0357	6.5071	1.2398
15	2014	31	28.6429	71.1224	929.8265	8.0929	0.8439
32	2014	31	24.1143	83.3163	873.8153	4.2061	1.9571
73	2014	31	29.3286	77.3878	958.0367	7.4143	1.0888
71	2014	31	26.8571	79.9898	926.2245	6.3357	1.2429
41	2014	31	24.6429	78.8061	873.1939	6.1020	1.5980
10	2014	31	27.5429	81.2041	960.1592	6.3541	1.1531
23	2014	31	18.4571	81.6122	777.0969	5.1031	1.8071
27	2014	31	22.8714	85.1429	858.5276	4.9163	1.5704
60	2014	31	26.3571	81.3878	936.0357	6.5071	1.2398
53	2014	31	22.8429	80.7857	853.5112	6.1418	1.8857
66	2014	31	24.2143	85.0918	895.7061	5.6847	1.8878
59	2014	31	25.4143	73.3980	885.3296	7.3704	2.3643
61	2014	31	29.3286	77.3878	958.0367	7.4143	1.0888

84	2014	31	29.3286	77.3878	958.0367	7.4143	1.0888
38	2014	31	25.4143	73.3980	885.3296	7.3704	2.3643
87	2014	31	25.2571	82.6735	897.8041	4.6306	1.1969
34	2014	31	25.4143	73.3980	885.3296	7.3704	2.3643
29	2014	31	27.2429	83.1020	936.7969	6.6612	1.2980
5	2014	31	21.4857	85.7041	832.1235	5.2714	1.5224
8	2014	31	22.8429	80.7857	853.5112	6.1418	1.8857
12	2014	31	21.4857	85.7041	832.1235	5.2714	1.5224
13	2014	31	27.6571	77.5000	941.6449	7.1765	1.0184
18	2014	31	28.4714	79.9694	959.9347	6.0173	1.1041
33	2014	31	26.3857	73.8980	899.3939	8.2898	1.5745
56	2014	31	27.8429	80.6020	972.8847	6.1296	1.1378
77	2014	31	28.6429	71.1224	929.8265	8.0929	0.8439
54	2014	31	21.4857	85.7041	832.1235	5.2714	1.5224
21	2014	31	26.3857	73.8980	899.3939	8.2898	1.5745
68	2014	31	29.7571	81.3776	973.4194	6.3694	1.1194
74	2014	31	29.3286	77.3878	958.0367	7.4143	1.0888
88	2014	31	24.1143	83.3163	873.8153	4.2061	1.9571
16	2014	31	26.6143	77.1224	918.3490	8.0214	1.4398
30	2014	31	24.2143	85.0918	895.7061	5.6847	1.8878
6	2014	31	29.7571	81.3776	973.4194	6.3694	1.1194
49	2014	31	27.2429	83.1020	936.7969	6.6612	1.2980
22	2014	31	24.1143	83.3163	873.8153	4.2061	1.9571
45	2014	31	21.0143	83.5102	817.6429	4.2357	1.2561
58	2014	31	27.2429	83.1020	936.7969	6.6612	1.2980
37	2014	31	29.7571	81.3776	973.4194	6.3694	1.1194
17	2014	31	24.7857	85.5714	900.3122	4.6112	1.9704
55	2014	31	23.6714	83.4286	876.5765	5.1694	1.5224
46	2014	31	26.6143	77.1224	918.3490	8.0214	1.4398
86	2014	31	24.3714	77.9184	864.9316	6.7571	0.7684
2	2014	31	24.3714	77.9184	864.9316	6.7571	0.7684
4	2014	31	26.3857	73.8980	899.3939	8.2898	1.5745
47	2014	31	28.0143	82.4082	955.5439	5.7480	0.4449
82	2014	31	24.1143	83.3163	873.8153	4.2061	1.9571
19	2014	31	27.4857	82.7041	957.1061	5.9724	1.1408
20	2014	31	22.8429	80.7857	853.5112	6.1418	1.8857
80	2014	31	24.1143	83.3163	873.8153	4.2061	1.9571
3	2014	31	27.6571	77.5000	941.6449	7.1765	1.0184
52	2014	31	24.7857	85.5714	900.3122	4.6112	1.9704
70	2014	31	27.4000	70.1122	907.7469	8.5418	1.5898
64	2014	31	18.4571	81.6122	777.0969	5.1031	1.8071
48	2014	31	28.6429	71.1224	929.8265	8.0929	0.8439
65	2014	31	24.7857	85.5714	900.3122	4.6112	1.9704
44	2014	31	27.4000	70.1122	907.7469	8.5418	1.5898
75	2014	31	18.4571	81.6122	777.0969	5.1031	1.8071
40	2014	31	26.9714	80.3163	942.1082	7.5347	1.1939
11	2014	31	23.6714	83.4286	876.5765	5.1694	1.5224
35	2014	31	26.3571	81.3878	936.0357	6.5071	1.2398
78	2014	31	25.2571	82.6735	897.8041	4.6306	1.1969
28	2014	31	26.8571	79.9898	926.2245	6.3357	1.2429

39	2014	31	24.7857	85.5714	900.3122	4.6112	1.9704
24	2014	31	27.2429	83.1020	936.7969	6.6612	1.2980
63	2014	31	26.9714	80.3163	942.1082	7.5347	1.1939
62	2014	31	24.6429	78.8061	873.1939	6.1020	1.5980
1	2014	31	24.1143	83.3163	873.8153	4.2061	1.9571
31	2014	32	23.8714	74.3367	844.2214	8.1908	1.1163
79	2014	32	28.6571	77.0816	971.9531	7.6347	1.2531
51	2014	32	26.5286	79.5102	934.7133	7.7245	1.2480
14	2014	32	25.2000	83.2857	894.6173	6.5724	1.7316
67	2014	32	25.5286	82.8776	899.0939	6.3888	2.1837
42	2014	32	25.6000	78.0510	872.5959	5.2520	2.0112
50	2014	32	27.5571	70.2857	897.9071	8.7745	1.6582
43	2014	32	25.6000	78.0510	872.5959	5.2520	2.0112
85	2014	32	28.6857	65.8061	906.1388	9.2133	1.8990
25	2014	32	28.4571	79.8980	971.5857	6.9735	1.2122
69	2014	32	27.3857	79.2857	935.3194	8.2235	1.3918
57	2014	32	26.4000	68.5612	883.6918	8.3357	2.5388
9	2014	32	24.1857	76.7959	852.4286	8.0755	2.0622
72	2014	32	25.0143	80.0408	875.4735	7.3061	1.3296
26	2014	32	24.5429	82.7143	865.2510	7.2867	1.7571
7	2014	32	24.0571	81.5612	857.5612	6.8643	1.5633
83	2014	32	28.6429	75.1429	940.4612	9.3704	0.9959
76	2014	32	27.3143	73.8163	916.8969	8.8439	1.5092
36	2014	32	26.5000	75.6327	924.9184	8.3082	1.3582
81	2014	32	26.5286	79.5102	934.7133	7.7245	1.2480
15	2014	32	29.1286	66.2755	928.2020	8.8449	0.9724
32	2014	32	25.6000	78.0510	872.5959	5.2520	2.0112
73	2014	32	29.3571	72.1939	956.4163	8.7510	1.3714
71	2014	32	26.5000	75.6327	924.9184	8.3082	1.3582
41	2014	32	25.9286	75.4898	871.8551	8.4082	1.6867
10	2014	32	26.8714	80.4490	958.8255	7.1531	1.0724
23	2014	32	19.7143	81.7245	776.1643	5.7143	1.8531
27	2014	32	24.0571	81.5612	857.5612	6.8643	1.5633
60	2014	32	26.5286	79.5102	934.7133	7.7245	1.2480
53	2014	32	24.1857	76.7959	852.4286	8.0755	2.0622
66	2014	32	25.2000	83.2857	894.6173	6.5724	1.7316
59	2014	32	26.4000	68.5612	883.6918	8.3357	2.5388
61	2014	32	29.3571	72.1939	956.4163	8.7510	1.3714
84	2014	32	29.3571	72.1939	956.4163	8.7510	1.3714
38	2014	32	26.4000	68.5612	883.6918	8.3357	2.5388
87	2014	32	26.5571	79.0714	896.5939	6.2939	1.0163
34	2014	32	26.4000	68.5612	883.6918	8.3357	2.5388
29	2014	32	27.3857	79.2857	935.3194	8.2235	1.3918
5	2014	32	22.1571	82.6939	831.1867	7.2959	1.2847
8	2014	32	24.1857	76.7959	852.4286	8.0755	2.0622
12	2014	32	22.1571	82.6939	831.1867	7.2959	1.2847
13	2014	32	28.6429	75.1429	940.4612	9.3704	0.9959
18	2014	32	27.1000	77.6633	958.4541	6.4194	1.2224
33	2014	32	27.5571	70.2857	897.9071	8.7745	1.6582
56	2014	32	28.4571	79.8980	971.5857	6.9735	1.2122

77	2014	32	29.1286	66.2755	928.2020	8.8449	0.9724
54	2014	32	22.1571	82.6939	831.1867	7.2959	1.2847
21	2014	32	27.5571	70.2857	897.9071	8.7745	1.6582
68	2014	32	28.6571	77.0816	971.9531	7.6347	1.2531
74	2014	32	29.3571	72.1939	956.4163	8.7510	1.3714
88	2014	32	25.6000	78.0510	872.5959	5.2520	2.0112
16	2014	32	27.3143	73.8163	916.8969	8.8439	1.5092
30	2014	32	25.2000	83.2857	894.6173	6.5724	1.7316
6	2014	32	28.6571	77.0816	971.9531	7.6347	1.2531
49	2014	32	27.3857	79.2857	935.3194	8.2235	1.3918
22	2014	32	25.6000	78.0510	872.5959	5.2520	2.0112
45	2014	32	21.5714	80.8776	816.7653	6.0806	1.3765
58	2014	32	27.3857	79.2857	935.3194	8.2235	1.3918
37	2014	32	28.6571	77.0816	971.9531	7.6347	1.2531
17	2014	32	25.5286	82.8776	899.0939	6.3888	2.1837
55	2014	32	25.0143	80.0408	875.4735	7.3061	1.3296
46	2014	32	27.3143	73.8163	916.8969	8.8439	1.5092
86	2014	32	25.8143	73.4184	863.6143	8.5561	0.7398
2	2014	32	25.8143	73.4184	863.6143	8.5561	0.7398
4	2014	32	27.5571	70.2857	897.9071	8.7745	1.6582
47	2014	32	29.5000	77.4592	954.1480	7.9643	0.5133
82	2014	32	25.6000	78.0510	872.5959	5.2520	2.0112
19	2014	32	28.0143	81.1735	955.8724	7.0918	1.1561
20	2014	32	24.1857	76.7959	852.4286	8.0755	2.0622
80	2014	32	25.6000	78.0510	872.5959	5.2520	2.0112
3	2014	32	28.6429	75.1429	940.4612	9.3704	0.9959
52	2014	32	25.5286	82.8776	899.0939	6.3888	2.1837
70	2014	32	28.6857	65.8061	906.1388	9.2133	1.8990
64	2014	32	19.7143	81.7245	776.1643	5.7143	1.8531
48	2014	32	29.1286	66.2755	928.2020	8.8449	0.9724
65	2014	32	25.5286	82.8776	899.0939	6.3888	2.1837
44	2014	32	28.6857	65.8061	906.1388	9.2133	1.8990
75	2014	32	19.7143	81.7245	776.1643	5.7143	1.8531
40	2014	32	26.4429	79.6020	940.8908	8.1143	1.0490
11	2014	32	25.0143	80.0408	875.4735	7.3061	1.3296
35	2014	32	26.5286	79.5102	934.7133	7.7245	1.2480
78	2014	32	26.5571	79.0714	896.5939	6.2939	1.0163
28	2014	32	26.5000	75.6327	924.9184	8.3082	1.3582
39	2014	32	25.5286	82.8776	899.0939	6.3888	2.1837
24	2014	32	27.3857	79.2857	935.3194	8.2235	1.3918
63	2014	32	26.4429	79.6020	940.8908	8.1143	1.0490
62	2014	32	25.9286	75.4898	871.8551	8.4082	1.6867
1	2014	32	25.6000	78.0510	872.5959	5.2520	2.0112
31	2014	33	19.4143	78.4490	843.6990	5.3398	1.0990
79	2014	33	24.3857	79.4184	971.9520	4.8704	1.1888
51	2014	33	22.7571	81.4592	934.2184	5.4531	1.4480
14	2014	33	21.9000	82.7041	893.7153	5.0061	2.0673
67	2014	33	21.9429	83.4490	898.2755	5.1000	2.4582
42	2014	33	21.6000	76.4388	871.7959	4.3480	2.3561
50	2014	33	22.3286	73.2449	897.3296	6.5092	1.9602

43	2014	33	21.6000	76.4388	871.7959	4.3480	2.3561
85	2014	33	23.1000	69.0612	905.6541	6.7847	2.0418
25	2014	33	25.1714	82.2449	970.8214	5.6245	1.0929
69	2014	33	23.8000	80.9082	934.6959	5.8347	1.4306
57	2014	33	21.5571	69.0612	883.0316	6.7745	2.6602
9	2014	33	21.2000	75.3980	851.6276	6.3633	2.4398
72	2014	33	22.0429	78.8265	874.5827	6.1367	1.6102
26	2014	33	22.0286	81.7143	864.3551	5.9031	1.8918
7	2014	33	21.3857	80.6224	856.6990	5.7867	1.7439
83	2014	33	25.9000	75.9184	939.4745	7.3816	1.0327
76	2014	33	22.9286	77.1327	916.3265	6.5857	1.6010
36	2014	33	23.1000	78.8878	924.2071	6.3653	1.3378
81	2014	33	22.7571	81.4592	934.2184	5.4531	1.4480
15	2014	33	24.6143	68.4082	927.6357	5.7745	1.1735
32	2014	33	21.6000	76.4388	871.7959	4.3480	2.3561
73	2014	33	25.2000	74.0102	956.0337	6.0286	1.3612
71	2014	33	23.1000	78.8878	924.2071	6.3653	1.3378
41	2014	33	22.1857	75.4898	871.1622	6.6929	1.7071
10	2014	33	23.4857	83.8469	958.4796	4.3796	1.1143
23	2014	33	15.7571	84.3061	775.4480	4.1520	2.1714
27	2014	33	21.3857	80.6224	856.6990	5.7867	1.7439
60	2014	33	22.7571	81.4592	934.2184	5.4531	1.4480
53	2014	33	21.2000	75.3980	851.6276	6.3633	2.4398
66	2014	33	21.9000	82.7041	893.7153	5.0061	2.0673
59	2014	33	21.5571	69.0612	883.0316	6.7745	2.6602
61	2014	33	25.2000	74.0102	956.0337	6.0286	1.3612
84	2014	33	25.2000	74.0102	956.0337	6.0286	1.3612
38	2014	33	21.5571	69.0612	883.0316	6.7745	2.6602
87	2014	33	23.1714	77.3163	895.7051	5.4969	1.3000
34	2014	33	21.5571	69.0612	883.0316	6.7745	2.6602
29	2014	33	23.8000	80.9082	934.6959	5.8347	1.4306
5	2014	33	19.8857	83.2959	830.3673	5.4918	1.1520
8	2014	33	21.2000	75.3980	851.6276	6.3633	2.4398
12	2014	33	19.8857	83.2959	830.3673	5.4918	1.1520
13	2014	33	25.9000	75.9184	939.4745	7.3816	1.0327
18	2014	33	23.5429	80.4082	958.4051	3.4724	1.2622
33	2014	33	22.3286	73.2449	897.3296	6.5092	1.9602
56	2014	33	25.1714	82.2449	970.8214	5.6245	1.0929
77	2014	33	24.6143	68.4082	927.6357	5.7745	1.1735
54	2014	33	19.8857	83.2959	830.3673	5.4918	1.1520
21	2014	33	22.3286	73.2449	897.3296	6.5092	1.9602
68	2014	33	24.3857	79.4184	971.9520	4.8704	1.1888
74	2014	33	25.2000	74.0102	956.0337	6.0286	1.3612
88	2014	33	21.6000	76.4388	871.7959	4.3480	2.3561
16	2014	33	22.9286	77.1327	916.3265	6.5857	1.6010
30	2014	33	21.9000	82.7041	893.7153	5.0061	2.0673
6	2014	33	24.3857	79.4184	971.9520	4.8704	1.1888
49	2014	33	23.8000	80.9082	934.6959	5.8347	1.4306
22	2014	33	21.6000	76.4388	871.7959	4.3480	2.3561
45	2014	33	19.2000	81.8061	815.9388	4.5194	1.5408

58	2014	33	23.8000	80.9082	934.6959	5.8347	1.4306
37	2014	33	24.3857	79.4184	971.9520	4.8704	1.1888
17	2014	33	21.9429	83.4490	898.2755	5.1000	2.4582
55	2014	33	22.0429	78.8265	874.5827	6.1367	1.6102
46	2014	33	22.9286	77.1327	916.3265	6.5857	1.6010
86	2014	33	22.0571	73.0816	862.9000	6.3347	0.7408
2	2014	33	22.0571	73.0816	862.9000	6.3347	0.7408
4	2014	33	22.3286	73.2449	897.3296	6.5092	1.9602
47	2014	33	26.4429	75.3061	953.1112	6.5367	0.5306
82	2014	33	21.6000	76.4388	871.7959	4.3480	2.3561
19	2014	33	25.6429	80.8878	954.8888	5.3622	1.1531
20	2014	33	21.2000	75.3980	851.6276	6.3633	2.4398
80	2014	33	21.6000	76.4388	871.7959	4.3480	2.3561
3	2014	33	25.9000	75.9184	939.4745	7.3816	1.0327
52	2014	33	21.9429	83.4490	898.2755	5.1000	2.4582
70	2014	33	23.1000	69.0612	905.6541	6.7847	2.0418
64	2014	33	15.7571	84.3061	775.4480	4.1520	2.1714
48	2014	33	24.6143	68.4082	927.6357	5.7745	1.1735
65	2014	33	21.9429	83.4490	898.2755	5.1000	2.4582
44	2014	33	23.1000	69.0612	905.6541	6.7847	2.0418
75	2014	33	15.7571	84.3061	775.4480	4.1520	2.1714
40	2014	33	22.7857	84.2449	940.2786	5.6847	1.2510
11	2014	33	22.0429	78.8265	874.5827	6.1367	1.6102
35	2014	33	22.7571	81.4592	934.2184	5.4531	1.4480
78	2014	33	23.1714	77.3163	895.7051	5.4969	1.3000
28	2014	33	23.1000	78.8878	924.2071	6.3653	1.3378
39	2014	33	21.9429	83.4490	898.2755	5.1000	2.4582
24	2014	33	23.8000	80.9082	934.6959	5.8347	1.4306
63	2014	33	22.7857	84.2449	940.2786	5.6847	1.2510
62	2014	33	22.1857	75.4898	871.1622	6.6929	1.7071
1	2014	33	21.6000	76.4388	871.7959	4.3480	2.3561
31	2014	34	19.2857	81.6939	845.8000	3.2837	0.9520
79	2014	34	25.2571	83.3469	974.8592	2.4122	1.0112
51	2014	34	22.8143	83.0714	936.6643	3.5745	1.5214
14	2014	34	21.4143	84.0306	895.6837	2.7276	2.1643
67	2014	34	21.3714	85.4898	900.3837	2.6327	2.6541
42	2014	34	20.5143	79.2959	873.7714	2.9704	2.6214
50	2014	34	21.3286	77.6429	899.8194	4.1745	1.9633
43	2014	34	20.5143	79.2959	873.7714	2.9704	2.6214
85	2014	34	22.1429	75.0000	908.3143	4.3388	1.7112
25	2014	34	25.4714	84.0102	973.4408	3.5082	0.9459
69	2014	34	23.3143	82.7449	937.2469	3.5837	1.3163
57	2014	34	20.4286	72.9388	885.2735	4.4776	2.7898
9	2014	34	20.0286	75.8469	853.4061	4.2459	2.5265
72	2014	34	21.3714	79.0000	876.4582	4.2112	2.0531
26	2014	34	21.7000	81.9490	866.1480	4.0071	1.9816
7	2014	34	21.3571	79.9388	858.4724	4.0133	1.8449
83	2014	34	25.1429	78.0612	941.7796	4.8663	1.0245
76	2014	34	22.2857	80.5306	918.8194	4.3949	1.5837
36	2014	34	22.8714	81.3980	926.5214	3.8510	1.2878

81	2014	34	22.8143	83.0714	936.6643	3.5745	1.5214
15	2014	34	23.1571	73.2551	930.4429	3.6265	1.1684
32	2014	34	20.5143	79.2959	873.7714	2.9704	2.6214
73	2014	34	24.5000	78.0714	958.9143	3.8245	1.2408
71	2014	34	22.8714	81.3980	926.5214	3.8510	1.2878
41	2014	34	20.6857	76.9592	873.2653	4.6612	1.5908
10	2014	34	24.1286	85.7245	961.0837	2.4163	1.0571
23	2014	34	15.8143	84.8673	776.8959	2.7020	2.3857
27	2014	34	21.3571	79.9388	858.4724	4.0133	1.8449
60	2014	34	22.8143	83.0714	936.6643	3.5745	1.5214
53	2014	34	20.0286	75.8469	853.4061	4.2459	2.5265
66	2014	34	21.4143	84.0306	895.6837	2.7276	2.1643
59	2014	34	20.4286	72.9388	885.2735	4.4776	2.7898
61	2014	34	24.5000	78.0714	958.9143	3.8245	1.2408
84	2014	34	24.5000	78.0714	958.9143	3.8245	1.2408
38	2014	34	20.4286	72.9388	885.2735	4.4776	2.7898
87	2014	34	22.0429	78.2449	897.7459	3.5153	1.7286
34	2014	34	20.4286	72.9388	885.2735	4.4776	2.7898
29	2014	34	23.3143	82.7449	937.2469	3.5837	1.3163
5	2014	34	19.5000	81.6429	831.9857	3.8041	1.2306
8	2014	34	20.0286	75.8469	853.4061	4.2459	2.5265
12	2014	34	19.5000	81.6429	831.9857	3.8041	1.2306
13	2014	34	25.1429	78.0612	941.7796	4.8663	1.0245
18	2014	34	23.7429	82.7857	961.2286	2.1878	1.1724
33	2014	34	21.3286	77.6429	899.8194	4.1745	1.9633
56	2014	34	25.4714	84.0102	973.4408	3.5082	0.9459
77	2014	34	23.1571	73.2551	930.4429	3.6265	1.1684
54	2014	34	19.5000	81.6429	831.9857	3.8041	1.2306
21	2014	34	21.3286	77.6429	899.8194	4.1745	1.9633
68	2014	34	25.2571	83.3469	974.8592	2.4122	1.0112
74	2014	34	24.5000	78.0714	958.9143	3.8245	1.2408
88	2014	34	20.5143	79.2959	873.7714	2.9704	2.6214
16	2014	34	22.2857	80.5306	918.8194	4.3949	1.5837
30	2014	34	21.4143	84.0306	895.6837	2.7276	2.1643
6	2014	34	25.2571	83.3469	974.8592	2.4122	1.0112
49	2014	34	23.3143	82.7449	937.2469	3.5837	1.3163
22	2014	34	20.5143	79.2959	873.7714	2.9704	2.6214
45	2014	34	19.2286	80.7959	817.4724	3.3367	1.5939
58	2014	34	23.3143	82.7449	937.2469	3.5837	1.3163
37	2014	34	25.2571	83.3469	974.8592	2.4122	1.0112
17	2014	34	21.3714	85.4898	900.3837	2.6327	2.6541
55	2014	34	21.3714	79.0000	876.4582	4.2112	2.0531
46	2014	34	22.2857	80.5306	918.8194	4.3949	1.5837
86	2014	34	20.8571	74.5510	864.9265	3.9673	0.7469
2	2014	34	20.8571	74.5510	864.9265	3.9673	0.7469
4	2014	34	21.3286	77.6429	899.8194	4.1745	1.9633
47	2014	34	24.9143	78.0918	955.5969	3.9337	0.4724
82	2014	34	20.5143	79.2959	873.7714	2.9704	2.6214
19	2014	34	25.1000	81.7959	957.3235	3.1592	1.1316
20	2014	34	20.0286	75.8469	853.4061	4.2459	2.5265

80	2014	34	20.5143	79.2959	873.7714	2.9704	2.6214
3	2014	34	25.1429	78.0612	941.7796	4.8663	1.0245
52	2014	34	21.3714	85.4898	900.3837	2.6327	2.6541
70	2014	34	22.1429	75.0000	908.3143	4.3388	1.7112
64	2014	34	15.8143	84.8673	776.8959	2.7020	2.3857
48	2014	34	23.1571	73.2551	930.4429	3.6265	1.1684
65	2014	34	21.3714	85.4898	900.3837	2.6327	2.6541
44	2014	34	22.1429	75.0000	908.3143	4.3388	1.7112
75	2014	34	15.8143	84.8673	776.8959	2.7020	2.3857
40	2014	34	23.9429	87.5612	942.6735	3.6592	1.4714
11	2014	34	21.3714	79.0000	876.4582	4.2112	2.0531
35	2014	34	22.8143	83.0714	936.6643	3.5745	1.5214
78	2014	34	22.0429	78.2449	897.7459	3.5153	1.7286
28	2014	34	22.8714	81.3980	926.5214	3.8510	1.2878
39	2014	34	21.3714	85.4898	900.3837	2.6327	2.6541
24	2014	34	23.3143	82.7449	937.2469	3.5837	1.3163
63	2014	34	23.9429	87.5612	942.6735	3.6592	1.4714
62	2014	34	20.6857	76.9592	873.2653	4.6612	1.5908
1	2014	34	20.5143	79.2959	873.7714	2.9704	2.6214
31	2014	35	20.7286	81.0000	848.1959	3.3837	0.9000
79	2014	35	26.7571	84.2041	977.4888	3.5510	0.9582
51	2014	35	23.3000	83.3265	939.4388	4.0653	1.3306
14	2014	35	22.5286	84.7857	898.4653	3.5939	1.9724
67	2014	35	22.7143	86.4490	903.2041	2.5796	2.4153
42	2014	35	22.3429	82.0918	876.4276	3.9827	2.2122
50	2014	35	23.2429	79.6327	902.6520	3.3092	1.6949
43	2014	35	22.3429	82.0918	876.4276	3.9827	2.2122
85	2014	35	24.0143	78.1429	911.1704	4.0755	1.6184
25	2014	35	26.0143	83.7449	976.4969	3.8714	0.9765
69	2014	35	24.7143	82.7959	940.2143	3.5694	1.1551
57	2014	35	22.8143	76.6327	887.9745	3.9286	2.4837
9	2014	35	21.3000	78.0714	855.9082	4.5531	2.4194
72	2014	35	22.1429	80.0000	879.1204	4.6969	1.9918
26	2014	35	21.9143	82.2959	868.7061	4.5051	1.9459
7	2014	35	21.7286	78.6327	860.9939	5.0643	1.8969
83	2014	35	25.6714	79.3878	944.8531	5.1796	0.9755
76	2014	35	24.2429	80.7755	921.6959	3.8276	1.4520
36	2014	35	24.1000	80.6020	929.4000	4.0490	1.1602
81	2014	35	23.3000	83.3265	939.4388	4.0653	1.3306
15	2014	35	25.0143	76.3163	933.5204	3.4020	0.8857
32	2014	35	22.3429	82.0918	876.4276	3.9827	2.2122
73	2014	35	26.3429	80.0714	961.8796	3.9857	1.1020
71	2014	35	24.1000	80.6020	929.4000	4.0490	1.1602
41	2014	35	23.1429	79.9694	875.8745	4.6480	1.4602
10	2014	35	25.3143	84.3163	963.8592	3.5480	1.1020
23	2014	35	17.0429	83.4082	778.8857	2.5837	2.3184
27	2014	35	21.7286	78.6327	860.9939	5.0643	1.8969
60	2014	35	23.3000	83.3265	939.4388	4.0653	1.3306
53	2014	35	21.3000	78.0714	855.9082	4.5531	2.4194
66	2014	35	22.5286	84.7857	898.4653	3.5939	1.9724

59	2014	35	22.8143	76.6327	887.9745	3.9286	2.4837
61	2014	35	26.3429	80.0714	961.8796	3.9857	1.1020
84	2014	35	26.3429	80.0714	961.8796	3.9857	1.1020
38	2014	35	22.8143	76.6327	887.9745	3.9286	2.4837
87	2014	35	23.3857	80.7449	900.5602	3.9235	1.6112
34	2014	35	22.8143	76.6327	887.9745	3.9286	2.4837
29	2014	35	24.7143	82.7959	940.2143	3.5694	1.1551
5	2014	35	20.2286	80.3061	834.3622	4.7071	1.3398
8	2014	35	21.3000	78.0714	855.9082	4.5531	2.4194
12	2014	35	20.2286	80.3061	834.3622	4.7071	1.3398
13	2014	35	25.6714	79.3878	944.8531	5.1796	0.9755
18	2014	35	25.3143	84.3673	963.8500	3.1980	0.9388
33	2014	35	23.2429	79.6327	902.6520	3.3092	1.6949
56	2014	35	26.0143	83.7449	976.4969	3.8714	0.9765
77	2014	35	25.0143	76.3163	933.5204	3.4020	0.8857
54	2014	35	20.2286	80.3061	834.3622	4.7071	1.3398
21	2014	35	23.2429	79.6327	902.6520	3.3092	1.6949
68	2014	35	26.7571	84.2041	977.4888	3.5510	0.9582
74	2014	35	26.3429	80.0714	961.8796	3.9857	1.1020
88	2014	35	22.3429	82.0918	876.4276	3.9827	2.2122
16	2014	35	24.2429	80.7755	921.6959	3.8276	1.4520
30	2014	35	22.5286	84.7857	898.4653	3.5939	1.9724
6	2014	35	26.7571	84.2041	977.4888	3.5510	0.9582
49	2014	35	24.7143	82.7959	940.2143	3.5694	1.1551
22	2014	35	22.3429	82.0918	876.4276	3.9827	2.2122
45	2014	35	19.4286	80.0102	819.7694	4.0735	1.4837
58	2014	35	24.7143	82.7959	940.2143	3.5694	1.1551
37	2014	35	26.7571	84.2041	977.4888	3.5510	0.9582
17	2014	35	22.7143	86.4490	903.2041	2.5796	2.4153
55	2014	35	22.1429	80.0000	879.1204	4.6969	1.9918
46	2014	35	24.2429	80.7755	921.6959	3.8276	1.4520
86	2014	35	22.7714	76.5714	867.5010	4.4378	0.7184
2	2014	35	22.7714	76.5714	867.5010	4.4378	0.7184
4	2014	35	23.2429	79.6327	902.6520	3.3092	1.6949
47	2014	35	25.6286	82.8571	958.8418	4.1357	0.4918
82	2014	35	22.3429	82.0918	876.4276	3.9827	2.2122
19	2014	35	25.9714	81.9082	960.4296	3.8163	1.0755
20	2014	35	21.3000	78.0714	855.9082	4.5531	2.4194
80	2014	35	22.3429	82.0918	876.4276	3.9827	2.2122
3	2014	35	25.6714	79.3878	944.8531	5.1796	0.9755
52	2014	35	22.7143	86.4490	903.2041	2.5796	2.4153
70	2014	35	24.0143	78.1429	911.1704	4.0755	1.6184
64	2014	35	17.0429	83.4082	778.8857	2.5837	2.3184
48	2014	35	25.0143	76.3163	933.5204	3.4020	0.8857
65	2014	35	22.7143	86.4490	903.2041	2.5796	2.4153
44	2014	35	24.0143	78.1429	911.1704	4.0755	1.6184
75	2014	35	17.0429	83.4082	778.8857	2.5837	2.3184
40	2014	35	24.7000	85.4184	945.5214	4.6357	1.3878
11	2014	35	22.1429	80.0000	879.1204	4.6969	1.9918
35	2014	35	23.3000	83.3265	939.4388	4.0653	1.3306

78	2014	35	23.3857	80.7449	900.5602	3.9235	1.6112
28	2014	35	24.1000	80.6020	929.4000	4.0490	1.1602
39	2014	35	22.7143	86.4490	903.2041	2.5796	2.4153
24	2014	35	24.7143	82.7959	940.2143	3.5694	1.1551
63	2014	35	24.7000	85.4184	945.5214	4.6357	1.3878
62	2014	35	23.1429	79.9694	875.8745	4.6480	1.4602
1	2014	35	22.3429	82.0918	876.4276	3.9827	2.2122
31	2014	36	20.5286	79.2143	848.8316	3.7347	0.9582
79	2014	36	25.5714	81.9796	978.2010	4.8367	1.0704
51	2014	36	23.8286	81.4184	940.3337	5.3204	1.3367
14	2014	36	23.2429	83.8469	899.5765	5.2429	2.0102
67	2014	36	22.6143	85.1939	904.1878	3.6439	2.3684
42	2014	36	22.0000	81.2347	877.3571	5.3112	2.0857
50	2014	36	22.4714	78.3571	903.3204	4.0224	1.8724
43	2014	36	22.0000	81.2347	877.3571	5.3112	2.0857
85	2014	36	23.4286	76.3061	911.8214	4.9878	1.9296
25	2014	36	26.8143	82.5510	977.5031	5.2816	1.1235
69	2014	36	24.1857	80.4694	941.0357	5.0082	1.2439
57	2014	36	21.6286	74.6633	888.7469	4.9184	2.8327
9	2014	36	21.4286	78.0000	856.8806	5.8327	2.5571
72	2014	36	22.6857	80.0918	880.1776	6.0847	1.7143
26	2014	36	22.7857	82.2653	869.7520	5.7245	1.9265
7	2014	36	22.2429	78.5510	862.0051	6.4541	1.8255
83	2014	36	26.9714	78.1429	945.9327	7.2898	0.9745
76	2014	36	22.6857	78.6020	922.4327	4.4398	1.5071
36	2014	36	24.3571	77.3265	930.3133	5.5224	1.2082
81	2014	36	23.8286	81.4184	940.3337	5.3204	1.3367
15	2014	36	23.5857	75.6531	934.1500	3.5051	0.8571
32	2014	36	22.0000	81.2347	877.3571	5.3112	2.0857
73	2014	36	25.0000	78.3163	962.5480	4.8092	1.1561
71	2014	36	24.3571	77.3265	930.3133	5.5224	1.2082
41	2014	36	21.9000	77.8878	876.6714	5.2020	1.8020
10	2014	36	24.9286	81.2653	964.7347	5.2531	1.2816
23	2014	36	17.3571	82.3776	779.5786	3.1878	2.3837
27	2014	36	22.2429	78.5510	862.0051	6.4541	1.8255
60	2014	36	23.8286	81.4184	940.3337	5.3204	1.3367
53	2014	36	21.4286	78.0000	856.8806	5.8327	2.5571
66	2014	36	23.2429	83.8469	899.5765	5.2429	2.0102
59	2014	36	21.6286	74.6633	888.7469	4.9184	2.8327
61	2014	36	25.0000	78.3163	962.5480	4.8092	1.1561
84	2014	36	25.0000	78.3163	962.5480	4.8092	1.1561
38	2014	36	21.6286	74.6633	888.7469	4.9184	2.8327
87	2014	36	24.2857	79.8469	901.5653	5.5327	1.4255
34	2014	36	21.6286	74.6633	888.7469	4.9184	2.8327
29	2014	36	24.1857	80.4694	941.0357	5.0082	1.2439
5	2014	36	20.7286	80.4082	835.3031	6.3602	1.4684
8	2014	36	21.4286	78.0000	856.8806	5.8327	2.5571
12	2014	36	20.7286	80.4082	835.3031	6.3602	1.4684
13	2014	36	26.9714	78.1429	945.9327	7.2898	0.9745
18	2014	36	24.0429	82.7347	964.5184	3.8918	0.9969

33	2014	36	22.4714	78.3571	903.3204	4.0224	1.8724
56	2014	36	26.8143	82.5510	977.5031	5.2816	1.1235
77	2014	36	23.5857	75.6531	934.1500	3.5051	0.8571
54	2014	36	20.7286	80.4082	835.3031	6.3602	1.4684
21	2014	36	22.4714	78.3571	903.3204	4.0224	1.8724
68	2014	36	25.5714	81.9796	978.2010	4.8367	1.0704
74	2014	36	25.0000	78.3163	962.5480	4.8092	1.1561
88	2014	36	22.0000	81.2347	877.3571	5.3112	2.0857
16	2014	36	22.6857	78.6020	922.4327	4.4398	1.5071
30	2014	36	23.2429	83.8469	899.5765	5.2429	2.0102
6	2014	36	25.5714	81.9796	978.2010	4.8367	1.0704
49	2014	36	24.1857	80.4694	941.0357	5.0082	1.2439
22	2014	36	22.0000	81.2347	877.3571	5.3112	2.0857
45	2014	36	20.4143	79.9082	820.6684	4.7888	1.4031
58	2014	36	24.1857	80.4694	941.0357	5.0082	1.2439
37	2014	36	25.5714	81.9796	978.2010	4.8367	1.0704
17	2014	36	22.6143	85.1939	904.1878	3.6439	2.3684
55	2014	36	22.6857	80.0918	880.1776	6.0847	1.7143
46	2014	36	22.6857	78.6020	922.4327	4.4398	1.5071
86	2014	36	22.1857	75.2755	868.3112	5.0480	0.7357
2	2014	36	22.1857	75.2755	868.3112	5.0480	0.7357
4	2014	36	22.4714	78.3571	903.3204	4.0224	1.8724
47	2014	36	26.5000	82.7041	959.9429	5.8265	0.6041
82	2014	36	22.0000	81.2347	877.3571	5.3112	2.0857
19	2014	36	26.8714	81.1224	961.5092	5.5847	0.9684
20	2014	36	21.4286	78.0000	856.8806	5.8327	2.5571
80	2014	36	22.0000	81.2347	877.3571	5.3112	2.0857
3	2014	36	26.9714	78.1429	945.9327	7.2898	0.9745
52	2014	36	22.6143	85.1939	904.1878	3.6439	2.3684
70	2014	36	23.4286	76.3061	911.8214	4.9878	1.9296
64	2014	36	17.3571	82.3776	779.5786	3.1878	2.3837
48	2014	36	23.5857	75.6531	934.1500	3.5051	0.8571
65	2014	36	22.6143	85.1939	904.1878	3.6439	2.3684
44	2014	36	23.4286	76.3061	911.8214	4.9878	1.9296
75	2014	36	17.3571	82.3776	779.5786	3.1878	2.3837
40	2014	36	24.7000	82.2551	946.5602	6.2602	1.3571
11	2014	36	22.6857	80.0918	880.1776	6.0847	1.7143
35	2014	36	23.8286	81.4184	940.3337	5.3204	1.3367
78	2014	36	24.2857	79.8469	901.5653	5.5327	1.4255
28	2014	36	24.3571	77.3265	930.3133	5.5224	1.2082
39	2014	36	22.6143	85.1939	904.1878	3.6439	2.3684
24	2014	36	24.1857	80.4694	941.0357	5.0082	1.2439
63	2014	36	24.7000	82.2551	946.5602	6.2602	1.3571
62	2014	36	21.9000	77.8878	876.6714	5.2020	1.8020
1	2014	36	22.0000	81.2347	877.3571	5.3112	2.0857
31	2014	37	23.9000	77.2449	847.1449	5.4888	1.0235
79	2014	37	26.8714	81.7245	976.8265	5.0296	1.0265
51	2014	37	24.7286	79.4898	938.9480	6.2816	1.4265
14	2014	37	23.9000	82.2245	898.3184	6.1612	2.1704
67	2014	37	24.3000	84.3061	902.8214	4.7827	2.5194

42	2014	37	24.4429	78.8265	875.9643	6.3092	2.2622
50	2014	37	26.5143	75.0408	901.6204	5.6837	2.2255
43	2014	37	24.4429	78.8265	875.9643	6.3092	2.2622
85	2014	37	28.1429	71.0918	909.8592	6.8286	2.1020
25	2014	37	27.5571	81.1531	976.0133	6.0847	1.1745
69	2014	37	26.5286	78.6429	939.4969	5.8510	1.4949
57	2014	37	25.7571	71.2143	887.1776	6.4408	3.2490
9	2014	37	23.4714	77.3265	855.5796	7.2439	2.5969
72	2014	37	24.0714	79.8878	878.8561	7.2388	1.7061
26	2014	37	23.8286	82.0102	868.4643	6.7276	1.9755
7	2014	37	23.9286	78.1633	860.6908	7.3571	1.8520
83	2014	37	28.3000	75.7653	944.3490	8.4347	0.9633
76	2014	37	26.2429	77.7245	920.9367	5.6388	1.5449
36	2014	37	25.8429	75.2653	928.8571	6.3714	1.4173
81	2014	37	24.7286	79.4898	938.9480	6.2816	1.4265
15	2014	37	27.5429	74.8469	932.5082	3.8102	0.9939
32	2014	37	24.4429	78.8265	875.9643	6.3092	2.2622
73	2014	37	27.8714	77.4388	961.0020	5.6520	1.1214
71	2014	37	25.8429	75.2653	928.8571	6.3714	1.4173
41	2014	37	25.5714	75.9898	875.1194	6.3092	1.8776
10	2014	37	25.7143	80.6735	963.3469	6.1857	1.1847
23	2014	37	19.3857	81.2449	778.3704	4.9684	2.5378
27	2014	37	23.9286	78.1633	860.6908	7.3571	1.8520
60	2014	37	24.7286	79.4898	938.9480	6.2816	1.4265
53	2014	37	23.4714	77.3265	855.5796	7.2439	2.5969
66	2014	37	23.9000	82.2245	898.3184	6.1612	2.1704
59	2014	37	25.7571	71.2143	887.1776	6.4408	3.2490
61	2014	37	27.8714	77.4388	961.0020	5.6520	1.1214
84	2014	37	27.8714	77.4388	961.0020	5.6520	1.1214
38	2014	37	25.7571	71.2143	887.1776	6.4408	3.2490
87	2014	37	25.6857	77.2551	900.1010	6.8806	1.3612
34	2014	37	25.7571	71.2143	887.1776	6.4408	3.2490
29	2014	37	26.5286	78.6429	939.4969	5.8510	1.4949
5	2014	37	21.9857	80.3980	834.0490	7.3347	1.4980
8	2014	37	23.4714	77.3265	855.5796	7.2439	2.5969
12	2014	37	21.9857	80.3980	834.0490	7.3347	1.4980
13	2014	37	28.3000	75.7653	944.3490	8.4347	0.9633
18	2014	37	26.3714	82.0204	963.1694	4.3857	1.0071
33	2014	37	26.5143	75.0408	901.6204	5.6837	2.2255
56	2014	37	27.5571	81.1531	976.0133	6.0847	1.1745
77	2014	37	27.5429	74.8469	932.5082	3.8102	0.9939
54	2014	37	21.9857	80.3980	834.0490	7.3347	1.4980
21	2014	37	26.5143	75.0408	901.6204	5.6837	2.2255
68	2014	37	26.8714	81.7245	976.8265	5.0296	1.0265
74	2014	37	27.8714	77.4388	961.0020	5.6520	1.1214
88	2014	37	24.4429	78.8265	875.9643	6.3092	2.2622
16	2014	37	26.2429	77.7245	920.9367	5.6388	1.5449
30	2014	37	23.9000	82.2245	898.3184	6.1612	2.1704
6	2014	37	26.8714	81.7245	976.8265	5.0296	1.0265
49	2014	37	26.5286	78.6429	939.4969	5.8510	1.4949

22	2014	37	24.4429	78.8265	875.9643	6.3092	2.2622
45	2014	37	21.5286	79.3469	819.4633	5.4276	1.5388
58	2014	37	26.5286	78.6429	939.4969	5.8510	1.4949
37	2014	37	26.8714	81.7245	976.8265	5.0296	1.0265
17	2014	37	24.3000	84.3061	902.8214	4.7827	2.5194
55	2014	37	24.0714	79.8878	878.8561	7.2388	1.7061
46	2014	37	26.2429	77.7245	920.9367	5.6388	1.5449
86	2014	37	24.9143	74.0000	866.8082	5.8000	0.7622
2	2014	37	24.9143	74.0000	866.8082	5.8000	0.7622
4	2014	37	26.5143	75.0408	901.6204	5.6837	2.2255
47	2014	37	27.5714	80.4490	958.3276	6.8847	0.6092
82	2014	37	24.4429	78.8265	875.9643	6.3092	2.2622
19	2014	37	27.0429	80.7041	960.0857	6.3378	0.9592
20	2014	37	23.4714	77.3265	855.5796	7.2439	2.5969
80	2014	37	24.4429	78.8265	875.9643	6.3092	2.2622
3	2014	37	28.3000	75.7653	944.3490	8.4347	0.9633
52	2014	37	24.3000	84.3061	902.8214	4.7827	2.5194
70	2014	37	28.1429	71.0918	909.8592	6.8286	2.1020
64	2014	37	19.3857	81.2449	778.3704	4.9684	2.5378
48	2014	37	27.5429	74.8469	932.5082	3.8102	0.9939
65	2014	37	24.3000	84.3061	902.8214	4.7827	2.5194
44	2014	37	28.1429	71.0918	909.8592	6.8286	2.1020
75	2014	37	19.3857	81.2449	778.3704	4.9684	2.5378
40	2014	37	26.2000	80.3469	945.2163	6.6163	1.3704
11	2014	37	24.0714	79.8878	878.8561	7.2388	1.7061
35	2014	37	24.7286	79.4898	938.9480	6.2816	1.4265
78	2014	37	25.6857	77.2551	900.1010	6.8806	1.3612
28	2014	37	25.8429	75.2653	928.8571	6.3714	1.4173
39	2014	37	24.3000	84.3061	902.8214	4.7827	2.5194
24	2014	37	26.5286	78.6429	939.4969	5.8510	1.4949
63	2014	37	26.2000	80.3469	945.2163	6.6163	1.3704
62	2014	37	25.5714	75.9898	875.1194	6.3092	1.8776
1	2014	37	24.4429	78.8265	875.9643	6.3092	2.2622
31	2014	38	17.9571	78.9184	846.3337	5.2806	1.0255
79	2014	38	22.3286	84.0408	976.9102	3.1092	0.9480
51	2014	38	19.5143	84.5000	938.5459	4.0408	1.5796
14	2014	38	20.9571	84.4184	897.1786	4.5429	2.3061
67	2014	38	19.9429	87.9286	901.8918	3.7663	2.8031
42	2014	38	19.8143	80.6939	874.9694	5.6653	2.6010
50	2014	38	20.5714	76.6633	900.8663	4.7010	2.4051
43	2014	38	19.8143	80.6939	874.9694	5.6653	2.6010
85	2014	38	20.2714	73.8265	909.0796	6.1204	2.1847
25	2014	38	24.3857	81.2551	975.0551	4.3612	1.2765
69	2014	38	22.1286	79.6633	938.8551	4.0327	1.7092
57	2014	38	19.5429	73.2449	886.3000	6.1255	2.9847
9	2014	38	19.3571	79.2245	854.5000	6.4878	2.6469
72	2014	38	20.7571	80.6837	877.6122	5.9327	2.1276
26	2014	38	21.3143	83.1837	867.0388	5.8551	2.0816
7	2014	38	20.3571	79.9388	859.3939	5.9510	1.9163
83	2014	38	25.4571	76.7143	942.7347	5.8765	0.9939

76	2014	38	20.9429	80.3776	920.3102	4.4265	1.7367
36	2014	38	20.7143	79.6531	928.1276	4.5286	1.5847
81	2014	38	19.5143	84.5000	938.5459	4.0408	1.5796
15	2014	38	23.0857	74.6939	931.8684	2.8602	1.2306
32	2014	38	19.8143	80.6939	874.9694	5.6653	2.6010
73	2014	38	23.8571	78.2449	960.5929	4.0969	1.1276
71	2014	38	20.7143	79.6531	928.1276	4.5286	1.5847
41	2014	38	19.7571	78.0612	874.1806	5.8235	1.8490
10	2014	38	21.2286	84.8673	963.0745	3.5796	1.2490
23	2014	38	15.4857	82.2347	777.5112	5.6459	2.6327
27	2014	38	20.3571	79.9388	859.3939	5.9510	1.9163
60	2014	38	19.5143	84.5000	938.5459	4.0408	1.5796
53	2014	38	19.3571	79.2245	854.5000	6.4878	2.6469
66	2014	38	20.9571	84.4184	897.1786	4.5429	2.3061
59	2014	38	19.5429	73.2449	886.3000	6.1255	2.9847
61	2014	38	23.8571	78.2449	960.5929	4.0969	1.1276
84	2014	38	23.8571	78.2449	960.5929	4.0969	1.1276
38	2014	38	19.5429	73.2449	886.3000	6.1255	2.9847
87	2014	38	21.8714	78.6633	898.9694	5.5622	1.4755
34	2014	38	19.5429	73.2449	886.3000	6.1255	2.9847
29	2014	38	22.1286	79.6633	938.8551	4.0327	1.7092
5	2014	38	18.2143	83.0408	832.9296	5.7541	1.5612
8	2014	38	19.3571	79.2245	854.5000	6.4878	2.6469
12	2014	38	18.2143	83.0408	832.9296	5.7541	1.5612
13	2014	38	25.4571	76.7143	942.7347	5.8765	0.9939
18	2014	38	21.8429	83.4490	963.1898	3.1357	1.0337
33	2014	38	20.5714	76.6633	900.8663	4.7010	2.4051
56	2014	38	24.3857	81.2551	975.0551	4.3612	1.2765
77	2014	38	23.0857	74.6939	931.8684	2.8602	1.2306
54	2014	38	18.2143	83.0408	832.9296	5.7541	1.5612
21	2014	38	20.5714	76.6633	900.8663	4.7010	2.4051
68	2014	38	22.3286	84.0408	976.9102	3.1092	0.9480
74	2014	38	23.8571	78.2449	960.5929	4.0969	1.1276
88	2014	38	19.8143	80.6939	874.9694	5.6653	2.6010
16	2014	38	20.9429	80.3776	920.3102	4.4265	1.7367
30	2014	38	20.9571	84.4184	897.1786	4.5429	2.3061
6	2014	38	22.3286	84.0408	976.9102	3.1092	0.9480
49	2014	38	22.1286	79.6633	938.8551	4.0327	1.7092
22	2014	38	19.8143	80.6939	874.9694	5.6653	2.6010
45	2014	38	18.1143	81.4286	818.3653	4.7255	1.6980
58	2014	38	22.1286	79.6633	938.8551	4.0327	1.7092
37	2014	38	22.3286	84.0408	976.9102	3.1092	0.9480
17	2014	38	19.9429	87.9286	901.8918	3.7663	2.8031
55	2014	38	20.7571	80.6837	877.6122	5.9327	2.1276
46	2014	38	20.9429	80.3776	920.3102	4.4265	1.7367
86	2014	38	19.9571	76.2347	865.8459	5.6255	0.7500
2	2014	38	19.9571	76.2347	865.8459	5.6255	0.7500
4	2014	38	20.5714	76.6633	900.8663	4.7010	2.4051
47	2014	38	25.3000	80.8469	956.8622	5.4714	0.5173
82	2014	38	19.8143	80.6939	874.9694	5.6653	2.6010

19	2014	38	24.4429	81.6633	958.8133	4.8714	1.2265
20	2014	38	19.3571	79.2245	854.5000	6.4878	2.6469
80	2014	38	19.8143	80.6939	874.9694	5.6653	2.6010
3	2014	38	25.4571	76.7143	942.7347	5.8765	0.9939
52	2014	38	19.9429	87.9286	901.8918	3.7663	2.8031
70	2014	38	20.2714	73.8265	909.0796	6.1204	2.1847
64	2014	38	15.4857	82.2347	777.5112	5.6459	2.6327
48	2014	38	23.0857	74.6939	931.8684	2.8602	1.2306
65	2014	38	19.9429	87.9286	901.8918	3.7663	2.8031
44	2014	38	20.2714	73.8265	909.0796	6.1204	2.1847
75	2014	38	15.4857	82.2347	777.5112	5.6459	2.6327
40	2014	38	20.6429	84.7959	944.5214	4.2439	1.5010
11	2014	38	20.7571	80.6837	877.6122	5.9327	2.1276
35	2014	38	19.5143	84.5000	938.5459	4.0408	1.5796
78	2014	38	21.8714	78.6633	898.9694	5.5622	1.4755
28	2014	38	20.7143	79.6531	928.1276	4.5286	1.5847
39	2014	38	19.9429	87.9286	901.8918	3.7663	2.8031
24	2014	38	22.1286	79.6633	938.8551	4.0327	1.7092
63	2014	38	20.6429	84.7959	944.5214	4.2439	1.5010
62	2014	38	19.7571	78.0612	874.1806	5.8235	1.8490
1	2014	38	19.8143	80.6939	874.9694	5.6653	2.6010
31	2014	39	18.2000	80.3367	848.1847	3.8051	0.9878
79	2014	39	24.4143	79.9796	979.4102	3.2980	0.9031
51	2014	39	21.1857	85.0408	940.7265	3.4653	1.4592
14	2014	39	20.1143	83.3061	898.7010	4.1306	2.0673
67	2014	39	20.5286	86.2041	903.6561	3.1112	3.0980
42	2014	39	19.9857	80.9286	876.6327	4.7092	2.7000
50	2014	39	20.6714	78.2143	903.0418	3.9092	2.1878
43	2014	39	19.9857	80.9286	876.6327	4.7092	2.7000
85	2014	39	21.4286	76.3061	911.5694	4.5041	2.0051
25	2014	39	23.7571	80.1837	977.3357	3.5929	1.2724
69	2014	39	22.3571	79.2041	941.1276	3.3439	1.6510
57	2014	39	20.4857	74.5714	888.2745	4.9224	2.3837
9	2014	39	19.2571	78.0510	855.9724	4.5306	2.4347
72	2014	39	20.4000	79.2551	879.1102	4.5388	2.1337
26	2014	39	20.1429	83.3367	868.4184	4.3857	1.9112
7	2014	39	19.7429	82.0816	860.8265	4.3367	1.7000
83	2014	39	24.3857	78.3061	944.5949	4.4867	1.0071
76	2014	39	21.6143	79.8367	922.4561	4.2408	1.6449
36	2014	39	22.2429	80.0000	930.1786	3.9378	1.4561
81	2014	39	21.1857	85.0408	940.7265	3.4653	1.4592
15	2014	39	22.1286	73.2653	934.1990	2.9041	1.1306
32	2014	39	19.9857	80.9286	876.6327	4.7092	2.7000
73	2014	39	24.3571	76.5816	963.0112	3.7378	1.1969
71	2014	39	22.2429	80.0000	930.1786	3.9378	1.4561
41	2014	39	20.3571	79.5816	876.1143	4.6276	1.6888
10	2014	39	22.3429	83.9082	965.3806	3.4214	1.2480
23	2014	39	15.1429	83.4286	778.5643	4.2724	2.4980
27	2014	39	19.7429	82.0816	860.8265	4.3367	1.7000
60	2014	39	21.1857	85.0408	940.7265	3.4653	1.4592

53	2014	39	19.2571	78.0510	855.9724	4.5306	2.4347
66	2014	39	20.1143	83.3061	898.7010	4.1306	2.0673
59	2014	39	20.4857	74.5714	888.2745	4.9224	2.3837
61	2014	39	24.3571	76.5816	963.0112	3.7378	1.1969
84	2014	39	24.3571	76.5816	963.0112	3.7378	1.1969
38	2014	39	20.4857	74.5714	888.2745	4.9224	2.3837
87	2014	39	21.5714	78.2041	900.7327	4.6265	1.7020
34	2014	39	20.4857	74.5714	888.2745	4.9224	2.3837
29	2014	39	22.3571	79.2041	941.1276	3.3439	1.6510
5	2014	39	17.8000	85.0918	834.2204	4.2020	1.6031
8	2014	39	19.2571	78.0510	855.9724	4.5306	2.4347
12	2014	39	17.8000	85.0918	834.2204	4.2020	1.6031
13	2014	39	24.3857	78.3061	944.5949	4.4867	1.0071
18	2014	39	22.9000	81.4490	965.5898	3.4724	1.0112
33	2014	39	20.6714	78.2143	903.0418	3.9092	2.1878
56	2014	39	23.7571	80.1837	977.3357	3.5929	1.2724
77	2014	39	22.1286	73.2653	934.1990	2.9041	1.1306
54	2014	39	17.8000	85.0918	834.2204	4.2020	1.6031
21	2014	39	20.6714	78.2143	903.0418	3.9092	2.1878
68	2014	39	24.4143	79.9796	979.4102	3.2980	0.9031
74	2014	39	24.3571	76.5816	963.0112	3.7378	1.1969
88	2014	39	19.9857	80.9286	876.6327	4.7092	2.7000
16	2014	39	21.6143	79.8367	922.4561	4.2408	1.6449
30	2014	39	20.1143	83.3061	898.7010	4.1306	2.0673
6	2014	39	24.4143	79.9796	979.4102	3.2980	0.9031
49	2014	39	22.3571	79.2041	941.1276	3.3439	1.6510
22	2014	39	19.9857	80.9286	876.6327	4.7092	2.7000
45	2014	39	17.3571	83.7143	819.5714	3.6418	1.5490
58	2014	39	22.3571	79.2041	941.1276	3.3439	1.6510
37	2014	39	24.4143	79.9796	979.4102	3.2980	0.9031
17	2014	39	20.5286	86.2041	903.6561	3.1112	3.0980
55	2014	39	20.4000	79.2551	879.1102	4.5388	2.1337
46	2014	39	21.6143	79.8367	922.4561	4.2408	1.6449
86	2014	39	20.2143	77.7857	867.6755	4.0541	0.7204
2	2014	39	20.2143	77.7857	867.6755	4.0541	0.7204
4	2014	39	20.6714	78.2143	903.0418	3.9092	2.1878
47	2014	39	23.9286	79.6837	958.8837	4.7490	0.4296
82	2014	39	19.9857	80.9286	876.6327	4.7092	2.7000
19	2014	39	23.1571	80.5408	960.7469	4.7510	1.3235
20	2014	39	19.2571	78.0510	855.9724	4.5306	2.4347
80	2014	39	19.9857	80.9286	876.6327	4.7092	2.7000
3	2014	39	24.3857	78.3061	944.5949	4.4867	1.0071
52	2014	39	20.5286	86.2041	903.6561	3.1112	3.0980
70	2014	39	21.4286	76.3061	911.5694	4.5041	2.0051
64	2014	39	15.1429	83.4286	778.5643	4.2724	2.4980
48	2014	39	22.1286	73.2653	934.1990	2.9041	1.1306
65	2014	39	20.5286	86.2041	903.6561	3.1112	3.0980
44	2014	39	21.4286	76.3061	911.5694	4.5041	2.0051
75	2014	39	15.1429	83.4286	778.5643	4.2724	2.4980
40	2014	39	22.1429	85.6939	946.5398	3.8602	1.4439

11	2014	39	20.4000	79.2551	879.1102	4.5388	2.1337
35	2014	39	21.1857	85.0408	940.7265	3.4653	1.4592
78	2014	39	21.5714	78.2041	900.7327	4.6265	1.7020
28	2014	39	22.2429	80.0000	930.1786	3.9378	1.4561
39	2014	39	20.5286	86.2041	903.6561	3.1112	3.0980
24	2014	39	22.3571	79.2041	941.1276	3.3439	1.6510
63	2014	39	22.1429	85.6939	946.5398	3.8602	1.4439
62	2014	39	20.3571	79.5816	876.1143	4.6276	1.6888
1	2014	39	19.9857	80.9286	876.6327	4.7092	2.7000
31	2014	40	18.3571	79.5306	849.7000	3.7418	1.0102
79	2014	40	22.9000	76.2959	980.8061	3.8265	0.8112
51	2014	40	20.3143	81.6633	942.3531	4.0418	1.3102
14	2014	40	19.7857	83.0816	900.6755	4.0612	1.9184
67	2014	40	19.0143	85.5714	905.5490	2.5051	2.9918
42	2014	40	18.9286	81.5510	878.4061	3.9092	2.4980
50	2014	40	20.5143	77.0714	904.8163	3.6286	1.8786
43	2014	40	18.9286	81.5510	878.4061	3.9092	2.4980
85	2014	40	21.2429	75.0102	913.4296	3.7122	1.6806
25	2014	40	23.3857	81.0816	979.5673	3.9571	1.0745
69	2014	40	21.4143	78.6531	942.9551	3.0663	1.5684
57	2014	40	19.1571	74.5204	890.0286	4.3408	2.2612
9	2014	40	18.8000	78.1735	857.6929	3.9837	2.2980
72	2014	40	19.8286	79.8980	881.0561	4.6316	1.6959
26	2014	40	20.2571	83.6020	870.5082	4.2551	1.7459
7	2014	40	19.7429	81.9592	862.7622	5.1337	1.6939
83	2014	40	23.7571	79.1735	947.1184	5.5724	0.9837
76	2014	40	20.6571	77.1735	924.1959	4.7592	1.4153
36	2014	40	20.3857	78.6122	932.0520	4.1510	1.3582
81	2014	40	20.3143	81.6633	942.3531	4.0418	1.3102
15	2014	40	21.5571	73.2347	936.1143	3.2031	0.8990
32	2014	40	18.9286	81.5510	878.4061	3.9092	2.4980
73	2014	40	23.1429	75.7755	964.7765	3.7265	1.0102
71	2014	40	20.3857	78.6122	932.0520	4.1510	1.3582
41	2014	40	19.5429	80.2755	877.8163	4.0796	1.4857
10	2014	40	21.7714	81.7143	967.0286	4.1735	0.9735
23	2014	40	14.9143	81.8776	779.8510	4.3857	2.4245
27	2014	40	19.7429	81.9592	862.7622	5.1337	1.6939
60	2014	40	20.3143	81.6633	942.3531	4.0418	1.3102
53	2014	40	18.8000	78.1735	857.6929	3.9837	2.2980
66	2014	40	19.7857	83.0816	900.6755	4.0612	1.9184
59	2014	40	19.1571	74.5204	890.0286	4.3408	2.2612
61	2014	40	23.1429	75.7755	964.7765	3.7265	1.0102
84	2014	40	23.1429	75.7755	964.7765	3.7265	1.0102
38	2014	40	19.1571	74.5204	890.0286	4.3408	2.2612
87	2014	40	20.7000	78.6429	902.7051	4.5643	1.5776
34	2014	40	19.1571	74.5204	890.0286	4.3408	2.2612
29	2014	40	21.4143	78.6531	942.9551	3.0663	1.5684
5	2014	40	17.9143	85.3367	835.8847	4.4929	1.3459
8	2014	40	18.8000	78.1735	857.6929	3.9837	2.2980
12	2014	40	17.9143	85.3367	835.8847	4.4929	1.3459

13	2014	40	23.7571	79.1735	947.1184	5.5724	0.9837
18	2014	40	21.7571	79.9694	967.0224	4.1500	0.9786
33	2014	40	20.5143	77.0714	904.8163	3.6286	1.8786
56	2014	40	23.3857	81.0816	979.5673	3.9571	1.0745
77	2014	40	21.5571	73.2347	936.1143	3.2031	0.8990
54	2014	40	17.9143	85.3367	835.8847	4.4929	1.3459
21	2014	40	20.5143	77.0714	904.8163	3.6286	1.8786
68	2014	40	22.9000	76.2959	980.8061	3.8265	0.8112
74	2014	40	23.1429	75.7755	964.7765	3.7265	1.0102
88	2014	40	18.9286	81.5510	878.4061	3.9092	2.4980
16	2014	40	20.6571	77.1735	924.1959	4.7592	1.4153
30	2014	40	19.7857	83.0816	900.6755	4.0612	1.9184
6	2014	40	22.9000	76.2959	980.8061	3.8265	0.8112
49	2014	40	21.4143	78.6531	942.9551	3.0663	1.5684
22	2014	40	18.9286	81.5510	878.4061	3.9092	2.4980
45	2014	40	17.5857	82.7857	821.2122	3.8918	1.5388
58	2014	40	21.4143	78.6531	942.9551	3.0663	1.5684
37	2014	40	22.9000	76.2959	980.8061	3.8265	0.8112
17	2014	40	19.0143	85.5714	905.5490	2.5051	2.9918
55	2014	40	19.8286	79.8980	881.0561	4.6316	1.6959
46	2014	40	20.6571	77.1735	924.1959	4.7592	1.4153
86	2014	40	19.9000	76.6020	869.3582	3.5367	0.7184
2	2014	40	19.9000	76.6020	869.3582	3.5367	0.7184
4	2014	40	20.5143	77.0714	904.8163	3.6286	1.8786
47	2014	40	23.8857	80.1735	961.3296	4.7765	0.3327
82	2014	40	18.9286	81.5510	878.4061	3.9092	2.4980
19	2014	40	23.7857	80.5510	963.0980	4.4378	1.1163
20	2014	40	18.8000	78.1735	857.6929	3.9837	2.2980
80	2014	40	18.9286	81.5510	878.4061	3.9092	2.4980
3	2014	40	23.7571	79.1735	947.1184	5.5724	0.9837
52	2014	40	19.0143	85.5714	905.5490	2.5051	2.9918
70	2014	40	21.2429	75.0102	913.4296	3.7122	1.6806
64	2014	40	14.9143	81.8776	779.8510	4.3857	2.4245
48	2014	40	21.5571	73.2347	936.1143	3.2031	0.8990
65	2014	40	19.0143	85.5714	905.5490	2.5051	2.9918
44	2014	40	21.2429	75.0102	913.4296	3.7122	1.6806
75	2014	40	14.9143	81.8776	779.8510	4.3857	2.4245
40	2014	40	21.0714	83.1939	948.3867	4.6571	1.3776
11	2014	40	19.8286	79.8980	881.0561	4.6316	1.6959
35	2014	40	20.3143	81.6633	942.3531	4.0418	1.3102
78	2014	40	20.7000	78.6429	902.7051	4.5643	1.5776
28	2014	40	20.3857	78.6122	932.0520	4.1510	1.3582
39	2014	40	19.0143	85.5714	905.5490	2.5051	2.9918
24	2014	40	21.4143	78.6531	942.9551	3.0663	1.5684
63	2014	40	21.0714	83.1939	948.3867	4.6571	1.3776
62	2014	40	19.5429	80.2755	877.8163	4.0796	1.4857
1	2014	40	18.9286	81.5510	878.4061	3.9092	2.4980
31	2014	41	16.3143	79.3061	851.3102	4.8724	1.0347
79	2014	41	19.8857	75.8163	983.0592	4.1031	0.8439
51	2014	41	16.5571	82.2143	944.4439	4.1194	1.2888

14	2014	41	16.8429	83.9490	902.4929	4.0622	1.6796
67	2014	41	16.5286	86.9796	907.4378	2.9122	2.8541
42	2014	41	17.0429	81.0408	880.1388	4.7939	2.3878
50	2014	41	18.0143	74.2755	906.7122	4.8724	1.7408
43	2014	41	17.0429	81.0408	880.1388	4.7939	2.3878
85	2014	41	19.8286	73.2347	915.2255	4.7133	1.5276
25	2014	41	20.1000	82.2143	981.7755	4.4143	1.1143
69	2014	41	18.1429	78.6837	945.0908	3.6429	1.5112
57	2014	41	17.3429	73.9796	891.7918	5.2786	2.1327
9	2014	41	16.9143	77.7041	859.3531	5.1276	2.1357
72	2014	41	17.2143	81.6531	882.8184	4.9980	1.5582
26	2014	41	18.0429	81.8367	872.2541	4.6949	1.6765
7	2014	41	16.9857	80.1531	864.4224	5.9878	1.7276
83	2014	41	20.8714	79.8265	949.2571	5.7908	0.9010
76	2014	41	17.6714	76.0816	926.2490	5.6867	1.3990
36	2014	41	17.4857	79.9592	934.0878	4.3653	1.4459
81	2014	41	16.5571	82.2143	944.4439	4.1194	1.2888
15	2014	41	19.0143	73.2041	938.2949	4.4755	0.8898
32	2014	41	17.0429	81.0408	880.1388	4.7939	2.3878
73	2014	41	20.5571	75.6327	967.0541	4.6306	0.9827
71	2014	41	17.4857	79.9592	934.0878	4.3653	1.4459
41	2014	41	17.6571	79.4898	879.5092	4.6929	1.3827
10	2014	41	17.7571	81.3776	969.1878	4.1531	0.9265
23	2014	41	13.2571	79.6633	781.2908	5.7143	2.4398
27	2014	41	16.9857	80.1531	864.4224	5.9878	1.7276
60	2014	41	16.5571	82.2143	944.4439	4.1194	1.2888
53	2014	41	16.9143	77.7041	859.3531	5.1276	2.1357
66	2014	41	16.8429	83.9490	902.4929	4.0622	1.6796
59	2014	41	17.3429	73.9796	891.7918	5.2786	2.1327
61	2014	41	20.5571	75.6327	967.0541	4.6306	0.9827
84	2014	41	20.5571	75.6327	967.0541	4.6306	0.9827
38	2014	41	17.3429	73.9796	891.7918	5.2786	2.1327
87	2014	41	17.9714	80.6837	904.5439	5.1827	1.3806
34	2014	41	17.3429	73.9796	891.7918	5.2786	2.1327
29	2014	41	18.1429	78.6837	945.0908	3.6429	1.5112
5	2014	41	15.2714	83.0306	837.4816	5.6745	1.2459
8	2014	41	16.9143	77.7041	859.3531	5.1276	2.1357
12	2014	41	15.2714	83.0306	837.4816	5.6745	1.2459
13	2014	41	20.8714	79.8265	949.2571	5.7908	0.9010
18	2014	41	18.7143	78.2449	969.2367	4.9908	0.9673
33	2014	41	18.0143	74.2755	906.7122	4.8724	1.7408
56	2014	41	20.1000	82.2143	981.7755	4.4143	1.1143
77	2014	41	19.0143	73.2041	938.2949	4.4755	0.8898
54	2014	41	15.2714	83.0306	837.4816	5.6745	1.2459
21	2014	41	18.0143	74.2755	906.7122	4.8724	1.7408
68	2014	41	19.8857	75.8163	983.0592	4.1031	0.8439
74	2014	41	20.5571	75.6327	967.0541	4.6306	0.9827
88	2014	41	17.0429	81.0408	880.1388	4.7939	2.3878
16	2014	41	17.6714	76.0816	926.2490	5.6867	1.3990
30	2014	41	16.8429	83.9490	902.4929	4.0622	1.6796

6	2014	41	19.8857	75.8163	983.0592	4.1031	0.8439
49	2014	41	18.1429	78.6837	945.0908	3.6429	1.5112
22	2014	41	17.0429	81.0408	880.1388	4.7939	2.3878
45	2014	41	15.0857	79.5816	822.7561	5.2837	1.4827
58	2014	41	18.1429	78.6837	945.0908	3.6429	1.5112
37	2014	41	19.8857	75.8163	983.0592	4.1031	0.8439
17	2014	41	16.5286	86.9796	907.4378	2.9122	2.8541
55	2014	41	17.2143	81.6531	882.8184	4.9980	1.5582
46	2014	41	17.6714	76.0816	926.2490	5.6867	1.3990
86	2014	41	17.5000	75.3367	870.9969	4.5082	0.7224
2	2014	41	17.5000	75.3367	870.9969	4.5082	0.7224
4	2014	41	18.0143	74.2755	906.7122	4.8724	1.7408
47	2014	41	21.0143	81.8980	963.4347	4.9000	0.2653
82	2014	41	17.0429	81.0408	880.1388	4.7939	2.3878
19	2014	41	20.1857	79.9796	965.1306	4.5735	0.9969
20	2014	41	16.9143	77.7041	859.3531	5.1276	2.1357
80	2014	41	17.0429	81.0408	880.1388	4.7939	2.3878
3	2014	41	20.8714	79.8265	949.2571	5.7908	0.9010
52	2014	41	16.5286	86.9796	907.4378	2.9122	2.8541
70	2014	41	19.8286	73.2347	915.2255	4.7133	1.5276
64	2014	41	13.2571	79.6633	781.2908	5.7143	2.4398
48	2014	41	19.0143	73.2041	938.2949	4.4755	0.8898
65	2014	41	16.5286	86.9796	907.4378	2.9122	2.8541
44	2014	41	19.8286	73.2347	915.2255	4.7133	1.5276
75	2014	41	13.2571	79.6633	781.2908	5.7143	2.4398
40	2014	41	17.6143	82.7857	950.4071	4.7500	1.3245
11	2014	41	17.2143	81.6531	882.8184	4.9980	1.5582
35	2014	41	16.5571	82.2143	944.4439	4.1194	1.2888
78	2014	41	17.9714	80.6837	904.5439	5.1827	1.3806
28	2014	41	17.4857	79.9592	934.0878	4.3653	1.4459
39	2014	41	16.5286	86.9796	907.4378	2.9122	2.8541
24	2014	41	18.1429	78.6837	945.0908	3.6429	1.5112
63	2014	41	17.6143	82.7857	950.4071	4.7500	1.3245
62	2014	41	17.6571	79.4898	879.5092	4.6929	1.3827
1	2014	41	17.0429	81.0408	880.1388	4.7939	2.3878
31	2014	42	12.5143	82.0510	853.0459	3.5898	0.8888
79	2014	42	20.6714	71.8367	986.1857	4.6439	0.9000
51	2014	42	18.2000	79.5510	947.0286	4.7143	1.1214
14	2014	42	17.8429	77.8163	904.3735	5.5684	1.3602
67	2014	42	17.4000	81.3673	909.4847	3.6908	2.8071
42	2014	42	15.7143	76.9898	881.9806	5.0480	2.3653
50	2014	42	14.7000	74.6531	909.1255	4.8520	1.6755
43	2014	42	15.7143	76.9898	881.9806	5.0480	2.3653
85	2014	42	14.3429	73.7347	917.7776	4.5673	1.5051
25	2014	42	21.0857	78.6837	984.5327	5.2520	1.0724
69	2014	42	19.4286	76.1735	947.7327	4.1020	1.3745
57	2014	42	14.8857	73.3776	893.9051	4.8827	1.8092
9	2014	42	14.9286	75.0204	860.9908	3.6939	1.9337
72	2014	42	16.9000	78.6735	884.5908	4.0092	1.3082
26	2014	42	17.0857	79.9082	873.8520	3.1857	1.5735

7	2014	42	16.7714	79.2449	865.9888	3.8347	1.5194
83	2014	42	21.7000	77.2959	951.5673	4.5816	0.8214
76	2014	42	17.4000	74.6224	928.7480	5.5265	1.2806
36	2014	42	19.0571	75.6837	936.4561	4.8010	1.3980
81	2014	42	18.2000	79.5510	947.0286	4.7143	1.1214
15	2014	42	17.2571	72.8061	940.9622	4.9673	0.7949
32	2014	42	15.7143	76.9898	881.9806	5.0480	2.3653
73	2014	42	20.5286	73.1122	969.9847	4.8949	0.9245
71	2014	42	19.0571	75.6837	936.4561	4.8010	1.3980
41	2014	42	14.7429	78.7857	881.5357	3.9041	1.2041
10	2014	42	19.0143	78.4490	972.0184	5.0949	0.9949
23	2014	42	10.4429	84.7041	782.0918	3.9276	2.2245
27	2014	42	16.7714	79.2449	865.9888	3.8347	1.5194
60	2014	42	18.2000	79.5510	947.0286	4.7143	1.1214
53	2014	42	14.9286	75.0204	860.9908	3.6939	1.9337
66	2014	42	17.8429	77.8163	904.3735	5.5684	1.3602
59	2014	42	14.8857	73.3776	893.9051	4.8827	1.8092
61	2014	42	20.5286	73.1122	969.9847	4.8949	0.9245
84	2014	42	20.5286	73.1122	969.9847	4.8949	0.9245
38	2014	42	14.8857	73.3776	893.9051	4.8827	1.8092
87	2014	42	17.7000	77.9184	906.5643	5.1745	1.2459
34	2014	42	14.8857	73.3776	893.9051	4.8827	1.8092
29	2014	42	19.4286	76.1735	947.7327	4.1020	1.3745
5	2014	42	14.3000	84.1531	838.8786	3.9173	1.3745
8	2014	42	14.9286	75.0204	860.9908	3.6939	1.9337
12	2014	42	14.3000	84.1531	838.8786	3.9173	1.3745
13	2014	42	21.7000	77.2959	951.5673	4.5816	0.8214
18	2014	42	19.2714	73.9286	972.2327	5.2745	0.9408
33	2014	42	14.7000	74.6531	909.1255	4.8520	1.6755
56	2014	42	21.0857	78.6837	984.5327	5.2520	1.0724
77	2014	42	17.2571	72.8061	940.9622	4.9673	0.7949
54	2014	42	14.3000	84.1531	838.8786	3.9173	1.3745
21	2014	42	14.7000	74.6531	909.1255	4.8520	1.6755
68	2014	42	20.6714	71.8367	986.1857	4.6439	0.9000
74	2014	42	20.5286	73.1122	969.9847	4.8949	0.9245
88	2014	42	15.7143	76.9898	881.9806	5.0480	2.3653
16	2014	42	17.4000	74.6224	928.7480	5.5265	1.2806
30	2014	42	17.8429	77.8163	904.3735	5.5684	1.3602
6	2014	42	20.6714	71.8367	986.1857	4.6439	0.9000
49	2014	42	19.4286	76.1735	947.7327	4.1020	1.3745
22	2014	42	15.7143	76.9898	881.9806	5.0480	2.3653
45	2014	42	14.4429	82.6020	823.9551	3.8214	1.2296
58	2014	42	19.4286	76.1735	947.7327	4.1020	1.3745
37	2014	42	20.6714	71.8367	986.1857	4.6439	0.9000
17	2014	42	17.4000	81.3673	909.4847	3.6908	2.8071
55	2014	42	16.9000	78.6735	884.5908	4.0092	1.3082
46	2014	42	17.4000	74.6224	928.7480	5.5265	1.2806
86	2014	42	15.3571	75.7449	872.8694	3.3010	0.6959
2	2014	42	15.3571	75.7449	872.8694	3.3010	0.6959
4	2014	42	14.7000	74.6531	909.1255	4.8520	1.6755

47	2014	42	21.1571	78.3673	965.9000	4.6755	0.2704
82	2014	42	15.7143	76.9898	881.9806	5.0480	2.3653
19	2014	42	21.5000	74.4286	967.5337	6.3224	0.9612
20	2014	42	14.9286	75.0204	860.9908	3.6939	1.9337
80	2014	42	15.7143	76.9898	881.9806	5.0480	2.3653
3	2014	42	21.7000	77.2959	951.5673	4.5816	0.8214
52	2014	42	17.4000	81.3673	909.4847	3.6908	2.8071
70	2014	42	14.3429	73.7347	917.7776	4.5673	1.5051
64	2014	42	10.4429	84.7041	782.0918	3.9276	2.2245
48	2014	42	17.2571	72.8061	940.9622	4.9673	0.7949
65	2014	42	17.4000	81.3673	909.4847	3.6908	2.8071
44	2014	42	14.3429	73.7347	917.7776	4.5673	1.5051
75	2014	42	10.4429	84.7041	782.0918	3.9276	2.2245
40	2014	42	19.2000	78.8367	952.8684	5.4663	1.0602
11	2014	42	16.9000	78.6735	884.5908	4.0092	1.3082
35	2014	42	18.2000	79.5510	947.0286	4.7143	1.1214
78	2014	42	17.7000	77.9184	906.5643	5.1745	1.2459
28	2014	42	19.0571	75.6837	936.4561	4.8010	1.3980
39	2014	42	17.4000	81.3673	909.4847	3.6908	2.8071
24	2014	42	19.4286	76.1735	947.7327	4.1020	1.3745
63	2014	42	19.2000	78.8367	952.8684	5.4663	1.0602
62	2014	42	14.7429	78.7857	881.5357	3.9041	1.2041
1	2014	42	15.7143	76.9898	881.9806	5.0480	2.3653
31	2014	43	14.5143	86.7449	852.9500	1.8000	0.7908
79	2014	43	21.2143	69.3571	985.9551	4.1429	0.8204
51	2014	43	18.5857	76.1939	946.8224	4.6439	1.1735
14	2014	43	18.2857	74.5102	904.4918	4.9969	1.7378
67	2014	43	18.1429	79.1735	909.4071	2.4031	2.8592
42	2014	43	16.8857	79.1122	881.9051	3.0643	2.3816
50	2014	43	16.2143	81.8776	909.1510	2.6939	1.5520
43	2014	43	16.8857	79.1122	881.9051	3.0643	2.3816
85	2014	43	17.3571	82.2551	918.0469	2.7704	1.2388
25	2014	43	21.0857	76.3980	984.6561	5.4388	0.9918
69	2014	43	19.8143	73.1327	947.4745	2.8837	1.4857
57	2014	43	16.1286	78.4592	893.8827	2.7163	1.9888
9	2014	43	16.3857	78.6531	860.8459	2.4347	2.1551
72	2014	43	17.7429	78.0102	884.5827	3.5133	1.4265
26	2014	43	18.7429	81.4388	873.7561	2.8867	1.8745
7	2014	43	18.1429	79.7245	865.8561	3.1745	1.7214
83	2014	43	22.0000	74.3776	951.6714	4.3388	0.8490
76	2014	43	17.7857	77.0000	928.5582	3.3051	1.1929
36	2014	43	19.2286	71.8469	936.3133	4.1061	1.2918
81	2014	43	18.5857	76.1939	946.8224	4.6439	1.1735
15	2014	43	17.6143	79.1224	940.9224	2.8388	0.6378
32	2014	43	16.8857	79.1122	881.9051	3.0643	2.3816
73	2014	43	21.1714	72.6531	969.7561	3.1980	0.8031
71	2014	43	19.2286	71.8469	936.3133	4.1061	1.2918
41	2014	43	16.3857	83.9286	881.4633	2.2582	1.0173
10	2014	43	19.3571	77.0816	971.9551	5.2633	1.0735
23	2014	43	12.9286	87.2653	781.6214	3.3796	2.0867

27	2014	43	18.1429	79.7245	865.8561	3.1745	1.7214
60	2014	43	18.5857	76.1939	946.8224	4.6439	1.1735
53	2014	43	16.3857	78.6531	860.8459	2.4347	2.1551
66	2014	43	18.2857	74.5102	904.4918	4.9969	1.7378
59	2014	43	16.1286	78.4592	893.8827	2.7163	1.9888
61	2014	43	21.1714	72.6531	969.7561	3.1980	0.8031
84	2014	43	21.1714	72.6531	969.7561	3.1980	0.8031
38	2014	43	16.1286	78.4592	893.8827	2.7163	1.9888
87	2014	43	18.1000	78.2857	906.5663	3.7092	1.1735
34	2014	43	16.1286	78.4592	893.8827	2.7163	1.9888
29	2014	43	19.8143	73.1327	947.4745	2.8837	1.4857
5	2014	43	16.4571	84.7653	838.6133	3.3633	1.4786
8	2014	43	16.3857	78.6531	860.8459	2.4347	2.1551
12	2014	43	16.4571	84.7653	838.6133	3.3633	1.4786
13	2014	43	22.0000	74.3776	951.6714	4.3388	0.8490
18	2014	43	20.1857	72.1122	971.9714	4.3378	1.1194
33	2014	43	16.2143	81.8776	909.1510	2.6939	1.5520
56	2014	43	21.0857	76.3980	984.6561	5.4388	0.9918
77	2014	43	17.6143	79.1224	940.9224	2.8388	0.6378
54	2014	43	16.4571	84.7653	838.6133	3.3633	1.4786
21	2014	43	16.2143	81.8776	909.1510	2.6939	1.5520
68	2014	43	21.2143	69.3571	985.9551	4.1429	0.8204
74	2014	43	21.1714	72.6531	969.7561	3.1980	0.8031
88	2014	43	16.8857	79.1122	881.9051	3.0643	2.3816
16	2014	43	17.7857	77.0000	928.5582	3.3051	1.1929
30	2014	43	18.2857	74.5102	904.4918	4.9969	1.7378
6	2014	43	21.2143	69.3571	985.9551	4.1429	0.8204
49	2014	43	19.8143	73.1327	947.4745	2.8837	1.4857
22	2014	43	16.8857	79.1122	881.9051	3.0643	2.3816
45	2014	43	16.0143	83.3265	823.6286	3.0796	1.5316
58	2014	43	19.8143	73.1327	947.4745	2.8837	1.4857
37	2014	43	21.2143	69.3571	985.9551	4.1429	0.8204
17	2014	43	18.1429	79.1735	909.4071	2.4031	2.8592
55	2014	43	17.7429	78.0102	884.5827	3.5133	1.4265
46	2014	43	17.7857	77.0000	928.5582	3.3051	1.1929
86	2014	43	16.9429	79.5102	872.7439	1.7571	0.6857
2	2014	43	16.9429	79.5102	872.7439	1.7571	0.6857
4	2014	43	16.2143	81.8776	909.1510	2.6939	1.5520
47	2014	43	21.7000	76.3061	966.1000	3.7224	0.3480
82	2014	43	16.8857	79.1122	881.9051	3.0643	2.3816
19	2014	43	21.2714	73.2755	967.8531	5.9255	0.9214
20	2014	43	16.3857	78.6531	860.8459	2.4347	2.1551
80	2014	43	16.8857	79.1122	881.9051	3.0643	2.3816
3	2014	43	22.0000	74.3776	951.6714	4.3388	0.8490
52	2014	43	18.1429	79.1735	909.4071	2.4031	2.8592
70	2014	43	17.3571	82.2551	918.0469	2.7704	1.2388
64	2014	43	12.9286	87.2653	781.6214	3.3796	2.0867
48	2014	43	17.6143	79.1224	940.9224	2.8388	0.6378
65	2014	43	18.1429	79.1735	909.4071	2.4031	2.8592
44	2014	43	17.3571	82.2551	918.0469	2.7704	1.2388

75	2014	43	12.9286	87.2653	781.6214	3.3796	2.0867
40	2014	43	19.2000	75.4694	952.9704	5.4908	1.1092
11	2014	43	17.7429	78.0102	884.5827	3.5133	1.4265
35	2014	43	18.5857	76.1939	946.8224	4.6439	1.1735
78	2014	43	18.1000	78.2857	906.5663	3.7092	1.1735
28	2014	43	19.2286	71.8469	936.3133	4.1061	1.2918
39	2014	43	18.1429	79.1735	909.4071	2.4031	2.8592
24	2014	43	19.8143	73.1327	947.4745	2.8837	1.4857
63	2014	43	19.2000	75.4694	952.9704	5.4908	1.1092
62	2014	43	16.3857	83.9286	881.4633	2.2582	1.0173
1	2014	43	16.8857	79.1122	881.9051	3.0643	2.3816
31	2014	44	14.4000	86.9184	851.8122	2.5929	0.8684
79	2014	44	16.1000	74.9490	984.8847	3.8673	0.7378
51	2014	44	14.8429	80.2959	945.7102	3.9408	1.3673
14	2014	44	18.0143	77.9388	903.6673	4.0959	2.0429
67	2014	44	16.7571	83.1327	908.3490	2.5673	2.9745
42	2014	44	16.3429	82.8776	880.8990	3.4133	2.2480
50	2014	44	16.4286	84.7755	907.6520	2.8041	1.3704
43	2014	44	16.3429	82.8776	880.8990	3.4133	2.2480
85	2014	44	17.0286	85.3061	916.4449	3.1602	1.1082
25	2014	44	20.7571	79.2245	983.4745	4.9531	1.0571
69	2014	44	17.2000	76.5816	946.1551	2.6367	1.5449
57	2014	44	15.8143	80.6224	892.6500	2.8327	2.4959
9	2014	44	16.1000	81.2449	859.9408	4.1122	2.6112
72	2014	44	18.2286	79.7347	883.7337	4.9704	1.8867
26	2014	44	18.8143	81.7959	873.0143	5.0306	2.1867
7	2014	44	18.2000	78.6633	865.0755	5.2857	1.9776
83	2014	44	22.7286	74.7143	950.7082	5.3561	0.9337
76	2014	44	15.4714	82.7959	927.2429	2.9398	1.2500
36	2014	44	16.9714	75.7551	935.1204	3.9582	1.4194
81	2014	44	14.8429	80.2959	945.7102	3.9408	1.3673
15	2014	44	16.4714	85.7551	939.5959	1.8071	0.6316
32	2014	44	16.3429	82.8776	880.8990	3.4133	2.2480
73	2014	44	17.3429	78.8571	968.5041	3.1031	0.8918
71	2014	44	16.9714	75.7551	935.1204	3.9582	1.4194
41	2014	44	16.2714	86.1020	880.2347	2.9837	1.1867
10	2014	44	16.1571	81.2857	970.8776	4.2918	1.0439
23	2014	44	11.2714	80.7857	781.0388	4.6857	2.2541
27	2014	44	18.2000	78.6633	865.0755	5.2857	1.9776
60	2014	44	14.8429	80.2959	945.7102	3.9408	1.3673
53	2014	44	16.1000	81.2449	859.9408	4.1122	2.6112
66	2014	44	18.0143	77.9388	903.6673	4.0959	2.0429
59	2014	44	15.8143	80.6224	892.6500	2.8327	2.4959
61	2014	44	17.3429	78.8571	968.5041	3.1031	0.8918
84	2014	44	17.3429	78.8571	968.5041	3.1031	0.8918
38	2014	44	15.8143	80.6224	892.6500	2.8327	2.4959
87	2014	44	18.6429	81.2653	905.5806	4.5316	1.4020
34	2014	44	15.8143	80.6224	892.6500	2.8327	2.4959
29	2014	44	17.2000	76.5816	946.1551	2.6367	1.5449
5	2014	44	15.8857	81.0204	837.8531	5.5071	1.5582

8	2014	44	16.1000	81.2449	859.9408	4.1122	2.6112
12	2014	44	15.8857	81.0204	837.8531	5.5071	1.5582
13	2014	44	22.7286	74.7143	950.7082	5.3561	0.9337
18	2014	44	15.2714	78.0714	970.8245	4.1776	1.0745
33	2014	44	16.4286	84.7755	907.6520	2.8041	1.3704
56	2014	44	20.7571	79.2245	983.4745	4.9531	1.0571
77	2014	44	16.4714	85.7551	939.5959	1.8071	0.6316
54	2014	44	15.8857	81.0204	837.8531	5.5071	1.5582
21	2014	44	16.4286	84.7755	907.6520	2.8041	1.3704
68	2014	44	16.1000	74.9490	984.8847	3.8673	0.7378
74	2014	44	17.3429	78.8571	968.5041	3.1031	0.8918
88	2014	44	16.3429	82.8776	880.8990	3.4133	2.2480
16	2014	44	15.4714	82.7959	927.2429	2.9398	1.2500
30	2014	44	18.0143	77.9388	903.6673	4.0959	2.0429
6	2014	44	16.1000	74.9490	984.8847	3.8673	0.7378
49	2014	44	17.2000	76.5816	946.1551	2.6367	1.5449
22	2014	44	16.3429	82.8776	880.8990	3.4133	2.2480
45	2014	44	15.1429	78.5306	822.9776	4.7673	1.9235
58	2014	44	17.2000	76.5816	946.1551	2.6367	1.5449
37	2014	44	16.1000	74.9490	984.8847	3.8673	0.7378
17	2014	44	16.7571	83.1327	908.3490	2.5673	2.9745
55	2014	44	18.2286	79.7347	883.7337	4.9704	1.8867
46	2014	44	15.4714	82.7959	927.2429	2.9398	1.2500
86	2014	44	16.6714	81.0918	871.6367	2.4500	0.7102
2	2014	44	16.6714	81.0918	871.6367	2.4500	0.7102
4	2014	44	16.4286	84.7755	907.6520	2.8041	1.3704
47	2014	44	22.2714	78.7857	965.0286	4.0337	0.4112
82	2014	44	16.3429	82.8776	880.8990	3.4133	2.2480
19	2014	44	21.1286	79.2551	966.9092	4.5684	0.8959
20	2014	44	16.1000	81.2449	859.9408	4.1122	2.6112
80	2014	44	16.3429	82.8776	880.8990	3.4133	2.2480
3	2014	44	22.7286	74.7143	950.7082	5.3561	0.9337
52	2014	44	16.7571	83.1327	908.3490	2.5673	2.9745
70	2014	44	17.0286	85.3061	916.4449	3.1602	1.1082
64	2014	44	11.2714	80.7857	781.0388	4.6857	2.2541
48	2014	44	16.4714	85.7551	939.5959	1.8071	0.6316
65	2014	44	16.7571	83.1327	908.3490	2.5673	2.9745
44	2014	44	17.0286	85.3061	916.4449	3.1602	1.1082
75	2014	44	11.2714	80.7857	781.0388	4.6857	2.2541
40	2014	44	16.8857	78.6327	951.9684	4.5367	1.2847
11	2014	44	18.2286	79.7347	883.7337	4.9704	1.8867
35	2014	44	14.8429	80.2959	945.7102	3.9408	1.3673
78	2014	44	18.6429	81.2653	905.5806	4.5316	1.4020
28	2014	44	16.9714	75.7551	935.1204	3.9582	1.4194
39	2014	44	16.7571	83.1327	908.3490	2.5673	2.9745
24	2014	44	17.2000	76.5816	946.1551	2.6367	1.5449
63	2014	44	16.8857	78.6327	951.9684	4.5367	1.2847
62	2014	44	16.2714	86.1020	880.2347	2.9837	1.1867
1	2014	44	16.3429	82.8776	880.8990	3.4133	2.2480
31	2014	45	9.4000	85.4796	851.8061	1.7000	0.9704

79	2014	45	14.5857	79.7449	986.6010	1.8306	0.7429
51	2014	45	12.0714	84.2551	946.7480	1.7092	1.6255
14	2014	45	11.5714	81.6735	903.8061	2.3867	2.0245
67	2014	45	11.3000	87.7449	908.7306	1.6735	3.0051
42	2014	45	10.2000	86.2143	881.0286	2.5510	2.3265
50	2014	45	10.5571	84.3673	908.0765	1.5541	1.4316
43	2014	45	10.2000	86.2143	881.0286	2.5510	2.3265
85	2014	45	11.4000	85.0408	916.9061	2.1255	1.3531
25	2014	45	15.4571	82.4592	984.5122	2.5776	1.0949
69	2014	45	12.8429	81.3163	947.1949	1.2520	1.4684
57	2014	45	9.9286	82.6633	892.9878	1.7959	2.4663
9	2014	45	9.8286	83.2959	859.8939	2.7959	2.8163
72	2014	45	11.3714	81.8469	883.7786	3.2541	2.1571
26	2014	45	12.4429	84.1939	872.9602	3.8204	1.9837
7	2014	45	11.8429	80.6429	864.9888	3.7827	2.0000
83	2014	45	16.0857	77.5408	951.1755	3.3561	0.9694
76	2014	45	10.9571	86.9898	928.0776	1.4980	1.4643
36	2014	45	12.1571	80.7653	935.9082	2.3010	1.5429
81	2014	45	12.0714	84.2551	946.7480	1.7092	1.6255
15	2014	45	12.1286	87.1020	940.4337	0.7112	0.7031
32	2014	45	10.2000	86.2143	881.0286	2.5510	2.3265
73	2014	45	14.0286	86.1224	969.9429	1.4929	0.9184
71	2014	45	12.1571	80.7653	935.9082	2.3010	1.5429
41	2014	45	10.5571	85.3980	880.4469	1.9755	1.5561
10	2014	45	13.3857	85.6327	972.2000	2.0684	0.9378
23	2014	45	7.5000	83.3061	780.5490	3.5704	2.3929
27	2014	45	11.8429	80.6429	864.9888	3.7827	2.0000
60	2014	45	12.0714	84.2551	946.7480	1.7092	1.6255
53	2014	45	9.8286	83.2959	859.8939	2.7959	2.8163
66	2014	45	11.5714	81.6735	903.8061	2.3867	2.0245
59	2014	45	9.9286	82.6633	892.9878	1.7959	2.4663
61	2014	45	14.0286	86.1224	969.9429	1.4929	0.9184
84	2014	45	14.0286	86.1224	969.9429	1.4929	0.9184
38	2014	45	9.9286	82.6633	892.9878	1.7959	2.4663
87	2014	45	12.2000	83.0204	905.8847	3.2735	1.8745
34	2014	45	9.9286	82.6633	892.9878	1.7959	2.4663
29	2014	45	12.8429	81.3163	947.1949	1.2520	1.4684
5	2014	45	9.7714	84.4694	837.6255	3.8980	1.5592
8	2014	45	9.8286	83.2959	859.8939	2.7959	2.8163
12	2014	45	9.7714	84.4694	837.6255	3.8980	1.5592
13	2014	45	16.0857	77.5408	951.1755	3.3561	0.9694
18	2014	45	13.8286	83.3980	972.3418	2.1378	0.9908
33	2014	45	10.5571	84.3673	908.0765	1.5541	1.4316
56	2014	45	15.4571	82.4592	984.5122	2.5776	1.0949
77	2014	45	12.1286	87.1020	940.4337	0.7112	0.7031
54	2014	45	9.7714	84.4694	837.6255	3.8980	1.5592
21	2014	45	10.5571	84.3673	908.0765	1.5541	1.4316
68	2014	45	14.5857	79.7449	986.6010	1.8306	0.7429
74	2014	45	14.0286	86.1224	969.9429	1.4929	0.9184
88	2014	45	10.2000	86.2143	881.0286	2.5510	2.3265

16	2014	45	10.9571	86.9898	928.0776	1.4980	1.4643
30	2014	45	11.5714	81.6735	903.8061	2.3867	2.0245
6	2014	45	14.5857	79.7449	986.6010	1.8306	0.7429
49	2014	45	12.8429	81.3163	947.1949	1.2520	1.4684
22	2014	45	10.2000	86.2143	881.0286	2.5510	2.3265
45	2014	45	11.1571	82.0918	822.6653	3.7643	1.9071
58	2014	45	12.8429	81.3163	947.1949	1.2520	1.4684
37	2014	45	14.5857	79.7449	986.6010	1.8306	0.7429
17	2014	45	11.3000	87.7449	908.7306	1.6735	3.0051
55	2014	45	11.3714	81.8469	883.7786	3.2541	2.1571
46	2014	45	10.9571	86.9898	928.0776	1.4980	1.4643
86	2014	45	11.3429	80.6122	871.7469	1.5224	0.7429
2	2014	45	11.3429	80.6122	871.7469	1.5224	0.7429
4	2014	45	10.5571	84.3673	908.0765	1.5541	1.4316
47	2014	45	16.1286	80.0816	965.6061	2.6429	0.4194
82	2014	45	10.2000	86.2143	881.0286	2.5510	2.3265
19	2014	45	15.8857	79.9490	967.5255	2.5765	1.0214
20	2014	45	9.8286	83.2959	859.8939	2.7959	2.8163
80	2014	45	10.2000	86.2143	881.0286	2.5510	2.3265
3	2014	45	16.0857	77.5408	951.1755	3.3561	0.9694
52	2014	45	11.3000	87.7449	908.7306	1.6735	3.0051
70	2014	45	11.4000	85.0408	916.9061	2.1255	1.3531
64	2014	45	7.5000	83.3061	780.5490	3.5704	2.3929
48	2014	45	12.1286	87.1020	940.4337	0.7112	0.7031
65	2014	45	11.3000	87.7449	908.7306	1.6735	3.0051
44	2014	45	11.4000	85.0408	916.9061	2.1255	1.3531
75	2014	45	7.5000	83.3061	780.5490	3.5704	2.3929
40	2014	45	12.7143	84.8367	952.8500	2.2918	1.4643
11	2014	45	11.3714	81.8469	883.7786	3.2541	2.1571
35	2014	45	12.0714	84.2551	946.7480	1.7092	1.6255
78	2014	45	12.2000	83.0204	905.8847	3.2735	1.8745
28	2014	45	12.1571	80.7653	935.9082	2.3010	1.5429
39	2014	45	11.3000	87.7449	908.7306	1.6735	3.0051
24	2014	45	12.8429	81.3163	947.1949	1.2520	1.4684
63	2014	45	12.7143	84.8367	952.8500	2.2918	1.4643
62	2014	45	10.5571	85.3980	880.4469	1.9755	1.5561
1	2014	45	10.2000	86.2143	881.0286	2.5510	2.3265
31	2014	46	8.4429	86.6939	852.8041	0.4051	0.9327
79	2014	46	12.7571	79.8163	989.2429	0.4092	0.6969
51	2014	46	10.3714	84.9286	948.8224	0.4929	1.5765
14	2014	46	10.1000	85.0816	904.9592	0.7816	1.8745
67	2014	46	9.6857	90.3878	910.2653	0.2214	2.7408
42	2014	46	9.0429	89.9082	882.1918	0.5755	2.3429
50	2014	46	9.7571	85.7041	910.0480	0.1714	1.3204
43	2014	46	9.0429	89.9082	882.1918	0.5755	2.3429
85	2014	46	10.6143	87.0612	919.0265	0.5378	1.3061
25	2014	46	13.6857	84.6224	987.1122	0.8173	0.8643
69	2014	46	11.7714	82.4490	949.5276	0.0429	1.3255
57	2014	46	9.0571	86.8878	894.5612	0.2673	2.0500
9	2014	46	8.8000	87.9898	860.6837	0.5133	2.4031

72	2014	46	10.7286	85.2347	884.7173	0.5643	2.1255
26	2014	46	11.5857	88.9694	873.6224	1.0673	1.5398
7	2014	46	10.5000	86.5714	865.6551	0.8969	1.7796
83	2014	46	15.2571	83.0102	952.9520	0.6367	0.8704
76	2014	46	10.2571	88.6122	930.2327	0.0694	1.4520
36	2014	46	10.6429	84.0306	937.9582	0.7204	1.4153
81	2014	46	10.3714	84.9286	948.8224	0.4929	1.5765
15	2014	46	11.0429	89.1429	942.7939	0.0214	0.5918
32	2014	46	9.0429	89.9082	882.1918	0.5755	2.3429
73	2014	46	13.1286	88.4898	972.6000	0.2684	0.7602
71	2014	46	10.6429	84.0306	937.9582	0.7204	1.4153
41	2014	46	9.5143	88.8163	881.8490	0.3245	1.3786
10	2014	46	11.6714	86.4898	974.5980	1.0204	0.9388
23	2014	46	5.3000	90.1224	780.4459	2.0878	2.2398
27	2014	46	10.5000	86.5714	865.6551	0.8969	1.7796
60	2014	46	10.3714	84.9286	948.8224	0.4929	1.5765
53	2014	46	8.8000	87.9898	860.6837	0.5133	2.4031
66	2014	46	10.1000	85.0816	904.9592	0.7816	1.8745
59	2014	46	9.0571	86.8878	894.5612	0.2673	2.0500
61	2014	46	13.1286	88.4898	972.6000	0.2684	0.7602
84	2014	46	13.1286	88.4898	972.6000	0.2684	0.7602
38	2014	46	9.0571	86.8878	894.5612	0.2673	2.0500
87	2014	46	11.2571	85.4286	907.2847	0.6439	2.0367
34	2014	46	9.0571	86.8878	894.5612	0.2673	2.0500
29	2014	46	11.7714	82.4490	949.5276	0.0429	1.3255
5	2014	46	8.5286	91.5204	838.0061	1.1439	1.4143
8	2014	46	8.8000	87.9898	860.6837	0.5133	2.4031
12	2014	46	8.5286	91.5204	838.0061	1.1439	1.4143
13	2014	46	15.2571	83.0102	952.9520	0.6367	0.8704
18	2014	46	11.8714	83.2041	974.8398	0.8051	1.0224
33	2014	46	9.7571	85.7041	910.0480	0.1714	1.3204
56	2014	46	13.6857	84.6224	987.1122	0.8173	0.8643
77	2014	46	11.0429	89.1429	942.7939	0.0214	0.5918
54	2014	46	8.5286	91.5204	838.0061	1.1439	1.4143
21	2014	46	9.7571	85.7041	910.0480	0.1714	1.3204
68	2014	46	12.7571	79.8163	989.2429	0.4092	0.6969
74	2014	46	13.1286	88.4898	972.6000	0.2684	0.7602
88	2014	46	9.0429	89.9082	882.1918	0.5755	2.3429
16	2014	46	10.2571	88.6122	930.2327	0.0694	1.4520
30	2014	46	10.1000	85.0816	904.9592	0.7816	1.8745
6	2014	46	12.7571	79.8163	989.2429	0.4092	0.6969
49	2014	46	11.7714	82.4490	949.5276	0.0429	1.3255
22	2014	46	9.0429	89.9082	882.1918	0.5755	2.3429
45	2014	46	8.8286	88.5204	822.7520	1.9459	1.6327
58	2014	46	11.7714	82.4490	949.5276	0.0429	1.3255
37	2014	46	12.7571	79.8163	989.2429	0.4092	0.6969
17	2014	46	9.6857	90.3878	910.2653	0.2214	2.7408
55	2014	46	10.7286	85.2347	884.7173	0.5643	2.1255
46	2014	46	10.2571	88.6122	930.2327	0.0694	1.4520
86	2014	46	9.8714	84.2041	872.9122	0.2357	0.7306

2	2014	46	9.8714	84.2041	872.9122	0.2357	0.7306
4	2014	46	9.7571	85.7041	910.0480	0.1714	1.3204
47	2014	46	15.1143	81.7449	967.7378	0.5051	0.3684
82	2014	46	9.0429	89.9082	882.1918	0.5755	2.3429
19	2014	46	14.1714	77.8265	969.6347	0.7939	1.1816
20	2014	46	8.8000	87.9898	860.6837	0.5133	2.4031
80	2014	46	9.0429	89.9082	882.1918	0.5755	2.3429
3	2014	46	15.2571	83.0102	952.9520	0.6367	0.8704
52	2014	46	9.6857	90.3878	910.2653	0.2214	2.7408
70	2014	46	10.6143	87.0612	919.0265	0.5378	1.3061
64	2014	46	5.3000	90.1224	780.4459	2.0878	2.2398
48	2014	46	11.0429	89.1429	942.7939	0.0214	0.5918
65	2014	46	9.6857	90.3878	910.2653	0.2214	2.7408
44	2014	46	10.6143	87.0612	919.0265	0.5378	1.3061
75	2014	46	5.3000	90.1224	780.4459	2.0878	2.2398
40	2014	46	10.8571	88.8980	954.8898	0.9612	1.5061
11	2014	46	10.7286	85.2347	884.7173	0.5643	2.1255
35	2014	46	10.3714	84.9286	948.8224	0.4929	1.5765
78	2014	46	11.2571	85.4286	907.2847	0.6439	2.0367
28	2014	46	10.6429	84.0306	937.9582	0.7204	1.4153
39	2014	46	9.6857	90.3878	910.2653	0.2214	2.7408
24	2014	46	11.7714	82.4490	949.5276	0.0429	1.3255
63	2014	46	10.8571	88.8980	954.8898	0.9612	1.5061
62	2014	46	9.5143	88.8163	881.8490	0.3245	1.3786
1	2014	46	9.0429	89.9082	882.1918	0.5755	2.3429
31	2014	47	8.0571	88.9694	853.5806	0.1582	0.8163
79	2014	47	13.5000	77.4694	990.8153	0.8551	0.6714
51	2014	47	11.0143	84.8776	950.2776	0.5724	1.2949
14	2014	47	11.0286	86.6735	906.2806	0.4694	1.8102
67	2014	47	10.9143	90.3571	911.6031	0.0000	2.5765
42	2014	47	10.1143	89.5510	883.2949	0.0745	2.2633
50	2014	47	9.7571	84.7959	911.3296	0.2265	1.1429
43	2014	47	10.1143	89.5510	883.2949	0.0745	2.2633
85	2014	47	10.1143	86.7653	920.3265	0.2102	1.1796
25	2014	47	14.5143	86.8163	989.0337	0.6347	0.5327
69	2014	47	11.6429	81.5306	951.0592	0.1000	1.1735
57	2014	47	8.8286	86.2653	895.7194	0.0357	1.9990
9	2014	47	9.9714	89.4592	861.6224	0.0388	2.2408
72	2014	47	11.4571	85.5918	885.8541	0.1112	1.9122
26	2014	47	11.9571	90.7245	874.6612	0.4031	1.4184
7	2014	47	11.6143	88.8673	866.6857	0.2429	1.6031
83	2014	47	15.5286	86.3878	954.7194	0.1959	0.7051
76	2014	47	10.3286	85.8571	931.6286	0.4347	1.3133
36	2014	47	11.6714	83.8469	939.4673	0.5724	1.2633
81	2014	47	11.0143	84.8776	950.2776	0.5724	1.2949
15	2014	47	10.7143	89.9490	944.2939	0.4582	0.5112
32	2014	47	10.1143	89.5510	883.2949	0.0745	2.2633
73	2014	47	13.1286	85.3469	974.1796	1.0000	0.6357
71	2014	47	11.6714	83.8469	939.4673	0.5724	1.2633
41	2014	47	9.2714	90.8776	882.9153	0.0000	1.2143

10	2014	47	12.4714	85.9388	976.1704	0.9531	0.8357
23	2014	47	7.1286	91.7653	780.7765	1.6633	2.0602
27	2014	47	11.6143	88.8673	866.6857	0.2429	1.6031
60	2014	47	11.0143	84.8776	950.2776	0.5724	1.2949
53	2014	47	9.9714	89.4592	861.6224	0.0388	2.2408
66	2014	47	11.0286	86.6735	906.2806	0.4694	1.8102
59	2014	47	8.8286	86.2653	895.7194	0.0357	1.9990
61	2014	47	13.1286	85.3469	974.1796	1.0000	0.6357
84	2014	47	13.1286	85.3469	974.1796	1.0000	0.6357
38	2014	47	8.8286	86.2653	895.7194	0.0357	1.9990
87	2014	47	12.4143	84.5714	908.5469	0.1245	1.9449
34	2014	47	8.8286	86.2653	895.7194	0.0357	1.9990
29	2014	47	11.6429	81.5306	951.0592	0.1000	1.1735
5	2014	47	9.9286	93.9796	838.8020	0.6224	1.4388
8	2014	47	9.9714	89.4592	861.6224	0.0388	2.2408
12	2014	47	9.9286	93.9796	838.8020	0.6224	1.4388
13	2014	47	15.5286	86.3878	954.7194	0.1959	0.7051
18	2014	47	12.1571	82.3878	976.3673	1.2112	0.8469
33	2014	47	9.7571	84.7959	911.3296	0.2265	1.1429
56	2014	47	14.5143	86.8163	989.0337	0.6347	0.5327
77	2014	47	10.7143	89.9490	944.2939	0.4582	0.5112
54	2014	47	9.9286	93.9796	838.8020	0.6224	1.4388
21	2014	47	9.7571	84.7959	911.3296	0.2265	1.1429
68	2014	47	13.5000	77.4694	990.8153	0.8551	0.6714
74	2014	47	13.1286	85.3469	974.1796	1.0000	0.6357
88	2014	47	10.1143	89.5510	883.2949	0.0745	2.2633
16	2014	47	10.3286	85.8571	931.6286	0.4347	1.3133
30	2014	47	11.0286	86.6735	906.2806	0.4694	1.8102
6	2014	47	13.5000	77.4694	990.8153	0.8551	0.6714
49	2014	47	11.6429	81.5306	951.0592	0.1000	1.1735
22	2014	47	10.1143	89.5510	883.2949	0.0745	2.2633
45	2014	47	10.1857	90.5714	823.4000	1.2561	1.3133
58	2014	47	11.6429	81.5306	951.0592	0.1000	1.1735
37	2014	47	13.5000	77.4694	990.8153	0.8551	0.6714
17	2014	47	10.9143	90.3571	911.6031	0.0000	2.5765
55	2014	47	11.4571	85.5918	885.8541	0.1112	1.9122
46	2014	47	10.3286	85.8571	931.6286	0.4347	1.3133
86	2014	47	10.2143	86.8673	873.9459	0.0653	0.6653
2	2014	47	10.2143	86.8673	873.9459	0.0653	0.6653
4	2014	47	9.7571	84.7959	911.3296	0.2265	1.1429
47	2014	47	15.4857	82.6531	969.6439	0.0020	0.3112
82	2014	47	10.1143	89.5510	883.2949	0.0745	2.2633
19	2014	47	14.6286	79.2551	971.4827	0.3469	1.0724
20	2014	47	9.9714	89.4592	861.6224	0.0388	2.2408
80	2014	47	10.1143	89.5510	883.2949	0.0745	2.2633
3	2014	47	15.5286	86.3878	954.7194	0.1959	0.7051
52	2014	47	10.9143	90.3571	911.6031	0.0000	2.5765
70	2014	47	10.1143	86.7653	920.3265	0.2102	1.1796
64	2014	47	7.1286	91.7653	780.7765	1.6633	2.0602
48	2014	47	10.7143	89.9490	944.2939	0.4582	0.5112

65	2014	47	10.9143	90.3571	911.6031	0.0000	2.5765
44	2014	47	10.1143	86.7653	920.3265	0.2102	1.1796
75	2014	47	7.1286	91.7653	780.7765	1.6633	2.0602
40	2014	47	12.5286	88.6939	956.3878	0.9245	1.2837
11	2014	47	11.4571	85.5918	885.8541	0.1112	1.9122
35	2014	47	11.0143	84.8776	950.2776	0.5724	1.2949
78	2014	47	12.4143	84.5714	908.5469	0.1245	1.9449
28	2014	47	11.6714	83.8469	939.4673	0.5724	1.2633
39	2014	47	10.9143	90.3571	911.6031	0.0000	2.5765
24	2014	47	11.6429	81.5306	951.0592	0.1000	1.1735
63	2014	47	12.5286	88.6939	956.3878	0.9245	1.2837
62	2014	47	9.2714	90.8776	882.9153	0.0000	1.2143
1	2014	47	10.1143	89.5510	883.2949	0.0745	2.2633
31	2014	48	10.1429	88.4694	851.2847	0.9969	0.7847
79	2014	48	11.7857	79.4388	988.2204	0.7796	0.6990
51	2014	48	9.8857	87.5816	947.7694	0.2969	1.3551
14	2014	48	14.7143	86.5408	903.9531	0.9490	2.2694
67	2014	48	12.3429	91.7245	909.0490	0.1857	2.6520
42	2014	48	12.7714	88.6837	880.8133	0.7367	2.1888
50	2014	48	11.5143	85.5918	908.7143	0.4214	1.3184
43	2014	48	12.7714	88.6837	880.8133	0.7367	2.1888
85	2014	48	12.5143	86.2347	917.7316	0.9255	1.2429
25	2014	48	16.5571	87.2959	986.1908	0.9316	0.6776
69	2014	48	12.4714	82.8776	948.2857	0.0878	1.2592
57	2014	48	10.9857	86.7449	893.1745	0.3378	2.0418
9	2014	48	12.9143	86.9898	859.2806	0.9878	2.5949
72	2014	48	14.4286	83.5918	883.4673	1.9908	1.9561
26	2014	48	15.8429	86.0000	872.3633	3.2490	1.9378
7	2014	48	14.8286	83.7041	864.3633	3.0439	2.0296
83	2014	48	18.5714	83.4184	951.9520	2.6337	0.7592
76	2014	48	11.0286	86.8878	928.9031	0.5378	1.3735
36	2014	48	12.0000	84.8265	936.8102	0.7265	1.1867
81	2014	48	9.8857	87.5816	947.7694	0.2969	1.3551
15	2014	48	12.3857	91.6429	941.4684	0.6531	0.5510
32	2014	48	12.7714	88.6837	880.8133	0.7367	2.1888
73	2014	48	13.0000	86.4592	971.3939	0.9031	0.6918
71	2014	48	12.0000	84.8265	936.8102	0.7265	1.1867
41	2014	48	11.7000	90.2347	880.3969	0.6408	1.2133
10	2014	48	11.3286	88.5510	973.6153	0.5347	0.8041
23	2014	48	8.7000	84.3367	778.7541	4.3510	2.1786
27	2014	48	14.8286	83.7041	864.3633	3.0439	2.0296
60	2014	48	9.8857	87.5816	947.7694	0.2969	1.3551
53	2014	48	12.9143	86.9898	859.2806	0.9878	2.5949
66	2014	48	14.7143	86.5408	903.9531	0.9490	2.2694
59	2014	48	10.9857	86.7449	893.1745	0.3378	2.0418
61	2014	48	13.0000	86.4592	971.3939	0.9031	0.6918
84	2014	48	13.0000	86.4592	971.3939	0.9031	0.6918
38	2014	48	10.9857	86.7449	893.1745	0.3378	2.0418
87	2014	48	15.2857	82.9286	905.9408	1.2276	1.9786
34	2014	48	10.9857	86.7449	893.1745	0.3378	2.0418

29	2014	48	12.4714	82.8776	948.2857	0.0878	1.2592
5	2014	48	13.3714	85.0918	836.5724	3.4776	1.8541
8	2014	48	12.9143	86.9898	859.2806	0.9878	2.5949
12	2014	48	13.3714	85.0918	836.5724	3.4776	1.8541
13	2014	48	18.5714	83.4184	951.9520	2.6337	0.7592
18	2014	48	11.2286	85.4286	973.7612	0.9469	0.7684
33	2014	48	11.5143	85.5918	908.7143	0.4214	1.3184
56	2014	48	16.5571	87.2959	986.1908	0.9316	0.6776
77	2014	48	12.3857	91.6429	941.4684	0.6531	0.5510
54	2014	48	13.3714	85.0918	836.5724	3.4776	1.8541
21	2014	48	11.5143	85.5918	908.7143	0.4214	1.3184
68	2014	48	11.7857	79.4388	988.2204	0.7796	0.6990
74	2014	48	13.0000	86.4592	971.3939	0.9031	0.6918
88	2014	48	12.7714	88.6837	880.8133	0.7367	2.1888
16	2014	48	11.0286	86.8878	928.9031	0.5378	1.3735
30	2014	48	14.7143	86.5408	903.9531	0.9490	2.2694
6	2014	48	11.7857	79.4388	988.2204	0.7796	0.6990
49	2014	48	12.4714	82.8776	948.2857	0.0878	1.2592
22	2014	48	12.7714	88.6837	880.8133	0.7367	2.1888
45	2014	48	13.1571	79.6939	821.3357	4.2418	1.6827
58	2014	48	12.4714	82.8776	948.2857	0.0878	1.2592
37	2014	48	11.7857	79.4388	988.2204	0.7796	0.6990
17	2014	48	12.3429	91.7245	909.0490	0.1857	2.6520
55	2014	48	14.4286	83.5918	883.4673	1.9908	1.9561
46	2014	48	11.0286	86.8878	928.9031	0.5378	1.3735
86	2014	48	13.1286	86.1327	871.4980	0.7020	0.6898
2	2014	48	13.1286	86.1327	871.4980	0.7020	0.6898
4	2014	48	11.5143	85.5918	908.7143	0.4214	1.3184
47	2014	48	18.5143	81.9388	966.7204	0.9408	0.3194
82	2014	48	12.7714	88.6837	880.8133	0.7367	2.1888
19	2014	48	18.2000	82.6939	968.7745	0.9184	0.8816
20	2014	48	12.9143	86.9898	859.2806	0.9878	2.5949
80	2014	48	12.7714	88.6837	880.8133	0.7367	2.1888
3	2014	48	18.5714	83.4184	951.9520	2.6337	0.7592
52	2014	48	12.3429	91.7245	909.0490	0.1857	2.6520
70	2014	48	12.5143	86.2347	917.7316	0.9255	1.2429
64	2014	48	8.7000	84.3367	778.7541	4.3510	2.1786
48	2014	48	12.3857	91.6429	941.4684	0.6531	0.5510
65	2014	48	12.3429	91.7245	909.0490	0.1857	2.6520
44	2014	48	12.5143	86.2347	917.7316	0.9255	1.2429
75	2014	48	8.7000	84.3367	778.7541	4.3510	2.1786
40	2014	48	11.8714	89.6224	953.8929	0.9561	1.3000
11	2014	48	14.4286	83.5918	883.4673	1.9908	1.9561
35	2014	48	9.8857	87.5816	947.7694	0.2969	1.3551
78	2014	48	15.2857	82.9286	905.9408	1.2276	1.9786
28	2014	48	12.0000	84.8265	936.8102	0.7265	1.1867
39	2014	48	12.3429	91.7245	909.0490	0.1857	2.6520
24	2014	48	12.4714	82.8776	948.2857	0.0878	1.2592
63	2014	48	11.8714	89.6224	953.8929	0.9561	1.3000
62	2014	48	11.7000	90.2347	880.3969	0.6408	1.2133

1	2014	48	12.7714	88.6837	880.8133	0.7367	2.1888
31	2014	49	5.9286	85.8469	849.4286	1.4847	0.9092
79	2014	49	9.9000	78.4082	987.0612	0.9122	0.7429
51	2014	49	8.0429	85.0918	946.2633	1.0092	1.6418
14	2014	49	7.5000	83.0714	902.0276	1.9714	2.5612
67	2014	49	7.4857	88.8469	907.1724	0.7092	2.6755
42	2014	49	6.7000	85.3163	878.8888	1.7429	2.4020
50	2014	49	7.7000	82.8878	906.8204	0.3735	1.5459
43	2014	49	6.7000	85.3163	878.8888	1.7429	2.4020
85	2014	49	8.3000	82.5408	915.7969	1.1786	1.3867
25	2014	49	11.2143	80.2041	984.3316	1.6704	1.1357
69	2014	49	9.7000	77.9694	946.5714	1.0133	1.6112
57	2014	49	6.3143	85.8163	891.2704	0.6449	2.1112
9	2014	49	6.9429	81.7959	857.3520	2.5102	2.6969
72	2014	49	8.5429	79.1837	881.4684	3.8796	2.2357
26	2014	49	9.4143	78.4796	870.4092	4.7847	2.2459
7	2014	49	8.7571	77.4490	862.4061	4.7010	2.2857
83	2014	49	13.8429	75.2245	949.5918	4.6612	0.9469
76	2014	49	7.8286	84.8571	927.1408	0.7418	1.5898
36	2014	49	8.4714	82.3980	935.0265	1.1837	1.4337
81	2014	49	8.0429	85.0918	946.2633	1.0092	1.6418
15	2014	49	9.1857	87.2347	939.6306	0.4480	0.8255
32	2014	49	6.7000	85.3163	878.8888	1.7429	2.4020
73	2014	49	10.5714	83.3367	969.8490	0.5694	0.8224
71	2014	49	8.4714	82.3980	935.0265	1.1837	1.4337
41	2014	49	6.8143	87.3367	878.4653	1.3204	1.2847
10	2014	49	9.0286	86.4592	972.2184	1.1551	1.0276
23	2014	49	3.9429	78.4184	777.0653	5.3388	2.5388
27	2014	49	8.7571	77.4490	862.4061	4.7010	2.2857
60	2014	49	8.0429	85.0918	946.2633	1.0092	1.6418
53	2014	49	6.9429	81.7959	857.3520	2.5102	2.6969
66	2014	49	7.5000	83.0714	902.0276	1.9714	2.5612
59	2014	49	6.3143	85.8163	891.2704	0.6449	2.1112
61	2014	49	10.5714	83.3367	969.8490	0.5694	0.8224
84	2014	49	10.5714	83.3367	969.8490	0.5694	0.8224
38	2014	49	6.3143	85.8163	891.2704	0.6449	2.1112
87	2014	49	9.3714	78.4898	903.8878	2.6082	2.1622
34	2014	49	6.3143	85.8163	891.2704	0.6449	2.1112
29	2014	49	9.7000	77.9694	946.5714	1.0133	1.6112
5	2014	49	6.6571	75.6531	834.7327	4.5133	2.1663
8	2014	49	6.9429	81.7959	857.3520	2.5102	2.6969
12	2014	49	6.6571	75.6531	834.7327	4.5133	2.1663
13	2014	49	13.8429	75.2245	949.5918	4.6612	0.9469
18	2014	49	8.7429	84.0918	972.5327	0.6520	0.9388
33	2014	49	7.7000	82.8878	906.8204	0.3735	1.5459
56	2014	49	11.2143	80.2041	984.3316	1.6704	1.1357
77	2014	49	9.1857	87.2347	939.6306	0.4480	0.8255
54	2014	49	6.6571	75.6531	834.7327	4.5133	2.1663
21	2014	49	7.7000	82.8878	906.8204	0.3735	1.5459
68	2014	49	9.9000	78.4082	987.0612	0.9122	0.7429

74	2014	49	10.5714	83.3367	969.8490	0.5694	0.8224
88	2014	49	6.7000	85.3163	878.8888	1.7429	2.4020
16	2014	49	7.8286	84.8571	927.1408	0.7418	1.5898
30	2014	49	7.5000	83.0714	902.0276	1.9714	2.5612
6	2014	49	9.9000	78.4082	987.0612	0.9122	0.7429
49	2014	49	9.7000	77.9694	946.5714	1.0133	1.6112
22	2014	49	6.7000	85.3163	878.8888	1.7429	2.4020
45	2014	49	7.4286	70.6020	819.5347	5.6582	2.0898
58	2014	49	9.7000	77.9694	946.5714	1.0133	1.6112
37	2014	49	9.9000	78.4082	987.0612	0.9122	0.7429
17	2014	49	7.4857	88.8469	907.1724	0.7092	2.6755
55	2014	49	8.5429	79.1837	881.4684	3.8796	2.2357
46	2014	49	7.8286	84.8571	927.1408	0.7418	1.5898
86	2014	49	8.3857	81.4898	869.4806	1.4969	0.7684
2	2014	49	8.3857	81.4898	869.4806	1.4969	0.7684
4	2014	49	7.7000	82.8878	906.8204	0.3735	1.5459
47	2014	49	13.3143	77.0000	964.4143	2.4184	0.5255
82	2014	49	6.7000	85.3163	878.8888	1.7429	2.4020
19	2014	49	11.9429	78.3673	966.7031	1.6908	1.1204
20	2014	49	6.9429	81.7959	857.3520	2.5102	2.6969
80	2014	49	6.7000	85.3163	878.8888	1.7429	2.4020
3	2014	49	13.8429	75.2245	949.5918	4.6612	0.9469
52	2014	49	7.4857	88.8469	907.1724	0.7092	2.6755
70	2014	49	8.3000	82.5408	915.7969	1.1786	1.3867
64	2014	49	3.9429	78.4184	777.0653	5.3388	2.5388
48	2014	49	9.1857	87.2347	939.6306	0.4480	0.8255
65	2014	49	7.4857	88.8469	907.1724	0.7092	2.6755
44	2014	49	8.3000	82.5408	915.7969	1.1786	1.3867
75	2014	49	3.9429	78.4184	777.0653	5.3388	2.5388
40	2014	49	7.8143	88.6837	952.3429	1.1857	1.6480
11	2014	49	8.5429	79.1837	881.4684	3.8796	2.2357
35	2014	49	8.0429	85.0918	946.2633	1.0092	1.6418
78	2014	49	9.3714	78.4898	903.8878	2.6082	2.1622
28	2014	49	8.4714	82.3980	935.0265	1.1837	1.4337
39	2014	49	7.4857	88.8469	907.1724	0.7092	2.6755
24	2014	49	9.7000	77.9694	946.5714	1.0133	1.6112
63	2014	49	7.8143	88.6837	952.3429	1.1857	1.6480
62	2014	49	6.8143	87.3367	878.4653	1.3204	1.2847
1	2014	49	6.7000	85.3163	878.8888	1.7429	2.4020
31	2014	50	2.7286	84.1939	852.0133	0.5939	1.0102
79	2014	50	7.8000	67.3469	991.3898	1.4796	0.7867
51	2014	50	5.8286	71.5204	950.0500	2.2378	1.7224
14	2014	50	5.6857	74.8061	905.1143	2.0878	2.3122
67	2014	50	5.1429	77.7245	910.4980	1.1714	2.8439
42	2014	50	4.1857	79.3061	881.9020	1.4204	2.6306
50	2014	50	5.2429	75.5510	910.2388	0.1602	1.6541
43	2014	50	4.1857	79.3061	881.9020	1.4204	2.6306
85	2014	50	5.6571	78.7857	919.2643	0.6837	1.3980
25	2014	50	9.6286	71.4082	988.7214	2.4888	1.2306
69	2014	50	6.9143	66.2245	950.5286	1.9265	1.6888

57	2014	50	4.0286	81.7551	894.4857	0.7837	1.9806
9	2014	50	3.4714	80.4184	860.0449	1.8122	2.4480
72	2014	50	5.2571	75.8776	884.4286	2.2878	2.3163
26	2014	50	6.0429	80.8469	873.1653	1.9500	1.9041
7	2014	50	5.0286	80.0612	865.1245	2.0561	1.9643
83	2014	50	11.2429	68.3571	953.4388	2.5694	0.9684
76	2014	50	5.5143	75.4082	930.8765	1.1286	1.6531
36	2014	50	6.1429	71.1837	938.7684	1.6541	1.5806
81	2014	50	5.8286	71.5204	950.0500	2.2378	1.7224
15	2014	50	6.6571	79.1633	943.5204	0.6031	0.9939
32	2014	50	4.1857	79.3061	881.9020	1.4204	2.6306
73	2014	50	8.3286	71.2959	974.0827	0.9286	0.8857
71	2014	50	6.1429	71.1837	938.7684	1.6541	1.5806
41	2014	50	4.2714	83.7143	881.5173	0.7378	1.3776
10	2014	50	7.0571	74.9592	976.3082	2.6061	1.1510
23	2014	50	0.1571	87.0816	778.6633	2.1133	2.4888
27	2014	50	5.0286	80.0612	865.1245	2.0561	1.9643
60	2014	50	5.8286	71.5204	950.0500	2.2378	1.7224
53	2014	50	3.4714	80.4184	860.0449	1.8122	2.4480
66	2014	50	5.6857	74.8061	905.1143	2.0878	2.3122
59	2014	50	4.0286	81.7551	894.4857	0.7837	1.9806
61	2014	50	8.3286	71.2959	974.0827	0.9286	0.8857
84	2014	50	8.3286	71.2959	974.0827	0.9286	0.8857
38	2014	50	4.0286	81.7551	894.4857	0.7837	1.9806
87	2014	50	6.5143	72.1224	907.2214	1.8357	2.3184
34	2014	50	4.0286	81.7551	894.4857	0.7837	1.9806
29	2014	50	6.9143	66.2245	950.5286	1.9265	1.6888
5	2014	50	2.8143	82.6429	837.1408	1.5316	2.0020
8	2014	50	3.4714	80.4184	860.0449	1.8122	2.4480
12	2014	50	2.8143	82.6429	837.1408	1.5316	2.0020
13	2014	50	11.2429	68.3571	953.4388	2.5694	0.9684
18	2014	50	6.5000	74.3367	976.7194	1.0051	1.0867
33	2014	50	5.2429	75.5510	910.2388	0.1602	1.6541
56	2014	50	9.6286	71.4082	988.7214	2.4888	1.2306
77	2014	50	6.6571	79.1633	943.5204	0.6031	0.9939
54	2014	50	2.8143	82.6429	837.1408	1.5316	2.0020
21	2014	50	5.2429	75.5510	910.2388	0.1602	1.6541
68	2014	50	7.8000	67.3469	991.3898	1.4796	0.7867
74	2014	50	8.3286	71.2959	974.0827	0.9286	0.8857
88	2014	50	4.1857	79.3061	881.9020	1.4204	2.6306
16	2014	50	5.5143	75.4082	930.8765	1.1286	1.6531
30	2014	50	5.6857	74.8061	905.1143	2.0878	2.3122
6	2014	50	7.8000	67.3469	991.3898	1.4796	0.7867
49	2014	50	6.9143	66.2245	950.5286	1.9265	1.6888
22	2014	50	4.1857	79.3061	881.9020	1.4204	2.6306
45	2014	50	3.5143	80.8265	821.5888	2.4867	1.7551
58	2014	50	6.9143	66.2245	950.5286	1.9265	1.6888
37	2014	50	7.8000	67.3469	991.3898	1.4796	0.7867
17	2014	50	5.1429	77.7245	910.4980	1.1714	2.8439
55	2014	50	5.2571	75.8776	884.4286	2.2878	2.3163

46	2014	50	5.5143	75.4082	930.8765	1.1286	1.6531
86	2014	50	4.6857	79.1633	872.3429	0.9337	0.8071
2	2014	50	4.6857	79.1633	872.3429	0.9337	0.8071
4	2014	50	5.2429	75.5510	910.2388	0.1602	1.6541
47	2014	50	11.3429	68.2245	968.5398	2.0276	0.7255
82	2014	50	4.1857	79.3061	881.9020	1.4204	2.6306
19	2014	50	10.2429	66.8878	970.6980	2.0204	1.3918
20	2014	50	3.4714	80.4184	860.0449	1.8122	2.4480
80	2014	50	4.1857	79.3061	881.9020	1.4204	2.6306
3	2014	50	11.2429	68.3571	953.4388	2.5694	0.9684
52	2014	50	5.1429	77.7245	910.4980	1.1714	2.8439
70	2014	50	5.6571	78.7857	919.2643	0.6837	1.3980
64	2014	50	0.1571	87.0816	778.6633	2.1133	2.4888
48	2014	50	6.6571	79.1633	943.5204	0.6031	0.9939
65	2014	50	5.1429	77.7245	910.4980	1.1714	2.8439
44	2014	50	5.6571	78.7857	919.2643	0.6837	1.3980
75	2014	50	0.1571	87.0816	778.6633	2.1133	2.4888
40	2014	50	6.2857	80.1020	956.1306	2.0939	1.7592
11	2014	50	5.2571	75.8776	884.4286	2.2878	2.3163
35	2014	50	5.8286	71.5204	950.0500	2.2378	1.7224
78	2014	50	6.5143	72.1224	907.2214	1.8357	2.3184
28	2014	50	6.1429	71.1837	938.7684	1.6541	1.5806
39	2014	50	5.1429	77.7245	910.4980	1.1714	2.8439
24	2014	50	6.9143	66.2245	950.5286	1.9265	1.6888
63	2014	50	6.2857	80.1020	956.1306	2.0939	1.7592
62	2014	50	4.2714	83.7143	881.5173	0.7378	1.3776
1	2014	50	4.1857	79.3061	881.9020	1.4204	2.6306
31	2014	51	1.9429	84.5408	855.1265	0.3051	0.9735
79	2014	51	8.7571	61.1429	995.0398	1.7398	0.7939
51	2014	51	6.0143	63.9490	953.5490	1.9796	1.4980
14	2014	51	6.0429	67.8469	908.4704	1.5816	2.1000
67	2014	51	5.2857	70.2551	913.9490	0.8888	3.0449
42	2014	51	3.6571	78.6531	885.2337	0.9092	2.4112
50	2014	51	4.0000	75.9490	913.9265	0.0235	1.5245
43	2014	51	3.6571	78.6531	885.2337	0.9092	2.4112
85	2014	51	4.2429	81.2245	923.2276	0.4398	1.2286
25	2014	51	9.0429	67.0612	992.8316	3.1418	1.1194
69	2014	51	7.3286	62.8980	954.2898	1.4020	1.4867
57	2014	51	2.9286	83.1531	898.0214	0.8316	1.7092
9	2014	51	3.6714	83.0408	863.1694	0.5449	2.2806
72	2014	51	5.3429	76.7755	887.8296	1.0418	2.2673
26	2014	51	6.1429	85.8571	876.4235	0.4184	1.6398
7	2014	51	5.0429	85.0102	868.3031	0.4898	1.6653
83	2014	51	10.5286	66.0000	957.8071	1.2663	0.9847
76	2014	51	5.7429	71.8265	934.5092	0.7469	1.5449
36	2014	51	6.5143	63.6429	942.4367	1.3531	1.5020
81	2014	51	6.0143	63.9490	953.5490	1.9796	1.4980
15	2014	51	6.1429	79.7347	947.3643	0.7224	0.8684
32	2014	51	3.6571	78.6531	885.2337	0.9092	2.4112
73	2014	51	8.9143	66.7245	977.9551	1.0408	0.9643

71	2014	51	6.5143	63.6429	942.4367	1.3531	1.5020
41	2014	51	3.6286	83.3571	884.9357	0.4020	1.1980
10	2014	51	6.9000	65.8367	979.9765	3.4480	1.2082
23	2014	51	-1.0143	92.6735	780.6643	0.8837	2.2939
27	2014	51	5.0429	85.0102	868.3031	0.4898	1.6653
60	2014	51	6.0143	63.9490	953.5490	1.9796	1.4980
53	2014	51	3.6714	83.0408	863.1694	0.5449	2.2806
66	2014	51	6.0429	67.8469	908.4704	1.5816	2.1000
59	2014	51	2.9286	83.1531	898.0214	0.8316	1.7092
61	2014	51	8.9143	66.7245	977.9551	1.0408	0.9643
84	2014	51	8.9143	66.7245	977.9551	1.0408	0.9643
38	2014	51	2.9286	83.1531	898.0214	0.8316	1.7092
87	2014	51	5.7714	71.3061	910.9602	0.8306	2.4041
34	2014	51	2.9286	83.1531	898.0214	0.8316	1.7092
29	2014	51	7.3286	62.8980	954.2898	1.4020	1.4867
5	2014	51	3.1571	90.8061	839.9316	0.3878	1.7194
8	2014	51	3.6714	83.0408	863.1694	0.5449	2.2806
12	2014	51	3.1571	90.8061	839.9316	0.3878	1.7194
13	2014	51	10.5286	66.0000	957.8071	1.2663	0.9847
18	2014	51	7.5429	68.0918	980.2265	1.2908	1.0204
33	2014	51	4.0000	75.9490	913.9265	0.0235	1.5245
56	2014	51	9.0429	67.0612	992.8316	3.1418	1.1194
77	2014	51	6.1429	79.7347	947.3643	0.7224	0.8684
54	2014	51	3.1571	90.8061	839.9316	0.3878	1.7194
21	2014	51	4.0000	75.9490	913.9265	0.0235	1.5245
68	2014	51	8.7571	61.1429	995.0398	1.7398	0.7939
74	2014	51	8.9143	66.7245	977.9551	1.0408	0.9643
88	2014	51	3.6571	78.6531	885.2337	0.9092	2.4112
16	2014	51	5.7429	71.8265	934.5092	0.7469	1.5449
30	2014	51	6.0429	67.8469	908.4704	1.5816	2.1000
6	2014	51	8.7571	61.1429	995.0398	1.7398	0.7939
49	2014	51	7.3286	62.8980	954.2898	1.4020	1.4867
22	2014	51	3.6571	78.6531	885.2337	0.9092	2.4112
45	2014	51	3.4000	89.9592	824.1357	0.9102	1.3806
58	2014	51	7.3286	62.8980	954.2898	1.4020	1.4867
37	2014	51	8.7571	61.1429	995.0398	1.7398	0.7939
17	2014	51	5.2857	70.2551	913.9490	0.8888	3.0449
55	2014	51	5.3429	76.7755	887.8296	1.0418	2.2673
46	2014	51	5.7429	71.8265	934.5092	0.7469	1.5449
86	2014	51	4.3286	82.0612	875.9918	0.2010	1.0071
2	2014	51	4.3286	82.0612	875.9918	0.2010	1.0071
4	2014	51	4.0000	75.9490	913.9265	0.0235	1.5245
47	2014	51	10.9143	62.7755	972.9306	1.4418	0.6969
82	2014	51	3.6571	78.6531	885.2337	0.9092	2.4112
19	2014	51	10.4429	58.9898	974.7735	2.4612	1.3592
20	2014	51	3.6714	83.0408	863.1694	0.5449	2.2806
80	2014	51	3.6571	78.6531	885.2337	0.9092	2.4112
3	2014	51	10.5286	66.0000	957.8071	1.2663	0.9847
52	2014	51	5.2857	70.2551	913.9490	0.8888	3.0449
70	2014	51	4.2429	81.2245	923.2276	0.4398	1.2286

64	2014	51	-1.0143	92.6735	780.6643	0.8837	2.2939
48	2014	51	6.1429	79.7347	947.3643	0.7224	0.8684
65	2014	51	5.2857	70.2551	913.9490	0.8888	3.0449
44	2014	51	4.2429	81.2245	923.2276	0.4398	1.2286
75	2014	51	-1.0143	92.6735	780.6643	0.8837	2.2939
40	2014	51	5.8286	70.1429	959.7000	3.0541	1.6102
11	2014	51	5.3429	76.7755	887.8296	1.0418	2.2673
35	2014	51	6.0143	63.9490	953.5490	1.9796	1.4980
78	2014	51	5.7714	71.3061	910.9602	0.8306	2.4041
28	2014	51	6.5143	63.6429	942.4367	1.3531	1.5020
39	2014	51	5.2857	70.2551	913.9490	0.8888	3.0449
24	2014	51	7.3286	62.8980	954.2898	1.4020	1.4867
63	2014	51	5.8286	70.1429	959.7000	3.0541	1.6102
62	2014	51	3.6286	83.3571	884.9357	0.4020	1.1980
1	2014	51	3.6571	78.6531	885.2337	0.9092	2.4112
31	2014	52	3.5286	83.9082	855.2337	1.0888	1.0357
79	2014	52	7.8286	60.5816	994.9735	3.4143	0.7929
51	2014	52	5.5000	65.9184	953.5857	2.5214	1.2612
14	2014	52	6.2571	67.9898	908.6694	2.1755	1.9459
67	2014	52	5.9000	72.2551	914.0949	1.0694	2.9000
42	2014	52	5.2286	79.8367	885.3673	1.9051	2.1255
50	2014	52	5.1429	78.6939	914.0602	0.6663	1.4622
43	2014	52	5.2286	79.8367	885.3673	1.9051	2.1255
85	2014	52	5.7143	82.8163	923.4418	0.8724	1.3194
25	2014	52	8.5571	69.1020	993.1296	3.7786	1.1286
69	2014	52	6.6000	66.4898	954.3888	1.6459	1.3806
57	2014	52	4.4714	83.6224	898.1429	1.3184	1.8245
9	2014	52	4.7857	81.6939	863.3214	1.4500	2.1816
72	2014	52	6.4571	77.3776	888.0643	2.1806	2.0816
26	2014	52	7.8857	82.1122	876.6908	1.6173	1.7112
7	2014	52	6.8286	82.5408	868.5051	1.8041	1.5745
83	2014	52	10.8857	69.1939	958.2510	2.5500	0.9663
76	2014	52	6.0143	72.3061	934.5286	1.1337	1.5561
36	2014	52	6.0857	65.6531	942.5571	2.1102	1.5449
81	2014	52	5.5000	65.9184	953.5857	2.5214	1.2612
15	2014	52	6.1286	82.5102	947.4622	1.2541	0.7612
32	2014	52	5.2286	79.8367	885.3673	1.9051	2.1255
73	2014	52	8.2857	68.5714	977.9816	2.0194	1.0755
71	2014	52	6.0857	65.6531	942.5571	2.1102	1.5449
41	2014	52	4.6571	83.4796	885.0408	0.9684	1.0724
10	2014	52	6.2714	66.5000	980.0551	4.5898	1.1837
23	2014	52	1.9000	87.7041	780.7459	2.9112	2.4582
27	2014	52	6.8286	82.5408	868.5051	1.8041	1.5745
60	2014	52	5.5000	65.9184	953.5857	2.5214	1.2612
53	2014	52	4.7857	81.6939	863.3214	1.4500	2.1816
66	2014	52	6.2571	67.9898	908.6694	2.1755	1.9459
59	2014	52	4.4714	83.6224	898.1429	1.3184	1.8245
61	2014	52	8.2857	68.5714	977.9816	2.0194	1.0755
84	2014	52	8.2857	68.5714	977.9816	2.0194	1.0755
38	2014	52	4.4714	83.6224	898.1429	1.3184	1.8245

87	2014	52	6.7714	74.6939	911.1990	1.6122	2.3061
34	2014	52	4.4714	83.6224	898.1429	1.3184	1.8245
29	2014	52	6.6000	66.4898	954.3888	1.6459	1.3806
5	2014	52	5.0143	86.9796	840.0602	2.0520	1.7082
8	2014	52	4.7857	81.6939	863.3214	1.4500	2.1816
12	2014	52	5.0143	86.9796	840.0602	2.0520	1.7082
13	2014	52	10.8857	69.1939	958.2510	2.5500	0.9663
18	2014	52	7.0286	65.0204	980.0663	2.6704	0.9194
33	2014	52	5.1429	78.6939	914.0602	0.6663	1.4622
56	2014	52	8.5571	69.1020	993.1296	3.7786	1.1286
77	2014	52	6.1286	82.5102	947.4622	1.2541	0.7612
54	2014	52	5.0143	86.9796	840.0602	2.0520	1.7082
21	2014	52	5.1429	78.6939	914.0602	0.6663	1.4622
68	2014	52	7.8286	60.5816	994.9735	3.4143	0.7929
74	2014	52	8.2857	68.5714	977.9816	2.0194	1.0755
88	2014	52	5.2286	79.8367	885.3673	1.9051	2.1255
16	2014	52	6.0143	72.3061	934.5286	1.1337	1.5561
30	2014	52	6.2571	67.9898	908.6694	2.1755	1.9459
6	2014	52	7.8286	60.5816	994.9735	3.4143	0.7929
49	2014	52	6.6000	66.4898	954.3888	1.6459	1.3806
22	2014	52	5.2286	79.8367	885.3673	1.9051	2.1255
45	2014	52	6.4714	83.4796	824.2786	2.5173	1.6429
58	2014	52	6.6000	66.4898	954.3888	1.6459	1.3806
37	2014	52	7.8286	60.5816	994.9735	3.4143	0.7929
17	2014	52	5.9000	72.2551	914.0949	1.0694	2.9000
55	2014	52	6.4571	77.3776	888.0643	2.1806	2.0816
46	2014	52	6.0143	72.3061	934.5286	1.1337	1.5561
86	2014	52	5.3000	82.4592	876.4939	0.1786	1.0867
2	2014	52	5.3000	82.4592	876.4939	0.1786	1.0867
4	2014	52	5.1429	78.6939	914.0602	0.6663	1.4622
47	2014	52	11.0000	66.4286	973.3245	2.1918	0.6776
82	2014	52	5.2286	79.8367	885.3673	1.9051	2.1255
19	2014	52	9.2286	62.1224	975.0929	3.1765	1.2133
20	2014	52	4.7857	81.6939	863.3214	1.4500	2.1816
80	2014	52	5.2286	79.8367	885.3673	1.9051	2.1255
3	2014	52	10.8857	69.1939	958.2510	2.5500	0.9663
52	2014	52	5.9000	72.2551	914.0949	1.0694	2.9000
70	2014	52	5.7143	82.8163	923.4418	0.8724	1.3194
64	2014	52	1.9000	87.7041	780.7459	2.9112	2.4582
48	2014	52	6.1286	82.5102	947.4622	1.2541	0.7612
65	2014	52	5.9000	72.2551	914.0949	1.0694	2.9000
44	2014	52	5.7143	82.8163	923.4418	0.8724	1.3194
75	2014	52	1.9000	87.7041	780.7459	2.9112	2.4582
40	2014	52	5.9857	68.6224	959.8296	3.6878	1.3286
11	2014	52	6.4571	77.3776	888.0643	2.1806	2.0816
35	2014	52	5.5000	65.9184	953.5857	2.5214	1.2612
78	2014	52	6.7714	74.6939	911.1990	1.6122	2.3061
28	2014	52	6.0857	65.6531	942.5571	2.1102	1.5449
39	2014	52	5.9000	72.2551	914.0949	1.0694	2.9000
24	2014	52	6.6000	66.4898	954.3888	1.6459	1.3806

63	2014	52	5.9857	68.6224	959.8296	3.6878	1.3286
62	2014	52	4.6571	83.4796	885.0408	0.9684	1.0724
1	2014	52	5.2286	79.8367	885.3673	1.9051	2.1255
31	2015	1	6.5286	79.1327	853.7245	2.2755	1.0184
79	2015	1	9.1286	62.8776	992.0347	3.6143	0.8102
51	2015	1	8.0429	67.0204	951.7010	3.2643	1.1980
14	2015	1	9.4286	68.1837	907.1316	3.1745	1.8929
67	2015	1	8.9857	71.1837	912.3847	2.1051	2.7071
42	2015	1	8.2429	73.7857	883.7684	2.9031	2.1959
50	2015	1	7.4571	74.8265	912.2347	1.8582	1.3092
43	2015	1	8.2429	73.7857	883.7684	2.9031	2.1959
85	2015	1	7.7000	80.0204	921.4714	1.9020	1.3429
25	2015	1	10.7429	73.6735	991.2837	3.9204	1.0582
69	2015	1	9.1286	66.8163	952.4367	2.3776	1.1633
57	2015	1	7.9143	73.5102	896.3551	2.2786	2.2622
9	2015	1	7.6429	75.4490	861.8908	2.8000	2.0755
72	2015	1	9.2857	73.3469	886.5000	3.1847	1.8337
26	2015	1	9.7714	77.2551	875.2265	3.2816	1.8765
7	2015	1	8.7857	77.0714	867.0673	3.3061	1.5398
83	2015	1	12.4571	73.9592	956.3255	3.7439	0.8031
76	2015	1	7.7857	70.0306	932.6367	2.5694	1.5449
36	2015	1	9.1571	66.8673	940.6878	3.2867	1.5224
81	2015	1	8.0429	67.0204	951.7010	3.2643	1.1980
15	2015	1	6.8286	83.7245	945.6490	1.6765	0.6337
32	2015	1	8.2429	73.7857	883.7684	2.9031	2.1959
73	2015	1	9.3000	70.4490	976.0306	2.3306	0.9204
71	2015	1	9.1571	66.8673	940.6878	3.2867	1.5224
41	2015	1	7.7143	78.7449	883.3673	2.1816	1.0439
10	2015	1	8.9000	70.8061	978.0714	4.4582	0.9949
23	2015	1	6.1429	79.8061	780.0061	4.3541	2.4327
27	2015	1	8.7857	77.0714	867.0673	3.3061	1.5398
60	2015	1	8.0429	67.0204	951.7010	3.2643	1.1980
53	2015	1	7.6429	75.4490	861.8908	2.8000	2.0755
66	2015	1	9.4286	68.1837	907.1316	3.1745	1.8929
59	2015	1	7.9143	73.5102	896.3551	2.2786	2.2622
61	2015	1	9.3000	70.4490	976.0306	2.3306	0.9204
84	2015	1	9.3000	70.4490	976.0306	2.3306	0.9204
38	2015	1	7.9143	73.5102	896.3551	2.2786	2.2622
87	2015	1	9.6429	73.2347	909.4459	2.9316	2.1847
34	2015	1	7.9143	73.5102	896.3551	2.2786	2.2622
29	2015	1	9.1286	66.8163	952.4367	2.3776	1.1633
5	2015	1	7.6714	79.6837	838.8388	3.6122	1.7541
8	2015	1	7.6429	75.4490	861.8908	2.8000	2.0755
12	2015	1	7.6714	79.6837	838.8388	3.6122	1.7541
13	2015	1	12.4571	73.9592	956.3255	3.7439	0.8031
18	2015	1	8.2143	64.8367	978.0745	3.2827	1.0214
33	2015	1	7.4571	74.8265	912.2347	1.8582	1.3092
56	2015	1	10.7429	73.6735	991.2837	3.9204	1.0582
77	2015	1	6.8286	83.7245	945.6490	1.6765	0.6337
54	2015	1	7.6714	79.6837	838.8388	3.6122	1.7541

21	2015	1	7.4571	74.8265	912.2347	1.8582	1.3092
68	2015	1	9.1286	62.8776	992.0347	3.6143	0.8102
74	2015	1	9.3000	70.4490	976.0306	2.3306	0.9204
88	2015	1	8.2429	73.7857	883.7684	2.9031	2.1959
16	2015	1	7.7857	70.0306	932.6367	2.5694	1.5449
30	2015	1	9.4286	68.1837	907.1316	3.1745	1.8929
6	2015	1	9.1286	62.8776	992.0347	3.6143	0.8102
49	2015	1	9.1286	66.8163	952.4367	2.3776	1.1633
22	2015	1	8.2429	73.7857	883.7684	2.9031	2.1959
45	2015	1	8.4000	76.2143	823.2041	4.3663	1.8408
58	2015	1	9.1286	66.8163	952.4367	2.3776	1.1633
37	2015	1	9.1286	62.8776	992.0347	3.6143	0.8102
17	2015	1	8.9857	71.1837	912.3847	2.1051	2.7071
55	2015	1	9.2857	73.3469	886.5000	3.1847	1.8337
46	2015	1	7.7857	70.0306	932.6367	2.5694	1.5449
86	2015	1	8.1143	77.4796	874.7949	0.0510	0.9398
2	2015	1	8.1143	77.4796	874.7949	0.0510	0.9398
4	2015	1	7.4571	74.8265	912.2347	1.8582	1.3092
47	2015	1	12.6143	71.0000	971.2929	3.0510	0.6235
82	2015	1	8.2429	73.7857	883.7684	2.9031	2.1959
19	2015	1	10.7571	69.4796	973.3582	3.5888	0.9847
20	2015	1	7.6429	75.4490	861.8908	2.8000	2.0755
80	2015	1	8.2429	73.7857	883.7684	2.9031	2.1959
3	2015	1	12.4571	73.9592	956.3255	3.7439	0.8031
52	2015	1	8.9857	71.1837	912.3847	2.1051	2.7071
70	2015	1	7.7000	80.0204	921.4714	1.9020	1.3429
64	2015	1	6.1429	79.8061	780.0061	4.3541	2.4327
48	2015	1	6.8286	83.7245	945.6490	1.6765	0.6337
65	2015	1	8.9857	71.1837	912.3847	2.1051	2.7071
44	2015	1	7.7000	80.0204	921.4714	1.9020	1.3429
75	2015	1	6.1429	79.8061	780.0061	4.3541	2.4327
40	2015	1	9.3571	70.2143	958.0112	3.7704	1.1918
11	2015	1	9.2857	73.3469	886.5000	3.1847	1.8337
35	2015	1	8.0429	67.0204	951.7010	3.2643	1.1980
78	2015	1	9.6429	73.2347	909.4459	2.9316	2.1847
28	2015	1	9.1571	66.8673	940.6878	3.2867	1.5224
39	2015	1	8.9857	71.1837	912.3847	2.1051	2.7071
24	2015	1	9.1286	66.8163	952.4367	2.3776	1.1633
63	2015	1	9.3571	70.2143	958.0112	3.7704	1.1918
62	2015	1	7.7143	78.7449	883.3673	2.1816	1.0439
1	2015	1	8.2429	73.7857	883.7684	2.9031	2.1959
31	2015	2	3.4429	77.3878	852.4286	2.3908	0.9633
79	2015	2	8.4714	67.0918	985.8867	1.4571	1.0490
51	2015	2	7.1000	69.4796	949.8327	1.7163	1.2878
14	2015	2	6.0714	71.8878	905.5786	2.0878	2.1102
67	2015	2	5.6714	73.4796	910.7959	1.3520	2.9918
42	2015	2	4.6571	74.3776	882.3541	1.5878	2.5357
50	2015	2	6.1571	71.9388	910.6500	1.4408	1.2500
43	2015	2	4.6571	74.3776	882.3541	1.5878	2.5357
85	2015	2	6.1143	77.8571	919.9163	1.3276	1.2929

25	2015	2	10.0286	77.0306	989.0714	2.6561	0.8122
69	2015	2	7.9143	67.8469	950.6092	1.2898	1.1673
57	2015	2	4.6000	69.9490	894.8724	1.3888	2.3010
9	2015	2	4.2571	76.9082	860.5704	1.7286	2.1500
72	2015	2	6.4571	73.1020	884.9918	2.0265	2.0796
26	2015	2	6.9857	80.1020	873.8071	2.7255	1.7694
7	2015	2	6.0000	78.4082	865.7000	2.6316	1.5184
83	2015	2	11.1286	75.6531	954.4010	3.2827	0.7520
76	2015	2	7.0143	69.3469	930.9449	1.8980	1.5204
36	2015	2	7.0571	68.1531	938.9265	1.7735	1.5439
81	2015	2	7.1000	69.4796	949.8327	1.7163	1.2878
15	2015	2	8.1000	82.6939	943.9082	1.0908	0.5602
32	2015	2	4.6571	74.3776	882.3541	1.5878	2.5357
73	2015	2	9.5571	69.0000	974.0867	0.9316	0.7092
71	2015	2	7.0571	68.1531	938.9265	1.7735	1.5439
41	2015	2	5.0571	76.6327	881.9500	1.7500	1.2276
10	2015	2	8.3571	73.4490	976.0214	2.1602	0.8857
23	2015	2	0.9429	78.5510	779.1541	3.7980	2.2418
27	2015	2	6.0000	78.4082	865.7000	2.6316	1.5184
60	2015	2	7.1000	69.4796	949.8327	1.7163	1.2878
53	2015	2	4.2571	76.9082	860.5704	1.7286	2.1500
66	2015	2	6.0714	71.8878	905.5786	2.0878	2.1102
59	2015	2	4.6000	69.9490	894.8724	1.3888	2.3010
61	2015	2	9.5571	69.0000	974.0867	0.9316	0.7092
84	2015	2	9.5571	69.0000	974.0867	0.9316	0.7092
38	2015	2	4.6000	69.9490	894.8724	1.3888	2.3010
87	2015	2	6.8857	72.6224	907.8745	1.9357	2.3449
34	2015	2	4.6000	69.9490	894.8724	1.3888	2.3010
29	2015	2	7.9143	67.8469	950.6092	1.2898	1.1673
5	2015	2	3.8857	81.9592	837.6520	2.7837	1.7010
8	2015	2	4.2571	76.9082	860.5704	1.7286	2.1500
12	2015	2	3.8857	81.9592	837.6520	2.7837	1.7010
13	2015	2	11.1286	75.6531	954.4010	3.2827	0.7520
18	2015	2	8.4286	69.8061	976.2378	1.7337	1.0204
33	2015	2	6.1571	71.9388	910.6500	1.4408	1.2500
56	2015	2	10.0286	77.0306	989.0714	2.6561	0.8122
77	2015	2	8.1000	82.6939	943.9082	1.0908	0.5602
54	2015	2	3.8857	81.9592	837.6520	2.7837	1.7010
21	2015	2	6.1571	71.9388	910.6500	1.4408	1.2500
68	2015	2	8.4714	67.0918	985.8867	1.4571	1.0490
74	2015	2	9.5571	69.0000	974.0867	0.9316	0.7092
88	2015	2	4.6571	74.3776	882.3541	1.5878	2.5357
16	2015	2	7.0143	69.3469	930.9449	1.8980	1.5204
30	2015	2	6.0714	71.8878	905.5786	2.0878	2.1102
6	2015	2	8.4714	67.0918	985.8867	1.4571	1.0490
49	2015	2	7.9143	67.8469	950.6092	1.2898	1.1673
22	2015	2	4.6571	74.3776	882.3541	1.5878	2.5357
45	2015	2	5.3429	78.9082	822.1235	3.6469	1.7439
58	2015	2	7.9143	67.8469	950.6092	1.2898	1.1673
37	2015	2	8.4714	67.0918	985.8867	1.4571	1.0490

17	2015	2	5.6714	73.4796	910.7959	1.3520	2.9918
55	2015	2	6.4571	73.1020	884.9918	2.0265	2.0796
46	2015	2	7.0143	69.3469	930.9449	1.8980	1.5204
86	2015	2	5.1000	76.0510	873.2082	0.0000	0.9990
2	2015	2	5.1000	76.0510	873.2082	0.0000	0.9990
4	2015	2	6.1571	71.9388	910.6500	1.4408	1.2500
47	2015	2	11.0857	72.5816	969.3214	2.2469	0.6663
82	2015	2	4.6571	74.3776	882.3541	1.5878	2.5357
19	2015	2	10.0286	73.5408	971.4592	2.2827	0.9551
20	2015	2	4.2571	76.9082	860.5704	1.7286	2.1500
80	2015	2	4.6571	74.3776	882.3541	1.5878	2.5357
3	2015	2	11.1286	75.6531	954.4010	3.2827	0.7520
52	2015	2	5.6714	73.4796	910.7959	1.3520	2.9918
70	2015	2	6.1143	77.8571	919.9163	1.3276	1.2929
64	2015	2	0.9429	78.5510	779.1541	3.7980	2.2418
48	2015	2	8.1000	82.6939	943.9082	1.0908	0.5602
65	2015	2	5.6714	73.4796	910.7959	1.3520	2.9918
44	2015	2	6.1143	77.8571	919.9163	1.3276	1.2929
75	2015	2	0.9429	78.5510	779.1541	3.7980	2.2418
40	2015	2	7.6143	71.7857	956.0878	2.4500	1.5531
11	2015	2	6.4571	73.1020	884.9918	2.0265	2.0796
35	2015	2	7.1000	69.4796	949.8327	1.7163	1.2878
78	2015	2	6.8857	72.6224	907.8745	1.9357	2.3449
28	2015	2	7.0571	68.1531	938.9265	1.7735	1.5439
39	2015	2	5.6714	73.4796	910.7959	1.3520	2.9918
24	2015	2	7.9143	67.8469	950.6092	1.2898	1.1673
63	2015	2	7.6143	71.7857	956.0878	2.4500	1.5531
62	2015	2	5.0571	76.6327	881.9500	1.7500	1.2276
1	2015	2	4.6571	74.3776	882.3541	1.5878	2.5357
31	2015	3	4.2857	82.0306	853.2224	1.2551	0.8990
79	2015	3	9.0429	71.8980	983.2010	1.4388	1.2031
51	2015	3	6.7714	77.3571	950.7786	1.0592	1.1694
14	2015	3	6.3714	80.4796	906.4051	1.5010	1.8408
67	2015	3	6.3286	82.9796	911.7561	0.5041	2.8663
42	2015	3	5.5571	82.9490	883.2337	0.4622	2.3347
50	2015	3	6.4286	74.4898	911.5276	1.1531	1.3041
43	2015	3	5.5571	82.9490	883.2337	0.4622	2.3347
85	2015	3	7.4143	79.4592	920.8255	1.1031	1.3520
25	2015	3	9.3286	84.0714	990.1714	1.3724	0.5684
69	2015	3	7.7571	76.5408	951.6153	0.4898	1.1163
57	2015	3	5.1571	77.8469	895.8459	0.7316	1.7306
9	2015	3	4.9714	83.5204	861.3510	0.6939	2.1194
72	2015	3	5.9714	79.6531	885.8500	0.9337	2.0357
26	2015	3	7.0143	85.7653	874.7469	1.2245	1.4898
7	2015	3	5.7000	84.5204	866.5939	1.2510	1.3745
83	2015	3	9.4857	81.6224	955.6949	1.8378	0.7388
76	2015	3	7.0286	74.7449	931.8684	1.3480	1.4918
36	2015	3	6.9571	76.2449	939.9224	0.8857	1.5367
81	2015	3	6.7714	77.3571	950.7786	1.0592	1.1694
15	2015	3	8.0429	80.2551	944.6235	1.1408	0.5724

32	2015	3	5.5571	82.9490	883.2337	0.4622	2.3347
73	2015	3	10.0429	68.4694	974.8133	0.7316	0.8541
71	2015	3	6.9571	76.2449	939.9224	0.8857	1.5367
41	2015	3	5.8000	81.6939	882.8561	0.7949	1.2153
10	2015	3	7.8143	78.8265	977.0071	1.5439	0.8755
23	2015	3	1.6571	84.4286	779.5490	3.2388	2.0520
27	2015	3	5.7000	84.5204	866.5939	1.2510	1.3745
60	2015	3	6.7714	77.3571	950.7786	1.0592	1.1694
53	2015	3	4.9714	83.5204	861.3510	0.6939	2.1194
66	2015	3	6.3714	80.4796	906.4051	1.5010	1.8408
59	2015	3	5.1571	77.8469	895.8459	0.7316	1.7306
61	2015	3	10.0429	68.4694	974.8133	0.7316	0.8541
84	2015	3	10.0429	68.4694	974.8133	0.7316	0.8541
38	2015	3	5.1571	77.8469	895.8459	0.7316	1.7306
87	2015	3	6.8429	79.3265	908.8735	0.5367	2.1316
34	2015	3	5.1571	77.8469	895.8459	0.7316	1.7306
29	2015	3	7.7571	76.5408	951.6153	0.4898	1.1163
5	2015	3	4.3143	88.9592	838.3786	1.2939	1.6173
8	2015	3	4.9714	83.5204	861.3510	0.6939	2.1194
12	2015	3	4.3143	88.9592	838.3786	1.2939	1.6173
13	2015	3	9.4857	81.6224	955.6949	1.8378	0.7388
18	2015	3	8.5571	74.4796	977.0959	1.2735	0.8776
33	2015	3	6.4286	74.4898	911.5276	1.1531	1.3041
56	2015	3	9.3286	84.0714	990.1714	1.3724	0.5684
77	2015	3	8.0429	80.2551	944.6235	1.1408	0.5724
54	2015	3	4.3143	88.9592	838.3786	1.2939	1.6173
21	2015	3	6.4286	74.4898	911.5276	1.1531	1.3041
68	2015	3	9.0429	71.8980	983.2010	1.4388	1.2031
74	2015	3	10.0429	68.4694	974.8133	0.7316	0.8541
88	2015	3	5.5571	82.9490	883.2337	0.4622	2.3347
16	2015	3	7.0286	74.7449	931.8684	1.3480	1.4918
30	2015	3	6.3714	80.4796	906.4051	1.5010	1.8408
6	2015	3	9.0429	71.8980	983.2010	1.4388	1.2031
49	2015	3	7.7571	76.5408	951.6153	0.4898	1.1163
22	2015	3	5.5571	82.9490	883.2337	0.4622	2.3347
45	2015	3	5.2571	84.6837	822.7571	3.0143	1.5327
58	2015	3	7.7571	76.5408	951.6153	0.4898	1.1163
37	2015	3	9.0429	71.8980	983.2010	1.4388	1.2031
17	2015	3	6.3286	82.9796	911.7561	0.5041	2.8663
55	2015	3	5.9714	79.6531	885.8500	0.9337	2.0357
46	2015	3	7.0286	74.7449	931.8684	1.3480	1.4918
86	2015	3	5.5714	80.2245	874.0602	0.3061	0.9153
2	2015	3	5.5714	80.2245	874.0602	0.3061	0.9153
4	2015	3	6.4286	74.4898	911.5276	1.1531	1.3041
47	2015	3	10.7286	78.6122	970.6500	1.3673	0.7041
82	2015	3	5.5571	82.9490	883.2337	0.4622	2.3347
19	2015	3	9.9000	77.9898	972.4918	1.7418	0.9622
20	2015	3	4.9714	83.5204	861.3510	0.6939	2.1194
80	2015	3	5.5571	82.9490	883.2337	0.4622	2.3347
3	2015	3	9.4857	81.6224	955.6949	1.8378	0.7388

52	2015	3	6.3286	82.9796	911.7561	0.5041	2.8663
70	2015	3	7.4143	79.4592	920.8255	1.1031	1.3520
64	2015	3	1.6571	84.4286	779.5490	3.2388	2.0520
48	2015	3	8.0429	80.2551	944.6235	1.1408	0.5724
65	2015	3	6.3286	82.9796	911.7561	0.5041	2.8663
44	2015	3	7.4143	79.4592	920.8255	1.1031	1.3520
75	2015	3	1.6571	84.4286	779.5490	3.2388	2.0520
40	2015	3	6.8286	80.6224	957.0276	1.4153	1.4622
11	2015	3	5.9714	79.6531	885.8500	0.9337	2.0357
35	2015	3	6.7714	77.3571	950.7786	1.0592	1.1694
78	2015	3	6.8429	79.3265	908.8735	0.5367	2.1316
28	2015	3	6.9571	76.2449	939.9224	0.8857	1.5367
39	2015	3	6.3286	82.9796	911.7561	0.5041	2.8663
24	2015	3	7.7571	76.5408	951.6153	0.4898	1.1163
63	2015	3	6.8286	80.6224	957.0276	1.4153	1.4622
62	2015	3	5.8000	81.6939	882.8561	0.7949	1.2153
1	2015	3	5.5571	82.9490	883.2337	0.4622	2.3347
31	2015	4	6.8714	84.9592	853.8571	1.4388	0.8092
79	2015	4	10.2000	71.7857	983.6806	2.6765	0.9633
51	2015	4	9.2286	80.9592	951.6663	1.7224	0.9398
14	2015	4	9.8571	81.0816	907.3827	2.3888	1.5735
67	2015	4	9.3286	84.5612	912.6663	1.0704	2.2582
42	2015	4	8.4429	84.3776	884.0194	1.1163	1.9878
50	2015	4	8.6857	75.1122	912.1980	1.6000	1.3490
43	2015	4	8.4429	84.3776	884.0194	1.1163	1.9878
85	2015	4	9.6429	79.5204	921.3622	1.8388	1.3878
25	2015	4	12.0000	85.7449	991.3276	1.7786	0.5408
69	2015	4	10.1857	79.4490	952.4704	1.0612	0.8622
57	2015	4	8.3000	80.4694	896.5684	1.4837	1.5561
9	2015	4	8.1000	83.8367	862.1388	1.3133	2.0010
72	2015	4	9.6000	81.8878	886.7633	1.7643	1.6214
26	2015	4	10.2286	87.3571	875.7071	1.4714	1.5694
7	2015	4	9.4857	85.7347	867.4980	1.5510	1.4194
83	2015	4	13.2714	85.1327	956.8898	2.0541	0.7694
76	2015	4	9.2571	75.0714	932.6010	2.1776	1.3633
36	2015	4	10.2714	79.7245	940.7990	1.5541	1.3939
81	2015	4	9.2286	80.9592	951.6663	1.7224	0.9398
15	2015	4	9.7571	77.6939	945.2204	1.3898	0.5265
32	2015	4	8.4429	84.3776	884.0194	1.1163	1.9878
73	2015	4	11.2571	67.0714	975.4949	1.6071	1.0449
71	2015	4	10.2714	79.7245	940.7990	1.5541	1.3939
41	2015	4	8.4000	85.0612	883.5643	1.7245	0.9418
10	2015	4	9.9714	80.8163	977.9276	2.4082	0.7694
23	2015	4	5.2000	85.4184	780.1663	3.5286	2.0459
27	2015	4	9.4857	85.7347	867.4980	1.5510	1.4194
60	2015	4	9.2286	80.9592	951.6663	1.7224	0.9398
53	2015	4	8.1000	83.8367	862.1388	1.3133	2.0010
66	2015	4	9.8571	81.0816	907.3827	2.3888	1.5735
59	2015	4	8.3000	80.4694	896.5684	1.4837	1.5561
61	2015	4	11.2571	67.0714	975.4949	1.6071	1.0449

84	2015	4	11.2571	67.0714	975.4949	1.6071	1.0449
38	2015	4	8.3000	80.4694	896.5684	1.4837	1.5561
87	2015	4	10.1857	81.7755	909.7408	1.3102	1.5959
34	2015	4	8.3000	80.4694	896.5684	1.4837	1.5561
29	2015	4	10.1857	79.4490	952.4704	1.0612	0.8622
5	2015	4	7.6571	89.1224	839.2041	1.4980	1.6286
8	2015	4	8.1000	83.8367	862.1388	1.3133	2.0010
12	2015	4	7.6571	89.1224	839.2041	1.4980	1.6286
13	2015	4	13.2714	85.1327	956.8898	2.0541	0.7694
18	2015	4	9.1143	73.1327	977.8010	2.0500	0.7918
33	2015	4	8.6857	75.1122	912.1980	1.6000	1.3490
56	2015	4	12.0000	85.7449	991.3276	1.7786	0.5408
77	2015	4	9.7571	77.6939	945.2204	1.3898	0.5265
54	2015	4	7.6571	89.1224	839.2041	1.4980	1.6286
21	2015	4	8.6857	75.1122	912.1980	1.6000	1.3490
68	2015	4	10.2000	71.7857	983.6806	2.6765	0.9633
74	2015	4	11.2571	67.0714	975.4949	1.6071	1.0449
88	2015	4	8.4429	84.3776	884.0194	1.1163	1.9878
16	2015	4	9.2571	75.0714	932.6010	2.1776	1.3633
30	2015	4	9.8571	81.0816	907.3827	2.3888	1.5735
6	2015	4	10.2000	71.7857	983.6806	2.6765	0.9633
49	2015	4	10.1857	79.4490	952.4704	1.0612	0.8622
22	2015	4	8.4429	84.3776	884.0194	1.1163	1.9878
45	2015	4	8.4857	84.4592	823.5449	3.4408	1.4745
58	2015	4	10.1857	79.4490	952.4704	1.0612	0.8622
37	2015	4	10.2000	71.7857	983.6806	2.6765	0.9633
17	2015	4	9.3286	84.5612	912.6663	1.0704	2.2582
55	2015	4	9.6000	81.8878	886.7633	1.7643	1.6214
46	2015	4	9.2571	75.0714	932.6010	2.1776	1.3633
86	2015	4	8.7000	81.9184	874.7898	1.1173	0.8714
2	2015	4	8.7000	81.9184	874.7898	1.1173	0.8714
4	2015	4	8.6857	75.1122	912.1980	1.6000	1.3490
47	2015	4	13.2714	81.3878	971.7929	1.6684	0.6173
82	2015	4	8.4429	84.3776	884.0194	1.1163	1.9878
19	2015	4	13.0429	77.4388	973.4990	2.7867	0.9684
20	2015	4	8.1000	83.8367	862.1388	1.3133	2.0010
80	2015	4	8.4429	84.3776	884.0194	1.1163	1.9878
3	2015	4	13.2714	85.1327	956.8898	2.0541	0.7694
52	2015	4	9.3286	84.5612	912.6663	1.0704	2.2582
70	2015	4	9.6429	79.5204	921.3622	1.8388	1.3878
64	2015	4	5.2000	85.4184	780.1663	3.5286	2.0459
48	2015	4	9.7571	77.6939	945.2204	1.3898	0.5265
65	2015	4	9.3286	84.5612	912.6663	1.0704	2.2582
44	2015	4	9.6429	79.5204	921.3622	1.8388	1.3878
75	2015	4	5.2000	85.4184	780.1663	3.5286	2.0459
40	2015	4	10.3143	84.0204	958.0561	2.1173	1.0673
11	2015	4	9.6000	81.8878	886.7633	1.7643	1.6214
35	2015	4	9.2286	80.9592	951.6663	1.7224	0.9398
78	2015	4	10.1857	81.7755	909.7408	1.3102	1.5959
28	2015	4	10.2714	79.7245	940.7990	1.5541	1.3939

39	2015	4	9.3286	84.5612	912.6663	1.0704	2.2582
24	2015	4	10.1857	79.4490	952.4704	1.0612	0.8622
63	2015	4	10.3143	84.0204	958.0561	2.1173	1.0673
62	2015	4	8.4000	85.0612	883.5643	1.7245	0.9418
1	2015	4	8.4429	84.3776	884.0194	1.1163	1.9878
31	2015	5	3.4429	85.5918	850.5510	1.8571	0.8520
79	2015	5	2.6857	74.2143	981.1531	1.8837	1.4745
51	2015	5	1.3286	84.4184	948.7571	1.2173	1.3510
14	2015	5	4.7286	83.8776	904.2224	1.7194	2.4878
67	2015	5	1.8714	88.7959	909.4510	0.9429	2.1143
42	2015	5	3.3571	85.7143	880.6122	1.1643	2.2531
50	2015	5	3.6000	77.8061	908.7408	0.9867	1.6061
43	2015	5	3.3571	85.7143	880.6122	1.1643	2.2531
85	2015	5	4.6000	80.1020	917.7214	1.4673	1.4531
25	2015	5	6.8000	83.0918	987.8908	1.3959	0.9061
69	2015	5	3.5429	79.5816	949.2265	0.9531	1.1592
57	2015	5	2.0143	81.9592	893.1133	1.2776	1.8990
9	2015	5	5.6857	82.0204	858.8398	1.6204	2.4255
72	2015	5	7.9429	79.6837	883.2694	2.8214	1.9796
26	2015	5	10.0000	83.7041	872.1153	3.7735	2.0480
7	2015	5	8.7286	81.6429	863.9847	3.9204	1.9112
83	2015	5	12.7143	78.3673	952.4480	4.0031	1.1133
76	2015	5	2.4286	78.9082	929.3612	1.4949	1.5500
36	2015	5	2.2714	82.1020	937.5735	1.1551	1.6000
81	2015	5	1.3286	84.4184	948.7571	1.2173	1.3510
15	2015	5	4.3429	81.8571	941.9796	0.7541	0.7051
32	2015	5	3.3571	85.7143	880.6122	1.1643	2.2531
73	2015	5	5.1857	68.3163	972.4439	1.3918	1.1306
71	2015	5	2.2714	82.1020	937.5735	1.1551	1.6000
41	2015	5	3.0143	87.8571	880.1194	1.9724	1.2092
10	2015	5	2.8286	83.9592	975.0724	1.5520	0.9714
23	2015	5	5.6857	78.1633	777.4602	5.6622	2.2276
27	2015	5	8.7286	81.6429	863.9847	3.9204	1.9112
60	2015	5	1.3286	84.4184	948.7571	1.2173	1.3510
53	2015	5	5.6857	82.0204	858.8398	1.6204	2.4255
66	2015	5	4.7286	83.8776	904.2224	1.7194	2.4878
59	2015	5	2.0143	81.9592	893.1133	1.2776	1.8990
61	2015	5	5.1857	68.3163	972.4439	1.3918	1.1306
84	2015	5	5.1857	68.3163	972.4439	1.3918	1.1306
38	2015	5	2.0143	81.9592	893.1133	1.2776	1.8990
87	2015	5	7.5286	79.7755	906.0357	2.0837	1.8612
34	2015	5	2.0143	81.9592	893.1133	1.2776	1.8990
29	2015	5	3.5429	79.5816	949.2265	0.9531	1.1592
5	2015	5	6.9429	83.7857	835.9786	3.9959	1.7990
8	2015	5	5.6857	82.0204	858.8398	1.6204	2.4255
12	2015	5	6.9429	83.7857	835.9786	3.9959	1.7990
13	2015	5	12.7143	78.3673	952.4480	4.0031	1.1133
18	2015	5	3.1143	77.6327	975.2714	1.4694	0.9633
33	2015	5	3.6000	77.8061	908.7408	0.9867	1.6061
56	2015	5	6.8000	83.0918	987.8908	1.3959	0.9061

77	2015	5	4.3429	81.8571	941.9796	0.7541	0.7051
54	2015	5	6.9429	83.7857	835.9786	3.9959	1.7990
21	2015	5	3.6000	77.8061	908.7408	0.9867	1.6061
68	2015	5	2.6857	74.2143	981.1531	1.8837	1.4745
74	2015	5	5.1857	68.3163	972.4439	1.3918	1.1306
88	2015	5	3.3571	85.7143	880.6122	1.1643	2.2531
16	2015	5	2.4286	78.9082	929.3612	1.4949	1.5500
30	2015	5	4.7286	83.8776	904.2224	1.7194	2.4878
6	2015	5	2.6857	74.2143	981.1531	1.8837	1.4745
49	2015	5	3.5429	79.5816	949.2265	0.9531	1.1592
22	2015	5	3.3571	85.7143	880.6122	1.1643	2.2531
45	2015	5	9.0143	73.7143	820.4796	5.6765	1.8490
58	2015	5	3.5429	79.5816	949.2265	0.9531	1.1592
37	2015	5	2.6857	74.2143	981.1531	1.8837	1.4745
17	2015	5	1.8714	88.7959	909.4510	0.9429	2.1143
55	2015	5	7.9429	79.6837	883.2694	2.8214	1.9796
46	2015	5	2.4286	78.9082	929.3612	1.4949	1.5500
86	2015	5	4.8714	82.4694	871.2735	1.7306	0.9673
2	2015	5	4.8714	82.4694	871.2735	1.7306	0.9673
4	2015	5	3.6000	77.8061	908.7408	0.9867	1.6061
47	2015	5	12.6571	77.3878	967.4173	1.8459	0.7296
82	2015	5	3.3571	85.7143	880.6122	1.1643	2.2531
19	2015	5	8.8571	78.1429	969.8918	1.8837	1.1612
20	2015	5	5.6857	82.0204	858.8398	1.6204	2.4255
80	2015	5	3.3571	85.7143	880.6122	1.1643	2.2531
3	2015	5	12.7143	78.3673	952.4480	4.0031	1.1133
52	2015	5	1.8714	88.7959	909.4510	0.9429	2.1143
70	2015	5	4.6000	80.1020	917.7214	1.4673	1.4531
64	2015	5	5.6857	78.1633	777.4602	5.6622	2.2276
48	2015	5	4.3429	81.8571	941.9796	0.7541	0.7051
65	2015	5	1.8714	88.7959	909.4510	0.9429	2.1143
44	2015	5	4.6000	80.1020	917.7214	1.4673	1.4531
75	2015	5	5.6857	78.1633	777.4602	5.6622	2.2276
40	2015	5	2.2571	85.6837	955.1776	1.6714	1.3827
11	2015	5	7.9429	79.6837	883.2694	2.8214	1.9796
35	2015	5	1.3286	84.4184	948.7571	1.2173	1.3510
78	2015	5	7.5286	79.7755	906.0357	2.0837	1.8612
28	2015	5	2.2714	82.1020	937.5735	1.1551	1.6000
39	2015	5	1.8714	88.7959	909.4510	0.9429	2.1143
24	2015	5	3.5429	79.5816	949.2265	0.9531	1.1592
63	2015	5	2.2571	85.6837	955.1776	1.6714	1.3827
62	2015	5	3.0143	87.8571	880.1194	1.9724	1.2092
1	2015	5	3.3571	85.7143	880.6122	1.1643	2.2531
31	2015	6	2.4286	87.3980	850.9980	0.7745	0.9551
79	2015	6	6.0571	77.9898	983.6061	0.8592	1.8490
51	2015	6	4.2000	86.6939	950.5898	0.4929	1.5184
14	2015	6	4.4857	87.7959	904.9622	0.5786	2.5061
67	2015	6	3.9571	92.6735	910.5235	0.3898	2.4531
42	2015	6	3.4429	89.3265	881.2500	0.4520	2.4112
50	2015	6	4.1000	81.0306	909.8939	0.3143	1.6061

43	2015	6	3.4429	89.3265	881.2500	0.4520	2.4112
85	2015	6	4.9714	83.3878	918.9480	0.6122	1.3929
25	2015	6	7.7571	81.7143	989.8673	0.3582	1.1592
69	2015	6	5.4429	80.6429	951.0031	0.2857	1.4286
57	2015	6	2.8143	86.2347	894.0531	0.4520	1.6755
9	2015	6	3.0143	85.3673	859.1765	1.0510	2.7020
72	2015	6	5.1286	81.3571	883.6867	2.0163	2.5173
26	2015	6	5.4429	86.1429	872.2439	3.4143	2.1122
7	2015	6	5.0143	83.9898	864.1418	3.6633	2.1449
83	2015	6	9.8857	76.9388	953.2398	2.9041	1.1837
76	2015	6	4.1143	86.9082	930.9316	0.3908	1.6439
36	2015	6	4.8000	84.5510	939.1571	0.3949	1.7990
81	2015	6	4.2000	86.6939	950.5898	0.4929	1.5184
15	2015	6	5.3857	87.0510	943.6867	0.2276	0.8520
32	2015	6	3.4429	89.3265	881.2500	0.4520	2.4112
73	2015	6	6.8286	73.8061	974.6510	0.5133	1.0469
71	2015	6	4.8000	84.5510	939.1571	0.3949	1.7990
41	2015	6	3.0571	92.1122	880.8816	0.8500	1.5408
10	2015	6	5.3571	86.5204	977.2633	0.8163	1.1622
23	2015	6	0.5714	77.9796	777.2265	5.7520	2.2418
27	2015	6	5.0143	83.9898	864.1418	3.6633	2.1449
60	2015	6	4.2000	86.6939	950.5898	0.4929	1.5184
53	2015	6	3.0143	85.3673	859.1765	1.0510	2.7020
66	2015	6	4.4857	87.7959	904.9622	0.5786	2.5061
59	2015	6	2.8143	86.2347	894.0531	0.4520	1.6755
61	2015	6	6.8286	73.8061	974.6510	0.5133	1.0469
84	2015	6	6.8286	73.8061	974.6510	0.5133	1.0469
38	2015	6	2.8143	86.2347	894.0531	0.4520	1.6755
87	2015	6	6.1000	79.8673	906.7878	1.4592	2.4459
34	2015	6	2.8143	86.2347	894.0531	0.4520	1.6755
29	2015	6	5.4429	80.6429	951.0031	0.2857	1.4286
5	2015	6	2.9286	86.0408	836.0143	3.8306	1.9367
8	2015	6	3.0143	85.3673	859.1765	1.0510	2.7020
12	2015	6	2.9286	86.0408	836.0143	3.8306	1.9367
13	2015	6	9.8857	76.9388	953.2398	2.9041	1.1837
18	2015	6	5.9571	81.5714	977.6184	0.7520	1.0847
33	2015	6	4.1000	81.0306	909.8939	0.3143	1.6061
56	2015	6	7.7571	81.7143	989.8673	0.3582	1.1592
77	2015	6	5.3857	87.0510	943.6867	0.2276	0.8520
54	2015	6	2.9286	86.0408	836.0143	3.8306	1.9367
21	2015	6	4.1000	81.0306	909.8939	0.3143	1.6061
68	2015	6	6.0571	77.9898	983.6061	0.8592	1.8490
74	2015	6	6.8286	73.8061	974.6510	0.5133	1.0469
88	2015	6	3.4429	89.3265	881.2500	0.4520	2.4112
16	2015	6	4.1143	86.9082	930.9316	0.3908	1.6439
30	2015	6	4.4857	87.7959	904.9622	0.5786	2.5061
6	2015	6	6.0571	77.9898	983.6061	0.8592	1.8490
49	2015	6	5.4429	80.6429	951.0031	0.2857	1.4286
22	2015	6	3.4429	89.3265	881.2500	0.4520	2.4112
45	2015	6	3.9143	74.5306	820.4071	5.5367	1.9480

58	2015	6	5.4429	80.6429	951.0031	0.2857	1.4286
37	2015	6	6.0571	77.9898	983.6061	0.8592	1.8490
17	2015	6	3.9571	92.6735	910.5235	0.3898	2.4531
55	2015	6	5.1286	81.3571	883.6867	2.0163	2.5173
46	2015	6	4.1143	86.9082	930.9316	0.3908	1.6439
86	2015	6	3.4857	85.8163	871.8133	0.9327	0.7980
2	2015	6	3.4857	85.8163	871.8133	0.9327	0.7980
4	2015	6	4.1000	81.0306	909.8939	0.3143	1.6061
47	2015	6	9.9714	73.5714	968.6102	1.2306	0.8439
82	2015	6	3.4429	89.3265	881.2500	0.4520	2.4112
19	2015	6	8.6714	78.5714	971.4224	0.4827	1.3347
20	2015	6	3.0143	85.3673	859.1765	1.0510	2.7020
80	2015	6	3.4429	89.3265	881.2500	0.4520	2.4112
3	2015	6	9.8857	76.9388	953.2398	2.9041	1.1837
52	2015	6	3.9571	92.6735	910.5235	0.3898	2.4531
70	2015	6	4.9714	83.3878	918.9480	0.6122	1.3929
64	2015	6	0.5714	77.9796	777.2265	5.7520	2.2418
48	2015	6	5.3857	87.0510	943.6867	0.2276	0.8520
65	2015	6	3.9571	92.6735	910.5235	0.3898	2.4531
44	2015	6	4.9714	83.3878	918.9480	0.6122	1.3929
75	2015	6	0.5714	77.9796	777.2265	5.7520	2.2418
40	2015	6	4.3000	88.9490	956.9071	0.7286	1.5939
11	2015	6	5.1286	81.3571	883.6867	2.0163	2.5173
35	2015	6	4.2000	86.6939	950.5898	0.4929	1.5184
78	2015	6	6.1000	79.8673	906.7878	1.4592	2.4459
28	2015	6	4.8000	84.5510	939.1571	0.3949	1.7990
39	2015	6	3.9571	92.6735	910.5235	0.3898	2.4531
24	2015	6	5.4429	80.6429	951.0031	0.2857	1.4286
63	2015	6	4.3000	88.9490	956.9071	0.7286	1.5939
62	2015	6	3.0571	92.1122	880.8816	0.8500	1.5408
1	2015	6	3.4429	89.3265	881.2500	0.4520	2.4112
31	2015	7	6.6571	86.5000	852.7735	0.8469	1.0010
79	2015	7	10.6857	69.1327	984.6786	1.8122	1.1847
51	2015	7	9.4571	75.6735	952.0082	1.2694	1.1684
14	2015	7	8.8714	79.1837	906.8480	1.2327	1.7939
67	2015	7	9.4000	80.7041	912.3184	0.6949	2.7592
42	2015	7	8.6143	83.1224	883.1786	0.8214	2.2408
50	2015	7	8.9286	73.3571	911.8602	0.7541	1.5061
43	2015	7	8.6143	83.1224	883.1786	0.8214	2.2408
85	2015	7	10.2714	76.6224	920.9551	0.9898	1.3010
25	2015	7	11.6000	74.9184	991.7908	0.8929	0.9296
69	2015	7	9.9857	71.6020	952.7265	0.8429	1.2755
57	2015	7	8.7857	77.9796	895.9459	0.8806	1.6714
9	2015	7	7.3714	86.6020	861.0582	0.9367	2.4296
72	2015	7	8.8714	80.4490	885.7633	1.2755	2.1694
26	2015	7	8.8286	91.9898	874.3663	1.1204	1.6959
7	2015	7	8.0286	88.8980	866.2010	1.4776	1.9571
83	2015	7	12.4143	77.9898	956.0153	1.0051	0.8745
76	2015	7	8.7429	78.2347	932.6918	0.8612	1.4653
36	2015	7	10.1857	73.6020	940.8969	1.2724	1.5745

81	2015	7	9.4571	75.6735	952.0082	1.2694	1.1684
15	2015	7	9.3000	80.4082	945.5378	0.7949	0.6908
32	2015	7	8.6143	83.1224	883.1786	0.8214	2.2408
73	2015	7	10.9286	68.0612	976.2735	0.9327	0.8735
71	2015	7	10.1857	73.6020	940.8969	1.2724	1.5745
41	2015	7	8.1857	88.0612	882.8041	0.9398	1.3153
10	2015	7	10.7000	76.7755	978.6265	1.7633	0.9051
23	2015	7	4.2714	86.6429	778.5265	3.2918	2.1949
27	2015	7	8.0286	88.8980	866.2010	1.4776	1.9571
60	2015	7	9.4571	75.6735	952.0082	1.2694	1.1684
53	2015	7	7.3714	86.6020	861.0582	0.9367	2.4296
66	2015	7	8.8714	79.1837	906.8480	1.2327	1.7939
59	2015	7	8.7857	77.9796	895.9459	0.8806	1.6714
61	2015	7	10.9286	68.0612	976.2735	0.9327	0.8735
84	2015	7	10.9286	68.0612	976.2735	0.9327	0.8735
38	2015	7	8.7857	77.9796	895.9459	0.8806	1.6714
87	2015	7	9.9143	75.4388	908.9765	1.4245	2.1582
34	2015	7	8.7857	77.9796	895.9459	0.8806	1.6714
29	2015	7	9.9857	71.6020	952.7265	0.8429	1.2755
5	2015	7	7.0429	91.9898	837.8306	1.6510	2.0204
8	2015	7	7.3714	86.6020	861.0582	0.9367	2.4296
12	2015	7	7.0429	91.9898	837.8306	1.6510	2.0204
13	2015	7	12.4143	77.9898	956.0153	1.0051	0.8745
18	2015	7	9.8429	72.4796	978.7286	1.7704	0.8684
33	2015	7	8.9286	73.3571	911.8602	0.7541	1.5061
56	2015	7	11.6000	74.9184	991.7908	0.8929	0.9296
77	2015	7	9.3000	80.4082	945.5378	0.7949	0.6908
54	2015	7	7.0429	91.9898	837.8306	1.6510	2.0204
21	2015	7	8.9286	73.3571	911.8602	0.7541	1.5061
68	2015	7	10.6857	69.1327	984.6786	1.8122	1.1847
74	2015	7	10.9286	68.0612	976.2735	0.9327	0.8735
88	2015	7	8.6143	83.1224	883.1786	0.8214	2.2408
16	2015	7	8.7429	78.2347	932.6918	0.8612	1.4653
30	2015	7	8.8714	79.1837	906.8480	1.2327	1.7939
6	2015	7	10.6857	69.1327	984.6786	1.8122	1.1847
49	2015	7	9.9857	71.6020	952.7265	0.8429	1.2755
22	2015	7	8.6143	83.1224	883.1786	0.8214	2.2408
45	2015	7	7.8571	85.8878	822.0765	2.8276	1.6929
58	2015	7	9.9857	71.6020	952.7265	0.8429	1.2755
37	2015	7	10.6857	69.1327	984.6786	1.8122	1.1847
17	2015	7	9.4000	80.7041	912.3184	0.6949	2.7592
55	2015	7	8.8714	80.4490	885.7633	1.2755	2.1694
46	2015	7	8.7429	78.2347	932.6918	0.8612	1.4653
86	2015	7	7.7714	86.5816	873.8133	0.9408	0.6980
2	2015	7	7.7714	86.5816	873.8133	0.9408	0.6980
4	2015	7	8.9286	73.3571	911.8602	0.7541	1.5061
47	2015	7	12.7857	70.3163	971.3847	0.7582	0.7265
82	2015	7	8.6143	83.1224	883.1786	0.8214	2.2408
19	2015	7	11.5429	70.4184	973.6510	1.0714	1.2184
20	2015	7	7.3714	86.6020	861.0582	0.9367	2.4296

80	2015	7	8.6143	83.1224	883.1786	0.8214	2.2408
3	2015	7	12.4143	77.9898	956.0153	1.0051	0.8745
52	2015	7	9.4000	80.7041	912.3184	0.6949	2.7592
70	2015	7	10.2714	76.6224	920.9551	0.9898	1.3010
64	2015	7	4.2714	86.6429	778.5265	3.2918	2.1949
48	2015	7	9.3000	80.4082	945.5378	0.7949	0.6908
65	2015	7	9.4000	80.7041	912.3184	0.6949	2.7592
44	2015	7	10.2714	76.6224	920.9551	0.9898	1.3010
75	2015	7	4.2714	86.6429	778.5265	3.2918	2.1949
40	2015	7	10.1714	78.6837	958.4347	1.3684	1.3204
11	2015	7	8.8714	80.4490	885.7633	1.2755	2.1694
35	2015	7	9.4571	75.6735	952.0082	1.2694	1.1684
78	2015	7	9.9143	75.4388	908.9765	1.4245	2.1582
28	2015	7	10.1857	73.6020	940.8969	1.2724	1.5745
39	2015	7	9.4000	80.7041	912.3184	0.6949	2.7592
24	2015	7	9.9857	71.6020	952.7265	0.8429	1.2755
63	2015	7	10.1714	78.6837	958.4347	1.3684	1.3204
62	2015	7	8.1857	88.0612	882.8041	0.9398	1.3153
1	2015	7	8.6143	83.1224	883.1786	0.8214	2.2408
31	2015	8	11.3714	79.8061	849.6041	3.0714	1.0153
79	2015	8	10.6714	70.3673	978.5602	2.4143	1.1327
51	2015	8	9.8000	73.8673	946.7071	2.0122	1.1959
14	2015	8	14.2143	77.4490	903.1663	2.2867	2.1888
67	2015	8	13.2714	76.3265	908.0480	1.5408	3.1316
42	2015	8	13.1429	76.2755	879.5867	2.9184	2.4224
50	2015	8	12.2571	69.8265	907.3827	1.9439	1.6286
43	2015	8	13.1429	76.2755	879.5867	2.9184	2.4224
85	2015	8	14.0000	70.1122	916.1643	2.7918	1.4102
25	2015	8	17.0286	74.9286	985.8827	2.3327	0.9847
69	2015	8	13.0857	70.3980	947.2245	2.2235	1.3837
57	2015	8	12.3429	68.0102	891.7653	2.9612	2.5500
9	2015	8	13.4143	78.9592	858.0337	3.4286	2.5286
72	2015	8	15.6143	75.6429	882.4449	3.5204	1.8888
26	2015	8	15.9143	83.4796	871.5367	3.3510	1.8796
7	2015	8	14.8714	81.0102	863.3969	3.9490	2.1133
83	2015	8	18.5857	76.6020	951.5714	3.6092	0.7969
76	2015	8	11.2714	74.7347	927.7041	1.7347	1.5827
36	2015	8	12.4000	70.8673	935.8143	2.8418	1.4500
81	2015	8	9.8000	73.8673	946.7071	2.0122	1.1959
15	2015	8	12.4714	78.2551	940.3347	1.3633	0.8163
32	2015	8	13.1429	76.2755	879.5867	2.9184	2.4224
73	2015	8	12.7000	68.5000	970.4102	1.4347	0.8071
71	2015	8	12.4000	70.8673	935.8143	2.8418	1.4500
41	2015	8	12.7143	78.9388	878.9520	3.2398	1.1459
10	2015	8	11.3857	75.7959	972.7531	2.1612	0.8000
23	2015	8	9.4714	77.3061	776.8867	4.9429	2.2082
27	2015	8	14.8714	81.0102	863.3969	3.9490	2.1133
60	2015	8	9.8000	73.8673	946.7071	2.0122	1.1959
53	2015	8	13.4143	78.9592	858.0337	3.4286	2.5286
66	2015	8	14.2143	77.4490	903.1663	2.2867	2.1888

59	2015	8	12.3429	68.0102	891.7653	2.9612	2.5500
61	2015	8	12.7000	68.5000	970.4102	1.4347	0.8071
84	2015	8	12.7000	68.5000	970.4102	1.4347	0.8071
38	2015	8	12.3429	68.0102	891.7653	2.9612	2.5500
87	2015	8	15.9286	72.0408	905.0071	3.5592	1.8204
34	2015	8	12.3429	68.0102	891.7653	2.9612	2.5500
29	2015	8	13.0857	70.3980	947.2245	2.2235	1.3837
5	2015	8	13.1286	81.1327	835.3939	4.2051	2.2490
8	2015	8	13.4143	78.9592	858.0337	3.4286	2.5286
12	2015	8	13.1286	81.1327	835.3939	4.2051	2.2490
13	2015	8	18.5857	76.6020	951.5714	3.6092	0.7969
18	2015	8	10.8714	75.2653	972.8051	2.1388	0.9102
33	2015	8	12.2571	69.8265	907.3827	1.9439	1.6286
56	2015	8	17.0286	74.9286	985.8827	2.3327	0.9847
77	2015	8	12.4714	78.2551	940.3347	1.3633	0.8163
54	2015	8	13.1286	81.1327	835.3939	4.2051	2.2490
21	2015	8	12.2571	69.8265	907.3827	1.9439	1.6286
68	2015	8	10.6714	70.3673	978.5602	2.4143	1.1327
74	2015	8	12.7000	68.5000	970.4102	1.4347	0.8071
88	2015	8	13.1429	76.2755	879.5867	2.9184	2.4224
16	2015	8	11.2714	74.7347	927.7041	1.7347	1.5827
30	2015	8	14.2143	77.4490	903.1663	2.2867	2.1888
6	2015	8	10.6714	70.3673	978.5602	2.4143	1.1327
49	2015	8	13.0857	70.3980	947.2245	2.2235	1.3837
22	2015	8	13.1429	76.2755	879.5867	2.9184	2.4224
45	2015	8	13.1000	75.0102	820.0194	5.2612	1.9653
58	2015	8	13.0857	70.3980	947.2245	2.2235	1.3837
37	2015	8	10.6714	70.3673	978.5602	2.4143	1.1327
17	2015	8	13.2714	76.3265	908.0480	1.5408	3.1316
55	2015	8	15.6143	75.6429	882.4449	3.5204	1.8888
46	2015	8	11.2714	74.7347	927.7041	1.7347	1.5827
86	2015	8	13.4714	78.6224	870.3296	3.5684	0.9092
2	2015	8	13.4714	78.6224	870.3296	3.5684	0.9092
4	2015	8	12.2571	69.8265	907.3827	1.9439	1.6286
47	2015	8	19.2857	72.7347	966.3398	2.3286	0.6878
82	2015	8	13.1429	76.2755	879.5867	2.9184	2.4224
19	2015	8	17.6714	73.8367	968.6663	1.8908	1.0796
20	2015	8	13.4143	78.9592	858.0337	3.4286	2.5286
80	2015	8	13.1429	76.2755	879.5867	2.9184	2.4224
3	2015	8	18.5857	76.6020	951.5714	3.6092	0.7969
52	2015	8	13.2714	76.3265	908.0480	1.5408	3.1316
70	2015	8	14.0000	70.1122	916.1643	2.7918	1.4102
64	2015	8	9.4714	77.3061	776.8867	4.9429	2.2082
48	2015	8	12.4714	78.2551	940.3347	1.3633	0.8163
65	2015	8	13.2714	76.3265	908.0480	1.5408	3.1316
44	2015	8	14.0000	70.1122	916.1643	2.7918	1.4102
75	2015	8	9.4714	77.3061	776.8867	4.9429	2.2082
40	2015	8	12.8714	75.9592	953.2582	2.3724	1.4694
11	2015	8	15.6143	75.6429	882.4449	3.5204	1.8888
35	2015	8	9.8000	73.8673	946.7071	2.0122	1.1959

78	2015	8	15.9286	72.0408	905.0071	3.5592	1.8204
28	2015	8	12.4000	70.8673	935.8143	2.8418	1.4500
39	2015	8	13.2714	76.3265	908.0480	1.5408	3.1316
24	2015	8	13.0857	70.3980	947.2245	2.2235	1.3837
63	2015	8	12.8714	75.9592	953.2582	2.3724	1.4694
62	2015	8	12.7143	78.9388	878.9520	3.2398	1.1459
1	2015	8	13.1429	76.2755	879.5867	2.9184	2.4224
31	2015	9	7.7429	78.9898	846.4531	4.1000	1.0969
79	2015	9	7.8714	81.3673	975.3153	1.1102	1.6918
51	2015	9	6.2571	86.4490	943.4173	0.9949	1.5102
14	2015	9	9.1714	86.1939	899.5214	1.6633	2.6776
67	2015	9	6.5857	88.1531	904.5429	1.1622	3.3204
42	2015	9	7.9000	82.3265	876.1480	3.4245	2.8071
50	2015	9	8.0429	77.7449	903.6959	1.4102	1.9214
43	2015	9	7.9000	82.3265	876.1480	3.4245	2.8071
85	2015	9	9.1143	76.5714	912.3000	2.6255	1.5929
25	2015	9	11.6143	79.1735	981.6316	2.1898	1.3469
69	2015	9	8.1000	79.7551	943.5490	1.8214	1.6418
57	2015	9	6.9571	75.3980	888.2133	3.0133	2.8694
9	2015	9	9.6857	77.0612	854.7092	5.1888	3.0888
72	2015	9	12.5286	74.2347	878.6765	5.6235	2.4816
26	2015	9	17.6714	63.3571	867.8092	7.5163	2.5949
7	2015	9	15.4571	66.6531	859.7959	7.3694	2.6704
83	2015	9	18.2571	73.8469	946.3918	6.1847	1.0449
76	2015	9	6.7857	84.9184	924.2184	0.9531	1.8684
36	2015	9	7.1286	82.4592	932.2347	2.2847	1.7082
81	2015	9	6.2571	86.4490	943.4173	0.9949	1.5102
15	2015	9	8.6000	85.2347	936.6112	0.6551	1.1541
32	2015	9	7.9000	82.3265	876.1480	3.4245	2.8071
73	2015	9	9.2286	77.8163	966.8418	0.6816	0.8857
71	2015	9	7.1286	82.4592	932.2347	2.2847	1.7082
41	2015	9	8.7571	80.0306	875.4214	3.8806	1.4796
10	2015	9	7.6000	87.6429	969.3622	0.8898	0.9235
23	2015	9	9.7286	62.5306	774.8480	8.0204	2.4000
27	2015	9	15.4571	66.6531	859.7959	7.3694	2.6704
60	2015	9	6.2571	86.4490	943.4173	0.9949	1.5102
53	2015	9	9.6857	77.0612	854.7092	5.1888	3.0888
66	2015	9	9.1714	86.1939	899.5214	1.6633	2.6776
59	2015	9	6.9571	75.3980	888.2133	3.0133	2.8694
61	2015	9	9.2286	77.8163	966.8418	0.6816	0.8857
84	2015	9	9.2286	77.8163	966.8418	0.6816	0.8857
38	2015	9	6.9571	75.3980	888.2133	3.0133	2.8694
87	2015	9	11.2714	76.6020	901.1071	4.3541	2.4184
34	2015	9	6.9571	75.3980	888.2133	3.0133	2.8694
29	2015	9	8.1000	79.7551	943.5490	1.8214	1.6418
5	2015	9	12.9429	64.8265	832.3082	7.8806	2.6429
8	2015	9	9.6857	77.0612	854.7092	5.1888	3.0888
12	2015	9	12.9429	64.8265	832.3082	7.8806	2.6429
13	2015	9	18.2571	73.8469	946.3918	6.1847	1.0449
18	2015	9	7.8143	86.0408	969.5673	0.7653	1.0622

33	2015	9	8.0429	77.7449	903.6959	1.4102	1.9214
56	2015	9	11.6143	79.1735	981.6316	2.1898	1.3469
77	2015	9	8.6000	85.2347	936.6112	0.6551	1.1541
54	2015	9	12.9429	64.8265	832.3082	7.8806	2.6429
21	2015	9	8.0429	77.7449	903.6959	1.4102	1.9214
68	2015	9	7.8714	81.3673	975.3153	1.1102	1.6918
74	2015	9	9.2286	77.8163	966.8418	0.6816	0.8857
88	2015	9	7.9000	82.3265	876.1480	3.4245	2.8071
16	2015	9	6.7857	84.9184	924.2184	0.9531	1.8684
30	2015	9	9.1714	86.1939	899.5214	1.6633	2.6776
6	2015	9	7.8714	81.3673	975.3153	1.1102	1.6918
49	2015	9	8.1000	79.7551	943.5490	1.8214	1.6418
22	2015	9	7.9000	82.3265	876.1480	3.4245	2.8071
45	2015	9	15.5000	52.0612	817.3531	8.9980	2.7969
58	2015	9	8.1000	79.7551	943.5490	1.8214	1.6418
37	2015	9	7.8714	81.3673	975.3153	1.1102	1.6918
17	2015	9	6.5857	88.1531	904.5429	1.1622	3.3204
55	2015	9	12.5286	74.2347	878.6765	5.6235	2.4816
46	2015	9	6.7857	84.9184	924.2184	0.9531	1.8684
86	2015	9	9.8143	75.2347	866.8153	5.0714	1.0724
2	2015	9	9.8143	75.2347	866.8153	5.0714	1.0724
4	2015	9	8.0429	77.7449	903.6959	1.4102	1.9214
47	2015	9	15.7714	75.5612	961.3327	3.4102	0.8367
82	2015	9	7.9000	82.3265	876.1480	3.4245	2.8071
19	2015	9	12.7429	81.7755	964.2786	1.1367	1.4531
20	2015	9	9.6857	77.0612	854.7092	5.1888	3.0888
80	2015	9	7.9000	82.3265	876.1480	3.4245	2.8071
3	2015	9	18.2571	73.8469	946.3918	6.1847	1.0449
52	2015	9	6.5857	88.1531	904.5429	1.1622	3.3204
70	2015	9	9.1143	76.5714	912.3000	2.6255	1.5929
64	2015	9	9.7286	62.5306	774.8480	8.0204	2.4000
48	2015	9	8.6000	85.2347	936.6112	0.6551	1.1541
65	2015	9	6.5857	88.1531	904.5429	1.1622	3.3204
44	2015	9	9.1143	76.5714	912.3000	2.6255	1.5929
75	2015	9	9.7286	62.5306	774.8480	8.0204	2.4000
40	2015	9	6.9000	87.8571	949.7082	1.8102	1.8949
11	2015	9	12.5286	74.2347	878.6765	5.6235	2.4816
35	2015	9	6.2571	86.4490	943.4173	0.9949	1.5102
78	2015	9	11.2714	76.6020	901.1071	4.3541	2.4184
28	2015	9	7.1286	82.4592	932.2347	2.2847	1.7082
39	2015	9	6.5857	88.1531	904.5429	1.1622	3.3204
24	2015	9	8.1000	79.7551	943.5490	1.8214	1.6418
63	2015	9	6.9000	87.8571	949.7082	1.8102	1.8949
62	2015	9	8.7571	80.0306	875.4214	3.8806	1.4796
1	2015	9	7.9000	82.3265	876.1480	3.4245	2.8071
31	2015	10	4.9143	85.0408	846.5337	2.0551	1.1082
79	2015	10	8.0143	78.6531	976.8092	0.1388	1.8469
51	2015	10	5.6286	89.1327	944.5316	0.1255	1.6092
14	2015	10	5.2429	88.1735	899.4765	0.7490	2.4531
67	2015	10	4.8571	93.1327	905.0582	0.2622	3.0163

42	2015	10	4.3714	89.1735	876.3122	1.6694	2.8071
50	2015	10	6.6857	79.2347	904.3918	0.2204	2.0051
43	2015	10	4.3714	89.1735	876.3122	1.6694	2.8071
85	2015	10	7.3571	80.5816	913.2306	0.8602	1.5449
25	2015	10	8.6143	79.5000	982.8837	0.9490	1.3898
69	2015	10	7.2143	80.1327	944.8296	0.5439	1.5663
57	2015	10	4.4714	84.5408	888.8276	1.0418	2.3337
9	2015	10	5.0143	83.2449	854.4592	2.9878	3.1571
72	2015	10	6.7857	78.1633	878.4388	3.5398	2.9061
26	2015	10	11.0000	59.9490	866.9806	7.3776	2.9878
7	2015	10	9.3714	66.6633	859.1245	5.5908	2.8224
83	2015	10	11.8000	70.5102	946.2224	4.3378	1.3367
76	2015	10	6.5429	85.3571	925.1969	0.1306	1.9000
36	2015	10	5.9286	85.2245	933.2337	0.7214	1.9194
81	2015	10	5.6286	89.1327	944.5316	0.1255	1.6092
15	2015	10	8.8857	81.9490	937.6316	0.3847	1.2020
32	2015	10	4.3714	89.1735	876.3122	1.6694	2.8071
73	2015	10	9.1143	74.6429	968.1908	0.1194	1.0235
71	2015	10	5.9286	85.2245	933.2337	0.7214	1.9194
41	2015	10	4.8143	88.4184	875.8622	1.6827	1.6051
10	2015	10	6.7143	89.9490	970.7184	0.1051	0.9418
23	2015	10	8.4286	64.1122	774.0031	8.1684	2.6969
27	2015	10	9.3714	66.6633	859.1245	5.5908	2.8224
60	2015	10	5.6286	89.1327	944.5316	0.1255	1.6092
53	2015	10	5.0143	83.2449	854.4592	2.9878	3.1571
66	2015	10	5.2429	88.1735	899.4765	0.7490	2.4531
59	2015	10	4.4714	84.5408	888.8276	1.0418	2.3337
61	2015	10	9.1143	74.6429	968.1908	0.1194	1.0235
84	2015	10	9.1143	74.6429	968.1908	0.1194	1.0235
38	2015	10	4.4714	84.5408	888.8276	1.0418	2.3337
87	2015	10	6.8571	81.0102	901.3633	2.5633	2.7827
34	2015	10	4.4714	84.5408	888.8276	1.0418	2.3337
29	2015	10	7.2143	80.1327	944.8296	0.5439	1.5663
5	2015	10	7.3143	69.3571	831.6020	6.7990	2.8378
8	2015	10	5.0143	83.2449	854.4592	2.9878	3.1571
12	2015	10	7.3143	69.3571	831.6020	6.7990	2.8378
13	2015	10	11.8000	70.5102	946.2224	4.3378	1.3367
18	2015	10	8.0429	82.1939	970.9827	0.0490	1.1041
33	2015	10	6.6857	79.2347	904.3918	0.2204	2.0051
56	2015	10	8.6143	79.5000	982.8837	0.9490	1.3898
77	2015	10	8.8857	81.9490	937.6316	0.3847	1.2020
54	2015	10	7.3143	69.3571	831.6020	6.7990	2.8378
21	2015	10	6.6857	79.2347	904.3918	0.2204	2.0051
68	2015	10	8.0143	78.6531	976.8092	0.1388	1.8469
74	2015	10	9.1143	74.6429	968.1908	0.1194	1.0235
88	2015	10	4.3714	89.1735	876.3122	1.6694	2.8071
16	2015	10	6.5429	85.3571	925.1969	0.1306	1.9000
30	2015	10	5.2429	88.1735	899.4765	0.7490	2.4531
6	2015	10	8.0143	78.6531	976.8092	0.1388	1.8469
49	2015	10	7.2143	80.1327	944.8296	0.5439	1.5663

22	2015	10	4.3714	89.1735	876.3122	1.6694	2.8071
45	2015	10	13.1143	50.6633	816.3878	8.8449	3.2776
58	2015	10	7.2143	80.1327	944.8296	0.5439	1.5663
37	2015	10	8.0143	78.6531	976.8092	0.1388	1.8469
17	2015	10	4.8571	93.1327	905.0582	0.2622	3.0163
55	2015	10	6.7857	78.1633	878.4388	3.5398	2.9061
46	2015	10	6.5429	85.3571	925.1969	0.1306	1.9000
86	2015	10	5.7857	82.2959	866.9551	2.9520	0.9500
2	2015	10	5.7857	82.2959	866.9551	2.9520	0.9500
4	2015	10	6.6857	79.2347	904.3918	0.2204	2.0051
47	2015	10	11.0429	72.5510	961.8255	2.3622	0.9480
82	2015	10	4.3714	89.1735	876.3122	1.6694	2.8071
19	2015	10	9.2857	79.1020	964.7633	0.3327	1.6714
20	2015	10	5.0143	83.2449	854.4592	2.9878	3.1571
80	2015	10	4.3714	89.1735	876.3122	1.6694	2.8071
3	2015	10	11.8000	70.5102	946.2224	4.3378	1.3367
52	2015	10	4.8571	93.1327	905.0582	0.2622	3.0163
70	2015	10	7.3571	80.5816	913.2306	0.8602	1.5449
64	2015	10	8.4286	64.1122	774.0031	8.1684	2.6969
48	2015	10	8.8857	81.9490	937.6316	0.3847	1.2020
65	2015	10	4.8571	93.1327	905.0582	0.2622	3.0163
44	2015	10	7.3571	80.5816	913.2306	0.8602	1.5449
75	2015	10	8.4286	64.1122	774.0031	8.1684	2.6969
40	2015	10	5.7143	91.8367	950.6051	0.6786	1.8061
11	2015	10	6.7857	78.1633	878.4388	3.5398	2.9061
35	2015	10	5.6286	89.1327	944.5316	0.1255	1.6092
78	2015	10	6.8571	81.0102	901.3633	2.5633	2.7827
28	2015	10	5.9286	85.2245	933.2337	0.7214	1.9194
39	2015	10	4.8571	93.1327	905.0582	0.2622	3.0163
24	2015	10	7.2143	80.1327	944.8296	0.5439	1.5663
63	2015	10	5.7143	91.8367	950.6051	0.6786	1.8061
62	2015	10	4.8143	88.4184	875.8622	1.6827	1.6051
1	2015	10	4.3714	89.1735	876.3122	1.6694	2.8071
31	2015	11	10.1143	85.3980	848.7214	1.2949	0.9806
79	2015	11	11.8000	74.2857	978.8541	0.6173	1.8745
51	2015	11	10.4143	85.2347	946.6235	0.7949	1.4765
14	2015	11	11.2286	88.0102	901.7643	0.9755	1.9592
67	2015	11	10.6286	90.6531	907.3071	0.5255	2.7051
42	2015	11	10.8286	88.6429	878.5806	1.1969	2.6541
50	2015	11	11.6857	74.1122	906.7357	0.4143	1.6847
43	2015	11	10.8286	88.6429	878.5806	1.1969	2.6541
85	2015	11	12.5857	76.7551	915.7612	0.9745	1.4908
25	2015	11	13.6286	83.1939	985.6449	0.8276	0.9714
69	2015	11	12.4571	76.8980	947.1500	1.0724	1.4020
57	2015	11	10.7286	81.4490	891.1878	1.0163	2.0204
9	2015	11	10.6429	87.0816	856.6684	1.1337	2.6837
72	2015	11	12.4143	84.0408	880.9020	0.8133	2.5847
26	2015	11	13.0857	78.4694	869.2378	3.8918	2.4980
7	2015	11	12.4429	79.9490	861.3857	2.4204	2.3663
83	2015	11	16.6143	77.3061	949.5878	1.2878	1.1786

76	2015	11	11.5429	79.0714	927.3765	1.0520	1.6908
36	2015	11	11.2143	82.7959	935.5286	0.8929	1.7847
81	2015	11	10.4143	85.2347	946.6235	0.7949	1.4765
15	2015	11	12.8000	73.2551	939.8112	1.6388	1.0418
32	2015	11	10.8286	88.6429	878.5806	1.1969	2.6541
73	2015	11	13.4286	69.1735	970.2816	0.8000	1.1561
71	2015	11	11.2143	82.7959	935.5286	0.8929	1.7847
41	2015	11	11.1714	88.6429	878.2643	1.0480	1.3827
10	2015	11	10.8429	87.3061	972.8735	0.6173	0.9255
23	2015	11	9.8286	68.6327	775.4398	7.8408	2.6265
27	2015	11	12.4429	79.9490	861.3857	2.4204	2.3663
60	2015	11	10.4143	85.2347	946.6235	0.7949	1.4765
53	2015	11	10.6429	87.0816	856.6684	1.1337	2.6837
66	2015	11	11.2286	88.0102	901.7643	0.9755	1.9592
59	2015	11	10.7286	81.4490	891.1878	1.0163	2.0204
61	2015	11	13.4286	69.1735	970.2816	0.8000	1.1561
84	2015	11	13.4286	69.1735	970.2816	0.8000	1.1561
38	2015	11	10.7286	81.4490	891.1878	1.0163	2.0204
87	2015	11	13.4714	82.6429	903.8878	1.7031	2.2531
34	2015	11	10.7286	81.4490	891.1878	1.0163	2.0204
29	2015	11	12.4571	76.8980	947.1500	1.0724	1.4020
5	2015	11	10.4143	84.5306	833.5867	3.8316	2.5316
8	2015	11	10.6429	87.0816	856.6684	1.1337	2.6837
12	2015	11	10.4143	84.5306	833.5867	3.8316	2.5316
13	2015	11	16.6143	77.3061	949.5878	1.2878	1.1786
18	2015	11	11.7000	76.0816	973.0000	0.5837	1.2388
33	2015	11	11.6857	74.1122	906.7357	0.4143	1.6847
56	2015	11	13.6286	83.1939	985.6449	0.8276	0.9714
77	2015	11	12.8000	73.2551	939.8112	1.6388	1.0418
54	2015	11	10.4143	84.5306	833.5867	3.8316	2.5316
21	2015	11	11.6857	74.1122	906.7357	0.4143	1.6847
68	2015	11	11.8000	74.2857	978.8541	0.6173	1.8745
74	2015	11	13.4286	69.1735	970.2816	0.8000	1.1561
88	2015	11	10.8286	88.6429	878.5806	1.1969	2.6541
16	2015	11	11.5429	79.0714	927.3765	1.0520	1.6908
30	2015	11	11.2286	88.0102	901.7643	0.9755	1.9592
6	2015	11	11.8000	74.2857	978.8541	0.6173	1.8745
49	2015	11	12.4571	76.8980	947.1500	1.0724	1.4020
22	2015	11	10.8286	88.6429	878.5806	1.1969	2.6541
45	2015	11	12.2143	64.2245	817.9439	7.8439	3.1000
58	2015	11	12.4571	76.8980	947.1500	1.0724	1.4020
37	2015	11	11.8000	74.2857	978.8541	0.6173	1.8745
17	2015	11	10.6286	90.6531	907.3071	0.5255	2.7051
55	2015	11	12.4143	84.0408	880.9020	0.8133	2.5847
46	2015	11	11.5429	79.0714	927.3765	1.0520	1.6908
86	2015	11	11.2143	85.2857	869.3235	1.5724	0.8306
2	2015	11	11.2143	85.2857	869.3235	1.5724	0.8306
4	2015	11	11.6857	74.1122	906.7357	0.4143	1.6847
47	2015	11	16.5286	75.2143	965.1276	0.9929	0.8071
82	2015	11	10.8286	88.6429	878.5806	1.1969	2.6541

19	2015	11	14.0429	79.3673	967.5582	0.3969	1.3041
20	2015	11	10.6429	87.0816	856.6684	1.1337	2.6837
80	2015	11	10.8286	88.6429	878.5806	1.1969	2.6541
3	2015	11	16.6143	77.3061	949.5878	1.2878	1.1786
52	2015	11	10.6286	90.6531	907.3071	0.5255	2.7051
70	2015	11	12.5857	76.7551	915.7612	0.9745	1.4908
64	2015	11	9.8286	68.6327	775.4398	7.8408	2.6265
48	2015	11	12.8000	73.2551	939.8112	1.6388	1.0418
65	2015	11	10.6286	90.6531	907.3071	0.5255	2.7051
44	2015	11	12.5857	76.7551	915.7612	0.9745	1.4908
75	2015	11	9.8286	68.6327	775.4398	7.8408	2.6265
40	2015	11	10.5286	90.9082	952.8449	0.7071	1.4561
11	2015	11	12.4143	84.0408	880.9020	0.8133	2.5847
35	2015	11	10.4143	85.2347	946.6235	0.7949	1.4765
78	2015	11	13.4714	82.6429	903.8878	1.7031	2.2531
28	2015	11	11.2143	82.7959	935.5286	0.8929	1.7847
39	2015	11	10.6286	90.6531	907.3071	0.5255	2.7051
24	2015	11	12.4571	76.8980	947.1500	1.0724	1.4020
63	2015	11	10.5286	90.9082	952.8449	0.7071	1.4561
62	2015	11	11.1714	88.6429	878.2643	1.0480	1.3827
1	2015	11	10.8286	88.6429	878.5806	1.1969	2.6541
31	2015	12	14.8000	78.0918	848.5082	3.8878	1.0276
79	2015	12	14.3286	78.0408	976.9418	1.1429	1.8327
51	2015	12	13.1714	85.5510	945.0622	1.6031	1.5051
14	2015	12	17.2143	86.9286	900.9663	2.3051	2.4439
67	2015	12	15.2857	89.0714	906.2265	1.2592	2.5122
42	2015	12	16.0286	83.5918	877.8714	3.2071	2.7265
50	2015	12	14.7143	74.5000	905.6704	1.3418	1.6000
43	2015	12	16.0286	83.5918	877.8714	3.2071	2.7265
85	2015	12	16.1000	74.4082	914.5357	2.4959	1.5469
25	2015	12	18.9000	82.2143	983.4796	1.7673	0.9500
69	2015	12	15.2714	77.3367	945.4112	2.0286	1.3520
57	2015	12	13.6714	77.5408	890.2582	2.3449	2.1276
9	2015	12	16.5714	80.8265	856.4061	3.4959	2.5469
72	2015	12	18.5429	78.4286	880.3500	3.1082	2.3112
26	2015	12	21.0286	75.0510	869.2173	4.9429	2.3316
7	2015	12	19.3429	74.7347	861.3143	4.5837	2.1816
83	2015	12	23.1000	75.0204	948.2347	3.0286	1.0582
76	2015	12	13.9714	79.9082	926.0020	1.9153	1.5684
36	2015	12	15.2286	82.3367	933.9673	1.9133	1.6806
81	2015	12	13.1714	85.5510	945.0622	1.6031	1.5051
15	2015	12	15.5714	76.5000	938.3133	2.2796	0.9898
32	2015	12	16.0286	83.5918	877.8714	3.2071	2.7265
73	2015	12	15.9857	71.2959	968.3622	1.5204	1.1459
71	2015	12	15.2286	82.3367	933.9673	1.9133	1.6806
41	2015	12	15.7714	81.5000	877.5020	3.3684	1.4010
10	2015	12	14.3143	88.3367	971.0122	1.3367	0.9653
23	2015	12	14.1000	64.3673	776.3684	8.6082	2.4592
27	2015	12	19.3429	74.7347	861.3143	4.5837	2.1816
60	2015	12	13.1714	85.5510	945.0622	1.6031	1.5051

53	2015	12	16.5714	80.8265	856.4061	3.4959	2.5469
66	2015	12	17.2143	86.9286	900.9663	2.3051	2.4439
59	2015	12	13.6714	77.5408	890.2582	2.3449	2.1276
61	2015	12	15.9857	71.2959	968.3622	1.5204	1.1459
84	2015	12	15.9857	71.2959	968.3622	1.5204	1.1459
38	2015	12	13.6714	77.5408	890.2582	2.3449	2.1276
87	2015	12	19.5286	76.8776	902.7776	4.2571	1.9959
34	2015	12	13.6714	77.5408	890.2582	2.3449	2.1276
29	2015	12	15.2714	77.3367	945.4112	2.0286	1.3520
5	2015	12	18.5857	75.2347	833.8316	5.5214	2.5092
8	2015	12	16.5714	80.8265	856.4061	3.4959	2.5469
12	2015	12	18.5857	75.2347	833.8316	5.5214	2.5092
13	2015	12	23.1000	75.0204	948.2347	3.0286	1.0582
18	2015	12	14.2143	79.8571	971.1643	0.7357	1.1633
33	2015	12	14.7143	74.5000	905.6704	1.3418	1.6000
56	2015	12	18.9000	82.2143	983.4796	1.7673	0.9500
77	2015	12	15.5714	76.5000	938.3133	2.2796	0.9898
54	2015	12	18.5857	75.2347	833.8316	5.5214	2.5092
21	2015	12	14.7143	74.5000	905.6704	1.3418	1.6000
68	2015	12	14.3286	78.0408	976.9418	1.1429	1.8327
74	2015	12	15.9857	71.2959	968.3622	1.5204	1.1459
88	2015	12	16.0286	83.5918	877.8714	3.2071	2.7265
16	2015	12	13.9714	79.9082	926.0020	1.9153	1.5684
30	2015	12	17.2143	86.9286	900.9663	2.3051	2.4439
6	2015	12	14.3286	78.0408	976.9418	1.1429	1.8327
49	2015	12	15.2714	77.3367	945.4112	2.0286	1.3520
22	2015	12	16.0286	83.5918	877.8714	3.2071	2.7265
45	2015	12	18.8000	61.1020	818.5367	8.4949	2.8673
58	2015	12	15.2714	77.3367	945.4112	2.0286	1.3520
37	2015	12	14.3286	78.0408	976.9418	1.1429	1.8327
17	2015	12	15.2857	89.0714	906.2265	1.2592	2.5122
55	2015	12	18.5429	78.4286	880.3500	3.1082	2.3112
46	2015	12	13.9714	79.9082	926.0020	1.9153	1.5684
86	2015	12	16.2857	78.3061	868.7786	3.5735	0.9031
2	2015	12	16.2857	78.3061	868.7786	3.5735	0.9031
4	2015	12	14.7143	74.5000	905.6704	1.3418	1.6000
47	2015	12	23.0571	74.0102	963.1612	2.6133	0.7327
82	2015	12	16.0286	83.5918	877.8714	3.2071	2.7265
19	2015	12	20.6571	80.9694	965.8612	1.1551	1.1786
20	2015	12	16.5714	80.8265	856.4061	3.4959	2.5469
80	2015	12	16.0286	83.5918	877.8714	3.2071	2.7265
3	2015	12	23.1000	75.0204	948.2347	3.0286	1.0582
52	2015	12	15.2857	89.0714	906.2265	1.2592	2.5122
70	2015	12	16.1000	74.4082	914.5357	2.4959	1.5469
64	2015	12	14.1000	64.3673	776.3684	8.6082	2.4592
48	2015	12	15.5714	76.5000	938.3133	2.2796	0.9898
65	2015	12	15.2857	89.0714	906.2265	1.2592	2.5122
44	2015	12	16.1000	74.4082	914.5357	2.4959	1.5469
75	2015	12	14.1000	64.3673	776.3684	8.6082	2.4592
40	2015	12	14.7000	90.2653	951.3143	1.3908	1.4796

11	2015	12	18.5429	78.4286	880.3500	3.1082	2.3112
35	2015	12	13.1714	85.5510	945.0622	1.6031	1.5051
78	2015	12	19.5286	76.8776	902.7776	4.2571	1.9959
28	2015	12	15.2286	82.3367	933.9673	1.9133	1.6806
39	2015	12	15.2857	89.0714	906.2265	1.2592	2.5122
24	2015	12	15.2714	77.3367	945.4112	2.0286	1.3520
63	2015	12	14.7000	90.2653	951.3143	1.3908	1.4796
62	2015	12	15.7714	81.5000	877.5020	3.3684	1.4010
1	2015	12	16.0286	83.5918	877.8714	3.2071	2.7265
31	2015	13	13.2143	76.2857	849.4622	4.1398	1.1133
79	2015	13	14.1429	80.7347	976.9153	1.3918	1.4643
51	2015	13	12.5571	87.2449	945.3194	1.3837	1.4745
14	2015	13	13.7286	85.7143	901.5847	2.1071	2.7000
67	2015	13	12.5857	91.1939	906.7714	0.9816	2.4561
42	2015	13	12.7143	85.1633	878.6500	3.0163	2.6776
50	2015	13	13.6429	77.8163	906.2531	1.3531	1.7092
43	2015	13	12.7143	85.1633	878.6500	3.0163	2.6776
85	2015	13	14.8143	75.8061	914.9520	2.4990	1.6602
25	2015	13	16.8429	80.9286	983.1224	1.9255	1.0827
69	2015	13	14.1143	80.7653	945.6755	1.5092	1.3643
57	2015	13	12.5857	80.7041	890.9286	2.1602	2.1306
9	2015	13	12.5286	78.9592	857.3551	3.5102	2.5714
72	2015	13	14.4714	75.6531	881.0969	3.6531	2.3204
26	2015	13	15.5857	67.7959	870.1653	5.3898	2.5541
7	2015	13	14.8000	69.0510	862.2704	5.1684	2.3051
83	2015	13	19.4143	68.5102	948.2959	3.9306	1.1061
76	2015	13	13.3286	84.1327	926.4510	1.3429	1.5378
36	2015	13	13.2429	83.5510	934.2592	1.5500	1.6480
81	2015	13	12.5571	87.2449	945.3194	1.3837	1.4745
15	2015	13	14.9857	82.0612	938.6939	1.7857	1.0357
32	2015	13	12.7143	85.1633	878.6500	3.0163	2.6776
73	2015	13	15.3000	75.5918	968.5133	1.3878	1.1316
71	2015	13	13.2429	83.5510	934.2592	1.5500	1.6480
41	2015	13	13.6143	81.8571	878.2082	3.3306	1.5653
10	2015	13	13.7714	88.5306	970.9765	1.4439	0.9694
23	2015	13	10.9571	65.8367	777.9745	7.2755	2.6602
27	2015	13	14.8000	69.0510	862.2704	5.1684	2.3051
60	2015	13	12.5571	87.2449	945.3194	1.3837	1.4745
53	2015	13	12.5286	78.9592	857.3551	3.5102	2.5714
66	2015	13	13.7286	85.7143	901.5847	2.1071	2.7000
59	2015	13	12.5857	80.7041	890.9286	2.1602	2.1306
61	2015	13	15.3000	75.5918	968.5133	1.3878	1.1316
84	2015	13	15.3000	75.5918	968.5133	1.3878	1.1316
38	2015	13	12.5857	80.7041	890.9286	2.1602	2.1306
87	2015	13	14.7857	76.1224	903.3122	4.1051	2.0898
34	2015	13	12.5857	80.7041	890.9286	2.1602	2.1306
29	2015	13	14.1143	80.7653	945.6755	1.5092	1.3643
5	2015	13	13.1857	68.1531	834.9969	6.0612	2.6714
8	2015	13	12.5286	78.9592	857.3551	3.5102	2.5714
12	2015	13	13.1857	68.1531	834.9969	6.0612	2.6714

13	2015	13	19.4143	68.5102	948.2959	3.9306	1.1061
18	2015	13	14.3429	83.1224	971.1673	0.4204	1.0316
33	2015	13	13.6429	77.8163	906.2531	1.3531	1.7092
56	2015	13	16.8429	80.9286	983.1224	1.9255	1.0827
77	2015	13	14.9857	82.0612	938.6939	1.7857	1.0357
54	2015	13	13.1857	68.1531	834.9969	6.0612	2.6714
21	2015	13	13.6429	77.8163	906.2531	1.3531	1.7092
68	2015	13	14.1429	80.7347	976.9153	1.3918	1.4643
74	2015	13	15.3000	75.5918	968.5133	1.3878	1.1316
88	2015	13	12.7143	85.1633	878.6500	3.0163	2.6776
16	2015	13	13.3286	84.1327	926.4510	1.3429	1.5378
30	2015	13	13.7286	85.7143	901.5847	2.1071	2.7000
6	2015	13	14.1429	80.7347	976.9153	1.3918	1.4643
49	2015	13	14.1143	80.7653	945.6755	1.5092	1.3643
22	2015	13	12.7143	85.1633	878.6500	3.0163	2.6776
45	2015	13	13.4714	60.7143	819.9745	6.9296	2.5235
58	2015	13	14.1143	80.7653	945.6755	1.5092	1.3643
37	2015	13	14.1429	80.7347	976.9153	1.3918	1.4643
17	2015	13	12.5857	91.1939	906.7714	0.9816	2.4561
55	2015	13	14.4714	75.6531	881.0969	3.6531	2.3204
46	2015	13	13.3286	84.1327	926.4510	1.3429	1.5378
86	2015	13	13.6571	76.4286	869.5867	3.4622	1.0143
2	2015	13	13.6571	76.4286	869.5867	3.4622	1.0143
4	2015	13	13.6429	77.8163	906.2531	1.3531	1.7092
47	2015	13	19.1429	71.5510	962.9684	2.9541	0.7347
82	2015	13	12.7143	85.1633	878.6500	3.0163	2.6776
19	2015	13	17.1714	79.8061	965.7449	1.4378	1.2571
20	2015	13	12.5286	78.9592	857.3551	3.5102	2.5714
80	2015	13	12.7143	85.1633	878.6500	3.0163	2.6776
3	2015	13	19.4143	68.5102	948.2959	3.9306	1.1061
52	2015	13	12.5857	91.1939	906.7714	0.9816	2.4561
70	2015	13	14.8143	75.8061	914.9520	2.4990	1.6602
64	2015	13	10.9571	65.8367	777.9745	7.2755	2.6602
48	2015	13	14.9857	82.0612	938.6939	1.7857	1.0357
65	2015	13	12.5857	91.1939	906.7714	0.9816	2.4561
44	2015	13	14.8143	75.8061	914.9520	2.4990	1.6602
75	2015	13	10.9571	65.8367	777.9745	7.2755	2.6602
40	2015	13	13.7143	89.6837	951.4571	1.5357	1.5500
11	2015	13	14.4714	75.6531	881.0969	3.6531	2.3204
35	2015	13	12.5571	87.2449	945.3194	1.3837	1.4745
78	2015	13	14.7857	76.1224	903.3122	4.1051	2.0898
28	2015	13	13.2429	83.5510	934.2592	1.5500	1.6480
39	2015	13	12.5857	91.1939	906.7714	0.9816	2.4561
24	2015	13	14.1143	80.7653	945.6755	1.5092	1.3643
63	2015	13	13.7143	89.6837	951.4571	1.5357	1.5500
62	2015	13	13.6143	81.8571	878.2082	3.3306	1.5653
1	2015	13	12.7143	85.1633	878.6500	3.0163	2.6776
31	2015	14	20.3286	73.3878	848.0316	4.9357	1.1612
79	2015	14	19.6571	80.9286	974.7214	1.8704	1.3622
51	2015	14	20.3857	83.6429	943.5337	2.2061	1.4490

14	2015	14	22.3429	83.2245	900.8929	1.9551	2.8796
67	2015	14	22.3143	88.1939	905.6357	1.3112	3.1684
42	2015	14	22.0857	82.8776	877.6959	3.2622	2.8163
50	2015	14	21.8714	74.8571	904.3490	2.9541	1.8296
43	2015	14	22.0857	82.8776	877.6959	3.2622	2.8163
85	2015	14	24.1143	70.7755	912.7765	3.7918	2.1908
25	2015	14	25.7714	79.5714	981.5255	2.2969	1.1694
69	2015	14	22.9571	78.4898	943.7786	1.8612	1.5469
57	2015	14	22.6714	73.8163	889.4286	3.9500	2.9898
9	2015	14	21.6714	76.8673	856.6378	3.7949	2.7622
72	2015	14	23.8143	73.3367	880.4816	3.7347	2.4520
26	2015	14	24.3286	67.3367	869.8327	5.5622	2.7265
7	2015	14	23.1571	68.9286	861.8571	5.1020	2.9704
83	2015	14	26.1286	67.4388	947.6541	4.5714	1.1520
76	2015	14	21.4714	81.3265	924.5010	2.9510	1.6929
36	2015	14	23.0286	79.9694	932.7582	2.6163	1.9684
81	2015	14	20.3857	83.6429	943.5337	2.2061	1.4490
15	2015	14	21.8571	80.4490	936.4857	2.6367	1.1102
32	2015	14	22.0857	82.8776	877.6959	3.2622	2.8163
73	2015	14	22.4429	75.4184	966.2031	2.8500	1.2531
71	2015	14	23.0286	79.9694	932.7582	2.6163	1.9684
41	2015	14	21.6857	77.6531	876.8153	4.2000	1.6888
10	2015	14	21.7714	84.5816	969.0429	2.4408	1.1704
23	2015	14	17.3571	65.9388	777.5020	6.8204	2.9622
27	2015	14	23.1571	68.9286	861.8571	5.1020	2.9704
60	2015	14	20.3857	83.6429	943.5337	2.2061	1.4490
53	2015	14	21.6714	76.8673	856.6378	3.7949	2.7622
66	2015	14	22.3429	83.2245	900.8929	1.9551	2.8796
59	2015	14	22.6714	73.8163	889.4286	3.9500	2.9898
61	2015	14	22.4429	75.4184	966.2031	2.8500	1.2531
84	2015	14	22.4429	75.4184	966.2031	2.8500	1.2531
38	2015	14	22.6714	73.8163	889.4286	3.9500	2.9898
87	2015	14	23.2429	77.6939	902.4633	3.3031	1.7071
34	2015	14	22.6714	73.8163	889.4286	3.9500	2.9898
29	2015	14	22.9571	78.4898	943.7786	1.8612	1.5469
5	2015	14	21.3857	68.7041	834.5633	6.0163	2.9173
8	2015	14	21.6714	76.8673	856.6378	3.7949	2.7622
12	2015	14	21.3857	68.7041	834.5633	6.0163	2.9173
13	2015	14	26.1286	67.4388	947.6541	4.5714	1.1520
18	2015	14	19.3714	82.6224	968.9673	0.9551	1.1827
33	2015	14	21.8714	74.8571	904.3490	2.9541	1.8296
56	2015	14	25.7714	79.5714	981.5255	2.2969	1.1694
77	2015	14	21.8571	80.4490	936.4857	2.6367	1.1102
54	2015	14	21.3857	68.7041	834.5633	6.0163	2.9173
21	2015	14	21.8714	74.8571	904.3490	2.9541	1.8296
68	2015	14	19.6571	80.9286	974.7214	1.8704	1.3622
74	2015	14	22.4429	75.4184	966.2031	2.8500	1.2531
88	2015	14	22.0857	82.8776	877.6959	3.2622	2.8163
16	2015	14	21.4714	81.3265	924.5010	2.9510	1.6929
30	2015	14	22.3429	83.2245	900.8929	1.9551	2.8796

6	2015	14	19.6571	80.9286	974.7214	1.8704	1.3622
49	2015	14	22.9571	78.4898	943.7786	1.8612	1.5469
22	2015	14	22.0857	82.8776	877.6959	3.2622	2.8163
45	2015	14	20.5000	63.1020	819.7143	6.7102	2.5347
58	2015	14	22.9571	78.4898	943.7786	1.8612	1.5469
37	2015	14	19.6571	80.9286	974.7214	1.8704	1.3622
17	2015	14	22.3143	88.1939	905.6357	1.3112	3.1684
55	2015	14	23.8143	73.3367	880.4816	3.7347	2.4520
46	2015	14	21.4714	81.3265	924.5010	2.9510	1.6929
86	2015	14	21.7857	73.4592	868.4245	4.0765	1.2163
2	2015	14	21.7857	73.4592	868.4245	4.0765	1.2163
4	2015	14	21.8714	74.8571	904.3490	2.9541	1.8296
47	2015	14	26.8000	70.3163	962.0786	2.4724	0.7827
82	2015	14	22.0857	82.8776	877.6959	3.2622	2.8163
19	2015	14	25.2429	79.3367	964.7500	1.4867	1.2480
20	2015	14	21.6714	76.8673	856.6378	3.7949	2.7622
80	2015	14	22.0857	82.8776	877.6959	3.2622	2.8163
3	2015	14	26.1286	67.4388	947.6541	4.5714	1.1520
52	2015	14	22.3143	88.1939	905.6357	1.3112	3.1684
70	2015	14	24.1143	70.7755	912.7765	3.7918	2.1908
64	2015	14	17.3571	65.9388	777.5020	6.8204	2.9622
48	2015	14	21.8571	80.4490	936.4857	2.6367	1.1102
65	2015	14	22.3143	88.1939	905.6357	1.3112	3.1684
44	2015	14	24.1143	70.7755	912.7765	3.7918	2.1908
75	2015	14	17.3571	65.9388	777.5020	6.8204	2.9622
40	2015	14	22.8571	85.7143	950.0184	2.3153	1.8847
11	2015	14	23.8143	73.3367	880.4816	3.7347	2.4520
35	2015	14	20.3857	83.6429	943.5337	2.2061	1.4490
78	2015	14	23.2429	77.6939	902.4633	3.3031	1.7071
28	2015	14	23.0286	79.9694	932.7582	2.6163	1.9684
39	2015	14	22.3143	88.1939	905.6357	1.3112	3.1684
24	2015	14	22.9571	78.4898	943.7786	1.8612	1.5469
63	2015	14	22.8571	85.7143	950.0184	2.3153	1.8847
62	2015	14	21.6857	77.6531	876.8153	4.2000	1.6888
1	2015	14	22.0857	82.8776	877.6959	3.2622	2.8163
31	2015	15	9.6857	70.2041	845.1939	5.5582	1.2490
79	2015	15	12.5143	80.3061	971.5388	1.9816	2.1439
51	2015	15	10.7714	81.2551	940.3306	2.6622	1.8112
14	2015	15	11.4571	77.3980	897.8357	3.3449	3.2449
67	2015	15	10.6714	80.9592	902.4990	2.6745	3.8255
42	2015	15	10.4286	74.0918	874.6704	4.8500	3.2980
50	2015	15	11.4857	72.2041	901.0347	4.1316	2.0541
43	2015	15	10.4286	74.0918	874.6704	4.8500	3.2980
85	2015	15	12.1714	68.3163	909.4245	4.7898	2.4224
25	2015	15	14.9143	72.8571	977.9857	3.7929	1.6408
69	2015	15	12.3143	73.7041	940.3857	2.4857	1.8378
57	2015	15	10.5857	64.3980	886.2265	5.0990	3.5847
9	2015	15	10.7286	67.7857	853.7541	5.4469	3.1643
72	2015	15	13.2000	63.3163	877.4765	5.6153	2.7316
26	2015	15	14.4429	57.6633	867.1245	6.7878	2.9041

7	2015	15	13.2571	59.9184	859.1112	6.5684	3.5245
83	2015	15	18.5429	60.7449	944.2071	6.1469	1.4031
76	2015	15	11.6286	78.0000	921.1214	4.1102	1.9357
36	2015	15	11.5571	74.7143	929.4806	3.8684	2.3347
81	2015	15	10.7714	81.2551	940.3306	2.6622	1.8112
15	2015	15	13.1143	78.3980	933.0357	3.0745	1.2929
32	2015	15	10.4286	74.0918	874.6704	4.8500	3.2980
73	2015	15	13.8000	72.6020	962.6867	4.0031	1.3490
71	2015	15	11.5571	74.7143	929.4806	3.8684	2.3347
41	2015	15	10.9429	71.1429	873.7398	5.2949	1.7673
10	2015	15	11.6286	81.8776	965.8071	3.2765	1.5061
23	2015	15	6.7429	63.9082	775.1908	7.0327	2.9041
27	2015	15	13.2571	59.9184	859.1112	6.5684	3.5245
60	2015	15	10.7714	81.2551	940.3306	2.6622	1.8112
53	2015	15	10.7286	67.7857	853.7541	5.4469	3.1643
66	2015	15	11.4571	77.3980	897.8357	3.3449	3.2449
59	2015	15	10.5857	64.3980	886.2265	5.0990	3.5847
61	2015	15	13.8000	72.6020	962.6867	4.0031	1.3490
84	2015	15	13.8000	72.6020	962.6867	4.0031	1.3490
38	2015	15	10.5857	64.3980	886.2265	5.0990	3.5847
87	2015	15	13.2286	70.7041	899.3051	4.7796	1.9337
34	2015	15	10.5857	64.3980	886.2265	5.0990	3.5847
29	2015	15	12.3143	73.7041	940.3857	2.4857	1.8378
5	2015	15	11.1286	61.1633	831.9878	7.0337	3.3235
8	2015	15	10.7286	67.7857	853.7541	5.4469	3.1643
12	2015	15	11.1286	61.1633	831.9878	7.0337	3.3235
13	2015	15	18.5429	60.7449	944.2071	6.1469	1.4031
18	2015	15	12.1857	83.3776	965.8418	1.3592	1.3653
33	2015	15	11.4857	72.2041	901.0347	4.1316	2.0541
56	2015	15	14.9143	72.8571	977.9857	3.7929	1.6408
77	2015	15	13.1143	78.3980	933.0357	3.0745	1.2929
54	2015	15	11.1286	61.1633	831.9878	7.0337	3.3235
21	2015	15	11.4857	72.2041	901.0347	4.1316	2.0541
68	2015	15	12.5143	80.3061	971.5388	1.9816	2.1439
74	2015	15	13.8000	72.6020	962.6867	4.0031	1.3490
88	2015	15	10.4286	74.0918	874.6704	4.8500	3.2980
16	2015	15	11.6286	78.0000	921.1214	4.1102	1.9357
30	2015	15	11.4571	77.3980	897.8357	3.3449	3.2449
6	2015	15	12.5143	80.3061	971.5388	1.9816	2.1439
49	2015	15	12.3143	73.7041	940.3857	2.4857	1.8378
22	2015	15	10.4286	74.0918	874.6704	4.8500	3.2980
45	2015	15	11.3286	57.7245	817.3367	7.5990	2.9898
58	2015	15	12.3143	73.7041	940.3857	2.4857	1.8378
37	2015	15	12.5143	80.3061	971.5388	1.9816	2.1439
17	2015	15	10.6714	80.9592	902.4990	2.6745	3.8255
55	2015	15	13.2000	63.3163	877.4765	5.6153	2.7316
46	2015	15	11.6286	78.0000	921.1214	4.1102	1.9357
86	2015	15	11.1857	67.5102	865.4418	5.2306	1.3041
2	2015	15	11.1857	67.5102	865.4418	5.2306	1.3041
4	2015	15	11.4857	72.2041	901.0347	4.1316	2.0541

47	2015	15	18.4714	63.1429	958.4735	3.8429	0.9755
82	2015	15	10.4286	74.0918	874.6704	4.8500	3.2980
19	2015	15	15.2429	76.3776	961.4102	2.4245	1.5388
20	2015	15	10.7286	67.7857	853.7541	5.4469	3.1643
80	2015	15	10.4286	74.0918	874.6704	4.8500	3.2980
3	2015	15	18.5429	60.7449	944.2071	6.1469	1.4031
52	2015	15	10.6714	80.9592	902.4990	2.6745	3.8255
70	2015	15	12.1714	68.3163	909.4245	4.7898	2.4224
64	2015	15	6.7429	63.9082	775.1908	7.0327	2.9041
48	2015	15	13.1143	78.3980	933.0357	3.0745	1.2929
65	2015	15	10.6714	80.9592	902.4990	2.6745	3.8255
44	2015	15	12.1714	68.3163	909.4245	4.7898	2.4224
75	2015	15	6.7429	63.9082	775.1908	7.0327	2.9041
40	2015	15	10.8000	81.8163	946.8010	3.3306	2.4408
11	2015	15	13.2000	63.3163	877.4765	5.6153	2.7316
35	2015	15	10.7714	81.2551	940.3306	2.6622	1.8112
78	2015	15	13.2286	70.7041	899.3051	4.7796	1.9337
28	2015	15	11.5571	74.7143	929.4806	3.8684	2.3347
39	2015	15	10.6714	80.9592	902.4990	2.6745	3.8255
24	2015	15	12.3143	73.7041	940.3857	2.4857	1.8378
63	2015	15	10.8000	81.8163	946.8010	3.3306	2.4408
62	2015	15	10.9429	71.1429	873.7398	5.2949	1.7673
1	2015	15	10.4286	74.0918	874.6704	4.8500	3.2980
31	2015	16	17.6429	70.9592	848.6347	5.1041	1.2296
79	2015	16	21.0714	70.9490	974.6786	4.4306	2.2245
51	2015	16	20.1000	76.0102	943.4357	4.5204	1.7796
14	2015	16	19.7857	69.5102	900.5235	5.3051	2.8143
67	2015	16	19.4857	73.5102	905.4755	4.4582	3.3561
42	2015	16	20.0000	66.9388	877.6765	5.4867	3.1255
50	2015	16	19.4571	68.9898	904.7439	4.2653	1.9551
43	2015	16	20.0000	66.9388	877.6765	5.4867	3.1255
85	2015	16	20.6857	68.5102	913.3990	5.0357	1.8245
25	2015	16	22.7429	68.3265	981.2459	5.5459	1.5357
69	2015	16	20.7286	70.6531	943.8490	4.2582	1.7888
57	2015	16	19.7857	60.0408	889.6286	5.0551	3.0980
9	2015	16	20.0714	62.1020	856.6296	5.6776	2.9306
72	2015	16	21.0000	59.2449	880.3173	5.8408	2.4684
26	2015	16	22.7857	58.9898	869.7724	5.8122	2.3214
7	2015	16	21.7571	59.4490	861.7643	6.2694	2.8255
83	2015	16	24.9571	57.0408	947.3571	5.8622	1.3429
76	2015	16	20.5286	71.2245	924.5653	5.1194	1.8255
36	2015	16	21.1714	69.2551	932.6806	5.2704	2.1092
81	2015	16	20.1000	76.0102	943.4357	4.5204	1.7796
15	2015	16	20.0857	72.7041	936.7888	4.3020	1.2245
32	2015	16	20.0000	66.9388	877.6765	5.4867	3.1255
73	2015	16	22.0857	67.1939	966.1612	5.1490	1.2949
71	2015	16	21.1714	69.2551	932.6806	5.2704	2.1092
41	2015	16	19.7857	69.3673	877.1286	5.2398	1.6776
10	2015	16	20.8286	76.9592	968.9908	5.2061	1.3071
23	2015	16	16.7429	65.4592	777.7704	6.1684	2.5173

27	2015	16	21.7571	59.4490	861.7643	6.2694	2.8255
60	2015	16	20.1000	76.0102	943.4357	4.5204	1.7796
53	2015	16	20.0714	62.1020	856.6296	5.6776	2.9306
66	2015	16	19.7857	69.5102	900.5235	5.3051	2.8143
59	2015	16	19.7857	60.0408	889.6286	5.0551	3.0980
61	2015	16	22.0857	67.1939	966.1612	5.1490	1.2949
84	2015	16	22.0857	67.1939	966.1612	5.1490	1.2949
38	2015	16	19.7857	60.0408	889.6286	5.0551	3.0980
87	2015	16	20.9429	63.0816	902.3510	5.8163	2.2806
34	2015	16	19.7857	60.0408	889.6286	5.0551	3.0980
29	2015	16	20.7286	70.6531	943.8490	4.2582	1.7888
5	2015	16	20.8857	60.6327	834.6265	6.7184	2.7327
8	2015	16	20.0714	62.1020	856.6296	5.6776	2.9306
12	2015	16	20.8857	60.6327	834.6265	6.7184	2.7327
13	2015	16	24.9571	57.0408	947.3571	5.8622	1.3429
18	2015	16	20.2571	75.2041	968.9847	4.0684	1.2612
33	2015	16	19.4571	68.9898	904.7439	4.2653	1.9551
56	2015	16	22.7429	68.3265	981.2459	5.5459	1.5357
77	2015	16	20.0857	72.7041	936.7888	4.3020	1.2245
54	2015	16	20.8857	60.6327	834.6265	6.7184	2.7327
21	2015	16	19.4571	68.9898	904.7439	4.2653	1.9551
68	2015	16	21.0714	70.9490	974.6786	4.4306	2.2245
74	2015	16	22.0857	67.1939	966.1612	5.1490	1.2949
88	2015	16	20.0000	66.9388	877.6765	5.4867	3.1255
16	2015	16	20.5286	71.2245	924.5653	5.1194	1.8255
30	2015	16	19.7857	69.5102	900.5235	5.3051	2.8143
6	2015	16	21.0714	70.9490	974.6786	4.4306	2.2245
49	2015	16	20.7286	70.6531	943.8490	4.2582	1.7888
22	2015	16	20.0000	66.9388	877.6765	5.4867	3.1255
45	2015	16	20.1429	60.4898	819.8031	6.6316	2.4929
58	2015	16	20.7286	70.6531	943.8490	4.2582	1.7888
37	2015	16	21.0714	70.9490	974.6786	4.4306	2.2245
17	2015	16	19.4857	73.5102	905.4755	4.4582	3.3561
55	2015	16	21.0000	59.2449	880.3173	5.8408	2.4684
46	2015	16	20.5286	71.2245	924.5653	5.1194	1.8255
86	2015	16	19.1000	66.0918	868.7255	5.4918	1.0867
2	2015	16	19.1000	66.0918	868.7255	5.4918	1.0867
4	2015	16	19.4571	68.9898	904.7439	4.2653	1.9551
47	2015	16	24.5857	58.0816	961.7327	5.0551	0.9571
82	2015	16	20.0000	66.9388	877.6765	5.4867	3.1255
19	2015	16	22.4429	69.0510	964.3990	4.5112	1.6429
20	2015	16	20.0714	62.1020	856.6296	5.6776	2.9306
80	2015	16	20.0000	66.9388	877.6765	5.4867	3.1255
3	2015	16	24.9571	57.0408	947.3571	5.8622	1.3429
52	2015	16	19.4857	73.5102	905.4755	4.4582	3.3561
70	2015	16	20.6857	68.5102	913.3990	5.0357	1.8245
64	2015	16	16.7429	65.4592	777.7704	6.1684	2.5173
48	2015	16	20.0857	72.7041	936.7888	4.3020	1.2245
65	2015	16	19.4857	73.5102	905.4755	4.4582	3.3561
44	2015	16	20.6857	68.5102	913.3990	5.0357	1.8245

75	2015	16	16.7429	65.4592	777.7704	6.1684	2.5173
40	2015	16	21.1429	76.0000	949.7459	5.4133	2.1745
11	2015	16	21.0000	59.2449	880.3173	5.8408	2.4684
35	2015	16	20.1000	76.0102	943.4357	4.5204	1.7796
78	2015	16	20.9429	63.0816	902.3510	5.8163	2.2806
28	2015	16	21.1714	69.2551	932.6806	5.2704	2.1092
39	2015	16	19.4857	73.5102	905.4755	4.4582	3.3561
24	2015	16	20.7286	70.6531	943.8490	4.2582	1.7888
63	2015	16	21.1429	76.0000	949.7459	5.4133	2.1745
62	2015	16	19.7857	69.3673	877.1286	5.2398	1.6776
1	2015	16	20.0000	66.9388	877.6765	5.4867	3.1255
31	2015	17	12.6429	71.6633	849.5663	4.9714	1.1939
79	2015	17	17.6143	67.4490	974.1918	5.2653	1.6255
51	2015	17	14.9286	73.3776	943.5041	5.3082	1.5235
14	2015	17	14.8286	69.6633	901.2082	5.3173	2.5745
67	2015	17	13.8143	74.6531	906.0735	4.0694	3.2204
42	2015	17	13.3000	68.1633	878.4827	5.0796	2.7041
50	2015	17	15.0286	68.0408	905.5224	3.7082	1.8592
43	2015	17	13.3000	68.1633	878.4827	5.0796	2.7041
85	2015	17	15.5571	68.8673	914.2102	5.0949	1.6786
25	2015	17	17.9571	71.4388	981.5704	4.9612	1.1276
69	2015	17	15.9571	69.9286	944.1571	4.5224	1.5398
57	2015	17	13.5429	61.4898	890.4327	4.6092	2.9041
9	2015	17	13.2143	62.6837	857.5010	5.4765	2.7510
72	2015	17	14.8857	61.9082	881.1408	5.2071	2.3480
26	2015	17	15.6000	62.4694	870.5541	5.3796	2.1020
7	2015	17	14.6857	61.4694	862.5673	6.1786	2.4633
83	2015	17	19.8286	58.1122	948.1816	5.6796	1.2806
76	2015	17	15.0857	68.9694	925.0296	4.9184	1.5806
36	2015	17	15.0286	68.7755	933.0786	5.0357	1.8704
81	2015	17	14.9286	73.3776	943.5041	5.3082	1.5235
15	2015	17	16.4000	73.6429	937.2755	4.0816	1.1265
32	2015	17	13.3000	68.1633	878.4827	5.0796	2.7041
73	2015	17	17.0571	68.3367	966.2133	4.3378	1.2163
71	2015	17	15.0286	68.7755	933.0786	5.0357	1.8704
41	2015	17	14.1857	69.1020	877.9786	4.9980	1.6378
10	2015	17	16.1286	74.1327	968.8133	5.4857	1.0959
23	2015	17	9.4286	65.2245	778.7929	6.1735	2.4602
27	2015	17	14.6857	61.4694	862.5673	6.1786	2.4633
60	2015	17	14.9286	73.3776	943.5041	5.3082	1.5235
53	2015	17	13.2143	62.6837	857.5010	5.4765	2.7510
66	2015	17	14.8286	69.6633	901.2082	5.3173	2.5745
59	2015	17	13.5429	61.4898	890.4327	4.6092	2.9041
61	2015	17	17.0571	68.3367	966.2133	4.3378	1.2163
84	2015	17	17.0571	68.3367	966.2133	4.3378	1.2163
38	2015	17	13.5429	61.4898	890.4327	4.6092	2.9041
87	2015	17	15.7571	63.9082	903.1684	5.5347	2.0490
34	2015	17	13.5429	61.4898	890.4327	4.6092	2.9041
29	2015	17	15.9571	69.9286	944.1571	4.5224	1.5398
5	2015	17	12.7000	61.8571	835.4745	6.4816	2.4898

8	2015	17	13.2143	62.6837	857.5010	5.4765	2.7510
12	2015	17	12.7000	61.8571	835.4745	6.4816	2.4898
13	2015	17	19.8286	58.1122	948.1816	5.6796	1.2806
18	2015	17	17.6000	70.5204	968.4837	4.7378	1.1827
33	2015	17	15.0286	68.0408	905.5224	3.7082	1.8592
56	2015	17	17.9571	71.4388	981.5704	4.9612	1.1276
77	2015	17	16.4000	73.6429	937.2755	4.0816	1.1265
54	2015	17	12.7000	61.8571	835.4745	6.4816	2.4898
21	2015	17	15.0286	68.0408	905.5224	3.7082	1.8592
68	2015	17	17.6143	67.4490	974.1918	5.2653	1.6255
74	2015	17	17.0571	68.3367	966.2133	4.3378	1.2163
88	2015	17	13.3000	68.1633	878.4827	5.0796	2.7041
16	2015	17	15.0857	68.9694	925.0296	4.9184	1.5806
30	2015	17	14.8286	69.6633	901.2082	5.3173	2.5745
6	2015	17	17.6143	67.4490	974.1918	5.2653	1.6255
49	2015	17	15.9571	69.9286	944.1571	4.5224	1.5398
22	2015	17	13.3000	68.1633	878.4827	5.0796	2.7041
45	2015	17	12.6857	62.3776	820.6827	6.2612	2.2071
58	2015	17	15.9571	69.9286	944.1571	4.5224	1.5398
37	2015	17	17.6143	67.4490	974.1918	5.2653	1.6255
17	2015	17	13.8143	74.6531	906.0735	4.0694	3.2204
55	2015	17	14.8857	61.9082	881.1408	5.2071	2.3480
46	2015	17	15.0857	68.9694	925.0296	4.9184	1.5806
86	2015	17	14.0143	67.0918	869.6000	5.4520	0.9745
2	2015	17	14.0143	67.0918	869.6000	5.4520	0.9745
4	2015	17	15.0286	68.0408	905.5224	3.7082	1.8592
47	2015	17	20.3000	59.9082	962.4969	5.3000	0.8724
82	2015	17	13.3000	68.1633	878.4827	5.0796	2.7041
19	2015	17	19.0143	67.5000	964.8796	4.4398	1.5776
20	2015	17	13.2143	62.6837	857.5010	5.4765	2.7510
80	2015	17	13.3000	68.1633	878.4827	5.0796	2.7041
3	2015	17	19.8286	58.1122	948.1816	5.6796	1.2806
52	2015	17	13.8143	74.6531	906.0735	4.0694	3.2204
70	2015	17	15.5571	68.8673	914.2102	5.0949	1.6786
64	2015	17	9.4286	65.2245	778.7929	6.1735	2.4602
48	2015	17	16.4000	73.6429	937.2755	4.0816	1.1265
65	2015	17	13.8143	74.6531	906.0735	4.0694	3.2204
44	2015	17	15.5571	68.8673	914.2102	5.0949	1.6786
75	2015	17	9.4286	65.2245	778.7929	6.1735	2.4602
40	2015	17	15.1857	73.4286	949.8684	5.8612	1.9327
11	2015	17	14.8857	61.9082	881.1408	5.2071	2.3480
35	2015	17	14.9286	73.3776	943.5041	5.3082	1.5235
78	2015	17	15.7571	63.9082	903.1684	5.5347	2.0490
28	2015	17	15.0286	68.7755	933.0786	5.0357	1.8704
39	2015	17	13.8143	74.6531	906.0735	4.0694	3.2204
24	2015	17	15.9571	69.9286	944.1571	4.5224	1.5398
63	2015	17	15.1857	73.4286	949.8684	5.8612	1.9327
62	2015	17	14.1857	69.1020	877.9786	4.9980	1.6378
1	2015	17	13.3000	68.1633	878.4827	5.0796	2.7041
31	2015	18	16.9571	79.2857	849.0296	3.1020	1.0051

79	2015	18	22.4143	75.4796	972.6592	3.4398	1.3643
51	2015	18	20.5571	81.3367	942.3153	2.8510	1.2898
14	2015	18	20.4714	77.8980	900.2490	3.3847	2.2296
67	2015	18	19.6571	84.2959	905.1337	2.1378	2.6592
42	2015	18	19.4429	79.4898	877.7786	3.2898	2.2939
50	2015	18	19.4571	74.0408	904.6867	2.1041	1.5490
43	2015	18	19.4429	79.4898	877.7786	3.2898	2.2939
85	2015	18	20.0000	76.9592	913.3908	3.4388	1.5010
25	2015	18	23.5286	77.9694	980.1765	3.1724	1.0020
69	2015	18	20.8286	77.4796	943.1122	2.5143	1.4327
57	2015	18	18.4571	73.1837	889.7316	2.7010	2.3133
9	2015	18	19.2143	73.0102	856.8306	3.6276	2.2929
72	2015	18	20.9143	71.3469	880.3367	3.8704	2.0214
26	2015	18	21.5571	70.1122	869.7663	4.5000	1.7673
7	2015	18	20.6571	69.7449	861.8827	5.0265	2.0837
83	2015	18	25.3143	66.6327	947.0071	4.6551	1.2500
76	2015	18	19.9429	77.0714	924.1673	2.8276	1.3500
36	2015	18	20.7714	78.3163	932.0051	2.8194	1.5286
81	2015	18	20.5571	81.3367	942.3153	2.8510	1.2898
15	2015	18	20.7714	80.1429	936.2439	2.4837	0.9847
32	2015	18	19.4429	79.4898	877.7786	3.2898	2.2939
73	2015	18	21.5714	77.4388	965.1102	1.8827	1.1561
71	2015	18	20.7714	78.3163	932.0051	2.8194	1.5286
41	2015	18	18.5143	79.7347	877.3337	3.2765	1.3755
10	2015	18	21.7714	80.8673	967.3418	3.0969	0.9592
23	2015	18	14.3857	73.9082	778.4020	4.4102	2.2133
27	2015	18	20.6571	69.7449	861.8827	5.0265	2.0837
60	2015	18	20.5571	81.3367	942.3153	2.8510	1.2898
53	2015	18	19.2143	73.0102	856.8306	3.6276	2.2929
66	2015	18	20.4714	77.8980	900.2490	3.3847	2.2296
59	2015	18	18.4571	73.1837	889.7316	2.7010	2.3133
61	2015	18	21.5714	77.4388	965.1102	1.8827	1.1561
84	2015	18	21.5714	77.4388	965.1102	1.8827	1.1561
38	2015	18	18.4571	73.1837	889.7316	2.7010	2.3133
87	2015	18	21.8571	73.5714	902.2918	3.5245	1.7755
34	2015	18	18.4571	73.1837	889.7316	2.7010	2.3133
29	2015	18	20.8286	77.4796	943.1122	2.5143	1.4327
5	2015	18	18.6286	71.8469	834.8837	4.7082	2.1367
8	2015	18	19.2143	73.0102	856.8306	3.6276	2.2929
12	2015	18	18.6286	71.8469	834.8837	4.7082	2.1367
13	2015	18	25.3143	66.6327	947.0071	4.6551	1.2500
18	2015	18	21.8000	76.6327	966.9908	2.6786	1.1173
33	2015	18	19.4571	74.0408	904.6867	2.1041	1.5490
56	2015	18	23.5286	77.9694	980.1765	3.1724	1.0020
77	2015	18	20.7714	80.1429	936.2439	2.4837	0.9847
54	2015	18	18.6286	71.8469	834.8837	4.7082	2.1367
21	2015	18	19.4571	74.0408	904.6867	2.1041	1.5490
68	2015	18	22.4143	75.4796	972.6592	3.4398	1.3643
74	2015	18	21.5714	77.4388	965.1102	1.8827	1.1561
88	2015	18	19.4429	79.4898	877.7786	3.2898	2.2939

16	2015	18	19.9429	77.0714	924.1673	2.8276	1.3500
30	2015	18	20.4714	77.8980	900.2490	3.3847	2.2296
6	2015	18	22.4143	75.4796	972.6592	3.4398	1.3643
49	2015	18	20.8286	77.4796	943.1122	2.5143	1.4327
22	2015	18	19.4429	79.4898	877.7786	3.2898	2.2939
45	2015	18	18.8286	68.9592	820.0827	4.6929	1.8980
58	2015	18	20.8286	77.4796	943.1122	2.5143	1.4327
37	2015	18	22.4143	75.4796	972.6592	3.4398	1.3643
17	2015	18	19.6571	84.2959	905.1337	2.1378	2.6592
55	2015	18	20.9143	71.3469	880.3367	3.8704	2.0214
46	2015	18	19.9429	77.0714	924.1673	2.8276	1.3500
86	2015	18	19.0286	76.0714	868.9265	3.5184	0.8082
2	2015	18	19.0286	76.0714	868.9265	3.5184	0.8082
4	2015	18	19.4571	74.0408	904.6867	2.1041	1.5490
47	2015	18	26.1857	66.6224	961.1898	4.0214	0.7367
82	2015	18	19.4429	79.4898	877.7786	3.2898	2.2939
19	2015	18	24.3571	72.0204	963.4388	3.1173	1.4204
20	2015	18	19.2143	73.0102	856.8306	3.6276	2.2929
80	2015	18	19.4429	79.4898	877.7786	3.2898	2.2939
3	2015	18	25.3143	66.6327	947.0071	4.6551	1.2500
52	2015	18	19.6571	84.2959	905.1337	2.1378	2.6592
70	2015	18	20.0000	76.9592	913.3908	3.4388	1.5010
64	2015	18	14.3857	73.9082	778.4020	4.4102	2.2133
48	2015	18	20.7714	80.1429	936.2439	2.4837	0.9847
65	2015	18	19.6571	84.2959	905.1337	2.1378	2.6592
44	2015	18	20.0000	76.9592	913.3908	3.4388	1.5010
75	2015	18	14.3857	73.9082	778.4020	4.4102	2.2133
40	2015	18	21.7143	79.9388	948.5490	3.5551	1.4908
11	2015	18	20.9143	71.3469	880.3367	3.8704	2.0214
35	2015	18	20.5571	81.3367	942.3153	2.8510	1.2898
78	2015	18	21.8571	73.5714	902.2918	3.5245	1.7755
28	2015	18	20.7714	78.3163	932.0051	2.8194	1.5286
39	2015	18	19.6571	84.2959	905.1337	2.1378	2.6592
24	2015	18	20.8286	77.4796	943.1122	2.5143	1.4327
63	2015	18	21.7143	79.9388	948.5490	3.5551	1.4908
62	2015	18	18.5143	79.7347	877.3337	3.2765	1.3755
1	2015	18	19.4429	79.4898	877.7786	3.2898	2.2939
31	2015	19	16.6429	81.0102	848.2143	3.0245	0.9786
79	2015	19	20.9143	78.6020	970.9327	3.7439	1.4184
51	2015	19	19.7000	83.3776	940.7347	2.9602	1.2847
14	2015	19	19.3571	82.4796	898.8786	3.2010	2.0786
67	2015	19	18.6857	87.4286	903.7888	2.2031	2.1286
42	2015	19	18.0286	83.2857	876.6388	3.5347	2.1939
50	2015	19	19.2714	76.3367	903.4531	2.9663	1.3949
43	2015	19	18.0286	83.2857	876.6388	3.5347	2.1939
85	2015	19	19.9429	78.8776	912.1510	3.6765	1.2235
25	2015	19	22.7571	81.9184	978.2878	3.5898	0.8612
69	2015	19	20.8714	80.3571	941.5857	3.2867	1.5969
57	2015	19	18.0857	77.1633	888.6000	3.4439	2.0888
9	2015	19	18.0571	76.7449	855.8041	3.8653	1.9827

72	2015	19	19.8000	75.5612	879.1531	4.5408	1.7194
26	2015	19	21.6286	72.7755	868.3888	6.1755	1.6173
7	2015	19	21.0286	73.1837	860.6571	5.9480	1.9122
83	2015	19	25.5000	69.4694	945.1786	5.1010	1.2286
76	2015	19	19.6000	78.9592	922.8010	3.5306	1.4102
36	2015	19	20.0571	82.5510	930.7061	3.3745	1.2990
81	2015	19	19.7000	83.3776	940.7347	2.9602	1.2847
15	2015	19	19.7714	81.7551	934.9031	3.3857	0.7918
32	2015	19	18.0286	83.2857	876.6388	3.5347	2.1939
73	2015	19	21.5857	78.5612	963.5143	2.6010	1.0592
71	2015	19	20.0571	82.5510	930.7061	3.3745	1.2990
41	2015	19	18.5000	83.4694	876.3112	3.6551	1.2020
10	2015	19	20.6000	84.3776	965.5735	3.0847	0.8245
23	2015	19	14.5714	76.7041	777.7520	4.8755	2.2031
27	2015	19	21.0286	73.1837	860.6571	5.9480	1.9122
60	2015	19	19.7000	83.3776	940.7347	2.9602	1.2847
53	2015	19	18.0571	76.7449	855.8041	3.8653	1.9827
66	2015	19	19.3571	82.4796	898.8786	3.2010	2.0786
59	2015	19	18.0857	77.1633	888.6000	3.4439	2.0888
61	2015	19	21.5857	78.5612	963.5143	2.6010	1.0592
84	2015	19	21.5857	78.5612	963.5143	2.6010	1.0592
38	2015	19	18.0857	77.1633	888.6000	3.4439	2.0888
87	2015	19	20.1714	77.4592	900.9378	3.6653	1.7061
34	2015	19	18.0857	77.1633	888.6000	3.4439	2.0888
29	2015	19	20.8714	80.3571	941.5857	3.2867	1.5969
5	2015	19	19.0286	74.7551	833.8622	5.8561	1.9337
8	2015	19	18.0571	76.7449	855.8041	3.8653	1.9827
12	2015	19	19.0286	74.7551	833.8622	5.8561	1.9337
13	2015	19	25.5000	69.4694	945.1786	5.1010	1.2286
18	2015	19	20.3143	81.0408	965.4286	3.1694	1.0071
33	2015	19	19.2714	76.3367	903.4531	2.9663	1.3949
56	2015	19	22.7571	81.9184	978.2878	3.5898	0.8612
77	2015	19	19.7714	81.7551	934.9031	3.3857	0.7918
54	2015	19	19.0286	74.7551	833.8622	5.8561	1.9337
21	2015	19	19.2714	76.3367	903.4531	2.9663	1.3949
68	2015	19	20.9143	78.6020	970.9327	3.7439	1.4184
74	2015	19	21.5857	78.5612	963.5143	2.6010	1.0592
88	2015	19	18.0286	83.2857	876.6388	3.5347	2.1939
16	2015	19	19.6000	78.9592	922.8010	3.5306	1.4102
30	2015	19	19.3571	82.4796	898.8786	3.2010	2.0786
6	2015	19	20.9143	78.6020	970.9327	3.7439	1.4184
49	2015	19	20.8714	80.3571	941.5857	3.2867	1.5969
22	2015	19	18.0286	83.2857	876.6388	3.5347	2.1939
45	2015	19	19.7000	68.4592	818.9653	5.7439	1.5296
58	2015	19	20.8714	80.3571	941.5857	3.2867	1.5969
37	2015	19	20.9143	78.6020	970.9327	3.7439	1.4184
17	2015	19	18.6857	87.4286	903.7888	2.2031	2.1286
55	2015	19	19.8000	75.5612	879.1531	4.5408	1.7194
46	2015	19	19.6000	78.9592	922.8010	3.5306	1.4102
86	2015	19	18.3429	78.2857	867.8929	4.0337	0.7980

2	2015	19	18.3429	78.2857	867.8929	4.0337	0.7980
4	2015	19	19.2714	76.3367	903.4531	2.9663	1.3949
47	2015	19	24.6714	70.2959	959.4031	4.0939	0.6949
82	2015	19	18.0286	83.2857	876.6388	3.5347	2.1939
19	2015	19	23.2000	76.8265	961.5653	3.5010	1.1724
20	2015	19	18.0571	76.7449	855.8041	3.8653	1.9827
80	2015	19	18.0286	83.2857	876.6388	3.5347	2.1939
3	2015	19	25.5000	69.4694	945.1786	5.1010	1.2286
52	2015	19	18.6857	87.4286	903.7888	2.2031	2.1286
70	2015	19	19.9429	78.8776	912.1510	3.6765	1.2235
64	2015	19	14.5714	76.7041	777.7520	4.8755	2.2031
48	2015	19	19.7714	81.7551	934.9031	3.3857	0.7918
65	2015	19	18.6857	87.4286	903.7888	2.2031	2.1286
44	2015	19	19.9429	78.8776	912.1510	3.6765	1.2235
75	2015	19	14.5714	76.7041	777.7520	4.8755	2.2031
40	2015	19	19.9143	84.5714	946.9163	3.1694	1.1184
11	2015	19	19.8000	75.5612	879.1531	4.5408	1.7194
35	2015	19	19.7000	83.3776	940.7347	2.9602	1.2847
78	2015	19	20.1714	77.4592	900.9378	3.6653	1.7061
28	2015	19	20.0571	82.5510	930.7061	3.3745	1.2990
39	2015	19	18.6857	87.4286	903.7888	2.2031	2.1286
24	2015	19	20.8714	80.3571	941.5857	3.2867	1.5969
63	2015	19	19.9143	84.5714	946.9163	3.1694	1.1184
62	2015	19	18.5000	83.4694	876.3112	3.6551	1.2020
1	2015	19	18.0286	83.2857	876.6388	3.5347	2.1939
31	2015	20	18.3571	76.6531	846.2102	4.0816	1.1398
79	2015	20	21.0714	79.2857	968.7561	3.7776	1.7276
51	2015	20	20.5571	80.7653	938.4031	3.6765	1.5245
14	2015	20	20.7286	83.0408	896.8327	3.8276	2.6102
67	2015	20	20.3286	86.6735	901.6235	2.3459	2.8888
42	2015	20	19.8429	81.9082	874.5439	4.1663	2.6490
50	2015	20	19.6714	74.0816	901.1204	3.9602	1.6429
43	2015	20	19.8429	81.9082	874.5439	4.1663	2.6490
85	2015	20	20.7714	73.8163	909.6867	5.1816	1.5643
25	2015	20	23.4143	81.5816	975.7214	4.2327	0.8204
69	2015	20	21.3143	78.5306	939.0306	3.7194	1.6378
57	2015	20	19.6714	72.7551	886.3439	3.8990	2.8265
9	2015	20	19.7714	73.8571	853.8704	5.0949	2.6276
72	2015	20	22.0571	72.8061	877.0816	6.1418	2.3612
26	2015	20	22.8857	68.3776	866.2755	7.4276	2.1398
7	2015	20	22.3571	68.1633	858.5918	6.9510	2.4020
83	2015	20	26.6000	65.5714	942.5837	6.7918	1.2867
76	2015	20	20.8714	76.3469	920.3092	3.8071	1.6439
36	2015	20	21.6857	80.0918	928.3765	4.1276	1.4480
81	2015	20	20.5571	80.7653	938.4031	3.6765	1.5245
15	2015	20	20.8429	81.3673	932.4765	3.5694	0.8847
32	2015	20	19.8429	81.9082	874.5439	4.1663	2.6490
73	2015	20	22.1714	75.4796	960.8602	3.1439	1.1245
71	2015	20	21.6857	80.0918	928.3765	4.1276	1.4480
41	2015	20	20.0429	78.5510	874.1735	4.3255	1.4398

10	2015	20	21.4571	83.7245	963.2143	3.6816	1.0327
23	2015	20	16.6857	71.6735	776.2316	6.3776	2.7510
27	2015	20	22.3571	68.1633	858.5918	6.9510	2.4020
60	2015	20	20.5571	80.7653	938.4031	3.6765	1.5245
53	2015	20	19.7714	73.8571	853.8704	5.0949	2.6276
66	2015	20	20.7286	83.0408	896.8327	3.8276	2.6102
59	2015	20	19.6714	72.7551	886.3439	3.8990	2.8265
61	2015	20	22.1714	75.4796	960.8602	3.1439	1.1245
84	2015	20	22.1714	75.4796	960.8602	3.1439	1.1245
38	2015	20	19.6714	72.7551	886.3439	3.8990	2.8265
87	2015	20	22.4143	76.1020	898.7327	4.5582	1.9735
34	2015	20	19.6714	72.7551	886.3439	3.8990	2.8265
29	2015	20	21.3143	78.5306	939.0306	3.7194	1.6378
5	2015	20	20.8143	68.1327	831.9500	7.1704	2.3367
8	2015	20	19.7714	73.8571	853.8704	5.0949	2.6276
12	2015	20	20.8143	68.1327	831.9500	7.1704	2.3367
13	2015	20	26.6000	65.5714	942.5837	6.7918	1.2867
18	2015	20	20.5857	83.0000	963.2878	3.0714	1.0561
33	2015	20	19.6714	74.0816	901.1204	3.9602	1.6429
56	2015	20	23.4143	81.5816	975.7214	4.2327	0.8204
77	2015	20	20.8429	81.3673	932.4765	3.5694	0.8847
54	2015	20	20.8143	68.1327	831.9500	7.1704	2.3367
21	2015	20	19.6714	74.0816	901.1204	3.9602	1.6429
68	2015	20	21.0714	79.2857	968.7561	3.7776	1.7276
74	2015	20	22.1714	75.4796	960.8602	3.1439	1.1245
88	2015	20	19.8429	81.9082	874.5439	4.1663	2.6490
16	2015	20	20.8714	76.3469	920.3092	3.8071	1.6439
30	2015	20	20.7286	83.0408	896.8327	3.8276	2.6102
6	2015	20	21.0714	79.2857	968.7561	3.7776	1.7276
49	2015	20	21.3143	78.5306	939.0306	3.7194	1.6378
22	2015	20	19.8429	81.9082	874.5439	4.1663	2.6490
45	2015	20	20.1714	63.3469	817.1633	7.3163	1.9357
58	2015	20	21.3143	78.5306	939.0306	3.7194	1.6378
37	2015	20	21.0714	79.2857	968.7561	3.7776	1.7276
17	2015	20	20.3286	86.6735	901.6235	2.3459	2.8888
55	2015	20	22.0571	72.8061	877.0816	6.1418	2.3612
46	2015	20	20.8714	76.3469	920.3092	3.8071	1.6439
86	2015	20	20.2143	73.8776	865.8245	4.9000	0.9908
2	2015	20	20.2143	73.8776	865.8245	4.9000	0.9908
4	2015	20	19.6714	74.0816	901.1204	3.9602	1.6429
47	2015	20	25.8286	70.6327	956.9531	5.4469	0.8092
82	2015	20	19.8429	81.9082	874.5439	4.1663	2.6490
19	2015	20	24.0571	78.9184	959.1888	3.9959	1.2378
20	2015	20	19.7714	73.8571	853.8704	5.0949	2.6276
80	2015	20	19.8429	81.9082	874.5439	4.1663	2.6490
3	2015	20	26.6000	65.5714	942.5837	6.7918	1.2867
52	2015	20	20.3286	86.6735	901.6235	2.3459	2.8888
70	2015	20	20.7714	73.8163	909.6867	5.1816	1.5643
64	2015	20	16.6857	71.6735	776.2316	6.3776	2.7510
48	2015	20	20.8429	81.3673	932.4765	3.5694	0.8847

65	2015	20	20.3286	86.6735	901.6235	2.3459	2.8888
44	2015	20	20.7714	73.8163	909.6867	5.1816	1.5643
75	2015	20	16.6857	71.6735	776.2316	6.3776	2.7510
40	2015	20	21.6571	84.3265	944.6367	3.7276	1.4959
11	2015	20	22.0571	72.8061	877.0816	6.1418	2.3612
35	2015	20	20.5571	80.7653	938.4031	3.6765	1.5245
78	2015	20	22.4143	76.1020	898.7327	4.5582	1.9735
28	2015	20	21.6857	80.0918	928.3765	4.1276	1.4480
39	2015	20	20.3286	86.6735	901.6235	2.3459	2.8888
24	2015	20	21.3143	78.5306	939.0306	3.7194	1.6378
63	2015	20	21.6571	84.3265	944.6367	3.7276	1.4959
62	2015	20	20.0429	78.5510	874.1735	4.3255	1.4398
1	2015	20	19.8429	81.9082	874.5439	4.1663	2.6490
31	2015	21	18.1286	77.4898	845.7082	3.5041	1.0663
79	2015	21	22.0714	81.1224	967.9173	2.7541	1.4929
51	2015	21	20.3714	82.5000	937.6296	2.4000	1.3316
14	2015	21	20.0286	84.4694	896.2806	3.0980	2.7214
67	2015	21	19.6286	88.6224	901.0000	1.6224	3.0031
42	2015	21	18.7571	84.5204	874.0816	3.2316	2.7408
50	2015	21	19.9000	76.9184	900.4837	2.7408	1.5418
43	2015	21	18.7571	84.5204	874.0816	3.2316	2.7408
85	2015	21	20.5286	76.3878	909.0153	4.1888	1.5857
25	2015	21	23.0857	83.8265	974.9235	3.0408	0.7500
69	2015	21	21.7714	81.1122	938.2439	2.4561	1.3000
57	2015	21	18.7143	74.6837	885.7592	2.7327	2.8694
9	2015	21	18.7286	76.8878	853.4571	4.3429	2.6010
72	2015	21	20.2429	74.7041	876.5633	5.4122	2.5796
26	2015	21	21.1429	75.8878	865.9143	5.7214	2.1102
7	2015	21	20.5429	72.5000	858.2184	5.0969	2.2673
83	2015	21	24.5000	71.7551	942.0684	6.3214	1.1500
76	2015	21	20.6714	78.9490	919.6153	2.7806	1.6031
36	2015	21	20.7857	80.9184	927.4612	2.9510	1.4296
81	2015	21	20.3714	82.5000	937.6296	2.4000	1.3316
15	2015	21	21.5857	82.9490	931.6776	2.2357	0.8051
32	2015	21	18.7571	84.5204	874.0816	3.2316	2.7408
73	2015	21	22.6286	77.7551	960.0224	2.0878	1.0745
71	2015	21	20.7857	80.9184	927.4612	2.9510	1.4296
41	2015	21	19.2857	81.4796	873.6143	3.3408	1.3745
10	2015	21	21.3714	85.3673	962.3296	2.6112	0.9500
23	2015	21	15.3429	72.0714	776.1184	5.2816	2.7235
27	2015	21	20.5429	72.5000	858.2184	5.0969	2.2673
60	2015	21	20.3714	82.5000	937.6296	2.4000	1.3316
53	2015	21	18.7286	76.8878	853.4571	4.3429	2.6010
66	2015	21	20.0286	84.4694	896.2806	3.0980	2.7214
59	2015	21	18.7143	74.6837	885.7592	2.7327	2.8694
61	2015	21	22.6286	77.7551	960.0224	2.0878	1.0745
84	2015	21	22.6286	77.7551	960.0224	2.0878	1.0745
38	2015	21	18.7143	74.6837	885.7592	2.7327	2.8694
87	2015	21	20.8143	78.6327	898.1929	4.1133	1.9561
34	2015	21	18.7143	74.6837	885.7592	2.7327	2.8694

29	2015	21	21.7714	81.1122	938.2439	2.4561	1.3000
5	2015	21	18.5714	72.4898	831.6449	5.6306	2.1765
8	2015	21	18.7286	76.8878	853.4571	4.3429	2.6010
12	2015	21	18.5714	72.4898	831.6449	5.6306	2.1765
13	2015	21	24.5000	71.7551	942.0684	6.3214	1.1500
18	2015	21	21.9286	83.7653	962.4510	2.3541	1.0908
33	2015	21	19.9000	76.9184	900.4837	2.7408	1.5418
56	2015	21	23.0857	83.8265	974.9235	3.0408	0.7500
77	2015	21	21.5857	82.9490	931.6776	2.2357	0.8051
54	2015	21	18.5714	72.4898	831.6449	5.6306	2.1765
21	2015	21	19.9000	76.9184	900.4837	2.7408	1.5418
68	2015	21	22.0714	81.1224	967.9173	2.7541	1.4929
74	2015	21	22.6286	77.7551	960.0224	2.0878	1.0745
88	2015	21	18.7571	84.5204	874.0816	3.2316	2.7408
16	2015	21	20.6714	78.9490	919.6153	2.7806	1.6031
30	2015	21	20.0286	84.4694	896.2806	3.0980	2.7214
6	2015	21	22.0714	81.1224	967.9173	2.7541	1.4929
49	2015	21	21.7714	81.1122	938.2439	2.4561	1.3000
22	2015	21	18.7571	84.5204	874.0816	3.2316	2.7408
45	2015	21	19.0000	70.9694	816.9551	5.8551	1.7571
58	2015	21	21.7714	81.1122	938.2439	2.4561	1.3000
37	2015	21	22.0714	81.1224	967.9173	2.7541	1.4929
17	2015	21	19.6286	88.6224	901.0000	1.6224	3.0031
55	2015	21	20.2429	74.7041	876.5633	5.4122	2.5796
46	2015	21	20.6714	78.9490	919.6153	2.7806	1.6031
86	2015	21	19.4000	76.2449	865.2969	3.8867	0.9837
2	2015	21	19.4000	76.2449	865.2969	3.8867	0.9837
4	2015	21	19.9000	76.9184	900.4837	2.7408	1.5418
47	2015	21	24.6000	74.3878	956.3276	4.3541	0.8184
82	2015	21	18.7571	84.5204	874.0816	3.2316	2.7408
19	2015	21	23.8714	80.8163	958.5327	3.2388	1.1173
20	2015	21	18.7286	76.8878	853.4571	4.3429	2.6010
80	2015	21	18.7571	84.5204	874.0816	3.2316	2.7408
3	2015	21	24.5000	71.7551	942.0684	6.3214	1.1500
52	2015	21	19.6286	88.6224	901.0000	1.6224	3.0031
70	2015	21	20.5286	76.3878	909.0153	4.1888	1.5857
64	2015	21	15.3429	72.0714	776.1184	5.2816	2.7235
48	2015	21	21.5857	82.9490	931.6776	2.2357	0.8051
65	2015	21	19.6286	88.6224	901.0000	1.6224	3.0031
44	2015	21	20.5286	76.3878	909.0153	4.1888	1.5857
75	2015	21	15.3429	72.0714	776.1184	5.2816	2.7235
40	2015	21	20.5143	85.1429	943.8571	2.8939	1.6071
11	2015	21	20.2429	74.7041	876.5633	5.4122	2.5796
35	2015	21	20.3714	82.5000	937.6296	2.4000	1.3316
78	2015	21	20.8143	78.6327	898.1929	4.1133	1.9561
28	2015	21	20.7857	80.9184	927.4612	2.9510	1.4296
39	2015	21	19.6286	88.6224	901.0000	1.6224	3.0031
24	2015	21	21.7714	81.1122	938.2439	2.4561	1.3000
63	2015	21	20.5143	85.1429	943.8571	2.8939	1.6071
62	2015	21	19.2857	81.4796	873.6143	3.3408	1.3745

1	2015	21	18.7571	84.5204	874.0816	3.2316	2.7408
31	2015	22	18.9571	81.8367	845.0857	1.7347	0.9704
79	2015	22	22.6714	82.1224	966.9408	1.6551	1.3878
51	2015	22	22.5429	85.0816	936.6929	1.6480	1.4061
14	2015	22	23.3286	86.0204	895.2041	2.3041	2.6347
67	2015	22	22.2714	91.1837	900.0173	1.0194	2.6041
42	2015	22	21.3857	87.5510	873.2306	2.1551	2.5398
50	2015	22	20.7143	82.1837	899.7439	0.9031	1.3847
43	2015	22	21.3857	87.5510	873.2306	2.1551	2.5398
85	2015	22	20.8857	82.5816	908.3582	1.6990	1.4214
25	2015	22	26.3857	86.5510	973.5867	2.7949	0.7561
69	2015	22	23.2714	82.7347	937.3020	1.9398	1.2051
57	2015	22	20.0286	80.2755	884.9612	1.8806	2.2582
9	2015	22	21.4857	80.8878	852.5724	3.1612	2.2714
72	2015	22	23.5571	78.4286	875.5765	4.5867	2.1184
26	2015	22	24.5429	80.9898	864.9541	5.6439	1.9735
7	2015	22	24.0286	76.9490	857.3143	4.7143	2.1092
83	2015	22	28.1857	76.1429	940.9071	5.7582	1.0663
76	2015	22	21.7714	83.6837	918.8663	1.9561	1.5582
36	2015	22	23.6286	83.0204	926.3929	1.9000	1.3878
81	2015	22	22.5429	85.0816	936.6929	1.6480	1.4061
15	2015	22	21.7571	87.2959	930.9051	0.9255	0.7194
32	2015	22	21.3857	87.5510	873.2306	2.1551	2.5398
73	2015	22	23.4429	80.2143	959.2520	1.2408	0.9724
71	2015	22	23.6286	83.0204	926.3929	1.9000	1.3878
41	2015	22	20.8714	86.0612	872.8133	2.0724	1.1316
10	2015	22	23.5000	87.7857	961.2439	1.5684	0.9224
23	2015	22	18.0857	76.7857	775.4806	4.8337	2.5633
27	2015	22	24.0286	76.9490	857.3143	4.7143	2.1092
60	2015	22	22.5429	85.0816	936.6929	1.6480	1.4061
53	2015	22	21.4857	80.8878	852.5724	3.1612	2.2714
66	2015	22	23.3286	86.0204	895.2041	2.3041	2.6347
59	2015	22	20.0286	80.2755	884.9612	1.8806	2.2582
61	2015	22	23.4429	80.2143	959.2520	1.2408	0.9724
84	2015	22	23.4429	80.2143	959.2520	1.2408	0.9724
38	2015	22	20.0286	80.2755	884.9612	1.8806	2.2582
87	2015	22	24.1429	81.0714	897.1786	3.7378	1.8133
34	2015	22	20.0286	80.2755	884.9612	1.8806	2.2582
29	2015	22	23.2714	82.7347	937.3020	1.9398	1.2051
5	2015	22	22.3286	78.3980	830.8388	5.5837	2.0184
8	2015	22	21.4857	80.8878	852.5724	3.1612	2.2714
12	2015	22	22.3286	78.3980	830.8388	5.5837	2.0184
13	2015	22	28.1857	76.1429	940.9071	5.7582	1.0663
18	2015	22	22.0857	84.6429	961.5837	1.4888	1.0755
33	2015	22	20.7143	82.1837	899.7439	0.9031	1.3847
56	2015	22	26.3857	86.5510	973.5867	2.7949	0.7561
77	2015	22	21.7571	87.2959	930.9051	0.9255	0.7194
54	2015	22	22.3286	78.3980	830.8388	5.5837	2.0184
21	2015	22	20.7143	82.1837	899.7439	0.9031	1.3847
68	2015	22	22.6714	82.1224	966.9408	1.6551	1.3878

74	2015	22	23.4429	80.2143	959.2520	1.2408	0.9724
88	2015	22	21.3857	87.5510	873.2306	2.1551	2.5398
16	2015	22	21.7714	83.6837	918.8663	1.9561	1.5582
30	2015	22	23.3286	86.0204	895.2041	2.3041	2.6347
6	2015	22	22.6714	82.1224	966.9408	1.6551	1.3878
49	2015	22	23.2714	82.7347	937.3020	1.9398	1.2051
22	2015	22	21.3857	87.5510	873.2306	2.1551	2.5398
45	2015	22	22.1571	75.1531	816.1388	5.8684	1.5980
58	2015	22	23.2714	82.7347	937.3020	1.9398	1.2051
37	2015	22	22.6714	82.1224	966.9408	1.6551	1.3878
17	2015	22	22.2714	91.1837	900.0173	1.0194	2.6041
55	2015	22	23.5571	78.4286	875.5765	4.5867	2.1184
46	2015	22	21.7714	83.6837	918.8663	1.9561	1.5582
86	2015	22	21.2571	80.4286	864.4612	2.7551	0.8490
2	2015	22	21.2571	80.4286	864.4612	2.7551	0.8490
4	2015	22	20.7143	82.1837	899.7439	0.9031	1.3847
47	2015	22	28.1143	77.3061	955.0276	3.1918	0.7602
82	2015	22	21.3857	87.5510	873.2306	2.1551	2.5398
19	2015	22	26.7143	82.4796	957.2153	2.2510	0.9673
20	2015	22	21.4857	80.8878	852.5724	3.1612	2.2714
80	2015	22	21.3857	87.5510	873.2306	2.1551	2.5398
3	2015	22	28.1857	76.1429	940.9071	5.7582	1.0663
52	2015	22	22.2714	91.1837	900.0173	1.0194	2.6041
70	2015	22	20.8857	82.5816	908.3582	1.6990	1.4214
64	2015	22	18.0857	76.7857	775.4806	4.8337	2.5633
48	2015	22	21.7571	87.2959	930.9051	0.9255	0.7194
65	2015	22	22.2714	91.1837	900.0173	1.0194	2.6041
44	2015	22	20.8857	82.5816	908.3582	1.6990	1.4214
75	2015	22	18.0857	76.7857	775.4806	4.8337	2.5633
40	2015	22	24.0857	88.1122	942.6969	2.2633	1.4776
11	2015	22	23.5571	78.4286	875.5765	4.5867	2.1184
35	2015	22	22.5429	85.0816	936.6929	1.6480	1.4061
78	2015	22	24.1429	81.0714	897.1786	3.7378	1.8133
28	2015	22	23.6286	83.0204	926.3929	1.9000	1.3878
39	2015	22	22.2714	91.1837	900.0173	1.0194	2.6041
24	2015	22	23.2714	82.7347	937.3020	1.9398	1.2051
63	2015	22	24.0857	88.1122	942.6969	2.2633	1.4776
62	2015	22	20.8714	86.0612	872.8133	2.0724	1.1316
1	2015	22	21.3857	87.5510	873.2306	2.1551	2.5398
31	2015	23	18.6857	85.0306	844.5776	1.3918	0.9541
79	2015	23	23.5000	84.2449	965.9796	1.7102	1.5429
51	2015	23	23.2143	84.6122	935.7878	2.5398	1.5898
14	2015	23	23.1714	84.7653	894.5622	2.5806	2.9643
67	2015	23	22.2429	91.4898	899.2867	1.0204	2.8480
42	2015	23	21.0429	88.6939	872.5857	2.5949	2.4633
50	2015	23	20.6143	84.4388	899.0133	0.5286	1.3653
43	2015	23	21.0429	88.6939	872.5857	2.5949	2.4633
85	2015	23	21.2429	84.0918	907.7173	1.1500	1.4153
25	2015	23	27.0143	83.2041	972.4796	4.6776	1.1255
69	2015	23	23.7000	83.2755	936.3296	2.1510	1.3020

57	2015	23	20.1571	82.3367	884.3031	1.7378	2.1327
9	2015	23	20.6143	82.3469	851.9990	2.7898	2.3367
72	2015	23	22.5143	79.4184	875.0204	4.6276	2.0490
26	2015	23	23.6714	79.8367	864.4122	6.8163	2.0857
7	2015	23	23.1429	76.4796	856.7786	5.9949	2.3051
83	2015	23	27.6286	74.8061	940.1092	6.7602	1.1776
76	2015	23	21.8286	85.1735	917.9592	1.6520	1.5194
36	2015	23	23.7000	82.6837	925.5112	2.1051	1.3827
81	2015	23	23.2143	84.6122	935.7878	2.5398	1.5898
15	2015	23	21.6857	89.3469	930.0082	0.6908	0.7061
32	2015	23	21.0429	88.6939	872.5857	2.5949	2.4633
73	2015	23	23.1143	82.3571	958.2480	1.3418	0.9939
71	2015	23	23.7000	82.6837	925.5112	2.1051	1.3827
41	2015	23	20.7857	87.7959	872.1724	1.7378	1.0469
10	2015	23	24.6143	87.9796	960.2847	2.4388	1.1071
23	2015	23	16.6857	80.6122	775.0449	4.8286	2.5469
27	2015	23	23.1429	76.4796	856.7786	5.9949	2.3051
60	2015	23	23.2143	84.6122	935.7878	2.5398	1.5898
53	2015	23	20.6143	82.3469	851.9990	2.7898	2.3367
66	2015	23	23.1714	84.7653	894.5622	2.5806	2.9643
59	2015	23	20.1571	82.3367	884.3031	1.7378	2.1327
61	2015	23	23.1143	82.3571	958.2480	1.3418	0.9939
84	2015	23	23.1143	82.3571	958.2480	1.3418	0.9939
38	2015	23	20.1571	82.3367	884.3031	1.7378	2.1327
87	2015	23	23.3143	82.0000	896.4582	3.8969	1.8143
34	2015	23	20.1571	82.3367	884.3031	1.7378	2.1327
29	2015	23	23.7000	83.2755	936.3296	2.1510	1.3020
5	2015	23	20.9571	79.1020	830.3204	6.7245	1.9643
8	2015	23	20.6143	82.3469	851.9990	2.7898	2.3367
12	2015	23	20.9571	79.1020	830.3204	6.7245	1.9643
13	2015	23	27.6286	74.8061	940.1092	6.7602	1.1776
18	2015	23	22.7000	86.8163	960.6663	1.0969	0.9969
33	2015	23	20.6143	84.4388	899.0133	0.5286	1.3653
56	2015	23	27.0143	83.2041	972.4796	4.6776	1.1255
77	2015	23	21.6857	89.3469	930.0082	0.6908	0.7061
54	2015	23	20.9571	79.1020	830.3204	6.7245	1.9643
21	2015	23	20.6143	84.4388	899.0133	0.5286	1.3653
68	2015	23	23.5000	84.2449	965.9796	1.7102	1.5429
74	2015	23	23.1143	82.3571	958.2480	1.3418	0.9939
88	2015	23	21.0429	88.6939	872.5857	2.5949	2.4633
16	2015	23	21.8286	85.1735	917.9592	1.6520	1.5194
30	2015	23	23.1714	84.7653	894.5622	2.5806	2.9643
6	2015	23	23.5000	84.2449	965.9796	1.7102	1.5429
49	2015	23	23.7000	83.2755	936.3296	2.1510	1.3020
22	2015	23	21.0429	88.6939	872.5857	2.5949	2.4633
45	2015	23	21.7571	72.5510	815.6388	7.3010	1.9469
58	2015	23	23.7000	83.2755	936.3296	2.1510	1.3020
37	2015	23	23.5000	84.2449	965.9796	1.7102	1.5429
17	2015	23	22.2429	91.4898	899.2867	1.0204	2.8480
55	2015	23	22.5143	79.4184	875.0204	4.6276	2.0490

46	2015	23	21.8286	85.1735	917.9592	1.6520	1.5194
86	2015	23	20.8286	82.0918	863.8469	2.6276	0.8255
2	2015	23	20.8286	82.0918	863.8469	2.6276	0.8255
4	2015	23	20.6143	84.4388	899.0133	0.5286	1.3653
47	2015	23	26.7714	77.9082	954.1204	4.1224	0.6939
82	2015	23	21.0429	88.6939	872.5857	2.5949	2.4633
19	2015	23	26.8714	80.8265	956.3041	2.4653	1.1214
20	2015	23	20.6143	82.3469	851.9990	2.7898	2.3367
80	2015	23	21.0429	88.6939	872.5857	2.5949	2.4633
3	2015	23	27.6286	74.8061	940.1092	6.7602	1.1776
52	2015	23	22.2429	91.4898	899.2867	1.0204	2.8480
70	2015	23	21.2429	84.0918	907.7173	1.1500	1.4153
64	2015	23	16.6857	80.6122	775.0449	4.8286	2.5469
48	2015	23	21.6857	89.3469	930.0082	0.6908	0.7061
65	2015	23	22.2429	91.4898	899.2867	1.0204	2.8480
44	2015	23	21.2429	84.0918	907.7173	1.1500	1.4153
75	2015	23	16.6857	80.6122	775.0449	4.8286	2.5469
40	2015	23	24.8857	85.8980	941.8510	3.8347	1.6908
11	2015	23	22.5143	79.4184	875.0204	4.6276	2.0490
35	2015	23	23.2143	84.6122	935.7878	2.5398	1.5898
78	2015	23	23.3143	82.0000	896.4582	3.8969	1.8143
28	2015	23	23.7000	82.6837	925.5112	2.1051	1.3827
39	2015	23	22.2429	91.4898	899.2867	1.0204	2.8480
24	2015	23	23.7000	83.2755	936.3296	2.1510	1.3020
63	2015	23	24.8857	85.8980	941.8510	3.8347	1.6908
62	2015	23	20.7857	87.7959	872.1724	1.7378	1.0469
1	2015	23	21.0429	88.6939	872.5857	2.5949	2.4633
31	2015	24	18.5286	87.0408	845.1980	1.2316	0.9092
79	2015	24	23.5714	85.7857	965.9010	2.3816	1.3051
51	2015	24	22.0429	84.3469	935.9510	2.4418	1.3561
14	2015	24	21.4286	85.6224	894.9378	1.8153	2.5194
67	2015	24	20.7714	92.7143	899.6327	0.7296	2.7388
42	2015	24	20.2571	90.2857	873.0755	1.9816	2.2663
50	2015	24	20.7714	85.7551	899.4000	0.9816	1.3051
43	2015	24	20.2571	90.2857	873.0755	1.9816	2.2663
85	2015	24	21.3571	85.2245	908.1837	1.2316	1.2735
25	2015	24	24.5286	82.4694	972.6929	3.7806	1.1898
69	2015	24	22.4857	84.7857	936.6541	2.0633	1.1786
57	2015	24	20.3429	83.2245	884.7867	1.1653	2.1092
9	2015	24	20.0429	84.0408	852.5520	1.5622	2.2276
72	2015	24	21.5714	81.1020	875.5714	2.8806	1.9888
26	2015	24	21.6286	83.1020	864.9990	5.5745	2.1133
7	2015	24	21.3143	78.5102	857.3735	4.4898	2.0061
83	2015	24	25.4857	77.8980	940.6929	5.4857	1.2755
76	2015	24	21.7429	86.9592	918.2541	1.6857	1.3337
36	2015	24	22.0857	84.2143	925.8684	1.7490	1.2786
81	2015	24	22.0429	84.3469	935.9510	2.4418	1.3561
15	2015	24	22.6714	90.3265	930.2449	1.1745	0.5735
32	2015	24	20.2571	90.2857	873.0755	1.9816	2.2663
73	2015	24	23.4429	85.1939	958.4276	1.3602	0.9224

71	2015	24	22.0857	84.2143	925.8684	1.7490	1.2786
41	2015	24	20.7143	89.1224	872.7327	1.4010	0.9306
10	2015	24	23.3286	86.9082	960.3520	2.7143	0.9500
23	2015	24	15.6571	84.5408	775.7490	3.1296	2.4531
27	2015	24	21.3143	78.5102	857.3735	4.4898	2.0061
60	2015	24	22.0429	84.3469	935.9510	2.4418	1.3561
53	2015	24	20.0429	84.0408	852.5520	1.5622	2.2276
66	2015	24	21.4286	85.6224	894.9378	1.8153	2.5194
59	2015	24	20.3429	83.2245	884.7867	1.1653	2.1092
61	2015	24	23.4429	85.1939	958.4276	1.3602	0.9224
84	2015	24	23.4429	85.1939	958.4276	1.3602	0.9224
38	2015	24	20.3429	83.2245	884.7867	1.1653	2.1092
87	2015	24	22.4286	84.2245	896.9602	2.5929	1.6776
34	2015	24	20.3429	83.2245	884.7867	1.1653	2.1092
29	2015	24	22.4857	84.7857	936.6541	2.0633	1.1786
5	2015	24	19.3286	81.7755	830.9408	5.1633	1.8816
8	2015	24	20.0429	84.0408	852.5520	1.5622	2.2276
12	2015	24	19.3286	81.7755	830.9408	5.1633	1.8816
13	2015	24	25.4857	77.8980	940.6929	5.4857	1.2755
18	2015	24	23.1714	87.8163	960.5663	2.2367	0.9898
33	2015	24	20.7714	85.7551	899.4000	0.9816	1.3051
56	2015	24	24.5286	82.4694	972.6929	3.7806	1.1898
77	2015	24	22.6714	90.3265	930.2449	1.1745	0.5735
54	2015	24	19.3286	81.7755	830.9408	5.1633	1.8816
21	2015	24	20.7714	85.7551	899.4000	0.9816	1.3051
68	2015	24	23.5714	85.7857	965.9010	2.3816	1.3051
74	2015	24	23.4429	85.1939	958.4276	1.3602	0.9224
88	2015	24	20.2571	90.2857	873.0755	1.9816	2.2663
16	2015	24	21.7429	86.9592	918.2541	1.6857	1.3337
30	2015	24	21.4286	85.6224	894.9378	1.8153	2.5194
6	2015	24	23.5714	85.7857	965.9010	2.3816	1.3051
49	2015	24	22.4857	84.7857	936.6541	2.0633	1.1786
22	2015	24	20.2571	90.2857	873.0755	1.9816	2.2663
45	2015	24	18.6857	76.0714	816.2510	5.4153	1.8571
58	2015	24	22.4857	84.7857	936.6541	2.0633	1.1786
37	2015	24	23.5714	85.7857	965.9010	2.3816	1.3051
17	2015	24	20.7714	92.7143	899.6327	0.7296	2.7388
55	2015	24	21.5714	81.1020	875.5714	2.8806	1.9888
46	2015	24	21.7429	86.9592	918.2541	1.6857	1.3337
86	2015	24	20.4143	83.5000	864.4408	2.1347	0.7633
2	2015	24	20.4143	83.5000	864.4408	2.1347	0.7633
4	2015	24	20.7714	85.7551	899.4000	0.9816	1.3051
47	2015	24	25.5571	81.3367	954.6439	3.0796	0.7051
82	2015	24	20.2571	90.2857	873.0755	1.9816	2.2663
19	2015	24	24.7714	81.2653	956.6724	1.7480	1.1296
20	2015	24	20.0429	84.0408	852.5520	1.5622	2.2276
80	2015	24	20.2571	90.2857	873.0755	1.9816	2.2663
3	2015	24	25.4857	77.8980	940.6929	5.4857	1.2755
52	2015	24	20.7714	92.7143	899.6327	0.7296	2.7388
70	2015	24	21.3571	85.2245	908.1837	1.2316	1.2735

64	2015	24	15.6571	84.5408	775.7490	3.1296	2.4531
48	2015	24	22.6714	90.3265	930.2449	1.1745	0.5735
65	2015	24	20.7714	92.7143	899.6327	0.7296	2.7388
44	2015	24	21.3571	85.2245	908.1837	1.2316	1.2735
75	2015	24	15.6571	84.5408	775.7490	3.1296	2.4531
40	2015	24	22.6429	84.1327	942.0418	3.7316	1.6633
11	2015	24	21.5714	81.1020	875.5714	2.8806	1.9888
35	2015	24	22.0429	84.3469	935.9510	2.4418	1.3561
78	2015	24	22.4286	84.2245	896.9602	2.5929	1.6776
28	2015	24	22.0857	84.2143	925.8684	1.7490	1.2786
39	2015	24	20.7714	92.7143	899.6327	0.7296	2.7388
24	2015	24	22.4857	84.7857	936.6541	2.0633	1.1786
63	2015	24	22.6429	84.1327	942.0418	3.7316	1.6633
62	2015	24	20.7143	89.1224	872.7327	1.4010	0.9306
1	2015	24	20.2571	90.2857	873.0755	1.9816	2.2663
31	2015	25	20.8143	85.1429	845.1010	1.7469	0.9173
79	2015	25	25.4143	82.2959	965.5408	3.0918	1.2071
51	2015	25	24.2000	82.3163	935.8959	2.9194	1.3071
14	2015	25	22.6143	86.8980	895.1214	1.6306	2.1980
67	2015	25	22.6000	92.6327	899.7357	0.6418	2.4939
42	2015	25	21.9286	89.9490	873.1102	1.2806	2.1694
50	2015	25	22.6857	83.8061	899.2071	1.8694	1.3418
43	2015	25	21.9286	89.9490	873.1102	1.2806	2.1694
85	2015	25	23.3143	84.3061	907.9133	2.2847	1.3643
25	2015	25	25.4143	86.0204	972.9918	1.9490	0.9051
69	2015	25	24.7714	83.0714	936.6439	2.4633	1.1061
57	2015	25	22.0571	80.2857	884.6673	1.5612	2.2010
9	2015	25	21.1857	84.2551	852.6837	1.3806	2.1653
72	2015	25	22.7143	82.0408	875.7704	2.5918	1.7633
26	2015	25	23.3000	85.0918	865.3357	4.4224	1.9551
7	2015	25	22.7429	81.0204	857.6867	3.3939	1.7847
83	2015	25	26.7857	81.6531	941.0980	4.7398	1.1204
76	2015	25	23.5857	84.7449	918.1276	2.2133	1.3000
36	2015	25	24.4143	83.1224	925.9520	1.9418	1.3561
81	2015	25	24.2000	82.3163	935.8959	2.9194	1.3071
15	2015	25	24.0000	88.1122	929.9714	1.8439	0.5724
32	2015	25	21.9286	89.9490	873.1102	1.2806	2.1694
73	2015	25	25.6286	82.0714	958.1633	1.7582	0.9806
71	2015	25	24.4143	83.1224	925.9520	1.9418	1.3561
41	2015	25	22.7143	86.3980	872.6388	2.4990	1.1469
10	2015	25	25.1857	84.6531	960.3020	3.2612	0.8714
23	2015	25	17.5286	83.8776	775.9337	2.6327	2.3653
27	2015	25	22.7429	81.0204	857.6867	3.3939	1.7847
60	2015	25	24.2000	82.3163	935.8959	2.9194	1.3071
53	2015	25	21.1857	84.2551	852.6837	1.3806	2.1653
66	2015	25	22.6143	86.8980	895.1214	1.6306	2.1980
59	2015	25	22.0571	80.2857	884.6673	1.5612	2.2010
61	2015	25	25.6286	82.0714	958.1633	1.7582	0.9806
84	2015	25	25.6286	82.0714	958.1633	1.7582	0.9806
38	2015	25	22.0571	80.2857	884.6673	1.5612	2.2010

87	2015	25	23.8571	84.1224	897.0969	2.2776	1.4408
34	2015	25	22.0571	80.2857	884.6673	1.5612	2.2010
29	2015	25	24.7714	83.0714	936.6439	2.4633	1.1061
5	2015	25	20.9429	83.1531	831.2194	4.0582	1.6816
8	2015	25	21.1857	84.2551	852.6837	1.3806	2.1653
12	2015	25	20.9429	83.1531	831.2194	4.0582	1.6816
13	2015	25	26.7857	81.6531	941.0980	4.7398	1.1204
18	2015	25	24.9571	84.7857	960.1296	3.0827	1.0898
33	2015	25	22.6857	83.8061	899.2071	1.8694	1.3418
56	2015	25	25.4143	86.0204	972.9918	1.9490	0.9051
77	2015	25	24.0000	88.1122	929.9714	1.8439	0.5724
54	2015	25	20.9429	83.1531	831.2194	4.0582	1.6816
21	2015	25	22.6857	83.8061	899.2071	1.8694	1.3418
68	2015	25	25.4143	82.2959	965.5408	3.0918	1.2071
74	2015	25	25.6286	82.0714	958.1633	1.7582	0.9806
88	2015	25	21.9286	89.9490	873.1102	1.2806	2.1694
16	2015	25	23.5857	84.7449	918.1276	2.2133	1.3000
30	2015	25	22.6143	86.8980	895.1214	1.6306	2.1980
6	2015	25	25.4143	82.2959	965.5408	3.0918	1.2071
49	2015	25	24.7714	83.0714	936.6439	2.4633	1.1061
22	2015	25	21.9286	89.9490	873.1102	1.2806	2.1694
45	2015	25	20.6286	80.8163	816.5918	3.5224	1.8816
58	2015	25	24.7714	83.0714	936.6439	2.4633	1.1061
37	2015	25	25.4143	82.2959	965.5408	3.0918	1.2071
17	2015	25	22.6000	92.6327	899.7357	0.6418	2.4939
55	2015	25	22.7143	82.0408	875.7704	2.5918	1.7633
46	2015	25	23.5857	84.7449	918.1276	2.2133	1.3000
86	2015	25	22.2000	82.8673	864.4459	2.6255	0.9010
2	2015	25	22.2000	82.8673	864.4459	2.6255	0.9010
4	2015	25	22.6857	83.8061	899.2071	1.8694	1.3418
47	2015	25	26.5143	83.8776	954.9847	2.6133	0.6837
82	2015	25	21.9286	89.9490	873.1102	1.2806	2.1694
19	2015	25	25.8571	85.0000	956.9816	1.1367	1.0561
20	2015	25	21.1857	84.2551	852.6837	1.3806	2.1653
80	2015	25	21.9286	89.9490	873.1102	1.2806	2.1694
3	2015	25	26.7857	81.6531	941.0980	4.7398	1.1204
52	2015	25	22.6000	92.6327	899.7357	0.6418	2.4939
70	2015	25	23.3143	84.3061	907.9133	2.2847	1.3643
64	2015	25	17.5286	83.8776	775.9337	2.6327	2.3653
48	2015	25	24.0000	88.1122	929.9714	1.8439	0.5724
65	2015	25	22.6000	92.6327	899.7357	0.6418	2.4939
44	2015	25	23.3143	84.3061	907.9133	2.2847	1.3643
75	2015	25	17.5286	83.8776	775.9337	2.6327	2.3653
40	2015	25	24.4143	85.5714	942.1653	2.5990	1.3602
11	2015	25	22.7143	82.0408	875.7704	2.5918	1.7633
35	2015	25	24.2000	82.3163	935.8959	2.9194	1.3071
78	2015	25	23.8571	84.1224	897.0969	2.2776	1.4408
28	2015	25	24.4143	83.1224	925.9520	1.9418	1.3561
39	2015	25	22.6000	92.6327	899.7357	0.6418	2.4939
24	2015	25	24.7714	83.0714	936.6439	2.4633	1.1061

63	2015	25	24.4143	85.5714	942.1653	2.5990	1.3602
62	2015	25	22.7143	86.3980	872.6388	2.4990	1.1469
1	2015	25	21.9286	89.9490	873.1102	1.2806	2.1694
31	2015	26	24.7857	80.7041	843.3990	3.1408	1.0735
79	2015	26	28.1000	79.5000	964.0704	3.5673	1.3398
51	2015	26	26.5000	80.6429	934.5898	3.5898	1.5143
14	2015	26	24.1714	87.3673	894.1041	2.4235	2.5714
67	2015	26	24.3571	92.3163	898.6000	0.8000	2.3929
42	2015	26	23.7571	89.4796	871.8612	1.5724	2.2041
50	2015	26	26.2429	80.7347	897.4898	1.9224	1.5112
43	2015	26	23.7571	89.4796	871.8612	1.5724	2.2041
85	2015	26	27.8571	79.0510	905.9051	3.6653	1.8878
25	2015	26	27.9857	87.1429	971.7724	2.5908	0.8541
69	2015	26	27.1857	80.7245	935.1122	3.0724	1.3102
57	2015	26	24.9714	77.5306	883.1388	2.8888	2.6378
9	2015	26	22.7000	84.8673	851.5337	2.0327	2.2173
72	2015	26	23.9429	82.8367	874.6684	3.3367	1.7694
26	2015	26	24.1857	84.2449	864.3092	4.6071	1.8041
7	2015	26	23.6857	81.7347	856.6327	3.7184	1.9735
83	2015	26	27.7857	83.3367	939.8592	5.4061	0.9490
76	2015	26	26.2143	82.3673	916.5694	2.2592	1.5051
36	2015	26	26.5857	81.0612	924.6010	2.6010	1.5184
81	2015	26	26.5000	80.6429	934.5898	3.5898	1.5143
15	2015	26	27.8714	83.9694	928.1459	1.9173	0.7643
32	2015	26	23.7571	89.4796	871.8612	1.5724	2.2041
73	2015	26	28.3000	78.4694	956.4276	2.2337	1.1561
71	2015	26	26.5857	81.0612	924.6010	2.6010	1.5184
41	2015	26	25.2857	84.0408	871.0816	3.5969	1.5296
10	2015	26	27.2000	84.1020	959.0551	3.7571	0.9765
23	2015	26	20.0714	82.8367	774.8398	3.4051	2.4776
27	2015	26	23.6857	81.7347	856.6327	3.7184	1.9735
60	2015	26	26.5000	80.6429	934.5898	3.5898	1.5143
53	2015	26	22.7000	84.8673	851.5337	2.0327	2.2173
66	2015	26	24.1714	87.3673	894.1041	2.4235	2.5714
59	2015	26	24.9714	77.5306	883.1388	2.8888	2.6378
61	2015	26	28.3000	78.4694	956.4276	2.2337	1.1561
84	2015	26	28.3000	78.4694	956.4276	2.2337	1.1561
38	2015	26	24.9714	77.5306	883.1388	2.8888	2.6378
87	2015	26	25.3143	84.9796	895.8867	2.8969	1.3490
34	2015	26	24.9714	77.5306	883.1388	2.8888	2.6378
29	2015	26	27.1857	80.7245	935.1122	3.0724	1.3102
5	2015	26	22.4286	82.6939	830.2051	4.4551	1.5378
8	2015	26	22.7000	84.8673	851.5337	2.0327	2.2173
12	2015	26	22.4286	82.6939	830.2051	4.4551	1.5378
13	2015	26	27.7857	83.3367	939.8592	5.4061	0.9490
18	2015	26	27.8714	82.3673	958.6194	3.0286	1.2245
33	2015	26	26.2429	80.7347	897.4898	1.9224	1.5112
56	2015	26	27.9857	87.1429	971.7724	2.5908	0.8541
77	2015	26	27.8714	83.9694	928.1459	1.9173	0.7643
54	2015	26	22.4286	82.6939	830.2051	4.4551	1.5378

21	2015	26	26.2429	80.7347	897.4898	1.9224	1.5112
68	2015	26	28.1000	79.5000	964.0704	3.5673	1.3398
74	2015	26	28.3000	78.4694	956.4276	2.2337	1.1561
88	2015	26	23.7571	89.4796	871.8612	1.5724	2.2041
16	2015	26	26.2143	82.3673	916.5694	2.2592	1.5051
30	2015	26	24.1714	87.3673	894.1041	2.4235	2.5714
6	2015	26	28.1000	79.5000	964.0704	3.5673	1.3398
49	2015	26	27.1857	80.7245	935.1122	3.0724	1.3102
22	2015	26	23.7571	89.4796	871.8612	1.5724	2.2041
45	2015	26	21.8714	81.2857	815.6837	3.8959	1.9949
58	2015	26	27.1857	80.7245	935.1122	3.0724	1.3102
37	2015	26	28.1000	79.5000	964.0704	3.5673	1.3398
17	2015	26	24.3571	92.3163	898.6000	0.8000	2.3929
55	2015	26	23.9429	82.8367	874.6684	3.3367	1.7694
46	2015	26	26.2143	82.3673	916.5694	2.2592	1.5051
86	2015	26	24.3143	80.9694	862.9908	3.0918	1.3112
2	2015	26	24.3143	80.9694	862.9908	3.0918	1.3112
4	2015	26	26.2429	80.7347	897.4898	1.9224	1.5112
47	2015	26	27.8000	85.2347	953.7367	3.2316	0.5990
82	2015	26	23.7571	89.4796	871.8612	1.5724	2.2041
19	2015	26	27.7143	86.3469	955.7704	2.2316	1.0541
20	2015	26	22.7000	84.8673	851.5337	2.0327	2.2173
80	2015	26	23.7571	89.4796	871.8612	1.5724	2.2041
3	2015	26	27.7857	83.3367	939.8592	5.4061	0.9490
52	2015	26	24.3571	92.3163	898.6000	0.8000	2.3929
70	2015	26	27.8571	79.0510	905.9051	3.6653	1.8878
64	2015	26	20.0714	82.8367	774.8398	3.4051	2.4776
48	2015	26	27.8714	83.9694	928.1459	1.9173	0.7643
65	2015	26	24.3571	92.3163	898.6000	0.8000	2.3929
44	2015	26	27.8571	79.0510	905.9051	3.6653	1.8878
75	2015	26	20.0714	82.8367	774.8398	3.4051	2.4776
40	2015	26	26.1857	85.9286	941.1041	3.3724	1.5122
11	2015	26	23.9429	82.8367	874.6684	3.3367	1.7694
35	2015	26	26.5000	80.6429	934.5898	3.5898	1.5143
78	2015	26	25.3143	84.9796	895.8867	2.8969	1.3490
28	2015	26	26.5857	81.0612	924.6010	2.6010	1.5184
39	2015	26	24.3571	92.3163	898.6000	0.8000	2.3929
24	2015	26	27.1857	80.7245	935.1122	3.0724	1.3102
63	2015	26	26.1857	85.9286	941.1041	3.3724	1.5122
62	2015	26	25.2857	84.0408	871.0816	3.5969	1.5296
1	2015	26	23.7571	89.4796	871.8612	1.5724	2.2041
31	2015	27	20.6000	75.8469	842.0214	4.5051	1.3439
79	2015	27	24.8714	78.6633	962.5929	4.2867	1.5643
51	2015	27	23.9429	78.1224	933.2092	4.4602	1.7704
14	2015	27	23.1286	85.9490	893.0306	3.4235	2.8653
67	2015	27	22.6143	90.7959	897.4122	1.3847	3.0082
42	2015	27	21.8143	87.2857	870.7347	2.0969	2.5194
50	2015	27	22.0143	78.1735	895.9837	2.0520	1.7867
43	2015	27	21.8143	87.2857	870.7347	2.0969	2.5194
85	2015	27	22.8857	74.1735	904.1541	4.6265	2.3480

25	2015	27	26.1571	83.8367	970.2857	4.1378	1.0959
69	2015	27	24.3000	78.9898	933.6337	3.8827	1.6265
57	2015	27	21.4857	74.7857	881.7765	4.1031	3.7194
9	2015	27	21.2429	83.8878	850.5163	2.9327	2.5184
72	2015	27	22.8571	81.6735	873.6122	4.2837	2.0531
26	2015	27	22.9000	81.3163	863.4041	5.3776	1.9316
7	2015	27	22.4000	79.6224	855.6847	4.3010	2.2367
83	2015	27	26.5857	81.9082	938.6316	5.9571	0.9714
76	2015	27	23.0429	81.0816	915.1071	2.3929	1.6429
36	2015	27	24.2571	78.0918	923.2337	3.0867	1.8143
81	2015	27	23.9429	78.1224	933.2092	4.4602	1.7704
15	2015	27	23.7429	80.7143	926.4612	1.8204	0.9602
32	2015	27	21.8143	87.2857	870.7347	2.0969	2.5194
73	2015	27	25.4857	75.8163	954.7602	2.7082	1.4776
71	2015	27	24.2571	78.0918	923.2337	3.0867	1.8143
41	2015	27	22.4571	81.4286	869.7806	4.3112	1.9010
10	2015	27	24.8429	82.8163	957.6480	4.0357	1.2031
23	2015	27	16.6857	82.2449	774.0510	4.0439	2.6867
27	2015	27	22.4000	79.6224	855.6847	4.3010	2.2367
60	2015	27	23.9429	78.1224	933.2092	4.4602	1.7704
53	2015	27	21.2429	83.8878	850.5163	2.9327	2.5184
66	2015	27	23.1286	85.9490	893.0306	3.4235	2.8653
59	2015	27	21.4857	74.7857	881.7765	4.1031	3.7194
61	2015	27	25.4857	75.8163	954.7602	2.7082	1.4776
84	2015	27	25.4857	75.8163	954.7602	2.7082	1.4776
38	2015	27	21.4857	74.7857	881.7765	4.1031	3.7194
87	2015	27	24.0286	83.2347	894.7020	3.5776	1.6153
34	2015	27	21.4857	74.7857	881.7765	4.1031	3.7194
29	2015	27	24.3000	78.9898	933.6337	3.8827	1.6265
5	2015	27	20.4286	80.9082	829.3316	4.8439	1.7235
8	2015	27	21.2429	83.8878	850.5163	2.9327	2.5184
12	2015	27	20.4286	80.9082	829.3316	4.8439	1.7235
13	2015	27	26.5857	81.9082	938.6316	5.9571	0.9714
18	2015	27	24.8000	80.3265	957.1082	3.9184	1.4510
33	2015	27	22.0143	78.1735	895.9837	2.0520	1.7867
56	2015	27	26.1571	83.8367	970.2857	4.1378	1.0959
77	2015	27	23.7429	80.7143	926.4612	1.8204	0.9602
54	2015	27	20.4286	80.9082	829.3316	4.8439	1.7235
21	2015	27	22.0143	78.1735	895.9837	2.0520	1.7867
68	2015	27	24.8714	78.6633	962.5929	4.2867	1.5643
74	2015	27	25.4857	75.8163	954.7602	2.7082	1.4776
88	2015	27	21.8143	87.2857	870.7347	2.0969	2.5194
16	2015	27	23.0429	81.0816	915.1071	2.3929	1.6429
30	2015	27	23.1286	85.9490	893.0306	3.4235	2.8653
6	2015	27	24.8714	78.6633	962.5929	4.2867	1.5643
49	2015	27	24.3000	78.9898	933.6337	3.8827	1.6265
22	2015	27	21.8143	87.2857	870.7347	2.0969	2.5194
45	2015	27	19.5714	79.3367	814.9102	4.3847	2.1398
58	2015	27	24.3000	78.9898	933.6337	3.8827	1.6265
37	2015	27	24.8714	78.6633	962.5929	4.2867	1.5643

17	2015	27	22.6143	90.7959	897.4122	1.3847	3.0082
55	2015	27	22.8571	81.6735	873.6122	4.2837	2.0531
46	2015	27	23.0429	81.0816	915.1071	2.3929	1.6429
86	2015	27	22.1571	78.6939	861.7561	3.7806	1.7776
2	2015	27	22.1571	78.6939	861.7561	3.7806	1.7776
4	2015	27	22.0143	78.1735	895.9837	2.0520	1.7867
47	2015	27	27.1571	83.2143	952.3673	4.3704	0.6276
82	2015	27	21.8143	87.2857	870.7347	2.0969	2.5194
19	2015	27	26.6286	83.7041	954.4194	3.2857	1.1429
20	2015	27	21.2429	83.8878	850.5163	2.9327	2.5184
80	2015	27	21.8143	87.2857	870.7347	2.0969	2.5194
3	2015	27	26.5857	81.9082	938.6316	5.9571	0.9714
52	2015	27	22.6143	90.7959	897.4122	1.3847	3.0082
70	2015	27	22.8857	74.1735	904.1541	4.6265	2.3480
64	2015	27	16.6857	82.2449	774.0510	4.0439	2.6867
48	2015	27	23.7429	80.7143	926.4612	1.8204	0.9602
65	2015	27	22.6143	90.7959	897.4122	1.3847	3.0082
44	2015	27	22.8857	74.1735	904.1541	4.6265	2.3480
75	2015	27	16.6857	82.2449	774.0510	4.0439	2.6867
40	2015	27	24.4714	82.8673	939.8163	4.9347	2.0071
11	2015	27	22.8571	81.6735	873.6122	4.2837	2.0531
35	2015	27	23.9429	78.1224	933.2092	4.4602	1.7704
78	2015	27	24.0286	83.2347	894.7020	3.5776	1.6153
28	2015	27	24.2571	78.0918	923.2337	3.0867	1.8143
39	2015	27	22.6143	90.7959	897.4122	1.3847	3.0082
24	2015	27	24.3000	78.9898	933.6337	3.8827	1.6265
63	2015	27	24.4714	82.8673	939.8163	4.9347	2.0071
62	2015	27	22.4571	81.4286	869.7806	4.3112	1.9010
1	2015	27	21.8143	87.2857	870.7347	2.0969	2.5194
31	2015	28	21.0286	74.0816	843.7867	5.4071	1.3439
79	2015	28	24.0857	80.5306	964.1888	3.9898	1.5020
51	2015	28	21.9429	78.3673	934.6327	4.3143	1.7418
14	2015	28	21.1714	81.2755	893.9224	3.9286	2.5878
67	2015	28	20.7286	88.6429	898.5929	2.0633	3.3112
42	2015	28	21.0429	84.0510	872.0449	3.2245	2.7041
50	2015	28	23.1714	77.5816	897.8918	3.2847	1.5694
43	2015	28	21.0429	84.0510	872.0449	3.2245	2.7041
85	2015	28	24.0429	76.1531	906.1663	5.1143	1.7663
25	2015	28	24.4000	81.6837	971.5255	3.8622	1.0980
69	2015	28	23.3714	78.8367	935.3031	4.1459	1.5378
57	2015	28	20.9429	74.8367	883.3878	4.1888	3.3602
9	2015	28	20.8000	78.9898	851.7204	4.1469	2.5020
72	2015	28	21.8714	77.4490	874.7214	4.7837	2.1898
26	2015	28	22.6714	78.0714	864.5235	5.8133	1.8306
7	2015	28	21.5857	76.3673	856.8520	4.9673	1.9551
83	2015	28	24.9143	80.3265	939.9776	5.5755	1.0459
76	2015	28	23.3000	79.4490	916.8020	4.0959	1.4694
36	2015	28	22.1143	77.4694	924.6827	3.2490	1.7653
81	2015	28	21.9429	78.3673	934.6327	4.3143	1.7418
15	2015	28	24.8000	80.4490	928.3633	3.7898	0.9102

32	2015	28	21.0429	84.0510	872.0449	3.2245	2.7041
73	2015	28	25.1714	74.9592	956.5235	3.1490	1.3571
71	2015	28	22.1143	77.4694	924.6827	3.2490	1.7653
41	2015	28	22.0857	79.5816	871.3806	5.4949	1.5857
10	2015	28	22.9714	83.6020	958.9949	4.0755	1.1969
23	2015	28	16.8286	79.5714	775.3653	4.9929	2.4214
27	2015	28	21.5857	76.3673	856.8520	4.9673	1.9551
60	2015	28	21.9429	78.3673	934.6327	4.3143	1.7418
53	2015	28	20.8000	78.9898	851.7204	4.1469	2.5020
66	2015	28	21.1714	81.2755	893.9224	3.9286	2.5878
59	2015	28	20.9429	74.8367	883.3878	4.1888	3.3602
61	2015	28	25.1714	74.9592	956.5235	3.1490	1.3571
84	2015	28	25.1714	74.9592	956.5235	3.1490	1.3571
38	2015	28	20.9429	74.8367	883.3878	4.1888	3.3602
87	2015	28	22.2143	79.1939	895.9561	4.2745	2.1857
34	2015	28	20.9429	74.8367	883.3878	4.1888	3.3602
29	2015	28	23.3714	78.8367	935.3031	4.1459	1.5378
5	2015	28	20.1143	78.5612	830.5010	5.9857	1.7133
8	2015	28	20.8000	78.9898	851.7204	4.1469	2.5020
12	2015	28	20.1143	78.5612	830.5010	5.9857	1.7133
13	2015	28	24.9143	80.3265	939.9776	5.5755	1.0459
18	2015	28	24.2857	81.2857	958.7276	4.0469	1.3622
33	2015	28	23.1714	77.5816	897.8918	3.2847	1.5694
56	2015	28	24.4000	81.6837	971.5255	3.8622	1.0980
77	2015	28	24.8000	80.4490	928.3633	3.7898	0.9102
54	2015	28	20.1143	78.5612	830.5010	5.9857	1.7133
21	2015	28	23.1714	77.5816	897.8918	3.2847	1.5694
68	2015	28	24.0857	80.5306	964.1888	3.9898	1.5020
74	2015	28	25.1714	74.9592	956.5235	3.1490	1.3571
88	2015	28	21.0429	84.0510	872.0449	3.2245	2.7041
16	2015	28	23.3000	79.4490	916.8020	4.0959	1.4694
30	2015	28	21.1714	81.2755	893.9224	3.9286	2.5878
6	2015	28	24.0857	80.5306	964.1888	3.9898	1.5020
49	2015	28	23.3714	78.8367	935.3031	4.1459	1.5378
22	2015	28	21.0429	84.0510	872.0449	3.2245	2.7041
45	2015	28	19.5000	78.1531	816.0071	5.1071	1.8418
58	2015	28	23.3714	78.8367	935.3031	4.1459	1.5378
37	2015	28	24.0857	80.5306	964.1888	3.9898	1.5020
17	2015	28	20.7286	88.6429	898.5929	2.0633	3.3112
55	2015	28	21.8714	77.4490	874.7214	4.7837	2.1898
46	2015	28	23.3000	79.4490	916.8020	4.0959	1.4694
86	2015	28	21.5143	76.3571	863.2776	5.3857	1.4490
2	2015	28	21.5143	76.3571	863.2776	5.3857	1.4490
4	2015	28	23.1714	77.5816	897.8918	3.2847	1.5694
47	2015	28	24.8571	80.5306	953.6184	4.6918	0.6633
82	2015	28	21.0429	84.0510	872.0449	3.2245	2.7041
19	2015	28	24.6429	80.3163	955.4633	3.4378	1.2357
20	2015	28	20.8000	78.9898	851.7204	4.1469	2.5020
80	2015	28	21.0429	84.0510	872.0449	3.2245	2.7041
3	2015	28	24.9143	80.3265	939.9776	5.5755	1.0459

52	2015	28	20.7286	88.6429	898.5929	2.0633	3.3112
70	2015	28	24.0429	76.1531	906.1663	5.1143	1.7663
64	2015	28	16.8286	79.5714	775.3653	4.9929	2.4214
48	2015	28	24.8000	80.4490	928.3633	3.7898	0.9102
65	2015	28	20.7286	88.6429	898.5929	2.0633	3.3112
44	2015	28	24.0429	76.1531	906.1663	5.1143	1.7663
75	2015	28	16.8286	79.5714	775.3653	4.9929	2.4214
40	2015	28	22.2714	82.9796	940.7949	4.3265	1.9806
11	2015	28	21.8714	77.4490	874.7214	4.7837	2.1898
35	2015	28	21.9429	78.3673	934.6327	4.3143	1.7418
78	2015	28	22.2143	79.1939	895.9561	4.2745	2.1857
28	2015	28	22.1143	77.4694	924.6827	3.2490	1.7653
39	2015	28	20.7286	88.6429	898.5929	2.0633	3.3112
24	2015	28	23.3714	78.8367	935.3031	4.1459	1.5378
63	2015	28	22.2714	82.9796	940.7949	4.3265	1.9806
62	2015	28	22.0857	79.5816	871.3806	5.4949	1.5857
1	2015	28	21.0429	84.0510	872.0449	3.2245	2.7041
31	2015	29	21.4000	74.5510	845.3224	4.7255	1.1041
79	2015	29	27.3143	79.9082	965.3469	4.4122	1.1633
51	2015	29	25.2857	80.3980	935.7439	3.8367	1.3816
14	2015	29	23.9571	77.8265	894.6582	4.2041	1.8796
67	2015	29	23.7571	85.5612	899.6031	2.8500	2.4816
42	2015	29	23.1429	79.1122	873.1276	4.1235	2.4500
50	2015	29	23.7571	75.2653	899.4908	4.5806	1.3765
43	2015	29	23.1429	79.1122	873.1276	4.1235	2.4500
85	2015	29	24.5286	75.5510	907.8143	5.6214	1.2020
25	2015	29	27.5857	80.3469	972.3592	4.3459	0.9980
69	2015	29	25.5143	79.1939	936.6429	4.3306	1.2020
57	2015	29	22.9286	73.6224	884.7755	3.9337	2.0898
9	2015	29	22.3429	73.3367	852.7500	4.7378	2.1500
72	2015	29	23.1000	74.4592	875.6551	4.8765	1.8694
26	2015	29	23.3429	75.1224	865.3643	6.1827	1.5112
7	2015	29	22.5143	74.2653	857.7827	5.5786	1.4541
83	2015	29	27.2429	77.7143	940.9398	5.5847	1.0776
76	2015	29	25.3714	76.7755	918.1827	6.2214	1.3327
36	2015	29	25.5571	78.3265	925.8143	4.1204	1.4082
81	2015	29	25.2857	80.3980	935.7439	3.8367	1.3816
15	2015	29	25.9286	78.4490	929.9112	6.4612	0.8561
32	2015	29	23.1429	79.1122	873.1276	4.1235	2.4500
73	2015	29	27.5143	73.5000	957.9194	3.8990	1.0786
71	2015	29	25.5571	78.3265	925.8143	4.1204	1.4082
41	2015	29	23.1714	77.5000	872.7776	5.5633	0.9867
10	2015	29	26.1143	83.9184	959.9459	4.9939	1.0316
23	2015	29	17.3143	78.9286	776.4235	4.7663	2.0643
27	2015	29	22.5143	74.2653	857.7827	5.5786	1.4541
60	2015	29	25.2857	80.3980	935.7439	3.8367	1.3816
53	2015	29	22.3429	73.3367	852.7500	4.7378	2.1500
66	2015	29	23.9571	77.8265	894.6582	4.2041	1.8796
59	2015	29	22.9286	73.6224	884.7755	3.9337	2.0898
61	2015	29	27.5143	73.5000	957.9194	3.8990	1.0786

84	2015	29	27.5143	73.5000	957.9194	3.8990	1.0786
38	2015	29	22.9286	73.6224	884.7755	3.9337	2.0898
87	2015	29	24.3143	76.7449	897.0388	4.8653	2.0490
34	2015	29	22.9286	73.6224	884.7755	3.9337	2.0898
29	2015	29	25.5143	79.1939	936.6429	4.3306	1.2020
5	2015	29	20.7000	77.8265	831.4745	6.5122	1.4582
8	2015	29	22.3429	73.3367	852.7500	4.7378	2.1500
12	2015	29	20.7000	77.8265	831.4745	6.5122	1.4582
13	2015	29	27.2429	77.7143	940.9398	5.5847	1.0776
18	2015	29	26.5143	81.1531	959.9480	3.9204	1.1020
33	2015	29	23.7571	75.2653	899.4908	4.5806	1.3765
56	2015	29	27.5857	80.3469	972.3592	4.3459	0.9980
77	2015	29	25.9286	78.4490	929.9112	6.4612	0.8561
54	2015	29	20.7000	77.8265	831.4745	6.5122	1.4582
21	2015	29	23.7571	75.2653	899.4908	4.5806	1.3765
68	2015	29	27.3143	79.9082	965.3469	4.4122	1.1633
74	2015	29	27.5143	73.5000	957.9194	3.8990	1.0786
88	2015	29	23.1429	79.1122	873.1276	4.1235	2.4500
16	2015	29	25.3714	76.7755	918.1827	6.2214	1.3327
30	2015	29	23.9571	77.8265	894.6582	4.2041	1.8796
6	2015	29	27.3143	79.9082	965.3469	4.4122	1.1633
49	2015	29	25.5143	79.1939	936.6429	4.3306	1.2020
22	2015	29	23.1429	79.1122	873.1276	4.1235	2.4500
45	2015	29	19.9714	76.4694	816.8847	5.0051	1.3153
58	2015	29	25.5143	79.1939	936.6429	4.3306	1.2020
37	2015	29	27.3143	79.9082	965.3469	4.4122	1.1633
17	2015	29	23.7571	85.5612	899.6031	2.8500	2.4816
55	2015	29	23.1000	74.4592	875.6551	4.8765	1.8694
46	2015	29	25.3714	76.7755	918.1827	6.2214	1.3327
86	2015	29	22.5571	74.6327	864.6204	5.7378	0.8888
2	2015	29	22.5571	74.6327	864.6204	5.7378	0.8888
4	2015	29	23.7571	75.2653	899.4908	4.5806	1.3765
47	2015	29	27.3286	79.4898	954.6449	5.1122	0.7112
82	2015	29	23.1429	79.1122	873.1276	4.1235	2.4500
19	2015	29	27.7429	76.9388	956.1612	4.9184	1.1439
20	2015	29	22.3429	73.3367	852.7500	4.7378	2.1500
80	2015	29	23.1429	79.1122	873.1276	4.1235	2.4500
3	2015	29	27.2429	77.7143	940.9398	5.5847	1.0776
52	2015	29	23.7571	85.5612	899.6031	2.8500	2.4816
70	2015	29	24.5286	75.5510	907.8143	5.6214	1.2020
64	2015	29	17.3143	78.9286	776.4235	4.7663	2.0643
48	2015	29	25.9286	78.4490	929.9112	6.4612	0.8561
65	2015	29	23.7571	85.5612	899.6031	2.8500	2.4816
44	2015	29	24.5286	75.5510	907.8143	5.6214	1.2020
75	2015	29	17.3143	78.9286	776.4235	4.7663	2.0643
40	2015	29	26.1429	81.7653	941.4622	4.6786	1.4000
11	2015	29	23.1000	74.4592	875.6551	4.8765	1.8694
35	2015	29	25.2857	80.3980	935.7439	3.8367	1.3816
78	2015	29	24.3143	76.7449	897.0388	4.8653	2.0490
28	2015	29	25.5571	78.3265	925.8143	4.1204	1.4082

39	2015	29	23.7571	85.5612	899.6031	2.8500	2.4816
24	2015	29	25.5143	79.1939	936.6429	4.3306	1.2020
63	2015	29	26.1429	81.7653	941.4622	4.6786	1.4000
62	2015	29	23.1714	77.5000	872.7776	5.5633	0.9867
1	2015	29	23.1429	79.1122	873.1276	4.1235	2.4500
31	2015	30	21.2857	74.2143	844.7939	3.2429	1.0286
79	2015	30	27.4143	75.1633	963.9959	5.9286	1.2163
51	2015	30	25.2429	77.1224	934.6878	4.9612	1.3031
14	2015	30	23.5143	77.4388	894.2020	4.0408	1.5357
67	2015	30	23.3571	82.2959	898.9398	3.8051	2.1949
42	2015	30	23.0429	76.2449	872.5347	3.2551	2.3969
50	2015	30	24.2714	73.7755	898.6439	4.5592	1.4776
43	2015	30	23.0429	76.2449	872.5347	3.2551	2.3969
85	2015	30	24.9143	72.2653	906.8531	6.0429	1.2469
25	2015	30	27.2000	77.7245	971.3122	6.1520	1.2000
69	2015	30	25.6857	78.0918	935.6010	4.7153	1.1469
57	2015	30	23.0714	70.3469	884.0571	3.7429	1.8459
9	2015	30	22.3286	71.5306	852.3418	4.3194	1.8918
72	2015	30	23.1714	74.7755	875.2265	4.5898	1.5163
26	2015	30	22.8714	74.1020	865.1184	5.4898	1.3653
7	2015	30	22.3000	74.8980	857.4796	4.4143	1.3367
83	2015	30	26.2143	76.2449	940.2908	5.3561	1.0684
76	2015	30	24.8571	74.5102	917.1745	6.3776	1.3776
36	2015	30	25.1429	75.0612	924.8755	5.2816	1.4816
81	2015	30	25.2429	77.1224	934.6878	4.9612	1.3031
15	2015	30	25.9143	77.0102	928.7959	6.1163	0.8837
32	2015	30	23.0429	76.2449	872.5347	3.2551	2.3969
73	2015	30	27.3286	70.5408	956.6204	4.6327	1.1776
71	2015	30	25.1429	75.0612	924.8755	5.2816	1.4816
41	2015	30	23.4429	76.0816	872.1571	4.0459	0.9071
10	2015	30	26.1571	80.9592	958.7602	6.3643	1.1939
23	2015	30	17.2000	77.7551	776.3214	3.4031	1.9796
27	2015	30	22.3000	74.8980	857.4796	4.4143	1.3367
60	2015	30	25.2429	77.1224	934.6878	4.9612	1.3031
53	2015	30	22.3286	71.5306	852.3418	4.3194	1.8918
66	2015	30	23.5143	77.4388	894.2020	4.0408	1.5357
59	2015	30	23.0714	70.3469	884.0571	3.7429	1.8459
61	2015	30	27.3286	70.5408	956.6204	4.6327	1.1776
84	2015	30	27.3286	70.5408	956.6204	4.6327	1.1776
38	2015	30	23.0714	70.3469	884.0571	3.7429	1.8459
87	2015	30	24.0429	76.0612	896.4265	5.0531	1.3816
34	2015	30	23.0714	70.3469	884.0571	3.7429	1.8459
29	2015	30	25.6857	78.0918	935.6010	4.7153	1.1469
5	2015	30	20.8857	77.1633	831.2306	4.6531	1.2592
8	2015	30	22.3286	71.5306	852.3418	4.3194	1.8918
12	2015	30	20.8857	77.1633	831.2306	4.6531	1.2592
13	2015	30	26.2143	76.2449	940.2908	5.3561	1.0684
18	2015	30	26.5714	78.2755	958.6327	4.7714	1.0724
33	2015	30	24.2714	73.7755	898.6439	4.5592	1.4776
56	2015	30	27.2000	77.7245	971.3122	6.1520	1.2000

77	2015	30	25.9143	77.0102	928.7959	6.1163	0.8837
54	2015	30	20.8857	77.1633	831.2306	4.6531	1.2592
21	2015	30	24.2714	73.7755	898.6439	4.5592	1.4776
68	2015	30	27.4143	75.1633	963.9959	5.9286	1.2163
74	2015	30	27.3286	70.5408	956.6204	4.6327	1.1776
88	2015	30	23.0429	76.2449	872.5347	3.2551	2.3969
16	2015	30	24.8571	74.5102	917.1745	6.3776	1.3776
30	2015	30	23.5143	77.4388	894.2020	4.0408	1.5357
6	2015	30	27.4143	75.1633	963.9959	5.9286	1.2163
49	2015	30	25.6857	78.0918	935.6010	4.7153	1.1469
22	2015	30	23.0429	76.2449	872.5347	3.2551	2.3969
45	2015	30	20.2429	74.5816	816.7704	3.5184	1.2643
58	2015	30	25.6857	78.0918	935.6010	4.7153	1.1469
37	2015	30	27.4143	75.1633	963.9959	5.9286	1.2163
17	2015	30	23.3571	82.2959	898.9398	3.8051	2.1949
55	2015	30	23.1714	74.7755	875.2265	4.5898	1.5163
46	2015	30	24.8571	74.5102	917.1745	6.3776	1.3776
86	2015	30	22.8857	73.9184	864.0633	4.6347	0.9286
2	2015	30	22.8857	73.9184	864.0633	4.6347	0.9286
4	2015	30	24.2714	73.7755	898.6439	4.5592	1.4776
47	2015	30	26.3286	78.6735	953.8908	5.8571	0.7408
82	2015	30	23.0429	76.2449	872.5347	3.2551	2.3969
19	2015	30	26.8286	74.9898	955.4163	6.3041	1.0755
20	2015	30	22.3286	71.5306	852.3418	4.3194	1.8918
80	2015	30	23.0429	76.2449	872.5347	3.2551	2.3969
3	2015	30	26.2143	76.2449	940.2908	5.3561	1.0684
52	2015	30	23.3571	82.2959	898.9398	3.8051	2.1949
70	2015	30	24.9143	72.2653	906.8531	6.0429	1.2469
64	2015	30	17.2000	77.7551	776.3214	3.4031	1.9796
48	2015	30	25.9143	77.0102	928.7959	6.1163	0.8837
65	2015	30	23.3571	82.2959	898.9398	3.8051	2.1949
44	2015	30	24.9143	72.2653	906.8531	6.0429	1.2469
75	2015	30	17.2000	77.7551	776.3214	3.4031	1.9796
40	2015	30	25.6857	77.6122	940.5888	6.4459	1.2061
11	2015	30	23.1714	74.7755	875.2265	4.5898	1.5163
35	2015	30	25.2429	77.1224	934.6878	4.9612	1.3031
78	2015	30	24.0429	76.0612	896.4265	5.0531	1.3816
28	2015	30	25.1429	75.0612	924.8755	5.2816	1.4816
39	2015	30	23.3571	82.2959	898.9398	3.8051	2.1949
24	2015	30	25.6857	78.0918	935.6010	4.7153	1.1469
63	2015	30	25.6857	77.6122	940.5888	6.4459	1.2061
62	2015	30	23.4429	76.0816	872.1571	4.0459	0.9071
1	2015	30	23.0429	76.2449	872.5347	3.2551	2.3969
31	2015	31	21.0286	75.4184	845.3378	3.4255	1.1102
79	2015	31	27.0429	75.3878	965.0061	5.2122	1.1796
51	2015	31	24.9143	76.0102	935.6735	4.7857	1.4337
14	2015	31	21.4000	82.8980	895.1990	2.9204	1.8235
67	2015	31	21.9857	85.9388	899.8418	3.2153	2.0224
42	2015	31	21.5429	80.8571	873.3041	2.4449	2.1755
50	2015	31	24.8714	73.6939	899.3122	4.9357	1.4173

43	2015	31	21.5429	80.8571	873.3041	2.4449	2.1755
85	2015	31	26.1429	71.0408	907.4531	6.8980	1.5235
25	2015	31	25.8143	79.9184	972.6469	4.9969	1.1898
69	2015	31	24.8571	79.8163	936.5020	4.3459	1.2041
57	2015	31	23.0143	70.5816	884.7439	3.9092	2.1500
9	2015	31	20.4857	76.4082	853.0429	3.5031	1.7684
72	2015	31	21.2857	79.6122	876.0510	3.3694	1.3153
26	2015	31	21.0714	79.7959	865.9122	3.8663	1.3184
7	2015	31	20.5857	80.0612	858.2276	2.3776	1.2418
83	2015	31	24.9714	82.0612	941.3347	4.3000	1.0735
76	2015	31	24.9429	76.3571	918.0071	5.1806	1.3173
36	2015	31	24.5857	76.8163	925.8184	4.6347	1.5265
81	2015	31	24.9143	76.0102	935.6735	4.7857	1.4337
15	2015	31	26.9429	77.0714	929.5010	5.2531	0.9429
32	2015	31	21.5429	80.8571	873.3041	2.4449	2.1755
73	2015	31	27.4286	72.7551	957.5663	3.9939	1.2296
71	2015	31	24.5857	76.8163	925.8184	4.6347	1.5265
41	2015	31	22.9714	77.3980	872.8276	3.7673	1.1235
10	2015	31	25.2429	82.3980	959.9286	5.1908	1.1786
23	2015	31	17.2143	77.3776	776.8918	3.1602	1.9714
27	2015	31	20.5857	80.0612	858.2276	2.3776	1.2418
60	2015	31	24.9143	76.0102	935.6735	4.7857	1.4337
53	2015	31	20.4857	76.4082	853.0429	3.5031	1.7684
66	2015	31	21.4000	82.8980	895.1990	2.9204	1.8235
59	2015	31	23.0143	70.5816	884.7439	3.9092	2.1500
61	2015	31	27.4286	72.7551	957.5663	3.9939	1.2296
84	2015	31	27.4286	72.7551	957.5663	3.9939	1.2296
38	2015	31	23.0143	70.5816	884.7439	3.9092	2.1500
87	2015	31	23.2000	80.2857	897.2653	4.0194	0.9867
34	2015	31	23.0143	70.5816	884.7439	3.9092	2.1500
29	2015	31	24.8571	79.8163	936.5020	4.3459	1.2041
5	2015	31	18.8429	80.3163	831.8990	3.1694	1.1551
8	2015	31	20.4857	76.4082	853.0429	3.5031	1.7684
12	2015	31	18.8429	80.3163	831.8990	3.1694	1.1551
13	2015	31	24.9714	82.0612	941.3347	4.3000	1.0735
18	2015	31	26.5714	79.6633	959.5867	4.5490	1.0214
33	2015	31	24.8714	73.6939	899.3122	4.9357	1.4173
56	2015	31	25.8143	79.9184	972.6469	4.9969	1.1898
77	2015	31	26.9429	77.0714	929.5010	5.2531	0.9429
54	2015	31	18.8429	80.3163	831.8990	3.1694	1.1551
21	2015	31	24.8714	73.6939	899.3122	4.9357	1.4173
68	2015	31	27.0429	75.3878	965.0061	5.2122	1.1796
74	2015	31	27.4286	72.7551	957.5663	3.9939	1.2296
88	2015	31	21.5429	80.8571	873.3041	2.4449	2.1755
16	2015	31	24.9429	76.3571	918.0071	5.1806	1.3173
30	2015	31	21.4000	82.8980	895.1990	2.9204	1.8235
6	2015	31	27.0429	75.3878	965.0061	5.2122	1.1796
49	2015	31	24.8571	79.8163	936.5020	4.3459	1.2041
22	2015	31	21.5429	80.8571	873.3041	2.4449	2.1755
45	2015	31	18.4429	77.7449	817.4367	3.0173	1.1714

58	2015	31	24.8571	79.8163	936.5020	4.3459	1.2041
37	2015	31	27.0429	75.3878	965.0061	5.2122	1.1796
17	2015	31	21.9857	85.9388	899.8418	3.2153	2.0224
55	2015	31	21.2857	79.6122	876.0510	3.3694	1.3153
46	2015	31	24.9429	76.3571	918.0071	5.1806	1.3173
86	2015	31	21.8143	75.3265	864.7071	4.2296	0.9939
2	2015	31	21.8143	75.3265	864.7071	4.2296	0.9939
4	2015	31	24.8714	73.6939	899.3122	4.9357	1.4173
47	2015	31	25.1857	83.0612	955.0796	3.7367	0.7398
82	2015	31	21.5429	80.8571	873.3041	2.4449	2.1755
19	2015	31	24.8714	79.6327	956.7571	4.2622	1.0612
20	2015	31	20.4857	76.4082	853.0429	3.5031	1.7684
80	2015	31	21.5429	80.8571	873.3041	2.4449	2.1755
3	2015	31	24.9714	82.0612	941.3347	4.3000	1.0735
52	2015	31	21.9857	85.9388	899.8418	3.2153	2.0224
70	2015	31	26.1429	71.0408	907.4531	6.8980	1.5235
64	2015	31	17.2143	77.3776	776.8918	3.1602	1.9714
48	2015	31	26.9429	77.0714	929.5010	5.2531	0.9429
65	2015	31	21.9857	85.9388	899.8418	3.2153	2.0224
44	2015	31	26.1429	71.0408	907.4531	6.8980	1.5235
75	2015	31	17.2143	77.3776	776.8918	3.1602	1.9714
40	2015	31	24.4714	80.1633	941.8714	5.2112	1.2571
11	2015	31	21.2857	79.6122	876.0510	3.3694	1.3153
35	2015	31	24.9143	76.0102	935.6735	4.7857	1.4337
78	2015	31	23.2000	80.2857	897.2653	4.0194	0.9867
28	2015	31	24.5857	76.8163	925.8184	4.6347	1.5265
39	2015	31	21.9857	85.9388	899.8418	3.2153	2.0224
24	2015	31	24.8571	79.8163	936.5020	4.3459	1.2041
63	2015	31	24.4714	80.1633	941.8714	5.2112	1.2571
62	2015	31	22.9714	77.3980	872.8276	3.7673	1.1235
1	2015	31	21.5429	80.8571	873.3041	2.4449	2.1755
31	2015	32	20.3143	78.6939	845.9051	4.6408	1.0990
79	2015	32	27.9286	72.3265	966.4857	6.5418	1.3633
51	2015	32	25.4000	74.2857	937.2653	6.1327	1.5367
14	2015	32	22.4000	87.0612	896.9102	2.4776	2.2786
67	2015	32	22.7571	89.2041	901.4173	3.2235	2.6694
42	2015	32	22.1714	85.0408	874.5908	3.2908	2.2214
50	2015	32	24.1143	70.6837	900.2031	6.0561	2.0173
43	2015	32	22.1714	85.0408	874.5908	3.2908	2.2214
85	2015	32	24.3857	67.7755	908.2806	8.3653	2.4122
25	2015	32	27.1143	80.0510	974.7490	4.6388	1.1643
69	2015	32	25.4429	78.7143	937.9714	5.1561	1.3551
57	2015	32	22.7571	69.4796	885.8010	5.0173	3.0551
9	2015	32	20.5143	83.4286	854.1990	3.7735	2.1827
72	2015	32	21.5857	84.1224	877.4459	3.1000	1.4265
26	2015	32	21.1286	87.5204	867.0388	3.5541	1.4500
7	2015	32	20.5429	85.7449	859.3398	2.0663	1.4643
83	2015	32	25.1714	87.2755	943.1010	4.2265	1.0133
76	2015	32	25.1571	74.7449	919.2173	5.6367	1.4765
36	2015	32	25.0429	75.7143	927.4480	4.8714	1.6939

81	2015	32	25.4000	74.2857	937.2653	6.1327	1.5367
15	2015	32	25.9000	72.9184	930.4878	5.9031	1.1898
32	2015	32	22.1714	85.0408	874.5908	3.2908	2.2214
73	2015	32	27.8429	70.3061	958.9245	4.5908	1.4918
71	2015	32	25.0429	75.7143	927.4480	4.8714	1.6939
41	2015	32	22.6714	77.7347	873.7806	4.4643	1.8449
10	2015	32	26.2857	79.9796	961.6990	6.5939	1.1592
23	2015	32	16.6571	80.1531	777.2969	4.2337	2.0245
27	2015	32	20.5429	85.7449	859.3398	2.0663	1.4643
60	2015	32	25.4000	74.2857	937.2653	6.1327	1.5367
53	2015	32	20.5143	83.4286	854.1990	3.7735	2.1827
66	2015	32	22.4000	87.0612	896.9102	2.4776	2.2786
59	2015	32	22.7571	69.4796	885.8010	5.0173	3.0551
61	2015	32	27.8429	70.3061	958.9245	4.5908	1.4918
84	2015	32	27.8429	70.3061	958.9245	4.5908	1.4918
38	2015	32	22.7571	69.4796	885.8010	5.0173	3.0551
87	2015	32	23.4286	83.8673	898.7582	3.7133	1.0653
34	2015	32	22.7571	69.4796	885.8010	5.0173	3.0551
29	2015	32	25.4429	78.7143	937.9714	5.1561	1.3551
5	2015	32	18.7857	86.7857	832.7969	3.3776	1.3347
8	2015	32	20.5143	83.4286	854.1990	3.7735	2.1827
12	2015	32	18.7857	86.7857	832.7969	3.3776	1.3347
13	2015	32	25.1714	87.2755	943.1010	4.2265	1.0133
18	2015	32	27.1286	76.7653	960.8888	6.1112	1.2551
33	2015	32	24.1143	70.6837	900.2031	6.0561	2.0173
56	2015	32	27.1143	80.0510	974.7490	4.6388	1.1643
77	2015	32	25.9000	72.9184	930.4878	5.9031	1.1898
54	2015	32	18.7857	86.7857	832.7969	3.3776	1.3347
21	2015	32	24.1143	70.6837	900.2031	6.0561	2.0173
68	2015	32	27.9286	72.3265	966.4857	6.5418	1.3633
74	2015	32	27.8429	70.3061	958.9245	4.5908	1.4918
88	2015	32	22.1714	85.0408	874.5908	3.2908	2.2214
16	2015	32	25.1571	74.7449	919.2173	5.6367	1.4765
30	2015	32	22.4000	87.0612	896.9102	2.4776	2.2786
6	2015	32	27.9286	72.3265	966.4857	6.5418	1.3633
49	2015	32	25.4429	78.7143	937.9714	5.1561	1.3551
22	2015	32	22.1714	85.0408	874.5908	3.2908	2.2214
45	2015	32	18.5286	83.3673	818.1908	3.0337	1.2429
58	2015	32	25.4429	78.7143	937.9714	5.1561	1.3551
37	2015	32	27.9286	72.3265	966.4857	6.5418	1.3633
17	2015	32	22.7571	89.2041	901.4173	3.2235	2.6694
55	2015	32	21.5857	84.1224	877.4459	3.1000	1.4265
46	2015	32	25.1571	74.7449	919.2173	5.6367	1.4765
86	2015	32	21.2286	78.5102	865.7061	4.6255	1.5184
2	2015	32	21.2286	78.5102	865.7061	4.6255	1.5184
4	2015	32	24.1143	70.6837	900.2031	6.0561	2.0173
47	2015	32	25.8429	86.5510	957.0673	2.4469	0.6888
82	2015	32	22.1714	85.0408	874.5908	3.2908	2.2214
19	2015	32	26.5000	83.9796	958.8888	2.8265	1.0959
20	2015	32	20.5143	83.4286	854.1990	3.7735	2.1827

80	2015	32	22.1714	85.0408	874.5908	3.2908	2.2214
3	2015	32	25.1714	87.2755	943.1010	4.2265	1.0133
52	2015	32	22.7571	89.2041	901.4173	3.2235	2.6694
70	2015	32	24.3857	67.7755	908.2806	8.3653	2.4122
64	2015	32	16.6571	80.1531	777.2969	4.2337	2.0245
48	2015	32	25.9000	72.9184	930.4878	5.9031	1.1898
65	2015	32	22.7571	89.2041	901.4173	3.2235	2.6694
44	2015	32	24.3857	67.7755	908.2806	8.3653	2.4122
75	2015	32	16.6571	80.1531	777.2969	4.2337	2.0245
40	2015	32	25.5429	80.0510	943.8357	5.7806	1.3878
11	2015	32	21.5857	84.1224	877.4459	3.1000	1.4265
35	2015	32	25.4000	74.2857	937.2653	6.1327	1.5367
78	2015	32	23.4286	83.8673	898.7582	3.7133	1.0653
28	2015	32	25.0429	75.7143	927.4480	4.8714	1.6939
39	2015	32	22.7571	89.2041	901.4173	3.2235	2.6694
24	2015	32	25.4429	78.7143	937.9714	5.1561	1.3551
63	2015	32	25.5429	80.0510	943.8357	5.7806	1.3878
62	2015	32	22.6714	77.7347	873.7806	4.4643	1.8449
1	2015	32	22.1714	85.0408	874.5908	3.2908	2.2214
31	2015	33	21.5714	84.7449	846.3888	3.3163	0.9031
79	2015	33	26.5571	71.0408	966.7143	7.8245	1.5051
51	2015	33	24.8143	74.9490	937.4153	6.8735	1.4276
14	2015	33	24.0429	83.9388	897.0224	3.3122	1.8418
67	2015	33	23.5857	87.5000	901.5949	4.2490	2.7276
42	2015	33	23.3571	84.3980	874.8735	3.9755	2.2878
50	2015	33	24.3714	73.7755	900.6837	4.2061	2.0122
43	2015	33	23.3571	84.3980	874.8735	3.9755	2.2878
85	2015	33	25.1143	73.3980	908.8592	6.4378	2.2704
25	2015	33	27.8000	77.0408	974.5173	6.1786	1.2673
69	2015	33	26.0143	77.2959	938.1827	5.9143	1.3051
57	2015	33	23.3714	72.0000	886.1643	4.3214	2.7602
9	2015	33	22.2571	84.6531	854.5214	4.0929	2.0735
72	2015	33	23.7000	83.8878	877.6867	3.5571	1.3908
26	2015	33	22.9571	87.9286	867.3439	3.8786	1.5041
7	2015	33	22.7286	86.7143	859.6571	2.4418	1.3918
83	2015	33	27.1286	85.5612	943.2327	4.9163	0.9520
76	2015	33	24.6429	75.6122	919.5480	5.5571	1.5949
36	2015	33	25.1000	75.8776	927.6133	5.5398	1.6327
81	2015	33	24.8143	74.9490	937.4153	6.8735	1.4276
15	2015	33	24.9571	78.2551	931.1184	4.7571	1.1000
32	2015	33	23.3571	84.3980	874.8735	3.9755	2.2878
73	2015	33	27.2429	68.6633	959.2276	5.3520	1.4265
71	2015	33	25.1000	75.8776	927.6133	5.5398	1.6327
41	2015	33	23.2857	81.3878	874.1898	3.2173	1.7204
10	2015	33	25.7571	78.4898	961.7143	7.8133	1.1816
23	2015	33	17.8143	85.2143	777.6031	3.6704	1.8255
27	2015	33	22.7286	86.7143	859.6571	2.4418	1.3918
60	2015	33	24.8143	74.9490	937.4153	6.8735	1.4276
53	2015	33	22.2571	84.6531	854.5214	4.0929	2.0735
66	2015	33	24.0429	83.9388	897.0224	3.3122	1.8418

59	2015	33	23.3714	72.0000	886.1643	4.3214	2.7602
61	2015	33	27.2429	68.6633	959.2276	5.3520	1.4265
84	2015	33	27.2429	68.6633	959.2276	5.3520	1.4265
38	2015	33	23.3714	72.0000	886.1643	4.3214	2.7602
87	2015	33	25.1429	82.9694	898.9847	4.8092	1.2959
34	2015	33	23.3714	72.0000	886.1643	4.3214	2.7602
29	2015	33	26.0143	77.2959	938.1827	5.9143	1.3051
5	2015	33	20.6857	89.9286	833.1306	2.8122	1.1602
8	2015	33	22.2571	84.6531	854.5214	4.0929	2.0735
12	2015	33	20.6857	89.9286	833.1306	2.8122	1.1602
13	2015	33	27.1286	85.5612	943.2327	4.9163	0.9520
18	2015	33	25.9857	75.8980	961.2449	6.8163	1.4337
33	2015	33	24.3714	73.7755	900.6837	4.2061	2.0122
56	2015	33	27.8000	77.0408	974.5173	6.1786	1.2673
77	2015	33	24.9571	78.2551	931.1184	4.7571	1.1000
54	2015	33	20.6857	89.9286	833.1306	2.8122	1.1602
21	2015	33	24.3714	73.7755	900.6837	4.2061	2.0122
68	2015	33	26.5571	71.0408	966.7143	7.8245	1.5051
74	2015	33	27.2429	68.6633	959.2276	5.3520	1.4265
88	2015	33	23.3571	84.3980	874.8735	3.9755	2.2878
16	2015	33	24.6429	75.6122	919.5480	5.5571	1.5949
30	2015	33	24.0429	83.9388	897.0224	3.3122	1.8418
6	2015	33	26.5571	71.0408	966.7143	7.8245	1.5051
49	2015	33	26.0143	77.2959	938.1827	5.9143	1.3051
22	2015	33	23.3571	84.3980	874.8735	3.9755	2.2878
45	2015	33	20.1286	86.4388	818.5245	2.8010	1.2480
58	2015	33	26.0143	77.2959	938.1827	5.9143	1.3051
37	2015	33	26.5571	71.0408	966.7143	7.8245	1.5051
17	2015	33	23.5857	87.5000	901.5949	4.2490	2.7276
55	2015	33	23.7000	83.8878	877.6867	3.5571	1.3908
46	2015	33	24.6429	75.6122	919.5480	5.5571	1.5949
86	2015	33	22.7429	81.9082	866.1082	3.8245	1.4367
2	2015	33	22.7429	81.9082	866.1082	3.8245	1.4367
4	2015	33	24.3714	73.7755	900.6837	4.2061	2.0122
47	2015	33	27.8000	83.9388	957.0000	4.5684	0.5612
82	2015	33	23.3571	84.3980	874.8735	3.9755	2.2878
19	2015	33	27.6714	81.2245	958.7041	4.9816	1.1408
20	2015	33	22.2571	84.6531	854.5214	4.0929	2.0735
80	2015	33	23.3571	84.3980	874.8735	3.9755	2.2878
3	2015	33	27.1286	85.5612	943.2327	4.9163	0.9520
52	2015	33	23.5857	87.5000	901.5949	4.2490	2.7276
70	2015	33	25.1143	73.3980	908.8592	6.4378	2.2704
64	2015	33	17.8143	85.2143	777.6031	3.6704	1.8255
48	2015	33	24.9571	78.2551	931.1184	4.7571	1.1000
65	2015	33	23.5857	87.5000	901.5949	4.2490	2.7276
44	2015	33	25.1143	73.3980	908.8592	6.4378	2.2704
75	2015	33	17.8143	85.2143	777.6031	3.6704	1.8255
40	2015	33	25.6571	78.3776	943.7153	7.4102	1.2878
11	2015	33	23.7000	83.8878	877.6867	3.5571	1.3908
35	2015	33	24.8143	74.9490	937.4153	6.8735	1.4276

78	2015	33	25.1429	82.9694	898.9847	4.8092	1.2959
28	2015	33	25.1000	75.8776	927.6133	5.5398	1.6327
39	2015	33	23.5857	87.5000	901.5949	4.2490	2.7276
24	2015	33	26.0143	77.2959	938.1827	5.9143	1.3051
63	2015	33	25.6571	78.3776	943.7153	7.4102	1.2878
62	2015	33	23.2857	81.3878	874.1898	3.2173	1.7204
1	2015	33	23.3571	84.3980	874.8735	3.9755	2.2878
31	2015	34	18.9429	86.7143	846.5378	2.0316	0.8531
79	2015	34	25.7571	78.3571	966.3704	4.8286	1.3378
51	2015	34	24.1286	80.5000	936.8296	4.3663	1.3000
14	2015	34	22.5429	83.1020	896.2163	3.4194	1.7061
67	2015	34	22.4143	87.8878	900.8673	3.5929	2.3347
42	2015	34	21.5714	83.7551	874.3806	3.6520	2.0622
50	2015	34	21.9143	80.5918	900.6469	2.3959	1.5061
43	2015	34	21.5714	83.7551	874.3806	3.6520	2.0622
85	2015	34	22.9000	80.2755	908.9173	4.0173	1.5327
25	2015	34	26.4000	79.3776	973.5102	5.4510	1.1990
69	2015	34	24.1429	80.3571	937.6265	4.6082	1.1551
57	2015	34	21.2000	76.8878	885.8929	3.0714	1.9816
9	2015	34	20.2143	81.8265	854.0969	3.9133	1.8051
72	2015	34	21.6286	80.9286	877.0878	4.1388	1.5837
26	2015	34	22.0143	83.1224	866.8929	4.7143	1.6408
7	2015	34	21.1286	82.3571	859.1959	3.3429	1.3694
83	2015	34	25.3143	82.0102	942.3939	5.5133	0.9531
76	2015	34	23.1286	81.7755	919.2551	3.3878	1.5092
36	2015	34	23.7857	81.4082	926.9388	3.9612	1.2704
81	2015	34	24.1286	80.5000	936.8296	4.3663	1.3000
15	2015	34	23.4429	88.6327	931.1602	2.4918	0.8010
32	2015	34	21.5714	83.7551	874.3806	3.6520	2.0622
73	2015	34	25.3571	76.1735	958.9143	3.9102	1.0286
71	2015	34	23.7857	81.4082	926.9388	3.9612	1.2704
41	2015	34	21.5000	84.2449	873.9694	2.5816	1.2265
10	2015	34	24.7429	84.7551	961.0939	4.8663	1.0357
23	2015	34	15.5286	85.9796	777.6929	3.0418	1.7806
27	2015	34	21.1286	82.3571	859.1959	3.3429	1.3694
60	2015	34	24.1286	80.5000	936.8296	4.3663	1.3000
53	2015	34	20.2143	81.8265	854.0969	3.9133	1.8051
66	2015	34	22.5429	83.1020	896.2163	3.4194	1.7061
59	2015	34	21.2000	76.8878	885.8929	3.0714	1.9816
61	2015	34	25.3571	76.1735	958.9143	3.9102	1.0286
84	2015	34	25.3571	76.1735	958.9143	3.9102	1.0286
38	2015	34	21.2000	76.8878	885.8929	3.0714	1.9816
87	2015	34	22.4857	81.9184	898.3306	5.1602	1.4878
34	2015	34	21.2000	76.8878	885.8929	3.0714	1.9816
29	2015	34	24.1429	80.3571	937.6265	4.6082	1.1551
5	2015	34	19.2286	86.1633	832.8276	3.1357	1.0786
8	2015	34	20.2143	81.8265	854.0969	3.9133	1.8051
12	2015	34	19.2286	86.1633	832.8276	3.1357	1.0786
13	2015	34	25.3143	82.0102	942.3939	5.5133	0.9531
18	2015	34	25.2286	82.7857	961.0694	4.1367	1.1551

33	2015	34	21.9143	80.5918	900.6469	2.3959	1.5061
56	2015	34	26.4000	79.3776	973.5102	5.4510	1.1990
77	2015	34	23.4429	88.6327	931.1602	2.4918	0.8010
54	2015	34	19.2286	86.1633	832.8276	3.1357	1.0786
21	2015	34	21.9143	80.5918	900.6469	2.3959	1.5061
68	2015	34	25.7571	78.3571	966.3704	4.8286	1.3378
74	2015	34	25.3571	76.1735	958.9143	3.9102	1.0286
88	2015	34	21.5714	83.7551	874.3806	3.6520	2.0622
16	2015	34	23.1286	81.7755	919.2551	3.3878	1.5092
30	2015	34	22.5429	83.1020	896.2163	3.4194	1.7061
6	2015	34	25.7571	78.3571	966.3704	4.8286	1.3378
49	2015	34	24.1429	80.3571	937.6265	4.6082	1.1551
22	2015	34	21.5714	83.7551	874.3806	3.6520	2.0622
45	2015	34	18.8286	83.0408	818.2939	3.8704	1.4020
58	2015	34	24.1429	80.3571	937.6265	4.6082	1.1551
37	2015	34	25.7571	78.3571	966.3704	4.8286	1.3378
17	2015	34	22.4143	87.8878	900.8673	3.5929	2.3347
55	2015	34	21.6286	80.9286	877.0878	4.1388	1.5837
46	2015	34	23.1286	81.7755	919.2551	3.3878	1.5092
86	2015	34	20.4429	82.0204	865.8143	3.5092	1.0490
2	2015	34	20.4429	82.0204	865.8143	3.5092	1.0490
4	2015	34	21.9143	80.5918	900.6469	2.3959	1.5061
47	2015	34	25.8714	81.4592	955.9755	5.1918	0.5724
82	2015	34	21.5714	83.7551	874.3806	3.6520	2.0622
19	2015	34	25.9857	81.1735	957.6214	4.9776	1.0265
20	2015	34	20.2143	81.8265	854.0969	3.9133	1.8051
80	2015	34	21.5714	83.7551	874.3806	3.6520	2.0622
3	2015	34	25.3143	82.0102	942.3939	5.5133	0.9531
52	2015	34	22.4143	87.8878	900.8673	3.5929	2.3347
70	2015	34	22.9000	80.2755	908.9173	4.0173	1.5327
64	2015	34	15.5286	85.9796	777.6929	3.0418	1.7806
48	2015	34	23.4429	88.6327	931.1602	2.4918	0.8010
65	2015	34	22.4143	87.8878	900.8673	3.5929	2.3347
44	2015	34	22.9000	80.2755	908.9173	4.0173	1.5327
75	2015	34	15.5286	85.9796	777.6929	3.0418	1.7806
40	2015	34	24.5286	82.1429	942.8816	5.7224	1.2194
11	2015	34	21.6286	80.9286	877.0878	4.1388	1.5837
35	2015	34	24.1286	80.5000	936.8296	4.3663	1.3000
78	2015	34	22.4857	81.9184	898.3306	5.1602	1.4878
28	2015	34	23.7857	81.4082	926.9388	3.9612	1.2704
39	2015	34	22.4143	87.8878	900.8673	3.5929	2.3347
24	2015	34	24.1429	80.3571	937.6265	4.6082	1.1551
63	2015	34	24.5286	82.1429	942.8816	5.7224	1.2194
62	2015	34	21.5000	84.2449	873.9694	2.5816	1.2265
1	2015	34	21.5714	83.7551	874.3806	3.6520	2.0622
31	2015	35	17.9857	84.7449	846.8878	2.1173	0.8827
79	2015	35	23.3714	81.5714	967.1388	3.6214	1.1551
51	2015	35	21.3857	81.9286	937.4551	3.6602	1.3000
14	2015	35	20.4286	82.3673	896.5224	3.2204	1.5276
67	2015	35	19.7286	88.5816	901.2776	3.2673	2.4724

42	2015	35	18.9000	84.8571	874.7551	2.7663	1.9684
50	2015	35	21.0571	81.1327	901.1949	3.0020	1.4969
43	2015	35	18.9000	84.8571	874.7551	2.7663	1.9684
85	2015	35	21.1286	80.6735	909.4847	3.7959	1.3500
25	2015	35	23.8143	82.2551	974.2327	4.2418	1.0092
69	2015	35	21.9714	82.8776	938.2929	3.9327	1.0245
57	2015	35	18.8000	79.4694	886.4153	3.0837	1.6959
9	2015	35	18.5286	81.0102	854.3704	3.2337	1.8173
72	2015	35	19.9571	80.8673	877.3847	3.4204	1.6520
26	2015	35	20.4857	82.2143	867.0398	3.7378	1.6459
7	2015	35	19.5571	81.6429	859.3622	2.9173	1.4122
83	2015	35	23.9714	82.3878	942.8276	4.5510	0.9724
76	2015	35	20.9429	83.2551	919.8888	2.9633	1.5041
36	2015	35	21.3857	82.7245	927.4755	3.3694	1.2469
81	2015	35	21.3857	81.9286	937.4551	3.6602	1.3000
15	2015	35	22.5571	88.4592	931.7510	2.4163	0.7429
32	2015	35	18.9000	84.8571	874.7551	2.7663	1.9684
73	2015	35	23.7429	78.9796	959.6796	3.5867	0.9786
71	2015	35	21.3857	82.7245	927.4755	3.3694	1.2469
41	2015	35	19.0857	85.3469	874.4327	2.6439	0.9704
10	2015	35	22.4714	86.3061	961.8276	3.9490	0.8837
23	2015	35	14.9857	84.0408	777.7663	2.7582	1.9459
27	2015	35	19.5571	81.6429	859.3622	2.9173	1.4122
60	2015	35	21.3857	81.9286	937.4551	3.6602	1.3000
53	2015	35	18.5286	81.0102	854.3704	3.2337	1.8173
66	2015	35	20.4286	82.3673	896.5224	3.2204	1.5276
59	2015	35	18.8000	79.4694	886.4153	3.0837	1.6959
61	2015	35	23.7429	78.9796	959.6796	3.5867	0.9786
84	2015	35	23.7429	78.9796	959.6796	3.5867	0.9786
38	2015	35	18.8000	79.4694	886.4153	3.0837	1.6959
87	2015	35	20.6429	84.9388	898.7531	3.6092	1.3592
34	2015	35	18.8000	79.4694	886.4153	3.0837	1.6959
29	2015	35	21.9714	82.8776	938.2929	3.9327	1.0245
5	2015	35	17.8857	84.3265	832.9735	3.1245	1.0765
8	2015	35	18.5286	81.0102	854.3704	3.2337	1.8173
12	2015	35	17.8857	84.3265	832.9735	3.1245	1.0765
13	2015	35	23.9714	82.3878	942.8276	4.5510	0.9724
18	2015	35	23.4571	83.5612	961.7510	3.7306	0.9296
33	2015	35	21.0571	81.1327	901.1949	3.0020	1.4969
56	2015	35	23.8143	82.2551	974.2327	4.2418	1.0092
77	2015	35	22.5571	88.4592	931.7510	2.4163	0.7429
54	2015	35	17.8857	84.3265	832.9735	3.1245	1.0765
21	2015	35	21.0571	81.1327	901.1949	3.0020	1.4969
68	2015	35	23.3714	81.5714	967.1388	3.6214	1.1551
74	2015	35	23.7429	78.9796	959.6796	3.5867	0.9786
88	2015	35	18.9000	84.8571	874.7551	2.7663	1.9684
16	2015	35	20.9429	83.2551	919.8888	2.9633	1.5041
30	2015	35	20.4286	82.3673	896.5224	3.2204	1.5276
6	2015	35	23.3714	81.5714	967.1388	3.6214	1.1551
49	2015	35	21.9714	82.8776	938.2929	3.9327	1.0245

22	2015	35	18.9000	84.8571	874.7551	2.7663	1.9684
45	2015	35	17.6571	81.2449	818.3673	3.5949	1.4684
58	2015	35	21.9714	82.8776	938.2929	3.9327	1.0245
37	2015	35	23.3714	81.5714	967.1388	3.6214	1.1551
17	2015	35	19.7286	88.5816	901.2776	3.2673	2.4724
55	2015	35	19.9571	80.8673	877.3847	3.4204	1.6520
46	2015	35	20.9429	83.2551	919.8888	2.9633	1.5041
86	2015	35	19.0571	81.9796	866.1969	2.9449	0.8796
2	2015	35	19.0571	81.9796	866.1969	2.9449	0.8796
4	2015	35	21.0571	81.1327	901.1949	3.0020	1.4969
47	2015	35	24.3286	82.4184	956.5367	3.3429	0.6071
82	2015	35	18.9000	84.8571	874.7551	2.7663	1.9684
19	2015	35	24.0286	83.2857	958.1561	3.5378	0.9143
20	2015	35	18.5286	81.0102	854.3704	3.2337	1.8173
80	2015	35	18.9000	84.8571	874.7551	2.7663	1.9684
3	2015	35	23.9714	82.3878	942.8276	4.5510	0.9724
52	2015	35	19.7286	88.5816	901.2776	3.2673	2.4724
70	2015	35	21.1286	80.6735	909.4847	3.7959	1.3500
64	2015	35	14.9857	84.0408	777.7663	2.7582	1.9459
48	2015	35	22.5571	88.4592	931.7510	2.4163	0.7429
65	2015	35	19.7286	88.5816	901.2776	3.2673	2.4724
44	2015	35	21.1286	80.6735	909.4847	3.7959	1.3500
75	2015	35	14.9857	84.0408	777.7663	2.7582	1.9459
40	2015	35	22.2000	83.3163	943.4551	4.5724	1.2684
11	2015	35	19.9571	80.8673	877.3847	3.4204	1.6520
35	2015	35	21.3857	81.9286	937.4551	3.6602	1.3000
78	2015	35	20.6429	84.9388	898.7531	3.6092	1.3592
28	2015	35	21.3857	82.7245	927.4755	3.3694	1.2469
39	2015	35	19.7286	88.5816	901.2776	3.2673	2.4724
24	2015	35	21.9714	82.8776	938.2929	3.9327	1.0245
63	2015	35	22.2000	83.3163	943.4551	4.5724	1.2684
62	2015	35	19.0857	85.3469	874.4327	2.6439	0.9704
1	2015	35	18.9000	84.8571	874.7551	2.7663	1.9684
31	2015	36	20.5000	83.5918	847.6806	2.5245	0.8378
79	2015	36	25.3857	82.5306	968.5204	3.9888	0.9010
51	2015	36	23.9857	82.9694	938.7245	4.0643	1.2439
14	2015	36	21.9429	82.8673	897.4500	3.4510	1.1694
67	2015	36	22.2143	88.3776	902.3898	3.5653	2.4510
42	2015	36	21.9429	85.2755	875.7245	3.0133	2.1286
50	2015	36	22.9429	78.2653	902.3194	3.6755	1.4612
43	2015	36	21.9429	85.2755	875.7245	3.0133	2.1286
85	2015	36	24.0000	79.0918	910.5786	4.0286	1.3765
25	2015	36	25.7714	84.5918	975.5367	3.5806	0.8429
69	2015	36	24.3857	83.7551	939.6439	3.5929	0.9255
57	2015	36	21.9857	79.0816	887.4847	3.8245	1.7786
9	2015	36	20.8571	80.4796	855.2245	3.8031	1.9071
72	2015	36	21.6714	81.9184	878.2531	3.4918	1.3990
26	2015	36	21.9000	82.0918	867.8327	3.1184	1.4510
7	2015	36	21.3714	81.9184	860.2061	2.8449	1.3296
83	2015	36	25.2000	84.4082	943.8592	4.6020	0.9582

76	2015	36	23.5000	82.1429	921.1847	3.6561	1.4837
36	2015	36	23.7286	83.6531	928.6816	3.7969	1.3214
81	2015	36	23.9857	82.9694	938.7245	4.0643	1.2439
15	2015	36	24.1857	85.1429	933.0051	2.9622	0.7153
32	2015	36	21.9429	85.2755	875.7245	3.0133	2.1286
73	2015	36	25.7286	78.7449	961.1184	3.5439	1.0510
71	2015	36	23.7286	83.6531	928.6816	3.7969	1.3214
41	2015	36	22.1000	84.8571	875.4704	3.1490	0.8020
10	2015	36	25.0714	85.9694	963.1041	4.6347	0.8694
23	2015	36	17.0429	84.4796	778.2939	1.7929	1.7786
27	2015	36	21.3714	81.9184	860.2061	2.8449	1.3296
60	2015	36	23.9857	82.9694	938.7245	4.0643	1.2439
53	2015	36	20.8571	80.4796	855.2245	3.8031	1.9071
66	2015	36	21.9429	82.8673	897.4500	3.4510	1.1694
59	2015	36	21.9857	79.0816	887.4847	3.8245	1.7786
61	2015	36	25.7286	78.7449	961.1184	3.5439	1.0510
84	2015	36	25.7286	78.7449	961.1184	3.5439	1.0510
38	2015	36	21.9857	79.0816	887.4847	3.8245	1.7786
87	2015	36	22.8286	85.8367	899.7735	3.3908	1.2347
34	2015	36	21.9857	79.0816	887.4847	3.8245	1.7786
29	2015	36	24.3857	83.7551	939.6439	3.5929	0.9255
5	2015	36	19.8429	83.7449	833.7378	3.0061	1.0337
8	2015	36	20.8571	80.4796	855.2245	3.8031	1.9071
12	2015	36	19.8429	83.7449	833.7378	3.0061	1.0337
13	2015	36	25.2000	84.4082	943.8592	4.6020	0.9582
18	2015	36	25.3429	82.5102	963.0929	4.2694	0.9041
33	2015	36	22.9429	78.2653	902.3194	3.6755	1.4612
56	2015	36	25.7714	84.5918	975.5367	3.5806	0.8429
77	2015	36	24.1857	85.1429	933.0051	2.9622	0.7153
54	2015	36	19.8429	83.7449	833.7378	3.0061	1.0337
21	2015	36	22.9429	78.2653	902.3194	3.6755	1.4612
68	2015	36	25.3857	82.5306	968.5204	3.9888	0.9010
74	2015	36	25.7286	78.7449	961.1184	3.5439	1.0510
88	2015	36	21.9429	85.2755	875.7245	3.0133	2.1286
16	2015	36	23.5000	82.1429	921.1847	3.6561	1.4837
30	2015	36	21.9429	82.8673	897.4500	3.4510	1.1694
6	2015	36	25.3857	82.5306	968.5204	3.9888	0.9010
49	2015	36	24.3857	83.7551	939.6439	3.5929	0.9255
22	2015	36	21.9429	85.2755	875.7245	3.0133	2.1286
45	2015	36	19.2429	81.9796	819.0867	3.2276	1.3092
58	2015	36	24.3857	83.7551	939.6439	3.5929	0.9255
37	2015	36	25.3857	82.5306	968.5204	3.9888	0.9010
17	2015	36	22.2143	88.3776	902.3898	3.5653	2.4510
55	2015	36	21.6714	81.9184	878.2531	3.4918	1.3990
46	2015	36	23.5000	82.1429	921.1847	3.6561	1.4837
86	2015	36	21.7571	81.2857	867.1306	3.0949	0.7643
2	2015	36	21.7571	81.2857	867.1306	3.0949	0.7643
4	2015	36	22.9429	78.2653	902.3194	3.6755	1.4612
47	2015	36	25.7000	83.4184	957.6827	3.0378	0.5857
82	2015	36	21.9429	85.2755	875.7245	3.0133	2.1286

19	2015	36	25.5857	83.6122	959.2704	3.4602	0.9276
20	2015	36	20.8571	80.4796	855.2245	3.8031	1.9071
80	2015	36	21.9429	85.2755	875.7245	3.0133	2.1286
3	2015	36	25.2000	84.4082	943.8592	4.6020	0.9582
52	2015	36	22.2143	88.3776	902.3898	3.5653	2.4510
70	2015	36	24.0000	79.0918	910.5786	4.0286	1.3765
64	2015	36	17.0429	84.4796	778.2939	1.7929	1.7786
48	2015	36	24.1857	85.1429	933.0051	2.9622	0.7153
65	2015	36	22.2143	88.3776	902.3898	3.5653	2.4510
44	2015	36	24.0000	79.0918	910.5786	4.0286	1.3765
75	2015	36	17.0429	84.4796	778.2939	1.7929	1.7786
40	2015	36	24.3143	85.0306	944.5673	4.2602	1.0908
11	2015	36	21.6714	81.9184	878.2531	3.4918	1.3990
35	2015	36	23.9857	82.9694	938.7245	4.0643	1.2439
78	2015	36	22.8286	85.8367	899.7735	3.3908	1.2347
28	2015	36	23.7286	83.6531	928.6816	3.7969	1.3214
39	2015	36	22.2143	88.3776	902.3898	3.5653	2.4510
24	2015	36	24.3857	83.7551	939.6439	3.5929	0.9255
63	2015	36	24.3143	85.0306	944.5673	4.2602	1.0908
62	2015	36	22.1000	84.8571	875.4704	3.1490	0.8020
1	2015	36	21.9429	85.2755	875.7245	3.0133	2.1286
31	2015	37	18.6286	84.6020	848.4041	2.4786	0.9245
79	2015	37	23.7571	84.0306	969.8755	3.0980	1.1031
51	2015	37	21.8429	85.0000	939.9929	2.7378	1.2684
14	2015	37	20.2571	88.3265	898.7061	1.9429	1.4480
67	2015	37	20.4000	91.8469	903.5429	2.1184	2.6398
42	2015	37	19.4571	88.0204	876.7194	2.5071	2.3480
50	2015	37	20.7143	78.8673	903.2684	2.5347	1.6929
43	2015	37	19.4571	88.0204	876.7194	2.5071	2.3480
85	2015	37	21.5429	77.6327	911.4347	3.2949	1.6959
25	2015	37	23.6286	88.3163	976.9765	2.0796	0.7204
69	2015	37	22.3143	84.8776	940.7439	1.8214	0.8776
57	2015	37	19.5571	79.3469	888.3806	3.1520	2.3276
9	2015	37	19.0714	83.8061	856.2010	3.4459	2.2490
72	2015	37	20.4143	84.1122	879.3306	3.0235	1.4020
26	2015	37	20.8571	84.8571	868.8276	3.3531	1.5929
7	2015	37	20.3000	83.3980	861.1888	2.9653	1.5673
83	2015	37	24.5429	85.7857	945.0235	4.9347	0.9602
76	2015	37	21.4000	82.7653	922.2439	2.5367	1.4704
36	2015	37	21.7857	85.9694	929.8367	2.5255	1.3163
81	2015	37	21.8429	85.0000	939.9929	2.7378	1.2684
15	2015	37	22.0429	86.0000	934.0704	2.1694	0.6816
32	2015	37	19.4571	88.0204	876.7194	2.5071	2.3480
73	2015	37	23.7857	80.1531	962.2918	2.1163	0.9612
71	2015	37	21.7857	85.9694	929.8367	2.5255	1.3163
41	2015	37	20.1000	86.4796	876.3051	2.7398	1.1265
10	2015	37	22.9286	88.1531	964.4347	3.1582	0.9724
23	2015	37	15.0714	86.5816	779.0531	1.6980	1.8388
27	2015	37	20.3000	83.3980	861.1888	2.9653	1.5673
60	2015	37	21.8429	85.0000	939.9929	2.7378	1.2684

53	2015	37	19.0714	83.8061	856.2010	3.4459	2.2490
66	2015	37	20.2571	88.3265	898.7061	1.9429	1.4480
59	2015	37	19.5571	79.3469	888.3806	3.1520	2.3276
61	2015	37	23.7857	80.1531	962.2918	2.1163	0.9612
84	2015	37	23.7857	80.1531	962.2918	2.1163	0.9612
38	2015	37	19.5571	79.3469	888.3806	3.1520	2.3276
87	2015	37	21.2429	87.1122	900.8551	3.0092	1.4133
34	2015	37	19.5571	79.3469	888.3806	3.1520	2.3276
29	2015	37	22.3143	84.8776	940.7439	1.8214	0.8776
5	2015	37	18.5714	84.7347	834.6102	3.4306	1.2388
8	2015	37	19.0714	83.8061	856.2010	3.4459	2.2490
12	2015	37	18.5714	84.7347	834.6102	3.4306	1.2388
13	2015	37	24.5429	85.7857	945.0235	4.9347	0.9602
18	2015	37	23.5714	83.3265	964.4684	3.0378	0.9653
33	2015	37	20.7143	78.8673	903.2684	2.5347	1.6929
56	2015	37	23.6286	88.3163	976.9765	2.0796	0.7204
77	2015	37	22.0429	86.0000	934.0704	2.1694	0.6816
54	2015	37	18.5714	84.7347	834.6102	3.4306	1.2388
21	2015	37	20.7143	78.8673	903.2684	2.5347	1.6929
68	2015	37	23.7571	84.0306	969.8755	3.0980	1.1031
74	2015	37	23.7857	80.1531	962.2918	2.1163	0.9612
88	2015	37	19.4571	88.0204	876.7194	2.5071	2.3480
16	2015	37	21.4000	82.7653	922.2439	2.5367	1.4704
30	2015	37	20.2571	88.3265	898.7061	1.9429	1.4480
6	2015	37	23.7571	84.0306	969.8755	3.0980	1.1031
49	2015	37	22.3143	84.8776	940.7439	1.8214	0.8776
22	2015	37	19.4571	88.0204	876.7194	2.5071	2.3480
45	2015	37	17.9286	83.6224	819.9398	3.3908	1.3173
58	2015	37	22.3143	84.8776	940.7439	1.8214	0.8776
37	2015	37	23.7571	84.0306	969.8755	3.0980	1.1031
17	2015	37	20.4000	91.8469	903.5429	2.1184	2.6398
55	2015	37	20.4143	84.1122	879.3306	3.0235	1.4020
46	2015	37	21.4000	82.7653	922.2439	2.5367	1.4704
86	2015	37	19.5143	82.4694	867.9663	2.7367	1.0673
2	2015	37	19.5143	82.4694	867.9663	2.7367	1.0673
4	2015	37	20.7143	78.8673	903.2684	2.5347	1.6929
47	2015	37	24.7571	84.6224	958.9153	2.6153	0.6082
82	2015	37	19.4571	88.0204	876.7194	2.5071	2.3480
19	2015	37	24.1286	86.5816	960.7051	2.4010	0.9388
20	2015	37	19.0714	83.8061	856.2010	3.4459	2.2490
80	2015	37	19.4571	88.0204	876.7194	2.5071	2.3480
3	2015	37	24.5429	85.7857	945.0235	4.9347	0.9602
52	2015	37	20.4000	91.8469	903.5429	2.1184	2.6398
70	2015	37	21.5429	77.6327	911.4347	3.2949	1.6959
64	2015	37	15.0714	86.5816	779.0531	1.6980	1.8388
48	2015	37	22.0429	86.0000	934.0704	2.1694	0.6816
65	2015	37	20.4000	91.8469	903.5429	2.1184	2.6398
44	2015	37	21.5429	77.6327	911.4347	3.2949	1.6959
75	2015	37	15.0714	86.5816	779.0531	1.6980	1.8388
40	2015	37	22.0143	88.5306	946.0112	2.6786	1.0061

11	2015	37	20.4143	84.1122	879.3306	3.0235	1.4020
35	2015	37	21.8429	85.0000	939.9929	2.7378	1.2684
78	2015	37	21.2429	87.1122	900.8551	3.0092	1.4133
28	2015	37	21.7857	85.9694	929.8367	2.5255	1.3163
39	2015	37	20.4000	91.8469	903.5429	2.1184	2.6398
24	2015	37	22.3143	84.8776	940.7439	1.8214	0.8776
63	2015	37	22.0143	88.5306	946.0112	2.6786	1.0061
62	2015	37	20.1000	86.4796	876.3051	2.7398	1.1265
1	2015	37	19.4571	88.0204	876.7194	2.5071	2.3480
31	2015	38	16.9143	86.5204	850.3755	1.3255	1.0082
79	2015	38	21.9286	81.9694	972.8531	2.2520	1.2990
51	2015	38	20.6571	83.1224	942.6633	2.0490	1.2786
14	2015	38	20.1143	87.2959	901.0316	1.1469	1.5194
67	2015	38	19.5143	91.0306	905.9112	1.2878	2.6184
42	2015	38	18.9143	87.6837	878.9194	1.3684	2.2724
50	2015	38	18.8429	80.1939	905.7357	0.8276	1.6806
43	2015	38	18.9143	87.6837	878.9194	1.3684	2.2724
85	2015	38	19.6286	79.9592	913.9551	1.5459	1.6316
25	2015	38	23.6143	87.1020	979.8173	2.0357	0.6878
69	2015	38	21.3286	83.0306	943.3500	1.2776	0.8520
57	2015	38	18.2286	80.6735	890.6582	1.5102	2.1816
9	2015	38	18.1714	84.5408	858.2459	1.5051	2.3500
72	2015	38	19.7000	83.4184	881.5633	1.7469	1.4010
26	2015	38	19.8857	85.3776	870.9296	3.0143	1.6449
7	2015	38	19.1714	84.3571	863.2541	2.2531	1.6592
83	2015	38	23.6857	84.4184	947.6061	4.4429	0.9714
76	2015	38	19.9571	81.8878	924.7888	1.1959	1.4959
36	2015	38	21.0429	83.6122	932.3684	1.8245	1.3347
81	2015	38	20.6571	83.1224	942.6633	2.0490	1.2786
15	2015	38	20.1000	88.2347	936.7939	0.8327	0.6837
32	2015	38	18.9143	87.6837	878.9194	1.3684	2.2724
73	2015	38	22.2000	79.1224	965.1500	1.1010	0.8949
71	2015	38	21.0429	83.6122	932.3684	1.8245	1.3347
41	2015	38	18.6143	87.3367	878.4622	1.1643	1.2388
10	2015	38	21.5714	86.0000	967.2898	2.2347	1.0367
23	2015	38	14.2857	88.4796	780.4959	1.6245	2.1745
27	2015	38	19.1714	84.3571	863.2541	2.2531	1.6592
60	2015	38	20.6571	83.1224	942.6633	2.0490	1.2786
53	2015	38	18.1714	84.5408	858.2459	1.5051	2.3500
66	2015	38	20.1143	87.2959	901.0316	1.1469	1.5194
59	2015	38	18.2286	80.6735	890.6582	1.5102	2.1816
61	2015	38	22.2000	79.1224	965.1500	1.1010	0.8949
84	2015	38	22.2000	79.1224	965.1500	1.1010	0.8949
38	2015	38	18.2286	80.6735	890.6582	1.5102	2.1816
87	2015	38	20.7571	86.5204	903.1929	1.9643	1.4969
34	2015	38	18.2286	80.6735	890.6582	1.5102	2.1816
29	2015	38	21.3286	83.0306	943.3500	1.2776	0.8520
5	2015	38	17.4429	86.8265	836.4816	2.7316	1.2724
8	2015	38	18.1714	84.5408	858.2459	1.5051	2.3500
12	2015	38	17.4429	86.8265	836.4816	2.7316	1.2724

13	2015	38	23.6857	84.4184	947.6061	4.4429	0.9714
18	2015	38	21.6429	81.1735	967.4520	1.9724	0.9796
33	2015	38	18.8429	80.1939	905.7357	0.8276	1.6806
56	2015	38	23.6143	87.1020	979.8173	2.0357	0.6878
77	2015	38	20.1000	88.2347	936.7939	0.8327	0.6837
54	2015	38	17.4429	86.8265	836.4816	2.7316	1.2724
21	2015	38	18.8429	80.1939	905.7357	0.8276	1.6806
68	2015	38	21.9286	81.9694	972.8531	2.2520	1.2990
74	2015	38	22.2000	79.1224	965.1500	1.1010	0.8949
88	2015	38	18.9143	87.6837	878.9194	1.3684	2.2724
16	2015	38	19.9571	81.8878	924.7888	1.1959	1.4959
30	2015	38	20.1143	87.2959	901.0316	1.1469	1.5194
6	2015	38	21.9286	81.9694	972.8531	2.2520	1.2990
49	2015	38	21.3286	83.0306	943.3500	1.2776	0.8520
22	2015	38	18.9143	87.6837	878.9194	1.3684	2.2724
45	2015	38	17.3857	85.0306	821.6806	2.5929	1.3561
58	2015	38	21.3286	83.0306	943.3500	1.2776	0.8520
37	2015	38	21.9286	81.9694	972.8531	2.2520	1.2990
17	2015	38	19.5143	91.0306	905.9112	1.2878	2.6184
55	2015	38	19.7000	83.4184	881.5633	1.7469	1.4010
46	2015	38	19.9571	81.8878	924.7888	1.1959	1.4959
86	2015	38	18.2714	84.5204	870.0796	1.3265	1.0418
2	2015	38	18.2714	84.5204	870.0796	1.3265	1.0418
4	2015	38	18.8429	80.1939	905.7357	0.8276	1.6806
47	2015	38	24.1714	82.5306	961.6041	2.9071	0.6541
82	2015	38	18.9143	87.6837	878.9194	1.3684	2.2724
19	2015	38	23.7714	85.7347	963.4010	2.1582	1.0194
20	2015	38	18.1714	84.5408	858.2459	1.5051	2.3500
80	2015	38	18.9143	87.6837	878.9194	1.3684	2.2724
3	2015	38	23.6857	84.4184	947.6061	4.4429	0.9714
52	2015	38	19.5143	91.0306	905.9112	1.2878	2.6184
70	2015	38	19.6286	79.9592	913.9551	1.5459	1.6316
64	2015	38	14.2857	88.4796	780.4959	1.6245	2.1745
48	2015	38	20.1000	88.2347	936.7939	0.8327	0.6837
65	2015	38	19.5143	91.0306	905.9112	1.2878	2.6184
44	2015	38	19.6286	79.9592	913.9551	1.5459	1.6316
75	2015	38	14.2857	88.4796	780.4959	1.6245	2.1745
40	2015	38	21.3857	86.8265	948.6918	2.6071	1.0520
11	2015	38	19.7000	83.4184	881.5633	1.7469	1.4010
35	2015	38	20.6571	83.1224	942.6633	2.0490	1.2786
78	2015	38	20.7571	86.5204	903.1929	1.9643	1.4969
28	2015	38	21.0429	83.6122	932.3684	1.8245	1.3347
39	2015	38	19.5143	91.0306	905.9112	1.2878	2.6184
24	2015	38	21.3286	83.0306	943.3500	1.2776	0.8520
63	2015	38	21.3857	86.8265	948.6918	2.6071	1.0520
62	2015	38	18.6143	87.3367	878.4622	1.1643	1.2388
1	2015	38	18.9143	87.6837	878.9194	1.3684	2.2724
31	2015	39	16.6857	88.5918	850.1306	0.7480	0.9408
79	2015	39	21.1000	82.9184	973.1673	1.3663	1.2602
51	2015	39	19.9000	84.1327	942.7735	1.5786	1.2000

14	2015	39	19.4714	86.0612	900.8500	1.0224	1.5490
67	2015	39	18.9000	91.7551	905.8469	1.0663	2.3796
42	2015	39	18.1000	89.1837	878.7418	0.7276	2.1194
50	2015	39	18.6000	83.2551	905.6980	0.2122	1.3704
43	2015	39	18.1000	89.1837	878.7418	0.7276	2.1194
85	2015	39	19.4286	82.7347	914.0378	0.9510	1.3939
25	2015	39	23.3857	85.2755	979.8990	2.0990	0.6418
69	2015	39	20.4286	83.6020	943.5102	1.1398	0.8694
57	2015	39	17.8429	83.7653	890.5653	0.5867	1.7449
9	2015	39	17.5429	86.7245	857.9592	0.5031	2.1755
72	2015	39	19.0857	84.0000	881.3602	1.0490	1.3847
26	2015	39	19.7571	87.4694	870.7061	2.4786	1.5082
7	2015	39	19.2571	86.2857	862.9837	1.6265	1.5531
83	2015	39	23.3286	86.7347	947.5980	2.9469	0.7867
76	2015	39	19.1000	83.7551	924.8418	0.6684	1.3806
36	2015	39	20.3857	83.5000	932.4571	1.4082	1.3582
81	2015	39	19.9000	84.1327	942.7735	1.5786	1.2000
15	2015	39	19.8571	90.5918	936.9143	0.1602	0.6000
32	2015	39	18.1000	89.1837	878.7418	0.7276	2.1194
73	2015	39	21.2286	81.0408	965.4898	0.6388	0.8286
71	2015	39	20.3857	83.5000	932.4571	1.4082	1.3582
41	2015	39	18.2571	89.4796	878.2908	0.5755	1.0429
10	2015	39	21.2714	86.6531	967.5010	1.7745	0.9347
23	2015	39	14.4286	90.4592	779.9235	1.0776	2.2224
27	2015	39	19.2571	86.2857	862.9837	1.6265	1.5531
60	2015	39	19.9000	84.1327	942.7735	1.5786	1.2000
53	2015	39	17.5429	86.7245	857.9592	0.5031	2.1755
66	2015	39	19.4714	86.0612	900.8500	1.0224	1.5490
59	2015	39	17.8429	83.7653	890.5653	0.5867	1.7449
61	2015	39	21.2286	81.0408	965.4898	0.6388	0.8286
84	2015	39	21.2286	81.0408	965.4898	0.6388	0.8286
38	2015	39	17.8429	83.7653	890.5653	0.5867	1.7449
87	2015	39	20.1000	86.9694	903.0490	1.1429	1.4347
34	2015	39	17.8429	83.7653	890.5653	0.5867	1.7449
29	2015	39	20.4286	83.6020	943.5102	1.1398	0.8694
5	2015	39	17.6000	89.1939	836.1378	1.8806	1.2265
8	2015	39	17.5429	86.7245	857.9592	0.5031	2.1755
12	2015	39	17.6000	89.1939	836.1378	1.8806	1.2265
13	2015	39	23.3286	86.7347	947.5980	2.9469	0.7867
18	2015	39	20.5429	84.0816	967.7500	1.3214	0.9163
33	2015	39	18.6000	83.2551	905.6980	0.2122	1.3704
56	2015	39	23.3857	85.2755	979.8990	2.0990	0.6418
77	2015	39	19.8571	90.5918	936.9143	0.1602	0.6000
54	2015	39	17.6000	89.1939	836.1378	1.8806	1.2265
21	2015	39	18.6000	83.2551	905.6980	0.2122	1.3704
68	2015	39	21.1000	82.9184	973.1673	1.3663	1.2602
74	2015	39	21.2286	81.0408	965.4898	0.6388	0.8286
88	2015	39	18.1000	89.1837	878.7418	0.7276	2.1194
16	2015	39	19.1000	83.7551	924.8418	0.6684	1.3806
30	2015	39	19.4714	86.0612	900.8500	1.0224	1.5490

6	2015	39	21.1000	82.9184	973.1673	1.3663	1.2602
49	2015	39	20.4286	83.6020	943.5102	1.1398	0.8694
22	2015	39	18.1000	89.1837	878.7418	0.7276	2.1194
45	2015	39	18.1143	85.9286	821.2520	1.9276	1.4439
58	2015	39	20.4286	83.6020	943.5102	1.1398	0.8694
37	2015	39	21.1000	82.9184	973.1673	1.3663	1.2602
17	2015	39	18.9000	91.7551	905.8469	1.0663	2.3796
55	2015	39	19.0857	84.0000	881.3602	1.0490	1.3847
46	2015	39	19.1000	83.7551	924.8418	0.6684	1.3806
86	2015	39	18.1429	87.2449	869.8765	0.8745	0.8633
2	2015	39	18.1429	87.2449	869.8765	0.8745	0.8633
4	2015	39	18.6000	83.2551	905.6980	0.2122	1.3704
47	2015	39	23.4000	83.1633	961.6612	2.5286	0.6643
82	2015	39	18.1000	89.1837	878.7418	0.7276	2.1194
19	2015	39	23.1571	84.6020	963.3918	1.9643	0.9653
20	2015	39	17.5429	86.7245	857.9592	0.5031	2.1755
80	2015	39	18.1000	89.1837	878.7418	0.7276	2.1194
3	2015	39	23.3286	86.7347	947.5980	2.9469	0.7867
52	2015	39	18.9000	91.7551	905.8469	1.0663	2.3796
70	2015	39	19.4286	82.7347	914.0378	0.9510	1.3939
64	2015	39	14.4286	90.4592	779.9235	1.0776	2.2224
48	2015	39	19.8571	90.5918	936.9143	0.1602	0.6000
65	2015	39	18.9000	91.7551	905.8469	1.0663	2.3796
44	2015	39	19.4286	82.7347	914.0378	0.9510	1.3939
75	2015	39	14.4286	90.4592	779.9235	1.0776	2.2224
40	2015	39	20.9571	86.9184	948.6918	2.3602	1.1347
11	2015	39	19.0857	84.0000	881.3602	1.0490	1.3847
35	2015	39	19.9000	84.1327	942.7735	1.5786	1.2000
78	2015	39	20.1000	86.9694	903.0490	1.1429	1.4347
28	2015	39	20.3857	83.5000	932.4571	1.4082	1.3582
39	2015	39	18.9000	91.7551	905.8469	1.0663	2.3796
24	2015	39	20.4286	83.6020	943.5102	1.1398	0.8694
63	2015	39	20.9571	86.9184	948.6918	2.3602	1.1347
62	2015	39	18.2571	89.4796	878.2908	0.5755	1.0429
1	2015	39	18.1000	89.1837	878.7418	0.7276	2.1194
31	2015	40	16.5571	90.0510	848.9724	0.9163	0.8592
79	2015	40	21.8286	83.8265	971.9449	1.6694	1.2827
51	2015	40	20.5714	85.7245	941.5510	1.6071	1.2571
14	2015	40	20.4857	87.8163	899.5388	1.4500	1.5184
67	2015	40	19.7286	94.3163	904.5490	1.2388	2.3663
42	2015	40	18.6286	92.1939	877.4449	0.9214	2.1990
50	2015	40	18.7857	86.1327	904.3847	0.7633	1.5031
43	2015	40	18.6286	92.1939	877.4449	0.9214	2.1990
85	2015	40	19.1714	84.8776	912.7898	1.4347	1.3163
25	2015	40	24.0000	84.6633	978.4439	2.0296	0.6694
69	2015	40	21.0286	85.6122	942.2500	1.2816	0.8408
57	2015	40	17.7429	87.0918	889.2704	0.6816	1.7398
9	2015	40	18.3286	90.3878	856.6561	0.6520	2.1276
72	2015	40	19.9143	86.5000	880.0041	0.9316	1.5663
26	2015	40	21.4714	89.3367	869.2684	2.6112	1.6571

7	2015	40	20.3286	88.2959	861.5878	1.6990	1.6622
83	2015	40	24.5857	87.9796	945.9602	2.6786	0.6857
76	2015	40	19.9286	87.0306	923.5459	0.8806	1.4571
36	2015	40	20.9286	85.4694	931.1745	1.4949	1.4571
81	2015	40	20.5714	85.7245	941.5510	1.6071	1.2571
15	2015	40	20.3000	92.2245	935.5857	0.4173	0.6082
32	2015	40	18.6286	92.1939	877.4449	0.9214	2.1990
73	2015	40	22.2000	83.2857	964.2367	0.9418	0.7582
71	2015	40	20.9286	85.4694	931.1745	1.4949	1.4571
41	2015	40	18.2857	93.5714	877.0112	0.6806	0.8286
10	2015	40	21.6286	88.1327	966.2245	1.8245	0.8878
23	2015	40	14.8286	89.8061	778.8469	2.4316	2.1684
27	2015	40	20.3286	88.2959	861.5878	1.6990	1.6622
60	2015	40	20.5714	85.7245	941.5510	1.6071	1.2571
53	2015	40	18.3286	90.3878	856.6561	0.6520	2.1276
66	2015	40	20.4857	87.8163	899.5388	1.4500	1.5184
59	2015	40	17.7429	87.0918	889.2704	0.6816	1.7398
61	2015	40	22.2000	83.2857	964.2367	0.9418	0.7582
84	2015	40	22.2000	83.2857	964.2367	0.9418	0.7582
38	2015	40	17.7429	87.0918	889.2704	0.6816	1.7398
87	2015	40	20.9000	89.3878	901.6745	1.3959	1.3010
34	2015	40	17.7429	87.0918	889.2704	0.6816	1.7398
29	2015	40	21.0286	85.6122	942.2500	1.2816	0.8408
5	2015	40	18.2429	91.5306	834.8408	2.3633	1.4194
8	2015	40	18.3286	90.3878	856.6561	0.6520	2.1276
12	2015	40	18.2429	91.5306	834.8408	2.3633	1.4194
13	2015	40	24.5857	87.9796	945.9602	2.6786	0.6857
18	2015	40	21.2571	87.9082	966.5429	1.5102	0.8194
33	2015	40	18.7857	86.1327	904.3847	0.7633	1.5031
56	2015	40	24.0000	84.6633	978.4439	2.0296	0.6694
77	2015	40	20.3000	92.2245	935.5857	0.4173	0.6082
54	2015	40	18.2429	91.5306	834.8408	2.3633	1.4194
21	2015	40	18.7857	86.1327	904.3847	0.7633	1.5031
68	2015	40	21.8286	83.8265	971.9449	1.6694	1.2827
74	2015	40	22.2000	83.2857	964.2367	0.9418	0.7582
88	2015	40	18.6286	92.1939	877.4449	0.9214	2.1990
16	2015	40	19.9286	87.0306	923.5459	0.8806	1.4571
30	2015	40	20.4857	87.8163	899.5388	1.4500	1.5184
6	2015	40	21.8286	83.8265	971.9449	1.6694	1.2827
49	2015	40	21.0286	85.6122	942.2500	1.2816	0.8408
22	2015	40	18.6286	92.1939	877.4449	0.9214	2.1990
45	2015	40	19.1714	85.0000	819.9459	2.6816	1.5500
58	2015	40	21.0286	85.6122	942.2500	1.2816	0.8408
37	2015	40	21.8286	83.8265	971.9449	1.6694	1.2827
17	2015	40	19.7286	94.3163	904.5490	1.2388	2.3663
55	2015	40	19.9143	86.5000	880.0041	0.9316	1.5663
46	2015	40	19.9286	87.0306	923.5459	0.8806	1.4571
86	2015	40	18.2857	90.1531	868.5816	0.7184	0.8337
2	2015	40	18.2857	90.1531	868.5816	0.7184	0.8337
4	2015	40	18.7857	86.1327	904.3847	0.7633	1.5031

47	2015	40	24.6429	85.1020	960.1041	2.1735	0.6010
82	2015	40	18.6286	92.1939	877.4449	0.9214	2.1990
19	2015	40	24.6286	84.4388	961.8969	2.3214	0.9061
20	2015	40	18.3286	90.3878	856.6561	0.6520	2.1276
80	2015	40	18.6286	92.1939	877.4449	0.9214	2.1990
3	2015	40	24.5857	87.9796	945.9602	2.6786	0.6857
52	2015	40	19.7286	94.3163	904.5490	1.2388	2.3663
70	2015	40	19.1714	84.8776	912.7898	1.4347	1.3163
64	2015	40	14.8286	89.8061	778.8469	2.4316	2.1684
48	2015	40	20.3000	92.2245	935.5857	0.4173	0.6082
65	2015	40	19.7286	94.3163	904.5490	1.2388	2.3663
44	2015	40	19.1714	84.8776	912.7898	1.4347	1.3163
75	2015	40	14.8286	89.8061	778.8469	2.4316	2.1684
40	2015	40	21.7714	86.9898	947.3980	2.5694	1.3041
11	2015	40	19.9143	86.5000	880.0041	0.9316	1.5663
35	2015	40	20.5714	85.7245	941.5510	1.6071	1.2571
78	2015	40	20.9000	89.3878	901.6745	1.3959	1.3010
28	2015	40	20.9286	85.4694	931.1745	1.4949	1.4571
39	2015	40	19.7286	94.3163	904.5490	1.2388	2.3663
24	2015	40	21.0286	85.6122	942.2500	1.2816	0.8408
63	2015	40	21.7714	86.9898	947.3980	2.5694	1.3041
62	2015	40	18.2857	93.5714	877.0112	0.6806	0.8286
1	2015	40	18.6286	92.1939	877.4449	0.9214	2.1990
31	2015	41	13.6143	88.9286	851.6102	0.9837	0.8020
79	2015	41	19.3000	82.0102	974.7224	2.1663	1.1133
51	2015	41	17.4000	83.8367	944.1306	2.1796	1.2265
14	2015	41	15.6857	86.7857	901.7010	2.1433	1.4408
67	2015	41	15.5429	92.4184	906.8888	1.6133	2.6867
42	2015	41	14.7143	91.7347	879.8286	1.2327	2.2306
50	2015	41	16.1571	86.7551	907.1582	1.3878	1.5663
43	2015	41	14.7143	91.7347	879.8286	1.2327	2.2306
85	2015	41	16.7857	87.1224	915.7500	1.4786	1.1378
25	2015	41	19.5429	83.8265	981.1184	2.5592	0.7602
69	2015	41	18.0000	84.8469	944.9612	1.9684	0.9235
57	2015	41	14.8714	87.5510	891.9000	1.0143	1.8561
9	2015	41	14.0286	88.4490	858.9306	1.0082	2.1500
72	2015	41	15.3429	85.4286	882.2643	1.1327	1.9459
26	2015	41	15.5714	86.7245	871.4847	2.3765	1.7684
7	2015	41	14.5429	87.1327	863.8388	1.4745	1.7306
83	2015	41	19.2857	86.7653	948.5153	2.8480	0.8000
76	2015	41	17.2143	86.0714	926.1908	1.3827	1.4643
36	2015	41	17.2000	85.1122	933.7418	2.0847	1.4500
81	2015	41	17.4000	83.8367	944.1306	2.1796	1.2265
15	2015	41	18.5857	91.5510	938.3418	0.6551	0.6755
32	2015	41	14.7143	91.7347	879.8286	1.2327	2.2306
73	2015	41	20.0286	81.2449	966.9469	1.5153	0.8286
71	2015	41	17.2000	85.1122	933.7418	2.0847	1.4500
41	2015	41	15.0000	93.5714	879.6582	0.8235	0.6551
10	2015	41	18.4286	86.6429	968.9286	2.4306	0.8612
23	2015	41	10.4429	88.3061	780.9908	2.9214	2.1755

27	2015	41	14.5429	87.1327	863.8388	1.4745	1.7306
60	2015	41	17.4000	83.8367	944.1306	2.1796	1.2265
53	2015	41	14.0286	88.4490	858.9306	1.0082	2.1500
66	2015	41	15.6857	86.7857	901.7010	2.1433	1.4408
59	2015	41	14.8714	87.5510	891.9000	1.0143	1.8561
61	2015	41	20.0286	81.2449	966.9469	1.5153	0.8286
84	2015	41	20.0286	81.2449	966.9469	1.5153	0.8286
38	2015	41	14.8714	87.5510	891.9000	1.0143	1.8561
87	2015	41	16.1857	89.5306	904.1306	1.7786	1.6061
34	2015	41	14.8714	87.5510	891.9000	1.0143	1.8561
29	2015	41	18.0000	84.8469	944.9612	1.9684	0.9235
5	2015	41	13.0000	91.3980	837.0286	2.1531	1.3857
8	2015	41	14.0286	88.4490	858.9306	1.0082	2.1500
12	2015	41	13.0000	91.3980	837.0286	2.1531	1.3857
13	2015	41	19.2857	86.7653	948.5153	2.8480	0.8000
18	2015	41	19.2571	85.6020	969.2602	2.0500	0.8031
33	2015	41	16.1571	86.7551	907.1582	1.3878	1.5663
56	2015	41	19.5429	83.8265	981.1184	2.5592	0.7602
77	2015	41	18.5857	91.5510	938.3418	0.6551	0.6755
54	2015	41	13.0000	91.3980	837.0286	2.1531	1.3857
21	2015	41	16.1571	86.7551	907.1582	1.3878	1.5663
68	2015	41	19.3000	82.0102	974.7224	2.1663	1.1133
74	2015	41	20.0286	81.2449	966.9469	1.5153	0.8286
88	2015	41	14.7143	91.7347	879.8286	1.2327	2.2306
16	2015	41	17.2143	86.0714	926.1908	1.3827	1.4643
30	2015	41	15.6857	86.7857	901.7010	2.1433	1.4408
6	2015	41	19.3000	82.0102	974.7224	2.1663	1.1133
49	2015	41	18.0000	84.8469	944.9612	1.9684	0.9235
22	2015	41	14.7143	91.7347	879.8286	1.2327	2.2306
45	2015	41	13.1286	84.1735	822.0347	2.8643	1.5837
58	2015	41	18.0000	84.8469	944.9612	1.9684	0.9235
37	2015	41	19.3000	82.0102	974.7224	2.1663	1.1133
17	2015	41	15.5429	92.4184	906.8888	1.6133	2.6867
55	2015	41	15.3429	85.4286	882.2643	1.1327	1.9459
46	2015	41	17.2143	86.0714	926.1908	1.3827	1.4643
86	2015	41	14.5000	88.9490	871.1418	0.7704	0.7582
2	2015	41	14.5000	88.9490	871.1418	0.7704	0.7582
4	2015	41	16.1571	86.7551	907.1582	1.3878	1.5663
47	2015	41	19.5143	83.9388	962.7020	2.6969	0.6347
82	2015	41	14.7143	91.7347	879.8286	1.2327	2.2306
19	2015	41	19.4286	83.1939	964.2929	2.8388	1.0398
20	2015	41	14.0286	88.4490	858.9306	1.0082	2.1500
80	2015	41	14.7143	91.7347	879.8286	1.2327	2.2306
3	2015	41	19.2857	86.7653	948.5153	2.8480	0.8000
52	2015	41	15.5429	92.4184	906.8888	1.6133	2.6867
70	2015	41	16.7857	87.1224	915.7500	1.4786	1.1378
64	2015	41	10.4429	88.3061	780.9908	2.9214	2.1755
48	2015	41	18.5857	91.5510	938.3418	0.6551	0.6755
65	2015	41	15.5429	92.4184	906.8888	1.6133	2.6867
44	2015	41	16.7857	87.1224	915.7500	1.4786	1.1378

75	2015	41	10.4429	88.3061	780.9908	2.9214	2.1755
40	2015	41	17.5714	85.0000	949.8694	3.5633	1.4296
11	2015	41	15.3429	85.4286	882.2643	1.1327	1.9459
35	2015	41	17.4000	83.8367	944.1306	2.1796	1.2265
78	2015	41	16.1857	89.5306	904.1306	1.7786	1.6061
28	2015	41	17.2000	85.1122	933.7418	2.0847	1.4500
39	2015	41	15.5429	92.4184	906.8888	1.6133	2.6867
24	2015	41	18.0000	84.8469	944.9612	1.9684	0.9235
63	2015	41	17.5714	85.0000	949.8694	3.5633	1.4296
62	2015	41	15.0000	93.5714	879.6582	0.8235	0.6551
1	2015	41	14.7143	91.7347	879.8286	1.2327	2.2306
31	2015	42	15.1857	84.9490	854.1929	1.8898	0.8469
79	2015	42	20.2429	78.0816	977.7418	3.5010	0.8592
51	2015	42	17.7143	80.8878	947.1204	2.9602	1.0888
14	2015	42	17.6143	82.8163	904.6735	3.0207	1.1541
67	2015	42	17.5857	86.6531	909.8286	2.6000	2.2714
42	2015	42	17.3143	85.4286	882.6235	2.6337	1.9398
50	2015	42	17.7286	82.1735	910.0469	2.9847	1.2867
43	2015	42	17.3143	85.4286	882.6235	2.6337	1.9398
85	2015	42	18.8286	81.3980	918.6633	2.6184	1.0888
25	2015	42	20.1143	83.8878	984.8398	3.3286	0.8653
69	2015	42	18.7143	82.8367	948.0949	2.7908	1.0051
57	2015	42	17.0286	81.3163	894.7653	2.2735	1.7490
9	2015	42	16.7000	82.0714	861.6918	2.7816	1.6459
72	2015	42	17.1857	83.2041	885.2551	2.1612	1.6796
26	2015	42	17.7571	84.0408	874.6122	2.5592	1.4837
7	2015	42	16.9429	84.1020	866.8224	2.1500	1.3714
83	2015	42	20.7143	85.2857	952.4316	3.3306	1.0194
76	2015	42	18.0857	82.0408	929.1286	2.7837	1.2520
36	2015	42	18.4714	83.5918	936.8571	3.2959	1.3102
81	2015	42	17.7143	80.8878	947.1204	2.9602	1.0888
15	2015	42	18.3429	86.0816	941.3010	2.0520	0.6020
32	2015	42	17.3143	85.4286	882.6235	2.6337	1.9398
73	2015	42	20.0857	77.3163	970.0480	2.9531	0.8959
71	2015	42	18.4714	83.5918	936.8571	3.2959	1.3102
41	2015	42	17.1000	88.1531	882.5010	2.1929	0.5969
10	2015	42	18.8143	82.9694	972.0949	3.8796	0.8357
23	2015	42	12.4143	85.0102	783.2000	2.3918	1.9031
27	2015	42	16.9429	84.1020	866.8224	2.1500	1.3714
60	2015	42	17.7143	80.8878	947.1204	2.9602	1.0888
53	2015	42	16.7000	82.0714	861.6918	2.7816	1.6459
66	2015	42	17.6143	82.8163	904.6735	3.0207	1.1541
59	2015	42	17.0286	81.3163	894.7653	2.2735	1.7490
61	2015	42	20.0857	77.3163	970.0480	2.9531	0.8959
84	2015	42	20.0857	77.3163	970.0480	2.9531	0.8959
38	2015	42	17.0286	81.3163	894.7653	2.2735	1.7490
87	2015	42	18.1857	87.2449	907.2980	2.5898	1.4469
34	2015	42	17.0286	81.3163	894.7653	2.2735	1.7490
29	2015	42	18.7143	82.8367	948.0949	2.7908	1.0051
5	2015	42	15.4286	87.5000	839.7520	2.1296	1.1092

8	2015	42	16.7000	82.0714	861.6918	2.7816	1.6459
12	2015	42	15.4286	87.5000	839.7520	2.1296	1.1092
13	2015	42	20.7143	85.2857	952.4316	3.3306	1.0194
18	2015	42	19.5429	80.3367	972.1449	3.4714	0.8592
33	2015	42	17.7286	82.1735	910.0469	2.9847	1.2867
56	2015	42	20.1143	83.8878	984.8398	3.3286	0.8653
77	2015	42	18.3429	86.0816	941.3010	2.0520	0.6020
54	2015	42	15.4286	87.5000	839.7520	2.1296	1.1092
21	2015	42	17.7286	82.1735	910.0469	2.9847	1.2867
68	2015	42	20.2429	78.0816	977.7418	3.5010	0.8592
74	2015	42	20.0857	77.3163	970.0480	2.9531	0.8959
88	2015	42	17.3143	85.4286	882.6235	2.6337	1.9398
16	2015	42	18.0857	82.0408	929.1286	2.7837	1.2520
30	2015	42	17.6143	82.8163	904.6735	3.0207	1.1541
6	2015	42	20.2429	78.0816	977.7418	3.5010	0.8592
49	2015	42	18.7143	82.8367	948.0949	2.7908	1.0051
22	2015	42	17.3143	85.4286	882.6235	2.6337	1.9398
45	2015	42	14.9429	84.1327	824.7082	2.4663	1.3918
58	2015	42	18.7143	82.8367	948.0949	2.7908	1.0051
37	2015	42	20.2429	78.0816	977.7418	3.5010	0.8592
17	2015	42	17.5857	86.6531	909.8286	2.6000	2.2714
55	2015	42	17.1857	83.2041	885.2551	2.1612	1.6796
46	2015	42	18.0857	82.0408	929.1286	2.7837	1.2520
86	2015	42	16.7000	84.7959	873.9857	2.3418	0.7163
2	2015	42	16.7000	84.7959	873.9857	2.3418	0.7163
4	2015	42	17.7286	82.1735	910.0469	2.9847	1.2867
47	2015	42	21.0429	82.6837	966.6031	2.8939	0.6689
82	2015	42	17.3143	85.4286	882.6235	2.6337	1.9398
19	2015	42	20.3429	82.9388	967.9633	3.3398	1.0449
20	2015	42	16.7000	82.0714	861.6918	2.7816	1.6459
80	2015	42	17.3143	85.4286	882.6235	2.6337	1.9398
3	2015	42	20.7143	85.2857	952.4316	3.3306	1.0194
52	2015	42	17.5857	86.6531	909.8286	2.6000	2.2714
70	2015	42	18.8286	81.3980	918.6633	2.6184	1.0888
64	2015	42	12.4143	85.0102	783.2000	2.3918	1.9031
48	2015	42	18.3429	86.0816	941.3010	2.0520	0.6020
65	2015	42	17.5857	86.6531	909.8286	2.6000	2.2714
44	2015	42	18.8286	81.3980	918.6633	2.6184	1.0888
75	2015	42	12.4143	85.0102	783.2000	2.3918	1.9031
40	2015	42	18.3143	83.5816	953.0235	4.3347	1.0888
11	2015	42	17.1857	83.2041	885.2551	2.1612	1.6796
35	2015	42	17.7143	80.8878	947.1204	2.9602	1.0888
78	2015	42	18.1857	87.2449	907.2980	2.5898	1.4469
28	2015	42	18.4714	83.5918	936.8571	3.2959	1.3102
39	2015	42	17.5857	86.6531	909.8286	2.6000	2.2714
24	2015	42	18.7143	82.8367	948.0949	2.7908	1.0051
63	2015	42	18.3143	83.5816	953.0235	4.3347	1.0888
62	2015	42	17.1000	88.1531	882.5010	2.1929	0.5969
1	2015	42	17.3143	85.4286	882.6235	2.6337	1.9398
31	2015	43	17.6286	82.9388	853.0235	3.8469	0.8776

79	2015	43	20.8571	73.2755	976.9092	6.0612	0.8622
51	2015	43	18.5571	76.6735	946.4735	6.1612	0.9990
14	2015	43	19.0857	76.3673	904.3357	5.3284	0.7194
67	2015	43	18.9714	79.0714	909.2735	4.9204	1.8061
42	2015	43	18.4286	77.3469	881.8939	5.5255	1.9010
50	2015	43	19.9143	75.2347	908.9776	5.1980	1.2959
43	2015	43	18.4286	77.3469	881.8939	5.5255	1.9010
85	2015	43	21.3571	73.7959	917.3092	4.5684	1.2571
25	2015	43	20.9286	81.0204	984.4163	5.5684	0.9969
69	2015	43	19.7714	78.0204	947.3439	4.8480	1.0531
57	2015	43	18.5714	75.3469	893.8092	4.0653	1.7000
9	2015	43	17.8571	78.0714	861.0765	5.7755	1.3776
72	2015	43	18.7000	80.8367	884.7735	4.6418	1.1224
26	2015	43	18.9714	82.0306	874.2112	5.1857	1.4714
7	2015	43	18.7286	81.1633	866.2929	4.4653	1.2388
83	2015	43	22.0857	81.9286	951.9918	6.0163	1.1255
76	2015	43	19.5571	78.0408	928.3459	4.4092	1.2490
36	2015	43	19.6000	77.7653	936.1786	5.7143	1.3429
81	2015	43	18.5571	76.6735	946.4735	6.1612	0.9990
15	2015	43	20.2571	82.1837	940.4459	3.6765	0.5541
32	2015	43	18.4286	77.3469	881.8939	5.5255	1.9010
73	2015	43	21.0429	74.9490	969.3714	4.6633	0.8765
71	2015	43	19.6000	77.7653	936.1786	5.7143	1.3429
41	2015	43	18.7857	82.6531	881.4980	4.1449	0.8194
10	2015	43	19.2000	77.8367	971.4092	7.1643	0.9429
23	2015	43	14.6143	80.0204	782.4357	4.7867	1.8867
27	2015	43	18.7286	81.1633	866.2929	4.4653	1.2388
60	2015	43	18.5571	76.6735	946.4735	6.1612	0.9990
53	2015	43	17.8571	78.0714	861.0765	5.7755	1.3776
66	2015	43	19.0857	76.3673	904.3357	5.3284	0.7194
59	2015	43	18.5714	75.3469	893.8092	4.0653	1.7000
61	2015	43	21.0429	74.9490	969.3714	4.6633	0.8765
84	2015	43	21.0429	74.9490	969.3714	4.6633	0.8765
38	2015	43	18.5714	75.3469	893.8092	4.0653	1.7000
87	2015	43	19.7429	81.6531	906.6704	5.3367	0.9163
34	2015	43	18.5714	75.3469	893.8092	4.0653	1.7000
29	2015	43	19.7714	78.0204	947.3439	4.8480	1.0531
5	2015	43	17.1714	83.2959	839.1878	4.0622	1.1847
8	2015	43	17.8571	78.0714	861.0765	5.7755	1.3776
12	2015	43	17.1714	83.2959	839.1878	4.0622	1.1847
13	2015	43	22.0857	81.9286	951.9918	6.0163	1.1255
18	2015	43	20.4286	75.4490	971.3449	5.7102	0.8357
33	2015	43	19.9143	75.2347	908.9776	5.1980	1.2959
56	2015	43	20.9286	81.0204	984.4163	5.5684	0.9969
77	2015	43	20.2571	82.1837	940.4459	3.6765	0.5541
54	2015	43	17.1714	83.2959	839.1878	4.0622	1.1847
21	2015	43	19.9143	75.2347	908.9776	5.1980	1.2959
68	2015	43	20.8571	73.2755	976.9092	6.0612	0.8622
74	2015	43	21.0429	74.9490	969.3714	4.6633	0.8765
88	2015	43	18.4286	77.3469	881.8939	5.5255	1.9010

16	2015	43	19.5571	78.0408	928.3459	4.4092	1.2490
30	2015	43	19.0857	76.3673	904.3357	5.3284	0.7194
6	2015	43	20.8571	73.2755	976.9092	6.0612	0.8622
49	2015	43	19.7714	78.0204	947.3439	4.8480	1.0531
22	2015	43	18.4286	77.3469	881.8939	5.5255	1.9010
45	2015	43	16.5286	81.9694	824.2449	4.4612	1.3765
58	2015	43	19.7714	78.0204	947.3439	4.8480	1.0531
37	2015	43	20.8571	73.2755	976.9092	6.0612	0.8622
17	2015	43	18.9714	79.0714	909.2735	4.9204	1.8061
55	2015	43	18.7000	80.8367	884.7735	4.6418	1.1224
46	2015	43	19.5571	78.0408	928.3459	4.4092	1.2490
86	2015	43	19.0000	80.3469	873.0245	4.9786	1.1020
2	2015	43	19.0000	80.3469	873.0245	4.9786	1.1020
4	2015	43	19.9143	75.2347	908.9776	5.1980	1.2959
47	2015	43	22.0714	80.6735	966.1041	4.8561	0.5832
82	2015	43	18.4286	77.3469	881.8939	5.5255	1.9010
19	2015	43	21.4429	79.8673	967.6602	5.7204	0.8980
20	2015	43	17.8571	78.0714	861.0765	5.7755	1.3776
80	2015	43	18.4286	77.3469	881.8939	5.5255	1.9010
3	2015	43	22.0857	81.9286	951.9918	6.0163	1.1255
52	2015	43	18.9714	79.0714	909.2735	4.9204	1.8061
70	2015	43	21.3571	73.7959	917.3092	4.5684	1.2571
64	2015	43	14.6143	80.0204	782.4357	4.7867	1.8867
48	2015	43	20.2571	82.1837	940.4459	3.6765	0.5541
65	2015	43	18.9714	79.0714	909.2735	4.9204	1.8061
44	2015	43	21.3571	73.7959	917.3092	4.5684	1.2571
75	2015	43	14.6143	80.0204	782.4357	4.7867	1.8867
40	2015	43	18.9286	78.8980	952.5296	7.4031	0.7224
11	2015	43	18.7000	80.8367	884.7735	4.6418	1.1224
35	2015	43	18.5571	76.6735	946.4735	6.1612	0.9990
78	2015	43	19.7429	81.6531	906.6704	5.3367	0.9163
28	2015	43	19.6000	77.7653	936.1786	5.7143	1.3429
39	2015	43	18.9714	79.0714	909.2735	4.9204	1.8061
24	2015	43	19.7714	78.0204	947.3439	4.8480	1.0531
63	2015	43	18.9286	78.8980	952.5296	7.4031	0.7224
62	2015	43	18.7857	82.6531	881.4980	4.1449	0.8194
1	2015	43	18.4286	77.3469	881.8939	5.5255	1.9010
31	2015	44	13.3714	81.4490	851.1633	4.8602	0.9571
79	2015	44	14.7857	75.2857	976.1102	5.6806	1.2439
51	2015	44	13.0857	78.4082	945.4010	6.4622	1.2112
14	2015	44	16.1143	75.9082	903.0316	5.3061	1.4827
67	2015	44	14.6714	79.9490	907.8898	4.6143	2.3408
42	2015	44	14.7857	76.7959	880.2827	6.1327	2.2776
50	2015	44	14.2429	74.2959	907.3408	4.6786	1.7633
43	2015	44	14.7857	76.7959	880.2827	6.1327	2.2776
85	2015	44	14.9857	71.5306	915.5490	4.1235	1.6643
25	2015	44	18.5714	80.1122	983.0153	5.5827	1.0857
69	2015	44	15.5571	76.7959	945.9102	4.7684	1.3694
57	2015	44	13.4857	74.6735	892.1071	4.3510	2.4735
9	2015	44	14.8429	79.2551	859.4724	6.2296	2.0663

72	2015	44	16.8143	78.7245	883.1755	5.3939	1.4122
26	2015	44	17.5714	81.8061	872.5153	7.0724	2.0786
7	2015	44	16.8857	79.3776	864.5969	6.0439	1.6939
83	2015	44	20.8143	80.1429	950.0112	7.0429	1.1480
76	2015	44	14.6286	77.3469	926.8765	4.0061	1.4714
36	2015	44	14.7143	76.7653	934.8061	5.6857	1.5888
81	2015	44	13.0857	78.4082	945.4010	6.4622	1.2112
15	2015	44	15.7571	82.9388	938.9704	2.3939	0.7663
32	2015	44	14.7857	76.7959	880.2827	6.1327	2.2776
73	2015	44	16.8143	74.8571	968.1122	4.0837	0.9265
71	2015	44	14.7143	76.7653	934.8061	5.6857	1.5888
41	2015	44	14.6000	81.2041	879.6980	4.2776	1.1949
10	2015	44	14.6857	80.0000	970.4704	6.6939	1.0235
23	2015	44	10.9000	76.0816	780.7898	7.0857	2.3276
27	2015	44	16.8857	79.3776	864.5969	6.0439	1.6939
60	2015	44	13.0857	78.4082	945.4010	6.4622	1.2112
53	2015	44	14.8429	79.2551	859.4724	6.2296	2.0663
66	2015	44	16.1143	75.9082	903.0316	5.3061	1.4827
59	2015	44	13.4857	74.6735	892.1071	4.3510	2.4735
61	2015	44	16.8143	74.8571	968.1122	4.0837	0.9265
84	2015	44	16.8143	74.8571	968.1122	4.0837	0.9265
38	2015	44	13.4857	74.6735	892.1071	4.3510	2.4735
87	2015	44	17.5000	78.7347	904.9786	6.1255	1.2061
34	2015	44	13.4857	74.6735	892.1071	4.3510	2.4735
29	2015	44	15.5571	76.7959	945.9102	4.7684	1.3694
5	2015	44	15.1857	79.3367	837.4969	6.2684	1.5449
8	2015	44	14.8429	79.2551	859.4724	6.2296	2.0663
12	2015	44	15.1857	79.3367	837.4969	6.2684	1.5449
13	2015	44	20.8143	80.1429	950.0112	7.0429	1.1480
18	2015	44	14.7714	76.0612	970.5755	5.0194	1.0449
33	2015	44	14.2429	74.2959	907.3408	4.6786	1.7633
56	2015	44	18.5714	80.1122	983.0153	5.5827	1.0857
77	2015	44	15.7571	82.9388	938.9704	2.3939	0.7663
54	2015	44	15.1857	79.3367	837.4969	6.2684	1.5449
21	2015	44	14.2429	74.2959	907.3408	4.6786	1.7633
68	2015	44	14.7857	75.2857	976.1102	5.6806	1.2439
74	2015	44	16.8143	74.8571	968.1122	4.0837	0.9265
88	2015	44	14.7857	76.7959	880.2827	6.1327	2.2776
16	2015	44	14.6286	77.3469	926.8765	4.0061	1.4714
30	2015	44	16.1143	75.9082	903.0316	5.3061	1.4827
6	2015	44	14.7857	75.2857	976.1102	5.6806	1.2439
49	2015	44	15.5571	76.7959	945.9102	4.7684	1.3694
22	2015	44	14.7857	76.7959	880.2827	6.1327	2.2776
45	2015	44	14.8571	76.1837	822.6071	7.1480	1.8541
58	2015	44	15.5571	76.7959	945.9102	4.7684	1.3694
37	2015	44	14.7857	75.2857	976.1102	5.6806	1.2439
17	2015	44	14.6714	79.9490	907.8898	4.6143	2.3408
55	2015	44	16.8143	78.7245	883.1755	5.3939	1.4122
46	2015	44	14.6286	77.3469	926.8765	4.0061	1.4714
86	2015	44	15.4571	77.3061	871.1918	5.2561	1.8653

2	2015	44	15.4571	77.3061	871.1918	5.2561	1.8653
4	2015	44	14.2429	74.2959	907.3408	4.6786	1.7633
47	2015	44	20.6286	80.1224	964.1510	5.8469	0.5980
82	2015	44	14.7857	76.7959	880.2827	6.1327	2.2776
19	2015	44	20.0429	78.7755	966.1551	6.2194	0.9276
20	2015	44	14.8429	79.2551	859.4724	6.2296	2.0663
80	2015	44	14.7857	76.7959	880.2827	6.1327	2.2776
3	2015	44	20.8143	80.1429	950.0112	7.0429	1.1480
52	2015	44	14.6714	79.9490	907.8898	4.6143	2.3408
70	2015	44	14.9857	71.5306	915.5490	4.1235	1.6643
64	2015	44	10.9000	76.0816	780.7898	7.0857	2.3276
48	2015	44	15.7571	82.9388	938.9704	2.3939	0.7663
65	2015	44	14.6714	79.9490	907.8898	4.6143	2.3408
44	2015	44	14.9857	71.5306	915.5490	4.1235	1.6643
75	2015	44	10.9000	76.0816	780.7898	7.0857	2.3276
40	2015	44	14.5429	79.4796	951.4918	7.4204	1.1071
11	2015	44	16.8143	78.7245	883.1755	5.3939	1.4122
35	2015	44	13.0857	78.4082	945.4010	6.4622	1.2112
78	2015	44	17.5000	78.7347	904.9786	6.1255	1.2061
28	2015	44	14.7143	76.7653	934.8061	5.6857	1.5888
39	2015	44	14.6714	79.9490	907.8898	4.6143	2.3408
24	2015	44	15.5571	76.7959	945.9102	4.7684	1.3694
63	2015	44	14.5429	79.4796	951.4918	7.4204	1.1071
62	2015	44	14.6000	81.2041	879.6980	4.2776	1.1949
1	2015	44	14.7857	76.7959	880.2827	6.1327	2.2776
31	2015	45	15.1714	79.7143	851.8133	3.8337	1.0561
79	2015	45	15.4571	78.8061	978.8980	2.6959	1.4102
51	2015	45	15.4286	82.6224	947.5449	3.5490	1.5194
14	2015	45	17.1429	80.7959	904.5367	2.8612	2.4163
67	2015	45	17.1571	86.0408	909.4184	2.2143	3.1378
42	2015	45	16.6000	80.5612	881.3418	4.0969	2.6204
50	2015	45	16.2286	75.8571	908.6806	2.9765	1.8582
43	2015	45	16.6000	80.5612	881.3418	4.0969	2.6204
85	2015	45	16.3000	74.2041	917.0214	2.6398	1.7571
25	2015	45	19.5000	81.8673	985.4449	3.3367	1.0806
69	2015	45	18.2143	78.6429	947.8204	2.5969	1.6694
57	2015	45	17.1286	75.6735	893.2459	3.2276	3.0163
9	2015	45	15.8143	82.8776	860.3010	4.1541	2.6500
72	2015	45	16.9857	81.6122	884.2673	3.7694	1.8633
26	2015	45	17.0429	83.5306	873.4582	5.8867	2.2714
7	2015	45	17.0000	80.2959	865.5102	5.2357	2.1806
83	2015	45	20.4143	82.2959	951.7612	5.1316	1.0939
76	2015	45	16.1000	80.4184	928.5806	2.4112	1.6061
36	2015	45	18.1571	80.6224	936.7061	3.1888	1.7398
81	2015	45	15.4286	82.6224	947.5449	3.5490	1.5194
15	2015	45	16.6714	84.3367	940.7235	1.2837	0.7949
32	2015	45	16.6000	80.5612	881.3418	4.0969	2.6204
73	2015	45	17.4429	75.5510	970.4071	2.0622	0.9622
71	2015	45	18.1571	80.6224	936.7061	3.1888	1.7398
41	2015	45	17.2429	81.4694	880.6347	3.4490	1.5582

10	2015	45	16.2714	84.4898	973.0520	3.3153	0.9643
23	2015	45	12.4714	76.2143	780.8908	5.9520	2.4204
27	2015	45	17.0000	80.2959	865.5102	5.2357	2.1806
60	2015	45	15.4286	82.6224	947.5449	3.5490	1.5194
53	2015	45	15.8143	82.8776	860.3010	4.1541	2.6500
66	2015	45	17.1429	80.7959	904.5367	2.8612	2.4163
59	2015	45	17.1286	75.6735	893.2459	3.2276	3.0163
61	2015	45	17.4429	75.5510	970.4071	2.0622	0.9622
84	2015	45	17.4429	75.5510	970.4071	2.0622	0.9622
38	2015	45	17.1286	75.6735	893.2459	3.2276	3.0163
87	2015	45	18.2857	80.1224	906.3143	3.7163	1.7724
34	2015	45	17.1286	75.6735	893.2459	3.2276	3.0163
29	2015	45	18.2143	78.6429	947.8204	2.5969	1.6694
5	2015	45	16.2286	78.1939	838.0837	6.1908	1.9306
8	2015	45	15.8143	82.8776	860.3010	4.1541	2.6500
12	2015	45	16.2286	78.1939	838.0837	6.1908	1.9306
13	2015	45	20.4143	82.2959	951.7612	5.1316	1.0939
18	2015	45	14.9286	81.7755	973.2694	1.9837	1.1296
33	2015	45	16.2286	75.8571	908.6806	2.9765	1.8582
56	2015	45	19.5000	81.8673	985.4449	3.3367	1.0806
77	2015	45	16.6714	84.3367	940.7235	1.2837	0.7949
54	2015	45	16.2286	78.1939	838.0837	6.1908	1.9306
21	2015	45	16.2286	75.8571	908.6806	2.9765	1.8582
68	2015	45	15.4571	78.8061	978.8980	2.6959	1.4102
74	2015	45	17.4429	75.5510	970.4071	2.0622	0.9622
88	2015	45	16.6000	80.5612	881.3418	4.0969	2.6204
16	2015	45	16.1000	80.4184	928.5806	2.4112	1.6061
30	2015	45	17.1429	80.7959	904.5367	2.8612	2.4163
6	2015	45	15.4571	78.8061	978.8980	2.6959	1.4102
49	2015	45	18.2143	78.6429	947.8204	2.5969	1.6694
22	2015	45	16.6000	80.5612	881.3418	4.0969	2.6204
45	2015	45	15.5571	73.2653	823.0786	6.6847	2.1092
58	2015	45	18.2143	78.6429	947.8204	2.5969	1.6694
37	2015	45	15.4571	78.8061	978.8980	2.6959	1.4102
17	2015	45	17.1571	86.0408	909.4184	2.2143	3.1378
55	2015	45	16.9857	81.6122	884.2673	3.7694	1.8633
46	2015	45	16.1000	80.4184	928.5806	2.4112	1.6061
86	2015	45	16.9714	78.7653	872.1071	2.9643	2.3224
2	2015	45	16.9714	78.7653	872.1071	2.9643	2.3224
4	2015	45	16.2286	75.8571	908.6806	2.9765	1.8582
47	2015	45	20.6571	80.3265	966.1265	3.7990	0.6224
82	2015	45	16.6000	80.5612	881.3418	4.0969	2.6204
19	2015	45	19.9286	82.1327	968.3980	3.5480	1.0133
20	2015	45	15.8143	82.8776	860.3010	4.1541	2.6500
80	2015	45	16.6000	80.5612	881.3418	4.0969	2.6204
3	2015	45	20.4143	82.2959	951.7612	5.1316	1.0939
52	2015	45	17.1571	86.0408	909.4184	2.2143	3.1378
70	2015	45	16.3000	74.2041	917.0214	2.6398	1.7571
64	2015	45	12.4714	76.2143	780.8908	5.9520	2.4204
48	2015	45	16.6714	84.3367	940.7235	1.2837	0.7949

65	2015	45	17.1571	86.0408	909.4184	2.2143	3.1378
44	2015	45	16.3000	74.2041	917.0214	2.6398	1.7571
75	2015	45	12.4714	76.2143	780.8908	5.9520	2.4204
40	2015	45	17.3857	84.8776	953.7531	3.9714	1.5357
11	2015	45	16.9857	81.6122	884.2673	3.7694	1.8633
35	2015	45	15.4286	82.6224	947.5449	3.5490	1.5194
78	2015	45	18.2857	80.1224	906.3143	3.7163	1.7724
28	2015	45	18.1571	80.6224	936.7061	3.1888	1.7398
39	2015	45	17.1571	86.0408	909.4184	2.2143	3.1378
24	2015	45	18.2143	78.6429	947.8204	2.5969	1.6694
63	2015	45	17.3857	84.8776	953.7531	3.9714	1.5357
62	2015	45	17.2429	81.4694	880.6347	3.4490	1.5582
1	2015	45	16.6000	80.5612	881.3418	4.0969	2.6204
31	2015	46	12.7571	80.7755	851.2265	3.2418	1.0490
79	2015	46	12.1429	81.5918	979.6133	0.9500	1.5082
51	2015	46	10.8143	84.3878	947.8735	2.2704	1.5959
14	2015	46	12.1000	84.6020	904.4235	2.0714	2.4184
67	2015	46	10.9143	89.8571	909.3704	1.4327	3.4765
42	2015	46	11.5000	83.8571	880.9980	2.6969	2.9133
50	2015	46	11.7571	77.8673	908.4857	2.0990	1.9490
43	2015	46	11.5000	83.8571	880.9980	2.6969	2.9133
85	2015	46	13.0000	78.9388	916.9020	2.0163	1.6194
25	2015	46	15.0857	82.6020	986.0092	2.0714	1.1367
69	2015	46	12.6000	78.7245	948.0531	1.8837	1.7214
57	2015	46	10.6857	78.0918	892.9571	2.5194	2.9408
9	2015	46	13.3286	86.1633	859.8010	3.9071	2.8051
72	2015	46	14.8857	86.4388	883.8480	3.3724	2.0112
26	2015	46	17.0143	82.8265	872.9888	5.7031	2.3439
7	2015	46	16.3429	81.3469	865.0173	5.1347	2.3541
83	2015	46	19.6714	84.0918	951.5837	4.5357	1.0306
76	2015	46	10.9429	83.0408	928.6541	2.0286	1.7051
36	2015	46	11.8714	81.9490	936.8663	1.9031	1.8663
81	2015	46	10.8143	84.3878	947.8735	2.2704	1.5959
15	2015	46	12.4143	85.5510	940.8531	1.5143	0.8459
32	2015	46	11.5000	83.8571	880.9980	2.6969	2.9133
73	2015	46	13.4000	76.5612	970.8898	1.3235	1.0388
71	2015	46	11.8714	81.9490	936.8663	1.9031	1.8663
41	2015	46	12.0857	83.8776	880.2531	3.2643	1.7837
10	2015	46	12.2571	85.4286	973.6010	1.7051	1.0286
23	2015	46	11.7571	73.4796	780.1969	6.1184	2.3357
27	2015	46	16.3429	81.3469	865.0173	5.1347	2.3541
60	2015	46	10.8143	84.3878	947.8735	2.2704	1.5959
53	2015	46	13.3286	86.1633	859.8010	3.9071	2.8051
66	2015	46	12.1000	84.6020	904.4235	2.0714	2.4184
59	2015	46	10.6857	78.0918	892.9571	2.5194	2.9408
61	2015	46	13.4000	76.5612	970.8898	1.3235	1.0388
84	2015	46	13.4000	76.5612	970.8898	1.3235	1.0388
38	2015	46	10.6857	78.0918	892.9571	2.5194	2.9408
87	2015	46	14.7714	83.0612	906.0571	2.3847	1.9796
34	2015	46	10.6857	78.0918	892.9571	2.5194	2.9408

29	2015	46	12.6000	78.7245	948.0531	1.8837	1.7214
5	2015	46	14.4714	77.8571	837.4990	6.1449	2.1918
8	2015	46	13.3286	86.1633	859.8010	3.9071	2.8051
12	2015	46	14.4714	77.8571	837.4990	6.1449	2.1918
13	2015	46	19.6714	84.0918	951.5837	4.5357	1.0306
18	2015	46	12.1000	85.4898	973.8980	0.6929	1.0173
33	2015	46	11.7571	77.8673	908.4857	2.0990	1.9490
56	2015	46	15.0857	82.6020	986.0092	2.0714	1.1367
77	2015	46	12.4143	85.5510	940.8531	1.5143	0.8459
54	2015	46	14.4714	77.8571	837.4990	6.1449	2.1918
21	2015	46	11.7571	77.8673	908.4857	2.0990	1.9490
68	2015	46	12.1429	81.5918	979.6133	0.9500	1.5082
74	2015	46	13.4000	76.5612	970.8898	1.3235	1.0388
88	2015	46	11.5000	83.8571	880.9980	2.6969	2.9133
16	2015	46	10.9429	83.0408	928.6541	2.0286	1.7051
30	2015	46	12.1000	84.6020	904.4235	2.0714	2.4184
6	2015	46	12.1429	81.5918	979.6133	0.9500	1.5082
49	2015	46	12.6000	78.7245	948.0531	1.8837	1.7214
22	2015	46	11.5000	83.8571	880.9980	2.6969	2.9133
45	2015	46	14.7857	71.8469	822.5378	6.6745	2.1980
58	2015	46	12.6000	78.7245	948.0531	1.8837	1.7214
37	2015	46	12.1429	81.5918	979.6133	0.9500	1.5082
17	2015	46	10.9143	89.8571	909.3704	1.4327	3.4765
55	2015	46	14.8857	86.4388	883.8480	3.3724	2.0112
46	2015	46	10.9429	83.0408	928.6541	2.0286	1.7051
86	2015	46	13.3000	81.5408	871.6765	1.7173	2.2204
2	2015	46	13.3000	81.5408	871.6765	1.7173	2.2204
4	2015	46	11.7571	77.8673	908.4857	2.0990	1.9490
47	2015	46	18.1286	80.8367	966.2143	2.4418	0.5684
82	2015	46	11.5000	83.8571	880.9980	2.6969	2.9133
19	2015	46	16.0143	83.9694	968.7612	2.0173	1.1735
20	2015	46	13.3286	86.1633	859.8010	3.9071	2.8051
80	2015	46	11.5000	83.8571	880.9980	2.6969	2.9133
3	2015	46	19.6714	84.0918	951.5837	4.5357	1.0306
52	2015	46	10.9143	89.8571	909.3704	1.4327	3.4765
70	2015	46	13.0000	78.9388	916.9020	2.0163	1.6194
64	2015	46	11.7571	73.4796	780.1969	6.1184	2.3357
48	2015	46	12.4143	85.5510	940.8531	1.5143	0.8459
65	2015	46	10.9143	89.8571	909.3704	1.4327	3.4765
44	2015	46	13.0000	78.9388	916.9020	2.0163	1.6194
75	2015	46	11.7571	73.4796	780.1969	6.1184	2.3357
40	2015	46	11.7571	86.9796	954.1480	2.0786	1.7439
11	2015	46	14.8857	86.4388	883.8480	3.3724	2.0112
35	2015	46	10.8143	84.3878	947.8735	2.2704	1.5959
78	2015	46	14.7714	83.0612	906.0571	2.3847	1.9796
28	2015	46	11.8714	81.9490	936.8663	1.9031	1.8663
39	2015	46	10.9143	89.8571	909.3704	1.4327	3.4765
24	2015	46	12.6000	78.7245	948.0531	1.8837	1.7214
63	2015	46	11.7571	86.9796	954.1480	2.0786	1.7439
62	2015	46	12.0857	83.8776	880.2531	3.2643	1.7837

1	2015	46	11.5000	83.8571	880.9980	2.6969	2.9133
31	2015	47	14.0000	83.2347	849.2724	2.6204	0.9398
79	2015	47	13.1000	86.6939	977.7316	0.3765	1.5378
51	2015	47	11.9857	89.0204	945.9531	1.1306	1.5939
14	2015	47	15.3571	89.1837	902.1663	1.8276	2.0327
67	2015	47	13.2714	94.1327	907.2827	1.0633	3.1204
42	2015	47	14.4571	87.4388	878.8694	2.2051	2.7776
50	2015	47	13.7714	82.0306	906.5796	0.9592	1.8939
43	2015	47	14.4571	87.4388	878.8694	2.2051	2.7776
85	2015	47	14.8714	82.3163	914.9612	1.1337	1.4694
25	2015	47	16.9857	84.3776	983.8837	1.2633	0.9449
69	2015	47	14.4714	82.5510	946.1898	1.1378	1.4531
57	2015	47	12.9857	84.1122	891.0276	1.9010	2.5357
9	2015	47	15.7571	85.7755	857.6510	4.6602	2.7082
72	2015	47	17.4000	86.6633	881.5112	4.0429	1.8602
26	2015	47	17.9000	79.0510	870.6776	6.7418	2.4765
7	2015	47	17.2286	79.0918	862.7398	5.6500	2.1010
83	2015	47	21.3000	82.9388	948.8296	5.7684	1.0357
76	2015	47	12.8714	86.8265	926.7918	1.3296	1.7796
36	2015	47	13.3571	86.5816	934.8265	1.3194	1.8265
81	2015	47	11.9857	89.0204	945.9531	1.1306	1.5939
15	2015	47	14.0286	88.9082	939.0816	0.7102	0.8337
32	2015	47	14.4571	87.4388	878.8694	2.2051	2.7776
73	2015	47	14.9714	80.8673	969.0714	0.8194	0.9418
71	2015	47	13.3571	86.5816	934.8265	1.3194	1.8265
41	2015	47	14.0571	88.9082	878.3255	2.6949	1.3337
10	2015	47	13.2857	88.7143	971.6582	0.6949	1.0724
23	2015	47	11.2429	70.3469	778.3673	7.1367	2.2286
27	2015	47	17.2286	79.0918	862.7398	5.6500	2.1010
60	2015	47	11.9857	89.0204	945.9531	1.1306	1.5939
53	2015	47	15.7571	85.7755	857.6510	4.6602	2.7082
66	2015	47	15.3571	89.1837	902.1663	1.8276	2.0327
59	2015	47	12.9857	84.1122	891.0276	1.9010	2.5357
61	2015	47	14.9714	80.8673	969.0714	0.8194	0.9418
84	2015	47	14.9714	80.8673	969.0714	0.8194	0.9418
38	2015	47	12.9857	84.1122	891.0276	1.9010	2.5357
87	2015	47	17.7286	85.7041	903.7163	2.6112	1.8020
34	2015	47	12.9857	84.1122	891.0276	1.9010	2.5357
29	2015	47	14.4714	82.5510	946.1898	1.1378	1.4531
5	2015	47	15.0857	77.5510	835.4265	6.4582	2.0204
8	2015	47	15.7571	85.7755	857.6510	4.6602	2.7082
12	2015	47	15.0857	77.5510	835.4265	6.4582	2.0204
13	2015	47	21.3000	82.9388	948.8296	5.7684	1.0357
18	2015	47	13.1857	88.6020	971.9949	0.4724	0.8847
33	2015	47	13.7714	82.0306	906.5796	0.9592	1.8939
56	2015	47	16.9857	84.3776	983.8837	1.2633	0.9449
77	2015	47	14.0286	88.9082	939.0816	0.7102	0.8337
54	2015	47	15.0857	77.5510	835.4265	6.4582	2.0204
21	2015	47	13.7714	82.0306	906.5796	0.9592	1.8939
68	2015	47	13.1000	86.6939	977.7316	0.3765	1.5378

74	2015	47	14.9714	80.8673	969.0714	0.8194	0.9418
88	2015	47	14.4571	87.4388	878.8694	2.2051	2.7776
16	2015	47	12.8714	86.8265	926.7918	1.3296	1.7796
30	2015	47	15.3571	89.1837	902.1663	1.8276	2.0327
6	2015	47	13.1000	86.6939	977.7316	0.3765	1.5378
49	2015	47	14.4714	82.5510	946.1898	1.1378	1.4531
22	2015	47	14.4571	87.4388	878.8694	2.2051	2.7776
45	2015	47	14.8286	67.1224	820.5459	7.4337	2.3020
58	2015	47	14.4714	82.5510	946.1898	1.1378	1.4531
37	2015	47	13.1000	86.6939	977.7316	0.3765	1.5378
17	2015	47	13.2714	94.1327	907.2827	1.0633	3.1204
55	2015	47	17.4000	86.6633	881.5112	4.0429	1.8602
46	2015	47	12.8714	86.8265	926.7918	1.3296	1.7796
86	2015	47	14.9571	84.4490	869.6204	1.4051	1.6551
2	2015	47	14.9571	84.4490	869.6204	1.4051	1.6551
4	2015	47	13.7714	82.0306	906.5796	0.9592	1.8939
47	2015	47	21.4000	81.2653	963.5398	2.9469	0.5041
82	2015	47	14.4571	87.4388	878.8694	2.2051	2.7776
19	2015	47	18.9429	85.5408	966.3735	1.7745	1.2531
20	2015	47	15.7571	85.7755	857.6510	4.6602	2.7082
80	2015	47	14.4571	87.4388	878.8694	2.2051	2.7776
3	2015	47	21.3000	82.9388	948.8296	5.7684	1.0357
52	2015	47	13.2714	94.1327	907.2827	1.0633	3.1204
70	2015	47	14.8714	82.3163	914.9612	1.1337	1.4694
64	2015	47	11.2429	70.3469	778.3673	7.1367	2.2286
48	2015	47	14.0286	88.9082	939.0816	0.7102	0.8337
65	2015	47	13.2714	94.1327	907.2827	1.0633	3.1204
44	2015	47	14.8714	82.3163	914.9612	1.1337	1.4694
75	2015	47	11.2429	70.3469	778.3673	7.1367	2.2286
40	2015	47	12.8857	90.8980	952.1592	1.0918	1.7235
11	2015	47	17.4000	86.6633	881.5112	4.0429	1.8602
35	2015	47	11.9857	89.0204	945.9531	1.1306	1.5939
78	2015	47	17.7286	85.7041	903.7163	2.6112	1.8020
28	2015	47	13.3571	86.5816	934.8265	1.3194	1.8265
39	2015	47	13.2714	94.1327	907.2827	1.0633	3.1204
24	2015	47	14.4714	82.5510	946.1898	1.1378	1.4531
63	2015	47	12.8857	90.8980	952.1592	1.0918	1.7235
62	2015	47	14.0571	88.9082	878.3255	2.6949	1.3337
1	2015	47	14.4571	87.4388	878.8694	2.2051	2.7776
31	2015	48	9.8286	82.1531	850.1786	2.2602	0.9367
79	2015	48	8.4857	87.9184	979.2245	0.3357	1.7663
51	2015	48	7.9571	92.1224	947.2265	0.2643	1.5633
14	2015	48	10.4857	89.0510	903.0265	1.4755	2.0357
67	2015	48	10.0571	94.5204	908.1816	0.9071	2.6296
42	2015	48	10.5429	85.6837	879.7061	2.2561	2.5337
50	2015	48	10.9143	83.4898	907.5827	0.3367	1.6694
43	2015	48	10.5429	85.6837	879.7061	2.2561	2.5337
85	2015	48	12.1000	82.7551	915.9041	0.8694	1.4582
25	2015	48	12.7571	86.3878	985.1888	0.8622	0.6990
69	2015	48	10.5286	84.5204	947.4429	0.3878	1.5398

57	2015	48	10.0286	84.9694	891.9765	1.4112	2.3939
9	2015	48	10.6714	82.3878	858.4857	4.2857	2.7357
72	2015	48	12.1857	82.4796	882.3878	4.2214	2.0673
26	2015	48	12.3857	78.7551	871.5561	5.9173	2.2286
7	2015	48	11.9571	77.3571	863.6092	4.9459	1.8990
83	2015	48	15.9143	82.2857	950.0102	5.8898	1.0500
76	2015	48	9.5571	88.8776	927.9898	0.2541	1.8276
36	2015	48	9.5000	89.6020	935.9633	0.9663	1.6347
81	2015	48	7.9571	92.1224	947.2265	0.2643	1.5633
15	2015	48	11.1857	91.3265	940.3357	0.0347	0.7429
32	2015	48	10.5429	85.6837	879.7061	2.2561	2.5337
73	2015	48	11.4286	82.3571	970.4643	0.2143	0.8408
71	2015	48	9.5000	89.6020	935.9633	0.9663	1.6347
41	2015	48	11.0714	88.1939	879.2194	2.5827	1.0898
10	2015	48	8.9857	91.0408	973.1112	0.1276	1.0378
23	2015	48	6.9429	75.2449	778.9776	5.5724	2.1735
27	2015	48	11.9571	77.3571	863.6092	4.9459	1.8990
60	2015	48	7.9571	92.1224	947.2265	0.2643	1.5633
53	2015	48	10.6714	82.3878	858.4857	4.2857	2.7357
66	2015	48	10.4857	89.0510	903.0265	1.4755	2.0357
59	2015	48	10.0286	84.9694	891.9765	1.4112	2.3939
61	2015	48	11.4286	82.3571	970.4643	0.2143	0.8408
84	2015	48	11.4286	82.3571	970.4643	0.2143	0.8408
38	2015	48	10.0286	84.9694	891.9765	1.4112	2.3939
87	2015	48	12.8286	82.9898	904.6490	2.6827	1.7673
34	2015	48	10.0286	84.9694	891.9765	1.4112	2.3939
29	2015	48	10.5286	84.5204	947.4429	0.3878	1.5398
5	2015	48	10.2000	78.9082	836.2551	5.5000	1.7286
8	2015	48	10.6714	82.3878	858.4857	4.2857	2.7357
12	2015	48	10.2000	78.9082	836.2551	5.5000	1.7286
13	2015	48	15.9143	82.2857	950.0102	5.8898	1.0500
18	2015	48	8.8857	91.3673	973.4184	0.1235	0.8020
33	2015	48	10.9143	83.4898	907.5827	0.3367	1.6694
56	2015	48	12.7571	86.3878	985.1888	0.8622	0.6990
77	2015	48	11.1857	91.3265	940.3357	0.0347	0.7429
54	2015	48	10.2000	78.9082	836.2551	5.5000	1.7286
21	2015	48	10.9143	83.4898	907.5827	0.3367	1.6694
68	2015	48	8.4857	87.9184	979.2245	0.3357	1.7663
74	2015	48	11.4286	82.3571	970.4643	0.2143	0.8408
88	2015	48	10.5429	85.6837	879.7061	2.2561	2.5337
16	2015	48	9.5571	88.8776	927.9898	0.2541	1.8276
30	2015	48	10.4857	89.0510	903.0265	1.4755	2.0357
6	2015	48	8.4857	87.9184	979.2245	0.3357	1.7663
49	2015	48	10.5286	84.5204	947.4429	0.3878	1.5398
22	2015	48	10.5429	85.6837	879.7061	2.2561	2.5337
45	2015	48	10.5571	68.9082	821.2245	6.3000	2.1143
58	2015	48	10.5286	84.5204	947.4429	0.3878	1.5398
37	2015	48	8.4857	87.9184	979.2245	0.3357	1.7663
17	2015	48	10.0571	94.5204	908.1816	0.9071	2.6296
55	2015	48	12.1857	82.4796	882.3878	4.2214	2.0673

46	2015	48	9.5571	88.8776	927.9898	0.2541	1.8276
86	2015	48	11.4571	82.7959	870.4990	1.4469	1.5531
2	2015	48	11.4571	82.7959	870.4990	1.4469	1.5531
4	2015	48	10.9143	83.4898	907.5827	0.3367	1.6694
47	2015	48	15.3857	81.2347	964.6980	3.2429	0.4949
82	2015	48	10.5429	85.6837	879.7061	2.2561	2.5337
19	2015	48	14.1286	86.3265	967.4653	1.3122	1.1684
20	2015	48	10.6714	82.3878	858.4857	4.2857	2.7357
80	2015	48	10.5429	85.6837	879.7061	2.2561	2.5337
3	2015	48	15.9143	82.2857	950.0102	5.8898	1.0500
52	2015	48	10.0571	94.5204	908.1816	0.9071	2.6296
70	2015	48	12.1000	82.7551	915.9041	0.8694	1.4582
64	2015	48	6.9429	75.2449	778.9776	5.5724	2.1735
48	2015	48	11.1857	91.3265	940.3357	0.0347	0.7429
65	2015	48	10.0571	94.5204	908.1816	0.9071	2.6296
44	2015	48	12.1000	82.7551	915.9041	0.8694	1.4582
75	2015	48	6.9429	75.2449	778.9776	5.5724	2.1735
40	2015	48	8.7000	94.2143	953.4153	0.4224	1.4378
11	2015	48	12.1857	82.4796	882.3878	4.2214	2.0673
35	2015	48	7.9571	92.1224	947.2265	0.2643	1.5633
78	2015	48	12.8286	82.9898	904.6490	2.6827	1.7673
28	2015	48	9.5000	89.6020	935.9633	0.9663	1.6347
39	2015	48	10.0571	94.5204	908.1816	0.9071	2.6296
24	2015	48	10.5286	84.5204	947.4429	0.3878	1.5398
63	2015	48	8.7000	94.2143	953.4153	0.4224	1.4378
62	2015	48	11.0714	88.1939	879.2194	2.5827	1.0898
1	2015	48	10.5429	85.6837	879.7061	2.2561	2.5337
31	2015	49	7.4286	81.4796	852.4653	2.1551	0.9153
79	2015	49	8.9429	86.2755	982.2347	0.7235	1.7112
51	2015	49	7.8571	90.1122	949.9684	0.4653	1.4235
14	2015	49	7.9857	87.1735	905.5327	1.2265	1.9796
67	2015	49	7.7000	91.9796	910.7143	0.8704	2.2449
42	2015	49	7.5143	83.1633	882.1133	2.0531	2.4418
50	2015	49	8.2143	81.1020	910.2541	0.6990	1.5551
43	2015	49	7.5143	83.1633	882.1133	2.0531	2.4418
85	2015	49	8.9000	80.6939	918.7745	1.1173	1.4776
25	2015	49	11.5000	87.1327	988.5112	1.0469	0.6673
69	2015	49	9.4857	82.7245	950.3041	0.6082	1.4378
57	2015	49	7.1571	83.0000	894.5347	1.4449	2.1898
9	2015	49	7.4857	83.5816	860.6980	2.6949	2.5990
72	2015	49	8.9000	82.8776	884.8480	2.6939	2.1224
26	2015	49	10.5000	84.3571	873.7980	3.1949	2.0337
7	2015	49	9.9571	80.4184	865.8133	3.0663	1.9490
83	2015	49	13.5714	83.9796	953.3786	3.4510	1.0133
76	2015	49	7.9857	86.3367	930.6714	0.4398	1.6561
36	2015	49	8.6857	87.8367	938.7673	1.0918	1.5031
81	2015	49	7.8571	90.1122	949.9684	0.4653	1.4235
15	2015	49	8.8857	90.4082	943.1449	0.0929	0.8531
32	2015	49	7.5143	83.1633	882.1133	2.0531	2.4418
73	2015	49	10.2000	81.2857	973.4480	0.5061	0.8959

71	2015	49	8.6857	87.8367	938.7673	1.0918	1.5031
41	2015	49	7.8000	85.9898	881.6735	2.2418	1.1133
10	2015	49	8.8143	89.5918	976.1367	0.4143	1.0714
23	2015	49	5.8286	83.1429	780.3204	3.7092	2.1184
27	2015	49	9.9571	80.4184	865.8133	3.0663	1.9490
60	2015	49	7.8571	90.1122	949.9684	0.4653	1.4235
53	2015	49	7.4857	83.5816	860.6980	2.6949	2.5990
66	2015	49	7.9857	87.1735	905.5327	1.2265	1.9796
59	2015	49	7.1571	83.0000	894.5347	1.4449	2.1898
61	2015	49	10.2000	81.2857	973.4480	0.5061	0.8959
84	2015	49	10.2000	81.2857	973.4480	0.5061	0.8959
38	2015	49	7.1571	83.0000	894.5347	1.4449	2.1898
87	2015	49	9.5429	82.5102	907.4082	2.1408	1.7306
34	2015	49	7.1571	83.0000	894.5347	1.4449	2.1898
29	2015	49	9.4857	82.7245	950.3041	0.6082	1.4378
5	2015	49	7.9857	84.3469	838.1469	3.1633	1.7735
8	2015	49	7.4857	83.5816	860.6980	2.6949	2.5990
12	2015	49	7.9857	84.3469	838.1469	3.1633	1.7735
13	2015	49	13.5714	83.9796	953.3786	3.4510	1.0133
18	2015	49	8.6571	90.9592	976.3265	0.3980	0.8316
33	2015	49	8.2143	81.1020	910.2541	0.6990	1.5551
56	2015	49	11.5000	87.1327	988.5112	1.0469	0.6673
77	2015	49	8.8857	90.4082	943.1449	0.0929	0.8531
54	2015	49	7.9857	84.3469	838.1469	3.1633	1.7735
21	2015	49	8.2143	81.1020	910.2541	0.6990	1.5551
68	2015	49	8.9429	86.2755	982.2347	0.7235	1.7112
74	2015	49	10.2000	81.2857	973.4480	0.5061	0.8959
88	2015	49	7.5143	83.1633	882.1133	2.0531	2.4418
16	2015	49	7.9857	86.3367	930.6714	0.4398	1.6561
30	2015	49	7.9857	87.1735	905.5327	1.2265	1.9796
6	2015	49	8.9429	86.2755	982.2347	0.7235	1.7112
49	2015	49	9.4857	82.7245	950.3041	0.6082	1.4378
22	2015	49	7.5143	83.1633	882.1133	2.0531	2.4418
45	2015	49	9.0714	78.6837	822.8122	4.7551	1.7867
58	2015	49	9.4857	82.7245	950.3041	0.6082	1.4378
37	2015	49	8.9429	86.2755	982.2347	0.7235	1.7112
17	2015	49	7.7000	91.9796	910.7143	0.8704	2.2449
55	2015	49	8.9000	82.8776	884.8480	2.6939	2.1224
46	2015	49	7.9857	86.3367	930.6714	0.4398	1.6561
86	2015	49	8.2571	81.7245	872.9031	1.3071	1.4265
2	2015	49	8.2571	81.7245	872.9031	1.3071	1.4265
4	2015	49	8.2143	81.1020	910.2541	0.6990	1.5551
47	2015	49	13.0286	82.4082	968.3276	1.9296	0.4571
82	2015	49	7.5143	83.1633	882.1133	2.0531	2.4418
19	2015	49	11.9714	84.4388	970.6786	1.1194	1.0857
20	2015	49	7.4857	83.5816	860.6980	2.6949	2.5990
80	2015	49	7.5143	83.1633	882.1133	2.0531	2.4418
3	2015	49	13.5714	83.9796	953.3786	3.4510	1.0133
52	2015	49	7.7000	91.9796	910.7143	0.8704	2.2449
70	2015	49	8.9000	80.6939	918.7745	1.1173	1.4776

64	2015	49	5.8286	83.1429	780.3204	3.7092	2.1184
48	2015	49	8.8857	90.4082	943.1449	0.0929	0.8531
65	2015	49	7.7000	91.9796	910.7143	0.8704	2.2449
44	2015	49	8.9000	80.6939	918.7745	1.1173	1.4776
75	2015	49	5.8286	83.1429	780.3204	3.7092	2.1184
40	2015	49	8.2000	92.9490	956.2316	0.5571	1.3031
11	2015	49	8.9000	82.8776	884.8480	2.6939	2.1224
35	2015	49	7.8571	90.1122	949.9684	0.4653	1.4235
78	2015	49	9.5429	82.5102	907.4082	2.1408	1.7306
28	2015	49	8.6857	87.8367	938.7673	1.0918	1.5031
39	2015	49	7.7000	91.9796	910.7143	0.8704	2.2449
24	2015	49	9.4857	82.7245	950.3041	0.6082	1.4378
63	2015	49	8.2000	92.9490	956.2316	0.5571	1.3031
62	2015	49	7.8000	85.9898	881.6735	2.2418	1.1133
1	2015	49	7.5143	83.1633	882.1133	2.0531	2.4418
31	2015	50	7.4571	85.9286	852.8837	0.6857	0.7918
79	2015	50	8.1143	83.8265	983.1643	0.9643	1.2112
51	2015	50	6.4714	88.0510	950.8745	0.7612	1.2653
14	2015	50	6.4143	89.2347	906.1847	0.8286	1.4255
67	2015	50	6.3143	92.2857	911.5561	0.6837	2.1959
42	2015	50	6.9000	85.8061	882.7827	1.1061	2.2888
50	2015	50	7.7714	80.1224	911.2459	0.7398	1.4561
43	2015	50	6.9000	85.8061	882.7827	1.1061	2.2888
85	2015	50	8.9286	80.0102	920.0041	0.6306	1.1398
25	2015	50	9.7857	87.1429	990.0337	1.3194	0.6388
69	2015	50	7.9571	83.0816	951.5245	0.9888	1.0714
57	2015	50	6.3714	85.9694	895.4776	0.7561	1.6102
9	2015	50	7.2571	88.7347	861.0867	0.4980	2.0990
72	2015	50	8.4571	89.1224	885.4714	0.5633	1.5684
26	2015	50	10.0857	89.9694	874.1898	1.0510	1.8531
7	2015	50	9.7571	86.1224	866.1673	0.9122	1.7735
83	2015	50	12.5857	87.9082	954.8602	0.9633	0.9010
76	2015	50	7.1000	84.6224	931.7000	0.7735	1.4041
36	2015	50	6.6286	88.4694	939.8653	0.8449	1.3949
81	2015	50	6.4714	88.0510	950.8745	0.7612	1.2653
15	2015	50	8.8429	89.1122	944.2898	0.8265	0.8408
32	2015	50	6.9000	85.8061	882.7827	1.1061	2.2888
73	2015	50	9.1000	81.7347	974.7786	0.9643	0.8990
71	2015	50	6.6286	88.4694	939.8653	0.8449	1.3949
41	2015	50	7.2000	89.8980	882.4878	0.6204	0.8918
10	2015	50	7.8000	87.6327	977.1633	0.7653	1.0265
23	2015	50	6.5714	87.5000	779.8153	2.6296	1.8551
27	2015	50	9.7571	86.1224	866.1673	0.9122	1.7735
60	2015	50	6.4714	88.0510	950.8745	0.7612	1.2653
53	2015	50	7.2571	88.7347	861.0867	0.4980	2.0990
66	2015	50	6.4143	89.2347	906.1847	0.8286	1.4255
59	2015	50	6.3714	85.9694	895.4776	0.7561	1.6102
61	2015	50	9.1000	81.7347	974.7786	0.9643	0.8990
84	2015	50	9.1000	81.7347	974.7786	0.9643	0.8990
38	2015	50	6.3714	85.9694	895.4776	0.7561	1.6102

87	2015	50	9.1429	87.1837	908.3296	1.4959	1.5265
34	2015	50	6.3714	85.9694	895.4776	0.7561	1.6102
29	2015	50	7.9571	83.0816	951.5245	0.9888	1.0714
5	2015	50	7.9286	92.7959	838.1990	1.0490	1.6684
8	2015	50	7.2571	88.7347	861.0867	0.4980	2.0990
12	2015	50	7.9286	92.7959	838.1990	1.0490	1.6684
13	2015	50	12.5857	87.9082	954.8602	0.9633	0.9010
18	2015	50	7.7286	88.5000	977.3061	0.7765	0.7500
33	2015	50	7.7714	80.1224	911.2459	0.7398	1.4561
56	2015	50	9.7857	87.1429	990.0337	1.3194	0.6388
77	2015	50	8.8429	89.1122	944.2898	0.8265	0.8408
54	2015	50	7.9286	92.7959	838.1990	1.0490	1.6684
21	2015	50	7.7714	80.1224	911.2459	0.7398	1.4561
68	2015	50	8.1143	83.8265	983.1643	0.9643	1.2112
74	2015	50	9.1000	81.7347	974.7786	0.9643	0.8990
88	2015	50	6.9000	85.8061	882.7827	1.1061	2.2888
16	2015	50	7.1000	84.6224	931.7000	0.7735	1.4041
30	2015	50	6.4143	89.2347	906.1847	0.8286	1.4255
6	2015	50	8.1143	83.8265	983.1643	0.9643	1.2112
49	2015	50	7.9571	83.0816	951.5245	0.9888	1.0714
22	2015	50	6.9000	85.8061	882.7827	1.1061	2.2888
45	2015	50	9.4429	85.1020	822.6184	3.3653	1.5643
58	2015	50	7.9571	83.0816	951.5245	0.9888	1.0714
37	2015	50	8.1143	83.8265	983.1643	0.9643	1.2112
17	2015	50	6.3143	92.2857	911.5561	0.6837	2.1959
55	2015	50	8.4571	89.1224	885.4714	0.5633	1.5684
46	2015	50	7.1000	84.6224	931.7000	0.7735	1.4041
86	2015	50	8.2286	86.1429	873.5827	0.3306	0.8786
2	2015	50	8.2286	86.1429	873.5827	0.3306	0.8786
4	2015	50	7.7714	80.1224	911.2459	0.7398	1.4561
47	2015	50	11.9429	85.3878	970.0388	0.6306	0.3980
82	2015	50	6.9000	85.8061	882.7827	1.1061	2.2888
19	2015	50	10.3571	83.8878	972.0908	1.1357	1.0602
20	2015	50	7.2571	88.7347	861.0867	0.4980	2.0990
80	2015	50	6.9000	85.8061	882.7827	1.1061	2.2888
3	2015	50	12.5857	87.9082	954.8602	0.9633	0.9010
52	2015	50	6.3143	92.2857	911.5561	0.6837	2.1959
70	2015	50	8.9286	80.0102	920.0041	0.6306	1.1398
64	2015	50	6.5714	87.5000	779.8153	2.6296	1.8551
48	2015	50	8.8429	89.1122	944.2898	0.8265	0.8408
65	2015	50	6.3143	92.2857	911.5561	0.6837	2.1959
44	2015	50	8.9286	80.0102	920.0041	0.6306	1.1398
75	2015	50	6.5714	87.5000	779.8153	2.6296	1.8551
40	2015	50	6.8571	91.0000	957.1500	0.7980	1.2867
11	2015	50	8.4571	89.1224	885.4714	0.5633	1.5684
35	2015	50	6.4714	88.0510	950.8745	0.7612	1.2653
78	2015	50	9.1429	87.1837	908.3296	1.4959	1.5265
28	2015	50	6.6286	88.4694	939.8653	0.8449	1.3949
39	2015	50	6.3143	92.2857	911.5561	0.6837	2.1959
24	2015	50	7.9571	83.0816	951.5245	0.9888	1.0714

63	2015	50	6.8571	91.0000	957.1500	0.7980	1.2867
62	2015	50	7.2000	89.8980	882.4878	0.6204	0.8918
1	2015	50	6.9000	85.8061	882.7827	1.1061	2.2888
31	2015	51	1.6143	86.1633	852.4602	0.5235	0.9398
79	2015	51	5.7286	79.9184	983.5847	0.8786	1.3398
51	2015	51	4.1143	86.4490	951.1316	0.6878	1.3520
14	2015	51	4.1857	88.2551	905.9959	0.7714	1.5429
67	2015	51	3.2714	92.2959	911.4888	0.8143	2.5643
42	2015	51	2.9714	86.3367	882.5133	0.7051	2.4776
50	2015	51	3.3714	80.7041	911.2041	0.7969	1.8051
43	2015	51	2.9714	86.3367	882.5133	0.7051	2.4776
85	2015	51	4.1143	80.1735	920.0949	0.5663	1.1449
25	2015	51	8.6857	83.0000	990.3786	1.2194	0.8051
69	2015	51	5.4000	81.9694	951.7633	1.0714	1.2020
57	2015	51	2.3714	86.7041	895.3357	0.4959	1.6163
9	2015	51	2.5429	88.4490	860.6439	0.0980	2.1357
72	2015	51	4.1714	88.8265	885.1898	0.2306	1.6929
26	2015	51	5.0286	89.4796	873.7816	0.9592	1.8133
7	2015	51	4.5714	86.1224	865.7439	0.6255	1.7704
83	2015	51	9.6429	85.1531	954.9378	0.5122	0.9153
76	2015	51	4.3000	84.0714	931.8102	0.9714	1.5857
36	2015	51	4.5143	87.3980	940.0102	0.7418	1.6194
81	2015	51	4.1143	86.4490	951.1316	0.6878	1.3520
15	2015	51	5.2571	87.9184	944.4551	1.2388	0.8418
32	2015	51	2.9714	86.3367	882.5133	0.7051	2.4776
73	2015	51	6.5571	80.2653	975.1929	0.7388	0.9255
71	2015	51	4.5143	87.3980	940.0102	0.7418	1.6194
41	2015	51	2.8714	90.6735	882.2816	0.2480	0.8765
10	2015	51	5.4286	84.9490	977.4878	0.8510	0.9786
23	2015	51	-0.4000	86.7245	778.8153	2.1071	1.9490
27	2015	51	4.5714	86.1224	865.7439	0.6255	1.7704
60	2015	51	4.1143	86.4490	951.1316	0.6878	1.3520
53	2015	51	2.5429	88.4490	860.6439	0.0980	2.1357
66	2015	51	4.1857	88.2551	905.9959	0.7714	1.5429
59	2015	51	2.3714	86.7041	895.3357	0.4959	1.6163
61	2015	51	6.5571	80.2653	975.1929	0.7388	0.9255
84	2015	51	6.5571	80.2653	975.1929	0.7388	0.9255
38	2015	51	2.3714	86.7041	895.3357	0.4959	1.6163
87	2015	51	5.4429	85.8776	908.2112	1.0673	1.8561
34	2015	51	2.3714	86.7041	895.3357	0.4959	1.6163
29	2015	51	5.4000	81.9694	951.7633	1.0714	1.2020
5	2015	51	2.6143	92.5204	837.5531	0.9786	1.6153
8	2015	51	2.5429	88.4490	860.6439	0.0980	2.1357
12	2015	51	2.6143	92.5204	837.5531	0.9786	1.6153
13	2015	51	9.6429	85.1531	954.9378	0.5122	0.9153
18	2015	51	5.3857	85.3163	977.7235	0.7347	0.7622
33	2015	51	3.3714	80.7041	911.2041	0.7969	1.8051
56	2015	51	8.6857	83.0000	990.3786	1.2194	0.8051
77	2015	51	5.2571	87.9184	944.4551	1.2388	0.8418
54	2015	51	2.6143	92.5204	837.5531	0.9786	1.6153

21	2015	51	3.3714	80.7041	911.2041	0.7969	1.8051
68	2015	51	5.7286	79.9184	983.5847	0.8786	1.3398
74	2015	51	6.5571	80.2653	975.1929	0.7388	0.9255
88	2015	51	2.9714	86.3367	882.5133	0.7051	2.4776
16	2015	51	4.3000	84.0714	931.8102	0.9714	1.5857
30	2015	51	4.1857	88.2551	905.9959	0.7714	1.5429
6	2015	51	5.7286	79.9184	983.5847	0.8786	1.3398
49	2015	51	5.4000	81.9694	951.7633	1.0714	1.2020
22	2015	51	2.9714	86.3367	882.5133	0.7051	2.4776
45	2015	51	3.6000	83.5408	821.8827	2.6449	1.6592
58	2015	51	5.4000	81.9694	951.7633	1.0714	1.2020
37	2015	51	5.7286	79.9184	983.5847	0.8786	1.3398
17	2015	51	3.2714	92.2959	911.4888	0.8143	2.5643
55	2015	51	4.1714	88.8265	885.1898	0.2306	1.6929
46	2015	51	4.3000	84.0714	931.8102	0.9714	1.5857
86	2015	51	3.2286	86.0102	873.2847	0.1837	0.8827
2	2015	51	3.2286	86.0102	873.2847	0.1837	0.8827
4	2015	51	3.3714	80.7041	911.2041	0.7969	1.8051
47	2015	51	9.8286	81.4184	970.1571	0.6245	0.3878
82	2015	51	2.9714	86.3367	882.5133	0.7051	2.4776
19	2015	51	9.1143	80.0000	972.2694	0.9367	1.2388
20	2015	51	2.5429	88.4490	860.6439	0.0980	2.1357
80	2015	51	2.9714	86.3367	882.5133	0.7051	2.4776
3	2015	51	9.6429	85.1531	954.9378	0.5122	0.9153
52	2015	51	3.2714	92.2959	911.4888	0.8143	2.5643
70	2015	51	4.1143	80.1735	920.0949	0.5663	1.1449
64	2015	51	-0.4000	86.7245	778.8153	2.1071	1.9490
48	2015	51	5.2571	87.9184	944.4551	1.2388	0.8418
65	2015	51	3.2714	92.2959	911.4888	0.8143	2.5643
44	2015	51	4.1143	80.1735	920.0949	0.5663	1.1449
75	2015	51	-0.4000	86.7245	778.8153	2.1071	1.9490
40	2015	51	4.7286	88.7551	957.3296	1.0765	1.4347
11	2015	51	4.1714	88.8265	885.1898	0.2306	1.6929
35	2015	51	4.1143	86.4490	951.1316	0.6878	1.3520
78	2015	51	5.4429	85.8776	908.2112	1.0673	1.8561
28	2015	51	4.5143	87.3980	940.0102	0.7418	1.6194
39	2015	51	3.2714	92.2959	911.4888	0.8143	2.5643
24	2015	51	5.4000	81.9694	951.7633	1.0714	1.2020
63	2015	51	4.7286	88.7551	957.3296	1.0765	1.4347
62	2015	51	2.8714	90.6735	882.2816	0.2480	0.8765
1	2015	51	2.9714	86.3367	882.5133	0.7051	2.4776
31	2015	52	4.2571	84.1429	852.4847	1.1969	0.9724
79	2015	52	6.9429	78.0102	983.9367	0.8204	1.4173
51	2015	52	5.6571	84.0000	951.2857	0.6286	1.5010
14	2015	52	9.5143	82.5000	906.0888	1.7929	1.9316
67	2015	52	7.9143	89.4388	911.4786	1.5276	2.9939
42	2015	52	7.5571	84.4694	882.4561	1.5867	2.5633
50	2015	52	5.8286	81.5918	911.4296	0.7418	1.7684
43	2015	52	7.5571	84.4694	882.4561	1.5867	2.5633
85	2015	52	6.1857	82.4898	920.4816	0.5735	1.2939

25	2015	52	11.6000	75.7143	990.4092	1.6449	0.9939
69	2015	52	7.3571	80.1939	951.8816	0.7745	1.2469
57	2015	52	5.8286	85.4286	895.3469	0.9255	1.7969
9	2015	52	7.8286	84.7347	860.5551	1.5582	2.4276
72	2015	52	9.6000	83.7449	885.2245	1.7214	1.9306
26	2015	52	10.2714	84.9796	873.9194	2.1214	2.0500
7	2015	52	9.6286	81.3980	865.7755	1.7265	2.0347
83	2015	52	13.5000	78.5816	955.0878	1.5796	1.0071
76	2015	52	6.1714	84.4592	931.9582	0.7153	1.6867
36	2015	52	7.6000	82.9796	940.0510	1.1173	1.6990
81	2015	52	5.6571	84.0000	951.2857	0.6286	1.5010
15	2015	52	6.9143	88.8878	944.7490	0.6867	0.7265
32	2015	52	7.5571	84.4694	882.4561	1.5867	2.5633
73	2015	52	7.9143	80.3367	975.5245	0.1735	0.8990
71	2015	52	7.6000	82.9796	940.0510	1.1173	1.6990
41	2015	52	5.9286	88.7857	882.3194	0.9102	1.0082
10	2015	52	7.1143	82.2041	977.7602	0.9745	0.9367
23	2015	52	4.4143	79.5204	778.5653	3.3418	2.3184
27	2015	52	9.6286	81.3980	865.7755	1.7265	2.0347
60	2015	52	5.6571	84.0000	951.2857	0.6286	1.5010
53	2015	52	7.8286	84.7347	860.5551	1.5582	2.4276
66	2015	52	9.5143	82.5000	906.0888	1.7929	1.9316
59	2015	52	5.8286	85.4286	895.3469	0.9255	1.7969
61	2015	52	7.9143	80.3367	975.5245	0.1735	0.8990
84	2015	52	7.9143	80.3367	975.5245	0.1735	0.8990
38	2015	52	5.8286	85.4286	895.3469	0.9255	1.7969
87	2015	52	10.0143	81.4082	908.2735	1.6908	2.1357
34	2015	52	5.8286	85.4286	895.3469	0.9255	1.7969
29	2015	52	7.3571	80.1939	951.8816	0.7745	1.2469
5	2015	52	8.6000	82.8061	837.4214	2.3184	2.0959
8	2015	52	7.8286	84.7347	860.5551	1.5582	2.4276
12	2015	52	8.6000	82.8061	837.4214	2.3184	2.0959
13	2015	52	13.5000	78.5816	955.0878	1.5796	1.0071
18	2015	52	6.7429	84.7143	978.0020	0.6908	0.7480
33	2015	52	5.8286	81.5918	911.4296	0.7418	1.7684
56	2015	52	11.6000	75.7143	990.4092	1.6449	0.9939
77	2015	52	6.9143	88.8878	944.7490	0.6867	0.7265
54	2015	52	8.6000	82.8061	837.4214	2.3184	2.0959
21	2015	52	5.8286	81.5918	911.4296	0.7418	1.7684
68	2015	52	6.9429	78.0102	983.9367	0.8204	1.4173
74	2015	52	7.9143	80.3367	975.5245	0.1735	0.8990
88	2015	52	7.5571	84.4694	882.4561	1.5867	2.5633
16	2015	52	6.1714	84.4592	931.9582	0.7153	1.6867
30	2015	52	9.5143	82.5000	906.0888	1.7929	1.9316
6	2015	52	6.9429	78.0102	983.9367	0.8204	1.4173
49	2015	52	7.3571	80.1939	951.8816	0.7745	1.2469
22	2015	52	7.5571	84.4694	882.4561	1.5867	2.5633
45	2015	52	8.2714	76.2653	821.8276	3.5020	2.0133
58	2015	52	7.3571	80.1939	951.8816	0.7745	1.2469
37	2015	52	6.9429	78.0102	983.9367	0.8204	1.4173

17	2015	52	7.9143	89.4388	911.4786	1.5276	2.9939
55	2015	52	9.6000	83.7449	885.2245	1.7214	1.9306
46	2015	52	6.1714	84.4592	931.9582	0.7153	1.6867
86	2015	52	6.6143	84.7347	873.2806	0.7418	1.2286
2	2015	52	6.6143	84.7347	873.2806	0.7418	1.2286
4	2015	52	5.8286	81.5918	911.4296	0.7418	1.7684
47	2015	52	13.4143	74.8980	970.1959	1.1592	0.4398
82	2015	52	7.5571	84.4694	882.4561	1.5867	2.5633
19	2015	52	12.9429	74.0306	972.3918	1.0235	1.2469
20	2015	52	7.8286	84.7347	860.5551	1.5582	2.4276
80	2015	52	7.5571	84.4694	882.4561	1.5867	2.5633
3	2015	52	13.5000	78.5816	955.0878	1.5796	1.0071
52	2015	52	7.9143	89.4388	911.4786	1.5276	2.9939
70	2015	52	6.1857	82.4898	920.4816	0.5735	1.2939
64	2015	52	4.4143	79.5204	778.5653	3.3418	2.3184
48	2015	52	6.9143	88.8878	944.7490	0.6867	0.7265
65	2015	52	7.9143	89.4388	911.4786	1.5276	2.9939
44	2015	52	6.1857	82.4898	920.4816	0.5735	1.2939
75	2015	52	4.4143	79.5204	778.5653	3.3418	2.3184
40	2015	52	7.3000	84.7245	957.5020	2.0235	1.4684
11	2015	52	9.6000	83.7449	885.2245	1.7214	1.9306
35	2015	52	5.6571	84.0000	951.2857	0.6286	1.5010
78	2015	52	10.0143	81.4082	908.2735	1.6908	2.1357
28	2015	52	7.6000	82.9796	940.0510	1.1173	1.6990
39	2015	52	7.9143	89.4388	911.4786	1.5276	2.9939
24	2015	52	7.3571	80.1939	951.8816	0.7745	1.2469
63	2015	52	7.3000	84.7245	957.5020	2.0235	1.4684
62	2015	52	5.9286	88.7857	882.3194	0.9102	1.0082
1	2015	52	7.5571	84.4694	882.4561	1.5867	2.5633
31	2015	53	4.8143	83.3571	854.1888	0.8347	0.8051
79	2015	53	9.0143	79.1633	984.9245	1.1847	1.3357
51	2015	53	8.4286	83.3776	952.4184	0.9541	1.3500
14	2015	53	8.0000	79.5510	907.5969	2.4684	1.7408
67	2015	53	7.9857	86.3061	912.8408	2.0102	2.8286
42	2015	53	7.0429	82.5510	883.9837	2.1857	2.4918
50	2015	53	7.6429	80.0204	912.8949	0.8633	1.3980
43	2015	53	7.0429	82.5510	883.9837	2.1857	2.4918
85	2015	53	8.1429	82.7143	921.9684	0.6480	1.1643
25	2015	53	11.4714	75.8367	991.6000	1.8429	0.9878
69	2015	53	9.4000	82.4184	953.1194	0.7276	1.0010
57	2015	53	6.8000	83.9694	896.8469	1.2449	1.7102
9	2015	53	6.3429	82.7755	862.2102	1.9122	2.3173
72	2015	53	8.0000	81.4694	886.8173	2.1929	1.7194
26	2015	53	8.5571	83.5204	875.6735	2.3112	1.9184
7	2015	53	7.8143	80.3776	867.4878	1.9010	1.8867
83	2015	53	12.3143	79.0612	956.7061	2.0449	0.9643
76	2015	53	7.9571	84.4082	933.2663	0.6408	1.4184
36	2015	53	8.9000	81.1429	941.3306	1.4867	1.5133
81	2015	53	8.4286	83.3776	952.4184	0.9541	1.3500
15	2015	53	8.2429	89.8367	946.0633	0.3755	0.5969

32	2015	53	7.0429	82.5510	883.9837	2.1857	2.4918
73	2015	53	9.7143	82.4796	976.7092	0.1327	0.8000
71	2015	53	8.9000	81.1429	941.3306	1.4867	1.5133
41	2015	53	6.6857	86.3878	883.9051	0.8990	0.8306
10	2015	53	9.2286	82.3980	978.8633	1.1347	0.9296
23	2015	53	3.0286	79.1735	780.2837	3.5745	2.2918
27	2015	53	7.8143	80.3776	867.4878	1.9010	1.8867
60	2015	53	8.4286	83.3776	952.4184	0.9541	1.3500
53	2015	53	6.3429	82.7755	862.2102	1.9122	2.3173
66	2015	53	8.0000	79.5510	907.5969	2.4684	1.7408
59	2015	53	6.8000	83.9694	896.8469	1.2449	1.7102
61	2015	53	9.7143	82.4796	976.7092	0.1327	0.8000
84	2015	53	9.7143	82.4796	976.7092	0.1327	0.8000
38	2015	53	6.8000	83.9694	896.8469	1.2449	1.7102
87	2015	53	9.0143	79.4286	909.7755	2.5255	1.9724
34	2015	53	6.8000	83.9694	896.8469	1.2449	1.7102
29	2015	53	9.4000	82.4184	953.1194	0.7276	1.0010
5	2015	53	5.8571	80.7857	839.1735	2.4459	1.9939
8	2015	53	6.3429	82.7755	862.2102	1.9122	2.3173
12	2015	53	5.8571	80.7857	839.1735	2.4459	1.9939
13	2015	53	12.3143	79.0612	956.7061	2.0449	0.9643
18	2015	53	8.6143	85.3367	978.9633	1.0847	0.8133
33	2015	53	7.6429	80.0204	912.8949	0.8633	1.3980
56	2015	53	11.4714	75.8367	991.6000	1.8429	0.9878
77	2015	53	8.2429	89.8367	946.0633	0.3755	0.5969
54	2015	53	5.8571	80.7857	839.1735	2.4459	1.9939
21	2015	53	7.6429	80.0204	912.8949	0.8633	1.3980
68	2015	53	9.0143	79.1633	984.9245	1.1847	1.3357
74	2015	53	9.7143	82.4796	976.7092	0.1327	0.8000
88	2015	53	7.0429	82.5510	883.9837	2.1857	2.4918
16	2015	53	7.9571	84.4082	933.2663	0.6408	1.4184
30	2015	53	8.0000	79.5510	907.5969	2.4684	1.7408
6	2015	53	9.0143	79.1633	984.9245	1.1847	1.3357
49	2015	53	9.4000	82.4184	953.1194	0.7276	1.0010
22	2015	53	7.0429	82.5510	883.9837	2.1857	2.4918
45	2015	53	6.6714	77.6531	823.6296	3.2122	1.8673
58	2015	53	9.4000	82.4184	953.1194	0.7276	1.0010
37	2015	53	9.0143	79.1633	984.9245	1.1847	1.3357
17	2015	53	7.9857	86.3061	912.8408	2.0102	2.8286
55	2015	53	8.0000	81.4694	886.8173	2.1929	1.7194
46	2015	53	7.9571	84.4082	933.2663	0.6408	1.4184
86	2015	53	7.0714	84.1429	874.9337	0.7378	1.2255
2	2015	53	7.0714	84.1429	874.9337	0.7378	1.2255
4	2015	53	7.6429	80.0204	912.8949	0.8633	1.3980
47	2015	53	12.1429	76.1735	971.6490	1.6643	0.4480
82	2015	53	7.0429	82.5510	883.9837	2.1857	2.4918
19	2015	53	11.7429	73.4388	973.7765	1.7531	1.0184
20	2015	53	6.3429	82.7755	862.2102	1.9122	2.3173
80	2015	53	7.0429	82.5510	883.9837	2.1857	2.4918
3	2015	53	12.3143	79.0612	956.7061	2.0449	0.9643

52	2015	53	7.9857	86.3061	912.8408	2.0102	2.8286
70	2015	53	8.1429	82.7143	921.9684	0.6480	1.1643
64	2015	53	3.0286	79.1735	780.2837	3.5745	2.2918
48	2015	53	8.2429	89.8367	946.0633	0.3755	0.5969
65	2015	53	7.9857	86.3061	912.8408	2.0102	2.8286
44	2015	53	8.1429	82.7143	921.9684	0.6480	1.1643
75	2015	53	3.0286	79.1735	780.2837	3.5745	2.2918
40	2015	53	8.9571	83.7347	958.7082	2.1643	1.2388
11	2015	53	8.0000	81.4694	886.8173	2.1929	1.7194
35	2015	53	8.4286	83.3776	952.4184	0.9541	1.3500
78	2015	53	9.0143	79.4286	909.7755	2.5255	1.9724
28	2015	53	8.9000	81.1429	941.3306	1.4867	1.5133
39	2015	53	7.9857	86.3061	912.8408	2.0102	2.8286
24	2015	53	9.4000	82.4184	953.1194	0.7276	1.0010
63	2015	53	8.9571	83.7347	958.7082	2.1643	1.2388
62	2015	53	6.6857	86.3878	883.9051	0.8990	0.8306
1	2015	53	7.0429	82.5510	883.9837	2.1857	2.4918
31	2016	1	5.7429	84.5918	853.9735	0.8673	0.7816
79	2016	1	7.4000	82.3673	983.6592	0.7082	1.4306
51	2016	1	6.8286	86.1327	951.3908	0.9286	1.3041
14	2016	1	8.6571	85.1224	906.9939	1.5745	1.6071
67	2016	1	7.3000	89.0714	912.2265	1.2408	2.4480
42	2016	1	6.9000	86.1633	883.5378	1.7378	2.4020
50	2016	1	7.2000	80.0918	912.2143	1.1602	1.3612
43	2016	1	6.9000	86.1633	883.5378	1.7378	2.4020
85	2016	1	7.9857	82.9898	921.1755	1.2510	1.1724
25	2016	1	11.6571	81.5714	990.4888	1.1622	0.9490
69	2016	1	8.3571	84.3776	952.1480	0.6286	0.8173
57	2016	1	6.3000	87.5000	896.3082	1.2745	1.6633
9	2016	1	7.9286	85.7347	861.8949	2.0480	2.1653
72	2016	1	9.9857	85.2245	886.2480	2.0520	1.5347
26	2016	1	11.8286	84.6939	875.1102	3.0316	1.7969
7	2016	1	10.8571	82.2143	867.0194	2.7541	1.5694
83	2016	1	14.8571	82.3776	955.6163	2.3490	0.8510
76	2016	1	6.7857	84.7551	932.4796	0.5929	1.3153
36	2016	1	7.7286	84.8061	940.4500	1.3276	1.4755
81	2016	1	6.8286	86.1327	951.3908	0.9286	1.3041
15	2016	1	7.7429	88.3571	945.1571	0.3020	0.5357
32	2016	1	6.9000	86.1633	883.5378	1.7378	2.4020
73	2016	1	9.0143	82.7653	975.5541	0.0408	0.7286
71	2016	1	7.7286	84.8061	940.4500	1.3276	1.4755
41	2016	1	7.1286	87.7449	883.4429	1.4918	0.6806
10	2016	1	8.0571	86.0714	977.6204	0.6755	0.9827
23	2016	1	4.6857	81.3776	780.3959	3.7898	1.9255
27	2016	1	10.8571	82.2143	867.0194	2.7541	1.5694
60	2016	1	6.8286	86.1327	951.3908	0.9286	1.3041
53	2016	1	7.9286	85.7347	861.8949	2.0480	2.1653
66	2016	1	8.6571	85.1224	906.9939	1.5745	1.6071
59	2016	1	6.3000	87.5000	896.3082	1.2745	1.6633
61	2016	1	9.0143	82.7653	975.5541	0.0408	0.7286

84	2016	1	9.0143	82.7653	975.5541	0.0408	0.7286
38	2016	1	6.3000	87.5000	896.3082	1.2745	1.6633
87	2016	1	10.3143	82.8571	909.0735	2.4612	1.8622
34	2016	1	6.3000	87.5000	896.3082	1.2745	1.6633
29	2016	1	8.3571	84.3776	952.1480	0.6286	0.8173
5	2016	1	8.9143	84.0306	838.9827	3.1367	1.5316
8	2016	1	7.9286	85.7347	861.8949	2.0480	2.1653
12	2016	1	8.9143	84.0306	838.9827	3.1367	1.5316
13	2016	1	14.8571	82.3776	955.6163	2.3490	0.8510
18	2016	1	7.4857	86.2755	977.7878	0.6449	0.8724
33	2016	1	7.2000	80.0918	912.2143	1.1602	1.3612
56	2016	1	11.6571	81.5714	990.4888	1.1622	0.9490
77	2016	1	7.7429	88.3571	945.1571	0.3020	0.5357
54	2016	1	8.9143	84.0306	838.9827	3.1367	1.5316
21	2016	1	7.2000	80.0918	912.2143	1.1602	1.3612
68	2016	1	7.4000	82.3673	983.6592	0.7082	1.4306
74	2016	1	9.0143	82.7653	975.5541	0.0408	0.7286
88	2016	1	6.9000	86.1633	883.5378	1.7378	2.4020
16	2016	1	6.7857	84.7551	932.4796	0.5929	1.3153
30	2016	1	8.6571	85.1224	906.9939	1.5745	1.6071
6	2016	1	7.4000	82.3673	983.6592	0.7082	1.4306
49	2016	1	8.3571	84.3776	952.1480	0.6286	0.8173
22	2016	1	6.9000	86.1633	883.5378	1.7378	2.4020
45	2016	1	9.7143	77.3776	823.4633	4.1765	1.6796
58	2016	1	8.3571	84.3776	952.1480	0.6286	0.8173
37	2016	1	7.4000	82.3673	983.6592	0.7082	1.4306
17	2016	1	7.3000	89.0714	912.2265	1.2408	2.4480
55	2016	1	9.9857	85.2245	886.2480	2.0520	1.5347
46	2016	1	6.7857	84.7551	932.4796	0.5929	1.3153
86	2016	1	7.5143	84.5510	874.5408	0.8969	1.1214
2	2016	1	7.5143	84.5510	874.5408	0.8969	1.1214
4	2016	1	7.2000	80.0918	912.2143	1.1602	1.3612
47	2016	1	14.4286	80.5816	970.5520	1.9857	0.3929
82	2016	1	6.9000	86.1633	883.5378	1.7378	2.4020
19	2016	1	12.7429	79.2857	972.8061	1.6643	1.0245
20	2016	1	7.9286	85.7347	861.8949	2.0480	2.1653
80	2016	1	6.9000	86.1633	883.5378	1.7378	2.4020
3	2016	1	14.8571	82.3776	955.6163	2.3490	0.8510
52	2016	1	7.3000	89.0714	912.2265	1.2408	2.4480
70	2016	1	7.9857	82.9898	921.1755	1.2510	1.1724
64	2016	1	4.6857	81.3776	780.3959	3.7898	1.9255
48	2016	1	7.7429	88.3571	945.1571	0.3020	0.5357
65	2016	1	7.3000	89.0714	912.2265	1.2408	2.4480
44	2016	1	7.9857	82.9898	921.1755	1.2510	1.1724
75	2016	1	4.6857	81.3776	780.3959	3.7898	1.9255
40	2016	1	7.8286	87.1224	957.6959	1.3041	1.1827
11	2016	1	9.9857	85.2245	886.2480	2.0520	1.5347
35	2016	1	6.8286	86.1327	951.3908	0.9286	1.3041
78	2016	1	10.3143	82.8571	909.0735	2.4612	1.8622
28	2016	1	7.7286	84.8061	940.4500	1.3276	1.4755

39	2016	1	7.3000	89.0714	912.2265	1.2408	2.4480
24	2016	1	8.3571	84.3776	952.1480	0.6286	0.8173
63	2016	1	7.8286	87.1224	957.6959	1.3041	1.1827
62	2016	1	7.1286	87.7449	883.4429	1.4918	0.6806
1	2016	1	6.9000	86.1633	883.5378	1.7378	2.4020
31	2016	2	4.1857	87.7551	850.9663	1.3255	0.7959
79	2016	2	5.1286	84.8163	981.6306	0.1184	1.7867
51	2016	2	3.7857	90.1939	949.1357	0.3286	1.5245
14	2016	2	4.6429	91.1020	904.2582	0.8857	1.6592
67	2016	2	4.1714	94.1633	909.6490	0.5133	2.5082
42	2016	2	3.9000	91.5816	880.7347	1.0429	2.4449
50	2016	2	5.0714	82.1327	909.4327	0.7735	1.6041
43	2016	2	3.9000	91.5816	880.7347	1.0429	2.4449
85	2016	2	5.9714	84.1429	918.2673	1.0010	1.3378
25	2016	2	8.0143	83.2347	988.1633	0.5153	0.9694
69	2016	2	5.5571	84.5000	949.7531	0.2918	1.0643
57	2016	2	3.9571	93.4388	893.5153	0.8092	1.7041
9	2016	2	4.7000	89.4490	858.9378	2.2837	2.3673
72	2016	2	6.3429	89.4082	883.2929	2.3184	1.8051
26	2016	2	8.5429	85.0714	871.9245	4.3184	2.0765
7	2016	2	7.3857	83.2551	863.9276	4.1163	1.7735
83	2016	2	10.9571	84.2347	952.4133	2.6153	0.9878
76	2016	2	4.4286	87.5816	929.9704	0.2959	1.5847
36	2016	2	4.7143	89.6327	938.0000	0.6541	1.6663
81	2016	2	3.7857	90.1939	949.1357	0.3286	1.5245
15	2016	2	6.2286	85.5714	942.4439	0.1806	0.6929
32	2016	2	3.9000	91.5816	880.7347	1.0429	2.4449
73	2016	2	7.0143	81.2449	973.1837	0.0673	0.8041
71	2016	2	4.7143	89.6327	938.0000	0.6541	1.6663
41	2016	2	4.3714	92.8571	880.5316	1.5561	0.8816
10	2016	2	4.9143	89.1224	975.4857	0.1429	1.1194
23	2016	2	3.1286	78.8163	777.5459	5.4122	1.9653
27	2016	2	7.3857	83.2551	863.9276	4.1163	1.7735
60	2016	2	3.7857	90.1939	949.1357	0.3286	1.5245
53	2016	2	4.7000	89.4490	858.9378	2.2837	2.3673
66	2016	2	4.6429	91.1020	904.2582	0.8857	1.6592
59	2016	2	3.9571	93.4388	893.5153	0.8092	1.7041
61	2016	2	7.0143	81.2449	973.1837	0.0673	0.8041
84	2016	2	7.0143	81.2449	973.1837	0.0673	0.8041
38	2016	2	3.9571	93.4388	893.5153	0.8092	1.7041
87	2016	2	6.6714	85.0000	906.1510	1.9184	2.0878
34	2016	2	3.9571	93.4388	893.5153	0.8092	1.7041
29	2016	2	5.5571	84.5000	949.7531	0.2918	1.0643
5	2016	2	5.3000	85.2041	835.9561	4.5235	1.7888
8	2016	2	4.7000	89.4490	858.9378	2.2837	2.3673
12	2016	2	5.3000	85.2041	835.9561	4.5235	1.7888
13	2016	2	10.9571	84.2347	952.4133	2.6153	0.9878
18	2016	2	5.4429	86.9388	975.7480	0.2041	0.9633
33	2016	2	5.0714	82.1327	909.4327	0.7735	1.6041
56	2016	2	8.0143	83.2347	988.1633	0.5153	0.9694

77	2016	2	6.2286	85.5714	942.4439	0.1806	0.6929
54	2016	2	5.3000	85.2041	835.9561	4.5235	1.7888
21	2016	2	5.0714	82.1327	909.4327	0.7735	1.6041
68	2016	2	5.1286	84.8163	981.6306	0.1184	1.7867
74	2016	2	7.0143	81.2449	973.1837	0.0673	0.8041
88	2016	2	3.9000	91.5816	880.7347	1.0429	2.4449
16	2016	2	4.4286	87.5816	929.9704	0.2959	1.5847
30	2016	2	4.6429	91.1020	904.2582	0.8857	1.6592
6	2016	2	5.1286	84.8163	981.6306	0.1184	1.7867
49	2016	2	5.5571	84.5000	949.7531	0.2918	1.0643
22	2016	2	3.9000	91.5816	880.7347	1.0429	2.4449
45	2016	2	6.8000	72.8469	820.4184	6.3020	1.9694
58	2016	2	5.5571	84.5000	949.7531	0.2918	1.0643
37	2016	2	5.1286	84.8163	981.6306	0.1184	1.7867
17	2016	2	4.1714	94.1633	909.6490	0.5133	2.5082
55	2016	2	6.3429	89.4082	883.2929	2.3184	1.8051
46	2016	2	4.4286	87.5816	929.9704	0.2959	1.5847
86	2016	2	5.2286	87.0918	871.5735	1.1459	1.1653
2	2016	2	5.2286	87.0918	871.5735	1.1459	1.1653
4	2016	2	5.0714	82.1327	909.4327	0.7735	1.6041
47	2016	2	10.3571	82.3776	967.6367	1.5531	0.3520
82	2016	2	3.9000	91.5816	880.7347	1.0429	2.4449
19	2016	2	8.7143	82.9082	970.2173	0.8306	1.3000
20	2016	2	4.7000	89.4490	858.9378	2.2837	2.3673
80	2016	2	3.9000	91.5816	880.7347	1.0429	2.4449
3	2016	2	10.9571	84.2347	952.4133	2.6153	0.9878
52	2016	2	4.1714	94.1633	909.6490	0.5133	2.5082
70	2016	2	5.9714	84.1429	918.2673	1.0010	1.3378
64	2016	2	3.1286	78.8163	777.5459	5.4122	1.9653
48	2016	2	6.2286	85.5714	942.4439	0.1806	0.6929
65	2016	2	4.1714	94.1633	909.6490	0.5133	2.5082
44	2016	2	5.9714	84.1429	918.2673	1.0010	1.3378
75	2016	2	3.1286	78.8163	777.5459	5.4122	1.9653
40	2016	2	4.0429	92.1327	955.4480	0.4806	1.5071
11	2016	2	6.3429	89.4082	883.2929	2.3184	1.8051
35	2016	2	3.7857	90.1939	949.1357	0.3286	1.5245
78	2016	2	6.6714	85.0000	906.1510	1.9184	2.0878
28	2016	2	4.7143	89.6327	938.0000	0.6541	1.6663
39	2016	2	4.1714	94.1633	909.6490	0.5133	2.5082
24	2016	2	5.5571	84.5000	949.7531	0.2918	1.0643
63	2016	2	4.0429	92.1327	955.4480	0.4806	1.5071
62	2016	2	4.3714	92.8571	880.5316	1.5561	0.8816
1	2016	2	3.9000	91.5816	880.7347	1.0429	2.4449
31	2016	3	0.6429	88.1735	849.4816	1.0786	0.8153
79	2016	3	2.7286	81.7653	981.6388	0.6153	1.9459
51	2016	3	1.2714	89.6633	948.8194	0.0704	1.6633
14	2016	3	1.9857	90.6122	903.2163	0.4684	1.6888
67	2016	3	0.9143	94.1327	908.7929	0.1347	2.5367
42	2016	3	0.5286	91.8878	879.6020	0.4469	2.6092
50	2016	3	2.0714	81.7857	908.4551	0.7071	1.6684

43	2016	3	0.5286	91.8878	879.6020	0.4469	2.6092
85	2016	3	2.8429	83.1020	917.2969	0.7173	1.3541
25	2016	3	6.0714	81.1224	988.0684	0.1276	1.0367
69	2016	3	3.1429	83.4286	949.3602	0.1337	1.3112
57	2016	3	0.5000	94.2755	892.4959	0.5786	1.5214
9	2016	3	1.4000	90.2857	857.5439	1.2714	2.4449
72	2016	3	3.3714	89.2143	882.0745	1.6051	2.0867
26	2016	3	6.0286	85.6224	870.3878	3.8010	2.1704
7	2016	3	4.8286	84.3776	862.4633	3.9735	2.0520
83	2016	3	8.8857	84.3571	951.6286	1.6786	1.1102
76	2016	3	1.7143	87.5612	929.3031	0.3378	1.6633
36	2016	3	1.9000	88.6020	937.5102	0.0000	1.7837
81	2016	3	1.2714	89.6633	948.8194	0.0704	1.6633
15	2016	3	3.4714	82.0612	941.6837	0.2837	1.0163
32	2016	3	0.5286	91.8878	879.6020	0.4469	2.6092
73	2016	3	4.4714	77.9286	972.8837	0.1939	0.9898
71	2016	3	1.9000	88.6020	937.5102	0.0000	1.7837
41	2016	3	1.1714	94.4490	879.3276	0.8449	1.0480
10	2016	3	2.7286	87.4592	975.4347	0.0878	1.1786
23	2016	3	0.1286	77.7245	775.6316	5.4031	2.2776
27	2016	3	4.8286	84.3776	862.4633	3.9735	2.0520
60	2016	3	1.2714	89.6633	948.8194	0.0704	1.6633
53	2016	3	1.4000	90.2857	857.5439	1.2714	2.4449
66	2016	3	1.9857	90.6122	903.2163	0.4684	1.6888
59	2016	3	0.5000	94.2755	892.4959	0.5786	1.5214
61	2016	3	4.4714	77.9286	972.8837	0.1939	0.9898
84	2016	3	4.4714	77.9286	972.8837	0.1939	0.9898
38	2016	3	0.5000	94.2755	892.4959	0.5786	1.5214
87	2016	3	3.1857	83.4286	905.1694	1.1286	2.4296
34	2016	3	0.5000	94.2755	892.4959	0.5786	1.5214
29	2016	3	3.1429	83.4286	949.3602	0.1337	1.3112
5	2016	3	2.3286	89.0204	834.2857	3.8898	2.2214
8	2016	3	1.4000	90.2857	857.5439	1.2714	2.4449
12	2016	3	2.3286	89.0204	834.2857	3.8898	2.2214
13	2016	3	8.8857	84.3571	951.6286	1.6786	1.1102
18	2016	3	2.7143	84.4082	975.6704	0.6571	1.0765
33	2016	3	2.0714	81.7857	908.4551	0.7071	1.6684
56	2016	3	6.0714	81.1224	988.0684	0.1276	1.0367
77	2016	3	3.4714	82.0612	941.6837	0.2837	1.0163
54	2016	3	2.3286	89.0204	834.2857	3.8898	2.2214
21	2016	3	2.0714	81.7857	908.4551	0.7071	1.6684
68	2016	3	2.7286	81.7653	981.6388	0.6153	1.9459
74	2016	3	4.4714	77.9286	972.8837	0.1939	0.9898
88	2016	3	0.5286	91.8878	879.6020	0.4469	2.6092
16	2016	3	1.7143	87.5612	929.3031	0.3378	1.6633
30	2016	3	1.9857	90.6122	903.2163	0.4684	1.6888
6	2016	3	2.7286	81.7653	981.6388	0.6153	1.9459
49	2016	3	3.1429	83.4286	949.3602	0.1337	1.3112
22	2016	3	0.5286	91.8878	879.6020	0.4469	2.6092
45	2016	3	5.1143	74.2959	818.5653	5.8878	2.1765

58	2016	3	3.1429	83.4286	949.3602	0.1337	1.3112
37	2016	3	2.7286	81.7653	981.6388	0.6153	1.9459
17	2016	3	0.9143	94.1327	908.7929	0.1347	2.5367
55	2016	3	3.3714	89.2143	882.0745	1.6051	2.0867
46	2016	3	1.7143	87.5612	929.3031	0.3378	1.6633
86	2016	3	2.0286	88.1939	870.2673	0.7041	1.0469
2	2016	3	2.0286	88.1939	870.2673	0.7041	1.0469
4	2016	3	2.0714	81.7857	908.4551	0.7071	1.6684
47	2016	3	8.0571	80.3265	967.2041	0.7908	0.4122
82	2016	3	0.5286	91.8878	879.6020	0.4469	2.6092
19	2016	3	6.5286	80.1939	969.7265	0.5480	1.3592
20	2016	3	1.4000	90.2857	857.5439	1.2714	2.4449
80	2016	3	0.5286	91.8878	879.6020	0.4469	2.6092
3	2016	3	8.8857	84.3571	951.6286	1.6786	1.1102
52	2016	3	0.9143	94.1327	908.7929	0.1347	2.5367
70	2016	3	2.8429	83.1020	917.2969	0.7173	1.3541
64	2016	3	0.1286	77.7245	775.6316	5.4031	2.2776
48	2016	3	3.4714	82.0612	941.6837	0.2837	1.0163
65	2016	3	0.9143	94.1327	908.7929	0.1347	2.5367
44	2016	3	2.8429	83.1020	917.2969	0.7173	1.3541
75	2016	3	0.1286	77.7245	775.6316	5.4031	2.2776
40	2016	3	1.8571	92.5204	955.0929	0.1551	1.6878
11	2016	3	3.3714	89.2143	882.0745	1.6051	2.0867
35	2016	3	1.2714	89.6633	948.8194	0.0704	1.6633
78	2016	3	3.1857	83.4286	905.1694	1.1286	2.4296
28	2016	3	1.9000	88.6020	937.5102	0.0000	1.7837
39	2016	3	0.9143	94.1327	908.7929	0.1347	2.5367
24	2016	3	3.1429	83.4286	949.3602	0.1337	1.3112
63	2016	3	1.8571	92.5204	955.0929	0.1551	1.6878
62	2016	3	1.1714	94.4490	879.3276	0.8449	1.0480
1	2016	3	0.5286	91.8878	879.6020	0.4469	2.6092
31	2016	4	0.9429	84.3265	851.6622	0.8633	0.9265
79	2016	4	3.8571	76.5000	984.8816	1.1645	1.7571
51	2016	4	2.6714	83.3878	951.7745	0.2643	1.7378
14	2016	4	4.0000	83.2857	905.9071	0.3102	1.8694
67	2016	4	3.2714	86.6633	911.4296	0.1806	2.7367
42	2016	4	2.7286	85.3163	882.0531	0.5092	2.6867
50	2016	4	2.2857	78.6531	911.2735	0.6388	1.7041
43	2016	4	2.7286	85.3163	882.0531	0.5092	2.6867
85	2016	4	2.9857	81.6327	920.2908	0.5959	1.2949
25	2016	4	7.3143	74.2857	991.3918	0.6847	1.2347
69	2016	4	4.4000	77.2245	952.3337	0.6276	1.4643
57	2016	4	1.9857	89.3061	895.0878	0.6000	1.5490
9	2016	4	2.5429	85.3980	859.8388	0.8684	2.4673
72	2016	4	4.7000	82.5612	884.6510	1.2071	2.2847
26	2016	4	6.2571	80.7959	872.9000	2.6480	2.3133
7	2016	4	5.5000	80.0204	864.8796	2.9684	2.1714
83	2016	4	9.7429	77.1224	955.0786	1.6316	1.1449
76	2016	4	2.7714	81.9286	932.1520	0.4418	1.6265
36	2016	4	3.9286	80.6633	940.3827	0.0000	1.7878

81	2016	4	2.6714	83.3878	951.7745	0.2643	1.7378
15	2016	4	3.4857	78.2245	944.8480	0.4837	1.0602
32	2016	4	2.7286	85.3163	882.0531	0.5092	2.6867
73	2016	4	5.3286	72.7959	976.0776	0.2398	1.0735
71	2016	4	3.9286	80.6633	940.3827	0.0000	1.7878
41	2016	4	2.5857	88.3265	881.7714	0.8622	1.2724
10	2016	4	4.1143	81.0000	978.5857	0.3071	1.1827
23	2016	4	1.4571	76.3878	776.9704	4.3388	2.5173
27	2016	4	5.5000	80.0204	864.8796	2.9684	2.1714
60	2016	4	2.6714	83.3878	951.7745	0.2643	1.7378
53	2016	4	2.5429	85.3980	859.8388	0.8684	2.4673
66	2016	4	4.0000	83.2857	905.9071	0.3102	1.8694
59	2016	4	1.9857	89.3061	895.0878	0.6000	1.5490
61	2016	4	5.3286	72.7959	976.0776	0.2398	1.0735
84	2016	4	5.3286	72.7959	976.0776	0.2398	1.0735
38	2016	4	1.9857	89.3061	895.0878	0.6000	1.5490
87	2016	4	5.2571	76.6531	907.9673	0.9398	2.6949
34	2016	4	1.9857	89.3061	895.0878	0.6000	1.5490
29	2016	4	4.4000	77.2245	952.3337	0.6276	1.4643
5	2016	4	3.7143	86.3878	836.3357	3.0214	2.3082
8	2016	4	2.5429	85.3980	859.8388	0.8684	2.4673
12	2016	4	3.7143	86.3878	836.3357	3.0214	2.3082
13	2016	4	9.7429	77.1224	955.0786	1.6316	1.1449
18	2016	4	3.9429	80.3265	978.8653	0.8214	0.8735
33	2016	4	2.2857	78.6531	911.2735	0.6388	1.7041
56	2016	4	7.3143	74.2857	991.3918	0.6847	1.2347
77	2016	4	3.4857	78.2245	944.8480	0.4837	1.0602
54	2016	4	3.7143	86.3878	836.3357	3.0214	2.3082
21	2016	4	2.2857	78.6531	911.2735	0.6388	1.7041
68	2016	4	3.8571	76.5000	984.8816	1.1645	1.7571
74	2016	4	5.3286	72.7959	976.0776	0.2398	1.0735
88	2016	4	2.7286	85.3163	882.0531	0.5092	2.6867
16	2016	4	2.7714	81.9286	932.1520	0.4418	1.6265
30	2016	4	4.0000	83.2857	905.9071	0.3102	1.8694
6	2016	4	3.8571	76.5000	984.8816	1.1645	1.7571
49	2016	4	4.4000	77.2245	952.3337	0.6276	1.4643
22	2016	4	2.7286	85.3163	882.0531	0.5092	2.6867
45	2016	4	5.1429	72.4898	820.4786	4.5959	2.2408
58	2016	4	4.4000	77.2245	952.3337	0.6276	1.4643
37	2016	4	3.8571	76.5000	984.8816	1.1645	1.7571
17	2016	4	3.2714	86.6633	911.4296	0.1806	2.7367
55	2016	4	4.7000	82.5612	884.6510	1.2071	2.2847
46	2016	4	2.7714	81.9286	932.1520	0.4418	1.6265
86	2016	4	2.8714	83.2143	872.6071	0.4398	1.2745
2	2016	4	2.8714	83.2143	872.6071	0.4398	1.2745
4	2016	4	2.2857	78.6531	911.2735	0.6388	1.7041
47	2016	4	9.5857	71.6939	970.7245	0.8245	0.5551
82	2016	4	2.7286	85.3163	882.0531	0.5092	2.6867
19	2016	4	7.9714	72.8163	973.1061	0.6112	1.2388
20	2016	4	2.5429	85.3980	859.8388	0.8684	2.4673

80	2016	4	2.7286	85.3163	882.0531	0.5092	2.6867
3	2016	4	9.7429	77.1224	955.0786	1.6316	1.1449
52	2016	4	3.2714	86.6633	911.4296	0.1806	2.7367
70	2016	4	2.9857	81.6327	920.2908	0.5959	1.2949
64	2016	4	1.4571	76.3878	776.9704	4.3388	2.5173
48	2016	4	3.4857	78.2245	944.8480	0.4837	1.0602
65	2016	4	3.2714	86.6633	911.4296	0.1806	2.7367
44	2016	4	2.9857	81.6327	920.2908	0.5959	1.2949
75	2016	4	1.4571	76.3878	776.9704	4.3388	2.5173
40	2016	4	3.6571	84.7653	958.0459	0.7286	1.6153
11	2016	4	4.7000	82.5612	884.6510	1.2071	2.2847
35	2016	4	2.6714	83.3878	951.7745	0.2643	1.7378
78	2016	4	5.2571	76.6531	907.9673	0.9398	2.6949
28	2016	4	3.9286	80.6633	940.3827	0.0000	1.7878
39	2016	4	3.2714	86.6633	911.4296	0.1806	2.7367
24	2016	4	4.4000	77.2245	952.3337	0.6276	1.4643
63	2016	4	3.6571	84.7653	958.0459	0.7286	1.6153
62	2016	4	2.5857	88.3265	881.7714	0.8622	1.2724
1	2016	4	2.7286	85.3163	882.0531	0.5092	2.6867
31	2016	5	1.4857	83.0408	853.7000	0.8204	0.9653
79	2016	5	4.5000	76.5918	987.1490	1.4482	1.5939
51	2016	5	2.7429	80.6429	953.9612	0.9704	1.6306
14	2016	5	3.2000	77.8980	907.9520	0.4724	2.0204
67	2016	5	2.9571	81.6735	913.5061	0.2694	2.9112
42	2016	5	1.9429	82.4490	884.1041	0.3857	2.6235
50	2016	5	2.9571	78.0306	913.5449	0.2490	1.6592
43	2016	5	1.9429	82.4490	884.1041	0.3857	2.6235
85	2016	5	3.5714	82.1429	922.6653	0.1469	1.3398
25	2016	5	6.5429	71.1633	993.6418	1.3724	1.2408
69	2016	5	4.4429	75.0816	954.5694	1.1653	1.4806
57	2016	5	1.9286	88.3980	897.2245	0.2990	1.5847
9	2016	5	1.9143	83.9286	861.8245	0.5163	2.4755
72	2016	5	3.4857	79.5918	886.7765	0.7449	2.3663
26	2016	5	4.7000	79.4796	875.0429	1.1571	2.1092
7	2016	5	3.6429	79.2857	866.9714	1.3786	2.1643
83	2016	5	9.1000	71.7245	957.5071	1.4112	1.2459
76	2016	5	3.1429	80.4184	934.3735	0.6439	1.6122
36	2016	5	3.5714	76.5918	942.5684	0.4633	1.8980
81	2016	5	2.7429	80.6429	953.9612	0.9704	1.6306
15	2016	5	4.2286	78.3980	947.2398	0.7102	0.9214
32	2016	5	1.9429	82.4490	884.1041	0.3857	2.6235
73	2016	5	5.4143	73.1429	978.4582	0.5582	1.0520
71	2016	5	3.5714	76.5918	942.5684	0.4633	1.8980
41	2016	5	1.9429	86.5408	883.9061	0.5480	1.2500
10	2016	5	3.4714	79.2959	980.8020	0.9204	1.0449
23	2016	5	-2.0286	81.6633	778.6551	2.7092	2.5857
27	2016	5	3.6429	79.2857	866.9714	1.3786	2.1643
60	2016	5	2.7429	80.6429	953.9612	0.9704	1.6306
53	2016	5	1.9143	83.9286	861.8245	0.5163	2.4755
66	2016	5	3.2000	77.8980	907.9520	0.4724	2.0204

59	2016	5	1.9286	88.3980	897.2245	0.2990	1.5847
61	2016	5	5.4143	73.1429	978.4582	0.5582	1.0520
84	2016	5	5.4143	73.1429	978.4582	0.5582	1.0520
38	2016	5	1.9286	88.3980	897.2245	0.2990	1.5847
87	2016	5	4.2714	72.1531	910.1500	0.5418	2.9643
34	2016	5	1.9286	88.3980	897.2245	0.2990	1.5847
29	2016	5	4.4429	75.0816	954.5694	1.1653	1.4806
5	2016	5	1.6429	86.1633	838.3010	1.8541	2.1071
8	2016	5	1.9143	83.9286	861.8245	0.5163	2.4755
12	2016	5	1.6429	86.1633	838.3010	1.8541	2.1071
13	2016	5	9.1000	71.7245	957.5071	1.4112	1.2459
18	2016	5	4.1571	81.0000	981.0816	1.1449	0.7724
33	2016	5	2.9571	78.0306	913.5449	0.2490	1.6592
56	2016	5	6.5429	71.1633	993.6418	1.3724	1.2408
77	2016	5	4.2286	78.3980	947.2398	0.7102	0.9214
54	2016	5	1.6429	86.1633	838.3010	1.8541	2.1071
21	2016	5	2.9571	78.0306	913.5449	0.2490	1.6592
68	2016	5	4.5000	76.5918	987.1490	1.4482	1.5939
74	2016	5	5.4143	73.1429	978.4582	0.5582	1.0520
88	2016	5	1.9429	82.4490	884.1041	0.3857	2.6235
16	2016	5	3.1429	80.4184	934.3735	0.6439	1.6122
30	2016	5	3.2000	77.8980	907.9520	0.4724	2.0204
6	2016	5	4.5000	76.5918	987.1490	1.4482	1.5939
49	2016	5	4.4429	75.0816	954.5694	1.1653	1.4806
22	2016	5	1.9429	82.4490	884.1041	0.3857	2.6235
45	2016	5	1.6286	77.0204	822.4000	2.7745	1.9429
58	2016	5	4.4429	75.0816	954.5694	1.1653	1.4806
37	2016	5	4.5000	76.5918	987.1490	1.4482	1.5939
17	2016	5	2.9571	81.6735	913.5061	0.2694	2.9112
55	2016	5	3.4857	79.5918	886.7765	0.7449	2.3663
46	2016	5	3.1429	80.4184	934.3735	0.6439	1.6122
86	2016	5	2.4429	82.0204	874.7082	0.2898	1.3112
2	2016	5	2.4429	82.0204	874.7082	0.2898	1.3112
4	2016	5	2.9571	78.0306	913.5449	0.2490	1.6592
47	2016	5	8.6429	67.3980	973.0969	0.7990	0.5929
82	2016	5	1.9429	82.4490	884.1041	0.3857	2.6235
19	2016	5	7.1714	68.7143	975.3684	0.9143	1.2724
20	2016	5	1.9143	83.9286	861.8245	0.5163	2.4755
80	2016	5	1.9429	82.4490	884.1041	0.3857	2.6235
3	2016	5	9.1000	71.7245	957.5071	1.4112	1.2459
52	2016	5	2.9571	81.6735	913.5061	0.2694	2.9112
70	2016	5	3.5714	82.1429	922.6653	0.1469	1.3398
64	2016	5	-2.0286	81.6633	778.6551	2.7092	2.5857
48	2016	5	4.2286	78.3980	947.2398	0.7102	0.9214
65	2016	5	2.9571	81.6735	913.5061	0.2694	2.9112
44	2016	5	3.5714	82.1429	922.6653	0.1469	1.3398
75	2016	5	-2.0286	81.6633	778.6551	2.7092	2.5857
40	2016	5	2.8714	81.6735	960.1714	1.4357	1.6878
11	2016	5	3.4857	79.5918	886.7765	0.7449	2.3663
35	2016	5	2.7429	80.6429	953.9612	0.9704	1.6306

78	2016	5	4.2714	72.1531	910.1500	0.5418	2.9643
28	2016	5	3.5714	76.5918	942.5684	0.4633	1.8980
39	2016	5	2.9571	81.6735	913.5061	0.2694	2.9112
24	2016	5	4.4429	75.0816	954.5694	1.1653	1.4806
63	2016	5	2.8714	81.6735	960.1714	1.4357	1.6878
62	2016	5	1.9429	86.5408	883.9061	0.5480	1.2500
1	2016	5	1.9429	82.4490	884.1041	0.3857	2.6235
31	2016	6	6.8714	77.4592	851.8449	2.7582	1.0245
79	2016	6	11.7857	73.2449	982.6378	2.9929	1.3663
51	2016	6	9.7714	76.0714	950.2684	3.6561	1.3071
14	2016	6	11.7000	68.9082	905.6306	2.9796	2.4449
67	2016	6	11.5571	71.7143	910.8653	1.9571	3.2439
42	2016	6	10.9143	73.4490	881.9367	3.0541	2.7449
50	2016	6	9.7857	73.1122	910.6592	2.2755	1.4418
43	2016	6	10.9143	73.4490	881.9367	3.0541	2.7449
85	2016	6	10.1000	76.0000	919.5673	1.9786	1.4296
25	2016	6	13.2714	70.6633	989.8582	3.8694	1.1051
69	2016	6	11.2857	73.8367	950.9531	2.9337	1.3827
57	2016	6	11.8286	76.8469	894.5714	2.6949	2.2408
9	2016	6	10.5286	73.8367	860.0112	3.2918	2.4908
72	2016	6	12.1571	72.0612	884.7724	3.1316	2.1888
26	2016	6	14.3857	70.0612	873.4735	2.8837	2.0694
7	2016	6	12.6143	71.1837	865.3429	3.4745	2.2296
83	2016	6	14.4286	68.2959	954.7449	3.5898	1.2265
76	2016	6	10.5857	76.4286	931.0408	3.0643	1.4622
36	2016	6	12.1714	70.1224	939.2745	2.7837	1.8378
81	2016	6	9.7714	76.0714	950.2684	3.6561	1.3071
15	2016	6	10.6143	76.1122	943.7061	1.9827	0.8082
32	2016	6	10.9143	73.4490	881.9367	3.0541	2.7449
73	2016	6	11.9714	74.0510	974.4286	2.2582	0.9510
71	2016	6	12.1714	70.1224	939.2745	2.7837	1.8378
41	2016	6	9.8286	80.3163	881.5582	2.5408	1.1449
10	2016	6	11.8286	74.8163	976.6531	3.6388	0.9622
23	2016	6	6.9000	75.5204	778.0949	2.9602	2.5357
27	2016	6	12.6143	71.1837	865.3429	3.4745	2.2296
60	2016	6	9.7714	76.0714	950.2684	3.6561	1.3071
53	2016	6	10.5286	73.8367	860.0112	3.2918	2.4908
66	2016	6	11.7000	68.9082	905.6306	2.9796	2.4449
59	2016	6	11.8286	76.8469	894.5714	2.6949	2.2408
61	2016	6	11.9714	74.0510	974.4286	2.2582	0.9510
84	2016	6	11.9714	74.0510	974.4286	2.2582	0.9510
38	2016	6	11.8286	76.8469	894.5714	2.6949	2.2408
87	2016	6	11.9857	67.1633	907.7173	2.9112	2.6122
34	2016	6	11.8286	76.8469	894.5714	2.6949	2.2408
29	2016	6	11.2857	73.8367	950.9531	2.9337	1.3827
5	2016	6	11.8429	74.6633	837.0041	3.5133	2.2592
8	2016	6	10.5286	73.8367	860.0112	3.2918	2.4908
12	2016	6	11.8429	74.6633	837.0041	3.5133	2.2592
13	2016	6	14.4286	68.2959	954.7449	3.5898	1.2265
18	2016	6	10.5571	78.7551	976.7357	2.5551	0.8357

33	2016	6	9.7857	73.1122	910.6592	2.2755	1.4418
56	2016	6	13.2714	70.6633	989.8582	3.8694	1.1051
77	2016	6	10.6143	76.1122	943.7061	1.9827	0.8082
54	2016	6	11.8429	74.6633	837.0041	3.5133	2.2592
21	2016	6	9.7857	73.1122	910.6592	2.2755	1.4418
68	2016	6	11.7857	73.2449	982.6378	2.9929	1.3663
74	2016	6	11.9714	74.0510	974.4286	2.2582	0.9510
88	2016	6	10.9143	73.4490	881.9367	3.0541	2.7449
16	2016	6	10.5857	76.4286	931.0408	3.0643	1.4622
30	2016	6	11.7000	68.9082	905.6306	2.9796	2.4449
6	2016	6	11.7857	73.2449	982.6378	2.9929	1.3663
49	2016	6	11.2857	73.8367	950.9531	2.9337	1.3827
22	2016	6	10.9143	73.4490	881.9367	3.0541	2.7449
45	2016	6	11.9429	70.7755	821.4500	3.7071	2.0714
58	2016	6	11.2857	73.8367	950.9531	2.9337	1.3827
37	2016	6	11.7857	73.2449	982.6378	2.9929	1.3663
17	2016	6	11.5571	71.7143	910.8653	1.9571	3.2439
55	2016	6	12.1571	72.0612	884.7724	3.1316	2.1888
46	2016	6	10.5857	76.4286	931.0408	3.0643	1.4622
86	2016	6	10.6143	73.9082	872.6000	2.2980	1.4173
2	2016	6	10.6143	73.9082	872.6000	2.2980	1.4173
4	2016	6	9.7857	73.1122	910.6592	2.2755	1.4418
47	2016	6	14.5857	66.8878	969.9520	3.1714	0.5704
82	2016	6	10.9143	73.4490	881.9367	3.0541	2.7449
19	2016	6	14.1857	67.8980	972.0969	3.6398	1.2531
20	2016	6	10.5286	73.8367	860.0112	3.2918	2.4908
80	2016	6	10.9143	73.4490	881.9367	3.0541	2.7449
3	2016	6	14.4286	68.2959	954.7449	3.5898	1.2265
52	2016	6	11.5571	71.7143	910.8653	1.9571	3.2439
70	2016	6	10.1000	76.0000	919.5673	1.9786	1.4296
64	2016	6	6.9000	75.5204	778.0949	2.9602	2.5357
48	2016	6	10.6143	76.1122	943.7061	1.9827	0.8082
65	2016	6	11.5571	71.7143	910.8653	1.9571	3.2439
44	2016	6	10.1000	76.0000	919.5673	1.9786	1.4296
75	2016	6	6.9000	75.5204	778.0949	2.9602	2.5357
40	2016	6	12.6714	73.9490	956.7194	4.1531	1.8857
11	2016	6	12.1571	72.0612	884.7724	3.1316	2.1888
35	2016	6	9.7714	76.0714	950.2684	3.6561	1.3071
78	2016	6	11.9857	67.1633	907.7173	2.9112	2.6122
28	2016	6	12.1714	70.1224	939.2745	2.7837	1.8378
39	2016	6	11.5571	71.7143	910.8653	1.9571	3.2439
24	2016	6	11.2857	73.8367	950.9531	2.9337	1.3827
63	2016	6	12.6714	73.9490	956.7194	4.1531	1.8857
62	2016	6	9.8286	80.3163	881.5582	2.5408	1.1449
1	2016	6	10.9143	73.4490	881.9367	3.0541	2.7449
31	2016	7	3.7857	71.2551	850.7837	3.1980	1.1439
79	2016	7	9.3000	65.2245	979.8337	4.0051	1.6337
51	2016	7	7.6143	68.4184	947.8582	4.9745	1.3684
14	2016	7	7.5714	61.5102	903.9816	3.5735	2.5571
67	2016	7	7.5143	63.1735	909.0286	2.3582	3.5010

42	2016	7	5.5286	66.0408	880.4959	4.0898	3.1908
50	2016	7	5.6000	69.5918	908.9500	3.0408	1.5255
43	2016	7	5.5286	66.0408	880.4959	4.0898	3.1908
85	2016	7	6.9571	68.8673	917.8469	2.5520	1.4000
25	2016	7	10.6143	66.4286	987.2143	4.1367	1.1276
69	2016	7	8.8286	67.9592	948.5112	4.0224	1.4990
57	2016	7	5.4857	68.5510	892.9276	3.6980	2.6061
9	2016	7	4.3571	65.3265	858.8041	4.4990	2.7418
72	2016	7	6.6286	63.3469	883.3204	4.2408	2.1765
26	2016	7	5.2857	62.2959	872.3306	3.9888	2.4235
7	2016	7	4.7286	64.0408	864.1939	4.4408	2.3694
83	2016	7	11.1714	64.5918	952.8020	4.4612	1.2653
76	2016	7	6.8714	70.4592	928.8653	3.9694	1.4449
36	2016	7	8.6143	61.6224	937.0214	3.7663	1.7888
81	2016	7	7.6143	68.4184	947.8582	4.9745	1.3684
15	2016	7	7.3000	73.5306	941.4898	2.0908	0.8184
32	2016	7	5.5286	66.0408	880.4959	4.0898	3.1908
73	2016	7	10.4143	66.8776	971.6888	2.9694	0.8459
71	2016	7	8.6143	61.6224	937.0214	3.7663	1.7888
41	2016	7	5.6857	70.0102	880.0908	3.2765	1.2969
10	2016	7	8.3143	66.5204	973.9653	5.0888	1.1806
23	2016	7	-0.6857	72.2653	777.4653	3.2918	2.5633
27	2016	7	4.7286	64.0408	864.1939	4.4408	2.3694
60	2016	7	7.6143	68.4184	947.8582	4.9745	1.3684
53	2016	7	4.3571	65.3265	858.8041	4.4990	2.7418
66	2016	7	7.5714	61.5102	903.9816	3.5735	2.5571
59	2016	7	5.4857	68.5510	892.9276	3.6980	2.6061
61	2016	7	10.4143	66.8776	971.6888	2.9694	0.8459
84	2016	7	10.4143	66.8776	971.6888	2.9694	0.8459
38	2016	7	5.4857	68.5510	892.9276	3.6980	2.6061
87	2016	7	7.9429	62.2143	906.0204	3.9602	2.4704
34	2016	7	5.4857	68.5510	892.9276	3.6980	2.6061
29	2016	7	8.8286	67.9592	948.5112	4.0224	1.4990
5	2016	7	2.4857	66.3980	836.0541	4.1582	2.6459
8	2016	7	4.3571	65.3265	858.8041	4.4990	2.7418
12	2016	7	2.4857	66.3980	836.0541	4.1582	2.6459
13	2016	7	11.1714	64.5918	952.8020	4.4612	1.2653
18	2016	7	8.4571	71.9490	974.1541	3.2684	1.0510
33	2016	7	5.6000	69.5918	908.9500	3.0408	1.5255
56	2016	7	10.6143	66.4286	987.2143	4.1367	1.1276
77	2016	7	7.3000	73.5306	941.4898	2.0908	0.8184
54	2016	7	2.4857	66.3980	836.0541	4.1582	2.6459
21	2016	7	5.6000	69.5918	908.9500	3.0408	1.5255
68	2016	7	9.3000	65.2245	979.8337	4.0051	1.6337
74	2016	7	10.4143	66.8776	971.6888	2.9694	0.8459
88	2016	7	5.5286	66.0408	880.4959	4.0898	3.1908
16	2016	7	6.8714	70.4592	928.8653	3.9694	1.4449
30	2016	7	7.5714	61.5102	903.9816	3.5735	2.5571
6	2016	7	9.3000	65.2245	979.8337	4.0051	1.6337
49	2016	7	8.8286	67.9592	948.5112	4.0224	1.4990

22	2016	7	5.5286	66.0408	880.4959	4.0898	3.1908
45	2016	7	2.2857	66.3163	820.6908	4.0643	2.3459
58	2016	7	8.8286	67.9592	948.5112	4.0224	1.4990
37	2016	7	9.3000	65.2245	979.8337	4.0051	1.6337
17	2016	7	7.5143	63.1735	909.0286	2.3582	3.5010
55	2016	7	6.6286	63.3469	883.3204	4.2408	2.1765
46	2016	7	6.8714	70.4592	928.8653	3.9694	1.4449
86	2016	7	5.1714	65.0612	871.2959	3.3204	1.5827
2	2016	7	5.1714	65.0612	871.2959	3.3204	1.5827
4	2016	7	5.6000	69.5918	908.9500	3.0408	1.5255
47	2016	7	11.6857	63.1327	967.6602	4.0255	0.5939
82	2016	7	5.5286	66.0408	880.4959	4.0898	3.1908
19	2016	7	11.0714	63.4286	969.7786	4.0112	1.3286
20	2016	7	4.3571	65.3265	858.8041	4.4990	2.7418
80	2016	7	5.5286	66.0408	880.4959	4.0898	3.1908
3	2016	7	11.1714	64.5918	952.8020	4.4612	1.2653
52	2016	7	7.5143	63.1735	909.0286	2.3582	3.5010
70	2016	7	6.9571	68.8673	917.8469	2.5520	1.4000
64	2016	7	-0.6857	72.2653	777.4653	3.2918	2.5633
48	2016	7	7.3000	73.5306	941.4898	2.0908	0.8184
65	2016	7	7.5143	63.1735	909.0286	2.3582	3.5010
44	2016	7	6.9571	68.8673	917.8469	2.5520	1.4000
75	2016	7	-0.6857	72.2653	777.4653	3.2918	2.5633
40	2016	7	8.1429	65.4592	954.3673	5.4173	2.0418
11	2016	7	6.6286	63.3469	883.3204	4.2408	2.1765
35	2016	7	7.6143	68.4184	947.8582	4.9745	1.3684
78	2016	7	7.9429	62.2143	906.0204	3.9602	2.4704
28	2016	7	8.6143	61.6224	937.0214	3.7663	1.7888
39	2016	7	7.5143	63.1735	909.0286	2.3582	3.5010
24	2016	7	8.8286	67.9592	948.5112	4.0224	1.4990
63	2016	7	8.1429	65.4592	954.3673	5.4173	2.0418
62	2016	7	5.6857	70.0102	880.0908	3.2765	1.2969
1	2016	7	5.5286	66.0408	880.4959	4.0898	3.1908
31	2016	8	5.3857	74.2347	853.2214	1.7163	1.0959
79	2016	8	9.1429	66.2041	982.9827	3.0745	1.7408
51	2016	8	7.0000	68.7449	950.6980	3.0949	1.5643
14	2016	8	6.7286	64.6224	906.2194	1.3255	2.2000
67	2016	8	6.5286	67.5510	911.5184	0.8531	3.1888
42	2016	8	5.9000	71.2959	882.9469	1.7031	3.1071
50	2016	8	6.9000	72.7959	911.7500	1.6673	1.4418
43	2016	8	5.9000	71.2959	882.9469	1.7031	3.1071
85	2016	8	8.0857	71.0306	920.6776	1.3888	1.2520
25	2016	8	9.6857	68.5102	989.9020	1.5357	0.8633
69	2016	8	8.0429	66.9694	951.4010	2.3633	1.4969
57	2016	8	6.2143	76.9898	895.6418	1.6531	2.1265
9	2016	8	5.4857	73.8265	861.1510	1.7847	2.7235
72	2016	8	6.7857	68.1327	885.6449	1.7296	2.1071
26	2016	8	7.6429	74.3571	874.5224	1.9888	2.0265
7	2016	8	6.4571	75.2551	866.4480	1.8112	1.8888
83	2016	8	11.4857	66.0612	955.3418	1.8796	1.2224

76	2016	8	7.2571	72.6939	931.7816	2.0204	1.4286
36	2016	8	7.1286	63.7653	939.7531	1.9255	1.7888
81	2016	8	7.0000	68.7449	950.6980	3.0949	1.5643
15	2016	8	8.2429	76.3469	944.4214	1.2714	0.8082
32	2016	8	5.9000	71.2959	882.9469	1.7031	3.1071
73	2016	8	9.4429	63.7857	974.7194	2.0306	0.9173
71	2016	8	7.1286	63.7653	939.7531	1.9255	1.7888
41	2016	8	6.2286	74.1327	882.7327	1.5724	1.3561
10	2016	8	8.2571	68.7551	976.9214	3.1776	1.1276
23	2016	8	2.3857	81.5816	779.2969	2.3306	2.4082
27	2016	8	6.4571	75.2551	866.4480	1.8112	1.8888
60	2016	8	7.0000	68.7449	950.6980	3.0949	1.5643
53	2016	8	5.4857	73.8265	861.1510	1.7847	2.7235
66	2016	8	6.7286	64.6224	906.2194	1.3255	2.2000
59	2016	8	6.2143	76.9898	895.6418	1.6531	2.1265
61	2016	8	9.4429	63.7857	974.7194	2.0306	0.9173
84	2016	8	9.4429	63.7857	974.7194	2.0306	0.9173
38	2016	8	6.2143	76.9898	895.6418	1.6531	2.1265
87	2016	8	7.3143	65.0714	908.4592	1.8633	2.6969
34	2016	8	6.2143	76.9898	895.6418	1.6531	2.1265
29	2016	8	8.0429	66.9694	951.4010	2.3633	1.4969
5	2016	8	5.0143	79.7755	838.2082	1.8122	2.1561
8	2016	8	5.4857	73.8265	861.1510	1.7847	2.7235
12	2016	8	5.0143	79.7755	838.2082	1.8122	2.1561
13	2016	8	11.4857	66.0612	955.3418	1.8796	1.2224
18	2016	8	8.8714	71.7959	977.2735	2.8898	1.1000
33	2016	8	6.9000	72.7959	911.7500	1.6673	1.4418
56	2016	8	9.6857	68.5102	989.9020	1.5357	0.8633
77	2016	8	8.2429	76.3469	944.4214	1.2714	0.8082
54	2016	8	5.0143	79.7755	838.2082	1.8122	2.1561
21	2016	8	6.9000	72.7959	911.7500	1.6673	1.4418
68	2016	8	9.1429	66.2041	982.9827	3.0745	1.7408
74	2016	8	9.4429	63.7857	974.7194	2.0306	0.9173
88	2016	8	5.9000	71.2959	882.9469	1.7031	3.1071
16	2016	8	7.2571	72.6939	931.7816	2.0204	1.4286
30	2016	8	6.7286	64.6224	906.2194	1.3255	2.2000
6	2016	8	9.1429	66.2041	982.9827	3.0745	1.7408
49	2016	8	8.0429	66.9694	951.4010	2.3633	1.4969
22	2016	8	5.9000	71.2959	882.9469	1.7031	3.1071
45	2016	8	4.6143	80.6224	822.6133	1.7990	1.8092
58	2016	8	8.0429	66.9694	951.4010	2.3633	1.4969
37	2016	8	9.1429	66.2041	982.9827	3.0745	1.7408
17	2016	8	6.5286	67.5510	911.5184	0.8531	3.1888
55	2016	8	6.7857	68.1327	885.6449	1.7296	2.1071
46	2016	8	7.2571	72.6939	931.7816	2.0204	1.4286
86	2016	8	6.3857	71.6735	873.8969	1.7000	1.4704
2	2016	8	6.3857	71.6735	873.8969	1.7000	1.4704
4	2016	8	6.9000	72.7959	911.7500	1.6673	1.4418
47	2016	8	11.2714	62.8469	970.2143	1.5969	0.5806
82	2016	8	5.9000	71.2959	882.9469	1.7031	3.1071

19	2016	8	10.5000	61.4796	972.1908	1.4980	1.3602
20	2016	8	5.4857	73.8265	861.1510	1.7847	2.7235
80	2016	8	5.9000	71.2959	882.9469	1.7031	3.1071
3	2016	8	11.4857	66.0612	955.3418	1.8796	1.2224
52	2016	8	6.5286	67.5510	911.5184	0.8531	3.1888
70	2016	8	8.0857	71.0306	920.6776	1.3888	1.2520
64	2016	8	2.3857	81.5816	779.2969	2.3306	2.4082
48	2016	8	8.2429	76.3469	944.4214	1.2714	0.8082
65	2016	8	6.5286	67.5510	911.5184	0.8531	3.1888
44	2016	8	8.0857	71.0306	920.6776	1.3888	1.2520
75	2016	8	2.3857	81.5816	779.2969	2.3306	2.4082
40	2016	8	7.1714	71.0102	956.9204	2.8918	1.7061
11	2016	8	6.7857	68.1327	885.6449	1.7296	2.1071
35	2016	8	7.0000	68.7449	950.6980	3.0949	1.5643
78	2016	8	7.3143	65.0714	908.4592	1.8633	2.6969
28	2016	8	7.1286	63.7653	939.7531	1.9255	1.7888
39	2016	8	6.5286	67.5510	911.5184	0.8531	3.1888
24	2016	8	8.0429	66.9694	951.4010	2.3633	1.4969
63	2016	8	7.1714	71.0102	956.9204	2.8918	1.7061
62	2016	8	6.2286	74.1327	882.7327	1.5724	1.3561
1	2016	8	5.9000	71.2959	882.9469	1.7031	3.1071
31	2016	9	12.5714	73.1939	854.9429	3.8490	0.9796
79	2016	9	15.1000	76.5612	983.8898	4.1847	1.1969
51	2016	9	13.7429	72.5306	952.0582	4.0561	1.2653
14	2016	9	13.8143	66.2449	908.2653	3.0071	1.9000
67	2016	9	14.0429	68.9184	913.3592	2.8439	2.8622
42	2016	9	14.2286	68.2857	884.8755	3.5888	2.4306
50	2016	9	14.5571	70.6531	913.0622	3.9388	1.3163
43	2016	9	14.2286	68.2857	884.8755	3.5888	2.4306
85	2016	9	16.5571	69.0408	921.7041	3.9133	1.2582
25	2016	9	15.5714	75.1327	991.5765	2.8092	0.6071
69	2016	9	15.2429	70.3673	952.7990	3.3316	1.1602
57	2016	9	14.9571	73.2449	897.2204	3.6398	2.3612
9	2016	9	13.6143	72.3469	863.2602	3.6153	2.3449
72	2016	9	14.3429	69.9796	887.7653	3.3735	1.8122
26	2016	9	14.8000	74.5408	876.7276	3.9745	1.7653
7	2016	9	13.7143	76.3163	868.6388	3.8592	1.4684
83	2016	9	17.7429	67.7347	957.3429	3.9153	1.0878
76	2016	9	14.6143	73.3673	933.1224	3.7418	1.3235
36	2016	9	15.1429	68.2449	941.3337	3.4122	1.6133
81	2016	9	13.7429	72.5306	952.0582	4.0561	1.2653
15	2016	9	14.7429	75.2959	945.5367	3.4827	0.6959
32	2016	9	14.2286	68.2857	884.8755	3.5888	2.4306
73	2016	9	15.9857	68.7143	975.8000	3.8082	0.9163
71	2016	9	15.1429	68.2449	941.3337	3.4122	1.6133
41	2016	9	13.3571	76.2551	884.4286	3.9857	1.1949
10	2016	9	14.8143	73.3571	978.1265	4.0592	0.8867
23	2016	9	9.7143	75.6122	781.8663	4.2745	1.9235
27	2016	9	13.7143	76.3163	868.6388	3.8592	1.4684
60	2016	9	13.7429	72.5306	952.0582	4.0561	1.2653

53	2016	9	13.6143	72.3469	863.2602	3.6153	2.3449
66	2016	9	13.8143	66.2449	908.2653	3.0071	1.9000
59	2016	9	14.9571	73.2449	897.2204	3.6398	2.3612
61	2016	9	15.9857	68.7143	975.8000	3.8082	0.9163
84	2016	9	15.9857	68.7143	975.8000	3.8082	0.9163
38	2016	9	14.9571	73.2449	897.2204	3.6398	2.3612
87	2016	9	14.8714	65.6837	910.3857	3.6622	2.2163
34	2016	9	14.9571	73.2449	897.2204	3.6398	2.3612
29	2016	9	15.2429	70.3673	952.7990	3.3316	1.1602
5	2016	9	12.9714	78.0408	840.5000	3.8704	1.6357
8	2016	9	13.6143	72.3469	863.2602	3.6153	2.3449
12	2016	9	12.9714	78.0408	840.5000	3.8704	1.6357
13	2016	9	17.7429	67.7347	957.3429	3.9153	1.0878
18	2016	9	14.4143	74.8673	978.0908	4.2163	0.9102
33	2016	9	14.5571	70.6531	913.0622	3.9388	1.3163
56	2016	9	15.5714	75.1327	991.5765	2.8092	0.6071
77	2016	9	14.7429	75.2959	945.5367	3.4827	0.6959
54	2016	9	12.9714	78.0408	840.5000	3.8704	1.6357
21	2016	9	14.5571	70.6531	913.0622	3.9388	1.3163
68	2016	9	15.1000	76.5612	983.8898	4.1847	1.1969
74	2016	9	15.9857	68.7143	975.8000	3.8082	0.9163
88	2016	9	14.2286	68.2857	884.8755	3.5888	2.4306
16	2016	9	14.6143	73.3673	933.1224	3.7418	1.3235
30	2016	9	13.8143	66.2449	908.2653	3.0071	1.9000
6	2016	9	15.1000	76.5612	983.8898	4.1847	1.1969
49	2016	9	15.2429	70.3673	952.7990	3.3316	1.1602
22	2016	9	14.2286	68.2857	884.8755	3.5888	2.4306
45	2016	9	12.0429	79.8673	825.0337	3.5510	1.3867
58	2016	9	15.2429	70.3673	952.7990	3.3316	1.1602
37	2016	9	15.1000	76.5612	983.8898	4.1847	1.1969
17	2016	9	14.0429	68.9184	913.3592	2.8439	2.8622
55	2016	9	14.3429	69.9796	887.7653	3.3735	1.8122
46	2016	9	14.6143	73.3673	933.1224	3.7418	1.3235
86	2016	9	14.0714	70.6020	875.7316	3.8888	1.3633
2	2016	9	14.0714	70.6020	875.7316	3.8888	1.3633
4	2016	9	14.5571	70.6531	913.0622	3.9388	1.3163
47	2016	9	17.2857	66.7551	972.1480	3.1490	0.4735
82	2016	9	14.2286	68.2857	884.8755	3.5888	2.4306
19	2016	9	16.3571	66.5510	974.1235	3.2388	1.1347
20	2016	9	13.6143	72.3469	863.2602	3.6153	2.3449
80	2016	9	14.2286	68.2857	884.8755	3.5888	2.4306
3	2016	9	17.7429	67.7347	957.3429	3.9153	1.0878
52	2016	9	14.0429	68.9184	913.3592	2.8439	2.8622
70	2016	9	16.5571	69.0408	921.7041	3.9133	1.2582
64	2016	9	9.7143	75.6122	781.8663	4.2745	1.9235
48	2016	9	14.7429	75.2959	945.5367	3.4827	0.6959
65	2016	9	14.0429	68.9184	913.3592	2.8439	2.8622
44	2016	9	16.5571	69.0408	921.7041	3.9133	1.2582
75	2016	9	9.7143	75.6122	781.8663	4.2745	1.9235
40	2016	9	14.8571	74.2449	958.4469	3.6510	1.2551

11	2016	9	14.3429	69.9796	887.7653	3.3735	1.8122
35	2016	9	13.7429	72.5306	952.0582	4.0561	1.2653
78	2016	9	14.8714	65.6837	910.3857	3.6622	2.2163
28	2016	9	15.1429	68.2449	941.3337	3.4122	1.6133
39	2016	9	14.0429	68.9184	913.3592	2.8439	2.8622
24	2016	9	15.2429	70.3673	952.7990	3.3316	1.1602
63	2016	9	14.8571	74.2449	958.4469	3.6510	1.2551
62	2016	9	13.3571	76.2551	884.4286	3.9857	1.1949
1	2016	9	14.2286	68.2857	884.8755	3.5888	2.4306
31	2016	10	9.4143	74.3061	851.0214	5.3112	1.0459
79	2016	10	9.2429	84.8571	979.0204	3.4490	1.3184
51	2016	10	7.8143	79.5714	947.4806	3.5510	1.3337
14	2016	10	8.7429	74.9286	904.2888	3.3582	2.2163
67	2016	10	8.4000	76.6327	909.1592	3.3449	3.1398
42	2016	10	8.8714	70.3571	880.7867	4.7755	2.3592
50	2016	10	9.3429	72.1837	908.3378	4.5357	1.8112
43	2016	10	8.8714	70.3571	880.7867	4.7755	2.3592
85	2016	10	10.8714	68.3061	916.7010	5.3092	1.6786
25	2016	10	12.4429	78.2959	986.6398	3.1296	1.0480
69	2016	10	10.3571	74.2143	947.9276	3.5010	1.4163
57	2016	10	8.9429	72.6633	892.7704	4.9959	2.7602
9	2016	10	9.5857	71.6939	859.4194	5.5816	2.4122
72	2016	10	11.4429	71.9490	883.6204	5.1173	1.7398
26	2016	10	13.6143	66.7755	872.6367	6.7153	2.2367
7	2016	10	12.7714	68.3673	864.6265	6.7898	1.8000
83	2016	10	16.5714	67.2857	952.0357	6.3878	1.2714
76	2016	10	8.9857	75.5612	928.3255	4.0980	1.5949
36	2016	10	9.5143	73.3469	936.7286	3.9255	1.7582
81	2016	10	7.8143	79.5714	947.4806	3.5510	1.3337
15	2016	10	9.5714	76.1939	940.5796	3.5153	0.8102
32	2016	10	8.8714	70.3571	880.7867	4.7755	2.3592
73	2016	10	11.2143	73.0816	970.6418	3.7541	0.9173
71	2016	10	9.5143	73.3469	936.7286	3.9255	1.7582
41	2016	10	9.9143	76.7653	880.1092	5.7296	1.3235
10	2016	10	9.4571	78.8469	973.3429	3.7061	1.1388
23	2016	10	8.2714	66.3571	779.3122	6.7990	1.8194
27	2016	10	12.7714	68.3673	864.6265	6.7898	1.8000
60	2016	10	7.8143	79.5714	947.4806	3.5510	1.3337
53	2016	10	9.5857	71.6939	859.4194	5.5816	2.4122
66	2016	10	8.7429	74.9286	904.2888	3.3582	2.2163
59	2016	10	8.9429	72.6633	892.7704	4.9959	2.7602
61	2016	10	11.2143	73.0816	970.6418	3.7541	0.9173
84	2016	10	11.2143	73.0816	970.6418	3.7541	0.9173
38	2016	10	8.9429	72.6633	892.7704	4.9959	2.7602
87	2016	10	11.5857	68.3265	905.9776	4.8092	1.9408
34	2016	10	8.9429	72.6633	892.7704	4.9959	2.7602
29	2016	10	10.3571	74.2143	947.9276	3.5010	1.4163
5	2016	10	10.7143	68.1224	836.9378	6.9469	1.9908
8	2016	10	9.5857	71.6939	859.4194	5.5816	2.4122
12	2016	10	10.7143	68.1224	836.9378	6.9469	1.9908

13	2016	10	16.5714	67.2857	952.0357	6.3878	1.2714
18	2016	10	8.8571	80.8878	973.2918	3.2265	1.0296
33	2016	10	9.3429	72.1837	908.3378	4.5357	1.8112
56	2016	10	12.4429	78.2959	986.6398	3.1296	1.0480
77	2016	10	9.5714	76.1939	940.5796	3.5153	0.8102
54	2016	10	10.7143	68.1224	836.9378	6.9469	1.9908
21	2016	10	9.3429	72.1837	908.3378	4.5357	1.8112
68	2016	10	9.2429	84.8571	979.0204	3.4490	1.3184
74	2016	10	11.2143	73.0816	970.6418	3.7541	0.9173
88	2016	10	8.8714	70.3571	880.7867	4.7755	2.3592
16	2016	10	8.9857	75.5612	928.3255	4.0980	1.5949
30	2016	10	8.7429	74.9286	904.2888	3.3582	2.2163
6	2016	10	9.2429	84.8571	979.0204	3.4490	1.3184
49	2016	10	10.3571	74.2143	947.9276	3.5010	1.4163
22	2016	10	8.8714	70.3571	880.7867	4.7755	2.3592
45	2016	10	12.5143	64.5714	821.7990	6.9378	1.8112
58	2016	10	10.3571	74.2143	947.9276	3.5010	1.4163
37	2016	10	9.2429	84.8571	979.0204	3.4490	1.3184
17	2016	10	8.4000	76.6327	909.1592	3.3449	3.1398
55	2016	10	11.4429	71.9490	883.6204	5.1173	1.7398
46	2016	10	8.9857	75.5612	928.3255	4.0980	1.5949
86	2016	10	10.2714	69.8571	871.5112	5.6061	1.4173
2	2016	10	10.2714	69.8571	871.5112	5.6061	1.4173
4	2016	10	9.3429	72.1837	908.3378	4.5357	1.8112
47	2016	10	15.7571	69.7959	966.9071	4.8255	0.5694
82	2016	10	8.8714	70.3571	880.7867	4.7755	2.3592
19	2016	10	12.8286	75.7755	969.4204	3.5735	1.2306
20	2016	10	9.5857	71.6939	859.4194	5.5816	2.4122
80	2016	10	8.8714	70.3571	880.7867	4.7755	2.3592
3	2016	10	16.5714	67.2857	952.0357	6.3878	1.2714
52	2016	10	8.4000	76.6327	909.1592	3.3449	3.1398
70	2016	10	10.8714	68.3061	916.7010	5.3092	1.6786
64	2016	10	8.2714	66.3571	779.3122	6.7990	1.8194
48	2016	10	9.5714	76.1939	940.5796	3.5153	0.8102
65	2016	10	8.4000	76.6327	909.1592	3.3449	3.1398
44	2016	10	10.8714	68.3061	916.7010	5.3092	1.6786
75	2016	10	8.2714	66.3571	779.3122	6.7990	1.8194
40	2016	10	8.8286	78.4286	954.0765	3.5857	1.7010
11	2016	10	11.4429	71.9490	883.6204	5.1173	1.7398
35	2016	10	7.8143	79.5714	947.4806	3.5510	1.3337
78	2016	10	11.5857	68.3265	905.9776	4.8092	1.9408
28	2016	10	9.5143	73.3469	936.7286	3.9255	1.7582
39	2016	10	8.4000	76.6327	909.1592	3.3449	3.1398
24	2016	10	10.3571	74.2143	947.9276	3.5010	1.4163
63	2016	10	8.8286	78.4286	954.0765	3.5857	1.7010
62	2016	10	9.9143	76.7653	880.1092	5.7296	1.3235
1	2016	10	8.8714	70.3571	880.7867	4.7755	2.3592
31	2016	11	11.4000	77.5408	847.6316	3.2255	1.2224
79	2016	11	12.9571	81.7347	976.4837	1.2694	1.5429
51	2016	11	12.2571	82.9694	944.5806	1.5316	1.6418

14	2016	11	12.9000	83.7347	900.8439	1.4653	2.4429
67	2016	11	12.6143	85.8673	905.8969	1.4163	3.0092
42	2016	11	12.7857	77.8776	877.3357	2.4102	2.5378
50	2016	11	12.6286	74.2857	905.2765	1.6918	2.0337
43	2016	11	12.7857	77.8776	877.3357	2.4102	2.5378
85	2016	11	13.8857	71.8673	913.6704	2.7102	1.8418
25	2016	11	15.6714	79.0714	983.3898	1.7520	1.1918
69	2016	11	13.5857	76.5612	944.9827	1.4653	1.7367
57	2016	11	11.8286	81.5918	889.5469	2.5959	2.4694
9	2016	11	12.3571	77.5714	855.9143	3.4531	2.5643
72	2016	11	13.7143	76.3265	879.9878	2.9153	2.0071
26	2016	11	15.9857	69.3673	868.7673	5.2000	2.4133
7	2016	11	14.8000	69.5306	860.8296	5.2163	2.2582
83	2016	11	18.7571	65.2551	948.0857	3.8704	1.4255
76	2016	11	11.9000	79.0408	925.4704	1.2622	1.7449
36	2016	11	13.4429	77.9694	933.5959	1.8459	1.8612
81	2016	11	12.2571	82.9694	944.5806	1.5316	1.6418
15	2016	11	12.9143	78.6224	937.8204	1.0378	0.8531
32	2016	11	12.7857	77.8776	877.3357	2.4102	2.5378
73	2016	11	13.7714	74.0408	967.9888	1.3408	0.9745
71	2016	11	13.4429	77.9694	933.5959	1.8459	1.8612
41	2016	11	12.4571	79.8367	876.7765	2.7776	1.4663
10	2016	11	13.1286	81.9694	970.5612	1.3622	1.2408
23	2016	11	11.3714	68.3878	775.6867	6.1622	2.2735
27	2016	11	14.8000	69.5306	860.8296	5.2163	2.2582
60	2016	11	12.2571	82.9694	944.5806	1.5316	1.6418
53	2016	11	12.3571	77.5714	855.9143	3.4531	2.5643
66	2016	11	12.9000	83.7347	900.8439	1.4653	2.4429
59	2016	11	11.8286	81.5918	889.5469	2.5959	2.4694
61	2016	11	13.7714	74.0408	967.9888	1.3408	0.9745
84	2016	11	13.7714	74.0408	967.9888	1.3408	0.9745
38	2016	11	11.8286	81.5918	889.5469	2.5959	2.4694
87	2016	11	14.9714	71.2959	902.4398	2.7133	2.2367
34	2016	11	11.8286	81.5918	889.5469	2.5959	2.4694
29	2016	11	13.5857	76.5612	944.9827	1.4653	1.7367
5	2016	11	13.2000	72.5816	833.2031	5.7918	2.3173
8	2016	11	12.3571	77.5714	855.9143	3.4531	2.5643
12	2016	11	13.2000	72.5816	833.2031	5.7918	2.3173
13	2016	11	18.7571	65.2551	948.0857	3.8704	1.4255
18	2016	11	13.0286	83.5612	970.7837	1.1520	1.0929
33	2016	11	12.6286	74.2857	905.2765	1.6918	2.0337
56	2016	11	15.6714	79.0714	983.3898	1.7520	1.1918
77	2016	11	12.9143	78.6224	937.8204	1.0378	0.8531
54	2016	11	13.2000	72.5816	833.2031	5.7918	2.3173
21	2016	11	12.6286	74.2857	905.2765	1.6918	2.0337
68	2016	11	12.9571	81.7347	976.4837	1.2694	1.5429
74	2016	11	13.7714	74.0408	967.9888	1.3408	0.9745
88	2016	11	12.7857	77.8776	877.3357	2.4102	2.5378
16	2016	11	11.9000	79.0408	925.4704	1.2622	1.7449
30	2016	11	12.9000	83.7347	900.8439	1.4653	2.4429

6	2016	11	12.9571	81.7347	976.4837	1.2694	1.5429
49	2016	11	13.5857	76.5612	944.9827	1.4653	1.7367
22	2016	11	12.7857	77.8776	877.3357	2.4102	2.5378
45	2016	11	16.0571	59.2041	817.9582	6.4867	2.5367
58	2016	11	13.5857	76.5612	944.9827	1.4653	1.7367
37	2016	11	12.9571	81.7347	976.4837	1.2694	1.5429
17	2016	11	12.6143	85.8673	905.8969	1.4163	3.0092
55	2016	11	13.7143	76.3265	879.9878	2.9153	2.0071
46	2016	11	11.9000	79.0408	925.4704	1.2622	1.7449
86	2016	11	12.8143	74.6939	868.0633	3.2173	1.4755
2	2016	11	12.8143	74.6939	868.0633	3.2173	1.4755
4	2016	11	12.6286	74.2857	905.2765	1.6918	2.0337
47	2016	11	17.9571	68.1633	963.2480	2.5357	0.7010
82	2016	11	12.7857	77.8776	877.3357	2.4102	2.5378
19	2016	11	16.6143	78.7041	965.9173	1.7867	1.3520
20	2016	11	12.3571	77.5714	855.9143	3.4531	2.5643
80	2016	11	12.7857	77.8776	877.3357	2.4102	2.5378
3	2016	11	18.7571	65.2551	948.0857	3.8704	1.4255
52	2016	11	12.6143	85.8673	905.8969	1.4163	3.0092
70	2016	11	13.8857	71.8673	913.6704	2.7102	1.8418
64	2016	11	11.3714	68.3878	775.6867	6.1622	2.2735
48	2016	11	12.9143	78.6224	937.8204	1.0378	0.8531
65	2016	11	12.6143	85.8673	905.8969	1.4163	3.0092
44	2016	11	13.8857	71.8673	913.6704	2.7102	1.8418
75	2016	11	11.3714	68.3878	775.6867	6.1622	2.2735
40	2016	11	12.6286	85.6531	951.0765	1.2694	1.7469
11	2016	11	13.7143	76.3265	879.9878	2.9153	2.0071
35	2016	11	12.2571	82.9694	944.5806	1.5316	1.6418
78	2016	11	14.9714	71.2959	902.4398	2.7133	2.2367
28	2016	11	13.4429	77.9694	933.5959	1.8459	1.8612
39	2016	11	12.6143	85.8673	905.8969	1.4163	3.0092
24	2016	11	13.5857	76.5612	944.9827	1.4653	1.7367
63	2016	11	12.6286	85.6531	951.0765	1.2694	1.7469
62	2016	11	12.4571	79.8367	876.7765	2.7776	1.4663
1	2016	11	12.7857	77.8776	877.3357	2.4102	2.5378
31	2016	12	9.6429	79.1531	847.9724	2.0663	1.3153
79	2016	12	11.4714	79.8265	976.6776	1.3806	1.4990
51	2016	12	9.8857	83.9592	944.8010	1.9796	1.6459
14	2016	12	9.7714	85.7959	900.7041	1.7969	2.2000
67	2016	12	8.9143	89.0714	905.9857	1.2541	2.4847
42	2016	12	8.9429	82.6633	877.4184	1.8959	2.5276
50	2016	12	10.7714	75.2959	905.6653	1.0837	1.8653
43	2016	12	8.9429	82.6633	877.4184	1.8959	2.5276
85	2016	12	11.7571	75.3367	914.1082	1.4969	1.5347
25	2016	12	13.1286	82.8469	983.2388	1.7776	0.9061
69	2016	12	11.1286	80.1531	945.2908	1.3306	1.5051
57	2016	12	9.1000	88.8265	889.8469	1.7276	2.1031
9	2016	12	9.8571	81.0612	855.9867	3.0071	2.4735
72	2016	12	11.7857	79.2653	879.9571	2.4418	2.1878
26	2016	12	14.1429	70.2857	868.5929	5.0520	2.6010

7	2016	12	12.7857	71.8265	860.7500	4.9184	2.2622
83	2016	12	17.1000	66.7653	947.8500	2.9184	1.4102
76	2016	12	10.4286	81.8980	925.9020	0.9541	1.7684
36	2016	12	10.0286	82.6531	933.7367	1.5959	1.8153
81	2016	12	9.8857	83.9592	944.8010	1.9796	1.6459
15	2016	12	11.7429	79.2347	938.2214	0.9643	0.8000
32	2016	12	8.9429	82.6633	877.4184	1.8959	2.5276
73	2016	12	12.7143	77.2653	968.3867	1.3694	0.9622
71	2016	12	10.0286	82.6531	933.7367	1.5959	1.8153
41	2016	12	9.8571	84.2041	877.0867	1.2520	1.3592
10	2016	12	11.1286	83.5816	970.6571	1.3010	1.0745
23	2016	12	7.1714	68.3878	775.8347	5.6010	2.3786
27	2016	12	12.7857	71.8265	860.7500	4.9184	2.2622
60	2016	12	9.8857	83.9592	944.8010	1.9796	1.6459
53	2016	12	9.8571	81.0612	855.9867	3.0071	2.4735
66	2016	12	9.7714	85.7959	900.7041	1.7969	2.2000
59	2016	12	9.1000	88.8265	889.8469	1.7276	2.1031
61	2016	12	12.7143	77.2653	968.3867	1.3694	0.9622
84	2016	12	12.7143	77.2653	968.3867	1.3694	0.9622
38	2016	12	9.1000	88.8265	889.8469	1.7276	2.1031
87	2016	12	11.6571	74.3776	902.4500	2.3622	2.3398
34	2016	12	9.1000	88.8265	889.8469	1.7276	2.1031
29	2016	12	11.1286	80.1531	945.2908	1.3306	1.5051
5	2016	12	10.7286	75.1429	833.2337	5.2061	2.3541
8	2016	12	9.8571	81.0612	855.9867	3.0071	2.4735
12	2016	12	10.7286	75.1429	833.2337	5.2061	2.3541
13	2016	12	17.1000	66.7653	947.8500	2.9184	1.4102
18	2016	12	10.8143	83.9490	971.0316	1.4888	1.0663
33	2016	12	10.7714	75.2959	905.6653	1.0837	1.8653
56	2016	12	13.1286	82.8469	983.2388	1.7776	0.9061
77	2016	12	11.7429	79.2347	938.2214	0.9643	0.8000
54	2016	12	10.7286	75.1429	833.2337	5.2061	2.3541
21	2016	12	10.7714	75.2959	905.6653	1.0837	1.8653
68	2016	12	11.4714	79.8265	976.6776	1.3806	1.4990
74	2016	12	12.7143	77.2653	968.3867	1.3694	0.9622
88	2016	12	8.9429	82.6633	877.4184	1.8959	2.5276
16	2016	12	10.4286	81.8980	925.9020	0.9541	1.7684
30	2016	12	9.7714	85.7959	900.7041	1.7969	2.2000
6	2016	12	11.4714	79.8265	976.6776	1.3806	1.4990
49	2016	12	11.1286	80.1531	945.2908	1.3306	1.5051
22	2016	12	8.9429	82.6633	877.4184	1.8959	2.5276
45	2016	12	11.2286	57.9694	817.9571	6.1959	2.5643
58	2016	12	11.1286	80.1531	945.2908	1.3306	1.5051
37	2016	12	11.4714	79.8265	976.6776	1.3806	1.4990
17	2016	12	8.9143	89.0714	905.9857	1.2541	2.4847
55	2016	12	11.7857	79.2653	879.9571	2.4418	2.1878
46	2016	12	10.4286	81.8980	925.9020	0.9541	1.7684
86	2016	12	10.3000	78.7041	868.3143	2.4776	1.1796
2	2016	12	10.3000	78.7041	868.3143	2.4776	1.1796
4	2016	12	10.7714	75.2959	905.6653	1.0837	1.8653

47	2016	12	15.6143	70.7959	963.0643	1.6531	0.6255
82	2016	12	8.9429	82.6633	877.4184	1.8959	2.5276
19	2016	12	13.8714	81.2959	965.5776	1.6806	1.2071
20	2016	12	9.8571	81.0612	855.9867	3.0071	2.4735
80	2016	12	8.9429	82.6633	877.4184	1.8959	2.5276
3	2016	12	17.1000	66.7653	947.8500	2.9184	1.4102
52	2016	12	8.9143	89.0714	905.9857	1.2541	2.4847
70	2016	12	11.7571	75.3367	914.1082	1.4969	1.5347
64	2016	12	7.1714	68.3878	775.8347	5.6010	2.3786
48	2016	12	11.7429	79.2347	938.2214	0.9643	0.8000
65	2016	12	8.9143	89.0714	905.9857	1.2541	2.4847
44	2016	12	11.7571	75.3367	914.1082	1.4969	1.5347
75	2016	12	7.1714	68.3878	775.8347	5.6010	2.3786
40	2016	12	9.9000	89.5102	950.9898	1.1347	1.3786
11	2016	12	11.7857	79.2653	879.9571	2.4418	2.1878
35	2016	12	9.8857	83.9592	944.8010	1.9796	1.6459
78	2016	12	11.6571	74.3776	902.4500	2.3622	2.3398
28	2016	12	10.0286	82.6531	933.7367	1.5959	1.8153
39	2016	12	8.9143	89.0714	905.9857	1.2541	2.4847
24	2016	12	11.1286	80.1531	945.2908	1.3306	1.5051
63	2016	12	9.9000	89.5102	950.9898	1.1347	1.3786
62	2016	12	9.8571	84.2041	877.0867	1.2520	1.3592
1	2016	12	8.9429	82.6633	877.4184	1.8959	2.5276
31	2016	13	13.3000	80.5102	849.9561	2.2735	1.1949
79	2016	13	16.2429	79.7041	977.8061	1.7643	1.3224
51	2016	13	15.4857	84.5510	946.3051	2.1316	1.3755
14	2016	13	13.9429	87.0816	902.7031	1.5041	2.0653
67	2016	13	14.1000	91.1020	907.8633	1.0755	2.3112
42	2016	13	13.6000	86.5102	879.3806	1.8724	2.3378
50	2016	13	13.6000	78.6122	907.3765	1.3592	1.7041
43	2016	13	13.6000	86.5102	879.3806	1.8724	2.3378
85	2016	13	14.6000	78.6735	915.8378	1.6735	1.3133
25	2016	13	17.3857	86.1531	984.9490	1.3561	0.7612
69	2016	13	15.8714	82.6224	946.8510	1.3816	1.2235
57	2016	13	13.7000	90.5204	891.7153	2.0020	2.0888
9	2016	13	13.0286	85.4694	858.0051	3.0031	2.2735
72	2016	13	14.2571	83.9796	882.0255	2.6980	1.8378
26	2016	13	14.9714	75.8878	870.8112	4.2694	2.4490
7	2016	13	14.6714	76.2653	862.9316	4.2939	1.9745
83	2016	13	18.6143	73.0918	950.0459	2.9776	1.1796
76	2016	13	14.3143	82.6224	927.4622	1.6531	1.6051
36	2016	13	15.3571	85.6633	935.5122	1.4449	1.6520
81	2016	13	15.4857	84.5510	946.3051	2.1316	1.3755
15	2016	13	14.0571	80.7857	939.6939	1.4541	0.7602
32	2016	13	13.6000	86.5102	879.3806	1.8724	2.3378
73	2016	13	16.0714	79.2449	969.7102	2.0408	0.8867
71	2016	13	15.3571	85.6633	935.5122	1.4449	1.6520
41	2016	13	14.3000	87.5306	878.9969	1.5776	1.2490
10	2016	13	16.3286	83.7143	972.0276	1.9704	0.9306
23	2016	13	11.2000	72.3061	777.9888	4.7398	2.3051

27	2016	13	14.6714	76.2653	862.9316	4.2939	1.9745
60	2016	13	15.4857	84.5510	946.3051	2.1316	1.3755
53	2016	13	13.0286	85.4694	858.0051	3.0031	2.2735
66	2016	13	13.9429	87.0816	902.7031	1.5041	2.0653
59	2016	13	13.7000	90.5204	891.7153	2.0020	2.0888
61	2016	13	16.0714	79.2449	969.7102	2.0408	0.8867
84	2016	13	16.0714	79.2449	969.7102	2.0408	0.8867
38	2016	13	13.7000	90.5204	891.7153	2.0020	2.0888
87	2016	13	15.3429	79.4490	904.4643	2.2122	1.9592
34	2016	13	13.7000	90.5204	891.7153	2.0020	2.0888
29	2016	13	15.8714	82.6224	946.8510	1.3816	1.2235
5	2016	13	13.7857	77.3163	835.4490	4.3224	2.1031
8	2016	13	13.0286	85.4694	858.0051	3.0031	2.2735
12	2016	13	13.7857	77.3163	835.4490	4.3224	2.1031
13	2016	13	18.6143	73.0918	950.0459	2.9776	1.1796
18	2016	13	15.7429	85.3367	972.1735	1.7194	0.9194
33	2016	13	13.6000	78.6122	907.3765	1.3592	1.7041
56	2016	13	17.3857	86.1531	984.9490	1.3561	0.7612
77	2016	13	14.0571	80.7857	939.6939	1.4541	0.7602
54	2016	13	13.7857	77.3163	835.4490	4.3224	2.1031
21	2016	13	13.6000	78.6122	907.3765	1.3592	1.7041
68	2016	13	16.2429	79.7041	977.8061	1.7643	1.3224
74	2016	13	16.0714	79.2449	969.7102	2.0408	0.8867
88	2016	13	13.6000	86.5102	879.3806	1.8724	2.3378
16	2016	13	14.3143	82.6224	927.4622	1.6531	1.6051
30	2016	13	13.9429	87.0816	902.7031	1.5041	2.0653
6	2016	13	16.2429	79.7041	977.8061	1.7643	1.3224
49	2016	13	15.8714	82.6224	946.8510	1.3816	1.2235
22	2016	13	13.6000	86.5102	879.3806	1.8724	2.3378
45	2016	13	13.9286	67.5510	820.2112	4.9816	1.9306
58	2016	13	15.8714	82.6224	946.8510	1.3816	1.2235
37	2016	13	16.2429	79.7041	977.8061	1.7643	1.3224
17	2016	13	14.1000	91.1020	907.8633	1.0755	2.3112
55	2016	13	14.2571	83.9796	882.0255	2.6980	1.8378
46	2016	13	14.3143	82.6224	927.4622	1.6531	1.6051
86	2016	13	14.2714	82.4184	870.2480	2.6541	1.2602
2	2016	13	14.2714	82.4184	870.2480	2.6541	1.2602
4	2016	13	13.6000	78.6122	907.3765	1.3592	1.7041
47	2016	13	17.7857	77.2857	965.0704	2.0408	0.4469
82	2016	13	13.6000	86.5102	879.3806	1.8724	2.3378
19	2016	13	16.9429	84.7143	967.5306	1.1031	0.9408
20	2016	13	13.0286	85.4694	858.0051	3.0031	2.2735
80	2016	13	13.6000	86.5102	879.3806	1.8724	2.3378
3	2016	13	18.6143	73.0918	950.0459	2.9776	1.1796
52	2016	13	14.1000	91.1020	907.8633	1.0755	2.3112
70	2016	13	14.6000	78.6735	915.8378	1.6735	1.3133
64	2016	13	11.2000	72.3061	777.9888	4.7398	2.3051
48	2016	13	14.0571	80.7857	939.6939	1.4541	0.7602
65	2016	13	14.1000	91.1020	907.8633	1.0755	2.3112
44	2016	13	14.6000	78.6735	915.8378	1.6735	1.3133

75	2016	13	11.2000	72.3061	777.9888	4.7398	2.3051
40	2016	13	16.3000	87.5714	952.6102	1.3663	1.3061
11	2016	13	14.2571	83.9796	882.0255	2.6980	1.8378
35	2016	13	15.4857	84.5510	946.3051	2.1316	1.3755
78	2016	13	15.3429	79.4490	904.4643	2.2122	1.9592
28	2016	13	15.3571	85.6633	935.5122	1.4449	1.6520
39	2016	13	14.1000	91.1020	907.8633	1.0755	2.3112
24	2016	13	15.8714	82.6224	946.8510	1.3816	1.2235
63	2016	13	16.3000	87.5714	952.6102	1.3663	1.3061
62	2016	13	14.3000	87.5306	878.9969	1.5776	1.2490
1	2016	13	13.6000	86.5102	879.3806	1.8724	2.3378
31	2016	14	15.5286	78.7653	848.6735	3.0112	0.9959
79	2016	14	16.0143	79.7857	975.1408	1.6143	1.2235
51	2016	14	15.4286	84.1327	943.9622	2.2378	1.4531
14	2016	14	18.1429	87.1531	901.2133	0.9051	2.4214
67	2016	14	17.1714	90.6020	906.0041	1.0612	2.4143
42	2016	14	17.6429	84.7959	877.8714	2.1724	2.2857
50	2016	14	16.0714	80.9592	905.5316	1.1500	1.4867
43	2016	14	17.6429	84.7959	877.8714	2.1724	2.2857
85	2016	14	17.2857	80.0918	913.9592	1.9939	1.3143
25	2016	14	20.4429	85.6327	982.4622	1.0449	0.7296
69	2016	14	17.5857	82.5102	944.4908	1.2469	1.2745
57	2016	14	15.5143	89.2143	889.9847	2.4776	2.1959
9	2016	14	17.7714	84.4592	856.7898	3.9469	2.4122
72	2016	14	19.4143	83.6939	880.7092	3.7816	1.5673
26	2016	14	21.5429	77.1837	869.8755	4.7929	2.2857
7	2016	14	20.2143	76.3061	861.9745	4.3163	2.1531
83	2016	14	24.2571	75.4898	948.4214	3.5673	1.1143
76	2016	14	15.6714	83.5204	925.2898	1.6918	1.4337
36	2016	14	17.9286	84.3469	933.2908	1.5071	1.5214
81	2016	14	15.4286	84.1327	943.9622	2.2378	1.4531
15	2016	14	16.7286	82.8469	937.4898	1.2133	0.7245
32	2016	14	17.6429	84.7959	877.8714	2.1724	2.2857
73	2016	14	17.3714	79.5918	967.1500	1.8755	0.8724
71	2016	14	17.9286	84.3469	933.2908	1.5071	1.5214
41	2016	14	17.0714	84.7449	877.3480	2.0929	1.1459
10	2016	14	16.8143	82.5102	969.4908	2.3857	1.0184
23	2016	14	14.5571	71.7857	777.4347	5.6214	2.3398
27	2016	14	20.2143	76.3061	861.9745	4.3163	2.1531
60	2016	14	15.4286	84.1327	943.9622	2.2378	1.4531
53	2016	14	17.7714	84.4592	856.7898	3.9469	2.4122
66	2016	14	18.1429	87.1531	901.2133	0.9051	2.4214
59	2016	14	15.5143	89.2143	889.9847	2.4776	2.1959
61	2016	14	17.3714	79.5918	967.1500	1.8755	0.8724
84	2016	14	17.3714	79.5918	967.1500	1.8755	0.8724
38	2016	14	15.5143	89.2143	889.9847	2.4776	2.1959
87	2016	14	19.8714	79.9286	902.7908	2.9214	1.7245
34	2016	14	15.5143	89.2143	889.9847	2.4776	2.1959
29	2016	14	17.5857	82.5102	944.4908	1.2469	1.2745
5	2016	14	18.6429	73.3776	834.5827	5.0000	2.3041

8	2016	14	17.7714	84.4592	856.7898	3.9469	2.4122
12	2016	14	18.6429	73.3776	834.5827	5.0000	2.3041
13	2016	14	24.2571	75.4898	948.4214	3.5673	1.1143
18	2016	14	16.0714	85.5102	969.5143	1.5061	0.8949
33	2016	14	16.0714	80.9592	905.5316	1.1500	1.4867
56	2016	14	20.4429	85.6327	982.4622	1.0449	0.7296
77	2016	14	16.7286	82.8469	937.4898	1.2133	0.7245
54	2016	14	18.6429	73.3776	834.5827	5.0000	2.3041
21	2016	14	16.0714	80.9592	905.5316	1.1500	1.4867
68	2016	14	16.0143	79.7857	975.1408	1.6143	1.2235
74	2016	14	17.3714	79.5918	967.1500	1.8755	0.8724
88	2016	14	17.6429	84.7959	877.8714	2.1724	2.2857
16	2016	14	15.6714	83.5204	925.2898	1.6918	1.4337
30	2016	14	18.1429	87.1531	901.2133	0.9051	2.4214
6	2016	14	16.0143	79.7857	975.1408	1.6143	1.2235
49	2016	14	17.5857	82.5102	944.4908	1.2469	1.2745
22	2016	14	17.6429	84.7959	877.8714	2.1724	2.2857
45	2016	14	18.7286	68.6429	819.5571	5.4112	2.0000
58	2016	14	17.5857	82.5102	944.4908	1.2469	1.2745
37	2016	14	16.0143	79.7857	975.1408	1.6143	1.2235
17	2016	14	17.1714	90.6020	906.0041	1.0612	2.4143
55	2016	14	19.4143	83.6939	880.7092	3.7816	1.5673
46	2016	14	15.6714	83.5204	925.2898	1.6918	1.4337
86	2016	14	17.4857	80.0000	868.7102	3.6745	1.4357
2	2016	14	17.4857	80.0000	868.7102	3.6745	1.4357
4	2016	14	16.0714	80.9592	905.5316	1.1500	1.4867
47	2016	14	23.4714	79.5000	963.0163	3.2571	0.4592
82	2016	14	17.6429	84.7959	877.8714	2.1724	2.2857
19	2016	14	21.3429	86.7143	965.5714	0.8878	0.8469
20	2016	14	17.7714	84.4592	856.7898	3.9469	2.4122
80	2016	14	17.6429	84.7959	877.8714	2.1724	2.2857
3	2016	14	24.2571	75.4898	948.4214	3.5673	1.1143
52	2016	14	17.1714	90.6020	906.0041	1.0612	2.4143
70	2016	14	17.2857	80.0918	913.9592	1.9939	1.3143
64	2016	14	14.5571	71.7857	777.4347	5.6214	2.3398
48	2016	14	16.7286	82.8469	937.4898	1.2133	0.7245
65	2016	14	17.1714	90.6020	906.0041	1.0612	2.4143
44	2016	14	17.2857	80.0918	913.9592	1.9939	1.3143
75	2016	14	14.5571	71.7857	777.4347	5.6214	2.3398
40	2016	14	17.4714	84.2245	950.4561	1.8031	1.5296
11	2016	14	19.4143	83.6939	880.7092	3.7816	1.5673
35	2016	14	15.4286	84.1327	943.9622	2.2378	1.4531
78	2016	14	19.8714	79.9286	902.7908	2.9214	1.7245
28	2016	14	17.9286	84.3469	933.2908	1.5071	1.5214
39	2016	14	17.1714	90.6020	906.0041	1.0612	2.4143
24	2016	14	17.5857	82.5102	944.4908	1.2469	1.2745
63	2016	14	17.4714	84.2245	950.4561	1.8031	1.5296
62	2016	14	17.0714	84.7449	877.3480	2.0929	1.1459
1	2016	14	17.6429	84.7959	877.8714	2.1724	2.2857
31	2016	15	16.0286	80.5918	845.4571	2.3990	1.0173

79	2016	15	18.8714	83.8469	970.0582	1.2224	1.1520
51	2016	15	17.7857	86.3673	939.1714	1.9520	1.6153
14	2016	15	18.4286	86.7959	896.7724	1.2184	2.6469
67	2016	15	18.4857	89.4694	901.5378	1.3173	2.4765
42	2016	15	17.6143	82.6735	873.9643	2.8837	2.5714
50	2016	15	17.4000	81.5918	901.4724	0.9735	1.5439
43	2016	15	17.6143	82.6735	873.9643	2.8837	2.5714
85	2016	15	18.1714	81.8367	909.7704	2.0224	1.4235
25	2016	15	21.3000	86.1531	976.5765	1.6745	0.8061
69	2016	15	19.0286	83.0306	939.6602	1.2378	1.4184
57	2016	15	16.7571	90.7143	886.0867	2.1898	2.1714
9	2016	15	17.5714	81.2653	853.1735	5.1857	2.8327
72	2016	15	19.2857	80.5612	876.6541	4.8051	1.8592
26	2016	15	21.0286	68.2755	865.8582	7.0408	2.4878
7	2016	15	19.1714	72.0612	858.1888	5.7092	2.4980
83	2016	15	24.2429	71.8776	942.9520	5.4622	1.3939
76	2016	15	17.4143	86.2449	920.8959	1.2592	1.5143
36	2016	15	19.0571	83.8163	928.4490	2.2041	1.4408
81	2016	15	17.7857	86.3673	939.1714	1.9520	1.6153
15	2016	15	18.4000	83.4286	932.9418	1.0796	0.8704
32	2016	15	17.6143	82.6735	873.9643	2.8837	2.5714
73	2016	15	19.2571	82.9898	962.1541	1.7173	0.8653
71	2016	15	19.0571	83.8163	928.4490	2.2041	1.4408
41	2016	15	17.3429	84.7755	873.6204	2.1510	1.0031
10	2016	15	18.7286	85.3061	964.2245	1.8541	1.1143
23	2016	15	13.3143	70.4388	775.0296	6.9837	2.2857
27	2016	15	19.1714	72.0612	858.1888	5.7092	2.4980
60	2016	15	17.7857	86.3673	939.1714	1.9520	1.6153
53	2016	15	17.5714	81.2653	853.1735	5.1857	2.8327
66	2016	15	18.4286	86.7959	896.7724	1.2184	2.6469
59	2016	15	16.7571	90.7143	886.0867	2.1898	2.1714
61	2016	15	19.2571	82.9898	962.1541	1.7173	0.8653
84	2016	15	19.2571	82.9898	962.1541	1.7173	0.8653
38	2016	15	16.7571	90.7143	886.0867	2.1898	2.1714
87	2016	15	20.0857	76.8776	898.3510	4.4092	1.9153
34	2016	15	16.7571	90.7143	886.0867	2.1898	2.1714
29	2016	15	19.0286	83.0306	939.6602	1.2378	1.4184
5	2016	15	17.2429	70.4388	831.2449	6.6929	2.6990
8	2016	15	17.5714	81.2653	853.1735	5.1857	2.8327
12	2016	15	17.2429	70.4388	831.2449	6.6929	2.6990
13	2016	15	24.2429	71.8776	942.9520	5.4622	1.3939
18	2016	15	18.5143	86.5306	964.4878	1.2316	0.9959
33	2016	15	17.4000	81.5918	901.4724	0.9735	1.5439
56	2016	15	21.3000	86.1531	976.5765	1.6745	0.8061
77	2016	15	18.4000	83.4286	932.9418	1.0796	0.8704
54	2016	15	17.2429	70.4388	831.2449	6.6929	2.6990
21	2016	15	17.4000	81.5918	901.4724	0.9735	1.5439
68	2016	15	18.8714	83.8469	970.0582	1.2224	1.1520
74	2016	15	19.2571	82.9898	962.1541	1.7173	0.8653
88	2016	15	17.6143	82.6735	873.9643	2.8837	2.5714

16	2016	15	17.4143	86.2449	920.8959	1.2592	1.5143
30	2016	15	18.4286	86.7959	896.7724	1.2184	2.6469
6	2016	15	18.8714	83.8469	970.0582	1.2224	1.1520
49	2016	15	19.0286	83.0306	939.6602	1.2378	1.4184
22	2016	15	17.6143	82.6735	873.9643	2.8837	2.5714
45	2016	15	18.2000	60.0204	816.4204	6.9633	2.2704
58	2016	15	19.0286	83.0306	939.6602	1.2378	1.4184
37	2016	15	18.8714	83.8469	970.0582	1.2224	1.1520
17	2016	15	18.4857	89.4694	901.5378	1.3173	2.4765
55	2016	15	19.2857	80.5612	876.6541	4.8051	1.8592
46	2016	15	17.4143	86.2449	920.8959	1.2592	1.5143
86	2016	15	17.8429	79.4388	865.0531	3.6694	1.1367
2	2016	15	17.8429	79.4388	865.0531	3.6694	1.1367
4	2016	15	17.4000	81.5918	901.4724	0.9735	1.5439
47	2016	15	23.2571	77.5102	957.3418	4.7776	0.6500
82	2016	15	17.6143	82.6735	873.9643	2.8837	2.5714
19	2016	15	22.2429	87.1122	959.8827	1.5337	0.9786
20	2016	15	17.5714	81.2653	853.1735	5.1857	2.8327
80	2016	15	17.6143	82.6735	873.9643	2.8837	2.5714
3	2016	15	24.2429	71.8776	942.9520	5.4622	1.3939
52	2016	15	18.4857	89.4694	901.5378	1.3173	2.4765
70	2016	15	18.1714	81.8367	909.7704	2.0224	1.4235
64	2016	15	13.3143	70.4388	775.0296	6.9837	2.2857
48	2016	15	18.4000	83.4286	932.9418	1.0796	0.8704
65	2016	15	18.4857	89.4694	901.5378	1.3173	2.4765
44	2016	15	18.1714	81.8367	909.7704	2.0224	1.4235
75	2016	15	13.3143	70.4388	775.0296	6.9837	2.2857
40	2016	15	18.4429	87.2857	945.3531	1.8520	1.4745
11	2016	15	19.2857	80.5612	876.6541	4.8051	1.8592
35	2016	15	17.7857	86.3673	939.1714	1.9520	1.6153
78	2016	15	20.0857	76.8776	898.3510	4.4092	1.9153
28	2016	15	19.0571	83.8163	928.4490	2.2041	1.4408
39	2016	15	18.4857	89.4694	901.5378	1.3173	2.4765
24	2016	15	19.0286	83.0306	939.6602	1.2378	1.4184
63	2016	15	18.4429	87.2857	945.3531	1.8520	1.4745
62	2016	15	17.3429	84.7755	873.6204	2.1510	1.0031
1	2016	15	17.6143	82.6735	873.9643	2.8837	2.5714
31	2016	16	12.8000	79.6531	845.8796	2.4908	1.2194
79	2016	16	17.7286	83.6837	969.9194	1.7735	1.0051
51	2016	16	16.4286	85.5510	939.0796	2.2857	1.4643
14	2016	16	16.1000	83.1122	896.7755	2.5367	2.5112
67	2016	16	15.7857	84.1837	901.6418	2.3010	2.4694
42	2016	16	14.7857	78.6939	874.3082	3.5684	2.8092
50	2016	16	14.3571	78.5102	901.7959	1.8612	1.6816
43	2016	16	14.7857	78.6939	874.3082	3.5684	2.8092
85	2016	16	15.4143	79.0612	910.1153	2.8653	1.4949
25	2016	16	19.2143	86.1122	976.4010	2.6755	0.8908
69	2016	16	17.4714	81.0000	939.7592	1.9184	1.3500
57	2016	16	14.5000	85.9082	886.4500	2.8694	2.4612
9	2016	16	14.3429	77.5714	853.5133	5.1255	2.7776

72	2016	16	15.9857	77.3571	876.9347	4.9745	2.0133
26	2016	16	17.2143	66.6939	866.0480	6.3092	2.5418
7	2016	16	16.1857	71.9388	858.4408	5.6316	2.4051
83	2016	16	20.2857	69.8265	943.1827	6.0306	1.4541
76	2016	16	15.5714	83.6837	921.0704	1.7388	1.6010
36	2016	16	17.2000	82.1531	928.5245	3.1561	1.4153
81	2016	16	16.4286	85.5510	939.0796	2.2857	1.4643
15	2016	16	15.7000	81.6531	933.0755	1.7459	0.9571
32	2016	16	14.7857	78.6939	874.3082	3.5684	2.8092
73	2016	16	17.7286	83.1327	962.1296	2.3316	0.8888
71	2016	16	17.2000	82.1531	928.5245	3.1561	1.4153
41	2016	16	14.5429	81.1735	874.1102	2.7194	1.1816
10	2016	16	17.6857	85.4286	964.0265	2.1316	0.9255
23	2016	16	10.3429	74.0204	775.3133	5.7531	2.3214
27	2016	16	16.1857	71.9388	858.4408	5.6316	2.4051
60	2016	16	16.4286	85.5510	939.0796	2.2857	1.4643
53	2016	16	14.3429	77.5714	853.5133	5.1255	2.7776
66	2016	16	16.1000	83.1122	896.7755	2.5367	2.5112
59	2016	16	14.5000	85.9082	886.4500	2.8694	2.4612
61	2016	16	17.7286	83.1327	962.1296	2.3316	0.8888
84	2016	16	17.7286	83.1327	962.1296	2.3316	0.8888
38	2016	16	14.5000	85.9082	886.4500	2.8694	2.4612
87	2016	16	16.5714	73.5612	898.7071	4.7898	2.1194
34	2016	16	14.5000	85.9082	886.4500	2.8694	2.4612
29	2016	16	17.4714	81.0000	939.7592	1.9184	1.3500
5	2016	16	14.5857	73.4184	831.5276	6.3612	2.5143
8	2016	16	14.3429	77.5714	853.5133	5.1255	2.7776
12	2016	16	14.5857	73.4184	831.5276	6.3612	2.5143
13	2016	16	20.2857	69.8265	943.1827	6.0306	1.4541
18	2016	16	17.3143	85.0204	964.2551	1.6816	0.9163
33	2016	16	14.3571	78.5102	901.7959	1.8612	1.6816
56	2016	16	19.2143	86.1122	976.4010	2.6755	0.8908
77	2016	16	15.7000	81.6531	933.0755	1.7459	0.9571
54	2016	16	14.5857	73.4184	831.5276	6.3612	2.5143
21	2016	16	14.3571	78.5102	901.7959	1.8612	1.6816
68	2016	16	17.7286	83.6837	969.9194	1.7735	1.0051
74	2016	16	17.7286	83.1327	962.1296	2.3316	0.8888
88	2016	16	14.7857	78.6939	874.3082	3.5684	2.8092
16	2016	16	15.5714	83.6837	921.0704	1.7388	1.6010
30	2016	16	16.1000	83.1122	896.7755	2.5367	2.5112
6	2016	16	17.7286	83.6837	969.9194	1.7735	1.0051
49	2016	16	17.4714	81.0000	939.7592	1.9184	1.3500
22	2016	16	14.7857	78.6939	874.3082	3.5684	2.8092
45	2016	16	15.1000	61.2347	816.6480	6.2959	2.3684
58	2016	16	17.4714	81.0000	939.7592	1.9184	1.3500
37	2016	16	17.7286	83.6837	969.9194	1.7735	1.0051
17	2016	16	15.7857	84.1837	901.6418	2.3010	2.4694
55	2016	16	15.9857	77.3571	876.9347	4.9745	2.0133
46	2016	16	15.5714	83.6837	921.0704	1.7388	1.6010
86	2016	16	14.4429	76.6429	865.5469	3.2214	1.2612

2	2016	16	14.4429	76.6429	865.5469	3.2214	1.2612
4	2016	16	14.3571	78.5102	901.7959	1.8612	1.6816
47	2016	16	19.9857	74.9490	957.5888	4.7847	0.6592
82	2016	16	14.7857	78.6939	874.3082	3.5684	2.8092
19	2016	16	19.7429	82.5510	959.6786	2.6663	0.9908
20	2016	16	14.3429	77.5714	853.5133	5.1255	2.7776
80	2016	16	14.7857	78.6939	874.3082	3.5684	2.8092
3	2016	16	20.2857	69.8265	943.1827	6.0306	1.4541
52	2016	16	15.7857	84.1837	901.6418	2.3010	2.4694
70	2016	16	15.4143	79.0612	910.1153	2.8653	1.4949
64	2016	16	10.3429	74.0204	775.3133	5.7531	2.3214
48	2016	16	15.7000	81.6531	933.0755	1.7459	0.9571
65	2016	16	15.7857	84.1837	901.6418	2.3010	2.4694
44	2016	16	15.4143	79.0612	910.1153	2.8653	1.4949
75	2016	16	10.3429	74.0204	775.3133	5.7531	2.3214
40	2016	16	17.7429	87.5000	945.1398	2.4031	1.1969
11	2016	16	15.9857	77.3571	876.9347	4.9745	2.0133
35	2016	16	16.4286	85.5510	939.0796	2.2857	1.4643
78	2016	16	16.5714	73.5612	898.7071	4.7898	2.1194
28	2016	16	17.2000	82.1531	928.5245	3.1561	1.4153
39	2016	16	15.7857	84.1837	901.6418	2.3010	2.4694
24	2016	16	17.4714	81.0000	939.7592	1.9184	1.3500
63	2016	16	17.7429	87.5000	945.1398	2.4031	1.1969
62	2016	16	14.5429	81.1735	874.1102	2.7194	1.1816
1	2016	16	14.7857	78.6939	874.3082	3.5684	2.8092
31	2016	17	14.8857	82.0510	846.7449	2.5408	1.0612
79	2016	17	19.3286	82.9082	970.5469	1.6490	1.1510
51	2016	17	17.6571	86.0816	939.7551	2.3051	1.3347
14	2016	17	16.6571	84.6020	897.5714	1.9582	1.9990
67	2016	17	16.3857	86.2755	902.5051	1.9224	2.3847
42	2016	17	16.0714	81.2041	875.1541	2.5704	2.6316
50	2016	17	16.7714	80.9286	902.6337	1.9949	1.5582
43	2016	17	16.0714	81.2041	875.1541	2.5704	2.6316
85	2016	17	17.4429	81.2143	911.1306	2.6510	1.4112
25	2016	17	19.9857	87.5510	977.3204	1.9704	0.7245
69	2016	17	18.5143	81.9490	940.5786	1.7194	1.1929
57	2016	17	16.1714	86.3367	887.2551	2.7408	2.4490
9	2016	17	15.4714	80.7551	854.3286	4.0388	2.4143
72	2016	17	17.0571	81.1735	877.7847	3.9347	1.8837
26	2016	17	18.7000	73.6837	866.8724	4.5592	2.1837
7	2016	17	17.5143	76.1429	859.2327	4.5816	2.0245
83	2016	17	21.3000	76.4490	944.4051	4.9163	1.2408
76	2016	17	17.4143	84.3367	921.7847	1.7357	1.5582
36	2016	17	17.9571	84.2143	929.3153	2.4061	1.4510
81	2016	17	17.6571	86.0816	939.7551	2.3051	1.3347
15	2016	17	18.0286	84.2653	933.8857	1.4898	0.7378
32	2016	17	16.0714	81.2041	875.1541	2.5704	2.6316
73	2016	17	19.5143	83.6735	962.8092	2.0102	0.9153
71	2016	17	17.9571	84.2143	929.3153	2.4061	1.4510
41	2016	17	16.3000	82.8571	874.9929	2.3520	1.0337

10	2016	17	18.4571	85.6020	964.7714	2.0378	0.7633
23	2016	17	12.7571	78.5612	775.9082	4.2276	2.2612
27	2016	17	17.5143	76.1429	859.2327	4.5816	2.0245
60	2016	17	17.6571	86.0816	939.7551	2.3051	1.3347
53	2016	17	15.4714	80.7551	854.3286	4.0388	2.4143
66	2016	17	16.6571	84.6020	897.5714	1.9582	1.9990
59	2016	17	16.1714	86.3367	887.2551	2.7408	2.4490
61	2016	17	19.5143	83.6735	962.8092	2.0102	0.9153
84	2016	17	19.5143	83.6735	962.8092	2.0102	0.9153
38	2016	17	16.1714	86.3367	887.2551	2.7408	2.4490
87	2016	17	17.7429	77.9388	899.6888	3.0704	2.1041
34	2016	17	16.1714	86.3367	887.2551	2.7408	2.4490
29	2016	17	18.5143	81.9490	940.5786	1.7194	1.1929
5	2016	17	15.2571	78.8163	832.2418	4.8745	2.0878
8	2016	17	15.4714	80.7551	854.3286	4.0388	2.4143
12	2016	17	15.2571	78.8163	832.2418	4.8745	2.0878
13	2016	17	21.3000	76.4490	944.4051	4.9163	1.2408
18	2016	17	19.4857	83.1429	964.8133	1.6082	0.9439
33	2016	17	16.7714	80.9286	902.6337	1.9949	1.5582
56	2016	17	19.9857	87.5510	977.3204	1.9704	0.7245
77	2016	17	18.0286	84.2653	933.8857	1.4898	0.7378
54	2016	17	15.2571	78.8163	832.2418	4.8745	2.0878
21	2016	17	16.7714	80.9286	902.6337	1.9949	1.5582
68	2016	17	19.3286	82.9082	970.5469	1.6490	1.1510
74	2016	17	19.5143	83.6735	962.8092	2.0102	0.9153
88	2016	17	16.0714	81.2041	875.1541	2.5704	2.6316
16	2016	17	17.4143	84.3367	921.7847	1.7357	1.5582
30	2016	17	16.6571	84.6020	897.5714	1.9582	1.9990
6	2016	17	19.3286	82.9082	970.5469	1.6490	1.1510
49	2016	17	18.5143	81.9490	940.5786	1.7194	1.1929
22	2016	17	16.0714	81.2041	875.1541	2.5704	2.6316
45	2016	17	16.5286	68.7143	817.3041	5.3378	2.1622
58	2016	17	18.5143	81.9490	940.5786	1.7194	1.1929
37	2016	17	19.3286	82.9082	970.5469	1.6490	1.1510
17	2016	17	16.3857	86.2755	902.5051	1.9224	2.3847
55	2016	17	17.0571	81.1735	877.7847	3.9347	1.8837
46	2016	17	17.4143	84.3367	921.7847	1.7357	1.5582
86	2016	17	16.3429	78.1224	866.4602	2.8429	1.1929
2	2016	17	16.3429	78.1224	866.4602	2.8429	1.1929
4	2016	17	16.7714	80.9286	902.6337	1.9949	1.5582
47	2016	17	21.0857	78.1735	958.7959	3.0776	0.5684
82	2016	17	16.0714	81.2041	875.1541	2.5704	2.6316
19	2016	17	20.2286	84.1020	960.6286	2.0755	0.9592
20	2016	17	15.4714	80.7551	854.3286	4.0388	2.4143
80	2016	17	16.0714	81.2041	875.1541	2.5704	2.6316
3	2016	17	21.3000	76.4490	944.4051	4.9163	1.2408
52	2016	17	16.3857	86.2755	902.5051	1.9224	2.3847
70	2016	17	17.4429	81.2143	911.1306	2.6510	1.4112
64	2016	17	12.7571	78.5612	775.9082	4.2276	2.2612
48	2016	17	18.0286	84.2653	933.8857	1.4898	0.7378

65	2016	17	16.3857	86.2755	902.5051	1.9224	2.3847
44	2016	17	17.4429	81.2143	911.1306	2.6510	1.4112
75	2016	17	12.7571	78.5612	775.9082	4.2276	2.2612
40	2016	17	17.9286	87.4796	945.8765	2.2418	1.2592
11	2016	17	17.0571	81.1735	877.7847	3.9347	1.8837
35	2016	17	17.6571	86.0816	939.7551	2.3051	1.3347
78	2016	17	17.7429	77.9388	899.6888	3.0704	2.1041
28	2016	17	17.9571	84.2143	929.3153	2.4061	1.4510
39	2016	17	16.3857	86.2755	902.5051	1.9224	2.3847
24	2016	17	18.5143	81.9490	940.5786	1.7194	1.1929
63	2016	17	17.9286	87.4796	945.8765	2.2418	1.2592
62	2016	17	16.3000	82.8571	874.9929	2.3520	1.0337
1	2016	17	16.0714	81.2041	875.1541	2.5704	2.6316
31	2016	18	19.2286	84.3980	846.3980	2.7551	0.8878
79	2016	18	22.4571	80.9592	969.4500	2.3265	1.2061
51	2016	18	21.1571	86.7449	939.0224	2.7673	1.2541
14	2016	18	21.8143	88.0714	897.3214	1.3459	1.6541
67	2016	18	21.8571	91.1531	902.1092	1.8959	2.3888
42	2016	18	21.8429	84.2143	874.7959	2.6878	2.2714
50	2016	18	21.7429	81.6633	901.8429	2.1541	1.5378
43	2016	18	21.8429	84.2143	874.7959	2.6878	2.2714
85	2016	18	22.9714	80.8776	910.2653	2.8653	1.4837
25	2016	18	24.0000	89.9184	976.7327	1.6245	0.6571
69	2016	18	23.1571	82.6633	939.7051	1.8265	1.0867
57	2016	18	21.7571	87.4184	886.6582	3.1480	2.1990
9	2016	18	21.1143	84.5714	854.0908	4.1112	2.2143
72	2016	18	22.2714	86.1837	877.4816	3.5949	1.6184
26	2016	18	24.0857	76.0306	866.6010	4.5276	2.0031
7	2016	18	22.9571	78.1735	858.9653	4.9194	1.7867
83	2016	18	26.7000	80.6735	943.7949	4.9153	1.1071
76	2016	18	22.2000	83.2857	920.9204	2.6163	1.4561
36	2016	18	22.5143	86.0000	928.6980	2.5245	1.3122
81	2016	18	21.1571	86.7449	939.0224	2.7673	1.2541
15	2016	18	22.2571	84.4388	932.8980	1.5388	0.6449
32	2016	18	21.8429	84.2143	874.7959	2.6878	2.2714
73	2016	18	23.1000	82.3265	961.6990	2.4347	0.8847
71	2016	18	22.5143	86.0000	928.6980	2.5245	1.3122
41	2016	18	21.1286	85.3673	874.4622	2.6816	0.8469
10	2016	18	22.2000	85.2449	963.9622	2.2949	0.7653
23	2016	18	17.5286	78.7041	775.9520	5.0051	2.0888
27	2016	18	22.9571	78.1735	858.9653	4.9194	1.7867
60	2016	18	21.1571	86.7449	939.0224	2.7673	1.2541
53	2016	18	21.1143	84.5714	854.0908	4.1112	2.2143
66	2016	18	21.8143	88.0714	897.3214	1.3459	1.6541
59	2016	18	21.7571	87.4184	886.6582	3.1480	2.1990
61	2016	18	23.1000	82.3265	961.6990	2.4347	0.8847
84	2016	18	23.1000	82.3265	961.6990	2.4347	0.8847
38	2016	18	21.7571	87.4184	886.6582	3.1480	2.1990
87	2016	18	23.2429	81.8265	899.2327	3.1551	1.7602
34	2016	18	21.7571	87.4184	886.6582	3.1480	2.1990

29	2016	18	23.1571	82.6633	939.7051	1.8265	1.0867
5	2016	18	20.6143	82.5204	832.1276	4.8480	1.7755
8	2016	18	21.1143	84.5714	854.0908	4.1112	2.2143
12	2016	18	20.6143	82.5204	832.1276	4.8480	1.7755
13	2016	18	26.7000	80.6735	943.7949	4.9153	1.1071
18	2016	18	22.4000	79.4898	963.7316	2.7704	1.1173
33	2016	18	21.7429	81.6633	901.8429	2.1541	1.5378
56	2016	18	24.0000	89.9184	976.7327	1.6245	0.6571
77	2016	18	22.2571	84.4388	932.8980	1.5388	0.6449
54	2016	18	20.6143	82.5204	832.1276	4.8480	1.7755
21	2016	18	21.7429	81.6633	901.8429	2.1541	1.5378
68	2016	18	22.4571	80.9592	969.4500	2.3265	1.2061
74	2016	18	23.1000	82.3265	961.6990	2.4347	0.8847
88	2016	18	21.8429	84.2143	874.7959	2.6878	2.2714
16	2016	18	22.2000	83.2857	920.9204	2.6163	1.4561
30	2016	18	21.8143	88.0714	897.3214	1.3459	1.6541
6	2016	18	22.4571	80.9592	969.4500	2.3265	1.2061
49	2016	18	23.1571	82.6633	939.7051	1.8265	1.0867
22	2016	18	21.8429	84.2143	874.7959	2.6878	2.2714
45	2016	18	20.9857	70.8367	817.1847	5.7214	1.7500
58	2016	18	23.1571	82.6633	939.7051	1.8265	1.0867
37	2016	18	22.4571	80.9592	969.4500	2.3265	1.2061
17	2016	18	21.8571	91.1531	902.1092	1.8959	2.3888
55	2016	18	22.2714	86.1837	877.4816	3.5949	1.6184
46	2016	18	22.2000	83.2857	920.9204	2.6163	1.4561
86	2016	18	21.6143	80.8571	866.0265	3.2184	1.0439
2	2016	18	21.6143	80.8571	866.0265	3.2184	1.0439
4	2016	18	21.7429	81.6633	901.8429	2.1541	1.5378
47	2016	18	25.7714	81.7449	958.1653	2.7500	0.4663
82	2016	18	21.8429	84.2143	874.7959	2.6878	2.2714
19	2016	18	24.6857	88.5510	960.1990	1.6592	0.9663
20	2016	18	21.1143	84.5714	854.0908	4.1112	2.2143
80	2016	18	21.8429	84.2143	874.7959	2.6878	2.2714
3	2016	18	26.7000	80.6735	943.7949	4.9153	1.1071
52	2016	18	21.8571	91.1531	902.1092	1.8959	2.3888
70	2016	18	22.9714	80.8776	910.2653	2.8653	1.4837
64	2016	18	17.5286	78.7041	775.9520	5.0051	2.0888
48	2016	18	22.2571	84.4388	932.8980	1.5388	0.6449
65	2016	18	21.8571	91.1531	902.1092	1.8959	2.3888
44	2016	18	22.9714	80.8776	910.2653	2.8653	1.4837
75	2016	18	17.5286	78.7041	775.9520	5.0051	2.0888
40	2016	18	22.2571	87.3469	945.3490	2.5582	1.2276
11	2016	18	22.2714	86.1837	877.4816	3.5949	1.6184
35	2016	18	21.1571	86.7449	939.0224	2.7673	1.2541
78	2016	18	23.2429	81.8265	899.2327	3.1551	1.7602
28	2016	18	22.5143	86.0000	928.6980	2.5245	1.3122
39	2016	18	21.8571	91.1531	902.1092	1.8959	2.3888
24	2016	18	23.1571	82.6633	939.7051	1.8265	1.0867
63	2016	18	22.2571	87.3469	945.3490	2.5582	1.2276
62	2016	18	21.1286	85.3673	874.4622	2.6816	0.8469

1	2016	18	21.8429	84.2143	874.7959	2.6878	2.2714
31	2016	19	18.2143	77.2449	845.9235	4.9908	1.1143
79	2016	19	20.2857	78.6735	968.8296	3.5806	1.4908
51	2016	19	19.5143	84.1837	938.4469	3.8551	1.4082
14	2016	19	19.2857	84.8469	896.9643	2.9653	2.2337
67	2016	19	18.7714	87.0918	901.6051	3.4378	2.8571
42	2016	19	18.8429	78.2041	874.3816	4.7918	2.4857
50	2016	19	20.1571	76.1327	900.9847	4.1398	1.9388
43	2016	19	18.8429	78.2041	874.3816	4.7918	2.4857
85	2016	19	21.3143	72.7245	909.1714	5.1102	1.8704
25	2016	19	22.0143	86.6837	975.9776	2.8449	0.9286
69	2016	19	20.6571	80.1429	938.8918	3.2276	1.3031
57	2016	19	19.6429	80.4286	885.9694	5.4694	2.5929
9	2016	19	18.6571	78.1633	853.8214	6.6265	2.4449
72	2016	19	20.0286	81.0408	877.1122	5.7878	1.6816
26	2016	19	21.7429	67.7143	866.2908	7.3429	2.4684
7	2016	19	20.6571	70.8980	858.7194	7.5541	2.1143
83	2016	19	24.6571	72.9184	942.8367	7.1714	1.3561
76	2016	19	19.9143	78.6837	920.1316	4.5439	1.5531
36	2016	19	20.0857	82.9490	928.0602	4.0714	1.4663
81	2016	19	19.5143	84.1837	938.4469	3.8551	1.4082
15	2016	19	21.1571	79.6939	931.8265	3.2459	0.8367
32	2016	19	18.8429	78.2041	874.3816	4.7918	2.4857
73	2016	19	21.6000	79.3265	960.7582	3.6347	0.9796
71	2016	19	20.0857	82.9490	928.0602	4.0714	1.4663
41	2016	19	20.2286	77.6429	873.8347	5.1439	1.2918
10	2016	19	20.3429	82.6837	963.3510	3.2255	1.0082
23	2016	19	14.8000	70.9592	776.3500	6.7133	2.1867
27	2016	19	20.6571	70.8980	858.7194	7.5541	2.1143
60	2016	19	19.5143	84.1837	938.4469	3.8551	1.4082
53	2016	19	18.6571	78.1633	853.8214	6.6265	2.4449
66	2016	19	19.2857	84.8469	896.9643	2.9653	2.2337
59	2016	19	19.6429	80.4286	885.9694	5.4694	2.5929
61	2016	19	21.6000	79.3265	960.7582	3.6347	0.9796
84	2016	19	21.6000	79.3265	960.7582	3.6347	0.9796
38	2016	19	19.6429	80.4286	885.9694	5.4694	2.5929
87	2016	19	20.4571	76.9490	898.6367	5.4755	1.7908
34	2016	19	19.6429	80.4286	885.9694	5.4694	2.5929
29	2016	19	20.6571	80.1429	938.8918	3.2276	1.3031
5	2016	19	18.9143	73.1327	832.1000	7.7888	2.0459
8	2016	19	18.6571	78.1633	853.8214	6.6265	2.4449
12	2016	19	18.9143	73.1327	832.1000	7.7888	2.0459
13	2016	19	24.6571	72.9184	942.8367	7.1714	1.3561
18	2016	19	20.5429	77.6735	963.0724	4.1184	1.2367
33	2016	19	20.1571	76.1327	900.9847	4.1398	1.9388
56	2016	19	22.0143	86.6837	975.9776	2.8449	0.9286
77	2016	19	21.1571	79.6939	931.8265	3.2459	0.8367
54	2016	19	18.9143	73.1327	832.1000	7.7888	2.0459
21	2016	19	20.1571	76.1327	900.9847	4.1398	1.9388
68	2016	19	20.2857	78.6735	968.8296	3.5806	1.4908

74	2016	19	21.6000	79.3265	960.7582	3.6347	0.9796
88	2016	19	18.8429	78.2041	874.3816	4.7918	2.4857
16	2016	19	19.9143	78.6837	920.1316	4.5439	1.5531
30	2016	19	19.2857	84.8469	896.9643	2.9653	2.2337
6	2016	19	20.2857	78.6735	968.8296	3.5806	1.4908
49	2016	19	20.6571	80.1429	938.8918	3.2276	1.3031
22	2016	19	18.8429	78.2041	874.3816	4.7918	2.4857
45	2016	19	19.2429	60.9796	817.3327	8.0112	1.8867
58	2016	19	20.6571	80.1429	938.8918	3.2276	1.3031
37	2016	19	20.2857	78.6735	968.8296	3.5806	1.4908
17	2016	19	18.7714	87.0918	901.6051	3.4378	2.8571
55	2016	19	20.0286	81.0408	877.1122	5.7878	1.6816
46	2016	19	19.9143	78.6837	920.1316	4.5439	1.5531
86	2016	19	19.7143	74.2143	865.5071	5.6388	1.5041
2	2016	19	19.7143	74.2143	865.5071	5.6388	1.5041
4	2016	19	20.1571	76.1327	900.9847	4.1398	1.9388
47	2016	19	23.2000	79.2653	957.1500	4.5092	0.4806
82	2016	19	18.8429	78.2041	874.3816	4.7918	2.4857
19	2016	19	22.3000	86.5204	959.4969	2.6643	0.9980
20	2016	19	18.6571	78.1633	853.8214	6.6265	2.4449
80	2016	19	18.8429	78.2041	874.3816	4.7918	2.4857
3	2016	19	24.6571	72.9184	942.8367	7.1714	1.3561
52	2016	19	18.7714	87.0918	901.6051	3.4378	2.8571
70	2016	19	21.3143	72.7245	909.1714	5.1102	1.8704
64	2016	19	14.8000	70.9592	776.3500	6.7133	2.1867
48	2016	19	21.1571	79.6939	931.8265	3.2459	0.8367
65	2016	19	18.7714	87.0918	901.6051	3.4378	2.8571
44	2016	19	21.3143	72.7245	909.1714	5.1102	1.8704
75	2016	19	14.8000	70.9592	776.3500	6.7133	2.1867
40	2016	19	20.1143	84.1735	944.8633	4.1592	1.3571
11	2016	19	20.0286	81.0408	877.1122	5.7878	1.6816
35	2016	19	19.5143	84.1837	938.4469	3.8551	1.4082
78	2016	19	20.4571	76.9490	898.6367	5.4755	1.7908
28	2016	19	20.0857	82.9490	928.0602	4.0714	1.4663
39	2016	19	18.7714	87.0918	901.6051	3.4378	2.8571
24	2016	19	20.6571	80.1429	938.8918	3.2276	1.3031
63	2016	19	20.1143	84.1735	944.8633	4.1592	1.3571
62	2016	19	20.2286	77.6429	873.8347	5.1439	1.2918
1	2016	19	18.8429	78.2041	874.3816	4.7918	2.4857
31	2016	20	16.7571	72.5612	846.9888	4.8347	1.2520
79	2016	20	18.5857	78.0816	970.6571	3.3735	1.5041
51	2016	20	17.2571	82.1531	940.0163	3.4898	1.4673
14	2016	20	18.5143	80.6735	898.1112	3.0867	2.5327
67	2016	20	17.5857	83.9286	902.8724	3.0031	2.8459
42	2016	20	17.6857	74.6633	875.5245	4.2663	2.8714
50	2016	20	17.8143	72.7959	902.3388	4.5071	2.1969
43	2016	20	17.6857	74.6633	875.5245	4.2663	2.8714
85	2016	20	19.3286	68.7959	910.5908	5.0633	2.0286
25	2016	20	21.2571	82.8061	977.4133	3.4173	1.0296
69	2016	20	18.2714	79.0408	940.5296	3.0949	1.4510

57	2016	20	17.2714	77.2245	887.2510	4.7449	2.8857
9	2016	20	17.8286	73.7041	854.8408	6.6214	2.6929
72	2016	20	19.0571	76.3878	878.2041	5.7745	1.8347
26	2016	20	19.8000	66.9082	867.2827	7.8255	2.4071
7	2016	20	18.9714	69.1531	859.7194	7.2796	2.3347
83	2016	20	23.5571	69.4388	944.1816	6.5969	1.3592
76	2016	20	17.6857	76.4286	921.6296	4.1122	1.5214
36	2016	20	18.1000	81.4490	929.5561	3.9663	1.5480
81	2016	20	17.2571	82.1531	940.0163	3.4898	1.4673
15	2016	20	18.8286	75.8061	933.3194	3.9622	0.9286
32	2016	20	17.6857	74.6633	875.5245	4.2663	2.8714
73	2016	20	19.4000	77.7653	962.4531	3.5429	1.0755
71	2016	20	18.1000	81.4490	929.5561	3.9663	1.5480
41	2016	20	18.0571	72.1122	875.0122	5.1643	1.6204
10	2016	20	18.4143	80.3673	964.9490	3.1612	0.9918
23	2016	20	14.1286	69.9388	777.1878	5.6480	2.3837
27	2016	20	18.9714	69.1531	859.7194	7.2796	2.3347
60	2016	20	17.2571	82.1531	940.0163	3.4898	1.4673
53	2016	20	17.8286	73.7041	854.8408	6.6214	2.6929
66	2016	20	18.5143	80.6735	898.1112	3.0867	2.5327
59	2016	20	17.2714	77.2245	887.2510	4.7449	2.8857
61	2016	20	19.4000	77.7653	962.4531	3.5429	1.0755
84	2016	20	19.4000	77.7653	962.4531	3.5429	1.0755
38	2016	20	17.2714	77.2245	887.2510	4.7449	2.8857
87	2016	20	20.0571	72.5204	899.8316	5.1235	2.0939
34	2016	20	17.2714	77.2245	887.2510	4.7449	2.8857
29	2016	20	18.2714	79.0408	940.5296	3.0949	1.4510
5	2016	20	17.0571	69.9898	833.0133	7.7031	2.2582
8	2016	20	17.8286	73.7041	854.8408	6.6214	2.6929
12	2016	20	17.0571	69.9898	833.0133	7.7031	2.2582
13	2016	20	23.5571	69.4388	944.1816	6.5969	1.3592
18	2016	20	18.6000	78.6837	964.8551	3.5908	1.1133
33	2016	20	17.8143	72.7959	902.3388	4.5071	2.1969
56	2016	20	21.2571	82.8061	977.4133	3.4173	1.0296
77	2016	20	18.8286	75.8061	933.3194	3.9622	0.9286
54	2016	20	17.0571	69.9898	833.0133	7.7031	2.2582
21	2016	20	17.8143	72.7959	902.3388	4.5071	2.1969
68	2016	20	18.5857	78.0816	970.6571	3.3735	1.5041
74	2016	20	19.4000	77.7653	962.4531	3.5429	1.0755
88	2016	20	17.6857	74.6633	875.5245	4.2663	2.8714
16	2016	20	17.6857	76.4286	921.6296	4.1122	1.5214
30	2016	20	18.5143	80.6735	898.1112	3.0867	2.5327
6	2016	20	18.5857	78.0816	970.6571	3.3735	1.5041
49	2016	20	18.2714	79.0408	940.5296	3.0949	1.4510
22	2016	20	17.6857	74.6633	875.5245	4.2663	2.8714
45	2016	20	17.7000	60.6327	818.1827	7.1510	2.0653
58	2016	20	18.2714	79.0408	940.5296	3.0949	1.4510
37	2016	20	18.5857	78.0816	970.6571	3.3735	1.5041
17	2016	20	17.5857	83.9286	902.8724	3.0031	2.8459
55	2016	20	19.0571	76.3878	878.2041	5.7745	1.8347

46	2016	20	17.6857	76.4286	921.6296	4.1122	1.5214
86	2016	20	18.1429	70.4286	866.6520	5.7735	1.6704
2	2016	20	18.1429	70.4286	866.6520	5.7735	1.6704
4	2016	20	17.8143	72.7959	902.3388	4.5071	2.1969
47	2016	20	23.2000	76.3061	958.5582	4.5735	0.6122
82	2016	20	17.6857	74.6633	875.5245	4.2663	2.8714
19	2016	20	22.3000	81.9388	960.8276	3.2918	1.0816
20	2016	20	17.8286	73.7041	854.8408	6.6214	2.6929
80	2016	20	17.6857	74.6633	875.5245	4.2663	2.8714
3	2016	20	23.5571	69.4388	944.1816	6.5969	1.3592
52	2016	20	17.5857	83.9286	902.8724	3.0031	2.8459
70	2016	20	19.3286	68.7959	910.5908	5.0633	2.0286
64	2016	20	14.1286	69.9388	777.1878	5.6480	2.3837
48	2016	20	18.8286	75.8061	933.3194	3.9622	0.9286
65	2016	20	17.5857	83.9286	902.8724	3.0031	2.8459
44	2016	20	19.3286	68.7959	910.5908	5.0633	2.0286
75	2016	20	14.1286	69.9388	777.1878	5.6480	2.3837
40	2016	20	17.9143	82.2857	946.2031	3.9194	1.4439
11	2016	20	19.0571	76.3878	878.2041	5.7745	1.8347
35	2016	20	17.2571	82.1531	940.0163	3.4898	1.4673
78	2016	20	20.0571	72.5204	899.8316	5.1235	2.0939
28	2016	20	18.1000	81.4490	929.5561	3.9663	1.5480
39	2016	20	17.5857	83.9286	902.8724	3.0031	2.8459
24	2016	20	18.2714	79.0408	940.5296	3.0949	1.4510
63	2016	20	17.9143	82.2857	946.2031	3.9194	1.4439
62	2016	20	18.0571	72.1122	875.0122	5.1643	1.6204
1	2016	20	17.6857	74.6633	875.5245	4.2663	2.8714
31	2016	21	16.1571	78.8673	847.1255	2.7704	1.1561
79	2016	21	18.8714	81.9286	971.1602	1.5990	1.0286
51	2016	21	18.1286	84.4184	940.4153	1.5745	1.4122
14	2016	21	18.4571	83.1429	898.2602	1.7071	2.1224
67	2016	21	17.7857	88.7449	903.1224	1.1602	2.5806
42	2016	21	16.5429	81.0816	875.7418	2.0724	2.7908
50	2016	21	17.4000	77.9184	902.7541	2.3643	1.8622
43	2016	21	16.5429	81.0816	875.7418	2.0724	2.7908
85	2016	21	18.2143	73.7143	911.1092	3.0847	1.7990
25	2016	21	21.5571	85.1735	977.7724	2.4214	0.8122
69	2016	21	19.3000	82.3980	941.0510	1.2041	1.1867
57	2016	21	16.7286	83.8265	887.6276	2.3500	2.4439
9	2016	21	16.7714	80.6837	854.9459	3.5153	2.4847
72	2016	21	18.5286	81.8776	878.3898	3.3949	1.6765
26	2016	21	20.1571	78.7653	867.5367	4.5286	1.9357
7	2016	21	19.8429	78.3367	859.8735	4.0398	2.0010
83	2016	21	22.9714	78.1633	944.6969	3.3469	1.0286
76	2016	21	18.0857	81.1429	922.0990	1.7071	1.3929
36	2016	21	18.8857	85.4490	929.9735	1.8745	1.4224
81	2016	21	18.1286	84.4184	940.4153	1.5745	1.4122
15	2016	21	19.2143	79.2551	933.9122	2.3561	0.8408
32	2016	21	16.5429	81.0816	875.7418	2.0724	2.7908
73	2016	21	19.9714	81.0306	963.0520	1.8898	0.9827

71	2016	21	18.8857	85.4490	929.9735	1.8745	1.4224
41	2016	21	17.4143	78.6224	875.2837	2.8378	1.5633
10	2016	21	18.9857	83.7143	965.3673	1.5724	0.7245
23	2016	21	14.2857	79.0204	776.9602	3.4786	2.2898
27	2016	21	19.8429	78.3367	859.8735	4.0398	2.0010
60	2016	21	18.1286	84.4184	940.4153	1.5745	1.4122
53	2016	21	16.7714	80.6837	854.9459	3.5153	2.4847
66	2016	21	18.4571	83.1429	898.2602	1.7071	2.1224
59	2016	21	16.7286	83.8265	887.6276	2.3500	2.4439
61	2016	21	19.9714	81.0306	963.0520	1.8898	0.9827
84	2016	21	19.9714	81.0306	963.0520	1.8898	0.9827
38	2016	21	16.7286	83.8265	887.6276	2.3500	2.4439
87	2016	21	18.6000	76.6939	900.1051	3.0082	1.9806
34	2016	21	16.7286	83.8265	887.6276	2.3500	2.4439
29	2016	21	19.3000	82.3980	941.0510	1.2041	1.1867
5	2016	21	17.6286	80.3367	833.0520	4.0520	1.7827
8	2016	21	16.7714	80.6837	854.9459	3.5153	2.4847
12	2016	21	17.6286	80.3367	833.0520	4.0520	1.7827
13	2016	21	22.9714	78.1633	944.6969	3.3469	1.0286
18	2016	21	19.1000	81.8980	965.3643	1.7439	0.8020
33	2016	21	17.4000	77.9184	902.7541	2.3643	1.8622
56	2016	21	21.5571	85.1735	977.7724	2.4214	0.8122
77	2016	21	19.2143	79.2551	933.9122	2.3561	0.8408
54	2016	21	17.6286	80.3367	833.0520	4.0520	1.7827
21	2016	21	17.4000	77.9184	902.7541	2.3643	1.8622
68	2016	21	18.8714	81.9286	971.1602	1.5990	1.0286
74	2016	21	19.9714	81.0306	963.0520	1.8898	0.9827
88	2016	21	16.5429	81.0816	875.7418	2.0724	2.7908
16	2016	21	18.0857	81.1429	922.0990	1.7071	1.3929
30	2016	21	18.4571	83.1429	898.2602	1.7071	2.1224
6	2016	21	18.8714	81.9286	971.1602	1.5990	1.0286
49	2016	21	19.3000	82.3980	941.0510	1.2041	1.1867
22	2016	21	16.5429	81.0816	875.7418	2.0724	2.7908
45	2016	21	18.5714	73.6735	818.1184	3.7408	2.0000
58	2016	21	19.3000	82.3980	941.0510	1.2041	1.1867
37	2016	21	18.8714	81.9286	971.1602	1.5990	1.0286
17	2016	21	17.7857	88.7449	903.1224	1.1602	2.5806
55	2016	21	18.5286	81.8776	878.3898	3.3949	1.6765
46	2016	21	18.0857	81.1429	922.0990	1.7071	1.3929
86	2016	21	17.1571	77.5714	866.8949	3.3041	1.3653
2	2016	21	17.1571	77.5714	866.8949	3.3041	1.3653
4	2016	21	17.4000	77.9184	902.7541	2.3643	1.8622
47	2016	21	21.9143	78.7245	959.0480	2.9796	0.5827
82	2016	21	16.5429	81.0816	875.7418	2.0724	2.7908
19	2016	21	22.1857	82.9490	961.1224	2.5939	1.0806
20	2016	21	16.7714	80.6837	854.9459	3.5153	2.4847
80	2016	21	16.5429	81.0816	875.7418	2.0724	2.7908
3	2016	21	22.9714	78.1633	944.6969	3.3469	1.0286
52	2016	21	17.7857	88.7449	903.1224	1.1602	2.5806
70	2016	21	18.2143	73.7143	911.1092	3.0847	1.7990

64	2016	21	14.2857	79.0204	776.9602	3.4786	2.2898
48	2016	21	19.2143	79.2551	933.9122	2.3561	0.8408
65	2016	21	17.7857	88.7449	903.1224	1.1602	2.5806
44	2016	21	18.2143	73.7143	911.1092	3.0847	1.7990
75	2016	21	14.2857	79.0204	776.9602	3.4786	2.2898
40	2016	21	18.7000	85.9286	946.4592	1.7531	1.4102
11	2016	21	18.5286	81.8776	878.3898	3.3949	1.6765
35	2016	21	18.1286	84.4184	940.4153	1.5745	1.4122
78	2016	21	18.6000	76.6939	900.1051	3.0082	1.9806
28	2016	21	18.8857	85.4490	929.9735	1.8745	1.4224
39	2016	21	17.7857	88.7449	903.1224	1.1602	2.5806
24	2016	21	19.3000	82.3980	941.0510	1.2041	1.1867
63	2016	21	18.7000	85.9286	946.4592	1.7531	1.4102
62	2016	21	17.4143	78.6224	875.2837	2.8378	1.5633
1	2016	21	16.5429	81.0816	875.7418	2.0724	2.7908
31	2016	22	22.2571	81.5102	845.2357	3.6704	1.0745
79	2016	22	24.5143	84.0816	968.2959	1.4327	1.0173
51	2016	22	24.2286	84.4082	937.8296	1.9122	1.5092
14	2016	22	23.4429	86.6735	896.2296	1.6296	2.0224
67	2016	22	23.3286	91.7143	900.9837	1.1418	3.0510
42	2016	22	22.9286	85.6531	873.8490	2.7378	2.5633
50	2016	22	24.2571	81.5102	900.3735	2.0347	1.7602
43	2016	22	22.9286	85.6531	873.8490	2.7378	2.5633
85	2016	22	25.3714	76.4796	908.7061	4.0592	1.8918
25	2016	22	26.5143	87.5714	975.0133	2.2643	0.7429
69	2016	22	25.7714	83.5816	938.3541	1.4857	1.1296
57	2016	22	23.8000	86.8980	885.5153	3.4490	2.5214
9	2016	22	22.0857	86.4082	853.1990	3.4592	2.6214
72	2016	22	23.5857	86.0306	876.5612	3.6704	1.8388
26	2016	22	24.0714	82.5918	865.9082	4.4714	2.0480
7	2016	22	23.4429	81.0918	858.2143	4.2286	2.2418
83	2016	22	27.5571	82.2959	942.3898	3.6480	1.0245
76	2016	22	24.7571	84.4490	919.5102	2.2714	1.4020
36	2016	22	25.1143	85.9898	927.5337	1.9010	1.5459
81	2016	22	24.2286	84.4082	937.8296	1.9122	1.5092
15	2016	22	25.1286	81.9796	931.1745	2.2847	0.7765
32	2016	22	22.9286	85.6531	873.8490	2.7378	2.5633
73	2016	22	26.3714	82.5306	960.0939	1.6306	0.9745
71	2016	22	25.1143	85.9898	927.5337	1.9010	1.5459
41	2016	22	24.2000	82.8878	873.2551	3.3643	1.5306
10	2016	22	24.6143	86.1837	962.6908	1.1439	0.7378
23	2016	22	18.9714	82.2857	775.5857	4.9949	2.3408
27	2016	22	23.4429	81.0918	858.2143	4.2286	2.2418
60	2016	22	24.2286	84.4082	937.8296	1.9122	1.5092
53	2016	22	22.0857	86.4082	853.1990	3.4592	2.6214
66	2016	22	23.4429	86.6735	896.2296	1.6296	2.0224
59	2016	22	23.8000	86.8980	885.5153	3.4490	2.5214
61	2016	22	26.3714	82.5306	960.0939	1.6306	0.9745
84	2016	22	26.3714	82.5306	960.0939	1.6306	0.9745
38	2016	22	23.8000	86.8980	885.5153	3.4490	2.5214

87	2016	22	24.6857	81.3367	898.0867	3.5306	1.9378
34	2016	22	23.8000	86.8980	885.5153	3.4490	2.5214
29	2016	22	25.7714	83.5816	938.3541	1.4857	1.1296
5	2016	22	21.9429	84.0306	831.5082	3.9898	1.7898
8	2016	22	22.0857	86.4082	853.1990	3.4592	2.6214
12	2016	22	21.9429	84.0306	831.5082	3.9898	1.7898
13	2016	22	27.5571	82.2959	942.3898	3.6480	1.0245
18	2016	22	24.4857	83.8061	962.4969	1.1306	0.7643
33	2016	22	24.2571	81.5102	900.3735	2.0347	1.7602
56	2016	22	26.5143	87.5714	975.0133	2.2643	0.7429
77	2016	22	25.1286	81.9796	931.1745	2.2847	0.7765
54	2016	22	21.9429	84.0306	831.5082	3.9898	1.7898
21	2016	22	24.2571	81.5102	900.3735	2.0347	1.7602
68	2016	22	24.5143	84.0816	968.2959	1.4327	1.0173
74	2016	22	26.3714	82.5306	960.0939	1.6306	0.9745
88	2016	22	22.9286	85.6531	873.8490	2.7378	2.5633
16	2016	22	24.7571	84.4490	919.5102	2.2714	1.4020
30	2016	22	23.4429	86.6735	896.2296	1.6296	2.0224
6	2016	22	24.5143	84.0816	968.2959	1.4327	1.0173
49	2016	22	25.7714	83.5816	938.3541	1.4857	1.1296
22	2016	22	22.9286	85.6531	873.8490	2.7378	2.5633
45	2016	22	21.6143	78.3367	816.7224	3.9245	2.3224
58	2016	22	25.7714	83.5816	938.3541	1.4857	1.1296
37	2016	22	24.5143	84.0816	968.2959	1.4327	1.0173
17	2016	22	23.3286	91.7143	900.9837	1.1418	3.0510
55	2016	22	23.5857	86.0306	876.5612	3.6704	1.8388
46	2016	22	24.7571	84.4490	919.5102	2.2714	1.4020
86	2016	22	23.4857	81.4592	864.9857	3.4378	1.5480
2	2016	22	23.4857	81.4592	864.9857	3.4378	1.5480
4	2016	22	24.2571	81.5102	900.3735	2.0347	1.7602
47	2016	22	26.4714	81.3163	956.5245	3.2071	0.6255
82	2016	22	22.9286	85.6531	873.8490	2.7378	2.5633
19	2016	22	27.0857	86.3673	958.5663	2.8000	1.0929
20	2016	22	22.0857	86.4082	853.1990	3.4592	2.6214
80	2016	22	22.9286	85.6531	873.8490	2.7378	2.5633
3	2016	22	27.5571	82.2959	942.3898	3.6480	1.0245
52	2016	22	23.3286	91.7143	900.9837	1.1418	3.0510
70	2016	22	25.3714	76.4796	908.7061	4.0592	1.8918
64	2016	22	18.9714	82.2857	775.5857	4.9949	2.3408
48	2016	22	25.1286	81.9796	931.1745	2.2847	0.7765
65	2016	22	23.3286	91.7143	900.9837	1.1418	3.0510
44	2016	22	25.3714	76.4796	908.7061	4.0592	1.8918
75	2016	22	18.9714	82.2857	775.5857	4.9949	2.3408
40	2016	22	24.4857	88.5714	944.0980	1.7408	1.6939
11	2016	22	23.5857	86.0306	876.5612	3.6704	1.8388
35	2016	22	24.2286	84.4082	937.8296	1.9122	1.5092
78	2016	22	24.6857	81.3367	898.0867	3.5306	1.9378
28	2016	22	25.1143	85.9898	927.5337	1.9010	1.5459
39	2016	22	23.3286	91.7143	900.9837	1.1418	3.0510
24	2016	22	25.7714	83.5816	938.3541	1.4857	1.1296

63	2016	22	24.4857	88.5714	944.0980	1.7408	1.6939
62	2016	22	24.2000	82.8878	873.2551	3.3643	1.5306
1	2016	22	22.9286	85.6531	873.8490	2.7378	2.5633
31	2016	23	19.5000	76.6735	845.1663	6.1704	1.0990
79	2016	23	24.7714	79.9796	966.5612	3.5857	1.2592
51	2016	23	23.5000	80.6429	936.5837	4.0286	1.4898
14	2016	23	21.9000	85.1837	895.6806	2.9214	1.8663
67	2016	23	21.6429	88.5816	900.2990	2.4143	3.0224
42	2016	23	21.0571	80.1735	873.4398	4.9112	2.4653
50	2016	23	22.2857	74.8367	899.6031	4.5439	1.9173
43	2016	23	21.0571	80.1735	873.4398	4.9112	2.4653
85	2016	23	22.6571	71.9388	907.8857	6.5020	1.9796
25	2016	23	25.7143	86.5306	973.5490	3.4898	0.7857
69	2016	23	24.1286	79.0816	937.0796	3.7622	1.2571
57	2016	23	21.4000	81.6531	884.9449	5.7806	2.7020
9	2016	23	20.6857	81.5000	853.0051	5.6949	2.7388
72	2016	23	21.8143	81.6020	876.2490	5.8020	1.7806
26	2016	23	21.8143	77.1633	865.8306	6.9204	2.2051
7	2016	23	21.4286	75.1531	858.1510	6.4378	2.3235
83	2016	23	25.6143	77.8980	941.6684	5.9949	1.2602
76	2016	23	23.2857	78.5714	918.4071	5.3306	1.5031
36	2016	23	23.5000	82.3061	926.4724	4.0612	1.4929
81	2016	23	23.5000	80.6429	936.5837	4.0286	1.4898
15	2016	23	23.1286	77.9898	929.9704	4.7092	0.7520
32	2016	23	21.0571	80.1735	873.4398	4.9112	2.4653
73	2016	23	24.8571	76.6531	958.4939	3.8510	1.0755
71	2016	23	23.5000	82.3061	926.4724	4.0612	1.4929
41	2016	23	21.0857	78.1224	872.8541	5.8510	1.5398
10	2016	23	24.6857	82.6633	961.1245	2.9051	0.9092
23	2016	23	16.3000	76.0306	776.0306	7.5776	2.3429
27	2016	23	21.4286	75.1531	858.1510	6.4378	2.3235
60	2016	23	23.5000	80.6429	936.5837	4.0286	1.4898
53	2016	23	20.6857	81.5000	853.0051	5.6949	2.7388
66	2016	23	21.9000	85.1837	895.6806	2.9214	1.8663
59	2016	23	21.4000	81.6531	884.9449	5.7806	2.7020
61	2016	23	24.8571	76.6531	958.4939	3.8510	1.0755
84	2016	23	24.8571	76.6531	958.4939	3.8510	1.0755
38	2016	23	21.4000	81.6531	884.9449	5.7806	2.7020
87	2016	23	22.4857	78.6837	897.5429	5.4490	1.8531
34	2016	23	21.4000	81.6531	884.9449	5.7806	2.7020
29	2016	23	24.1286	79.0816	937.0796	3.7622	1.2571
5	2016	23	20.0286	76.7755	831.6000	6.7704	1.9459
8	2016	23	20.6857	81.5000	853.0051	5.6949	2.7388
12	2016	23	20.0286	76.7755	831.6000	6.7704	1.9459
13	2016	23	25.6143	77.8980	941.6684	5.9949	1.2602
18	2016	23	24.0286	80.5612	960.8643	3.1582	1.0184
33	2016	23	22.2857	74.8367	899.6031	4.5439	1.9173
56	2016	23	25.7143	86.5306	973.5490	3.4898	0.7857
77	2016	23	23.1286	77.9898	929.9704	4.7092	0.7520
54	2016	23	20.0286	76.7755	831.6000	6.7704	1.9459

21	2016	23	22.2857	74.8367	899.6031	4.5439	1.9173
68	2016	23	24.7714	79.9796	966.5612	3.5857	1.2592
74	2016	23	24.8571	76.6531	958.4939	3.8510	1.0755
88	2016	23	21.0571	80.1735	873.4398	4.9112	2.4653
16	2016	23	23.2857	78.5714	918.4071	5.3306	1.5031
30	2016	23	21.9000	85.1837	895.6806	2.9214	1.8663
6	2016	23	24.7714	79.9796	966.5612	3.5857	1.2592
49	2016	23	24.1286	79.0816	937.0796	3.7622	1.2571
22	2016	23	21.0571	80.1735	873.4398	4.9112	2.4653
45	2016	23	19.2571	72.9184	817.0112	5.7847	2.2122
58	2016	23	24.1286	79.0816	937.0796	3.7622	1.2571
37	2016	23	24.7714	79.9796	966.5612	3.5857	1.2592
17	2016	23	21.6429	88.5816	900.2990	2.4143	3.0224
55	2016	23	21.8143	81.6020	876.2490	5.8020	1.7806
46	2016	23	23.2857	78.5714	918.4071	5.3306	1.5031
86	2016	23	20.8000	75.9286	864.6724	5.9806	1.6276
2	2016	23	20.8000	75.9286	864.6724	5.9806	1.6276
4	2016	23	22.2857	74.8367	899.6031	4.5439	1.9173
47	2016	23	24.7000	79.3265	955.5347	4.7541	0.6806
82	2016	23	21.0571	80.1735	873.4398	4.9112	2.4653
19	2016	23	25.6000	85.5408	957.4520	4.3694	1.1347
20	2016	23	20.6857	81.5000	853.0051	5.6949	2.7388
80	2016	23	21.0571	80.1735	873.4398	4.9112	2.4653
3	2016	23	25.6143	77.8980	941.6684	5.9949	1.2602
52	2016	23	21.6429	88.5816	900.2990	2.4143	3.0224
70	2016	23	22.6571	71.9388	907.8857	6.5020	1.9796
64	2016	23	16.3000	76.0306	776.0306	7.5776	2.3429
48	2016	23	23.1286	77.9898	929.9704	4.7092	0.7520
65	2016	23	21.6429	88.5816	900.2990	2.4143	3.0224
44	2016	23	22.6571	71.9388	907.8857	6.5020	1.9796
75	2016	23	16.3000	76.0306	776.0306	7.5776	2.3429
40	2016	23	24.0857	85.0000	942.8929	3.7316	1.6531
11	2016	23	21.8143	81.6020	876.2490	5.8020	1.7806
35	2016	23	23.5000	80.6429	936.5837	4.0286	1.4898
78	2016	23	22.4857	78.6837	897.5429	5.4490	1.8531
28	2016	23	23.5000	82.3061	926.4724	4.0612	1.4929
39	2016	23	21.6429	88.5816	900.2990	2.4143	3.0224
24	2016	23	24.1286	79.0816	937.0796	3.7622	1.2571
63	2016	23	24.0857	85.0000	942.8929	3.7316	1.6531
62	2016	23	21.0857	78.1224	872.8541	5.8510	1.5398
1	2016	23	21.0571	80.1735	873.4398	4.9112	2.4653
31	2016	24	20.2286	81.0612	845.5776	4.4592	1.0122
79	2016	24	25.6000	79.3878	966.0255	4.2480	1.2112
51	2016	24	23.7429	82.0612	936.2908	3.4469	1.2918
14	2016	24	22.2286	86.5714	895.4255	2.4827	1.3878
67	2016	24	22.2000	88.7755	900.1265	1.6918	2.4173
42	2016	24	21.5714	80.8469	873.4265	3.5296	2.3133
50	2016	24	22.8000	75.9898	899.8296	4.0959	1.6469
43	2016	24	21.5714	80.8469	873.4265	3.5296	2.3133
85	2016	24	23.5571	75.4082	908.1306	4.7653	1.4827

25	2016	24	25.7286	87.9898	973.0816	2.8561	0.6571
69	2016	24	24.3857	79.5408	936.9837	3.4429	1.2306
57	2016	24	22.1714	84.4592	885.1082	3.8653	2.1337
9	2016	24	20.5571	82.4592	853.1204	4.2276	2.2245
72	2016	24	21.7857	84.0510	876.2255	4.0888	1.5265
26	2016	24	21.7143	80.6020	865.9286	5.7469	1.7235
7	2016	24	21.3429	78.7959	858.2827	4.3857	1.7041
83	2016	24	25.8714	80.2755	941.5816	4.6929	1.1929
76	2016	24	23.2143	78.5816	918.5112	4.2194	1.4112
36	2016	24	24.2000	84.1020	926.2551	3.5939	1.2969
81	2016	24	23.7429	82.0612	936.2908	3.4469	1.2918
15	2016	24	24.1000	79.0510	930.1184	4.6235	0.6704
32	2016	24	21.5714	80.8469	873.4265	3.5296	2.3133
73	2016	24	25.5143	76.8367	958.4255	3.8480	1.0276
71	2016	24	24.2000	84.1020	926.2551	3.5939	1.2969
41	2016	24	21.9571	81.7041	873.1082	4.0429	1.0235
10	2016	24	24.6429	83.1020	960.5878	2.7847	0.8847
23	2016	24	16.8286	80.2143	776.4541	4.8592	2.0663
27	2016	24	21.3429	78.7959	858.2827	4.3857	1.7041
60	2016	24	23.7429	82.0612	936.2908	3.4469	1.2918
53	2016	24	20.5571	82.4592	853.1204	4.2276	2.2245
66	2016	24	22.2286	86.5714	895.4255	2.4827	1.3878
59	2016	24	22.1714	84.4592	885.1082	3.8653	2.1337
61	2016	24	25.5143	76.8367	958.4255	3.8480	1.0276
84	2016	24	25.5143	76.8367	958.4255	3.8480	1.0276
38	2016	24	22.1714	84.4592	885.1082	3.8653	2.1337
87	2016	24	23.0857	80.3163	897.4663	3.8704	1.5857
34	2016	24	22.1714	84.4592	885.1082	3.8653	2.1337
29	2016	24	24.3857	79.5408	936.9837	3.4429	1.2306
5	2016	24	19.9286	79.8061	831.8235	5.1827	1.5071
8	2016	24	20.5571	82.4592	853.1204	4.2276	2.2245
12	2016	24	19.9286	79.8061	831.8235	5.1827	1.5071
13	2016	24	25.8714	80.2755	941.5816	4.6929	1.1929
18	2016	24	25.2714	80.0000	960.4633	3.9816	1.0653
33	2016	24	22.8000	75.9898	899.8296	4.0959	1.6469
56	2016	24	25.7286	87.9898	973.0816	2.8561	0.6571
77	2016	24	24.1000	79.0510	930.1184	4.6235	0.6704
54	2016	24	19.9286	79.8061	831.8235	5.1827	1.5071
21	2016	24	22.8000	75.9898	899.8296	4.0959	1.6469
68	2016	24	25.6000	79.3878	966.0255	4.2480	1.2112
74	2016	24	25.5143	76.8367	958.4255	3.8480	1.0276
88	2016	24	21.5714	80.8469	873.4265	3.5296	2.3133
16	2016	24	23.2143	78.5816	918.5112	4.2194	1.4112
30	2016	24	22.2286	86.5714	895.4255	2.4827	1.3878
6	2016	24	25.6000	79.3878	966.0255	4.2480	1.2112
49	2016	24	24.3857	79.5408	936.9837	3.4429	1.2306
22	2016	24	21.5714	80.8469	873.4265	3.5296	2.3133
45	2016	24	19.9000	77.4898	817.2551	4.6143	1.5980
58	2016	24	24.3857	79.5408	936.9837	3.4429	1.2306
37	2016	24	25.6000	79.3878	966.0255	4.2480	1.2112

17	2016	24	22.2000	88.7755	900.1265	1.6918	2.4173
55	2016	24	21.7857	84.0510	876.2255	4.0888	1.5265
46	2016	24	23.2143	78.5816	918.5112	4.2194	1.4112
86	2016	24	21.3000	78.4694	864.9112	4.2296	1.2337
2	2016	24	21.3000	78.4694	864.9112	4.2296	1.2337
4	2016	24	22.8000	75.9898	899.8296	4.0959	1.6469
47	2016	24	24.9571	80.8265	955.3204	3.4276	0.5082
82	2016	24	21.5714	80.8469	873.4265	3.5296	2.3133
19	2016	24	25.5000	86.9388	957.0755	3.0612	1.0714
20	2016	24	20.5571	82.4592	853.1204	4.2276	2.2245
80	2016	24	21.5714	80.8469	873.4265	3.5296	2.3133
3	2016	24	25.8714	80.2755	941.5816	4.6929	1.1929
52	2016	24	22.2000	88.7755	900.1265	1.6918	2.4173
70	2016	24	23.5571	75.4082	908.1306	4.7653	1.4827
64	2016	24	16.8286	80.2143	776.4541	4.8592	2.0663
48	2016	24	24.1000	79.0510	930.1184	4.6235	0.6704
65	2016	24	22.2000	88.7755	900.1265	1.6918	2.4173
44	2016	24	23.5571	75.4082	908.1306	4.7653	1.4827
75	2016	24	16.8286	80.2143	776.4541	4.8592	2.0663
40	2016	24	24.1143	85.3571	942.3663	3.3776	1.2041
11	2016	24	21.7857	84.0510	876.2255	4.0888	1.5265
35	2016	24	23.7429	82.0612	936.2908	3.4469	1.2918
78	2016	24	23.0857	80.3163	897.4663	3.8704	1.5857
28	2016	24	24.2000	84.1020	926.2551	3.5939	1.2969
39	2016	24	22.2000	88.7755	900.1265	1.6918	2.4173
24	2016	24	24.3857	79.5408	936.9837	3.4429	1.2306
63	2016	24	24.1143	85.3571	942.3663	3.3776	1.2041
62	2016	24	21.9571	81.7041	873.1082	4.0429	1.0235
1	2016	24	21.5714	80.8469	873.4265	3.5296	2.3133
31	2016	25	21.9429	84.2551	844.4163	2.6827	0.9602
79	2016	25	27.0286	77.8571	964.8041	4.3347	1.1786
51	2016	25	26.3286	79.3571	935.3561	3.6173	1.3816
14	2016	25	24.6429	85.1837	895.0622	2.0357	2.1704
67	2016	25	24.6714	87.0102	899.4469	1.2908	3.3612
42	2016	25	23.6000	82.9082	872.6541	2.3714	2.7327
50	2016	25	24.3000	79.8571	898.6173	2.4010	1.5204
43	2016	25	23.6000	82.9082	872.6541	2.3714	2.7327
85	2016	25	25.4571	77.6327	906.7694	3.6347	1.5469
25	2016	25	27.9857	85.1633	972.6296	2.9245	0.9194
69	2016	25	27.1857	79.1735	935.9735	2.7745	1.4592
57	2016	25	24.6429	83.9796	884.0337	2.9878	2.5500
9	2016	25	22.0714	86.6122	852.5082	3.2276	2.6153
72	2016	25	24.0143	86.8469	875.7245	3.1388	1.9929
26	2016	25	23.6000	84.2347	865.4551	4.5929	1.7673
7	2016	25	23.5571	82.1531	857.7276	2.7429	1.9816
83	2016	25	28.0286	81.9286	941.1980	4.2306	1.0541
76	2016	25	25.5571	80.6939	917.3480	3.1684	1.4786
36	2016	25	27.0143	79.9082	925.4653	3.6010	1.6571
81	2016	25	26.3286	79.3571	935.3561	3.6173	1.3816
15	2016	25	25.8857	80.7959	928.7622	3.4408	0.7551

32	2016	25	23.6000	82.9082	872.6541	2.3714	2.7327
73	2016	25	28.0143	76.8673	957.1337	3.3418	1.2806
71	2016	25	27.0143	79.9082	925.4653	3.6010	1.6571
41	2016	25	24.0714	83.4490	872.0571	2.8163	1.2041
10	2016	25	27.0571	79.5918	959.7306	2.9561	1.0459
23	2016	25	18.1143	85.3776	775.6357	2.1745	2.1082
27	2016	25	23.5571	82.1531	857.7276	2.7429	1.9816
60	2016	25	26.3286	79.3571	935.3561	3.6173	1.3816
53	2016	25	22.0714	86.6122	852.5082	3.2276	2.6153
66	2016	25	24.6429	85.1837	895.0622	2.0357	2.1704
59	2016	25	24.6429	83.9796	884.0337	2.9878	2.5500
61	2016	25	28.0143	76.8673	957.1337	3.3418	1.2806
84	2016	25	28.0143	76.8673	957.1337	3.3418	1.2806
38	2016	25	24.6429	83.9796	884.0337	2.9878	2.5500
87	2016	25	25.6143	80.9286	896.8082	3.0684	1.6806
34	2016	25	24.6429	83.9796	884.0337	2.9878	2.5500
29	2016	25	27.1857	79.1735	935.9735	2.7745	1.4592
5	2016	25	21.6000	83.6327	831.1867	3.2633	1.5847
8	2016	25	22.0714	86.6122	852.5082	3.2276	2.6153
12	2016	25	21.6000	83.6327	831.1867	3.2633	1.5847
13	2016	25	28.0286	81.9286	941.1980	4.2306	1.0541
18	2016	25	26.4857	80.0204	959.1357	4.0816	1.2418
33	2016	25	24.3000	79.8571	898.6173	2.4010	1.5204
56	2016	25	27.9857	85.1633	972.6296	2.9245	0.9194
77	2016	25	25.8857	80.7959	928.7622	3.4408	0.7551
54	2016	25	21.6000	83.6327	831.1867	3.2633	1.5847
21	2016	25	24.3000	79.8571	898.6173	2.4010	1.5204
68	2016	25	27.0286	77.8571	964.8041	4.3347	1.1786
74	2016	25	28.0143	76.8673	957.1337	3.3418	1.2806
88	2016	25	23.6000	82.9082	872.6541	2.3714	2.7327
16	2016	25	25.5571	80.6939	917.3480	3.1684	1.4786
30	2016	25	24.6429	85.1837	895.0622	2.0357	2.1704
6	2016	25	27.0286	77.8571	964.8041	4.3347	1.1786
49	2016	25	27.1857	79.1735	935.9735	2.7745	1.4592
22	2016	25	23.6000	82.9082	872.6541	2.3714	2.7327
45	2016	25	21.4714	79.4184	816.6388	3.1816	1.9429
58	2016	25	27.1857	79.1735	935.9735	2.7745	1.4592
37	2016	25	27.0286	77.8571	964.8041	4.3347	1.1786
17	2016	25	24.6714	87.0102	899.4469	1.2908	3.3612
55	2016	25	24.0143	86.8469	875.7245	3.1388	1.9929
46	2016	25	25.5571	80.6939	917.3480	3.1684	1.4786
86	2016	25	22.9571	81.7041	863.9673	2.6878	1.9918
2	2016	25	22.9571	81.7041	863.9673	2.6878	1.9918
4	2016	25	24.3000	79.8571	898.6173	2.4010	1.5204
47	2016	25	27.1857	81.2653	954.8704	3.0143	0.4449
82	2016	25	23.6000	82.9082	872.6541	2.3714	2.7327
19	2016	25	27.9429	87.3571	956.8878	2.5806	1.1316
20	2016	25	22.0714	86.6122	852.5082	3.2276	2.6153
80	2016	25	23.6000	82.9082	872.6541	2.3714	2.7327
3	2016	25	28.0286	81.9286	941.1980	4.2306	1.0541

52	2016	25	24.6714	87.0102	899.4469	1.2908	3.3612
70	2016	25	25.4571	77.6327	906.7694	3.6347	1.5469
64	2016	25	18.1143	85.3776	775.6357	2.1745	2.1082
48	2016	25	25.8857	80.7959	928.7622	3.4408	0.7551
65	2016	25	24.6714	87.0102	899.4469	1.2908	3.3612
44	2016	25	25.4571	77.6327	906.7694	3.6347	1.5469
75	2016	25	18.1143	85.3776	775.6357	2.1745	2.1082
40	2016	25	26.9714	81.2143	941.8908	4.1092	2.0235
11	2016	25	24.0143	86.8469	875.7245	3.1388	1.9929
35	2016	25	26.3286	79.3571	935.3561	3.6173	1.3816
78	2016	25	25.6143	80.9286	896.8082	3.0684	1.6806
28	2016	25	27.0143	79.9082	925.4653	3.6010	1.6571
39	2016	25	24.6714	87.0102	899.4469	1.2908	3.3612
24	2016	25	27.1857	79.1735	935.9735	2.7745	1.4592
63	2016	25	26.9714	81.2143	941.8908	4.1092	2.0235
62	2016	25	24.0714	83.4490	872.0571	2.8163	1.2041
1	2016	25	23.6000	82.9082	872.6541	2.3714	2.7327
31	2016	26	20.7714	84.3469	844.8061	2.1602	0.9592
79	2016	26	25.1000	77.2653	965.3847	3.7643	1.3520
51	2016	26	24.9571	76.1939	935.8449	4.4684	1.6327
14	2016	26	23.8714	80.7143	895.9276	3.3092	2.8235
67	2016	26	23.9286	83.3265	900.0582	2.5173	4.1592
42	2016	26	23.1429	80.2959	873.2031	3.2949	2.9235
50	2016	26	22.7429	80.2755	898.9765	1.8867	1.6643
43	2016	26	23.1429	80.2959	873.2031	3.2949	2.9235
85	2016	26	23.5286	76.9082	907.1153	3.3010	1.8173
25	2016	26	27.4000	80.6531	973.2010	5.1031	1.2276
69	2016	26	24.7000	77.5408	936.3653	2.8337	1.7592
57	2016	26	22.6143	83.1531	884.4653	3.3449	2.7643
9	2016	26	22.1571	84.2857	853.1112	4.3041	3.0949
72	2016	26	23.5286	83.9898	876.4102	5.1163	2.2449
26	2016	26	23.2143	83.1429	866.0929	5.8500	2.1541
7	2016	26	23.0000	79.4388	858.3367	4.5541	2.4520
83	2016	26	27.5143	77.2959	941.8408	6.6000	1.1704
76	2016	26	23.3286	81.4898	917.7133	3.2143	1.5980
36	2016	26	25.7714	73.8878	925.9276	5.1306	1.9653
81	2016	26	24.9571	76.1939	935.8449	4.4684	1.6327
15	2016	26	23.5429	82.0612	929.0929	2.2602	0.8306
32	2016	26	23.1429	80.2959	873.2031	3.2949	2.9235
73	2016	26	24.7143	77.6837	957.5367	3.1041	1.4439
71	2016	26	25.7714	73.8878	925.9276	5.1306	1.9653
41	2016	26	22.3571	83.1939	872.4816	2.9071	1.6786
10	2016	26	26.0571	76.9898	960.3010	4.3602	1.2010
23	2016	26	17.9714	85.5204	776.0857	1.4490	2.1704
27	2016	26	23.0000	79.4388	858.3367	4.5541	2.4520
60	2016	26	24.9571	76.1939	935.8449	4.4684	1.6327
53	2016	26	22.1571	84.2857	853.1112	4.3041	3.0949
66	2016	26	23.8714	80.7143	895.9276	3.3092	2.8235
59	2016	26	22.6143	83.1531	884.4653	3.3449	2.7643
61	2016	26	24.7143	77.6837	957.5367	3.1041	1.4439

84	2016	26	24.7143	77.6837	957.5367	3.1041	1.4439
38	2016	26	22.6143	83.1531	884.4653	3.3449	2.7643
87	2016	26	24.9286	76.5510	897.3663	5.2143	1.7878
34	2016	26	22.6143	83.1531	884.4653	3.3449	2.7643
29	2016	26	24.7000	77.5408	936.3653	2.8337	1.7592
5	2016	26	21.4000	82.5714	831.7459	4.1378	1.7704
8	2016	26	22.1571	84.2857	853.1112	4.3041	3.0949
12	2016	26	21.4000	82.5714	831.7459	4.1378	1.7704
13	2016	26	27.5143	77.2959	941.8408	6.6000	1.1704
18	2016	26	24.4429	81.1020	959.6857	3.3694	1.3908
33	2016	26	22.7429	80.2755	898.9765	1.8867	1.6643
56	2016	26	27.4000	80.6531	973.2010	5.1031	1.2276
77	2016	26	23.5429	82.0612	929.0929	2.2602	0.8306
54	2016	26	21.4000	82.5714	831.7459	4.1378	1.7704
21	2016	26	22.7429	80.2755	898.9765	1.8867	1.6643
68	2016	26	25.1000	77.2653	965.3847	3.7643	1.3520
74	2016	26	24.7143	77.6837	957.5367	3.1041	1.4439
88	2016	26	23.1429	80.2959	873.2031	3.2949	2.9235
16	2016	26	23.3286	81.4898	917.7133	3.2143	1.5980
30	2016	26	23.8714	80.7143	895.9276	3.3092	2.8235
6	2016	26	25.1000	77.2653	965.3847	3.7643	1.3520
49	2016	26	24.7000	77.5408	936.3653	2.8337	1.7592
22	2016	26	23.1429	80.2959	873.2031	3.2949	2.9235
45	2016	26	21.1143	77.6020	817.2041	3.7980	2.2102
58	2016	26	24.7000	77.5408	936.3653	2.8337	1.7592
37	2016	26	25.1000	77.2653	965.3847	3.7643	1.3520
17	2016	26	23.9286	83.3265	900.0582	2.5173	4.1592
55	2016	26	23.5286	83.9898	876.4102	5.1163	2.2449
46	2016	26	23.3286	81.4898	917.7133	3.2143	1.5980
86	2016	26	22.3857	81.8265	864.3796	3.2112	2.3510
2	2016	26	22.3857	81.8265	864.3796	3.2112	2.3510
4	2016	26	22.7429	80.2755	898.9765	1.8867	1.6643
47	2016	26	26.2429	78.2653	955.4918	5.0184	0.4633
82	2016	26	23.1429	80.2959	873.2031	3.2949	2.9235
19	2016	26	27.4000	83.1531	957.6786	5.1990	1.2367
20	2016	26	22.1571	84.2857	853.1112	4.3041	3.0949
80	2016	26	23.1429	80.2959	873.2031	3.2949	2.9235
3	2016	26	27.5143	77.2959	941.8408	6.6000	1.1704
52	2016	26	23.9286	83.3265	900.0582	2.5173	4.1592
70	2016	26	23.5286	76.9082	907.1153	3.3010	1.8173
64	2016	26	17.9714	85.5204	776.0857	1.4490	2.1704
48	2016	26	23.5429	82.0612	929.0929	2.2602	0.8306
65	2016	26	23.9286	83.3265	900.0582	2.5173	4.1592
44	2016	26	23.5286	76.9082	907.1153	3.3010	1.8173
75	2016	26	17.9714	85.5204	776.0857	1.4490	2.1704
40	2016	26	26.5286	74.6531	942.5878	6.8276	2.5969
11	2016	26	23.5286	83.9898	876.4102	5.1163	2.2449
35	2016	26	24.9571	76.1939	935.8449	4.4684	1.6327
78	2016	26	24.9286	76.5510	897.3663	5.2143	1.7878
28	2016	26	25.7714	73.8878	925.9276	5.1306	1.9653

39	2016	26	23.9286	83.3265	900.0582	2.5173	4.1592
24	2016	26	24.7000	77.5408	936.3653	2.8337	1.7592
63	2016	26	26.5286	74.6531	942.5878	6.8276	2.5969
62	2016	26	22.3571	83.1939	872.4816	2.9071	1.6786
1	2016	26	23.1429	80.2959	873.2031	3.2949	2.9235
31	2016	27	23.0143	83.2551	844.7592	3.3633	0.9714
79	2016	27	26.5143	83.4388	965.5082	2.5878	1.3786
51	2016	27	25.5714	82.1224	935.6847	3.2531	1.6112
14	2016	27	24.2571	83.6531	895.4469	3.1194	2.4531
67	2016	27	24.3286	86.3776	899.7735	2.7490	3.5561
42	2016	27	23.7857	81.3980	873.0214	3.6429	2.5153
50	2016	27	25.9571	79.5102	898.9714	2.5531	1.9439
43	2016	27	23.7857	81.3980	873.0214	3.6429	2.5153
85	2016	27	27.1857	76.4898	907.1092	3.7286	1.7969
25	2016	27	27.6571	85.5612	972.6980	4.6367	0.9082
69	2016	27	26.6000	80.9796	936.2592	2.5102	1.5929
57	2016	27	24.7714	85.9592	884.3990	3.4990	2.5367
9	2016	27	22.9429	82.0816	852.8847	5.1765	2.7612
72	2016	27	24.0714	85.2755	876.0357	5.5959	1.8490
26	2016	27	23.8000	84.3878	865.6796	5.6102	2.0796
7	2016	27	23.8000	80.3878	858.0214	5.0306	2.1265
83	2016	27	27.8286	78.2041	941.2388	6.8184	1.2204
76	2016	27	25.9714	83.3571	917.7827	2.9816	1.5122
36	2016	27	26.1000	79.0918	925.6939	4.2133	1.6020
81	2016	27	25.5714	82.1224	935.6847	3.2531	1.6112
15	2016	27	26.9286	82.2653	929.2398	2.0735	0.8276
32	2016	27	23.7857	81.3980	873.0214	3.6429	2.5153
73	2016	27	27.5714	81.9082	957.7031	2.9959	1.1000
71	2016	27	26.1000	79.0918	925.6939	4.2133	1.6020
41	2016	27	24.5429	84.1429	872.4265	3.4765	1.5459
10	2016	27	26.6571	83.4388	960.0316	3.4245	0.9500
23	2016	27	19.1000	85.2653	776.0133	1.3010	2.0531
27	2016	27	23.8000	80.3878	858.0214	5.0306	2.1265
60	2016	27	25.5714	82.1224	935.6847	3.2531	1.6112
53	2016	27	22.9429	82.0816	852.8847	5.1765	2.7612
66	2016	27	24.2571	83.6531	895.4469	3.1194	2.4531
59	2016	27	24.7714	85.9592	884.3990	3.4990	2.5367
61	2016	27	27.5714	81.9082	957.7031	2.9959	1.1000
84	2016	27	27.5714	81.9082	957.7031	2.9959	1.1000
38	2016	27	24.7714	85.9592	884.3990	3.4990	2.5367
87	2016	27	25.1286	77.8878	897.0500	5.4388	1.6122
34	2016	27	24.7714	85.9592	884.3990	3.4990	2.5367
29	2016	27	26.6000	80.9796	936.2592	2.5102	1.5929
5	2016	27	21.8143	83.7755	831.5439	4.4663	1.6592
8	2016	27	22.9429	82.0816	852.8847	5.1765	2.7612
12	2016	27	21.8143	83.7755	831.5439	4.4663	1.6592
13	2016	27	27.8286	78.2041	941.2388	6.8184	1.2204
18	2016	27	26.4286	85.3673	959.9316	2.2153	1.1847
33	2016	27	25.9571	79.5102	898.9714	2.5531	1.9439
56	2016	27	27.6571	85.5612	972.6980	4.6367	0.9082

77	2016	27	26.9286	82.2653	929.2398	2.0735	0.8276
54	2016	27	21.8143	83.7755	831.5439	4.4663	1.6592
21	2016	27	25.9571	79.5102	898.9714	2.5531	1.9439
68	2016	27	26.5143	83.4388	965.5082	2.5878	1.3786
74	2016	27	27.5714	81.9082	957.7031	2.9959	1.1000
88	2016	27	23.7857	81.3980	873.0214	3.6429	2.5153
16	2016	27	25.9714	83.3571	917.7827	2.9816	1.5122
30	2016	27	24.2571	83.6531	895.4469	3.1194	2.4531
6	2016	27	26.5143	83.4388	965.5082	2.5878	1.3786
49	2016	27	26.6000	80.9796	936.2592	2.5102	1.5929
22	2016	27	23.7857	81.3980	873.0214	3.6429	2.5153
45	2016	27	21.1714	80.4796	816.9633	3.9827	1.8694
58	2016	27	26.6000	80.9796	936.2592	2.5102	1.5929
37	2016	27	26.5143	83.4388	965.5082	2.5878	1.3786
17	2016	27	24.3286	86.3776	899.7735	2.7490	3.5561
55	2016	27	24.0714	85.2755	876.0357	5.5959	1.8490
46	2016	27	25.9714	83.3571	917.7827	2.9816	1.5122
86	2016	27	24.1857	81.3980	864.2520	4.0337	1.8602
2	2016	27	24.1857	81.3980	864.2520	4.0337	1.8602
4	2016	27	25.9571	79.5102	898.9714	2.5531	1.9439
47	2016	27	26.6000	81.2857	954.9980	4.7684	0.4153
82	2016	27	23.7857	81.3980	873.0214	3.6429	2.5153
19	2016	27	27.4571	85.9898	957.0306	4.3990	1.0429
20	2016	27	22.9429	82.0816	852.8847	5.1765	2.7612
80	2016	27	23.7857	81.3980	873.0214	3.6429	2.5153
3	2016	27	27.8286	78.2041	941.2388	6.8184	1.2204
52	2016	27	24.3286	86.3776	899.7735	2.7490	3.5561
70	2016	27	27.1857	76.4898	907.1092	3.7286	1.7969
64	2016	27	19.1000	85.2653	776.0133	1.3010	2.0531
48	2016	27	26.9286	82.2653	929.2398	2.0735	0.8276
65	2016	27	24.3286	86.3776	899.7735	2.7490	3.5561
44	2016	27	27.1857	76.4898	907.1092	3.7286	1.7969
75	2016	27	19.1000	85.2653	776.0133	1.3010	2.0531
40	2016	27	26.7571	78.8163	942.0898	5.8796	1.9306
11	2016	27	24.0714	85.2755	876.0357	5.5959	1.8490
35	2016	27	25.5714	82.1224	935.6847	3.2531	1.6112
78	2016	27	25.1286	77.8878	897.0500	5.4388	1.6122
28	2016	27	26.1000	79.0918	925.6939	4.2133	1.6020
39	2016	27	24.3286	86.3776	899.7735	2.7490	3.5561
24	2016	27	26.6000	80.9796	936.2592	2.5102	1.5929
63	2016	27	26.7571	78.8163	942.0898	5.8796	1.9306
62	2016	27	24.5429	84.1429	872.4265	3.4765	1.5459
1	2016	27	23.7857	81.3980	873.0214	3.6429	2.5153
31	2016	28	22.6429	80.9082	843.3582	6.0316	1.0490
79	2016	28	27.9429	84.0816	963.5337	4.4888	1.1439
51	2016	28	26.7000	83.3673	934.0469	4.4847	1.3378
14	2016	28	24.5857	86.0510	893.9673	3.0112	2.0000
67	2016	28	25.0429	87.2245	898.3510	3.8969	2.8663
42	2016	28	24.2143	80.7245	871.7367	4.8000	2.2827
50	2016	28	25.7571	75.6122	897.3663	5.3602	2.0592

43	2016	28	24.2143	80.7245	871.7367	4.8000	2.2827
85	2016	28	25.6714	72.7143	905.3510	6.5980	1.8827
25	2016	28	28.9714	86.0204	970.9510	5.1296	0.8051
69	2016	28	27.2714	80.5102	934.6398	4.9051	1.2980
57	2016	28	24.7857	82.8469	882.9061	4.8908	2.6194
9	2016	28	23.5286	79.9490	851.6378	6.4959	2.4939
72	2016	28	24.3714	86.4388	874.6643	6.2582	1.4531
26	2016	28	24.2857	83.2347	864.3531	5.8745	1.9500
7	2016	28	23.7429	80.0306	856.7571	5.8235	1.7969
83	2016	28	28.8000	79.1633	939.5571	7.1776	1.2245
76	2016	28	26.3571	80.4694	916.1796	5.3531	1.4959
36	2016	28	27.0571	81.0204	924.1878	4.8398	1.0796
81	2016	28	26.7000	83.3673	934.0469	4.4847	1.3378
15	2016	28	27.3571	77.7551	927.4500	4.4582	0.8245
32	2016	28	24.2143	80.7245	871.7367	4.8000	2.2827
73	2016	28	28.2714	79.4796	955.7857	4.8255	0.9724
71	2016	28	27.0571	81.0204	924.1878	4.8398	1.0796
41	2016	28	24.6429	80.6939	870.9745	5.9122	1.4745
10	2016	28	27.7857	84.1735	958.1898	3.9949	0.7694
23	2016	28	19.3286	82.2857	775.1276	3.4898	2.0592
27	2016	28	23.7429	80.0306	856.7571	5.8235	1.7969
60	2016	28	26.7000	83.3673	934.0469	4.4847	1.3378
53	2016	28	23.5286	79.9490	851.6378	6.4959	2.4939
66	2016	28	24.5857	86.0510	893.9673	3.0112	2.0000
59	2016	28	24.7857	82.8469	882.9061	4.8908	2.6194
61	2016	28	28.2714	79.4796	955.7857	4.8255	0.9724
84	2016	28	28.2714	79.4796	955.7857	4.8255	0.9724
38	2016	28	24.7857	82.8469	882.9061	4.8908	2.6194
87	2016	28	25.7571	79.5204	895.6388	6.3051	1.3847
34	2016	28	24.7857	82.8469	882.9061	4.8908	2.6194
29	2016	28	27.2714	80.5102	934.6398	4.9051	1.2980
5	2016	28	21.6143	83.7245	830.4582	5.8082	1.5480
8	2016	28	23.5286	79.9490	851.6378	6.4959	2.4939
12	2016	28	21.6143	83.7245	830.4582	5.8082	1.5480
13	2016	28	28.8000	79.1633	939.5571	7.1776	1.2245
18	2016	28	28.1571	83.5510	957.9673	4.3418	0.9704
33	2016	28	25.7571	75.6122	897.3663	5.3602	2.0592
56	2016	28	28.9714	86.0204	970.9510	5.1296	0.8051
77	2016	28	27.3571	77.7551	927.4500	4.4582	0.8245
54	2016	28	21.6143	83.7245	830.4582	5.8082	1.5480
21	2016	28	25.7571	75.6122	897.3663	5.3602	2.0592
68	2016	28	27.9429	84.0816	963.5337	4.4888	1.1439
74	2016	28	28.2714	79.4796	955.7857	4.8255	0.9724
88	2016	28	24.2143	80.7245	871.7367	4.8000	2.2827
16	2016	28	26.3571	80.4694	916.1796	5.3531	1.4959
30	2016	28	24.5857	86.0510	893.9673	3.0112	2.0000
6	2016	28	27.9429	84.0816	963.5337	4.4888	1.1439
49	2016	28	27.2714	80.5102	934.6398	4.9051	1.2980
22	2016	28	24.2143	80.7245	871.7367	4.8000	2.2827
45	2016	28	21.6000	79.7245	815.9398	5.1694	1.6469

58	2016	28	27.2714	80.5102	934.6398	4.9051	1.2980
37	2016	28	27.9429	84.0816	963.5337	4.4888	1.1439
17	2016	28	25.0429	87.2245	898.3510	3.8969	2.8663
55	2016	28	24.3714	86.4388	874.6643	6.2582	1.4531
46	2016	28	26.3571	80.4694	916.1796	5.3531	1.4959
86	2016	28	24.3143	77.1939	862.8888	6.5898	1.9337
2	2016	28	24.3143	77.1939	862.8888	6.5898	1.9337
4	2016	28	25.7571	75.6122	897.3663	5.3602	2.0592
47	2016	28	27.8286	82.5816	953.3204	4.5776	0.4102
82	2016	28	24.2143	80.7245	871.7367	4.8000	2.2827
19	2016	28	28.6143	86.9184	955.2959	3.9643	0.9061
20	2016	28	23.5286	79.9490	851.6378	6.4959	2.4939
80	2016	28	24.2143	80.7245	871.7367	4.8000	2.2827
3	2016	28	28.8000	79.1633	939.5571	7.1776	1.2245
52	2016	28	25.0429	87.2245	898.3510	3.8969	2.8663
70	2016	28	25.6714	72.7143	905.3510	6.5980	1.8827
64	2016	28	19.3286	82.2857	775.1276	3.4898	2.0592
48	2016	28	27.3571	77.7551	927.4500	4.4582	0.8245
65	2016	28	25.0429	87.2245	898.3510	3.8969	2.8663
44	2016	28	25.6714	72.7143	905.3510	6.5980	1.8827
75	2016	28	19.3286	82.2857	775.1276	3.4898	2.0592
40	2016	28	27.7143	80.4286	940.3459	5.8500	1.3786
11	2016	28	24.3714	86.4388	874.6643	6.2582	1.4531
35	2016	28	26.7000	83.3673	934.0469	4.4847	1.3378
78	2016	28	25.7571	79.5204	895.6388	6.3051	1.3847
28	2016	28	27.0571	81.0204	924.1878	4.8398	1.0796
39	2016	28	25.0429	87.2245	898.3510	3.8969	2.8663
24	2016	28	27.2714	80.5102	934.6398	4.9051	1.2980
63	2016	28	27.7143	80.4286	940.3459	5.8500	1.3786
62	2016	28	24.6429	80.6939	870.9745	5.9122	1.4745
1	2016	28	24.2143	80.7245	871.7367	4.8000	2.2827
31	2016	29	23.8000	81.5000	842.7786	5.9786	1.0745
79	2016	29	29.0571	80.5816	962.4592	5.3949	1.0622
51	2016	29	27.8286	78.6837	933.3918	6.3051	1.3796
14	2016	29	24.8857	84.1224	893.8092	2.4673	2.3296
67	2016	29	25.1714	85.2449	897.9827	3.7745	3.3837
42	2016	29	24.5429	79.2959	871.3337	5.2847	2.6041
50	2016	29	26.7571	74.8571	896.5245	5.7214	2.0980
43	2016	29	24.5429	79.2959	871.3337	5.2847	2.6041
85	2016	29	27.9714	72.2959	904.5276	7.1296	2.0724
25	2016	29	28.5857	82.1939	970.5194	5.9153	1.0347
69	2016	29	28.4429	76.8265	933.8980	5.7653	1.4908
57	2016	29	25.9714	81.0714	882.2724	5.1357	3.2449
9	2016	29	23.1143	79.6327	851.3112	6.1000	2.7857
72	2016	29	24.4571	85.4184	874.4337	6.1173	1.6031
26	2016	29	23.8571	81.2143	864.2286	6.1612	2.0908
7	2016	29	23.6000	79.0612	856.5684	6.4102	2.0908
83	2016	29	28.0714	76.9694	939.2959	7.7163	1.2429
76	2016	29	27.4714	78.1429	915.3245	5.8969	1.5663
36	2016	29	27.9143	76.9898	923.6612	5.5520	1.3949

81	2016	29	27.8286	78.6837	933.3918	6.3051	1.3796
15	2016	29	28.3429	75.8878	926.3724	5.4480	0.8745
32	2016	29	24.5429	79.2959	871.3337	5.2847	2.6041
73	2016	29	29.6857	76.1224	954.7153	5.1112	1.3102
71	2016	29	27.9143	76.9898	923.6612	5.5520	1.3949
41	2016	29	25.5714	78.3061	870.3776	5.2204	1.7847
10	2016	29	28.6429	80.0102	957.5531	4.5153	0.8847
23	2016	29	18.9286	80.0102	774.8571	4.0265	2.1990
27	2016	29	23.6000	79.0612	856.5684	6.4102	2.0908
60	2016	29	27.8286	78.6837	933.3918	6.3051	1.3796
53	2016	29	23.1143	79.6327	851.3112	6.1000	2.7857
66	2016	29	24.8857	84.1224	893.8092	2.4673	2.3296
59	2016	29	25.9714	81.0714	882.2724	5.1357	3.2449
61	2016	29	29.6857	76.1224	954.7153	5.1112	1.3102
84	2016	29	29.6857	76.1224	954.7153	5.1112	1.3102
38	2016	29	25.9714	81.0714	882.2724	5.1357	3.2449
87	2016	29	26.0429	78.6122	895.2929	6.6857	1.5163
34	2016	29	25.9714	81.0714	882.2724	5.1357	3.2449
29	2016	29	28.4429	76.8265	933.8980	5.7653	1.4908
5	2016	29	22.1857	82.4388	830.2327	6.0582	1.5571
8	2016	29	23.1143	79.6327	851.3112	6.1000	2.7857
12	2016	29	22.1857	82.4388	830.2327	6.0582	1.5571
13	2016	29	28.0714	76.9694	939.2959	7.7163	1.2429
18	2016	29	28.7286	80.4388	956.7827	5.2806	1.1602
33	2016	29	26.7571	74.8571	896.5245	5.7214	2.0980
56	2016	29	28.5857	82.1939	970.5194	5.9153	1.0347
77	2016	29	28.3429	75.8878	926.3724	5.4480	0.8745
54	2016	29	22.1857	82.4388	830.2327	6.0582	1.5571
21	2016	29	26.7571	74.8571	896.5245	5.7214	2.0980
68	2016	29	29.0571	80.5816	962.4592	5.3949	1.0622
74	2016	29	29.6857	76.1224	954.7153	5.1112	1.3102
88	2016	29	24.5429	79.2959	871.3337	5.2847	2.6041
16	2016	29	27.4714	78.1429	915.3245	5.8969	1.5663
30	2016	29	24.8857	84.1224	893.8092	2.4673	2.3296
6	2016	29	29.0571	80.5816	962.4592	5.3949	1.0622
49	2016	29	28.4429	76.8265	933.8980	5.7653	1.4908
22	2016	29	24.5429	79.2959	871.3337	5.2847	2.6041
45	2016	29	21.3143	76.6633	815.7724	5.2755	2.0378
58	2016	29	28.4429	76.8265	933.8980	5.7653	1.4908
37	2016	29	29.0571	80.5816	962.4592	5.3949	1.0622
17	2016	29	25.1714	85.2449	897.9827	3.7745	3.3837
55	2016	29	24.4571	85.4184	874.4337	6.1173	1.6031
46	2016	29	27.4714	78.1429	915.3245	5.8969	1.5663
86	2016	29	24.5000	75.9082	862.4010	6.9082	2.5061
2	2016	29	24.5000	75.9082	862.4010	6.9082	2.5061
4	2016	29	26.7571	74.8571	896.5245	5.7214	2.0980
47	2016	29	27.2714	79.8980	952.9745	4.8255	0.4602
82	2016	29	24.5429	79.2959	871.3337	5.2847	2.6041
19	2016	29	28.2571	85.1327	955.0969	4.6500	1.0959
20	2016	29	23.1143	79.6327	851.3112	6.1000	2.7857

80	2016	29	24.5429	79.2959	871.3337	5.2847	2.6041
3	2016	29	28.0714	76.9694	939.2959	7.7163	1.2429
52	2016	29	25.1714	85.2449	897.9827	3.7745	3.3837
70	2016	29	27.9714	72.2959	904.5276	7.1296	2.0724
64	2016	29	18.9286	80.0102	774.8571	4.0265	2.1990
48	2016	29	28.3429	75.8878	926.3724	5.4480	0.8745
65	2016	29	25.1714	85.2449	897.9827	3.7745	3.3837
44	2016	29	27.9714	72.2959	904.5276	7.1296	2.0724
75	2016	29	18.9286	80.0102	774.8571	4.0265	2.1990
40	2016	29	27.6857	76.6327	940.0143	6.8949	1.9888
11	2016	29	24.4571	85.4184	874.4337	6.1173	1.6031
35	2016	29	27.8286	78.6837	933.3918	6.3051	1.3796
78	2016	29	26.0429	78.6122	895.2929	6.6857	1.5163
28	2016	29	27.9143	76.9898	923.6612	5.5520	1.3949
39	2016	29	25.1714	85.2449	897.9827	3.7745	3.3837
24	2016	29	28.4429	76.8265	933.8980	5.7653	1.4908
63	2016	29	27.6857	76.6327	940.0143	6.8949	1.9888
62	2016	29	25.5714	78.3061	870.3776	5.2204	1.7847
1	2016	29	24.5429	79.2959	871.3337	5.2847	2.6041
31	2016	30	23.5000	75.2959	844.1745	7.1031	1.2561
79	2016	30	30.6857	74.4286	964.1020	7.1592	1.2541
51	2016	30	28.1143	72.3673	935.2184	8.3224	1.5031
14	2016	30	25.4143	79.3673	895.7969	3.7673	2.7163
67	2016	30	25.3143	81.3469	899.9051	4.4857	3.8235
42	2016	30	25.2286	74.7245	873.0480	6.9806	3.0776
50	2016	30	26.7286	71.3571	897.9612	6.1480	2.1439
43	2016	30	25.2286	74.7245	873.0480	6.9806	3.0776
85	2016	30	28.2429	66.7653	905.9286	8.3020	2.5684
25	2016	30	28.9286	79.8571	972.7245	6.1041	1.0367
69	2016	30	28.2857	71.8163	935.6306	7.1082	1.7194
57	2016	30	26.4571	74.5102	883.8776	7.0265	4.0459
9	2016	30	23.6286	76.9490	853.0347	7.2061	3.0500
72	2016	30	23.7143	81.3265	876.3051	6.6071	1.6898
26	2016	30	23.1857	78.7449	866.1510	6.3714	2.0571
7	2016	30	23.1143	76.4694	858.3694	6.7571	2.2653
83	2016	30	27.4429	74.9286	941.5102	8.0929	1.3357
76	2016	30	28.3286	72.8061	916.8806	7.0551	1.6816
36	2016	30	28.1429	70.5816	925.5684	6.8133	1.9408
81	2016	30	28.1143	72.3673	935.2184	8.3224	1.5031
15	2016	30	29.0143	71.1122	927.7878	6.9622	1.1051
32	2016	30	25.2286	74.7245	873.0480	6.9806	3.0776
73	2016	30	30.4286	69.8469	956.2663	6.8541	1.5265
71	2016	30	28.1429	70.5816	925.5684	6.8133	1.9408
41	2016	30	25.7000	72.7857	871.9643	5.8367	2.2082
10	2016	30	28.6857	74.5510	959.5051	6.1918	1.0480
23	2016	30	18.7143	77.6429	776.1857	5.0684	2.4184
27	2016	30	23.1143	76.4694	858.3694	6.7571	2.2653
60	2016	30	28.1143	72.3673	935.2184	8.3224	1.5031
53	2016	30	23.6286	76.9490	853.0347	7.2061	3.0500
66	2016	30	25.4143	79.3673	895.7969	3.7673	2.7163

59	2016	30	26.4571	74.5102	883.8776	7.0265	4.0459
61	2016	30	30.4286	69.8469	956.2663	6.8541	1.5265
84	2016	30	30.4286	69.8469	956.2663	6.8541	1.5265
38	2016	30	26.4571	74.5102	883.8776	7.0265	4.0459
87	2016	30	25.9000	74.9286	897.1888	7.3112	1.6163
34	2016	30	26.4571	74.5102	883.8776	7.0265	4.0459
29	2016	30	28.2857	71.8163	935.6306	7.1082	1.7194
5	2016	30	21.0714	78.5816	831.8888	6.2571	1.7020
8	2016	30	23.6286	76.9490	853.0347	7.2061	3.0500
12	2016	30	21.0714	78.5816	831.8888	6.2571	1.7020
13	2016	30	27.4429	74.9286	941.5102	8.0929	1.3357
18	2016	30	30.3714	75.0102	958.3031	7.0918	1.4510
33	2016	30	26.7286	71.3571	897.9612	6.1480	2.1439
56	2016	30	28.9286	79.8571	972.7245	6.1041	1.0367
77	2016	30	29.0143	71.1122	927.7878	6.9622	1.1051
54	2016	30	21.0714	78.5816	831.8888	6.2571	1.7020
21	2016	30	26.7286	71.3571	897.9612	6.1480	2.1439
68	2016	30	30.6857	74.4286	964.1020	7.1592	1.2541
74	2016	30	30.4286	69.8469	956.2663	6.8541	1.5265
88	2016	30	25.2286	74.7245	873.0480	6.9806	3.0776
16	2016	30	28.3286	72.8061	916.8806	7.0551	1.6816
30	2016	30	25.4143	79.3673	895.7969	3.7673	2.7163
6	2016	30	30.6857	74.4286	964.1020	7.1592	1.2541
49	2016	30	28.2857	71.8163	935.6306	7.1082	1.7194
22	2016	30	25.2286	74.7245	873.0480	6.9806	3.0776
45	2016	30	19.8857	75.8061	817.4276	4.8296	2.2541
58	2016	30	28.2857	71.8163	935.6306	7.1082	1.7194
37	2016	30	30.6857	74.4286	964.1020	7.1592	1.2541
17	2016	30	25.3143	81.3469	899.9051	4.4857	3.8235
55	2016	30	23.7143	81.3265	876.3051	6.6071	1.6898
46	2016	30	28.3286	72.8061	916.8806	7.0551	1.6816
86	2016	30	24.4143	73.5306	864.0551	7.4765	3.0153
2	2016	30	24.4143	73.5306	864.0551	7.4765	3.0153
4	2016	30	26.7286	71.3571	897.9612	6.1480	2.1439
47	2016	30	26.7714	77.7041	955.1755	5.8827	0.4806
82	2016	30	25.2286	74.7245	873.0480	6.9806	3.0776
19	2016	30	28.5429	83.6429	957.2980	5.6531	1.2571
20	2016	30	23.6286	76.9490	853.0347	7.2061	3.0500
80	2016	30	25.2286	74.7245	873.0480	6.9806	3.0776
3	2016	30	27.4429	74.9286	941.5102	8.0929	1.3357
52	2016	30	25.3143	81.3469	899.9051	4.4857	3.8235
70	2016	30	28.2429	66.7653	905.9286	8.3020	2.5684
64	2016	30	18.7143	77.6429	776.1857	5.0684	2.4184
48	2016	30	29.0143	71.1122	927.7878	6.9622	1.1051
65	2016	30	25.3143	81.3469	899.9051	4.4857	3.8235
44	2016	30	28.2429	66.7653	905.9286	8.3020	2.5684
75	2016	30	18.7143	77.6429	776.1857	5.0684	2.4184
40	2016	30	28.5429	72.7449	942.1296	8.2020	2.4469
11	2016	30	23.7143	81.3265	876.3051	6.6071	1.6898
35	2016	30	28.1143	72.3673	935.2184	8.3224	1.5031

78	2016	30	25.9000	74.9286	897.1888	7.3112	1.6163
28	2016	30	28.1429	70.5816	925.5684	6.8133	1.9408
39	2016	30	25.3143	81.3469	899.9051	4.4857	3.8235
24	2016	30	28.2857	71.8163	935.6306	7.1082	1.7194
63	2016	30	28.5429	72.7449	942.1296	8.2020	2.4469
62	2016	30	25.7000	72.7857	871.9643	5.8367	2.2082
1	2016	30	25.2286	74.7245	873.0480	6.9806	3.0776
31	2016	31	21.1000	74.7653	845.9847	7.3316	1.3327
79	2016	31	26.7000	71.1735	965.7898	8.5531	1.6031
51	2016	31	25.1857	70.9592	936.6500	8.8459	1.5378
14	2016	31	23.0000	77.3163	896.7041	5.7673	2.2071
67	2016	31	22.9286	80.5408	901.1490	5.7071	2.9316
42	2016	31	22.9857	70.9388	874.4133	7.9163	2.9857
50	2016	31	24.4286	69.8571	899.8980	6.8776	1.7969
43	2016	31	22.9857	70.9388	874.4133	7.9163	2.9857
85	2016	31	24.5857	65.3163	907.9143	8.3837	2.3898
25	2016	31	26.8000	79.2041	973.7265	6.9469	1.0347
69	2016	31	25.1429	71.5714	937.3041	7.9612	1.6622
57	2016	31	22.8143	71.5000	885.5796	7.4163	3.5061
9	2016	31	21.8286	73.6429	854.3337	8.1643	2.7031
72	2016	31	22.8714	77.4082	877.4082	7.4031	1.4653
26	2016	31	23.2714	76.7245	867.2378	6.5510	1.7633
7	2016	31	22.2857	74.6224	859.4378	6.8327	1.8949
83	2016	31	26.9286	73.5918	942.6316	8.2755	1.3571
76	2016	31	24.7286	69.9490	918.7337	8.3806	1.6000
36	2016	31	24.3857	70.8061	926.9520	8.0602	1.8429
81	2016	31	25.1857	70.9592	936.6500	8.8459	1.5378
15	2016	31	26.0571	68.6020	929.8745	7.5184	1.1949
32	2016	31	22.9857	70.9388	874.4133	7.9163	2.9857
73	2016	31	26.4857	68.3469	958.1541	7.6367	1.2837
71	2016	31	24.3857	70.8061	926.9520	8.0602	1.8429
41	2016	31	22.7714	70.9490	873.6459	6.9867	2.0531
10	2016	31	26.1143	73.8163	960.8102	7.6561	1.0673
23	2016	31	17.5286	78.4286	777.5663	5.9347	2.2571
27	2016	31	22.2857	74.6224	859.4378	6.8327	1.8949
60	2016	31	25.1857	70.9592	936.6500	8.8459	1.5378
53	2016	31	21.8286	73.6429	854.3337	8.1643	2.7031
66	2016	31	23.0000	77.3163	896.7041	5.7673	2.2071
59	2016	31	22.8143	71.5000	885.5796	7.4163	3.5061
61	2016	31	26.4857	68.3469	958.1541	7.6367	1.2837
84	2016	31	26.4857	68.3469	958.1541	7.6367	1.2837
38	2016	31	22.8143	71.5000	885.5796	7.4163	3.5061
87	2016	31	23.7429	73.2959	898.4827	8.3173	1.4959
34	2016	31	22.8143	71.5000	885.5796	7.4163	3.5061
29	2016	31	25.1429	71.5714	937.3041	7.9612	1.6622
5	2016	31	20.5143	79.1735	833.1694	6.4918	1.4673
8	2016	31	21.8286	73.6429	854.3337	8.1643	2.7031
12	2016	31	20.5143	79.1735	833.1694	6.4918	1.4673
13	2016	31	26.9286	73.5918	942.6316	8.2755	1.3571
18	2016	31	25.6571	73.0510	960.1316	8.1316	1.3408

33	2016	31	24.4286	69.8571	899.8980	6.8776	1.7969
56	2016	31	26.8000	79.2041	973.7265	6.9469	1.0347
77	2016	31	26.0571	68.6020	929.8745	7.5184	1.1949
54	2016	31	20.5143	79.1735	833.1694	6.4918	1.4673
21	2016	31	24.4286	69.8571	899.8980	6.8776	1.7969
68	2016	31	26.7000	71.1735	965.7898	8.5531	1.6031
74	2016	31	26.4857	68.3469	958.1541	7.6367	1.2837
88	2016	31	22.9857	70.9388	874.4133	7.9163	2.9857
16	2016	31	24.7286	69.9490	918.7337	8.3806	1.6000
30	2016	31	23.0000	77.3163	896.7041	5.7673	2.2071
6	2016	31	26.7000	71.1735	965.7898	8.5531	1.6031
49	2016	31	25.1429	71.5714	937.3041	7.9612	1.6622
22	2016	31	22.9857	70.9388	874.4133	7.9163	2.9857
45	2016	31	19.8714	78.2857	818.6735	5.3990	1.7020
58	2016	31	25.1429	71.5714	937.3041	7.9612	1.6622
37	2016	31	26.7000	71.1735	965.7898	8.5531	1.6031
17	2016	31	22.9286	80.5408	901.1490	5.7071	2.9316
55	2016	31	22.8714	77.4082	877.4082	7.4031	1.4653
46	2016	31	24.7286	69.9490	918.7337	8.3806	1.6000
86	2016	31	22.4857	71.8571	865.6571	7.6735	2.5306
2	2016	31	22.4857	71.8571	865.6571	7.6735	2.5306
4	2016	31	24.4286	69.8571	899.8980	6.8776	1.7969
47	2016	31	25.4000	77.3878	956.3163	6.7490	0.5276
82	2016	31	22.9857	70.9388	874.4133	7.9163	2.9857
19	2016	31	26.1429	81.5918	958.0408	6.9500	1.1551
20	2016	31	21.8286	73.6429	854.3337	8.1643	2.7031
80	2016	31	22.9857	70.9388	874.4133	7.9163	2.9857
3	2016	31	26.9286	73.5918	942.6316	8.2755	1.3571
52	2016	31	22.9286	80.5408	901.1490	5.7071	2.9316
70	2016	31	24.5857	65.3163	907.9143	8.3837	2.3898
64	2016	31	17.5286	78.4286	777.5663	5.9347	2.2571
48	2016	31	26.0571	68.6020	929.8745	7.5184	1.1949
65	2016	31	22.9286	80.5408	901.1490	5.7071	2.9316
44	2016	31	24.5857	65.3163	907.9143	8.3837	2.3898
75	2016	31	17.5286	78.4286	777.5663	5.9347	2.2571
40	2016	31	25.7143	72.4286	943.0357	8.8959	1.8214
11	2016	31	22.8714	77.4082	877.4082	7.4031	1.4653
35	2016	31	25.1857	70.9592	936.6500	8.8459	1.5378
78	2016	31	23.7429	73.2959	898.4827	8.3173	1.4959
28	2016	31	24.3857	70.8061	926.9520	8.0602	1.8429
39	2016	31	22.9286	80.5408	901.1490	5.7071	2.9316
24	2016	31	25.1429	71.5714	937.3041	7.9612	1.6622
63	2016	31	25.7143	72.4286	943.0357	8.8959	1.8214
62	2016	31	22.7714	70.9490	873.6459	6.9867	2.0531
1	2016	31	22.9857	70.9388	874.4133	7.9163	2.9857
31	2016	32	21.8143	84.8265	845.9204	5.0561	1.0796
79	2016	32	27.0714	78.6939	965.2816	5.7112	1.5102
51	2016	32	26.1000	78.8878	935.7571	5.6469	1.4490
14	2016	32	23.8714	82.8469	895.2786	4.4429	1.6214
67	2016	32	24.0857	87.1735	899.9633	3.6816	2.2143

42	2016	32	23.7857	77.6633	873.5082	5.1592	2.5776
50	2016	32	25.3286	76.2653	899.7143	5.3622	1.4704
43	2016	32	23.7857	77.6633	873.5082	5.1592	2.5776
85	2016	32	25.9286	74.7653	907.9173	6.0898	1.5316
25	2016	32	27.3143	83.8265	972.3184	5.9367	1.0092
69	2016	32	26.7143	79.2857	936.6092	5.2755	1.5184
57	2016	32	23.7714	82.4388	885.0867	5.2704	2.3929
9	2016	32	22.5857	78.9286	853.4439	5.7745	2.2153
72	2016	32	23.3000	81.3265	876.2490	5.6908	1.2806
26	2016	32	22.5143	80.6633	866.1520	5.1000	1.4449
7	2016	32	22.6143	79.6429	858.3735	5.0163	1.4194
83	2016	32	25.9571	79.4286	941.3724	6.1857	1.1500
76	2016	32	26.1429	77.8163	918.3000	6.4694	1.3908
36	2016	32	26.1286	81.0102	925.9020	5.7235	1.5531
81	2016	32	26.1000	78.8878	935.7571	5.6469	1.4490
15	2016	32	26.7286	76.4796	929.8245	5.4469	0.9694
32	2016	32	23.7857	77.6633	873.5082	5.1592	2.5776
73	2016	32	27.9429	77.0816	957.8143	5.3990	0.9510
71	2016	32	26.1286	81.0102	925.9020	5.7235	1.5531
41	2016	32	23.8429	81.3571	873.2765	4.8459	1.3582
10	2016	32	26.7429	81.4694	959.8143	5.0031	0.8878
23	2016	32	18.2143	83.8776	777.2112	4.2724	1.7296
27	2016	32	22.6143	79.6429	858.3735	5.0163	1.4194
60	2016	32	26.1000	78.8878	935.7571	5.6469	1.4490
53	2016	32	22.5857	78.9286	853.4439	5.7745	2.2153
66	2016	32	23.8714	82.8469	895.2786	4.4429	1.6214
59	2016	32	23.7714	82.4388	885.0867	5.2704	2.3929
61	2016	32	27.9429	77.0816	957.8143	5.3990	0.9510
84	2016	32	27.9429	77.0816	957.8143	5.3990	0.9510
38	2016	32	23.7714	82.4388	885.0867	5.2704	2.3929
87	2016	32	24.0714	81.1633	897.4337	6.0704	1.3429
34	2016	32	23.7714	82.4388	885.0867	5.2704	2.3929
29	2016	32	26.7143	79.2857	936.6092	5.2755	1.5184
5	2016	32	21.4429	83.4388	832.3429	5.4806	1.2755
8	2016	32	22.5857	78.9286	853.4439	5.7745	2.2153
12	2016	32	21.4429	83.4388	832.3429	5.4806	1.2755
13	2016	32	25.9571	79.4286	941.3724	6.1857	1.1500
18	2016	32	26.9429	81.7551	959.8327	4.8837	1.0888
33	2016	32	25.3286	76.2653	899.7143	5.3622	1.4704
56	2016	32	27.3143	83.8265	972.3184	5.9367	1.0092
77	2016	32	26.7286	76.4796	929.8245	5.4469	0.9694
54	2016	32	21.4429	83.4388	832.3429	5.4806	1.2755
21	2016	32	25.3286	76.2653	899.7143	5.3622	1.4704
68	2016	32	27.0714	78.6939	965.2816	5.7112	1.5102
74	2016	32	27.9429	77.0816	957.8143	5.3990	0.9510
88	2016	32	23.7857	77.6633	873.5082	5.1592	2.5776
16	2016	32	26.1429	77.8163	918.3000	6.4694	1.3908
30	2016	32	23.8714	82.8469	895.2786	4.4429	1.6214
6	2016	32	27.0714	78.6939	965.2816	5.7112	1.5102
49	2016	32	26.7143	79.2857	936.6092	5.2755	1.5184

22	2016	32	23.7857	77.6633	873.5082	5.1592	2.5776
45	2016	32	20.4286	82.6429	817.8398	4.4173	1.1092
58	2016	32	26.7143	79.2857	936.6092	5.2755	1.5184
37	2016	32	27.0714	78.6939	965.2816	5.7112	1.5102
17	2016	32	24.0857	87.1735	899.9633	3.6816	2.2143
55	2016	32	23.3000	81.3265	876.2490	5.6908	1.2806
46	2016	32	26.1429	77.8163	918.3000	6.4694	1.3908
86	2016	32	23.0429	78.7653	865.1531	5.1888	1.4000
2	2016	32	23.0429	78.7653	865.1531	5.1888	1.4000
4	2016	32	25.3286	76.2653	899.7143	5.3622	1.4704
47	2016	32	25.7000	81.3571	955.0510	5.5357	0.5276
82	2016	32	23.7857	77.6633	873.5082	5.1592	2.5776
19	2016	32	27.2000	85.8163	956.5153	5.8143	1.0255
20	2016	32	22.5857	78.9286	853.4439	5.7745	2.2153
80	2016	32	23.7857	77.6633	873.5082	5.1592	2.5776
3	2016	32	25.9571	79.4286	941.3724	6.1857	1.1500
52	2016	32	24.0857	87.1735	899.9633	3.6816	2.2143
70	2016	32	25.9286	74.7653	907.9173	6.0898	1.5316
64	2016	32	18.2143	83.8776	777.2112	4.2724	1.7296
48	2016	32	26.7286	76.4796	929.8245	5.4469	0.9694
65	2016	32	24.0857	87.1735	899.9633	3.6816	2.2143
44	2016	32	25.9286	74.7653	907.9173	6.0898	1.5316
75	2016	32	18.2143	83.8776	777.2112	4.2724	1.7296
40	2016	32	26.5286	80.0000	941.6449	6.2224	1.4347
11	2016	32	23.3000	81.3265	876.2490	5.6908	1.2806
35	2016	32	26.1000	78.8878	935.7571	5.6469	1.4490
78	2016	32	24.0714	81.1633	897.4337	6.0704	1.3429
28	2016	32	26.1286	81.0102	925.9020	5.7235	1.5531
39	2016	32	24.0857	87.1735	899.9633	3.6816	2.2143
24	2016	32	26.7143	79.2857	936.6092	5.2755	1.5184
63	2016	32	26.5286	80.0000	941.6449	6.2224	1.4347
62	2016	32	23.8429	81.3571	873.2765	4.8459	1.3582
1	2016	32	23.7857	77.6633	873.5082	5.1592	2.5776
31	2016	33	22.7286	86.8571	844.9082	5.0459	0.9510
79	2016	33	29.5714	81.2041	963.5755	5.8520	1.1378
51	2016	33	26.5286	82.8265	934.0449	4.8653	1.3000
14	2016	33	24.4286	84.9592	893.3929	4.2378	1.4827
67	2016	33	24.7143	88.1531	898.2510	2.8286	2.4959
42	2016	33	23.7857	82.7143	872.0663	4.5908	2.4388
50	2016	33	26.2857	77.5204	898.5347	6.1520	1.5378
43	2016	33	23.7857	82.7143	872.0663	4.5908	2.4388
85	2016	33	26.5571	77.7245	906.7561	6.2745	1.1520
25	2016	33	27.7857	87.3265	970.1959	5.0796	1.0969
69	2016	33	27.2571	81.2755	934.9643	5.3867	1.3837
57	2016	33	23.8286	89.1224	883.8112	5.1816	2.0133
9	2016	33	22.4714	82.9082	852.0571	5.5204	2.2510
72	2016	33	23.9286	82.7551	874.5745	5.2582	1.6663
26	2016	33	23.0286	84.1531	864.5367	4.3133	1.3827
7	2016	33	22.7571	82.0102	856.8306	4.7673	1.3408
83	2016	33	26.4857	87.1122	939.4929	4.8327	1.0510

76	2016	33	26.8857	79.8469	916.8816	7.0082	1.3357
36	2016	33	26.6571	83.4388	924.1459	5.3908	1.6041
81	2016	33	26.5286	82.8265	934.0449	4.8653	1.3000
15	2016	33	28.2429	79.1531	928.5500	5.8837	0.8541
32	2016	33	23.7857	82.7143	872.0663	4.5908	2.4388
73	2016	33	29.5429	78.2245	956.2082	5.8724	0.9418
71	2016	33	26.6571	83.4388	924.1459	5.3908	1.6041
41	2016	33	23.8571	86.1429	872.1367	5.1061	1.0541
10	2016	33	28.1429	83.5918	957.9265	4.7806	0.8602
23	2016	33	19.0429	84.7347	776.2653	4.3469	1.7286
27	2016	33	22.7571	82.0102	856.8306	4.7673	1.3408
60	2016	33	26.5286	82.8265	934.0449	4.8653	1.3000
53	2016	33	22.4714	82.9082	852.0571	5.5204	2.2510
66	2016	33	24.4286	84.9592	893.3929	4.2378	1.4827
59	2016	33	23.8286	89.1224	883.8112	5.1816	2.0133
61	2016	33	29.5429	78.2245	956.2082	5.8724	0.9418
84	2016	33	29.5429	78.2245	956.2082	5.8724	0.9418
38	2016	33	23.8286	89.1224	883.8112	5.1816	2.0133
87	2016	33	24.6429	85.5204	895.7786	5.4633	1.6622
34	2016	33	23.8286	89.1224	883.8112	5.1816	2.0133
29	2016	33	27.2571	81.2755	934.9643	5.3867	1.3837
5	2016	33	21.0714	83.6633	830.9867	5.6388	1.4847
8	2016	33	22.4714	82.9082	852.0571	5.5204	2.2510
12	2016	33	21.0714	83.6633	830.9867	5.6388	1.4847
13	2016	33	26.4857	87.1122	939.4929	4.8327	1.0510
18	2016	33	29.2143	83.0510	958.2408	5.2673	1.0878
33	2016	33	26.2857	77.5204	898.5347	6.1520	1.5378
56	2016	33	27.7857	87.3265	970.1959	5.0796	1.0969
77	2016	33	28.2429	79.1531	928.5500	5.8837	0.8541
54	2016	33	21.0714	83.6633	830.9867	5.6388	1.4847
21	2016	33	26.2857	77.5204	898.5347	6.1520	1.5378
68	2016	33	29.5714	81.2041	963.5755	5.8520	1.1378
74	2016	33	29.5429	78.2245	956.2082	5.8724	0.9418
88	2016	33	23.7857	82.7143	872.0663	4.5908	2.4388
16	2016	33	26.8857	79.8469	916.8816	7.0082	1.3357
30	2016	33	24.4286	84.9592	893.3929	4.2378	1.4827
6	2016	33	29.5714	81.2041	963.5755	5.8520	1.1378
49	2016	33	27.2571	81.2755	934.9643	5.3867	1.3837
22	2016	33	23.7857	82.7143	872.0663	4.5908	2.4388
45	2016	33	20.7000	83.0102	816.4969	4.7092	1.1051
58	2016	33	27.2571	81.2755	934.9643	5.3867	1.3837
37	2016	33	29.5714	81.2041	963.5755	5.8520	1.1378
17	2016	33	24.7143	88.1531	898.2510	2.8286	2.4959
55	2016	33	23.9286	82.7551	874.5745	5.2582	1.6663
46	2016	33	26.8857	79.8469	916.8816	7.0082	1.3357
86	2016	33	22.8714	83.5510	863.9612	4.7388	0.8337
2	2016	33	22.8714	83.5510	863.9612	4.7388	0.8337
4	2016	33	26.2857	77.5204	898.5347	6.1520	1.5378
47	2016	33	26.5143	83.5918	953.0204	5.4786	0.4561
82	2016	33	23.7857	82.7143	872.0663	4.5908	2.4388

19	2016	33	28.0143	87.0714	954.3204	5.4408	1.0745
20	2016	33	22.4714	82.9082	852.0571	5.5204	2.2510
80	2016	33	23.7857	82.7143	872.0663	4.5908	2.4388
3	2016	33	26.4857	87.1122	939.4929	4.8327	1.0510
52	2016	33	24.7143	88.1531	898.2510	2.8286	2.4959
70	2016	33	26.5571	77.7245	906.7561	6.2745	1.1520
64	2016	33	19.0429	84.7347	776.2653	4.3469	1.7286
48	2016	33	28.2429	79.1531	928.5500	5.8837	0.8541
65	2016	33	24.7143	88.1531	898.2510	2.8286	2.4959
44	2016	33	26.5571	77.7245	906.7561	6.2745	1.1520
75	2016	33	19.0429	84.7347	776.2653	4.3469	1.7286
40	2016	33	26.9429	83.8571	939.6724	5.3337	1.3776
11	2016	33	23.9286	82.7551	874.5745	5.2582	1.6663
35	2016	33	26.5286	82.8265	934.0449	4.8653	1.3000
78	2016	33	24.6429	85.5204	895.7786	5.4633	1.6622
28	2016	33	26.6571	83.4388	924.1459	5.3908	1.6041
39	2016	33	24.7143	88.1531	898.2510	2.8286	2.4959
24	2016	33	27.2571	81.2755	934.9643	5.3867	1.3837
63	2016	33	26.9429	83.8571	939.6724	5.3337	1.3776
62	2016	33	23.8571	86.1429	872.1367	5.1061	1.0541
1	2016	33	23.7857	82.7143	872.0663	4.5908	2.4388
31	2016	34	22.6714	81.1633	845.3092	6.5745	0.9347
79	2016	34	27.6000	75.4592	963.9296	7.2755	1.1827
51	2016	34	25.4571	80.6020	934.5296	5.5357	1.2653
14	2016	34	23.2857	83.0306	893.8694	4.8490	1.3041
67	2016	34	23.8286	84.7551	898.7939	2.9010	2.5276
42	2016	34	23.3429	80.6837	872.6306	5.4500	2.3133
50	2016	34	25.2286	72.9082	898.8378	7.6796	1.6214
43	2016	34	23.3429	80.6837	872.6306	5.4500	2.3133
85	2016	34	26.1000	72.7143	907.0224	7.4235	1.2776
25	2016	34	27.5000	85.8163	970.5939	4.7745	1.1898
69	2016	34	26.2429	78.7347	935.4378	6.4888	1.2245
57	2016	34	23.4857	87.0510	884.3000	5.8724	1.7755
9	2016	34	22.5714	80.5510	852.5806	6.4612	2.2847
72	2016	34	23.3286	80.3265	875.0663	5.9255	1.8061
26	2016	34	23.3143	83.4184	864.9816	4.5031	1.4796
7	2016	34	22.6000	81.1020	857.2929	5.3837	1.4561
83	2016	34	26.7857	85.9898	939.8602	5.6153	1.1041
76	2016	34	25.5714	75.6633	917.3582	7.8663	1.4449
36	2016	34	25.7429	79.6837	924.6653	5.9347	1.6663
81	2016	34	25.4571	80.6020	934.5296	5.5357	1.2653
15	2016	34	27.1857	73.2755	928.8490	7.6194	0.9755
32	2016	34	23.3429	80.6837	872.6306	5.4500	2.3133
73	2016	34	28.0714	73.0408	956.5276	7.1714	1.0786
71	2016	34	25.7429	79.6837	924.6653	5.9347	1.6663
41	2016	34	23.9000	81.1531	872.5929	6.6847	1.1602
10	2016	34	26.2286	80.0714	958.3357	5.5765	0.9398
23	2016	34	19.1857	81.9388	776.7571	5.3755	1.7816
27	2016	34	22.6000	81.1020	857.2929	5.3837	1.4561
60	2016	34	25.4571	80.6020	934.5296	5.5357	1.2653

53	2016	34	22.5714	80.5510	852.5806	6.4612	2.2847
66	2016	34	23.2857	83.0306	893.8694	4.8490	1.3041
59	2016	34	23.4857	87.0510	884.3000	5.8724	1.7755
61	2016	34	28.0714	73.0408	956.5276	7.1714	1.0786
84	2016	34	28.0714	73.0408	956.5276	7.1714	1.0786
38	2016	34	23.4857	87.0510	884.3000	5.8724	1.7755
87	2016	34	24.3000	83.1122	896.2939	6.1296	1.8061
34	2016	34	23.4857	87.0510	884.3000	5.8724	1.7755
29	2016	34	26.2429	78.7347	935.4378	6.4888	1.2245
5	2016	34	21.2429	82.4694	831.4929	5.9969	1.6071
8	2016	34	22.5714	80.5510	852.5806	6.4612	2.2847
12	2016	34	21.2429	82.4694	831.4929	5.9969	1.6071
13	2016	34	26.7857	85.9898	939.8602	5.6153	1.1041
18	2016	34	27.2143	76.6633	958.5867	7.0571	1.1704
33	2016	34	25.2286	72.9082	898.8378	7.6796	1.6214
56	2016	34	27.5000	85.8163	970.5939	4.7745	1.1898
77	2016	34	27.1857	73.2755	928.8490	7.6194	0.9755
54	2016	34	21.2429	82.4694	831.4929	5.9969	1.6071
21	2016	34	25.2286	72.9082	898.8378	7.6796	1.6214
68	2016	34	27.6000	75.4592	963.9296	7.2755	1.1827
74	2016	34	28.0714	73.0408	956.5276	7.1714	1.0786
88	2016	34	23.3429	80.6837	872.6306	5.4500	2.3133
16	2016	34	25.5714	75.6633	917.3582	7.8663	1.4449
30	2016	34	23.2857	83.0306	893.8694	4.8490	1.3041
6	2016	34	27.6000	75.4592	963.9296	7.2755	1.1827
49	2016	34	26.2429	78.7347	935.4378	6.4888	1.2245
22	2016	34	23.3429	80.6837	872.6306	5.4500	2.3133
45	2016	34	20.5714	80.6735	816.9857	5.7582	1.2653
58	2016	34	26.2429	78.7347	935.4378	6.4888	1.2245
37	2016	34	27.6000	75.4592	963.9296	7.2755	1.1827
17	2016	34	23.8286	84.7551	898.7939	2.9010	2.5276
55	2016	34	23.3286	80.3265	875.0663	5.9255	1.8061
46	2016	34	25.5714	75.6633	917.3582	7.8663	1.4449
86	2016	34	23.3571	80.0714	864.4490	5.7980	0.8765
2	2016	34	23.3571	80.0714	864.4490	5.7980	0.8765
4	2016	34	25.2286	72.9082	898.8378	7.6796	1.6214
47	2016	34	25.4286	82.5816	953.3908	5.7949	0.4429
82	2016	34	23.3429	80.6837	872.6306	5.4500	2.3133
19	2016	34	27.0286	84.9184	954.6490	5.5765	1.1449
20	2016	34	22.5714	80.5510	852.5806	6.4612	2.2847
80	2016	34	23.3429	80.6837	872.6306	5.4500	2.3133
3	2016	34	26.7857	85.9898	939.8602	5.6153	1.1041
52	2016	34	23.8286	84.7551	898.7939	2.9010	2.5276
70	2016	34	26.1000	72.7143	907.0224	7.4235	1.2776
64	2016	34	19.1857	81.9388	776.7571	5.3755	1.7816
48	2016	34	27.1857	73.2755	928.8490	7.6194	0.9755
65	2016	34	23.8286	84.7551	898.7939	2.9010	2.5276
44	2016	34	26.1000	72.7143	907.0224	7.4235	1.2776
75	2016	34	19.1857	81.9388	776.7571	5.3755	1.7816
40	2016	34	25.2143	82.5612	940.1286	5.7286	1.2153

11	2016	34	23.3286	80.3265	875.0663	5.9255	1.8061
35	2016	34	25.4571	80.6020	934.5296	5.5357	1.2653
78	2016	34	24.3000	83.1122	896.2939	6.1296	1.8061
28	2016	34	25.7429	79.6837	924.6653	5.9347	1.6663
39	2016	34	23.8286	84.7551	898.7939	2.9010	2.5276
24	2016	34	26.2429	78.7347	935.4378	6.4888	1.2245
63	2016	34	25.2143	82.5612	940.1286	5.7286	1.2153
62	2016	34	23.9000	81.1531	872.5929	6.6847	1.1602
1	2016	34	23.3429	80.6837	872.6306	5.4500	2.3133
31	2016	35	18.5000	80.6633	847.0878	4.7398	0.9786
79	2016	35	24.1857	71.6633	967.0316	6.2398	1.3480
51	2016	35	22.8286	75.6735	937.3765	5.9092	1.3235
14	2016	35	21.6143	79.4082	896.4561	4.6602	1.1704
67	2016	35	21.1571	81.2143	901.3000	3.4694	2.2051
42	2016	35	20.6000	77.7653	874.8571	4.7071	2.1980
50	2016	35	20.5857	73.9898	901.0245	5.2347	1.4827
43	2016	35	20.6000	77.7653	874.8571	4.7071	2.1980
85	2016	35	21.3429	73.4694	909.3898	5.5204	1.3857
25	2016	35	25.1429	79.6837	973.8490	5.6153	1.2469
69	2016	35	22.9571	76.0714	938.2612	5.6867	1.2000
57	2016	35	20.2000	84.8776	886.5378	5.1051	1.5255
9	2016	35	19.5857	78.8061	854.6245	4.5765	1.9418
72	2016	35	20.7714	80.3571	877.4531	4.8153	1.2561
26	2016	35	21.4143	80.9184	867.1898	5.0041	1.5367
7	2016	35	20.2714	80.9184	859.4347	4.1245	1.5398
83	2016	35	24.9000	80.3367	942.6510	5.7122	1.1469
76	2016	35	21.8429	75.5000	919.9327	5.8041	1.4898
36	2016	35	23.2429	75.2449	927.4071	5.8776	1.6316
81	2016	35	22.8286	75.6735	937.3765	5.9092	1.3235
15	2016	35	22.1143	72.1327	931.4571	5.9082	1.0378
32	2016	35	20.6000	77.7653	874.8571	4.7071	2.1980
73	2016	35	24.0571	71.5816	959.5449	5.9173	1.0898
71	2016	35	23.2429	75.2449	927.4071	5.8776	1.6316
41	2016	35	20.3286	80.2347	874.6510	4.8010	1.1102
10	2016	35	23.3714	76.7041	961.5020	5.7204	0.9561
23	2016	35	15.0857	82.4490	778.0827	3.6816	1.8561
27	2016	35	20.2714	80.9184	859.4347	4.1245	1.5398
60	2016	35	22.8286	75.6735	937.3765	5.9092	1.3235
53	2016	35	19.5857	78.8061	854.6245	4.5765	1.9418
66	2016	35	21.6143	79.4082	896.4561	4.6602	1.1704
59	2016	35	20.2000	84.8776	886.5378	5.1051	1.5255
61	2016	35	24.0571	71.5816	959.5449	5.9173	1.0898
84	2016	35	24.0571	71.5816	959.5449	5.9173	1.0898
38	2016	35	20.2000	84.8776	886.5378	5.1051	1.5255
87	2016	35	21.7571	80.0918	898.7878	5.5102	1.5276
34	2016	35	20.2000	84.8776	886.5378	5.1051	1.5255
29	2016	35	22.9571	76.0714	938.2612	5.6867	1.2000
5	2016	35	18.6286	82.1837	833.3235	4.7122	1.5520
8	2016	35	19.5857	78.8061	854.6245	4.5765	1.9418
12	2016	35	18.6286	82.1837	833.3235	4.7122	1.5520

13	2016	35	24.9000	80.3367	942.6510	5.7122	1.1469
18	2016	35	23.7286	73.1939	961.5786	5.6990	1.1102
33	2016	35	20.5857	73.9898	901.0245	5.2347	1.4827
56	2016	35	25.1429	79.6837	973.8490	5.6153	1.2469
77	2016	35	22.1143	72.1327	931.4571	5.9082	1.0378
54	2016	35	18.6286	82.1837	833.3235	4.7122	1.5520
21	2016	35	20.5857	73.9898	901.0245	5.2347	1.4827
68	2016	35	24.1857	71.6633	967.0316	6.2398	1.3480
74	2016	35	24.0571	71.5816	959.5449	5.9173	1.0898
88	2016	35	20.6000	77.7653	874.8571	4.7071	2.1980
16	2016	35	21.8429	75.5000	919.9327	5.8041	1.4898
30	2016	35	21.6143	79.4082	896.4561	4.6602	1.1704
6	2016	35	24.1857	71.6633	967.0316	6.2398	1.3480
49	2016	35	22.9571	76.0714	938.2612	5.6867	1.2000
22	2016	35	20.6000	77.7653	874.8571	4.7071	2.1980
45	2016	35	18.5571	80.1327	818.7224	4.6939	1.3143
58	2016	35	22.9571	76.0714	938.2612	5.6867	1.2000
37	2016	35	24.1857	71.6633	967.0316	6.2398	1.3480
17	2016	35	21.1571	81.2143	901.3000	3.4694	2.2051
55	2016	35	20.7714	80.3571	877.4531	4.8153	1.2561
46	2016	35	21.8429	75.5000	919.9327	5.8041	1.4898
86	2016	35	19.8143	77.2653	866.4459	4.3643	1.0296
2	2016	35	19.8143	77.2653	866.4459	4.3643	1.0296
4	2016	35	20.5857	73.9898	901.0245	5.2347	1.4827
47	2016	35	23.6857	81.0714	956.4327	5.0296	0.4276
82	2016	35	20.6000	77.7653	874.8571	4.7071	2.1980
19	2016	35	24.8143	81.6122	957.8408	5.6316	1.1949
20	2016	35	19.5857	78.8061	854.6245	4.5765	1.9418
80	2016	35	20.6000	77.7653	874.8571	4.7071	2.1980
3	2016	35	24.9000	80.3367	942.6510	5.7122	1.1469
52	2016	35	21.1571	81.2143	901.3000	3.4694	2.2051
70	2016	35	21.3429	73.4694	909.3898	5.5204	1.3857
64	2016	35	15.0857	82.4490	778.0827	3.6816	1.8561
48	2016	35	22.1143	72.1327	931.4571	5.9082	1.0378
65	2016	35	21.1571	81.2143	901.3000	3.4694	2.2051
44	2016	35	21.3429	73.4694	909.3898	5.5204	1.3857
75	2016	35	15.0857	82.4490	778.0827	3.6816	1.8561
40	2016	35	22.8000	80.0816	943.1796	6.3929	1.0684
11	2016	35	20.7714	80.3571	877.4531	4.8153	1.2561
35	2016	35	22.8286	75.6735	937.3765	5.9092	1.3235
78	2016	35	21.7571	80.0918	898.7878	5.5102	1.5276
28	2016	35	23.2429	75.2449	927.4071	5.8776	1.6316
39	2016	35	21.1571	81.2143	901.3000	3.4694	2.2051
24	2016	35	22.9571	76.0714	938.2612	5.6867	1.2000
63	2016	35	22.8000	80.0816	943.1796	6.3929	1.0684
62	2016	35	20.3286	80.2347	874.6510	4.8010	1.1102
1	2016	35	20.6000	77.7653	874.8571	4.7071	2.1980
31	2016	36	17.4857	86.0816	847.8949	1.6031	0.9306
79	2016	36	24.1429	70.7449	968.4224	4.6020	1.3041
51	2016	36	22.8286	70.4388	938.5245	5.3969	1.4092

14	2016	36	21.9143	74.7755	897.2347	3.8776	1.2408
67	2016	36	21.4143	77.3673	902.1684	3.3102	2.4357
42	2016	36	20.2143	76.3571	875.6633	3.1071	2.2724
50	2016	36	19.7429	79.7755	902.3776	2.0612	1.3959
43	2016	36	20.2143	76.3571	875.6633	3.1071	2.2724
85	2016	36	20.0143	81.8776	910.9214	2.1571	1.2082
25	2016	36	25.7714	75.7551	975.0735	6.4776	1.3439
69	2016	36	22.6571	74.7245	939.5704	3.7969	1.3010
57	2016	36	18.9714	86.2755	887.6082	3.0092	1.5847
9	2016	36	19.1286	78.5816	855.2755	2.0561	1.8459
72	2016	36	20.9000	79.4286	878.2276	2.9806	1.2245
26	2016	36	20.9714	79.3980	867.8347	3.7398	1.4745
7	2016	36	20.0286	80.6837	860.0745	1.9745	1.4041
83	2016	36	25.1571	76.3265	943.6582	4.2296	1.1857
76	2016	36	20.9000	77.5714	921.2367	3.6092	1.3378
36	2016	36	23.1714	70.3163	928.5112	5.3633	1.6969
81	2016	36	22.8286	70.4388	938.5245	5.3969	1.4092
15	2016	36	20.9286	79.6020	933.2276	2.7337	0.8694
32	2016	36	20.2143	76.3571	875.6633	3.1071	2.2724
73	2016	36	23.2857	73.2143	961.2347	3.4745	1.0388
71	2016	36	23.1714	70.3163	928.5112	5.3633	1.6969
41	2016	36	19.2714	83.3673	875.6276	2.1663	0.9163
10	2016	36	23.6714	73.5000	962.8071	5.5520	1.0041
23	2016	36	14.1000	85.7653	778.1796	1.0306	1.7296
27	2016	36	20.0286	80.6837	860.0745	1.9745	1.4041
60	2016	36	22.8286	70.4388	938.5245	5.3969	1.4092
53	2016	36	19.1286	78.5816	855.2755	2.0561	1.8459
66	2016	36	21.9143	74.7755	897.2347	3.8776	1.2408
59	2016	36	18.9714	86.2755	887.6082	3.0092	1.5847
61	2016	36	23.2857	73.2143	961.2347	3.4745	1.0388
84	2016	36	23.2857	73.2143	961.2347	3.4745	1.0388
38	2016	36	18.9714	86.2755	887.6082	3.0092	1.5847
87	2016	36	21.8000	77.3878	899.6847	3.6745	1.5276
34	2016	36	18.9714	86.2755	887.6082	3.0092	1.5847
29	2016	36	22.6571	74.7245	939.5704	3.7969	1.3010
5	2016	36	18.1857	82.7245	833.7296	2.2959	1.2673
8	2016	36	19.1286	78.5816	855.2755	2.0561	1.8459
12	2016	36	18.1857	82.7245	833.7296	2.2959	1.2673
13	2016	36	25.1571	76.3265	943.6582	4.2296	1.1857
18	2016	36	23.7000	72.2755	962.9541	3.7541	1.0061
33	2016	36	19.7429	79.7755	902.3776	2.0612	1.3959
56	2016	36	25.7714	75.7551	975.0735	6.4776	1.3439
77	2016	36	20.9286	79.6020	933.2276	2.7337	0.8694
54	2016	36	18.1857	82.7245	833.7296	2.2959	1.2673
21	2016	36	19.7429	79.7755	902.3776	2.0612	1.3959
68	2016	36	24.1429	70.7449	968.4224	4.6020	1.3041
74	2016	36	23.2857	73.2143	961.2347	3.4745	1.0388
88	2016	36	20.2143	76.3571	875.6633	3.1071	2.2724
16	2016	36	20.9000	77.5714	921.2367	3.6092	1.3378
30	2016	36	21.9143	74.7755	897.2347	3.8776	1.2408

6	2016	36	24.1429	70.7449	968.4224	4.6020	1.3041
49	2016	36	22.6571	74.7245	939.5704	3.7969	1.3010
22	2016	36	20.2143	76.3571	875.6633	3.1071	2.2724
45	2016	36	17.8429	82.2857	819.0929	2.1551	1.2704
58	2016	36	22.6571	74.7245	939.5704	3.7969	1.3010
37	2016	36	24.1429	70.7449	968.4224	4.6020	1.3041
17	2016	36	21.4143	77.3673	902.1684	3.3102	2.4357
55	2016	36	20.9000	79.4286	878.2276	2.9806	1.2245
46	2016	36	20.9000	77.5714	921.2367	3.6092	1.3378
86	2016	36	19.2571	78.7755	867.2827	1.8847	0.9816
2	2016	36	19.2571	78.7755	867.2827	1.8847	0.9816
4	2016	36	19.7429	79.7755	902.3776	2.0612	1.3959
47	2016	36	24.7429	78.0816	957.4786	4.4949	0.4092
82	2016	36	20.2143	76.3571	875.6633	3.1071	2.2724
19	2016	36	26.0000	76.1939	958.9061	5.9633	1.1561
20	2016	36	19.1286	78.5816	855.2755	2.0561	1.8459
80	2016	36	20.2143	76.3571	875.6633	3.1071	2.2724
3	2016	36	25.1571	76.3265	943.6582	4.2296	1.1857
52	2016	36	21.4143	77.3673	902.1684	3.3102	2.4357
70	2016	36	20.0143	81.8776	910.9214	2.1571	1.2082
64	2016	36	14.1000	85.7653	778.1796	1.0306	1.7296
48	2016	36	20.9286	79.6020	933.2276	2.7337	0.8694
65	2016	36	21.4143	77.3673	902.1684	3.3102	2.4357
44	2016	36	20.0143	81.8776	910.9214	2.1571	1.2082
75	2016	36	14.1000	85.7653	778.1796	1.0306	1.7296
40	2016	36	23.5286	76.6735	944.2714	6.7582	1.1388
11	2016	36	20.9000	79.4286	878.2276	2.9806	1.2245
35	2016	36	22.8286	70.4388	938.5245	5.3969	1.4092
78	2016	36	21.8000	77.3878	899.6847	3.6745	1.5276
28	2016	36	23.1714	70.3163	928.5112	5.3633	1.6969
39	2016	36	21.4143	77.3673	902.1684	3.3102	2.4357
24	2016	36	22.6571	74.7245	939.5704	3.7969	1.3010
63	2016	36	23.5286	76.6735	944.2714	6.7582	1.1388
62	2016	36	19.2714	83.3673	875.6276	2.1663	0.9163
1	2016	36	20.2143	76.3571	875.6633	3.1071	2.2724
31	2016	37	19.1286	86.0204	849.1000	2.2296	0.9224
79	2016	37	24.9286	72.0000	969.7786	4.5490	1.1837
51	2016	37	22.4286	72.4490	939.8490	4.6704	1.2592
14	2016	37	21.4000	75.7755	898.3194	2.9633	1.3265
67	2016	37	21.1286	78.2755	903.3163	2.6561	2.5867
42	2016	37	20.6571	76.0918	876.8449	2.9735	2.3531
50	2016	37	21.6714	78.5816	903.7520	3.0378	1.3776
43	2016	37	20.6571	76.0918	876.8449	2.9735	2.3531
85	2016	37	22.6429	81.5204	912.2449	3.1327	1.1061
25	2016	37	25.2571	78.3776	976.4153	5.7745	1.2837
69	2016	37	23.2857	75.2347	940.8745	3.4214	1.3908
57	2016	37	20.6857	87.1224	888.8806	2.8724	1.5735
9	2016	37	19.4143	79.0306	856.4071	2.1592	1.7806
72	2016	37	20.8857	78.7041	879.3939	3.1429	1.3408
26	2016	37	20.8857	80.7857	868.9990	2.9990	1.3092

7	2016	37	19.9714	81.4490	861.2184	1.9663	1.1908
83	2016	37	24.7714	75.9796	945.1490	3.4245	1.1327
76	2016	37	22.6714	77.1429	922.5510	4.3755	1.3000
36	2016	37	23.1286	71.7551	929.8439	4.7776	1.5265
81	2016	37	22.4286	72.4490	939.8490	4.6704	1.2592
15	2016	37	23.1143	81.0612	934.6878	3.4857	0.6653
32	2016	37	20.6571	76.0918	876.8449	2.9735	2.3531
73	2016	37	25.0143	74.1939	962.6612	3.8469	0.9755
71	2016	37	23.1286	71.7551	929.8439	4.7776	1.5265
41	2016	37	20.8571	82.8469	876.8663	2.6561	0.8888
10	2016	37	23.5429	74.1531	964.1337	5.2214	1.0031
23	2016	37	15.2571	84.9694	779.1010	2.2010	1.4163
27	2016	37	19.9714	81.4490	861.2184	1.9663	1.1908
60	2016	37	22.4286	72.4490	939.8490	4.6704	1.2592
53	2016	37	19.4143	79.0306	856.4071	2.1592	1.7806
66	2016	37	21.4000	75.7755	898.3194	2.9633	1.3265
59	2016	37	20.6857	87.1224	888.8806	2.8724	1.5735
61	2016	37	25.0143	74.1939	962.6612	3.8469	0.9755
84	2016	37	25.0143	74.1939	962.6612	3.8469	0.9755
38	2016	37	20.6857	87.1224	888.8806	2.8724	1.5735
87	2016	37	22.2857	77.3980	900.8908	3.3735	1.6102
34	2016	37	20.6857	87.1224	888.8806	2.8724	1.5735
29	2016	37	23.2857	75.2347	940.8745	3.4214	1.3908
5	2016	37	18.1286	82.8571	834.7745	2.5245	1.1602
8	2016	37	19.4143	79.0306	856.4071	2.1592	1.7806
12	2016	37	18.1286	82.8571	834.7745	2.5245	1.1602
13	2016	37	24.7714	75.9796	945.1490	3.4245	1.1327
18	2016	37	24.1714	73.3878	964.3265	4.3316	0.9388
33	2016	37	21.6714	78.5816	903.7520	3.0378	1.3776
56	2016	37	25.2571	78.3776	976.4153	5.7745	1.2837
77	2016	37	23.1143	81.0612	934.6878	3.4857	0.6653
54	2016	37	18.1286	82.8571	834.7745	2.5245	1.1602
21	2016	37	21.6714	78.5816	903.7520	3.0378	1.3776
68	2016	37	24.9286	72.0000	969.7786	4.5490	1.1837
74	2016	37	25.0143	74.1939	962.6612	3.8469	0.9755
88	2016	37	20.6571	76.0918	876.8449	2.9735	2.3531
16	2016	37	22.6714	77.1429	922.5510	4.3755	1.3000
30	2016	37	21.4000	75.7755	898.3194	2.9633	1.3265
6	2016	37	24.9286	72.0000	969.7786	4.5490	1.1837
49	2016	37	23.2857	75.2347	940.8745	3.4214	1.3908
22	2016	37	20.6571	76.0918	876.8449	2.9735	2.3531
45	2016	37	17.8429	82.8061	820.1643	1.8112	1.1755
58	2016	37	23.2857	75.2347	940.8745	3.4214	1.3908
37	2016	37	24.9286	72.0000	969.7786	4.5490	1.1837
17	2016	37	21.1286	78.2755	903.3163	2.6561	2.5867
55	2016	37	20.8857	78.7041	879.3939	3.1429	1.3408
46	2016	37	22.6714	77.1429	922.5510	4.3755	1.3000
86	2016	37	19.8143	79.8163	868.5071	2.3133	0.8061
2	2016	37	19.8143	79.8163	868.5071	2.3133	0.8061
4	2016	37	21.6714	78.5816	903.7520	3.0378	1.3776

47	2016	37	24.0286	77.6122	958.7357	4.1102	0.3173
82	2016	37	20.6571	76.0918	876.8449	2.9735	2.3531
19	2016	37	25.0000	75.1633	960.0867	5.2245	1.1449
20	2016	37	19.4143	79.0306	856.4071	2.1592	1.7806
80	2016	37	20.6571	76.0918	876.8449	2.9735	2.3531
3	2016	37	24.7714	75.9796	945.1490	3.4245	1.1327
52	2016	37	21.1286	78.2755	903.3163	2.6561	2.5867
70	2016	37	22.6429	81.5204	912.2449	3.1327	1.1061
64	2016	37	15.2571	84.9694	779.1010	2.2010	1.4163
48	2016	37	23.1143	81.0612	934.6878	3.4857	0.6653
65	2016	37	21.1286	78.2755	903.3163	2.6561	2.5867
44	2016	37	22.6429	81.5204	912.2449	3.1327	1.1061
75	2016	37	15.2571	84.9694	779.1010	2.2010	1.4163
40	2016	37	23.2143	77.5510	945.5020	5.9724	1.1673
11	2016	37	20.8857	78.7041	879.3939	3.1429	1.3408
35	2016	37	22.4286	72.4490	939.8490	4.6704	1.2592
78	2016	37	22.2857	77.3980	900.8908	3.3735	1.6102
28	2016	37	23.1286	71.7551	929.8439	4.7776	1.5265
39	2016	37	21.1286	78.2755	903.3163	2.6561	2.5867
24	2016	37	23.2857	75.2347	940.8745	3.4214	1.3908
63	2016	37	23.2143	77.5510	945.5020	5.9724	1.1673
62	2016	37	20.8571	82.8469	876.8663	2.6561	0.8888
1	2016	37	20.6571	76.0918	876.8449	2.9735	2.3531
31	2016	38	16.9429	86.0102	850.7449	2.1194	0.9653
79	2016	38	24.4286	68.5612	972.3980	5.7653	1.4031
51	2016	38	21.7571	72.3571	942.2918	5.4806	1.2214
14	2016	38	20.5143	74.9592	900.4020	3.9806	1.4051
67	2016	38	20.5571	77.1735	905.4133	3.8633	2.5439
42	2016	38	19.9286	73.9490	878.7745	3.6561	2.3878
50	2016	38	20.0571	75.0612	905.6684	3.7316	1.3286
43	2016	38	19.9286	73.9490	878.7745	3.6561	2.3878
85	2016	38	20.5143	76.9184	914.1143	3.8541	1.1510
25	2016	38	23.7000	78.6327	979.1367	6.2582	1.3092
69	2016	38	22.4714	72.9286	943.1194	4.3490	1.4480
57	2016	38	19.9857	83.7245	890.7602	3.0571	1.5694
9	2016	38	18.6429	78.8367	858.2510	2.3643	1.7908
72	2016	38	20.1857	77.2143	881.3704	3.2184	1.3520
26	2016	38	19.4143	83.3469	870.9745	2.7133	1.1480
7	2016	38	18.9143	82.2857	863.1316	1.9286	1.1837
83	2016	38	23.9143	74.6939	947.9520	4.0122	1.0724
76	2016	38	21.7000	73.5714	924.6490	5.2112	1.2939
36	2016	38	22.4000	71.3571	932.1612	5.8388	1.4347
81	2016	38	21.7571	72.3571	942.2918	5.4806	1.2214
15	2016	38	21.7286	74.9592	936.6510	4.4224	0.6653
32	2016	38	19.9286	73.9490	878.7745	3.6561	2.3878
73	2016	38	24.6143	68.6837	964.9102	5.0684	0.9918
71	2016	38	22.4000	71.3571	932.1612	5.8388	1.4347
41	2016	38	19.6000	80.2347	878.6745	2.4714	1.0908
10	2016	38	22.1857	73.2551	966.7541	5.6673	1.0449
23	2016	38	14.5000	85.9082	780.4469	2.3673	1.4224

27	2016	38	18.9143	82.2857	863.1316	1.9286	1.1837
60	2016	38	21.7571	72.3571	942.2918	5.4806	1.2214
53	2016	38	18.6429	78.8367	858.2510	2.3643	1.7908
66	2016	38	20.5143	74.9592	900.4020	3.9806	1.4051
59	2016	38	19.9857	83.7245	890.7602	3.0571	1.5694
61	2016	38	24.6143	68.6837	964.9102	5.0684	0.9918
84	2016	38	24.6143	68.6837	964.9102	5.0684	0.9918
38	2016	38	19.9857	83.7245	890.7602	3.0571	1.5694
87	2016	38	21.3286	74.6224	902.9337	4.7378	1.8000
34	2016	38	19.9857	83.7245	890.7602	3.0571	1.5694
29	2016	38	22.4714	72.9286	943.1194	4.3490	1.4480
5	2016	38	17.4571	85.3673	836.5122	2.5408	1.2561
8	2016	38	18.6429	78.8367	858.2510	2.3643	1.7908
12	2016	38	17.4571	85.3673	836.5122	2.5408	1.2561
13	2016	38	23.9143	74.6939	947.9520	4.0122	1.0724
18	2016	38	24.0429	70.1224	966.8929	5.3102	1.0571
33	2016	38	20.0571	75.0612	905.6684	3.7316	1.3286
56	2016	38	23.7000	78.6327	979.1367	6.2582	1.3092
77	2016	38	21.7286	74.9592	936.6510	4.4224	0.6653
54	2016	38	17.4571	85.3673	836.5122	2.5408	1.2561
21	2016	38	20.0571	75.0612	905.6684	3.7316	1.3286
68	2016	38	24.4286	68.5612	972.3980	5.7653	1.4031
74	2016	38	24.6143	68.6837	964.9102	5.0684	0.9918
88	2016	38	19.9286	73.9490	878.7745	3.6561	2.3878
16	2016	38	21.7000	73.5714	924.6490	5.2112	1.2939
30	2016	38	20.5143	74.9592	900.4020	3.9806	1.4051
6	2016	38	24.4286	68.5612	972.3980	5.7653	1.4031
49	2016	38	22.4714	72.9286	943.1194	4.3490	1.4480
22	2016	38	19.9286	73.9490	878.7745	3.6561	2.3878
45	2016	38	16.9429	83.9388	821.8398	1.6684	1.2061
58	2016	38	22.4714	72.9286	943.1194	4.3490	1.4480
37	2016	38	24.4286	68.5612	972.3980	5.7653	1.4031
17	2016	38	20.5571	77.1735	905.4133	3.8633	2.5439
55	2016	38	20.1857	77.2143	881.3704	3.2184	1.3520
46	2016	38	21.7000	73.5714	924.6490	5.2112	1.2939
86	2016	38	19.3000	80.0612	870.3112	2.3694	0.7622
2	2016	38	19.3000	80.0612	870.3112	2.3694	0.7622
4	2016	38	20.0571	75.0612	905.6684	3.7316	1.3286
47	2016	38	23.1429	77.0204	961.1908	4.7133	0.2663
82	2016	38	19.9286	73.9490	878.7745	3.6561	2.3878
19	2016	38	23.9143	73.3673	962.5551	6.3214	1.2245
20	2016	38	18.6429	78.8367	858.2510	2.3643	1.7908
80	2016	38	19.9286	73.9490	878.7745	3.6561	2.3878
3	2016	38	23.9143	74.6939	947.9520	4.0122	1.0724
52	2016	38	20.5571	77.1735	905.4133	3.8633	2.5439
70	2016	38	20.5143	76.9184	914.1143	3.8541	1.1510
64	2016	38	14.5000	85.9082	780.4469	2.3673	1.4224
48	2016	38	21.7286	74.9592	936.6510	4.4224	0.6653
65	2016	38	20.5571	77.1735	905.4133	3.8633	2.5439
44	2016	38	20.5143	76.9184	914.1143	3.8541	1.1510

75	2016	38	14.5000	85.9082	780.4469	2.3673	1.4224
40	2016	38	22.0429	76.3163	948.0051	6.6694	1.1857
11	2016	38	20.1857	77.2143	881.3704	3.2184	1.3520
35	2016	38	21.7571	72.3571	942.2918	5.4806	1.2214
78	2016	38	21.3286	74.6224	902.9337	4.7378	1.8000
28	2016	38	22.4000	71.3571	932.1612	5.8388	1.4347
39	2016	38	20.5571	77.1735	905.4133	3.8633	2.5439
24	2016	38	22.4714	72.9286	943.1194	4.3490	1.4480
63	2016	38	22.0429	76.3163	948.0051	6.6694	1.1857
62	2016	38	19.6000	80.2347	878.6745	2.4714	1.0908
1	2016	38	19.9286	73.9490	878.7745	3.6561	2.3878
31	2016	39	18.7286	86.5714	850.4990	1.7000	0.8816
79	2016	39	22.8143	66.4796	972.2949	5.1929	1.5929
51	2016	39	21.1571	71.3878	942.2449	5.5010	1.4214
14	2016	39	21.0429	73.2755	900.4939	4.5561	1.6776
67	2016	39	20.3143	76.1531	905.3908	4.1439	2.6990
42	2016	39	20.2571	72.6224	878.6765	3.7929	2.4357
50	2016	39	20.1857	77.6837	905.4306	2.2520	1.4592
43	2016	39	20.2571	72.6224	878.6765	3.7929	2.4357
85	2016	39	21.2571	77.7245	913.9347	2.8959	1.3500
25	2016	39	24.0714	76.9286	979.3765	6.0133	1.3327
69	2016	39	23.0286	70.6224	942.8929	4.2888	1.5663
57	2016	39	20.2429	81.2041	890.5490	2.5918	2.0908
9	2016	39	19.6429	76.5612	858.1929	3.0582	2.2306
72	2016	39	21.1571	74.8980	881.3643	3.7541	1.5051
26	2016	39	21.3429	81.8061	870.9827	3.4163	1.2888
7	2016	39	20.6429	79.6429	863.1112	2.7041	1.4071
83	2016	39	24.7429	71.3980	947.8418	5.7663	1.1224
76	2016	39	21.8429	72.3367	924.3347	3.8918	1.2857
36	2016	39	21.8143	70.1122	932.1214	5.8490	1.4908
81	2016	39	21.1571	71.3878	942.2449	5.5010	1.4214
15	2016	39	22.3286	74.8367	936.3153	2.7490	0.7704
32	2016	39	20.2571	72.6224	878.6765	3.7929	2.4357
73	2016	39	24.5714	65.1327	964.5061	4.5888	1.0837
71	2016	39	21.8143	70.1122	932.1214	5.8490	1.4908
41	2016	39	20.5286	78.1327	878.4296	2.4265	1.2204
10	2016	39	21.8286	72.4796	966.8398	5.4378	1.0347
23	2016	39	14.6143	87.3163	780.2459	1.7204	1.9163
27	2016	39	20.6429	79.6429	863.1112	2.7041	1.4071
60	2016	39	21.1571	71.3878	942.2449	5.5010	1.4214
53	2016	39	19.6429	76.5612	858.1929	3.0582	2.2306
66	2016	39	21.0429	73.2755	900.4939	4.5561	1.6776
59	2016	39	20.2429	81.2041	890.5490	2.5918	2.0908
61	2016	39	24.5714	65.1327	964.5061	4.5888	1.0837
84	2016	39	24.5714	65.1327	964.5061	4.5888	1.0837
38	2016	39	20.2429	81.2041	890.5490	2.5918	2.0908
87	2016	39	21.9714	71.6735	902.9092	5.5898	1.9347
34	2016	39	20.2429	81.2041	890.5490	2.5918	2.0908
29	2016	39	23.0286	70.6224	942.8929	4.2888	1.5663
5	2016	39	18.8429	83.1531	836.3867	3.1408	1.3337

8	2016	39	19.6429	76.5612	858.1929	3.0582	2.2306
12	2016	39	18.8429	83.1531	836.3867	3.1408	1.3337
13	2016	39	24.7429	71.3980	947.8418	5.7663	1.1224
18	2016	39	23.2571	67.2551	966.6276	4.6235	1.2327
33	2016	39	20.1857	77.6837	905.4306	2.2520	1.4592
56	2016	39	24.0714	76.9286	979.3765	6.0133	1.3327
77	2016	39	22.3286	74.8367	936.3153	2.7490	0.7704
54	2016	39	18.8429	83.1531	836.3867	3.1408	1.3337
21	2016	39	20.1857	77.6837	905.4306	2.2520	1.4592
68	2016	39	22.8143	66.4796	972.2949	5.1929	1.5929
74	2016	39	24.5714	65.1327	964.5061	4.5888	1.0837
88	2016	39	20.2571	72.6224	878.6765	3.7929	2.4357
16	2016	39	21.8429	72.3367	924.3347	3.8918	1.2857
30	2016	39	21.0429	73.2755	900.4939	4.5561	1.6776
6	2016	39	22.8143	66.4796	972.2949	5.1929	1.5929
49	2016	39	23.0286	70.6224	942.8929	4.2888	1.5663
22	2016	39	20.2571	72.6224	878.6765	3.7929	2.4357
45	2016	39	18.0286	82.6735	821.7143	2.2112	1.3214
58	2016	39	23.0286	70.6224	942.8929	4.2888	1.5663
37	2016	39	22.8143	66.4796	972.2949	5.1929	1.5929
17	2016	39	20.3143	76.1531	905.3908	4.1439	2.6990
55	2016	39	21.1571	74.8980	881.3643	3.7541	1.5051
46	2016	39	21.8429	72.3367	924.3347	3.8918	1.2857
86	2016	39	20.2857	77.2449	870.1092	2.3429	1.3439
2	2016	39	20.2857	77.2449	870.1092	2.3429	1.3439
4	2016	39	20.1857	77.6837	905.4306	2.2520	1.4592
47	2016	39	25.1286	74.3061	961.2969	5.3918	0.4633
82	2016	39	20.2571	72.6224	878.6765	3.7929	2.4357
19	2016	39	24.9429	70.9898	962.7673	6.7939	1.2143
20	2016	39	19.6429	76.5612	858.1929	3.0582	2.2306
80	2016	39	20.2571	72.6224	878.6765	3.7929	2.4357
3	2016	39	24.7429	71.3980	947.8418	5.7663	1.1224
52	2016	39	20.3143	76.1531	905.3908	4.1439	2.6990
70	2016	39	21.2571	77.7245	913.9347	2.8959	1.3500
64	2016	39	14.6143	87.3163	780.2459	1.7204	1.9163
48	2016	39	22.3286	74.8367	936.3153	2.7490	0.7704
65	2016	39	20.3143	76.1531	905.3908	4.1439	2.6990
44	2016	39	21.2571	77.7245	913.9347	2.8959	1.3500
75	2016	39	14.6143	87.3163	780.2459	1.7204	1.9163
40	2016	39	20.9286	74.8469	948.1827	6.4735	1.3102
11	2016	39	21.1571	74.8980	881.3643	3.7541	1.5051
35	2016	39	21.1571	71.3878	942.2449	5.5010	1.4214
78	2016	39	21.9714	71.6735	902.9092	5.5898	1.9347
28	2016	39	21.8143	70.1122	932.1214	5.8490	1.4908
39	2016	39	20.3143	76.1531	905.3908	4.1439	2.6990
24	2016	39	23.0286	70.6224	942.8929	4.2888	1.5663
63	2016	39	20.9286	74.8469	948.1827	6.4735	1.3102
62	2016	39	20.5286	78.1327	878.4296	2.4265	1.2204
1	2016	39	20.2571	72.6224	878.6765	3.7929	2.4357
31	2016	40	17.1857	81.6327	849.9765	3.7745	0.8653

79	2016	40	23.4286	68.4490	971.8663	4.9673	1.4102
51	2016	40	20.4286	72.3265	941.7765	5.2867	1.4133
14	2016	40	20.4429	72.8469	899.9306	4.7020	1.7224
67	2016	40	19.9571	76.7959	904.8531	4.0194	2.7571
42	2016	40	19.5000	71.1327	878.1337	4.7255	2.4531
50	2016	40	19.3143	76.9082	904.8102	3.3908	1.4449
43	2016	40	19.5000	71.1327	878.1337	4.7255	2.4531
85	2016	40	21.1857	73.1939	913.2092	4.4867	1.5204
25	2016	40	23.1143	76.0612	978.9459	4.9847	1.3684
69	2016	40	21.8857	68.6020	942.3449	4.7990	1.5316
57	2016	40	19.7000	76.4490	889.9643	3.7857	2.3092
9	2016	40	18.6571	73.2857	857.6765	4.9102	2.1561
72	2016	40	19.7143	74.3776	880.8224	5.1347	1.3786
26	2016	40	19.5143	78.3673	870.4735	4.9592	1.3888
7	2016	40	18.2857	76.8776	862.6316	4.2429	1.4235
83	2016	40	23.1714	71.9082	946.8316	6.1561	1.1459
76	2016	40	20.2143	72.7347	923.7306	4.4684	1.3214
36	2016	40	21.6714	70.5918	931.6051	5.5357	1.5031
81	2016	40	20.4286	72.3265	941.7765	5.2867	1.4133
15	2016	40	20.1286	75.8571	935.6816	3.2337	0.8031
32	2016	40	19.5000	71.1327	878.1337	4.7255	2.4531
73	2016	40	23.5714	65.3265	963.9276	4.8714	1.0908
71	2016	40	21.6714	70.5918	931.6051	5.5357	1.5031
41	2016	40	19.2714	73.6429	877.8480	4.3663	1.2265
10	2016	40	21.1714	73.1735	966.4612	5.4398	0.9337
23	2016	40	14.1857	82.8673	780.0122	3.9378	2.1857
27	2016	40	18.2857	76.8776	862.6316	4.2429	1.4235
60	2016	40	20.4286	72.3265	941.7765	5.2867	1.4133
53	2016	40	18.6571	73.2857	857.6765	4.9102	2.1561
66	2016	40	20.4429	72.8469	899.9306	4.7020	1.7224
59	2016	40	19.7000	76.4490	889.9643	3.7857	2.3092
61	2016	40	23.5714	65.3265	963.9276	4.8714	1.0908
84	2016	40	23.5714	65.3265	963.9276	4.8714	1.0908
38	2016	40	19.7000	76.4490	889.9643	3.7857	2.3092
87	2016	40	21.1286	70.9388	902.3469	5.9071	1.8194
34	2016	40	19.7000	76.4490	889.9643	3.7857	2.3092
29	2016	40	21.8857	68.6020	942.3449	4.7990	1.5316
5	2016	40	16.9000	78.8776	835.9684	4.9959	1.3306
8	2016	40	18.6571	73.2857	857.6765	4.9102	2.1561
12	2016	40	16.9000	78.8776	835.9684	4.9959	1.3306
13	2016	40	23.1714	71.9082	946.8316	6.1561	1.1459
18	2016	40	23.0714	69.1224	966.0959	4.8010	1.2582
33	2016	40	19.3143	76.9082	904.8102	3.3908	1.4449
56	2016	40	23.1143	76.0612	978.9459	4.9847	1.3684
77	2016	40	20.1286	75.8571	935.6816	3.2337	0.8031
54	2016	40	16.9000	78.8776	835.9684	4.9959	1.3306
21	2016	40	19.3143	76.9082	904.8102	3.3908	1.4449
68	2016	40	23.4286	68.4490	971.8663	4.9673	1.4102
74	2016	40	23.5714	65.3265	963.9276	4.8714	1.0908
88	2016	40	19.5000	71.1327	878.1337	4.7255	2.4531

16	2016	40	20.2143	72.7347	923.7306	4.4684	1.3214
30	2016	40	20.4429	72.8469	899.9306	4.7020	1.7224
6	2016	40	23.4286	68.4490	971.8663	4.9673	1.4102
49	2016	40	21.8857	68.6020	942.3449	4.7990	1.5316
22	2016	40	19.5000	71.1327	878.1337	4.7255	2.4531
45	2016	40	16.7000	79.6837	821.3418	4.3276	1.2847
58	2016	40	21.8857	68.6020	942.3449	4.7990	1.5316
37	2016	40	23.4286	68.4490	971.8663	4.9673	1.4102
17	2016	40	19.9571	76.7959	904.8531	4.0194	2.7571
55	2016	40	19.7143	74.3776	880.8224	5.1347	1.3786
46	2016	40	20.2143	72.7347	923.7306	4.4684	1.3214
86	2016	40	18.4143	72.7041	869.5908	4.1173	1.4245
2	2016	40	18.4143	72.7041	869.5908	4.1173	1.4245
4	2016	40	19.3143	76.9082	904.8102	3.3908	1.4449
47	2016	40	24.1000	72.9898	960.6796	5.2561	0.6408
82	2016	40	19.5000	71.1327	878.1337	4.7255	2.4531
19	2016	40	24.1000	69.4796	962.2367	5.7245	1.1653
20	2016	40	18.6571	73.2857	857.6765	4.9102	2.1561
80	2016	40	19.5000	71.1327	878.1337	4.7255	2.4531
3	2016	40	23.1714	71.9082	946.8316	6.1561	1.1459
52	2016	40	19.9571	76.7959	904.8531	4.0194	2.7571
70	2016	40	21.1857	73.1939	913.2092	4.4867	1.5204
64	2016	40	14.1857	82.8673	780.0122	3.9378	2.1857
48	2016	40	20.1286	75.8571	935.6816	3.2337	0.8031
65	2016	40	19.9571	76.7959	904.8531	4.0194	2.7571
44	2016	40	21.1857	73.1939	913.2092	4.4867	1.5204
75	2016	40	14.1857	82.8673	780.0122	3.9378	2.1857
40	2016	40	20.7000	75.7755	947.7837	5.5827	1.3296
11	2016	40	19.7143	74.3776	880.8224	5.1347	1.3786
35	2016	40	20.4286	72.3265	941.7765	5.2867	1.4133
78	2016	40	21.1286	70.9388	902.3469	5.9071	1.8194
28	2016	40	21.6714	70.5918	931.6051	5.5357	1.5031
39	2016	40	19.9571	76.7959	904.8531	4.0194	2.7571
24	2016	40	21.8857	68.6020	942.3449	4.7990	1.5316
63	2016	40	20.7000	75.7755	947.7837	5.5827	1.3296
62	2016	40	19.2714	73.6429	877.8480	4.3663	1.2265
1	2016	40	19.5000	71.1327	878.1337	4.7255	2.4531
31	2016	41	13.1571	85.1735	850.7429	2.9837	0.8031
79	2016	41	18.0286	64.3980	974.5143	3.9092	1.6990
51	2016	41	16.1857	69.3673	943.8816	3.8010	1.4633
14	2016	41	16.4714	71.8673	901.1000	3.8827	1.4398
67	2016	41	15.1000	77.0612	906.2949	3.0949	2.7204
42	2016	41	14.0000	75.9286	879.2163	3.6541	2.4347
50	2016	41	14.7143	77.8061	906.3347	3.1102	1.2347
43	2016	41	14.0000	75.9286	879.2163	3.6541	2.4347
85	2016	41	15.0143	75.1735	914.7163	3.5704	1.4653
25	2016	41	21.6143	70.0000	980.8602	4.5092	1.4857
69	2016	41	17.2714	66.9796	944.4582	3.7041	1.4857
57	2016	41	14.0714	80.4796	891.2918	2.9816	1.9582
9	2016	41	14.0286	78.2959	858.4551	3.4235	1.9010

72	2016	41	15.5857	78.2959	881.7439	3.3878	1.3000
26	2016	41	16.3429	84.6224	871.2082	3.1735	1.1235
7	2016	41	15.6571	82.9388	863.3673	2.7622	1.3051
83	2016	41	19.6571	77.2755	948.2602	3.5133	1.0929
76	2016	41	14.7143	75.7857	925.7571	3.5796	1.4204
36	2016	41	16.8143	67.2551	933.3888	4.3173	1.6857
81	2016	41	16.1857	69.3673	943.8816	3.8010	1.4633
15	2016	41	15.4286	78.8265	937.8541	2.8531	0.7255
32	2016	41	14.0000	75.9286	879.2163	3.6541	2.4347
73	2016	41	17.3714	66.4898	966.6051	3.7449	0.9969
71	2016	41	16.8143	67.2551	933.3888	4.3173	1.6857
41	2016	41	14.5429	79.1939	879.0112	3.2224	1.1959
10	2016	41	18.2143	69.9694	968.7429	4.1184	0.9490
23	2016	41	11.2000	85.5408	780.0776	3.2204	1.9643
27	2016	41	15.6571	82.9388	863.3673	2.7622	1.3051
60	2016	41	16.1857	69.3673	943.8816	3.8010	1.4633
53	2016	41	14.0286	78.2959	858.4551	3.4235	1.9010
66	2016	41	16.4714	71.8673	901.1000	3.8827	1.4398
59	2016	41	14.0714	80.4796	891.2918	2.9816	1.9582
61	2016	41	17.3714	66.4898	966.6051	3.7449	0.9969
84	2016	41	17.3714	66.4898	966.6051	3.7449	0.9969
38	2016	41	14.0714	80.4796	891.2918	2.9816	1.9582
87	2016	41	16.2429	74.0408	903.5816	3.8378	1.7622
34	2016	41	14.0714	80.4796	891.2918	2.9816	1.9582
29	2016	41	17.2714	66.9796	944.4582	3.7041	1.4857
5	2016	41	14.1429	85.4388	836.5235	3.4071	1.3082
8	2016	41	14.0286	78.2959	858.4551	3.4235	1.9010
12	2016	41	14.1429	85.4388	836.5235	3.4071	1.3082
13	2016	41	19.6571	77.2755	948.2602	3.5133	1.0929
18	2016	41	17.1143	67.0612	968.8388	3.3724	1.2990
33	2016	41	14.7143	77.8061	906.3347	3.1102	1.2347
56	2016	41	21.6143	70.0000	980.8602	4.5092	1.4857
77	2016	41	15.4286	78.8265	937.8541	2.8531	0.7255
54	2016	41	14.1429	85.4388	836.5235	3.4071	1.3082
21	2016	41	14.7143	77.8061	906.3347	3.1102	1.2347
68	2016	41	18.0286	64.3980	974.5143	3.9092	1.6990
74	2016	41	17.3714	66.4898	966.6051	3.7449	0.9969
88	2016	41	14.0000	75.9286	879.2163	3.6541	2.4347
16	2016	41	14.7143	75.7857	925.7571	3.5796	1.4204
30	2016	41	16.4714	71.8673	901.1000	3.8827	1.4398
6	2016	41	18.0286	64.3980	974.5143	3.9092	1.6990
49	2016	41	17.2714	66.9796	944.4582	3.7041	1.4857
22	2016	41	14.0000	75.9286	879.2163	3.6541	2.4347
45	2016	41	14.6571	84.8980	821.7286	3.2745	1.2429
58	2016	41	17.2714	66.9796	944.4582	3.7041	1.4857
37	2016	41	18.0286	64.3980	974.5143	3.9092	1.6990
17	2016	41	15.1000	77.0612	906.2949	3.0949	2.7204
55	2016	41	15.5857	78.2959	881.7439	3.3878	1.3000
46	2016	41	14.7143	75.7857	925.7571	3.5796	1.4204
86	2016	41	14.6857	78.7449	870.5898	3.1041	0.9755

2	2016	41	14.6857	78.7449	870.5898	3.1041	0.9755
4	2016	41	14.7143	77.8061	906.3347	3.1102	1.2347
47	2016	41	21.0571	71.1939	962.2347	3.6898	0.6306
82	2016	41	14.0000	75.9286	879.2163	3.6541	2.4347
19	2016	41	21.4857	64.5000	963.8194	5.0745	1.3694
20	2016	41	14.0286	78.2959	858.4551	3.4235	1.9010
80	2016	41	14.0000	75.9286	879.2163	3.6541	2.4347
3	2016	41	19.6571	77.2755	948.2602	3.5133	1.0929
52	2016	41	15.1000	77.0612	906.2949	3.0949	2.7204
70	2016	41	15.0143	75.1735	914.7163	3.5704	1.4653
64	2016	41	11.2000	85.5408	780.0776	3.2204	1.9643
48	2016	41	15.4286	78.8265	937.8541	2.8531	0.7255
65	2016	41	15.1000	77.0612	906.2949	3.0949	2.7204
44	2016	41	15.0143	75.1735	914.7163	3.5704	1.4653
75	2016	41	11.2000	85.5408	780.0776	3.2204	1.9643
40	2016	41	17.7857	73.3878	949.6837	4.6092	1.4429
11	2016	41	15.5857	78.2959	881.7439	3.3878	1.3000
35	2016	41	16.1857	69.3673	943.8816	3.8010	1.4633
78	2016	41	16.2429	74.0408	903.5816	3.8378	1.7622
28	2016	41	16.8143	67.2551	933.3888	4.3173	1.6857
39	2016	41	15.1000	77.0612	906.2949	3.0949	2.7204
24	2016	41	17.2714	66.9796	944.4582	3.7041	1.4857
63	2016	41	17.7857	73.3878	949.6837	4.6092	1.4429
62	2016	41	14.5429	79.1939	879.0112	3.2224	1.1959
1	2016	41	14.0000	75.9286	879.2163	3.6541	2.4347
31	2016	42	16.7429	92.2245	851.1551	0.8663	0.7245
79	2016	42	20.2286	68.7143	975.9286	1.4286	1.6908
51	2016	42	18.9143	74.3265	944.9806	1.1745	1.4327
14	2016	42	18.3429	78.2449	901.7429	1.4153	1.5878
67	2016	42	18.0429	85.0204	907.0449	0.8847	2.8735
42	2016	42	17.1000	86.7143	879.8296	1.0918	2.3255
50	2016	42	18.1857	85.2245	907.1816	0.7571	1.1194
43	2016	42	17.1000	86.7143	879.8296	1.0918	2.3255
85	2016	42	18.7286	87.5918	915.7694	0.7908	1.1459
25	2016	42	21.9000	71.3571	981.7214	2.2898	1.2071
69	2016	42	19.6286	76.2245	945.6214	1.1490	1.3969
57	2016	42	17.2714	93.0816	892.0622	0.7449	1.8327
9	2016	42	16.2857	87.2041	858.8857	0.9541	2.0378
72	2016	42	17.7143	84.2143	882.2939	1.1173	1.6327
26	2016	42	18.7571	89.6633	871.6031	1.3847	1.1122
7	2016	42	17.5286	88.0918	863.7194	1.3612	1.5010
83	2016	42	21.5143	82.1633	949.1837	1.3633	0.9888
76	2016	42	18.5429	84.1224	926.8296	1.0878	1.3429
36	2016	42	19.2571	74.4694	934.3592	1.7684	1.7510
81	2016	42	18.9143	74.3265	944.9806	1.1745	1.4327
15	2016	42	19.5286	85.8673	939.0694	0.6112	0.5837
32	2016	42	17.1000	86.7143	879.8296	1.0918	2.3255
73	2016	42	20.6714	76.1939	968.1316	1.3388	0.8735
71	2016	42	19.2571	74.4694	934.3592	1.7684	1.7510
41	2016	42	17.2571	90.5918	879.6429	0.7816	1.0357

10	2016	42	20.0429	74.0102	969.8551	1.1786	0.8551
23	2016	42	14.2000	89.5000	779.8908	2.2969	1.9898
27	2016	42	17.5286	88.0918	863.7194	1.3612	1.5010
60	2016	42	18.9143	74.3265	944.9806	1.1745	1.4327
53	2016	42	16.2857	87.2041	858.8857	0.9541	2.0378
66	2016	42	18.3429	78.2449	901.7429	1.4153	1.5878
59	2016	42	17.2714	93.0816	892.0622	0.7449	1.8327
61	2016	42	20.6714	76.1939	968.1316	1.3388	0.8735
84	2016	42	20.6714	76.1939	968.1316	1.3388	0.8735
38	2016	42	17.2714	93.0816	892.0622	0.7449	1.8327
87	2016	42	18.6571	83.0918	904.3367	1.0765	1.7694
34	2016	42	17.2714	93.0816	892.0622	0.7449	1.8327
29	2016	42	19.6286	76.2245	945.6214	1.1490	1.3969
5	2016	42	16.0714	90.7449	836.7122	1.8684	1.4469
8	2016	42	16.2857	87.2041	858.8857	0.9541	2.0378
12	2016	42	16.0714	90.7449	836.7122	1.8684	1.4469
13	2016	42	21.5143	82.1633	949.1837	1.3633	0.9888
18	2016	42	20.1429	72.0510	970.3367	1.0990	1.1571
33	2016	42	18.1857	85.2245	907.1816	0.7571	1.1194
56	2016	42	21.9000	71.3571	981.7214	2.2898	1.2071
77	2016	42	19.5286	85.8673	939.0694	0.6112	0.5837
54	2016	42	16.0714	90.7449	836.7122	1.8684	1.4469
21	2016	42	18.1857	85.2245	907.1816	0.7571	1.1194
68	2016	42	20.2286	68.7143	975.9286	1.4286	1.6908
74	2016	42	20.6714	76.1939	968.1316	1.3388	0.8735
88	2016	42	17.1000	86.7143	879.8296	1.0918	2.3255
16	2016	42	18.5429	84.1224	926.8296	1.0878	1.3429
30	2016	42	18.3429	78.2449	901.7429	1.4153	1.5878
6	2016	42	20.2286	68.7143	975.9286	1.4286	1.6908
49	2016	42	19.6286	76.2245	945.6214	1.1490	1.3969
22	2016	42	17.1000	86.7143	879.8296	1.0918	2.3255
45	2016	42	16.3714	87.6837	821.7602	1.9888	1.3980
58	2016	42	19.6286	76.2245	945.6214	1.1490	1.3969
37	2016	42	20.2286	68.7143	975.9286	1.4286	1.6908
17	2016	42	18.0429	85.0204	907.0449	0.8847	2.8735
55	2016	42	17.7143	84.2143	882.2939	1.1173	1.6327
46	2016	42	18.5429	84.1224	926.8296	1.0878	1.3429
86	2016	42	16.8286	87.2551	871.0908	0.8735	0.9571
2	2016	42	16.8286	87.2551	871.0908	0.8735	0.9571
4	2016	42	18.1857	85.2245	907.1816	0.7571	1.1194
47	2016	42	22.0143	75.0000	963.2776	1.3531	0.5286
82	2016	42	17.1000	86.7143	879.8296	1.0918	2.3255
19	2016	42	22.2714	67.8878	964.6908	2.4265	1.3857
20	2016	42	16.2857	87.2041	858.8857	0.9541	2.0378
80	2016	42	17.1000	86.7143	879.8296	1.0918	2.3255
3	2016	42	21.5143	82.1633	949.1837	1.3633	0.9888
52	2016	42	18.0429	85.0204	907.0449	0.8847	2.8735
70	2016	42	18.7286	87.5918	915.7694	0.7908	1.1459
64	2016	42	14.2000	89.5000	779.8908	2.2969	1.9898
48	2016	42	19.5286	85.8673	939.0694	0.6112	0.5837

65	2016	42	18.0429	85.0204	907.0449	0.8847	2.8735
44	2016	42	18.7286	87.5918	915.7694	0.7908	1.1459
75	2016	42	14.2000	89.5000	779.8908	2.2969	1.9898
40	2016	42	19.1000	77.3163	950.5929	2.0520	1.6582
11	2016	42	17.7143	84.2143	882.2939	1.1173	1.6327
35	2016	42	18.9143	74.3265	944.9806	1.1745	1.4327
78	2016	42	18.6571	83.0918	904.3367	1.0765	1.7694
28	2016	42	19.2571	74.4694	934.3592	1.7684	1.7510
39	2016	42	18.0429	85.0204	907.0449	0.8847	2.8735
24	2016	42	19.6286	76.2245	945.6214	1.1490	1.3969
63	2016	42	19.1000	77.3163	950.5929	2.0520	1.6582
62	2016	42	17.2571	90.5918	879.6429	0.7816	1.0357
1	2016	42	17.1000	86.7143	879.8296	1.0918	2.3255
31	2016	43	16.0286	85.5612	849.2245	3.2337	0.9755
79	2016	43	13.3714	82.1429	974.3786	0.5684	1.6633
51	2016	43	13.5714	86.0102	943.1561	0.8276	1.4602
14	2016	43	18.3857	83.4388	900.4102	1.6796	2.3459
67	2016	43	18.3857	87.6735	905.3296	0.8602	3.3133
42	2016	43	17.7857	85.8980	878.1837	2.5571	2.6010
50	2016	43	17.1286	84.4694	904.8092	1.6276	1.6071
43	2016	43	17.7857	85.8980	878.1837	2.5571	2.6010
85	2016	43	17.1000	85.9082	913.3449	2.4061	1.4735
25	2016	43	21.7286	79.7755	979.8653	1.5714	0.8551
69	2016	43	19.3429	83.0816	943.3755	1.4337	1.5133
57	2016	43	18.1857	90.6633	889.9388	1.5592	2.7694
9	2016	43	16.7857	85.8469	857.4612	2.5051	2.5990
72	2016	43	18.4857	83.0204	880.9469	2.8847	2.0878
26	2016	43	18.8286	84.7653	870.4173	3.2510	1.5622
7	2016	43	18.6857	83.3061	862.4490	3.8337	1.9459
83	2016	43	22.2571	80.2755	947.6500	3.0051	0.9990
76	2016	43	17.1714	86.7551	924.4673	2.1276	1.3786
36	2016	43	19.7143	81.4694	932.3786	2.4388	1.7867
81	2016	43	13.5714	86.0102	943.1561	0.8276	1.4602
15	2016	43	17.5000	85.8878	936.4786	0.4398	0.7571
32	2016	43	17.7857	85.8980	878.1837	2.5571	2.6010
73	2016	43	17.7000	82.0612	965.8653	1.5673	0.8204
71	2016	43	19.7143	81.4694	932.3786	2.4388	1.7867
41	2016	43	18.4429	85.9286	877.6082	2.9194	1.4939
10	2016	43	15.5286	84.3571	968.0878	0.4408	0.9694
23	2016	43	12.9000	82.2449	778.7827	5.2459	2.2531
27	2016	43	18.6857	83.3061	862.4490	3.8337	1.9459
60	2016	43	13.5714	86.0102	943.1561	0.8276	1.4602
53	2016	43	16.7857	85.8469	857.4612	2.5051	2.5990
66	2016	43	18.3857	83.4388	900.4102	1.6796	2.3459
59	2016	43	18.1857	90.6633	889.9388	1.5592	2.7694
61	2016	43	17.7000	82.0612	965.8653	1.5673	0.8204
84	2016	43	17.7000	82.0612	965.8653	1.5673	0.8204
38	2016	43	18.1857	90.6633	889.9388	1.5592	2.7694
87	2016	43	19.6000	83.5918	902.7092	2.3265	1.8490
34	2016	43	18.1857	90.6633	889.9388	1.5592	2.7694

29	2016	43	19.3429	83.0816	943.3755	1.4337	1.5133
5	2016	43	17.1000	83.9082	835.4735	4.2112	1.8878
8	2016	43	16.7857	85.8469	857.4612	2.5051	2.5990
12	2016	43	17.1000	83.9082	835.4735	4.2112	1.8878
13	2016	43	22.2571	80.2755	947.6500	3.0051	0.9990
18	2016	43	13.5429	84.7857	968.6898	0.5929	0.9133
33	2016	43	17.1286	84.4694	904.8092	1.6276	1.6071
56	2016	43	21.7286	79.7755	979.8653	1.5714	0.8551
77	2016	43	17.5000	85.8878	936.4786	0.4398	0.7571
54	2016	43	17.1000	83.9082	835.4735	4.2112	1.8878
21	2016	43	17.1286	84.4694	904.8092	1.6276	1.6071
68	2016	43	13.3714	82.1429	974.3786	0.5684	1.6633
74	2016	43	17.7000	82.0612	965.8653	1.5673	0.8204
88	2016	43	17.7857	85.8980	878.1837	2.5571	2.6010
16	2016	43	17.1714	86.7551	924.4673	2.1276	1.3786
30	2016	43	18.3857	83.4388	900.4102	1.6796	2.3459
6	2016	43	13.3714	82.1429	974.3786	0.5684	1.6633
49	2016	43	19.3429	83.0816	943.3755	1.4337	1.5133
22	2016	43	17.7857	85.8980	878.1837	2.5571	2.6010
45	2016	43	16.8429	80.5204	820.6867	4.7582	1.8031
58	2016	43	19.3429	83.0816	943.3755	1.4337	1.5133
37	2016	43	13.3714	82.1429	974.3786	0.5684	1.6633
17	2016	43	18.3857	87.6735	905.3296	0.8602	3.3133
55	2016	43	18.4857	83.0204	880.9469	2.8847	2.0878
46	2016	43	17.1714	86.7551	924.4673	2.1276	1.3786
86	2016	43	18.0286	83.4796	869.2520	2.4531	1.6510
2	2016	43	18.0286	83.4796	869.2520	2.4531	1.6510
4	2016	43	17.1286	84.4694	904.8092	1.6276	1.6071
47	2016	43	22.1571	80.9796	961.7000	2.0582	0.5071
82	2016	43	17.7857	85.8980	878.1837	2.5571	2.6010
19	2016	43	22.2286	76.4388	963.2276	1.6418	1.1520
20	2016	43	16.7857	85.8469	857.4612	2.5051	2.5990
80	2016	43	17.7857	85.8980	878.1837	2.5571	2.6010
3	2016	43	22.2571	80.2755	947.6500	3.0051	0.9990
52	2016	43	18.3857	87.6735	905.3296	0.8602	3.3133
70	2016	43	17.1000	85.9082	913.3449	2.4061	1.4735
64	2016	43	12.9000	82.2449	778.7827	5.2459	2.2531
48	2016	43	17.5000	85.8878	936.4786	0.4398	0.7571
65	2016	43	18.3857	87.6735	905.3296	0.8602	3.3133
44	2016	43	17.1000	85.9082	913.3449	2.4061	1.4735
75	2016	43	12.9000	82.2449	778.7827	5.2459	2.2531
40	2016	43	18.3143	85.2551	948.8561	1.5816	1.6561
11	2016	43	18.4857	83.0204	880.9469	2.8847	2.0878
35	2016	43	13.5714	86.0102	943.1561	0.8276	1.4602
78	2016	43	19.6000	83.5918	902.7092	2.3265	1.8490
28	2016	43	19.7143	81.4694	932.3786	2.4388	1.7867
39	2016	43	18.3857	87.6735	905.3296	0.8602	3.3133
24	2016	43	19.3429	83.0816	943.3755	1.4337	1.5133
63	2016	43	18.3143	85.2551	948.8561	1.5816	1.6561
62	2016	43	18.4429	85.9286	877.6082	2.9194	1.4939

1	2016	43	17.7857	85.8980	878.1837	2.5571	2.6010
31	2016	44	11.5714	83.2347	851.1194	3.3969	1.1888
79	2016	44	15.6571	85.8776	977.9929	0.5357	1.8541
51	2016	44	14.6429	89.0612	946.0929	1.3224	1.5673
14	2016	44	13.4143	84.0408	903.1143	1.9010	2.5796
67	2016	44	13.4714	86.7755	907.9857	0.9469	3.5449
42	2016	44	12.3429	84.7755	880.4633	2.7102	2.8408
50	2016	44	13.5143	83.0816	907.2847	1.9184	1.9194
43	2016	44	12.3429	84.7755	880.4633	2.7102	2.8408
85	2016	44	13.7571	83.2041	915.9592	2.6327	1.7327
25	2016	44	17.2000	80.1735	983.6051	1.8245	0.9184
69	2016	44	15.8714	80.3469	946.2908	1.8510	1.7847
57	2016	44	12.9714	88.5408	892.2827	1.5898	3.0786
9	2016	44	12.1571	85.3163	859.5408	2.5388	2.9245
72	2016	44	13.6286	82.0918	883.3204	2.9163	2.2980
26	2016	44	14.5429	86.4694	872.6163	3.5010	1.6918
7	2016	44	13.9286	83.2347	864.5510	4.0020	2.0500
83	2016	44	17.6143	80.3163	950.7163	3.3153	1.0378
76	2016	44	14.0286	85.6020	927.1214	2.3806	1.5449
36	2016	44	15.2571	79.7551	935.2827	2.7429	1.9847
81	2016	44	14.6429	89.0612	946.0929	1.3224	1.5673
15	2016	44	14.7714	83.6122	939.1837	0.6133	0.8347
32	2016	44	12.3429	84.7755	880.4633	2.7102	2.8408
73	2016	44	16.2000	81.5306	969.0490	1.8480	0.8459
71	2016	44	15.2571	79.7551	935.2827	2.7429	1.9847
41	2016	44	13.1143	82.9388	879.7622	3.2551	1.9745
10	2016	44	15.1857	87.4898	971.5969	1.0214	1.0408
23	2016	44	10.1143	82.9184	780.1102	4.5878	2.4020
27	2016	44	13.9286	83.2347	864.5510	4.0020	2.0500
60	2016	44	14.6429	89.0612	946.0929	1.3224	1.5673
53	2016	44	12.1571	85.3163	859.5408	2.5388	2.9245
66	2016	44	13.4143	84.0408	903.1143	1.9010	2.5796
59	2016	44	12.9714	88.5408	892.2827	1.5898	3.0786
61	2016	44	16.2000	81.5306	969.0490	1.8480	0.8459
84	2016	44	16.2000	81.5306	969.0490	1.8480	0.8459
38	2016	44	12.9714	88.5408	892.2827	1.5898	3.0786
87	2016	44	14.3571	81.0306	905.2796	2.6357	2.2337
34	2016	44	12.9714	88.5408	892.2827	1.5898	3.0786
29	2016	44	15.8714	80.3469	946.2908	1.8510	1.7847
5	2016	44	12.1429	84.5714	837.2837	4.1520	2.1092
8	2016	44	12.1571	85.3163	859.5408	2.5388	2.9245
12	2016	44	12.1429	84.5714	837.2837	4.1520	2.1092
13	2016	44	17.6143	80.3163	950.7163	3.3153	1.0378
18	2016	44	15.5143	88.7347	972.1224	0.6888	0.8561
33	2016	44	13.5143	83.0816	907.2847	1.9184	1.9194
56	2016	44	17.2000	80.1735	983.6051	1.8245	0.9184
77	2016	44	14.7714	83.6122	939.1837	0.6133	0.8347
54	2016	44	12.1429	84.5714	837.2837	4.1520	2.1092
21	2016	44	13.5143	83.0816	907.2847	1.9184	1.9194
68	2016	44	15.6571	85.8776	977.9929	0.5357	1.8541

74	2016	44	16.2000	81.5306	969.0490	1.8480	0.8459
88	2016	44	12.3429	84.7755	880.4633	2.7102	2.8408
16	2016	44	14.0286	85.6020	927.1214	2.3806	1.5449
30	2016	44	13.4143	84.0408	903.1143	1.9010	2.5796
6	2016	44	15.6571	85.8776	977.9929	0.5357	1.8541
49	2016	44	15.8714	80.3469	946.2908	1.8510	1.7847
22	2016	44	12.3429	84.7755	880.4633	2.7102	2.8408
45	2016	44	13.2000	80.7245	822.3592	4.9265	1.8663
58	2016	44	15.8714	80.3469	946.2908	1.8510	1.7847
37	2016	44	15.6571	85.8776	977.9929	0.5357	1.8541
17	2016	44	13.4714	86.7755	907.9857	0.9469	3.5449
55	2016	44	13.6286	82.0918	883.3204	2.9163	2.2980
46	2016	44	14.0286	85.6020	927.1214	2.3806	1.5449
86	2016	44	13.0429	81.5102	871.3500	2.5653	2.0520
2	2016	44	13.0429	81.5102	871.3500	2.5653	2.0520
4	2016	44	13.5143	83.0816	907.2847	1.9184	1.9194
47	2016	44	17.7143	81.4694	965.0755	2.2153	0.5276
82	2016	44	12.3429	84.7755	880.4633	2.7102	2.8408
19	2016	44	17.2286	77.6224	966.7949	1.7224	1.1776
20	2016	44	12.1571	85.3163	859.5408	2.5388	2.9245
80	2016	44	12.3429	84.7755	880.4633	2.7102	2.8408
3	2016	44	17.6143	80.3163	950.7163	3.3153	1.0378
52	2016	44	13.4714	86.7755	907.9857	0.9469	3.5449
70	2016	44	13.7571	83.2041	915.9592	2.6327	1.7327
64	2016	44	10.1143	82.9184	780.1102	4.5878	2.4020
48	2016	44	14.7714	83.6122	939.1837	0.6133	0.8347
65	2016	44	13.4714	86.7755	907.9857	0.9469	3.5449
44	2016	44	13.7571	83.2041	915.9592	2.6327	1.7327
75	2016	44	10.1143	82.9184	780.1102	4.5878	2.4020
40	2016	44	14.4714	87.5408	952.1480	1.9255	1.8367
11	2016	44	13.6286	82.0918	883.3204	2.9163	2.2980
35	2016	44	14.6429	89.0612	946.0929	1.3224	1.5673
78	2016	44	14.3571	81.0306	905.2796	2.6357	2.2337
28	2016	44	15.2571	79.7551	935.2827	2.7429	1.9847
39	2016	44	13.4714	86.7755	907.9857	0.9469	3.5449
24	2016	44	15.8714	80.3469	946.2908	1.8510	1.7847
63	2016	44	14.4714	87.5408	952.1480	1.9255	1.8367
62	2016	44	13.1143	82.9388	879.7622	3.2551	1.9745
1	2016	44	12.3429	84.7755	880.4633	2.7102	2.8408
31	2016	45	8.0143	88.8163	853.8878	1.3929	1.1551
79	2016	45	12.3571	83.0816	981.1439	1.4082	1.6286
51	2016	45	11.3429	85.5000	949.0541	1.4898	1.4551
14	2016	45	11.1000	85.8265	905.6776	1.3704	2.2541
67	2016	45	10.5429	90.1122	910.8235	0.5847	2.9245
42	2016	45	10.0429	88.6939	883.0602	0.9816	2.3765
50	2016	45	10.2857	86.1633	910.7551	1.0827	1.6296
43	2016	45	10.0429	88.6939	883.0602	0.9816	2.3765
85	2016	45	10.3000	87.3980	919.7184	0.9959	1.3204
25	2016	45	14.6857	80.4490	987.4908	1.6031	0.9133
69	2016	45	12.7000	81.5612	949.8735	1.4541	1.5071

57	2016	45	9.6714	93.0612	895.4194	0.4969	2.1949
9	2016	45	9.8571	88.2857	861.7633	0.8980	2.4388
72	2016	45	11.3000	85.4898	885.7276	1.0602	2.0010
26	2016	45	12.1000	91.4796	874.7337	2.4449	1.4765
7	2016	45	12.0000	87.4184	866.6194	1.9622	1.8327
83	2016	45	15.9286	81.4592	953.9643	2.0796	0.9816
76	2016	45	11.1429	87.2347	930.5622	1.0010	1.4235
36	2016	45	11.6000	80.2551	938.6020	1.5214	1.7765
81	2016	45	11.3429	85.5000	949.0541	1.4898	1.4551
15	2016	45	11.9143	84.8571	942.9173	0.7122	0.5347
32	2016	45	10.0429	88.6939	883.0602	0.9816	2.3765
73	2016	45	13.6286	82.7653	972.7143	1.4051	0.7673
71	2016	45	11.6000	80.2551	938.6020	1.5214	1.7765
41	2016	45	10.0286	88.4898	882.7204	1.1133	1.4939
10	2016	45	12.4143	85.4898	974.9653	1.7245	0.8571
23	2016	45	7.5143	87.6020	781.5092	3.0082	2.2276
27	2016	45	12.0000	87.4184	866.6194	1.9622	1.8327
60	2016	45	11.3429	85.5000	949.0541	1.4898	1.4551
53	2016	45	9.8571	88.2857	861.7633	0.8980	2.4388
66	2016	45	11.1000	85.8265	905.6776	1.3704	2.2541
59	2016	45	9.6714	93.0612	895.4194	0.4969	2.1949
61	2016	45	13.6286	82.7653	972.7143	1.4051	0.7673
84	2016	45	13.6286	82.7653	972.7143	1.4051	0.7673
38	2016	45	9.6714	93.0612	895.4194	0.4969	2.1949
87	2016	45	12.0571	84.5510	908.1265	1.1378	2.1480
34	2016	45	9.6714	93.0612	895.4194	0.4969	2.1949
29	2016	45	12.7000	81.5612	949.8735	1.4541	1.5071
5	2016	45	10.0714	91.7755	839.0929	2.4337	1.7694
8	2016	45	9.8571	88.2857	861.7633	0.8980	2.4388
12	2016	45	10.0714	91.7755	839.0929	2.4337	1.7694
13	2016	45	15.9286	81.4592	953.9643	2.0796	0.9816
18	2016	45	12.4429	85.8367	975.1929	1.5898	0.8643
33	2016	45	10.2857	86.1633	910.7551	1.0827	1.6296
56	2016	45	14.6857	80.4490	987.4908	1.6031	0.9133
77	2016	45	11.9143	84.8571	942.9173	0.7122	0.5347
54	2016	45	10.0714	91.7755	839.0929	2.4337	1.7694
21	2016	45	10.2857	86.1633	910.7551	1.0827	1.6296
68	2016	45	12.3571	83.0816	981.1439	1.4082	1.6286
74	2016	45	13.6286	82.7653	972.7143	1.4051	0.7673
88	2016	45	10.0429	88.6939	883.0602	0.9816	2.3765
16	2016	45	11.1429	87.2347	930.5622	1.0010	1.4235
30	2016	45	11.1000	85.8265	905.6776	1.3704	2.2541
6	2016	45	12.3571	83.0816	981.1439	1.4082	1.6286
49	2016	45	12.7000	81.5612	949.8735	1.4541	1.5071
22	2016	45	10.0429	88.6939	883.0602	0.9816	2.3765
45	2016	45	11.7857	84.6837	823.8653	3.8306	1.5459
58	2016	45	12.7000	81.5612	949.8735	1.4541	1.5071
37	2016	45	12.3571	83.0816	981.1439	1.4082	1.6286
17	2016	45	10.5429	90.1122	910.8235	0.5847	2.9245
55	2016	45	11.3000	85.4898	885.7276	1.0602	2.0010

46	2016	45	11.1429	87.2347	930.5622	1.0010	1.4235
86	2016	45	9.8714	85.1122	874.0643	0.7949	1.4837
2	2016	45	9.8714	85.1122	874.0643	0.7949	1.4837
4	2016	45	10.2857	86.1633	910.7551	1.0827	1.6296
47	2016	45	15.3857	81.9694	968.6704	1.2714	0.5582
82	2016	45	10.0429	88.6939	883.0602	0.9816	2.3765
19	2016	45	14.7000	78.0408	970.3459	1.5255	1.1724
20	2016	45	9.8571	88.2857	861.7633	0.8980	2.4388
80	2016	45	10.0429	88.6939	883.0602	0.9816	2.3765
3	2016	45	15.9286	81.4592	953.9643	2.0796	0.9816
52	2016	45	10.5429	90.1122	910.8235	0.5847	2.9245
70	2016	45	10.3000	87.3980	919.7184	0.9959	1.3204
64	2016	45	7.5143	87.6020	781.5092	3.0082	2.2276
48	2016	45	11.9143	84.8571	942.9173	0.7122	0.5347
65	2016	45	10.5429	90.1122	910.8235	0.5847	2.9245
44	2016	45	10.3000	87.3980	919.7184	0.9959	1.3204
75	2016	45	7.5143	87.6020	781.5092	3.0082	2.2276
40	2016	45	12.0000	85.7449	955.3755	2.0561	1.8694
11	2016	45	11.3000	85.4898	885.7276	1.0602	2.0010
35	2016	45	11.3429	85.5000	949.0541	1.4898	1.4551
78	2016	45	12.0571	84.5510	908.1265	1.1378	2.1480
28	2016	45	11.6000	80.2551	938.6020	1.5214	1.7765
39	2016	45	10.5429	90.1122	910.8235	0.5847	2.9245
24	2016	45	12.7000	81.5612	949.8735	1.4541	1.5071
63	2016	45	12.0000	85.7449	955.3755	2.0561	1.8694
62	2016	45	10.0286	88.4898	882.7204	1.1133	1.4939
1	2016	45	10.0429	88.6939	883.0602	0.9816	2.3765
31	2016	46	13.5714	87.7959	851.8959	2.1806	0.9755
79	2016	46	16.3000	85.0000	978.3684	1.3184	1.2827
51	2016	46	15.5286	87.1735	946.5265	1.2235	1.4071
14	2016	46	18.1857	87.2551	903.5408	1.4694	2.2776
67	2016	46	18.2571	90.2347	908.5082	1.2163	2.5898
42	2016	46	16.6714	88.2449	880.8510	1.8602	2.1857
50	2016	46	14.9143	86.5204	908.5969	1.1388	1.5235
43	2016	46	16.6714	88.2449	880.8510	1.8602	2.1857
85	2016	46	15.9000	86.5612	917.4878	1.6469	1.1367
25	2016	46	21.2000	83.6122	984.8969	2.0969	0.6837
69	2016	46	17.9714	84.6837	947.4265	1.3857	1.1878
57	2016	46	14.9143	94.5714	893.2388	0.6459	1.8663
9	2016	46	16.2286	87.5204	859.6541	2.2776	2.2786
72	2016	46	17.8286	85.0714	883.5541	2.3255	1.7939
26	2016	46	17.6286	90.9082	872.6847	3.8796	1.5092
7	2016	46	17.5571	85.2143	864.5143	3.7684	1.8531
83	2016	46	21.5286	79.3673	951.5510	3.6439	0.9245
76	2016	46	15.8429	88.3776	928.2847	0.8949	1.2449
36	2016	46	18.6143	82.9286	936.1878	1.6663	1.3602
81	2016	46	15.5286	87.1735	946.5265	1.2235	1.4071
15	2016	46	15.9143	86.6633	940.7020	0.8592	0.3816
32	2016	46	16.6714	88.2449	880.8510	1.8602	2.1857
73	2016	46	17.5286	83.6020	970.2204	1.2204	0.6867

71	2016	46	18.6143	82.9286	936.1878	1.6663	1.3602
41	2016	46	15.2714	88.8571	880.6194	1.9020	0.9786
10	2016	46	17.1857	87.1020	972.3408	1.7847	0.7878
23	2016	46	11.7571	80.7551	779.7041	5.1245	1.7469
27	2016	46	17.5571	85.2143	864.5143	3.7684	1.8531
60	2016	46	15.5286	87.1735	946.5265	1.2235	1.4071
53	2016	46	16.2286	87.5204	859.6541	2.2776	2.2786
66	2016	46	18.1857	87.2551	903.5408	1.4694	2.2776
59	2016	46	14.9143	94.5714	893.2388	0.6459	1.8663
61	2016	46	17.5286	83.6020	970.2204	1.2204	0.6867
84	2016	46	17.5286	83.6020	970.2204	1.2204	0.6867
38	2016	46	14.9143	94.5714	893.2388	0.6459	1.8663
87	2016	46	18.7143	84.6837	905.8561	2.1276	1.8173
34	2016	46	14.9143	94.5714	893.2388	0.6459	1.8663
29	2016	46	17.9714	84.6837	947.4265	1.3857	1.1878
5	2016	46	15.3143	88.5816	837.0786	4.2684	1.5541
8	2016	46	16.2286	87.5204	859.6541	2.2776	2.2786
12	2016	46	15.3143	88.5816	837.0786	4.2684	1.5541
13	2016	46	21.5286	79.3673	951.5510	3.6439	0.9245
18	2016	46	15.9857	86.6224	972.4673	1.3888	0.7612
33	2016	46	14.9143	86.5204	908.5969	1.1388	1.5235
56	2016	46	21.2000	83.6122	984.8969	2.0969	0.6837
77	2016	46	15.9143	86.6633	940.7020	0.8592	0.3816
54	2016	46	15.3143	88.5816	837.0786	4.2684	1.5541
21	2016	46	14.9143	86.5204	908.5969	1.1388	1.5235
68	2016	46	16.3000	85.0000	978.3684	1.3184	1.2827
74	2016	46	17.5286	83.6020	970.2204	1.2204	0.6867
88	2016	46	16.6714	88.2449	880.8510	1.8602	2.1857
16	2016	46	15.8429	88.3776	928.2847	0.8949	1.2449
30	2016	46	18.1857	87.2551	903.5408	1.4694	2.2776
6	2016	46	16.3000	85.0000	978.3684	1.3184	1.2827
49	2016	46	17.9714	84.6837	947.4265	1.3857	1.1878
22	2016	46	16.6714	88.2449	880.8510	1.8602	2.1857
45	2016	46	15.0143	77.5102	821.9418	6.1378	1.7724
58	2016	46	17.9714	84.6837	947.4265	1.3857	1.1878
37	2016	46	16.3000	85.0000	978.3684	1.3184	1.2827
17	2016	46	18.2571	90.2347	908.5082	1.2163	2.5898
55	2016	46	17.8286	85.0714	883.5541	2.3255	1.7939
46	2016	46	15.8429	88.3776	928.2847	0.8949	1.2449
86	2016	46	16.0571	85.6122	871.9459	1.2306	1.3112
2	2016	46	16.0571	85.6122	871.9459	1.2306	1.3112
4	2016	46	14.9143	86.5204	908.5969	1.1388	1.5235
47	2016	46	21.5429	82.8367	966.1898	2.3082	0.5816
82	2016	46	16.6714	88.2449	880.8510	1.8602	2.1857
19	2016	46	21.7143	80.6122	968.0245	1.8816	0.9939
20	2016	46	16.2286	87.5204	859.6541	2.2776	2.2786
80	2016	46	16.6714	88.2449	880.8510	1.8602	2.1857
3	2016	46	21.5286	79.3673	951.5510	3.6439	0.9245
52	2016	46	18.2571	90.2347	908.5082	1.2163	2.5898
70	2016	46	15.9000	86.5612	917.4878	1.6469	1.1367

64	2016	46	11.7571	80.7551	779.7041	5.1245	1.7469
48	2016	46	15.9143	86.6633	940.7020	0.8592	0.3816
65	2016	46	18.2571	90.2347	908.5082	1.2163	2.5898
44	2016	46	15.9000	86.5612	917.4878	1.6469	1.1367
75	2016	46	11.7571	80.7551	779.7041	5.1245	1.7469
40	2016	46	18.1000	87.1633	952.9367	2.1633	1.5276
11	2016	46	17.8286	85.0714	883.5541	2.3255	1.7939
35	2016	46	15.5286	87.1735	946.5265	1.2235	1.4071
78	2016	46	18.7143	84.6837	905.8561	2.1276	1.8173
28	2016	46	18.6143	82.9286	936.1878	1.6663	1.3602
39	2016	46	18.2571	90.2347	908.5082	1.2163	2.5898
24	2016	46	17.9714	84.6837	947.4265	1.3857	1.1878
63	2016	46	18.1000	87.1633	952.9367	2.1633	1.5276
62	2016	46	15.2714	88.8571	880.6194	1.9020	0.9786
1	2016	46	16.6714	88.2449	880.8510	1.8602	2.1857
31	2016	47	7.2571	82.9898	850.2969	3.1878	0.8184
79	2016	47	7.3000	85.0816	977.8000	0.5133	1.5000
51	2016	47	6.1857	90.0816	945.6224	1.0316	1.5959
14	2016	47	8.8714	84.1224	902.1786	2.3173	2.4122
67	2016	47	7.4000	86.4796	907.1163	2.5847	2.9980
42	2016	47	7.7571	82.3163	879.3000	3.8816	2.4663
50	2016	47	8.8000	84.2041	907.0388	1.3143	1.5602
43	2016	47	7.7571	82.3163	879.3000	3.8816	2.4663
85	2016	47	9.3000	84.8469	915.7143	2.3612	1.1959
25	2016	47	11.3286	81.3673	983.5367	2.9969	0.8908
69	2016	47	8.8857	82.6633	946.2102	1.4837	1.3602
57	2016	47	7.4286	93.0102	891.6531	1.6551	1.8949
9	2016	47	8.7143	80.4286	858.1755	4.9204	2.4857
72	2016	47	9.9571	78.8980	882.0296	4.7724	1.8020
26	2016	47	11.7286	84.7755	871.2561	6.2235	1.7051
7	2016	47	10.4714	79.1429	863.1020	6.6653	1.9276
83	2016	47	14.2857	75.8265	949.7500	5.4102	1.1122
76	2016	47	7.7571	87.3469	927.0418	1.2847	1.4612
36	2016	47	7.7857	81.9592	934.8786	2.9378	1.6214
81	2016	47	6.1857	90.0816	945.6224	1.0316	1.5959
15	2016	47	9.3857	84.9796	939.3663	0.6837	0.6276
32	2016	47	7.7571	82.3163	879.3000	3.8816	2.4663
73	2016	47	9.9714	80.8163	969.2367	1.3633	0.8327
71	2016	47	7.7857	81.9592	934.8786	2.9378	1.6214
41	2016	47	8.1571	83.9082	878.9602	3.1510	1.0714
10	2016	47	7.4571	87.9388	971.5204	1.2765	0.9714
23	2016	47	5.7571	71.9082	778.4092	6.9786	1.6020
27	2016	47	10.4714	79.1429	863.1020	6.6653	1.9276
60	2016	47	6.1857	90.0816	945.6224	1.0316	1.5959
53	2016	47	8.7143	80.4286	858.1755	4.9204	2.4857
66	2016	47	8.8714	84.1224	902.1786	2.3173	2.4122
59	2016	47	7.4286	93.0102	891.6531	1.6551	1.8949
61	2016	47	9.9714	80.8163	969.2367	1.3633	0.8327
84	2016	47	9.9714	80.8163	969.2367	1.3633	0.8327
38	2016	47	7.4286	93.0102	891.6531	1.6551	1.8949

87	2016	47	10.2143	78.1224	904.2194	4.5214	1.9388
34	2016	47	7.4286	93.0102	891.6531	1.6551	1.8949
29	2016	47	8.8857	82.6633	946.2102	1.4837	1.3602
5	2016	47	8.9143	79.7449	835.7235	6.7378	1.5316
8	2016	47	8.7143	80.4286	858.1755	4.9204	2.4857
12	2016	47	8.9143	79.7449	835.7235	6.7378	1.5316
13	2016	47	14.2857	75.8265	949.7500	5.4102	1.1122
18	2016	47	7.4571	87.1327	971.9133	0.5857	0.8724
33	2016	47	8.8000	84.2041	907.0388	1.3143	1.5602
56	2016	47	11.3286	81.3673	983.5367	2.9969	0.8908
77	2016	47	9.3857	84.9796	939.3663	0.6837	0.6276
54	2016	47	8.9143	79.7449	835.7235	6.7378	1.5316
21	2016	47	8.8000	84.2041	907.0388	1.3143	1.5602
68	2016	47	7.3000	85.0816	977.8000	0.5133	1.5000
74	2016	47	9.9714	80.8163	969.2367	1.3633	0.8327
88	2016	47	7.7571	82.3163	879.3000	3.8816	2.4663
16	2016	47	7.7571	87.3469	927.0418	1.2847	1.4612
30	2016	47	8.8714	84.1224	902.1786	2.3173	2.4122
6	2016	47	7.3000	85.0816	977.8000	0.5133	1.5000
49	2016	47	8.8857	82.6633	946.2102	1.4837	1.3602
22	2016	47	7.7571	82.3163	879.3000	3.8816	2.4663
45	2016	47	9.7429	68.3265	820.7245	8.2133	1.8969
58	2016	47	8.8857	82.6633	946.2102	1.4837	1.3602
37	2016	47	7.3000	85.0816	977.8000	0.5133	1.5000
17	2016	47	7.4000	86.4796	907.1163	2.5847	2.9980
55	2016	47	9.9571	78.8980	882.0296	4.7724	1.8020
46	2016	47	7.7571	87.3469	927.0418	1.2847	1.4612
86	2016	47	8.3286	81.9796	870.3235	2.4408	1.3959
2	2016	47	8.3286	81.9796	870.3235	2.4408	1.3959
4	2016	47	8.8000	84.2041	907.0388	1.3143	1.5602
47	2016	47	13.9857	79.2755	964.3602	3.7367	0.6327
82	2016	47	7.7571	82.3163	879.3000	3.8816	2.4663
19	2016	47	12.6143	78.7449	966.4276	2.6847	1.1337
20	2016	47	8.7143	80.4286	858.1755	4.9204	2.4857
80	2016	47	7.7571	82.3163	879.3000	3.8816	2.4663
3	2016	47	14.2857	75.8265	949.7500	5.4102	1.1122
52	2016	47	7.4000	86.4796	907.1163	2.5847	2.9980
70	2016	47	9.3000	84.8469	915.7143	2.3612	1.1959
64	2016	47	5.7571	71.9082	778.4092	6.9786	1.6020
48	2016	47	9.3857	84.9796	939.3663	0.6837	0.6276
65	2016	47	7.4000	86.4796	907.1163	2.5847	2.9980
44	2016	47	9.3000	84.8469	915.7143	2.3612	1.1959
75	2016	47	5.7571	71.9082	778.4092	6.9786	1.6020
40	2016	47	6.8286	89.1429	951.9643	2.1500	1.5694
11	2016	47	9.9571	78.8980	882.0296	4.7724	1.8020
35	2016	47	6.1857	90.0816	945.6224	1.0316	1.5959
78	2016	47	10.2143	78.1224	904.2194	4.5214	1.9388
28	2016	47	7.7857	81.9592	934.8786	2.9378	1.6214
39	2016	47	7.4000	86.4796	907.1163	2.5847	2.9980
24	2016	47	8.8857	82.6633	946.2102	1.4837	1.3602

63	2016	47	6.8286	89.1429	951.9643	2.1500	1.5694
62	2016	47	8.1571	83.9082	878.9602	3.1510	1.0714
1	2016	47	7.7571	82.3163	879.3000	3.8816	2.4663
31	2016	48	5.6857	80.6837	852.4490	2.6031	0.8939
79	2016	48	9.0143	81.4796	982.1602	1.0480	1.5796
51	2016	48	7.3143	86.6531	949.4357	1.7837	1.5745
14	2016	48	8.2714	79.1837	904.8255	2.9949	2.2643
67	2016	48	7.4571	83.2959	910.1622	2.7551	3.1214
42	2016	48	6.9571	78.6939	881.8551	3.5939	2.6673
50	2016	48	7.4143	79.1429	910.0837	1.4888	1.6551
43	2016	48	6.9571	78.6939	881.8551	3.5939	2.6673
85	2016	48	7.7857	83.8163	918.8694	2.0786	1.2755
25	2016	48	11.2571	79.0714	987.8878	2.9010	1.1286
69	2016	48	8.8857	79.3878	950.1184	1.6786	1.4265
57	2016	48	6.2571	90.4592	894.4500	2.0622	2.0061
9	2016	48	6.6714	77.3571	860.3694	4.3337	2.3439
72	2016	48	8.5000	75.1020	884.5010	4.0408	1.8776
26	2016	48	9.2714	82.1633	873.4306	5.0765	1.5520
7	2016	48	8.3857	78.9592	865.3051	5.1786	1.9429
83	2016	48	13.2571	73.4694	953.1204	3.8520	1.1551
76	2016	48	7.4571	84.2857	930.5184	1.6745	1.6276
36	2016	48	8.0143	79.1939	938.4918	2.7755	1.8480
81	2016	48	7.3143	86.6531	949.4357	1.7837	1.5745
15	2016	48	8.0714	80.8878	942.9796	0.8949	0.9000
32	2016	48	6.9571	78.6939	881.8551	3.5939	2.6673
73	2016	48	9.2429	78.1633	973.4592	1.3980	0.9439
71	2016	48	8.0143	79.1939	938.4918	2.7755	1.8480
41	2016	48	6.7429	79.3673	881.5327	2.9010	1.3776
10	2016	48	8.5286	84.0918	975.7541	2.0153	1.0520
23	2016	48	3.9000	77.7143	779.4735	5.4765	2.0439
27	2016	48	8.3857	78.9592	865.3051	5.1786	1.9429
60	2016	48	7.3143	86.6531	949.4357	1.7837	1.5745
53	2016	48	6.6714	77.3571	860.3694	4.3337	2.3439
66	2016	48	8.2714	79.1837	904.8255	2.9949	2.2643
59	2016	48	6.2571	90.4592	894.4500	2.0622	2.0061
61	2016	48	9.2429	78.1633	973.4592	1.3980	0.9439
84	2016	48	9.2429	78.1633	973.4592	1.3980	0.9439
38	2016	48	6.2571	90.4592	894.4500	2.0622	2.0061
87	2016	48	9.3571	73.9388	907.1102	4.0694	2.1847
34	2016	48	6.2571	90.4592	894.4500	2.0622	2.0061
29	2016	48	8.8857	79.3878	950.1184	1.6786	1.4265
5	2016	48	6.7000	80.6633	837.5561	5.2337	1.6296
8	2016	48	6.6714	77.3571	860.3694	4.3337	2.3439
12	2016	48	6.7000	80.6633	837.5561	5.2337	1.6296
13	2016	48	13.2571	73.4694	953.1204	3.8520	1.1551
18	2016	48	8.4143	84.8265	976.1898	1.1010	0.9867
33	2016	48	7.4143	79.1429	910.0837	1.4888	1.6551
56	2016	48	11.2571	79.0714	987.8878	2.9010	1.1286
77	2016	48	8.0714	80.8878	942.9796	0.8949	0.9000
54	2016	48	6.7000	80.6633	837.5561	5.2337	1.6296

21	2016	48	7.4143	79.1429	910.0837	1.4888	1.6551
68	2016	48	9.0143	81.4796	982.1602	1.0480	1.5796
74	2016	48	9.2429	78.1633	973.4592	1.3980	0.9439
88	2016	48	6.9571	78.6939	881.8551	3.5939	2.6673
16	2016	48	7.4571	84.2857	930.5184	1.6745	1.6276
30	2016	48	8.2714	79.1837	904.8255	2.9949	2.2643
6	2016	48	9.0143	81.4796	982.1602	1.0480	1.5796
49	2016	48	8.8857	79.3878	950.1184	1.6786	1.4265
22	2016	48	6.9571	78.6939	881.8551	3.5939	2.6673
45	2016	48	7.3000	74.3265	822.2898	5.9949	1.6582
58	2016	48	8.8857	79.3878	950.1184	1.6786	1.4265
37	2016	48	9.0143	81.4796	982.1602	1.0480	1.5796
17	2016	48	7.4571	83.2959	910.1622	2.7551	3.1214
55	2016	48	8.5000	75.1020	884.5010	4.0408	1.8776
46	2016	48	7.4571	84.2857	930.5184	1.6745	1.6276
86	2016	48	7.3000	79.6327	872.7959	2.2776	1.2449
2	2016	48	7.3000	79.6327	872.7959	2.2776	1.2449
4	2016	48	7.4143	79.1429	910.0837	1.4888	1.6551
47	2016	48	13.2571	74.3571	968.0663	3.0031	0.6969
82	2016	48	6.9571	78.6939	881.8551	3.5939	2.6673
19	2016	48	12.5429	73.1224	969.9959	3.1633	1.3316
20	2016	48	6.6714	77.3571	860.3694	4.3337	2.3439
80	2016	48	6.9571	78.6939	881.8551	3.5939	2.6673
3	2016	48	13.2571	73.4694	953.1204	3.8520	1.1551
52	2016	48	7.4571	83.2959	910.1622	2.7551	3.1214
70	2016	48	7.7857	83.8163	918.8694	2.0786	1.2755
64	2016	48	3.9000	77.7143	779.4735	5.4765	2.0439
48	2016	48	8.0714	80.8878	942.9796	0.8949	0.9000
65	2016	48	7.4571	83.2959	910.1622	2.7551	3.1214
44	2016	48	7.7857	83.8163	918.8694	2.0786	1.2755
75	2016	48	3.9000	77.7143	779.4735	5.4765	2.0439
40	2016	48	7.6714	87.1735	955.7224	2.5796	1.6500
11	2016	48	8.5000	75.1020	884.5010	4.0408	1.8776
35	2016	48	7.3143	86.6531	949.4357	1.7837	1.5745
78	2016	48	9.3571	73.9388	907.1102	4.0694	2.1847
28	2016	48	8.0143	79.1939	938.4918	2.7755	1.8480
39	2016	48	7.4571	83.2959	910.1622	2.7551	3.1214
24	2016	48	8.8857	79.3878	950.1184	1.6786	1.4265
63	2016	48	7.6714	87.1735	955.7224	2.5796	1.6500
62	2016	48	6.7429	79.3673	881.5327	2.9010	1.3776
1	2016	48	6.9571	78.6939	881.8551	3.5939	2.6673
31	2016	49	9.1857	81.8878	855.0020	2.7235	0.9255
79	2016	49	11.0857	78.7653	984.3959	2.9745	0.9714
51	2016	49	9.5286	82.3265	951.8990	3.5694	1.0194
14	2016	49	11.6143	76.9694	907.4551	3.5949	1.7224
67	2016	49	11.1143	81.0918	912.8867	3.2694	2.6918
42	2016	49	10.8143	79.6327	884.5776	3.0898	2.3184
50	2016	49	9.6143	77.4388	913.0786	2.3765	1.5724
43	2016	49	10.8143	79.6327	884.5776	3.0898	2.3184
85	2016	49	9.9429	84.2143	922.0633	1.7847	1.0714

25	2016	49	12.3000	80.2041	991.0684	3.2510	0.9061
69	2016	49	11.0714	79.7347	953.0571	2.6582	1.0143
57	2016	49	10.0571	90.1224	897.3378	2.0337	2.0031
9	2016	49	10.2143	81.4286	862.9622	3.5531	1.8694
72	2016	49	10.9857	77.9592	887.2724	3.2714	1.6367
26	2016	49	12.1571	85.2857	876.1500	2.7765	1.1776
7	2016	49	11.0714	83.3980	867.9378	3.2388	1.5663
83	2016	49	14.1000	74.9082	956.5816	3.1224	0.9224
76	2016	49	9.8857	81.6531	933.3612	2.7214	1.3500
36	2016	49	10.9857	77.8265	941.3255	3.3173	1.5061
81	2016	49	9.5286	82.3265	951.8990	3.5694	1.0194
15	2016	49	8.7143	82.8469	946.1306	1.5000	0.7143
32	2016	49	10.8143	79.6327	884.5776	3.0898	2.3184
73	2016	49	10.7714	81.1122	976.5020	1.9745	0.7429
71	2016	49	10.9857	77.8265	941.3255	3.3173	1.5061
41	2016	49	10.2429	79.0612	884.3020	2.9327	1.2531
10	2016	49	10.1714	81.4286	978.2735	3.8959	0.6949
23	2016	49	7.7143	83.4898	781.4929	4.5031	2.0122
27	2016	49	11.0714	83.3980	867.9378	3.2388	1.5663
60	2016	49	9.5286	82.3265	951.8990	3.5694	1.0194
53	2016	49	10.2143	81.4286	862.9622	3.5531	1.8694
66	2016	49	11.6143	76.9694	907.4551	3.5949	1.7224
59	2016	49	10.0571	90.1224	897.3378	2.0337	2.0031
61	2016	49	10.7714	81.1122	976.5020	1.9745	0.7429
84	2016	49	10.7714	81.1122	976.5020	1.9745	0.7429
38	2016	49	10.0571	90.1224	897.3378	2.0337	2.0031
87	2016	49	11.5714	76.1531	910.1133	3.4786	1.9908
34	2016	49	10.0571	90.1224	897.3378	2.0337	2.0031
29	2016	49	11.0714	79.7347	953.0571	2.6582	1.0143
5	2016	49	10.4429	85.7041	839.9959	3.4173	1.4888
8	2016	49	10.2143	81.4286	862.9622	3.5531	1.8694
12	2016	49	10.4429	85.7041	839.9959	3.4173	1.4888
13	2016	49	14.1000	74.9082	956.5816	3.1224	0.9224
18	2016	49	10.4000	81.8367	978.4510	3.0704	0.7551
33	2016	49	9.6143	77.4388	913.0786	2.3765	1.5724
56	2016	49	12.3000	80.2041	991.0684	3.2510	0.9061
77	2016	49	8.7143	82.8469	946.1306	1.5000	0.7143
54	2016	49	10.4429	85.7041	839.9959	3.4173	1.4888
21	2016	49	9.6143	77.4388	913.0786	2.3765	1.5724
68	2016	49	11.0857	78.7653	984.3959	2.9745	0.9714
74	2016	49	10.7714	81.1122	976.5020	1.9745	0.7429
88	2016	49	10.8143	79.6327	884.5776	3.0898	2.3184
16	2016	49	9.8857	81.6531	933.3612	2.7214	1.3500
30	2016	49	11.6143	76.9694	907.4551	3.5949	1.7224
6	2016	49	11.0857	78.7653	984.3959	2.9745	0.9714
49	2016	49	11.0714	79.7347	953.0571	2.6582	1.0143
22	2016	49	10.8143	79.6327	884.5776	3.0898	2.3184
45	2016	49	10.5571	82.6735	824.5673	3.6204	1.3724
58	2016	49	11.0714	79.7347	953.0571	2.6582	1.0143
37	2016	49	11.0857	78.7653	984.3959	2.9745	0.9714

17	2016	49	11.1143	81.0918	912.8867	3.2694	2.6918
55	2016	49	10.9857	77.9592	887.2724	3.2714	1.6367
46	2016	49	9.8857	81.6531	933.3612	2.7214	1.3500
86	2016	49	10.6000	81.7755	875.5643	1.9837	1.0082
2	2016	49	10.6000	81.7755	875.5643	1.9837	1.0082
4	2016	49	9.6143	77.4388	913.0786	2.3765	1.5724
47	2016	49	13.9000	74.8878	971.5857	2.6327	0.6051
82	2016	49	10.8143	79.6327	884.5776	3.0898	2.3184
19	2016	49	13.4857	70.9286	973.1367	4.3337	1.0612
20	2016	49	10.2143	81.4286	862.9622	3.5531	1.8694
80	2016	49	10.8143	79.6327	884.5776	3.0898	2.3184
3	2016	49	14.1000	74.9082	956.5816	3.1224	0.9224
52	2016	49	11.1143	81.0918	912.8867	3.2694	2.6918
70	2016	49	9.9429	84.2143	922.0633	1.7847	1.0714
64	2016	49	7.7143	83.4898	781.4929	4.5031	2.0122
48	2016	49	8.7143	82.8469	946.1306	1.5000	0.7143
65	2016	49	11.1143	81.0918	912.8867	3.2694	2.6918
44	2016	49	9.9429	84.2143	922.0633	1.7847	1.0714
75	2016	49	7.7143	83.4898	781.4929	4.5031	2.0122
40	2016	49	9.7143	83.9286	958.2857	4.0265	1.1888
11	2016	49	10.9857	77.9592	887.2724	3.2714	1.6367
35	2016	49	9.5286	82.3265	951.8990	3.5694	1.0194
78	2016	49	11.5714	76.1531	910.1133	3.4786	1.9908
28	2016	49	10.9857	77.8265	941.3255	3.3173	1.5061
39	2016	49	11.1143	81.0918	912.8867	3.2694	2.6918
24	2016	49	11.0714	79.7347	953.0571	2.6582	1.0143
63	2016	49	9.7143	83.9286	958.2857	4.0265	1.1888
62	2016	49	10.2429	79.0612	884.3020	2.9327	1.2531
1	2016	49	10.8143	79.6327	884.5776	3.0898	2.3184
31	2016	50	6.9286	80.8367	852.8378	3.3541	0.9765
79	2016	50	8.1714	75.4286	981.9102	2.8908	1.2173
51	2016	50	6.6286	80.9388	949.5582	3.1969	1.0765
14	2016	50	8.7143	74.4286	905.4510	3.6857	1.7571
67	2016	50	7.8000	79.3469	910.7143	3.5694	2.5245
42	2016	50	7.8857	78.4082	882.5061	3.1500	2.2082
50	2016	50	7.8000	78.5306	910.6806	2.4245	1.5429
43	2016	50	7.8857	78.4082	882.5061	3.1500	2.2082
85	2016	50	8.7000	82.9796	919.4837	1.5949	1.1469
25	2016	50	10.8429	80.1531	988.5092	3.2378	0.7663
69	2016	50	8.6000	77.2347	950.5857	2.5153	1.0357
57	2016	50	7.1143	87.6837	895.0633	2.0378	2.0061
9	2016	50	7.8714	81.7041	861.0643	3.2041	2.0367
72	2016	50	9.7000	78.6735	885.2827	3.0153	1.4214
26	2016	50	10.7429	83.6429	874.2918	3.0173	1.3224
7	2016	50	9.7571	82.4796	866.0786	3.4786	1.4714
83	2016	50	13.9143	75.5204	954.2163	4.1837	0.8786
76	2016	50	7.1000	80.2143	930.9755	2.4531	1.4245
36	2016	50	8.0857	75.4898	938.9184	3.8806	1.5286
81	2016	50	6.6286	80.9388	949.5582	3.1969	1.0765
15	2016	50	8.0571	84.4286	943.6888	1.1837	0.6163

32	2016	50	7.8857	78.4082	882.5061	3.1500	2.2082
73	2016	50	9.2143	78.9082	973.9745	2.0490	0.6071
71	2016	50	8.0857	75.4898	938.9184	3.8806	1.5286
41	2016	50	8.1571	78.3878	882.0133	2.6551	1.2367
10	2016	50	7.9143	79.8673	975.7857	3.4745	0.6235
23	2016	50	5.1000	79.3673	780.1663	5.0327	2.0694
27	2016	50	9.7571	82.4796	866.0786	3.4786	1.4714
60	2016	50	6.6286	80.9388	949.5582	3.1969	1.0765
53	2016	50	7.8714	81.7041	861.0643	3.2041	2.0367
66	2016	50	8.7143	74.4286	905.4510	3.6857	1.7571
59	2016	50	7.1143	87.6837	895.0633	2.0378	2.0061
61	2016	50	9.2143	78.9082	973.9745	2.0490	0.6071
84	2016	50	9.2143	78.9082	973.9745	2.0490	0.6071
38	2016	50	7.1143	87.6837	895.0633	2.0378	2.0061
87	2016	50	10.3429	75.7041	907.9194	3.5949	1.7918
34	2016	50	7.1143	87.6837	895.0633	2.0378	2.0061
29	2016	50	8.6000	77.2347	950.5857	2.5153	1.0357
5	2016	50	7.2143	84.8571	838.2949	3.4071	1.4459
8	2016	50	7.8714	81.7041	861.0643	3.2041	2.0367
12	2016	50	7.2143	84.8571	838.2949	3.4071	1.4459
13	2016	50	13.9143	75.5204	954.2163	4.1837	0.8786
18	2016	50	7.5714	78.8673	976.0051	3.2388	0.8031
33	2016	50	7.8000	78.5306	910.6806	2.4245	1.5429
56	2016	50	10.8429	80.1531	988.5092	3.2378	0.7663
77	2016	50	8.0571	84.4286	943.6888	1.1837	0.6163
54	2016	50	7.2143	84.8571	838.2949	3.4071	1.4459
21	2016	50	7.8000	78.5306	910.6806	2.4245	1.5429
68	2016	50	8.1714	75.4286	981.9102	2.8908	1.2173
74	2016	50	9.2143	78.9082	973.9745	2.0490	0.6071
88	2016	50	7.8857	78.4082	882.5061	3.1500	2.2082
16	2016	50	7.1000	80.2143	930.9755	2.4531	1.4245
30	2016	50	8.7143	74.4286	905.4510	3.6857	1.7571
6	2016	50	8.1714	75.4286	981.9102	2.8908	1.2173
49	2016	50	8.6000	77.2347	950.5857	2.5153	1.0357
22	2016	50	7.8857	78.4082	882.5061	3.1500	2.2082
45	2016	50	8.3143	79.3980	823.0153	4.0061	1.2918
58	2016	50	8.6000	77.2347	950.5857	2.5153	1.0357
37	2016	50	8.1714	75.4286	981.9102	2.8908	1.2173
17	2016	50	7.8000	79.3469	910.7143	3.5694	2.5245
55	2016	50	9.7000	78.6735	885.2827	3.0153	1.4214
46	2016	50	7.1000	80.2143	930.9755	2.4531	1.4245
86	2016	50	8.6000	81.7857	873.4265	1.6612	1.1602
2	2016	50	8.6000	81.7857	873.4265	1.6612	1.1602
4	2016	50	7.8000	78.5306	910.6806	2.4245	1.5429
47	2016	50	13.6571	76.3265	969.0551	2.5714	0.5429
82	2016	50	7.8857	78.4082	882.5061	3.1500	2.2082
19	2016	50	12.2714	71.4184	970.7500	4.5327	0.9673
20	2016	50	7.8714	81.7041	861.0643	3.2041	2.0367
80	2016	50	7.8857	78.4082	882.5061	3.1500	2.2082
3	2016	50	13.9143	75.5204	954.2163	4.1837	0.8786

52	2016	50	7.8000	79.3469	910.7143	3.5694	2.5245
70	2016	50	8.7000	82.9796	919.4837	1.5949	1.1469
64	2016	50	5.1000	79.3673	780.1663	5.0327	2.0694
48	2016	50	8.0571	84.4286	943.6888	1.1837	0.6163
65	2016	50	7.8000	79.3469	910.7143	3.5694	2.5245
44	2016	50	8.7000	82.9796	919.4837	1.5949	1.1469
75	2016	50	5.1000	79.3673	780.1663	5.0327	2.0694
40	2016	50	7.3571	81.6735	955.9704	3.9418	1.0714
11	2016	50	9.7000	78.6735	885.2827	3.0153	1.4214
35	2016	50	6.6286	80.9388	949.5582	3.1969	1.0765
78	2016	50	10.3429	75.7041	907.9194	3.5949	1.7918
28	2016	50	8.0857	75.4898	938.9184	3.8806	1.5286
39	2016	50	7.8000	79.3469	910.7143	3.5694	2.5245
24	2016	50	8.6000	77.2347	950.5857	2.5153	1.0357
63	2016	50	7.3571	81.6735	955.9704	3.9418	1.0714
62	2016	50	8.1571	78.3878	882.0133	2.6551	1.2367
1	2016	50	7.8857	78.4082	882.5061	3.1500	2.2082
31	2016	51	6.4286	83.0918	851.2786	1.5643	0.9612
79	2016	51	8.8429	77.5306	981.3469	1.2439	1.3020
51	2016	51	8.0286	81.6939	948.7490	1.0724	1.2878
14	2016	51	10.2143	76.5306	904.2418	2.0122	2.0459
67	2016	51	8.2857	81.6224	909.5480	1.8439	2.5663
42	2016	51	7.9000	79.1939	881.1214	1.6439	2.4439
50	2016	51	8.2286	77.2245	909.3327	1.2112	1.4704
43	2016	51	7.9000	79.1939	881.1214	1.6439	2.4439
85	2016	51	8.9714	79.8878	918.0786	1.0388	1.2653
25	2016	51	12.2714	81.6837	987.5806	1.7755	0.6592
69	2016	51	9.3429	77.2959	949.5857	0.8704	1.1071
57	2016	51	7.0429	85.0816	893.6878	1.1327	1.9071
9	2016	51	8.8000	81.9694	859.5571	1.0418	2.4214
72	2016	51	11.5143	77.4082	883.7592	1.0122	1.5194
26	2016	51	13.1286	81.7755	872.6163	2.7367	1.5663
7	2016	51	12.6286	81.2551	864.4531	2.1694	1.8133
83	2016	51	16.8000	73.0714	952.5102	3.2898	0.8857
76	2016	51	7.9143	81.1837	929.9194	0.8408	1.5092
36	2016	51	8.7857	77.1429	937.8847	1.8316	1.5378
81	2016	51	8.0286	81.6939	948.7490	1.0724	1.2878
15	2016	51	8.8143	82.5918	942.4592	0.7102	0.5724
32	2016	51	7.9000	79.1939	881.1214	1.6439	2.4439
73	2016	51	10.2143	76.6939	973.0265	0.9020	0.5296
71	2016	51	8.7857	77.1429	937.8847	1.8316	1.5378
41	2016	51	7.5429	79.3980	880.6296	0.9663	1.2418
10	2016	51	9.1857	81.0714	975.1031	1.4204	0.7745
23	2016	51	6.3857	82.0714	778.4194	3.7612	2.1959
27	2016	51	12.6286	81.2551	864.4531	2.1694	1.8133
60	2016	51	8.0286	81.6939	948.7490	1.0724	1.2878
53	2016	51	8.8000	81.9694	859.5571	1.0418	2.4214
66	2016	51	10.2143	76.5306	904.2418	2.0122	2.0459
59	2016	51	7.0429	85.0816	893.6878	1.1327	1.9071
61	2016	51	10.2143	76.6939	973.0265	0.9020	0.5296

84	2016	51	10.2143	76.6939	973.0265	0.9020	0.5296
38	2016	51	7.0429	85.0816	893.6878	1.1327	1.9071
87	2016	51	10.9000	74.5510	906.5071	1.7398	1.9653
34	2016	51	7.0429	85.0816	893.6878	1.1327	1.9071
29	2016	51	9.3429	77.2959	949.5857	0.8704	1.1071
5	2016	51	10.7143	86.6735	836.6531	1.8571	1.7429
8	2016	51	8.8000	81.9694	859.5571	1.0418	2.4214
12	2016	51	10.7143	86.6735	836.6531	1.8571	1.7429
13	2016	51	16.8000	73.0714	952.5102	3.2898	0.8857
18	2016	51	8.8429	81.3265	975.3714	1.3469	0.8031
33	2016	51	8.2286	77.2245	909.3327	1.2112	1.4704
56	2016	51	12.2714	81.6837	987.5806	1.7755	0.6592
77	2016	51	8.8143	82.5918	942.4592	0.7102	0.5724
54	2016	51	10.7143	86.6735	836.6531	1.8571	1.7429
21	2016	51	8.2286	77.2245	909.3327	1.2112	1.4704
68	2016	51	8.8429	77.5306	981.3469	1.2439	1.3020
74	2016	51	10.2143	76.6939	973.0265	0.9020	0.5296
88	2016	51	7.9000	79.1939	881.1214	1.6439	2.4439
16	2016	51	7.9143	81.1837	929.9194	0.8408	1.5092
30	2016	51	10.2143	76.5306	904.2418	2.0122	2.0459
6	2016	51	8.8429	77.5306	981.3469	1.2439	1.3020
49	2016	51	9.3429	77.2959	949.5857	0.8704	1.1071
22	2016	51	7.9000	79.1939	881.1214	1.6439	2.4439
45	2016	51	11.9571	78.1429	821.3071	2.9592	1.5459
58	2016	51	9.3429	77.2959	949.5857	0.8704	1.1071
37	2016	51	8.8429	77.5306	981.3469	1.2439	1.3020
17	2016	51	8.2857	81.6224	909.5480	1.8439	2.5663
55	2016	51	11.5143	77.4082	883.7592	1.0122	1.5194
46	2016	51	7.9143	81.1837	929.9194	0.8408	1.5092
86	2016	51	8.1571	83.5000	871.9888	0.5153	1.2980
2	2016	51	8.1571	83.5000	871.9888	0.5153	1.2980
4	2016	51	8.2286	77.2245	909.3327	1.2112	1.4704
47	2016	51	15.8429	73.2449	967.5102	1.5684	0.5520
82	2016	51	7.9000	79.1939	881.1214	1.6439	2.4439
19	2016	51	13.9714	73.3469	969.7051	2.1990	1.0051
20	2016	51	8.8000	81.9694	859.5571	1.0418	2.4214
80	2016	51	7.9000	79.1939	881.1214	1.6439	2.4439
3	2016	51	16.8000	73.0714	952.5102	3.2898	0.8857
52	2016	51	8.2857	81.6224	909.5480	1.8439	2.5663
70	2016	51	8.9714	79.8878	918.0786	1.0388	1.2653
64	2016	51	6.3857	82.0714	778.4194	3.7612	2.1959
48	2016	51	8.8143	82.5918	942.4592	0.7102	0.5724
65	2016	51	8.2857	81.6224	909.5480	1.8439	2.5663
44	2016	51	8.9714	79.8878	918.0786	1.0388	1.2653
75	2016	51	6.3857	82.0714	778.4194	3.7612	2.1959
40	2016	51	8.7143	83.7143	955.1500	1.8888	1.2143
11	2016	51	11.5143	77.4082	883.7592	1.0122	1.5194
35	2016	51	8.0286	81.6939	948.7490	1.0724	1.2878
78	2016	51	10.9000	74.5510	906.5071	1.7398	1.9653
28	2016	51	8.7857	77.1429	937.8847	1.8316	1.5378

39	2016	51	8.2857	81.6224	909.5480	1.8439	2.5663
24	2016	51	9.3429	77.2959	949.5857	0.8704	1.1071
63	2016	51	8.7143	83.7143	955.1500	1.8888	1.2143
62	2016	51	7.5429	79.3980	880.6296	0.9663	1.2418
1	2016	51	7.9000	79.1939	881.1214	1.6439	2.4439
31	2016	52	5.0286	85.3878	852.4051	0.3061	0.9990
79	2016	52	6.9857	80.6020	983.0000	1.3408	1.2122
51	2016	52	5.1143	83.1939	950.1867	1.1316	1.2500
14	2016	52	7.3714	79.2347	905.3092	1.1316	2.2867
67	2016	52	6.4429	84.8367	910.7429	0.8827	2.5878
42	2016	52	6.5000	82.8878	882.1541	1.0010	2.5551
50	2016	52	6.8143	77.4592	910.7071	0.9929	1.5224
43	2016	52	6.5000	82.8878	882.1541	1.0010	2.5551
85	2016	52	7.6857	79.9694	919.5765	0.9990	1.1071
25	2016	52	9.3286	82.2143	989.0837	1.6020	0.7224
69	2016	52	7.2571	81.6837	951.0367	0.7694	0.9347
57	2016	52	5.8857	86.8367	894.9020	0.6061	1.8071
9	2016	52	6.4286	82.7653	860.4398	0.4092	2.4704
72	2016	52	7.9857	76.2245	884.7204	0.9143	1.9112
26	2016	52	8.8286	81.0714	873.4816	2.4378	1.8806
7	2016	52	7.8571	80.0204	865.2959	1.7735	2.2306
83	2016	52	12.1143	68.5510	953.6776	2.6306	1.0276
76	2016	52	6.8286	83.1020	931.2827	0.8918	1.3776
36	2016	52	6.5571	79.6224	939.2898	1.0867	1.5204
81	2016	52	5.1143	83.1939	950.1867	1.1316	1.2500
15	2016	52	7.6000	82.3469	943.6755	0.8500	0.5908
32	2016	52	6.5000	82.8878	882.1541	1.0010	2.5551
73	2016	52	8.5857	79.4286	974.5592	0.8133	0.5459
71	2016	52	6.5571	79.6224	939.2898	1.0867	1.5204
41	2016	52	7.1714	80.8776	881.8408	0.4531	1.1827
10	2016	52	6.4000	82.9286	976.6969	1.3929	0.8653
23	2016	52	4.1857	84.4286	778.8245	3.2827	2.2316
27	2016	52	7.8571	80.0204	865.2959	1.7735	2.2306
60	2016	52	5.1143	83.1939	950.1867	1.1316	1.2500
53	2016	52	6.4286	82.7653	860.4398	0.4092	2.4704
66	2016	52	7.3714	79.2347	905.3092	1.1316	2.2867
59	2016	52	5.8857	86.8367	894.9020	0.6061	1.8071
61	2016	52	8.5857	79.4286	974.5592	0.8133	0.5459
84	2016	52	8.5857	79.4286	974.5592	0.8133	0.5459
38	2016	52	5.8857	86.8367	894.9020	0.6061	1.8071
87	2016	52	8.4571	76.0816	907.6337	1.0459	2.2357
34	2016	52	5.8857	86.8367	894.9020	0.6061	1.8071
29	2016	52	7.2571	81.6837	951.0367	0.7694	0.9347
5	2016	52	5.7857	86.7755	837.3490	1.7173	1.8592
8	2016	52	6.4286	82.7653	860.4398	0.4092	2.4704
12	2016	52	5.7857	86.7755	837.3490	1.7173	1.8592
13	2016	52	12.1143	68.5510	953.6776	2.6306	1.0276
18	2016	52	6.7571	83.4388	976.9551	0.8061	0.7918
33	2016	52	6.8143	77.4592	910.7071	0.9929	1.5224
56	2016	52	9.3286	82.2143	989.0837	1.6020	0.7224

77	2016	52	7.6000	82.3469	943.6755	0.8500	0.5908
54	2016	52	5.7857	86.7755	837.3490	1.7173	1.8592
21	2016	52	6.8143	77.4592	910.7071	0.9929	1.5224
68	2016	52	6.9857	80.6020	983.0000	1.3408	1.2122
74	2016	52	8.5857	79.4286	974.5592	0.8133	0.5459
88	2016	52	6.5000	82.8878	882.1541	1.0010	2.5551
16	2016	52	6.8286	83.1020	931.2827	0.8918	1.3776
30	2016	52	7.3714	79.2347	905.3092	1.1316	2.2867
6	2016	52	6.9857	80.6020	983.0000	1.3408	1.2122
49	2016	52	7.2571	81.6837	951.0367	0.7694	0.9347
22	2016	52	6.5000	82.8878	882.1541	1.0010	2.5551
45	2016	52	6.9714	78.1429	821.8776	2.5429	2.0316
58	2016	52	7.2571	81.6837	951.0367	0.7694	0.9347
37	2016	52	6.9857	80.6020	983.0000	1.3408	1.2122
17	2016	52	6.4429	84.8367	910.7429	0.8827	2.5878
55	2016	52	7.9857	76.2245	884.7204	0.9143	1.9112
46	2016	52	6.8286	83.1020	931.2827	0.8918	1.3776
86	2016	52	7.4143	84.9898	873.1092	0.3408	1.2571
2	2016	52	7.4143	84.9898	873.1092	0.3408	1.2571
4	2016	52	6.8143	77.4592	910.7071	0.9929	1.5224
47	2016	52	11.3571	71.4082	968.7827	1.8337	0.5827
82	2016	52	6.5000	82.8878	882.1541	1.0010	2.5551
19	2016	52	10.8714	72.9592	971.0867	1.7204	0.9867
20	2016	52	6.4286	82.7653	860.4398	0.4092	2.4704
80	2016	52	6.5000	82.8878	882.1541	1.0010	2.5551
3	2016	52	12.1143	68.5510	953.6776	2.6306	1.0276
52	2016	52	6.4429	84.8367	910.7429	0.8827	2.5878
70	2016	52	7.6857	79.9694	919.5765	0.9990	1.1071
64	2016	52	4.1857	84.4286	778.8245	3.2827	2.2316
48	2016	52	7.6000	82.3469	943.6755	0.8500	0.5908
65	2016	52	6.4429	84.8367	910.7429	0.8827	2.5878
44	2016	52	7.6857	79.9694	919.5765	0.9990	1.1071
75	2016	52	4.1857	84.4286	778.8245	3.2827	2.2316
40	2016	52	6.1000	85.5204	956.5541	1.8878	1.2673
11	2016	52	7.9857	76.2245	884.7204	0.9143	1.9112
35	2016	52	5.1143	83.1939	950.1867	1.1316	1.2500
78	2016	52	8.4571	76.0816	907.6337	1.0459	2.2357
28	2016	52	6.5571	79.6224	939.2898	1.0867	1.5204
39	2016	52	6.4429	84.8367	910.7429	0.8827	2.5878
24	2016	52	7.2571	81.6837	951.0367	0.7694	0.9347
63	2016	52	6.1000	85.5204	956.5541	1.8878	1.2673
62	2016	52	7.1714	80.8776	881.8408	0.4531	1.1827
1	2016	52	6.5000	82.8878	882.1541	1.0010	2.5551
31	2017	1	8.7429	82.6020	852.3929	1.0224	0.9602
79	2017	1	9.5857	78.8061	982.8786	1.4245	1.3194
51	2017	1	8.7571	83.7143	950.0663	1.5194	1.1633
14	2017	1	12.2429	78.7245	905.5429	1.2031	2.1714
67	2017	1	11.5429	83.9694	910.7643	0.8541	2.9102
42	2017	1	10.9000	83.0000	882.2541	1.4031	2.4408
50	2017	1	10.1429	78.1837	910.5367	1.1327	1.5755

43	2017	1	10.9000	83.0000	882.2541	1.4031	2.4408
85	2017	1	10.4571	79.4796	919.3806	1.4214	1.1061
25	2017	1	14.8000	81.7857	988.9194	1.9806	0.8276
69	2017	1	11.7571	81.5918	950.7449	1.2786	1.0010
57	2017	1	10.7286	85.6327	894.7592	1.1878	1.9469
9	2017	1	10.5286	82.3265	860.7041	1.1306	2.3061
72	2017	1	12.3286	77.6020	885.0929	1.6337	1.9031
26	2017	1	13.3571	83.1122	874.1020	2.2255	1.8867
7	2017	1	12.9143	79.4898	865.8051	2.2071	2.0571
83	2017	1	16.1571	72.9286	954.2561	2.4469	1.0102
76	2017	1	10.0571	81.5918	930.9949	1.3102	1.3469
36	2017	1	10.9286	79.5408	939.2010	1.3276	1.4510
81	2017	1	8.7571	83.7143	950.0663	1.5194	1.1633
15	2017	1	9.7857	82.1633	938.1796	0.6612	0.9796
32	2017	1	10.9000	83.0000	882.2541	1.4031	2.4408
73	2017	1	11.8714	77.1429	974.2204	0.8959	0.5990
71	2017	1	10.9286	79.5408	939.2010	1.3276	1.4510
41	2017	1	10.8000	77.7041	881.7276	1.1388	1.2888
10	2017	1	9.8714	82.8571	976.5939	1.4776	0.7980
23	2017	1	8.0143	78.5204	779.3010	4.3745	2.2776
27	2017	1	12.9143	79.4898	865.8051	2.2071	2.0571
60	2017	1	8.7571	83.7143	950.0663	1.5194	1.1633
53	2017	1	10.5286	82.3265	860.7041	1.1306	2.3061
66	2017	1	12.2429	78.7245	905.5429	1.2031	2.1714
59	2017	1	10.7286	85.6327	894.7592	1.1878	1.9469
61	2017	1	11.8714	77.1429	974.2204	0.8959	0.5990
84	2017	1	11.8714	77.1429	974.2204	0.8959	0.5990
38	2017	1	10.7286	85.6327	894.7592	1.1878	1.9469
87	2017	1	12.5571	78.2245	907.8051	1.6102	2.0929
34	2017	1	10.7286	85.6327	894.7592	1.1878	1.9469
29	2017	1	11.7571	81.5918	950.7449	1.2786	1.0010
5	2017	1	11.0429	83.7551	837.8306	2.6510	1.7480
8	2017	1	10.5286	82.3265	860.7041	1.1306	2.3061
12	2017	1	11.0429	83.7551	837.8306	2.6510	1.7480
13	2017	1	16.1571	72.9286	954.2561	2.4469	1.0102
18	2017	1	9.7571	81.5306	976.8214	0.7786	0.7633
33	2017	1	10.1429	78.1837	910.5367	1.1327	1.5755
56	2017	1	14.8000	81.7857	988.9194	1.9806	0.8276
77	2017	1	9.7857	82.1633	938.1796	0.6612	0.9796
54	2017	1	11.0429	83.7551	837.8306	2.6510	1.7480
21	2017	1	10.1429	78.1837	910.5367	1.1327	1.5755
68	2017	1	9.5857	78.8061	982.8786	1.4245	1.3194
74	2017	1	11.8714	77.1429	974.2204	0.8959	0.5990
88	2017	1	10.9000	83.0000	882.2541	1.4031	2.4408
16	2017	1	10.0571	81.5918	930.9949	1.3102	1.3469
30	2017	1	12.2429	78.7245	905.5429	1.2031	2.1714
6	2017	1	9.5857	78.8061	982.8786	1.4245	1.3194
49	2017	1	11.7571	81.5918	950.7449	1.2786	1.0010
22	2017	1	10.9000	83.0000	882.2541	1.4031	2.4408
45	2017	1	10.9714	75.6122	822.5418	3.6378	2.2378

58	2017	1	11.7571	81.5918	950.7449	1.2786	1.0010
37	2017	1	9.5857	78.8061	982.8786	1.4245	1.3194
17	2017	1	11.5429	83.9694	910.7643	0.8541	2.9102
55	2017	1	12.3286	77.6020	885.0929	1.6337	1.9031
46	2017	1	10.0571	81.5918	930.9949	1.3102	1.3469
86	2017	1	11.1857	81.4286	873.1245	0.7735	1.7898
2	2017	1	11.1857	81.4286	873.1245	0.7735	1.7898
4	2017	1	10.1429	78.1837	910.5367	1.1327	1.5755
47	2017	1	15.9714	75.9082	969.1806	2.3755	0.5806
82	2017	1	10.9000	83.0000	882.2541	1.4031	2.4408
19	2017	1	15.6000	72.8163	971.2163	2.2031	0.9745
20	2017	1	10.5286	82.3265	860.7041	1.1306	2.3061
80	2017	1	10.9000	83.0000	882.2541	1.4031	2.4408
3	2017	1	16.1571	72.9286	954.2561	2.4469	1.0102
52	2017	1	11.5429	83.9694	910.7643	0.8541	2.9102
70	2017	1	10.4571	79.4796	919.3806	1.4214	1.1061
64	2017	1	8.0143	78.5204	779.3010	4.3745	2.2776
48	2017	1	9.7857	82.1633	938.1796	0.6612	0.9796
65	2017	1	11.5429	83.9694	910.7643	0.8541	2.9102
44	2017	1	10.4571	79.4796	919.3806	1.4214	1.1061
75	2017	1	8.0143	78.5204	779.3010	4.3745	2.2776
40	2017	1	10.8857	84.5408	956.4959	2.3000	1.2000
11	2017	1	12.3286	77.6020	885.0929	1.6337	1.9031
35	2017	1	8.7571	83.7143	950.0663	1.5194	1.1633
78	2017	1	12.5571	78.2245	907.8051	1.6102	2.0929
28	2017	1	10.9286	79.5408	939.2010	1.3276	1.4510
39	2017	1	11.5429	83.9694	910.7643	0.8541	2.9102
24	2017	1	11.7571	81.5918	950.7449	1.2786	1.0010
63	2017	1	10.8857	84.5408	956.4959	2.3000	1.2000
62	2017	1	10.8000	77.7041	881.7276	1.1388	1.2888
1	2017	1	10.9000	83.0000	882.2541	1.4031	2.4408
31	2017	2	3.0429	82.0408	850.3194	1.2510	1.0071
79	2017	2	6.3714	81.0408	980.7520	0.4949	1.5347
51	2017	2	4.4571	87.7245	947.9571	0.5878	1.3684
14	2017	2	4.7429	84.9286	903.5337	0.6714	1.9061
67	2017	2	3.6714	88.2347	908.7194	0.4969	3.2520
42	2017	2	3.2571	86.9694	880.2092	1.1143	2.4327
50	2017	2	4.7429	80.1020	908.3551	0.6153	1.6551
43	2017	2	3.2571	86.9694	880.2092	1.1143	2.4327
85	2017	2	5.3429	81.7041	917.1694	1.1418	1.3000
25	2017	2	8.6286	82.4694	986.6898	1.1765	0.9704
69	2017	2	6.0143	82.4796	948.5378	0.6520	1.2000
57	2017	2	3.3714	86.0918	892.6347	1.2551	2.0337
9	2017	2	4.2571	86.6327	858.6776	1.1337	2.4245
72	2017	2	6.4143	84.0408	882.9980	1.3776	1.7582
26	2017	2	8.6857	85.5510	872.0327	2.9122	1.8643
7	2017	2	7.5714	81.3673	863.7378	3.2724	1.7204
83	2017	2	12.1000	79.4286	951.8816	2.3622	0.9776
76	2017	2	4.4857	85.0102	928.8582	0.6551	1.4663
36	2017	2	4.8000	83.7755	937.0867	0.7347	1.6041

81	2017	2	4.4571	87.7245	947.9571	0.5878	1.3684
15	2017	2	4.7286	85.5000	928.5357	0.2531	1.3633
32	2017	2	3.2571	86.9694	880.2092	1.1143	2.4327
73	2017	2	7.2571	76.5408	972.0276	0.3888	0.6633
71	2017	2	4.8000	83.7755	937.0867	0.7347	1.6041
41	2017	2	3.8571	79.3163	879.6051	1.1031	1.4051
10	2017	2	5.8857	86.7041	974.4724	0.4643	0.8296
23	2017	2	2.4286	74.0816	777.5765	5.1908	2.1714
27	2017	2	7.5714	81.3673	863.7378	3.2724	1.7204
60	2017	2	4.4571	87.7245	947.9571	0.5878	1.3684
53	2017	2	4.2571	86.6327	858.6776	1.1337	2.4245
66	2017	2	4.7429	84.9286	903.5337	0.6714	1.9061
59	2017	2	3.3714	86.0918	892.6347	1.2551	2.0337
61	2017	2	7.2571	76.5408	972.0276	0.3888	0.6633
84	2017	2	7.2571	76.5408	972.0276	0.3888	0.6633
38	2017	2	3.3714	86.0918	892.6347	1.2551	2.0337
87	2017	2	6.3429	83.4796	905.7041	1.3480	2.1663
34	2017	2	3.3714	86.0918	892.6347	1.2551	2.0337
29	2017	2	6.0143	82.4796	948.5378	0.6520	1.2000
5	2017	2	5.6714	81.8061	835.8908	3.6939	1.8459
8	2017	2	4.2571	86.6327	858.6776	1.1337	2.4245
12	2017	2	5.6714	81.8061	835.8908	3.6939	1.8459
13	2017	2	12.1000	79.4286	951.8816	2.3622	0.9776
18	2017	2	6.2143	82.8673	974.7378	0.2357	0.8888
33	2017	2	4.7429	80.1020	908.3551	0.6153	1.6551
56	2017	2	8.6286	82.4694	986.6898	1.1765	0.9704
77	2017	2	4.7286	85.5000	928.5357	0.2531	1.3633
54	2017	2	5.6714	81.8061	835.8908	3.6939	1.8459
21	2017	2	4.7429	80.1020	908.3551	0.6153	1.6551
68	2017	2	6.3714	81.0408	980.7520	0.4949	1.5347
74	2017	2	7.2571	76.5408	972.0276	0.3888	0.6633
88	2017	2	3.2571	86.9694	880.2092	1.1143	2.4327
16	2017	2	4.4857	85.0102	928.8582	0.6551	1.4663
30	2017	2	4.7429	84.9286	903.5337	0.6714	1.9061
6	2017	2	6.3714	81.0408	980.7520	0.4949	1.5347
49	2017	2	6.0143	82.4796	948.5378	0.6520	1.2000
22	2017	2	3.2571	86.9694	880.2092	1.1143	2.4327
45	2017	2	6.7143	72.3061	820.8020	4.6398	2.1714
58	2017	2	6.0143	82.4796	948.5378	0.6520	1.2000
37	2017	2	6.3714	81.0408	980.7520	0.4949	1.5347
17	2017	2	3.6714	88.2347	908.7194	0.4969	3.2520
55	2017	2	6.4143	84.0408	882.9980	1.3776	1.7582
46	2017	2	4.4857	85.0102	928.8582	0.6551	1.4663
86	2017	2	4.5571	84.4796	871.0408	0.7418	1.8714
2	2017	2	4.5571	84.4796	871.0408	0.7418	1.8714
4	2017	2	4.7429	80.1020	908.3551	0.6153	1.6551
47	2017	2	10.8857	80.9388	966.9735	1.6571	0.6112
82	2017	2	3.2571	86.9694	880.2092	1.1143	2.4327
19	2017	2	9.0143	78.4694	969.1194	1.2255	1.1306
20	2017	2	4.2571	86.6327	858.6776	1.1337	2.4245

80	2017	2	3.2571	86.9694	880.2092	1.1143	2.4327
3	2017	2	12.1000	79.4286	951.8816	2.3622	0.9776
52	2017	2	3.6714	88.2347	908.7194	0.4969	3.2520
70	2017	2	5.3429	81.7041	917.1694	1.1418	1.3000
64	2017	2	2.4286	74.0816	777.5765	5.1908	2.1714
48	2017	2	4.7286	85.5000	928.5357	0.2531	1.3633
65	2017	2	3.6714	88.2347	908.7194	0.4969	3.2520
44	2017	2	5.3429	81.7041	917.1694	1.1418	1.3000
75	2017	2	2.4286	74.0816	777.5765	5.1908	2.1714
40	2017	2	4.9143	88.5612	954.3867	0.9296	1.4173
11	2017	2	6.4143	84.0408	882.9980	1.3776	1.7582
35	2017	2	4.4571	87.7245	947.9571	0.5878	1.3684
78	2017	2	6.3429	83.4796	905.7041	1.3480	2.1663
28	2017	2	4.8000	83.7755	937.0867	0.7347	1.6041
39	2017	2	3.6714	88.2347	908.7194	0.4969	3.2520
24	2017	2	6.0143	82.4796	948.5378	0.6520	1.2000
63	2017	2	4.9143	88.5612	954.3867	0.9296	1.4173
62	2017	2	3.8571	79.3163	879.6051	1.1031	1.4051
1	2017	2	3.2571	86.9694	880.2092	1.1143	2.4327
31	2017	3	3.3857	83.6327	850.3755	0.4041	1.1245
79	2017	3	6.3857	78.3469	981.8480	0.2520	1.5714
51	2017	3	4.1286	87.5510	948.8724	0.1469	1.6163
14	2017	3	4.3429	90.2449	903.6245	0.2255	1.7684
67	2017	3	3.7429	94.0204	909.1857	0.0816	2.9173
42	2017	3	3.1714	92.5612	880.3592	0.2214	2.5510
50	2017	3	4.8857	80.6327	909.0602	0.0857	1.7173
43	2017	3	3.1714	92.5612	880.3592	0.2214	2.5510
85	2017	3	5.3571	84.7449	917.9673	0.4867	1.3092
25	2017	3	8.2857	80.3673	987.9388	0.4245	1.1102
69	2017	3	5.4286	83.3163	949.6337	0.1112	1.3296
57	2017	3	3.2143	91.1224	893.1827	0.3694	1.6704
9	2017	3	3.8429	90.8469	858.4571	0.2622	2.4929
72	2017	3	5.7429	87.4082	882.8694	0.4408	1.9041
26	2017	3	6.8714	90.2449	871.5184	2.0673	1.5633
7	2017	3	6.4857	86.4490	863.2918	2.4071	1.8031
83	2017	3	10.6286	81.2041	952.1714	1.3071	1.0990
76	2017	3	4.5429	87.6837	929.7684	0.1143	1.5490
36	2017	3	4.5000	86.0102	937.9082	0.2561	1.8816
81	2017	3	4.1286	87.5510	948.8724	0.1469	1.6163
15	2017	3	5.3857	82.6429	927.0184	0.3041	1.4051
32	2017	3	3.1714	92.5612	880.3592	0.2214	2.5510
73	2017	3	7.3286	76.0408	973.2398	0.3765	0.8173
71	2017	3	4.5000	86.0102	937.9082	0.2561	1.8816
41	2017	3	3.7000	85.7551	880.0173	0.2663	1.3643
10	2017	3	5.3571	87.2347	975.5316	0.1541	0.7378
23	2017	3	2.6429	80.0714	776.5469	4.5388	2.1184
27	2017	3	6.4857	86.4490	863.2918	2.4071	1.8031
60	2017	3	4.1286	87.5510	948.8724	0.1469	1.6163
53	2017	3	3.8429	90.8469	858.4571	0.2622	2.4929
66	2017	3	4.3429	90.2449	903.6245	0.2255	1.7684

59	2017	3	3.2143	91.1224	893.1827	0.3694	1.6704
61	2017	3	7.3286	76.0408	973.2398	0.3765	0.8173
84	2017	3	7.3286	76.0408	973.2398	0.3765	0.8173
38	2017	3	3.2143	91.1224	893.1827	0.3694	1.6704
87	2017	3	5.8286	86.0000	905.9765	0.2827	2.4796
34	2017	3	3.2143	91.1224	893.1827	0.3694	1.6704
29	2017	3	5.4286	83.3163	949.6337	0.1112	1.3296
5	2017	3	4.1000	89.0714	835.2735	2.4041	1.9071
8	2017	3	3.8429	90.8469	858.4571	0.2622	2.4929
12	2017	3	4.1000	89.0714	835.2735	2.4041	1.9071
13	2017	3	10.6286	81.2041	952.1714	1.3071	1.0990
18	2017	3	6.3143	78.9796	975.8286	0.3959	1.0735
33	2017	3	4.8857	80.6327	909.0602	0.0857	1.7173
56	2017	3	8.2857	80.3673	987.9388	0.4245	1.1102
77	2017	3	5.3857	82.6429	927.0184	0.3041	1.4051
54	2017	3	4.1000	89.0714	835.2735	2.4041	1.9071
21	2017	3	4.8857	80.6327	909.0602	0.0857	1.7173
68	2017	3	6.3857	78.3469	981.8480	0.2520	1.5714
74	2017	3	7.3286	76.0408	973.2398	0.3765	0.8173
88	2017	3	3.1714	92.5612	880.3592	0.2214	2.5510
16	2017	3	4.5429	87.6837	929.7684	0.1143	1.5490
30	2017	3	4.3429	90.2449	903.6245	0.2255	1.7684
6	2017	3	6.3857	78.3469	981.8480	0.2520	1.5714
49	2017	3	5.4286	83.3163	949.6337	0.1112	1.3296
22	2017	3	3.1714	92.5612	880.3592	0.2214	2.5510
45	2017	3	6.4143	78.2551	819.8092	3.9051	2.0296
58	2017	3	5.4286	83.3163	949.6337	0.1112	1.3296
37	2017	3	6.3857	78.3469	981.8480	0.2520	1.5714
17	2017	3	3.7429	94.0204	909.1857	0.0816	2.9173
55	2017	3	5.7429	87.4082	882.8694	0.4408	1.9041
46	2017	3	4.5429	87.6837	929.7684	0.1143	1.5490
86	2017	3	4.4286	90.8980	871.2224	0.1878	1.2367
2	2017	3	4.4286	90.8980	871.2224	0.1878	1.2367
4	2017	3	4.8857	80.6327	909.0602	0.0857	1.7173
47	2017	3	10.1857	81.3878	967.6500	0.4969	0.6133
82	2017	3	3.1714	92.5612	880.3592	0.2214	2.5510
19	2017	3	8.5714	78.4490	969.8898	0.3214	1.3653
20	2017	3	3.8429	90.8469	858.4571	0.2622	2.4929
80	2017	3	3.1714	92.5612	880.3592	0.2214	2.5510
3	2017	3	10.6286	81.2041	952.1714	1.3071	1.0990
52	2017	3	3.7429	94.0204	909.1857	0.0816	2.9173
70	2017	3	5.3571	84.7449	917.9673	0.4867	1.3092
64	2017	3	2.6429	80.0714	776.5469	4.5388	2.1184
48	2017	3	5.3857	82.6429	927.0184	0.3041	1.4051
65	2017	3	3.7429	94.0204	909.1857	0.0816	2.9173
44	2017	3	5.3571	84.7449	917.9673	0.4867	1.3092
75	2017	3	2.6429	80.0714	776.5469	4.5388	2.1184
40	2017	3	4.5571	91.4490	955.2112	0.1898	1.6755
11	2017	3	5.7429	87.4082	882.8694	0.4408	1.9041
35	2017	3	4.1286	87.5510	948.8724	0.1469	1.6163

78	2017	3	5.8286	86.0000	905.9765	0.2827	2.4796
28	2017	3	4.5000	86.0102	937.9082	0.2561	1.8816
39	2017	3	3.7429	94.0204	909.1857	0.0816	2.9173
24	2017	3	5.4286	83.3163	949.6337	0.1112	1.3296
63	2017	3	4.5571	91.4490	955.2112	0.1898	1.6755
62	2017	3	3.7000	85.7551	880.0173	0.2663	1.3643
1	2017	3	3.1714	92.5612	880.3592	0.2214	2.5510
31	2017	4	6.9143	85.2551	853.2388	0.4020	1.0500
79	2017	4	10.2857	67.5102	984.7204	1.3633	1.2153
51	2017	4	9.6000	78.0918	951.8541	0.8184	1.3439
14	2017	4	10.3857	82.3265	906.8306	0.6265	1.7082
67	2017	4	10.0143	83.7959	912.2959	0.0612	2.5408
42	2017	4	8.6286	86.2143	883.4510	0.2245	2.5000
50	2017	4	8.2429	74.5000	912.2153	0.1643	1.6082
43	2017	4	8.6286	86.2143	883.4510	0.2245	2.5000
85	2017	4	9.2857	79.9694	921.1235	0.6357	1.1918
25	2017	4	13.2429	75.3673	991.2888	1.1908	0.9531
69	2017	4	10.7857	78.3163	952.8143	0.5520	1.1378
57	2017	4	8.7286	86.4388	896.3112	0.2520	1.6367
9	2017	4	7.7857	90.2347	861.5357	0.1531	2.1296
72	2017	4	9.6714	84.7755	886.1163	0.2122	1.6092
26	2017	4	10.5714	94.4184	874.7378	0.9000	1.0939
7	2017	4	9.6714	90.1939	866.4235	0.7061	1.8306
83	2017	4	13.5286	81.4592	956.0102	0.3265	0.9214
76	2017	4	8.3714	80.0000	932.9143	0.5051	1.3878
36	2017	4	11.0286	77.4184	941.0092	0.6765	1.5816
81	2017	4	9.6000	78.0918	951.8541	0.8184	1.3439
15	2017	4	7.4714	75.9490	930.2082	0.8102	1.3398
32	2017	4	8.6286	86.2143	883.4510	0.2245	2.5000
73	2017	4	10.5143	71.9694	976.3929	1.0337	0.7235
71	2017	4	11.0286	77.4184	941.0092	0.6765	1.5816
41	2017	4	8.2857	84.9184	883.1163	0.2500	1.3194
10	2017	4	10.6286	78.6327	978.5031	1.0143	0.4786
23	2017	4	5.6143	86.0510	779.0633	3.4133	2.1143
27	2017	4	9.6714	90.1939	866.4235	0.7061	1.8306
60	2017	4	9.6000	78.0918	951.8541	0.8184	1.3439
53	2017	4	7.7857	90.2347	861.5357	0.1531	2.1296
66	2017	4	10.3857	82.3265	906.8306	0.6265	1.7082
59	2017	4	8.7286	86.4388	896.3112	0.2520	1.6367
61	2017	4	10.5143	71.9694	976.3929	1.0337	0.7235
84	2017	4	10.5143	71.9694	976.3929	1.0337	0.7235
38	2017	4	8.7286	86.4388	896.3112	0.2520	1.6367
87	2017	4	11.0714	79.5102	909.2551	0.1622	2.1827
34	2017	4	8.7286	86.4388	896.3112	0.2520	1.6367
29	2017	4	10.7857	78.3163	952.8143	0.5520	1.1378
5	2017	4	8.4571	95.2449	838.2367	0.9133	1.7663
8	2017	4	7.7857	90.2347	861.5357	0.1531	2.1296
12	2017	4	8.4571	95.2449	838.2367	0.9133	1.7663
13	2017	4	13.5286	81.4592	956.0102	0.3265	0.9214
18	2017	4	9.3000	70.5204	978.7357	1.4388	0.9418

33	2017	4	8.2429	74.5000	912.2153	0.1643	1.6082
56	2017	4	13.2429	75.3673	991.2888	1.1908	0.9531
77	2017	4	7.4714	75.9490	930.2082	0.8102	1.3398
54	2017	4	8.4571	95.2449	838.2367	0.9133	1.7663
21	2017	4	8.2429	74.5000	912.2153	0.1643	1.6082
68	2017	4	10.2857	67.5102	984.7204	1.3633	1.2153
74	2017	4	10.5143	71.9694	976.3929	1.0337	0.7235
88	2017	4	8.6286	86.2143	883.4510	0.2245	2.5000
16	2017	4	8.3714	80.0000	932.9143	0.5051	1.3878
30	2017	4	10.3857	82.3265	906.8306	0.6265	1.7082
6	2017	4	10.2857	67.5102	984.7204	1.3633	1.2153
49	2017	4	10.7857	78.3163	952.8143	0.5520	1.1378
22	2017	4	8.6286	86.2143	883.4510	0.2245	2.5000
45	2017	4	10.0143	85.4490	822.5143	2.6051	1.8102
58	2017	4	10.7857	78.3163	952.8143	0.5520	1.1378
37	2017	4	10.2857	67.5102	984.7204	1.3633	1.2153
17	2017	4	10.0143	83.7959	912.2959	0.0612	2.5408
55	2017	4	9.6714	84.7755	886.1163	0.2122	1.6092
46	2017	4	8.3714	80.0000	932.9143	0.5051	1.3878
86	2017	4	8.4714	91.4184	874.2908	0.0510	1.0918
2	2017	4	8.4714	91.4184	874.2908	0.0510	1.0918
4	2017	4	8.2429	74.5000	912.2153	0.1643	1.6082
47	2017	4	14.2286	76.4388	971.3327	0.5388	0.5714
82	2017	4	8.6286	86.2143	883.4510	0.2245	2.5000
19	2017	4	13.6571	72.8980	973.3276	0.6418	1.1806
20	2017	4	7.7857	90.2347	861.5357	0.1531	2.1296
80	2017	4	8.6286	86.2143	883.4510	0.2245	2.5000
3	2017	4	13.5286	81.4592	956.0102	0.3265	0.9214
52	2017	4	10.0143	83.7959	912.2959	0.0612	2.5408
70	2017	4	9.2857	79.9694	921.1235	0.6357	1.1918
64	2017	4	5.6143	86.0510	779.0633	3.4133	2.1143
48	2017	4	7.4714	75.9490	930.2082	0.8102	1.3398
65	2017	4	10.0143	83.7959	912.2959	0.0612	2.5408
44	2017	4	9.2857	79.9694	921.1235	0.6357	1.1918
75	2017	4	5.6143	86.0510	779.0633	3.4133	2.1143
40	2017	4	11.3714	81.9796	958.2969	1.4276	1.3929
11	2017	4	9.6714	84.7755	886.1163	0.2122	1.6092
35	2017	4	9.6000	78.0918	951.8541	0.8184	1.3439
78	2017	4	11.0714	79.5102	909.2551	0.1622	2.1827
28	2017	4	11.0286	77.4184	941.0092	0.6765	1.5816
39	2017	4	10.0143	83.7959	912.2959	0.0612	2.5408
24	2017	4	10.7857	78.3163	952.8143	0.5520	1.1378
63	2017	4	11.3714	81.9796	958.2969	1.4276	1.3929
62	2017	4	8.2857	84.9184	883.1163	0.2500	1.3194
1	2017	4	8.6286	86.2143	883.4510	0.2245	2.5000
31	2017	5	5.7714	84.6122	852.4908	1.4673	1.1878
79	2017	5	5.4714	66.4388	983.8469	1.5163	1.6429
51	2017	5	4.3143	75.0102	950.9051	1.0000	1.5551
14	2017	5	5.6714	79.4490	906.2663	0.6980	2.0520
67	2017	5	4.4714	80.0204	911.5296	0.3735	2.7745

42	2017	5	4.8000	83.8061	882.7398	0.7541	2.6510
50	2017	5	5.6571	75.8163	911.2061	0.2306	1.7286
43	2017	5	4.8000	83.8061	882.7398	0.7541	2.6510
85	2017	5	6.2143	78.7041	919.9806	0.9673	1.3214
25	2017	5	8.7429	72.9694	990.0755	1.3480	1.0714
69	2017	5	6.1714	75.0612	951.6776	0.8204	1.4031
57	2017	5	4.4286	83.8061	895.3898	0.9316	2.0459
9	2017	5	6.3286	90.0204	861.0224	0.7908	2.2571
72	2017	5	7.6714	84.5918	885.5204	0.8235	1.6541
26	2017	5	12.6143	89.1020	874.1980	2.8765	1.6112
7	2017	5	11.3714	86.9082	865.9245	2.5531	2.0112
83	2017	5	12.7429	80.3367	954.9571	1.0153	0.8265
76	2017	5	5.1000	77.9490	931.9010	0.7806	1.4020
36	2017	5	4.8286	73.4796	940.0173	0.9612	1.5571
81	2017	5	4.3143	75.0102	950.9051	1.0000	1.5551
15	2017	5	5.5143	79.2755	929.2337	0.9663	1.5969
32	2017	5	4.8000	83.8061	882.7398	0.7541	2.6510
73	2017	5	6.9571	70.5510	975.3010	0.8561	0.6224
71	2017	5	4.8286	73.4796	940.0173	0.9612	1.5571
41	2017	5	5.5143	83.4388	882.2541	1.0469	1.5265
10	2017	5	5.2286	74.8163	977.4551	1.1949	0.7306
23	2017	5	8.5714	79.5612	779.1429	4.3969	2.4194
27	2017	5	11.3714	86.9082	865.9245	2.5531	2.0112
60	2017	5	4.3143	75.0102	950.9051	1.0000	1.5551
53	2017	5	6.3286	90.0204	861.0224	0.7908	2.2571
66	2017	5	5.6714	79.4490	906.2663	0.6980	2.0520
59	2017	5	4.4286	83.8061	895.3898	0.9316	2.0459
61	2017	5	6.9571	70.5510	975.3010	0.8561	0.6224
84	2017	5	6.9571	70.5510	975.3010	0.8561	0.6224
38	2017	5	4.4286	83.8061	895.3898	0.9316	2.0459
87	2017	5	7.0714	78.9694	908.4490	0.6980	2.0806
34	2017	5	4.4286	83.8061	895.3898	0.9316	2.0459
29	2017	5	6.1714	75.0612	951.6776	0.8204	1.4031
5	2017	5	9.6429	88.6939	837.8714	3.1571	2.0255
8	2017	5	6.3286	90.0204	861.0224	0.7908	2.2571
12	2017	5	9.6429	88.6939	837.8714	3.1571	2.0255
13	2017	5	12.7429	80.3367	954.9571	1.0153	0.8265
18	2017	5	5.6714	70.8469	977.9000	1.3031	1.0143
33	2017	5	5.6571	75.8163	911.2061	0.2306	1.7286
56	2017	5	8.7429	72.9694	990.0755	1.3480	1.0714
77	2017	5	5.5143	79.2755	929.2337	0.9663	1.5969
54	2017	5	9.6429	88.6939	837.8714	3.1571	2.0255
21	2017	5	5.6571	75.8163	911.2061	0.2306	1.7286
68	2017	5	5.4714	66.4388	983.8469	1.5163	1.6429
74	2017	5	6.9571	70.5510	975.3010	0.8561	0.6224
88	2017	5	4.8000	83.8061	882.7398	0.7541	2.6510
16	2017	5	5.1000	77.9490	931.9010	0.7806	1.4020
30	2017	5	5.6714	79.4490	906.2663	0.6980	2.0520
6	2017	5	5.4714	66.4388	983.8469	1.5163	1.6429
49	2017	5	6.1714	75.0612	951.6776	0.8204	1.4031

22	2017	5	4.8000	83.8061	882.7398	0.7541	2.6510
45	2017	5	11.9000	76.3776	822.3918	4.4071	2.1837
58	2017	5	6.1714	75.0612	951.6776	0.8204	1.4031
37	2017	5	5.4714	66.4388	983.8469	1.5163	1.6429
17	2017	5	4.4714	80.0204	911.5296	0.3735	2.7745
55	2017	5	7.6714	84.5918	885.5204	0.8235	1.6541
46	2017	5	5.1000	77.9490	931.9010	0.7806	1.4020
86	2017	5	6.9000	90.3571	873.5204	0.2633	1.5020
2	2017	5	6.9000	90.3571	873.5204	0.2633	1.5020
4	2017	5	5.6571	75.8163	911.2061	0.2306	1.7286
47	2017	5	11.3286	75.4286	970.1643	0.9724	0.6173
82	2017	5	4.8000	83.8061	882.7398	0.7541	2.6510
19	2017	5	9.8143	72.2959	972.3092	0.7245	1.1041
20	2017	5	6.3286	90.0204	861.0224	0.7908	2.2571
80	2017	5	4.8000	83.8061	882.7398	0.7541	2.6510
3	2017	5	12.7429	80.3367	954.9571	1.0153	0.8265
52	2017	5	4.4714	80.0204	911.5296	0.3735	2.7745
70	2017	5	6.2143	78.7041	919.9806	0.9673	1.3214
64	2017	5	8.5714	79.5612	779.1429	4.3969	2.4194
48	2017	5	5.5143	79.2755	929.2337	0.9663	1.5969
65	2017	5	4.4714	80.0204	911.5296	0.3735	2.7745
44	2017	5	6.2143	78.7041	919.9806	0.9673	1.3214
75	2017	5	8.5714	79.5612	779.1429	4.3969	2.4194
40	2017	5	4.8857	76.1122	957.3980	1.8500	1.6337
11	2017	5	7.6714	84.5918	885.5204	0.8235	1.6541
35	2017	5	4.3143	75.0102	950.9051	1.0000	1.5551
78	2017	5	7.0714	78.9694	908.4490	0.6980	2.0806
28	2017	5	4.8286	73.4796	940.0173	0.9612	1.5571
39	2017	5	4.4714	80.0204	911.5296	0.3735	2.7745
24	2017	5	6.1714	75.0612	951.6776	0.8204	1.4031
63	2017	5	4.8857	76.1122	957.3980	1.8500	1.6337
62	2017	5	5.5143	83.4388	882.2541	1.0469	1.5265
1	2017	5	4.8000	83.8061	882.7398	0.7541	2.6510
31	2017	6	3.4429	83.0408	849.8990	2.1765	1.2327
79	2017	6	6.3286	74.7959	981.5276	1.2031	1.8429
51	2017	6	4.8714	80.8673	948.4214	1.0469	1.8857
14	2017	6	6.6857	84.9184	903.5449	0.7786	2.4357
67	2017	6	5.1857	88.2143	908.8388	0.9969	2.5449
42	2017	6	5.7571	86.8367	880.0122	1.3306	2.5480
50	2017	6	6.1000	80.2347	908.3520	0.9347	1.5837
43	2017	6	5.7571	86.8367	880.0122	1.3306	2.5480
85	2017	6	6.2000	83.1837	917.1908	1.0061	1.4153
25	2017	6	9.8286	75.8163	987.4347	0.9255	1.2214
69	2017	6	6.6857	77.6531	948.9816	1.2327	1.5776
57	2017	6	5.2714	85.6837	892.5878	1.4888	2.2418
9	2017	6	5.5571	87.7449	858.2898	1.4510	2.4153
72	2017	6	7.3714	84.7857	882.7133	1.3439	1.9500
26	2017	6	8.7857	82.7041	871.2153	4.4714	2.1724
7	2017	6	8.0857	80.6735	863.0735	4.3459	2.2306
83	2017	6	12.8143	77.4184	951.7531	1.4490	0.9673

76	2017	6	5.6143	82.6020	929.1071	1.2378	1.5143
36	2017	6	5.8286	79.3673	937.3949	1.1490	1.7837
81	2017	6	4.8714	80.8673	948.4214	1.0469	1.8857
15	2017	6	6.2429	82.1735	926.3388	1.3561	1.6776
32	2017	6	5.7571	86.8367	880.0122	1.3306	2.5480
73	2017	6	7.8429	74.1837	972.6276	0.9694	0.6929
71	2017	6	5.8286	79.3673	937.3949	1.1490	1.7837
41	2017	6	5.7714	82.8673	879.4541	1.5133	1.6520
10	2017	6	5.8857	81.4184	975.0918	0.7959	0.9041
23	2017	6	1.5857	70.3469	776.8224	6.1010	2.6020
27	2017	6	8.0857	80.6735	863.0735	4.3459	2.2306
60	2017	6	4.8714	80.8673	948.4214	1.0469	1.8857
53	2017	6	5.5571	87.7449	858.2898	1.4510	2.4153
66	2017	6	6.6857	84.9184	903.5449	0.7786	2.4357
59	2017	6	5.2714	85.6837	892.5878	1.4888	2.2418
61	2017	6	7.8429	74.1837	972.6276	0.9694	0.6929
84	2017	6	7.8429	74.1837	972.6276	0.9694	0.6929
38	2017	6	5.2714	85.6837	892.5878	1.4888	2.2418
87	2017	6	7.9714	82.8673	905.6622	1.0510	2.3806
34	2017	6	5.2714	85.6837	892.5878	1.4888	2.2418
29	2017	6	6.6857	77.6531	948.9816	1.2327	1.5776
5	2017	6	5.8571	82.3061	835.1296	5.0061	2.0694
8	2017	6	5.5571	87.7449	858.2898	1.4510	2.4153
12	2017	6	5.8571	82.3061	835.1296	5.0061	2.0694
13	2017	6	12.8143	77.4184	951.7531	1.4490	0.9673
18	2017	6	5.9857	78.0612	975.4571	1.0755	1.0520
33	2017	6	6.1000	80.2347	908.3520	0.9347	1.5837
56	2017	6	9.8286	75.8163	987.4347	0.9255	1.2214
77	2017	6	6.2429	82.1735	926.3388	1.3561	1.6776
54	2017	6	5.8571	82.3061	835.1296	5.0061	2.0694
21	2017	6	6.1000	80.2347	908.3520	0.9347	1.5837
68	2017	6	6.3286	74.7959	981.5276	1.2031	1.8429
74	2017	6	7.8429	74.1837	972.6276	0.9694	0.6929
88	2017	6	5.7571	86.8367	880.0122	1.3306	2.5480
16	2017	6	5.6143	82.6020	929.1071	1.2378	1.5143
30	2017	6	6.6857	84.9184	903.5449	0.7786	2.4357
6	2017	6	6.3286	74.7959	981.5276	1.2031	1.8429
49	2017	6	6.6857	77.6531	948.9816	1.2327	1.5776
22	2017	6	5.7571	86.8367	880.0122	1.3306	2.5480
45	2017	6	5.5571	67.7347	819.7673	6.1837	2.3204
58	2017	6	6.6857	77.6531	948.9816	1.2327	1.5776
37	2017	6	6.3286	74.7959	981.5276	1.2031	1.8429
17	2017	6	5.1857	88.2143	908.8388	0.9969	2.5449
55	2017	6	7.3714	84.7857	882.7133	1.3439	1.9500
46	2017	6	5.6143	82.6020	929.1071	1.2378	1.5143
86	2017	6	6.2000	86.3980	870.7000	0.5286	1.8102
2	2017	6	6.2000	86.3980	870.7000	0.5286	1.8102
4	2017	6	6.1000	80.2347	908.3520	0.9347	1.5837
47	2017	6	12.3571	77.7857	967.1847	0.9041	0.6531
82	2017	6	5.7571	86.8367	880.0122	1.3306	2.5480

19	2017	6	11.0857	74.4898	969.5429	0.7908	1.2755
20	2017	6	5.5571	87.7449	858.2898	1.4510	2.4153
80	2017	6	5.7571	86.8367	880.0122	1.3306	2.5480
3	2017	6	12.8143	77.4184	951.7531	1.4490	0.9673
52	2017	6	5.1857	88.2143	908.8388	0.9969	2.5449
70	2017	6	6.2000	83.1837	917.1908	1.0061	1.4153
64	2017	6	1.5857	70.3469	776.8224	6.1010	2.6020
48	2017	6	6.2429	82.1735	926.3388	1.3561	1.6776
65	2017	6	5.1857	88.2143	908.8388	0.9969	2.5449
44	2017	6	6.2000	83.1837	917.1908	1.0061	1.4153
75	2017	6	1.5857	70.3469	776.8224	6.1010	2.6020
40	2017	6	5.5714	83.2755	954.9000	1.3673	1.7990
11	2017	6	7.3714	84.7857	882.7133	1.3439	1.9500
35	2017	6	4.8714	80.8673	948.4214	1.0469	1.8857
78	2017	6	7.9714	82.8673	905.6622	1.0510	2.3806
28	2017	6	5.8286	79.3673	937.3949	1.1490	1.7837
39	2017	6	5.1857	88.2143	908.8388	0.9969	2.5449
24	2017	6	6.6857	77.6531	948.9816	1.2327	1.5776
63	2017	6	5.5714	83.2755	954.9000	1.3673	1.7990
62	2017	6	5.7714	82.8673	879.4541	1.5133	1.6520
1	2017	6	5.7571	86.8367	880.0122	1.3306	2.5480
31	2017	7	9.1429	81.1633	851.9439	1.6776	1.0622
79	2017	7	12.8286	72.2959	982.6306	2.7173	1.1980
51	2017	7	12.2143	75.3673	949.8306	2.5908	1.5367
14	2017	7	12.9714	74.3980	905.3612	1.6857	2.4306
67	2017	7	13.0857	77.6735	910.5327	2.1439	2.3143
42	2017	7	12.5286	74.8776	881.8286	2.0184	2.2633
50	2017	7	11.0286	73.2449	910.1939	1.9969	1.2510
43	2017	7	12.5286	74.8776	881.8286	2.0184	2.2633
85	2017	7	11.5714	78.9592	919.1602	1.2612	1.3133
25	2017	7	15.4429	71.9490	989.1224	2.3122	1.0541
69	2017	7	13.1286	73.8061	950.5969	2.1745	1.1867
57	2017	7	11.6000	76.5000	894.4163	1.9949	2.2500
9	2017	7	11.2571	77.0918	860.2031	1.8592	2.1673
72	2017	7	13.2714	72.2245	884.6388	1.8061	1.9194
26	2017	7	12.8286	77.1837	873.3092	3.2204	1.9378
7	2017	7	12.0286	74.8980	865.0929	3.1939	2.0031
83	2017	7	16.9286	66.1327	953.9653	2.0449	1.0582
76	2017	7	11.0571	78.4490	930.7449	2.1755	1.3408
36	2017	7	13.2143	73.0204	938.9908	2.6480	1.6898
81	2017	7	12.2143	75.3673	949.8306	2.5908	1.5367
15	2017	7	9.7429	79.0816	928.0235	2.2265	1.3827
32	2017	7	12.5286	74.8776	881.8286	2.0184	2.2633
73	2017	7	12.9857	72.8265	974.0643	2.3776	0.6347
71	2017	7	13.2143	73.0204	938.9908	2.6480	1.6898
41	2017	7	11.0000	74.4082	881.3765	1.2633	1.4643
10	2017	7	12.8143	77.1327	976.4602	2.1745	0.8173
23	2017	7	7.1714	76.8673	778.4051	4.4949	2.2898
27	2017	7	12.0286	74.8980	865.0929	3.1939	2.0031
60	2017	7	12.2143	75.3673	949.8306	2.5908	1.5367

53	2017	7	11.2571	77.0918	860.2031	1.8592	2.1673
66	2017	7	12.9714	74.3980	905.3612	1.6857	2.4306
59	2017	7	11.6000	76.5000	894.4163	1.9949	2.2500
61	2017	7	12.9857	72.8265	974.0643	2.3776	0.6347
84	2017	7	12.9857	72.8265	974.0643	2.3776	0.6347
38	2017	7	11.6000	76.5000	894.4163	1.9949	2.2500
87	2017	7	14.4857	70.3061	907.5551	2.0224	2.1653
34	2017	7	11.6000	76.5000	894.4163	1.9949	2.2500
29	2017	7	13.1286	73.8061	950.5969	2.1745	1.1867
5	2017	7	10.2286	81.7959	837.0673	3.6214	1.6908
8	2017	7	11.2571	77.0918	860.2031	1.8592	2.1673
12	2017	7	10.2286	81.7959	837.0673	3.6214	1.6908
13	2017	7	16.9286	66.1327	953.9653	2.0449	1.0582
18	2017	7	11.6429	77.9796	976.5786	2.5092	0.8816
33	2017	7	11.0286	73.2449	910.1939	1.9969	1.2510
56	2017	7	15.4429	71.9490	989.1224	2.3122	1.0541
77	2017	7	9.7429	79.0816	928.0235	2.2265	1.3827
54	2017	7	10.2286	81.7959	837.0673	3.6214	1.6908
21	2017	7	11.0286	73.2449	910.1939	1.9969	1.2510
68	2017	7	12.8286	72.2959	982.6306	2.7173	1.1980
74	2017	7	12.9857	72.8265	974.0643	2.3776	0.6347
88	2017	7	12.5286	74.8776	881.8286	2.0184	2.2633
16	2017	7	11.0571	78.4490	930.7449	2.1755	1.3408
30	2017	7	12.9714	74.3980	905.3612	1.6857	2.4306
6	2017	7	12.8286	72.2959	982.6306	2.7173	1.1980
49	2017	7	13.1286	73.8061	950.5969	2.1745	1.1867
22	2017	7	12.5286	74.8776	881.8286	2.0184	2.2633
45	2017	7	10.3286	75.6633	821.6163	4.0612	1.7755
58	2017	7	13.1286	73.8061	950.5969	2.1745	1.1867
37	2017	7	12.8286	72.2959	982.6306	2.7173	1.1980
17	2017	7	13.0857	77.6735	910.5327	2.1439	2.3143
55	2017	7	13.2714	72.2245	884.6388	1.8061	1.9194
46	2017	7	11.0571	78.4490	930.7449	2.1755	1.3408
86	2017	7	11.8857	76.7041	872.6418	1.2194	1.7041
2	2017	7	11.8857	76.7041	872.6418	1.2194	1.7041
4	2017	7	11.0286	73.2449	910.1939	1.9969	1.2510
47	2017	7	17.2286	66.7857	969.2327	1.3653	0.6735
82	2017	7	12.5286	74.8776	881.8286	2.0184	2.2633
19	2017	7	15.8571	67.5204	971.4439	1.8755	1.1827
20	2017	7	11.2571	77.0918	860.2031	1.8592	2.1673
80	2017	7	12.5286	74.8776	881.8286	2.0184	2.2633
3	2017	7	16.9286	66.1327	953.9653	2.0449	1.0582
52	2017	7	13.0857	77.6735	910.5327	2.1439	2.3143
70	2017	7	11.5714	78.9592	919.1602	1.2612	1.3133
64	2017	7	7.1714	76.8673	778.4051	4.4949	2.2898
48	2017	7	9.7429	79.0816	928.0235	2.2265	1.3827
65	2017	7	13.0857	77.6735	910.5327	2.1439	2.3143
44	2017	7	11.5714	78.9592	919.1602	1.2612	1.3133
75	2017	7	7.1714	76.8673	778.4051	4.4949	2.2898
40	2017	7	13.8000	78.2143	956.4265	2.5102	1.4286

11	2017	7	13.2714	72.2245	884.6388	1.8061	1.9194
35	2017	7	12.2143	75.3673	949.8306	2.5908	1.5367
78	2017	7	14.4857	70.3061	907.5551	2.0224	2.1653
28	2017	7	13.2143	73.0204	938.9908	2.6480	1.6898
39	2017	7	13.0857	77.6735	910.5327	2.1439	2.3143
24	2017	7	13.1286	73.8061	950.5969	2.1745	1.1867
63	2017	7	13.8000	78.2143	956.4265	2.5102	1.4286
62	2017	7	11.0000	74.4082	881.3765	1.2633	1.4643
1	2017	7	12.5286	74.8776	881.8286	2.0184	2.2633
31	2017	8	4.9571	78.3469	851.5653	2.0969	1.1071
79	2017	8	7.3714	69.7755	981.2010	2.5265	1.1745
51	2017	8	6.2286	71.9082	948.6735	2.6765	1.5265
14	2017	8	7.7571	70.4286	904.9306	1.4571	2.9541
67	2017	8	7.1857	72.6122	909.8173	2.0347	3.0429
42	2017	8	6.7286	71.2857	881.3459	2.3745	2.5755
50	2017	8	7.1143	72.9388	909.3714	1.8592	1.3408
43	2017	8	6.7286	71.2857	881.3459	2.3745	2.5755
85	2017	8	8.2143	74.6633	918.1510	1.8714	1.4939
25	2017	8	11.2143	70.4592	987.7153	2.5663	1.0102
69	2017	8	8.3714	70.4490	949.3480	1.8449	1.5020
57	2017	8	6.9571	73.0306	893.6663	2.5959	2.8765
9	2017	8	6.6857	73.7653	859.9449	3.0980	2.4510
72	2017	8	8.5571	67.6122	884.3000	2.7612	2.1776
26	2017	8	10.2571	72.7857	873.2541	3.4786	2.1143
7	2017	8	8.3571	74.4796	865.0276	3.2041	1.9622
83	2017	8	13.6571	62.3163	953.3418	3.6735	1.2592
76	2017	8	6.3571	77.5918	929.7806	1.9724	1.4020
36	2017	8	8.0857	67.7755	937.9122	3.2510	1.8265
81	2017	8	6.2286	71.9082	948.6735	2.6765	1.5265
15	2017	8	6.1143	82.0816	927.1908	1.6796	1.5316
32	2017	8	6.7286	71.2857	881.3459	2.3745	2.5755
73	2017	8	8.2429	71.8469	972.7847	2.2653	0.6551
71	2017	8	8.0857	67.7755	937.9122	3.2510	1.8265
41	2017	8	7.3000	70.8061	880.7694	2.0592	1.5296
10	2017	8	7.3571	73.6122	975.0500	2.4878	1.0133
23	2017	8	2.5286	79.0612	778.5551	3.8245	2.3122
27	2017	8	8.3571	74.4796	865.0276	3.2041	1.9622
60	2017	8	6.2286	71.9082	948.6735	2.6765	1.5265
53	2017	8	6.6857	73.7653	859.9449	3.0980	2.4510
66	2017	8	7.7571	70.4286	904.9306	1.4571	2.9541
59	2017	8	6.9571	73.0306	893.6663	2.5959	2.8765
61	2017	8	8.2429	71.8469	972.7847	2.2653	0.6551
84	2017	8	8.2429	71.8469	972.7847	2.2653	0.6551
38	2017	8	6.9571	73.0306	893.6663	2.5959	2.8765
87	2017	8	8.9571	65.9388	906.9082	2.7735	2.1265
34	2017	8	6.9571	73.0306	893.6663	2.5959	2.8765
29	2017	8	8.3714	70.4490	949.3480	1.8449	1.5020
5	2017	8	6.9143	79.2653	837.0724	3.4796	2.1194
8	2017	8	6.6857	73.7653	859.9449	3.0980	2.4510
12	2017	8	6.9143	79.2653	837.0724	3.4796	2.1194

13	2017	8	13.6571	62.3163	953.3418	3.6735	1.2592
18	2017	8	6.8143	77.3980	975.3235	2.2500	0.8582
33	2017	8	7.1143	72.9388	909.3714	1.8592	1.3408
56	2017	8	11.2143	70.4592	987.7153	2.5663	1.0102
77	2017	8	6.1143	82.0816	927.1908	1.6796	1.5316
54	2017	8	6.9143	79.2653	837.0724	3.4796	2.1194
21	2017	8	7.1143	72.9388	909.3714	1.8592	1.3408
68	2017	8	7.3714	69.7755	981.2010	2.5265	1.1745
74	2017	8	8.2429	71.8469	972.7847	2.2653	0.6551
88	2017	8	6.7286	71.2857	881.3459	2.3745	2.5755
16	2017	8	6.3571	77.5918	929.7806	1.9724	1.4020
30	2017	8	7.7571	70.4286	904.9306	1.4571	2.9541
6	2017	8	7.3714	69.7755	981.2010	2.5265	1.1745
49	2017	8	8.3714	70.4490	949.3480	1.8449	1.5020
22	2017	8	6.7286	71.2857	881.3459	2.3745	2.5755
45	2017	8	7.4143	75.8265	821.7765	3.8153	2.0531
58	2017	8	8.3714	70.4490	949.3480	1.8449	1.5020
37	2017	8	7.3714	69.7755	981.2010	2.5265	1.1745
17	2017	8	7.1857	72.6122	909.8173	2.0347	3.0429
55	2017	8	8.5571	67.6122	884.3000	2.7612	2.1776
46	2017	8	6.3571	77.5918	929.7806	1.9724	1.4020
86	2017	8	7.4714	72.7857	872.2082	2.5214	2.1173
2	2017	8	7.4714	72.7857	872.2082	2.5214	2.1173
4	2017	8	7.1143	72.9388	909.3714	1.8592	1.3408
47	2017	8	13.3857	62.8980	968.2071	2.0082	0.7469
82	2017	8	6.7286	71.2857	881.3459	2.3745	2.5755
19	2017	8	12.1714	65.7347	970.5133	1.9245	1.1622
20	2017	8	6.6857	73.7653	859.9449	3.0980	2.4510
80	2017	8	6.7286	71.2857	881.3459	2.3745	2.5755
3	2017	8	13.6571	62.3163	953.3418	3.6735	1.2592
52	2017	8	7.1857	72.6122	909.8173	2.0347	3.0429
70	2017	8	8.2143	74.6633	918.1510	1.8714	1.4939
64	2017	8	2.5286	79.0612	778.5551	3.8245	2.3122
48	2017	8	6.1143	82.0816	927.1908	1.6796	1.5316
65	2017	8	7.1857	72.6122	909.8173	2.0347	3.0429
44	2017	8	8.2143	74.6633	918.1510	1.8714	1.4939
75	2017	8	2.5286	79.0612	778.5551	3.8245	2.3122
40	2017	8	7.2143	73.5000	955.3163	2.6704	1.6684
11	2017	8	8.5571	67.6122	884.3000	2.7612	2.1776
35	2017	8	6.2286	71.9082	948.6735	2.6765	1.5265
78	2017	8	8.9571	65.9388	906.9082	2.7735	2.1265
28	2017	8	8.0857	67.7755	937.9122	3.2510	1.8265
39	2017	8	7.1857	72.6122	909.8173	2.0347	3.0429
24	2017	8	8.3714	70.4490	949.3480	1.8449	1.5020
63	2017	8	7.2143	73.5000	955.3163	2.6704	1.6684
62	2017	8	7.3000	70.8061	880.7694	2.0592	1.5296
1	2017	8	6.7286	71.2857	881.3459	2.3745	2.5755
31	2017	9	6.4143	78.7755	850.5398	1.8561	1.1480
79	2017	9	10.3714	73.2551	979.8847	1.9694	1.2898
51	2017	9	9.5571	74.8061	947.4000	2.0408	1.5684

14	2017	9	9.7000	75.7653	903.5061	0.6816	2.8316
67	2017	9	9.4714	78.3878	908.5612	0.9939	2.9918
42	2017	9	8.4429	78.7551	880.2398	1.5878	2.6755
50	2017	9	8.6571	78.0816	908.3602	1.3000	1.4122
43	2017	9	8.4429	78.7551	880.2398	1.5878	2.6755
85	2017	9	9.7714	76.9184	917.1490	1.7510	1.4010
25	2017	9	13.0571	74.8980	986.2827	1.9010	0.9980
69	2017	9	10.4000	73.2041	948.2520	1.2990	1.6827
57	2017	9	7.9714	80.2245	892.6367	1.5673	2.6214
9	2017	9	7.6000	78.6122	858.7214	2.3541	2.4694
72	2017	9	9.8714	73.4592	883.0633	1.8857	2.1969
26	2017	9	10.8143	77.1633	871.9735	3.2980	2.0245
7	2017	9	8.7571	79.4082	863.7949	2.6816	1.8510
83	2017	9	14.6429	66.9082	952.0714	2.8571	1.2388
76	2017	9	9.3000	80.1020	928.7194	1.4520	1.4878
36	2017	9	10.4857	70.8367	936.7031	2.3857	1.8082
81	2017	9	9.5571	74.8061	947.4000	2.0408	1.5684
15	2017	9	8.6429	83.6531	926.1531	1.3092	1.6449
32	2017	9	8.4429	78.7551	880.2398	1.5878	2.6755
73	2017	9	11.2286	74.6531	971.7071	1.7898	0.8041
71	2017	9	10.4857	70.8367	936.7031	2.3857	1.8082
41	2017	9	8.1000	75.3980	879.7418	1.6347	1.5122
10	2017	9	10.4571	76.4796	973.6735	2.1449	0.9429
23	2017	9	3.7714	79.6020	777.4133	3.1816	2.2020
27	2017	9	8.7571	79.4082	863.7949	2.6816	1.8510
60	2017	9	9.5571	74.8061	947.4000	2.0408	1.5684
53	2017	9	7.6000	78.6122	858.7214	2.3541	2.4694
66	2017	9	9.7000	75.7653	903.5061	0.6816	2.8316
59	2017	9	7.9714	80.2245	892.6367	1.5673	2.6214
61	2017	9	11.2286	74.6531	971.7071	1.7898	0.8041
84	2017	9	11.2286	74.6531	971.7071	1.7898	0.8041
38	2017	9	7.9714	80.2245	892.6367	1.5673	2.6214
87	2017	9	10.5143	73.7551	905.7296	1.6684	2.2786
34	2017	9	7.9714	80.2245	892.6367	1.5673	2.6214
29	2017	9	10.4000	73.2041	948.2520	1.2990	1.6827
5	2017	9	6.7429	82.6939	835.8561	2.9408	2.1592
8	2017	9	7.6000	78.6122	858.7214	2.3541	2.4694
12	2017	9	6.7429	82.6939	835.8561	2.9408	2.1592
13	2017	9	14.6429	66.9082	952.0714	2.8571	1.2388
18	2017	9	9.6714	79.3265	974.0143	1.7031	0.8704
33	2017	9	8.6571	78.0816	908.3602	1.3000	1.4122
56	2017	9	13.0571	74.8980	986.2827	1.9010	0.9980
77	2017	9	8.6429	83.6531	926.1531	1.3092	1.6449
54	2017	9	6.7429	82.6939	835.8561	2.9408	2.1592
21	2017	9	8.6571	78.0816	908.3602	1.3000	1.4122
68	2017	9	10.3714	73.2551	979.8847	1.9694	1.2898
74	2017	9	11.2286	74.6531	971.7071	1.7898	0.8041
88	2017	9	8.4429	78.7551	880.2398	1.5878	2.6755
16	2017	9	9.3000	80.1020	928.7194	1.4520	1.4878
30	2017	9	9.7000	75.7653	903.5061	0.6816	2.8316

6	2017	9	10.3714	73.2551	979.8847	1.9694	1.2898
49	2017	9	10.4000	73.2041	948.2520	1.2990	1.6827
22	2017	9	8.4429	78.7551	880.2398	1.5878	2.6755
45	2017	9	8.1857	76.5408	820.5286	3.4500	2.0347
58	2017	9	10.4000	73.2041	948.2520	1.2990	1.6827
37	2017	9	10.3714	73.2551	979.8847	1.9694	1.2898
17	2017	9	9.4714	78.3878	908.5612	0.9939	2.9918
55	2017	9	9.8714	73.4592	883.0633	1.8857	2.1969
46	2017	9	9.3000	80.1020	928.7194	1.4520	1.4878
86	2017	9	7.9429	78.0306	871.1735	1.7092	1.8663
2	2017	9	7.9429	78.0306	871.1735	1.7092	1.8663
4	2017	9	8.6571	78.0816	908.3602	1.3000	1.4122
47	2017	9	14.4143	67.8061	966.9173	1.4092	0.7184
82	2017	9	8.4429	78.7551	880.2398	1.5878	2.6755
19	2017	9	13.6571	69.7041	969.0102	1.0847	1.2276
20	2017	9	7.6000	78.6122	858.7214	2.3541	2.4694
80	2017	9	8.4429	78.7551	880.2398	1.5878	2.6755
3	2017	9	14.6429	66.9082	952.0714	2.8571	1.2388
52	2017	9	9.4714	78.3878	908.5612	0.9939	2.9918
70	2017	9	9.7714	76.9184	917.1490	1.7510	1.4010
64	2017	9	3.7714	79.6020	777.4133	3.1816	2.2020
48	2017	9	8.6429	83.6531	926.1531	1.3092	1.6449
65	2017	9	9.4714	78.3878	908.5612	0.9939	2.9918
44	2017	9	9.7714	76.9184	917.1490	1.7510	1.4010
75	2017	9	3.7714	79.6020	777.4133	3.1816	2.2020
40	2017	9	9.8714	78.6837	953.9071	2.1980	1.6490
11	2017	9	9.8714	73.4592	883.0633	1.8857	2.1969
35	2017	9	9.5571	74.8061	947.4000	2.0408	1.5684
78	2017	9	10.5143	73.7551	905.7296	1.6684	2.2786
28	2017	9	10.4857	70.8367	936.7031	2.3857	1.8082
39	2017	9	9.4714	78.3878	908.5612	0.9939	2.9918
24	2017	9	10.4000	73.2041	948.2520	1.2990	1.6827
63	2017	9	9.8714	78.6837	953.9071	2.1980	1.6490
62	2017	9	8.1000	75.3980	879.7418	1.6347	1.5122
1	2017	9	8.4429	78.7551	880.2398	1.5878	2.6755
31	2017	10	6.3571	82.6429	850.2796	0.4276	0.9602
79	2017	10	10.9286	76.0102	979.1755	1.8245	1.0031
51	2017	10	9.4286	75.7143	946.7898	1.6929	1.3459
14	2017	10	8.6857	79.7857	902.7061	0.4020	1.9551
67	2017	10	8.3571	82.3673	908.0439	0.5327	2.4367
42	2017	10	7.8714	82.8265	879.7755	0.5704	2.3633
50	2017	10	8.5429	78.1122	908.2541	0.7704	1.3469
43	2017	10	7.8714	82.8265	879.7755	0.5704	2.3633
85	2017	10	8.9714	80.1633	917.1378	0.9776	1.2010
25	2017	10	12.3000	79.8673	985.7622	1.3969	0.7316
69	2017	10	10.4571	75.7551	948.0112	0.9633	1.2531
57	2017	10	7.6571	84.6122	892.4255	0.2204	1.7510
9	2017	10	7.8429	82.2347	858.1622	0.4418	2.1224
72	2017	10	9.9571	76.8571	882.4286	0.3010	1.8990
26	2017	10	11.4429	82.5408	871.2000	2.1082	1.5867

7	2017	10	10.5714	83.8673	863.0867	1.0837	1.5673
83	2017	10	14.8714	69.9796	951.4684	1.0255	0.9806
76	2017	10	8.8429	78.0102	928.4520	1.0061	1.4296
36	2017	10	9.6857	73.5408	936.2541	1.3673	1.6582
81	2017	10	9.4286	75.7143	946.7898	1.6929	1.3459
15	2017	10	8.2571	82.2959	925.9367	1.2071	1.4439
32	2017	10	7.8714	82.8265	879.7755	0.5704	2.3633
73	2017	10	11.3714	73.3571	971.2898	1.4122	0.7408
71	2017	10	9.6857	73.5408	936.2541	1.3673	1.6582
41	2017	10	7.7571	80.2041	879.5704	0.1878	1.2398
10	2017	10	10.8286	77.5816	972.9561	1.8214	0.6276
23	2017	10	6.6714	85.9592	776.6408	2.0500	1.9235
27	2017	10	10.5714	83.8673	863.0867	1.0837	1.5673
60	2017	10	9.4286	75.7143	946.7898	1.6929	1.3459
53	2017	10	7.8429	82.2347	858.1622	0.4418	2.1224
66	2017	10	8.6857	79.7857	902.7061	0.4020	1.9551
59	2017	10	7.6571	84.6122	892.4255	0.2204	1.7510
61	2017	10	11.3714	73.3571	971.2898	1.4122	0.7408
84	2017	10	11.3714	73.3571	971.2898	1.4122	0.7408
38	2017	10	7.6571	84.6122	892.4255	0.2204	1.7510
87	2017	10	10.5714	76.9796	905.2265	0.6204	2.0378
34	2017	10	7.6571	84.6122	892.4255	0.2204	1.7510
29	2017	10	10.4571	75.7551	948.0112	0.9633	1.2531
5	2017	10	8.6000	89.8061	835.2020	1.4286	1.6908
8	2017	10	7.8429	82.2347	858.1622	0.4418	2.1224
12	2017	10	8.6000	89.8061	835.2020	1.4286	1.6908
13	2017	10	14.8714	69.9796	951.4684	1.0255	0.9806
18	2017	10	10.4000	80.0918	973.2980	1.5469	0.6908
33	2017	10	8.5429	78.1122	908.2541	0.7704	1.3469
56	2017	10	12.3000	79.8673	985.7622	1.3969	0.7316
77	2017	10	8.2571	82.2959	925.9367	1.2071	1.4439
54	2017	10	8.6000	89.8061	835.2020	1.4286	1.6908
21	2017	10	8.5429	78.1122	908.2541	0.7704	1.3469
68	2017	10	10.9286	76.0102	979.1755	1.8245	1.0031
74	2017	10	11.3714	73.3571	971.2898	1.4122	0.7408
88	2017	10	7.8714	82.8265	879.7755	0.5704	2.3633
16	2017	10	8.8429	78.0102	928.4520	1.0061	1.4296
30	2017	10	8.6857	79.7857	902.7061	0.4020	1.9551
6	2017	10	10.9286	76.0102	979.1755	1.8245	1.0031
49	2017	10	10.4571	75.7551	948.0112	0.9633	1.2531
22	2017	10	7.8714	82.8265	879.7755	0.5704	2.3633
45	2017	10	10.5714	81.6939	819.6796	2.6255	1.6929
58	2017	10	10.4571	75.7551	948.0112	0.9633	1.2531
37	2017	10	10.9286	76.0102	979.1755	1.8245	1.0031
17	2017	10	8.3571	82.3673	908.0439	0.5327	2.4367
55	2017	10	9.9571	76.8571	882.4286	0.3010	1.8990
46	2017	10	8.8429	78.0102	928.4520	1.0061	1.4296
86	2017	10	8.0571	84.8878	870.9143	0.1020	1.0296
2	2017	10	8.0571	84.8878	870.9143	0.1020	1.0296
4	2017	10	8.5429	78.1122	908.2541	0.7704	1.3469

47	2017	10	14.4286	69.9082	966.4816	0.5102	0.6286
82	2017	10	7.8714	82.8265	879.7755	0.5704	2.3633
19	2017	10	12.7714	72.3265	968.2694	0.5214	1.0765
20	2017	10	7.8429	82.2347	858.1622	0.4418	2.1224
80	2017	10	7.8714	82.8265	879.7755	0.5704	2.3633
3	2017	10	14.8714	69.9796	951.4684	1.0255	0.9806
52	2017	10	8.3571	82.3673	908.0439	0.5327	2.4367
70	2017	10	8.9714	80.1633	917.1378	0.9776	1.2010
64	2017	10	6.6714	85.9592	776.6408	2.0500	1.9235
48	2017	10	8.2571	82.2959	925.9367	1.2071	1.4439
65	2017	10	8.3571	82.3673	908.0439	0.5327	2.4367
44	2017	10	8.9714	80.1633	917.1378	0.9776	1.2010
75	2017	10	6.6714	85.9592	776.6408	2.0500	1.9235
40	2017	10	9.7286	81.9796	953.1827	1.6510	1.1990
11	2017	10	9.9571	76.8571	882.4286	0.3010	1.8990
35	2017	10	9.4286	75.7143	946.7898	1.6929	1.3459
78	2017	10	10.5714	76.9796	905.2265	0.6204	2.0378
28	2017	10	9.6857	73.5408	936.2541	1.3673	1.6582
39	2017	10	8.3571	82.3673	908.0439	0.5327	2.4367
24	2017	10	10.4571	75.7551	948.0112	0.9633	1.2531
63	2017	10	9.7286	81.9796	953.1827	1.6510	1.1990
62	2017	10	7.7571	80.2041	879.5704	0.1878	1.2398
1	2017	10	7.8714	82.8265	879.7755	0.5704	2.3633
31	2017	11	6.8000	87.7245	847.5735	0.3265	0.9051
79	2017	11	8.1143	82.0612	976.6908	0.5449	1.1684
51	2017	11	6.5571	84.4796	944.2347	0.5214	1.5020
14	2017	11	8.3714	88.9184	899.9204	0.5724	2.0867
67	2017	11	6.9000	92.3878	905.3031	0.2245	2.5969
42	2017	11	7.3571	90.4592	876.9459	0.2724	2.4622
50	2017	11	7.9000	82.0102	905.3429	0.1418	1.4245
43	2017	11	7.3571	90.4592	876.9459	0.2724	2.4622
85	2017	11	8.4286	85.2449	914.1255	0.3112	1.2510
25	2017	11	10.7143	85.0306	982.8592	0.6439	0.8092
69	2017	11	8.1286	82.9388	945.1898	0.1878	1.0939
57	2017	11	6.2000	90.7449	889.5449	0.0490	1.7867
9	2017	11	9.0286	88.4796	855.3929	0.7663	2.4224
72	2017	11	10.7286	83.5510	879.4663	0.9194	1.9347
26	2017	11	14.4429	80.7449	868.1398	3.7551	2.0602
7	2017	11	14.0143	81.3061	860.0990	3.2439	2.0969
83	2017	11	16.1286	72.7959	947.7010	1.8969	1.0939
76	2017	11	7.3714	83.0000	925.6082	0.1990	1.5500
36	2017	11	7.0143	82.6122	933.5296	0.3643	1.8143
81	2017	11	6.5571	84.4796	944.2347	0.5214	1.5020
15	2017	11	8.1286	86.1633	923.0255	0.2898	1.5235
32	2017	11	7.3571	90.4592	876.9459	0.2724	2.4622
73	2017	11	9.6000	76.7551	968.4082	0.3357	0.6255
71	2017	11	7.0143	82.6122	933.5296	0.3643	1.8143
41	2017	11	6.9000	86.5408	876.7102	0.2500	1.3296
10	2017	11	7.7286	85.5408	970.3857	0.5102	0.7143
23	2017	11	10.1429	76.9388	774.4020	4.6592	2.2908

27	2017	11	14.0143	81.3061	860.0990	3.2439	2.0969
60	2017	11	6.5571	84.4796	944.2347	0.5214	1.5020
53	2017	11	9.0286	88.4796	855.3929	0.7663	2.4224
66	2017	11	8.3714	88.9184	899.9204	0.5724	2.0867
59	2017	11	6.2000	90.7449	889.5449	0.0490	1.7867
61	2017	11	9.6000	76.7551	968.4082	0.3357	0.6255
84	2017	11	9.6000	76.7551	968.4082	0.3357	0.6255
38	2017	11	6.2000	90.7449	889.5449	0.0490	1.7867
87	2017	11	10.5429	83.2653	902.1510	1.1388	2.0449
34	2017	11	6.2000	90.7449	889.5449	0.0490	1.7867
29	2017	11	8.1286	82.9388	945.1898	0.1878	1.0939
5	2017	11	11.7571	84.9388	832.4980	3.9347	2.1429
8	2017	11	9.0286	88.4796	855.3929	0.7663	2.4224
12	2017	11	11.7571	84.9388	832.4980	3.9347	2.1429
13	2017	11	16.1286	72.7959	947.7010	1.8969	1.0939
18	2017	11	8.3286	84.7857	970.8214	0.4531	0.7735
33	2017	11	7.9000	82.0102	905.3429	0.1418	1.4245
56	2017	11	10.7143	85.0306	982.8592	0.6439	0.8092
77	2017	11	8.1286	86.1633	923.0255	0.2898	1.5235
54	2017	11	11.7571	84.9388	832.4980	3.9347	2.1429
21	2017	11	7.9000	82.0102	905.3429	0.1418	1.4245
68	2017	11	8.1143	82.0612	976.6908	0.5449	1.1684
74	2017	11	9.6000	76.7551	968.4082	0.3357	0.6255
88	2017	11	7.3571	90.4592	876.9459	0.2724	2.4622
16	2017	11	7.3714	83.0000	925.6082	0.1990	1.5500
30	2017	11	8.3714	88.9184	899.9204	0.5724	2.0867
6	2017	11	8.1143	82.0612	976.6908	0.5449	1.1684
49	2017	11	8.1286	82.9388	945.1898	0.1878	1.0939
22	2017	11	7.3571	90.4592	876.9459	0.2724	2.4622
45	2017	11	13.6000	71.5510	817.1622	5.4122	2.3857
58	2017	11	8.1286	82.9388	945.1898	0.1878	1.0939
37	2017	11	8.1143	82.0612	976.6908	0.5449	1.1684
17	2017	11	6.9000	92.3878	905.3031	0.2245	2.5969
55	2017	11	10.7286	83.5510	879.4663	0.9194	1.9347
46	2017	11	7.3714	83.0000	925.6082	0.1990	1.5500
86	2017	11	8.3000	92.3776	868.0541	0.0857	1.0112
2	2017	11	8.3000	92.3776	868.0541	0.0857	1.0112
4	2017	11	7.9000	82.0102	905.3429	0.1418	1.4245
47	2017	11	14.9286	74.7857	962.9633	1.3224	0.8010
82	2017	11	7.3571	90.4592	876.9459	0.2724	2.4622
19	2017	11	11.9714	79.0510	965.1990	0.4571	1.1459
20	2017	11	9.0286	88.4796	855.3929	0.7663	2.4224
80	2017	11	7.3571	90.4592	876.9459	0.2724	2.4622
3	2017	11	16.1286	72.7959	947.7010	1.8969	1.0939
52	2017	11	6.9000	92.3878	905.3031	0.2245	2.5969
70	2017	11	8.4286	85.2449	914.1255	0.3112	1.2510
64	2017	11	10.1429	76.9388	774.4020	4.6592	2.2908
48	2017	11	8.1286	86.1633	923.0255	0.2898	1.5235
65	2017	11	6.9000	92.3878	905.3031	0.2245	2.5969
44	2017	11	8.4286	85.2449	914.1255	0.3112	1.2510

75	2017	11	10.1429	76.9388	774.4020	4.6592	2.2908
40	2017	11	6.8857	89.8265	950.5296	0.3980	1.4031
11	2017	11	10.7286	83.5510	879.4663	0.9194	1.9347
35	2017	11	6.5571	84.4796	944.2347	0.5214	1.5020
78	2017	11	10.5429	83.2653	902.1510	1.1388	2.0449
28	2017	11	7.0143	82.6122	933.5296	0.3643	1.8143
39	2017	11	6.9000	92.3878	905.3031	0.2245	2.5969
24	2017	11	8.1286	82.9388	945.1898	0.1878	1.0939
63	2017	11	6.8857	89.8265	950.5296	0.3980	1.4031
62	2017	11	6.9000	86.5408	876.7102	0.2500	1.3296
1	2017	11	7.3571	90.4592	876.9459	0.2724	2.4622
31	2017	12	10.0143	86.3367	847.4694	1.8786	1.0500
79	2017	12	10.7429	85.7653	977.3082	0.1469	1.3969
51	2017	12	9.2286	90.4796	944.7735	0.1735	1.5694
14	2017	12	10.0857	92.6531	900.0857	0.6582	2.1612
67	2017	12	9.1429	96.3776	905.5510	0.2184	2.4888
42	2017	12	9.6857	93.4388	877.0531	0.7357	2.5796
50	2017	12	10.1000	84.8367	905.4561	0.3133	1.5837
43	2017	12	9.6857	93.4388	877.0531	0.7357	2.5796
85	2017	12	11.0000	86.3163	914.0816	0.7469	1.1704
25	2017	12	12.7714	86.4694	983.4020	0.4265	0.8714
69	2017	12	10.8000	87.0000	945.5469	0.1255	1.1408
57	2017	12	8.8857	94.5714	889.6296	0.4122	1.7806
9	2017	12	10.7857	89.1327	855.4020	1.5990	2.6255
72	2017	12	12.7286	86.7245	879.4500	1.7357	2.1561
26	2017	12	15.0000	82.0408	868.1245	4.3847	2.1337
7	2017	12	13.4571	80.1735	860.0827	4.4755	2.3551
83	2017	12	17.5000	76.0000	947.4061	2.3173	1.1714
76	2017	12	9.4286	87.7041	925.8459	0.1633	1.5898
36	2017	12	9.7571	88.7551	933.9153	0.1245	1.8112
81	2017	12	9.2286	90.4796	944.7735	0.1735	1.5694
15	2017	12	9.8143	86.1531	923.1327	0.3531	1.7520
32	2017	12	9.6857	93.4388	877.0531	0.7357	2.5796
73	2017	12	11.7000	81.0306	968.8265	0.3592	0.6653
71	2017	12	9.7571	88.7551	933.9153	0.1245	1.8112
41	2017	12	10.3857	88.1531	876.6806	0.7286	1.4265
10	2017	12	10.4286	90.9898	971.0204	0.0694	0.7633
23	2017	12	9.1143	66.1939	774.7827	6.7602	2.4143
27	2017	12	13.4571	80.1735	860.0827	4.4755	2.3551
60	2017	12	9.2286	90.4796	944.7735	0.1735	1.5694
53	2017	12	10.7857	89.1327	855.4020	1.5990	2.6255
66	2017	12	10.0857	92.6531	900.0857	0.6582	2.1612
59	2017	12	8.8857	94.5714	889.6296	0.4122	1.7806
61	2017	12	11.7000	81.0306	968.8265	0.3592	0.6653
84	2017	12	11.7000	81.0306	968.8265	0.3592	0.6653
38	2017	12	8.8857	94.5714	889.6296	0.4122	1.7806
87	2017	12	12.6714	86.7959	902.1418	1.6235	2.2357
34	2017	12	8.8857	94.5714	889.6296	0.4122	1.7806
29	2017	12	10.8000	87.0000	945.5469	0.1255	1.1408
5	2017	12	11.9857	80.0306	832.5959	5.4337	2.1878

8	2017	12	10.7857	89.1327	855.4020	1.5990	2.6255
12	2017	12	11.9857	80.0306	832.5959	5.4337	2.1878
13	2017	12	17.5000	76.0000	947.4061	2.3173	1.1714
18	2017	12	10.8143	86.6020	971.3816	0.1827	1.0214
33	2017	12	10.1000	84.8367	905.4561	0.3133	1.5837
56	2017	12	12.7714	86.4694	983.4020	0.4265	0.8714
77	2017	12	9.8143	86.1531	923.1327	0.3531	1.7520
54	2017	12	11.9857	80.0306	832.5959	5.4337	2.1878
21	2017	12	10.1000	84.8367	905.4561	0.3133	1.5837
68	2017	12	10.7429	85.7653	977.3082	0.1469	1.3969
74	2017	12	11.7000	81.0306	968.8265	0.3592	0.6653
88	2017	12	9.6857	93.4388	877.0531	0.7357	2.5796
16	2017	12	9.4286	87.7041	925.8459	0.1633	1.5898
30	2017	12	10.0857	92.6531	900.0857	0.6582	2.1612
6	2017	12	10.7429	85.7653	977.3082	0.1469	1.3969
49	2017	12	10.8000	87.0000	945.5469	0.1255	1.1408
22	2017	12	9.6857	93.4388	877.0531	0.7357	2.5796
45	2017	12	13.8000	62.7245	817.3857	7.2969	2.5500
58	2017	12	10.8000	87.0000	945.5469	0.1255	1.1408
37	2017	12	10.7429	85.7653	977.3082	0.1469	1.3969
17	2017	12	9.1429	96.3776	905.5510	0.2184	2.4888
55	2017	12	12.7286	86.7245	879.4500	1.7357	2.1561
46	2017	12	9.4286	87.7041	925.8459	0.1633	1.5898
86	2017	12	11.6571	91.9184	867.9592	1.2082	1.2031
2	2017	12	11.6571	91.9184	867.9592	1.2082	1.2031
4	2017	12	10.1000	84.8367	905.4561	0.3133	1.5837
47	2017	12	16.7286	77.7449	962.8041	1.7408	0.9204
82	2017	12	9.6857	93.4388	877.0531	0.7357	2.5796
19	2017	12	13.6714	82.2551	965.4316	0.5490	1.2561
20	2017	12	10.7857	89.1327	855.4020	1.5990	2.6255
80	2017	12	9.6857	93.4388	877.0531	0.7357	2.5796
3	2017	12	17.5000	76.0000	947.4061	2.3173	1.1714
52	2017	12	9.1429	96.3776	905.5510	0.2184	2.4888
70	2017	12	11.0000	86.3163	914.0816	0.7469	1.1704
64	2017	12	9.1143	66.1939	774.7827	6.7602	2.4143
48	2017	12	9.8143	86.1531	923.1327	0.3531	1.7520
65	2017	12	9.1429	96.3776	905.5510	0.2184	2.4888
44	2017	12	11.0000	86.3163	914.0816	0.7469	1.1704
75	2017	12	9.1143	66.1939	774.7827	6.7602	2.4143
40	2017	12	9.4429	94.7245	951.0745	0.1347	1.5643
11	2017	12	12.7286	86.7245	879.4500	1.7357	2.1561
35	2017	12	9.2286	90.4796	944.7735	0.1735	1.5694
78	2017	12	12.6714	86.7959	902.1418	1.6235	2.2357
28	2017	12	9.7571	88.7551	933.9153	0.1245	1.8112
39	2017	12	9.1429	96.3776	905.5510	0.2184	2.4888
24	2017	12	10.8000	87.0000	945.5469	0.1255	1.1408
63	2017	12	9.4429	94.7245	951.0745	0.1347	1.5643
62	2017	12	10.3857	88.1531	876.6806	0.7286	1.4265
1	2017	12	9.6857	93.4388	877.0531	0.7357	2.5796
31	2017	13	11.9714	79.6837	849.6704	3.1255	1.1714

79	2017	13	15.6143	82.3673	978.4418	1.3561	1.1806
51	2017	13	13.6143	86.5306	946.3133	1.1561	1.3592
14	2017	13	12.9857	87.9694	902.3500	1.3480	2.0888
67	2017	13	13.2143	90.1020	907.5878	1.2010	2.5480
42	2017	13	13.1571	85.6633	879.3592	2.1776	2.4888
50	2017	13	13.8571	81.0714	907.4265	1.2163	1.7143
43	2017	13	13.1571	85.6633	879.3592	2.1776	2.4888
85	2017	13	15.2429	80.5306	915.9102	2.1357	1.3235
25	2017	13	15.6714	84.2245	985.2112	1.1765	0.6908
69	2017	13	14.6429	82.0510	947.1388	1.2000	1.1459
57	2017	13	13.7286	87.2653	891.6602	1.4735	2.0255
9	2017	13	12.4000	81.6327	857.7816	2.5235	2.5520
72	2017	13	13.5429	81.8469	881.8776	2.1224	2.2949
26	2017	13	14.2429	81.2143	870.6735	3.7908	1.7724
7	2017	13	13.7571	79.2857	862.5827	3.6265	2.0959
83	2017	13	17.3571	75.4898	950.0163	2.5184	1.1112
76	2017	13	14.3429	83.0102	927.5500	1.5429	1.4816
36	2017	13	14.2000	83.8163	935.7061	1.2500	1.7092
81	2017	13	13.6143	86.5306	946.3133	1.1561	1.3592
15	2017	13	14.4143	81.3265	924.8857	1.5837	1.8418
32	2017	13	13.1571	85.6633	879.3592	2.1776	2.4888
73	2017	13	16.1571	78.5306	970.1755	1.7776	0.7337
71	2017	13	14.2000	83.8163	935.7061	1.2500	1.7092
41	2017	13	13.8571	79.1531	878.7612	1.3878	1.4551
10	2017	13	14.6286	85.9184	972.4378	0.8204	0.7837
23	2017	13	9.1286	71.2245	777.3888	4.9551	2.4500
27	2017	13	13.7571	79.2857	862.5827	3.6265	2.0959
60	2017	13	13.6143	86.5306	946.3133	1.1561	1.3592
53	2017	13	12.4000	81.6327	857.7816	2.5235	2.5520
66	2017	13	12.9857	87.9694	902.3500	1.3480	2.0888
59	2017	13	13.7286	87.2653	891.6602	1.4735	2.0255
61	2017	13	16.1571	78.5306	970.1755	1.7776	0.7337
84	2017	13	16.1571	78.5306	970.1755	1.7776	0.7337
38	2017	13	13.7286	87.2653	891.6602	1.4735	2.0255
87	2017	13	14.1429	82.0102	904.4643	2.1827	2.2449
34	2017	13	13.7286	87.2653	891.6602	1.4735	2.0255
29	2017	13	14.6429	82.0510	947.1388	1.2000	1.1459
5	2017	13	12.0286	79.2653	835.0939	4.5735	1.9633
8	2017	13	12.4000	81.6327	857.7816	2.5235	2.5520
12	2017	13	12.0286	79.2653	835.0939	4.5735	1.9633
13	2017	13	17.3571	75.4898	950.0163	2.5184	1.1112
18	2017	13	15.4286	82.2653	972.4959	1.2531	1.0173
33	2017	13	13.8571	81.0714	907.4265	1.2163	1.7143
56	2017	13	15.6714	84.2245	985.2112	1.1765	0.6908
77	2017	13	14.4143	81.3265	924.8857	1.5837	1.8418
54	2017	13	12.0286	79.2653	835.0939	4.5735	1.9633
21	2017	13	13.8571	81.0714	907.4265	1.2163	1.7143
68	2017	13	15.6143	82.3673	978.4418	1.3561	1.1806
74	2017	13	16.1571	78.5306	970.1755	1.7776	0.7337
88	2017	13	13.1571	85.6633	879.3592	2.1776	2.4888

16	2017	13	14.3429	83.0102	927.5500	1.5429	1.4816
30	2017	13	12.9857	87.9694	902.3500	1.3480	2.0888
6	2017	13	15.6143	82.3673	978.4418	1.3561	1.1806
49	2017	13	14.6429	82.0510	947.1388	1.2000	1.1459
22	2017	13	13.1571	85.6633	879.3592	2.1776	2.4888
45	2017	13	12.3857	64.8367	819.8388	5.8214	2.1592
58	2017	13	14.6429	82.0510	947.1388	1.2000	1.1459
37	2017	13	15.6143	82.3673	978.4418	1.3561	1.1806
17	2017	13	13.2143	90.1020	907.5878	1.2010	2.5480
55	2017	13	13.5429	81.8469	881.8776	2.1224	2.2949
46	2017	13	14.3429	83.0102	927.5500	1.5429	1.4816
86	2017	13	13.4429	81.9184	870.1357	2.5857	1.4776
2	2017	13	13.4429	81.9184	870.1357	2.5857	1.4776
4	2017	13	13.8571	81.0714	907.4265	1.2163	1.7143
47	2017	13	16.7000	77.2347	965.2184	1.6327	0.7939
82	2017	13	13.1571	85.6633	879.3592	2.1776	2.4888
19	2017	13	15.7143	81.1939	967.5857	1.1837	1.0500
20	2017	13	12.4000	81.6327	857.7816	2.5235	2.5520
80	2017	13	13.1571	85.6633	879.3592	2.1776	2.4888
3	2017	13	17.3571	75.4898	950.0163	2.5184	1.1112
52	2017	13	13.2143	90.1020	907.5878	1.2010	2.5480
70	2017	13	15.2429	80.5306	915.9102	2.1357	1.3235
64	2017	13	9.1286	71.2245	777.3888	4.9551	2.4500
48	2017	13	14.4143	81.3265	924.8857	1.5837	1.8418
65	2017	13	13.2143	90.1020	907.5878	1.2010	2.5480
44	2017	13	15.2429	80.5306	915.9102	2.1357	1.3235
75	2017	13	9.1286	71.2245	777.3888	4.9551	2.4500
40	2017	13	14.5714	88.8980	952.7633	1.3653	1.4735
11	2017	13	13.5429	81.8469	881.8776	2.1224	2.2949
35	2017	13	13.6143	86.5306	946.3133	1.1561	1.3592
78	2017	13	14.1429	82.0102	904.4643	2.1827	2.2449
28	2017	13	14.2000	83.8163	935.7061	1.2500	1.7092
39	2017	13	13.2143	90.1020	907.5878	1.2010	2.5480
24	2017	13	14.6429	82.0510	947.1388	1.2000	1.1459
63	2017	13	14.5714	88.8980	952.7633	1.3653	1.4735
62	2017	13	13.8571	79.1531	878.7612	1.3878	1.4551
1	2017	13	13.1571	85.6633	879.3592	2.1776	2.4888
31	2017	14	17.5286	73.2857	849.4378	3.5571	1.2612
79	2017	14	19.9429	75.8061	976.2337	2.6510	1.1500
51	2017	14	20.2000	78.7551	944.7429	2.7041	1.3704
14	2017	14	19.2000	80.1327	902.2531	2.6469	2.7133
67	2017	14	19.7857	80.7959	906.9541	2.6031	3.3102
42	2017	14	19.6571	74.0204	879.0612	3.7776	2.7235
50	2017	14	18.7143	74.1837	906.4092	2.5469	1.8378
43	2017	14	19.6571	74.0204	879.0612	3.7776	2.7235
85	2017	14	20.3000	70.0714	914.6888	3.3663	1.8592
25	2017	14	22.6143	79.4082	983.7224	2.2939	0.8184
69	2017	14	21.8286	75.2143	945.4418	2.1429	1.2531
57	2017	14	20.5429	73.8367	890.8776	2.8031	2.9204
9	2017	14	19.2000	71.9082	857.8367	4.0959	2.5908

72	2017	14	20.4571	72.8163	881.9490	3.4633	2.3378
26	2017	14	21.5429	72.4184	871.1112	4.7327	1.8633
7	2017	14	20.4000	72.1735	862.8857	4.7092	2.2786
83	2017	14	24.2429	69.0204	949.9551	4.6418	1.1796
76	2017	14	19.9857	74.4490	926.0735	3.2561	1.5071
36	2017	14	21.6143	74.2551	934.4827	2.8408	1.6724
81	2017	14	20.2000	78.7551	944.7429	2.7041	1.3704
15	2017	14	18.0000	75.1939	923.5347	2.3827	1.8245
32	2017	14	19.6571	74.0204	879.0612	3.7776	2.7235
73	2017	14	20.9571	73.0408	968.0194	2.9990	0.7612
71	2017	14	21.6143	74.2551	934.4827	2.8408	1.6724
41	2017	14	20.1714	68.3061	878.1643	2.6704	1.6102
10	2017	14	21.0286	78.2755	970.6255	2.5816	0.9459
23	2017	14	16.4857	73.4286	777.8898	4.4755	2.6051
27	2017	14	20.4000	72.1735	862.8857	4.7092	2.2786
60	2017	14	20.2000	78.7551	944.7429	2.7041	1.3704
53	2017	14	19.2000	71.9082	857.8367	4.0959	2.5908
66	2017	14	19.2000	80.1327	902.2531	2.6469	2.7133
59	2017	14	20.5429	73.8367	890.8776	2.8031	2.9204
61	2017	14	20.9571	73.0408	968.0194	2.9990	0.7612
84	2017	14	20.9571	73.0408	968.0194	2.9990	0.7612
38	2017	14	20.5429	73.8367	890.8776	2.8031	2.9204
87	2017	14	21.7429	73.4796	904.1510	4.4010	2.1867
34	2017	14	20.5429	73.8367	890.8776	2.8031	2.9204
29	2017	14	21.8286	75.2143	945.4418	2.1429	1.2531
5	2017	14	20.0429	71.2245	835.4347	5.2898	2.0010
8	2017	14	19.2000	71.9082	857.8367	4.0959	2.5908
12	2017	14	20.0429	71.2245	835.4347	5.2898	2.0010
13	2017	14	24.2429	69.0204	949.9551	4.6418	1.1796
18	2017	14	18.6143	76.7449	970.3755	2.2173	0.9969
33	2017	14	18.7143	74.1837	906.4092	2.5469	1.8378
56	2017	14	22.6143	79.4082	983.7224	2.2939	0.8184
77	2017	14	18.0000	75.1939	923.5347	2.3827	1.8245
54	2017	14	20.0429	71.2245	835.4347	5.2898	2.0010
21	2017	14	18.7143	74.1837	906.4092	2.5469	1.8378
68	2017	14	19.9429	75.8061	976.2337	2.6510	1.1500
74	2017	14	20.9571	73.0408	968.0194	2.9990	0.7612
88	2017	14	19.6571	74.0204	879.0612	3.7776	2.7235
16	2017	14	19.9857	74.4490	926.0735	3.2561	1.5071
30	2017	14	19.2000	80.1327	902.2531	2.6469	2.7133
6	2017	14	19.9429	75.8061	976.2337	2.6510	1.1500
49	2017	14	21.8286	75.2143	945.4418	2.1429	1.2531
22	2017	14	19.6571	74.0204	879.0612	3.7776	2.7235
45	2017	14	19.9143	64.4286	820.4143	4.8806	2.3908
58	2017	14	21.8286	75.2143	945.4418	2.1429	1.2531
37	2017	14	19.9429	75.8061	976.2337	2.6510	1.1500
17	2017	14	19.7857	80.7959	906.9541	2.6031	3.3102
55	2017	14	20.4571	72.8163	881.9490	3.4633	2.3378
46	2017	14	19.9857	74.4490	926.0735	3.2561	1.5071
86	2017	14	20.0000	72.4388	869.7806	3.6888	2.1561

2	2017	14	20.0000	72.4388	869.7806	3.6888	2.1561
4	2017	14	18.7143	74.1837	906.4092	2.5469	1.8378
47	2017	14	24.3143	72.7449	964.7173	3.2306	0.7684
82	2017	14	19.6571	74.0204	879.0612	3.7776	2.7235
19	2017	14	21.9429	78.5102	966.9520	2.1418	1.0082
20	2017	14	19.2000	71.9082	857.8367	4.0959	2.5908
80	2017	14	19.6571	74.0204	879.0612	3.7776	2.7235
3	2017	14	24.2429	69.0204	949.9551	4.6418	1.1796
52	2017	14	19.7857	80.7959	906.9541	2.6031	3.3102
70	2017	14	20.3000	70.0714	914.6888	3.3663	1.8592
64	2017	14	16.4857	73.4286	777.8898	4.4755	2.6051
48	2017	14	18.0000	75.1939	923.5347	2.3827	1.8245
65	2017	14	19.7857	80.7959	906.9541	2.6031	3.3102
44	2017	14	20.3000	70.0714	914.6888	3.3663	1.8592
75	2017	14	16.4857	73.4286	777.8898	4.4755	2.6051
40	2017	14	21.1000	78.3878	951.5847	2.7388	1.7561
11	2017	14	20.4571	72.8163	881.9490	3.4633	2.3378
35	2017	14	20.2000	78.7551	944.7429	2.7041	1.3704
78	2017	14	21.7429	73.4796	904.1510	4.4010	2.1867
28	2017	14	21.6143	74.2551	934.4827	2.8408	1.6724
39	2017	14	19.7857	80.7959	906.9541	2.6031	3.3102
24	2017	14	21.8286	75.2143	945.4418	2.1429	1.2531
63	2017	14	21.1000	78.3878	951.5847	2.7388	1.7561
62	2017	14	20.1714	68.3061	878.1643	2.6704	1.6102
1	2017	14	19.6571	74.0204	879.0612	3.7776	2.7235
31	2017	15	15.0429	70.2857	846.3480	4.3347	1.3357
79	2017	15	17.6286	74.3265	971.9020	2.8796	1.6092
51	2017	15	16.7714	75.2449	940.5235	3.8796	1.7847
14	2017	15	18.2000	75.4694	898.4704	4.0480	3.2378
67	2017	15	17.3857	76.5204	903.0908	4.0929	3.7429
42	2017	15	16.8714	71.5000	875.4776	4.5000	2.9490
50	2017	15	17.3000	73.7959	902.5480	3.6224	1.9582
43	2017	15	16.8714	71.5000	875.4776	4.5000	2.9490
85	2017	15	18.3857	67.8265	910.6449	3.9765	2.1745
25	2017	15	20.3714	73.7041	978.7867	3.6990	1.2439
69	2017	15	18.8429	70.3367	940.9714	2.6214	1.6939
57	2017	15	16.8286	71.2755	887.1286	3.5286	3.1796
9	2017	15	16.7429	68.4388	854.5214	6.0551	2.8602
72	2017	15	18.7286	67.9184	878.2847	5.5622	2.4082
26	2017	15	20.1286	60.5204	867.6918	6.8112	2.4949
7	2017	15	19.2571	62.9082	859.5724	6.7633	2.7010
83	2017	15	23.8000	61.0510	945.1133	6.9031	1.3163
76	2017	15	17.5429	72.3980	921.8908	4.0551	1.6837
36	2017	15	18.2571	68.6939	930.2153	4.4184	1.9306
81	2017	15	16.7714	75.2449	940.5235	3.8796	1.7847
15	2017	15	16.5857	77.6429	919.4449	2.1398	1.8367
32	2017	15	16.8714	71.5000	875.4776	4.5000	2.9490
73	2017	15	19.1857	69.0204	963.4010	3.1724	0.8724
71	2017	15	18.2571	68.6939	930.2153	4.4184	1.9306
41	2017	15	17.3857	66.1224	874.5929	4.1204	1.7663

10	2017	15	17.3571	76.6633	966.1551	3.8959	1.0296
23	2017	15	13.8714	63.1837	775.6541	7.4898	2.4490
27	2017	15	19.2571	62.9082	859.5724	6.7633	2.7010
60	2017	15	16.7714	75.2449	940.5235	3.8796	1.7847
53	2017	15	16.7429	68.4388	854.5214	6.0551	2.8602
66	2017	15	18.2000	75.4694	898.4704	4.0480	3.2378
59	2017	15	16.8286	71.2755	887.1286	3.5286	3.1796
61	2017	15	19.1857	69.0204	963.4010	3.1724	0.8724
84	2017	15	19.1857	69.0204	963.4010	3.1724	0.8724
38	2017	15	16.8286	71.2755	887.1286	3.5286	3.1796
87	2017	15	19.7429	68.0408	900.1286	6.9112	2.5051
34	2017	15	16.8286	71.2755	887.1286	3.5286	3.1796
29	2017	15	18.8429	70.3367	940.9714	2.6214	1.6939
5	2017	15	17.4000	58.9796	832.4398	7.2296	2.1724
8	2017	15	16.7429	68.4388	854.5214	6.0551	2.8602
12	2017	15	17.4000	58.9796	832.4398	7.2296	2.1724
13	2017	15	23.8000	61.0510	945.1133	6.9031	1.3163
18	2017	15	17.3714	77.5306	966.1847	2.2122	1.0133
33	2017	15	17.3000	73.7959	902.5480	3.6224	1.9582
56	2017	15	20.3714	73.7041	978.7867	3.6990	1.2439
77	2017	15	16.5857	77.6429	919.4449	2.1398	1.8367
54	2017	15	17.4000	58.9796	832.4398	7.2296	2.1724
21	2017	15	17.3000	73.7959	902.5480	3.6224	1.9582
68	2017	15	17.6286	74.3265	971.9020	2.8796	1.6092
74	2017	15	19.1857	69.0204	963.4010	3.1724	0.8724
88	2017	15	16.8714	71.5000	875.4776	4.5000	2.9490
16	2017	15	17.5429	72.3980	921.8908	4.0551	1.6837
30	2017	15	18.2000	75.4694	898.4704	4.0480	3.2378
6	2017	15	17.6286	74.3265	971.9020	2.8796	1.6092
49	2017	15	18.8429	70.3367	940.9714	2.6214	1.6939
22	2017	15	16.8714	71.5000	875.4776	4.5000	2.9490
45	2017	15	17.9429	54.5408	817.7980	7.2561	2.7827
58	2017	15	18.8429	70.3367	940.9714	2.6214	1.6939
37	2017	15	17.6286	74.3265	971.9020	2.8796	1.6092
17	2017	15	17.3857	76.5204	903.0908	4.0929	3.7429
55	2017	15	18.7286	67.9184	878.2847	5.5622	2.4082
46	2017	15	17.5429	72.3980	921.8908	4.0551	1.6837
86	2017	15	17.6429	69.3265	866.3020	4.8347	2.1776
2	2017	15	17.6429	69.3265	866.3020	4.8347	2.1776
4	2017	15	17.3000	73.7959	902.5480	3.6224	1.9582
47	2017	15	24.1429	64.9388	959.5633	5.9755	0.9480
82	2017	15	16.8714	71.5000	875.4776	4.5000	2.9490
19	2017	15	21.5714	73.5714	962.1980	3.3612	1.3388
20	2017	15	16.7429	68.4388	854.5214	6.0551	2.8602
80	2017	15	16.8714	71.5000	875.4776	4.5000	2.9490
3	2017	15	23.8000	61.0510	945.1133	6.9031	1.3163
52	2017	15	17.3857	76.5204	903.0908	4.0929	3.7429
70	2017	15	18.3857	67.8265	910.6449	3.9765	2.1745
64	2017	15	13.8714	63.1837	775.6541	7.4898	2.4490
48	2017	15	16.5857	77.6429	919.4449	2.1398	1.8367

65	2017	15	17.3857	76.5204	903.0908	4.0929	3.7429
44	2017	15	18.3857	67.8265	910.6449	3.9765	2.1745
75	2017	15	13.8714	63.1837	775.6541	7.4898	2.4490
40	2017	15	17.1571	75.2143	947.4214	4.1633	2.2000
11	2017	15	18.7286	67.9184	878.2847	5.5622	2.4082
35	2017	15	16.7714	75.2449	940.5235	3.8796	1.7847
78	2017	15	19.7429	68.0408	900.1286	6.9112	2.5051
28	2017	15	18.2571	68.6939	930.2153	4.4184	1.9306
39	2017	15	17.3857	76.5204	903.0908	4.0929	3.7429
24	2017	15	18.8429	70.3367	940.9714	2.6214	1.6939
63	2017	15	17.1571	75.2143	947.4214	4.1633	2.2000
62	2017	15	17.3857	66.1224	874.5929	4.1204	1.7663
1	2017	15	16.8714	71.5000	875.4776	4.5000	2.9490
31	2017	16	14.2857	70.7143	846.2867	3.8194	1.2398
79	2017	16	18.2286	76.3673	970.6122	3.0245	1.4214
51	2017	16	16.8571	76.6429	939.4010	4.0000	1.6378
14	2017	16	16.6000	75.7857	897.4469	4.4163	2.8898
67	2017	16	16.3286	77.4082	902.2204	4.2959	3.6122
42	2017	16	15.8143	73.8469	874.9561	4.0633	2.6888
50	2017	16	16.8714	73.5102	902.0592	3.9571	1.9082
43	2017	16	15.8143	73.8469	874.9561	4.0633	2.6888
85	2017	16	17.7000	69.3265	910.2551	3.8469	1.8878
25	2017	16	20.0429	72.6122	977.2459	4.4316	1.3031
69	2017	16	18.0143	69.2449	940.0653	3.0663	1.7735
57	2017	16	15.9714	74.2245	886.7541	3.0408	2.7102
9	2017	16	15.0000	71.0816	854.0969	5.6000	2.8622
72	2017	16	16.1571	70.5918	877.6316	4.7898	2.2224
26	2017	16	16.6571	64.4388	867.0684	5.4990	2.4061
7	2017	16	16.2000	64.7959	859.0592	5.5633	2.4173
83	2017	16	20.4143	62.2449	944.1041	5.9367	1.3429
76	2017	16	17.4571	71.8061	921.1806	3.8582	1.6571
36	2017	16	17.5714	69.4796	929.2592	4.8918	1.8806
81	2017	16	16.8571	76.6429	939.4010	4.0000	1.6378
15	2017	16	16.9000	79.4388	918.7847	2.6133	1.6837
32	2017	16	15.8143	73.8469	874.9561	4.0633	2.6888
73	2017	16	18.9857	69.3673	962.4061	3.0908	0.8449
71	2017	16	17.5714	69.4796	929.2592	4.8918	1.8806
41	2017	16	16.3000	66.7551	874.2837	4.0000	1.6908
10	2017	16	17.7286	77.1837	964.8031	3.7500	0.9959
23	2017	16	10.4286	64.2755	775.7561	6.8020	2.0592
27	2017	16	16.2000	64.7959	859.0592	5.5633	2.4173
60	2017	16	16.8571	76.6429	939.4010	4.0000	1.6378
53	2017	16	15.0000	71.0816	854.0969	5.6000	2.8622
66	2017	16	16.6000	75.7857	897.4469	4.4163	2.8898
59	2017	16	15.9714	74.2245	886.7541	3.0408	2.7102
61	2017	16	18.9857	69.3673	962.4061	3.0908	0.8449
84	2017	16	18.9857	69.3673	962.4061	3.0908	0.8449
38	2017	16	15.9714	74.2245	886.7541	3.0408	2.7102
87	2017	16	17.6286	68.5306	899.3867	6.2133	2.4643
34	2017	16	15.9714	74.2245	886.7541	3.0408	2.7102

29	2017	16	18.0143	69.2449	940.0653	3.0663	1.7735
5	2017	16	14.0286	64.4082	832.0908	6.0735	2.0541
8	2017	16	15.0000	71.0816	854.0969	5.6000	2.8622
12	2017	16	14.0286	64.4082	832.0908	6.0735	2.0541
13	2017	16	20.4143	62.2449	944.1041	5.9367	1.3429
18	2017	16	18.1857	78.7755	964.8939	2.8163	0.9316
33	2017	16	16.8714	73.5102	902.0592	3.9571	1.9082
56	2017	16	20.0429	72.6122	977.2459	4.4316	1.3031
77	2017	16	16.9000	79.4388	918.7847	2.6133	1.6837
54	2017	16	14.0286	64.4082	832.0908	6.0735	2.0541
21	2017	16	16.8714	73.5102	902.0592	3.9571	1.9082
68	2017	16	18.2286	76.3673	970.6122	3.0245	1.4214
74	2017	16	18.9857	69.3673	962.4061	3.0908	0.8449
88	2017	16	15.8143	73.8469	874.9561	4.0633	2.6888
16	2017	16	17.4571	71.8061	921.1806	3.8582	1.6571
30	2017	16	16.6000	75.7857	897.4469	4.4163	2.8898
6	2017	16	18.2286	76.3673	970.6122	3.0245	1.4214
49	2017	16	18.0143	69.2449	940.0653	3.0663	1.7735
22	2017	16	15.8143	73.8469	874.9561	4.0633	2.6888
45	2017	16	13.5286	57.7653	817.4663	6.5633	2.5847
58	2017	16	18.0143	69.2449	940.0653	3.0663	1.7735
37	2017	16	18.2286	76.3673	970.6122	3.0245	1.4214
17	2017	16	16.3286	77.4082	902.2204	4.2959	3.6122
55	2017	16	16.1571	70.5918	877.6316	4.7898	2.2224
46	2017	16	17.4571	71.8061	921.1806	3.8582	1.6571
86	2017	16	15.8571	70.2755	866.0306	4.7173	1.7806
2	2017	16	15.8571	70.2755	866.0306	4.7173	1.7806
4	2017	16	16.8714	73.5102	902.0592	3.9571	1.9082
47	2017	16	20.6286	64.8265	958.3684	5.7071	0.9245
82	2017	16	15.8143	73.8469	874.9561	4.0633	2.6888
19	2017	16	20.4286	71.2755	960.6602	3.5980	1.3745
20	2017	16	15.0000	71.0816	854.0969	5.6000	2.8622
80	2017	16	15.8143	73.8469	874.9561	4.0633	2.6888
3	2017	16	20.4143	62.2449	944.1041	5.9367	1.3429
52	2017	16	16.3286	77.4082	902.2204	4.2959	3.6122
70	2017	16	17.7000	69.3265	910.2551	3.8469	1.8878
64	2017	16	10.4286	64.2755	775.7561	6.8020	2.0592
48	2017	16	16.9000	79.4388	918.7847	2.6133	1.6837
65	2017	16	16.3286	77.4082	902.2204	4.2959	3.6122
44	2017	16	17.7000	69.3265	910.2551	3.8469	1.8878
75	2017	16	10.4286	64.2755	775.7561	6.8020	2.0592
40	2017	16	17.9143	76.7041	945.9990	4.3745	2.0520
11	2017	16	16.1571	70.5918	877.6316	4.7898	2.2224
35	2017	16	16.8571	76.6429	939.4010	4.0000	1.6378
78	2017	16	17.6286	68.5306	899.3867	6.2133	2.4643
28	2017	16	17.5714	69.4796	929.2592	4.8918	1.8806
39	2017	16	16.3286	77.4082	902.2204	4.2959	3.6122
24	2017	16	18.0143	69.2449	940.0653	3.0663	1.7735
63	2017	16	17.9143	76.7041	945.9990	4.3745	2.0520
62	2017	16	16.3000	66.7551	874.2837	4.0000	1.6908

1	2017	16	15.8143	73.8469	874.9561	4.0633	2.6888
31	2017	17	13.5429	75.7245	847.9827	2.1918	1.0990
79	2017	17	17.1286	78.7143	972.1755	2.8194	1.0306
51	2017	17	15.8286	80.9694	941.0929	2.8745	1.2908
14	2017	17	15.7286	78.7653	899.1286	2.9133	2.5112
67	2017	17	15.6000	82.2041	904.0143	2.5959	3.1776
42	2017	17	14.5143	77.8673	876.7827	2.5204	2.3704
50	2017	17	15.0571	75.6224	903.9531	2.8449	1.7133
43	2017	17	14.5143	77.8673	876.7827	2.5204	2.3704
85	2017	17	15.9000	73.1633	912.2765	2.8163	1.4286
25	2017	17	19.5143	76.2959	979.1122	3.6418	0.9378
69	2017	17	16.8714	75.7245	942.0867	2.3184	1.3480
57	2017	17	14.8286	77.5000	888.7337	1.9061	2.3776
9	2017	17	14.1143	76.5714	855.8765	2.9469	2.3949
72	2017	17	15.4571	75.9796	879.4888	2.2694	1.8388
26	2017	17	16.2857	77.9286	868.9051	2.3898	1.6796
7	2017	17	15.4571	75.8469	860.8500	2.5286	1.8276
83	2017	17	20.8286	70.0408	946.6796	3.2490	1.1653
76	2017	17	15.7143	74.9388	923.1184	2.8490	1.4694
36	2017	17	16.7143	75.1633	931.1347	3.5418	1.5092
81	2017	17	15.8286	80.9694	941.0929	2.8745	1.2908
15	2017	17	14.6143	79.9592	920.6847	2.8582	1.5102
32	2017	17	14.5143	77.8673	876.7827	2.5204	2.3704
73	2017	17	16.9286	75.1633	964.4041	2.5969	0.7469
71	2017	17	16.7143	75.1633	931.1347	3.5418	1.5092
41	2017	17	15.0000	72.4898	876.1806	2.3633	1.4510
10	2017	17	17.2286	79.0714	966.4653	3.0163	0.8153
23	2017	17	10.2571	78.9898	777.1041	2.7898	1.7429
27	2017	17	15.4571	75.8469	860.8500	2.5286	1.8276
60	2017	17	15.8286	80.9694	941.0929	2.8745	1.2908
53	2017	17	14.1143	76.5714	855.8765	2.9469	2.3949
66	2017	17	15.7286	78.7653	899.1286	2.9133	2.5112
59	2017	17	14.8286	77.5000	888.7337	1.9061	2.3776
61	2017	17	16.9286	75.1633	964.4041	2.5969	0.7469
84	2017	17	16.9286	75.1633	964.4041	2.5969	0.7469
38	2017	17	14.8286	77.5000	888.7337	1.9061	2.3776
87	2017	17	16.8143	73.8571	901.4378	3.1306	2.0531
34	2017	17	14.8286	77.5000	888.7337	1.9061	2.3776
29	2017	17	16.8714	75.7245	942.0867	2.3184	1.3480
5	2017	17	13.1571	79.8265	833.7694	2.6796	1.6735
8	2017	17	14.1143	76.5714	855.8765	2.9469	2.3949
12	2017	17	13.1571	79.8265	833.7694	2.6796	1.6735
13	2017	17	20.8286	70.0408	946.6796	3.2490	1.1653
18	2017	17	16.6429	78.9184	966.4204	3.1612	0.8878
33	2017	17	15.0571	75.6224	903.9531	2.8449	1.7133
56	2017	17	19.5143	76.2959	979.1122	3.6418	0.9378
77	2017	17	14.6143	79.9592	920.6847	2.8582	1.5102
54	2017	17	13.1571	79.8265	833.7694	2.6796	1.6735
21	2017	17	15.0571	75.6224	903.9531	2.8449	1.7133
68	2017	17	17.1286	78.7143	972.1755	2.8194	1.0306

74	2017	17	16.9286	75.1633	964.4041	2.5969	0.7469
88	2017	17	14.5143	77.8673	876.7827	2.5204	2.3704
16	2017	17	15.7143	74.9388	923.1184	2.8490	1.4694
30	2017	17	15.7286	78.7653	899.1286	2.9133	2.5112
6	2017	17	17.1286	78.7143	972.1755	2.8194	1.0306
49	2017	17	16.8714	75.7245	942.0867	2.3184	1.3480
22	2017	17	14.5143	77.8673	876.7827	2.5204	2.3704
45	2017	17	13.3143	75.7245	818.9102	3.0827	1.9439
58	2017	17	16.8714	75.7245	942.0867	2.3184	1.3480
37	2017	17	17.1286	78.7143	972.1755	2.8194	1.0306
17	2017	17	15.6000	82.2041	904.0143	2.5959	3.1776
55	2017	17	15.4571	75.9796	879.4888	2.2694	1.8388
46	2017	17	15.7143	74.9388	923.1184	2.8490	1.4694
86	2017	17	15.0286	76.0408	867.9133	3.0561	1.7214
2	2017	17	15.0286	76.0408	867.9133	3.0561	1.7214
4	2017	17	15.0571	75.6224	903.9531	2.8449	1.7133
47	2017	17	20.8000	71.3980	960.9071	2.8112	0.7816
82	2017	17	14.5143	77.8673	876.7827	2.5204	2.3704
19	2017	17	19.8286	73.0000	962.5867	2.6500	1.1469
20	2017	17	14.1143	76.5714	855.8765	2.9469	2.3949
80	2017	17	14.5143	77.8673	876.7827	2.5204	2.3704
3	2017	17	20.8286	70.0408	946.6796	3.2490	1.1653
52	2017	17	15.6000	82.2041	904.0143	2.5959	3.1776
70	2017	17	15.9000	73.1633	912.2765	2.8163	1.4286
64	2017	17	10.2571	78.9898	777.1041	2.7898	1.7429
48	2017	17	14.6143	79.9592	920.6847	2.8582	1.5102
65	2017	17	15.6000	82.2041	904.0143	2.5959	3.1776
44	2017	17	15.9000	73.1633	912.2765	2.8163	1.4286
75	2017	17	10.2571	78.9898	777.1041	2.7898	1.7429
40	2017	17	17.0286	77.8776	947.4806	3.2837	1.6000
11	2017	17	15.4571	75.9796	879.4888	2.2694	1.8388
35	2017	17	15.8286	80.9694	941.0929	2.8745	1.2908
78	2017	17	16.8143	73.8571	901.4378	3.1306	2.0531
28	2017	17	16.7143	75.1633	931.1347	3.5418	1.5092
39	2017	17	15.6000	82.2041	904.0143	2.5959	3.1776
24	2017	17	16.8714	75.7245	942.0867	2.3184	1.3480
63	2017	17	17.0286	77.8776	947.4806	3.2837	1.6000
62	2017	17	15.0000	72.4898	876.1806	2.3633	1.4510
1	2017	17	14.5143	77.8673	876.7827	2.5204	2.3704
31	2017	18	16.2143	77.1939	848.3541	3.0163	1.0816
79	2017	18	19.9429	79.0306	972.7684	2.6092	1.1367
51	2017	18	19.4857	82.0510	941.5367	2.4153	1.4806
14	2017	18	19.2571	79.3571	899.4296	2.3745	2.4765
67	2017	18	18.5143	84.4184	904.3765	2.0724	2.7408
42	2017	18	17.4714	80.7857	877.1071	2.8735	2.3561
50	2017	18	17.6143	81.4286	904.4459	1.9929	1.6776
43	2017	18	17.4714	80.7857	877.1071	2.8735	2.3561
85	2017	18	18.6000	78.3571	912.7367	2.7133	1.4357
25	2017	18	23.3143	75.7959	979.3429	3.8449	0.8980
69	2017	18	20.0000	80.0408	942.5327	1.4480	1.3459

57	2017	18	17.0000	81.5816	889.1806	2.1388	2.2490
9	2017	18	17.6286	77.0000	856.1704	3.5724	2.3224
72	2017	18	18.9429	75.8673	879.7020	3.0857	1.7551
26	2017	18	21.7000	74.4694	869.0173	4.0735	1.6357
7	2017	18	20.2571	74.1633	861.0235	3.7143	1.7929
83	2017	18	24.3286	68.1837	946.6255	4.2796	1.1724
76	2017	18	18.2143	80.6939	923.6959	2.5276	1.3490
36	2017	18	20.1571	76.9388	931.4796	2.6918	1.4204
81	2017	18	19.4857	82.0510	941.5367	2.4153	1.4806
15	2017	18	17.9000	83.2245	921.2704	2.3796	1.6918
32	2017	18	17.4714	80.7857	877.1071	2.8735	2.3561
73	2017	18	20.1000	79.0714	965.0388	1.9204	0.7980
71	2017	18	20.1571	76.9388	931.4796	2.6918	1.4204
41	2017	18	17.7857	76.4490	876.5867	2.6908	1.4041
10	2017	18	20.5143	80.3878	966.9051	2.9571	0.8531
23	2017	18	13.9143	80.5918	777.4286	4.0480	1.7449
27	2017	18	20.2571	74.1633	861.0235	3.7143	1.7929
60	2017	18	19.4857	82.0510	941.5367	2.4153	1.4806
53	2017	18	17.6286	77.0000	856.1704	3.5724	2.3224
66	2017	18	19.2571	79.3571	899.4296	2.3745	2.4765
59	2017	18	17.0000	81.5816	889.1806	2.1388	2.2490
61	2017	18	20.1000	79.0714	965.0388	1.9204	0.7980
84	2017	18	20.1000	79.0714	965.0388	1.9204	0.7980
38	2017	18	17.0000	81.5816	889.1806	2.1388	2.2490
87	2017	18	19.9714	74.7245	901.7224	3.2092	1.9449
34	2017	18	17.0000	81.5816	889.1806	2.1388	2.2490
29	2017	18	20.0000	80.0408	942.5327	1.4480	1.3459
5	2017	18	18.1571	78.8367	834.0388	3.8520	1.8327
8	2017	18	17.6286	77.0000	856.1704	3.5724	2.3224
12	2017	18	18.1571	78.8367	834.0388	3.8520	1.8327
13	2017	18	24.3286	68.1837	946.6255	4.2796	1.1724
18	2017	18	19.5429	80.2449	967.0878	2.5398	0.8724
33	2017	18	17.6143	81.4286	904.4459	1.9929	1.6776
56	2017	18	23.3143	75.7959	979.3429	3.8449	0.8980
77	2017	18	17.9000	83.2245	921.2704	2.3796	1.6918
54	2017	18	18.1571	78.8367	834.0388	3.8520	1.8327
21	2017	18	17.6143	81.4286	904.4459	1.9929	1.6776
68	2017	18	19.9429	79.0306	972.7684	2.6092	1.1367
74	2017	18	20.1000	79.0714	965.0388	1.9204	0.7980
88	2017	18	17.4714	80.7857	877.1071	2.8735	2.3561
16	2017	18	18.2143	80.6939	923.6959	2.5276	1.3490
30	2017	18	19.2571	79.3571	899.4296	2.3745	2.4765
6	2017	18	19.9429	79.0306	972.7684	2.6092	1.1367
49	2017	18	20.0000	80.0408	942.5327	1.4480	1.3459
22	2017	18	17.4714	80.7857	877.1071	2.8735	2.3561
45	2017	18	18.7571	77.2551	819.1388	4.3490	1.6939
58	2017	18	20.0000	80.0408	942.5327	1.4480	1.3459
37	2017	18	19.9429	79.0306	972.7684	2.6092	1.1367
17	2017	18	18.5143	84.4184	904.3765	2.0724	2.7408
55	2017	18	18.9429	75.8673	879.7020	3.0857	1.7551

46	2017	18	18.2143	80.6939	923.6959	2.5276	1.3490
86	2017	18	17.8143	78.4796	868.2459	3.7122	1.4622
2	2017	18	17.8143	78.4796	868.2459	3.7122	1.4622
4	2017	18	17.6143	81.4286	904.4459	1.9929	1.6776
47	2017	18	24.3714	70.2245	960.9367	2.8969	0.8092
82	2017	18	17.4714	80.7857	877.1071	2.8735	2.3561
19	2017	18	23.2571	72.8469	962.7398	2.6531	1.1071
20	2017	18	17.6286	77.0000	856.1704	3.5724	2.3224
80	2017	18	17.4714	80.7857	877.1071	2.8735	2.3561
3	2017	18	24.3286	68.1837	946.6255	4.2796	1.1724
52	2017	18	18.5143	84.4184	904.3765	2.0724	2.7408
70	2017	18	18.6000	78.3571	912.7367	2.7133	1.4357
64	2017	18	13.9143	80.5918	777.4286	4.0480	1.7449
48	2017	18	17.9000	83.2245	921.2704	2.3796	1.6918
65	2017	18	18.5143	84.4184	904.3765	2.0724	2.7408
44	2017	18	18.6000	78.3571	912.7367	2.7133	1.4357
75	2017	18	13.9143	80.5918	777.4286	4.0480	1.7449
40	2017	18	20.4143	79.8469	947.8929	3.3714	1.4469
11	2017	18	18.9429	75.8673	879.7020	3.0857	1.7551
35	2017	18	19.4857	82.0510	941.5367	2.4153	1.4806
78	2017	18	19.9714	74.7245	901.7224	3.2092	1.9449
28	2017	18	20.1571	76.9388	931.4796	2.6918	1.4204
39	2017	18	18.5143	84.4184	904.3765	2.0724	2.7408
24	2017	18	20.0000	80.0408	942.5327	1.4480	1.3459
63	2017	18	20.4143	79.8469	947.8929	3.3714	1.4469
62	2017	18	17.7857	76.4490	876.5867	2.6908	1.4041
1	2017	18	17.4714	80.7857	877.1071	2.8735	2.3561
31	2017	19	17.6857	71.9388	849.0327	5.1337	1.1643
79	2017	19	21.7286	77.5306	972.5959	4.2306	1.3031
51	2017	19	20.7429	78.5816	941.5541	3.7163	1.5031
14	2017	19	19.7857	80.1939	899.7980	3.3480	2.2571
67	2017	19	19.8429	82.3571	904.6429	3.4357	2.7745
42	2017	19	19.1143	78.8367	877.5622	4.4908	2.5520
50	2017	19	19.0429	80.1837	904.9388	3.1265	1.8622
43	2017	19	19.1143	78.8367	877.5622	4.4908	2.5520
85	2017	19	20.3000	74.5306	913.2663	3.6755	1.5980
25	2017	19	22.7143	75.8469	979.2429	4.4847	0.9429
69	2017	19	20.6714	78.2041	942.6286	2.2245	1.3969
57	2017	19	19.4714	79.1837	889.6510	3.7663	2.3500
9	2017	19	18.9429	73.6122	856.6480	5.7316	2.5429
72	2017	19	19.3571	74.7959	880.0837	4.6082	1.9571
26	2017	19	20.6143	71.1224	869.3398	6.6214	1.8245
7	2017	19	19.7286	70.6939	861.4755	6.2847	2.0276
83	2017	19	23.8000	67.0408	946.6327	5.8796	1.3276
76	2017	19	19.7857	78.4184	923.9582	3.8663	1.4143
36	2017	19	21.2429	75.2653	931.6265	3.7765	1.3837
81	2017	19	20.7429	78.5816	941.5541	3.7163	1.5031
15	2017	19	18.9000	81.4694	921.6214	3.1000	1.7908
32	2017	19	19.1143	78.8367	877.5622	4.4908	2.5520
73	2017	19	21.3286	76.5000	964.9867	2.9357	0.8204

71	2017	19	21.2429	75.2653	931.6265	3.7765	1.3837
41	2017	19	19.8714	72.1939	877.1020	4.2592	1.5296
10	2017	19	21.8714	78.2449	966.7602	4.5602	0.9969
23	2017	19	15.2714	73.0714	778.3010	6.7765	1.9031
27	2017	19	19.7286	70.6939	861.4755	6.2847	2.0276
60	2017	19	20.7429	78.5816	941.5541	3.7163	1.5031
53	2017	19	18.9429	73.6122	856.6480	5.7316	2.5429
66	2017	19	19.7857	80.1939	899.7980	3.3480	2.2571
59	2017	19	19.4714	79.1837	889.6510	3.7663	2.3500
61	2017	19	21.3286	76.5000	964.9867	2.9357	0.8204
84	2017	19	21.3286	76.5000	964.9867	2.9357	0.8204
38	2017	19	19.4714	79.1837	889.6510	3.7663	2.3500
87	2017	19	20.6714	73.2347	902.0449	4.9959	2.1112
34	2017	19	19.4714	79.1837	889.6510	3.7663	2.3500
29	2017	19	20.6714	78.2041	942.6286	2.2245	1.3969
5	2017	19	18.4714	73.5918	834.6020	6.6520	1.8776
8	2017	19	18.9429	73.6122	856.6480	5.7316	2.5429
12	2017	19	18.4714	73.5918	834.6020	6.6520	1.8776
13	2017	19	23.8000	67.0408	946.6327	5.8796	1.3276
18	2017	19	21.1857	77.6633	966.9439	3.5531	0.8990
33	2017	19	19.0429	80.1837	904.9388	3.1265	1.8622
56	2017	19	22.7143	75.8469	979.2429	4.4847	0.9429
77	2017	19	18.9000	81.4694	921.6214	3.1000	1.7908
54	2017	19	18.4714	73.5918	834.6020	6.6520	1.8776
21	2017	19	19.0429	80.1837	904.9388	3.1265	1.8622
68	2017	19	21.7286	77.5306	972.5959	4.2306	1.3031
74	2017	19	21.3286	76.5000	964.9867	2.9357	0.8204
88	2017	19	19.1143	78.8367	877.5622	4.4908	2.5520
16	2017	19	19.7857	78.4184	923.9582	3.8663	1.4143
30	2017	19	19.7857	80.1939	899.7980	3.3480	2.2571
6	2017	19	21.7286	77.5306	972.5959	4.2306	1.3031
49	2017	19	20.6714	78.2041	942.6286	2.2245	1.3969
22	2017	19	19.1143	78.8367	877.5622	4.4908	2.5520
45	2017	19	18.5286	68.2245	819.7173	6.7990	1.8571
58	2017	19	20.6714	78.2041	942.6286	2.2245	1.3969
37	2017	19	21.7286	77.5306	972.5959	4.2306	1.3031
17	2017	19	19.8429	82.3571	904.6429	3.4357	2.7745
55	2017	19	19.3571	74.7959	880.0837	4.6082	1.9571
46	2017	19	19.7857	78.4184	923.9582	3.8663	1.4143
86	2017	19	19.5286	75.1122	868.7949	5.5510	1.4020
2	2017	19	19.5286	75.1122	868.7949	5.5510	1.4020
4	2017	19	19.0429	80.1837	904.9388	3.1265	1.8622
47	2017	19	23.2714	71.1837	960.9316	4.2296	0.8745
82	2017	19	19.1143	78.8367	877.5622	4.4908	2.5520
19	2017	19	23.0286	74.6122	962.7898	3.4031	1.2276
20	2017	19	18.9429	73.6122	856.6480	5.7316	2.5429
80	2017	19	19.1143	78.8367	877.5622	4.4908	2.5520
3	2017	19	23.8000	67.0408	946.6327	5.8796	1.3276
52	2017	19	19.8429	82.3571	904.6429	3.4357	2.7745
70	2017	19	20.3000	74.5306	913.2663	3.6755	1.5980

64	2017	19	15.2714	73.0714	778.3010	6.7765	1.9031
48	2017	19	18.9000	81.4694	921.6214	3.1000	1.7908
65	2017	19	19.8429	82.3571	904.6429	3.4357	2.7745
44	2017	19	20.3000	74.5306	913.2663	3.6755	1.5980
75	2017	19	15.2714	73.0714	778.3010	6.7765	1.9031
40	2017	19	21.4429	79.7551	947.9908	5.0449	1.4378
11	2017	19	19.3571	74.7959	880.0837	4.6082	1.9571
35	2017	19	20.7429	78.5816	941.5541	3.7163	1.5031
78	2017	19	20.6714	73.2347	902.0449	4.9959	2.1112
28	2017	19	21.2429	75.2653	931.6265	3.7765	1.3837
39	2017	19	19.8429	82.3571	904.6429	3.4357	2.7745
24	2017	19	20.6714	78.2041	942.6286	2.2245	1.3969
63	2017	19	21.4429	79.7551	947.9908	5.0449	1.4378
62	2017	19	19.8714	72.1939	877.1020	4.2592	1.5296
1	2017	19	19.1143	78.8367	877.5622	4.4908	2.5520
31	2017	20	18.8714	67.3061	849.2735	4.7551	1.2153
79	2017	20	22.5857	75.3776	971.9051	5.9031	1.2990
51	2017	20	20.7000	76.2245	941.2082	4.9357	1.3082
14	2017	20	18.5714	79.5102	899.8459	3.7612	1.9071
67	2017	20	18.9571	80.5714	904.5969	4.2704	2.7510
42	2017	20	18.5571	74.3980	877.7500	4.4010	2.5367
50	2017	20	20.9286	73.3061	904.8184	4.6755	1.7633
43	2017	20	18.5571	74.3980	877.7500	4.4010	2.5367
85	2017	20	22.1000	67.7857	913.1449	4.6245	1.6857
25	2017	20	22.5000	77.2959	978.9612	4.6439	0.7633
69	2017	20	21.6286	76.1939	942.4020	3.4408	1.1918
57	2017	20	19.7571	73.4082	889.6480	4.8357	2.5327
9	2017	20	17.8143	70.9490	856.9551	5.1041	2.4551
72	2017	20	18.2571	74.6837	880.3582	3.9122	1.8306
26	2017	20	18.3429	74.8571	869.8561	5.2194	1.5490
7	2017	20	18.4286	72.9286	861.9276	5.1959	2.0337
83	2017	20	21.9857	71.9388	947.0827	4.6459	1.2939
76	2017	20	21.4571	72.5000	923.6765	5.3133	1.4929
36	2017	20	21.0286	74.2449	931.4694	5.1490	1.3796
81	2017	20	20.7000	76.2245	941.2082	4.9357	1.3082
15	2017	20	21.2857	75.8469	921.3143	4.8908	1.6235
32	2017	20	18.5571	74.3980	877.7500	4.4010	2.5367
73	2017	20	23.4429	71.6735	964.3643	4.7357	0.8061
71	2017	20	21.0286	74.2449	931.4694	5.1490	1.3796
41	2017	20	20.2571	65.2653	877.2041	4.1500	1.5939
10	2017	20	21.4571	75.5102	966.2296	5.8878	0.9000
23	2017	20	14.8571	71.6633	778.8480	5.2918	1.8418
27	2017	20	18.4286	72.9286	861.9276	5.1959	2.0337
60	2017	20	20.7000	76.2245	941.2082	4.9357	1.3082
53	2017	20	17.8143	70.9490	856.9551	5.1041	2.4551
66	2017	20	18.5714	79.5102	899.8459	3.7612	1.9071
59	2017	20	19.7571	73.4082	889.6480	4.8357	2.5327
61	2017	20	23.4429	71.6735	964.3643	4.7357	0.8061
84	2017	20	23.4429	71.6735	964.3643	4.7357	0.8061
38	2017	20	19.7571	73.4082	889.6480	4.8357	2.5327

87	2017	20	19.8143	73.3367	902.2490	5.0571	2.0173
34	2017	20	19.7571	73.4082	889.6480	4.8357	2.5327
29	2017	20	21.6286	76.1939	942.4020	3.4408	1.1918
5	2017	20	17.2000	73.1224	835.0796	5.3398	1.5347
8	2017	20	17.8143	70.9490	856.9551	5.1041	2.4551
12	2017	20	17.2000	73.1224	835.0796	5.3398	1.5347
13	2017	20	21.9857	71.9388	947.0827	4.6459	1.2939
18	2017	20	22.4714	74.7143	966.2245	5.1490	0.8857
33	2017	20	20.9286	73.3061	904.8184	4.6755	1.7633
56	2017	20	22.5000	77.2959	978.9612	4.6439	0.7633
77	2017	20	21.2857	75.8469	921.3143	4.8908	1.6235
54	2017	20	17.2000	73.1224	835.0796	5.3398	1.5347
21	2017	20	20.9286	73.3061	904.8184	4.6755	1.7633
68	2017	20	22.5857	75.3776	971.9051	5.9031	1.2990
74	2017	20	23.4429	71.6735	964.3643	4.7357	0.8061
88	2017	20	18.5571	74.3980	877.7500	4.4010	2.5367
16	2017	20	21.4571	72.5000	923.6765	5.3133	1.4929
30	2017	20	18.5714	79.5102	899.8459	3.7612	1.9071
6	2017	20	22.5857	75.3776	971.9051	5.9031	1.2990
49	2017	20	21.6286	76.1939	942.4020	3.4408	1.1918
22	2017	20	18.5571	74.3980	877.7500	4.4010	2.5367
45	2017	20	16.9143	68.6122	820.2429	5.2888	1.7786
58	2017	20	21.6286	76.1939	942.4020	3.4408	1.1918
37	2017	20	22.5857	75.3776	971.9051	5.9031	1.2990
17	2017	20	18.9571	80.5714	904.5969	4.2704	2.7510
55	2017	20	18.2571	74.6837	880.3582	3.9122	1.8306
46	2017	20	21.4571	72.5000	923.6765	5.3133	1.4929
86	2017	20	19.0429	70.3469	869.0602	5.0286	1.6296
2	2017	20	19.0429	70.3469	869.0602	5.0286	1.6296
4	2017	20	20.9286	73.3061	904.8184	4.6755	1.7633
47	2017	20	21.9000	77.1633	961.2276	3.9031	0.7388
82	2017	20	18.5571	74.3980	877.7500	4.4010	2.5367
19	2017	20	21.7857	77.4388	962.7408	3.5510	1.2133
20	2017	20	17.8143	70.9490	856.9551	5.1041	2.4551
80	2017	20	18.5571	74.3980	877.7500	4.4010	2.5367
3	2017	20	21.9857	71.9388	947.0827	4.6459	1.2939
52	2017	20	18.9571	80.5714	904.5969	4.2704	2.7510
70	2017	20	22.1000	67.7857	913.1449	4.6245	1.6857
64	2017	20	14.8571	71.6633	778.8480	5.2918	1.8418
48	2017	20	21.2857	75.8469	921.3143	4.8908	1.6235
65	2017	20	18.9571	80.5714	904.5969	4.2704	2.7510
44	2017	20	22.1000	67.7857	913.1449	4.6245	1.6857
75	2017	20	14.8571	71.6633	778.8480	5.2918	1.8418
40	2017	20	20.7857	77.0102	947.6133	6.1204	1.2673
11	2017	20	18.2571	74.6837	880.3582	3.9122	1.8306
35	2017	20	20.7000	76.2245	941.2082	4.9357	1.3082
78	2017	20	19.8143	73.3367	902.2490	5.0571	2.0173
28	2017	20	21.0286	74.2449	931.4694	5.1490	1.3796
39	2017	20	18.9571	80.5714	904.5969	4.2704	2.7510
24	2017	20	21.6286	76.1939	942.4020	3.4408	1.1918

63	2017	20	20.7857	77.0102	947.6133	6.1204	1.2673
62	2017	20	20.2571	65.2653	877.2041	4.1500	1.5939
1	2017	20	18.5571	74.3980	877.7500	4.4010	2.5367
31	2017	21	16.9714	69.0816	847.9857	2.9980	1.1908
79	2017	21	22.4143	75.8673	970.0980	4.5724	1.1408
51	2017	21	20.4714	77.6224	939.6173	3.9582	1.3224
14	2017	21	19.3000	80.1633	898.5357	2.8531	1.9102
67	2017	21	18.8714	82.8469	903.2663	3.0847	2.6214
42	2017	21	18.4571	75.8163	876.4816	3.3980	2.3469
50	2017	21	19.4000	70.9286	903.2194	4.1306	1.7480
43	2017	21	18.4571	75.8163	876.4816	3.3980	2.3469
85	2017	21	20.1857	67.8265	911.4143	4.7102	1.7633
25	2017	21	22.2143	80.1837	977.3908	3.4653	0.5949
69	2017	21	20.6143	77.2653	940.8082	2.9276	1.1051
57	2017	21	18.4571	74.1224	888.2071	4.0755	2.5102
9	2017	21	17.8714	73.7041	855.7990	3.6163	2.3969
72	2017	21	18.9000	76.3673	879.1224	2.7388	1.7347
26	2017	21	20.0143	78.7143	868.7949	3.6459	1.4561
7	2017	21	18.9571	75.9592	860.7878	3.5990	1.9071
83	2017	21	22.9714	76.0306	945.7867	3.6531	1.2061
76	2017	21	20.3000	72.6429	922.0724	4.2857	1.4143
36	2017	21	20.4429	75.4388	929.9878	3.9806	1.4367
81	2017	21	20.4714	77.6224	939.6173	3.9582	1.3224
15	2017	21	19.6857	74.8878	919.6633	4.2245	1.4939
32	2017	21	18.4571	75.8163	876.4816	3.3980	2.3469
73	2017	21	21.9714	73.1020	962.5449	4.0327	0.8357
71	2017	21	20.4429	75.4388	929.9878	3.9806	1.4367
41	2017	21	18.7000	66.6122	875.8367	3.1398	1.6490
10	2017	21	21.3857	77.7245	964.5827	4.2194	0.7806
23	2017	21	13.6429	75.2653	777.9908	3.9724	1.8929
27	2017	21	18.9571	75.9592	860.7878	3.5990	1.9071
60	2017	21	20.4714	77.6224	939.6173	3.9582	1.3224
53	2017	21	17.8714	73.7041	855.7990	3.6163	2.3969
66	2017	21	19.3000	80.1633	898.5357	2.8531	1.9102
59	2017	21	18.4571	74.1224	888.2071	4.0755	2.5102
61	2017	21	21.9714	73.1020	962.5449	4.0327	0.8357
84	2017	21	21.9714	73.1020	962.5449	4.0327	0.8357
38	2017	21	18.4571	74.1224	888.2071	4.0755	2.5102
87	2017	21	19.7571	75.5612	900.9235	3.7980	1.8898
34	2017	21	18.4571	74.1224	888.2071	4.0755	2.5102
29	2017	21	20.6143	77.2653	940.8082	2.9276	1.1051
5	2017	21	17.1429	76.3571	834.0408	3.2816	1.4551
8	2017	21	17.8714	73.7041	855.7990	3.6163	2.3969
12	2017	21	17.1429	76.3571	834.0408	3.2816	1.4551
13	2017	21	22.9714	76.0306	945.7867	3.6531	1.2061
18	2017	21	21.8000	77.5204	964.4418	3.9357	0.8480
33	2017	21	19.4000	70.9286	903.2194	4.1306	1.7480
56	2017	21	22.2143	80.1837	977.3908	3.4653	0.5949
77	2017	21	19.6857	74.8878	919.6633	4.2245	1.4939
54	2017	21	17.1429	76.3571	834.0408	3.2816	1.4551

21	2017	21	19.4000	70.9286	903.2194	4.1306	1.7480
68	2017	21	22.4143	75.8673	970.0980	4.5724	1.1408
74	2017	21	21.9714	73.1020	962.5449	4.0327	0.8357
88	2017	21	18.4571	75.8163	876.4816	3.3980	2.3469
16	2017	21	20.3000	72.6429	922.0724	4.2857	1.4143
30	2017	21	19.3000	80.1633	898.5357	2.8531	1.9102
6	2017	21	22.4143	75.8673	970.0980	4.5724	1.1408
49	2017	21	20.6143	77.2653	940.8082	2.9276	1.1051
22	2017	21	18.4571	75.8163	876.4816	3.3980	2.3469
45	2017	21	17.0143	73.6735	819.2735	3.5347	1.6918
58	2017	21	20.6143	77.2653	940.8082	2.9276	1.1051
37	2017	21	22.4143	75.8673	970.0980	4.5724	1.1408
17	2017	21	18.8714	82.8469	903.2663	3.0847	2.6214
55	2017	21	18.9000	76.3673	879.1224	2.7388	1.7347
46	2017	21	20.3000	72.6429	922.0724	4.2857	1.4143
86	2017	21	18.1286	73.4796	867.7786	3.9490	1.6408
2	2017	21	18.1286	73.4796	867.7786	3.9490	1.6408
4	2017	21	19.4000	70.9286	903.2194	4.1306	1.7480
47	2017	21	22.7714	80.0204	959.8633	2.7347	0.5612
82	2017	21	18.4571	75.8163	876.4816	3.3980	2.3469
19	2017	21	22.5286	79.2245	961.2949	2.4531	1.0194
20	2017	21	17.8714	73.7041	855.7990	3.6163	2.3969
80	2017	21	18.4571	75.8163	876.4816	3.3980	2.3469
3	2017	21	22.9714	76.0306	945.7867	3.6531	1.2061
52	2017	21	18.8714	82.8469	903.2663	3.0847	2.6214
70	2017	21	20.1857	67.8265	911.4143	4.7102	1.7633
64	2017	21	13.6429	75.2653	777.9908	3.9724	1.8929
48	2017	21	19.6857	74.8878	919.6633	4.2245	1.4939
65	2017	21	18.8714	82.8469	903.2663	3.0847	2.6214
44	2017	21	20.1857	67.8265	911.4143	4.7102	1.7633
75	2017	21	13.6429	75.2653	777.9908	3.9724	1.8929
40	2017	21	20.7143	78.6224	946.0602	4.3796	1.0745
11	2017	21	18.9000	76.3673	879.1224	2.7388	1.7347
35	2017	21	20.4714	77.6224	939.6173	3.9582	1.3224
78	2017	21	19.7571	75.5612	900.9235	3.7980	1.8898
28	2017	21	20.4429	75.4388	929.9878	3.9806	1.4367
39	2017	21	18.8714	82.8469	903.2663	3.0847	2.6214
24	2017	21	20.6143	77.2653	940.8082	2.9276	1.1051
63	2017	21	20.7143	78.6224	946.0602	4.3796	1.0745
62	2017	21	18.7000	66.6122	875.8367	3.1398	1.6490
1	2017	21	18.4571	75.8163	876.4816	3.3980	2.3469
31	2017	22	20.0286	71.7959	847.0092	2.2153	1.1500
79	2017	22	23.5714	73.8265	968.7429	4.3602	1.0469
51	2017	22	22.4857	75.7041	938.3724	4.1204	1.2714
14	2017	22	22.3429	75.1020	897.3898	4.4235	1.9857
67	2017	22	22.1571	78.2551	902.1378	3.8122	2.6622
42	2017	22	21.8857	72.0204	875.3857	4.7337	2.3704
50	2017	22	21.4857	70.4082	902.0898	4.0061	1.8520
43	2017	22	21.8857	72.0204	875.3857	4.7337	2.3704
85	2017	22	22.7000	68.2857	910.2520	4.8347	1.7490

25	2017	22	25.3143	78.0408	976.0367	4.1622	0.7367
69	2017	22	23.4286	74.6837	939.5480	3.9286	1.1837
57	2017	22	21.6143	72.5816	887.1000	4.1857	2.2704
9	2017	22	21.7000	71.9694	854.7633	5.0378	2.3357
72	2017	22	22.6571	72.7449	877.9786	4.9806	1.7480
26	2017	22	24.1857	73.9592	867.6102	5.9490	1.7490
7	2017	22	23.4714	72.7449	859.6571	5.6020	1.9092
83	2017	22	27.3571	71.7653	944.1959	6.4051	1.1531
76	2017	22	22.1429	71.2755	920.9122	4.3082	1.4357
36	2017	22	23.5286	71.7857	928.7398	4.2786	1.4704
81	2017	22	22.4857	75.7041	938.3724	4.1204	1.2714
15	2017	22	20.4286	76.5816	918.5980	3.4776	1.3816
32	2017	22	21.8857	72.0204	875.3857	4.7337	2.3704
73	2017	22	23.0286	73.8163	961.3408	3.6082	0.8806
71	2017	22	23.5286	71.7857	928.7398	4.2786	1.4704
41	2017	22	22.2571	68.1735	874.8061	3.7235	1.6122
10	2017	22	23.5857	76.2857	963.2255	3.9245	0.8429
23	2017	22	17.7714	76.3776	777.2173	4.1082	1.8592
27	2017	22	23.4714	72.7449	859.6571	5.6020	1.9092
60	2017	22	22.4857	75.7041	938.3724	4.1204	1.2714
53	2017	22	21.7000	71.9694	854.7633	5.0378	2.3357
66	2017	22	22.3429	75.1020	897.3898	4.4235	1.9857
59	2017	22	21.6143	72.5816	887.1000	4.1857	2.2704
61	2017	22	23.0286	73.8163	961.3408	3.6082	0.8806
84	2017	22	23.0286	73.8163	961.3408	3.6082	0.8806
38	2017	22	21.6143	72.5816	887.1000	4.1857	2.2704
87	2017	22	23.8571	71.9694	899.6724	5.2582	1.9724
34	2017	22	21.6143	72.5816	887.1000	4.1857	2.2704
29	2017	22	23.4286	74.6837	939.5480	3.9286	1.1837
5	2017	22	21.6857	74.7551	833.0418	5.0724	1.6051
8	2017	22	21.7000	71.9694	854.7633	5.0378	2.3357
12	2017	22	21.6857	74.7551	833.0418	5.0724	1.6051
13	2017	22	27.3571	71.7653	944.1959	6.4051	1.1531
18	2017	22	22.3571	78.0510	963.1357	3.3786	0.8439
33	2017	22	21.4857	70.4082	902.0898	4.0061	1.8520
56	2017	22	25.3143	78.0408	976.0367	4.1622	0.7367
77	2017	22	20.4286	76.5816	918.5980	3.4776	1.3816
54	2017	22	21.6857	74.7551	833.0418	5.0724	1.6051
21	2017	22	21.4857	70.4082	902.0898	4.0061	1.8520
68	2017	22	23.5714	73.8265	968.7429	4.3602	1.0469
74	2017	22	23.0286	73.8163	961.3408	3.6082	0.8806
88	2017	22	21.8857	72.0204	875.3857	4.7337	2.3704
16	2017	22	22.1429	71.2755	920.9122	4.3082	1.4357
30	2017	22	22.3429	75.1020	897.3898	4.4235	1.9857
6	2017	22	23.5714	73.8265	968.7429	4.3602	1.0469
49	2017	22	23.4286	74.6837	939.5480	3.9286	1.1837
22	2017	22	21.8857	72.0204	875.3857	4.7337	2.3704
45	2017	22	22.0286	71.8980	818.3071	5.0255	1.7969
58	2017	22	23.4286	74.6837	939.5480	3.9286	1.1837
37	2017	22	23.5714	73.8265	968.7429	4.3602	1.0469

17	2017	22	22.1571	78.2551	902.1378	3.8122	2.6622
55	2017	22	22.6571	72.7449	877.9786	4.9806	1.7480
46	2017	22	22.1429	71.2755	920.9122	4.3082	1.4357
86	2017	22	22.0857	74.4490	866.6939	4.8776	1.4939
2	2017	22	22.0857	74.4490	866.6939	4.8776	1.4939
4	2017	22	21.4857	70.4082	902.0898	4.0061	1.8520
47	2017	22	26.8429	75.1327	958.2939	4.9612	0.6245
82	2017	22	21.8857	72.0204	875.3857	4.7337	2.3704
19	2017	22	25.9286	74.2041	959.9010	3.7204	1.0204
20	2017	22	21.7000	71.9694	854.7633	5.0378	2.3357
80	2017	22	21.8857	72.0204	875.3857	4.7337	2.3704
3	2017	22	27.3571	71.7653	944.1959	6.4051	1.1531
52	2017	22	22.1571	78.2551	902.1378	3.8122	2.6622
70	2017	22	22.7000	68.2857	910.2520	4.8347	1.7490
64	2017	22	17.7714	76.3776	777.2173	4.1082	1.8592
48	2017	22	20.4286	76.5816	918.5980	3.4776	1.3816
65	2017	22	22.1571	78.2551	902.1378	3.8122	2.6622
44	2017	22	22.7000	68.2857	910.2520	4.8347	1.7490
75	2017	22	17.7714	76.3776	777.2173	4.1082	1.8592
40	2017	22	23.6143	75.8061	944.7990	4.9959	1.1969
11	2017	22	22.6571	72.7449	877.9786	4.9806	1.7480
35	2017	22	22.4857	75.7041	938.3724	4.1204	1.2714
78	2017	22	23.8571	71.9694	899.6724	5.2582	1.9724
28	2017	22	23.5286	71.7857	928.7398	4.2786	1.4704
39	2017	22	22.1571	78.2551	902.1378	3.8122	2.6622
24	2017	22	23.4286	74.6837	939.5480	3.9286	1.1837
63	2017	22	23.6143	75.8061	944.7990	4.9959	1.1969
62	2017	22	22.2571	68.1735	874.8061	3.7235	1.6122
1	2017	22	21.8857	72.0204	875.3857	4.7337	2.3704
31	2017	23	21.1714	71.9490	845.4439	3.2010	1.0888
79	2017	23	24.6857	76.4592	966.5347	4.3684	1.1735
51	2017	23	23.9286	77.9184	936.3041	4.1765	1.1122
14	2017	23	22.8286	76.3367	895.5612	4.8735	1.9133
67	2017	23	22.5286	78.8571	900.2224	4.1367	2.5704
42	2017	23	21.9286	71.4490	873.6082	5.8714	2.4980
50	2017	23	22.9429	72.3061	900.1980	5.1704	1.6408
43	2017	23	21.9286	71.4490	873.6082	5.8714	2.4980
85	2017	23	23.9143	69.2449	908.3602	5.3357	1.7398
25	2017	23	26.5143	77.4490	973.6480	4.9827	0.8143
69	2017	23	24.5143	74.4388	937.4092	4.5173	1.3755
57	2017	23	22.2286	73.6122	885.3041	4.6224	2.1378
9	2017	23	21.4429	69.5102	853.1510	6.6704	2.3439
72	2017	23	22.5429	71.1531	876.1816	7.0663	1.8714
26	2017	23	23.1143	68.9082	865.8224	8.4408	1.8388
7	2017	23	22.8571	68.1429	857.9643	7.7459	2.0755
83	2017	23	27.1143	67.7449	941.7163	8.4929	1.1684
76	2017	23	23.7429	71.5510	918.9102	5.0867	1.5041
36	2017	23	24.3429	72.7959	926.6490	4.6510	1.4388
81	2017	23	23.9286	77.9184	936.3041	4.1765	1.1122
15	2017	23	23.2714	77.7755	916.6337	3.7143	1.4031

32	2017	23	21.9286	71.4490	873.6082	5.8714	2.4980
73	2017	23	25.5000	75.3061	959.1500	3.7327	0.8398
71	2017	23	24.3429	72.7959	926.6490	4.6510	1.4388
41	2017	23	22.5571	67.0102	873.1122	4.4041	1.6276
10	2017	23	25.0000	78.5816	961.0214	3.6857	0.9755
23	2017	23	17.9143	74.7347	776.0316	4.8673	1.7500
27	2017	23	22.8571	68.1429	857.9643	7.7459	2.0755
60	2017	23	23.9286	77.9184	936.3041	4.1765	1.1122
53	2017	23	21.4429	69.5102	853.1510	6.6704	2.3439
66	2017	23	22.8286	76.3367	895.5612	4.8735	1.9133
59	2017	23	22.2286	73.6122	885.3041	4.6224	2.1378
61	2017	23	25.5000	75.3061	959.1500	3.7327	0.8398
84	2017	23	25.5000	75.3061	959.1500	3.7327	0.8398
38	2017	23	22.2286	73.6122	885.3041	4.6224	2.1378
87	2017	23	23.7143	71.0204	897.7214	6.8000	2.0378
34	2017	23	22.2286	73.6122	885.3041	4.6224	2.1378
29	2017	23	24.5143	74.4388	937.4092	4.5173	1.3755
5	2017	23	21.3714	70.7041	831.5112	7.6071	1.6031
8	2017	23	21.4429	69.5102	853.1510	6.6704	2.3439
12	2017	23	21.3714	70.7041	831.5112	7.6071	1.6031
13	2017	23	27.1143	67.7449	941.7163	8.4929	1.1684
18	2017	23	25.4714	79.8878	960.9020	3.5684	0.9418
33	2017	23	22.9429	72.3061	900.1980	5.1704	1.6408
56	2017	23	26.5143	77.4490	973.6480	4.9827	0.8143
77	2017	23	23.2714	77.7755	916.6337	3.7143	1.4031
54	2017	23	21.3714	70.7041	831.5112	7.6071	1.6031
21	2017	23	22.9429	72.3061	900.1980	5.1704	1.6408
68	2017	23	24.6857	76.4592	966.5347	4.3684	1.1735
74	2017	23	25.5000	75.3061	959.1500	3.7327	0.8398
88	2017	23	21.9286	71.4490	873.6082	5.8714	2.4980
16	2017	23	23.7429	71.5510	918.9102	5.0867	1.5041
30	2017	23	22.8286	76.3367	895.5612	4.8735	1.9133
6	2017	23	24.6857	76.4592	966.5347	4.3684	1.1735
49	2017	23	24.5143	74.4388	937.4092	4.5173	1.3755
22	2017	23	21.9286	71.4490	873.6082	5.8714	2.4980
45	2017	23	21.3000	66.4082	816.9061	7.3878	2.0143
58	2017	23	24.5143	74.4388	937.4092	4.5173	1.3755
37	2017	23	24.6857	76.4592	966.5347	4.3684	1.1735
17	2017	23	22.5286	78.8571	900.2224	4.1367	2.5704
55	2017	23	22.5429	71.1531	876.1816	7.0663	1.8714
46	2017	23	23.7429	71.5510	918.9102	5.0867	1.5041
86	2017	23	21.8571	72.7653	865.0173	5.6010	1.5449
2	2017	23	21.8571	72.7653	865.0173	5.6010	1.5449
4	2017	23	22.9429	72.3061	900.1980	5.1704	1.6408
47	2017	23	26.6429	72.6939	955.7969	6.4776	0.6776
82	2017	23	21.9286	71.4490	873.6082	5.8714	2.4980
19	2017	23	26.3143	74.1837	957.5694	4.0051	1.1520
20	2017	23	21.4429	69.5102	853.1510	6.6704	2.3439
80	2017	23	21.9286	71.4490	873.6082	5.8714	2.4980
3	2017	23	27.1143	67.7449	941.7163	8.4929	1.1684

52	2017	23	22.5286	78.8571	900.2224	4.1367	2.5704
70	2017	23	23.9143	69.2449	908.3602	5.3357	1.7398
64	2017	23	17.9143	74.7347	776.0316	4.8673	1.7500
48	2017	23	23.2714	77.7755	916.6337	3.7143	1.4031
65	2017	23	22.5286	78.8571	900.2224	4.1367	2.5704
44	2017	23	23.9143	69.2449	908.3602	5.3357	1.7398
75	2017	23	17.9143	74.7347	776.0316	4.8673	1.7500
40	2017	23	24.7571	77.4694	942.7398	4.8327	1.2673
11	2017	23	22.5429	71.1531	876.1816	7.0663	1.8714
35	2017	23	23.9286	77.9184	936.3041	4.1765	1.1122
78	2017	23	23.7143	71.0204	897.7214	6.8000	2.0378
28	2017	23	24.3429	72.7959	926.6490	4.6510	1.4388
39	2017	23	22.5286	78.8571	900.2224	4.1367	2.5704
24	2017	23	24.5143	74.4388	937.4092	4.5173	1.3755
63	2017	23	24.7571	77.4694	942.7398	4.8327	1.2673
62	2017	23	22.5571	67.0102	873.1122	4.4041	1.6276
1	2017	23	21.9286	71.4490	873.6082	5.8714	2.4980
31	2017	24	17.5000	77.5204	845.0765	2.9796	1.0163
79	2017	24	22.1429	83.8673	966.2602	2.4796	1.1663
51	2017	24	20.6429	85.8469	935.9571	2.2224	1.1929
14	2017	24	20.5286	86.5306	895.0745	2.3071	1.8398
67	2017	24	19.7857	90.2245	899.7449	2.1041	2.4224
42	2017	24	18.8143	84.0714	873.1745	3.6551	2.3969
50	2017	24	19.6000	82.5408	899.7633	3.7092	1.5490
43	2017	24	18.8143	84.0714	873.1745	3.6551	2.3969
85	2017	24	19.7143	79.0714	907.9898	3.7765	1.5418
25	2017	24	24.1143	83.1939	972.9694	3.4378	0.6520
69	2017	24	21.7286	80.8776	936.9143	2.3806	1.4357
57	2017	24	18.4286	85.0918	884.9163	2.8408	2.0857
9	2017	24	18.6000	79.1327	852.7133	4.4908	2.5143
72	2017	24	20.0000	80.4898	875.6551	4.4949	1.9153
26	2017	24	20.6857	79.1939	865.2602	6.2337	1.6531
7	2017	24	19.9571	75.7551	857.4816	5.5429	2.0143
83	2017	24	24.3571	74.8980	941.0592	5.6490	1.1357
76	2017	24	20.2571	80.1837	918.4704	3.2878	1.4541
36	2017	24	21.2429	81.4388	926.1969	2.6265	1.4112
81	2017	24	20.6429	85.8469	935.9571	2.2224	1.1929
15	2017	24	19.7571	84.5408	916.0888	2.1041	1.5102
32	2017	24	18.8143	84.0714	873.1745	3.6551	2.3969
73	2017	24	22.6571	79.8061	958.6082	2.4571	0.9010
71	2017	24	21.2429	81.4388	926.1969	2.6265	1.4112
41	2017	24	18.8143	75.7857	872.7051	2.8276	1.6837
10	2017	24	21.7143	86.6429	960.6827	1.7663	1.0316
23	2017	24	14.7429	80.4184	775.6908	4.0898	1.7163
27	2017	24	19.9571	75.7551	857.4816	5.5429	2.0143
60	2017	24	20.6429	85.8469	935.9571	2.2224	1.1929
53	2017	24	18.6000	79.1327	852.7133	4.4908	2.5143
66	2017	24	20.5286	86.5306	895.0745	2.3071	1.8398
59	2017	24	18.4286	85.0918	884.9163	2.8408	2.0857
61	2017	24	22.6571	79.8061	958.6082	2.4571	0.9010

84	2017	24	22.6571	79.8061	958.6082	2.4571	0.9010
38	2017	24	18.4286	85.0918	884.9163	2.8408	2.0857
87	2017	24	20.9143	81.1020	897.2276	4.1918	2.0163
34	2017	24	18.4286	85.0918	884.9163	2.8408	2.0857
29	2017	24	21.7286	80.8776	936.9143	2.3806	1.4357
5	2017	24	18.1714	79.2449	831.0500	5.5408	1.4857
8	2017	24	18.6000	79.1327	852.7133	4.4908	2.5143
12	2017	24	18.1714	79.2449	831.0500	5.5408	1.4857
13	2017	24	24.3571	74.8980	941.0592	5.6490	1.1357
18	2017	24	21.8857	84.0306	960.5990	2.3857	1.1449
33	2017	24	19.6000	82.5408	899.7633	3.7092	1.5490
56	2017	24	24.1143	83.1939	972.9694	3.4378	0.6520
77	2017	24	19.7571	84.5408	916.0888	2.1041	1.5102
54	2017	24	18.1714	79.2449	831.0500	5.5408	1.4857
21	2017	24	19.6000	82.5408	899.7633	3.7092	1.5490
68	2017	24	22.1429	83.8673	966.2602	2.4796	1.1663
74	2017	24	22.6571	79.8061	958.6082	2.4571	0.9010
88	2017	24	18.8143	84.0714	873.1745	3.6551	2.3969
16	2017	24	20.2571	80.1837	918.4704	3.2878	1.4541
30	2017	24	20.5286	86.5306	895.0745	2.3071	1.8398
6	2017	24	22.1429	83.8673	966.2602	2.4796	1.1663
49	2017	24	21.7286	80.8776	936.9143	2.3806	1.4357
22	2017	24	18.8143	84.0714	873.1745	3.6551	2.3969
45	2017	24	18.3143	73.9286	816.4112	5.7520	1.9367
58	2017	24	21.7286	80.8776	936.9143	2.3806	1.4357
37	2017	24	22.1429	83.8673	966.2602	2.4796	1.1663
17	2017	24	19.7857	90.2245	899.7449	2.1041	2.4224
55	2017	24	20.0000	80.4898	875.6551	4.4949	1.9153
46	2017	24	20.2571	80.1837	918.4704	3.2878	1.4541
86	2017	24	19.0143	82.3265	864.5980	3.3184	1.4786
2	2017	24	19.0143	82.3265	864.5980	3.3184	1.4786
4	2017	24	19.6000	82.5408	899.7633	3.7092	1.5490
47	2017	24	24.5429	80.6224	955.1449	3.4276	0.6704
82	2017	24	18.8143	84.0714	873.1745	3.6551	2.3969
19	2017	24	24.5857	80.4796	956.8939	2.1745	1.1745
20	2017	24	18.6000	79.1327	852.7133	4.4908	2.5143
80	2017	24	18.8143	84.0714	873.1745	3.6551	2.3969
3	2017	24	24.3571	74.8980	941.0592	5.6490	1.1357
52	2017	24	19.7857	90.2245	899.7449	2.1041	2.4224
70	2017	24	19.7143	79.0714	907.9898	3.7765	1.5418
64	2017	24	14.7429	80.4184	775.6908	4.0898	1.7163
48	2017	24	19.7571	84.5408	916.0888	2.1041	1.5102
65	2017	24	19.7857	90.2245	899.7449	2.1041	2.4224
44	2017	24	19.7143	79.0714	907.9898	3.7765	1.5418
75	2017	24	14.7429	80.4184	775.6908	4.0898	1.7163
40	2017	24	21.3000	86.4388	942.3051	2.9663	1.4214
11	2017	24	20.0000	80.4898	875.6551	4.4949	1.9153
35	2017	24	20.6429	85.8469	935.9571	2.2224	1.1929
78	2017	24	20.9143	81.1020	897.2276	4.1918	2.0163
28	2017	24	21.2429	81.4388	926.1969	2.6265	1.4112

39	2017	24	19.7857	90.2245	899.7449	2.1041	2.4224
24	2017	24	21.7286	80.8776	936.9143	2.3806	1.4357
63	2017	24	21.3000	86.4388	942.3051	2.9663	1.4214
62	2017	24	18.8143	75.7857	872.7051	2.8276	1.6837
1	2017	24	18.8143	84.0714	873.1745	3.6551	2.3969
31	2017	25	19.1000	84.2959	845.2337	1.4327	0.8980
79	2017	25	24.6286	83.5918	966.3571	2.1888	0.9582
51	2017	25	23.5571	87.1224	936.1122	1.3551	1.1143
14	2017	25	21.9429	87.8061	895.1224	1.2112	1.5469
67	2017	25	21.8000	93.6122	899.8541	1.0561	2.1776
42	2017	25	20.4571	89.2041	873.3204	1.5541	2.0929
50	2017	25	21.1286	89.0714	900.0031	1.5316	1.4418
43	2017	25	20.4571	89.2041	873.3204	1.5541	2.0929
85	2017	25	21.4286	86.6531	908.2663	1.9418	1.3153
25	2017	25	25.7857	84.5714	973.2582	2.8898	0.4582
69	2017	25	23.3286	84.1122	937.1429	1.3316	1.1286
57	2017	25	20.4143	91.5408	885.0929	1.1602	1.8612
9	2017	25	20.1000	85.0714	852.8265	2.0286	2.1643
72	2017	25	20.8143	85.5408	875.8347	1.6663	1.4541
26	2017	25	21.1571	89.7653	865.5133	3.4398	1.2714
7	2017	25	20.9857	84.7653	857.6704	2.1469	1.5214
83	2017	25	25.1857	81.3367	941.5531	2.8071	1.0418
76	2017	25	21.8714	84.7245	918.7439	1.7224	1.2592
36	2017	25	23.8429	82.3469	926.3531	1.6102	1.3204
81	2017	25	23.5571	87.1224	936.1122	1.3551	1.1143
15	2017	25	21.8000	89.1122	916.2643	1.1612	1.4561
32	2017	25	20.4571	89.2041	873.3204	1.5541	2.0929
73	2017	25	24.0000	81.3061	958.8510	1.8418	0.8796
71	2017	25	23.8429	82.3469	926.3531	1.6102	1.3204
41	2017	25	20.5571	84.0510	872.8520	1.1765	1.3112
10	2017	25	24.9857	86.3367	960.7592	1.5173	0.8643
23	2017	25	16.0429	85.0816	775.7857	1.9745	1.3724
27	2017	25	20.9857	84.7653	857.6704	2.1469	1.5214
60	2017	25	23.5571	87.1224	936.1122	1.3551	1.1143
53	2017	25	20.1000	85.0714	852.8265	2.0286	2.1643
66	2017	25	21.9429	87.8061	895.1224	1.2112	1.5469
59	2017	25	20.4143	91.5408	885.0929	1.1602	1.8612
61	2017	25	24.0000	81.3061	958.8510	1.8418	0.8796
84	2017	25	24.0000	81.3061	958.8510	1.8418	0.8796
38	2017	25	20.4143	91.5408	885.0929	1.1602	1.8612
87	2017	25	22.2857	86.3061	897.4204	2.0561	1.6316
34	2017	25	20.4143	91.5408	885.0929	1.1602	1.8612
29	2017	25	23.3286	84.1122	937.1429	1.3316	1.1286
5	2017	25	19.3714	87.5510	831.2000	2.5990	1.2949
8	2017	25	20.1000	85.0714	852.8265	2.0286	2.1643
12	2017	25	19.3714	87.5510	831.2000	2.5990	1.2949
13	2017	25	25.1857	81.3367	941.5531	2.8071	1.0418
18	2017	25	24.0286	84.0408	960.7571	2.0561	1.0265
33	2017	25	21.1286	89.0714	900.0031	1.5316	1.4418
56	2017	25	25.7857	84.5714	973.2582	2.8898	0.4582

77	2017	25	21.8000	89.1122	916.2643	1.1612	1.4561
54	2017	25	19.3714	87.5510	831.2000	2.5990	1.2949
21	2017	25	21.1286	89.0714	900.0031	1.5316	1.4418
68	2017	25	24.6286	83.5918	966.3571	2.1888	0.9582
74	2017	25	24.0000	81.3061	958.8510	1.8418	0.8796
88	2017	25	20.4571	89.2041	873.3204	1.5541	2.0929
16	2017	25	21.8714	84.7245	918.7439	1.7224	1.2592
30	2017	25	21.9429	87.8061	895.1224	1.2112	1.5469
6	2017	25	24.6286	83.5918	966.3571	2.1888	0.9582
49	2017	25	23.3286	84.1122	937.1429	1.3316	1.1286
22	2017	25	20.4571	89.2041	873.3204	1.5541	2.0929
45	2017	25	19.4143	84.3673	816.5235	2.8102	1.4378
58	2017	25	23.3286	84.1122	937.1429	1.3316	1.1286
37	2017	25	24.6286	83.5918	966.3571	2.1888	0.9582
17	2017	25	21.8000	93.6122	899.8541	1.0561	2.1776
55	2017	25	20.8143	85.5408	875.8347	1.6663	1.4541
46	2017	25	21.8714	84.7245	918.7439	1.7224	1.2592
86	2017	25	20.6571	89.1735	864.7235	1.5133	1.1082
2	2017	25	20.6571	89.1735	864.7235	1.5133	1.1082
4	2017	25	21.1286	89.0714	900.0031	1.5316	1.4418
47	2017	25	25.1000	85.3673	955.5184	1.6153	0.5939
82	2017	25	20.4571	89.2041	873.3204	1.5541	2.0929
19	2017	25	25.5286	81.4184	957.0714	1.8582	1.0959
20	2017	25	20.1000	85.0714	852.8265	2.0286	2.1643
80	2017	25	20.4571	89.2041	873.3204	1.5541	2.0929
3	2017	25	25.1857	81.3367	941.5531	2.8071	1.0418
52	2017	25	21.8000	93.6122	899.8541	1.0561	2.1776
70	2017	25	21.4286	86.6531	908.2663	1.9418	1.3153
64	2017	25	16.0429	85.0816	775.7857	1.9745	1.3724
48	2017	25	21.8000	89.1122	916.2643	1.1612	1.4561
65	2017	25	21.8000	93.6122	899.8541	1.0561	2.1776
44	2017	25	21.4286	86.6531	908.2663	1.9418	1.3153
75	2017	25	16.0429	85.0816	775.7857	1.9745	1.3724
40	2017	25	24.5714	86.5918	942.3765	2.8622	1.4214
11	2017	25	20.8143	85.5408	875.8347	1.6663	1.4541
35	2017	25	23.5571	87.1224	936.1122	1.3551	1.1143
78	2017	25	22.2857	86.3061	897.4204	2.0561	1.6316
28	2017	25	23.8429	82.3469	926.3531	1.6102	1.3204
39	2017	25	21.8000	93.6122	899.8541	1.0561	2.1776
24	2017	25	23.3286	84.1122	937.1429	1.3316	1.1286
63	2017	25	24.5714	86.5918	942.3765	2.8622	1.4214
62	2017	25	20.5571	84.0510	872.8520	1.1765	1.3112
1	2017	25	20.4571	89.2041	873.3204	1.5541	2.0929
31	2017	26	18.6286	88.3469	844.9520	0.9908	0.7898
79	2017	26	23.0000	85.6020	965.6255	2.0990	0.8980
51	2017	26	21.3429	88.6939	935.4847	1.3378	0.8327
14	2017	26	20.6143	89.6327	894.7214	0.8010	1.5082
67	2017	26	20.0714	94.9082	899.4092	0.6776	2.2296
42	2017	26	19.3429	92.0714	872.9480	0.9133	1.9082
50	2017	26	20.7143	90.3163	899.6582	1.2510	1.2857

43	2017	26	19.3429	92.0714	872.9480	0.9133	1.9082
85	2017	26	20.8857	89.3367	907.9847	1.3510	1.1929
25	2017	26	23.5143	87.8980	972.8388	1.8224	0.2633
69	2017	26	21.5857	87.7245	936.7071	1.0235	0.8724
57	2017	26	18.9143	94.4184	884.7622	0.6786	1.5673
9	2017	26	19.0000	87.4082	852.4949	1.0020	1.7531
72	2017	26	19.6429	87.9082	875.5551	0.6122	1.2082
26	2017	26	19.8286	93.9082	865.3388	2.4531	1.1327
7	2017	26	19.5571	88.3571	857.3776	0.8796	1.3061
83	2017	26	23.4286	84.8163	941.4439	1.4878	1.0122
76	2017	26	20.7429	87.9184	918.3612	1.5020	1.0827
36	2017	26	21.2857	84.8673	925.8776	0.9867	1.1214
81	2017	26	21.3429	88.6939	935.4847	1.3378	0.8327
15	2017	26	21.2429	90.2857	915.8612	1.6571	1.4255
32	2017	26	19.3429	92.0714	872.9480	0.9133	1.9082
73	2017	26	22.6714	86.0102	958.4276	1.6776	0.6327
71	2017	26	21.2857	84.8673	925.8776	0.9867	1.1214
41	2017	26	19.3714	87.8878	872.5051	0.5531	0.9827
10	2017	26	22.2429	87.6122	960.1092	1.0071	0.5837
23	2017	26	15.3714	87.2551	775.5510	1.2755	1.1714
27	2017	26	19.5571	88.3571	857.3776	0.8796	1.3061
60	2017	26	21.3429	88.6939	935.4847	1.3378	0.8327
53	2017	26	19.0000	87.4082	852.4949	1.0020	1.7531
66	2017	26	20.6143	89.6327	894.7214	0.8010	1.5082
59	2017	26	18.9143	94.4184	884.7622	0.6786	1.5673
61	2017	26	22.6714	86.0102	958.4276	1.6776	0.6327
84	2017	26	22.6714	86.0102	958.4276	1.6776	0.6327
38	2017	26	18.9143	94.4184	884.7622	0.6786	1.5673
87	2017	26	20.8286	89.6633	897.1051	1.1235	1.3735
34	2017	26	18.9143	94.4184	884.7622	0.6786	1.5673
29	2017	26	21.5857	87.7245	936.7071	1.0235	0.8724
5	2017	26	18.0571	90.2041	830.9367	1.7398	1.1000
8	2017	26	19.0000	87.4082	852.4949	1.0020	1.7531
12	2017	26	18.0571	90.2041	830.9367	1.7398	1.1000
13	2017	26	23.4286	84.8163	941.4439	1.4878	1.0122
18	2017	26	23.0857	87.2347	960.0286	2.2398	0.8765
33	2017	26	20.7143	90.3163	899.6582	1.2510	1.2857
56	2017	26	23.5143	87.8980	972.8388	1.8224	0.2633
77	2017	26	21.2429	90.2857	915.8612	1.6571	1.4255
54	2017	26	18.0571	90.2041	830.9367	1.7398	1.1000
21	2017	26	20.7143	90.3163	899.6582	1.2510	1.2857
68	2017	26	23.0000	85.6020	965.6255	2.0990	0.8980
74	2017	26	22.6714	86.0102	958.4276	1.6776	0.6327
88	2017	26	19.3429	92.0714	872.9480	0.9133	1.9082
16	2017	26	20.7429	87.9184	918.3612	1.5020	1.0827
30	2017	26	20.6143	89.6327	894.7214	0.8010	1.5082
6	2017	26	23.0000	85.6020	965.6255	2.0990	0.8980
49	2017	26	21.5857	87.7245	936.7071	1.0235	0.8724
22	2017	26	19.3429	92.0714	872.9480	0.9133	1.9082
45	2017	26	17.8429	88.1531	816.2969	2.0010	1.2816

58	2017	26	21.5857	87.7245	936.7071	1.0235	0.8724
37	2017	26	23.0000	85.6020	965.6255	2.0990	0.8980
17	2017	26	20.0714	94.9082	899.4092	0.6776	2.2296
55	2017	26	19.6429	87.9082	875.5551	0.6122	1.2082
46	2017	26	20.7429	87.9184	918.3612	1.5020	1.0827
86	2017	26	19.4286	92.1531	864.4051	1.0786	0.8010
2	2017	26	19.4286	92.1531	864.4051	1.0786	0.8010
4	2017	26	20.7143	90.3163	899.6582	1.2510	1.2857
47	2017	26	24.1429	87.9184	955.3173	1.3357	0.4551
82	2017	26	19.3429	92.0714	872.9480	0.9133	1.9082
19	2017	26	24.2143	85.0816	956.7551	1.2020	0.8541
20	2017	26	19.0000	87.4082	852.4949	1.0020	1.7531
80	2017	26	19.3429	92.0714	872.9480	0.9133	1.9082
3	2017	26	23.4286	84.8163	941.4439	1.4878	1.0122
52	2017	26	20.0714	94.9082	899.4092	0.6776	2.2296
70	2017	26	20.8857	89.3367	907.9847	1.3510	1.1929
64	2017	26	15.3714	87.2551	775.5510	1.2755	1.1714
48	2017	26	21.2429	90.2857	915.8612	1.6571	1.4255
65	2017	26	20.0714	94.9082	899.4092	0.6776	2.2296
44	2017	26	20.8857	89.3367	907.9847	1.3510	1.1929
75	2017	26	15.3714	87.2551	775.5510	1.2755	1.1714
40	2017	26	21.7000	87.9490	941.8561	1.5612	1.3173
11	2017	26	19.6429	87.9082	875.5551	0.6122	1.2082
35	2017	26	21.3429	88.6939	935.4847	1.3378	0.8327
78	2017	26	20.8286	89.6633	897.1051	1.1235	1.3735
28	2017	26	21.2857	84.8673	925.8776	0.9867	1.1214
39	2017	26	20.0714	94.9082	899.4092	0.6776	2.2296
24	2017	26	21.5857	87.7245	936.7071	1.0235	0.8724
63	2017	26	21.7000	87.9490	941.8561	1.5612	1.3173
62	2017	26	19.3714	87.8878	872.5051	0.5531	0.9827
1	2017	26	19.3429	92.0714	872.9480	0.9133	1.9082
31	2017	27	21.7143	85.4694	844.7827	1.9582	0.8418
79	2017	27	27.9286	83.9082	965.2827	3.9388	0.8612
51	2017	27	25.5143	86.8571	935.2949	3.3602	0.8714
14	2017	27	22.9286	89.8878	894.7602	1.9806	1.8122
67	2017	27	23.4857	91.8980	899.3316	2.0449	2.4449
42	2017	27	22.5143	88.9592	872.8622	2.6520	2.0184
50	2017	27	24.2857	85.1735	899.2786	3.1347	1.4041
43	2017	27	22.5143	88.9592	872.8622	2.6520	2.0184
85	2017	27	24.7286	84.0612	907.5316	3.0704	1.3531
25	2017	27	26.9000	88.2245	972.5510	2.5582	0.3469
69	2017	27	25.7714	85.3469	936.3939	2.2929	1.0112
57	2017	27	23.2429	90.1224	884.5459	2.1398	1.8265
9	2017	27	21.3714	86.1327	852.4959	1.5898	1.7612
72	2017	27	22.2429	87.1429	875.5622	1.7367	1.3204
26	2017	27	21.7714	94.7755	865.4224	1.4418	1.2806
7	2017	27	21.7429	88.2755	857.4000	1.2102	1.3735
83	2017	27	26.5857	84.2041	941.3245	2.3602	1.0214
76	2017	27	25.1286	85.1224	918.0061	3.5449	1.2082
36	2017	27	25.8857	83.3265	925.6959	2.9745	1.2622

81	2017	27	25.5143	86.8571	935.2949	3.3602	0.8714
15	2017	27	24.8571	85.8061	915.4378	2.9061	1.5296
32	2017	27	22.5143	88.9592	872.8622	2.6520	2.0184
73	2017	27	27.0714	84.2653	957.9633	3.4173	0.6602
71	2017	27	25.8857	83.3265	925.6959	2.9745	1.2622
41	2017	27	23.0714	84.6122	872.3163	1.6878	1.2245
10	2017	27	26.6286	86.6735	959.9194	2.1133	0.5224
23	2017	27	17.7429	86.8980	775.6592	2.0582	1.1306
27	2017	27	21.7429	88.2755	857.4000	1.2102	1.3735
60	2017	27	25.5143	86.8571	935.2949	3.3602	0.8714
53	2017	27	21.3714	86.1327	852.4959	1.5898	1.7612
66	2017	27	22.9286	89.8878	894.7602	1.9806	1.8122
59	2017	27	23.2429	90.1224	884.5459	2.1398	1.8265
61	2017	27	27.0714	84.2653	957.9633	3.4173	0.6602
84	2017	27	27.0714	84.2653	957.9633	3.4173	0.6602
38	2017	27	23.2429	90.1224	884.5459	2.1398	1.8265
87	2017	27	23.9714	88.1224	896.9888	2.0827	1.4235
34	2017	27	23.2429	90.1224	884.5459	2.1398	1.8265
29	2017	27	25.7714	85.3469	936.3939	2.2929	1.0112
5	2017	27	20.1857	89.9592	831.0316	2.1571	1.2561
8	2017	27	21.3714	86.1327	852.4959	1.5898	1.7612
12	2017	27	20.1857	89.9592	831.0316	2.1571	1.2561
13	2017	27	26.5857	84.2041	941.3245	2.3602	1.0214
18	2017	27	27.5429	85.1531	959.6276	4.1714	0.9653
33	2017	27	24.2857	85.1735	899.2786	3.1347	1.4041
56	2017	27	26.9000	88.2245	972.5510	2.5582	0.3469
77	2017	27	24.8571	85.8061	915.4378	2.9061	1.5296
54	2017	27	20.1857	89.9592	831.0316	2.1571	1.2561
21	2017	27	24.2857	85.1735	899.2786	3.1347	1.4041
68	2017	27	27.9286	83.9082	965.2827	3.9388	0.8612
74	2017	27	27.0714	84.2653	957.9633	3.4173	0.6602
88	2017	27	22.5143	88.9592	872.8622	2.6520	2.0184
16	2017	27	25.1286	85.1224	918.0061	3.5449	1.2082
30	2017	27	22.9286	89.8878	894.7602	1.9806	1.8122
6	2017	27	27.9286	83.9082	965.2827	3.9388	0.8612
49	2017	27	25.7714	85.3469	936.3939	2.2929	1.0112
22	2017	27	22.5143	88.9592	872.8622	2.6520	2.0184
45	2017	27	19.5571	89.2245	816.4704	1.4878	1.4551
58	2017	27	25.7714	85.3469	936.3939	2.2929	1.0112
37	2017	27	27.9286	83.9082	965.2827	3.9388	0.8612
17	2017	27	23.4857	91.8980	899.3316	2.0449	2.4449
55	2017	27	22.2429	87.1429	875.5622	1.7367	1.3204
46	2017	27	25.1286	85.1224	918.0061	3.5449	1.2082
86	2017	27	21.9429	90.8265	864.3031	1.7418	0.9602
2	2017	27	21.9429	90.8265	864.3031	1.7418	0.9602
4	2017	27	24.2857	85.1735	899.2786	3.1347	1.4041
47	2017	27	26.8000	86.2959	955.1143	2.9582	0.4531
82	2017	27	22.5143	88.9592	872.8622	2.6520	2.0184
19	2017	27	26.7857	85.1531	956.6724	1.9959	0.7765
20	2017	27	21.3714	86.1327	852.4959	1.5898	1.7612

80	2017	27	22.5143	88.9592	872.8622	2.6520	2.0184
3	2017	27	26.5857	84.2041	941.3245	2.3602	1.0214
52	2017	27	23.4857	91.8980	899.3316	2.0449	2.4449
70	2017	27	24.7286	84.0612	907.5316	3.0704	1.3531
64	2017	27	17.7429	86.8980	775.6592	2.0582	1.1306
48	2017	27	24.8571	85.8061	915.4378	2.9061	1.5296
65	2017	27	23.4857	91.8980	899.3316	2.0449	2.4449
44	2017	27	24.7286	84.0612	907.5316	3.0704	1.3531
75	2017	27	17.7429	86.8980	775.6592	2.0582	1.1306
40	2017	27	26.0429	87.3673	941.7592	3.0806	1.4745
11	2017	27	22.2429	87.1429	875.5622	1.7367	1.3204
35	2017	27	25.5143	86.8571	935.2949	3.3602	0.8714
78	2017	27	23.9714	88.1224	896.9888	2.0827	1.4235
28	2017	27	25.8857	83.3265	925.6959	2.9745	1.2622
39	2017	27	23.4857	91.8980	899.3316	2.0449	2.4449
24	2017	27	25.7714	85.3469	936.3939	2.2929	1.0112
63	2017	27	26.0429	87.3673	941.7592	3.0806	1.4745
62	2017	27	23.0714	84.6122	872.3163	1.6878	1.2245
1	2017	27	22.5143	88.9592	872.8622	2.6520	2.0184
31	2017	28	22.1714	77.9286	844.8510	3.4092	1.0071
79	2017	28	28.5571	76.2959	965.0367	5.7980	1.0653
51	2017	28	25.7143	81.4898	935.4714	4.8643	1.1949
14	2017	28	22.5286	88.4184	895.3378	2.3235	1.9898
67	2017	28	23.0143	89.5510	899.7408	2.5684	2.5388
42	2017	28	22.6714	84.1429	873.2153	3.8286	2.0551
50	2017	28	25.3571	77.7347	899.0551	4.8969	1.7184
43	2017	28	22.6714	84.1429	873.2153	3.8286	2.0551
85	2017	28	26.1714	75.4286	907.1449	4.7878	1.6918
25	2017	28	26.5714	85.5204	972.9735	3.6214	0.5184
69	2017	28	26.2429	79.3571	936.3878	3.1173	1.1929
57	2017	28	23.9714	82.2653	884.5684	3.6429	2.4194
9	2017	28	21.5714	83.4898	852.9122	2.9806	1.9122
72	2017	28	21.6429	85.9388	876.0173	2.3296	1.2245
26	2017	28	21.9143	92.4490	865.8622	0.9153	1.4286
7	2017	28	21.5429	85.5816	857.8827	1.9776	1.5031
83	2017	28	25.8857	82.1735	941.6510	3.3520	1.0929
76	2017	28	25.5857	78.4796	917.8337	5.0000	1.3878
36	2017	28	25.4286	77.7041	925.9551	4.4612	1.4867
81	2017	28	25.7143	81.4898	935.4714	4.8643	1.1949
15	2017	28	25.8857	77.8061	915.0735	4.4796	1.7143
32	2017	28	22.6714	84.1429	873.2153	3.8286	2.0551
73	2017	28	28.1429	76.6429	957.5378	5.0949	1.1296
71	2017	28	25.4286	77.7041	925.9551	4.4612	1.4867
41	2017	28	23.2857	78.6633	872.4337	2.4949	1.5480
10	2017	28	26.2857	82.5204	960.1173	3.7102	0.7010
23	2017	28	17.8714	80.4286	776.2306	2.8347	1.2418
27	2017	28	21.5429	85.5816	857.8827	1.9776	1.5031
60	2017	28	25.7143	81.4898	935.4714	4.8643	1.1949
53	2017	28	21.5714	83.4898	852.9122	2.9806	1.9122
66	2017	28	22.5286	88.4184	895.3378	2.3235	1.9898

59	2017	28	23.9714	82.2653	884.5684	3.6429	2.4194
61	2017	28	28.1429	76.6429	957.5378	5.0949	1.1296
84	2017	28	28.1429	76.6429	957.5378	5.0949	1.1296
38	2017	28	23.9714	82.2653	884.5684	3.6429	2.4194
87	2017	28	23.7429	85.1735	897.3724	3.5327	1.4041
34	2017	28	23.9714	82.2653	884.5684	3.6429	2.4194
29	2017	28	26.2429	79.3571	936.3878	3.1173	1.1929
5	2017	28	19.9857	86.9796	831.5418	2.7194	1.3561
8	2017	28	21.5714	83.4898	852.9122	2.9806	1.9122
12	2017	28	19.9857	86.9796	831.5418	2.7194	1.3561
13	2017	28	25.8857	82.1735	941.6510	3.3520	1.0929
18	2017	28	28.1429	77.7755	959.2867	5.5255	1.1255
33	2017	28	25.3571	77.7347	899.0551	4.8969	1.7184
56	2017	28	26.5714	85.5204	972.9735	3.6214	0.5184
77	2017	28	25.8857	77.8061	915.0735	4.4796	1.7143
54	2017	28	19.9857	86.9796	831.5418	2.7194	1.3561
21	2017	28	25.3571	77.7347	899.0551	4.8969	1.7184
68	2017	28	28.5571	76.2959	965.0367	5.7980	1.0653
74	2017	28	28.1429	76.6429	957.5378	5.0949	1.1296
88	2017	28	22.6714	84.1429	873.2153	3.8286	2.0551
16	2017	28	25.5857	78.4796	917.8337	5.0000	1.3878
30	2017	28	22.5286	88.4184	895.3378	2.3235	1.9898
6	2017	28	28.5571	76.2959	965.0367	5.7980	1.0653
49	2017	28	26.2429	79.3571	936.3878	3.1173	1.1929
22	2017	28	22.6714	84.1429	873.2153	3.8286	2.0551
45	2017	28	19.2000	86.7551	817.0571	1.6245	1.3367
58	2017	28	26.2429	79.3571	936.3878	3.1173	1.1929
37	2017	28	28.5571	76.2959	965.0367	5.7980	1.0653
17	2017	28	23.0143	89.5510	899.7408	2.5684	2.5388
55	2017	28	21.6429	85.9388	876.0173	2.3296	1.2245
46	2017	28	25.5857	78.4796	917.8337	5.0000	1.3878
86	2017	28	22.1143	87.3878	864.5398	2.6735	1.3684
2	2017	28	22.1143	87.3878	864.5398	2.6735	1.3684
4	2017	28	25.3571	77.7347	899.0551	4.8969	1.7184
47	2017	28	25.8714	84.4082	955.5194	3.4592	0.5714
82	2017	28	22.6714	84.1429	873.2153	3.8286	2.0551
19	2017	28	25.7714	84.0816	957.1878	2.4990	0.8469
20	2017	28	21.5714	83.4898	852.9122	2.9806	1.9122
80	2017	28	22.6714	84.1429	873.2153	3.8286	2.0551
3	2017	28	25.8857	82.1735	941.6510	3.3520	1.0929
52	2017	28	23.0143	89.5510	899.7408	2.5684	2.5388
70	2017	28	26.1714	75.4286	907.1449	4.7878	1.6918
64	2017	28	17.8714	80.4286	776.2306	2.8347	1.2418
48	2017	28	25.8857	77.8061	915.0735	4.4796	1.7143
65	2017	28	23.0143	89.5510	899.7408	2.5684	2.5388
44	2017	28	26.1714	75.4286	907.1449	4.7878	1.6918
75	2017	28	17.8714	80.4286	776.2306	2.8347	1.2418
40	2017	28	25.8286	83.0102	942.1663	4.9980	1.5469
11	2017	28	21.6429	85.9388	876.0173	2.3296	1.2245
35	2017	28	25.7143	81.4898	935.4714	4.8643	1.1949

78	2017	28	23.7429	85.1735	897.3724	3.5327	1.4041
28	2017	28	25.4286	77.7041	925.9551	4.4612	1.4867
39	2017	28	23.0143	89.5510	899.7408	2.5684	2.5388
24	2017	28	26.2429	79.3571	936.3878	3.1173	1.1929
63	2017	28	25.8286	83.0102	942.1663	4.9980	1.5469
62	2017	28	23.2857	78.6633	872.4337	2.4949	1.5480
1	2017	28	22.6714	84.1429	873.2153	3.8286	2.0551
31	2017	29	22.3429	75.7143	845.8908	3.7245	1.0939
79	2017	29	30.0714	70.6837	966.1490	7.0327	1.3633
51	2017	29	27.2571	77.0510	936.8214	6.6571	1.4224
14	2017	29	23.4857	87.0000	896.9173	2.6837	1.9796
67	2017	29	24.1857	88.8061	901.2255	2.9898	2.6561
42	2017	29	23.5000	81.7755	874.6214	4.5949	2.0898
50	2017	29	25.8571	75.0612	900.1265	5.4102	2.0041
43	2017	29	23.5000	81.7755	874.6214	4.5949	2.0898
85	2017	29	26.4857	70.3878	908.0857	5.5878	2.0480
25	2017	29	27.9143	81.9592	974.4765	4.5245	0.6265
69	2017	29	27.8429	74.8265	937.6235	5.2684	1.3469
57	2017	29	24.6429	77.1327	885.7980	5.3265	2.9122
9	2017	29	22.0000	81.2245	854.3408	4.7112	2.2194
72	2017	29	22.1143	85.2041	877.4939	2.6357	1.2541
26	2017	29	21.2714	90.3571	867.2673	2.0571	1.5173
7	2017	29	22.0429	83.3571	859.2867	2.9184	1.6867
83	2017	29	26.4571	79.3673	943.2031	4.4857	1.1592
76	2017	29	26.9571	75.0102	918.9816	5.8531	1.3143
36	2017	29	26.7429	75.1224	927.3633	5.4918	1.5704
81	2017	29	27.2571	77.0510	936.8214	6.6571	1.4224
15	2017	29	26.8714	74.0612	916.1245	6.0347	1.8327
32	2017	29	23.5000	81.7755	874.6214	4.5949	2.0898
73	2017	29	30.0857	69.9694	958.5776	5.9163	1.4735
71	2017	29	26.7429	75.1224	927.3633	5.4918	1.5704
41	2017	29	23.6714	76.2857	873.6786	3.5806	1.7776
10	2017	29	27.8857	79.2041	961.4398	5.4969	0.9765
23	2017	29	18.0000	80.1429	777.3837	3.0082	1.4184
27	2017	29	22.0429	83.3571	859.2867	2.9184	1.6867
60	2017	29	27.2571	77.0510	936.8214	6.6571	1.4224
53	2017	29	22.0000	81.2245	854.3408	4.7112	2.2194
66	2017	29	23.4857	87.0000	896.9173	2.6837	1.9796
59	2017	29	24.6429	77.1327	885.7980	5.3265	2.9122
61	2017	29	30.0857	69.9694	958.5776	5.9163	1.4735
84	2017	29	30.0857	69.9694	958.5776	5.9163	1.4735
38	2017	29	24.6429	77.1327	885.7980	5.3265	2.9122
87	2017	29	24.8429	83.0816	898.8827	5.0306	1.2847
34	2017	29	24.6429	77.1327	885.7980	5.3265	2.9122
29	2017	29	27.8429	74.8265	937.6235	5.2684	1.3469
5	2017	29	20.2143	86.1633	832.8949	3.0990	1.1684
8	2017	29	22.0000	81.2245	854.3408	4.7112	2.2194
12	2017	29	20.2143	86.1633	832.8949	3.0990	1.1684
13	2017	29	26.4571	79.3673	943.2031	4.4857	1.1592
18	2017	29	29.1571	74.3061	960.4051	6.2214	1.3000

33	2017	29	25.8571	75.0612	900.1265	5.4102	2.0041
56	2017	29	27.9143	81.9592	974.4765	4.5245	0.6265
77	2017	29	26.8714	74.0612	916.1245	6.0347	1.8327
54	2017	29	20.2143	86.1633	832.8949	3.0990	1.1684
21	2017	29	25.8571	75.0612	900.1265	5.4102	2.0041
68	2017	29	30.0714	70.6837	966.1490	7.0327	1.3633
74	2017	29	30.0857	69.9694	958.5776	5.9163	1.4735
88	2017	29	23.5000	81.7755	874.6214	4.5949	2.0898
16	2017	29	26.9571	75.0102	918.9816	5.8531	1.3143
30	2017	29	23.4857	87.0000	896.9173	2.6837	1.9796
6	2017	29	30.0714	70.6837	966.1490	7.0327	1.3633
49	2017	29	27.8429	74.8265	937.6235	5.2684	1.3469
22	2017	29	23.5000	81.7755	874.6214	4.5949	2.0898
45	2017	29	19.2857	86.4592	818.3949	2.1347	1.2378
58	2017	29	27.8429	74.8265	937.6235	5.2684	1.3469
37	2017	29	30.0714	70.6837	966.1490	7.0327	1.3633
17	2017	29	24.1857	88.8061	901.2255	2.9898	2.6561
55	2017	29	22.1143	85.2041	877.4939	2.6357	1.2541
46	2017	29	26.9571	75.0102	918.9816	5.8531	1.3143
86	2017	29	22.8571	84.7857	865.8306	3.9520	1.8010
2	2017	29	22.8571	84.7857	865.8306	3.9520	1.8010
4	2017	29	25.8571	75.0612	900.1265	5.4102	2.0041
47	2017	29	26.7857	83.8673	957.1602	3.7071	0.6143
82	2017	29	23.5000	81.7755	874.6214	4.5949	2.0898
19	2017	29	26.7143	84.1429	958.8357	3.1000	0.8408
20	2017	29	22.0000	81.2245	854.3408	4.7112	2.2194
80	2017	29	23.5000	81.7755	874.6214	4.5949	2.0898
3	2017	29	26.4571	79.3673	943.2031	4.4857	1.1592
52	2017	29	24.1857	88.8061	901.2255	2.9898	2.6561
70	2017	29	26.4857	70.3878	908.0857	5.5878	2.0480
64	2017	29	18.0000	80.1429	777.3837	3.0082	1.4184
48	2017	29	26.8714	74.0612	916.1245	6.0347	1.8327
65	2017	29	24.1857	88.8061	901.2255	2.9898	2.6561
44	2017	29	26.4857	70.3878	908.0857	5.5878	2.0480
75	2017	29	18.0000	80.1429	777.3837	3.0082	1.4184
40	2017	29	26.9571	77.8367	943.6510	6.7561	1.5776
11	2017	29	22.1143	85.2041	877.4939	2.6357	1.2541
35	2017	29	27.2571	77.0510	936.8214	6.6571	1.4224
78	2017	29	24.8429	83.0816	898.8827	5.0306	1.2847
28	2017	29	26.7429	75.1224	927.3633	5.4918	1.5704
39	2017	29	24.1857	88.8061	901.2255	2.9898	2.6561
24	2017	29	27.8429	74.8265	937.6235	5.2684	1.3469
63	2017	29	26.9571	77.8367	943.6510	6.7561	1.5776
62	2017	29	23.6714	76.2857	873.6786	3.5806	1.7776
1	2017	29	23.5000	81.7755	874.6214	4.5949	2.0898
31	2017	30	24.1000	75.0714	846.0959	5.5316	1.0724
79	2017	30	31.7000	64.7347	966.0867	9.6714	1.4520
51	2017	30	28.5143	71.6122	936.8990	9.0888	1.4061
14	2017	30	25.6143	82.8163	897.0184	5.2082	1.6296
67	2017	30	25.9000	83.1224	901.3480	5.9520	2.3184

42	2017	30	25.5571	75.7347	874.8561	6.6755	2.1867
50	2017	30	27.6857	71.5102	900.3541	7.8316	2.0418
43	2017	30	25.5571	75.7347	874.8561	6.6755	2.1867
85	2017	30	29.3000	65.0000	908.1643	8.4837	2.1622
25	2017	30	29.3429	76.2755	974.2245	6.9847	0.6816
69	2017	30	28.1571	70.2551	937.6582	8.7949	1.4184
57	2017	30	26.2571	73.6939	886.0429	7.8908	2.7776
9	2017	30	24.1143	77.2959	854.6571	6.6765	2.2337
72	2017	30	23.6857	82.3673	877.7571	4.9908	1.2796
26	2017	30	24.1429	88.2245	867.6265	4.4765	1.4939
7	2017	30	23.2143	81.3980	859.5990	4.8816	1.5102
83	2017	30	27.6143	75.4184	943.3449	6.3929	1.1867
76	2017	30	28.2143	69.7653	919.1388	8.6306	1.2878
36	2017	30	27.8571	71.1327	927.3816	7.9439	1.5969
81	2017	30	28.5143	71.6122	936.8990	9.0888	1.4061
15	2017	30	28.0857	70.5102	916.3561	8.3755	1.8633
32	2017	30	25.5571	75.7347	874.8561	6.6755	2.1867
73	2017	30	30.7286	63.6224	958.5694	7.9204	1.4367
71	2017	30	27.8571	71.1327	927.3816	7.9439	1.5969
41	2017	30	25.6286	73.4286	873.9265	6.4857	1.9173
10	2017	30	28.7286	74.1531	961.2571	8.3694	1.1112
23	2017	30	20.0857	82.0612	777.6367	4.7673	1.5082
27	2017	30	23.2143	81.3980	859.5990	4.8816	1.5102
60	2017	30	28.5143	71.6122	936.8990	9.0888	1.4061
53	2017	30	24.1143	77.2959	854.6571	6.6765	2.2337
66	2017	30	25.6143	82.8163	897.0184	5.2082	1.6296
59	2017	30	26.2571	73.6939	886.0429	7.8908	2.7776
61	2017	30	30.7286	63.6224	958.5694	7.9204	1.4367
84	2017	30	30.7286	63.6224	958.5694	7.9204	1.4367
38	2017	30	26.2571	73.6939	886.0429	7.8908	2.7776
87	2017	30	26.1143	77.6122	899.0194	7.2214	1.2092
34	2017	30	26.2571	73.6939	886.0429	7.8908	2.7776
29	2017	30	28.1571	70.2551	937.6582	8.7949	1.4184
5	2017	30	21.3571	85.6735	833.2480	4.5745	0.9357
8	2017	30	24.1143	77.2959	854.6571	6.6765	2.2337
12	2017	30	21.3571	85.6735	833.2480	4.5745	0.9357
13	2017	30	27.6143	75.4184	943.3449	6.3929	1.1867
18	2017	30	30.9857	70.2959	960.4592	8.5133	1.2602
33	2017	30	27.6857	71.5102	900.3541	7.8316	2.0418
56	2017	30	29.3429	76.2755	974.2245	6.9847	0.6816
77	2017	30	28.0857	70.5102	916.3561	8.3755	1.8633
54	2017	30	21.3571	85.6735	833.2480	4.5745	0.9357
21	2017	30	27.6857	71.5102	900.3541	7.8316	2.0418
68	2017	30	31.7000	64.7347	966.0867	9.6714	1.4520
74	2017	30	30.7286	63.6224	958.5694	7.9204	1.4367
88	2017	30	25.5571	75.7347	874.8561	6.6755	2.1867
16	2017	30	28.2143	69.7653	919.1388	8.6306	1.2878
30	2017	30	25.6143	82.8163	897.0184	5.2082	1.6296
6	2017	30	31.7000	64.7347	966.0867	9.6714	1.4520
49	2017	30	28.1571	70.2551	937.6582	8.7949	1.4184

22	2017	30	25.5571	75.7347	874.8561	6.6755	2.1867
45	2017	30	20.7286	85.4796	818.7918	4.0898	1.2837
58	2017	30	28.1571	70.2551	937.6582	8.7949	1.4184
37	2017	30	31.7000	64.7347	966.0867	9.6714	1.4520
17	2017	30	25.9000	83.1224	901.3480	5.9520	2.3184
55	2017	30	23.6857	82.3673	877.7571	4.9908	1.2796
46	2017	30	28.2143	69.7653	919.1388	8.6306	1.2878
86	2017	30	24.9286	79.8980	866.0888	6.6663	1.9224
2	2017	30	24.9286	79.8980	866.0888	6.6663	1.9224
4	2017	30	27.6857	71.5102	900.3541	7.8316	2.0418
47	2017	30	28.7286	79.4388	957.1143	5.7184	0.5092
82	2017	30	25.5571	75.7347	874.8561	6.6755	2.1867
19	2017	30	28.8286	79.8673	958.6878	5.4571	0.9224
20	2017	30	24.1143	77.2959	854.6571	6.6765	2.2337
80	2017	30	25.5571	75.7347	874.8561	6.6755	2.1867
3	2017	30	27.6143	75.4184	943.3449	6.3929	1.1867
52	2017	30	25.9000	83.1224	901.3480	5.9520	2.3184
70	2017	30	29.3000	65.0000	908.1643	8.4837	2.1622
64	2017	30	20.0857	82.0612	777.6367	4.7673	1.5082
48	2017	30	28.0857	70.5102	916.3561	8.3755	1.8633
65	2017	30	25.9000	83.1224	901.3480	5.9520	2.3184
44	2017	30	29.3000	65.0000	908.1643	8.4837	2.1622
75	2017	30	20.0857	82.0612	777.6367	4.7673	1.5082
40	2017	30	28.3429	72.0408	943.5337	9.2847	1.4878
11	2017	30	23.6857	82.3673	877.7571	4.9908	1.2796
35	2017	30	28.5143	71.6122	936.8990	9.0888	1.4061
78	2017	30	26.1143	77.6122	899.0194	7.2214	1.2092
28	2017	30	27.8571	71.1327	927.3816	7.9439	1.5969
39	2017	30	25.9000	83.1224	901.3480	5.9520	2.3184
24	2017	30	28.1571	70.2551	937.6582	8.7949	1.4184
63	2017	30	28.3429	72.0408	943.5337	9.2847	1.4878
62	2017	30	25.6286	73.4286	873.9265	6.4857	1.9173
1	2017	30	25.5571	75.7347	874.8561	6.6755	2.1867
31	2017	31	22.3857	75.8878	844.8439	7.1827	1.0316
79	2017	31	30.6000	63.4082	963.6633	9.8041	1.4622
51	2017	31	27.5429	70.8673	934.5776	8.5306	1.3020
14	2017	31	24.4143	80.7041	894.7918	6.5112	1.2235
67	2017	31	24.6429	80.5102	899.2459	7.1418	1.7673
42	2017	31	24.1000	74.3061	872.9816	7.2378	2.0653
50	2017	31	25.6286	71.2755	898.6714	9.0051	1.8214
43	2017	31	24.1000	74.3061	872.9816	7.2378	2.0653
85	2017	31	26.5429	65.2449	906.4622	9.8337	1.7439
25	2017	31	29.3286	74.3061	971.4071	7.8816	0.5235
69	2017	31	27.9286	71.5000	935.4408	8.9449	1.4061
57	2017	31	24.8286	75.3367	884.2347	8.6357	2.0837
9	2017	31	22.7429	76.0816	852.9378	7.0276	1.7714
72	2017	31	23.0000	81.8776	875.8367	6.2990	1.0082
26	2017	31	22.9286	84.3878	865.8551	6.8449	1.5041
7	2017	31	22.9286	79.5204	857.9092	6.7429	1.3204
83	2017	31	27.0857	75.7245	941.0071	7.5061	1.1786

76	2017	31	27.2714	69.1531	917.1673	9.8929	1.4173
36	2017	31	27.7571	69.1939	925.0286	8.6837	1.5969
81	2017	31	27.5429	70.8673	934.5776	8.5306	1.3020
15	2017	31	26.2857	71.0714	914.5837	9.2796	1.7939
32	2017	31	24.1000	74.3061	872.9816	7.2378	2.0653
73	2017	31	29.7286	64.1633	956.3337	8.4092	1.2796
71	2017	31	27.7571	69.1939	925.0286	8.6837	1.5969
41	2017	31	24.6429	72.1429	872.2704	7.2010	1.6592
10	2017	31	28.2857	74.1837	958.6704	9.0633	1.1194
23	2017	31	19.2857	79.3265	776.6184	6.7776	1.2520
27	2017	31	22.9286	79.5204	857.9092	6.7429	1.3204
60	2017	31	27.5429	70.8673	934.5776	8.5306	1.3020
53	2017	31	22.7429	76.0816	852.9378	7.0276	1.7714
66	2017	31	24.4143	80.7041	894.7918	6.5112	1.2235
59	2017	31	24.8286	75.3367	884.2347	8.6357	2.0837
61	2017	31	29.7286	64.1633	956.3337	8.4092	1.2796
84	2017	31	29.7286	64.1633	956.3337	8.4092	1.2796
38	2017	31	24.8286	75.3367	884.2347	8.6357	2.0837
87	2017	31	25.1714	75.8061	896.8663	7.5867	1.1000
34	2017	31	24.8286	75.3367	884.2347	8.6357	2.0837
29	2017	31	27.9286	71.5000	935.4408	8.9449	1.4061
5	2017	31	21.6714	82.8265	831.7418	6.7204	0.9449
8	2017	31	22.7429	76.0816	852.9378	7.0276	1.7714
12	2017	31	21.6714	82.8265	831.7418	6.7204	0.9449
13	2017	31	27.0857	75.7245	941.0071	7.5061	1.1786
18	2017	31	30.0571	67.7041	958.1235	8.6520	1.1337
33	2017	31	25.6286	71.2755	898.6714	9.0051	1.8214
56	2017	31	29.3286	74.3061	971.4071	7.8816	0.5235
77	2017	31	26.2857	71.0714	914.5837	9.2796	1.7939
54	2017	31	21.6714	82.8265	831.7418	6.7204	0.9449
21	2017	31	25.6286	71.2755	898.6714	9.0051	1.8214
68	2017	31	30.6000	63.4082	963.6633	9.8041	1.4622
74	2017	31	29.7286	64.1633	956.3337	8.4092	1.2796
88	2017	31	24.1000	74.3061	872.9816	7.2378	2.0653
16	2017	31	27.2714	69.1531	917.1673	9.8929	1.4173
30	2017	31	24.4143	80.7041	894.7918	6.5112	1.2235
6	2017	31	30.6000	63.4082	963.6633	9.8041	1.4622
49	2017	31	27.9286	71.5000	935.4408	8.9449	1.4061
22	2017	31	24.1000	74.3061	872.9816	7.2378	2.0653
45	2017	31	21.2000	81.9694	817.3990	6.3898	1.2041
58	2017	31	27.9286	71.5000	935.4408	8.9449	1.4061
37	2017	31	30.6000	63.4082	963.6633	9.8041	1.4622
17	2017	31	24.6429	80.5102	899.2459	7.1418	1.7673
55	2017	31	23.0000	81.8776	875.8367	6.2990	1.0082
46	2017	31	27.2714	69.1531	917.1673	9.8929	1.4173
86	2017	31	23.8857	77.3673	864.4408	8.4541	1.5745
2	2017	31	23.8857	77.3673	864.4408	8.4541	1.5745
4	2017	31	25.6286	71.2755	898.6714	9.0051	1.8214
47	2017	31	28.4286	76.3571	954.5469	7.2663	0.4980
82	2017	31	24.1000	74.3061	872.9816	7.2378	2.0653

19	2017	31	27.8714	77.2755	955.9480	6.4296	1.1051
20	2017	31	22.7429	76.0816	852.9378	7.0276	1.7714
80	2017	31	24.1000	74.3061	872.9816	7.2378	2.0653
3	2017	31	27.0857	75.7245	941.0071	7.5061	1.1786
52	2017	31	24.6429	80.5102	899.2459	7.1418	1.7673
70	2017	31	26.5429	65.2449	906.4622	9.8337	1.7439
64	2017	31	19.2857	79.3265	776.6184	6.7776	1.2520
48	2017	31	26.2857	71.0714	914.5837	9.2796	1.7939
65	2017	31	24.6429	80.5102	899.2459	7.1418	1.7673
44	2017	31	26.5429	65.2449	906.4622	9.8337	1.7439
75	2017	31	19.2857	79.3265	776.6184	6.7776	1.2520
40	2017	31	28.4429	71.0204	940.8816	9.0714	1.3582
11	2017	31	23.0000	81.8776	875.8367	6.2990	1.0082
35	2017	31	27.5429	70.8673	934.5776	8.5306	1.3020
78	2017	31	25.1714	75.8061	896.8663	7.5867	1.1000
28	2017	31	27.7571	69.1939	925.0286	8.6837	1.5969
39	2017	31	24.6429	80.5102	899.2459	7.1418	1.7673
24	2017	31	27.9286	71.5000	935.4408	8.9449	1.4061
63	2017	31	28.4429	71.0204	940.8816	9.0714	1.3582
62	2017	31	24.6429	72.1429	872.2704	7.2010	1.6592
1	2017	31	24.1000	74.3061	872.9816	7.2378	2.0653
31	2017	32	21.8000	78.2143	843.8357	6.0204	1.0704
79	2017	32	28.3000	66.7449	962.0255	8.4561	1.5898
51	2017	32	26.2571	74.5612	933.0418	7.4531	1.2929
14	2017	32	23.9714	84.1122	893.4531	5.0276	1.4694
67	2017	32	24.0571	83.6122	897.9592	5.4867	1.8969
42	2017	32	23.5143	77.5000	871.6255	5.8214	2.0735
50	2017	32	24.3000	76.3571	897.4071	6.5592	1.7643
43	2017	32	23.5143	77.5000	871.6255	5.8214	2.0735
85	2017	32	24.9000	72.0612	905.3408	7.9255	1.5816
25	2017	32	27.6143	76.0408	969.8745	6.5990	0.4102
69	2017	32	27.0429	73.9184	933.9092	7.3051	1.4776
57	2017	32	23.5000	79.1531	882.9276	7.4510	1.8980
9	2017	32	22.5143	77.6633	851.6357	5.7929	1.6673
72	2017	32	23.2143	82.8776	874.4347	4.8480	1.1378
26	2017	32	23.2429	85.7653	864.4612	6.9898	1.5612
7	2017	32	22.9857	81.4286	856.5633	6.3224	1.4163
83	2017	32	27.6000	77.6633	939.4316	7.2908	1.1765
76	2017	32	25.9429	72.7857	915.7102	8.5143	1.4245
36	2017	32	26.1714	71.7653	923.5551	7.7622	1.7653
81	2017	32	26.2571	74.5612	933.0418	7.4531	1.2929
15	2017	32	24.8857	77.9796	913.2959	7.3163	1.6163
32	2017	32	23.5143	77.5000	871.6255	5.8214	2.0735
73	2017	32	28.0286	69.6939	954.8276	6.9704	1.1133
71	2017	32	26.1714	71.7653	923.5551	7.7622	1.7653
41	2017	32	23.4714	74.2653	871.0173	5.6224	1.4133
10	2017	32	27.0571	76.8878	957.1204	8.0429	1.2112
23	2017	32	18.3429	79.7857	775.6673	5.9806	1.2398
27	2017	32	22.9857	81.4286	856.5633	6.3224	1.4163
60	2017	32	26.2571	74.5612	933.0418	7.4531	1.2929

53	2017	32	22.5143	77.6633	851.6357	5.7929	1.6673
66	2017	32	23.9714	84.1122	893.4531	5.0276	1.4694
59	2017	32	23.5000	79.1531	882.9276	7.4510	1.8980
61	2017	32	28.0286	69.6939	954.8276	6.9704	1.1133
84	2017	32	28.0286	69.6939	954.8276	6.9704	1.1133
38	2017	32	23.5000	79.1531	882.9276	7.4510	1.8980
87	2017	32	25.2571	77.7959	895.4551	6.2367	1.0551
34	2017	32	23.5000	79.1531	882.9276	7.4510	1.8980
29	2017	32	27.0429	73.9184	933.9092	7.3051	1.4776
5	2017	32	21.4857	83.0816	830.4765	6.5480	1.2143
8	2017	32	22.5143	77.6633	851.6357	5.7929	1.6673
12	2017	32	21.4857	83.0816	830.4765	6.5480	1.2143
13	2017	32	27.6000	77.6633	939.4316	7.2908	1.1765
18	2017	32	27.6571	70.2551	956.5061	6.8133	1.1327
33	2017	32	24.3000	76.3571	897.4071	6.5592	1.7643
56	2017	32	27.6143	76.0408	969.8745	6.5990	0.4102
77	2017	32	24.8857	77.9796	913.2959	7.3163	1.6163
54	2017	32	21.4857	83.0816	830.4765	6.5480	1.2143
21	2017	32	24.3000	76.3571	897.4071	6.5592	1.7643
68	2017	32	28.3000	66.7449	962.0255	8.4561	1.5898
74	2017	32	28.0286	69.6939	954.8276	6.9704	1.1133
88	2017	32	23.5143	77.5000	871.6255	5.8214	2.0735
16	2017	32	25.9429	72.7857	915.7102	8.5143	1.4245
30	2017	32	23.9714	84.1122	893.4531	5.0276	1.4694
6	2017	32	28.3000	66.7449	962.0255	8.4561	1.5898
49	2017	32	27.0429	73.9184	933.9092	7.3051	1.4776
22	2017	32	23.5143	77.5000	871.6255	5.8214	2.0735
45	2017	32	21.4143	81.1837	816.1500	5.9398	1.3061
58	2017	32	27.0429	73.9184	933.9092	7.3051	1.4776
37	2017	32	28.3000	66.7449	962.0255	8.4561	1.5898
17	2017	32	24.0571	83.6122	897.9592	5.4867	1.8969
55	2017	32	23.2143	82.8776	874.4347	4.8480	1.1378
46	2017	32	25.9429	72.7857	915.7102	8.5143	1.4245
86	2017	32	23.1000	78.7449	863.2122	7.4755	1.5418
2	2017	32	23.1000	78.7449	863.2122	7.4755	1.5418
4	2017	32	24.3000	76.3571	897.4071	6.5592	1.7643
47	2017	32	27.8000	80.5816	952.9480	6.6306	0.5653
82	2017	32	23.5143	77.5000	871.6255	5.8214	2.0735
19	2017	32	27.6429	79.6020	954.4878	4.7571	1.1020
20	2017	32	22.5143	77.6633	851.6357	5.7929	1.6673
80	2017	32	23.5143	77.5000	871.6255	5.8214	2.0735
3	2017	32	27.6000	77.6633	939.4316	7.2908	1.1765
52	2017	32	24.0571	83.6122	897.9592	5.4867	1.8969
70	2017	32	24.9000	72.0612	905.3408	7.9255	1.5816
64	2017	32	18.3429	79.7857	775.6673	5.9806	1.2398
48	2017	32	24.8857	77.9796	913.2959	7.3163	1.6163
65	2017	32	24.0571	83.6122	897.9592	5.4867	1.8969
44	2017	32	24.9000	72.0612	905.3408	7.9255	1.5816
75	2017	32	18.3429	79.7857	775.6673	5.9806	1.2398
40	2017	32	26.1714	73.9898	939.4194	8.2143	1.5837

11	2017	32	23.2143	82.8776	874.4347	4.8480	1.1378
35	2017	32	26.2571	74.5612	933.0418	7.4531	1.2929
78	2017	32	25.2571	77.7959	895.4551	6.2367	1.0551
28	2017	32	26.1714	71.7653	923.5551	7.7622	1.7653
39	2017	32	24.0571	83.6122	897.9592	5.4867	1.8969
24	2017	32	27.0429	73.9184	933.9092	7.3051	1.4776
63	2017	32	26.1714	73.9898	939.4194	8.2143	1.5837
62	2017	32	23.4714	74.2653	871.0173	5.6224	1.4133
1	2017	32	23.5143	77.5000	871.6255	5.8214	2.0735
31	2017	33	23.2857	76.9490	844.4735	5.0408	1.0755
79	2017	33	27.6857	74.7755	963.6071	6.2520	1.5480
51	2017	33	25.7571	80.3367	934.4265	5.6765	1.3102
14	2017	33	23.0143	88.7143	894.6867	2.9092	2.0602
67	2017	33	23.6429	87.6429	899.1194	3.9337	2.3000
42	2017	33	23.5571	80.1020	872.5071	4.5194	2.1847
50	2017	33	25.9143	77.3163	898.3490	5.0337	1.8480
43	2017	33	23.5571	80.1020	872.5071	4.5194	2.1847
85	2017	33	26.9571	73.9388	906.4031	6.5510	1.7082
25	2017	33	26.6857	82.2551	971.6867	4.3388	0.5122
69	2017	33	26.7286	75.7041	935.1551	5.4480	1.5184
57	2017	33	24.3857	80.5714	883.8847	6.4571	2.1163
9	2017	33	22.0857	79.7551	852.3837	4.8745	2.0429
72	2017	33	22.1000	84.0918	875.3602	3.1714	1.4663
26	2017	33	22.1429	90.0000	865.2980	5.3337	1.5612
7	2017	33	22.3571	84.0306	857.3204	5.3388	1.6041
83	2017	33	25.9429	80.1837	940.7031	5.5255	1.1459
76	2017	33	26.0714	74.9184	916.7857	6.7592	1.3602
36	2017	33	26.1286	77.4796	924.8735	5.7224	1.6990
81	2017	33	25.7571	80.3367	934.4265	5.6765	1.3102
15	2017	33	25.9714	80.6429	914.3459	5.8500	1.5459
32	2017	33	23.5571	80.1020	872.5071	4.5194	2.1847
73	2017	33	28.3286	73.2653	956.2408	5.4633	1.1520
71	2017	33	26.1286	77.4796	924.8735	5.7224	1.6990
41	2017	33	24.2286	75.5816	871.8398	4.4551	1.5684
10	2017	33	26.4286	81.5204	958.7480	5.3265	1.1837
23	2017	33	19.0286	81.9082	776.0653	4.1643	1.4194
27	2017	33	22.3571	84.0306	857.3204	5.3388	1.6041
60	2017	33	25.7571	80.3367	934.4265	5.6765	1.3102
53	2017	33	22.0857	79.7551	852.3837	4.8745	2.0429
66	2017	33	23.0143	88.7143	894.6867	2.9092	2.0602
59	2017	33	24.3857	80.5714	883.8847	6.4571	2.1163
61	2017	33	28.3286	73.2653	956.2408	5.4633	1.1520
84	2017	33	28.3286	73.2653	956.2408	5.4633	1.1520
38	2017	33	24.3857	80.5714	883.8847	6.4571	2.1163
87	2017	33	24.3000	80.9286	896.5480	4.8439	1.1673
34	2017	33	24.3857	80.5714	883.8847	6.4571	2.1163
29	2017	33	26.7286	75.7041	935.1551	5.4480	1.5184
5	2017	33	21.2000	84.7449	831.1061	5.2010	1.2959
8	2017	33	22.0857	79.7551	852.3837	4.8745	2.0429
12	2017	33	21.2000	84.7449	831.1061	5.2010	1.2959

13	2017	33	25.9429	80.1837	940.7031	5.5255	1.1459
18	2017	33	27.1000	78.6837	958.0153	4.3367	1.0684
33	2017	33	25.9143	77.3163	898.3490	5.0337	1.8480
56	2017	33	26.6857	82.2551	971.6867	4.3388	0.5122
77	2017	33	25.9714	80.6429	914.3459	5.8500	1.5459
54	2017	33	21.2000	84.7449	831.1061	5.2010	1.2959
21	2017	33	25.9143	77.3163	898.3490	5.0337	1.8480
68	2017	33	27.6857	74.7755	963.6071	6.2520	1.5480
74	2017	33	28.3286	73.2653	956.2408	5.4633	1.1520
88	2017	33	23.5571	80.1020	872.5071	4.5194	2.1847
16	2017	33	26.0714	74.9184	916.7857	6.7592	1.3602
30	2017	33	23.0143	88.7143	894.6867	2.9092	2.0602
6	2017	33	27.6857	74.7755	963.6071	6.2520	1.5480
49	2017	33	26.7286	75.7041	935.1551	5.4480	1.5184
22	2017	33	23.5571	80.1020	872.5071	4.5194	2.1847
45	2017	33	20.7429	80.6633	816.6867	4.9265	1.5459
58	2017	33	26.7286	75.7041	935.1551	5.4480	1.5184
37	2017	33	27.6857	74.7755	963.6071	6.2520	1.5480
17	2017	33	23.6429	87.6429	899.1194	3.9337	2.3000
55	2017	33	22.1000	84.0918	875.3602	3.1714	1.4663
46	2017	33	26.0714	74.9184	916.7857	6.7592	1.3602
86	2017	33	23.4143	80.9694	863.9990	5.8786	1.6429
2	2017	33	23.4143	80.9694	863.9990	5.8786	1.6429
4	2017	33	25.9143	77.3163	898.3490	5.0337	1.8480
47	2017	33	25.9143	88.5102	954.3735	4.1000	0.5582
82	2017	33	23.5571	80.1020	872.5071	4.5194	2.1847
19	2017	33	26.3571	84.6735	956.1235	2.4571	0.9133
20	2017	33	22.0857	79.7551	852.3837	4.8745	2.0429
80	2017	33	23.5571	80.1020	872.5071	4.5194	2.1847
3	2017	33	25.9429	80.1837	940.7031	5.5255	1.1459
52	2017	33	23.6429	87.6429	899.1194	3.9337	2.3000
70	2017	33	26.9571	73.9388	906.4031	6.5510	1.7082
64	2017	33	19.0286	81.9082	776.0653	4.1643	1.4194
48	2017	33	25.9714	80.6429	914.3459	5.8500	1.5459
65	2017	33	23.6429	87.6429	899.1194	3.9337	2.3000
44	2017	33	26.9571	73.9388	906.4031	6.5510	1.7082
75	2017	33	19.0286	81.9082	776.0653	4.1643	1.4194
40	2017	33	25.8429	80.7143	941.0622	5.7051	1.7776
11	2017	33	22.1000	84.0918	875.3602	3.1714	1.4663
35	2017	33	25.7571	80.3367	934.4265	5.6765	1.3102
78	2017	33	24.3000	80.9286	896.5480	4.8439	1.1673
28	2017	33	26.1286	77.4796	924.8735	5.7224	1.6990
39	2017	33	23.6429	87.6429	899.1194	3.9337	2.3000
24	2017	33	26.7286	75.7041	935.1551	5.4480	1.5184
63	2017	33	25.8429	80.7143	941.0622	5.7051	1.7776
62	2017	33	24.2286	75.5816	871.8398	4.4551	1.5684
1	2017	33	23.5571	80.1020	872.5071	4.5194	2.1847
31	2017	34	23.1714	75.4388	845.0694	6.4878	1.1357
79	2017	34	29.5429	75.9898	965.3714	6.4408	1.4541
51	2017	34	26.7571	79.8673	935.8990	6.0561	1.4051

14	2017	34	24.3857	85.5408	895.8735	4.4969	2.2092
67	2017	34	24.5857	84.9694	900.3020	5.2806	2.8694
42	2017	34	24.0714	78.3061	873.5663	4.9837	2.4949
50	2017	34	26.5000	73.4184	899.2949	6.0959	1.9867
43	2017	34	24.0714	78.3061	873.5663	4.9837	2.4949
85	2017	34	27.5286	68.0612	907.1745	7.6153	1.9194
25	2017	34	27.8571	82.7041	973.1306	5.4265	0.6020
69	2017	34	27.0000	75.5408	936.5153	5.7765	1.4469
57	2017	34	24.8714	77.4898	884.8510	7.3857	2.7276
9	2017	34	22.9714	79.1633	853.3531	6.0082	2.5429
72	2017	34	23.2286	82.0102	876.4214	4.3112	1.8031
26	2017	34	22.9000	88.7857	866.2786	5.7378	1.6122
7	2017	34	23.0571	82.0204	858.2939	6.3592	1.9480
83	2017	34	27.1143	79.3265	941.8561	6.5204	1.1571
76	2017	34	26.8714	73.4082	918.0235	6.9867	1.3459
36	2017	34	26.7857	75.9286	926.1816	6.1571	1.7388
81	2017	34	26.7571	79.8673	935.8990	6.0561	1.4051
15	2017	34	26.4286	76.7449	915.4837	6.3398	1.6918
32	2017	34	24.0714	78.3061	873.5663	4.9837	2.4949
73	2017	34	29.3429	70.1735	957.7102	6.4857	1.2388
71	2017	34	26.7857	75.9286	926.1816	6.1571	1.7388
41	2017	34	24.7429	73.5918	872.7020	5.9724	2.1020
10	2017	34	27.1429	82.1224	960.3347	5.3765	1.1724
23	2017	34	19.0000	82.0408	776.7245	5.3592	1.6306
27	2017	34	23.0571	82.0204	858.2939	6.3592	1.9480
60	2017	34	26.7571	79.8673	935.8990	6.0561	1.4051
53	2017	34	22.9714	79.1633	853.3531	6.0082	2.5429
66	2017	34	24.3857	85.5408	895.8735	4.4969	2.2092
59	2017	34	24.8714	77.4898	884.8510	7.3857	2.7276
61	2017	34	29.3429	70.1735	957.7102	6.4857	1.2388
84	2017	34	29.3429	70.1735	957.7102	6.4857	1.2388
38	2017	34	24.8714	77.4898	884.8510	7.3857	2.7276
87	2017	34	25.0571	80.3673	897.6857	5.7561	1.3429
34	2017	34	24.8714	77.4898	884.8510	7.3857	2.7276
29	2017	34	27.0000	75.5408	936.5153	5.7765	1.4469
5	2017	34	21.4857	82.6939	831.9796	6.5531	1.4122
8	2017	34	22.9714	79.1633	853.3531	6.0082	2.5429
12	2017	34	21.4857	82.6939	831.9796	6.5531	1.4122
13	2017	34	27.1143	79.3265	941.8561	6.5204	1.1571
18	2017	34	28.8143	79.8980	959.7969	4.7071	1.1041
33	2017	34	26.5000	73.4184	899.2949	6.0959	1.9867
56	2017	34	27.8571	82.7041	973.1306	5.4265	0.6020
77	2017	34	26.4286	76.7449	915.4837	6.3398	1.6918
54	2017	34	21.4857	82.6939	831.9796	6.5531	1.4122
21	2017	34	26.5000	73.4184	899.2949	6.0959	1.9867
68	2017	34	29.5429	75.9898	965.3714	6.4408	1.4541
74	2017	34	29.3429	70.1735	957.7102	6.4857	1.2388
88	2017	34	24.0714	78.3061	873.5663	4.9837	2.4949
16	2017	34	26.8714	73.4082	918.0235	6.9867	1.3459
30	2017	34	24.3857	85.5408	895.8735	4.4969	2.2092

6	2017	34	29.5429	75.9898	965.3714	6.4408	1.4541
49	2017	34	27.0000	75.5408	936.5153	5.7765	1.4469
22	2017	34	24.0714	78.3061	873.5663	4.9837	2.4949
45	2017	34	20.7286	78.6327	817.5316	5.8622	1.9755
58	2017	34	27.0000	75.5408	936.5153	5.7765	1.4469
37	2017	34	29.5429	75.9898	965.3714	6.4408	1.4541
17	2017	34	24.5857	84.9694	900.3020	5.2806	2.8694
55	2017	34	23.2286	82.0102	876.4214	4.3112	1.8031
46	2017	34	26.8714	73.4082	918.0235	6.9867	1.3459
86	2017	34	24.0000	80.0102	864.8337	6.9153	2.0704
2	2017	34	24.0000	80.0102	864.8337	6.9153	2.0704
4	2017	34	26.5000	73.4184	899.2949	6.0959	1.9867
47	2017	34	27.5429	87.9286	955.6214	5.4296	0.6439
82	2017	34	24.0714	78.3061	873.5663	4.9837	2.4949
19	2017	34	27.5714	83.9286	957.4194	4.4663	1.1520
20	2017	34	22.9714	79.1633	853.3531	6.0082	2.5429
80	2017	34	24.0714	78.3061	873.5663	4.9837	2.4949
3	2017	34	27.1143	79.3265	941.8561	6.5204	1.1571
52	2017	34	24.5857	84.9694	900.3020	5.2806	2.8694
70	2017	34	27.5286	68.0612	907.1745	7.6153	1.9194
64	2017	34	19.0000	82.0408	776.7245	5.3592	1.6306
48	2017	34	26.4286	76.7449	915.4837	6.3398	1.6918
65	2017	34	24.5857	84.9694	900.3020	5.2806	2.8694
44	2017	34	27.5286	68.0612	907.1745	7.6153	1.9194
75	2017	34	19.0000	82.0408	776.7245	5.3592	1.6306
40	2017	34	26.5000	80.9184	942.4602	6.0235	1.7480
11	2017	34	23.2286	82.0102	876.4214	4.3112	1.8031
35	2017	34	26.7571	79.8673	935.8990	6.0561	1.4051
78	2017	34	25.0571	80.3673	897.6857	5.7561	1.3429
28	2017	34	26.7857	75.9286	926.1816	6.1571	1.7388
39	2017	34	24.5857	84.9694	900.3020	5.2806	2.8694
24	2017	34	27.0000	75.5408	936.5153	5.7765	1.4469
63	2017	34	26.5000	80.9184	942.4602	6.0235	1.7480
62	2017	34	24.7429	73.5918	872.7020	5.9724	2.1020
1	2017	34	24.0714	78.3061	873.5663	4.9837	2.4949
31	2017	35	21.1000	76.8571	846.0980	6.8980	1.1653
79	2017	35	24.1857	74.5918	967.1163	6.1898	1.7418
51	2017	35	23.5429	80.0510	937.2888	6.5959	1.4990
14	2017	35	22.8143	84.2347	896.7786	5.1092	1.8929
67	2017	35	22.6857	83.9592	901.4173	5.3000	2.5724
42	2017	35	22.0286	79.4490	874.6796	4.4827	2.4806
50	2017	35	23.4714	74.9592	900.5755	5.7592	2.0418
43	2017	35	22.0286	79.4490	874.6796	4.4827	2.4806
85	2017	35	24.3143	67.6531	908.4020	7.3327	1.9571
25	2017	35	27.4143	81.3469	974.1367	6.5500	0.4857
69	2017	35	25.0286	77.8878	937.8745	5.2776	1.2347
57	2017	35	22.7000	78.7245	886.0316	6.7296	2.6571
9	2017	35	21.1714	79.5714	854.3561	5.4765	2.6469
72	2017	35	21.4571	82.5714	877.3490	4.4459	1.8082
26	2017	35	21.9571	88.7449	867.0755	5.4347	1.4500

7	2017	35	21.5143	83.3367	859.1582	5.9235	1.8898
83	2017	35	25.7286	79.5000	942.6796	6.8041	1.2357
76	2017	35	24.2571	75.8265	919.4520	6.3949	1.3337
36	2017	35	24.3571	75.9694	927.3939	5.4949	1.6867
81	2017	35	23.5429	80.0510	937.2888	6.5959	1.4990
15	2017	35	23.4571	79.1837	916.9490	5.5429	1.7582
32	2017	35	22.0286	79.4490	874.6796	4.4827	2.4806
73	2017	35	26.0286	71.7857	959.2714	6.4041	1.1122
71	2017	35	24.3571	75.9694	927.3939	5.4949	1.6867
41	2017	35	22.6857	74.3469	873.8306	5.9378	2.1704
10	2017	35	24.8286	82.1837	961.7612	5.5122	1.3020
23	2017	35	17.2571	82.8571	777.5184	5.6347	1.8571
27	2017	35	21.5143	83.3367	859.1582	5.9235	1.8898
60	2017	35	23.5429	80.0510	937.2888	6.5959	1.4990
53	2017	35	21.1714	79.5714	854.3561	5.4765	2.6469
66	2017	35	22.8143	84.2347	896.7786	5.1092	1.8929
59	2017	35	22.7000	78.7245	886.0316	6.7296	2.6571
61	2017	35	26.0286	71.7857	959.2714	6.4041	1.1122
84	2017	35	26.0286	71.7857	959.2714	6.4041	1.1122
38	2017	35	22.7000	78.7245	886.0316	6.7296	2.6571
87	2017	35	23.1857	81.2449	898.7480	5.4214	1.1653
34	2017	35	22.7000	78.7245	886.0316	6.7296	2.6571
29	2017	35	25.0286	77.8878	937.8745	5.2776	1.2347
5	2017	35	19.7571	84.4898	832.8449	5.7551	1.3959
8	2017	35	21.1714	79.5714	854.3561	5.4765	2.6469
12	2017	35	19.7571	84.4898	832.8449	5.7551	1.3959
13	2017	35	25.7286	79.5000	942.6796	6.8041	1.2357
18	2017	35	24.4857	77.1020	961.5990	5.2000	1.3235
33	2017	35	23.4714	74.9592	900.5755	5.7592	2.0418
56	2017	35	27.4143	81.3469	974.1367	6.5500	0.4857
77	2017	35	23.4571	79.1837	916.9490	5.5429	1.7582
54	2017	35	19.7571	84.4898	832.8449	5.7551	1.3959
21	2017	35	23.4714	74.9592	900.5755	5.7592	2.0418
68	2017	35	24.1857	74.5918	967.1163	6.1898	1.7418
74	2017	35	26.0286	71.7857	959.2714	6.4041	1.1122
88	2017	35	22.0286	79.4490	874.6796	4.4827	2.4806
16	2017	35	24.2571	75.8265	919.4520	6.3949	1.3337
30	2017	35	22.8143	84.2347	896.7786	5.1092	1.8929
6	2017	35	24.1857	74.5918	967.1163	6.1898	1.7418
49	2017	35	25.0286	77.8878	937.8745	5.2776	1.2347
22	2017	35	22.0286	79.4490	874.6796	4.4827	2.4806
45	2017	35	19.8286	80.8571	818.3429	5.1541	1.8592
58	2017	35	25.0286	77.8878	937.8745	5.2776	1.2347
37	2017	35	24.1857	74.5918	967.1163	6.1898	1.7418
17	2017	35	22.6857	83.9592	901.4173	5.3000	2.5724
55	2017	35	21.4571	82.5714	877.3490	4.4459	1.8082
46	2017	35	24.2571	75.8265	919.4520	6.3949	1.3337
86	2017	35	21.8429	81.8673	865.9010	6.7969	2.0245
2	2017	35	21.8429	81.8673	865.9010	6.7969	2.0245
4	2017	35	23.4714	74.9592	900.5755	5.7592	2.0418

47	2017	35	25.9143	87.2449	956.5469	5.7908	0.6663
82	2017	35	22.0286	79.4490	874.6796	4.4827	2.4806
19	2017	35	26.7143	82.6020	958.2867	5.8653	1.3592
20	2017	35	21.1714	79.5714	854.3561	5.4765	2.6469
80	2017	35	22.0286	79.4490	874.6796	4.4827	2.4806
3	2017	35	25.7286	79.5000	942.6796	6.8041	1.2357
52	2017	35	22.6857	83.9592	901.4173	5.3000	2.5724
70	2017	35	24.3143	67.6531	908.4020	7.3327	1.9571
64	2017	35	17.2571	82.8571	777.5184	5.6347	1.8571
48	2017	35	23.4571	79.1837	916.9490	5.5429	1.7582
65	2017	35	22.6857	83.9592	901.4173	5.3000	2.5724
44	2017	35	24.3143	67.6531	908.4020	7.3327	1.9571
75	2017	35	17.2571	82.8571	777.5184	5.6347	1.8571
40	2017	35	25.2429	80.7143	943.5786	7.0265	1.4255
11	2017	35	21.4571	82.5714	877.3490	4.4459	1.8082
35	2017	35	23.5429	80.0510	937.2888	6.5959	1.4990
78	2017	35	23.1857	81.2449	898.7480	5.4214	1.1653
28	2017	35	24.3571	75.9694	927.3939	5.4949	1.6867
39	2017	35	22.6857	83.9592	901.4173	5.3000	2.5724
24	2017	35	25.0286	77.8878	937.8745	5.2776	1.2347
63	2017	35	25.2429	80.7143	943.5786	7.0265	1.4255
62	2017	35	22.6857	74.3469	873.8306	5.9378	2.1704
1	2017	35	22.0286	79.4490	874.6796	4.4827	2.4806
31	2017	36	20.3143	80.8571	847.1163	4.4214	1.1020
79	2017	36	24.2714	81.7653	968.8673	2.5469	1.8061
51	2017	36	23.0571	87.8776	938.4847	3.4429	1.4561
14	2017	36	22.7857	88.8367	897.3102	3.0327	1.7010
67	2017	36	22.3714	89.5714	902.1327	2.5122	1.9204
42	2017	36	21.8286	83.3878	875.4010	2.8224	2.1184
50	2017	36	22.5000	82.5102	901.7714	3.0949	1.8561
43	2017	36	21.8286	83.3878	875.4010	2.8224	2.1184
85	2017	36	23.4571	75.1327	909.8622	4.6235	1.7480
25	2017	36	26.2429	84.7143	974.9071	5.2449	0.4643
69	2017	36	24.2857	83.6939	939.0357	2.4306	1.1765
57	2017	36	21.9429	84.0306	886.9949	4.3469	2.0133
9	2017	36	20.8571	82.2449	854.9582	3.3969	2.3071
72	2017	36	21.8571	84.9490	877.9449	3.2306	1.4949
26	2017	36	21.3429	92.2857	867.5816	3.6041	1.2939
7	2017	36	21.4286	87.1327	859.6837	3.6459	1.5061
83	2017	36	26.2714	81.3878	943.3816	5.1541	1.1582
76	2017	36	23.1286	82.9286	920.5959	3.6255	1.2684
36	2017	36	23.8714	84.1122	928.3510	2.6765	1.3143
81	2017	36	23.0571	87.8776	938.4847	3.4429	1.4561
15	2017	36	22.7143	86.6837	918.1633	2.7245	1.5224
32	2017	36	21.8286	83.3878	875.4010	2.8224	2.1184
73	2017	36	25.3000	79.5408	960.7439	3.1367	0.8531
71	2017	36	23.8714	84.1122	928.3510	2.6765	1.3143
41	2017	36	22.0286	78.4592	874.7714	3.5714	1.5704
10	2017	36	24.5857	86.9694	963.0704	2.6235	1.3082
23	2017	36	16.3857	86.1633	777.9051	3.3235	1.8092

27	2017	36	21.4286	87.1327	859.6837	3.6459	1.5061
60	2017	36	23.0571	87.8776	938.4847	3.4429	1.4561
53	2017	36	20.8571	82.2449	854.9582	3.3969	2.3071
66	2017	36	22.7857	88.8367	897.3102	3.0327	1.7010
59	2017	36	21.9429	84.0306	886.9949	4.3469	2.0133
61	2017	36	25.3000	79.5408	960.7439	3.1367	0.8531
84	2017	36	25.3000	79.5408	960.7439	3.1367	0.8531
38	2017	36	21.9429	84.0306	886.9949	4.3469	2.0133
87	2017	36	23.5286	84.2245	899.4551	3.8398	0.9939
34	2017	36	21.9429	84.0306	886.9949	4.3469	2.0133
29	2017	36	24.2857	83.6939	939.0357	2.4306	1.1765
5	2017	36	20.1143	88.2755	833.3153	3.0980	1.2724
8	2017	36	20.8571	82.2449	854.9582	3.3969	2.3071
12	2017	36	20.1143	88.2755	833.3153	3.0980	1.2724
13	2017	36	26.2714	81.3878	943.3816	5.1541	1.1582
18	2017	36	24.4000	83.9694	963.2388	2.3204	1.2214
33	2017	36	22.5000	82.5102	901.7714	3.0949	1.8561
56	2017	36	26.2429	84.7143	974.9071	5.2449	0.4643
77	2017	36	22.7143	86.6837	918.1633	2.7245	1.5224
54	2017	36	20.1143	88.2755	833.3153	3.0980	1.2724
21	2017	36	22.5000	82.5102	901.7714	3.0949	1.8561
68	2017	36	24.2714	81.7653	968.8673	2.5469	1.8061
74	2017	36	25.3000	79.5408	960.7439	3.1367	0.8531
88	2017	36	21.8286	83.3878	875.4010	2.8224	2.1184
16	2017	36	23.1286	82.9286	920.5959	3.6255	1.2684
30	2017	36	22.7857	88.8367	897.3102	3.0327	1.7010
6	2017	36	24.2714	81.7653	968.8673	2.5469	1.8061
49	2017	36	24.2857	83.6939	939.0357	2.4306	1.1765
22	2017	36	21.8286	83.3878	875.4010	2.8224	2.1184
45	2017	36	19.6571	85.7755	818.7214	2.6888	1.3837
58	2017	36	24.2857	83.6939	939.0357	2.4306	1.1765
37	2017	36	24.2714	81.7653	968.8673	2.5469	1.8061
17	2017	36	22.3714	89.5714	902.1327	2.5122	1.9204
55	2017	36	21.8571	84.9490	877.9449	3.2306	1.4949
46	2017	36	23.1286	82.9286	920.5959	3.6255	1.2684
86	2017	36	21.4429	86.6122	866.7408	4.1939	1.3653
2	2017	36	21.4429	86.6122	866.7408	4.1939	1.3653
4	2017	36	22.5000	82.5102	901.7714	3.0949	1.8561
47	2017	36	26.1000	89.9082	957.2663	3.8531	0.6041
82	2017	36	21.8286	83.3878	875.4010	2.8224	2.1184
19	2017	36	26.4714	84.7245	958.8939	4.2449	1.0908
20	2017	36	20.8571	82.2449	854.9582	3.3969	2.3071
80	2017	36	21.8286	83.3878	875.4010	2.8224	2.1184
3	2017	36	26.2714	81.3878	943.3816	5.1541	1.1582
52	2017	36	22.3714	89.5714	902.1327	2.5122	1.9204
70	2017	36	23.4571	75.1327	909.8622	4.6235	1.7480
64	2017	36	16.3857	86.1633	777.9051	3.3235	1.8092
48	2017	36	22.7143	86.6837	918.1633	2.7245	1.5224
65	2017	36	22.3714	89.5714	902.1327	2.5122	1.9204
44	2017	36	23.4571	75.1327	909.8622	4.6235	1.7480

75	2017	36	16.3857	86.1633	777.9051	3.3235	1.8092
40	2017	36	24.2429	86.5306	944.4765	4.7194	1.2622
11	2017	36	21.8571	84.9490	877.9449	3.2306	1.4949
35	2017	36	23.0571	87.8776	938.4847	3.4429	1.4561
78	2017	36	23.5286	84.2245	899.4551	3.8398	0.9939
28	2017	36	23.8714	84.1122	928.3510	2.6765	1.3143
39	2017	36	22.3714	89.5714	902.1327	2.5122	1.9204
24	2017	36	24.2857	83.6939	939.0357	2.4306	1.1765
63	2017	36	24.2429	86.5306	944.4765	4.7194	1.2622
62	2017	36	22.0286	78.4592	874.7714	3.5714	1.5704
1	2017	36	21.8286	83.3878	875.4010	2.8224	2.1184
31	2017	37	20.1571	77.6122	848.3276	4.7041	1.1878
79	2017	37	24.2429	82.4082	970.2122	1.7867	1.3990
51	2017	37	22.3714	88.4796	939.8806	2.0245	1.3051
14	2017	37	21.2143	88.7347	898.5653	2.0500	1.6888
67	2017	37	21.0571	90.1837	903.4520	1.4959	2.2969
42	2017	37	20.8000	81.2041	876.6520	2.3582	2.2786
50	2017	37	22.1000	82.6224	903.1857	2.6694	1.7194
43	2017	37	20.8000	81.2041	876.6520	2.3582	2.2786
85	2017	37	23.4857	75.4286	911.3643	4.5939	1.6704
25	2017	37	25.3714	85.6224	976.4245	4.0357	0.5959
69	2017	37	23.4429	82.7143	940.5520	1.4224	1.2480
57	2017	37	21.0286	84.1327	888.3153	3.6704	1.9918
9	2017	37	20.3143	79.6224	856.1510	3.9255	2.1276
72	2017	37	20.8714	81.9898	879.2337	3.3949	1.4582
26	2017	37	21.4429	89.5408	868.9194	3.7939	1.3459
7	2017	37	21.0143	83.5612	860.9827	3.7888	1.4582
83	2017	37	25.0286	79.4592	944.9704	4.3214	1.1378
76	2017	37	22.3571	82.8571	922.0133	2.1571	1.2041
36	2017	37	22.7571	84.6224	929.8071	2.0276	1.2449
81	2017	37	22.3714	88.4796	939.8806	2.0245	1.3051
15	2017	37	22.4857	84.6429	919.5316	2.5888	1.3898
32	2017	37	20.8000	81.2041	876.6520	2.3582	2.2786
73	2017	37	24.5143	80.2959	962.2816	1.5694	0.6694
71	2017	37	22.7571	84.6224	929.8071	2.0276	1.2449
41	2017	37	21.1000	77.5918	876.0531	3.8408	1.3204
10	2017	37	23.4000	87.2041	964.4592	1.9816	1.1745
23	2017	37	16.6857	84.0714	778.9061	3.3520	1.7224
27	2017	37	21.0143	83.5612	860.9827	3.7888	1.4582
60	2017	37	22.3714	88.4796	939.8806	2.0245	1.3051
53	2017	37	20.3143	79.6224	856.1510	3.9255	2.1276
66	2017	37	21.2143	88.7347	898.5653	2.0500	1.6888
59	2017	37	21.0286	84.1327	888.3153	3.6704	1.9918
61	2017	37	24.5143	80.2959	962.2816	1.5694	0.6694
84	2017	37	24.5143	80.2959	962.2816	1.5694	0.6694
38	2017	37	21.0286	84.1327	888.3153	3.6704	1.9918
87	2017	37	22.5000	81.5102	900.7745	3.8500	1.2163
34	2017	37	21.0286	84.1327	888.3153	3.6704	1.9918
29	2017	37	23.4429	82.7143	940.5520	1.4224	1.2480
5	2017	37	19.0000	84.2347	834.5163	3.9673	1.2980

8	2017	37	20.3143	79.6224	856.1510	3.9255	2.1276
12	2017	37	19.0000	84.2347	834.5163	3.9673	1.2980
13	2017	37	25.0286	79.4592	944.9704	4.3214	1.1378
18	2017	37	23.8857	85.4082	964.5153	1.3082	0.9245
33	2017	37	22.1000	82.6224	903.1857	2.6694	1.7194
56	2017	37	25.3714	85.6224	976.4245	4.0357	0.5959
77	2017	37	22.4857	84.6429	919.5316	2.5888	1.3898
54	2017	37	19.0000	84.2347	834.5163	3.9673	1.2980
21	2017	37	22.1000	82.6224	903.1857	2.6694	1.7194
68	2017	37	24.2429	82.4082	970.2122	1.7867	1.3990
74	2017	37	24.5143	80.2959	962.2816	1.5694	0.6694
88	2017	37	20.8000	81.2041	876.6520	2.3582	2.2786
16	2017	37	22.3571	82.8571	922.0133	2.1571	1.2041
30	2017	37	21.2143	88.7347	898.5653	2.0500	1.6888
6	2017	37	24.2429	82.4082	970.2122	1.7867	1.3990
49	2017	37	23.4429	82.7143	940.5520	1.4224	1.2480
22	2017	37	20.8000	81.2041	876.6520	2.3582	2.2786
45	2017	37	18.8714	82.9184	819.8959	3.0327	1.2622
58	2017	37	23.4429	82.7143	940.5520	1.4224	1.2480
37	2017	37	24.2429	82.4082	970.2122	1.7867	1.3990
17	2017	37	21.0571	90.1837	903.4520	1.4959	2.2969
55	2017	37	20.8714	81.9898	879.2337	3.3949	1.4582
46	2017	37	22.3571	82.8571	922.0133	2.1571	1.2041
86	2017	37	20.9714	82.3878	868.0092	3.7061	1.3082
2	2017	37	20.9714	82.3878	868.0092	3.7061	1.3082
4	2017	37	22.1000	82.6224	903.1857	2.6694	1.7194
47	2017	37	25.5714	88.2449	958.8071	3.6490	0.5378
82	2017	37	20.8000	81.2041	876.6520	2.3582	2.2786
19	2017	37	25.5000	83.9898	960.3347	2.9684	0.8735
20	2017	37	20.3143	79.6224	856.1510	3.9255	2.1276
80	2017	37	20.8000	81.2041	876.6520	2.3582	2.2786
3	2017	37	25.0286	79.4592	944.9704	4.3214	1.1378
52	2017	37	21.0571	90.1837	903.4520	1.4959	2.2969
70	2017	37	23.4857	75.4286	911.3643	4.5939	1.6704
64	2017	37	16.6857	84.0714	778.9061	3.3520	1.7224
48	2017	37	22.4857	84.6429	919.5316	2.5888	1.3898
65	2017	37	21.0571	90.1837	903.4520	1.4959	2.2969
44	2017	37	23.4857	75.4286	911.3643	4.5939	1.6704
75	2017	37	16.6857	84.0714	778.9061	3.3520	1.7224
40	2017	37	22.9429	87.8776	945.8265	3.5520	1.4082
11	2017	37	20.8714	81.9898	879.2337	3.3949	1.4582
35	2017	37	22.3714	88.4796	939.8806	2.0245	1.3051
78	2017	37	22.5000	81.5102	900.7745	3.8500	1.2163
28	2017	37	22.7571	84.6224	929.8071	2.0276	1.2449
39	2017	37	21.0571	90.1837	903.4520	1.4959	2.2969
24	2017	37	23.4429	82.7143	940.5520	1.4224	1.2480
63	2017	37	22.9429	87.8776	945.8265	3.5520	1.4082
62	2017	37	21.1000	77.5918	876.0531	3.8408	1.3204
1	2017	37	20.8000	81.2041	876.6520	2.3582	2.2786
31	2017	38	20.2429	76.3673	849.3449	5.4612	1.2173

79	2017	38	24.5571	79.0408	971.1867	3.1092	1.2296
51	2017	38	23.7286	84.2143	940.9827	2.9796	1.1469
14	2017	38	22.7143	86.3571	899.8306	2.7684	1.5449
67	2017	38	22.6429	87.3163	904.6878	2.6735	2.5898
42	2017	38	22.1143	78.5510	877.7694	3.1847	2.4398
50	2017	38	22.5857	81.1735	904.3184	3.2694	1.6694
43	2017	38	22.1143	78.5510	877.7694	3.1847	2.4398
85	2017	38	23.2857	73.9388	912.4704	4.9500	1.5510
25	2017	38	26.4857	83.3673	977.8551	4.1827	0.7704
69	2017	38	24.6857	79.7143	941.7214	2.7735	1.2908
57	2017	38	22.5000	82.2551	889.4286	4.1153	2.0490
9	2017	38	21.1857	76.8776	857.2969	5.0286	2.1622
72	2017	38	21.8286	79.8571	880.4653	3.5255	1.4398
26	2017	38	22.2000	85.3776	870.2224	4.8592	1.3612
7	2017	38	22.0429	79.4796	862.2398	5.0592	1.5490
83	2017	38	25.7286	78.2347	946.4367	4.4847	1.2163
76	2017	38	23.4714	80.6020	923.1806	2.7980	1.1847
36	2017	38	24.1857	80.1327	931.0010	3.3765	1.3888
81	2017	38	23.7286	84.2143	940.9827	2.9796	1.1469
15	2017	38	22.6000	82.2551	920.7031	3.5888	1.4286
32	2017	38	22.1143	78.5510	877.7694	3.1847	2.4398
73	2017	38	24.7000	78.6327	963.4837	2.3459	0.6837
71	2017	38	24.1857	80.1327	931.0010	3.3765	1.3888
41	2017	38	22.4286	75.1122	877.1684	4.3122	1.3357
10	2017	38	24.6143	84.6122	965.5643	3.2347	1.0561
23	2017	38	16.5143	81.6837	779.9102	4.2602	1.3878
27	2017	38	22.0429	79.4796	862.2398	5.0592	1.5490
60	2017	38	23.7286	84.2143	940.9827	2.9796	1.1469
53	2017	38	21.1857	76.8776	857.2969	5.0286	2.1622
66	2017	38	22.7143	86.3571	899.8306	2.7684	1.5449
59	2017	38	22.5000	82.2551	889.4286	4.1153	2.0490
61	2017	38	24.7000	78.6327	963.4837	2.3459	0.6837
84	2017	38	24.7000	78.6327	963.4837	2.3459	0.6837
38	2017	38	22.5000	82.2551	889.4286	4.1153	2.0490
87	2017	38	23.5429	78.5306	901.9806	4.6582	1.2786
34	2017	38	22.5000	82.2551	889.4286	4.1153	2.0490
29	2017	38	24.6857	79.7143	941.7214	2.7735	1.2908
5	2017	38	20.2286	81.5204	835.7286	5.3490	1.3745
8	2017	38	21.1857	76.8776	857.2969	5.0286	2.1622
12	2017	38	20.2286	81.5204	835.7286	5.3490	1.3745
13	2017	38	25.7286	78.2347	946.4367	4.4847	1.2163
18	2017	38	23.5429	83.2143	965.6122	1.9847	0.8582
33	2017	38	22.5857	81.1735	904.3184	3.2694	1.6694
56	2017	38	26.4857	83.3673	977.8551	4.1827	0.7704
77	2017	38	22.6000	82.2551	920.7031	3.5888	1.4286
54	2017	38	20.2286	81.5204	835.7286	5.3490	1.3745
21	2017	38	22.5857	81.1735	904.3184	3.2694	1.6694
68	2017	38	24.5571	79.0408	971.1867	3.1092	1.2296
74	2017	38	24.7000	78.6327	963.4837	2.3459	0.6837
88	2017	38	22.1143	78.5510	877.7694	3.1847	2.4398

16	2017	38	23.4714	80.6020	923.1806	2.7980	1.1847
30	2017	38	22.7143	86.3571	899.8306	2.7684	1.5449
6	2017	38	24.5571	79.0408	971.1867	3.1092	1.2296
49	2017	38	24.6857	79.7143	941.7214	2.7735	1.2908
22	2017	38	22.1143	78.5510	877.7694	3.1847	2.4398
45	2017	38	19.7000	79.9592	821.1112	4.6245	1.3694
58	2017	38	24.6857	79.7143	941.7214	2.7735	1.2908
37	2017	38	24.5571	79.0408	971.1867	3.1092	1.2296
17	2017	38	22.6429	87.3163	904.6878	2.6735	2.5898
55	2017	38	21.8286	79.8571	880.4653	3.5255	1.4398
46	2017	38	23.4714	80.6020	923.1806	2.7980	1.1847
86	2017	38	21.9857	78.7551	869.1255	4.2265	1.4102
2	2017	38	21.9857	78.7551	869.1255	4.2265	1.4102
4	2017	38	22.5857	81.1735	904.3184	3.2694	1.6694
47	2017	38	26.1286	84.8265	960.2286	4.4612	0.5378
82	2017	38	22.1143	78.5510	877.7694	3.1847	2.4398
19	2017	38	26.4429	81.3776	961.7347	3.5602	0.9347
20	2017	38	21.1857	76.8776	857.2969	5.0286	2.1622
80	2017	38	22.1143	78.5510	877.7694	3.1847	2.4398
3	2017	38	25.7286	78.2347	946.4367	4.4847	1.2163
52	2017	38	22.6429	87.3163	904.6878	2.6735	2.5898
70	2017	38	23.2857	73.9388	912.4704	4.9500	1.5510
64	2017	38	16.5143	81.6837	779.9102	4.2602	1.3878
48	2017	38	22.6000	82.2551	920.7031	3.5888	1.4286
65	2017	38	22.6429	87.3163	904.6878	2.6735	2.5898
44	2017	38	23.2857	73.9388	912.4704	4.9500	1.5510
75	2017	38	16.5143	81.6837	779.9102	4.2602	1.3878
40	2017	38	24.6143	84.6939	947.0561	4.3602	1.3092
11	2017	38	21.8286	79.8571	880.4653	3.5255	1.4398
35	2017	38	23.7286	84.2143	940.9827	2.9796	1.1469
78	2017	38	23.5429	78.5306	901.9806	4.6582	1.2786
28	2017	38	24.1857	80.1327	931.0010	3.3765	1.3888
39	2017	38	22.6429	87.3163	904.6878	2.6735	2.5898
24	2017	38	24.6857	79.7143	941.7214	2.7735	1.2908
63	2017	38	24.6143	84.6939	947.0561	4.3602	1.3092
62	2017	38	22.4286	75.1122	877.1684	4.3122	1.3357
1	2017	38	22.1143	78.5510	877.7694	3.1847	2.4398
31	2017	39	20.7143	78.7143	848.4796	5.1867	1.2316
79	2017	39	25.0286	78.9592	970.5184	3.8255	1.2153
51	2017	39	23.9143	82.1224	940.4031	4.3296	1.2847
14	2017	39	22.3571	86.4592	899.6449	2.9959	1.7133
67	2017	39	22.5571	86.6122	904.2388	3.0551	2.8622
42	2017	39	22.1143	78.5102	877.2408	3.9633	2.6724
50	2017	39	22.4286	81.5000	903.4051	2.8571	1.7133
43	2017	39	22.1143	78.5102	877.2408	3.9633	2.6724
85	2017	39	23.2857	74.3878	911.5010	4.6449	1.8214
25	2017	39	26.2000	82.1837	977.5531	4.6306	1.0224
69	2017	39	25.2143	77.8980	940.9663	4.2347	1.3959
57	2017	39	22.5857	80.0408	888.6541	5.0388	2.9673
9	2017	39	21.2429	78.0306	856.8357	5.1561	2.5745

72	2017	39	21.9286	80.2143	880.1214	4.0929	1.5633
26	2017	39	22.5000	86.3469	869.8816	5.7592	1.6143
7	2017	39	22.2143	79.3367	861.8755	5.8735	1.7694
83	2017	39	26.2429	78.5306	946.1163	5.9663	1.1908
76	2017	39	23.4286	79.8265	922.3255	3.9286	1.2490
36	2017	39	24.6429	78.5408	930.4612	4.0765	1.5276
81	2017	39	23.9143	82.1224	940.4031	4.3296	1.2847
15	2017	39	22.3143	83.0816	919.8112	2.8010	1.4786
32	2017	39	22.1143	78.5102	877.2408	3.9633	2.6724
73	2017	39	25.3143	78.5714	962.6847	3.3582	0.8010
71	2017	39	24.6429	78.5408	930.4612	4.0765	1.5276
41	2017	39	22.8286	73.4694	876.3888	4.7337	1.7531
10	2017	39	24.9571	82.4898	965.1010	4.3388	1.1286
23	2017	39	17.9429	83.3061	779.2592	4.1082	1.6612
27	2017	39	22.2143	79.3367	861.8755	5.8735	1.7694
60	2017	39	23.9143	82.1224	940.4031	4.3296	1.2847
53	2017	39	21.2429	78.0306	856.8357	5.1561	2.5745
66	2017	39	22.3571	86.4592	899.6449	2.9959	1.7133
59	2017	39	22.5857	80.0408	888.6541	5.0388	2.9673
61	2017	39	25.3143	78.5714	962.6847	3.3582	0.8010
84	2017	39	25.3143	78.5714	962.6847	3.3582	0.8010
38	2017	39	22.5857	80.0408	888.6541	5.0388	2.9673
87	2017	39	23.7714	79.2449	901.5684	5.0490	1.4255
34	2017	39	22.5857	80.0408	888.6541	5.0388	2.9673
29	2017	39	25.2143	77.8980	940.9663	4.2347	1.3959
5	2017	39	21.0857	81.0816	835.2663	5.8796	1.4980
8	2017	39	21.2429	78.0306	856.8357	5.1561	2.5745
12	2017	39	21.0857	81.0816	835.2663	5.8796	1.4980
13	2017	39	26.2429	78.5306	946.1163	5.9663	1.1908
18	2017	39	24.0571	84.8878	964.9571	2.8867	0.9439
33	2017	39	22.4286	81.5000	903.4051	2.8571	1.7133
56	2017	39	26.2000	82.1837	977.5531	4.6306	1.0224
77	2017	39	22.3143	83.0816	919.8112	2.8010	1.4786
54	2017	39	21.0857	81.0816	835.2663	5.8796	1.4980
21	2017	39	22.4286	81.5000	903.4051	2.8571	1.7133
68	2017	39	25.0286	78.9592	970.5184	3.8255	1.2153
74	2017	39	25.3143	78.5714	962.6847	3.3582	0.8010
88	2017	39	22.1143	78.5102	877.2408	3.9633	2.6724
16	2017	39	23.4286	79.8265	922.3255	3.9286	1.2490
30	2017	39	22.3571	86.4592	899.6449	2.9959	1.7133
6	2017	39	25.0286	78.9592	970.5184	3.8255	1.2153
49	2017	39	25.2143	77.8980	940.9663	4.2347	1.3959
22	2017	39	22.1143	78.5102	877.2408	3.9633	2.6724
45	2017	39	20.4714	80.7653	820.6643	5.2612	1.9439
58	2017	39	25.2143	77.8980	940.9663	4.2347	1.3959
37	2017	39	25.0286	78.9592	970.5184	3.8255	1.2153
17	2017	39	22.5571	86.6122	904.2388	3.0551	2.8622
55	2017	39	21.9286	80.2143	880.1214	4.0929	1.5633
46	2017	39	23.4286	79.8265	922.3255	3.9286	1.2490
86	2017	39	22.5000	79.0612	868.4265	4.2592	1.9592

2	2017	39	22.5000	79.0612	868.4265	4.2592	1.9592
4	2017	39	22.4286	81.5000	903.4051	2.8571	1.7133
47	2017	39	26.0429	84.6939	959.9306	4.9133	0.6429
82	2017	39	22.1143	78.5102	877.2408	3.9633	2.6724
19	2017	39	26.3286	81.2347	961.5592	4.1265	1.0061
20	2017	39	21.2429	78.0306	856.8357	5.1561	2.5745
80	2017	39	22.1143	78.5102	877.2408	3.9633	2.6724
3	2017	39	26.2429	78.5306	946.1163	5.9663	1.1908
52	2017	39	22.5571	86.6122	904.2388	3.0551	2.8622
70	2017	39	23.2857	74.3878	911.5010	4.6449	1.8214
64	2017	39	17.9429	83.3061	779.2592	4.1082	1.6612
48	2017	39	22.3143	83.0816	919.8112	2.8010	1.4786
65	2017	39	22.5571	86.6122	904.2388	3.0551	2.8622
44	2017	39	23.2857	74.3878	911.5010	4.6449	1.8214
75	2017	39	17.9429	83.3061	779.2592	4.1082	1.6612
40	2017	39	24.4143	82.1531	946.7694	5.3806	1.3571
11	2017	39	21.9286	80.2143	880.1214	4.0929	1.5633
35	2017	39	23.9143	82.1224	940.4031	4.3296	1.2847
78	2017	39	23.7714	79.2449	901.5684	5.0490	1.4255
28	2017	39	24.6429	78.5408	930.4612	4.0765	1.5276
39	2017	39	22.5571	86.6122	904.2388	3.0551	2.8622
24	2017	39	25.2143	77.8980	940.9663	4.2347	1.3959
63	2017	39	24.4143	82.1531	946.7694	5.3806	1.3571
62	2017	39	22.8286	73.4694	876.3888	4.7337	1.7531
1	2017	39	22.1143	78.5102	877.2408	3.9633	2.6724
31	2017	40	19.8000	79.9898	848.5786	4.8276	1.2449
79	2017	40	19.3143	81.5408	971.7735	2.8878	1.4418
51	2017	40	18.6429	83.8061	941.3469	4.0571	1.6316
14	2017	40	22.4143	87.1735	900.3541	2.6755	2.2490
67	2017	40	21.9857	87.2245	904.8255	2.2459	2.9806
42	2017	40	22.7571	78.0918	877.6337	4.2235	2.9061
50	2017	40	20.5714	84.0510	903.7469	2.1847	1.8571
43	2017	40	22.7571	78.0918	877.6337	4.2235	2.9061
85	2017	40	21.2714	76.9898	911.8898	4.4398	2.2500
25	2017	40	24.7571	83.5204	978.5827	3.6571	1.1857
69	2017	40	22.0143	77.2755	941.6469	4.0245	1.5714
57	2017	40	20.5857	82.3367	889.0041	4.8480	3.4806
9	2017	40	21.2714	78.7857	857.1735	5.5939	2.8551
72	2017	40	21.8286	80.5204	880.5918	5.1102	1.8418
26	2017	40	21.7286	87.6327	870.2867	6.2684	1.8980
7	2017	40	21.9429	78.7959	862.2561	6.1408	2.1102
83	2017	40	25.7857	77.6429	946.6510	6.8082	1.1949
76	2017	40	20.4571	81.9388	922.8439	3.7520	1.4296
36	2017	40	21.6286	79.0102	931.1684	3.3337	1.6929
81	2017	40	18.6429	83.8061	941.3469	4.0571	1.6316
15	2017	40	19.8143	86.5204	920.3337	1.4204	1.5673
32	2017	40	22.7571	78.0918	877.6337	4.2235	2.9061
73	2017	40	21.9143	78.6327	963.5418	2.9367	0.9490
71	2017	40	21.6286	79.0102	931.1684	3.3337	1.6929
41	2017	40	21.5571	75.0918	876.5990	4.6816	1.9898

10	2017	40	19.7571	83.3776	966.2969	3.4582	1.2867
23	2017	40	17.7000	83.1531	779.2898	3.8551	2.1990
27	2017	40	21.9429	78.7959	862.2561	6.1408	2.1102
60	2017	40	18.6429	83.8061	941.3469	4.0571	1.6316
53	2017	40	21.2714	78.7857	857.1735	5.5939	2.8551
66	2017	40	22.4143	87.1735	900.3541	2.6755	2.2490
59	2017	40	20.5857	82.3367	889.0041	4.8480	3.4806
61	2017	40	21.9143	78.6327	963.5418	2.9367	0.9490
84	2017	40	21.9143	78.6327	963.5418	2.9367	0.9490
38	2017	40	20.5857	82.3367	889.0041	4.8480	3.4806
87	2017	40	23.9286	79.3367	902.0490	4.8949	1.6102
34	2017	40	20.5857	82.3367	889.0041	4.8480	3.4806
29	2017	40	22.0143	77.2755	941.6469	4.0245	1.5714
5	2017	40	20.9429	78.6020	835.5204	6.8867	1.6694
8	2017	40	21.2714	78.7857	857.1735	5.5939	2.8551
12	2017	40	20.9429	78.6020	835.5204	6.8867	1.6694
13	2017	40	25.7857	77.6429	946.6510	6.8082	1.1949
18	2017	40	19.3000	87.0306	966.1112	2.0122	1.0459
33	2017	40	20.5714	84.0510	903.7469	2.1847	1.8571
56	2017	40	24.7571	83.5204	978.5827	3.6571	1.1857
77	2017	40	19.8143	86.5204	920.3337	1.4204	1.5673
54	2017	40	20.9429	78.6020	835.5204	6.8867	1.6694
21	2017	40	20.5714	84.0510	903.7469	2.1847	1.8571
68	2017	40	19.3143	81.5408	971.7735	2.8878	1.4418
74	2017	40	21.9143	78.6327	963.5418	2.9367	0.9490
88	2017	40	22.7571	78.0918	877.6337	4.2235	2.9061
16	2017	40	20.4571	81.9388	922.8439	3.7520	1.4296
30	2017	40	22.4143	87.1735	900.3541	2.6755	2.2490
6	2017	40	19.3143	81.5408	971.7735	2.8878	1.4418
49	2017	40	22.0143	77.2755	941.6469	4.0245	1.5714
22	2017	40	22.7571	78.0918	877.6337	4.2235	2.9061
45	2017	40	20.1714	79.6531	820.9000	5.3755	2.2684
58	2017	40	22.0143	77.2755	941.6469	4.0245	1.5714
37	2017	40	19.3143	81.5408	971.7735	2.8878	1.4418
17	2017	40	21.9857	87.2245	904.8255	2.2459	2.9806
55	2017	40	21.8286	80.5204	880.5918	5.1102	1.8418
46	2017	40	20.4571	81.9388	922.8439	3.7520	1.4296
86	2017	40	21.7857	79.8163	868.6173	4.5020	2.4337
2	2017	40	21.7857	79.8163	868.6173	4.5020	2.4337
4	2017	40	20.5714	84.0510	903.7469	2.1847	1.8571
47	2017	40	25.8571	85.3163	960.6204	4.6245	0.5939
82	2017	40	22.7571	78.0918	877.6337	4.2235	2.9061
19	2017	40	25.4714	83.6531	962.4643	3.4908	1.0061
20	2017	40	21.2714	78.7857	857.1735	5.5939	2.8551
80	2017	40	22.7571	78.0918	877.6337	4.2235	2.9061
3	2017	40	25.7857	77.6429	946.6510	6.8082	1.1949
52	2017	40	21.9857	87.2245	904.8255	2.2459	2.9806
70	2017	40	21.2714	76.9898	911.8898	4.4398	2.2500
64	2017	40	17.7000	83.1531	779.2898	3.8551	2.1990
48	2017	40	19.8143	86.5204	920.3337	1.4204	1.5673

65	2017	40	21.9857	87.2245	904.8255	2.2459	2.9806
44	2017	40	21.2714	76.9898	911.8898	4.4398	2.2500
75	2017	40	17.7000	83.1531	779.2898	3.8551	2.1990
40	2017	40	20.4714	84.2755	947.8582	4.7153	1.5194
11	2017	40	21.8286	80.5204	880.5918	5.1102	1.8418
35	2017	40	18.6429	83.8061	941.3469	4.0571	1.6316
78	2017	40	23.9286	79.3367	902.0490	4.8949	1.6102
28	2017	40	21.6286	79.0102	931.1684	3.3337	1.6929
39	2017	40	21.9857	87.2245	904.8255	2.2459	2.9806
24	2017	40	22.0143	77.2755	941.6469	4.0245	1.5714
63	2017	40	20.4714	84.2755	947.8582	4.7153	1.5194
62	2017	40	21.5571	75.0918	876.5990	4.6816	1.9898
1	2017	40	22.7571	78.0918	877.6337	4.2235	2.9061
31	2017	41	15.1000	80.7347	850.1643	4.0337	1.2296
79	2017	41	16.8571	84.6327	975.3010	1.0796	1.6316
51	2017	41	15.6000	89.0714	944.1153	1.4949	1.7806
14	2017	41	17.1571	86.8776	901.8735	3.1490	2.2000
67	2017	41	15.6571	89.3571	906.6439	2.2357	2.5867
42	2017	41	15.6714	79.8265	879.2327	3.9653	2.7724
50	2017	41	16.3143	86.7551	905.9327	2.1378	1.9857
43	2017	41	15.6714	79.8265	879.2327	3.9653	2.7724
85	2017	41	16.9714	79.7755	914.1265	3.6939	2.2561
25	2017	41	20.5000	83.0000	980.9796	3.0439	1.2939
69	2017	41	17.5714	79.5612	944.2714	2.7990	1.7908
57	2017	41	15.4000	85.9796	890.9235	3.7122	2.9235
9	2017	41	15.7429	80.9388	858.6214	5.1959	2.7000
72	2017	41	16.8000	82.5204	882.0449	4.8051	1.8796
26	2017	41	18.1714	89.1531	871.6184	5.2806	1.7510
7	2017	41	17.3286	80.4082	863.6204	5.0418	2.0133
83	2017	41	21.6857	78.9694	948.3337	5.8663	1.1459
76	2017	41	16.4000	85.6633	925.3490	2.9255	1.4694
36	2017	41	16.7429	82.7959	933.4255	2.6857	1.7551
81	2017	41	15.6000	89.0714	944.1153	1.4949	1.7806
15	2017	41	16.2857	88.8163	922.8010	1.1122	1.7663
32	2017	41	15.6714	79.8265	879.2327	3.9653	2.7724
73	2017	41	18.7714	78.9694	966.6459	1.8684	0.9786
71	2017	41	16.7429	82.7959	933.4255	2.6857	1.7551
41	2017	41	16.0429	77.9490	878.3776	3.7908	1.7367
10	2017	41	16.8857	87.6531	969.3469	1.2990	1.2367
23	2017	41	12.1429	83.4490	780.3776	3.5439	2.3337
27	2017	41	17.3286	80.4082	863.6204	5.0418	2.0133
60	2017	41	15.6000	89.0714	944.1153	1.4949	1.7806
53	2017	41	15.7429	80.9388	858.6214	5.1959	2.7000
66	2017	41	17.1571	86.8776	901.8735	3.1490	2.2000
59	2017	41	15.4000	85.9796	890.9235	3.7122	2.9235
61	2017	41	18.7714	78.9694	966.6459	1.8684	0.9786
84	2017	41	18.7714	78.9694	966.6459	1.8684	0.9786
38	2017	41	15.4000	85.9796	890.9235	3.7122	2.9235
87	2017	41	18.1000	79.2755	903.6776	4.6653	1.7214
34	2017	41	15.4000	85.9796	890.9235	3.7122	2.9235

29	2017	41	17.5714	79.5612	944.2714	2.7990	1.7908
5	2017	41	15.5571	81.2347	836.8122	5.9224	1.7020
8	2017	41	15.7429	80.9388	858.6214	5.1959	2.7000
12	2017	41	15.5571	81.2347	836.8122	5.9224	1.7020
13	2017	41	21.6857	78.9694	948.3337	5.8663	1.1459
18	2017	41	17.1571	88.1531	969.5224	0.2633	1.0612
33	2017	41	16.3143	86.7551	905.9327	2.1378	1.9857
56	2017	41	20.5000	83.0000	980.9796	3.0439	1.2939
77	2017	41	16.2857	88.8163	922.8010	1.1122	1.7663
54	2017	41	15.5571	81.2347	836.8122	5.9224	1.7020
21	2017	41	16.3143	86.7551	905.9327	2.1378	1.9857
68	2017	41	16.8571	84.6327	975.3010	1.0796	1.6316
74	2017	41	18.7714	78.9694	966.6459	1.8684	0.9786
88	2017	41	15.6714	79.8265	879.2327	3.9653	2.7724
16	2017	41	16.4000	85.6633	925.3490	2.9255	1.4694
30	2017	41	17.1571	86.8776	901.8735	3.1490	2.2000
6	2017	41	16.8571	84.6327	975.3010	1.0796	1.6316
49	2017	41	17.5714	79.5612	944.2714	2.7990	1.7908
22	2017	41	15.6714	79.8265	879.2327	3.9653	2.7724
45	2017	41	15.8429	80.5612	822.0949	4.7133	2.1816
58	2017	41	17.5714	79.5612	944.2714	2.7990	1.7908
37	2017	41	16.8571	84.6327	975.3010	1.0796	1.6316
17	2017	41	15.6571	89.3571	906.6439	2.2357	2.5867
55	2017	41	16.8000	82.5204	882.0449	4.8051	1.8796
46	2017	41	16.4000	85.6633	925.3490	2.9255	1.4694
86	2017	41	16.1143	82.4694	870.2367	4.1071	2.2714
2	2017	41	16.1143	82.4694	870.2367	4.1071	2.2714
4	2017	41	16.3143	86.7551	905.9327	2.1378	1.9857
47	2017	41	22.1000	84.6633	962.3704	4.4918	0.5102
82	2017	41	15.6714	79.8265	879.2327	3.9653	2.7724
19	2017	41	21.3857	83.1122	964.2847	3.4408	1.1214
20	2017	41	15.7429	80.9388	858.6214	5.1959	2.7000
80	2017	41	15.6714	79.8265	879.2327	3.9653	2.7724
3	2017	41	21.6857	78.9694	948.3337	5.8663	1.1459
52	2017	41	15.6571	89.3571	906.6439	2.2357	2.5867
70	2017	41	16.9714	79.7755	914.1265	3.6939	2.2561
64	2017	41	12.1429	83.4490	780.3776	3.5439	2.3337
48	2017	41	16.2857	88.8163	922.8010	1.1122	1.7663
65	2017	41	15.6571	89.3571	906.6439	2.2357	2.5867
44	2017	41	16.9714	79.7755	914.1265	3.6939	2.2561
75	2017	41	12.1429	83.4490	780.3776	3.5439	2.3337
40	2017	41	16.3429	88.5612	950.3224	2.9378	1.6520
11	2017	41	16.8000	82.5204	882.0449	4.8051	1.8796
35	2017	41	15.6000	89.0714	944.1153	1.4949	1.7806
78	2017	41	18.1000	79.2755	903.6776	4.6653	1.7214
28	2017	41	16.7429	82.7959	933.4255	2.6857	1.7551
39	2017	41	15.6571	89.3571	906.6439	2.2357	2.5867
24	2017	41	17.5714	79.5612	944.2714	2.7990	1.7908
63	2017	41	16.3429	88.5612	950.3224	2.9378	1.6520
62	2017	41	16.0429	77.9490	878.3776	3.7908	1.7367

1	2017	41	15.6714	79.8265	879.2327	3.9653	2.7724
31	2017	42	12.7000	85.0102	851.9143	2.0112	1.0459
79	2017	42	15.7571	85.9898	978.2510	0.3980	1.4480
51	2017	42	14.0429	91.4184	946.6112	0.2071	1.7520
14	2017	42	13.5143	87.9388	903.0694	1.7673	2.0286
67	2017	42	13.0714	93.6837	908.4857	1.1551	2.7245
42	2017	42	12.9429	87.6735	880.8704	1.7653	2.6592
50	2017	42	14.6429	88.0918	908.4286	1.2653	1.6867
43	2017	42	12.9429	87.6735	880.8704	1.7653	2.6592
85	2017	42	14.9857	85.1633	916.7531	1.8143	1.6510
25	2017	42	17.0857	83.2245	983.6755	1.6133	1.2051
69	2017	42	15.4857	83.2245	947.1143	1.1633	1.5816
57	2017	42	13.1714	90.8469	893.0704	1.6265	2.2541
9	2017	42	12.7286	86.5816	859.8082	2.1898	2.6929
72	2017	42	14.0000	85.2347	883.2122	2.0194	2.2163
26	2017	42	15.1429	91.2143	872.5357	2.0286	1.3622
7	2017	42	14.2714	86.2857	864.5929	2.0031	1.5398
83	2017	42	18.3429	83.1224	950.2429	2.3949	0.9704
76	2017	42	14.7000	87.1224	928.0929	1.3449	1.2265
36	2017	42	14.3286	86.5510	935.8653	1.1214	1.9112
81	2017	42	14.0429	91.4184	946.6112	0.2071	1.7520
15	2017	42	14.7429	89.4592	925.5041	0.8184	1.5337
32	2017	42	12.9429	87.6735	880.8704	1.7653	2.6592
73	2017	42	16.6429	79.9898	969.8245	0.8112	0.8347
71	2017	42	14.3286	86.5510	935.8653	1.1214	1.9112
41	2017	42	13.5143	83.9286	880.4204	1.7837	1.4827
10	2017	42	15.1857	90.1224	972.0235	0.3020	1.0867
23	2017	42	10.2857	88.3571	781.0041	1.8490	1.9286
27	2017	42	14.2714	86.2857	864.5929	2.0031	1.5398
60	2017	42	14.0429	91.4184	946.6112	0.2071	1.7520
53	2017	42	12.7286	86.5816	859.8082	2.1898	2.6929
66	2017	42	13.5143	87.9388	903.0694	1.7673	2.0286
59	2017	42	13.1714	90.8469	893.0704	1.6265	2.2541
61	2017	42	16.6429	79.9898	969.8245	0.8112	0.8347
84	2017	42	16.6429	79.9898	969.8245	0.8112	0.8347
38	2017	42	13.1714	90.8469	893.0704	1.6265	2.2541
87	2017	42	14.9286	83.3776	905.4051	2.1694	2.4224
34	2017	42	13.1714	90.8469	893.0704	1.6265	2.2541
29	2017	42	15.4857	83.2245	947.1143	1.1633	1.5816
5	2017	42	12.6714	89.5612	837.6459	2.1796	1.4612
8	2017	42	12.7286	86.5816	859.8082	2.1898	2.6929
12	2017	42	12.6714	89.5612	837.6459	2.1796	1.4612
13	2017	42	18.3429	83.1224	950.2429	2.3949	0.9704
18	2017	42	15.7429	88.3469	972.4418	0.1816	1.0990
33	2017	42	14.6429	88.0918	908.4286	1.2653	1.6867
56	2017	42	17.0857	83.2245	983.6755	1.6133	1.2051
77	2017	42	14.7429	89.4592	925.5041	0.8184	1.5337
54	2017	42	12.6714	89.5612	837.6459	2.1796	1.4612
21	2017	42	14.6429	88.0918	908.4286	1.2653	1.6867
68	2017	42	15.7571	85.9898	978.2510	0.3980	1.4480

74	2017	42	16.6429	79.9898	969.8245	0.8112	0.8347
88	2017	42	12.9429	87.6735	880.8704	1.7653	2.6592
16	2017	42	14.7000	87.1224	928.0929	1.3449	1.2265
30	2017	42	13.5143	87.9388	903.0694	1.7673	2.0286
6	2017	42	15.7571	85.9898	978.2510	0.3980	1.4480
49	2017	42	15.4857	83.2245	947.1143	1.1633	1.5816
22	2017	42	12.9429	87.6735	880.8704	1.7653	2.6592
45	2017	42	13.2571	86.8878	822.6918	2.1286	1.6347
58	2017	42	15.4857	83.2245	947.1143	1.1633	1.5816
37	2017	42	15.7571	85.9898	978.2510	0.3980	1.4480
17	2017	42	13.0714	93.6837	908.4857	1.1551	2.7245
55	2017	42	14.0000	85.2347	883.2122	2.0194	2.2163
46	2017	42	14.7000	87.1224	928.0929	1.3449	1.2265
86	2017	42	13.5571	90.3265	872.0204	1.7786	1.4867
2	2017	42	13.5571	90.3265	872.0204	1.7786	1.4867
4	2017	42	14.6429	88.0918	908.4286	1.2653	1.6867
47	2017	42	17.9857	85.3571	964.5459	2.3612	0.5571
82	2017	42	12.9429	87.6735	880.8704	1.7653	2.6592
19	2017	42	17.3571	81.2755	966.2908	1.7816	1.3561
20	2017	42	12.7286	86.5816	859.8082	2.1898	2.6929
80	2017	42	12.9429	87.6735	880.8704	1.7653	2.6592
3	2017	42	18.3429	83.1224	950.2429	2.3949	0.9704
52	2017	42	13.0714	93.6837	908.4857	1.1551	2.7245
70	2017	42	14.9857	85.1633	916.7531	1.8143	1.6510
64	2017	42	10.2857	88.3571	781.0041	1.8490	1.9286
48	2017	42	14.7429	89.4592	925.5041	0.8184	1.5337
65	2017	42	13.0714	93.6837	908.4857	1.1551	2.7245
44	2017	42	14.9857	85.1633	916.7531	1.8143	1.6510
75	2017	42	10.2857	88.3571	781.0041	1.8490	1.9286
40	2017	42	14.0000	91.8469	952.3990	1.0673	1.9571
11	2017	42	14.0000	85.2347	883.2122	2.0194	2.2163
35	2017	42	14.0429	91.4184	946.6112	0.2071	1.7520
78	2017	42	14.9286	83.3776	905.4051	2.1694	2.4224
28	2017	42	14.3286	86.5510	935.8653	1.1214	1.9112
39	2017	42	13.0714	93.6837	908.4857	1.1551	2.7245
24	2017	42	15.4857	83.2245	947.1143	1.1633	1.5816
63	2017	42	14.0000	91.8469	952.3990	1.0673	1.9571
62	2017	42	13.5143	83.9286	880.4204	1.7837	1.4827
1	2017	42	12.9429	87.6735	880.8704	1.7653	2.6592
31	2017	43	12.5429	90.0306	853.1602	0.2439	0.8031
79	2017	43	17.9571	81.8367	979.5092	1.4490	1.2071
51	2017	43	16.3429	85.4286	947.8878	1.6459	1.5551
14	2017	43	15.4714	84.4796	904.3224	1.1929	1.7857
67	2017	43	15.2714	89.8265	909.8469	0.8265	2.7908
42	2017	43	14.5571	86.8367	882.1173	0.6378	2.4765
50	2017	43	14.6857	88.0612	910.0459	0.4347	1.3480
43	2017	43	14.5571	86.8367	882.1173	0.6378	2.4765
85	2017	43	15.3000	87.9796	918.5633	0.2010	1.2561
25	2017	43	19.2286	82.1531	985.5918	1.2816	0.9214
69	2017	43	16.8143	81.9592	948.7388	0.9265	1.1602

57	2017	43	14.7000	89.7143	894.5224	0.4051	1.7796
9	2017	43	13.6143	86.8878	860.8061	0.5541	2.4378
72	2017	43	14.8571	82.6429	884.3857	0.4092	2.1867
26	2017	43	15.2857	91.2143	873.6510	0.5173	1.1929
7	2017	43	14.3429	88.9694	865.6204	0.4173	1.2429
83	2017	43	19.4429	81.9082	952.1612	1.0347	0.8949
76	2017	43	15.0286	84.9082	929.6765	0.5684	1.0204
36	2017	43	16.8429	81.3776	937.3663	0.7306	1.8224
81	2017	43	16.3429	85.4286	947.8878	1.6459	1.5551
15	2017	43	14.6571	89.5102	927.2347	0.3939	1.2184
32	2017	43	14.5571	86.8367	882.1173	0.6378	2.4765
73	2017	43	17.7429	80.0612	971.5378	0.6398	0.6867
71	2017	43	16.8429	81.3776	937.3663	0.7306	1.8224
41	2017	43	14.7000	85.6633	881.8286	0.5531	1.1724
10	2017	43	16.9571	85.1531	973.3643	1.4908	1.0214
23	2017	43	9.6857	91.8367	781.3357	0.9796	1.3949
27	2017	43	14.3429	88.9694	865.6204	0.4173	1.2429
60	2017	43	16.3429	85.4286	947.8878	1.6459	1.5551
53	2017	43	13.6143	86.8878	860.8061	0.5541	2.4378
66	2017	43	15.4714	84.4796	904.3224	1.1929	1.7857
59	2017	43	14.7000	89.7143	894.5224	0.4051	1.7796
61	2017	43	17.7429	80.0612	971.5378	0.6398	0.6867
84	2017	43	17.7429	80.0612	971.5378	0.6398	0.6867
38	2017	43	14.7000	89.7143	894.5224	0.4051	1.7796
87	2017	43	16.3429	82.3367	906.8602	0.8980	2.2204
34	2017	43	14.7000	89.7143	894.5224	0.4051	1.7796
29	2017	43	16.8143	81.9592	948.7388	0.9265	1.1602
5	2017	43	12.5857	93.5510	838.4449	0.5378	1.3255
8	2017	43	13.6143	86.8878	860.8061	0.5541	2.4378
12	2017	43	12.5857	93.5510	838.4449	0.5378	1.3255
13	2017	43	19.4429	81.9082	952.1612	1.0347	0.8949
18	2017	43	17.2714	85.4286	973.7796	1.2051	1.0031
33	2017	43	14.6857	88.0612	910.0459	0.4347	1.3480
56	2017	43	19.2286	82.1531	985.5918	1.2816	0.9214
77	2017	43	14.6571	89.5102	927.2347	0.3939	1.2184
54	2017	43	12.5857	93.5510	838.4449	0.5378	1.3255
21	2017	43	14.6857	88.0612	910.0459	0.4347	1.3480
68	2017	43	17.9571	81.8367	979.5092	1.4490	1.2071
74	2017	43	17.7429	80.0612	971.5378	0.6398	0.6867
88	2017	43	14.5571	86.8367	882.1173	0.6378	2.4765
16	2017	43	15.0286	84.9082	929.6765	0.5684	1.0204
30	2017	43	15.4714	84.4796	904.3224	1.1929	1.7857
6	2017	43	17.9571	81.8367	979.5092	1.4490	1.2071
49	2017	43	16.8143	81.9592	948.7388	0.9265	1.1602
22	2017	43	14.5571	86.8367	882.1173	0.6378	2.4765
45	2017	43	12.9714	89.9796	823.2827	1.0204	1.2296
58	2017	43	16.8143	81.9592	948.7388	0.9265	1.1602
37	2017	43	17.9571	81.8367	979.5092	1.4490	1.2071
17	2017	43	15.2714	89.8265	909.8469	0.8265	2.7908
55	2017	43	14.8571	82.6429	884.3857	0.4092	2.1867

46	2017	43	15.0286	84.9082	929.6765	0.5684	1.0204
86	2017	43	14.4857	92.4898	873.3031	0.2653	0.9704
2	2017	43	14.4857	92.4898	873.3031	0.2653	0.9704
4	2017	43	14.6857	88.0612	910.0459	0.4347	1.3480
47	2017	43	19.5000	84.0816	966.6531	0.7837	0.5245
82	2017	43	14.5571	86.8367	882.1173	0.6378	2.4765
19	2017	43	19.7857	77.1837	968.2010	0.9316	1.2306
20	2017	43	13.6143	86.8878	860.8061	0.5541	2.4378
80	2017	43	14.5571	86.8367	882.1173	0.6378	2.4765
3	2017	43	19.4429	81.9082	952.1612	1.0347	0.8949
52	2017	43	15.2714	89.8265	909.8469	0.8265	2.7908
70	2017	43	15.3000	87.9796	918.5633	0.2010	1.2561
64	2017	43	9.6857	91.8367	781.3357	0.9796	1.3949
48	2017	43	14.6571	89.5102	927.2347	0.3939	1.2184
65	2017	43	15.2714	89.8265	909.8469	0.8265	2.7908
44	2017	43	15.3000	87.9796	918.5633	0.2010	1.2561
75	2017	43	9.6857	91.8367	781.3357	0.9796	1.3949
40	2017	43	16.7000	87.8163	953.7255	1.5990	1.6163
11	2017	43	14.8571	82.6429	884.3857	0.4092	2.1867
35	2017	43	16.3429	85.4286	947.8878	1.6459	1.5551
78	2017	43	16.3429	82.3367	906.8602	0.8980	2.2204
28	2017	43	16.8429	81.3776	937.3663	0.7306	1.8224
39	2017	43	15.2714	89.8265	909.8469	0.8265	2.7908
24	2017	43	16.8143	81.9592	948.7388	0.9265	1.1602
63	2017	43	16.7000	87.8163	953.7255	1.5990	1.6163
62	2017	43	14.7000	85.6633	881.8286	0.5531	1.1724
1	2017	43	14.5571	86.8367	882.1173	0.6378	2.4765
31	2017	44	11.9571	83.2245	854.5612	2.0908	0.8082
79	2017	44	15.8714	73.9490	980.7173	4.4480	1.1133
51	2017	44	14.1286	77.9898	949.2827	4.7020	1.3184
14	2017	44	14.1429	77.8469	906.1867	4.3173	1.2510
67	2017	44	14.0857	79.8367	911.4582	3.9612	2.3806
42	2017	44	13.6857	75.3265	883.7357	4.0082	2.3551
50	2017	44	13.9143	81.7449	911.5541	3.3357	1.3786
43	2017	44	13.6857	75.3265	883.7357	4.0082	2.3551
85	2017	44	15.4857	78.4082	920.0347	2.4633	1.2867
25	2017	44	17.0000	78.4184	987.2796	3.9663	0.9031
69	2017	44	14.8000	76.9694	950.2367	3.5020	1.1286
57	2017	44	13.2000	79.4694	896.0051	2.6531	1.6408
9	2017	44	13.0571	77.3367	862.4776	3.9347	1.9827
72	2017	44	13.4857	76.2041	886.2184	3.5837	1.6541
26	2017	44	14.0429	86.2959	875.5633	3.5745	1.3122
7	2017	44	13.1429	83.9694	867.4541	3.8143	1.1214
83	2017	44	16.8286	77.0918	954.3000	4.2571	1.0163
76	2017	44	14.2000	80.0918	931.1143	3.5449	1.0980
36	2017	44	15.2857	73.7449	938.8194	3.6816	1.4929
81	2017	44	14.1286	77.9898	949.2827	4.7020	1.3184
15	2017	44	14.4857	82.6633	928.7163	2.9622	1.3408
32	2017	44	13.6857	75.3265	883.7357	4.0082	2.3551
73	2017	44	16.3571	76.2551	972.9520	3.4122	0.6520

71	2017	44	15.2857	73.7449	938.8194	3.6816	1.4929
41	2017	44	13.3000	77.4286	883.3418	3.4163	1.1122
10	2017	44	14.9143	77.9898	974.7704	4.7684	1.0592
23	2017	44	9.2286	85.6531	782.6122	2.7163	1.3918
27	2017	44	13.1429	83.9694	867.4541	3.8143	1.1214
60	2017	44	14.1286	77.9898	949.2827	4.7020	1.3184
53	2017	44	13.0571	77.3367	862.4776	3.9347	1.9827
66	2017	44	14.1429	77.8469	906.1867	4.3173	1.2510
59	2017	44	13.2000	79.4694	896.0051	2.6531	1.6408
61	2017	44	16.3571	76.2551	972.9520	3.4122	0.6520
84	2017	44	16.3571	76.2551	972.9520	3.4122	0.6520
38	2017	44	13.2000	79.4694	896.0051	2.6531	1.6408
87	2017	44	14.2000	75.8673	908.6520	4.4071	1.4367
34	2017	44	13.2000	79.4694	896.0051	2.6531	1.6408
29	2017	44	14.8000	76.9694	950.2367	3.5020	1.1286
5	2017	44	11.9000	85.9082	840.1051	3.4990	1.4673
8	2017	44	13.0571	77.3367	862.4776	3.9347	1.9827
12	2017	44	11.9000	85.9082	840.1051	3.4990	1.4673
13	2017	44	16.8286	77.0918	954.3000	4.2571	1.0163
18	2017	44	15.2000	78.4796	975.0122	3.8020	0.7827
33	2017	44	13.9143	81.7449	911.5541	3.3357	1.3786
56	2017	44	17.0000	78.4184	987.2796	3.9663	0.9031
77	2017	44	14.4857	82.6633	928.7163	2.9622	1.3408
54	2017	44	11.9000	85.9082	840.1051	3.4990	1.4673
21	2017	44	13.9143	81.7449	911.5541	3.3357	1.3786
68	2017	44	15.8714	73.9490	980.7173	4.4480	1.1133
74	2017	44	16.3571	76.2551	972.9520	3.4122	0.6520
88	2017	44	13.6857	75.3265	883.7357	4.0082	2.3551
16	2017	44	14.2000	80.0918	931.1143	3.5449	1.0980
30	2017	44	14.1429	77.8469	906.1867	4.3173	1.2510
6	2017	44	15.8714	73.9490	980.7173	4.4480	1.1133
49	2017	44	14.8000	76.9694	950.2367	3.5020	1.1286
22	2017	44	13.6857	75.3265	883.7357	4.0082	2.3551
45	2017	44	11.4286	83.6939	824.9245	3.3745	1.2827
58	2017	44	14.8000	76.9694	950.2367	3.5020	1.1286
37	2017	44	15.8714	73.9490	980.7173	4.4480	1.1133
17	2017	44	14.0857	79.8367	911.4582	3.9612	2.3806
55	2017	44	13.4857	76.2041	886.2184	3.5837	1.6541
46	2017	44	14.2000	80.0918	931.1143	3.5449	1.0980
86	2017	44	13.2571	82.2653	874.8714	1.8112	0.9888
2	2017	44	13.2571	82.2653	874.8714	1.8112	0.9888
4	2017	44	13.9143	81.7449	911.5541	3.3357	1.3786
47	2017	44	16.9429	80.5918	968.7408	3.3439	0.4939
82	2017	44	13.6857	75.3265	883.7357	4.0082	2.3551
19	2017	44	17.2571	72.7449	970.1582	4.0643	0.9684
20	2017	44	13.0571	77.3367	862.4776	3.9347	1.9827
80	2017	44	13.6857	75.3265	883.7357	4.0082	2.3551
3	2017	44	16.8286	77.0918	954.3000	4.2571	1.0163
52	2017	44	14.0857	79.8367	911.4582	3.9612	2.3806
70	2017	44	15.4857	78.4082	920.0347	2.4633	1.2867

64	2017	44	9.2286	85.6531	782.6122	2.7163	1.3918
48	2017	44	14.4857	82.6633	928.7163	2.9622	1.3408
65	2017	44	14.0857	79.8367	911.4582	3.9612	2.3806
44	2017	44	15.4857	78.4082	920.0347	2.4633	1.2867
75	2017	44	9.2286	85.6531	782.6122	2.7163	1.3918
40	2017	44	14.5857	79.6020	955.3429	5.0959	1.0367
11	2017	44	13.4857	76.2041	886.2184	3.5837	1.6541
35	2017	44	14.1286	77.9898	949.2827	4.7020	1.3184
78	2017	44	14.2000	75.8673	908.6520	4.4071	1.4367
28	2017	44	15.2857	73.7449	938.8194	3.6816	1.4929
39	2017	44	14.0857	79.8367	911.4582	3.9612	2.3806
24	2017	44	14.8000	76.9694	950.2367	3.5020	1.1286
63	2017	44	14.5857	79.6020	955.3429	5.0959	1.0367
62	2017	44	13.3000	77.4286	883.3418	3.4163	1.1122
1	2017	44	13.6857	75.3265	883.7357	4.0082	2.3551
31	2017	45	13.1571	75.7245	854.9643	4.1500	0.8796
79	2017	45	14.8000	71.4082	981.1480	5.6510	1.0214
51	2017	45	13.8000	77.3265	949.7071	5.5939	1.1939
14	2017	45	14.5571	77.1735	906.6969	5.6204	1.1776
67	2017	45	13.8143	76.1531	911.8908	5.5000	2.2878
42	2017	45	15.0714	68.5000	884.1459	6.2704	2.3969
50	2017	45	14.5286	76.2143	911.8878	5.3286	1.3633
43	2017	45	15.0714	68.5000	884.1459	6.2704	2.3969
85	2017	45	15.4143	71.1020	920.3571	4.7347	1.1551
25	2017	45	17.7286	78.2857	987.8837	4.8245	0.9878
69	2017	45	15.0286	75.0408	950.6745	4.9214	1.2143
57	2017	45	14.1857	74.4592	896.3418	3.8816	1.6510
9	2017	45	15.6571	69.6531	862.9214	6.6949	1.9459
72	2017	45	15.7714	73.2449	886.7265	5.5071	1.4980
26	2017	45	16.4286	81.2143	876.1276	5.6969	1.4755
7	2017	45	16.1143	78.4082	867.9776	6.3969	1.1796
83	2017	45	19.1429	76.4796	954.9582	5.6847	1.0204
76	2017	45	14.4286	76.7449	931.4347	5.8704	1.1755
36	2017	45	14.5857	73.1020	939.2673	5.0888	1.4286
81	2017	45	13.8000	77.3265	949.7071	5.5939	1.1939
15	2017	45	13.5143	77.9694	929.0153	4.5786	1.3990
32	2017	45	15.0714	68.5000	884.1459	6.2704	2.3969
73	2017	45	16.2000	72.6429	973.3255	5.4602	0.6663
71	2017	45	14.5857	73.1020	939.2673	5.0888	1.4286
41	2017	45	14.5571	72.0204	883.7163	5.4418	1.2133
10	2017	45	15.1143	76.6531	975.2531	5.8786	1.1061
23	2017	45	12.0429	75.1429	783.2184	4.7265	1.4806
27	2017	45	16.1143	78.4082	867.9776	6.3969	1.1796
60	2017	45	13.8000	77.3265	949.7071	5.5939	1.1939
53	2017	45	15.6571	69.6531	862.9214	6.6949	1.9459
66	2017	45	14.5571	77.1735	906.6969	5.6204	1.1776
59	2017	45	14.1857	74.4592	896.3418	3.8816	1.6510
61	2017	45	16.2000	72.6429	973.3255	5.4602	0.6663
84	2017	45	16.2000	72.6429	973.3255	5.4602	0.6663
38	2017	45	14.1857	74.4592	896.3418	3.8816	1.6510

87	2017	45	16.7143	73.3265	909.1204	6.1429	1.3582
34	2017	45	14.1857	74.4592	896.3418	3.8816	1.6510
29	2017	45	15.0286	75.0408	950.6745	4.9214	1.2143
5	2017	45	14.8714	78.0204	840.6102	6.1459	1.4929
8	2017	45	15.6571	69.6531	862.9214	6.6949	1.9459
12	2017	45	14.8714	78.0204	840.6102	6.1459	1.4929
13	2017	45	19.1429	76.4796	954.9582	5.6847	1.0204
18	2017	45	14.4000	75.1122	975.3837	4.7551	0.7020
33	2017	45	14.5286	76.2143	911.8878	5.3286	1.3633
56	2017	45	17.7286	78.2857	987.8837	4.8245	0.9878
77	2017	45	13.5143	77.9694	929.0153	4.5786	1.3990
54	2017	45	14.8714	78.0204	840.6102	6.1459	1.4929
21	2017	45	14.5286	76.2143	911.8878	5.3286	1.3633
68	2017	45	14.8000	71.4082	981.1480	5.6510	1.0214
74	2017	45	16.2000	72.6429	973.3255	5.4602	0.6663
88	2017	45	15.0714	68.5000	884.1459	6.2704	2.3969
16	2017	45	14.4286	76.7449	931.4347	5.8704	1.1755
30	2017	45	14.5571	77.1735	906.6969	5.6204	1.1776
6	2017	45	14.8000	71.4082	981.1480	5.6510	1.0214
49	2017	45	15.0286	75.0408	950.6745	4.9214	1.2143
22	2017	45	15.0714	68.5000	884.1459	6.2704	2.3969
45	2017	45	14.0857	77.5714	825.5510	5.4071	1.2714
58	2017	45	15.0286	75.0408	950.6745	4.9214	1.2143
37	2017	45	14.8000	71.4082	981.1480	5.6510	1.0214
17	2017	45	13.8143	76.1531	911.8908	5.5000	2.2878
55	2017	45	15.7714	73.2449	886.7265	5.5071	1.4980
46	2017	45	14.4286	76.7449	931.4347	5.8704	1.1755
86	2017	45	15.6286	75.0510	875.2959	2.6714	1.1378
2	2017	45	15.6286	75.0510	875.2959	2.6714	1.1378
4	2017	45	14.5286	76.2143	911.8878	5.3286	1.3633
47	2017	45	18.9429	81.1939	969.3929	4.7724	0.4602
82	2017	45	15.0714	68.5000	884.1459	6.2704	2.3969
19	2017	45	18.2143	74.5816	970.7796	5.1469	0.9051
20	2017	45	15.6571	69.6531	862.9214	6.6949	1.9459
80	2017	45	15.0714	68.5000	884.1459	6.2704	2.3969
3	2017	45	19.1429	76.4796	954.9582	5.6847	1.0204
52	2017	45	13.8143	76.1531	911.8908	5.5000	2.2878
70	2017	45	15.4143	71.1020	920.3571	4.7347	1.1551
64	2017	45	12.0429	75.1429	783.2184	4.7265	1.4806
48	2017	45	13.5143	77.9694	929.0153	4.5786	1.3990
65	2017	45	13.8143	76.1531	911.8908	5.5000	2.2878
44	2017	45	15.4143	71.1020	920.3571	4.7347	1.1551
75	2017	45	12.0429	75.1429	783.2184	4.7265	1.4806
40	2017	45	14.6571	78.9694	955.8316	5.8745	0.9704
11	2017	45	15.7714	73.2449	886.7265	5.5071	1.4980
35	2017	45	13.8000	77.3265	949.7071	5.5939	1.1939
78	2017	45	16.7143	73.3265	909.1204	6.1429	1.3582
28	2017	45	14.5857	73.1020	939.2673	5.0888	1.4286
39	2017	45	13.8143	76.1531	911.8908	5.5000	2.2878
24	2017	45	15.0286	75.0408	950.6745	4.9214	1.2143

63	2017	45	14.6571	78.9694	955.8316	5.8745	0.9704
62	2017	45	14.5571	72.0204	883.7163	5.4418	1.2133
1	2017	45	15.0714	68.5000	884.1459	6.2704	2.3969
31	2017	46	11.5143	79.7245	852.0429	3.1633	0.9173
79	2017	46	11.9429	77.5816	979.0000	2.8398	1.1000
51	2017	46	10.5143	83.8571	947.3439	2.6622	1.3704
14	2017	46	12.4429	86.2551	903.9908	2.5531	1.5306
67	2017	46	11.0000	85.4286	909.1959	2.6918	2.1602
42	2017	46	11.4714	77.9796	881.2000	3.7041	2.4102
50	2017	46	11.8143	80.9694	908.9735	2.6786	1.4582
43	2017	46	11.4714	77.9796	881.2000	3.7041	2.4102
85	2017	46	12.6857	77.6633	917.4582	2.9939	1.2367
25	2017	46	15.4857	81.8367	985.4235	2.2418	0.9276
69	2017	46	12.6571	78.9286	948.0449	2.6204	1.2929
57	2017	46	10.4571	83.7755	893.4602	1.8398	1.7847
9	2017	46	12.3571	76.0714	859.9296	5.1888	2.2582
72	2017	46	14.0286	78.7551	883.6847	3.3847	1.7071
26	2017	46	17.5143	82.5408	872.9929	6.0031	1.8255
7	2017	46	16.4857	78.4796	864.8418	6.4480	1.7041
83	2017	46	19.9429	77.9286	951.4582	4.3459	1.0276
76	2017	46	10.9429	82.0000	928.7510	3.3724	1.1622
36	2017	46	11.6429	80.6633	936.7337	2.3949	1.5204
81	2017	46	10.5143	83.8571	947.3439	2.6622	1.3704
15	2017	46	11.4286	81.7959	926.2418	2.1949	1.4704
32	2017	46	11.4714	77.9796	881.2000	3.7041	2.4102
73	2017	46	13.4000	75.0204	970.8439	3.0214	0.6398
71	2017	46	11.6429	80.6633	936.7337	2.3949	1.5204
41	2017	46	11.5714	78.6429	880.7490	2.9694	1.2918
10	2017	46	11.8429	82.7653	973.0357	2.8296	1.0653
23	2017	46	10.5429	72.3980	780.5408	6.1020	1.6306
27	2017	46	16.4857	78.4796	864.8418	6.4480	1.7041
60	2017	46	10.5143	83.8571	947.3439	2.6622	1.3704
53	2017	46	12.3571	76.0714	859.9296	5.1888	2.2582
66	2017	46	12.4429	86.2551	903.9908	2.5531	1.5306
59	2017	46	10.4571	83.7755	893.4602	1.8398	1.7847
61	2017	46	13.4000	75.0204	970.8439	3.0214	0.6398
84	2017	46	13.4000	75.0204	970.8439	3.0214	0.6398
38	2017	46	10.4571	83.7755	893.4602	1.8398	1.7847
87	2017	46	14.4571	78.2857	906.0296	3.5541	1.5622
34	2017	46	10.4571	83.7755	893.4602	1.8398	1.7847
29	2017	46	12.6571	78.9286	948.0449	2.6204	1.2929
5	2017	46	14.0571	79.6429	837.6398	6.3449	1.4929
8	2017	46	12.3571	76.0714	859.9296	5.1888	2.2582
12	2017	46	14.0571	79.6429	837.6398	6.3449	1.4929
13	2017	46	19.9429	77.9286	951.4582	4.3459	1.0276
18	2017	46	12.0000	79.1327	973.1459	2.4092	0.8990
33	2017	46	11.8143	80.9694	908.9735	2.6786	1.4582
56	2017	46	15.4857	81.8367	985.4235	2.2418	0.9276
77	2017	46	11.4286	81.7959	926.2418	2.1949	1.4704
54	2017	46	14.0571	79.6429	837.6398	6.3449	1.4929

21	2017	46	11.8143	80.9694	908.9735	2.6786	1.4582
68	2017	46	11.9429	77.5816	979.0000	2.8398	1.1000
74	2017	46	13.4000	75.0204	970.8439	3.0214	0.6398
88	2017	46	11.4714	77.9796	881.2000	3.7041	2.4102
16	2017	46	10.9429	82.0000	928.7510	3.3724	1.1622
30	2017	46	12.4429	86.2551	903.9908	2.5531	1.5306
6	2017	46	11.9429	77.5816	979.0000	2.8398	1.1000
49	2017	46	12.6571	78.9286	948.0449	2.6204	1.2929
22	2017	46	11.4714	77.9796	881.2000	3.7041	2.4102
45	2017	46	15.0857	75.0714	822.6969	6.8306	1.5418
58	2017	46	12.6571	78.9286	948.0449	2.6204	1.2929
37	2017	46	11.9429	77.5816	979.0000	2.8398	1.1000
17	2017	46	11.0000	85.4286	909.1959	2.6918	2.1602
55	2017	46	14.0286	78.7551	883.6847	3.3847	1.7071
46	2017	46	10.9429	82.0000	928.7510	3.3724	1.1622
86	2017	46	12.5571	81.3367	872.2714	1.2888	1.3398
2	2017	46	12.5571	81.3367	872.2714	1.2888	1.3398
4	2017	46	11.8143	80.9694	908.9735	2.6786	1.4582
47	2017	46	18.4714	84.1020	966.0551	2.8633	0.4245
82	2017	46	11.4714	77.9796	881.2000	3.7041	2.4102
19	2017	46	16.5571	80.5408	968.1194	2.0745	1.1214
20	2017	46	12.3571	76.0714	859.9296	5.1888	2.2582
80	2017	46	11.4714	77.9796	881.2000	3.7041	2.4102
3	2017	46	19.9429	77.9286	951.4582	4.3459	1.0276
52	2017	46	11.0000	85.4286	909.1959	2.6918	2.1602
70	2017	46	12.6857	77.6633	917.4582	2.9939	1.2367
64	2017	46	10.5429	72.3980	780.5408	6.1020	1.6306
48	2017	46	11.4286	81.7959	926.2418	2.1949	1.4704
65	2017	46	11.0000	85.4286	909.1959	2.6918	2.1602
44	2017	46	12.6857	77.6633	917.4582	2.9939	1.2367
75	2017	46	10.5429	72.3980	780.5408	6.1020	1.6306
40	2017	46	10.9857	87.4286	953.5408	2.3969	1.2071
11	2017	46	14.0286	78.7551	883.6847	3.3847	1.7071
35	2017	46	10.5143	83.8571	947.3439	2.6622	1.3704
78	2017	46	14.4571	78.2857	906.0296	3.5541	1.5622
28	2017	46	11.6429	80.6633	936.7337	2.3949	1.5204
39	2017	46	11.0000	85.4286	909.1959	2.6918	2.1602
24	2017	46	12.6571	78.9286	948.0449	2.6204	1.2929
63	2017	46	10.9857	87.4286	953.5408	2.3969	1.2071
62	2017	46	11.5714	78.6429	880.7490	2.9694	1.2918
1	2017	46	11.4714	77.9796	881.2000	3.7041	2.4102
31	2017	47	4.9000	85.0510	851.4245	1.0622	0.9827
79	2017	47	10.0286	76.3776	980.0878	0.6643	1.4010
51	2017	47	8.0429	82.1327	947.9541	0.4857	1.5571
14	2017	47	7.1571	85.5306	903.6724	0.3816	2.1480
67	2017	47	6.8714	87.4490	909.1133	0.4622	2.5122
42	2017	47	6.3286	85.7347	880.7857	1.1010	2.4898
50	2017	47	6.5714	86.3878	909.0990	0.4163	1.6633
43	2017	47	6.3286	85.7347	880.7857	1.1010	2.4898
85	2017	47	7.1714	86.0204	917.7327	0.6776	1.3571

25	2017	47	10.6857	80.0918	986.2520	0.3776	0.9520
69	2017	47	9.5857	76.1327	948.5786	0.7133	1.4622
57	2017	47	6.1286	92.0408	893.3776	0.4561	1.8010
9	2017	47	5.8571	83.9286	859.1571	2.2918	2.5020
72	2017	47	6.9857	82.2959	883.0296	1.3061	2.3551
26	2017	47	7.5857	85.7347	872.0449	4.3184	1.9000
7	2017	47	6.9286	81.8980	863.9224	4.2786	1.9969
83	2017	47	11.7857	75.0204	951.2031	2.5224	1.2143
76	2017	47	8.2857	83.3061	929.1531	0.7929	1.1704
36	2017	47	8.4000	78.7143	937.1010	0.4204	1.8214
81	2017	47	8.0429	82.1327	947.9541	0.4857	1.5571
15	2017	47	7.7286	80.8878	926.5898	0.4276	1.5163
32	2017	47	6.3286	85.7347	880.7857	1.1010	2.4898
73	2017	47	10.8714	71.9082	971.7367	0.6082	0.5969
71	2017	47	8.4000	78.7143	937.1010	0.4204	1.8214
41	2017	47	6.4000	85.4694	880.4194	0.4745	1.2469
10	2017	47	9.0286	82.3980	973.9398	0.8010	1.0908
23	2017	47	1.3286	80.7755	778.9694	4.1684	1.7408
27	2017	47	6.9286	81.8980	863.9224	4.2786	1.9969
60	2017	47	8.0429	82.1327	947.9541	0.4857	1.5571
53	2017	47	5.8571	83.9286	859.1571	2.2918	2.5020
66	2017	47	7.1571	85.5306	903.6724	0.3816	2.1480
59	2017	47	6.1286	92.0408	893.3776	0.4561	1.8010
61	2017	47	10.8714	71.9082	971.7367	0.6082	0.5969
84	2017	47	10.8714	71.9082	971.7367	0.6082	0.5969
38	2017	47	6.1286	92.0408	893.3776	0.4561	1.8010
87	2017	47	8.4143	79.0306	905.7582	1.1622	2.3755
34	2017	47	6.1286	92.0408	893.3776	0.4561	1.8010
29	2017	47	9.5857	76.1327	948.5786	0.7133	1.4622
5	2017	47	5.0143	86.1735	836.5235	3.9092	1.5673
8	2017	47	5.8571	83.9286	859.1571	2.2918	2.5020
12	2017	47	5.0143	86.1735	836.5235	3.9092	1.5673
13	2017	47	11.7857	75.0204	951.2031	2.5224	1.2143
18	2017	47	9.7143	78.4388	974.1745	0.6898	1.0500
33	2017	47	6.5714	86.3878	909.0990	0.4163	1.6633
56	2017	47	10.6857	80.0918	986.2520	0.3776	0.9520
77	2017	47	7.7286	80.8878	926.5898	0.4276	1.5163
54	2017	47	5.0143	86.1735	836.5235	3.9092	1.5673
21	2017	47	6.5714	86.3878	909.0990	0.4163	1.6633
68	2017	47	10.0286	76.3776	980.0878	0.6643	1.4010
74	2017	47	10.8714	71.9082	971.7367	0.6082	0.5969
88	2017	47	6.3286	85.7347	880.7857	1.1010	2.4898
16	2017	47	8.2857	83.3061	929.1531	0.7929	1.1704
30	2017	47	7.1571	85.5306	903.6724	0.3816	2.1480
6	2017	47	10.0286	76.3776	980.0878	0.6643	1.4010
49	2017	47	9.5857	76.1327	948.5786	0.7133	1.4622
22	2017	47	6.3286	85.7347	880.7857	1.1010	2.4898
45	2017	47	5.1857	77.6122	821.3357	4.9633	1.8653
58	2017	47	9.5857	76.1327	948.5786	0.7133	1.4622
37	2017	47	10.0286	76.3776	980.0878	0.6643	1.4010

17	2017	47	6.8714	87.4490	909.1133	0.4622	2.5122
55	2017	47	6.9857	82.2959	883.0296	1.3061	2.3551
46	2017	47	8.2857	83.3061	929.1531	0.7929	1.1704
86	2017	47	6.3286	90.2857	871.7857	0.2296	1.2602
2	2017	47	6.3286	90.2857	871.7857	0.2296	1.2602
4	2017	47	6.5714	86.3878	909.0990	0.4163	1.6633
47	2017	47	11.9429	78.4592	966.1582	1.2122	0.5571
82	2017	47	6.3286	85.7347	880.7857	1.1010	2.4898
19	2017	47	10.6429	78.0102	968.5592	0.3031	1.4898
20	2017	47	5.8571	83.9286	859.1571	2.2918	2.5020
80	2017	47	6.3286	85.7347	880.7857	1.1010	2.4898
3	2017	47	11.7857	75.0204	951.2031	2.5224	1.2143
52	2017	47	6.8714	87.4490	909.1133	0.4622	2.5122
70	2017	47	7.1714	86.0204	917.7327	0.6776	1.3571
64	2017	47	1.3286	80.7755	778.9694	4.1684	1.7408
48	2017	47	7.7286	80.8878	926.5898	0.4276	1.5163
65	2017	47	6.8714	87.4490	909.1133	0.4622	2.5122
44	2017	47	7.1714	86.0204	917.7327	0.6776	1.3571
75	2017	47	1.3286	80.7755	778.9694	4.1684	1.7408
40	2017	47	7.6571	88.8571	954.0980	0.3051	1.5765
11	2017	47	6.9857	82.2959	883.0296	1.3061	2.3551
35	2017	47	8.0429	82.1327	947.9541	0.4857	1.5571
78	2017	47	8.4143	79.0306	905.7582	1.1622	2.3755
28	2017	47	8.4000	78.7143	937.1010	0.4204	1.8214
39	2017	47	6.8714	87.4490	909.1133	0.4622	2.5122
24	2017	47	9.5857	76.1327	948.5786	0.7133	1.4622
63	2017	47	7.6571	88.8571	954.0980	0.3051	1.5765
62	2017	47	6.4000	85.4694	880.4194	0.4745	1.2469
1	2017	47	6.3286	85.7347	880.7857	1.1010	2.4898
31	2017	48	6.3714	84.3878	853.2153	0.5316	0.9551
79	2017	48	9.7857	69.9184	982.4633	1.3214	1.3092
51	2017	48	8.6429	73.7959	950.1786	1.3071	1.3939
14	2017	48	10.2000	75.3776	905.7541	1.3643	2.2327
67	2017	48	8.5000	78.9592	911.1959	1.2827	2.6714
42	2017	48	8.4143	79.4796	882.8122	1.6194	2.3092
50	2017	48	8.4857	83.7347	911.4429	0.8571	1.4398
43	2017	48	8.4143	79.4796	882.8122	1.6194	2.3092
85	2017	48	8.9714	83.8163	920.2582	0.9429	1.3684
25	2017	48	13.1429	76.7143	988.9857	1.1490	0.9786
69	2017	48	10.0857	69.7653	950.9449	1.7143	1.4082
57	2017	48	7.8429	86.8367	895.5071	1.3000	1.8745
9	2017	48	8.8429	81.2857	861.0020	1.3990	2.4959
72	2017	48	10.4286	79.3776	885.1745	1.3122	2.2010
26	2017	48	12.4429	86.6327	874.1857	2.6602	1.6755
7	2017	48	11.8286	82.7143	865.9265	2.6500	1.7633
83	2017	48	15.2429	73.2857	954.4133	2.1867	1.1602
76	2017	48	8.4429	76.7347	931.4071	1.4286	1.1520
36	2017	48	9.1857	70.2755	939.3510	1.1143	1.7378
81	2017	48	8.6429	73.7959	950.1786	1.3071	1.3939
15	2017	48	8.5286	77.3367	928.9367	0.9857	1.3327

32	2017	48	8.4143	79.4796	882.8122	1.6194	2.3092
73	2017	48	10.6000	67.5612	974.2888	1.2286	0.5592
71	2017	48	9.1857	70.2755	939.3510	1.1143	1.7378
41	2017	48	8.0286	81.4490	882.4918	0.9143	1.1092
10	2017	48	9.8143	76.2041	976.2888	1.4214	1.0765
23	2017	48	6.0571	85.9184	779.7561	2.7010	1.5429
27	2017	48	11.8286	82.7143	865.9265	2.6500	1.7633
60	2017	48	8.6429	73.7959	950.1786	1.3071	1.3939
53	2017	48	8.8429	81.2857	861.0020	1.3990	2.4959
66	2017	48	10.2000	75.3776	905.7541	1.3643	2.2327
59	2017	48	7.8429	86.8367	895.5071	1.3000	1.8745
61	2017	48	10.6000	67.5612	974.2888	1.2286	0.5592
84	2017	48	10.6000	67.5612	974.2888	1.2286	0.5592
38	2017	48	7.8429	86.8367	895.5071	1.3000	1.8745
87	2017	48	11.3286	73.8571	908.1286	1.4184	2.4867
34	2017	48	7.8429	86.8367	895.5071	1.3000	1.8745
29	2017	48	10.0857	69.7653	950.9449	1.7143	1.4082
5	2017	48	9.8857	87.4388	838.1031	2.4500	1.5449
8	2017	48	8.8429	81.2857	861.0020	1.3990	2.4959
12	2017	48	9.8857	87.4388	838.1031	2.4500	1.5449
13	2017	48	15.2429	73.2857	954.4133	2.1867	1.1602
18	2017	48	9.5857	74.9184	976.5082	1.1429	0.9337
33	2017	48	8.4857	83.7347	911.4429	0.8571	1.4398
56	2017	48	13.1429	76.7143	988.9857	1.1490	0.9786
77	2017	48	8.5286	77.3367	928.9367	0.9857	1.3327
54	2017	48	9.8857	87.4388	838.1031	2.4500	1.5449
21	2017	48	8.4857	83.7347	911.4429	0.8571	1.4398
68	2017	48	9.7857	69.9184	982.4633	1.3214	1.3092
74	2017	48	10.6000	67.5612	974.2888	1.2286	0.5592
88	2017	48	8.4143	79.4796	882.8122	1.6194	2.3092
16	2017	48	8.4429	76.7347	931.4071	1.4286	1.1520
30	2017	48	10.2000	75.3776	905.7541	1.3643	2.2327
6	2017	48	9.7857	69.9184	982.4633	1.3214	1.3092
49	2017	48	10.0857	69.7653	950.9449	1.7143	1.4082
22	2017	48	8.4143	79.4796	882.8122	1.6194	2.3092
45	2017	48	10.9571	80.9286	822.6908	2.6459	1.7980
58	2017	48	10.0857	69.7653	950.9449	1.7143	1.4082
37	2017	48	9.7857	69.9184	982.4633	1.3214	1.3092
17	2017	48	8.5000	78.9592	911.1959	1.2827	2.6714
55	2017	48	10.4286	79.3776	885.1745	1.3122	2.2010
46	2017	48	8.4429	76.7347	931.4071	1.4286	1.1520
86	2017	48	8.6857	87.9082	873.8378	0.3214	1.2184
2	2017	48	8.6857	87.9082	873.8378	0.3214	1.2184
4	2017	48	8.4857	83.7347	911.4429	0.8571	1.4398
47	2017	48	15.3143	74.1327	969.3449	1.6469	0.5531
82	2017	48	8.4143	79.4796	882.8122	1.6194	2.3092
19	2017	48	14.1714	73.3061	971.3541	1.2735	1.2643
20	2017	48	8.8429	81.2857	861.0020	1.3990	2.4959
80	2017	48	8.4143	79.4796	882.8122	1.6194	2.3092
3	2017	48	15.2429	73.2857	954.4133	2.1867	1.1602

52	2017	48	8.5000	78.9592	911.1959	1.2827	2.6714
70	2017	48	8.9714	83.8163	920.2582	0.9429	1.3684
64	2017	48	6.0571	85.9184	779.7561	2.7010	1.5429
48	2017	48	8.5286	77.3367	928.9367	0.9857	1.3327
65	2017	48	8.5000	78.9592	911.1959	1.2827	2.6714
44	2017	48	8.9714	83.8163	920.2582	0.9429	1.3684
75	2017	48	6.0571	85.9184	779.7561	2.7010	1.5429
40	2017	48	9.5286	82.7245	956.3286	1.0929	1.5031
11	2017	48	10.4286	79.3776	885.1745	1.3122	2.2010
35	2017	48	8.6429	73.7959	950.1786	1.3071	1.3939
78	2017	48	11.3286	73.8571	908.1286	1.4184	2.4867
28	2017	48	9.1857	70.2755	939.3510	1.1143	1.7378
39	2017	48	8.5000	78.9592	911.1959	1.2827	2.6714
24	2017	48	10.0857	69.7653	950.9449	1.7143	1.4082
63	2017	48	9.5286	82.7245	956.3286	1.0929	1.5031
62	2017	48	8.0286	81.4490	882.4918	0.9143	1.1092
1	2017	48	8.4143	79.4796	882.8122	1.6194	2.3092
31	2017	49	6.1000	86.0204	853.2724	0.4469	0.9173
79	2017	49	9.6571	71.5306	982.7061	1.3561	1.2153
51	2017	49	7.7857	76.0510	950.4184	1.3224	1.3520
14	2017	49	8.3286	79.5714	906.0786	1.5092	1.8398
67	2017	49	7.8429	83.3673	911.4908	1.5224	2.4469
42	2017	49	7.1429	81.2041	882.9816	1.6908	2.2786
50	2017	49	7.8286	84.2449	911.5908	0.8592	1.2388
43	2017	49	7.1429	81.2041	882.9816	1.6908	2.2786
85	2017	49	8.7714	83.5204	920.3969	0.9500	1.2490
25	2017	49	11.3429	78.9796	989.2745	1.3469	0.8459
69	2017	49	9.5857	72.6429	951.1867	1.7071	1.3143
57	2017	49	6.9143	87.7857	895.6388	1.2663	1.8551
9	2017	49	6.5286	83.3367	861.1857	1.1735	2.3755
72	2017	49	7.8286	81.4286	885.4500	1.3327	1.8500
26	2017	49	8.6857	89.1633	874.4061	2.3173	1.6745
7	2017	49	8.0571	84.6327	866.1000	2.4418	1.8388
83	2017	49	13.4000	74.0816	954.7561	1.9408	0.9571
76	2017	49	7.8286	79.7347	931.6163	1.3612	1.1112
36	2017	49	8.5857	75.1939	939.6214	1.2378	1.4949
81	2017	49	7.7857	76.0510	950.4184	1.3224	1.3520
15	2017	49	7.7286	81.2245	929.0847	1.0673	1.2918
32	2017	49	7.1429	81.2041	882.9816	1.6908	2.2786
73	2017	49	10.1857	72.9796	974.5837	1.2592	0.5735
71	2017	49	8.5857	75.1939	939.6214	1.2378	1.4949
41	2017	49	7.0857	82.3878	882.6500	0.9510	1.0918
10	2017	49	9.2143	77.5204	976.5327	1.4133	0.9122
23	2017	49	3.1000	87.1224	779.7204	2.8878	1.4214
27	2017	49	8.0571	84.6327	866.1000	2.4418	1.8388
60	2017	49	7.7857	76.0510	950.4184	1.3224	1.3520
53	2017	49	6.5286	83.3367	861.1857	1.1735	2.3755
66	2017	49	8.3286	79.5714	906.0786	1.5092	1.8398
59	2017	49	6.9143	87.7857	895.6388	1.2663	1.8551
61	2017	49	10.1857	72.9796	974.5837	1.2592	0.5735

84	2017	49	10.1857	72.9796	974.5837	1.2592	0.5735
38	2017	49	6.9143	87.7857	895.6388	1.2663	1.8551
87	2017	49	9.3714	76.5000	908.3724	1.5204	1.9306
34	2017	49	6.9143	87.7857	895.6388	1.2663	1.8551
29	2017	49	9.5857	72.6429	951.1867	1.7071	1.3143
5	2017	49	6.2000	89.5102	838.1969	2.2990	1.5204
8	2017	49	6.5286	83.3367	861.1857	1.1735	2.3755
12	2017	49	6.2000	89.5102	838.1969	2.2990	1.5204
13	2017	49	13.4000	74.0816	954.7561	1.9408	0.9571
18	2017	49	9.1286	77.4490	976.7194	1.2459	0.8755
33	2017	49	7.8286	84.2449	911.5908	0.8592	1.2388
56	2017	49	11.3429	78.9796	989.2745	1.3469	0.8459
77	2017	49	7.7286	81.2245	929.0847	1.0673	1.2918
54	2017	49	6.2000	89.5102	838.1969	2.2990	1.5204
21	2017	49	7.8286	84.2449	911.5908	0.8592	1.2388
68	2017	49	9.6571	71.5306	982.7061	1.3561	1.2153
74	2017	49	10.1857	72.9796	974.5837	1.2592	0.5735
88	2017	49	7.1429	81.2041	882.9816	1.6908	2.2786
16	2017	49	7.8286	79.7347	931.6163	1.3612	1.1112
30	2017	49	8.3286	79.5714	906.0786	1.5092	1.8398
6	2017	49	9.6571	71.5306	982.7061	1.3561	1.2153
49	2017	49	9.5857	72.6429	951.1867	1.7071	1.3143
22	2017	49	7.1429	81.2041	882.9816	1.6908	2.2786
45	2017	49	6.6714	83.1939	822.7592	2.7847	1.5418
58	2017	49	9.5857	72.6429	951.1867	1.7071	1.3143
37	2017	49	9.6571	71.5306	982.7061	1.3561	1.2153
17	2017	49	7.8429	83.3673	911.4908	1.5224	2.4469
55	2017	49	7.8286	81.4286	885.4500	1.3327	1.8500
46	2017	49	7.8286	79.7347	931.6163	1.3612	1.1112
86	2017	49	7.1714	88.6122	873.9816	0.2673	1.0551
2	2017	49	7.1714	88.6122	873.9816	0.2673	1.0551
4	2017	49	7.8286	84.2449	911.5908	0.8592	1.2388
47	2017	49	13.2857	75.5510	969.6622	1.7531	0.4643
82	2017	49	7.1429	81.2041	882.9816	1.6908	2.2786
19	2017	49	12.2286	73.6531	971.6816	1.3510	1.0888
20	2017	49	6.5286	83.3367	861.1857	1.1735	2.3755
80	2017	49	7.1429	81.2041	882.9816	1.6908	2.2786
3	2017	49	13.4000	74.0816	954.7561	1.9408	0.9571
52	2017	49	7.8429	83.3673	911.4908	1.5224	2.4469
70	2017	49	8.7714	83.5204	920.3969	0.9500	1.2490
64	2017	49	3.1000	87.1224	779.7204	2.8878	1.4214
48	2017	49	7.7286	81.2245	929.0847	1.0673	1.2918
65	2017	49	7.8429	83.3673	911.4908	1.5224	2.4469
44	2017	49	8.7714	83.5204	920.3969	0.9500	1.2490
75	2017	49	3.1000	87.1224	779.7204	2.8878	1.4214
40	2017	49	8.5286	82.8163	956.6255	1.2408	1.3592
11	2017	49	7.8286	81.4286	885.4500	1.3327	1.8500
35	2017	49	7.7857	76.0510	950.4184	1.3224	1.3520
78	2017	49	9.3714	76.5000	908.3724	1.5204	1.9306
28	2017	49	8.5857	75.1939	939.6214	1.2378	1.4949

39	2017	49	7.8429	83.3673	911.4908	1.5224	2.4469
24	2017	49	9.5857	72.6429	951.1867	1.7071	1.3143
63	2017	49	8.5286	82.8163	956.6255	1.2408	1.3592
62	2017	49	7.0857	82.3878	882.6500	0.9510	1.0918
1	2017	49	7.1429	81.2041	882.9816	1.6908	2.2786
31	2017	50	3.8571	88.4592	852.4204	0.4929	1.0000
79	2017	50	6.7857	69.3878	982.7776	0.9286	1.2500
51	2017	50	5.2429	76.0510	950.2990	0.7980	1.4276
14	2017	50	5.3429	82.5714	905.6163	0.9663	1.5622
67	2017	50	4.5714	83.8163	911.1235	0.9673	2.5235
42	2017	50	4.2286	83.3469	882.3980	0.7959	2.3714
50	2017	50	5.4286	83.3265	911.0541	0.5857	1.4388
43	2017	50	4.2286	83.3469	882.3980	0.7959	2.3714
85	2017	50	6.2000	81.7449	919.7510	0.5714	1.1571
25	2017	50	9.2429	78.9796	989.2469	0.7592	0.6776
69	2017	50	7.0571	70.9286	951.0439	0.7143	1.2622
57	2017	50	4.3571	88.4082	895.0796	0.6286	1.6429
9	2017	50	4.2571	87.8163	860.5051	0.4786	2.2235
72	2017	50	5.7000	83.4898	884.8531	0.6184	1.9357
26	2017	50	7.1571	94.0306	873.6969	0.6122	1.4663
7	2017	50	6.3571	89.7143	865.4184	0.7735	1.8602
83	2017	50	11.3000	74.3061	954.3071	0.7214	0.9408
76	2017	50	5.5429	78.9184	931.3714	0.6643	1.0592
36	2017	50	5.7000	75.6531	939.4000	0.9143	1.5510
81	2017	50	5.2429	76.0510	950.2990	0.7980	1.4276
15	2017	50	5.3286	81.8571	928.7469	0.7214	1.4071
32	2017	50	4.2286	83.3469	882.3980	0.7959	2.3714
73	2017	50	8.2286	72.0714	974.5439	0.6571	0.5612
71	2017	50	5.7000	75.6531	939.4000	0.9143	1.5510
41	2017	50	4.4857	85.6224	882.0010	0.4214	1.1541
10	2017	50	6.4286	76.5000	976.5296	1.0255	0.8612
23	2017	50	1.3286	93.1224	778.8184	1.3153	1.3776
27	2017	50	6.3571	89.7143	865.4184	0.7735	1.8602
60	2017	50	5.2429	76.0510	950.2990	0.7980	1.4276
53	2017	50	4.2571	87.8163	860.5051	0.4786	2.2235
66	2017	50	5.3429	82.5714	905.6163	0.9663	1.5622
59	2017	50	4.3571	88.4082	895.0796	0.6286	1.6429
61	2017	50	8.2286	72.0714	974.5439	0.6571	0.5612
84	2017	50	8.2286	72.0714	974.5439	0.6571	0.5612
38	2017	50	4.3571	88.4082	895.0796	0.6286	1.6429
87	2017	50	6.9714	77.7449	907.8990	0.6337	1.8327
34	2017	50	4.3571	88.4082	895.0796	0.6286	1.6429
29	2017	50	7.0571	70.9286	951.0439	0.7143	1.2622
5	2017	50	4.2857	96.1837	837.4602	0.8990	1.4429
8	2017	50	4.2571	87.8163	860.5051	0.4786	2.2235
12	2017	50	4.2857	96.1837	837.4602	0.8990	1.4429
13	2017	50	11.3000	74.3061	954.3071	0.7214	0.9408
18	2017	50	6.7571	74.9184	976.8082	0.8286	0.8908
33	2017	50	5.4286	83.3265	911.0541	0.5857	1.4388
56	2017	50	9.2429	78.9796	989.2469	0.7592	0.6776

77	2017	50	5.3286	81.8571	928.7469	0.7214	1.4071
54	2017	50	4.2857	96.1837	837.4602	0.8990	1.4429
21	2017	50	5.4286	83.3265	911.0541	0.5857	1.4388
68	2017	50	6.7857	69.3878	982.7776	0.9286	1.2500
74	2017	50	8.2286	72.0714	974.5439	0.6571	0.5612
88	2017	50	4.2286	83.3469	882.3980	0.7959	2.3714
16	2017	50	5.5429	78.9184	931.3714	0.6643	1.0592
30	2017	50	5.3429	82.5714	905.6163	0.9663	1.5622
6	2017	50	6.7857	69.3878	982.7776	0.9286	1.2500
49	2017	50	7.0571	70.9286	951.0439	0.7143	1.2622
22	2017	50	4.2286	83.3469	882.3980	0.7959	2.3714
45	2017	50	4.8000	90.2041	821.9347	1.4837	1.4796
58	2017	50	7.0571	70.9286	951.0439	0.7143	1.2622
37	2017	50	6.7857	69.3878	982.7776	0.9286	1.2500
17	2017	50	4.5714	83.8163	911.1235	0.9673	2.5235
55	2017	50	5.7000	83.4898	884.8531	0.6184	1.9357
46	2017	50	5.5429	78.9184	931.3714	0.6643	1.0592
86	2017	50	5.2714	91.9082	873.2612	0.0429	1.0306
2	2017	50	5.2714	91.9082	873.2612	0.0429	1.0306
4	2017	50	5.4286	83.3265	911.0541	0.5857	1.4388
47	2017	50	10.8714	75.2041	969.4184	0.8255	0.4265
82	2017	50	4.2286	83.3469	882.3980	0.7959	2.3714
19	2017	50	9.6143	72.1224	971.4357	0.6837	1.1633
20	2017	50	4.2571	87.8163	860.5051	0.4786	2.2235
80	2017	50	4.2286	83.3469	882.3980	0.7959	2.3714
3	2017	50	11.3000	74.3061	954.3071	0.7214	0.9408
52	2017	50	4.5714	83.8163	911.1235	0.9673	2.5235
70	2017	50	6.2000	81.7449	919.7510	0.5714	1.1571
64	2017	50	1.3286	93.1224	778.8184	1.3153	1.3776
48	2017	50	5.3286	81.8571	928.7469	0.7214	1.4071
65	2017	50	4.5714	83.8163	911.1235	0.9673	2.5235
44	2017	50	6.2000	81.7449	919.7510	0.5714	1.1571
75	2017	50	1.3286	93.1224	778.8184	1.3153	1.3776
40	2017	50	5.6571	80.0612	956.5143	0.8847	1.2949
11	2017	50	5.7000	83.4898	884.8531	0.6184	1.9357
35	2017	50	5.2429	76.0510	950.2990	0.7980	1.4276
78	2017	50	6.9714	77.7449	907.8990	0.6337	1.8327
28	2017	50	5.7000	75.6531	939.4000	0.9143	1.5510
39	2017	50	4.5714	83.8163	911.1235	0.9673	2.5235
24	2017	50	7.0571	70.9286	951.0439	0.7143	1.2622
63	2017	50	5.6571	80.0612	956.5143	0.8847	1.2949
62	2017	50	4.4857	85.6224	882.0010	0.4214	1.1541
1	2017	50	4.2286	83.3469	882.3980	0.7959	2.3714
31	2017	51	3.7714	83.5510	854.6786	1.8184	1.0092
79	2017	51	8.1000	65.3469	985.5643	2.5245	1.1622
51	2017	51	5.9714	71.5204	952.9163	2.2878	1.3490
14	2017	51	7.7000	71.6327	907.9469	2.7684	1.8010
67	2017	51	7.0429	73.2755	913.5010	2.3184	2.7622
42	2017	51	6.4143	74.3776	884.7622	2.2224	2.3224
50	2017	51	5.3714	79.2755	913.6745	1.9429	1.4153

43	2017	51	6.4143	74.3776	884.7622	2.2224	2.3224
85	2017	51	6.2429	77.1531	922.4806	1.8745	1.1520
25	2017	51	8.8714	74.4184	992.2194	2.2622	0.7663
69	2017	51	7.1857	67.5000	953.7969	1.6449	1.2082
57	2017	51	5.6000	79.2857	897.5500	2.0306	1.5878
9	2017	51	5.6143	79.1224	862.7051	2.1500	2.1592
72	2017	51	6.7000	73.7653	887.1837	2.2071	1.9949
26	2017	51	7.5571	84.2143	876.0490	1.6704	1.3153
7	2017	51	6.3000	81.7755	867.7153	1.9724	1.6765
83	2017	51	10.2143	70.7449	957.2735	2.5143	1.0296
76	2017	51	6.0571	73.7245	933.9939	2.0327	1.0439
36	2017	51	6.9857	68.2653	941.9837	2.1663	1.6867
81	2017	51	5.9714	71.5204	952.9163	2.2878	1.3490
15	2017	51	5.3714	81.3265	931.4663	1.4163	1.2755
32	2017	51	6.4143	74.3776	884.7622	2.2224	2.3224
73	2017	51	7.8000	69.2551	977.4337	1.4776	0.4367
71	2017	51	6.9857	68.2653	941.9837	2.1663	1.6867
41	2017	51	5.0714	80.2245	884.3929	1.9980	1.1602
10	2017	51	7.6000	71.5306	979.3102	2.8469	0.9184
23	2017	51	1.1429	90.2653	780.5092	2.0327	1.5980
27	2017	51	6.3000	81.7755	867.7153	1.9724	1.6765
60	2017	51	5.9714	71.5204	952.9163	2.2878	1.3490
53	2017	51	5.6143	79.1224	862.7051	2.1500	2.1592
66	2017	51	7.7000	71.6327	907.9469	2.7684	1.8010
59	2017	51	5.6000	79.2857	897.5500	2.0306	1.5878
61	2017	51	7.8000	69.2551	977.4337	1.4776	0.4367
84	2017	51	7.8000	69.2551	977.4337	1.4776	0.4367
38	2017	51	5.6000	79.2857	897.5500	2.0306	1.5878
87	2017	51	6.9857	70.0102	910.4571	2.0918	2.2102
34	2017	51	5.6000	79.2857	897.5500	2.0306	1.5878
29	2017	51	7.1857	67.5000	953.7969	1.6449	1.2082
5	2017	51	4.8857	87.6122	839.5786	2.0296	1.3612
8	2017	51	5.6143	79.1224	862.7051	2.1500	2.1592
12	2017	51	4.8857	87.6122	839.5786	2.0296	1.3612
13	2017	51	10.2143	70.7449	957.2735	2.5143	1.0296
18	2017	51	6.8143	72.4286	979.5867	2.2724	0.8214
33	2017	51	5.3714	79.2755	913.6745	1.9429	1.4153
56	2017	51	8.8714	74.4184	992.2194	2.2622	0.7663
77	2017	51	5.3714	81.3265	931.4663	1.4163	1.2755
54	2017	51	4.8857	87.6122	839.5786	2.0296	1.3612
21	2017	51	5.3714	79.2755	913.6745	1.9429	1.4153
68	2017	51	8.1000	65.3469	985.5643	2.5245	1.1622
74	2017	51	7.8000	69.2551	977.4337	1.4776	0.4367
88	2017	51	6.4143	74.3776	884.7622	2.2224	2.3224
16	2017	51	6.0571	73.7245	933.9939	2.0327	1.0439
30	2017	51	7.7000	71.6327	907.9469	2.7684	1.8010
6	2017	51	8.1000	65.3469	985.5643	2.5245	1.1622
49	2017	51	7.1857	67.5000	953.7969	1.6449	1.2082
22	2017	51	6.4143	74.3776	884.7622	2.2224	2.3224
45	2017	51	4.2000	87.7143	823.9714	2.0051	1.3612

58	2017	51	7.1857	67.5000	953.7969	1.6449	1.2082
37	2017	51	8.1000	65.3469	985.5643	2.5245	1.1622
17	2017	51	7.0429	73.2755	913.5010	2.3184	2.7622
55	2017	51	6.7000	73.7653	887.1837	2.2071	1.9949
46	2017	51	6.0571	73.7245	933.9939	2.0327	1.0439
86	2017	51	5.4857	83.4082	875.5929	0.7571	1.1541
2	2017	51	5.4857	83.4082	875.5929	0.7571	1.1541
4	2017	51	5.3714	79.2755	913.6745	1.9429	1.4153
47	2017	51	10.4714	70.7653	972.4367	2.2337	0.4622
82	2017	51	6.4143	74.3776	884.7622	2.2224	2.3224
19	2017	51	9.7286	66.6633	974.2765	2.9010	1.2143
20	2017	51	5.6143	79.1224	862.7051	2.1500	2.1592
80	2017	51	6.4143	74.3776	884.7622	2.2224	2.3224
3	2017	51	10.2143	70.7449	957.2735	2.5143	1.0296
52	2017	51	7.0429	73.2755	913.5010	2.3184	2.7622
70	2017	51	6.2429	77.1531	922.4806	1.8745	1.1520
64	2017	51	1.1429	90.2653	780.5092	2.0327	1.5980
48	2017	51	5.3714	81.3265	931.4663	1.4163	1.2755
65	2017	51	7.0429	73.2755	913.5010	2.3184	2.7622
44	2017	51	6.2429	77.1531	922.4806	1.8745	1.1520
75	2017	51	1.1429	90.2653	780.5092	2.0327	1.5980
40	2017	51	6.7857	73.6327	959.1122	3.4469	1.2939
11	2017	51	6.7000	73.7653	887.1837	2.2071	1.9949
35	2017	51	5.9714	71.5204	952.9163	2.2878	1.3490
78	2017	51	6.9857	70.0102	910.4571	2.0918	2.2102
28	2017	51	6.9857	68.2653	941.9837	2.1663	1.6867
39	2017	51	7.0429	73.2755	913.5010	2.3184	2.7622
24	2017	51	7.1857	67.5000	953.7969	1.6449	1.2082
63	2017	51	6.7857	73.6327	959.1122	3.4469	1.2939
62	2017	51	5.0714	80.2245	884.3929	1.9980	1.1602
1	2017	51	6.4143	74.3776	884.7622	2.2224	2.3224
31	2017	52	6.2714	72.6837	855.6429	3.8092	0.9694
79	2017	52	8.9571	66.1735	985.7663	4.1847	0.9837
51	2017	52	7.8143	69.9286	953.2827	4.1133	1.0622
14	2017	52	9.1143	63.2857	908.7612	4.4541	1.8439
67	2017	52	8.8143	65.5816	914.1235	4.3878	2.8204
42	2017	52	8.3571	64.0000	885.6010	4.6398	2.3755
50	2017	52	7.6143	74.5102	914.4082	3.4673	1.2796
43	2017	52	8.3571	64.0000	885.6010	4.6398	2.3755
85	2017	52	9.0286	71.9694	923.2469	3.4561	1.1980
25	2017	52	11.2714	74.1224	992.7286	3.9898	0.8561
69	2017	52	9.6857	68.1327	954.1612	3.4061	1.0847
57	2017	52	8.6000	68.8265	898.2520	3.8378	1.7949
9	2017	52	7.4286	67.9286	863.7010	4.3112	1.9796
72	2017	52	8.4571	65.5816	888.1347	4.1643	1.7602
26	2017	52	9.7429	73.3367	877.1653	4.5153	1.4418
7	2017	52	9.1714	71.6633	868.8031	4.8867	1.4500
83	2017	52	12.8714	69.2755	958.2357	5.0561	0.9745
76	2017	52	8.5857	70.2959	934.4031	4.2765	1.1663
36	2017	52	9.2286	65.7449	942.4592	4.5010	1.5959

81	2017	52	7.8143	69.9286	953.2827	4.1133	1.0622
15	2017	52	7.7714	77.2959	932.0194	2.8378	1.2510
32	2017	52	8.3571	64.0000	885.6010	4.6398	2.3755
73	2017	52	10.2143	71.1735	977.7286	3.0031	0.3908
71	2017	52	9.2286	65.7449	942.4592	4.5010	1.5959
41	2017	52	7.8000	71.8265	885.2214	4.1327	1.1143
10	2017	52	8.6857	68.9490	979.5878	5.0704	0.9051
23	2017	52	4.2000	79.2347	781.7633	4.3959	2.0684
27	2017	52	9.1714	71.6633	868.8031	4.8867	1.4500
60	2017	52	7.8143	69.9286	953.2827	4.1133	1.0622
53	2017	52	7.4286	67.9286	863.7010	4.3112	1.9796
66	2017	52	9.1143	63.2857	908.7612	4.4541	1.8439
59	2017	52	8.6000	68.8265	898.2520	3.8378	1.7949
61	2017	52	10.2143	71.1735	977.7286	3.0031	0.3908
84	2017	52	10.2143	71.1735	977.7286	3.0031	0.3908
38	2017	52	8.6000	68.8265	898.2520	3.8378	1.7949
87	2017	52	9.1571	65.6735	911.3214	4.4439	1.9959
34	2017	52	8.6000	68.8265	898.2520	3.8378	1.7949
29	2017	52	9.6857	68.1327	954.1612	3.4061	1.0847
5	2017	52	7.4571	75.0204	840.6980	4.5459	1.3143
8	2017	52	7.4286	67.9286	863.7010	4.3112	1.9796
12	2017	52	7.4571	75.0204	840.6980	4.5459	1.3143
13	2017	52	12.8714	69.2755	958.2357	5.0561	0.9745
18	2017	52	8.3857	73.1020	979.7939	3.8765	0.6143
33	2017	52	7.6143	74.5102	914.4082	3.4673	1.2796
56	2017	52	11.2714	74.1224	992.7286	3.9898	0.8561
77	2017	52	7.7714	77.2959	932.0194	2.8378	1.2510
54	2017	52	7.4571	75.0204	840.6980	4.5459	1.3143
21	2017	52	7.6143	74.5102	914.4082	3.4673	1.2796
68	2017	52	8.9571	66.1735	985.7663	4.1847	0.9837
74	2017	52	10.2143	71.1735	977.7286	3.0031	0.3908
88	2017	52	8.3571	64.0000	885.6010	4.6398	2.3755
16	2017	52	8.5857	70.2959	934.4031	4.2765	1.1663
30	2017	52	9.1143	63.2857	908.7612	4.4541	1.8439
6	2017	52	8.9571	66.1735	985.7663	4.1847	0.9837
49	2017	52	9.6857	68.1327	954.1612	3.4061	1.0847
22	2017	52	8.3571	64.0000	885.6010	4.6398	2.3755
45	2017	52	7.8714	77.4082	825.1704	4.5449	1.2878
58	2017	52	9.6857	68.1327	954.1612	3.4061	1.0847
37	2017	52	8.9571	66.1735	985.7663	4.1847	0.9837
17	2017	52	8.8143	65.5816	914.1235	4.3878	2.8204
55	2017	52	8.4571	65.5816	888.1347	4.1643	1.7602
46	2017	52	8.5857	70.2959	934.4031	4.2765	1.1663
86	2017	52	7.8429	73.8367	876.5153	2.1398	1.2265
2	2017	52	7.8429	73.8367	876.5153	2.1398	1.2265
4	2017	52	7.6143	74.5102	914.4082	3.4673	1.2796
47	2017	52	12.5286	70.1837	973.1898	4.0704	0.4929
82	2017	52	8.3571	64.0000	885.6010	4.6398	2.3755
19	2017	52	11.6571	66.8265	975.0031	4.7908	0.9663
20	2017	52	7.4286	67.9286	863.7010	4.3112	1.9796

80	2017	52	8.3571	64.0000	885.6010	4.6398	2.3755
3	2017	52	12.8714	69.2755	958.2357	5.0561	0.9745
52	2017	52	8.8143	65.5816	914.1235	4.3878	2.8204
70	2017	52	9.0286	71.9694	923.2469	3.4561	1.1980
64	2017	52	4.2000	79.2347	781.7633	4.3959	2.0684
48	2017	52	7.7714	77.2959	932.0194	2.8378	1.2510
65	2017	52	8.8143	65.5816	914.1235	4.3878	2.8204
44	2017	52	9.0286	71.9694	923.2469	3.4561	1.1980
75	2017	52	4.2000	79.2347	781.7633	4.3959	2.0684
40	2017	52	8.5143	71.5612	959.5673	5.3561	1.1010
11	2017	52	8.4571	65.5816	888.1347	4.1643	1.7602
35	2017	52	7.8143	69.9286	953.2827	4.1133	1.0622
78	2017	52	9.1571	65.6735	911.3214	4.4439	1.9959
28	2017	52	9.2286	65.7449	942.4592	4.5010	1.5959
39	2017	52	8.8143	65.5816	914.1235	4.3878	2.8204
24	2017	52	9.6857	68.1327	954.1612	3.4061	1.0847
63	2017	52	8.5143	71.5612	959.5673	5.3561	1.1010
62	2017	52	7.8000	71.8265	885.2214	4.1327	1.1143
1	2017	52	8.3571	64.0000	885.6010	4.6398	2.3755
31	2018	1	3.9571	74.5408	852.1071	2.8704	1.0357
79	2018	1	3.4714	74.0204	982.3806	2.5592	1.2571
51	2018	1	2.3429	78.5816	949.8092	2.8929	1.3163
14	2018	1	5.7286	76.7959	905.3765	2.6418	1.9592
67	2018	1	4.1429	77.0204	910.5582	2.8061	2.4837
42	2018	1	4.9000	74.1837	882.0133	3.1786	2.3602
50	2018	1	4.4000	77.5000	910.6235	2.0684	1.4622
43	2018	1	4.9000	74.1837	882.0133	3.1786	2.3602
85	2018	1	5.0714	75.4286	919.3827	2.1980	1.3398
25	2018	1	7.7143	81.7959	988.9327	2.6327	0.8224
69	2018	1	4.3857	76.5918	950.3684	2.3724	1.1694
57	2018	1	2.8429	76.2143	894.5296	2.5031	1.8592
9	2018	1	7.3429	75.9592	860.2388	2.8367	1.9694
72	2018	1	9.5571	75.1939	884.5510	2.9388	1.5980
26	2018	1	13.2000	79.8367	873.7082	4.6571	1.7776
7	2018	1	12.9429	75.4082	865.3500	5.0102	1.5673
83	2018	1	15.8143	74.1327	954.0378	3.5908	0.8847
76	2018	1	3.2857	75.0204	930.6663	2.9031	1.3541
36	2018	1	3.2429	77.2653	938.8020	3.3633	1.5316
81	2018	1	2.3429	78.5816	949.8092	2.8929	1.3163
15	2018	1	3.6857	77.5816	928.2490	2.2092	1.5031
32	2018	1	4.9000	74.1837	882.0133	3.1786	2.3602
73	2018	1	5.5143	73.6633	973.9684	2.0663	0.5010
71	2018	1	3.2429	77.2653	938.8020	3.3633	1.5316
41	2018	1	4.1714	77.2449	881.6316	2.8663	1.2235
10	2018	1	3.6857	77.9184	976.1418	3.2510	1.0133
23	2018	1	8.8571	73.0918	778.9316	5.0622	2.3102
27	2018	1	12.9429	75.4082	865.3500	5.0102	1.5673
60	2018	1	2.3429	78.5816	949.8092	2.8929	1.3163
53	2018	1	7.3429	75.9592	860.2388	2.8367	1.9694
66	2018	1	5.7286	76.7959	905.3765	2.6418	1.9592

59	2018	1	2.8429	76.2143	894.5296	2.5031	1.8592
61	2018	1	5.5143	73.6633	973.9684	2.0663	0.5010
84	2018	1	5.5143	73.6633	973.9684	2.0663	0.5010
38	2018	1	2.8429	76.2143	894.5296	2.5031	1.8592
87	2018	1	7.8857	76.7041	907.5694	2.9612	1.6388
34	2018	1	2.8429	76.2143	894.5296	2.5031	1.8592
29	2018	1	4.3857	76.5918	950.3684	2.3724	1.1694
5	2018	1	11.9000	76.7449	837.3847	4.9929	1.4082
8	2018	1	7.3429	75.9592	860.2388	2.8367	1.9694
12	2018	1	11.9000	76.7449	837.3847	4.9929	1.4082
13	2018	1	15.8143	74.1327	954.0378	3.5908	0.8847
18	2018	1	3.6857	78.3673	976.4102	2.2306	0.8031
33	2018	1	4.4000	77.5000	910.6235	2.0684	1.4622
56	2018	1	7.7143	81.7959	988.9327	2.6327	0.8224
77	2018	1	3.6857	77.5816	928.2490	2.2092	1.5031
54	2018	1	11.9000	76.7449	837.3847	4.9929	1.4082
21	2018	1	4.4000	77.5000	910.6235	2.0684	1.4622
68	2018	1	3.4714	74.0204	982.3806	2.5592	1.2571
74	2018	1	5.5143	73.6633	973.9684	2.0663	0.5010
88	2018	1	4.9000	74.1837	882.0133	3.1786	2.3602
16	2018	1	3.2857	75.0204	930.6663	2.9031	1.3541
30	2018	1	5.7286	76.7959	905.3765	2.6418	1.9592
6	2018	1	3.4714	74.0204	982.3806	2.5592	1.2571
49	2018	1	4.3857	76.5918	950.3684	2.3724	1.1694
22	2018	1	4.9000	74.1837	882.0133	3.1786	2.3602
45	2018	1	13.0000	71.7755	822.0663	5.0694	1.7918
58	2018	1	4.3857	76.5918	950.3684	2.3724	1.1694
37	2018	1	3.4714	74.0204	982.3806	2.5592	1.2571
17	2018	1	4.1429	77.0204	910.5582	2.8061	2.4837
55	2018	1	9.5571	75.1939	884.5510	2.9388	1.5980
46	2018	1	3.2857	75.0204	930.6663	2.9031	1.3541
86	2018	1	6.4571	79.1531	872.9071	1.6429	1.3276
2	2018	1	6.4571	79.1531	872.9071	1.6429	1.3276
4	2018	1	4.4000	77.5000	910.6235	2.0684	1.4622
47	2018	1	12.2714	79.4184	969.0357	2.2633	0.4286
82	2018	1	4.9000	74.1837	882.0133	3.1786	2.3602
19	2018	1	9.1571	79.1429	971.3194	2.6388	0.7010
20	2018	1	7.3429	75.9592	860.2388	2.8367	1.9694
80	2018	1	4.9000	74.1837	882.0133	3.1786	2.3602
3	2018	1	15.8143	74.1327	954.0378	3.5908	0.8847
52	2018	1	4.1429	77.0204	910.5582	2.8061	2.4837
70	2018	1	5.0714	75.4286	919.3827	2.1980	1.3398
64	2018	1	8.8571	73.0918	778.9316	5.0622	2.3102
48	2018	1	3.6857	77.5816	928.2490	2.2092	1.5031
65	2018	1	4.1429	77.0204	910.5582	2.8061	2.4837
44	2018	1	5.0714	75.4286	919.3827	2.1980	1.3398
75	2018	1	8.8571	73.0918	778.9316	5.0622	2.3102
40	2018	1	3.5000	81.0612	956.1949	3.0133	1.1327
11	2018	1	9.5571	75.1939	884.5510	2.9388	1.5980
35	2018	1	2.3429	78.5816	949.8092	2.8929	1.3163

78	2018	1	7.8857	76.7041	907.5694	2.9612	1.6388
28	2018	1	3.2429	77.2653	938.8020	3.3633	1.5316
39	2018	1	4.1429	77.0204	910.5582	2.8061	2.4837
24	2018	1	4.3857	76.5918	950.3684	2.3724	1.1694
63	2018	1	3.5000	81.0612	956.1949	3.0133	1.1327
62	2018	1	4.1714	77.2449	881.6316	2.8663	1.2235
1	2018	1	4.9000	74.1837	882.0133	3.1786	2.3602
31	2018	2	1.5857	80.7143	850.8969	0.9265	1.0847
79	2018	2	5.2714	80.2449	982.8041	1.1276	1.5867
51	2018	2	4.4857	86.8469	949.7490	0.7847	1.5898
14	2018	2	3.9429	86.1735	904.4082	0.2918	2.1806
67	2018	2	3.6714	87.5918	909.9000	0.1714	2.2918
42	2018	2	3.2429	84.4796	880.9357	0.6398	2.5286
50	2018	2	3.9429	82.9286	909.9010	0.6163	1.4673
43	2018	2	3.2429	84.4796	880.9357	0.6398	2.5286
85	2018	2	5.0000	81.7449	918.7929	0.6429	1.2969
25	2018	2	7.2857	83.8571	988.9235	0.7786	0.9857
69	2018	2	5.5571	82.7653	950.3408	0.4714	1.2969
57	2018	2	3.4000	87.3469	893.7653	0.3408	1.6796
9	2018	2	2.6143	81.5102	858.8357	0.8816	2.4173
72	2018	2	4.0000	78.7755	883.1990	1.3571	2.1612
26	2018	2	5.7286	80.5204	872.0857	3.6684	1.9990
7	2018	2	4.6000	74.5918	863.8041	4.0122	2.0796
83	2018	2	10.1000	71.0102	952.6255	1.9582	1.0184
76	2018	2	4.2429	83.7857	930.3929	0.7653	1.2714
36	2018	2	4.7714	85.3265	938.5582	0.4602	1.6704
81	2018	2	4.4857	86.8469	949.7490	0.7847	1.5898
15	2018	2	3.8571	83.1327	927.7806	1.3214	1.5990
32	2018	2	3.2429	84.4796	880.9357	0.6398	2.5286
73	2018	2	6.1857	77.2245	974.1990	0.8327	0.6316
71	2018	2	4.7714	85.3265	938.5582	0.4602	1.6704
41	2018	2	3.3571	83.8878	880.7214	0.6531	1.3490
10	2018	2	5.2714	85.8571	976.4245	1.1571	1.1020
23	2018	2	-1.8429	70.6327	777.0704	4.2653	2.3092
27	2018	2	4.6000	74.5918	863.8041	4.0122	2.0796
60	2018	2	4.4857	86.8469	949.7490	0.7847	1.5898
53	2018	2	2.6143	81.5102	858.8357	0.8816	2.4173
66	2018	2	3.9429	86.1735	904.4082	0.2918	2.1806
59	2018	2	3.4000	87.3469	893.7653	0.3408	1.6796
61	2018	2	6.1857	77.2245	974.1990	0.8327	0.6316
84	2018	2	6.1857	77.2245	974.1990	0.8327	0.6316
38	2018	2	3.4000	87.3469	893.7653	0.3408	1.6796
87	2018	2	5.0857	82.0816	906.5214	0.4561	2.2929
34	2018	2	3.4000	87.3469	893.7653	0.3408	1.6796
29	2018	2	5.5571	82.7653	950.3408	0.4714	1.2969
5	2018	2	2.1286	74.1020	835.7561	4.2122	1.8592
8	2018	2	2.6143	81.5102	858.8357	0.8816	2.4173
12	2018	2	2.1286	74.1020	835.7561	4.2122	1.8592
13	2018	2	10.1000	71.0102	952.6255	1.9582	1.0184
18	2018	2	4.5143	84.4694	976.7296	1.3245	1.0000

33	2018	2	3.9429	82.9286	909.9010	0.6163	1.4673
56	2018	2	7.2857	83.8571	988.9235	0.7786	0.9857
77	2018	2	3.8571	83.1327	927.7806	1.3214	1.5990
54	2018	2	2.1286	74.1020	835.7561	4.2122	1.8592
21	2018	2	3.9429	82.9286	909.9010	0.6163	1.4673
68	2018	2	5.2714	80.2449	982.8041	1.1276	1.5867
74	2018	2	6.1857	77.2245	974.1990	0.8327	0.6316
88	2018	2	3.2429	84.4796	880.9357	0.6398	2.5286
16	2018	2	4.2429	83.7857	930.3929	0.7653	1.2714
30	2018	2	3.9429	86.1735	904.4082	0.2918	2.1806
6	2018	2	5.2714	80.2449	982.8041	1.1276	1.5867
49	2018	2	5.5571	82.7653	950.3408	0.4714	1.2969
22	2018	2	3.2429	84.4796	880.9357	0.6398	2.5286
45	2018	2	2.2143	68.0612	820.4153	3.9398	2.2082
58	2018	2	5.5571	82.7653	950.3408	0.4714	1.2969
37	2018	2	5.2714	80.2449	982.8041	1.1276	1.5867
17	2018	2	3.6714	87.5918	909.9000	0.1714	2.2918
55	2018	2	4.0000	78.7755	883.1990	1.3571	2.1612
46	2018	2	4.2429	83.7857	930.3929	0.7653	1.2714
86	2018	2	3.5286	82.4592	871.7602	0.4745	1.3622
2	2018	2	3.5286	82.4592	871.7602	0.4745	1.3622
4	2018	2	3.9429	82.9286	909.9010	0.6163	1.4673
47	2018	2	9.1857	79.5510	968.1551	0.5520	0.5020
82	2018	2	3.2429	84.4796	880.9357	0.6398	2.5286
19	2018	2	7.9000	81.2245	970.8163	0.5796	1.0092
20	2018	2	2.6143	81.5102	858.8357	0.8816	2.4173
80	2018	2	3.2429	84.4796	880.9357	0.6398	2.5286
3	2018	2	10.1000	71.0102	952.6255	1.9582	1.0184
52	2018	2	3.6714	87.5918	909.9000	0.1714	2.2918
70	2018	2	5.0000	81.7449	918.7929	0.6429	1.2969
64	2018	2	-1.8429	70.6327	777.0704	4.2653	2.3092
48	2018	2	3.8571	83.1327	927.7806	1.3214	1.5990
65	2018	2	3.6714	87.5918	909.9000	0.1714	2.2918
44	2018	2	5.0000	81.7449	918.7929	0.6429	1.2969
75	2018	2	-1.8429	70.6327	777.0704	4.2653	2.3092
40	2018	2	4.7286	89.0918	956.0765	1.0724	1.2061
11	2018	2	4.0000	78.7755	883.1990	1.3571	2.1612
35	2018	2	4.4857	86.8469	949.7490	0.7847	1.5898
78	2018	2	5.0857	82.0816	906.5214	0.4561	2.2929
28	2018	2	4.7714	85.3265	938.5582	0.4602	1.6704
39	2018	2	3.6714	87.5918	909.9000	0.1714	2.2918
24	2018	2	5.5571	82.7653	950.3408	0.4714	1.2969
63	2018	2	4.7286	89.0918	956.0765	1.0724	1.2061
62	2018	2	3.3571	83.8878	880.7214	0.6531	1.3490
1	2018	2	3.2429	84.4796	880.9357	0.6398	2.5286
31	2018	3	5.3571	76.5612	850.6153	2.0663	1.0857
79	2018	3	7.3714	78.6837	982.2480	1.6265	1.2714
51	2018	3	6.7429	82.9082	949.3582	0.9429	1.2796
14	2018	3	7.6857	81.5000	904.4000	0.3102	2.0020
67	2018	3	6.5857	83.1122	909.8643	0.1520	2.3429

42	2018	3	6.3143	78.9490	880.8735	0.7480	2.4357
50	2018	3	6.0714	80.2857	909.6939	0.7500	1.2327
43	2018	3	6.3143	78.9490	880.8735	0.7480	2.4357
85	2018	3	6.8286	78.0714	918.5592	0.9847	1.1745
25	2018	3	11.0143	82.4592	988.8571	0.4092	0.8684
69	2018	3	7.8714	79.2041	950.1255	0.5918	1.0949
57	2018	3	6.1143	82.4592	893.5796	0.5357	1.8255
9	2018	3	6.3571	75.7551	858.7857	1.1867	2.4214
72	2018	3	7.4714	73.6735	883.2755	1.2429	2.1541
26	2018	3	9.6571	76.0714	872.0541	3.2143	1.8122
7	2018	3	9.1714	71.6633	863.7918	3.7949	2.0561
83	2018	3	13.0143	66.1633	953.0082	2.4082	1.0990
76	2018	3	6.6286	81.7449	930.1633	0.9143	1.1194
36	2018	3	7.5000	81.3061	938.3908	0.3561	1.4745
81	2018	3	6.7429	82.9082	949.3582	0.9429	1.2796
15	2018	3	6.4286	81.9490	927.4959	1.5286	1.4122
32	2018	3	6.3143	78.9490	880.8735	0.7480	2.4357
73	2018	3	8.5857	78.0306	973.8531	1.3786	0.5755
71	2018	3	7.5000	81.3061	938.3908	0.3561	1.4745
41	2018	3	6.5857	77.8367	880.5224	0.9010	1.2337
10	2018	3	7.8571	82.2245	976.0153	1.2571	0.9653
23	2018	3	4.0143	73.8571	776.6459	3.8296	1.9378
27	2018	3	9.1714	71.6633	863.7918	3.7949	2.0561
60	2018	3	6.7429	82.9082	949.3582	0.9429	1.2796
53	2018	3	6.3571	75.7551	858.7857	1.1867	2.4214
66	2018	3	7.6857	81.5000	904.4000	0.3102	2.0020
59	2018	3	6.1143	82.4592	893.5796	0.5357	1.8255
61	2018	3	8.5857	78.0306	973.8531	1.3786	0.5755
84	2018	3	8.5857	78.0306	973.8531	1.3786	0.5755
38	2018	3	6.1143	82.4592	893.5796	0.5357	1.8255
87	2018	3	8.3429	77.0306	906.6031	0.5112	2.1316
34	2018	3	6.1143	82.4592	893.5796	0.5357	1.8255
29	2018	3	7.8714	79.2041	950.1255	0.5918	1.0949
5	2018	3	6.8286	73.0612	835.7031	3.6031	1.9561
8	2018	3	6.3571	75.7551	858.7857	1.1867	2.4214
12	2018	3	6.8286	73.0612	835.7031	3.6031	1.9561
13	2018	3	13.0143	66.1633	953.0082	2.4082	1.0990
18	2018	3	7.4429	84.2347	976.1592	2.2388	0.7867
33	2018	3	6.0714	80.2857	909.6939	0.7500	1.2327
56	2018	3	11.0143	82.4592	988.8571	0.4092	0.8684
77	2018	3	6.4286	81.9490	927.4959	1.5286	1.4122
54	2018	3	6.8286	73.0612	835.7031	3.6031	1.9561
21	2018	3	6.0714	80.2857	909.6939	0.7500	1.2327
68	2018	3	7.3714	78.6837	982.2480	1.6265	1.2714
74	2018	3	8.5857	78.0306	973.8531	1.3786	0.5755
88	2018	3	6.3143	78.9490	880.8735	0.7480	2.4357
16	2018	3	6.6286	81.7449	930.1633	0.9143	1.1194
30	2018	3	7.6857	81.5000	904.4000	0.3102	2.0020
6	2018	3	7.3714	78.6837	982.2480	1.6265	1.2714
49	2018	3	7.8714	79.2041	950.1255	0.5918	1.0949

22	2018	3	6.3143	78.9490	880.8735	0.7480	2.4357
45	2018	3	8.1143	69.6122	820.1500	3.6796	2.0561
58	2018	3	7.8714	79.2041	950.1255	0.5918	1.0949
37	2018	3	7.3714	78.6837	982.2480	1.6265	1.2714
17	2018	3	6.5857	83.1122	909.8643	0.1520	2.3429
55	2018	3	7.4714	73.6735	883.2755	1.2429	2.1541
46	2018	3	6.6286	81.7449	930.1633	0.9143	1.1194
86	2018	3	6.9571	76.9490	871.5929	0.6204	1.6337
2	2018	3	6.9571	76.9490	871.5929	0.6204	1.6337
4	2018	3	6.0714	80.2857	909.6939	0.7500	1.2327
47	2018	3	12.4286	73.4694	968.4949	1.0357	0.4837
82	2018	3	6.3143	78.9490	880.8735	0.7480	2.4357
19	2018	3	11.2714	77.4388	970.9133	0.7724	0.9408
20	2018	3	6.3571	75.7551	858.7857	1.1867	2.4214
80	2018	3	6.3143	78.9490	880.8735	0.7480	2.4357
3	2018	3	13.0143	66.1633	953.0082	2.4082	1.0990
52	2018	3	6.5857	83.1122	909.8643	0.1520	2.3429
70	2018	3	6.8286	78.0714	918.5592	0.9847	1.1745
64	2018	3	4.0143	73.8571	776.6459	3.8296	1.9378
48	2018	3	6.4286	81.9490	927.4959	1.5286	1.4122
65	2018	3	6.5857	83.1122	909.8643	0.1520	2.3429
44	2018	3	6.8286	78.0714	918.5592	0.9847	1.1745
75	2018	3	4.0143	73.8571	776.6459	3.8296	1.9378
40	2018	3	7.7143	85.6327	955.8122	0.9816	1.0214
11	2018	3	7.4714	73.6735	883.2755	1.2429	2.1541
35	2018	3	6.7429	82.9082	949.3582	0.9429	1.2796
78	2018	3	8.3429	77.0306	906.6031	0.5112	2.1316
28	2018	3	7.5000	81.3061	938.3908	0.3561	1.4745
39	2018	3	6.5857	83.1122	909.8643	0.1520	2.3429
24	2018	3	7.8714	79.2041	950.1255	0.5918	1.0949
63	2018	3	7.7143	85.6327	955.8122	0.9816	1.0214
62	2018	3	6.5857	77.8367	880.5224	0.9010	1.2337
1	2018	3	6.3143	78.9490	880.8735	0.7480	2.4357
31	2018	4	3.5000	78.7143	848.3918	2.8816	1.1500
79	2018	4	2.1714	81.0204	979.6051	0.9378	1.5327
51	2018	4	1.3429	85.5102	946.8357	0.7184	1.4908
14	2018	4	4.7000	88.1735	902.0776	0.3286	2.1459
67	2018	4	2.2571	89.5510	907.4296	0.1194	2.2878
42	2018	4	4.1857	84.8878	878.5500	0.6490	2.2908
50	2018	4	3.0571	85.9694	907.1296	0.2592	1.3510
43	2018	4	4.1857	84.8878	878.5500	0.6490	2.2908
85	2018	4	4.4286	82.7245	915.8184	0.8796	1.1143
25	2018	4	6.5000	84.5408	985.9806	0.1163	0.8378
69	2018	4	4.3167	81.7245	947.1367	0.3724	1.1541
57	2018	4	2.1857	87.7041	891.0878	0.6786	1.8316
9	2018	4	5.7000	81.9490	856.6796	1.6510	2.2551
72	2018	4	7.0571	80.4592	881.0684	1.6480	1.8633
26	2018	4	10.3286	80.5714	869.8235	3.1724	1.9469
7	2018	4	9.5429	78.5204	861.6704	4.0020	1.9878
83	2018	4	12.4857	72.5102	950.4327	2.2806	1.0500

76	2018	4	3.1500	84.9796	927.4704	0.3867	1.2796
36	2018	4	2.3286	85.5612	935.7602	0.3388	1.4857
81	2018	4	1.3429	85.5102	946.8357	0.7184	1.4908
15	2018	4	2.5429	86.6939	924.9000	0.5980	1.3776
32	2018	4	4.1857	84.8878	878.5500	0.6490	2.2908
73	2018	4	4.5429	80.0000	970.9806	0.7071	0.6092
71	2018	4	2.3286	85.5612	935.7602	0.3388	1.4857
41	2018	4	3.5714	83.5612	878.0959	1.5408	1.2102
10	2018	4	1.5500	84.5510	973.1969	0.5898	1.0286
23	2018	4	6.0857	75.6837	775.0653	5.1490	1.7980
27	2018	4	9.5429	78.5204	861.6704	4.0020	1.9878
60	2018	4	1.3429	85.5102	946.8357	0.7184	1.4908
53	2018	4	5.7000	81.9490	856.6796	1.6510	2.2551
66	2018	4	4.7000	88.1735	902.0776	0.3286	2.1459
59	2018	4	2.1857	87.7041	891.0878	0.6786	1.8316
61	2018	4	4.5429	80.0000	970.9806	0.7071	0.6092
84	2018	4	4.5429	80.0000	970.9806	0.7071	0.6092
38	2018	4	2.1857	87.7041	891.0878	0.6786	1.8316
87	2018	4	7.2857	81.5204	904.1071	0.6041	1.6408
34	2018	4	2.1857	87.7041	891.0878	0.6786	1.8316
29	2018	4	4.3167	81.7245	947.1367	0.3724	1.1541
5	2018	4	8.0143	80.6531	833.7673	4.0541	1.9224
8	2018	4	5.7000	81.9490	856.6796	1.6510	2.2551
12	2018	4	8.0143	80.6531	833.7673	4.0541	1.9224
13	2018	4	12.4857	72.5102	950.4327	2.2806	1.0500
18	2018	4	2.6286	86.1735	973.5408	1.2990	0.8959
33	2018	4	3.0571	85.9694	907.1296	0.2592	1.3510
56	2018	4	6.5000	84.5408	985.9806	0.1163	0.8378
77	2018	4	2.5429	86.6939	924.9000	0.5980	1.3776
54	2018	4	8.0143	80.6531	833.7673	4.0541	1.9224
21	2018	4	3.0571	85.9694	907.1296	0.2592	1.3510
68	2018	4	2.1714	81.0204	979.6051	0.9378	1.5327
74	2018	4	4.5429	80.0000	970.9806	0.7071	0.6092
88	2018	4	4.1857	84.8878	878.5500	0.6490	2.2908
16	2018	4	3.1500	84.9796	927.4704	0.3867	1.2796
30	2018	4	4.7000	88.1735	902.0776	0.3286	2.1459
6	2018	4	2.1714	81.0204	979.6051	0.9378	1.5327
49	2018	4	4.3167	81.7245	947.1367	0.3724	1.1541
22	2018	4	4.1857	84.8878	878.5500	0.6490	2.2908
45	2018	4	10.5000	72.1429	818.2000	4.8051	1.9959
58	2018	4	4.3167	81.7245	947.1367	0.3724	1.1541
37	2018	4	2.1714	81.0204	979.6051	0.9378	1.5327
17	2018	4	2.2571	89.5510	907.4296	0.1194	2.2878
55	2018	4	7.0571	80.4592	881.0684	1.6480	1.8633
46	2018	4	3.1500	84.9796	927.4704	0.3867	1.2796
86	2018	4	6.0429	82.8980	869.2122	0.8673	1.6704
2	2018	4	6.0429	82.8980	869.2122	0.8673	1.6704
4	2018	4	3.0571	85.9694	907.1296	0.2592	1.3510
47	2018	4	11.3000	76.9184	965.6245	1.0051	0.3959
82	2018	4	4.1857	84.8878	878.5500	0.6490	2.2908

19	2018	4	8.2000	81.6735	968.1214	0.4653	0.8653
20	2018	4	5.7000	81.9490	856.6796	1.6510	2.2551
80	2018	4	4.1857	84.8878	878.5500	0.6490	2.2908
3	2018	4	12.4857	72.5102	950.4327	2.2806	1.0500
52	2018	4	2.2571	89.5510	907.4296	0.1194	2.2878
70	2018	4	4.4286	82.7245	915.8184	0.8796	1.1143
64	2018	4	6.0857	75.6837	775.0653	5.1490	1.7980
48	2018	4	2.5429	86.6939	924.9000	0.5980	1.3776
65	2018	4	2.2571	89.5510	907.4296	0.1194	2.2878
44	2018	4	4.4286	82.7245	915.8184	0.8796	1.1143
75	2018	4	6.0857	75.6837	775.0653	5.1490	1.7980
40	2018	4	2.3000	87.9592	953.3041	0.5367	1.2765
11	2018	4	7.0571	80.4592	881.0684	1.6480	1.8633
35	2018	4	1.3429	85.5102	946.8357	0.7184	1.4908
78	2018	4	7.2857	81.5204	904.1071	0.6041	1.6408
28	2018	4	2.3286	85.5612	935.7602	0.3388	1.4857
39	2018	4	2.2571	89.5510	907.4296	0.1194	2.2878
24	2018	4	4.3167	81.7245	947.1367	0.3724	1.1541
63	2018	4	2.3000	87.9592	953.3041	0.5367	1.2765
62	2018	4	3.5714	83.5612	878.0959	1.5408	1.2102
1	2018	4	4.1857	84.8878	878.5500	0.6490	2.2908
31	2018	5	-1.8143	83.9694	848.6653	1.2969	1.2633
79	2018	5	1.9429	80.5204	981.8867	0.3500	1.9276
51	2018	5	0.1857	87.5918	948.5388	0.2153	1.8071
14	2018	5	-0.2429	88.6939	902.4990	0.1439	2.3153
67	2018	5	-0.7571	93.1020	908.2429	0.0786	2.3245
42	2018	5	-1.2429	89.8776	878.9643	0.2878	2.6367
50	2018	5	0.2714	89.5510	908.0367	0.0000	1.6337
43	2018	5	-1.2429	89.8776	878.9643	0.2878	2.6367
85	2018	5	1.0333	85.8776	916.5592	0.3296	1.1837
25	2018	5	4.6857	77.2143	987.7500	0.3490	1.3929
69	2018	5	1.9286	80.0306	948.4745	0.0367	1.5071
57	2018	5	-1.2429	95.1531	891.8878	0.2000	1.6857
9	2018	5	-1.3143	86.8878	856.7490	1.2122	2.5357
72	2018	5	0.5000	81.6837	881.2092	1.5347	2.2051
26	2018	5	1.3286	84.2143	869.6459	2.3296	2.1969
7	2018	5	0.4857	83.6633	861.6082	2.9878	2.1276
83	2018	5	6.7286	72.1633	951.0276	1.6898	1.1714
76	2018	5	0.6143	87.1122	928.5959	0.0000	1.3439
36	2018	5	0.6429	83.8265	937.1347	0.1949	1.9755
81	2018	5	0.1857	87.5918	948.5388	0.2153	1.8071
15	2018	5	1.2571	86.4796	926.1092	0.0000	1.5306
32	2018	5	-1.2429	89.8776	878.9643	0.2878	2.6367
73	2018	5	3.3286	75.6939	972.9286	0.0000	0.8286
71	2018	5	0.6429	83.8265	937.1347	0.1949	1.9755
41	2018	5	-0.7143	91.6327	878.7041	0.8643	1.2990
10	2018	5	1.6714	83.8878	975.1888	0.2827	1.1898
23	2018	5	-4.7714	76.8265	774.3980	4.5724	2.0429
27	2018	5	0.4857	83.6633	861.6082	2.9878	2.1276
60	2018	5	0.1857	87.5918	948.5388	0.2153	1.8071

53	2018	5	-1.3143	86.8878	856.7490	1.2122	2.5357
66	2018	5	-0.2429	88.6939	902.4990	0.1439	2.3153
59	2018	5	-1.2429	95.1531	891.8878	0.2000	1.6857
61	2018	5	3.3286	75.6939	972.9286	0.0000	0.8286
84	2018	5	3.3286	75.6939	972.9286	0.0000	0.8286
38	2018	5	-1.2429	95.1531	891.8878	0.2000	1.6857
87	2018	5	1.6000	81.4490	904.5959	0.3980	2.1878
34	2018	5	-1.2429	95.1531	891.8878	0.2000	1.6857
29	2018	5	1.9286	80.0306	948.4745	0.0367	1.5071
5	2018	5	-1.8571	85.3061	833.4714	3.1898	2.0857
8	2018	5	-1.3143	86.8878	856.7490	1.2122	2.5357
12	2018	5	-1.8571	85.3061	833.4714	3.1898	2.0857
13	2018	5	6.7286	72.1633	951.0276	1.6898	1.1714
18	2018	5	1.7571	84.3571	975.7276	0.1898	1.0214
33	2018	5	0.2714	89.5510	908.0367	0.0000	1.6337
56	2018	5	4.6857	77.2143	987.7500	0.3490	1.3929
77	2018	5	1.2571	86.4796	926.1092	0.0000	1.5306
54	2018	5	-1.8571	85.3061	833.4714	3.1898	2.0857
21	2018	5	0.2714	89.5510	908.0367	0.0000	1.6337
68	2018	5	1.9429	80.5204	981.8867	0.3500	1.9276
74	2018	5	3.3286	75.6939	972.9286	0.0000	0.8286
88	2018	5	-1.2429	89.8776	878.9643	0.2878	2.6367
16	2018	5	0.6143	87.1122	928.5959	0.0000	1.3439
30	2018	5	-0.2429	88.6939	902.4990	0.1439	2.3153
6	2018	5	1.9429	80.5204	981.8867	0.3500	1.9276
49	2018	5	1.9286	80.0306	948.4745	0.0367	1.5071
22	2018	5	-1.2429	89.8776	878.9643	0.2878	2.6367
45	2018	5	-1.4857	74.0408	817.7357	4.3908	2.1561
58	2018	5	1.9286	80.0306	948.4745	0.0367	1.5071
37	2018	5	1.9429	80.5204	981.8867	0.3500	1.9276
17	2018	5	-0.7571	93.1020	908.2429	0.0786	2.3245
55	2018	5	0.5000	81.6837	881.2092	1.5347	2.2051
46	2018	5	0.6143	87.1122	928.5959	0.0000	1.3439
86	2018	5	-0.4429	89.4082	869.5684	0.5224	1.3827
2	2018	5	-0.4429	89.4082	869.5684	0.5224	1.3827
4	2018	5	0.2714	89.5510	908.0367	0.0000	1.6337
47	2018	5	6.3714	73.3469	966.5694	0.8469	0.6276
82	2018	5	-1.2429	89.8776	878.9643	0.2878	2.6367
19	2018	5	4.7143	76.5612	969.2398	0.2378	1.3347
20	2018	5	-1.3143	86.8878	856.7490	1.2122	2.5357
80	2018	5	-1.2429	89.8776	878.9643	0.2878	2.6367
3	2018	5	6.7286	72.1633	951.0276	1.6898	1.1714
52	2018	5	-0.7571	93.1020	908.2429	0.0786	2.3245
70	2018	5	1.0333	85.8776	916.5592	0.3296	1.1837
64	2018	5	-4.7714	76.8265	774.3980	4.5724	2.0429
48	2018	5	1.2571	86.4796	926.1092	0.0000	1.5306
65	2018	5	-0.7571	93.1020	908.2429	0.0786	2.3245
44	2018	5	1.0333	85.8776	916.5592	0.3296	1.1837
75	2018	5	-4.7714	76.8265	774.3980	4.5724	2.0429
40	2018	5	0.5857	88.3980	954.7663	0.4582	1.6133

11	2018	5	0.5000	81.6837	881.2092	1.5347	2.2051
35	2018	5	0.1857	87.5918	948.5388	0.2153	1.8071
78	2018	5	1.6000	81.4490	904.5959	0.3980	2.1878
28	2018	5	0.6429	83.8265	937.1347	0.1949	1.9755
39	2018	5	-0.7571	93.1020	908.2429	0.0786	2.3245
24	2018	5	1.9286	80.0306	948.4745	0.0367	1.5071
63	2018	5	0.5857	88.3980	954.7663	0.4582	1.6133
62	2018	5	-0.7143	91.6327	878.7041	0.8643	1.2990
1	2018	5	-1.2429	89.8776	878.9643	0.2878	2.6367
31	2018	6	0.7714	83.1735	851.9020	0.1541	1.2439
79	2018	6	6.1000	67.9490	985.5571	1.4102	1.4918
51	2018	6	4.9000	73.7449	952.1704	0.7918	1.4510
14	2018	6	3.7286	70.1939	906.1398	0.7480	2.0051
67	2018	6	4.2333	77.6020	911.4337	0.9500	2.5061
42	2018	6	2.8857	77.2755	882.5388	0.4071	2.5378
50	2018	6	2.9857	84.0204	911.9235	0.1071	1.5041
43	2018	6	2.8857	77.2755	882.5388	0.4071	2.5378
85	2018	6	3.6571	80.3367	920.4867	0.2918	1.1806
25	2018	6	7.0286	63.5102	992.0561	1.5245	1.3990
69	2018	6	5.5857	68.1327	952.6929	0.6000	1.5480
57	2018	6	2.3286	87.1429	895.6388	0.3786	1.5418
9	2018	6	1.7571	77.6429	860.0786	0.4806	2.4163
72	2018	6	3.2857	69.2755	884.8673	0.7602	2.1714
26	2018	6	3.6571	79.4388	873.2939	0.6990	1.8051
7	2018	6	2.7857	80.5408	865.1337	0.8194	1.7816
83	2018	6	8.4000	61.4388	955.8878	0.9816	1.1306
76	2018	6	3.9857	78.0918	932.5173	0.5347	1.0684
36	2018	6	5.2143	67.0408	940.9429	1.1469	1.8102
81	2018	6	4.9000	73.7449	952.1704	0.7918	1.4510
15	2018	6	3.9714	76.5408	929.9194	0.4898	1.5398
32	2018	6	2.8857	77.2755	882.5388	0.4071	2.5378
73	2018	6	7.0000	64.3265	976.9112	0.7745	0.8765
71	2018	6	5.2143	67.0408	940.9429	1.1469	1.8102
41	2018	6	2.2429	83.9592	882.3867	0.2449	1.1724
10	2018	6	5.9286	68.4592	979.1194	1.1755	1.0592
23	2018	6	-2.2857	86.3265	776.4245	1.4071	1.7602
27	2018	6	2.7857	80.5408	865.1337	0.8194	1.7816
60	2018	6	4.9000	73.7449	952.1704	0.7918	1.4510
53	2018	6	1.7571	77.6429	860.0786	0.4806	2.4163
66	2018	6	3.7286	70.1939	906.1398	0.7480	2.0051
59	2018	6	2.3286	87.1429	895.6388	0.3786	1.5418
61	2018	6	7.0000	64.3265	976.9112	0.7745	0.8765
84	2018	6	7.0000	64.3265	976.9112	0.7745	0.8765
38	2018	6	2.3286	87.1429	895.6388	0.3786	1.5418
87	2018	6	4.6857	68.8878	908.5531	0.3990	2.2898
34	2018	6	2.3286	87.1429	895.6388	0.3786	1.5418
29	2018	6	5.5857	68.1327	952.6929	0.6000	1.5480
5	2018	6	0.4429	87.7755	836.5520	0.7020	1.6918
8	2018	6	1.7571	77.6429	860.0786	0.4806	2.4163
12	2018	6	0.4429	87.7755	836.5520	0.7020	1.6918

13	2018	6	8.4000	61.4388	955.8878	0.9816	1.1306
18	2018	6	5.8429	71.2551	979.3663	0.9133	0.8510
33	2018	6	2.9857	84.0204	911.9235	0.1071	1.5041
56	2018	6	7.0286	63.5102	992.0561	1.5245	1.3990
77	2018	6	3.9714	76.5408	929.9194	0.4898	1.5398
54	2018	6	0.4429	87.7755	836.5520	0.7020	1.6918
21	2018	6	2.9857	84.0204	911.9235	0.1071	1.5041
68	2018	6	6.1000	67.9490	985.5571	1.4102	1.4918
74	2018	6	7.0000	64.3265	976.9112	0.7745	0.8765
88	2018	6	2.8857	77.2755	882.5388	0.4071	2.5378
16	2018	6	3.9857	78.0918	932.5173	0.5347	1.0684
30	2018	6	3.7286	70.1939	906.1398	0.7480	2.0051
6	2018	6	6.1000	67.9490	985.5571	1.4102	1.4918
49	2018	6	5.5857	68.1327	952.6929	0.6000	1.5480
22	2018	6	2.8857	77.2755	882.5388	0.4071	2.5378
45	2018	6	0.4143	83.6531	820.5194	1.6245	1.7316
58	2018	6	5.5857	68.1327	952.6929	0.6000	1.5480
37	2018	6	6.1000	67.9490	985.5571	1.4102	1.4918
17	2018	6	4.2333	77.6020	911.4337	0.9500	2.5061
55	2018	6	3.2857	69.2755	884.8673	0.7602	2.1714
46	2018	6	3.9857	78.0918	932.5173	0.5347	1.0684
86	2018	6	2.4000	82.3878	873.2000	0.0969	1.1612
2	2018	6	2.4000	82.3878	873.2000	0.0969	1.1612
4	2018	6	2.9857	84.0204	911.9235	0.1071	1.5041
47	2018	6	8.2000	60.1837	971.4776	1.1633	0.6806
82	2018	6	2.8857	77.2755	882.5388	0.4071	2.5378
19	2018	6	7.8286	59.1633	973.6633	1.1194	1.3541
20	2018	6	1.7571	77.6429	860.0786	0.4806	2.4163
80	2018	6	2.8857	77.2755	882.5388	0.4071	2.5378
3	2018	6	8.4000	61.4388	955.8878	0.9816	1.1306
52	2018	6	4.2333	77.6020	911.4337	0.9500	2.5061
70	2018	6	3.6571	80.3367	920.4867	0.2918	1.1806
64	2018	6	-2.2857	86.3265	776.4245	1.4071	1.7602
48	2018	6	3.9714	76.5408	929.9194	0.4898	1.5398
65	2018	6	4.2333	77.6020	911.4337	0.9500	2.5061
44	2018	6	3.6571	80.3367	920.4867	0.2918	1.1806
75	2018	6	-2.2857	86.3265	776.4245	1.4071	1.7602
40	2018	6	5.1857	71.8571	958.4265	1.4724	1.3847
11	2018	6	3.2857	69.2755	884.8673	0.7602	2.1714
35	2018	6	4.9000	73.7449	952.1704	0.7918	1.4510
78	2018	6	4.6857	68.8878	908.5531	0.3990	2.2898
28	2018	6	5.2143	67.0408	940.9429	1.1469	1.8102
39	2018	6	4.2333	77.6020	911.4337	0.9500	2.5061
24	2018	6	5.5857	68.1327	952.6929	0.6000	1.5480
63	2018	6	5.1857	71.8571	958.4265	1.4724	1.3847
62	2018	6	2.2429	83.9592	882.3867	0.2449	1.1724
1	2018	6	2.8857	77.2755	882.5388	0.4071	2.5378
31	2018	7	7.0286	83.0816	851.6786	1.0429	1.0133
79	2018	7	10.1857	61.0918	983.0133	1.9571	1.1990
51	2018	7	10.6286	63.8061	950.2459	1.3959	1.2694

14	2018	7	11.4143	64.4796	905.6020	1.5153	1.7694
67	2018	7	11.3714	67.8163	910.4112	1.6020	2.6000
42	2018	7	10.6714	68.2551	882.0643	1.1867	2.2388
50	2018	7	9.5714	81.5204	910.9551	0.8184	1.3143
43	2018	7	10.6714	68.2551	882.0643	1.1867	2.2388
85	2018	7	9.7286	79.8367	919.7531	1.0612	1.2398
25	2018	7	14.4000	63.3265	990.0541	1.9041	0.8653
69	2018	7	12.4429	62.0816	951.0990	1.0082	1.3857
57	2018	7	10.2429	77.8673	894.8092	1.0500	1.7296
9	2018	7	9.4286	75.0918	860.0418	1.1255	2.1878
72	2018	7	11.3571	67.1429	884.7031	1.5245	1.7837
26	2018	7	12.9714	76.4082	873.4949	1.7510	1.5551
7	2018	7	12.4429	77.6837	865.2561	1.6837	1.7020
83	2018	7	16.3000	61.3878	955.1867	1.7796	0.9122
76	2018	7	11.0143	70.0714	931.1755	1.1684	0.9888
36	2018	7	11.8714	60.3265	939.3551	1.7388	1.4959
81	2018	7	10.6286	63.8061	950.2459	1.3959	1.2694
15	2018	7	9.5286	70.9592	928.6031	1.1020	1.5939
32	2018	7	10.6714	68.2551	882.0643	1.1867	2.2388
73	2018	7	12.0143	59.7245	974.6929	1.5510	0.7776
71	2018	7	11.8714	60.3265	939.3551	1.7388	1.4959
41	2018	7	9.2143	79.4082	881.8051	1.1980	1.1173
10	2018	7	11.4000	61.9796	976.8439	1.4755	0.8418
23	2018	7	8.9429	85.1633	777.1173	2.0541	1.4276
27	2018	7	12.4429	77.6837	865.2561	1.6837	1.7020
60	2018	7	10.6286	63.8061	950.2459	1.3959	1.2694
53	2018	7	9.4286	75.0918	860.0418	1.1255	2.1878
66	2018	7	11.4143	64.4796	905.6020	1.5153	1.7694
59	2018	7	10.2429	77.8673	894.8092	1.0500	1.7296
61	2018	7	12.0143	59.7245	974.6929	1.5510	0.7776
84	2018	7	12.0143	59.7245	974.6929	1.5510	0.7776
38	2018	7	10.2429	77.8673	894.8092	1.0500	1.7296
87	2018	7	12.8143	63.8673	907.9459	1.3939	1.8520
34	2018	7	10.2429	77.8673	894.8092	1.0500	1.7296
29	2018	7	12.4429	62.0816	951.0990	1.0082	1.3857
5	2018	7	11.5571	83.5510	836.8980	1.3531	1.5898
8	2018	7	9.4286	75.0918	860.0418	1.1255	2.1878
12	2018	7	11.5571	83.5510	836.8980	1.3531	1.5898
13	2018	7	16.3000	61.3878	955.1867	1.7796	0.9122
18	2018	7	9.7286	65.2857	976.9929	1.3786	0.7796
33	2018	7	9.5714	81.5204	910.9551	0.8184	1.3143
56	2018	7	14.4000	63.3265	990.0541	1.9041	0.8653
77	2018	7	9.5286	70.9592	928.6031	1.1020	1.5939
54	2018	7	11.5571	83.5510	836.8980	1.3531	1.5898
21	2018	7	9.5714	81.5204	910.9551	0.8184	1.3143
68	2018	7	10.1857	61.0918	983.0133	1.9571	1.1990
74	2018	7	12.0143	59.7245	974.6929	1.5510	0.7776
88	2018	7	10.6714	68.2551	882.0643	1.1867	2.2388
16	2018	7	11.0143	70.0714	931.1755	1.1684	0.9888
30	2018	7	11.4143	64.4796	905.6020	1.5153	1.7694

6	2018	7	10.1857	61.0918	983.0133	1.9571	1.1990
49	2018	7	12.4429	62.0816	951.0990	1.0082	1.3857
22	2018	7	10.6714	68.2551	882.0643	1.1867	2.2388
45	2018	7	12.3143	83.1020	821.1398	1.6449	1.5827
58	2018	7	12.4429	62.0816	951.0990	1.0082	1.3857
37	2018	7	10.1857	61.0918	983.0133	1.9571	1.1990
17	2018	7	11.3714	67.8163	910.4112	1.6020	2.6000
55	2018	7	11.3571	67.1429	884.7031	1.5245	1.7837
46	2018	7	11.0143	70.0714	931.1755	1.1684	0.9888
86	2018	7	9.5857	80.4898	872.9041	1.0408	1.4082
2	2018	7	9.5857	80.4898	872.9041	1.0408	1.4082
4	2018	7	9.5714	81.5204	910.9551	0.8184	1.3143
47	2018	7	15.8143	59.9388	970.3439	1.7847	0.4908
82	2018	7	10.6714	68.2551	882.0643	1.1867	2.2388
19	2018	7	15.2571	54.4898	972.3378	1.4765	1.1020
20	2018	7	9.4286	75.0918	860.0418	1.1255	2.1878
80	2018	7	10.6714	68.2551	882.0643	1.1867	2.2388
3	2018	7	16.3000	61.3878	955.1867	1.7796	0.9122
52	2018	7	11.3714	67.8163	910.4112	1.6020	2.6000
70	2018	7	9.7286	79.8367	919.7531	1.0612	1.2398
64	2018	7	8.9429	85.1633	777.1173	2.0541	1.4276
48	2018	7	9.5286	70.9592	928.6031	1.1020	1.5939
65	2018	7	11.3714	67.8163	910.4112	1.6020	2.6000
44	2018	7	9.7286	79.8367	919.7531	1.0612	1.2398
75	2018	7	8.9429	85.1633	777.1173	2.0541	1.4276
40	2018	7	11.9000	62.8163	956.7296	1.9204	1.2357
11	2018	7	11.3571	67.1429	884.7031	1.5245	1.7837
35	2018	7	10.6286	63.8061	950.2459	1.3959	1.2694
78	2018	7	12.8143	63.8673	907.9459	1.3939	1.8520
28	2018	7	11.8714	60.3265	939.3551	1.7388	1.4959
39	2018	7	11.3714	67.8163	910.4112	1.6020	2.6000
24	2018	7	12.4429	62.0816	951.0990	1.0082	1.3857
63	2018	7	11.9000	62.8163	956.7296	1.9204	1.2357
62	2018	7	9.2143	79.4082	881.8051	1.1980	1.1173
1	2018	7	10.6714	68.2551	882.0643	1.1867	2.2388
31	2018	8	6.8857	78.8571	848.9878	2.7439	0.9694
79	2018	8	9.0429	70.0918	978.8010	1.6173	1.5510
51	2018	8	7.1571	74.7143	946.3898	1.6163	1.5827
14	2018	8	8.4143	77.3163	902.2306	2.2316	2.2204
67	2018	8	7.4857	75.6122	907.3184	1.9612	3.0827
42	2018	8	7.1143	73.8878	878.8806	2.2122	2.4745
50	2018	8	7.7143	81.0510	907.1806	1.7551	1.4296
43	2018	8	7.1143	73.8878	878.8806	2.2122	2.4745
85	2018	8	8.6143	78.7551	915.9592	2.0602	1.4163
25	2018	8	11.6143	72.1122	985.3061	1.8337	1.0653
69	2018	8	8.7143	69.3980	946.8969	1.1235	1.5500
57	2018	8	7.0857	76.7245	891.2827	1.9316	2.1745
9	2018	8	7.5857	78.7143	857.1878	2.3092	2.5020
72	2018	8	9.5143	72.8980	881.4031	3.1663	2.0306
26	2018	8	12.2000	72.7449	870.2990	4.6827	2.0235

7	2018	8	11.0286	74.2653	862.2000	4.3837	2.0888
83	2018	8	15.4571	65.6224	950.2949	3.5663	1.0510
76	2018	8	7.6286	73.4694	927.2153	1.5459	1.2592
36	2018	8	7.8714	71.5408	935.4388	1.8918	1.7776
81	2018	8	7.1571	74.7143	946.3898	1.6163	1.5827
15	2018	8	7.5857	75.8265	924.7480	1.2847	1.7337
32	2018	8	7.1143	73.8878	878.8806	2.2122	2.4745
73	2018	8	9.9857	65.9286	970.3143	1.5378	0.8235
71	2018	8	7.8714	71.5408	935.4388	1.8918	1.7776
41	2018	8	8.0000	78.4388	878.4796	2.3520	1.3959
10	2018	8	8.4857	75.1020	972.6724	1.1143	0.9347
23	2018	8	6.2143	68.4592	775.9296	5.5980	1.7184
27	2018	8	11.0286	74.2653	862.2000	4.3837	2.0888
60	2018	8	7.1571	74.7143	946.3898	1.6163	1.5827
53	2018	8	7.5857	78.7143	857.1878	2.3092	2.5020
66	2018	8	8.4143	77.3163	902.2306	2.2316	2.2204
59	2018	8	7.0857	76.7245	891.2827	1.9316	2.1745
61	2018	8	9.9857	65.9286	970.3143	1.5378	0.8235
84	2018	8	9.9857	65.9286	970.3143	1.5378	0.8235
38	2018	8	7.0857	76.7245	891.2827	1.9316	2.1745
87	2018	8	10.1143	68.9286	904.2714	2.6929	2.2163
34	2018	8	7.0857	76.7245	891.2827	1.9316	2.1745
29	2018	8	8.7143	69.3980	946.8969	1.1235	1.5500
5	2018	8	9.2000	71.7143	834.3898	4.6796	2.1582
8	2018	8	7.5857	78.7143	857.1878	2.3092	2.5020
12	2018	8	9.2000	71.7143	834.3898	4.6796	2.1582
13	2018	8	15.4571	65.6224	950.2949	3.5663	1.0510
18	2018	8	8.7000	74.5510	972.8990	0.9857	0.9592
33	2018	8	7.7143	81.0510	907.1806	1.7551	1.4296
56	2018	8	11.6143	72.1122	985.3061	1.8337	1.0653
77	2018	8	7.5857	75.8265	924.7480	1.2847	1.7337
54	2018	8	9.2000	71.7143	834.3898	4.6796	2.1582
21	2018	8	7.7143	81.0510	907.1806	1.7551	1.4296
68	2018	8	9.0429	70.0918	978.8010	1.6173	1.5510
74	2018	8	9.9857	65.9286	970.3143	1.5378	0.8235
88	2018	8	7.1143	73.8878	878.8806	2.2122	2.4745
16	2018	8	7.6286	73.4694	927.2153	1.5459	1.2592
30	2018	8	8.4143	77.3163	902.2306	2.2316	2.2204
6	2018	8	9.0429	70.0918	978.8010	1.6173	1.5510
49	2018	8	8.7143	69.3980	946.8969	1.1235	1.5500
22	2018	8	7.1143	73.8878	878.8806	2.2122	2.4745
45	2018	8	11.4000	61.3571	819.0765	5.1551	2.5163
58	2018	8	8.7143	69.3980	946.8969	1.1235	1.5500
37	2018	8	9.0429	70.0918	978.8010	1.6173	1.5510
17	2018	8	7.4857	75.6122	907.3184	1.9612	3.0827
55	2018	8	9.5143	72.8980	881.4031	3.1663	2.0306
46	2018	8	7.6286	73.4694	927.2153	1.5459	1.2592
86	2018	8	8.1571	80.5306	869.7510	2.5347	1.7357
2	2018	8	8.1571	80.5306	869.7510	2.5347	1.7357
4	2018	8	7.7143	81.0510	907.1806	1.7551	1.4296

47	2018	8	14.3571	67.3163	965.4112	2.4622	0.5949
82	2018	8	7.1143	73.8878	878.8806	2.2122	2.4745
19	2018	8	12.5429	65.8265	967.8633	1.2378	1.3816
20	2018	8	7.5857	78.7143	857.1878	2.3092	2.5020
80	2018	8	7.1143	73.8878	878.8806	2.2122	2.4745
3	2018	8	15.4571	65.6224	950.2949	3.5663	1.0510
52	2018	8	7.4857	75.6122	907.3184	1.9612	3.0827
70	2018	8	8.6143	78.7551	915.9592	2.0602	1.4163
64	2018	8	6.2143	68.4592	775.9296	5.5980	1.7184
48	2018	8	7.5857	75.8265	924.7480	1.2847	1.7337
65	2018	8	7.4857	75.6122	907.3184	1.9612	3.0827
44	2018	8	8.6143	78.7551	915.9592	2.0602	1.4163
75	2018	8	6.2143	68.4592	775.9296	5.5980	1.7184
40	2018	8	7.4429	75.8878	952.8235	1.8439	1.7990
11	2018	8	9.5143	72.8980	881.4031	3.1663	2.0306
35	2018	8	7.1571	74.7143	946.3898	1.6163	1.5827
78	2018	8	10.1143	68.9286	904.2714	2.6929	2.2163
28	2018	8	7.8714	71.5408	935.4388	1.8918	1.7776
39	2018	8	7.4857	75.6122	907.3184	1.9612	3.0827
24	2018	8	8.7143	69.3980	946.8969	1.1235	1.5500
63	2018	8	7.4429	75.8878	952.8235	1.8439	1.7990
62	2018	8	8.0000	78.4388	878.4796	2.3520	1.3959
1	2018	8	7.1143	73.8878	878.8806	2.2122	2.4745
31	2018	9	13.1714	72.9286	847.6684	3.6429	1.0531
79	2018	9	13.9571	76.9184	976.4500	1.8582	1.5673
51	2018	9	13.8286	85.1020	944.4010	2.1224	1.4990
14	2018	9	16.6571	81.7449	900.5653	2.4929	2.7337
67	2018	9	16.4286	81.4898	905.6541	2.3561	3.3653
42	2018	9	15.6714	78.1327	877.3408	2.9204	2.5255
50	2018	9	13.3571	81.7449	905.4163	2.5867	1.5633
43	2018	9	15.6714	78.1327	877.3408	2.9204	2.5255
85	2018	9	15.2429	76.8878	913.9531	3.1898	1.3092
25	2018	9	18.4000	77.0918	982.9184	2.1337	1.1357
69	2018	9	16.2714	77.8980	944.8908	1.7347	1.5408
57	2018	9	14.4429	78.5102	889.6439	2.4541	2.0908
9	2018	9	15.9143	74.9490	855.7867	3.3582	2.7673
72	2018	9	17.3857	70.9796	879.7898	4.1347	2.4408
26	2018	9	17.9714	70.7755	868.7153	5.9745	2.3918
7	2018	9	17.4143	71.9592	860.7204	5.1673	2.0908
83	2018	9	21.1000	65.6224	947.9898	4.3265	1.1633
76	2018	9	13.3286	80.6020	925.3990	1.9122	1.3827
36	2018	9	16.6143	78.8571	933.4704	2.2918	1.8204
81	2018	9	13.8286	85.1020	944.4010	2.1224	1.4990
15	2018	9	11.9857	83.5000	922.9327	1.6551	1.5888
32	2018	9	15.6714	78.1327	877.3408	2.9204	2.5255
73	2018	9	15.0571	73.5612	968.1133	1.5388	0.8490
71	2018	9	16.6143	78.8571	933.4704	2.2918	1.8204
41	2018	9	14.9000	76.3061	876.8786	2.9276	1.5296
10	2018	9	15.4143	84.0000	970.4531	1.4153	0.9194
23	2018	9	12.2143	63.0102	775.3010	6.7633	1.9500

27	2018	9	17.4143	71.9592	860.7204	5.1673	2.0908
60	2018	9	13.8286	85.1020	944.4010	2.1224	1.4990
53	2018	9	15.9143	74.9490	855.7867	3.3582	2.7673
66	2018	9	16.6571	81.7449	900.5653	2.4929	2.7337
59	2018	9	14.4429	78.5102	889.6439	2.4541	2.0908
61	2018	9	15.0571	73.5612	968.1133	1.5388	0.8490
84	2018	9	15.0571	73.5612	968.1133	1.5388	0.8490
38	2018	9	14.4429	78.5102	889.6439	2.4541	2.0908
87	2018	9	18.3714	70.2551	902.4265	3.3163	2.6163
34	2018	9	14.4429	78.5102	889.6439	2.4541	2.0908
29	2018	9	16.2714	77.8980	944.8908	1.7347	1.5408
5	2018	9	16.4143	67.2857	833.1541	5.9214	2.3745
8	2018	9	15.9143	74.9490	855.7867	3.3582	2.7673
12	2018	9	16.4143	67.2857	833.1541	5.9214	2.3745
13	2018	9	21.1000	65.6224	947.9898	4.3265	1.1633
18	2018	9	13.4571	81.1633	970.5806	1.2082	1.0082
33	2018	9	13.3571	81.7449	905.4163	2.5867	1.5633
56	2018	9	18.4000	77.0918	982.9184	2.1337	1.1357
77	2018	9	11.9857	83.5000	922.9327	1.6551	1.5888
54	2018	9	16.4143	67.2857	833.1541	5.9214	2.3745
21	2018	9	13.3571	81.7449	905.4163	2.5867	1.5633
68	2018	9	13.9571	76.9184	976.4500	1.8582	1.5673
74	2018	9	15.0571	73.5612	968.1133	1.5388	0.8490
88	2018	9	15.6714	78.1327	877.3408	2.9204	2.5255
16	2018	9	13.3286	80.6020	925.3990	1.9122	1.3827
30	2018	9	16.6571	81.7449	900.5653	2.4929	2.7337
6	2018	9	13.9571	76.9184	976.4500	1.8582	1.5673
49	2018	9	16.2714	77.8980	944.8908	1.7347	1.5408
22	2018	9	15.6714	78.1327	877.3408	2.9204	2.5255
45	2018	9	16.1714	54.2245	817.9551	7.0286	2.8020
58	2018	9	16.2714	77.8980	944.8908	1.7347	1.5408
37	2018	9	13.9571	76.9184	976.4500	1.8582	1.5673
17	2018	9	16.4286	81.4898	905.6541	2.3561	3.3653
55	2018	9	17.3857	70.9796	879.7898	4.1347	2.4408
46	2018	9	13.3286	80.6020	925.3990	1.9122	1.3827
86	2018	9	15.1714	76.9796	868.1918	2.8061	1.4694
2	2018	9	15.1714	76.9796	868.1918	2.8061	1.4694
4	2018	9	13.3571	81.7449	905.4163	2.5867	1.5633
47	2018	9	21.7143	67.1122	962.9306	3.4153	0.7224
82	2018	9	15.6714	78.1327	877.3408	2.9204	2.5255
19	2018	9	20.1286	71.6633	965.6276	1.8714	1.4765
20	2018	9	15.9143	74.9490	855.7867	3.3582	2.7673
80	2018	9	15.6714	78.1327	877.3408	2.9204	2.5255
3	2018	9	21.1000	65.6224	947.9898	4.3265	1.1633
52	2018	9	16.4286	81.4898	905.6541	2.3561	3.3653
70	2018	9	15.2429	76.8878	913.9531	3.1898	1.3092
64	2018	9	12.2143	63.0102	775.3010	6.7633	1.9500
48	2018	9	11.9857	83.5000	922.9327	1.6551	1.5888
65	2018	9	16.4286	81.4898	905.6541	2.3561	3.3653
44	2018	9	15.2429	76.8878	913.9531	3.1898	1.3092

75	2018	9	12.2143	63.0102	775.3010	6.7633	1.9500
40	2018	9	16.2286	85.8673	950.7929	1.8786	1.8561
11	2018	9	17.3857	70.9796	879.7898	4.1347	2.4408
35	2018	9	13.8286	85.1020	944.4010	2.1224	1.4990
78	2018	9	18.3714	70.2551	902.4265	3.3163	2.6163
28	2018	9	16.6143	78.8571	933.4704	2.2918	1.8204
39	2018	9	16.4286	81.4898	905.6541	2.3561	3.3653
24	2018	9	16.2714	77.8980	944.8908	1.7347	1.5408
63	2018	9	16.2286	85.8673	950.7929	1.8786	1.8561
62	2018	9	14.9000	76.3061	876.8786	2.9276	1.5296
1	2018	9	15.6714	78.1327	877.3408	2.9204	2.5255
31	2018	10	9.7429	70.2041	847.1092	4.1816	1.1306
79	2018	10	11.1143	75.9796	975.0806	2.3357	1.6051
51	2018	10	9.6286	82.3673	943.1082	2.9582	1.4776
14	2018	10	10.8429	77.7143	899.7837	2.8959	2.9133
67	2018	10	9.6857	78.4796	904.7918	2.9469	3.1765
42	2018	10	10.2143	74.1735	876.6245	3.8337	2.6643
50	2018	10	10.6429	80.6735	904.5061	3.5612	1.7612
43	2018	10	10.2143	74.1735	876.6245	3.8337	2.6643
85	2018	10	12.1571	73.7245	912.8837	4.0122	1.3653
25	2018	10	13.5000	75.7959	981.5122	3.0918	1.2357
69	2018	10	11.5429	74.7041	943.5745	2.8837	1.4592
57	2018	10	10.1857	75.2653	888.8041	3.1898	2.3898
9	2018	10	10.2429	70.0102	855.2816	4.7051	3.0582
72	2018	10	11.5429	66.7245	879.1398	4.9459	2.6673
26	2018	10	14.6714	66.3980	868.1163	6.8388	2.6592
7	2018	10	12.9714	67.5102	860.1551	6.1378	2.3429
83	2018	10	16.7714	64.1020	946.9776	5.5673	1.2265
76	2018	10	10.1000	79.8367	924.3398	2.6643	1.4204
36	2018	10	10.7000	75.4286	932.2867	3.2143	1.8745
81	2018	10	9.6286	82.3673	943.1082	2.9582	1.4776
15	2018	10	10.2143	82.5510	921.9347	2.2153	1.4480
32	2018	10	10.2143	74.1735	876.6245	3.8337	2.6643
73	2018	10	11.9429	73.1224	966.6643	2.2092	0.8500
71	2018	10	10.7000	75.4286	932.2867	3.2143	1.8745
41	2018	10	10.2000	74.4184	876.1357	3.7949	1.5918
10	2018	10	10.6857	80.2551	969.0245	2.3000	1.0786
23	2018	10	8.1000	65.2449	775.1704	6.8306	2.1184
27	2018	10	12.9714	67.5102	860.1551	6.1378	2.3429
60	2018	10	9.6286	82.3673	943.1082	2.9582	1.4776
53	2018	10	10.2429	70.0102	855.2816	4.7051	3.0582
66	2018	10	10.8429	77.7143	899.7837	2.8959	2.9133
59	2018	10	10.1857	75.2653	888.8041	3.1898	2.3898
61	2018	10	11.9429	73.1224	966.6643	2.2092	0.8500
84	2018	10	11.9429	73.1224	966.6643	2.2092	0.8500
38	2018	10	10.1857	75.2653	888.8041	3.1898	2.3898
87	2018	10	12.1429	67.7041	901.5541	4.2255	2.5276
34	2018	10	10.1857	75.2653	888.8041	3.1898	2.3898
29	2018	10	11.5429	74.7041	943.5745	2.8837	1.4592
5	2018	10	11.1571	65.5918	832.7776	6.7102	2.5980

8	2018	10	10.2429	70.0102	855.2816	4.7051	3.0582
12	2018	10	11.1571	65.5918	832.7776	6.7102	2.5980
13	2018	10	16.7714	64.1020	946.9776	5.5673	1.2265
18	2018	10	10.4143	81.1327	969.2908	1.4684	1.0847
33	2018	10	10.6429	80.6735	904.5061	3.5612	1.7612
56	2018	10	13.5000	75.7959	981.5122	3.0918	1.2357
77	2018	10	10.2143	82.5510	921.9347	2.2153	1.4480
54	2018	10	11.1571	65.5918	832.7776	6.7102	2.5980
21	2018	10	10.6429	80.6735	904.5061	3.5612	1.7612
68	2018	10	11.1143	75.9796	975.0806	2.3357	1.6051
74	2018	10	11.9429	73.1224	966.6643	2.2092	0.8500
88	2018	10	10.2143	74.1735	876.6245	3.8337	2.6643
16	2018	10	10.1000	79.8367	924.3398	2.6643	1.4204
30	2018	10	10.8429	77.7143	899.7837	2.8959	2.9133
6	2018	10	11.1143	75.9796	975.0806	2.3357	1.6051
49	2018	10	11.5429	74.7041	943.5745	2.8837	1.4592
22	2018	10	10.2143	74.1735	876.6245	3.8337	2.6643
45	2018	10	12.1286	58.1633	817.6510	7.6000	2.4051
58	2018	10	11.5429	74.7041	943.5745	2.8837	1.4592
37	2018	10	11.1143	75.9796	975.0806	2.3357	1.6051
17	2018	10	9.6857	78.4796	904.7918	2.9469	3.1765
55	2018	10	11.5429	66.7245	879.1398	4.9459	2.6673
46	2018	10	10.1000	79.8367	924.3398	2.6643	1.4204
86	2018	10	11.0857	73.7857	867.5806	3.3786	1.4296
2	2018	10	11.0857	73.7857	867.5806	3.3786	1.4296
4	2018	10	10.6429	80.6735	904.5061	3.5612	1.7612
47	2018	10	15.4714	66.8367	961.7204	4.4786	0.7133
82	2018	10	10.2143	74.1735	876.6245	3.8337	2.6643
19	2018	10	14.0857	71.1735	964.3714	3.0561	1.4418
20	2018	10	10.2429	70.0102	855.2816	4.7051	3.0582
80	2018	10	10.2143	74.1735	876.6245	3.8337	2.6643
3	2018	10	16.7714	64.1020	946.9776	5.5673	1.2265
52	2018	10	9.6857	78.4796	904.7918	2.9469	3.1765
70	2018	10	12.1571	73.7245	912.8837	4.0122	1.3653
64	2018	10	8.1000	65.2449	775.1704	6.8306	2.1184
48	2018	10	10.2143	82.5510	921.9347	2.2153	1.4480
65	2018	10	9.6857	78.4796	904.7918	2.9469	3.1765
44	2018	10	12.1571	73.7245	912.8837	4.0122	1.3653
75	2018	10	8.1000	65.2449	775.1704	6.8306	2.1184
40	2018	10	9.8714	83.6735	949.6224	2.5653	1.9235
11	2018	10	11.5429	66.7245	879.1398	4.9459	2.6673
35	2018	10	9.6286	82.3673	943.1082	2.9582	1.4776
78	2018	10	12.1429	67.7041	901.5541	4.2255	2.5276
28	2018	10	10.7000	75.4286	932.2867	3.2143	1.8745
39	2018	10	9.6857	78.4796	904.7918	2.9469	3.1765
24	2018	10	11.5429	74.7041	943.5745	2.8837	1.4592
63	2018	10	9.8714	83.6735	949.6224	2.5653	1.9235
62	2018	10	10.2000	74.4184	876.1357	3.7949	1.5918
1	2018	10	10.2143	74.1735	876.6245	3.8337	2.6643
31	2018	11	14.6571	69.7653	847.6337	4.7510	1.1153

79	2018	11	14.4429	75.3878	975.3582	3.1255	1.5969
51	2018	11	13.3571	79.8980	943.4969	3.5633	1.4265
14	2018	11	16.3286	76.1633	900.4949	3.4031	2.7378
67	2018	11	16.3714	76.0510	905.3622	3.8398	2.9837
42	2018	11	15.7714	69.8367	877.2398	4.3990	2.6724
50	2018	11	15.0571	74.6327	904.8061	3.9643	1.7327
43	2018	11	15.7714	69.8367	877.2398	4.3990	2.6724
85	2018	11	17.4286	66.6837	913.0439	4.1663	1.5408
25	2018	11	19.1571	74.2245	981.8663	4.1582	1.2847
69	2018	11	15.3714	72.3673	943.8592	3.7337	1.2796
57	2018	11	15.6000	71.5000	889.2133	4.1255	2.7041
9	2018	11	15.1143	67.4694	855.9980	5.4857	2.9878
72	2018	11	15.9000	67.1429	879.8653	5.0143	2.3714
26	2018	11	16.6857	64.6224	868.8214	7.1918	2.6010
7	2018	11	16.0000	67.1224	860.8776	6.3327	2.4878
83	2018	11	20.7000	63.2245	947.6980	6.1010	1.3265
76	2018	11	14.1143	76.5918	924.6541	3.6704	1.3786
36	2018	11	15.4000	73.0714	932.7531	4.4959	1.7163
81	2018	11	13.3571	79.8980	943.4969	3.5633	1.4265
15	2018	11	14.0857	77.9592	922.1765	2.8776	1.5582
32	2018	11	15.7714	69.8367	877.2398	4.3990	2.6724
73	2018	11	15.4857	72.7755	966.8510	3.0020	0.8194
71	2018	11	15.4000	73.0714	932.7531	4.4959	1.7163
41	2018	11	15.2857	74.0816	876.6235	4.5776	1.4939
10	2018	11	14.4857	78.0102	969.3847	3.3337	1.2214
23	2018	11	11.9143	66.4694	775.9969	6.5776	2.1694
27	2018	11	16.0000	67.1224	860.8776	6.3327	2.4878
60	2018	11	13.3571	79.8980	943.4969	3.5633	1.4265
53	2018	11	15.1143	67.4694	855.9980	5.4857	2.9878
66	2018	11	16.3286	76.1633	900.4949	3.4031	2.7378
59	2018	11	15.6000	71.5000	889.2133	4.1255	2.7041
61	2018	11	15.4857	72.7755	966.8510	3.0020	0.8194
84	2018	11	15.4857	72.7755	966.8510	3.0020	0.8194
38	2018	11	15.6000	71.5000	889.2133	4.1255	2.7041
87	2018	11	17.4000	67.8673	902.2500	4.8469	2.1061
34	2018	11	15.6000	71.5000	889.2133	4.1255	2.7041
29	2018	11	15.3714	72.3673	943.8592	3.7337	1.2796
5	2018	11	14.8000	66.0510	833.5684	7.0531	2.6847
8	2018	11	15.1143	67.4694	855.9980	5.4857	2.9878
12	2018	11	14.8000	66.0510	833.5684	7.0531	2.6847
13	2018	11	20.7000	63.2245	947.6980	6.1010	1.3265
18	2018	11	14.5429	81.4592	969.5704	2.2653	1.0755
33	2018	11	15.0571	74.6327	904.8061	3.9643	1.7327
56	2018	11	19.1571	74.2245	981.8663	4.1582	1.2847
77	2018	11	14.0857	77.9592	922.1765	2.8776	1.5582
54	2018	11	14.8000	66.0510	833.5684	7.0531	2.6847
21	2018	11	15.0571	74.6327	904.8061	3.9643	1.7327
68	2018	11	14.4429	75.3878	975.3582	3.1255	1.5969
74	2018	11	15.4857	72.7755	966.8510	3.0020	0.8194
88	2018	11	15.7714	69.8367	877.2398	4.3990	2.6724

16	2018	11	14.1143	76.5918	924.6541	3.6704	1.3786
30	2018	11	16.3286	76.1633	900.4949	3.4031	2.7378
6	2018	11	14.4429	75.3878	975.3582	3.1255	1.5969
49	2018	11	15.3714	72.3673	943.8592	3.7337	1.2796
22	2018	11	15.7714	69.8367	877.2398	4.3990	2.6724
45	2018	11	14.7857	60.2449	818.3776	7.3857	2.1633
58	2018	11	15.3714	72.3673	943.8592	3.7337	1.2796
37	2018	11	14.4429	75.3878	975.3582	3.1255	1.5969
17	2018	11	16.3714	76.0510	905.3622	3.8398	2.9837
55	2018	11	15.9000	67.1429	879.8653	5.0143	2.3714
46	2018	11	14.1143	76.5918	924.6541	3.6704	1.3786
86	2018	11	15.6714	70.0918	868.2010	4.9061	1.6082
2	2018	11	15.6714	70.0918	868.2010	4.9061	1.6082
4	2018	11	15.0571	74.6327	904.8061	3.9643	1.7327
47	2018	11	20.7000	70.1224	962.4510	4.4020	0.6531
82	2018	11	15.7714	69.8367	877.2398	4.3990	2.6724
19	2018	11	19.7000	73.2245	964.9776	3.7102	1.2786
20	2018	11	15.1143	67.4694	855.9980	5.4857	2.9878
80	2018	11	15.7714	69.8367	877.2398	4.3990	2.6724
3	2018	11	20.7000	63.2245	947.6980	6.1010	1.3265
52	2018	11	16.3714	76.0510	905.3622	3.8398	2.9837
70	2018	11	17.4286	66.6837	913.0439	4.1663	1.5408
64	2018	11	11.9143	66.4694	775.9969	6.5776	2.1694
48	2018	11	14.0857	77.9592	922.1765	2.8776	1.5582
65	2018	11	16.3714	76.0510	905.3622	3.8398	2.9837
44	2018	11	17.4286	66.6837	913.0439	4.1663	1.5408
75	2018	11	11.9143	66.4694	775.9969	6.5776	2.1694
40	2018	11	15.2857	80.0510	950.0806	3.8857	1.8122
11	2018	11	15.9000	67.1429	879.8653	5.0143	2.3714
35	2018	11	13.3571	79.8980	943.4969	3.5633	1.4265
78	2018	11	17.4000	67.8673	902.2500	4.8469	2.1061
28	2018	11	15.4000	73.0714	932.7531	4.4959	1.7163
39	2018	11	16.3714	76.0510	905.3622	3.8398	2.9837
24	2018	11	15.3714	72.3673	943.8592	3.7337	1.2796
63	2018	11	15.2857	80.0510	950.0806	3.8857	1.8122
62	2018	11	15.2857	74.0816	876.6235	4.5776	1.4939
1	2018	11	15.7714	69.8367	877.2398	4.3990	2.6724
31	2018	12	10.6143	72.7449	848.1847	4.5500	1.2245
79	2018	12	11.6429	79.0510	976.0500	2.5429	1.4990
51	2018	12	10.4857	83.6735	944.2898	2.5398	1.2224
14	2018	12	12.2857	77.4694	901.0551	3.2561	2.5122
67	2018	12	11.1286	79.0102	905.9184	3.2888	2.8316
42	2018	12	11.5143	72.7959	877.8061	3.6898	2.6092
50	2018	12	12.1857	75.2551	905.3051	3.3571	1.6194
43	2018	12	11.5143	72.7959	877.8061	3.6898	2.6092
85	2018	12	13.1000	68.3980	913.5061	3.6235	1.6061
25	2018	12	14.4571	76.8265	982.4714	3.7031	1.0827
69	2018	12	12.9167	77.0408	944.2990	3.0316	1.2224
57	2018	12	11.5000	75.3980	889.8010	3.9286	2.5990
9	2018	12	11.5429	69.8571	856.5878	4.8429	2.5643

72	2018	12	13.4000	68.6837	880.4694	4.2571	2.1224
26	2018	12	14.7143	67.6020	869.5337	5.8776	2.2735
7	2018	12	13.8143	70.6735	861.5643	4.9571	2.3367
83	2018	12	18.4857	62.7857	948.4429	5.0684	1.3786
76	2018	12	11.2000	79.2959	925.3357	3.2449	1.3296
36	2018	12	11.5143	77.2449	933.4776	4.2663	1.6755
81	2018	12	10.4857	83.6735	944.2898	2.5398	1.2224
15	2018	12	11.9286	77.8980	922.6378	3.1949	1.7337
32	2018	12	11.5143	72.7959	877.8061	3.6898	2.6092
73	2018	12	12.6571	75.5918	967.6551	2.5163	0.8480
71	2018	12	11.5143	77.2449	933.4776	4.2663	1.6755
41	2018	12	11.5429	78.4694	877.2163	3.8724	1.2929
10	2018	12	11.4714	82.8061	970.2133	2.6990	1.0867
23	2018	12	8.5571	69.0612	776.6194	5.4776	2.1408
27	2018	12	13.8143	70.6735	861.5643	4.9571	2.3367
60	2018	12	10.4857	83.6735	944.2898	2.5398	1.2224
53	2018	12	11.5429	69.8571	856.5878	4.8429	2.5643
66	2018	12	12.2857	77.4694	901.0551	3.2561	2.5122
59	2018	12	11.5000	75.3980	889.8010	3.9286	2.5990
61	2018	12	12.6571	75.5918	967.6551	2.5163	0.8480
84	2018	12	12.6571	75.5918	967.6551	2.5163	0.8480
38	2018	12	11.5000	75.3980	889.8010	3.9286	2.5990
87	2018	12	13.7714	70.9082	902.8643	4.2041	1.8714
34	2018	12	11.5000	75.3980	889.8010	3.9286	2.5990
29	2018	12	12.9167	77.0408	944.2990	3.0316	1.2224
5	2018	12	12.0143	68.3776	834.2337	5.5898	2.4806
8	2018	12	11.5429	69.8571	856.5878	4.8429	2.5643
12	2018	12	12.0143	68.3776	834.2337	5.5898	2.4806
13	2018	12	18.4857	62.7857	948.4429	5.0684	1.3786
18	2018	12	11.4714	85.1224	970.1724	2.3031	0.9061
33	2018	12	12.1857	75.2551	905.3051	3.3571	1.6194
56	2018	12	14.4571	76.8265	982.4714	3.7031	1.0827
77	2018	12	11.9286	77.8980	922.6378	3.1949	1.7337
54	2018	12	12.0143	68.3776	834.2337	5.5898	2.4806
21	2018	12	12.1857	75.2551	905.3051	3.3571	1.6194
68	2018	12	11.6429	79.0510	976.0500	2.5429	1.4990
74	2018	12	12.6571	75.5918	967.6551	2.5163	0.8480
88	2018	12	11.5143	72.7959	877.8061	3.6898	2.6092
16	2018	12	11.2000	79.2959	925.3357	3.2449	1.3296
30	2018	12	12.2857	77.4694	901.0551	3.2561	2.5122
6	2018	12	11.6429	79.0510	976.0500	2.5429	1.4990
49	2018	12	12.9167	77.0408	944.2990	3.0316	1.2224
22	2018	12	11.5143	72.7959	877.8061	3.6898	2.6092
45	2018	12	12.3429	63.3878	819.0041	5.9888	2.1010
58	2018	12	12.9167	77.0408	944.2990	3.0316	1.2224
37	2018	12	11.6429	79.0510	976.0500	2.5429	1.4990
17	2018	12	11.1286	79.0102	905.9184	3.2888	2.8316
55	2018	12	13.4000	68.6837	880.4694	4.2571	2.1224
46	2018	12	11.2000	79.2959	925.3357	3.2449	1.3296
86	2018	12	11.7286	75.2959	868.7847	4.4194	1.4980

2	2018	12	11.7286	75.2959	868.7847	4.4194	1.4980
4	2018	12	12.1857	75.2551	905.3051	3.3571	1.6194
47	2018	12	17.8571	68.7755	963.0724	3.6041	0.6541
82	2018	12	11.5143	72.7959	877.8061	3.6898	2.6092
19	2018	12	16.3714	72.4082	965.5704	3.5490	1.2633
20	2018	12	11.5429	69.8571	856.5878	4.8429	2.5643
80	2018	12	11.5143	72.7959	877.8061	3.6898	2.6092
3	2018	12	18.4857	62.7857	948.4429	5.0684	1.3786
52	2018	12	11.1286	79.0102	905.9184	3.2888	2.8316
70	2018	12	13.1000	68.3980	913.5061	3.6235	1.6061
64	2018	12	8.5571	69.0612	776.6194	5.4776	2.1408
48	2018	12	11.9286	77.8980	922.6378	3.1949	1.7337
65	2018	12	11.1286	79.0102	905.9184	3.2888	2.8316
44	2018	12	13.1000	68.3980	913.5061	3.6235	1.6061
75	2018	12	8.5571	69.0612	776.6194	5.4776	2.1408
40	2018	12	11.6000	81.6122	950.7306	3.3837	1.5133
11	2018	12	13.4000	68.6837	880.4694	4.2571	2.1224
35	2018	12	10.4857	83.6735	944.2898	2.5398	1.2224
78	2018	12	13.7714	70.9082	902.8643	4.2041	1.8714
28	2018	12	11.5143	77.2449	933.4776	4.2663	1.6755
39	2018	12	11.1286	79.0102	905.9184	3.2888	2.8316
24	2018	12	12.9167	77.0408	944.2990	3.0316	1.2224
63	2018	12	11.6000	81.6122	950.7306	3.3837	1.5133
62	2018	12	11.5429	78.4694	877.2163	3.8724	1.2929
1	2018	12	11.5143	72.7959	877.8061	3.6898	2.6092
31	2018	13	13.0143	78.1939	849.3133	2.8704	1.1408
79	2018	13	18.5714	82.0918	976.3061	2.1622	1.3541
51	2018	13	16.9000	86.1122	944.7347	1.7908	1.1408
14	2018	13	16.4143	80.1327	901.5204	2.3571	2.0061
67	2018	13	16.2429	82.4184	906.5306	1.9755	2.2724
42	2018	13	15.0286	80.2449	878.5561	2.3031	2.2918
50	2018	13	14.9714	82.1327	906.2051	2.0806	1.5429
43	2018	13	15.0286	80.2449	878.5561	2.3031	2.2918
85	2018	13	15.6714	77.0102	914.6112	2.5286	1.3755
25	2018	13	19.1429	80.0408	982.8459	3.0531	1.0500
69	2018	13	17.2286	79.4592	944.7398	2.2163	1.2112
57	2018	13	14.4857	81.9898	890.7122	2.7786	2.0735
9	2018	13	14.3714	76.7449	857.3633	2.9102	2.1449
72	2018	13	15.2429	73.6633	881.1255	2.7000	1.9408
26	2018	13	16.4000	73.9286	870.2214	4.0847	2.1184
7	2018	13	15.4143	77.2755	862.3092	3.5255	2.1173
83	2018	13	20.3000	67.4796	948.9214	3.4776	1.2531
76	2018	13	16.2714	83.5918	925.9939	2.1357	1.2918
36	2018	13	17.0714	81.7347	934.0418	3.0949	1.5673
81	2018	13	16.9000	86.1122	944.7347	1.7908	1.1408
15	2018	13	15.6000	82.1735	923.3214	2.7510	1.7327
32	2018	13	15.0286	80.2449	878.5561	2.3031	2.2918
73	2018	13	18.1857	78.0408	968.1429	2.1194	0.7694
71	2018	13	17.0714	81.7347	934.0418	3.0949	1.5673
41	2018	13	14.7571	84.0204	878.2255	2.1633	1.2561

10	2018	13	17.7286	85.1939	970.4867	2.1031	0.9602
23	2018	13	10.0429	77.0612	777.5949	4.0929	1.8776
27	2018	13	15.4143	77.2755	862.3092	3.5255	2.1173
60	2018	13	16.9000	86.1122	944.7347	1.7908	1.1408
53	2018	13	14.3714	76.7449	857.3633	2.9102	2.1449
66	2018	13	16.4143	80.1327	901.5204	2.3571	2.0061
59	2018	13	14.4857	81.9898	890.7122	2.7786	2.0735
61	2018	13	18.1857	78.0408	968.1429	2.1194	0.7694
84	2018	13	18.1857	78.0408	968.1429	2.1194	0.7694
38	2018	13	14.4857	81.9898	890.7122	2.7786	2.0735
87	2018	13	17.2714	75.6939	903.4806	2.8449	1.6704
34	2018	13	14.4857	81.9898	890.7122	2.7786	2.0735
29	2018	13	17.2286	79.4592	944.7398	2.2163	1.2112
5	2018	13	13.9000	75.4082	834.6653	3.5776	2.2245
8	2018	13	14.3714	76.7449	857.3633	2.9102	2.1449
12	2018	13	13.9000	75.4082	834.6653	3.5776	2.2245
13	2018	13	20.3000	67.4796	948.9214	3.4776	1.2531
18	2018	13	17.8714	87.6122	970.4378	2.3347	0.8061
33	2018	13	14.9714	82.1327	906.2051	2.0806	1.5429
56	2018	13	19.1429	80.0408	982.8459	3.0531	1.0500
77	2018	13	15.6000	82.1735	923.3214	2.7510	1.7327
54	2018	13	13.9000	75.4082	834.6653	3.5776	2.2245
21	2018	13	14.9714	82.1327	906.2051	2.0806	1.5429
68	2018	13	18.5714	82.0918	976.3061	2.1622	1.3541
74	2018	13	18.1857	78.0408	968.1429	2.1194	0.7694
88	2018	13	15.0286	80.2449	878.5561	2.3031	2.2918
16	2018	13	16.2714	83.5918	925.9939	2.1357	1.2918
30	2018	13	16.4143	80.1327	901.5204	2.3571	2.0061
6	2018	13	18.5714	82.0918	976.3061	2.1622	1.3541
49	2018	13	17.2286	79.4592	944.7398	2.2163	1.2112
22	2018	13	15.0286	80.2449	878.5561	2.3031	2.2918
45	2018	13	14.0571	71.4184	819.8541	4.0673	1.7051
58	2018	13	17.2286	79.4592	944.7398	2.2163	1.2112
37	2018	13	18.5714	82.0918	976.3061	2.1622	1.3541
17	2018	13	16.2429	82.4184	906.5306	1.9755	2.2724
55	2018	13	15.2429	73.6633	881.1255	2.7000	1.9408
46	2018	13	16.2714	83.5918	925.9939	2.1357	1.2918
86	2018	13	14.5143	84.4082	869.7163	2.5449	1.0214
2	2018	13	14.5143	84.4082	869.7163	2.5449	1.0214
4	2018	13	14.9714	82.1327	906.2051	2.0806	1.5429
47	2018	13	20.0000	72.5714	963.5082	2.5867	0.5724
82	2018	13	15.0286	80.2449	878.5561	2.3031	2.2918
19	2018	13	20.4143	72.0816	965.7622	2.6969	1.2173
20	2018	13	14.3714	76.7449	857.3633	2.9102	2.1449
80	2018	13	15.0286	80.2449	878.5561	2.3031	2.2918
3	2018	13	20.3000	67.4796	948.9214	3.4776	1.2531
52	2018	13	16.2429	82.4184	906.5306	1.9755	2.2724
70	2018	13	15.6714	77.0102	914.6112	2.5286	1.3755
64	2018	13	10.0429	77.0612	777.5949	4.0929	1.8776
48	2018	13	15.6000	82.1735	923.3214	2.7510	1.7327

65	2018	13	16.2429	82.4184	906.5306	1.9755	2.2724
44	2018	13	15.6714	77.0102	914.6112	2.5286	1.3755
75	2018	13	10.0429	77.0612	777.5949	4.0929	1.8776
40	2018	13	17.1143	83.2041	951.0622	2.8286	1.2908
11	2018	13	15.2429	73.6633	881.1255	2.7000	1.9408
35	2018	13	16.9000	86.1122	944.7347	1.7908	1.1408
78	2018	13	17.2714	75.6939	903.4806	2.8449	1.6704
28	2018	13	17.0714	81.7347	934.0418	3.0949	1.5673
39	2018	13	16.2429	82.4184	906.5306	1.9755	2.2724
24	2018	13	17.2286	79.4592	944.7398	2.2163	1.2112
63	2018	13	17.1143	83.2041	951.0622	2.8286	1.2908
62	2018	13	14.7571	84.0204	878.2255	2.1633	1.2561
1	2018	13	15.0286	80.2449	878.5561	2.3031	2.2918
31	2018	14	13.7857	81.1939	849.9031	2.5510	0.8694
79	2018	14	17.6286	79.0510	974.9878	3.0214	1.2061
51	2018	14	15.9857	83.4082	944.0633	2.5276	1.0980
14	2018	14	15.3429	84.7449	901.7898	1.5929	1.9265
67	2018	14	15.2571	83.2857	906.7194	2.4796	2.0673
42	2018	14	15.0429	83.0612	878.9827	2.6776	2.0041
50	2018	14	15.9857	81.9388	906.3898	2.5347	1.4561
43	2018	14	15.0429	83.0612	878.9827	2.6776	2.0041
85	2018	14	17.1429	78.9694	914.8204	2.9378	1.2051
25	2018	14	18.5571	83.7755	982.3969	2.9663	0.8265
69	2018	14	17.4714	79.1122	944.6531	2.1031	1.0429
57	2018	14	15.6143	82.9082	890.9867	3.1745	2.1418
9	2018	14	14.4857	80.3367	857.8816	3.5102	1.9684
72	2018	14	14.9714	80.4286	881.5898	2.6031	1.6867
26	2018	14	17.3143	79.8061	870.5806	4.6184	2.1694
7	2018	14	16.4857	83.3776	862.7724	3.9082	2.0633
83	2018	14	20.5714	71.7959	949.0633	3.8745	1.1449
76	2018	14	16.4000	81.8673	925.7194	2.5694	1.2459
36	2018	14	17.1571	80.6939	933.7857	3.3357	1.3408
81	2018	14	15.9857	83.4082	944.0633	2.5276	1.0980
15	2018	14	16.1857	81.4796	923.2510	2.8520	1.6388
32	2018	14	15.0429	83.0612	878.9827	2.6776	2.0041
73	2018	14	18.0429	77.3469	967.2827	2.8398	0.6918
71	2018	14	17.1571	80.6939	933.7857	3.3357	1.3408
41	2018	14	15.4000	84.9490	878.6051	2.2286	1.3796
10	2018	14	17.0000	81.9694	969.4133	2.6255	0.9306
23	2018	14	11.4857	79.9184	778.4418	4.4296	1.6367
27	2018	14	16.4857	83.3776	862.7724	3.9082	2.0633
60	2018	14	15.9857	83.4082	944.0633	2.5276	1.0980
53	2018	14	14.4857	80.3367	857.8816	3.5102	1.9684
66	2018	14	15.3429	84.7449	901.7898	1.5929	1.9265
59	2018	14	15.6143	82.9082	890.9867	3.1745	2.1418
61	2018	14	18.0429	77.3469	967.2827	2.8398	0.6918
84	2018	14	18.0429	77.3469	967.2827	2.8398	0.6918
38	2018	14	15.6143	82.9082	890.9867	3.1745	2.1418
87	2018	14	16.7143	78.4796	903.8286	3.3969	1.4745
34	2018	14	15.6143	82.9082	890.9867	3.1745	2.1418

29	2018	14	17.4714	79.1122	944.6531	2.1031	1.0429
5	2018	14	15.0429	82.5714	835.2031	3.9184	2.0214
8	2018	14	14.4857	80.3367	857.8816	3.5102	1.9684
12	2018	14	15.0429	82.5714	835.2031	3.9184	2.0214
13	2018	14	20.5714	71.7959	949.0633	3.8745	1.1449
18	2018	14	17.4286	83.6327	969.2092	3.0602	0.7867
33	2018	14	15.9857	81.9388	906.3898	2.5347	1.4561
56	2018	14	18.5571	83.7755	982.3969	2.9663	0.8265
77	2018	14	16.1857	81.4796	923.2510	2.8520	1.6388
54	2018	14	15.0429	82.5714	835.2031	3.9184	2.0214
21	2018	14	15.9857	81.9388	906.3898	2.5347	1.4561
68	2018	14	17.6286	79.0510	974.9878	3.0214	1.2061
74	2018	14	18.0429	77.3469	967.2827	2.8398	0.6918
88	2018	14	15.0429	83.0612	878.9827	2.6776	2.0041
16	2018	14	16.4000	81.8673	925.7194	2.5694	1.2459
30	2018	14	15.3429	84.7449	901.7898	1.5929	1.9265
6	2018	14	17.6286	79.0510	974.9878	3.0214	1.2061
49	2018	14	17.4714	79.1122	944.6531	2.1031	1.0429
22	2018	14	15.0429	83.0612	878.9827	2.6776	2.0041
45	2018	14	15.3857	75.8980	820.4031	4.4684	1.5092
58	2018	14	17.4714	79.1122	944.6531	2.1031	1.0429
37	2018	14	17.6286	79.0510	974.9878	3.0214	1.2061
17	2018	14	15.2571	83.2857	906.7194	2.4796	2.0673
55	2018	14	14.9714	80.4286	881.5898	2.6031	1.6867
46	2018	14	16.4000	81.8673	925.7194	2.5694	1.2459
86	2018	14	15.4571	84.7857	870.1306	3.2571	1.1041
2	2018	14	15.4571	84.7857	870.1306	3.2571	1.1041
4	2018	14	15.9857	81.9388	906.3898	2.5347	1.4561
47	2018	14	19.5143	79.5714	963.6327	2.8571	0.4735
82	2018	14	15.0429	83.0612	878.9827	2.6776	2.0041
19	2018	14	18.8714	78.0204	965.6357	2.0122	0.9010
20	2018	14	14.4857	80.3367	857.8816	3.5102	1.9684
80	2018	14	15.0429	83.0612	878.9827	2.6776	2.0041
3	2018	14	20.5714	71.7959	949.0633	3.8745	1.1449
52	2018	14	15.2571	83.2857	906.7194	2.4796	2.0673
70	2018	14	17.1429	78.9694	914.8204	2.9378	1.2051
64	2018	14	11.4857	79.9184	778.4418	4.4296	1.6367
48	2018	14	16.1857	81.4796	923.2510	2.8520	1.6388
65	2018	14	15.2571	83.2857	906.7194	2.4796	2.0673
44	2018	14	17.1429	78.9694	914.8204	2.9378	1.2051
75	2018	14	11.4857	79.9184	778.4418	4.4296	1.6367
40	2018	14	17.0857	81.3878	950.4776	3.3561	1.2082
11	2018	14	14.9714	80.4286	881.5898	2.6031	1.6867
35	2018	14	15.9857	83.4082	944.0633	2.5276	1.0980
78	2018	14	16.7143	78.4796	903.8286	3.3969	1.4745
28	2018	14	17.1571	80.6939	933.7857	3.3357	1.3408
39	2018	14	15.2571	83.2857	906.7194	2.4796	2.0673
24	2018	14	17.4714	79.1122	944.6531	2.1031	1.0429
63	2018	14	17.0857	81.3878	950.4776	3.3561	1.2082
62	2018	14	15.4000	84.9490	878.6051	2.2286	1.3796

1	2018	14	15.0429	83.0612	878.9827	2.6776	2.0041
31	2018	15	15.7000	74.6735	848.3990	4.9724	0.9439
79	2018	15	17.9857	75.2245	973.5735	3.4000	1.5735
51	2018	15	17.1286	79.4796	942.7235	2.9908	1.3347
14	2018	15	16.6857	82.9694	900.7551	2.8898	2.6327
67	2018	15	16.4000	80.5102	905.5194	3.5827	3.0255
42	2018	15	16.3143	75.6735	877.7745	4.2531	2.5796
50	2018	15	16.7286	75.4082	904.7388	4.2163	1.7153
43	2018	15	16.3143	75.6735	877.7745	4.2531	2.5796
85	2018	15	18.5286	70.6122	912.9755	4.6633	1.5469
25	2018	15	20.1857	81.0000	981.1031	3.4980	1.0102
69	2018	15	18.6429	75.0204	943.3194	2.7531	1.2643
57	2018	15	16.9286	74.4286	889.5214	5.3796	3.0663
9	2018	15	16.5000	73.5714	856.6755	6.3000	2.6929
72	2018	15	17.1571	75.9898	880.4235	5.2235	1.9194
26	2018	15	20.0000	69.7653	869.3929	7.0969	2.5010
7	2018	15	18.9429	72.6735	861.5612	6.9633	2.3112
83	2018	15	22.4714	66.6224	947.7102	6.5173	1.3327
76	2018	15	17.4000	77.3265	924.1673	3.9245	1.3980
36	2018	15	18.2143	74.0918	932.4439	4.0776	1.7041
81	2018	15	17.1286	79.4796	942.7235	2.9908	1.3347
15	2018	15	16.3571	76.2959	921.6041	3.3959	1.7122
32	2018	15	16.3143	75.6735	877.7745	4.2531	2.5796
73	2018	15	19.0857	72.3878	965.5622	3.6102	1.0245
71	2018	15	18.2143	74.0918	932.4439	4.0776	1.7041
41	2018	15	17.3571	75.3163	877.0561	4.7551	1.7571
10	2018	15	17.8571	78.0510	968.0806	2.9704	1.1214
23	2018	15	13.3571	68.6837	777.5092	6.3520	1.7724
27	2018	15	18.9429	72.6735	861.5612	6.9633	2.3112
60	2018	15	17.1286	79.4796	942.7235	2.9908	1.3347
53	2018	15	16.5000	73.5714	856.6755	6.3000	2.6929
66	2018	15	16.6857	82.9694	900.7551	2.8898	2.6327
59	2018	15	16.9286	74.4286	889.5214	5.3796	3.0663
61	2018	15	19.0857	72.3878	965.5622	3.6102	1.0245
84	2018	15	19.0857	72.3878	965.5622	3.6102	1.0245
38	2018	15	16.9286	74.4286	889.5214	5.3796	3.0663
87	2018	15	18.4429	73.3061	902.6071	5.2510	1.8184
34	2018	15	16.9286	74.4286	889.5214	5.3796	3.0663
29	2018	15	18.6429	75.0204	943.3194	2.7531	1.2643
5	2018	15	17.2000	71.8776	834.3990	6.9898	2.2908
8	2018	15	16.5000	73.5714	856.6755	6.3000	2.6929
12	2018	15	17.2000	71.8776	834.3990	6.9898	2.2908
13	2018	15	22.4714	66.6224	947.7102	6.5173	1.3327
18	2018	15	17.9429	78.0510	967.7122	3.2806	0.9908
33	2018	15	16.7286	75.4082	904.7388	4.2163	1.7153
56	2018	15	20.1857	81.0000	981.1031	3.4980	1.0102
77	2018	15	16.3571	76.2959	921.6041	3.3959	1.7122
54	2018	15	17.2000	71.8776	834.3990	6.9898	2.2908
21	2018	15	16.7286	75.4082	904.7388	4.2163	1.7153
68	2018	15	17.9857	75.2245	973.5735	3.4000	1.5735

74	2018	15	19.0857	72.3878	965.5622	3.6102	1.0245
88	2018	15	16.3143	75.6735	877.7745	4.2531	2.5796
16	2018	15	17.4000	77.3265	924.1673	3.9245	1.3980
30	2018	15	16.6857	82.9694	900.7551	2.8898	2.6327
6	2018	15	17.9857	75.2245	973.5735	3.4000	1.5735
49	2018	15	18.6429	75.0204	943.3194	2.7531	1.2643
22	2018	15	16.3143	75.6735	877.7745	4.2531	2.5796
45	2018	15	17.7714	62.5714	819.3755	7.5133	2.3388
58	2018	15	18.6429	75.0204	943.3194	2.7531	1.2643
37	2018	15	17.9857	75.2245	973.5735	3.4000	1.5735
17	2018	15	16.4000	80.5102	905.5194	3.5827	3.0255
55	2018	15	17.1571	75.9898	880.4235	5.2235	1.9194
46	2018	15	17.4000	77.3265	924.1673	3.9245	1.3980
86	2018	15	17.3857	73.1531	868.6990	5.6449	2.0684
2	2018	15	17.3857	73.1531	868.6990	5.6449	2.0684
4	2018	15	16.7286	75.4082	904.7388	4.2163	1.7153
47	2018	15	21.7857	76.3469	962.3622	4.5367	0.5847
82	2018	15	16.3143	75.6735	877.7745	4.2531	2.5796
19	2018	15	20.1000	79.1633	964.5663	2.5561	1.1112
20	2018	15	16.5000	73.5714	856.6755	6.3000	2.6929
80	2018	15	16.3143	75.6735	877.7745	4.2531	2.5796
3	2018	15	22.4714	66.6224	947.7102	6.5173	1.3327
52	2018	15	16.4000	80.5102	905.5194	3.5827	3.0255
70	2018	15	18.5286	70.6122	912.9755	4.6633	1.5469
64	2018	15	13.3571	68.6837	777.5092	6.3520	1.7724
48	2018	15	16.3571	76.2959	921.6041	3.3959	1.7122
65	2018	15	16.4000	80.5102	905.5194	3.5827	3.0255
44	2018	15	18.5286	70.6122	912.9755	4.6633	1.5469
75	2018	15	13.3571	68.6837	777.5092	6.3520	1.7724
40	2018	15	18.1857	77.6327	949.2918	4.2867	1.8684
11	2018	15	17.1571	75.9898	880.4235	5.2235	1.9194
35	2018	15	17.1286	79.4796	942.7235	2.9908	1.3347
78	2018	15	18.4429	73.3061	902.6071	5.2510	1.8184
28	2018	15	18.2143	74.0918	932.4439	4.0776	1.7041
39	2018	15	16.4000	80.5102	905.5194	3.5827	3.0255
24	2018	15	18.6429	75.0204	943.3194	2.7531	1.2643
63	2018	15	18.1857	77.6327	949.2918	4.2867	1.8684
62	2018	15	17.3571	75.3163	877.0561	4.7551	1.7571
1	2018	15	16.3143	75.6735	877.7745	4.2531	2.5796
31	2018	16	18.0857	67.0204	847.6571	6.3469	1.1286
79	2018	16	20.7500	72.2653	973.3918	4.1959	1.6531
51	2018	16	19.1429	76.2143	942.3316	3.6653	1.4327
14	2018	16	18.0714	76.4694	900.2847	4.5561	2.8000
67	2018	16	18.2000	76.3878	905.0163	4.0755	3.3531
42	2018	16	18.5714	68.0612	877.2051	5.0133	2.8480
50	2018	16	19.0571	71.8878	904.0602	4.7051	1.8031
43	2018	16	18.5714	68.0612	877.2051	5.0133	2.8480
85	2018	16	21.8286	62.7653	912.2347	5.5633	1.8265
25	2018	16	20.5714	75.7041	980.7561	4.4714	1.2469
69	2018	16	20.4000	69.7347	942.8204	4.0031	1.4490

57	2018	16	19.6286	67.2959	888.9173	6.6643	3.2765
9	2018	16	17.7714	65.9388	856.0735	6.9735	3.0622
72	2018	16	18.0571	68.1939	879.8857	6.1837	2.1184
26	2018	16	19.4857	62.2857	868.9429	7.2235	2.5837
7	2018	16	18.4429	63.3776	861.0337	7.7255	2.3398
83	2018	16	22.2571	61.6837	947.2500	6.8398	1.4245
76	2018	16	19.3000	74.2653	923.5796	4.9010	1.4082
36	2018	16	20.3143	68.4592	931.9837	4.4867	1.8684
81	2018	16	19.1429	76.2143	942.3316	3.6653	1.4327
15	2018	16	19.3286	75.5816	920.9500	3.9316	1.6408
32	2018	16	18.5714	68.0612	877.2051	5.0133	2.8480
73	2018	16	20.9571	69.2041	965.0602	4.2612	1.1806
71	2018	16	20.3143	68.4592	931.9837	4.4867	1.8684
41	2018	16	18.9286	66.5000	876.3459	6.2653	2.0061
10	2018	16	19.8571	75.9388	967.7765	4.1020	1.1622
23	2018	16	14.6571	63.2449	776.8837	7.0173	1.8980
27	2018	16	18.4429	63.3776	861.0337	7.7255	2.3398
60	2018	16	19.1429	76.2143	942.3316	3.6653	1.4327
53	2018	16	17.7714	65.9388	856.0735	6.9735	3.0622
66	2018	16	18.0714	76.4694	900.2847	4.5561	2.8000
59	2018	16	19.6286	67.2959	888.9173	6.6643	3.2765
61	2018	16	20.9571	69.2041	965.0602	4.2612	1.1806
84	2018	16	20.9571	69.2041	965.0602	4.2612	1.1806
38	2018	16	19.6286	67.2959	888.9173	6.6643	3.2765
87	2018	16	20.0286	67.1939	902.0418	5.6653	2.0714
34	2018	16	19.6286	67.2959	888.9173	6.6643	3.2765
29	2018	16	20.4000	69.7347	942.8204	4.0031	1.4490
5	2018	16	17.6714	63.6327	833.8735	7.4296	2.2653
8	2018	16	17.7714	65.9388	856.0735	6.9735	3.0622
12	2018	16	17.6714	63.6327	833.8735	7.4296	2.2653
13	2018	16	22.2571	61.6837	947.2500	6.8398	1.4245
18	2018	16	20.6000	75.8265	967.3816	4.0245	1.0694
33	2018	16	19.0571	71.8878	904.0602	4.7051	1.8031
56	2018	16	20.5714	75.7041	980.7561	4.4714	1.2469
77	2018	16	19.3286	75.5816	920.9500	3.9316	1.6408
54	2018	16	17.6714	63.6327	833.8735	7.4296	2.2653
21	2018	16	19.0571	71.8878	904.0602	4.7051	1.8031
68	2018	16	20.7500	72.2653	973.3918	4.1959	1.6531
74	2018	16	20.9571	69.2041	965.0602	4.2612	1.1806
88	2018	16	18.5714	68.0612	877.2051	5.0133	2.8480
16	2018	16	19.3000	74.2653	923.5796	4.9010	1.4082
30	2018	16	18.0714	76.4694	900.2847	4.5561	2.8000
6	2018	16	20.7500	72.2653	973.3918	4.1959	1.6531
49	2018	16	20.4000	69.7347	942.8204	4.0031	1.4490
22	2018	16	18.5714	68.0612	877.2051	5.0133	2.8480
45	2018	16	17.3286	58.6531	818.9408	8.0704	2.5622
58	2018	16	20.4000	69.7347	942.8204	4.0031	1.4490
37	2018	16	20.7500	72.2653	973.3918	4.1959	1.6531
17	2018	16	18.2000	76.3878	905.0163	4.0755	3.3531
55	2018	16	18.0571	68.1939	879.8857	6.1837	2.1184

46	2018	16	19.3000	74.2653	923.5796	4.9010	1.4082
86	2018	16	19.0571	64.4286	868.0847	6.8286	2.3214
2	2018	16	19.0571	64.4286	868.0847	6.8286	2.3214
4	2018	16	19.0571	71.8878	904.0602	4.7051	1.8031
47	2018	16	21.9286	68.9082	961.8867	5.1510	0.6469
82	2018	16	18.5714	68.0612	877.2051	5.0133	2.8480
19	2018	16	21.2000	73.3571	964.1765	3.8051	1.2071
20	2018	16	17.7714	65.9388	856.0735	6.9735	3.0622
80	2018	16	18.5714	68.0612	877.2051	5.0133	2.8480
3	2018	16	22.2571	61.6837	947.2500	6.8398	1.4245
52	2018	16	18.2000	76.3878	905.0163	4.0755	3.3531
70	2018	16	21.8286	62.7653	912.2347	5.5633	1.8265
64	2018	16	14.6571	63.2449	776.8837	7.0173	1.8980
48	2018	16	19.3286	75.5816	920.9500	3.9316	1.6408
65	2018	16	18.2000	76.3878	905.0163	4.0755	3.3531
44	2018	16	21.8286	62.7653	912.2347	5.5633	1.8265
75	2018	16	14.6571	63.2449	776.8837	7.0173	1.8980
40	2018	16	19.8714	74.5204	948.9867	5.4673	2.0469
11	2018	16	18.0571	68.1939	879.8857	6.1837	2.1184
35	2018	16	19.1429	76.2143	942.3316	3.6653	1.4327
78	2018	16	20.0286	67.1939	902.0418	5.6653	2.0714
28	2018	16	20.3143	68.4592	931.9837	4.4867	1.8684
39	2018	16	18.2000	76.3878	905.0163	4.0755	3.3531
24	2018	16	20.4000	69.7347	942.8204	4.0031	1.4490
63	2018	16	19.8714	74.5204	948.9867	5.4673	2.0469
62	2018	16	18.9286	66.5000	876.3459	6.2653	2.0061
1	2018	16	18.5714	68.0612	877.2051	5.0133	2.8480
31	2018	17	16.0143	70.1327	847.0847	5.0806	1.1612
79	2018	17	18.6429	74.3776	972.7469	3.6541	1.5969
51	2018	17	17.3000	79.0612	941.0837	3.6551	1.5000
14	2018	17	16.9857	80.9796	899.1551	3.2857	2.4714
67	2018	17	16.5286	80.3878	903.9306	3.1745	3.1173
42	2018	17	16.4000	72.3061	876.2847	3.7980	2.7755
50	2018	17	17.0143	77.1735	903.1398	3.0571	1.5684
43	2018	17	16.4000	72.3061	876.2847	3.7980	2.7755
85	2018	17	18.5857	67.0612	911.2908	4.4969	1.7031
25	2018	17	20.6000	79.3061	979.1663	3.7531	1.1694
69	2018	17	18.6714	72.2245	941.5673	3.1296	1.5316
57	2018	17	16.7571	71.3571	887.9929	5.4347	3.0827
9	2018	17	16.2286	70.2143	855.2898	4.9939	2.7000
72	2018	17	17.2286	71.2857	878.8969	4.8908	2.2031
26	2018	17	18.5286	67.5306	868.0184	5.7449	2.1357
7	2018	17	17.4286	70.9592	860.2143	6.0092	2.2122
83	2018	17	22.2429	65.8061	945.7827	5.6531	1.3316
76	2018	17	17.8571	76.9694	922.4776	3.9592	1.3153
36	2018	17	17.8714	72.7041	930.7592	3.8092	1.9347
81	2018	17	17.3000	79.0612	941.0837	3.6551	1.5000
15	2018	17	17.5000	78.9898	919.9133	3.2796	1.5918
32	2018	17	16.4000	72.3061	876.2847	3.7980	2.7755
73	2018	17	19.7286	72.8061	963.7439	3.4786	1.0643

71	2018	17	17.8714	72.7041	930.7592	3.8092	1.9347
41	2018	17	17.0429	72.1735	875.5490	4.9959	1.8735
10	2018	17	18.3286	78.3367	966.3398	3.9571	1.2214
23	2018	17	12.5286	70.6939	776.5082	5.6082	1.9622
27	2018	17	17.4286	70.9592	860.2143	6.0092	2.2122
60	2018	17	17.3000	79.0612	941.0837	3.6551	1.5000
53	2018	17	16.2286	70.2143	855.2898	4.9939	2.7000
66	2018	17	16.9857	80.9796	899.1551	3.2857	2.4714
59	2018	17	16.7571	71.3571	887.9929	5.4347	3.0827
61	2018	17	19.7286	72.8061	963.7439	3.4786	1.0643
84	2018	17	19.7286	72.8061	963.7439	3.4786	1.0643
38	2018	17	16.7571	71.3571	887.9929	5.4347	3.0827
87	2018	17	18.3571	71.2755	900.9276	4.1480	1.9612
34	2018	17	16.7571	71.3571	887.9929	5.4347	3.0827
29	2018	17	18.6714	72.2245	941.5673	3.1296	1.5316
5	2018	17	15.5286	69.5918	833.2561	5.7796	1.9010
8	2018	17	16.2286	70.2143	855.2898	4.9939	2.7000
12	2018	17	15.5286	69.5918	833.2561	5.7796	1.9010
13	2018	17	22.2429	65.8061	945.7827	5.6531	1.3316
18	2018	17	18.6286	79.8673	966.0316	3.1133	1.1653
33	2018	17	17.0143	77.1735	903.1398	3.0571	1.5684
56	2018	17	20.6000	79.3061	979.1663	3.7531	1.1694
77	2018	17	17.5000	78.9898	919.9133	3.2796	1.5918
54	2018	17	15.5286	69.5918	833.2561	5.7796	1.9010
21	2018	17	17.0143	77.1735	903.1398	3.0571	1.5684
68	2018	17	18.6429	74.3776	972.7469	3.6541	1.5969
74	2018	17	19.7286	72.8061	963.7439	3.4786	1.0643
88	2018	17	16.4000	72.3061	876.2847	3.7980	2.7755
16	2018	17	17.8571	76.9694	922.4776	3.9592	1.3153
30	2018	17	16.9857	80.9796	899.1551	3.2857	2.4714
6	2018	17	18.6429	74.3776	972.7469	3.6541	1.5969
49	2018	17	18.6714	72.2245	941.5673	3.1296	1.5316
22	2018	17	16.4000	72.3061	876.2847	3.7980	2.7755
45	2018	17	16.1857	65.8163	818.3214	6.7704	2.0531
58	2018	17	18.6714	72.2245	941.5673	3.1296	1.5316
37	2018	17	18.6429	74.3776	972.7469	3.6541	1.5969
17	2018	17	16.5286	80.3878	903.9306	3.1745	3.1173
55	2018	17	17.2286	71.2857	878.8969	4.8908	2.2031
46	2018	17	17.8571	76.9694	922.4776	3.9592	1.3153
86	2018	17	17.0286	70.7245	867.3041	5.5204	1.9449
2	2018	17	17.0286	70.7245	867.3041	5.5204	1.9449
4	2018	17	17.0143	77.1735	903.1398	3.0571	1.5684
47	2018	17	21.6286	72.6633	960.3265	4.4315	0.6041
82	2018	17	16.4000	72.3061	876.2847	3.7980	2.7755
19	2018	17	20.8286	75.2449	962.5714	2.9449	1.1133
20	2018	17	16.2286	70.2143	855.2898	4.9939	2.7000
80	2018	17	16.4000	72.3061	876.2847	3.7980	2.7755
3	2018	17	22.2429	65.8061	945.7827	5.6531	1.3316
52	2018	17	16.5286	80.3878	903.9306	3.1745	3.1173
70	2018	17	18.5857	67.0612	911.2908	4.4969	1.7031

64	2018	17	12.5286	70.6939	776.5082	5.6082	1.9622
48	2018	17	17.5000	78.9898	919.9133	3.2796	1.5918
65	2018	17	16.5286	80.3878	903.9306	3.1745	3.1173
44	2018	17	18.5857	67.0612	911.2908	4.4969	1.7031
75	2018	17	12.5286	70.6939	776.5082	5.6082	1.9622
40	2018	17	18.1857	78.3265	947.6194	4.5316	1.9286
11	2018	17	17.2286	71.2857	878.8969	4.8908	2.2031
35	2018	17	17.3000	79.0612	941.0837	3.6551	1.5000
78	2018	17	18.3571	71.2755	900.9276	4.1480	1.9612
28	2018	17	17.8714	72.7041	930.7592	3.8092	1.9347
39	2018	17	16.5286	80.3878	903.9306	3.1745	3.1173
24	2018	17	18.6714	72.2245	941.5673	3.1296	1.5316
63	2018	17	18.1857	78.3265	947.6194	4.5316	1.9286
62	2018	17	17.0429	72.1735	875.5490	4.9959	1.8735
1	2018	17	16.4000	72.3061	876.2847	3.7980	2.7755
31	2018	18	18.0857	75.8469	847.8235	3.4663	1.1745
79	2018	18	22.5571	79.0714	972.2847	2.9286	1.4327
51	2018	18	21.9571	83.4184	940.9469	2.6939	1.4980
14	2018	18	20.6000	87.6633	899.3469	1.7847	2.2908
67	2018	18	20.3857	86.5102	904.0949	2.0500	3.0071
42	2018	18	19.1714	81.1939	876.6827	2.3408	2.6306
50	2018	18	19.8714	83.2449	903.4735	1.7286	1.3612
43	2018	18	19.1714	81.1939	876.6827	2.3408	2.6306
85	2018	18	21.3000	74.3163	911.6459	3.9439	1.5643
25	2018	18	23.9571	83.7449	978.7827	3.1918	0.9918
69	2018	18	22.2143	78.8571	941.5327	2.2439	1.4531
57	2018	18	19.7857	78.1122	888.3827	4.0214	2.8163
9	2018	18	18.5571	78.4388	855.8418	3.4459	2.3500
72	2018	18	19.7000	78.2449	879.3031	3.5531	1.9082
26	2018	18	21.1143	76.2959	868.5745	4.5571	1.8020
7	2018	18	20.3571	80.7347	860.8082	4.5582	1.9531
83	2018	18	24.5143	73.5102	945.9776	4.7857	1.0990
76	2018	18	20.5286	81.1122	922.6316	3.0296	1.2684
36	2018	18	21.8857	80.2449	930.7806	2.6122	1.8020
81	2018	18	21.9571	83.4184	940.9469	2.6939	1.4980
15	2018	18	18.6429	82.9898	920.1827	2.8276	1.5663
32	2018	18	19.1714	81.1939	876.6827	2.3408	2.6306
73	2018	18	22.4143	76.8673	963.6163	2.9724	0.9673
71	2018	18	21.8857	80.2449	930.7806	2.6122	1.8020
41	2018	18	19.9857	79.3571	876.0990	3.4459	1.5459
10	2018	18	22.7571	83.1020	966.0010	2.9378	1.0673
23	2018	18	14.7286	79.3061	777.4173	3.7765	1.9143
27	2018	18	20.3571	80.7347	860.8082	4.5582	1.9531
60	2018	18	21.9571	83.4184	940.9469	2.6939	1.4980
53	2018	18	18.5571	78.4388	855.8418	3.4459	2.3500
66	2018	18	20.6000	87.6633	899.3469	1.7847	2.2908
59	2018	18	19.7857	78.1122	888.3827	4.0214	2.8163
61	2018	18	22.4143	76.8673	963.6163	2.9724	0.9673
84	2018	18	22.4143	76.8673	963.6163	2.9724	0.9673
38	2018	18	19.7857	78.1122	888.3827	4.0214	2.8163

87	2018	18	20.9429	80.3469	901.2102	2.4776	1.7061
34	2018	18	19.7857	78.1122	888.3827	4.0214	2.8163
29	2018	18	22.2143	78.8571	941.5327	2.2439	1.4531
5	2018	18	18.6857	78.5204	833.9541	4.7949	1.7673
8	2018	18	18.5571	78.4388	855.8418	3.4459	2.3500
12	2018	18	18.6857	78.5204	833.9541	4.7949	1.7673
13	2018	18	24.5143	73.5102	945.9776	4.7857	1.0990
18	2018	18	21.7857	84.9286	965.7408	2.4878	1.1418
33	2018	18	19.8714	83.2449	903.4735	1.7286	1.3612
56	2018	18	23.9571	83.7449	978.7827	3.1918	0.9918
77	2018	18	18.6429	82.9898	920.1827	2.8276	1.5663
54	2018	18	18.6857	78.5204	833.9541	4.7949	1.7673
21	2018	18	19.8714	83.2449	903.4735	1.7286	1.3612
68	2018	18	22.5571	79.0714	972.2847	2.9286	1.4327
74	2018	18	22.4143	76.8673	963.6163	2.9724	0.9673
88	2018	18	19.1714	81.1939	876.6827	2.3408	2.6306
16	2018	18	20.5286	81.1122	922.6316	3.0296	1.2684
30	2018	18	20.6000	87.6633	899.3469	1.7847	2.2908
6	2018	18	22.5571	79.0714	972.2847	2.9286	1.4327
49	2018	18	22.2143	78.8571	941.5327	2.2439	1.4531
22	2018	18	19.1714	81.1939	876.6827	2.3408	2.6306
45	2018	18	18.4429	72.1531	819.0306	5.6582	1.7071
58	2018	18	22.2143	78.8571	941.5327	2.2439	1.4531
37	2018	18	22.5571	79.0714	972.2847	2.9286	1.4327
17	2018	18	20.3857	86.5102	904.0949	2.0500	3.0071
55	2018	18	19.7000	78.2449	879.3031	3.5531	1.9082
46	2018	18	20.5286	81.1122	922.6316	3.0296	1.2684
86	2018	18	19.1714	81.5816	867.8378	3.4612	1.6327
2	2018	18	19.1714	81.5816	867.8378	3.4612	1.6327
4	2018	18	19.8714	83.2449	903.4735	1.7286	1.3612
47	2018	18	23.8000	81.1327	960.3480	3.1374	0.4694
82	2018	18	19.1714	81.1939	876.6827	2.3408	2.6306
19	2018	18	24.2143	81.1224	962.4010	1.9439	1.0602
20	2018	18	18.5571	78.4388	855.8418	3.4459	2.3500
80	2018	18	19.1714	81.1939	876.6827	2.3408	2.6306
3	2018	18	24.5143	73.5102	945.9776	4.7857	1.0990
52	2018	18	20.3857	86.5102	904.0949	2.0500	3.0071
70	2018	18	21.3000	74.3163	911.6459	3.9439	1.5643
64	2018	18	14.7286	79.3061	777.4173	3.7765	1.9143
48	2018	18	18.6429	82.9898	920.1827	2.8276	1.5663
65	2018	18	20.3857	86.5102	904.0949	2.0500	3.0071
44	2018	18	21.3000	74.3163	911.6459	3.9439	1.5643
75	2018	18	14.7286	79.3061	777.4173	3.7765	1.9143
40	2018	18	22.5857	82.7653	947.3980	3.1153	1.8031
11	2018	18	19.7000	78.2449	879.3031	3.5531	1.9082
35	2018	18	21.9571	83.4184	940.9469	2.6939	1.4980
78	2018	18	20.9429	80.3469	901.2102	2.4776	1.7061
28	2018	18	21.8857	80.2449	930.7806	2.6122	1.8020
39	2018	18	20.3857	86.5102	904.0949	2.0500	3.0071
24	2018	18	22.2143	78.8571	941.5327	2.2439	1.4531

63	2018	18	22.5857	82.7653	947.3980	3.1153	1.8031
62	2018	18	19.9857	79.3571	876.0990	3.4459	1.5459
1	2018	18	19.1714	81.1939	876.6827	2.3408	2.6306
31	2018	19	16.8143	76.6020	847.8939	3.2418	1.3316
79	2018	19	20.9429	79.5714	971.3255	3.1204	1.5898
51	2018	19	19.5571	83.3673	940.7459	2.1561	1.6541
14	2018	19	18.3857	89.3980	899.3235	1.5735	2.2173
67	2018	19	17.8857	88.1735	904.0857	1.7429	2.9786
42	2018	19	17.2286	85.2551	876.7816	1.9500	2.7133
50	2018	19	18.4286	81.6837	903.5449	2.2765	1.5184
43	2018	19	17.2286	85.2551	876.7816	1.9500	2.7133
85	2018	19	19.8714	73.5816	911.7388	4.4031	1.7582
25	2018	19	21.9286	86.2449	978.4990	2.4541	0.8122
69	2018	19	20.3857	78.8469	941.4602	3.2398	1.5276
57	2018	19	17.8143	79.9286	888.5082	3.6000	2.6847
9	2018	19	16.6571	81.9184	855.9918	3.5102	2.5194
72	2018	19	17.7857	81.8673	879.4224	2.8714	1.8878
26	2018	19	20.1857	78.8163	868.6816	4.6571	1.9541
7	2018	19	19.0714	82.1224	860.9153	4.3184	2.1837
83	2018	19	23.2000	76.7755	946.0000	3.7867	1.0041
76	2018	19	19.2000	79.6633	922.6878	3.5745	1.3663
36	2018	19	19.2714	82.6837	930.6980	1.9398	1.6969
81	2018	19	19.5571	83.3673	940.7459	2.1561	1.6541
15	2018	19	18.5429	83.6429	920.3469	3.1071	1.5480
32	2018	19	17.2286	85.2551	876.7816	1.9500	2.7133
73	2018	19	21.2571	74.4694	963.5622	3.3939	0.9918
71	2018	19	19.2714	82.6837	930.6980	1.9398	1.6969
41	2018	19	17.5571	80.3673	876.2429	2.6653	1.5745
10	2018	19	20.6143	85.0918	965.7694	2.5918	1.0490
23	2018	19	14.8714	79.9184	777.5143	4.5724	1.9765
27	2018	19	19.0714	82.1224	860.9153	4.3184	2.1837
60	2018	19	19.5571	83.3673	940.7459	2.1561	1.6541
53	2018	19	16.6571	81.9184	855.9918	3.5102	2.5194
66	2018	19	18.3857	89.3980	899.3235	1.5735	2.2173
59	2018	19	17.8143	79.9286	888.5082	3.6000	2.6847
61	2018	19	21.2571	74.4694	963.5622	3.3939	0.9918
84	2018	19	21.2571	74.4694	963.5622	3.3939	0.9918
38	2018	19	17.8143	79.9286	888.5082	3.6000	2.6847
87	2018	19	19.1714	85.1429	901.3071	2.3337	1.8184
34	2018	19	17.8143	79.9286	888.5082	3.6000	2.6847
29	2018	19	20.3857	78.8469	941.4602	3.2398	1.5276
5	2018	19	17.2143	80.9082	834.0918	4.7939	1.9827
8	2018	19	16.6571	81.9184	855.9918	3.5102	2.5194
12	2018	19	17.2143	80.9082	834.0918	4.7939	1.9827
13	2018	19	23.2000	76.7755	946.0000	3.7867	1.0041
18	2018	19	20.7286	84.4388	965.6500	3.2378	1.1010
33	2018	19	18.4286	81.6837	903.5449	2.2765	1.5184
56	2018	19	21.9286	86.2449	978.4990	2.4541	0.8122
77	2018	19	18.5429	83.6429	920.3469	3.1071	1.5480
54	2018	19	17.2143	80.9082	834.0918	4.7939	1.9827

21	2018	19	18.4286	81.6837	903.5449	2.2765	1.5184
68	2018	19	20.9429	79.5714	971.3255	3.1204	1.5898
74	2018	19	21.2571	74.4694	963.5622	3.3939	0.9918
88	2018	19	17.2286	85.2551	876.7816	1.9500	2.7133
16	2018	19	19.2000	79.6633	922.6878	3.5745	1.3663
30	2018	19	18.3857	89.3980	899.3235	1.5735	2.2173
6	2018	19	20.9429	79.5714	971.3255	3.1204	1.5898
49	2018	19	20.3857	78.8469	941.4602	3.2398	1.5276
22	2018	19	17.2286	85.2551	876.7816	1.9500	2.7133
45	2018	19	18.5286	75.1224	819.1857	5.6092	1.6980
58	2018	19	20.3857	78.8469	941.4602	3.2398	1.5276
37	2018	19	20.9429	79.5714	971.3255	3.1204	1.5898
17	2018	19	17.8857	88.1735	904.0857	1.7429	2.9786
55	2018	19	17.7857	81.8673	879.4224	2.8714	1.8878
46	2018	19	19.2000	79.6633	922.6878	3.5745	1.3663
86	2018	19	17.3429	84.1531	867.8980	2.5816	1.5510
2	2018	19	17.3429	84.1531	867.8980	2.5816	1.5510
4	2018	19	18.4286	81.6837	903.5449	2.2765	1.5184
47	2018	19	22.2571	85.6735	960.3480	2.1090	0.3714
82	2018	19	17.2286	85.2551	876.7816	1.9500	2.7133
19	2018	19	21.7571	84.7041	962.2663	1.7286	0.9520
20	2018	19	16.6571	81.9184	855.9918	3.5102	2.5194
80	2018	19	17.2286	85.2551	876.7816	1.9500	2.7133
3	2018	19	23.2000	76.7755	946.0000	3.7867	1.0041
52	2018	19	17.8857	88.1735	904.0857	1.7429	2.9786
70	2018	19	19.8714	73.5816	911.7388	4.4031	1.7582
64	2018	19	14.8714	79.9184	777.5143	4.5724	1.9765
48	2018	19	18.5429	83.6429	920.3469	3.1071	1.5480
65	2018	19	17.8857	88.1735	904.0857	1.7429	2.9786
44	2018	19	19.8714	73.5816	911.7388	4.4031	1.7582
75	2018	19	14.8714	79.9184	777.5143	4.5724	1.9765
40	2018	19	20.0143	85.5510	947.2255	2.1245	1.8010
11	2018	19	17.7857	81.8673	879.4224	2.8714	1.8878
35	2018	19	19.5571	83.3673	940.7459	2.1561	1.6541
78	2018	19	19.1714	85.1429	901.3071	2.3337	1.8184
28	2018	19	19.2714	82.6837	930.6980	1.9398	1.6969
39	2018	19	17.8857	88.1735	904.0857	1.7429	2.9786
24	2018	19	20.3857	78.8469	941.4602	3.2398	1.5276
63	2018	19	20.0143	85.5510	947.2255	2.1245	1.8010
62	2018	19	17.5571	80.3673	876.2429	2.6653	1.5745
1	2018	19	17.2286	85.2551	876.7816	1.9500	2.7133
31	2018	20	22.0714	73.3367	845.8316	5.2061	1.3286
79	2018	20	27.4143	77.5918	968.1827	4.5898	1.6051
51	2018	20	26.8286	79.5408	937.9510	4.1418	1.7582
14	2018	20	24.5857	86.8469	897.5602	3.2551	2.5510
67	2018	20	24.9857	83.8163	902.1847	3.3980	3.3878
42	2018	20	23.7000	81.5306	874.9031	3.8224	2.9643
50	2018	20	24.9000	75.5000	901.2735	4.2378	1.7459
43	2018	20	23.7000	81.5306	874.9031	3.8224	2.9643
85	2018	20	26.6000	69.4592	909.3745	5.7388	2.0888

25	2018	20	28.1286	84.8571	975.6949	3.6480	1.0173
69	2018	20	27.1571	74.3571	938.5296	5.4112	1.8653
57	2018	20	24.4143	76.0102	886.5878	5.3286	3.0337
9	2018	20	22.5714	80.0510	854.2480	5.1459	2.8367
72	2018	20	23.7143	79.8571	877.6469	4.8245	2.2663
26	2018	20	24.5286	75.2755	866.9367	6.5745	2.2041
7	2018	20	24.0286	78.2041	859.1612	6.2480	2.6296
83	2018	20	28.2714	74.3673	943.3949	5.3755	1.0755
76	2018	20	25.5571	75.6327	919.8122	5.0255	1.5949
36	2018	20	27.4857	77.9592	927.9918	3.9980	1.9163
81	2018	20	26.8286	79.5408	937.9510	4.1418	1.7582
15	2018	20	24.7857	77.9286	917.3235	4.3908	1.6796
32	2018	20	23.7000	81.5306	874.9031	3.8224	2.9643
73	2018	20	27.8000	71.1327	960.3092	4.4306	1.1806
71	2018	20	27.4857	77.9592	927.9918	3.9980	1.9163
41	2018	20	23.6857	79.1531	874.2153	3.5806	1.7276
10	2018	20	27.5143	81.7449	962.8592	4.0929	1.1633
23	2018	20	18.9571	72.8980	775.9357	6.7418	2.1633
27	2018	20	24.0286	78.2041	859.1612	6.2480	2.6296
60	2018	20	26.8286	79.5408	937.9510	4.1418	1.7582
53	2018	20	22.5714	80.0510	854.2480	5.1459	2.8367
66	2018	20	24.5857	86.8469	897.5602	3.2551	2.5510
59	2018	20	24.4143	76.0102	886.5878	5.3286	3.0337
61	2018	20	27.8000	71.1327	960.3092	4.4306	1.1806
84	2018	20	27.8000	71.1327	960.3092	4.4306	1.1806
38	2018	20	24.4143	76.0102	886.5878	5.3286	3.0337
87	2018	20	25.5571	80.9286	899.3765	5.1776	1.9827
34	2018	20	24.4143	76.0102	886.5878	5.3286	3.0337
29	2018	20	27.1571	74.3571	938.5296	5.4112	1.8653
5	2018	20	22.9000	74.8469	832.3888	6.2439	2.2510
8	2018	20	22.5714	80.0510	854.2480	5.1459	2.8367
12	2018	20	22.9000	74.8469	832.3888	6.2439	2.2510
13	2018	20	28.2714	74.3673	943.3949	5.3755	1.0755
18	2018	20	26.8000	80.8571	962.4837	4.3959	1.2653
33	2018	20	24.9000	75.5000	901.2735	4.2378	1.7459
56	2018	20	28.1286	84.8571	975.6949	3.6480	1.0173
77	2018	20	24.7857	77.9286	917.3235	4.3908	1.6796
54	2018	20	22.9000	74.8469	832.3888	6.2439	2.2510
21	2018	20	24.9000	75.5000	901.2735	4.2378	1.7459
68	2018	20	27.4143	77.5918	968.1827	4.5898	1.6051
74	2018	20	27.8000	71.1327	960.3092	4.4306	1.1806
88	2018	20	23.7000	81.5306	874.9031	3.8224	2.9643
16	2018	20	25.5571	75.6327	919.8122	5.0255	1.5949
30	2018	20	24.5857	86.8469	897.5602	3.2551	2.5510
6	2018	20	27.4143	77.5918	968.1827	4.5898	1.6051
49	2018	20	27.1571	74.3571	938.5296	5.4112	1.8653
22	2018	20	23.7000	81.5306	874.9031	3.8224	2.9643
45	2018	20	22.2571	69.9694	817.5806	7.4500	2.0714
58	2018	20	27.1571	74.3571	938.5296	5.4112	1.8653
37	2018	20	27.4143	77.5918	968.1827	4.5898	1.6051

17	2018	20	24.9857	83.8163	902.1847	3.3980	3.3878
55	2018	20	23.7143	79.8571	877.6469	4.8245	2.2663
46	2018	20	25.5571	75.6327	919.8122	5.0255	1.5949
86	2018	20	23.6429	78.5816	865.8776	4.8102	1.9888
2	2018	20	23.6429	78.5816	865.8776	4.8102	1.9888
4	2018	20	24.9000	75.5000	901.2735	4.2378	1.7459
47	2018	20	28.1429	83.4490	957.6316	4.0684	0.4959
82	2018	20	23.7000	81.5306	874.9031	3.8224	2.9643
19	2018	20	28.2286	83.5306	959.6949	2.3939	0.9612
20	2018	20	22.5714	80.0510	854.2480	5.1459	2.8367
80	2018	20	23.7000	81.5306	874.9031	3.8224	2.9643
3	2018	20	28.2714	74.3673	943.3949	5.3755	1.0755
52	2018	20	24.9857	83.8163	902.1847	3.3980	3.3878
70	2018	20	26.6000	69.4592	909.3745	5.7388	2.0888
64	2018	20	18.9571	72.8980	775.9357	6.7418	2.1633
48	2018	20	24.7857	77.9286	917.3235	4.3908	1.6796
65	2018	20	24.9857	83.8163	902.1847	3.3980	3.3878
44	2018	20	26.6000	69.4592	909.3745	5.7388	2.0888
75	2018	20	18.9571	72.8980	775.9357	6.7418	2.1633
40	2018	20	27.1571	84.0102	944.6888	3.9531	2.0918
11	2018	20	23.7143	79.8571	877.6469	4.8245	2.2663
35	2018	20	26.8286	79.5408	937.9510	4.1418	1.7582
78	2018	20	25.5571	80.9286	899.3765	5.1776	1.9827
28	2018	20	27.4857	77.9592	927.9918	3.9980	1.9163
39	2018	20	24.9857	83.8163	902.1847	3.3980	3.3878
24	2018	20	27.1571	74.3571	938.5296	5.4112	1.8653
63	2018	20	27.1571	84.0102	944.6888	3.9531	2.0918
62	2018	20	23.6857	79.1531	874.2153	3.5806	1.7276
1	2018	20	23.7000	81.5306	874.9031	3.8224	2.9643
31	2018	21	19.6571	70.0918	844.8102	6.7255	1.3429
79	2018	21	23.4857	76.8878	965.9337	5.9357	1.5929
51	2018	21	23.4857	74.8163	935.9673	6.2714	1.7571
14	2018	21	22.4429	80.9082	896.6367	5.0520	3.2806
67	2018	21	22.2429	77.5204	901.0622	5.2429	3.9184
42	2018	21	20.7000	75.4898	873.9245	5.4980	3.1510
50	2018	21	20.8429	73.9082	899.9092	4.5786	2.0082
43	2018	21	20.7000	75.4898	873.9245	5.4980	3.1510
85	2018	21	22.6571	68.5408	907.9612	6.2735	2.3633
25	2018	21	26.3286	77.3571	973.5357	7.1306	1.4041
69	2018	21	24.1000	72.2551	936.4245	6.6612	1.9949
57	2018	21	21.1857	71.9388	885.5429	7.0980	3.5347
9	2018	21	20.2143	75.3878	853.4082	7.1918	3.0418
72	2018	21	21.2857	74.3776	876.8184	6.7694	2.4918
26	2018	21	22.2571	72.2551	866.2612	8.6173	2.4000
7	2018	21	21.7714	73.3571	858.4663	8.2429	2.8765
83	2018	21	25.8429	68.7959	941.7214	8.4071	1.2031
76	2018	21	22.2429	75.4592	917.7929	4.9939	1.6408
36	2018	21	24.3429	70.2959	926.0439	6.8204	2.2969
81	2018	21	23.4857	74.8163	935.9673	6.2714	1.7571
15	2018	21	20.8286	77.2653	915.2092	4.6408	1.8214

32	2018	21	20.7000	75.4898	873.9245	5.4980	3.1510
73	2018	21	24.2143	71.6633	957.9112	5.1265	1.1633
71	2018	21	24.3429	70.2959	926.0439	6.8204	2.2969
41	2018	21	21.6571	74.0816	873.0531	4.9592	2.0082
10	2018	21	24.5714	77.0510	960.6867	6.1796	1.2367
23	2018	21	16.4714	67.9796	775.4673	7.6490	2.4520
27	2018	21	21.7714	73.3571	858.4663	8.2429	2.8765
60	2018	21	23.4857	74.8163	935.9673	6.2714	1.7571
53	2018	21	20.2143	75.3878	853.4082	7.1918	3.0418
66	2018	21	22.4429	80.9082	896.6367	5.0520	3.2806
59	2018	21	21.1857	71.9388	885.5429	7.0980	3.5347
61	2018	21	24.2143	71.6633	957.9112	5.1265	1.1633
84	2018	21	24.2143	71.6633	957.9112	5.1265	1.1633
38	2018	21	21.1857	71.9388	885.5429	7.0980	3.5347
87	2018	21	23.2000	73.0918	898.2816	7.2459	2.1031
34	2018	21	21.1857	71.9388	885.5429	7.0980	3.5347
29	2018	21	24.1000	72.2551	936.4245	6.6612	1.9949
5	2018	21	20.3571	69.3776	831.7092	8.2031	2.2286
8	2018	21	20.2143	75.3878	853.4082	7.1918	3.0418
12	2018	21	20.3571	69.3776	831.7092	8.2031	2.2286
13	2018	21	25.8429	68.7959	941.7214	8.4071	1.2031
18	2018	21	22.7143	80.3673	960.2969	4.5245	1.3561
33	2018	21	20.8429	73.9082	899.9092	4.5786	2.0082
56	2018	21	26.3286	77.3571	973.5357	7.1306	1.4041
77	2018	21	20.8286	77.2653	915.2092	4.6408	1.8214
54	2018	21	20.3571	69.3776	831.7092	8.2031	2.2286
21	2018	21	20.8429	73.9082	899.9092	4.5786	2.0082
68	2018	21	23.4857	76.8878	965.9337	5.9357	1.5929
74	2018	21	24.2143	71.6633	957.9112	5.1265	1.1633
88	2018	21	20.7000	75.4898	873.9245	5.4980	3.1510
16	2018	21	22.2429	75.4592	917.7929	4.9939	1.6408
30	2018	21	22.4429	80.9082	896.6367	5.0520	3.2806
6	2018	21	23.4857	76.8878	965.9337	5.9357	1.5929
49	2018	21	24.1000	72.2551	936.4245	6.6612	1.9949
22	2018	21	20.7000	75.4898	873.9245	5.4980	3.1510
45	2018	21	20.3286	64.4286	817.0122	8.6735	2.6490
58	2018	21	24.1000	72.2551	936.4245	6.6612	1.9949
37	2018	21	23.4857	76.8878	965.9337	5.9357	1.5929
17	2018	21	22.2429	77.5204	901.0622	5.2429	3.9184
55	2018	21	21.2857	74.3776	876.8184	6.7694	2.4918
46	2018	21	22.2429	75.4592	917.7929	4.9939	1.6408
86	2018	21	21.1286	72.9490	864.8296	6.7082	2.4888
2	2018	21	21.1286	72.9490	864.8296	6.7082	2.4888
4	2018	21	20.8429	73.9082	899.9092	4.5786	2.0082
47	2018	21	26.0286	76.6429	955.6429	6.2959	0.6031
82	2018	21	20.7000	75.4898	873.9245	5.4980	3.1510
19	2018	21	26.5286	76.0918	957.7867	3.7204	1.3286
20	2018	21	20.2143	75.3878	853.4082	7.1918	3.0418
80	2018	21	20.7000	75.4898	873.9245	5.4980	3.1510
3	2018	21	25.8429	68.7959	941.7214	8.4071	1.2031

52	2018	21	22.2429	77.5204	901.0622	5.2429	3.9184
70	2018	21	22.6571	68.5408	907.9612	6.2735	2.3633
64	2018	21	16.4714	67.9796	775.4673	7.6490	2.4520
48	2018	21	20.8286	77.2653	915.2092	4.6408	1.8214
65	2018	21	22.2429	77.5204	901.0622	5.2429	3.9184
44	2018	21	22.6571	68.5408	907.9612	6.2735	2.3633
75	2018	21	16.4714	67.9796	775.4673	7.6490	2.4520
40	2018	21	24.3571	76.8367	942.8673	7.8041	2.3439
11	2018	21	21.2857	74.3776	876.8184	6.7694	2.4918
35	2018	21	23.4857	74.8163	935.9673	6.2714	1.7571
78	2018	21	23.2000	73.0918	898.2816	7.2459	2.1031
28	2018	21	24.3429	70.2959	926.0439	6.8204	2.2969
39	2018	21	22.2429	77.5204	901.0622	5.2429	3.9184
24	2018	21	24.1000	72.2551	936.4245	6.6612	1.9949
63	2018	21	24.3571	76.8367	942.8673	7.8041	2.3439
62	2018	21	21.6571	74.0816	873.0531	4.9592	2.0082
1	2018	21	20.7000	75.4898	873.9245	5.4980	3.1510
31	2018	22	16.8857	70.6224	846.3296	4.2684	1.3357
79	2018	22	21.3857	80.9592	967.8418	3.4714	1.5092
51	2018	22	20.2857	78.6224	937.5888	4.0000	1.7490
14	2018	22	19.5000	82.6837	897.3051	3.3724	2.9031
67	2018	22	19.0143	79.9286	901.9163	3.5398	3.6663
42	2018	22	18.2286	76.8673	874.9949	3.3796	2.8898
50	2018	22	18.4857	79.8469	901.5092	1.9776	1.8459
43	2018	22	18.2286	76.8673	874.9949	3.3796	2.8898
85	2018	22	20.7143	71.6224	909.7459	3.5612	1.9367
25	2018	22	23.1143	79.6939	974.8888	5.3388	1.1878
69	2018	22	21.0286	75.6122	938.2633	4.1020	1.7204
57	2018	22	18.0143	75.7041	886.7337	4.6031	2.9704
9	2018	22	17.4714	75.6531	854.4112	5.1806	2.8786
72	2018	22	18.3714	76.0510	877.7480	4.3510	2.2949
26	2018	22	19.6857	77.0306	867.1388	6.0827	2.0735
7	2018	22	18.9429	77.7449	859.4224	5.3612	2.5286
83	2018	22	23.6714	70.1735	943.1765	6.7878	1.2867
76	2018	22	19.8429	79.2653	919.7347	2.8510	1.4857
36	2018	22	20.5429	73.6531	927.6092	4.9031	2.0827
81	2018	22	20.2857	78.6224	937.5888	4.0000	1.7490
15	2018	22	18.8143	84.2959	917.3929	2.4408	1.6092
32	2018	22	18.2286	76.8673	874.9949	3.3796	2.8898
73	2018	22	21.7143	78.2449	959.9827	2.7367	0.8786
71	2018	22	20.5429	73.6531	927.6092	4.9031	2.0827
41	2018	22	18.1571	74.3163	874.3980	3.4867	1.8745
10	2018	22	21.3571	79.9184	962.2786	4.3520	1.2490
23	2018	22	13.5429	74.8878	776.6224	4.8520	2.3653
27	2018	22	18.9429	77.7449	859.4224	5.3612	2.5286
60	2018	22	20.2857	78.6224	937.5888	4.0000	1.7490
53	2018	22	17.4714	75.6531	854.4112	5.1806	2.8786
66	2018	22	19.5000	82.6837	897.3051	3.3724	2.9031
59	2018	22	18.0143	75.7041	886.7337	4.6031	2.9704
61	2018	22	21.7143	78.2449	959.9827	2.7367	0.8786

84	2018	22	21.7143	78.2449	959.9827	2.7367	0.8786
38	2018	22	18.0143	75.7041	886.7337	4.6031	2.9704
87	2018	22	20.0857	74.4388	899.2837	4.7724	2.1459
34	2018	22	18.0143	75.7041	886.7337	4.6031	2.9704
29	2018	22	21.0286	75.6122	938.2633	4.1020	1.7204
5	2018	22	16.7286	76.3776	832.7337	5.3969	1.8857
8	2018	22	17.4714	75.6531	854.4112	5.1806	2.8786
12	2018	22	16.7286	76.3776	832.7337	5.3969	1.8857
13	2018	22	23.6714	70.1735	943.1765	6.7878	1.2867
18	2018	22	21.3143	85.5612	962.2878	2.1673	1.1612
33	2018	22	18.4857	79.8469	901.5092	1.9776	1.8459
56	2018	22	23.1143	79.6939	974.8888	5.3388	1.1878
77	2018	22	18.8143	84.2959	917.3929	2.4408	1.6092
54	2018	22	16.7286	76.3776	832.7337	5.3969	1.8857
21	2018	22	18.4857	79.8469	901.5092	1.9776	1.8459
68	2018	22	21.3857	80.9592	967.8418	3.4714	1.5092
74	2018	22	21.7143	78.2449	959.9827	2.7367	0.8786
88	2018	22	18.2286	76.8673	874.9949	3.3796	2.8898
16	2018	22	19.8429	79.2653	919.7347	2.8510	1.4857
30	2018	22	19.5000	82.6837	897.3051	3.3724	2.9031
6	2018	22	21.3857	80.9592	967.8418	3.4714	1.5092
49	2018	22	21.0286	75.6122	938.2633	4.1020	1.7204
22	2018	22	18.2286	76.8673	874.9949	3.3796	2.8898
45	2018	22	17.0571	72.6837	817.9510	5.4602	2.3408
58	2018	22	21.0286	75.6122	938.2633	4.1020	1.7204
37	2018	22	21.3857	80.9592	967.8418	3.4714	1.5092
17	2018	22	19.0143	79.9286	901.9163	3.5398	3.6663
55	2018	22	18.3714	76.0510	877.7480	4.3510	2.2949
46	2018	22	19.8429	79.2653	919.7347	2.8510	1.4857
86	2018	22	17.7571	75.7347	866.0969	3.7776	2.0776
2	2018	22	17.7571	75.7347	866.0969	3.7776	2.0776
4	2018	22	18.4857	79.8469	901.5092	1.9776	1.8459
47	2018	22	23.9143	77.5918	957.0500	4.7122	0.5663
82	2018	22	18.2286	76.8673	874.9949	3.3796	2.8898
19	2018	22	23.6000	76.1224	958.9469	2.7531	1.3704
20	2018	22	17.4714	75.6531	854.4112	5.1806	2.8786
80	2018	22	18.2286	76.8673	874.9949	3.3796	2.8898
3	2018	22	23.6714	70.1735	943.1765	6.7878	1.2867
52	2018	22	19.0143	79.9286	901.9163	3.5398	3.6663
70	2018	22	20.7143	71.6224	909.7459	3.5612	1.9367
64	2018	22	13.5429	74.8878	776.6224	4.8520	2.3653
48	2018	22	18.8143	84.2959	917.3929	2.4408	1.6092
65	2018	22	19.0143	79.9286	901.9163	3.5398	3.6663
44	2018	22	20.7143	71.6224	909.7459	3.5612	1.9367
75	2018	22	13.5429	74.8878	776.6224	4.8520	2.3653
40	2018	22	20.7429	79.8776	944.1439	5.8357	2.1102
11	2018	22	18.3714	76.0510	877.7480	4.3510	2.2949
35	2018	22	20.2857	78.6224	937.5888	4.0000	1.7490
78	2018	22	20.0857	74.4388	899.2837	4.7724	2.1459
28	2018	22	20.5429	73.6531	927.6092	4.9031	2.0827

39	2018	22	19.0143	79.9286	901.9163	3.5398	3.6663
24	2018	22	21.0286	75.6122	938.2633	4.1020	1.7204
63	2018	22	20.7429	79.8776	944.1439	5.8357	2.1102
62	2018	22	18.1571	74.3163	874.3980	3.4867	1.8745
1	2018	22	18.2286	76.8673	874.9949	3.3796	2.8898
31	2018	23	18.9571	74.4082	848.2255	2.9561	1.1204
79	2018	23	24.6286	83.3469	969.8490	2.3816	1.0235
51	2018	23	22.4143	84.2041	939.5633	2.4265	1.2888
14	2018	23	21.9143	84.2959	898.1020	2.7612	1.7622
67	2018	23	20.9000	83.8265	903.1031	2.9051	2.8020
42	2018	23	20.5429	79.9490	876.3143	2.9653	2.4398
50	2018	23	21.6857	81.9184	903.4112	2.2806	1.2878
43	2018	23	20.5429	79.9490	876.3143	2.9653	2.4398
85	2018	23	24.2286	75.2959	911.7959	2.8520	1.2316
25	2018	23	24.8286	86.6531	976.6878	2.7622	0.7531
69	2018	23	23.1571	79.5816	940.5316	2.8418	1.3541
57	2018	23	20.1286	81.1327	888.2847	3.3122	2.0327
9	2018	23	19.5857	77.7245	855.6929	3.3031	2.1480
72	2018	23	20.4571	79.2041	878.8071	2.8816	1.6908
26	2018	23	21.1714	80.7143	868.2296	3.5224	1.3010
7	2018	23	20.2571	83.4490	860.5704	3.2337	1.6357
83	2018	23	25.0000	75.6122	944.9010	4.3949	1.2449
76	2018	23	22.7143	80.7143	922.0153	2.6408	1.2296
36	2018	23	22.4571	80.4184	929.6143	3.4541	1.4827
81	2018	23	22.4143	84.2041	939.5633	2.4265	1.2888
15	2018	23	22.0714	84.3878	919.8031	2.7602	1.3235
32	2018	23	20.5429	79.9490	876.3143	2.9653	2.4398
73	2018	23	24.9286	81.0510	962.3714	2.2163	0.8102
71	2018	23	22.4571	80.4184	929.6143	3.4541	1.4827
41	2018	23	20.2286	80.1429	876.1847	3.0357	1.2224
10	2018	23	23.6000	84.6633	964.2235	2.4949	0.9847
23	2018	23	15.2857	81.3776	778.0673	3.0031	1.9418
27	2018	23	20.2571	83.4490	860.5704	3.2337	1.6357
60	2018	23	22.4143	84.2041	939.5633	2.4265	1.2888
53	2018	23	19.5857	77.7245	855.6929	3.3031	2.1480
66	2018	23	21.9143	84.2959	898.1020	2.7612	1.7622
59	2018	23	20.1286	81.1327	888.2847	3.3122	2.0327
61	2018	23	24.9286	81.0510	962.3714	2.2163	0.8102
84	2018	23	24.9286	81.0510	962.3714	2.2163	0.8102
38	2018	23	20.1286	81.1327	888.2847	3.3122	2.0327
87	2018	23	21.8143	80.3571	900.5520	3.4592	1.8806
34	2018	23	20.1286	81.1327	888.2847	3.3122	2.0327
29	2018	23	23.1571	79.5816	940.5316	2.8418	1.3541
5	2018	23	18.5857	82.6939	834.0153	2.9816	1.3582
8	2018	23	19.5857	77.7245	855.6929	3.3031	2.1480
12	2018	23	18.5857	82.6939	834.0153	2.9816	1.3582
13	2018	23	25.0000	75.6122	944.9010	4.3949	1.2449
18	2018	23	24.8714	86.6224	964.1908	2.2765	0.9143
33	2018	23	21.6857	81.9184	903.4112	2.2806	1.2878
56	2018	23	24.8286	86.6531	976.6878	2.7622	0.7531

77	2018	23	22.0714	84.3878	919.8031	2.7602	1.3235
54	2018	23	18.5857	82.6939	834.0153	2.9816	1.3582
21	2018	23	21.6857	81.9184	903.4112	2.2806	1.2878
68	2018	23	24.6286	83.3469	969.8490	2.3816	1.0235
74	2018	23	24.9286	81.0510	962.3714	2.2163	0.8102
88	2018	23	20.5429	79.9490	876.3143	2.9653	2.4398
16	2018	23	22.7143	80.7143	922.0153	2.6408	1.2296
30	2018	23	21.9143	84.2959	898.1020	2.7612	1.7622
6	2018	23	24.6286	83.3469	969.8490	2.3816	1.0235
49	2018	23	23.1571	79.5816	940.5316	2.8418	1.3541
22	2018	23	20.5429	79.9490	876.3143	2.9653	2.4398
45	2018	23	17.9857	81.3571	819.1786	3.1663	1.4439
58	2018	23	23.1571	79.5816	940.5316	2.8418	1.3541
37	2018	23	24.6286	83.3469	969.8490	2.3816	1.0235
17	2018	23	20.9000	83.8265	903.1031	2.9051	2.8020
55	2018	23	20.4571	79.2041	878.8071	2.8816	1.6908
46	2018	23	22.7143	80.7143	922.0153	2.6408	1.2296
86	2018	23	19.5571	81.1020	867.7857	2.7102	1.1929
2	2018	23	19.5571	81.1020	867.7857	2.7102	1.1929
4	2018	23	21.6857	81.9184	903.4112	2.2806	1.2878
47	2018	23	25.0000	81.3980	958.8010	3.6673	0.5347
82	2018	23	20.5429	79.9490	876.3143	2.9653	2.4398
19	2018	23	25.5000	79.2551	960.3684	2.9184	1.0265
20	2018	23	19.5857	77.7245	855.6929	3.3031	2.1480
80	2018	23	20.5429	79.9490	876.3143	2.9653	2.4398
3	2018	23	25.0000	75.6122	944.9010	4.3949	1.2449
52	2018	23	20.9000	83.8265	903.1031	2.9051	2.8020
70	2018	23	24.2286	75.2959	911.7959	2.8520	1.2316
64	2018	23	15.2857	81.3776	778.0673	3.0031	1.9418
48	2018	23	22.0714	84.3878	919.8031	2.7602	1.3235
65	2018	23	20.9000	83.8265	903.1031	2.9051	2.8020
44	2018	23	24.2286	75.2959	911.7959	2.8520	1.2316
75	2018	23	15.2857	81.3776	778.0673	3.0031	1.9418
40	2018	23	22.8286	86.1735	945.6735	3.2102	1.4908
11	2018	23	20.4571	79.2041	878.8071	2.8816	1.6908
35	2018	23	22.4143	84.2041	939.5633	2.4265	1.2888
78	2018	23	21.8143	80.3571	900.5520	3.4592	1.8806
28	2018	23	22.4571	80.4184	929.6143	3.4541	1.4827
39	2018	23	20.9000	83.8265	903.1031	2.9051	2.8020
24	2018	23	23.1571	79.5816	940.5316	2.8418	1.3541
63	2018	23	22.8286	86.1735	945.6735	3.2102	1.4908
62	2018	23	20.2286	80.1429	876.1847	3.0357	1.2224
1	2018	23	20.5429	79.9490	876.3143	2.9653	2.4398
31	2018	24	18.6000	78.8980	846.3531	3.1490	1.0204
79	2018	24	25.3429	77.9184	967.0755	4.4541	0.9673
51	2018	24	23.6000	81.4082	937.0449	3.9622	1.0704
14	2018	24	21.8286	80.0510	895.7755	3.2102	1.4633
67	2018	24	21.3714	82.0612	900.8561	3.4122	2.3000
42	2018	24	20.4286	77.7551	874.2092	3.5612	2.3592
50	2018	24	21.4143	78.6633	901.1735	3.4684	1.3510

43	2018	24	20.4286	77.7551	874.2092	3.5612	2.3592
85	2018	24	24.3714	76.5000	909.5082	3.4847	1.1480
25	2018	24	25.6714	85.9286	973.8745	3.0969	0.7265
69	2018	24	23.9143	77.7245	938.1061	3.6102	1.3735
57	2018	24	20.3571	80.8673	886.1806	3.7071	1.8745
9	2018	24	19.3286	76.9184	853.7265	3.1449	1.7908
72	2018	24	20.2714	78.2551	876.6337	2.8969	1.2408
26	2018	24	20.2857	81.3571	866.2398	3.1949	0.7337
7	2018	24	19.8857	85.4796	858.5990	3.3602	1.1296
83	2018	24	24.5429	77.7551	942.4041	3.7449	1.2143
76	2018	24	22.4429	77.1837	919.7122	3.4520	1.0929
36	2018	24	23.4429	78.3673	927.1949	4.1041	1.3847
81	2018	24	23.6000	81.4082	937.0449	3.9622	1.0704
15	2018	24	21.2286	79.4184	917.5367	3.6765	1.2949
32	2018	24	20.4286	77.7551	874.2092	3.5612	2.3592
73	2018	24	24.7286	75.0510	959.7949	3.8837	0.8357
71	2018	24	23.4429	78.3673	927.1949	4.1041	1.3847
41	2018	24	20.5714	81.6020	874.1653	3.5765	1.0296
10	2018	24	24.3857	82.0612	961.5439	3.8469	0.9561
23	2018	24	14.7857	85.2551	776.5367	2.9163	1.7827
27	2018	24	19.8857	85.4796	858.5990	3.3602	1.1296
60	2018	24	23.6000	81.4082	937.0449	3.9622	1.0704
53	2018	24	19.3286	76.9184	853.7265	3.1449	1.7908
66	2018	24	21.8286	80.0510	895.7755	3.2102	1.4633
59	2018	24	20.3571	80.8673	886.1806	3.7071	1.8745
61	2018	24	24.7286	75.0510	959.7949	3.8837	0.8357
84	2018	24	24.7286	75.0510	959.7949	3.8837	0.8357
38	2018	24	20.3571	80.8673	886.1806	3.7071	1.8745
87	2018	24	22.0714	80.7653	898.2939	3.4878	1.5980
34	2018	24	20.3571	80.8673	886.1806	3.7071	1.8745
29	2018	24	23.9143	77.7245	938.1061	3.6102	1.3735
5	2018	24	18.0857	83.9694	832.2163	3.1480	0.9888
8	2018	24	19.3286	76.9184	853.7265	3.1449	1.7908
12	2018	24	18.0857	83.9694	832.2163	3.1480	0.9888
13	2018	24	24.5429	77.7551	942.4041	3.7449	1.2143
18	2018	24	25.0857	79.6122	961.4214	3.8694	0.8684
33	2018	24	21.4143	78.6633	901.1735	3.4684	1.3510
56	2018	24	25.6714	85.9286	973.8745	3.0969	0.7265
77	2018	24	21.2286	79.4184	917.5367	3.6765	1.2949
54	2018	24	18.0857	83.9694	832.2163	3.1480	0.9888
21	2018	24	21.4143	78.6633	901.1735	3.4684	1.3510
68	2018	24	25.3429	77.9184	967.0755	4.4541	0.9673
74	2018	24	24.7286	75.0510	959.7949	3.8837	0.8357
88	2018	24	20.4286	77.7551	874.2092	3.5612	2.3592
16	2018	24	22.4429	77.1837	919.7122	3.4520	1.0929
30	2018	24	21.8286	80.0510	895.7755	3.2102	1.4633
6	2018	24	25.3429	77.9184	967.0755	4.4541	0.9673
49	2018	24	23.9143	77.7245	938.1061	3.6102	1.3735
22	2018	24	20.4286	77.7551	874.2092	3.5612	2.3592
45	2018	24	17.6143	84.1837	817.4847	3.0102	1.0633

58	2018	24	23.9143	77.7245	938.1061	3.6102	1.3735
37	2018	24	25.3429	77.9184	967.0755	4.4541	0.9673
17	2018	24	21.3714	82.0612	900.8561	3.4122	2.3000
55	2018	24	20.2714	78.2551	876.6337	2.8969	1.2408
46	2018	24	22.4429	77.1837	919.7122	3.4520	1.0929
86	2018	24	20.1571	80.9490	865.8051	3.4663	0.9592
2	2018	24	20.1571	80.9490	865.8051	3.4663	0.9592
4	2018	24	21.4143	78.6633	901.1735	3.4684	1.3510
47	2018	24	24.8000	82.1327	956.2133	3.5071	0.5469
82	2018	24	20.4286	77.7551	874.2092	3.5612	2.3592
19	2018	24	25.3000	78.0918	957.6092	3.5500	0.9378
20	2018	24	19.3286	76.9184	853.7265	3.1449	1.7908
80	2018	24	20.4286	77.7551	874.2092	3.5612	2.3592
3	2018	24	24.5429	77.7551	942.4041	3.7449	1.2143
52	2018	24	21.3714	82.0612	900.8561	3.4122	2.3000
70	2018	24	24.3714	76.5000	909.5082	3.4847	1.1480
64	2018	24	14.7857	85.2551	776.5367	2.9163	1.7827
48	2018	24	21.2286	79.4184	917.5367	3.6765	1.2949
65	2018	24	21.3714	82.0612	900.8561	3.4122	2.3000
44	2018	24	24.3714	76.5000	909.5082	3.4847	1.1480
75	2018	24	14.7857	85.2551	776.5367	2.9163	1.7827
40	2018	24	23.9429	83.2449	942.9888	4.3255	1.1235
11	2018	24	20.2714	78.2551	876.6337	2.8969	1.2408
35	2018	24	23.6000	81.4082	937.0449	3.9622	1.0704
78	2018	24	22.0714	80.7653	898.2939	3.4878	1.5980
28	2018	24	23.4429	78.3673	927.1949	4.1041	1.3847
39	2018	24	21.3714	82.0612	900.8561	3.4122	2.3000
24	2018	24	23.9143	77.7245	938.1061	3.6102	1.3735
63	2018	24	23.9429	83.2449	942.9888	4.3255	1.1235
62	2018	24	20.5714	81.6020	874.1653	3.5765	1.0296
1	2018	24	20.4286	77.7551	874.2092	3.5612	2.3592
31	2018	25	21.2286	82.1224	843.2602	1.8561	1.0694
79	2018	25	25.8286	75.7143	963.3551	4.2531	1.0918
51	2018	25	24.3857	80.1429	933.5847	4.2561	1.2745
14	2018	25	23.3000	81.3367	892.9571	2.0531	1.9092
67	2018	25	22.9714	83.2041	897.7714	2.1602	2.3102
42	2018	25	22.3143	79.2857	871.1929	2.2347	2.2643
50	2018	25	23.8000	81.0408	897.6816	1.7398	1.5143
43	2018	25	22.3143	79.2857	871.1929	2.2347	2.2643
85	2018	25	27.0571	78.6327	905.8857	2.4837	1.4908
25	2018	25	27.0429	83.7347	970.2286	3.6112	0.9714
69	2018	25	25.7857	75.7959	934.4796	2.6694	1.5786
57	2018	25	22.7571	81.4388	882.8959	2.3776	2.1367
9	2018	25	21.7429	79.5306	850.8469	2.3888	2.0816
72	2018	25	22.3857	80.5102	873.7602	2.1357	1.2561
26	2018	25	22.7571	85.2755	863.4592	2.7776	1.0459
7	2018	25	22.7857	87.6837	855.7908	2.6184	1.3204
83	2018	25	26.9143	79.0306	939.1541	2.9439	1.0847
76	2018	25	24.3429	77.5510	916.2010	2.2500	1.2449
36	2018	25	25.0000	76.9388	923.7755	3.3286	1.5276

81	2018	25	24.3857	80.1429	933.5847	4.2561	1.2745
15	2018	25	23.8714	81.6122	913.9949	2.0153	1.2214
32	2018	25	22.3143	79.2857	871.1929	2.2347	2.2643
73	2018	25	26.1000	74.7653	956.0296	3.0847	0.8653
71	2018	25	25.0000	76.9388	923.7755	3.3286	1.5276
41	2018	25	22.8714	82.8265	870.8939	2.1816	1.2214
10	2018	25	25.6857	81.2041	957.9153	4.3653	1.0806
23	2018	25	18.3857	89.5612	774.0184	2.5531	1.8847
27	2018	25	22.7857	87.6837	855.7908	2.6184	1.3204
60	2018	25	24.3857	80.1429	933.5847	4.2561	1.2745
53	2018	25	21.7429	79.5306	850.8469	2.3888	2.0816
66	2018	25	23.3000	81.3367	892.9571	2.0531	1.9092
59	2018	25	22.7571	81.4388	882.8959	2.3776	2.1367
61	2018	25	26.1000	74.7653	956.0296	3.0847	0.8653
84	2018	25	26.1000	74.7653	956.0296	3.0847	0.8653
38	2018	25	22.7571	81.4388	882.8959	2.3776	2.1367
87	2018	25	24.0429	82.2347	895.1837	2.7143	1.3990
34	2018	25	22.7571	81.4388	882.8959	2.3776	2.1367
29	2018	25	25.7857	75.7959	934.4796	2.6694	1.5786
5	2018	25	21.2714	86.6531	829.4898	2.6663	1.0898
8	2018	25	21.7429	79.5306	850.8469	2.3888	2.0816
12	2018	25	21.2714	86.6531	829.4898	2.6663	1.0898
13	2018	25	26.9143	79.0306	939.1541	2.9439	1.0847
18	2018	25	25.3714	79.3367	957.8133	2.7031	0.9102
33	2018	25	23.8000	81.0408	897.6816	1.7398	1.5143
56	2018	25	27.0429	83.7347	970.2286	3.6112	0.9714
77	2018	25	23.8714	81.6122	913.9949	2.0153	1.2214
54	2018	25	21.2714	86.6531	829.4898	2.6663	1.0898
21	2018	25	23.8000	81.0408	897.6816	1.7398	1.5143
68	2018	25	25.8286	75.7143	963.3551	4.2531	1.0918
74	2018	25	26.1000	74.7653	956.0296	3.0847	0.8653
88	2018	25	22.3143	79.2857	871.1929	2.2347	2.2643
16	2018	25	24.3429	77.5510	916.2010	2.2500	1.2449
30	2018	25	23.3000	81.3367	892.9571	2.0531	1.9092
6	2018	25	25.8286	75.7143	963.3551	4.2531	1.0918
49	2018	25	25.7857	75.7959	934.4796	2.6694	1.5786
22	2018	25	22.3143	79.2857	871.1929	2.2347	2.2643
45	2018	25	21.2429	85.1939	814.8898	2.8122	1.1735
58	2018	25	25.7857	75.7959	934.4796	2.6694	1.5786
37	2018	25	25.8286	75.7143	963.3551	4.2531	1.0918
17	2018	25	22.9714	83.2041	897.7714	2.1602	2.3102
55	2018	25	22.3857	80.5102	873.7602	2.1357	1.2561
46	2018	25	24.3429	77.5510	916.2010	2.2500	1.2449
86	2018	25	22.4143	82.6122	862.6306	2.5418	1.1184
2	2018	25	22.4143	82.6122	862.6306	2.5418	1.1184
4	2018	25	23.8000	81.0408	897.6816	1.7398	1.5143
47	2018	25	26.7571	83.7143	952.8633	3.3276	0.4286
82	2018	25	22.3143	79.2857	871.1929	2.2347	2.2643
19	2018	25	26.8143	78.9184	954.3969	2.2663	0.9010
20	2018	25	21.7429	79.5306	850.8469	2.3888	2.0816

80	2018	25	22.3143	79.2857	871.1929	2.2347	2.2643
3	2018	25	26.9143	79.0306	939.1541	2.9439	1.0847
52	2018	25	22.9714	83.2041	897.7714	2.1602	2.3102
70	2018	25	27.0571	78.6327	905.8857	2.4837	1.4908
64	2018	25	18.3857	89.5612	774.0184	2.5531	1.8847
48	2018	25	23.8714	81.6122	913.9949	2.0153	1.2214
65	2018	25	22.9714	83.2041	897.7714	2.1602	2.3102
44	2018	25	27.0571	78.6327	905.8857	2.4837	1.4908
75	2018	25	18.3857	89.5612	774.0184	2.5531	1.8847
40	2018	25	26.0857	79.7041	939.6153	4.5551	1.3112
11	2018	25	22.3857	80.5102	873.7602	2.1357	1.2561
35	2018	25	24.3857	80.1429	933.5847	4.2561	1.2745
78	2018	25	24.0429	82.2347	895.1837	2.7143	1.3990
28	2018	25	25.0000	76.9388	923.7755	3.3286	1.5276
39	2018	25	22.9714	83.2041	897.7714	2.1602	2.3102
24	2018	25	25.7857	75.7959	934.4796	2.6694	1.5786
63	2018	25	26.0857	79.7041	939.6153	4.5551	1.3112
62	2018	25	22.8714	82.8265	870.8939	2.1816	1.2214
1	2018	25	22.3143	79.2857	871.1929	2.2347	2.2643
31	2018	26	23.2571	83.7959	842.5663	2.0704	1.1020
79	2018	26	28.4571	78.9898	963.2745	3.7133	1.2265
51	2018	26	26.2857	83.1633	933.5959	3.8041	1.3133
14	2018	26	23.2143	89.0510	893.2663	1.2031	2.5378
67	2018	26	23.6143	88.8061	897.8255	1.2143	2.6235
42	2018	26	23.2571	85.6429	870.9929	1.8949	2.2449
50	2018	26	25.9000	83.6122	896.9592	1.7663	1.7469
43	2018	26	23.2571	85.6429	870.9929	1.8949	2.2449
85	2018	26	27.3571	78.3163	904.9020	3.3806	1.8673
25	2018	26	27.6429	86.5204	970.5592	3.5082	1.0184
69	2018	26	26.9571	77.5612	934.2010	2.3224	1.6112
57	2018	26	24.7143	83.3878	882.3561	2.5439	2.7602
9	2018	26	22.0286	85.1020	850.6010	2.5041	2.3245
72	2018	26	22.4571	86.0000	873.6327	1.8051	1.4888
26	2018	26	22.8286	89.1020	863.1694	3.0439	1.5888
7	2018	26	23.0000	90.0510	855.4918	2.6357	1.7643
83	2018	26	27.3429	80.8367	938.9806	3.1765	0.9724
76	2018	26	26.3143	80.2653	915.7776	1.3398	1.4337
36	2018	26	26.5429	78.5612	923.7306	3.0439	1.6551
81	2018	26	26.2857	83.1633	933.5959	3.8041	1.3133
15	2018	26	26.0857	83.2143	913.2949	1.7245	1.3878
32	2018	26	23.2571	85.6429	870.9929	1.8949	2.2449
73	2018	26	28.3143	78.1939	955.6112	2.3286	0.8602
71	2018	26	26.5429	78.5612	923.7306	3.0439	1.6551
41	2018	26	24.5143	86.1633	870.2939	2.2939	1.5408
10	2018	26	27.2286	83.4592	958.0204	3.4276	0.9878
23	2018	26	19.2143	90.4388	773.7704	2.7194	1.9755
27	2018	26	23.0000	90.0510	855.4918	2.6357	1.7643
60	2018	26	26.2857	83.1633	933.5959	3.8041	1.3133
53	2018	26	22.0286	85.1020	850.6010	2.5041	2.3245
66	2018	26	23.2143	89.0510	893.2663	1.2031	2.5378

59	2018	26	24.7143	83.3878	882.3561	2.5439	2.7602
61	2018	26	28.3143	78.1939	955.6112	2.3286	0.8602
84	2018	26	28.3143	78.1939	955.6112	2.3286	0.8602
38	2018	26	24.7143	83.3878	882.3561	2.5439	2.7602
87	2018	26	24.6571	87.7245	895.0531	2.3735	1.2133
34	2018	26	24.7143	83.3878	882.3561	2.5439	2.7602
29	2018	26	26.9571	77.5612	934.2010	2.3224	1.6112
5	2018	26	21.4000	89.0000	829.1582	2.5918	1.3071
8	2018	26	22.0286	85.1020	850.6010	2.5041	2.3245
12	2018	26	21.4000	89.0000	829.1582	2.5918	1.3071
13	2018	26	27.3429	80.8367	938.9806	3.1765	0.9724
18	2018	26	28.0857	82.4184	957.7082	2.2214	1.1347
33	2018	26	25.9000	83.6122	896.9592	1.7663	1.7469
56	2018	26	27.6429	86.5204	970.5592	3.5082	1.0184
77	2018	26	26.0857	83.2143	913.2949	1.7245	1.3878
54	2018	26	21.4000	89.0000	829.1582	2.5918	1.3071
21	2018	26	25.9000	83.6122	896.9592	1.7663	1.7469
68	2018	26	28.4571	78.9898	963.2745	3.7133	1.2265
74	2018	26	28.3143	78.1939	955.6112	2.3286	0.8602
88	2018	26	23.2571	85.6429	870.9929	1.8949	2.2449
16	2018	26	26.3143	80.2653	915.7776	1.3398	1.4337
30	2018	26	23.2143	89.0510	893.2663	1.2031	2.5378
6	2018	26	28.4571	78.9898	963.2745	3.7133	1.2265
49	2018	26	26.9571	77.5612	934.2010	2.3224	1.6112
22	2018	26	23.2571	85.6429	870.9929	1.8949	2.2449
45	2018	26	21.4000	84.8469	814.5990	2.6571	1.4571
58	2018	26	26.9571	77.5612	934.2010	2.3224	1.6112
37	2018	26	28.4571	78.9898	963.2745	3.7133	1.2265
17	2018	26	23.6143	88.8061	897.8255	1.2143	2.6235
55	2018	26	22.4571	86.0000	873.6327	1.8051	1.4888
46	2018	26	26.3143	80.2653	915.7776	1.3398	1.4337
86	2018	26	23.9143	86.0918	862.0776	2.3061	1.4378
2	2018	26	23.9143	86.0918	862.0776	2.3061	1.4378
4	2018	26	25.9000	83.6122	896.9592	1.7663	1.7469
47	2018	26	27.1000	87.0000	952.8857	3.1204	0.2490
82	2018	26	23.2571	85.6429	870.9929	1.8949	2.2449
19	2018	26	26.6857	85.0612	954.8663	1.2827	0.7510
20	2018	26	22.0286	85.1020	850.6010	2.5041	2.3245
80	2018	26	23.2571	85.6429	870.9929	1.8949	2.2449
3	2018	26	27.3429	80.8367	938.9806	3.1765	0.9724
52	2018	26	23.6143	88.8061	897.8255	1.2143	2.6235
70	2018	26	27.3571	78.3163	904.9020	3.3806	1.8673
64	2018	26	19.2143	90.4388	773.7704	2.7194	1.9755
48	2018	26	26.0857	83.2143	913.2949	1.7245	1.3878
65	2018	26	23.6143	88.8061	897.8255	1.2143	2.6235
44	2018	26	27.3571	78.3163	904.9020	3.3806	1.8673
75	2018	26	19.2143	90.4388	773.7704	2.7194	1.9755
40	2018	26	26.8429	79.9184	940.0082	4.4418	1.7276
11	2018	26	22.4571	86.0000	873.6327	1.8051	1.4888
35	2018	26	26.2857	83.1633	933.5959	3.8041	1.3133

78	2018	26	24.6571	87.7245	895.0531	2.3735	1.2133
28	2018	26	26.5429	78.5612	923.7306	3.0439	1.6551
39	2018	26	23.6143	88.8061	897.8255	1.2143	2.6235
24	2018	26	26.9571	77.5612	934.2010	2.3224	1.6112
63	2018	26	26.8429	79.9184	940.0082	4.4418	1.7276
62	2018	26	24.5143	86.1633	870.2939	2.2939	1.5408
1	2018	26	23.2571	85.6429	870.9929	1.8949	2.2449
31	2018	27	22.4857	83.0306	841.4071	3.0020	1.0439
79	2018	27	27.6000	79.6429	961.9990	4.3806	1.4582
51	2018	27	26.3857	82.4286	932.4490	4.4949	1.3204
14	2018	27	24.5714	91.1531	892.4765	1.6327	2.9122
67	2018	27	24.4143	88.9184	896.8480	1.8704	3.2082
42	2018	27	24.2571	84.6429	869.9806	2.9000	2.4500
50	2018	27	24.7429	82.8878	895.5714	2.5694	1.9418
43	2018	27	24.2571	84.6429	869.9806	2.9000	2.4500
85	2018	27	25.4571	75.0816	903.4000	4.3929	1.9827
25	2018	27	28.0429	87.3163	969.6306	3.5684	1.0429
69	2018	27	27.4714	74.8163	932.8918	3.5694	1.6051
57	2018	27	25.1286	79.7551	881.1031	3.7827	3.3551
9	2018	27	23.2714	84.2143	849.6867	3.4429	2.3796
72	2018	27	24.6000	84.8163	872.7306	2.6469	1.6622
26	2018	27	24.8143	86.0714	862.2929	4.8143	1.8449
7	2018	27	24.0571	86.6224	854.6061	3.7082	1.9551
83	2018	27	28.8571	78.1020	937.8949	5.0551	0.9929
76	2018	27	26.2429	79.8571	914.3878	1.9796	1.4092
36	2018	27	27.0286	76.7959	922.6224	4.1694	1.7306
81	2018	27	26.3857	82.4286	932.4490	4.4949	1.3204
15	2018	27	24.8857	83.1224	911.8867	2.5459	1.4653
32	2018	27	24.2571	84.6429	869.9806	2.9000	2.4500
73	2018	27	28.1143	76.7347	954.1306	3.2490	0.8827
71	2018	27	27.0286	76.7959	922.6224	4.1694	1.7306
41	2018	27	24.9000	83.4184	869.0735	3.6408	1.7673
10	2018	27	27.3667	82.2347	957.8918	3.3071	0.9959
23	2018	27	19.4000	88.6327	773.1245	2.3806	1.7908
27	2018	27	24.0571	86.6224	854.6061	3.7082	1.9551
60	2018	27	26.3857	82.4286	932.4490	4.4949	1.3204
53	2018	27	23.2714	84.2143	849.6867	3.4429	2.3796
66	2018	27	24.5714	91.1531	892.4765	1.6327	2.9122
59	2018	27	25.1286	79.7551	881.1031	3.7827	3.3551
61	2018	27	28.1143	76.7347	954.1306	3.2490	0.8827
84	2018	27	28.1143	76.7347	954.1306	3.2490	0.8827
38	2018	27	25.1286	79.7551	881.1031	3.7827	3.3551
87	2018	27	26.2000	86.6633	894.0347	3.2398	1.1939
34	2018	27	25.1286	79.7551	881.1031	3.7827	3.3551
29	2018	27	27.4714	74.8163	932.8918	3.5694	1.6051
5	2018	27	22.3286	86.1633	828.3316	2.9153	1.2796
8	2018	27	23.2714	84.2143	849.6867	3.4429	2.3796
12	2018	27	22.3286	86.1633	828.3316	2.9153	1.2796
13	2018	27	28.8571	78.1020	937.8949	5.0551	0.9929
18	2018	27	26.8143	81.8571	956.4520	2.4949	1.2306

33	2018	27	24.7429	82.8878	895.5714	2.5694	1.9418
56	2018	27	28.0429	87.3163	969.6306	3.5684	1.0429
77	2018	27	24.8857	83.1224	911.8867	2.5459	1.4653
54	2018	27	22.3286	86.1633	828.3316	2.9153	1.2796
21	2018	27	24.7429	82.8878	895.5714	2.5694	1.9418
68	2018	27	27.6000	79.6429	961.9990	4.3806	1.4582
74	2018	27	28.1143	76.7347	954.1306	3.2490	0.8827
88	2018	27	24.2571	84.6429	869.9806	2.9000	2.4500
16	2018	27	26.2429	79.8571	914.3878	1.9796	1.4092
30	2018	27	24.5714	91.1531	892.4765	1.6327	2.9122
6	2018	27	27.6000	79.6429	961.9990	4.3806	1.4582
49	2018	27	27.4714	74.8163	932.8918	3.5694	1.6051
22	2018	27	24.2571	84.6429	869.9806	2.9000	2.4500
45	2018	27	22.0714	80.9796	813.8520	2.8418	1.7439
58	2018	27	27.4714	74.8163	932.8918	3.5694	1.6051
37	2018	27	27.6000	79.6429	961.9990	4.3806	1.4582
17	2018	27	24.4143	88.9184	896.8480	1.8704	3.2082
55	2018	27	24.6000	84.8163	872.7306	2.6469	1.6622
46	2018	27	26.2429	79.8571	914.3878	1.9796	1.4092
86	2018	27	24.0000	83.7041	860.9245	2.9235	1.8347
2	2018	27	24.0000	83.7041	860.9245	2.9235	1.8347
4	2018	27	24.7429	82.8878	895.5714	2.5694	1.9418
47	2018	27	28.5714	85.4592	951.7684	3.2429	0.2469
82	2018	27	24.2571	84.6429	869.9806	2.9000	2.4500
19	2018	27	28.0857	87.7143	953.9592	1.5061	0.7418
20	2018	27	23.2714	84.2143	849.6867	3.4429	2.3796
80	2018	27	24.2571	84.6429	869.9806	2.9000	2.4500
3	2018	27	28.8571	78.1020	937.8949	5.0551	0.9929
52	2018	27	24.4143	88.9184	896.8480	1.8704	3.2082
70	2018	27	25.4571	75.0816	903.4000	4.3929	1.9827
64	2018	27	19.4000	88.6327	773.1245	2.3806	1.7908
48	2018	27	24.8857	83.1224	911.8867	2.5459	1.4653
65	2018	27	24.4143	88.9184	896.8480	1.8704	3.2082
44	2018	27	25.4571	75.0816	903.4000	4.3929	1.9827
75	2018	27	19.4000	88.6327	773.1245	2.3806	1.7908
40	2018	27	26.6714	80.0102	939.2051	5.0786	1.9898
11	2018	27	24.6000	84.8163	872.7306	2.6469	1.6622
35	2018	27	26.3857	82.4286	932.4490	4.4949	1.3204
78	2018	27	26.2000	86.6633	894.0347	3.2398	1.1939
28	2018	27	27.0286	76.7959	922.6224	4.1694	1.7306
39	2018	27	24.4143	88.9184	896.8480	1.8704	3.2082
24	2018	27	27.4714	74.8163	932.8918	3.5694	1.6051
63	2018	27	26.6714	80.0102	939.2051	5.0786	1.9898
62	2018	27	24.9000	83.4184	869.0735	3.6408	1.7673
1	2018	27	24.2571	84.6429	869.9806	2.9000	2.4500
31	2018	28	23.9571	81.6122	841.3663	3.6806	1.0449
79	2018	28	28.1143	78.6122	961.5347	4.5663	1.4918
51	2018	28	26.0571	81.6224	932.0143	4.7235	1.3571
14	2018	28	23.7143	89.1939	892.0816	2.2214	2.3398
67	2018	28	23.9714	87.0306	896.5173	2.9704	2.8939

42	2018	28	24.3714	79.7551	869.7969	4.0429	2.5143
50	2018	28	26.1000	80.5714	895.3663	3.0837	1.7949
43	2018	28	24.3714	79.7551	869.7969	4.0429	2.5143
85	2018	28	27.8286	73.5204	903.2786	4.3520	1.8857
25	2018	28	27.9429	85.8265	968.9745	4.1776	1.0531
69	2018	28	26.8571	69.7143	932.5163	4.3898	1.4847
57	2018	28	25.5000	76.2143	880.9357	4.3806	3.0816
9	2018	28	23.0143	81.6020	849.6010	4.4194	2.3061
72	2018	28	23.8571	81.2245	872.5704	4.4673	1.6939
26	2018	28	23.5286	81.9286	862.2327	6.8480	1.9204
7	2018	28	23.1000	83.2551	854.5643	4.9204	1.7531
83	2018	28	27.9571	75.3061	937.5571	6.7490	1.0122
76	2018	28	26.7429	79.3265	914.0745	3.3847	1.2735
36	2018	28	26.3571	74.8776	922.2112	4.5867	1.6204
81	2018	28	26.0571	81.6224	932.0143	4.7235	1.3571
15	2018	28	26.8714	82.4592	911.6276	3.9061	1.3735
32	2018	28	24.3714	79.7551	869.7969	4.0429	2.5143
73	2018	28	28.5000	76.1939	953.7163	3.9571	0.8480
71	2018	28	26.3571	74.8776	922.2112	4.5867	1.6204
41	2018	28	25.2000	80.0612	868.9602	4.4918	1.8745
10	2018	28	27.0286	81.8163	957.3255	3.6663	1.1082
23	2018	28	18.9000	89.0510	773.1704	2.2796	1.7520
27	2018	28	23.1000	83.2551	854.5643	4.9204	1.7531
60	2018	28	26.0571	81.6224	932.0143	4.7235	1.3571
53	2018	28	23.0143	81.6020	849.6010	4.4194	2.3061
66	2018	28	23.7143	89.1939	892.0816	2.2214	2.3398
59	2018	28	25.5000	76.2143	880.9357	4.3806	3.0816
61	2018	28	28.5000	76.1939	953.7163	3.9571	0.8480
84	2018	28	28.5000	76.1939	953.7163	3.9571	0.8480
38	2018	28	25.5000	76.2143	880.9357	4.3806	3.0816
87	2018	28	25.4714	83.4490	893.7602	4.8255	1.1520
34	2018	28	25.5000	76.2143	880.9357	4.3806	3.0816
29	2018	28	26.8571	69.7143	932.5163	4.3898	1.4847
5	2018	28	21.8286	83.4388	828.3582	3.7429	1.2449
8	2018	28	23.0143	81.6020	849.6010	4.4194	2.3061
12	2018	28	21.8286	83.4388	828.3582	3.7429	1.2449
13	2018	28	27.9571	75.3061	937.5571	6.7490	1.0122
18	2018	28	27.7286	82.8673	956.0480	2.4224	1.0806
33	2018	28	26.1000	80.5714	895.3663	3.0837	1.7949
56	2018	28	27.9429	85.8265	968.9745	4.1776	1.0531
77	2018	28	26.8714	82.4592	911.6276	3.9061	1.3735
54	2018	28	21.8286	83.4388	828.3582	3.7429	1.2449
21	2018	28	26.1000	80.5714	895.3663	3.0837	1.7949
68	2018	28	28.1143	78.6122	961.5347	4.5663	1.4918
74	2018	28	28.5000	76.1939	953.7163	3.9571	0.8480
88	2018	28	24.3714	79.7551	869.7969	4.0429	2.5143
16	2018	28	26.7429	79.3265	914.0745	3.3847	1.2735
30	2018	28	23.7143	89.1939	892.0816	2.2214	2.3398
6	2018	28	28.1143	78.6122	961.5347	4.5663	1.4918
49	2018	28	26.8571	69.7143	932.5163	4.3898	1.4847

22	2018	28	24.3714	79.7551	869.7969	4.0429	2.5143
45	2018	28	21.3429	78.9898	813.9051	3.6959	1.8714
58	2018	28	26.8571	69.7143	932.5163	4.3898	1.4847
37	2018	28	28.1143	78.6122	961.5347	4.5663	1.4918
17	2018	28	23.9714	87.0306	896.5173	2.9704	2.8939
55	2018	28	23.8571	81.2245	872.5704	4.4673	1.6939
46	2018	28	26.7429	79.3265	914.0745	3.3847	1.2735
86	2018	28	23.9857	82.2857	860.8500	3.8173	1.7490
2	2018	28	23.9857	82.2857	860.8500	3.8173	1.7490
4	2018	28	26.1000	80.5714	895.3663	3.0837	1.7949
47	2018	28	27.2857	83.6531	951.3235	4.0857	0.3510
82	2018	28	24.3714	79.7551	869.7969	4.0429	2.5143
19	2018	28	27.7429	85.0612	953.2888	2.7224	0.7337
20	2018	28	23.0143	81.6020	849.6010	4.4194	2.3061
80	2018	28	24.3714	79.7551	869.7969	4.0429	2.5143
3	2018	28	27.9571	75.3061	937.5571	6.7490	1.0122
52	2018	28	23.9714	87.0306	896.5173	2.9704	2.8939
70	2018	28	27.8286	73.5204	903.2786	4.3520	1.8857
64	2018	28	18.9000	89.0510	773.1704	2.2796	1.7520
48	2018	28	26.8714	82.4592	911.6276	3.9061	1.3735
65	2018	28	23.9714	87.0306	896.5173	2.9704	2.8939
44	2018	28	27.8286	73.5204	903.2786	4.3520	1.8857
75	2018	28	18.9000	89.0510	773.1704	2.2796	1.7520
40	2018	28	26.3571	81.0408	938.5898	5.3673	1.7531
11	2018	28	23.8571	81.2245	872.5704	4.4673	1.6939
35	2018	28	26.0571	81.6224	932.0143	4.7235	1.3571
78	2018	28	25.4714	83.4490	893.7602	4.8255	1.1520
28	2018	28	26.3571	74.8776	922.2112	4.5867	1.6204
39	2018	28	23.9714	87.0306	896.5173	2.9704	2.8939
24	2018	28	26.8571	69.7143	932.5163	4.3898	1.4847
63	2018	28	26.3571	81.0408	938.5898	5.3673	1.7531
62	2018	28	25.2000	80.0612	868.9602	4.4918	1.8745
1	2018	28	24.3714	79.7551	869.7969	4.0429	2.5143
31	2018	29	24.6429	73.2041	843.3816	6.5908	1.2551
79	2018	29	30.9286	74.9490	963.5602	6.7949	1.2745
51	2018	29	27.9286	78.7041	934.0714	6.3694	1.3296
14	2018	29	25.1000	85.2857	894.0051	4.3194	1.5816
67	2018	29	25.4857	83.0612	898.5673	5.7214	1.8837
42	2018	29	25.3571	73.2959	871.9184	6.2990	2.3908
50	2018	29	27.8143	70.9592	897.4500	6.7622	1.9347
43	2018	29	25.3571	73.2959	871.9184	6.2990	2.3908
85	2018	29	29.9000	64.3878	905.3306	7.7296	2.0898
25	2018	29	29.3000	81.4184	970.8031	6.1000	1.0092
69	2018	29	27.9429	69.1837	934.7694	6.3796	1.4276
57	2018	29	26.7857	70.3776	883.1265	7.3245	2.6235
9	2018	29	24.0571	76.6327	851.7194	6.5786	2.2969
72	2018	29	24.5143	77.3878	874.6969	6.6816	1.4102
26	2018	29	23.8571	80.0918	864.3673	7.6541	1.6745
7	2018	29	23.2857	82.6939	856.7051	6.3408	1.4735
83	2018	29	28.3000	72.7449	939.7327	8.1602	1.1847

76	2018	29	28.6000	73.1122	916.3153	7.2663	1.3449
36	2018	29	27.4429	74.9592	924.3398	6.0204	1.3265
81	2018	29	27.9286	78.7041	934.0714	6.3694	1.3296
15	2018	29	28.6571	72.6429	913.6449	7.9949	1.5265
32	2018	29	25.3571	73.2959	871.9184	6.2990	2.3908
73	2018	29	30.1429	72.7551	955.9337	6.1510	0.9031
71	2018	29	27.4429	74.9592	924.3398	6.0204	1.3265
41	2018	29	25.8000	74.4592	871.1551	7.1459	1.9582
10	2018	29	28.7571	79.7143	958.3010	5.9582	1.1408
23	2018	29	19.4000	87.0102	775.0704	3.9837	2.0806
27	2018	29	23.2857	82.6939	856.7051	6.3408	1.4735
60	2018	29	27.9286	78.7041	934.0714	6.3694	1.3296
53	2018	29	24.0571	76.6327	851.7194	6.5786	2.2969
66	2018	29	25.1000	85.2857	894.0051	4.3194	1.5816
59	2018	29	26.7857	70.3776	883.1265	7.3245	2.6235
61	2018	29	30.1429	72.7551	955.9337	6.1510	0.9031
84	2018	29	30.1429	72.7551	955.9337	6.1510	0.9031
38	2018	29	26.7857	70.3776	883.1265	7.3245	2.6235
87	2018	29	26.2286	78.5816	895.9061	7.1857	1.1735
34	2018	29	26.7857	70.3776	883.1265	7.3245	2.6235
29	2018	29	27.9429	69.1837	934.7694	6.3796	1.4276
5	2018	29	21.4429	82.5306	830.4969	5.2041	1.3143
8	2018	29	24.0571	76.6327	851.7194	6.5786	2.2969
12	2018	29	21.4429	82.5306	830.4969	5.2041	1.3143
13	2018	29	28.3000	72.7449	939.7327	8.1602	1.1847
18	2018	29	30.0571	79.2347	958.0541	5.6388	1.0378
33	2018	29	27.8143	70.9592	897.4500	6.7622	1.9347
56	2018	29	29.3000	81.4184	970.8031	6.1000	1.0092
77	2018	29	28.6571	72.6429	913.6449	7.9949	1.5265
54	2018	29	21.4429	82.5306	830.4969	5.2041	1.3143
21	2018	29	27.8143	70.9592	897.4500	6.7622	1.9347
68	2018	29	30.9286	74.9490	963.5602	6.7949	1.2745
74	2018	29	30.1429	72.7551	955.9337	6.1510	0.9031
88	2018	29	25.3571	73.2959	871.9184	6.2990	2.3908
16	2018	29	28.6000	73.1122	916.3153	7.2663	1.3449
30	2018	29	25.1000	85.2857	894.0051	4.3194	1.5816
6	2018	29	30.9286	74.9490	963.5602	6.7949	1.2745
49	2018	29	27.9429	69.1837	934.7694	6.3796	1.4276
22	2018	29	25.3571	73.2959	871.9184	6.2990	2.3908
45	2018	29	20.8714	80.7041	816.0112	4.4663	1.6102
58	2018	29	27.9429	69.1837	934.7694	6.3796	1.4276
37	2018	29	30.9286	74.9490	963.5602	6.7949	1.2745
17	2018	29	25.4857	83.0612	898.5673	5.7214	1.8837
55	2018	29	24.5143	77.3878	874.6969	6.6816	1.4102
46	2018	29	28.6000	73.1122	916.3153	7.2663	1.3449
86	2018	29	24.8000	78.4694	863.0714	6.5500	1.5173
2	2018	29	24.8000	78.4694	863.0714	6.5500	1.5173
4	2018	29	27.8143	70.9592	897.4500	6.7622	1.9347
47	2018	29	28.5000	81.0000	953.5143	5.9663	0.4745
82	2018	29	25.3571	73.2959	871.9184	6.2990	2.3908

19	2018	29	29.0667	79.1633	955.3173	5.7959	0.7929
20	2018	29	24.0571	76.6327	851.7194	6.5786	2.2969
80	2018	29	25.3571	73.2959	871.9184	6.2990	2.3908
3	2018	29	28.3000	72.7449	939.7327	8.1602	1.1847
52	2018	29	25.4857	83.0612	898.5673	5.7214	1.8837
70	2018	29	29.9000	64.3878	905.3306	7.7296	2.0898
64	2018	29	19.4000	87.0102	775.0704	3.9837	2.0806
48	2018	29	28.6571	72.6429	913.6449	7.9949	1.5265
65	2018	29	25.4857	83.0612	898.5673	5.7214	1.8837
44	2018	29	29.9000	64.3878	905.3306	7.7296	2.0898
75	2018	29	19.4000	87.0102	775.0704	3.9837	2.0806
40	2018	29	28.2286	78.1429	940.3010	7.3398	1.3602
11	2018	29	24.5143	77.3878	874.6969	6.6816	1.4102
35	2018	29	27.9286	78.7041	934.0714	6.3694	1.3296
78	2018	29	26.2286	78.5816	895.9061	7.1857	1.1735
28	2018	29	27.4429	74.9592	924.3398	6.0204	1.3265
39	2018	29	25.4857	83.0612	898.5673	5.7214	1.8837
24	2018	29	27.9429	69.1837	934.7694	6.3796	1.4276
63	2018	29	28.2286	78.1429	940.3010	7.3398	1.3602
62	2018	29	25.8000	74.4592	871.1551	7.1459	1.9582
1	2018	29	25.3571	73.2959	871.9184	6.2990	2.3908
31	2018	30	23.8286	66.8469	843.9561	8.1888	1.3469
79	2018	30	29.3857	70.1939	963.6582	8.9194	1.3122
51	2018	30	26.6571	75.4490	934.3541	7.6929	1.3602
14	2018	30	23.6857	80.8980	894.3265	6.2867	1.5184
67	2018	30	24.0714	78.6531	898.9194	6.9020	1.8714
42	2018	30	23.1714	71.5612	872.4143	6.9398	2.2418
50	2018	30	26.9000	63.0408	897.8622	8.8418	1.9500
43	2018	30	23.1714	71.5612	872.4143	6.9398	2.2418
85	2018	30	28.8714	55.2449	905.6735	10.0255	2.2092
25	2018	30	27.9714	76.2653	970.9898	7.6051	1.1602
69	2018	30	27.5143	69.1327	935.1724	8.0449	1.6337
57	2018	30	25.0286	66.3878	883.6378	8.7898	2.5714
9	2018	30	22.5714	72.1224	852.2408	7.5265	2.2551
72	2018	30	22.8714	76.0204	875.1939	7.1500	1.2133
26	2018	30	22.1714	79.0102	864.8949	7.7510	1.5449
7	2018	30	22.1857	81.1429	857.2673	7.2357	1.5673
83	2018	30	26.3429	73.6429	940.2020	7.8806	1.3286
76	2018	30	27.7429	65.4286	916.7327	9.2918	1.4173
36	2018	30	26.9429	73.5918	924.6949	7.4265	1.3918
81	2018	30	26.6571	75.4490	934.3541	7.6929	1.3602
15	2018	30	28.2571	62.9490	913.9173	10.2990	1.6265
32	2018	30	23.1714	71.5612	872.4143	6.9398	2.2418
73	2018	30	30.3286	65.6122	956.1724	8.1337	1.1786
71	2018	30	26.9429	73.5918	924.6949	7.4265	1.3918
41	2018	30	24.7000	69.9694	871.7265	8.4163	1.8255
10	2018	30	27.8286	76.2551	958.4847	8.0765	1.2449
23	2018	30	18.7429	81.6531	775.7704	6.0571	2.0490
27	2018	30	22.1857	81.1429	857.2673	7.2357	1.5673
60	2018	30	26.6571	75.4490	934.3541	7.6929	1.3602

53	2018	30	22.5714	72.1224	852.2408	7.5265	2.2551
66	2018	30	23.6857	80.8980	894.3265	6.2867	1.5184
59	2018	30	25.0286	66.3878	883.6378	8.7898	2.5714
61	2018	30	30.3286	65.6122	956.1724	8.1337	1.1786
84	2018	30	30.3286	65.6122	956.1724	8.1337	1.1786
38	2018	30	25.0286	66.3878	883.6378	8.7898	2.5714
87	2018	30	23.5714	78.7347	896.4214	7.2439	1.2031
34	2018	30	25.0286	66.3878	883.6378	8.7898	2.5714
29	2018	30	27.5143	69.1327	935.1724	8.0449	1.6337
5	2018	30	20.2429	82.1122	831.1418	6.3582	1.3949
8	2018	30	22.5714	72.1224	852.2408	7.5265	2.2551
12	2018	30	20.2429	82.1122	831.1418	6.3582	1.3949
13	2018	30	26.3429	73.6429	940.2020	7.8806	1.3286
18	2018	30	29.3143	72.5102	958.1714	8.2765	1.1929
33	2018	30	26.9000	63.0408	897.8622	8.8418	1.9500
56	2018	30	27.9714	76.2653	970.9898	7.6051	1.1602
77	2018	30	28.2571	62.9490	913.9173	10.2990	1.6265
54	2018	30	20.2429	82.1122	831.1418	6.3582	1.3949
21	2018	30	26.9000	63.0408	897.8622	8.8418	1.9500
68	2018	30	29.3857	70.1939	963.6582	8.9194	1.3122
74	2018	30	30.3286	65.6122	956.1724	8.1337	1.1786
88	2018	30	23.1714	71.5612	872.4143	6.9398	2.2418
16	2018	30	27.7429	65.4286	916.7327	9.2918	1.4173
30	2018	30	23.6857	80.8980	894.3265	6.2867	1.5184
6	2018	30	29.3857	70.1939	963.6582	8.9194	1.3122
49	2018	30	27.5143	69.1327	935.1724	8.0449	1.6337
22	2018	30	23.1714	71.5612	872.4143	6.9398	2.2418
45	2018	30	19.9571	79.9898	816.6592	5.3673	1.4102
58	2018	30	27.5143	69.1327	935.1724	8.0449	1.6337
37	2018	30	29.3857	70.1939	963.6582	8.9194	1.3122
17	2018	30	24.0714	78.6531	898.9194	6.9020	1.8714
55	2018	30	22.8714	76.0204	875.1939	7.1500	1.2133
46	2018	30	27.7429	65.4286	916.7327	9.2918	1.4173
86	2018	30	23.7429	73.3265	863.6765	7.9439	1.4316
2	2018	30	23.7429	73.3265	863.6765	7.9439	1.4316
4	2018	30	26.9000	63.0408	897.8622	8.8418	1.9500
47	2018	30	26.2714	79.3673	953.9153	6.2847	0.5684
82	2018	30	23.1714	71.5612	872.4143	6.9398	2.2418
19	2018	30	27.0429	76.7653	956.0102	6.9398	0.9898
20	2018	30	22.5714	72.1224	852.2408	7.5265	2.2551
80	2018	30	23.1714	71.5612	872.4143	6.9398	2.2418
3	2018	30	26.3429	73.6429	940.2020	7.8806	1.3286
52	2018	30	24.0714	78.6531	898.9194	6.9020	1.8714
70	2018	30	28.8714	55.2449	905.6735	10.0255	2.2092
64	2018	30	18.7429	81.6531	775.7704	6.0571	2.0490
48	2018	30	28.2571	62.9490	913.9173	10.2990	1.6265
65	2018	30	24.0714	78.6531	898.9194	6.9020	1.8714
44	2018	30	28.8714	55.2449	905.6735	10.0255	2.2092
75	2018	30	18.7429	81.6531	775.7704	6.0571	2.0490
40	2018	30	27.1286	74.2245	940.4724	8.6980	1.2551

11	2018	30	22.8714	76.0204	875.1939	7.1500	1.2133
35	2018	30	26.6571	75.4490	934.3541	7.6929	1.3602
78	2018	30	23.5714	78.7347	896.4214	7.2439	1.2031
28	2018	30	26.9429	73.5918	924.6949	7.4265	1.3918
39	2018	30	24.0714	78.6531	898.9194	6.9020	1.8714
24	2018	30	27.5143	69.1327	935.1724	8.0449	1.6337
63	2018	30	27.1286	74.2245	940.4724	8.6980	1.2551
62	2018	30	24.7000	69.9694	871.7265	8.4163	1.8255
1	2018	30	23.1714	71.5612	872.4143	6.9398	2.2418
31	2018	31	22.5000	70.0408	843.7806	6.7204	1.2459
79	2018	31	28.4143	70.0816	963.5490	8.8031	1.5959
51	2018	31	25.9714	76.1224	934.2908	7.0490	1.4071
14	2018	31	22.7000	84.2041	894.2663	4.8112	1.9020
67	2018	31	23.3286	81.0714	898.8592	5.1112	2.1265
42	2018	31	23.3714	76.8163	872.2745	5.3347	2.2061
50	2018	31	26.6857	62.4796	897.6704	7.9286	2.0214
43	2018	31	23.3714	76.8163	872.2745	5.3347	2.2061
85	2018	31	27.6429	56.2347	905.5184	9.0980	2.1163
25	2018	31	27.5000	77.3673	971.1194	6.7010	1.2827
69	2018	31	27.1000	68.5408	935.0724	7.8990	1.6918
57	2018	31	25.3857	68.0102	883.4469	8.0041	2.9071
9	2018	31	22.0000	76.2755	851.9796	6.1051	2.1245
72	2018	31	22.5714	80.2551	874.9939	5.0663	1.3031
26	2018	31	21.9143	83.0612	864.6214	6.1806	1.4898
7	2018	31	21.7429	84.6939	857.0000	5.4265	1.6724
83	2018	31	26.2286	78.5816	940.1571	6.2673	1.2673
76	2018	31	27.2429	63.7755	916.5592	7.1214	1.4561
36	2018	31	25.8429	72.9082	924.6439	6.4745	1.6673
81	2018	31	25.9714	76.1224	934.2908	7.0490	1.4071
15	2018	31	26.4000	64.0306	913.7582	9.5694	1.7265
32	2018	31	23.3714	76.8163	872.2745	5.3347	2.2061
73	2018	31	29.1000	63.5510	956.0020	7.8276	1.3724
71	2018	31	25.8429	72.9082	924.6439	6.4745	1.6673
41	2018	31	24.2571	72.1122	871.5316	7.3500	1.7827
10	2018	31	26.8286	74.6122	958.4296	8.0980	1.2112
23	2018	31	18.2286	82.9286	775.4827	5.0908	1.8694
27	2018	31	21.7429	84.6939	857.0000	5.4265	1.6724
60	2018	31	25.9714	76.1224	934.2908	7.0490	1.4071
53	2018	31	22.0000	76.2755	851.9796	6.1051	2.1245
66	2018	31	22.7000	84.2041	894.2663	4.8112	1.9020
59	2018	31	25.3857	68.0102	883.4469	8.0041	2.9071
61	2018	31	29.1000	63.5510	956.0020	7.8276	1.3724
84	2018	31	29.1000	63.5510	956.0020	7.8276	1.3724
38	2018	31	25.3857	68.0102	883.4469	8.0041	2.9071
87	2018	31	24.1000	85.0816	896.3378	5.2551	1.1082
34	2018	31	25.3857	68.0102	883.4469	8.0041	2.9071
29	2018	31	27.1000	68.5408	935.0724	7.8990	1.6918
5	2018	31	20.1571	85.6531	830.8224	5.3449	1.3796
8	2018	31	22.0000	76.2755	851.9796	6.1051	2.1245
12	2018	31	20.1571	85.6531	830.8224	5.3449	1.3796

13	2018	31	26.2286	78.5816	940.1571	6.2673	1.2673
18	2018	31	27.7429	72.6122	958.0531	7.3153	1.2327
33	2018	31	26.6857	62.4796	897.6704	7.9286	2.0214
56	2018	31	27.5000	77.3673	971.1194	6.7010	1.2827
77	2018	31	26.4000	64.0306	913.7582	9.5694	1.7265
54	2018	31	20.1571	85.6531	830.8224	5.3449	1.3796
21	2018	31	26.6857	62.4796	897.6704	7.9286	2.0214
68	2018	31	28.4143	70.0816	963.5490	8.8031	1.5959
74	2018	31	29.1000	63.5510	956.0020	7.8276	1.3724
88	2018	31	23.3714	76.8163	872.2745	5.3347	2.2061
16	2018	31	27.2429	63.7755	916.5592	7.1214	1.4561
30	2018	31	22.7000	84.2041	894.2663	4.8112	1.9020
6	2018	31	28.4143	70.0816	963.5490	8.8031	1.5959
49	2018	31	27.1000	68.5408	935.0724	7.8990	1.6918
22	2018	31	23.3714	76.8163	872.2745	5.3347	2.2061
45	2018	31	19.6571	82.2551	816.3173	4.1031	1.1776
58	2018	31	27.1000	68.5408	935.0724	7.8990	1.6918
37	2018	31	28.4143	70.0816	963.5490	8.8031	1.5959
17	2018	31	23.3286	81.0714	898.8592	5.1112	2.1265
55	2018	31	22.5714	80.2551	874.9939	5.0663	1.3031
46	2018	31	27.2429	63.7755	916.5592	7.1214	1.4561
86	2018	31	23.6429	74.6122	863.4327	6.6316	1.5408
2	2018	31	23.6429	74.6122	863.4327	6.6316	1.5408
4	2018	31	26.6857	62.4796	897.6704	7.9286	2.0214
47	2018	31	26.5143	83.4388	953.9684	4.1592	0.5061
82	2018	31	23.3714	76.8163	872.2745	5.3347	2.2061
19	2018	31	26.6000	80.7755	955.9980	4.8337	1.0602
20	2018	31	22.0000	76.2755	851.9796	6.1051	2.1245
80	2018	31	23.3714	76.8163	872.2745	5.3347	2.2061
3	2018	31	26.2286	78.5816	940.1571	6.2673	1.2673
52	2018	31	23.3286	81.0714	898.8592	5.1112	2.1265
70	2018	31	27.6429	56.2347	905.5184	9.0980	2.1163
64	2018	31	18.2286	82.9286	775.4827	5.0908	1.8694
48	2018	31	26.4000	64.0306	913.7582	9.5694	1.7265
65	2018	31	23.3286	81.0714	898.8592	5.1112	2.1265
44	2018	31	27.6429	56.2347	905.5184	9.0980	2.1163
75	2018	31	18.2286	82.9286	775.4827	5.0908	1.8694
40	2018	31	26.2714	74.2551	940.5459	8.3643	1.2071
11	2018	31	22.5714	80.2551	874.9939	5.0663	1.3031
35	2018	31	25.9714	76.1224	934.2908	7.0490	1.4071
78	2018	31	24.1000	85.0816	896.3378	5.2551	1.1082
28	2018	31	25.8429	72.9082	924.6439	6.4745	1.6673
39	2018	31	23.3286	81.0714	898.8592	5.1112	2.1265
24	2018	31	27.1000	68.5408	935.0724	7.8990	1.6918
63	2018	31	26.2714	74.2551	940.5459	8.3643	1.2071
62	2018	31	24.2571	72.1122	871.5316	7.3500	1.7827
1	2018	31	23.3714	76.8163	872.2745	5.3347	2.2061
31	2018	32	20.5286	80.1837	844.3214	4.2378	0.9337
79	2018	32	26.9714	75.2449	964.2357	7.6316	1.5918
51	2018	32	24.9714	80.4898	934.8510	6.0041	1.3969

14	2018	32	23.1000	90.1020	894.7520	2.9224	1.7112
67	2018	32	23.5571	86.0918	899.3582	3.5541	1.9898
42	2018	32	22.5286	81.5408	872.6520	4.0153	2.0663
50	2018	32	24.0143	69.5714	898.2592	6.4806	2.0204
43	2018	32	22.5286	81.5408	872.6520	4.0153	2.0663
85	2018	32	25.0143	66.9388	906.2888	7.2541	1.7367
25	2018	32	27.1571	81.6122	971.7929	5.2735	1.1776
69	2018	32	25.7143	68.7755	935.6571	6.3337	1.5949
57	2018	32	22.9000	74.6224	883.9102	6.2673	2.5878
9	2018	32	21.2143	82.6735	852.2959	4.5327	1.8878
72	2018	32	22.2000	84.3061	875.3684	3.6847	1.2663
26	2018	32	22.1429	87.5000	864.9837	4.3388	1.3357
7	2018	32	21.4429	90.5000	857.2949	3.4224	1.3980
83	2018	32	25.7714	81.7347	940.7510	5.3959	1.1510
76	2018	32	25.8286	68.6735	917.1153	5.7684	1.5133
36	2018	32	25.4000	77.8571	925.2184	4.8020	1.4418
81	2018	32	24.9714	80.4898	934.8510	6.0041	1.3969
15	2018	32	24.4286	73.3571	914.4306	7.7684	1.7367
32	2018	32	22.5286	81.5408	872.6520	4.0153	2.0663
73	2018	32	26.9857	69.7143	956.7163	6.4888	1.2276
71	2018	32	25.4000	77.8571	925.2184	4.8020	1.4418
41	2018	32	22.8286	79.5918	871.9806	5.7429	1.5622
10	2018	32	26.3571	77.9490	959.0704	6.4837	1.1255
23	2018	32	17.2714	88.0102	775.6908	3.0551	1.7224
27	2018	32	21.4429	90.5000	857.2949	3.4224	1.3980
60	2018	32	24.9714	80.4898	934.8510	6.0041	1.3969
53	2018	32	21.2143	82.6735	852.2959	4.5327	1.8878
66	2018	32	23.1000	90.1020	894.7520	2.9224	1.7112
59	2018	32	22.9000	74.6224	883.9102	6.2673	2.5878
61	2018	32	26.9857	69.7143	956.7163	6.4888	1.2276
84	2018	32	26.9857	69.7143	956.7163	6.4888	1.2276
38	2018	32	22.9000	74.6224	883.9102	6.2673	2.5878
87	2018	32	23.7286	88.5612	896.7551	4.3245	1.1163
34	2018	32	22.9000	74.6224	883.9102	6.2673	2.5878
29	2018	32	25.7143	68.7755	935.6571	6.3337	1.5949
5	2018	32	19.9000	89.6122	831.0194	3.4837	1.1143
8	2018	32	21.2143	82.6735	852.2959	4.5327	1.8878
12	2018	32	19.9000	89.6122	831.0194	3.4837	1.1143
13	2018	32	25.7714	81.7347	940.7510	5.3959	1.1510
18	2018	32	26.6857	78.0408	958.7163	5.9531	1.1439
33	2018	32	24.0143	69.5714	898.2592	6.4806	2.0204
56	2018	32	27.1571	81.6122	971.7929	5.2735	1.1776
77	2018	32	24.4286	73.3571	914.4306	7.7684	1.7367
54	2018	32	19.9000	89.6122	831.0194	3.4837	1.1143
21	2018	32	24.0143	69.5714	898.2592	6.4806	2.0204
68	2018	32	26.9714	75.2449	964.2357	7.6316	1.5918
74	2018	32	26.9857	69.7143	956.7163	6.4888	1.2276
88	2018	32	22.5286	81.5408	872.6520	4.0153	2.0663
16	2018	32	25.8286	68.6735	917.1153	5.7684	1.5133
30	2018	32	23.1000	90.1020	894.7520	2.9224	1.7112

6	2018	32	26.9714	75.2449	964.2357	7.6316	1.5918
49	2018	32	25.7143	68.7755	935.6571	6.3337	1.5949
22	2018	32	22.5286	81.5408	872.6520	4.0153	2.0663
45	2018	32	19.1000	86.1633	816.5306	2.2592	1.0163
58	2018	32	25.7143	68.7755	935.6571	6.3337	1.5949
37	2018	32	26.9714	75.2449	964.2357	7.6316	1.5918
17	2018	32	23.5571	86.0918	899.3582	3.5541	1.9898
55	2018	32	22.2000	84.3061	875.3684	3.6847	1.2663
46	2018	32	25.8286	68.6735	917.1153	5.7684	1.5133
86	2018	32	21.9714	81.5714	863.8092	4.6510	1.3541
2	2018	32	21.9714	81.5714	863.8092	4.6510	1.3541
4	2018	32	24.0143	69.5714	898.2592	6.4806	2.0204
47	2018	32	26.2286	86.5306	954.6357	3.0388	0.3735
82	2018	32	22.5286	81.5408	872.6520	4.0153	2.0663
19	2018	32	27.2429	84.8265	956.2347	3.7265	1.0133
20	2018	32	21.2143	82.6735	852.2959	4.5327	1.8878
80	2018	32	22.5286	81.5408	872.6520	4.0153	2.0663
3	2018	32	25.7714	81.7347	940.7510	5.3959	1.1510
52	2018	32	23.5571	86.0918	899.3582	3.5541	1.9898
70	2018	32	25.0143	66.9388	906.2888	7.2541	1.7367
64	2018	32	17.2714	88.0102	775.6908	3.0551	1.7224
48	2018	32	24.4286	73.3571	914.4306	7.7684	1.7367
65	2018	32	23.5571	86.0918	899.3582	3.5541	1.9898
44	2018	32	25.0143	66.9388	906.2888	7.2541	1.7367
75	2018	32	17.2714	88.0102	775.6908	3.0551	1.7224
40	2018	32	26.3000	77.6020	941.1265	7.3051	1.0796
11	2018	32	22.2000	84.3061	875.3684	3.6847	1.2663
35	2018	32	24.9714	80.4898	934.8510	6.0041	1.3969
78	2018	32	23.7286	88.5612	896.7551	4.3245	1.1163
28	2018	32	25.4000	77.8571	925.2184	4.8020	1.4418
39	2018	32	23.5571	86.0918	899.3582	3.5541	1.9898
24	2018	32	25.7143	68.7755	935.6571	6.3337	1.5949
63	2018	32	26.3000	77.6020	941.1265	7.3051	1.0796
62	2018	32	22.8286	79.5918	871.9806	5.7429	1.5622
1	2018	32	22.5286	81.5408	872.6520	4.0153	2.0663
31	2018	33	21.9000	84.6020	844.8510	3.8122	0.7173
79	2018	33	27.6857	77.8980	963.9092	6.7551	1.3367
51	2018	33	25.2000	83.6837	934.4459	5.3939	1.3194
14	2018	33	24.5143	86.3163	893.9888	4.2949	1.1776
67	2018	33	24.0000	84.3061	898.8337	4.4102	2.2806
42	2018	33	23.6571	80.7041	872.3949	4.9000	1.9949
50	2018	33	24.3857	75.9388	898.5653	5.6480	1.6316
43	2018	33	23.6571	80.7041	872.3949	4.9000	1.9949
85	2018	33	25.6571	75.0204	906.7592	6.2245	1.3459
25	2018	33	28.2286	81.3673	970.7765	5.7888	1.1459
69	2018	33	26.7143	70.2347	935.3959	6.1561	1.4980
57	2018	33	23.1286	80.9490	883.9949	5.2418	1.7418
9	2018	33	22.3571	82.6327	852.1847	5.0357	1.6776
72	2018	33	23.7857	82.7143	875.0061	5.0888	1.3173
26	2018	33	23.1000	84.8673	864.7643	5.3980	1.3061

7	2018	33	22.5571	88.7959	857.1235	4.8704	1.1245
83	2018	33	26.8857	83.4184	940.1327	6.0194	1.1020
76	2018	33	26.4571	70.5918	917.0704	7.0102	1.3959
36	2018	33	25.6286	78.7755	924.7276	5.4949	1.3531
81	2018	33	25.2000	83.6837	934.4459	5.3939	1.3194
15	2018	33	25.2857	78.3571	914.6173	7.1163	1.6765
32	2018	33	23.6571	80.7041	872.3949	4.9000	1.9949
73	2018	33	28.5429	73.9082	956.5776	6.1112	0.9020
71	2018	33	25.6286	78.7755	924.7276	5.4949	1.3531
41	2018	33	23.6857	83.6327	872.1684	5.6061	1.0878
10	2018	33	26.5571	80.3673	958.4204	5.8429	1.1582
23	2018	33	17.6143	89.2653	776.0837	3.2214	1.5316
27	2018	33	22.5571	88.7959	857.1235	4.8704	1.1245
60	2018	33	25.2000	83.6837	934.4459	5.3939	1.3194
53	2018	33	22.3571	82.6327	852.1847	5.0357	1.6776
66	2018	33	24.5143	86.3163	893.9888	4.2949	1.1776
59	2018	33	23.1286	80.9490	883.9949	5.2418	1.7418
61	2018	33	28.5429	73.9082	956.5776	6.1112	0.9020
84	2018	33	28.5429	73.9082	956.5776	6.1112	0.9020
38	2018	33	23.1286	80.9490	883.9949	5.2418	1.7418
87	2018	33	24.4143	87.7857	896.3235	4.9857	1.3000
34	2018	33	23.1286	80.9490	883.9949	5.2418	1.7418
29	2018	33	26.7143	70.2347	935.3959	6.1561	1.4980
5	2018	33	20.6714	87.8061	831.0255	3.9827	0.9010
8	2018	33	22.3571	82.6327	852.1847	5.0357	1.6776
12	2018	33	20.6714	87.8061	831.0255	3.9827	0.9010
13	2018	33	26.8857	83.4184	940.1327	6.0194	1.1020
18	2018	33	27.7857	79.4898	958.4490	5.4837	1.1224
33	2018	33	24.3857	75.9388	898.5653	5.6480	1.6316
56	2018	33	28.2286	81.3673	970.7765	5.7888	1.1459
77	2018	33	25.2857	78.3571	914.6173	7.1163	1.6765
54	2018	33	20.6714	87.8061	831.0255	3.9827	0.9010
21	2018	33	24.3857	75.9388	898.5653	5.6480	1.6316
68	2018	33	27.6857	77.8980	963.9092	6.7551	1.3367
74	2018	33	28.5429	73.9082	956.5776	6.1112	0.9020
88	2018	33	23.6571	80.7041	872.3949	4.9000	1.9949
16	2018	33	26.4571	70.5918	917.0704	7.0102	1.3959
30	2018	33	24.5143	86.3163	893.9888	4.2949	1.1776
6	2018	33	27.6857	77.8980	963.9092	6.7551	1.3367
49	2018	33	26.7143	70.2347	935.3959	6.1561	1.4980
22	2018	33	23.6571	80.7041	872.3949	4.9000	1.9949
45	2018	33	20.1000	84.0510	816.6276	3.4990	1.0122
58	2018	33	26.7143	70.2347	935.3959	6.1561	1.4980
37	2018	33	27.6857	77.8980	963.9092	6.7551	1.3367
17	2018	33	24.0000	84.3061	898.8337	4.4102	2.2806
55	2018	33	23.7857	82.7143	875.0061	5.0888	1.3173
46	2018	33	26.4571	70.5918	917.0704	7.0102	1.3959
86	2018	33	22.6857	85.1939	864.0224	4.4459	0.8704
2	2018	33	22.6857	85.1939	864.0224	4.4459	0.8704
4	2018	33	24.3857	75.9388	898.5653	5.6480	1.6316

47	2018	33	28.1286	83.8163	953.7653	4.4776	0.4347
82	2018	33	23.6571	80.7041	872.3949	4.9000	1.9949
19	2018	33	28.6000	80.1633	955.0449	5.8480	1.1367
20	2018	33	22.3571	82.6327	852.1847	5.0357	1.6776
80	2018	33	23.6571	80.7041	872.3949	4.9000	1.9949
3	2018	33	26.8857	83.4184	940.1327	6.0194	1.1020
52	2018	33	24.0000	84.3061	898.8337	4.4102	2.2806
70	2018	33	25.6571	75.0204	906.7592	6.2245	1.3459
64	2018	33	17.6143	89.2653	776.0837	3.2214	1.5316
48	2018	33	25.2857	78.3571	914.6173	7.1163	1.6765
65	2018	33	24.0000	84.3061	898.8337	4.4102	2.2806
44	2018	33	25.6571	75.0204	906.7592	6.2245	1.3459
75	2018	33	17.6143	89.2653	776.0837	3.2214	1.5316
40	2018	33	26.0000	79.2653	940.1806	7.1765	1.1459
11	2018	33	23.7857	82.7143	875.0061	5.0888	1.3173
35	2018	33	25.2000	83.6837	934.4459	5.3939	1.3194
78	2018	33	24.4143	87.7857	896.3235	4.9857	1.3000
28	2018	33	25.6286	78.7755	924.7276	5.4949	1.3531
39	2018	33	24.0000	84.3061	898.8337	4.4102	2.2806
24	2018	33	26.7143	70.2347	935.3959	6.1561	1.4980
63	2018	33	26.0000	79.2653	940.1806	7.1765	1.1459
62	2018	33	23.6857	83.6327	872.1684	5.6061	1.0878
1	2018	33	23.6571	80.7041	872.3949	4.9000	1.9949
31	2018	34	21.6143	80.5204	844.8469	5.7010	0.8694
79	2018	34	28.4143	73.6837	963.4918	6.9469	1.4235
51	2018	34	25.9143	81.0612	934.0041	5.5786	1.3245
14	2018	34	23.9571	80.5408	893.4061	6.0704	1.1214
67	2018	34	24.0143	80.3878	898.3724	5.9847	2.4663
42	2018	34	23.6286	76.8163	872.1316	6.4908	2.1418
50	2018	34	24.6286	75.7551	898.4490	5.6939	1.6592
43	2018	34	23.6286	76.8163	872.1316	6.4908	2.1418
85	2018	34	26.4429	73.9082	906.5990	6.7408	1.3714
25	2018	34	28.0286	77.0714	969.9939	7.1490	1.2643
69	2018	34	26.3143	71.2551	934.9755	7.1816	1.4235
57	2018	34	23.2571	80.8980	883.8480	5.8173	1.7265
9	2018	34	22.2857	79.2143	852.0041	6.6092	1.8235
72	2018	34	23.3143	78.3673	874.6990	6.6133	1.4847
26	2018	34	23.4143	80.1224	864.5663	6.8286	1.2908
7	2018	34	22.2857	84.7653	856.9786	6.4214	1.1010
83	2018	34	27.2143	80.0204	939.5163	7.5102	1.1449
76	2018	34	25.7000	70.7347	916.8133	7.3000	1.3755
36	2018	34	25.2714	77.0510	924.2602	6.8245	1.5337
81	2018	34	25.9143	81.0612	934.0041	5.5786	1.3245
15	2018	34	24.4143	79.2143	914.4653	7.3969	1.5602
32	2018	34	23.6286	76.8163	872.1316	6.4908	2.1418
73	2018	34	27.6000	71.4796	956.2347	6.6265	1.0459
71	2018	34	25.2714	77.0510	924.2602	6.8245	1.5337
41	2018	34	23.6143	81.6429	872.0408	6.6490	1.0776
10	2018	34	26.7571	77.0408	957.8092	6.1367	1.1153
23	2018	34	17.9000	87.2755	776.1939	4.9255	1.6459

27	2018	34	22.2857	84.7653	856.9786	6.4214	1.1010
60	2018	34	25.9143	81.0612	934.0041	5.5786	1.3245
53	2018	34	22.2857	79.2143	852.0041	6.6092	1.8235
66	2018	34	23.9571	80.5408	893.4061	6.0704	1.1214
59	2018	34	23.2571	80.8980	883.8480	5.8173	1.7265
61	2018	34	27.6000	71.4796	956.2347	6.6265	1.0459
84	2018	34	27.6000	71.4796	956.2347	6.6265	1.0459
38	2018	34	23.2571	80.8980	883.8480	5.8173	1.7265
87	2018	34	24.5429	83.0204	895.9418	6.5173	1.4786
34	2018	34	23.2571	80.8980	883.8480	5.8173	1.7265
29	2018	34	26.3143	71.2551	934.9755	7.1816	1.4235
5	2018	34	20.9429	84.1837	830.9694	5.1969	1.0388
8	2018	34	22.2857	79.2143	852.0041	6.6092	1.8235
12	2018	34	20.9429	84.1837	830.9694	5.1969	1.0388
13	2018	34	27.2143	80.0204	939.5163	7.5102	1.1449
18	2018	34	27.9857	74.8469	958.0816	4.8990	1.1704
33	2018	34	24.6286	75.7551	898.4490	5.6939	1.6592
56	2018	34	28.0286	77.0714	969.9939	7.1490	1.2643
77	2018	34	24.4143	79.2143	914.4653	7.3969	1.5602
54	2018	34	20.9429	84.1837	830.9694	5.1969	1.0388
21	2018	34	24.6286	75.7551	898.4490	5.6939	1.6592
68	2018	34	28.4143	73.6837	963.4918	6.9469	1.4235
74	2018	34	27.6000	71.4796	956.2347	6.6265	1.0459
88	2018	34	23.6286	76.8163	872.1316	6.4908	2.1418
16	2018	34	25.7000	70.7347	916.8133	7.3000	1.3755
30	2018	34	23.9571	80.5408	893.4061	6.0704	1.1214
6	2018	34	28.4143	73.6837	963.4918	6.9469	1.4235
49	2018	34	26.3143	71.2551	934.9755	7.1816	1.4235
22	2018	34	23.6286	76.8163	872.1316	6.4908	2.1418
45	2018	34	20.0000	80.8265	816.6112	5.2643	0.9837
58	2018	34	26.3143	71.2551	934.9755	7.1816	1.4235
37	2018	34	28.4143	73.6837	963.4918	6.9469	1.4235
17	2018	34	24.0143	80.3878	898.3724	5.9847	2.4663
55	2018	34	23.3143	78.3673	874.6990	6.6133	1.4847
46	2018	34	25.7000	70.7347	916.8133	7.3000	1.3755
86	2018	34	22.4000	82.9898	863.9520	5.7398	0.8102
2	2018	34	22.4000	82.9898	863.9520	5.7398	0.8102
4	2018	34	24.6286	75.7551	898.4490	5.6939	1.6592
47	2018	34	27.2714	79.6122	952.9837	7.1051	0.5224
82	2018	34	23.6286	76.8163	872.1316	6.4908	2.1418
19	2018	34	27.4857	74.4286	954.2000	7.7643	1.2796
20	2018	34	22.2857	79.2143	852.0041	6.6092	1.8235
80	2018	34	23.6286	76.8163	872.1316	6.4908	2.1418
3	2018	34	27.2143	80.0204	939.5163	7.5102	1.1449
52	2018	34	24.0143	80.3878	898.3724	5.9847	2.4663
70	2018	34	26.4429	73.9082	906.5990	6.7408	1.3714
64	2018	34	17.9000	87.2755	776.1939	4.9255	1.6459
48	2018	34	24.4143	79.2143	914.4653	7.3969	1.5602
65	2018	34	24.0143	80.3878	898.3724	5.9847	2.4663
44	2018	34	26.4429	73.9082	906.5990	6.7408	1.3714

75	2018	34	17.9000	87.2755	776.1939	4.9255	1.6459
40	2018	34	26.0143	77.8980	939.5071	7.6143	1.2837
11	2018	34	23.3143	78.3673	874.6990	6.6133	1.4847
35	2018	34	25.9143	81.0612	934.0041	5.5786	1.3245
78	2018	34	24.5429	83.0204	895.9418	6.5173	1.4786
28	2018	34	25.2714	77.0510	924.2602	6.8245	1.5337
39	2018	34	24.0143	80.3878	898.3724	5.9847	2.4663
24	2018	34	26.3143	71.2551	934.9755	7.1816	1.4235
63	2018	34	26.0143	77.8980	939.5071	7.6143	1.2837
62	2018	34	23.6143	81.6429	872.0408	6.6490	1.0776
1	2018	34	23.6286	76.8163	872.1316	6.4908	2.1418
31	2018	35	22.1286	76.6735	845.2133	7.1122	0.9837
79	2018	35	27.9429	70.6122	964.3806	6.6816	1.6531
51	2018	35	25.1429	79.9898	934.7592	4.9061	1.4000
14	2018	35	23.1714	81.3061	894.0735	5.0786	1.3459
67	2018	35	23.4000	80.9082	899.0276	5.2153	2.4214
42	2018	35	23.2857	74.9898	872.7194	6.4786	2.2704
50	2018	35	25.6857	72.2959	898.9949	5.9500	1.6980
43	2018	35	23.2857	74.9898	872.7194	6.4786	2.2704
85	2018	35	26.7286	70.1224	907.0990	6.7929	1.2888
25	2018	35	26.2429	79.1122	970.9908	5.6510	1.2378
69	2018	35	25.7571	73.2245	935.7500	6.2939	1.4214
57	2018	35	23.1714	79.7245	884.4622	5.3418	1.7306
9	2018	35	22.2857	76.6122	852.4969	6.9204	1.9878
72	2018	35	23.3000	76.0204	875.2439	5.8765	1.6816
26	2018	35	22.7571	78.6837	865.0551	6.1163	1.2163
7	2018	35	22.4571	83.3980	857.4816	5.9255	1.1867
83	2018	35	25.8000	79.3673	940.2051	6.6888	1.1449
76	2018	35	25.7143	73.5204	917.6041	6.4357	1.4163
36	2018	35	25.0143	78.4184	925.0153	5.8469	1.4827
81	2018	35	25.1429	79.9898	934.7592	4.9061	1.4000
15	2018	35	25.6143	78.6327	915.1806	7.2735	1.3888
32	2018	35	23.2857	74.9898	872.7194	6.4786	2.2704
73	2018	35	27.9429	71.1122	957.1286	6.0602	1.2010
71	2018	35	25.0143	78.4184	925.0153	5.8469	1.4827
41	2018	35	24.1000	79.2143	872.5857	6.6653	1.2051
10	2018	35	25.7714	77.9388	958.7306	4.6867	1.0847
23	2018	35	18.5286	83.1735	776.4847	5.7969	1.5908
27	2018	35	22.4571	83.3980	857.4816	5.9255	1.1867
60	2018	35	25.1429	79.9898	934.7592	4.9061	1.4000
53	2018	35	22.2857	76.6122	852.4969	6.9204	1.9878
66	2018	35	23.1714	81.3061	894.0735	5.0786	1.3459
59	2018	35	23.1714	79.7245	884.4622	5.3418	1.7306
61	2018	35	27.9429	71.1122	957.1286	6.0602	1.2010
84	2018	35	27.9429	71.1122	957.1286	6.0602	1.2010
38	2018	35	23.1714	79.7245	884.4622	5.3418	1.7306
87	2018	35	24.2429	80.3265	896.5571	6.2439	1.5449
34	2018	35	23.1714	79.7245	884.4622	5.3418	1.7306
29	2018	35	25.7571	73.2245	935.7500	6.2939	1.4214
5	2018	35	20.8714	82.6020	831.3806	6.0194	1.2276

8	2018	35	22.2857	76.6122	852.4969	6.9204	1.9878
12	2018	35	20.8714	82.6020	831.3806	6.0194	1.2276
13	2018	35	25.8000	79.3673	940.2051	6.6888	1.1449
18	2018	35	27.8143	70.6633	958.9551	4.0735	1.2122
33	2018	35	25.6857	72.2959	898.9949	5.9500	1.6980
56	2018	35	26.2429	79.1122	970.9908	5.6510	1.2378
77	2018	35	25.6143	78.6327	915.1806	7.2735	1.3888
54	2018	35	20.8714	82.6020	831.3806	6.0194	1.2276
21	2018	35	25.6857	72.2959	898.9949	5.9500	1.6980
68	2018	35	27.9429	70.6122	964.3806	6.6816	1.6531
74	2018	35	27.9429	71.1122	957.1286	6.0602	1.2010
88	2018	35	23.2857	74.9898	872.7194	6.4786	2.2704
16	2018	35	25.7143	73.5204	917.6041	6.4357	1.4163
30	2018	35	23.1714	81.3061	894.0735	5.0786	1.3459
6	2018	35	27.9429	70.6122	964.3806	6.6816	1.6531
49	2018	35	25.7571	73.2245	935.7500	6.2939	1.4214
22	2018	35	23.2857	74.9898	872.7194	6.4786	2.2704
45	2018	35	20.4286	79.3367	816.9622	5.7224	1.0980
58	2018	35	25.7571	73.2245	935.7500	6.2939	1.4214
37	2018	35	27.9429	70.6122	964.3806	6.6816	1.6531
17	2018	35	23.4000	80.9082	899.0276	5.2153	2.4214
55	2018	35	23.3000	76.0204	875.2439	5.8765	1.6816
46	2018	35	25.7143	73.5204	917.6041	6.4357	1.4163
86	2018	35	23.0857	80.8061	864.4276	5.6306	0.8398
2	2018	35	23.0857	80.8061	864.4276	5.6306	0.8398
4	2018	35	25.6857	72.2959	898.9949	5.9500	1.6980
47	2018	35	26.7857	79.9184	953.7704	6.7418	0.4520
82	2018	35	23.2857	74.9898	872.7194	6.4786	2.2704
19	2018	35	26.2000	78.6837	955.1582	6.0663	1.1265
20	2018	35	22.2857	76.6122	852.4969	6.9204	1.9878
80	2018	35	23.2857	74.9898	872.7194	6.4786	2.2704
3	2018	35	25.8000	79.3673	940.2051	6.6888	1.1449
52	2018	35	23.4000	80.9082	899.0276	5.2153	2.4214
70	2018	35	26.7286	70.1224	907.0990	6.7929	1.2888
64	2018	35	18.5286	83.1735	776.4847	5.7969	1.5908
48	2018	35	25.6143	78.6327	915.1806	7.2735	1.3888
65	2018	35	23.4000	80.9082	899.0276	5.2153	2.4214
44	2018	35	26.7286	70.1224	907.0990	6.7929	1.2888
75	2018	35	18.5286	83.1735	776.4847	5.7969	1.5908
40	2018	35	24.5286	81.1122	940.4163	5.5245	1.2459
11	2018	35	23.3000	76.0204	875.2439	5.8765	1.6816
35	2018	35	25.1429	79.9898	934.7592	4.9061	1.4000
78	2018	35	24.2429	80.3265	896.5571	6.2439	1.5449
28	2018	35	25.0143	78.4184	925.0153	5.8469	1.4827
39	2018	35	23.4000	80.9082	899.0276	5.2153	2.4214
24	2018	35	25.7571	73.2245	935.7500	6.2939	1.4214
63	2018	35	24.5286	81.1122	940.4163	5.5245	1.2459
62	2018	35	24.1000	79.2143	872.5857	6.6653	1.2051
1	2018	35	23.2857	74.9898	872.7194	6.4786	2.2704
31	2018	36	20.1286	76.3265	845.8796	6.8571	1.0214

79	2018	36	26.4571	71.8061	965.5745	6.4582	1.6235
51	2018	36	23.9429	81.0000	935.8980	5.0622	1.2959
14	2018	36	22.2714	83.6429	895.2939	3.9582	1.6735
67	2018	36	22.3143	82.0306	900.1459	4.7286	2.2888
42	2018	36	21.7143	74.1531	873.6704	6.4061	2.3061
50	2018	36	22.3571	70.6735	899.8286	5.7929	1.6194
43	2018	36	21.7143	74.1531	873.6704	6.4061	2.3061
85	2018	36	23.8000	67.7755	907.9204	6.9500	1.4010
25	2018	36	26.5429	81.5816	972.5000	4.6347	1.2204
69	2018	36	25.0857	75.6735	936.8561	6.3153	1.4122
57	2018	36	22.0000	76.7041	885.3490	5.5235	1.9316
9	2018	36	20.2571	77.1429	853.3786	6.6367	2.0153
72	2018	36	21.6286	76.9490	876.2602	4.7816	1.7408
26	2018	36	21.9857	79.9694	865.9449	5.4357	1.4173
7	2018	36	21.3429	83.2347	858.3480	5.8122	1.3714
83	2018	36	25.7286	82.0612	941.5347	5.6480	1.0888
76	2018	36	24.5429	72.9490	918.5561	6.3092	1.3908
36	2018	36	24.0857	78.6837	926.1776	5.8776	1.4173
81	2018	36	23.9429	81.0000	935.8980	5.0622	1.2959
15	2018	36	23.5857	76.0204	916.0255	6.9684	1.5194
32	2018	36	21.7143	74.1531	873.6704	6.4061	2.3061
73	2018	36	26.5571	72.0204	958.1602	5.6061	1.1194
71	2018	36	24.0857	78.6837	926.1776	5.8776	1.4173
41	2018	36	21.9429	75.8367	873.3888	6.7653	1.4531
10	2018	36	25.2143	79.9694	960.0592	4.6418	1.1531
23	2018	36	15.9571	83.5510	777.0327	5.3112	1.5939
27	2018	36	21.3429	83.2347	858.3480	5.8122	1.3714
60	2018	36	23.9429	81.0000	935.8980	5.0622	1.2959
53	2018	36	20.2571	77.1429	853.3786	6.6367	2.0153
66	2018	36	22.2714	83.6429	895.2939	3.9582	1.6735
59	2018	36	22.0000	76.7041	885.3490	5.5235	1.9316
61	2018	36	26.5571	72.0204	958.1602	5.6061	1.1194
84	2018	36	26.5571	72.0204	958.1602	5.6061	1.1194
38	2018	36	22.0000	76.7041	885.3490	5.5235	1.9316
87	2018	36	23.2000	80.4286	897.6153	5.8388	1.5327
34	2018	36	22.0000	76.7041	885.3490	5.5235	1.9316
29	2018	36	25.0857	75.6735	936.8561	6.3153	1.4122
5	2018	36	19.6571	82.4796	832.1133	6.2347	1.2898
8	2018	36	20.2571	77.1429	853.3786	6.6367	2.0153
12	2018	36	19.6571	82.4796	832.1133	6.2347	1.2898
13	2018	36	25.7286	82.0612	941.5347	5.6480	1.0888
18	2018	36	25.8857	73.8776	960.1102	3.5663	1.1663
33	2018	36	22.3571	70.6735	899.8286	5.7929	1.6194
56	2018	36	26.5429	81.5816	972.5000	4.6347	1.2204
77	2018	36	23.5857	76.0204	916.0255	6.9684	1.5194
54	2018	36	19.6571	82.4796	832.1133	6.2347	1.2898
21	2018	36	22.3571	70.6735	899.8286	5.7929	1.6194
68	2018	36	26.4571	71.8061	965.5745	6.4582	1.6235
74	2018	36	26.5571	72.0204	958.1602	5.6061	1.1194
88	2018	36	21.7143	74.1531	873.6704	6.4061	2.3061

16	2018	36	24.5429	72.9490	918.5561	6.3092	1.3908
30	2018	36	22.2714	83.6429	895.2939	3.9582	1.6735
6	2018	36	26.4571	71.8061	965.5745	6.4582	1.6235
49	2018	36	25.0857	75.6735	936.8561	6.3153	1.4122
22	2018	36	21.7143	74.1531	873.6704	6.4061	2.3061
45	2018	36	19.3143	78.4796	817.6276	5.8653	1.2969
58	2018	36	25.0857	75.6735	936.8561	6.3153	1.4122
37	2018	36	26.4571	71.8061	965.5745	6.4582	1.6235
17	2018	36	22.3143	82.0306	900.1459	4.7286	2.2888
55	2018	36	21.6286	76.9490	876.2602	4.7816	1.7408
46	2018	36	24.5429	72.9490	918.5561	6.3092	1.3908
86	2018	36	21.0571	79.8571	865.2041	4.8786	1.0490
2	2018	36	21.0571	79.8571	865.2041	4.8786	1.0490
4	2018	36	22.3571	70.6735	899.8286	5.7929	1.6194
47	2018	36	26.3571	80.2551	955.1459	5.9786	0.4408
82	2018	36	21.7143	74.1531	873.6704	6.4061	2.3061
19	2018	36	26.6429	81.6633	956.7082	5.0643	0.9878
20	2018	36	20.2571	77.1429	853.3786	6.6367	2.0153
80	2018	36	21.7143	74.1531	873.6704	6.4061	2.3061
3	2018	36	25.7286	82.0612	941.5347	5.6480	1.0888
52	2018	36	22.3143	82.0306	900.1459	4.7286	2.2888
70	2018	36	23.8000	67.7755	907.9204	6.9500	1.4010
64	2018	36	15.9571	83.5510	777.0327	5.3112	1.5939
48	2018	36	23.5857	76.0204	916.0255	6.9684	1.5194
65	2018	36	22.3143	82.0306	900.1459	4.7286	2.2888
44	2018	36	23.8000	67.7755	907.9204	6.9500	1.4010
75	2018	36	15.9571	83.5510	777.0327	5.3112	1.5939
40	2018	36	24.5714	81.7245	941.8061	5.2735	1.3204
11	2018	36	21.6286	76.9490	876.2602	4.7816	1.7408
35	2018	36	23.9429	81.0000	935.8980	5.0622	1.2959
78	2018	36	23.2000	80.4286	897.6153	5.8388	1.5327
28	2018	36	24.0857	78.6837	926.1776	5.8776	1.4173
39	2018	36	22.3143	82.0306	900.1459	4.7286	2.2888
24	2018	36	25.0857	75.6735	936.8561	6.3153	1.4122
63	2018	36	24.5714	81.7245	941.8061	5.2735	1.3204
62	2018	36	21.9429	75.8367	873.3888	6.7653	1.4531
1	2018	36	21.7143	74.1531	873.6704	6.4061	2.3061
31	2018	37	17.0143	81.3469	848.2296	3.5418	1.0510
79	2018	37	22.0714	74.7653	969.6337	5.0990	1.4745
51	2018	37	20.2857	81.8776	939.5469	4.5122	1.3143
14	2018	37	20.2571	82.8265	898.3347	3.8367	1.7061
67	2018	37	19.3429	82.8061	903.2490	4.3439	2.1663
42	2018	37	18.8571	77.2143	876.3796	4.7643	2.2806
50	2018	37	18.3857	77.2041	903.0194	2.9969	1.5531
43	2018	37	18.8571	77.2143	876.3796	4.7643	2.2806
85	2018	37	18.8286	75.1837	911.2602	4.2939	1.5469
25	2018	37	24.6429	77.3061	976.3296	4.6214	1.2980
69	2018	37	21.1000	76.1939	940.4296	4.6929	1.3684
57	2018	37	17.8286	80.0306	888.2296	4.0541	2.0837
9	2018	37	17.8286	80.9592	855.8633	4.4122	1.9367

72	2018	37	19.4714	79.8469	879.0102	3.5327	1.5041
26	2018	37	19.5857	82.7857	868.4776	3.6980	1.5622
7	2018	37	18.5857	85.3571	860.8398	4.2622	1.3337
83	2018	37	23.9143	79.9388	944.8684	4.9357	1.1469
76	2018	37	19.5000	76.0204	921.9337	3.9388	1.3765
36	2018	37	20.8000	78.7245	929.5765	5.3847	1.4612
81	2018	37	20.2857	81.8776	939.5469	4.5122	1.3143
15	2018	37	18.3429	80.4796	919.4561	4.1949	1.6010
32	2018	37	18.8571	77.2143	876.3796	4.7643	2.2806
73	2018	37	21.5571	75.4694	962.1490	3.9592	1.0224
71	2018	37	20.8000	78.7245	929.5765	5.3847	1.4612
41	2018	37	18.2286	80.0510	876.0918	4.2827	1.4245
10	2018	37	22.1286	78.4592	963.9786	4.0265	1.1490
23	2018	37	14.0571	90.8367	778.5929	2.7990	1.8173
27	2018	37	18.5857	85.3571	860.8398	4.2622	1.3337
60	2018	37	20.2857	81.8776	939.5469	4.5122	1.3143
53	2018	37	17.8286	80.9592	855.8633	4.4122	1.9367
66	2018	37	20.2571	82.8265	898.3347	3.8367	1.7061
59	2018	37	17.8286	80.0306	888.2296	4.0541	2.0837
61	2018	37	21.5571	75.4694	962.1490	3.9592	1.0224
84	2018	37	21.5571	75.4694	962.1490	3.9592	1.0224
38	2018	37	17.8286	80.0306	888.2296	4.0541	2.0837
87	2018	37	20.7714	80.9388	900.5755	5.0204	1.6184
34	2018	37	17.8286	80.0306	888.2296	4.0541	2.0837
29	2018	37	21.1000	76.1939	940.4296	4.6929	1.3684
5	2018	37	16.8857	85.0612	834.3041	3.6541	1.3388
8	2018	37	17.8286	80.9592	855.8633	4.4122	1.9367
12	2018	37	16.8857	85.0612	834.3041	3.6541	1.3388
13	2018	37	23.9143	79.9388	944.8684	4.9357	1.1469
18	2018	37	21.5286	78.8673	964.1296	2.7061	1.1449
33	2018	37	18.3857	77.2041	903.0194	2.9969	1.5531
56	2018	37	24.6429	77.3061	976.3296	4.6214	1.2980
77	2018	37	18.3429	80.4796	919.4561	4.1949	1.6010
54	2018	37	16.8857	85.0612	834.3041	3.6541	1.3388
21	2018	37	18.3857	77.2041	903.0194	2.9969	1.5531
68	2018	37	22.0714	74.7653	969.6337	5.0990	1.4745
74	2018	37	21.5571	75.4694	962.1490	3.9592	1.0224
88	2018	37	18.8571	77.2143	876.3796	4.7643	2.2806
16	2018	37	19.5000	76.0204	921.9337	3.9388	1.3765
30	2018	37	20.2571	82.8265	898.3347	3.8367	1.7061
6	2018	37	22.0714	74.7653	969.6337	5.0990	1.4745
49	2018	37	21.1000	76.1939	940.4296	4.6929	1.3684
22	2018	37	18.8571	77.2143	876.3796	4.7643	2.2806
45	2018	37	17.4571	81.4694	819.6000	3.6531	1.3776
58	2018	37	21.1000	76.1939	940.4296	4.6929	1.3684
37	2018	37	22.0714	74.7653	969.6337	5.0990	1.4745
17	2018	37	19.3429	82.8061	903.2490	4.3439	2.1663
55	2018	37	19.4714	79.8469	879.0102	3.5327	1.5041
46	2018	37	19.5000	76.0204	921.9337	3.9388	1.3765
86	2018	37	18.3286	83.6837	867.7714	2.7663	1.1102

2	2018	37	18.3286	83.6837	867.7714	2.7663	1.1102
4	2018	37	18.3857	77.2041	903.0194	2.9969	1.5531
47	2018	37	25.2714	76.4592	958.5796	5.9082	0.5480
82	2018	37	18.8571	77.2143	876.3796	4.7643	2.2806
19	2018	37	24.9571	76.3367	960.2755	5.1612	1.0327
20	2018	37	17.8286	80.9592	855.8633	4.4122	1.9367
80	2018	37	18.8571	77.2143	876.3796	4.7643	2.2806
3	2018	37	23.9143	79.9388	944.8684	4.9357	1.1469
52	2018	37	19.3429	82.8061	903.2490	4.3439	2.1663
70	2018	37	18.8286	75.1837	911.2602	4.2939	1.5469
64	2018	37	14.0571	90.8367	778.5929	2.7990	1.8173
48	2018	37	18.3429	80.4796	919.4561	4.1949	1.6010
65	2018	37	19.3429	82.8061	903.2490	4.3439	2.1663
44	2018	37	18.8286	75.1837	911.2602	4.2939	1.5469
75	2018	37	14.0571	90.8367	778.5929	2.7990	1.8173
40	2018	37	21.7000	79.2041	945.4663	4.7694	1.3500
11	2018	37	19.4714	79.8469	879.0102	3.5327	1.5041
35	2018	37	20.2857	81.8776	939.5469	4.5122	1.3143
78	2018	37	20.7714	80.9388	900.5755	5.0204	1.6184
28	2018	37	20.8000	78.7245	929.5765	5.3847	1.4612
39	2018	37	19.3429	82.8061	903.2490	4.3439	2.1663
24	2018	37	21.1000	76.1939	940.4296	4.6929	1.3684
63	2018	37	21.7000	79.2041	945.4663	4.7694	1.3500
62	2018	37	18.2286	80.0510	876.0918	4.2827	1.4245
1	2018	37	18.8571	77.2143	876.3796	4.7643	2.2806
31	2018	38	18.0571	86.3673	850.1398	1.7408	0.9959
79	2018	38	23.7857	78.6020	972.6378	3.5592	1.7286
51	2018	38	22.7429	84.1837	942.0408	2.9296	1.5480
14	2018	38	22.0143	84.6531	900.0531	3.2694	1.7990
67	2018	38	21.6286	86.1837	905.2051	2.7214	2.6531
42	2018	38	19.8857	83.7959	878.2112	2.6418	2.3694
50	2018	38	20.6429	83.6122	905.4633	1.0408	1.4673
43	2018	38	19.8857	83.7959	878.2112	2.6418	2.3694
85	2018	38	20.9714	84.2245	913.9214	1.7867	1.4500
25	2018	38	26.0571	77.8265	978.5010	4.0204	1.2837
69	2018	38	23.3571	80.0714	942.8918	1.8214	1.2735
57	2018	38	20.0857	87.0714	890.3173	2.2449	2.2306
9	2018	38	19.3429	84.7959	857.5408	2.3214	2.0908
72	2018	38	20.6857	83.3469	880.6786	2.7276	1.6571
26	2018	38	21.1857	84.9184	870.0480	3.1520	1.6265
7	2018	38	20.4143	88.9490	862.4510	2.8929	1.2204
83	2018	38	24.9571	80.0000	946.6592	4.5694	1.3133
76	2018	38	21.6857	82.0816	924.4296	1.6092	1.3633
36	2018	38	22.9429	81.5204	931.7949	3.2122	1.5684
81	2018	38	22.7429	84.1837	942.0408	2.9296	1.5480
15	2018	38	21.2143	87.3163	922.1194	1.4571	1.4847
32	2018	38	19.8857	83.7959	878.2112	2.6418	2.3694
73	2018	38	23.8571	80.5204	965.0918	2.0357	0.8806
71	2018	38	22.9429	81.5204	931.7949	3.2122	1.5684
41	2018	38	20.0000	87.6327	878.1327	2.2143	1.1214

10	2018	38	23.7857	79.9286	966.6214	2.5051	1.1531
23	2018	38	15.0857	94.5918	779.8786	1.4673	1.9990
27	2018	38	20.4143	88.9490	862.4510	2.8929	1.2204
60	2018	38	22.7429	84.1837	942.0408	2.9296	1.5480
53	2018	38	19.3429	84.7959	857.5408	2.3214	2.0908
66	2018	38	22.0143	84.6531	900.0531	3.2694	1.7990
59	2018	38	20.0857	87.0714	890.3173	2.2449	2.2306
61	2018	38	23.8571	80.5204	965.0918	2.0357	0.8806
84	2018	38	23.8571	80.5204	965.0918	2.0357	0.8806
38	2018	38	20.0857	87.0714	890.3173	2.2449	2.2306
87	2018	38	21.6857	85.5510	902.4459	3.6480	1.7408
34	2018	38	20.0857	87.0714	890.3173	2.2449	2.2306
29	2018	38	23.3571	80.0714	942.8918	1.8214	1.2735
5	2018	38	18.7857	88.2551	835.8327	2.2857	1.2724
8	2018	38	19.3429	84.7959	857.5408	2.3214	2.0908
12	2018	38	18.7857	88.2551	835.8327	2.2857	1.2724
13	2018	38	24.9571	80.0000	946.6592	4.5694	1.3133
18	2018	38	23.1000	83.0714	967.1490	1.6388	1.0622
33	2018	38	20.6429	83.6122	905.4633	1.0408	1.4673
56	2018	38	26.0571	77.8265	978.5010	4.0204	1.2837
77	2018	38	21.2143	87.3163	922.1194	1.4571	1.4847
54	2018	38	18.7857	88.2551	835.8327	2.2857	1.2724
21	2018	38	20.6429	83.6122	905.4633	1.0408	1.4673
68	2018	38	23.7857	78.6020	972.6378	3.5592	1.7286
74	2018	38	23.8571	80.5204	965.0918	2.0357	0.8806
88	2018	38	19.8857	83.7959	878.2112	2.6418	2.3694
16	2018	38	21.6857	82.0816	924.4296	1.6092	1.3633
30	2018	38	22.0143	84.6531	900.0531	3.2694	1.7990
6	2018	38	23.7857	78.6020	972.6378	3.5592	1.7286
49	2018	38	23.3571	80.0714	942.8918	1.8214	1.2735
22	2018	38	19.8857	83.7959	878.2112	2.6418	2.3694
45	2018	38	18.6286	83.5306	821.0133	2.3531	1.4490
58	2018	38	23.3571	80.0714	942.8918	1.8214	1.2735
37	2018	38	23.7857	78.6020	972.6378	3.5592	1.7286
17	2018	38	21.6286	86.1837	905.2051	2.7214	2.6531
55	2018	38	20.6857	83.3469	880.6786	2.7276	1.6571
46	2018	38	21.6857	82.0816	924.4296	1.6092	1.3633
86	2018	38	19.7429	88.1020	869.6776	1.3724	1.0347
2	2018	38	19.7429	88.1020	869.6776	1.3724	1.0347
4	2018	38	20.6429	83.6122	905.4633	1.0408	1.4673
47	2018	38	25.1571	77.7143	960.4867	4.7378	0.5541
82	2018	38	19.8857	83.7959	878.2112	2.6418	2.3694
19	2018	38	25.9286	76.2959	962.0918	4.1694	1.0735
20	2018	38	19.3429	84.7959	857.5408	2.3214	2.0908
80	2018	38	19.8857	83.7959	878.2112	2.6418	2.3694
3	2018	38	24.9571	80.0000	946.6592	4.5694	1.3133
52	2018	38	21.6286	86.1837	905.2051	2.7214	2.6531
70	2018	38	20.9714	84.2245	913.9214	1.7867	1.4500
64	2018	38	15.0857	94.5918	779.8786	1.4673	1.9990
48	2018	38	21.2143	87.3163	922.1194	1.4571	1.4847

65	2018	38	21.6286	86.1837	905.2051	2.7214	2.6531
44	2018	38	20.9714	84.2245	913.9214	1.7867	1.4500
75	2018	38	15.0857	94.5918	779.8786	1.4673	1.9990
40	2018	38	23.8286	81.6939	947.6786	3.6510	1.5102
11	2018	38	20.6857	83.3469	880.6786	2.7276	1.6571
35	2018	38	22.7429	84.1837	942.0408	2.9296	1.5480
78	2018	38	21.6857	85.5510	902.4459	3.6480	1.7408
28	2018	38	22.9429	81.5204	931.7949	3.2122	1.5684
39	2018	38	21.6286	86.1837	905.2051	2.7214	2.6531
24	2018	38	23.3571	80.0714	942.8918	1.8214	1.2735
63	2018	38	23.8286	81.6939	947.6786	3.6510	1.5102
62	2018	38	20.0000	87.6327	878.1327	2.2143	1.1214
1	2018	38	19.8857	83.7959	878.2112	2.6418	2.3694
31	2018	39	17.0286	87.6633	850.1184	1.7378	0.9041
79	2018	39	20.0143	82.9898	972.9306	2.6235	1.8643
51	2018	39	18.8714	86.5714	942.1633	2.0980	1.7327
14	2018	39	19.3857	87.6633	900.2173	2.6786	2.0990
67	2018	39	18.8857	87.7245	905.2643	1.7398	2.8408
42	2018	39	18.4000	86.3571	878.3184	2.1224	2.2857
50	2018	39	18.2571	84.7653	905.3204	0.9888	1.5010
43	2018	39	18.4000	86.3571	878.3184	2.1224	2.2857
85	2018	39	19.0857	86.1020	913.7418	1.7469	1.3541
25	2018	39	22.2714	82.6224	978.6867	3.7510	1.1551
69	2018	39	20.6143	82.7347	942.8367	0.9459	1.3918
57	2018	39	18.2143	87.2755	890.2673	2.2173	2.5163
9	2018	39	17.6000	85.4490	857.6224	2.1816	2.1163
72	2018	39	19.4286	83.8163	880.7939	2.8612	1.7459
26	2018	39	19.9286	85.2755	870.1612	4.2796	1.7541
7	2018	39	19.2714	89.5612	862.5276	3.3684	1.3571
83	2018	39	23.3571	81.6122	946.7143	5.3694	1.3082
76	2018	39	18.6714	85.9388	924.3867	1.2755	1.4051
36	2018	39	20.2000	83.1020	931.8500	2.5224	1.5776
81	2018	39	18.8714	86.5714	942.1633	2.0980	1.7327
15	2018	39	17.6714	89.6837	922.0602	0.9245	1.5398
32	2018	39	18.4000	86.3571	878.3184	2.1224	2.2857
73	2018	39	20.6429	84.1531	965.1429	1.2449	0.8051
71	2018	39	20.2000	83.1020	931.8500	2.5224	1.5776
41	2018	39	18.4571	89.6837	878.0622	2.2857	1.2296
10	2018	39	20.0714	83.8673	966.8592	2.0592	1.1000
23	2018	39	13.8571	93.9388	780.0122	1.6663	1.9612
27	2018	39	19.2714	89.5612	862.5276	3.3684	1.3571
60	2018	39	18.8714	86.5714	942.1633	2.0980	1.7327
53	2018	39	17.6000	85.4490	857.6224	2.1816	2.1163
66	2018	39	19.3857	87.6633	900.2173	2.6786	2.0990
59	2018	39	18.2143	87.2755	890.2673	2.2173	2.5163
61	2018	39	20.6429	84.1531	965.1429	1.2449	0.8051
84	2018	39	20.6429	84.1531	965.1429	1.2449	0.8051
38	2018	39	18.2143	87.2755	890.2673	2.2173	2.5163
87	2018	39	20.5000	87.6939	902.5367	3.1735	1.7571
34	2018	39	18.2143	87.2755	890.2673	2.2173	2.5163

29	2018	39	20.6143	82.7347	942.8367	0.9459	1.3918
5	2018	39	17.6571	88.1735	835.8918	3.2806	1.2643
8	2018	39	17.6000	85.4490	857.6224	2.1816	2.1163
12	2018	39	17.6571	88.1735	835.8918	3.2806	1.2643
13	2018	39	23.3571	81.6122	946.7143	5.3694	1.3082
18	2018	39	19.3143	89.8367	967.4306	1.0500	0.8816
33	2018	39	18.2571	84.7653	905.3204	0.9888	1.5010
56	2018	39	22.2714	82.6224	978.6867	3.7510	1.1551
77	2018	39	17.6714	89.6837	922.0602	0.9245	1.5398
54	2018	39	17.6571	88.1735	835.8918	3.2806	1.2643
21	2018	39	18.2571	84.7653	905.3204	0.9888	1.5010
68	2018	39	20.0143	82.9898	972.9306	2.6235	1.8643
74	2018	39	20.6429	84.1531	965.1429	1.2449	0.8051
88	2018	39	18.4000	86.3571	878.3184	2.1224	2.2857
16	2018	39	18.6714	85.9388	924.3867	1.2755	1.4051
30	2018	39	19.3857	87.6633	900.2173	2.6786	2.0990
6	2018	39	20.0143	82.9898	972.9306	2.6235	1.8643
49	2018	39	20.6143	82.7347	942.8367	0.9459	1.3918
22	2018	39	18.4000	86.3571	878.3184	2.1224	2.2857
45	2018	39	17.0857	83.1633	821.1378	2.8806	1.6061
58	2018	39	20.6143	82.7347	942.8367	0.9459	1.3918
37	2018	39	20.0143	82.9898	972.9306	2.6235	1.8643
17	2018	39	18.8857	87.7245	905.2643	1.7398	2.8408
55	2018	39	19.4286	83.8163	880.7939	2.8612	1.7459
46	2018	39	18.6714	85.9388	924.3867	1.2755	1.4051
86	2018	39	18.4857	88.4490	869.6469	1.1337	1.1418
2	2018	39	18.4857	88.4490	869.6469	1.1337	1.1418
4	2018	39	18.2571	84.7653	905.3204	0.9888	1.5010
47	2018	39	23.3286	82.9694	960.6061	4.1510	0.4010
82	2018	39	18.4000	86.3571	878.3184	2.1224	2.2857
19	2018	39	23.0000	80.8776	962.2918	3.9520	1.0745
20	2018	39	17.6000	85.4490	857.6224	2.1816	2.1163
80	2018	39	18.4000	86.3571	878.3184	2.1224	2.2857
3	2018	39	23.3571	81.6122	946.7143	5.3694	1.3082
52	2018	39	18.8857	87.7245	905.2643	1.7398	2.8408
70	2018	39	19.0857	86.1020	913.7418	1.7469	1.3541
64	2018	39	13.8571	93.9388	780.0122	1.6663	1.9612
48	2018	39	17.6714	89.6837	922.0602	0.9245	1.5398
65	2018	39	18.8857	87.7245	905.2643	1.7398	2.8408
44	2018	39	19.0857	86.1020	913.7418	1.7469	1.3541
75	2018	39	13.8571	93.9388	780.0122	1.6663	1.9612
40	2018	39	19.6857	84.8571	947.9071	3.7102	1.6398
11	2018	39	19.4286	83.8163	880.7939	2.8612	1.7459
35	2018	39	18.8714	86.5714	942.1633	2.0980	1.7327
78	2018	39	20.5000	87.6939	902.5367	3.1735	1.7571
28	2018	39	20.2000	83.1020	931.8500	2.5224	1.5776
39	2018	39	18.8857	87.7245	905.2643	1.7398	2.8408
24	2018	39	20.6143	82.7347	942.8367	0.9459	1.3918
63	2018	39	19.6857	84.8571	947.9071	3.7102	1.6398
62	2018	39	18.4571	89.6837	878.0622	2.2857	1.2296

1	2018	39	18.4000	86.3571	878.3184	2.1224	2.2857
31	2018	40	12.4429	86.9796	851.8245	0.6816	0.8449
79	2018	40	20.0000	79.1224	975.4918	2.1429	1.3357
51	2018	40	18.7000	81.3367	944.5949	2.3653	1.6316
14	2018	40	18.2857	78.1939	902.3143	2.7949	1.9265
67	2018	40	17.5143	79.4388	907.4296	1.8612	2.8510
42	2018	40	15.3000	80.8980	880.2959	1.6551	2.1622
50	2018	40	14.7143	84.5000	907.5745	0.3102	1.3571
43	2018	40	15.3000	80.8980	880.2959	1.6551	2.1622
85	2018	40	15.0429	87.7653	916.0561	0.7714	1.3449
25	2018	40	21.7714	78.6224	981.5673	4.2867	1.0867
69	2018	40	18.8857	78.1939	945.4051	1.0357	1.6133
57	2018	40	14.2857	85.9286	892.4082	1.4816	2.1153
9	2018	40	13.9857	83.1327	859.4031	1.7888	1.8102
72	2018	40	16.5571	77.8265	882.7878	2.1980	1.6194
26	2018	40	16.4857	83.4898	872.0551	2.7020	1.3827
7	2018	40	15.6286	86.3878	864.3408	2.4490	1.2612
83	2018	40	21.4286	78.5204	949.3316	4.0816	1.1429
76	2018	40	16.6143	84.1122	926.8143	0.6010	1.2745
36	2018	40	19.3286	74.6939	934.2051	2.8684	1.5898
81	2018	40	18.7000	81.3367	944.5949	2.3653	1.6316
15	2018	40	15.7429	89.8061	924.4694	0.5531	1.4163
32	2018	40	15.3000	80.8980	880.2959	1.6551	2.1622
73	2018	40	19.5429	81.3980	967.8663	0.9633	0.7592
71	2018	40	19.3286	74.6939	934.2051	2.8684	1.5898
41	2018	40	14.2857	88.9592	880.0888	1.1878	1.2184
10	2018	40	19.5429	78.7755	969.4224	2.6357	1.0918
23	2018	40	9.6286	95.1531	780.9408	0.7092	1.5735
27	2018	40	15.6286	86.3878	864.3408	2.4490	1.2612
60	2018	40	18.7000	81.3367	944.5949	2.3653	1.6316
53	2018	40	13.9857	83.1327	859.4031	1.7888	1.8102
66	2018	40	18.2857	78.1939	902.3143	2.7949	1.9265
59	2018	40	14.2857	85.9286	892.4082	1.4816	2.1153
61	2018	40	19.5429	81.3980	967.8663	0.9633	0.7592
84	2018	40	19.5429	81.3980	967.8663	0.9633	0.7592
38	2018	40	14.2857	85.9286	892.4082	1.4816	2.1153
87	2018	40	17.8429	79.5408	904.7224	2.2755	1.8806
34	2018	40	14.2857	85.9286	892.4082	1.4816	2.1153
29	2018	40	18.8857	78.1939	945.4051	1.0357	1.6133
5	2018	40	13.1286	86.6735	837.4469	2.0878	1.2898
8	2018	40	13.9857	83.1327	859.4031	1.7888	1.8102
12	2018	40	13.1286	86.6735	837.4469	2.0878	1.2898
13	2018	40	21.4286	78.5204	949.3316	4.0816	1.1429
18	2018	40	19.5714	85.9796	969.9469	1.2622	0.7265
33	2018	40	14.7143	84.5000	907.5745	0.3102	1.3571
56	2018	40	21.7714	78.6224	981.5673	4.2867	1.0867
77	2018	40	15.7429	89.8061	924.4694	0.5531	1.4163
54	2018	40	13.1286	86.6735	837.4469	2.0878	1.2898
21	2018	40	14.7143	84.5000	907.5745	0.3102	1.3571
68	2018	40	20.0000	79.1224	975.4918	2.1429	1.3357

74	2018	40	19.5429	81.3980	967.8663	0.9633	0.7592
88	2018	40	15.3000	80.8980	880.2959	1.6551	2.1622
16	2018	40	16.6143	84.1122	926.8143	0.6010	1.2745
30	2018	40	18.2857	78.1939	902.3143	2.7949	1.9265
6	2018	40	20.0000	79.1224	975.4918	2.1429	1.3357
49	2018	40	18.8857	78.1939	945.4051	1.0357	1.6133
22	2018	40	15.3000	80.8980	880.2959	1.6551	2.1622
45	2018	40	13.6571	84.6939	822.5459	1.5224	1.4112
58	2018	40	18.8857	78.1939	945.4051	1.0357	1.6133
37	2018	40	20.0000	79.1224	975.4918	2.1429	1.3357
17	2018	40	17.5143	79.4388	907.4296	1.8612	2.8510
55	2018	40	16.5571	77.8265	882.7878	2.1980	1.6194
46	2018	40	16.6143	84.1122	926.8143	0.6010	1.2745
86	2018	40	14.0571	87.0306	871.5551	0.4286	0.9827
2	2018	40	14.0571	87.0306	871.5551	0.4286	0.9827
4	2018	40	14.7143	84.5000	907.5745	0.3102	1.3571
47	2018	40	21.5714	79.4898	963.3786	3.4531	0.3204
82	2018	40	15.3000	80.8980	880.2959	1.6551	2.1622
19	2018	40	22.7000	73.1122	964.9990	4.8367	1.0582
20	2018	40	13.9857	83.1327	859.4031	1.7888	1.8102
80	2018	40	15.3000	80.8980	880.2959	1.6551	2.1622
3	2018	40	21.4286	78.5204	949.3316	4.0816	1.1429
52	2018	40	17.5143	79.4388	907.4296	1.8612	2.8510
70	2018	40	15.0429	87.7653	916.0561	0.7714	1.3449
64	2018	40	9.6286	95.1531	780.9408	0.7092	1.5735
48	2018	40	15.7429	89.8061	924.4694	0.5531	1.4163
65	2018	40	17.5143	79.4388	907.4296	1.8612	2.8510
44	2018	40	15.0429	87.7653	916.0561	0.7714	1.3449
75	2018	40	9.6286	95.1531	780.9408	0.7092	1.5735
40	2018	40	19.4571	78.8367	950.3673	4.3837	1.4704
11	2018	40	16.5571	77.8265	882.7878	2.1980	1.6194
35	2018	40	18.7000	81.3367	944.5949	2.3653	1.6316
78	2018	40	17.8429	79.5408	904.7224	2.2755	1.8806
28	2018	40	19.3286	74.6939	934.2051	2.8684	1.5898
39	2018	40	17.5143	79.4388	907.4296	1.8612	2.8510
24	2018	40	18.8857	78.1939	945.4051	1.0357	1.6133
63	2018	40	19.4571	78.8367	950.3673	4.3837	1.4704
62	2018	40	14.2857	88.9592	880.0888	1.1878	1.2184
1	2018	40	15.3000	80.8980	880.2959	1.6551	2.1622
31	2018	41	11.2000	89.2245	853.4969	0.1449	0.8449
79	2018	41	15.8429	74.6224	977.9653	2.7878	1.0551
51	2018	41	14.2000	77.7347	946.8908	2.8408	1.3612
14	2018	41	14.3714	72.4592	904.1439	2.3347	1.6255
67	2018	41	13.4714	75.9388	909.4398	1.6816	2.7367
42	2018	41	12.5143	80.3571	882.0745	1.0827	2.1041
50	2018	41	13.3857	84.7449	909.9163	0.2449	1.1112
43	2018	41	12.5143	80.3571	882.0745	1.0827	2.1041
85	2018	41	13.9714	88.7245	918.5653	0.2776	1.2694
25	2018	41	18.0000	74.8878	984.1867	4.3102	1.1337
69	2018	41	15.1571	76.2857	947.9745	1.1357	1.4867

57	2018	41	12.2571	89.1122	894.5153	0.7469	1.6327
9	2018	41	12.4143	84.3673	860.9480	0.6980	1.6847
72	2018	41	14.1857	77.4082	884.5071	0.9531	1.5622
26	2018	41	14.7000	86.2551	873.6837	1.0143	1.2276
7	2018	41	14.0857	89.1837	865.9020	1.0204	1.1765
83	2018	41	18.6143	78.7143	951.7378	2.0000	1.0551
76	2018	41	13.6857	83.3163	929.2133	0.4020	1.1255
36	2018	41	14.8857	70.6020	936.4898	2.6122	1.5704
81	2018	41	14.2000	77.7347	946.8908	2.8408	1.3612
15	2018	41	13.4143	90.0306	926.8245	0.4378	1.0969
32	2018	41	12.5143	80.3571	882.0745	1.0827	2.1041
73	2018	41	16.0286	79.4592	970.5541	1.3367	0.7388
71	2018	41	14.8857	70.6020	936.4898	2.6122	1.5704
41	2018	41	12.4286	90.5204	882.1112	0.3143	0.9224
10	2018	41	15.5143	73.3673	971.8643	3.2286	1.1316
23	2018	41	8.5571	96.3673	781.6143	0.5459	1.4173
27	2018	41	14.0857	89.1837	865.9020	1.0204	1.1765
60	2018	41	14.2000	77.7347	946.8908	2.8408	1.3612
53	2018	41	12.4143	84.3673	860.9480	0.6980	1.6847
66	2018	41	14.3714	72.4592	904.1439	2.3347	1.6255
59	2018	41	12.2571	89.1122	894.5153	0.7469	1.6327
61	2018	41	16.0286	79.4592	970.5541	1.3367	0.7388
84	2018	41	16.0286	79.4592	970.5541	1.3367	0.7388
38	2018	41	12.2571	89.1122	894.5153	0.7469	1.6327
87	2018	41	14.9429	78.3265	906.7276	1.2122	1.7827
34	2018	41	12.2571	89.1122	894.5153	0.7469	1.6327
29	2018	41	15.1571	76.2857	947.9745	1.1357	1.4867
5	2018	41	12.3714	89.9796	838.7367	0.5908	1.2112
8	2018	41	12.4143	84.3673	860.9480	0.6980	1.6847
12	2018	41	12.3714	89.9796	838.7367	0.5908	1.2112
13	2018	41	18.6143	78.7143	951.7378	2.0000	1.0551
18	2018	41	15.5857	78.7857	972.3653	2.1837	0.7541
33	2018	41	13.3857	84.7449	909.9163	0.2449	1.1112
56	2018	41	18.0000	74.8878	984.1867	4.3102	1.1337
77	2018	41	13.4143	90.0306	926.8245	0.4378	1.0969
54	2018	41	12.3714	89.9796	838.7367	0.5908	1.2112
21	2018	41	13.3857	84.7449	909.9163	0.2449	1.1112
68	2018	41	15.8429	74.6224	977.9653	2.7878	1.0551
74	2018	41	16.0286	79.4592	970.5541	1.3367	0.7388
88	2018	41	12.5143	80.3571	882.0745	1.0827	2.1041
16	2018	41	13.6857	83.3163	929.2133	0.4020	1.1255
30	2018	41	14.3714	72.4592	904.1439	2.3347	1.6255
6	2018	41	15.8429	74.6224	977.9653	2.7878	1.0551
49	2018	41	15.1571	76.2857	947.9745	1.1357	1.4867
22	2018	41	12.5143	80.3571	882.0745	1.0827	2.1041
45	2018	41	12.8000	87.6224	823.6082	0.9980	1.2867
58	2018	41	15.1571	76.2857	947.9745	1.1357	1.4867
37	2018	41	15.8429	74.6224	977.9653	2.7878	1.0551
17	2018	41	13.4714	75.9388	909.4398	1.6816	2.7367
55	2018	41	14.1857	77.4082	884.5071	0.9531	1.5622

46	2018	41	13.6857	83.3163	929.2133	0.4020	1.1255
86	2018	41	12.8286	89.3571	873.3857	0.0510	0.7531
2	2018	41	12.8286	89.3571	873.3857	0.0510	0.7531
4	2018	41	13.3857	84.7449	909.9163	0.2449	1.1112
47	2018	41	18.5000	77.8163	965.9847	1.8388	0.3408
82	2018	41	12.5143	80.3571	882.0745	1.0827	2.1041
19	2018	41	18.4714	68.1020	967.4204	4.5469	1.0235
20	2018	41	12.4143	84.3673	860.9480	0.6980	1.6847
80	2018	41	12.5143	80.3571	882.0745	1.0827	2.1041
3	2018	41	18.6143	78.7143	951.7378	2.0000	1.0551
52	2018	41	13.4714	75.9388	909.4398	1.6816	2.7367
70	2018	41	13.9714	88.7245	918.5653	0.2776	1.2694
64	2018	41	8.5571	96.3673	781.6143	0.5459	1.4173
48	2018	41	13.4143	90.0306	926.8245	0.4378	1.0969
65	2018	41	13.4714	75.9388	909.4398	1.6816	2.7367
44	2018	41	13.9714	88.7245	918.5653	0.2776	1.2694
75	2018	41	8.5571	96.3673	781.6143	0.5459	1.4173
40	2018	41	15.3000	73.3673	952.5939	5.1031	1.2541
11	2018	41	14.1857	77.4082	884.5071	0.9531	1.5622
35	2018	41	14.2000	77.7347	946.8908	2.8408	1.3612
78	2018	41	14.9429	78.3265	906.7276	1.2122	1.7827
28	2018	41	14.8857	70.6020	936.4898	2.6122	1.5704
39	2018	41	13.4714	75.9388	909.4398	1.6816	2.7367
24	2018	41	15.1571	76.2857	947.9745	1.1357	1.4867
63	2018	41	15.3000	73.3673	952.5939	5.1031	1.2541
62	2018	41	12.4286	90.5204	882.1112	0.3143	0.9224
1	2018	41	12.5143	80.3571	882.0745	1.0827	2.1041
31	2018	42	11.8429	91.4490	852.5459	0.1286	0.8857
79	2018	42	14.5143	79.5714	978.6847	2.1898	1.0837
51	2018	42	13.0429	84.9184	947.2776	1.7643	1.2429
14	2018	42	14.3000	82.6224	904.1194	1.0367	1.6867
67	2018	42	12.8143	84.6837	909.3959	0.9612	2.2316
42	2018	42	12.9286	85.4286	881.6276	0.7653	2.0622
50	2018	42	13.2429	83.7143	909.4347	0.4286	1.2347
43	2018	42	12.9286	85.4286	881.6276	0.7653	2.0622
85	2018	42	14.1429	85.6735	918.0235	0.3429	1.1786
25	2018	42	17.1000	82.5000	985.0306	2.1276	1.0398
69	2018	42	14.2714	81.5612	948.2204	0.8878	1.1480
57	2018	42	12.1571	91.1837	894.0459	0.5949	1.5306
9	2018	42	13.3571	86.8061	860.2837	0.3306	1.7735
72	2018	42	15.2857	82.9694	884.1112	0.5633	1.5051
26	2018	42	15.5714	91.4286	873.1827	1.0990	1.4112
7	2018	42	15.0714	94.0612	865.3112	0.8388	1.3694
83	2018	42	19.6000	84.2245	951.8980	1.2847	1.0184
76	2018	42	13.2000	85.9592	929.1265	0.4684	1.1367
36	2018	42	13.4857	79.6429	936.7582	1.5673	1.5816
81	2018	42	13.0429	84.9184	947.2776	1.7643	1.2429
15	2018	42	12.9429	90.0510	926.6000	0.4541	1.0939
32	2018	42	12.9286	85.4286	881.6276	0.7653	2.0622
73	2018	42	15.1000	83.0918	970.9571	1.2449	0.6704

71	2018	42	13.4857	79.6429	936.7582	1.5673	1.5816
41	2018	42	12.4429	93.3673	881.5071	0.2776	0.8561
10	2018	42	14.4714	80.0816	972.5990	1.9980	1.0000
23	2018	42	11.2286	95.8469	780.4469	1.2010	1.5837
27	2018	42	15.0714	94.0612	865.3112	0.8388	1.3694
60	2018	42	13.0429	84.9184	947.2776	1.7643	1.2429
53	2018	42	13.3571	86.8061	860.2837	0.3306	1.7735
66	2018	42	14.3000	82.6224	904.1194	1.0367	1.6867
59	2018	42	12.1571	91.1837	894.0459	0.5949	1.5306
61	2018	42	15.1000	83.0918	970.9571	1.2449	0.6704
84	2018	42	15.1000	83.0918	970.9571	1.2449	0.6704
38	2018	42	12.1571	91.1837	894.0459	0.5949	1.5306
87	2018	42	15.9286	85.3878	906.4857	0.7612	1.4561
34	2018	42	12.1571	91.1837	894.0459	0.5949	1.5306
29	2018	42	14.2714	81.5612	948.2204	0.8878	1.1480
5	2018	42	13.8833	92.3673	837.8000	0.8163	1.3194
8	2018	42	13.3571	86.8061	860.2837	0.3306	1.7735
12	2018	42	13.8833	92.3673	837.8000	0.8163	1.3194
13	2018	42	19.6000	84.2245	951.8980	1.2847	1.0184
18	2018	42	14.0167	82.8163	972.8092	1.8561	0.8327
33	2018	42	13.2429	83.7143	909.4347	0.4286	1.2347
56	2018	42	17.1000	82.5000	985.0306	2.1276	1.0398
77	2018	42	12.9429	90.0510	926.6000	0.4541	1.0939
54	2018	42	13.8833	92.3673	837.8000	0.8163	1.3194
21	2018	42	13.2429	83.7143	909.4347	0.4286	1.2347
68	2018	42	14.5143	79.5714	978.6847	2.1898	1.0837
74	2018	42	15.1000	83.0918	970.9571	1.2449	0.6704
88	2018	42	12.9286	85.4286	881.6276	0.7653	2.0622
16	2018	42	13.2000	85.9592	929.1265	0.4684	1.1367
30	2018	42	14.3000	82.6224	904.1194	1.0367	1.6867
6	2018	42	14.5143	79.5714	978.6847	2.1898	1.0837
49	2018	42	14.2714	81.5612	948.2204	0.8878	1.1480
22	2018	42	12.9286	85.4286	881.6276	0.7653	2.0622
45	2018	42	14.0143	88.8367	822.7163	1.5878	1.2939
58	2018	42	14.2714	81.5612	948.2204	0.8878	1.1480
37	2018	42	14.5143	79.5714	978.6847	2.1898	1.0837
17	2018	42	12.8143	84.6837	909.3959	0.9612	2.2316
55	2018	42	15.2857	82.9694	884.1112	0.5633	1.5051
46	2018	42	13.2000	85.9592	929.1265	0.4684	1.1367
86	2018	42	13.1571	91.7347	872.6633	0.0000	0.7602
2	2018	42	13.1571	91.7347	872.6633	0.0000	0.7602
4	2018	42	13.2429	83.7143	909.4347	0.4286	1.2347
47	2018	42	19.3571	83.4184	966.3898	0.8388	0.2735
82	2018	42	12.9286	85.4286	881.6276	0.7653	2.0622
19	2018	42	18.1714	77.0918	968.0653	2.1735	0.9480
20	2018	42	13.3571	86.8061	860.2837	0.3306	1.7735
80	2018	42	12.9286	85.4286	881.6276	0.7653	2.0622
3	2018	42	19.6000	84.2245	951.8980	1.2847	1.0184
52	2018	42	12.8143	84.6837	909.3959	0.9612	2.2316
70	2018	42	14.1429	85.6735	918.0235	0.3429	1.1786

64	2018	42	11.2286	95.8469	780.4469	1.2010	1.5837
48	2018	42	12.9429	90.0510	926.6000	0.4541	1.0939
65	2018	42	12.8143	84.6837	909.3959	0.9612	2.2316
44	2018	42	14.1429	85.6735	918.0235	0.3429	1.1786
75	2018	42	11.2286	95.8469	780.4469	1.2010	1.5837
40	2018	42	13.8714	82.0408	953.1255	2.8327	1.1408
11	2018	42	15.2857	82.9694	884.1112	0.5633	1.5051
35	2018	42	13.0429	84.9184	947.2776	1.7643	1.2429
78	2018	42	15.9286	85.3878	906.4857	0.7612	1.4561
28	2018	42	13.4857	79.6429	936.7582	1.5673	1.5816
39	2018	42	12.8143	84.6837	909.3959	0.9612	2.2316
24	2018	42	14.2714	81.5612	948.2204	0.8878	1.1480
63	2018	42	13.8714	82.0408	953.1255	2.8327	1.1408
62	2018	42	12.4429	93.3673	881.5071	0.2776	0.8561
1	2018	42	12.9286	85.4286	881.6276	0.7653	2.0622
31	2018	43	12.5571	91.6327	851.9194	0.2898	0.8949
79	2018	43	17.2429	83.8878	978.8490	1.3449	0.9122
51	2018	43	15.8167	89.7704	947.1816	0.7673	1.1959
14	2018	43	15.0857	91.2347	903.8990	0.4398	1.7867
67	2018	43	14.9143	91.8265	909.2092	0.4459	2.1316
42	2018	43	14.1143	88.2143	881.1847	0.5459	2.1592
50	2018	43	14.7857	83.8571	908.9153	0.1837	1.4612
43	2018	43	14.1143	88.2143	881.1847	0.5459	2.1592
85	2018	43	15.5286	85.1224	917.4510	0.1816	1.1531
25	2018	43	18.4500	89.0510	985.1061	0.6316	0.9115
69	2018	43	16.9286	85.4490	948.0898	0.6541	0.9398
57	2018	43	14.2571	90.9592	893.5490	0.2929	1.4653
9	2018	43	13.3857	88.5918	859.7990	0.4031	2.0092
72	2018	43	15.3143	85.0714	883.6765	0.7541	1.6714
26	2018	43	15.7857	92.0102	872.6724	1.6439	1.4633
7	2018	43	15.4333	92.2143	864.6918	1.8724	1.7163
83	2018	43	19.5143	86.3878	951.5367	1.7776	0.9776
76	2018	43	15.4000	87.1939	928.8347	0.4429	1.1684
36	2018	43	15.9143	87.6327	936.7296	0.7929	1.4939
81	2018	43	15.8167	89.7704	947.1816	0.7673	1.1959
15	2018	43	15.2000	89.2551	926.2694	0.3776	1.1367
32	2018	43	14.1143	88.2143	881.1847	0.5459	2.1592
73	2018	43	17.2667	84.8163	970.7571	0.6878	0.5936
71	2018	43	15.9143	87.6327	936.7296	0.7929	1.4939
41	2018	43	14.1833	93.7959	880.8908	0.2469	0.8878
10	2018	43	16.6000	87.1837	972.8051	0.8816	0.9184
23	2018	43	9.8000	92.4490	779.8429	3.0704	1.7796
27	2018	43	15.4333	92.2143	864.6918	1.8724	1.7163
60	2018	43	15.8167	89.7704	947.1816	0.7673	1.1959
53	2018	43	13.3857	88.5918	859.7990	0.4031	2.0092
66	2018	43	15.0857	91.2347	903.8990	0.4398	1.7867
59	2018	43	14.2571	90.9592	893.5490	0.2929	1.4653
61	2018	43	17.2667	84.8163	970.7571	0.6878	0.5936
84	2018	43	17.2667	84.8163	970.7571	0.6878	0.5936
38	2018	43	14.2571	90.9592	893.5490	0.2929	1.4653

87	2018	43	16.0143	89.5510	906.0827	0.7571	1.4520
34	2018	43	14.2571	90.9592	893.5490	0.2929	1.4653
29	2018	43	16.9286	85.4490	948.0898	0.6541	0.9398
5	2018	43	13.4857	90.9184	837.2755	2.1531	1.4878
8	2018	43	13.3857	88.5918	859.7990	0.4031	2.0092
12	2018	43	13.4857	90.9184	837.2755	2.1531	1.4878
13	2018	43	19.5143	86.3878	951.5367	1.7776	0.9776
18	2018	43	16.6714	88.4490	972.8520	0.5480	0.7816
33	2018	43	14.7857	83.8571	908.9153	0.1837	1.4612
56	2018	43	18.4500	89.0510	985.1061	0.6316	0.9115
77	2018	43	15.2000	89.2551	926.2694	0.3776	1.1367
54	2018	43	13.4857	90.9184	837.2755	2.1531	1.4878
21	2018	43	14.7857	83.8571	908.9153	0.1837	1.4612
68	2018	43	17.2429	83.8878	978.8490	1.3449	0.9122
74	2018	43	17.2667	84.8163	970.7571	0.6878	0.5936
88	2018	43	14.1143	88.2143	881.1847	0.5459	2.1592
16	2018	43	15.4000	87.1939	928.8347	0.4429	1.1684
30	2018	43	15.0857	91.2347	903.8990	0.4398	1.7867
6	2018	43	17.2429	83.8878	978.8490	1.3449	0.9122
49	2018	43	16.9286	85.4490	948.0898	0.6541	0.9398
22	2018	43	14.1143	88.2143	881.1847	0.5459	2.1592
45	2018	43	13.9000	86.5918	822.2459	2.9612	1.4786
58	2018	43	16.9286	85.4490	948.0898	0.6541	0.9398
37	2018	43	17.2429	83.8878	978.8490	1.3449	0.9122
17	2018	43	14.9143	91.8265	909.2092	0.4459	2.1316
55	2018	43	15.3143	85.0714	883.6765	0.7541	1.6714
46	2018	43	15.4000	87.1939	928.8347	0.4429	1.1684
86	2018	43	13.8429	92.9592	872.1112	0.0000	0.7582
2	2018	43	13.8429	92.9592	872.1112	0.0000	0.7582
4	2018	43	14.7857	83.8571	908.9153	0.1837	1.4612
47	2018	43	19.3143	86.5408	966.1388	1.2878	0.2000
82	2018	43	14.1143	88.2143	881.1847	0.5459	2.1592
19	2018	43	18.4429	85.7245	968.1612	0.6112	0.8286
20	2018	43	13.3857	88.5918	859.7990	0.4031	2.0092
80	2018	43	14.1143	88.2143	881.1847	0.5459	2.1592
3	2018	43	19.5143	86.3878	951.5367	1.7776	0.9776
52	2018	43	14.9143	91.8265	909.2092	0.4459	2.1316
70	2018	43	15.5286	85.1224	917.4510	0.1816	1.1531
64	2018	43	9.8000	92.4490	779.8429	3.0704	1.7796
48	2018	43	15.2000	89.2551	926.2694	0.3776	1.1367
65	2018	43	14.9143	91.8265	909.2092	0.4459	2.1316
44	2018	43	15.5286	85.1224	917.4510	0.1816	1.1531
75	2018	43	9.8000	92.4490	779.8429	3.0704	1.7796
40	2018	43	15.9000	90.8061	953.2735	0.7878	1.0888
11	2018	43	15.3143	85.0714	883.6765	0.7541	1.6714
35	2018	43	15.8167	89.7704	947.1816	0.7673	1.1959
78	2018	43	16.0143	89.5510	906.0827	0.7571	1.4520
28	2018	43	15.9143	87.6327	936.7296	0.7929	1.4939
39	2018	43	14.9143	91.8265	909.2092	0.4459	2.1316
24	2018	43	16.9286	85.4490	948.0898	0.6541	0.9398

63	2018	43	15.9000	90.8061	953.2735	0.7878	1.0888
62	2018	43	14.1833	93.7959	880.8908	0.2469	0.8878
1	2018	43	14.1143	88.2143	881.1847	0.5459	2.1592
31	2018	44	11.3857	86.5510	853.9357	1.4469	0.8418
79	2018	44	17.6286	77.6735	979.4755	3.8316	0.7367
51	2018	44	15.2571	83.4337	948.0510	2.8286	1.1316
14	2018	44	15.1571	83.4898	905.2347	2.0357	1.6286
67	2018	44	14.9429	82.6735	910.4980	1.5378	2.6878
42	2018	44	13.8143	82.1224	882.8418	1.4684	2.1806
50	2018	44	14.0571	82.4082	910.5776	1.0551	1.4633
43	2018	44	13.8143	82.1224	882.8418	1.4684	2.1806
85	2018	44	15.3714	83.3673	919.2184	0.6459	1.0286
25	2018	44	18.1000	83.5918	985.8694	2.6551	0.9873
69	2018	44	16.2714	80.7347	949.2112	2.2133	0.9857
57	2018	44	13.6429	85.8469	895.2020	0.8888	1.4755
9	2018	44	12.6714	83.9082	861.6163	0.8071	1.9245
72	2018	44	14.0714	80.0510	885.3816	1.7643	1.7184
26	2018	44	14.1143	86.0714	874.5102	2.5796	1.3806
7	2018	44	13.0714	86.6224	866.4847	2.9520	1.5949
83	2018	44	17.2714	84.8061	953.2061	2.9061	0.9327
76	2018	44	15.0571	82.8367	930.1500	1.5969	1.1816
36	2018	44	16.1857	80.8571	937.8561	2.7378	1.3388
81	2018	44	15.2571	83.4337	948.0510	2.8286	1.1316
15	2018	44	14.7714	83.8980	927.6592	1.4082	1.1133
32	2018	44	13.8143	82.1224	882.8418	1.4684	2.1806
73	2018	44	17.1429	81.9490	971.7265	1.6898	0.5693
71	2018	44	16.1857	80.8571	937.8561	2.7378	1.3388
41	2018	44	12.7571	88.8878	882.6459	1.3735	0.8265
10	2018	44	16.3429	80.5000	973.4959	3.0122	0.9612
23	2018	44	8.4286	87.0918	781.8653	3.9480	1.7980
27	2018	44	13.0714	86.6224	866.4847	2.9520	1.5949
60	2018	44	15.2571	83.4337	948.0510	2.8286	1.1316
53	2018	44	12.6714	83.9082	861.6163	0.8071	1.9245
66	2018	44	15.1571	83.4898	905.2347	2.0357	1.6286
59	2018	44	13.6429	85.8469	895.2020	0.8888	1.4755
61	2018	44	17.1429	81.9490	971.7265	1.6898	0.5693
84	2018	44	17.1429	81.9490	971.7265	1.6898	0.5693
38	2018	44	13.6429	85.8469	895.2020	0.8888	1.4755
87	2018	44	14.3857	86.3571	907.7490	1.8990	1.5459
34	2018	44	13.6429	85.8469	895.2020	0.8888	1.4755
29	2018	44	16.2714	80.7347	949.2112	2.2133	0.9857
5	2018	44	11.2143	86.9796	839.3408	3.1704	1.3265
8	2018	44	12.6714	83.9082	861.6163	0.8071	1.9245
12	2018	44	11.2143	86.9796	839.3408	3.1704	1.3265
13	2018	44	17.2714	84.8061	953.2061	2.9061	0.9327
18	2018	44	16.7714	82.4796	973.7071	1.8153	0.6857
33	2018	44	14.0571	82.4082	910.5776	1.0551	1.4633
56	2018	44	18.1000	83.5918	985.8694	2.6551	0.9873
77	2018	44	14.7714	83.8980	927.6592	1.4082	1.1133
54	2018	44	11.2143	86.9796	839.3408	3.1704	1.3265

21	2018	44	14.0571	82.4082	910.5776	1.0551	1.4633
68	2018	44	17.6286	77.6735	979.4755	3.8316	0.7367
74	2018	44	17.1429	81.9490	971.7265	1.6898	0.5693
88	2018	44	13.8143	82.1224	882.8418	1.4684	2.1806
16	2018	44	15.0571	82.8367	930.1500	1.5969	1.1816
30	2018	44	15.1571	83.4898	905.2347	2.0357	1.6286
6	2018	44	17.6286	77.6735	979.4755	3.8316	0.7367
49	2018	44	16.2714	80.7347	949.2112	2.2133	0.9857
22	2018	44	13.8143	82.1224	882.8418	1.4684	2.1806
45	2018	44	10.9571	83.2959	824.2133	3.7286	1.5857
58	2018	44	16.2714	80.7347	949.2112	2.2133	0.9857
37	2018	44	17.6286	77.6735	979.4755	3.8316	0.7367
17	2018	44	14.9429	82.6735	910.4980	1.5378	2.6878
55	2018	44	14.0714	80.0510	885.3816	1.7643	1.7184
46	2018	44	15.0571	82.8367	930.1500	1.5969	1.1816
86	2018	44	12.3143	89.9592	874.0704	0.7122	0.6847
2	2018	44	12.3143	89.9592	874.0704	0.7122	0.6847
4	2018	44	14.0571	82.4082	910.5776	1.0551	1.4633
47	2018	44	17.4714	84.3776	967.5673	2.3990	0.2980
82	2018	44	13.8143	82.1224	882.8418	1.4684	2.1806
19	2018	44	18.1143	82.6020	969.2388	2.0888	0.8122
20	2018	44	12.6714	83.9082	861.6163	0.8071	1.9245
80	2018	44	13.8143	82.1224	882.8418	1.4684	2.1806
3	2018	44	17.2714	84.8061	953.2061	2.9061	0.9327
52	2018	44	14.9429	82.6735	910.4980	1.5378	2.6878
70	2018	44	15.3714	83.3673	919.2184	0.6459	1.0286
64	2018	44	8.4286	87.0918	781.8653	3.9480	1.7980
48	2018	44	14.7714	83.8980	927.6592	1.4082	1.1133
65	2018	44	14.9429	82.6735	910.4980	1.5378	2.6878
44	2018	44	15.3714	83.3673	919.2184	0.6459	1.0286
75	2018	44	8.4286	87.0918	781.8653	3.9480	1.7980
40	2018	44	16.1143	82.9082	953.7010	3.3337	1.0041
11	2018	44	14.0714	80.0510	885.3816	1.7643	1.7184
35	2018	44	15.2571	83.4337	948.0510	2.8286	1.1316
78	2018	44	14.3857	86.3571	907.7490	1.8990	1.5459
28	2018	44	16.1857	80.8571	937.8561	2.7378	1.3388
39	2018	44	14.9429	82.6735	910.4980	1.5378	2.6878
24	2018	44	16.2714	80.7347	949.2112	2.2133	0.9857
63	2018	44	16.1143	82.9082	953.7010	3.3337	1.0041
62	2018	44	12.7571	88.8878	882.6459	1.3735	0.8265
1	2018	44	13.8143	82.1224	882.8418	1.4684	2.1806
31	2018	45	7.8000	80.4898	854.2031	2.9316	0.9622
79	2018	45	11.6429	73.6633	979.8592	4.8388	1.0990
51	2018	45	11.0714	80.6837	948.4020	4.3837	1.2510
14	2018	45	13.6714	76.7653	905.4388	3.8153	1.8602
67	2018	45	12.3000	75.9082	910.6071	2.7163	2.8918
42	2018	45	10.9714	76.7755	883.0827	2.8837	2.1153
50	2018	45	9.7286	78.5612	910.9245	2.6653	1.4908
43	2018	45	10.9714	76.7755	883.0827	2.8837	2.1153
85	2018	45	10.2000	78.7245	919.6724	1.1939	1.2092

25	2018	45	16.5000	77.8980	986.2653	4.4684	1.1768
69	2018	45	12.6714	78.3061	949.4490	3.5786	1.1092
57	2018	45	9.3000	81.3367	895.4663	2.0337	1.7776
9	2018	45	10.9857	77.6020	861.8082	2.3582	1.7990
72	2018	45	13.3429	75.3776	885.6418	3.7684	1.6724
26	2018	45	14.9143	80.4796	874.7510	4.7082	1.4653
7	2018	45	14.2571	82.8776	866.8153	4.3378	1.4439
83	2018	45	18.0286	81.2041	953.6837	4.6816	1.0204
76	2018	45	10.7143	79.8571	930.3663	2.5918	1.1612
36	2018	45	12.7429	75.6531	937.9827	4.3194	1.4867
81	2018	45	11.0714	80.6837	948.4020	4.3837	1.2510
15	2018	45	9.8143	80.7959	927.9276	2.4755	1.2112
32	2018	45	10.9714	76.7755	883.0827	2.8837	2.1153
73	2018	45	13.0857	79.4184	972.1296	2.8755	0.6919
71	2018	45	12.7429	75.6531	937.9827	4.3194	1.4867
41	2018	45	10.0143	84.8367	882.9418	3.1378	1.0296
10	2018	45	12.6571	75.4286	973.7908	4.4194	1.0898
23	2018	45	7.2143	83.0204	781.8949	4.1969	1.6888
27	2018	45	14.2571	82.8776	866.8153	4.3378	1.4439
60	2018	45	11.0714	80.6837	948.4020	4.3837	1.2510
53	2018	45	10.9857	77.6020	861.8082	2.3582	1.7990
66	2018	45	13.6714	76.7653	905.4388	3.8153	1.8602
59	2018	45	9.3000	81.3367	895.4663	2.0337	1.7776
61	2018	45	13.0857	79.4184	972.1296	2.8755	0.6919
84	2018	45	13.0857	79.4184	972.1296	2.8755	0.6919
38	2018	45	9.3000	81.3367	895.4663	2.0337	1.7776
87	2018	45	13.4714	81.8878	908.0878	3.8173	1.6827
34	2018	45	9.3000	81.3367	895.4663	2.0337	1.7776
29	2018	45	12.6714	78.3061	949.4490	3.5786	1.1092
5	2018	45	12.0429	82.8061	839.4939	4.2582	1.3561
8	2018	45	10.9857	77.6020	861.8082	2.3582	1.7990
12	2018	45	12.0429	82.8061	839.4939	4.2582	1.3561
13	2018	45	18.0286	81.2041	953.6837	4.6816	1.0204
18	2018	45	11.7429	77.1633	974.1071	3.3541	0.6520
33	2018	45	9.7286	78.5612	910.9245	2.6653	1.4908
56	2018	45	16.5000	77.8980	986.2653	4.4684	1.1768
77	2018	45	9.8143	80.7959	927.9276	2.4755	1.2112
54	2018	45	12.0429	82.8061	839.4939	4.2582	1.3561
21	2018	45	9.7286	78.5612	910.9245	2.6653	1.4908
68	2018	45	11.6429	73.6633	979.8592	4.8388	1.0990
74	2018	45	13.0857	79.4184	972.1296	2.8755	0.6919
88	2018	45	10.9714	76.7755	883.0827	2.8837	2.1153
16	2018	45	10.7143	79.8571	930.3663	2.5918	1.1612
30	2018	45	13.6714	76.7653	905.4388	3.8153	1.8602
6	2018	45	11.6429	73.6633	979.8592	4.8388	1.0990
49	2018	45	12.6714	78.3061	949.4490	3.5786	1.1092
22	2018	45	10.9714	76.7755	883.0827	2.8837	2.1153
45	2018	45	12.8571	78.9388	824.3286	4.5755	1.5184
58	2018	45	12.6714	78.3061	949.4490	3.5786	1.1092
37	2018	45	11.6429	73.6633	979.8592	4.8388	1.0990

17	2018	45	12.3000	75.9082	910.6071	2.7163	2.8918
55	2018	45	13.3429	75.3776	885.6418	3.7684	1.6724
46	2018	45	10.7143	79.8571	930.3663	2.5918	1.1612
86	2018	45	10.7429	84.1837	874.3561	1.8051	0.8520
2	2018	45	10.7429	84.1837	874.3561	1.8051	0.8520
4	2018	45	9.7286	78.5612	910.9245	2.6653	1.4908
47	2018	45	18.1286	79.6327	968.0061	3.9806	0.4378
82	2018	45	10.9714	76.7755	883.0827	2.8837	2.1153
19	2018	45	17.6571	77.6735	969.5378	3.5510	0.8378
20	2018	45	10.9857	77.6020	861.8082	2.3582	1.7990
80	2018	45	10.9714	76.7755	883.0827	2.8837	2.1153
3	2018	45	18.0286	81.2041	953.6837	4.6816	1.0204
52	2018	45	12.3000	75.9082	910.6071	2.7163	2.8918
70	2018	45	10.2000	78.7245	919.6724	1.1939	1.2092
64	2018	45	7.2143	83.0204	781.8949	4.1969	1.6888
48	2018	45	9.8143	80.7959	927.9276	2.4755	1.2112
65	2018	45	12.3000	75.9082	910.6071	2.7163	2.8918
44	2018	45	10.2000	78.7245	919.6724	1.1939	1.2092
75	2018	45	7.2143	83.0204	781.8949	4.1969	1.6888
40	2018	45	13.0714	76.5102	953.8112	5.3316	1.1347
11	2018	45	13.3429	75.3776	885.6418	3.7684	1.6724
35	2018	45	11.0714	80.6837	948.4020	4.3837	1.2510
78	2018	45	13.4714	81.8878	908.0878	3.8173	1.6827
28	2018	45	12.7429	75.6531	937.9827	4.3194	1.4867
39	2018	45	12.3000	75.9082	910.6071	2.7163	2.8918
24	2018	45	12.6714	78.3061	949.4490	3.5786	1.1092
63	2018	45	13.0714	76.5102	953.8112	5.3316	1.1347
62	2018	45	10.0143	84.8367	882.9418	3.1378	1.0296
1	2018	45	10.9714	76.7755	883.0827	2.8837	2.1153
31	2018	46	8.3000	83.4388	851.7663	2.2173	0.9827
79	2018	46	11.1429	83.2245	979.4755	1.7378	1.3408
51	2018	46	9.8429	87.8571	947.4765	2.3357	1.4959
14	2018	46	10.5143	85.8980	903.6245	2.5980	2.1786
67	2018	46	9.2143	86.1939	908.9245	1.5837	2.6592
42	2018	46	8.7286	83.9694	880.9184	2.1776	2.3061
50	2018	46	9.9000	80.3776	909.0480	2.0663	1.4959
43	2018	46	8.7286	83.9694	880.9184	2.1776	2.3061
85	2018	46	10.5714	81.0000	917.8204	0.5827	1.3367
25	2018	46	14.2000	81.9184	985.3633	2.4602	1.1765
69	2018	46	11.3286	83.3265	948.1490	1.8347	1.1878
57	2018	46	8.6286	87.6735	893.4480	1.4041	1.9408
9	2018	46	9.2714	82.4388	859.3735	2.5551	2.0867
72	2018	46	11.7143	80.1837	883.3143	3.5969	1.8796
26	2018	46	15.8857	82.4184	872.0592	5.9878	1.9531
7	2018	46	13.8000	83.7551	864.1888	5.4122	1.9082
83	2018	46	17.6143	80.1633	951.2857	4.4163	1.1092
76	2018	46	9.8714	85.9082	928.8663	1.3194	1.2286
36	2018	46	10.2571	84.0306	936.6000	2.4082	1.6980
81	2018	46	9.8429	87.8571	947.4765	2.3357	1.4959
15	2018	46	9.8000	87.4082	926.3531	1.4388	1.1592

32	2018	46	8.7286	83.9694	880.9184	2.1776	2.3061
73	2018	46	12.1714	82.8163	971.1898	1.5347	0.7388
71	2018	46	10.2571	84.0306	936.6000	2.4082	1.6980
41	2018	46	9.0286	88.2653	880.7480	2.4490	1.2265
10	2018	46	11.2286	83.2857	973.1704	2.1704	1.0776
23	2018	46	8.2857	81.3878	779.0602	6.3122	1.8612
27	2018	46	13.8000	83.7551	864.1888	5.4122	1.9082
60	2018	46	9.8429	87.8571	947.4765	2.3357	1.4959
53	2018	46	9.2714	82.4388	859.3735	2.5551	2.0867
66	2018	46	10.5143	85.8980	903.6245	2.5980	2.1786
59	2018	46	8.6286	87.6735	893.4480	1.4041	1.9408
61	2018	46	12.1714	82.8163	971.1898	1.5347	0.7388
84	2018	46	12.1714	82.8163	971.1898	1.5347	0.7388
38	2018	46	8.6286	87.6735	893.4480	1.4041	1.9408
87	2018	46	11.7571	85.7755	905.9459	3.0398	1.9633
34	2018	46	8.6286	87.6735	893.4480	1.4041	1.9408
29	2018	46	11.3286	83.3265	948.1490	1.8347	1.1878
5	2018	46	11.0000	84.2755	836.7214	5.6306	1.6367
8	2018	46	9.2714	82.4388	859.3735	2.5551	2.0867
12	2018	46	11.0000	84.2755	836.7214	5.6306	1.6367
13	2018	46	17.6143	80.1633	951.2857	4.4163	1.1092
18	2018	46	11.1429	85.1837	973.5735	1.7490	0.7500
33	2018	46	9.9000	80.3776	909.0480	2.0663	1.4959
56	2018	46	14.2000	81.9184	985.3633	2.4602	1.1765
77	2018	46	9.8000	87.4082	926.3531	1.4388	1.1592
54	2018	46	11.0000	84.2755	836.7214	5.6306	1.6367
21	2018	46	9.9000	80.3776	909.0480	2.0663	1.4959
68	2018	46	11.1429	83.2245	979.4755	1.7378	1.3408
74	2018	46	12.1714	82.8163	971.1898	1.5347	0.7388
88	2018	46	8.7286	83.9694	880.9184	2.1776	2.3061
16	2018	46	9.8714	85.9082	928.8663	1.3194	1.2286
30	2018	46	10.5143	85.8980	903.6245	2.5980	2.1786
6	2018	46	11.1429	83.2245	979.4755	1.7378	1.3408
49	2018	46	11.3286	83.3265	948.1490	1.8347	1.1878
22	2018	46	8.7286	83.9694	880.9184	2.1776	2.3061
45	2018	46	13.0333	73.3673	821.6143	6.5673	1.7837
58	2018	46	11.3286	83.3265	948.1490	1.8347	1.1878
37	2018	46	11.1429	83.2245	979.4755	1.7378	1.3408
17	2018	46	9.2143	86.1939	908.9245	1.5837	2.6592
55	2018	46	11.7143	80.1837	883.3143	3.5969	1.8796
46	2018	46	9.8714	85.9082	928.8663	1.3194	1.2286
86	2018	46	9.4857	85.8776	871.9571	1.5163	1.0449
2	2018	46	9.4857	85.8776	871.9571	1.5163	1.0449
4	2018	46	9.9000	80.3776	909.0480	2.0663	1.4959
47	2018	46	16.7000	78.5918	965.9122	3.4561	0.5571
82	2018	46	8.7286	83.9694	880.9184	2.1776	2.3061
19	2018	46	14.8857	78.7857	968.0480	2.1612	1.0194
20	2018	46	9.2714	82.4388	859.3735	2.5551	2.0867
80	2018	46	8.7286	83.9694	880.9184	2.1776	2.3061
3	2018	46	17.6143	80.1633	951.2857	4.4163	1.1092

52	2018	46	9.2143	86.1939	908.9245	1.5837	2.6592
70	2018	46	10.5714	81.0000	917.8204	0.5827	1.3367
64	2018	46	8.2857	81.3878	779.0602	6.3122	1.8612
48	2018	46	9.8000	87.4082	926.3531	1.4388	1.1592
65	2018	46	9.2143	86.1939	908.9245	1.5837	2.6592
44	2018	46	10.5714	81.0000	917.8204	0.5827	1.3367
75	2018	46	8.2857	81.3878	779.0602	6.3122	1.8612
40	2018	46	10.5143	85.1327	953.3112	2.7163	1.4551
11	2018	46	11.7143	80.1837	883.3143	3.5969	1.8796
35	2018	46	9.8429	87.8571	947.4765	2.3357	1.4959
78	2018	46	11.7571	85.7755	905.9459	3.0398	1.9633
28	2018	46	10.2571	84.0306	936.6000	2.4082	1.6980
39	2018	46	9.2143	86.1939	908.9245	1.5837	2.6592
24	2018	46	11.3286	83.3265	948.1490	1.8347	1.1878
63	2018	46	10.5143	85.1327	953.3112	2.7163	1.4551
62	2018	46	9.0286	88.2653	880.7480	2.4490	1.2265
1	2018	46	8.7286	83.9694	880.9184	2.1776	2.3061
31	2018	47	6.8000	86.8980	851.5357	0.7847	0.7510
79	2018	47	11.5000	85.7347	980.1214	0.6827	1.2357
51	2018	47	9.4000	90.5204	947.9561	0.8122	1.4122
14	2018	47	9.2143	92.9184	903.6327	0.9010	1.8847
67	2018	47	8.8857	93.2755	909.1480	0.5214	2.5735
42	2018	47	7.9143	89.6020	880.8388	0.9224	2.4847
50	2018	47	8.4143	84.1633	909.2653	0.7469	1.3745
43	2018	47	7.9143	89.6020	880.8388	0.9224	2.4847
85	2018	47	8.9286	86.2755	918.1286	0.0755	1.2276
25	2018	47	12.5857	85.8163	986.1714	0.9449	0.9867
69	2018	47	10.8857	86.2449	948.6306	0.4592	1.1276
57	2018	47	7.7714	92.8367	893.5398	0.5714	1.8429
9	2018	47	7.8000	88.8469	859.1020	1.2653	2.1827
72	2018	47	9.4429	85.9694	883.1765	1.4745	2.0857
26	2018	47	11.3000	86.3469	871.6398	4.2592	1.9806
7	2018	47	10.5000	87.2347	863.7949	3.9520	1.9980
83	2018	47	14.3000	83.9796	951.4378	1.9643	1.0510
76	2018	47	9.3000	89.2245	929.2265	0.3663	1.2847
36	2018	47	9.7571	89.0612	937.0633	0.7602	1.7031
81	2018	47	9.4000	90.5204	947.9561	0.8122	1.4122
15	2018	47	9.2000	91.4388	926.6714	0.4745	0.9561
32	2018	47	7.9143	89.6020	880.8388	0.9224	2.4847
73	2018	47	11.7286	85.5918	971.7980	0.3684	0.6000
71	2018	47	9.7571	89.0612	937.0633	0.7602	1.7031
41	2018	47	8.2429	91.9286	880.7673	0.9214	1.0735
10	2018	47	10.4857	87.7653	973.8082	0.9602	0.8990
23	2018	47	5.9714	83.0816	778.3020	6.6163	2.2061
27	2018	47	10.5000	87.2347	863.7949	3.9520	1.9980
60	2018	47	9.4000	90.5204	947.9561	0.8122	1.4122
53	2018	47	7.8000	88.8469	859.1020	1.2653	2.1827
66	2018	47	9.2143	92.9184	903.6327	0.9010	1.8847
59	2018	47	7.7714	92.8367	893.5398	0.5714	1.8429
61	2018	47	11.7286	85.5918	971.7980	0.3684	0.6000

84	2018	47	11.7286	85.5918	971.7980	0.3684	0.6000
38	2018	47	7.7714	92.8367	893.5398	0.5714	1.8429
87	2018	47	9.7571	90.7653	905.9929	1.0724	2.0755
34	2018	47	7.7714	92.8367	893.5398	0.5714	1.8429
29	2018	47	10.8857	86.2449	948.6306	0.4592	1.1276
5	2018	47	8.1857	90.2245	836.2367	4.2633	1.6735
8	2018	47	7.8000	88.8469	859.1020	1.2653	2.1827
12	2018	47	8.1857	90.2245	836.2367	4.2633	1.6735
13	2018	47	14.3000	83.9796	951.4378	1.9643	1.0510
18	2018	47	11.0714	89.8061	974.1663	0.6071	0.8714
33	2018	47	8.4143	84.1633	909.2653	0.7469	1.3745
56	2018	47	12.5857	85.8163	986.1714	0.9449	0.9867
77	2018	47	9.2000	91.4388	926.6714	0.4745	0.9561
54	2018	47	8.1857	90.2245	836.2367	4.2633	1.6735
21	2018	47	8.4143	84.1633	909.2653	0.7469	1.3745
68	2018	47	11.5000	85.7347	980.1214	0.6827	1.2357
74	2018	47	11.7286	85.5918	971.7980	0.3684	0.6000
88	2018	47	7.9143	89.6020	880.8388	0.9224	2.4847
16	2018	47	9.3000	89.2245	929.2265	0.3663	1.2847
30	2018	47	9.2143	92.9184	903.6327	0.9010	1.8847
6	2018	47	11.5000	85.7347	980.1214	0.6827	1.2357
49	2018	47	10.8857	86.2449	948.6306	0.4592	1.1276
22	2018	47	7.9143	89.6020	880.8388	0.9224	2.4847
45	2018	47	10.1429	73.4694	820.9337	6.9378	1.9031
58	2018	47	10.8857	86.2449	948.6306	0.4592	1.1276
37	2018	47	11.5000	85.7347	980.1214	0.6827	1.2357
17	2018	47	8.8857	93.2755	909.1480	0.5214	2.5735
55	2018	47	9.4429	85.9694	883.1765	1.4745	2.0857
46	2018	47	9.3000	89.2245	929.2265	0.3663	1.2847
86	2018	47	8.5857	89.3571	871.8367	0.6204	1.0316
2	2018	47	8.5857	89.3571	871.8367	0.6204	1.0316
4	2018	47	8.4143	84.1633	909.2653	0.7469	1.3745
47	2018	47	14.1000	80.4082	966.2959	1.5163	0.6112
82	2018	47	7.9143	89.6020	880.8388	0.9224	2.4847
19	2018	47	13.2429	78.5612	968.4173	0.9561	1.1714
20	2018	47	7.8000	88.8469	859.1020	1.2653	2.1827
80	2018	47	7.9143	89.6020	880.8388	0.9224	2.4847
3	2018	47	14.3000	83.9796	951.4378	1.9643	1.0510
52	2018	47	8.8857	93.2755	909.1480	0.5214	2.5735
70	2018	47	8.9286	86.2755	918.1286	0.0755	1.2276
64	2018	47	5.9714	83.0816	778.3020	6.6163	2.2061
48	2018	47	9.2000	91.4388	926.6714	0.4745	0.9561
65	2018	47	8.8857	93.2755	909.1480	0.5214	2.5735
44	2018	47	8.9286	86.2755	918.1286	0.0755	1.2276
75	2018	47	5.9714	83.0816	778.3020	6.6163	2.2061
40	2018	47	9.6286	91.7551	953.9092	1.0133	1.5378
11	2018	47	9.4429	85.9694	883.1765	1.4745	2.0857
35	2018	47	9.4000	90.5204	947.9561	0.8122	1.4122
78	2018	47	9.7571	90.7653	905.9929	1.0724	2.0755
28	2018	47	9.7571	89.0612	937.0633	0.7602	1.7031

39	2018	47	8.8857	93.2755	909.1480	0.5214	2.5735
24	2018	47	10.8857	86.2449	948.6306	0.4592	1.1276
63	2018	47	9.6286	91.7551	953.9092	1.0133	1.5378
62	2018	47	8.2429	91.9286	880.7673	0.9214	1.0735
1	2018	47	7.9143	89.6020	880.8388	0.9224	2.4847
31	2018	48	10.3000	81.7857	852.7194	2.3531	0.7612
79	2018	48	13.5714	82.2143	980.8561	2.4041	0.9786
51	2018	48	12.4857	87.0510	948.9357	2.1571	1.0357
14	2018	48	13.4000	87.0000	905.1745	2.2143	1.6398
67	2018	48	13.3286	86.9694	910.4806	2.8296	2.2561
42	2018	48	12.7000	84.1122	882.2592	3.1439	2.2633
50	2018	48	11.2286	83.4388	910.4500	2.2949	1.3469
43	2018	48	12.7000	84.1122	882.2592	3.1439	2.2633
85	2018	48	11.5714	86.5714	919.3480	1.2204	1.1367
25	2018	48	14.7000	85.7755	987.7898	2.1980	0.8561
69	2018	48	13.8571	84.6939	949.7663	2.1449	0.9959
57	2018	48	11.6571	88.5918	894.7827	2.7245	1.8939
9	2018	48	12.0571	84.7653	860.6071	2.9878	2.0939
72	2018	48	13.0286	83.2347	884.8602	3.1469	1.7735
26	2018	48	14.2000	84.0918	873.5490	4.5602	1.7112
7	2018	48	13.3000	85.0714	865.6102	4.6663	1.6214
83	2018	48	15.9714	83.1939	953.6235	3.4041	1.0235
76	2018	48	11.4429	87.2245	930.3633	1.7929	1.2592
36	2018	48	13.3429	85.5816	938.3204	2.3857	1.4408
81	2018	48	12.4857	87.0510	948.9357	2.1571	1.0357
15	2018	48	10.9143	89.8878	927.7673	1.5714	0.9857
32	2018	48	12.7000	84.1122	882.2592	3.1439	2.2633
73	2018	48	12.8571	86.8878	972.9755	1.1265	0.6173
71	2018	48	13.3429	85.5816	938.3204	2.3857	1.4408
41	2018	48	11.6857	86.0816	881.9888	2.0184	0.9663
10	2018	48	12.9000	84.7653	974.7969	2.9265	0.8255
23	2018	48	9.0143	77.3469	779.7082	6.2582	2.2908
27	2018	48	13.3000	85.0714	865.6102	4.6663	1.6214
60	2018	48	12.4857	87.0510	948.9357	2.1571	1.0357
53	2018	48	12.0571	84.7653	860.6071	2.9878	2.0939
66	2018	48	13.4000	87.0000	905.1745	2.2143	1.6398
59	2018	48	11.6571	88.5918	894.7827	2.7245	1.8939
61	2018	48	12.8571	86.8878	972.9755	1.1265	0.6173
84	2018	48	12.8571	86.8878	972.9755	1.1265	0.6173
38	2018	48	11.6571	88.5918	894.7827	2.7245	1.8939
87	2018	48	13.3429	87.1531	907.6061	3.1082	1.6316
34	2018	48	11.6571	88.5918	894.7827	2.7245	1.8939
29	2018	48	13.8571	84.6939	949.7663	2.1449	0.9959
5	2018	48	12.1571	86.8367	837.9224	4.6990	1.6347
8	2018	48	12.0571	84.7653	860.6071	2.9878	2.0939
12	2018	48	12.1571	86.8367	837.9224	4.6990	1.6347
13	2018	48	15.9714	83.1939	953.6235	3.4041	1.0235
18	2018	48	12.9143	87.9694	974.9582	2.0561	0.7367
33	2018	48	11.2286	83.4388	910.4500	2.2949	1.3469
56	2018	48	14.7000	85.7755	987.7898	2.1980	0.8561

77	2018	48	10.9143	89.8878	927.7673	1.5714	0.9857
54	2018	48	12.1571	86.8367	837.9224	4.6990	1.6347
21	2018	48	11.2286	83.4388	910.4500	2.2949	1.3469
68	2018	48	13.5714	82.2143	980.8561	2.4041	0.9786
74	2018	48	12.8571	86.8878	972.9755	1.1265	0.6173
88	2018	48	12.7000	84.1122	882.2592	3.1439	2.2633
16	2018	48	11.4429	87.2245	930.3633	1.7929	1.2592
30	2018	48	13.4000	87.0000	905.1745	2.2143	1.6398
6	2018	48	13.5714	82.2143	980.8561	2.4041	0.9786
49	2018	48	13.8571	84.6939	949.7663	2.1449	0.9959
22	2018	48	12.7000	84.1122	882.2592	3.1439	2.2633
45	2018	48	12.5571	72.6122	822.4735	7.1337	1.7918
58	2018	48	13.8571	84.6939	949.7663	2.1449	0.9959
37	2018	48	13.5714	82.2143	980.8561	2.4041	0.9786
17	2018	48	13.3286	86.9694	910.4806	2.8296	2.2561
55	2018	48	13.0286	83.2347	884.8602	3.1469	1.7735
46	2018	48	11.4429	87.2245	930.3633	1.7929	1.2592
86	2018	48	12.2857	84.4388	873.1673	1.5418	1.2031
2	2018	48	12.2857	84.4388	873.1673	1.5418	1.2031
4	2018	48	11.2286	83.4388	910.4500	2.2949	1.3469
47	2018	48	15.8429	80.2347	968.4000	2.6755	0.5908
82	2018	48	12.7000	84.1122	882.2592	3.1439	2.2633
19	2018	48	15.6857	76.7755	970.2061	2.7878	0.9980
20	2018	48	12.0571	84.7653	860.6071	2.9878	2.0939
80	2018	48	12.7000	84.1122	882.2592	3.1439	2.2633
3	2018	48	15.9714	83.1939	953.6235	3.4041	1.0235
52	2018	48	13.3286	86.9694	910.4806	2.8296	2.2561
70	2018	48	11.5714	86.5714	919.3480	1.2204	1.1367
64	2018	48	9.0143	77.3469	779.7082	6.2582	2.2908
48	2018	48	10.9143	89.8878	927.7673	1.5714	0.9857
65	2018	48	13.3286	86.9694	910.4806	2.8296	2.2561
44	2018	48	11.5714	86.5714	919.3480	1.2204	1.1367
75	2018	48	9.0143	77.3469	779.7082	6.2582	2.2908
40	2018	48	13.2571	86.3163	955.1000	2.9571	1.1653
11	2018	48	13.0286	83.2347	884.8602	3.1469	1.7735
35	2018	48	12.4857	87.0510	948.9357	2.1571	1.0357
78	2018	48	13.3429	87.1531	907.6061	3.1082	1.6316
28	2018	48	13.3429	85.5816	938.3204	2.3857	1.4408
39	2018	48	13.3286	86.9694	910.4806	2.8296	2.2561
24	2018	48	13.8571	84.6939	949.7663	2.1449	0.9959
63	2018	48	13.2571	86.3163	955.1000	2.9571	1.1653
62	2018	48	11.6857	86.0816	881.9888	2.0184	0.9663
1	2018	48	12.7000	84.1122	882.2592	3.1439	2.2633
31	2018	49	5.8286	79.1837	851.4378	3.6255	1.0653
79	2018	49	5.5571	83.3673	980.0224	2.1408	1.3694
51	2018	49	4.2000	88.1224	947.9276	2.1786	1.3612
14	2018	49	7.2857	82.7449	904.1224	3.1551	2.0316
67	2018	49	5.4714	83.1122	909.3173	3.9816	2.4704
42	2018	49	5.6429	80.1837	881.1020	4.4592	2.2929
50	2018	49	6.0571	82.6224	909.1031	2.9378	1.6082

43	2018	49	5.6429	80.1837	881.1020	4.4592	2.2929
85	2018	49	7.2429	84.3265	917.8520	1.7245	1.2990
25	2018	49	10.5286	82.7449	986.5092	2.9541	1.1571
69	2018	49	6.9000	81.7449	948.5163	2.6694	1.3347
57	2018	49	5.0286	85.4694	893.4765	3.4643	2.1531
9	2018	49	7.0571	80.1633	859.5378	4.5745	2.4316
72	2018	49	9.2286	78.7551	883.7816	4.9082	1.8204
26	2018	49	12.9667	75.3367	872.6429	5.9796	1.9296
7	2018	49	11.0429	77.8265	864.7439	6.5510	1.7469
83	2018	49	14.3714	79.9796	952.3214	5.5316	1.1337
76	2018	49	5.5286	87.0714	929.1602	1.7398	1.4684
36	2018	49	5.5286	83.7245	937.1122	3.5000	1.5224
81	2018	49	4.2000	88.1224	947.9276	2.1786	1.3612
15	2018	49	5.8286	88.7551	926.5510	1.5306	1.4673
32	2018	49	5.6429	80.1837	881.1020	4.4592	2.2929
73	2018	49	7.8571	84.8571	971.9122	1.0786	0.7010
71	2018	49	5.5286	83.7245	937.1122	3.5000	1.5224
41	2018	49	5.9143	78.7041	880.5520	2.2643	1.2602
10	2018	49	5.5857	86.2347	973.8306	2.8837	1.0622
23	2018	49	7.5000	68.4184	779.0153	6.6898	2.2367
27	2018	49	11.0429	77.8265	864.7439	6.5510	1.7469
60	2018	49	4.2000	88.1224	947.9276	2.1786	1.3612
53	2018	49	7.0571	80.1633	859.5378	4.5745	2.4316
66	2018	49	7.2857	82.7449	904.1224	3.1551	2.0316
59	2018	49	5.0286	85.4694	893.4765	3.4643	2.1531
61	2018	49	7.8571	84.8571	971.9122	1.0786	0.7010
84	2018	49	7.8571	84.8571	971.9122	1.0786	0.7010
38	2018	49	5.0286	85.4694	893.4765	3.4643	2.1531
87	2018	49	8.5143	83.1429	906.3867	4.3051	1.5827
34	2018	49	5.0286	85.4694	893.4765	3.4643	2.1531
29	2018	49	6.9000	81.7449	948.5163	2.6694	1.3347
5	2018	49	9.1429	75.1939	837.0827	6.1163	2.0694
8	2018	49	7.0571	80.1633	859.5378	4.5745	2.4316
12	2018	49	9.1429	75.1939	837.0827	6.1163	2.0694
13	2018	49	14.3714	79.9796	952.3214	5.5316	1.1337
18	2018	49	6.0286	86.1020	974.0592	1.8939	0.9092
33	2018	49	6.0571	82.6224	909.1031	2.9378	1.6082
56	2018	49	10.5286	82.7449	986.5092	2.9541	1.1571
77	2018	49	5.8286	88.7551	926.5510	1.5306	1.4673
54	2018	49	9.1429	75.1939	837.0827	6.1163	2.0694
21	2018	49	6.0571	82.6224	909.1031	2.9378	1.6082
68	2018	49	5.5571	83.3673	980.0224	2.1408	1.3694
74	2018	49	7.8571	84.8571	971.9122	1.0786	0.7010
88	2018	49	5.6429	80.1837	881.1020	4.4592	2.2929
16	2018	49	5.5286	87.0714	929.1602	1.7398	1.4684
30	2018	49	7.2857	82.7449	904.1224	3.1551	2.0316
6	2018	49	5.5571	83.3673	980.0224	2.1408	1.3694
49	2018	49	6.9000	81.7449	948.5163	2.6694	1.3347
22	2018	49	5.6429	80.1837	881.1020	4.4592	2.2929
45	2018	49	11.2714	64.9286	821.8694	7.6990	2.0347

58	2018	49	6.9000	81.7449	948.5163	2.6694	1.3347
37	2018	49	5.5571	83.3673	980.0224	2.1408	1.3694
17	2018	49	5.4714	83.1122	909.3173	3.9816	2.4704
55	2018	49	9.2286	78.7551	883.7816	4.9082	1.8204
46	2018	49	5.5286	87.0714	929.1602	1.7398	1.4684
86	2018	49	7.4429	77.7245	871.8398	2.2245	1.5235
2	2018	49	7.4429	77.7245	871.8398	2.2245	1.5235
4	2018	49	6.0571	82.6224	909.1031	2.9378	1.6082
47	2018	49	13.0143	78.8571	966.9888	4.1592	0.6194
82	2018	49	5.6429	80.1837	881.1020	4.4592	2.2929
19	2018	49	11.4429	76.3878	969.0500	3.5143	1.0949
20	2018	49	7.0571	80.1633	859.5378	4.5745	2.4316
80	2018	49	5.6429	80.1837	881.1020	4.4592	2.2929
3	2018	49	14.3714	79.9796	952.3214	5.5316	1.1337
52	2018	49	5.4714	83.1122	909.3173	3.9816	2.4704
70	2018	49	7.2429	84.3265	917.8520	1.7245	1.2990
64	2018	49	7.5000	68.4184	779.0153	6.6898	2.2367
48	2018	49	5.8286	88.7551	926.5510	1.5306	1.4673
65	2018	49	5.4714	83.1122	909.3173	3.9816	2.4704
44	2018	49	7.2429	84.3265	917.8520	1.7245	1.2990
75	2018	49	7.5000	68.4184	779.0153	6.6898	2.2367
40	2018	49	5.9857	83.1429	954.0694	3.6929	1.4153
11	2018	49	9.2286	78.7551	883.7816	4.9082	1.8204
35	2018	49	4.2000	88.1224	947.9276	2.1786	1.3612
78	2018	49	8.5143	83.1429	906.3867	4.3051	1.5827
28	2018	49	5.5286	83.7245	937.1122	3.5000	1.5224
39	2018	49	5.4714	83.1122	909.3173	3.9816	2.4704
24	2018	49	6.9000	81.7449	948.5163	2.6694	1.3347
63	2018	49	5.9857	83.1429	954.0694	3.6929	1.4153
62	2018	49	5.9143	78.7041	880.5520	2.2643	1.2602
1	2018	49	5.6429	80.1837	881.1020	4.4592	2.2929
31	2018	50	1.4857	87.1327	851.8276	1.6908	1.1878
79	2018	50	4.2000	82.0306	983.7520	0.4592	2.1327
51	2018	50	2.8429	89.8061	950.7204	0.4224	1.7265
14	2018	50	2.8143	87.8776	905.1520	1.2908	2.2480
67	2018	50	2.0286	88.9490	910.9082	1.4990	2.6694
42	2018	50	1.7000	85.4898	882.0153	1.7776	2.6133
50	2018	50	3.0429	80.7755	910.6796	0.9857	1.7520
43	2018	50	1.7000	85.4898	882.0153	1.7776	2.6133
85	2018	50	3.9143	82.3265	919.4837	0.6633	1.3837
25	2018	50	6.6000	78.9898	989.4867	1.3592	1.3929
69	2018	50	4.3143	78.9898	951.2061	0.7969	1.7408
57	2018	50	1.7857	87.8571	894.7786	1.1133	2.0592
9	2018	50	2.4714	85.8469	859.8602	2.1439	2.6041
72	2018	50	4.3000	81.5408	884.3857	2.2541	2.2643
26	2018	50	5.8857	80.1735	872.6439	3.3112	1.9704
7	2018	50	4.8714	84.3061	864.8969	3.5878	2.0010
83	2018	50	9.3000	79.2347	953.7337	2.9643	1.2378
76	2018	50	3.2143	86.8367	931.3510	0.2694	1.5602
36	2018	50	3.0571	85.4796	939.4449	1.4765	1.9704

81	2018	50	2.8429	89.8061	950.7204	0.4224	1.7265
15	2018	50	3.7143	84.7449	928.5857	0.4112	1.6602
32	2018	50	1.7000	85.4898	882.0153	1.7776	2.6133
73	2018	50	5.7286	78.1531	974.9520	0.2459	0.8347
71	2018	50	3.0571	85.4796	939.4449	1.4765	1.9704
41	2018	50	2.0143	81.9184	881.5163	0.6327	1.4367
10	2018	50	4.1286	87.9592	977.1439	0.5551	1.2071
23	2018	50	3.5286	74.8469	778.1337	5.5643	2.1480
27	2018	50	4.8714	84.3061	864.8969	3.5878	2.0010
60	2018	50	2.8429	89.8061	950.7204	0.4224	1.7265
53	2018	50	2.4714	85.8469	859.8602	2.1439	2.6041
66	2018	50	2.8143	87.8776	905.1520	1.2908	2.2480
59	2018	50	1.7857	87.8571	894.7786	1.1133	2.0592
61	2018	50	5.7286	78.1531	974.9520	0.2459	0.8347
84	2018	50	5.7286	78.1531	974.9520	0.2459	0.8347
38	2018	50	1.7857	87.8571	894.7786	1.1133	2.0592
87	2018	50	4.5286	85.8878	907.5490	1.6520	2.3786
34	2018	50	1.7857	87.8571	894.7786	1.1133	2.0592
29	2018	50	4.3143	78.9898	951.2061	0.7969	1.7408
5	2018	50	2.9286	82.4796	836.9133	3.3694	2.1439
8	2018	50	2.4714	85.8469	859.8602	2.1439	2.6041
12	2018	50	2.9286	82.4796	836.9133	3.3694	2.1439
13	2018	50	9.3000	79.2347	953.7337	2.9643	1.2378
18	2018	50	4.5429	82.4184	977.4878	0.3490	1.1673
33	2018	50	3.0429	80.7755	910.6796	0.9857	1.7520
56	2018	50	6.6000	78.9898	989.4867	1.3592	1.3929
77	2018	50	3.7143	84.7449	928.5857	0.4112	1.6602
54	2018	50	2.9286	82.4796	836.9133	3.3694	2.1439
21	2018	50	3.0429	80.7755	910.6796	0.9857	1.7520
68	2018	50	4.2000	82.0306	983.7520	0.4592	2.1327
74	2018	50	5.7286	78.1531	974.9520	0.2459	0.8347
88	2018	50	1.7000	85.4898	882.0153	1.7776	2.6133
16	2018	50	3.2143	86.8367	931.3510	0.2694	1.5602
30	2018	50	2.8143	87.8776	905.1520	1.2908	2.2480
6	2018	50	4.2000	82.0306	983.7520	0.4592	2.1327
49	2018	50	4.3143	78.9898	951.2061	0.7969	1.7408
22	2018	50	1.7000	85.4898	882.0153	1.7776	2.6133
45	2018	50	5.8571	72.1020	821.3898	5.6510	2.1061
58	2018	50	4.3143	78.9898	951.2061	0.7969	1.7408
37	2018	50	4.2000	82.0306	983.7520	0.4592	2.1327
17	2018	50	2.0286	88.9490	910.9082	1.4990	2.6694
55	2018	50	4.3000	81.5408	884.3857	2.2541	2.2643
46	2018	50	3.2143	86.8367	931.3510	0.2694	1.5602
86	2018	50	2.8000	82.3163	872.5520	0.9214	1.3429
2	2018	50	2.8000	82.3163	872.5520	0.9214	1.3429
4	2018	50	3.0429	80.7755	910.6796	0.9857	1.7520
47	2018	50	8.9714	76.3061	968.9337	2.1755	0.6561
82	2018	50	1.7000	85.4898	882.0153	1.7776	2.6133
19	2018	50	7.0143	75.1633	971.2837	1.3327	1.5235
20	2018	50	2.4714	85.8469	859.8602	2.1439	2.6041

80	2018	50	1.7000	85.4898	882.0153	1.7776	2.6133
3	2018	50	9.3000	79.2347	953.7337	2.9643	1.2378
52	2018	50	2.0286	88.9490	910.9082	1.4990	2.6694
70	2018	50	3.9143	82.3265	919.4837	0.6633	1.3837
64	2018	50	3.5286	74.8469	778.1337	5.5643	2.1480
48	2018	50	3.7143	84.7449	928.5857	0.4112	1.6602
65	2018	50	2.0286	88.9490	910.9082	1.4990	2.6694
44	2018	50	3.9143	82.3265	919.4837	0.6633	1.3837
75	2018	50	3.5286	74.8469	778.1337	5.5643	2.1480
40	2018	50	2.8143	88.1735	956.6898	1.3796	1.9561
11	2018	50	4.3000	81.5408	884.3857	2.2541	2.2643
35	2018	50	2.8429	89.8061	950.7204	0.4224	1.7265
78	2018	50	4.5286	85.8878	907.5490	1.6520	2.3786
28	2018	50	3.0571	85.4796	939.4449	1.4765	1.9704
39	2018	50	2.0286	88.9490	910.9082	1.4990	2.6694
24	2018	50	4.3143	78.9898	951.2061	0.7969	1.7408
63	2018	50	2.8143	88.1735	956.6898	1.3796	1.9561
62	2018	50	2.0143	81.9184	881.5163	0.6327	1.4367
1	2018	50	1.7000	85.4898	882.0153	1.7776	2.6133
31	2018	51	5.9286	89.6531	853.7255	0.9531	1.0061
79	2018	51	9.7571	78.8673	985.8112	1.3306	1.5704
51	2018	51	8.4857	84.8367	952.8867	0.8133	1.2786
14	2018	51	10.7857	85.0408	907.2745	0.9276	2.0214
67	2018	51	9.3000	85.0612	913.1082	1.0245	2.4837
42	2018	51	8.5429	82.8878	884.1265	0.7337	2.5071
50	2018	51	7.2571	79.0306	913.0071	1.0153	1.4306
43	2018	51	8.5429	82.8878	884.1265	0.7337	2.5071
85	2018	51	7.4571	82.0000	921.9796	1.3020	1.0908
25	2018	51	13.1000	78.2653	992.3031	1.1918	1.1541
69	2018	51	9.5429	76.5408	953.7480	0.4857	1.3296
57	2018	51	7.2714	86.3878	897.0010	0.4857	1.6724
9	2018	51	8.3857	87.8878	861.8041	0.3929	2.0316
72	2018	51	10.8571	82.3571	886.5286	0.4847	1.8673
26	2018	51	12.6143	88.3061	874.7878	1.1786	1.6959
7	2018	51	11.7857	91.2347	866.8133	1.1643	1.6714
83	2018	51	15.1333	78.4898	956.5786	1.0724	1.0959
76	2018	51	8.0000	82.3367	933.6806	0.9143	1.3082
36	2018	51	9.8000	81.1837	941.8204	1.1316	1.8133
81	2018	51	8.4857	84.8367	952.8867	0.8133	1.2786
15	2018	51	7.3571	80.3061	930.8541	1.0969	1.3224
32	2018	51	8.5429	82.8878	884.1265	0.7337	2.5071
73	2018	51	10.1857	75.9592	977.4469	0.8520	0.8510
71	2018	51	9.8000	81.1837	941.8204	1.1316	1.8133
41	2018	51	7.5000	83.7347	883.6633	0.4786	1.0480
10	2018	51	9.8429	81.6429	979.4041	1.0837	0.9602
23	2018	51	7.6143	80.5408	779.3684	4.0806	1.8786
27	2018	51	11.7857	91.2347	866.8133	1.1643	1.6714
60	2018	51	8.4857	84.8367	952.8867	0.8133	1.2786
53	2018	51	8.3857	87.8878	861.8041	0.3929	2.0316
66	2018	51	10.7857	85.0408	907.2745	0.9276	2.0214

59	2018	51	7.2714	86.3878	897.0010	0.4857	1.6724
61	2018	51	10.1857	75.9592	977.4469	0.8520	0.8510
84	2018	51	10.1857	75.9592	977.4469	0.8520	0.8510
38	2018	51	7.2714	86.3878	897.0010	0.4857	1.6724
87	2018	51	10.6429	83.8571	909.8898	0.7490	2.3327
34	2018	51	7.2714	86.3878	897.0010	0.4857	1.6724
29	2018	51	9.5429	76.5408	953.7480	0.4857	1.3296
5	2018	51	9.7286	93.2041	838.6082	1.4122	1.5469
8	2018	51	8.3857	87.8878	861.8041	0.3929	2.0316
12	2018	51	9.7286	93.2041	838.6082	1.4122	1.5469
13	2018	51	15.1333	78.4898	956.5786	1.0724	1.0959
18	2018	51	9.7000	78.6224	979.4653	1.1755	0.8398
33	2018	51	7.2571	79.0306	913.0071	1.0153	1.4306
56	2018	51	13.1000	78.2653	992.3031	1.1918	1.1541
77	2018	51	7.3571	80.3061	930.8541	1.0969	1.3224
54	2018	51	9.7286	93.2041	838.6082	1.4122	1.5469
21	2018	51	7.2571	79.0306	913.0071	1.0153	1.4306
68	2018	51	9.7571	78.8673	985.8112	1.3306	1.5704
74	2018	51	10.1857	75.9592	977.4469	0.8520	0.8510
88	2018	51	8.5429	82.8878	884.1265	0.7337	2.5071
16	2018	51	8.0000	82.3367	933.6806	0.9143	1.3082
30	2018	51	10.7857	85.0408	907.2745	0.9276	2.0214
6	2018	51	9.7571	78.8673	985.8112	1.3306	1.5704
49	2018	51	9.5429	76.5408	953.7480	0.4857	1.3296
22	2018	51	8.5429	82.8878	884.1265	0.7337	2.5071
45	2018	51	11.3143	82.8980	822.8500	3.4367	1.7143
58	2018	51	9.5429	76.5408	953.7480	0.4857	1.3296
37	2018	51	9.7571	78.8673	985.8112	1.3306	1.5704
17	2018	51	9.3000	85.0612	913.1082	1.0245	2.4837
55	2018	51	10.8571	82.3571	886.5286	0.4847	1.8673
46	2018	51	8.0000	82.3367	933.6806	0.9143	1.3082
86	2018	51	8.3857	85.6633	874.6786	0.2194	1.1418
2	2018	51	8.3857	85.6633	874.6786	0.2194	1.1418
4	2018	51	7.2571	79.0306	913.0071	1.0153	1.4306
47	2018	51	14.9571	74.0408	971.9000	0.7796	0.6041
82	2018	51	8.5429	82.8878	884.1265	0.7337	2.5071
19	2018	51	14.4143	71.6020	974.0133	1.0653	1.4500
20	2018	51	8.3857	87.8878	861.8041	0.3929	2.0316
80	2018	51	8.5429	82.8878	884.1265	0.7337	2.5071
3	2018	51	15.1333	78.4898	956.5786	1.0724	1.0959
52	2018	51	9.3000	85.0612	913.1082	1.0245	2.4837
70	2018	51	7.4571	82.0000	921.9796	1.3020	1.0908
64	2018	51	7.6143	80.5408	779.3684	4.0806	1.8786
48	2018	51	7.3571	80.3061	930.8541	1.0969	1.3224
65	2018	51	9.3000	85.0612	913.1082	1.0245	2.4837
44	2018	51	7.4571	82.0000	921.9796	1.3020	1.0908
75	2018	51	7.6143	80.5408	779.3684	4.0806	1.8786
40	2018	51	10.0571	86.0102	959.0296	1.4796	1.6020
11	2018	51	10.8571	82.3571	886.5286	0.4847	1.8673
35	2018	51	8.4857	84.8367	952.8867	0.8133	1.2786

78	2018	51	10.6429	83.8571	909.8898	0.7490	2.3327
28	2018	51	9.8000	81.1837	941.8204	1.1316	1.8133
39	2018	51	9.3000	85.0612	913.1082	1.0245	2.4837
24	2018	51	9.5429	76.5408	953.7480	0.4857	1.3296
63	2018	51	10.0571	86.0102	959.0296	1.4796	1.6020
62	2018	51	7.5000	83.7347	883.6633	0.4786	1.0480
1	2018	51	8.5429	82.8878	884.1265	0.7337	2.5071
31	2018	52	1.8143	87.8163	851.9163	1.0418	0.9418
79	2018	52	2.7286	79.1224	982.5786	1.2224	1.5755
51	2018	52	1.2857	86.0714	950.0582	0.7847	1.4031
14	2018	52	2.0857	86.2551	904.9541	1.0020	2.1194
67	2018	52	0.7714	86.8980	910.6755	1.0071	2.5102
42	2018	52	1.1143	84.2959	881.9510	0.6429	2.5071
50	2018	52	2.7000	82.4898	910.6561	1.0765	1.4357
43	2018	52	1.1143	84.2959	881.9510	0.6429	2.5071
85	2018	52	3.7571	85.3673	919.5724	1.3592	0.9939
25	2018	52	6.3429	78.8367	989.0265	1.1929	1.1071
69	2018	52	3.2143	79.1020	950.9204	0.4724	1.1602
57	2018	52	1.3714	88.1224	894.7265	0.4694	1.7857
9	2018	52	2.2857	88.6224	859.9071	0.4133	2.0449
72	2018	52	4.0429	83.3776	884.3684	0.6357	1.7908
26	2018	52	6.9286	86.9082	872.9061	2.5296	1.8735
7	2018	52	5.4714	86.9388	864.8153	2.6510	1.7673
83	2018	52	9.4429	77.7041	953.6531	1.7500	0.9531
76	2018	52	2.0429	83.7143	931.0867	0.8531	1.3194
36	2018	52	1.8714	81.9694	939.0929	1.0663	1.7255
81	2018	52	1.2857	86.0714	950.0582	0.7847	1.4031
15	2018	52	2.3286	82.9796	928.3847	0.9531	1.3133
32	2018	52	1.1143	84.2959	881.9510	0.6429	2.5071
73	2018	52	4.6143	77.2245	974.4143	0.7918	0.7133
71	2018	52	1.8714	81.9694	939.0929	1.0663	1.7255
41	2018	52	1.8286	84.0612	881.5143	0.5418	0.9653
10	2018	52	2.6429	83.1122	976.3367	1.0827	1.0378
23	2018	52	1.0429	78.9898	778.1102	4.1224	1.7918
27	2018	52	5.4714	86.9388	864.8153	2.6510	1.7673
60	2018	52	1.2857	86.0714	950.0582	0.7847	1.4031
53	2018	52	2.2857	88.6224	859.9071	0.4133	2.0449
66	2018	52	2.0857	86.2551	904.9541	1.0020	2.1194
59	2018	52	1.3714	88.1224	894.7265	0.4694	1.7857
61	2018	52	4.6143	77.2245	974.4143	0.7918	0.7133
84	2018	52	4.6143	77.2245	974.4143	0.7918	0.7133
38	2018	52	1.3714	88.1224	894.7265	0.4694	1.7857
87	2018	52	4.0857	85.6939	907.4520	0.9051	2.1684
34	2018	52	1.3714	88.1224	894.7265	0.4694	1.7857
29	2018	52	3.2143	79.1020	950.9204	0.4724	1.1602
5	2018	52	3.6571	90.6531	836.8265	2.9959	1.6051
8	2018	52	2.2857	88.6224	859.9071	0.4133	2.0449
12	2018	52	3.6571	90.6531	836.8265	2.9959	1.6051
13	2018	52	9.4429	77.7041	953.6531	1.7500	0.9531
18	2018	52	3.0571	79.5306	976.4357	1.1449	0.8755

33	2018	52	2.7000	82.4898	910.6561	1.0765	1.4357
56	2018	52	6.3429	78.8367	989.0265	1.1929	1.1071
77	2018	52	2.3286	82.9796	928.3847	0.9531	1.3133
54	2018	52	3.6571	90.6531	836.8265	2.9959	1.6051
21	2018	52	2.7000	82.4898	910.6561	1.0765	1.4357
68	2018	52	2.7286	79.1224	982.5786	1.2224	1.5755
74	2018	52	4.6143	77.2245	974.4143	0.7918	0.7133
88	2018	52	1.1143	84.2959	881.9510	0.6429	2.5071
16	2018	52	2.0429	83.7143	931.0867	0.8531	1.3194
30	2018	52	2.0857	86.2551	904.9541	1.0020	2.1194
6	2018	52	2.7286	79.1224	982.5786	1.2224	1.5755
49	2018	52	3.2143	79.1020	950.9204	0.4724	1.1602
22	2018	52	1.1143	84.2959	881.9510	0.6429	2.5071
45	2018	52	5.6000	77.1735	821.2847	4.5235	1.8255
58	2018	52	3.2143	79.1020	950.9204	0.4724	1.1602
37	2018	52	2.7286	79.1224	982.5786	1.2224	1.5755
17	2018	52	0.7714	86.8980	910.6755	1.0071	2.5102
55	2018	52	4.0429	83.3776	884.3684	0.6357	1.7908
46	2018	52	2.0429	83.7143	931.0867	0.8531	1.3194
86	2018	52	2.7714	85.7857	872.6837	0.2510	1.1418
2	2018	52	2.7714	85.7857	872.6837	0.2510	1.1418
4	2018	52	2.7000	82.4898	910.6561	1.0765	1.4357
47	2018	52	8.3286	75.6735	968.8592	0.9418	0.5582
82	2018	52	1.1143	84.2959	881.9510	0.6429	2.5071
19	2018	52	6.4571	74.0510	970.9694	1.2510	1.3898
20	2018	52	2.2857	88.6224	859.9071	0.4133	2.0449
80	2018	52	1.1143	84.2959	881.9510	0.6429	2.5071
3	2018	52	9.4429	77.7041	953.6531	1.7500	0.9531
52	2018	52	0.7714	86.8980	910.6755	1.0071	2.5102
70	2018	52	3.7571	85.3673	919.5724	1.3592	0.9939
64	2018	52	1.0429	78.9898	778.1102	4.1224	1.7918
48	2018	52	2.3286	82.9796	928.3847	0.9531	1.3133
65	2018	52	0.7714	86.8980	910.6755	1.0071	2.5102
44	2018	52	3.7571	85.3673	919.5724	1.3592	0.9939
75	2018	52	1.0429	78.9898	778.1102	4.1224	1.7918
40	2018	52	2.0143	86.4898	956.2031	1.4847	1.5622
11	2018	52	4.0429	83.3776	884.3684	0.6357	1.7908
35	2018	52	1.2857	86.0714	950.0582	0.7847	1.4031
78	2018	52	4.0857	85.6939	907.4520	0.9051	2.1684
28	2018	52	1.8714	81.9694	939.0929	1.0663	1.7255
39	2018	52	0.7714	86.8980	910.6755	1.0071	2.5102
24	2018	52	3.2143	79.1020	950.9204	0.4724	1.1602
63	2018	52	2.0143	86.4898	956.2031	1.4847	1.5622
62	2018	52	1.8286	84.0612	881.5143	0.5418	0.9653
1	2018	52	1.1143	84.2959	881.9510	0.6429	2.5071
31	2019	1	1.3857	90.2857	852.7367	0.4439	1.0612
79	2019	1	2.0429	80.4490	986.1724	0.0480	2.1041
51	2019	1	0.4571	89.6939	952.9490	0.0082	1.6908
14	2019	1	1.4143	93.7653	906.6347	0.1010	2.0378
67	2019	1	0.5143	95.3980	912.6633	0.0255	2.4765

42	2019	1	1.4429	92.2041	883.3061	0.0633	2.5643
50	2019	1	1.9286	84.1633	912.3878	0.1255	1.6663
43	2019	1	1.4429	92.2041	883.3061	0.0633	2.5643
85	2019	1	2.7714	85.8163	921.2888	0.1408	1.0837
25	2019	1	4.3714	81.6429	992.3112	0.0582	1.0663
69	2019	1	2.4429	82.5306	953.6184	0.0000	1.3684
57	2019	1	0.9000	92.9286	896.2837	0.0000	1.8541
9	2019	1	2.5286	92.8061	860.8612	0.3735	2.2276
72	2019	1	4.6000	87.9388	885.5704	0.4520	2.1286
26	2019	1	6.4143	90.8571	873.8990	2.0653	1.7092
7	2019	1	6.4000	87.5408	865.7694	2.6235	1.9653
83	2019	1	8.4714	80.1939	955.7092	0.9204	0.8776
76	2019	1	1.1857	87.8367	933.0082	0.0571	1.4969
36	2019	1	1.1429	86.7449	941.6776	0.0378	2.0184
81	2019	1	0.4571	89.6939	952.9490	0.0082	1.6908
15	2019	1	1.8714	85.3265	930.5796	0.0337	1.4214
32	2019	1	1.4429	92.2041	883.3061	0.0633	2.5643
73	2019	1	3.6714	76.4898	977.5214	0.0204	0.7724
71	2019	1	1.1429	86.7449	941.6776	0.0378	2.0184
41	2019	1	1.8571	86.8878	882.8571	0.3388	1.2582
10	2019	1	1.5000	87.8367	979.7337	0.0378	1.2541
23	2019	1	5.3143	81.7653	778.1265	4.4235	1.9316
27	2019	1	6.4000	87.5408	865.7694	2.6235	1.9653
60	2019	1	0.4571	89.6939	952.9490	0.0082	1.6908
53	2019	1	2.5286	92.8061	860.8612	0.3735	2.2276
66	2019	1	1.4143	93.7653	906.6347	0.1010	2.0378
59	2019	1	0.9000	92.9286	896.2837	0.0000	1.8541
61	2019	1	3.6714	76.4898	977.5214	0.0204	0.7724
84	2019	1	3.6714	76.4898	977.5214	0.0204	0.7724
38	2019	1	0.9000	92.9286	896.2837	0.0000	1.8541
87	2019	1	4.1429	90.8980	909.0245	0.4112	2.5112
34	2019	1	0.9000	92.9286	896.2837	0.0000	1.8541
29	2019	1	2.4429	82.5306	953.6184	0.0000	1.3684
5	2019	1	4.6429	92.6429	837.4541	2.8204	1.8265
8	2019	1	2.5286	92.8061	860.8612	0.3735	2.2276
12	2019	1	4.6429	92.6429	837.4541	2.8204	1.8265
13	2019	1	8.4714	80.1939	955.7092	0.9204	0.8776
18	2019	1	2.5429	82.3367	980.0010	0.0388	1.0663
33	2019	1	1.9286	84.1633	912.3878	0.1255	1.6663
56	2019	1	4.3714	81.6429	992.3112	0.0582	1.0663
77	2019	1	1.8714	85.3265	930.5796	0.0337	1.4214
54	2019	1	4.6429	92.6429	837.4541	2.8204	1.8265
21	2019	1	1.9286	84.1633	912.3878	0.1255	1.6663
68	2019	1	2.0429	80.4490	986.1724	0.0480	2.1041
74	2019	1	3.6714	76.4898	977.5214	0.0204	0.7724
88	2019	1	1.4429	92.2041	883.3061	0.0633	2.5643
16	2019	1	1.1857	87.8367	933.0082	0.0571	1.4969
30	2019	1	1.4143	93.7653	906.6347	0.1010	2.0378
6	2019	1	2.0429	80.4490	986.1724	0.0480	2.1041
49	2019	1	2.4429	82.5306	953.6184	0.0000	1.3684

22	2019	1	1.4429	92.2041	883.3061	0.0633	2.5643
45	2019	1	7.4143	78.4490	821.7337	4.3633	1.8133
58	2019	1	2.4429	82.5306	953.6184	0.0000	1.3684
37	2019	1	2.0429	80.4490	986.1724	0.0480	2.1041
17	2019	1	0.5143	95.3980	912.6633	0.0255	2.4765
55	2019	1	4.6000	87.9388	885.5704	0.4520	2.1286
46	2019	1	1.1857	87.8367	933.0082	0.0571	1.4969
86	2019	1	3.0857	89.1224	873.8235	0.1633	1.0704
2	2019	1	3.0857	89.1224	873.8235	0.1633	1.0704
4	2019	1	1.9286	84.1633	912.3878	0.1255	1.6663
47	2019	1	7.5857	79.2449	971.4357	0.3000	0.5622
82	2019	1	1.4429	92.2041	883.3061	0.0633	2.5643
19	2019	1	5.2714	78.8061	973.6796	0.2480	1.5490
20	2019	1	2.5286	92.8061	860.8612	0.3735	2.2276
80	2019	1	1.4429	92.2041	883.3061	0.0633	2.5643
3	2019	1	8.4714	80.1939	955.7092	0.9204	0.8776
52	2019	1	0.5143	95.3980	912.6633	0.0255	2.4765
70	2019	1	2.7714	85.8163	921.2888	0.1408	1.0837
64	2019	1	5.3143	81.7653	778.1265	4.4235	1.9316
48	2019	1	1.8714	85.3265	930.5796	0.0337	1.4214
65	2019	1	0.5143	95.3980	912.6633	0.0255	2.4765
44	2019	1	2.7714	85.8163	921.2888	0.1408	1.0837
75	2019	1	5.3143	81.7653	778.1265	4.4235	1.9316
40	2019	1	0.5000	91.7245	959.0153	0.0704	1.9653
11	2019	1	4.6000	87.9388	885.5704	0.4520	2.1286
35	2019	1	0.4571	89.6939	952.9490	0.0082	1.6908
78	2019	1	4.1429	90.8980	909.0245	0.4112	2.5112
28	2019	1	1.1429	86.7449	941.6776	0.0378	2.0184
39	2019	1	0.5143	95.3980	912.6633	0.0255	2.4765
24	2019	1	2.4429	82.5306	953.6184	0.0000	1.3684
63	2019	1	0.5000	91.7245	959.0153	0.0704	1.9653
62	2019	1	1.8571	86.8878	882.8571	0.3388	1.2582
1	2019	1	1.4429	92.2041	883.3061	0.0633	2.5643
31	2019	2	2.9714	92.1429	853.7102	0.7265	1.0031
79	2019	2	3.9286	83.8571	988.0276	0.0000	1.6878
51	2019	2	3.0000	90.6327	954.6255	0.0000	1.4010
14	2019	2	4.8143	95.6122	908.1449	0.0000	1.6939
67	2019	2	3.7857	96.0918	914.1061	0.0000	2.4673
42	2019	2	4.5286	93.7755	884.5041	0.1439	2.3061
50	2019	2	4.3286	84.4184	913.5908	0.0429	1.6051
43	2019	2	4.5286	93.7755	884.5041	0.1439	2.3061
85	2019	2	5.1000	86.5408	922.5204	0.0000	1.2051
25	2019	2	7.3000	87.0612	994.5245	0.0000	0.8306
69	2019	2	4.9571	85.1735	955.2214	0.0000	1.2816
57	2019	2	3.2286	94.2959	897.4673	0.0153	1.6796
9	2019	2	5.5429	93.7653	862.0286	0.8796	2.0184
72	2019	2	7.0571	90.6837	886.9388	0.7898	1.9684
26	2019	2	10.3571	93.7041	875.3898	1.8857	1.5041
7	2019	2	10.0571	87.0102	867.0643	3.0908	1.9704
83	2019	2	11.6857	84.4898	957.5633	0.0714	0.6551

76	2019	2	3.3857	90.8061	933.4694	0.0490	1.5714
36	2019	2	3.9429	88.5000	943.3337	0.0429	1.8316
81	2019	2	3.0000	90.6327	954.6255	0.0000	1.4010
15	2019	2	3.8857	87.1224	931.8776	0.1143	1.2888
32	2019	2	4.5286	93.7755	884.5041	0.1439	2.3061
73	2019	2	5.6571	79.4694	979.3071	0.0347	0.8112
71	2019	2	3.9429	88.5000	943.3337	0.0429	1.8316
41	2019	2	4.1857	87.0612	883.9255	0.7643	1.2214
10	2019	2	3.9000	89.2449	981.6633	0.0000	1.1245
23	2019	2	7.3714	71.1531	779.0673	5.9133	2.1439
27	2019	2	10.0571	87.0102	867.0643	3.0908	1.9704
60	2019	2	3.0000	90.6327	954.6255	0.0000	1.4010
53	2019	2	5.5429	93.7653	862.0286	0.8796	2.0184
66	2019	2	4.8143	95.6122	908.1449	0.0000	1.6939
59	2019	2	3.2286	94.2959	897.4673	0.0153	1.6796
61	2019	2	5.6571	79.4694	979.3071	0.0347	0.8112
84	2019	2	5.6571	79.4694	979.3071	0.0347	0.8112
38	2019	2	3.2286	94.2959	897.4673	0.0153	1.6796
87	2019	2	7.1571	92.7857	910.4184	0.1776	2.1847
34	2019	2	3.2286	94.2959	897.4673	0.0153	1.6796
29	2019	2	4.9571	85.1735	955.2214	0.0000	1.2816
5	2019	2	8.4000	89.7959	838.6082	3.6163	1.9541
8	2019	2	5.5429	93.7653	862.0286	0.8796	2.0184
12	2019	2	8.4000	89.7959	838.6082	3.6163	1.9541
13	2019	2	11.6857	84.4898	957.5633	0.0714	0.6551
18	2019	2	4.3429	85.2857	981.7398	0.0000	0.8816
33	2019	2	4.3286	84.4184	913.5908	0.0429	1.6051
56	2019	2	7.3000	87.0612	994.5245	0.0000	0.8306
77	2019	2	3.8857	87.1224	931.8776	0.1143	1.2888
54	2019	2	8.4000	89.7959	838.6082	3.6163	1.9541
21	2019	2	4.3286	84.4184	913.5908	0.0429	1.6051
68	2019	2	3.9286	83.8571	988.0276	0.0000	1.6878
74	2019	2	5.6571	79.4694	979.3071	0.0347	0.8112
88	2019	2	4.5286	93.7755	884.5041	0.1439	2.3061
16	2019	2	3.3857	90.8061	933.4694	0.0490	1.5714
30	2019	2	4.8143	95.6122	908.1449	0.0000	1.6939
6	2019	2	3.9286	83.8571	988.0276	0.0000	1.6878
49	2019	2	4.9571	85.1735	955.2214	0.0000	1.2816
22	2019	2	4.5286	93.7755	884.5041	0.1439	2.3061
45	2019	2	11.2000	73.5102	822.9653	4.9765	1.8520
58	2019	2	4.9571	85.1735	955.2214	0.0000	1.2816
37	2019	2	3.9286	83.8571	988.0276	0.0000	1.6878
17	2019	2	3.7857	96.0918	914.1061	0.0000	2.4673
55	2019	2	7.0571	90.6837	886.9388	0.7898	1.9684
46	2019	2	3.3857	90.8061	933.4694	0.0490	1.5714
86	2019	2	6.0000	88.9082	874.8510	0.4214	1.4949
2	2019	2	6.0000	88.9082	874.8510	0.4214	1.4949
4	2019	2	4.3286	84.4184	913.5908	0.0429	1.6051
47	2019	2	10.4714	83.4184	973.4388	0.0000	0.5000
82	2019	2	4.5286	93.7755	884.5041	0.1439	2.3061

19	2019	2	8.3714	81.4898	975.7571	0.0286	1.3765
20	2019	2	5.5429	93.7653	862.0286	0.8796	2.0184
80	2019	2	4.5286	93.7755	884.5041	0.1439	2.3061
3	2019	2	11.6857	84.4898	957.5633	0.0714	0.6551
52	2019	2	3.7857	96.0918	914.1061	0.0000	2.4673
70	2019	2	5.1000	86.5408	922.5204	0.0000	1.2051
64	2019	2	7.3714	71.1531	779.0673	5.9133	2.1439
48	2019	2	3.8857	87.1224	931.8776	0.1143	1.2888
65	2019	2	3.7857	96.0918	914.1061	0.0000	2.4673
44	2019	2	5.1000	86.5408	922.5204	0.0000	1.2051
75	2019	2	7.3714	71.1531	779.0673	5.9133	2.1439
40	2019	2	3.2429	93.4184	960.8490	0.0000	1.7061
11	2019	2	7.0571	90.6837	886.9388	0.7898	1.9684
35	2019	2	3.0000	90.6327	954.6255	0.0000	1.4010
78	2019	2	7.1571	92.7857	910.4184	0.1776	2.1847
28	2019	2	3.9429	88.5000	943.3337	0.0429	1.8316
39	2019	2	3.7857	96.0918	914.1061	0.0000	2.4673
24	2019	2	4.9571	85.1735	955.2214	0.0000	1.2816
63	2019	2	3.2429	93.4184	960.8490	0.0000	1.7061
62	2019	2	4.1857	87.0612	883.9255	0.7643	1.2214
1	2019	2	4.5286	93.7755	884.5041	0.1439	2.3061
31	2019	3	2.9714	92.0714	851.8735	0.9061	0.8806
79	2019	3	5.6000	84.7959	984.3643	0.1306	1.4061
51	2019	3	4.0286	91.0102	951.4051	0.0000	1.2571
14	2019	3	4.1714	96.4388	905.6378	0.0000	1.6173
67	2019	3	3.9571	94.6531	911.4122	0.0000	2.5102
42	2019	3	3.7143	93.3878	882.1418	0.4173	2.3510
50	2019	3	4.3429	85.8980	910.9561	0.0306	1.5541
43	2019	3	3.7143	93.3878	882.1418	0.4173	2.3510
85	2019	3	5.1857	89.3571	919.8694	0.0000	1.2143
25	2019	3	7.7286	89.6429	991.0224	0.0000	0.6633
69	2019	3	5.7429	85.7653	951.9796	0.0000	1.2112
57	2019	3	3.7143	94.7857	894.9857	0.0816	1.7908
9	2019	3	3.9857	93.5102	860.0041	1.1827	2.0092
72	2019	3	5.8286	91.1735	884.6939	1.1990	2.0908
26	2019	3	8.3714	92.1327	873.2051	2.5429	1.8143
7	2019	3	7.1714	86.3776	864.9367	3.4000	2.1102
83	2019	3	11.0857	85.2551	954.4724	0.2337	0.4969
76	2019	3	4.0571	93.3571	929.9633	0.0000	1.6245
36	2019	3	4.9000	88.7449	940.2673	0.0918	1.5745
81	2019	3	4.0286	91.0102	951.4051	0.0000	1.2571
15	2019	3	4.6857	89.4796	928.9541	0.2939	1.1367
32	2019	3	3.7143	93.3878	882.1418	0.4173	2.3510
73	2019	3	6.5714	82.7755	975.8551	0.0347	0.7092
71	2019	3	4.9000	88.7449	940.2673	0.0918	1.5745
41	2019	3	4.4714	85.9694	881.6194	0.7929	1.1235
10	2019	3	5.4143	89.7857	978.1520	0.0000	0.9959
23	2019	3	3.3286	66.0816	777.8796	6.8857	2.1541
27	2019	3	7.1714	86.3776	864.9367	3.4000	2.1102
60	2019	3	4.0286	91.0102	951.4051	0.0000	1.2571

53	2019	3	3.9857	93.5102	860.0041	1.1827	2.0092
66	2019	3	4.1714	96.4388	905.6378	0.0000	1.6173
59	2019	3	3.7143	94.7857	894.9857	0.0816	1.7908
61	2019	3	6.5714	82.7755	975.8551	0.0347	0.7092
84	2019	3	6.5714	82.7755	975.8551	0.0347	0.7092
38	2019	3	3.7143	94.7857	894.9857	0.0816	1.7908
87	2019	3	6.1571	93.4184	907.8418	0.3061	1.8796
34	2019	3	3.7143	94.7857	894.9857	0.0816	1.7908
29	2019	3	5.7429	85.7653	951.9796	0.0000	1.2112
5	2019	3	5.2143	88.1531	836.7745	4.5541	2.0143
8	2019	3	3.9857	93.5102	860.0041	1.1827	2.0092
12	2019	3	5.2143	88.1531	836.7745	4.5541	2.0143
13	2019	3	11.0857	85.2551	954.4724	0.2337	0.4969
18	2019	3	5.5286	86.6531	978.1357	0.1878	0.8541
33	2019	3	4.3429	85.8980	910.9561	0.0306	1.5541
56	2019	3	7.7286	89.6429	991.0224	0.0000	0.6633
77	2019	3	4.6857	89.4796	928.9541	0.2939	1.1367
54	2019	3	5.2143	88.1531	836.7745	4.5541	2.0143
21	2019	3	4.3429	85.8980	910.9561	0.0306	1.5541
68	2019	3	5.6000	84.7959	984.3643	0.1306	1.4061
74	2019	3	6.5714	82.7755	975.8551	0.0347	0.7092
88	2019	3	3.7143	93.3878	882.1418	0.4173	2.3510
16	2019	3	4.0571	93.3571	929.9633	0.0000	1.6245
30	2019	3	4.1714	96.4388	905.6378	0.0000	1.6173
6	2019	3	5.6000	84.7959	984.3643	0.1306	1.4061
49	2019	3	5.7429	85.7653	951.9796	0.0000	1.2112
22	2019	3	3.7143	93.3878	882.1418	0.4173	2.3510
45	2019	3	7.7714	67.5408	821.3306	6.7388	1.9816
58	2019	3	5.7429	85.7653	951.9796	0.0000	1.2112
37	2019	3	5.6000	84.7959	984.3643	0.1306	1.4061
17	2019	3	3.9571	94.6531	911.4122	0.0000	2.5102
55	2019	3	5.8286	91.1735	884.6939	1.1990	2.0908
46	2019	3	4.0571	93.3571	929.9633	0.0000	1.6245
86	2019	3	4.6571	87.8878	872.6908	0.6102	1.7592
2	2019	3	4.6571	87.8878	872.6908	0.6102	1.7592
4	2019	3	4.3429	85.8980	910.9561	0.0306	1.5541
47	2019	3	9.8286	85.5408	970.1561	0.0612	0.4867
82	2019	3	3.7143	93.3878	882.1418	0.4173	2.3510
19	2019	3	7.9571	82.8367	972.5735	0.0082	1.2122
20	2019	3	3.9857	93.5102	860.0041	1.1827	2.0092
80	2019	3	3.7143	93.3878	882.1418	0.4173	2.3510
3	2019	3	11.0857	85.2551	954.4724	0.2337	0.4969
52	2019	3	3.9571	94.6531	911.4122	0.0000	2.5102
70	2019	3	5.1857	89.3571	919.8694	0.0000	1.2143
64	2019	3	3.3286	66.0816	777.8796	6.8857	2.1541
48	2019	3	4.6857	89.4796	928.9541	0.2939	1.1367
65	2019	3	3.9571	94.6531	911.4122	0.0000	2.5102
44	2019	3	5.1857	89.3571	919.8694	0.0000	1.2143
75	2019	3	3.3286	66.0816	777.8796	6.8857	2.1541
40	2019	3	4.6571	93.9898	957.6990	0.0000	1.4480

11	2019	3	5.8286	91.1735	884.6939	1.1990	2.0908
35	2019	3	4.0286	91.0102	951.4051	0.0000	1.2571
78	2019	3	6.1571	93.4184	907.8418	0.3061	1.8796
28	2019	3	4.9000	88.7449	940.2673	0.0918	1.5745
39	2019	3	3.9571	94.6531	911.4122	0.0000	2.5102
24	2019	3	5.7429	85.7653	951.9796	0.0000	1.2112
63	2019	3	4.6571	93.9898	957.6990	0.0000	1.4480
62	2019	3	4.4714	85.9694	881.6194	0.7929	1.1235
1	2019	3	3.7143	93.3878	882.1418	0.4173	2.3510
31	2019	4	4.8000	87.9184	852.0388	0.8643	0.9480
79	2019	4	7.7571	78.1633	983.5122	0.7224	1.2265
51	2019	4	6.5714	85.0816	950.8714	0.0357	1.1398
14	2019	4	7.1429	90.4592	905.6071	0.6245	1.9357
67	2019	4	6.3714	87.1633	911.2745	0.2888	2.6418
42	2019	4	5.6571	89.1633	882.2622	0.4490	2.3245
50	2019	4	6.2571	82.3571	910.9735	0.2388	1.4837
43	2019	4	5.6571	89.1633	882.2622	0.4490	2.3245
85	2019	4	6.8000	85.5306	919.8980	0.0000	1.1735
25	2019	4	10.1857	85.5510	990.3612	0.6571	0.6041
69	2019	4	7.8429	79.4388	951.5796	0.0714	1.1673
57	2019	4	5.2857	91.0714	895.0520	0.0847	1.8949
9	2019	4	5.6571	91.0612	860.1449	0.6469	2.0500
72	2019	4	7.2143	87.4592	884.8296	0.8776	2.1102
26	2019	4	8.6286	89.2857	873.3153	1.6010	1.8796
7	2019	4	7.9571	86.7551	865.1092	2.3449	2.0071
83	2019	4	12.5714	78.9490	954.5194	0.7327	0.5327
76	2019	4	6.0429	87.2857	929.7551	0.0000	1.5776
36	2019	4	7.6571	81.6837	939.8673	0.2857	1.5122
81	2019	4	6.5714	85.0816	950.8714	0.0357	1.1398
15	2019	4	6.1143	83.2143	928.8122	0.8020	1.1867
32	2019	4	5.6571	89.1633	882.2622	0.4490	2.3245
73	2019	4	8.4429	78.2245	975.3347	0.0571	0.6296
71	2019	4	7.6571	81.6837	939.8673	0.2857	1.5122
41	2019	4	5.7857	82.8367	881.7449	0.6214	1.1786
10	2019	4	7.5143	84.1429	977.3847	0.0000	0.8622
23	2019	4	4.3857	76.3673	777.9582	5.5582	1.9673
27	2019	4	7.9571	86.7551	865.1092	2.3449	2.0071
60	2019	4	6.5714	85.0816	950.8714	0.0357	1.1398
53	2019	4	5.6571	91.0612	860.1449	0.6469	2.0500
66	2019	4	7.1429	90.4592	905.6071	0.6245	1.9357
59	2019	4	5.2857	91.0714	895.0520	0.0847	1.8949
61	2019	4	8.4429	78.2245	975.3347	0.0571	0.6296
84	2019	4	8.4429	78.2245	975.3347	0.0571	0.6296
38	2019	4	5.2857	91.0714	895.0520	0.0847	1.8949
87	2019	4	8.0571	87.4490	907.9204	0.5571	1.9541
34	2019	4	5.2857	91.0714	895.0520	0.0847	1.8949
29	2019	4	7.8429	79.4388	951.5796	0.0714	1.1673
5	2019	4	5.7143	92.4082	836.9480	2.6480	1.7990
8	2019	4	5.6571	91.0612	860.1449	0.6469	2.0500
12	2019	4	5.7143	92.4082	836.9480	2.6480	1.7990

13	2019	4	12.5714	78.9490	954.5194	0.7327	0.5327
18	2019	4	7.3857	82.5612	977.3980	0.7265	0.8061
33	2019	4	6.2571	82.3571	910.9735	0.2388	1.4837
56	2019	4	10.1857	85.5510	990.3612	0.6571	0.6041
77	2019	4	6.1143	83.2143	928.8122	0.8020	1.1867
54	2019	4	5.7143	92.4082	836.9480	2.6480	1.7990
21	2019	4	6.2571	82.3571	910.9735	0.2388	1.4837
68	2019	4	7.7571	78.1633	983.5122	0.7224	1.2265
74	2019	4	8.4429	78.2245	975.3347	0.0571	0.6296
88	2019	4	5.6571	89.1633	882.2622	0.4490	2.3245
16	2019	4	6.0429	87.2857	929.7551	0.0000	1.5776
30	2019	4	7.1429	90.4592	905.6071	0.6245	1.9357
6	2019	4	7.7571	78.1633	983.5122	0.7224	1.2265
49	2019	4	7.8429	79.4388	951.5796	0.0714	1.1673
22	2019	4	5.6571	89.1633	882.2622	0.4490	2.3245
45	2019	4	7.6857	74.2551	821.3490	5.2286	1.8500
58	2019	4	7.8429	79.4388	951.5796	0.0714	1.1673
37	2019	4	7.7571	78.1633	983.5122	0.7224	1.2265
17	2019	4	6.3714	87.1633	911.2745	0.2888	2.6418
55	2019	4	7.2143	87.4592	884.8296	0.8776	2.1102
46	2019	4	6.0429	87.2857	929.7551	0.0000	1.5776
86	2019	4	6.3000	86.5306	872.8724	0.4214	1.4806
2	2019	4	6.3000	86.5306	872.8724	0.4214	1.4806
4	2019	4	6.2571	82.3571	910.9735	0.2388	1.4837
47	2019	4	12.1286	80.6020	970.0429	0.5520	0.5551
82	2019	4	5.6571	89.1633	882.2622	0.4490	2.3245
19	2019	4	10.7429	78.7755	972.3296	0.4582	1.0010
20	2019	4	5.6571	91.0612	860.1449	0.6469	2.0500
80	2019	4	5.6571	89.1633	882.2622	0.4490	2.3245
3	2019	4	12.5714	78.9490	954.5194	0.7327	0.5327
52	2019	4	6.3714	87.1633	911.2745	0.2888	2.6418
70	2019	4	6.8000	85.5306	919.8980	0.0000	1.1735
64	2019	4	4.3857	76.3673	777.9582	5.5582	1.9673
48	2019	4	6.1143	83.2143	928.8122	0.8020	1.1867
65	2019	4	6.3714	87.1633	911.2745	0.2888	2.6418
44	2019	4	6.8000	85.5306	919.8980	0.0000	1.1735
75	2019	4	4.3857	76.3673	777.9582	5.5582	1.9673
40	2019	4	7.3714	88.8367	957.1163	0.3878	1.4449
11	2019	4	7.2143	87.4592	884.8296	0.8776	2.1102
35	2019	4	6.5714	85.0816	950.8714	0.0357	1.1398
78	2019	4	8.0571	87.4490	907.9204	0.5571	1.9541
28	2019	4	7.6571	81.6837	939.8673	0.2857	1.5122
39	2019	4	6.3714	87.1633	911.2745	0.2888	2.6418
24	2019	4	7.8429	79.4388	951.5796	0.0714	1.1673
63	2019	4	7.3714	88.8367	957.1163	0.3878	1.4449
62	2019	4	5.7857	82.8367	881.7449	0.6214	1.1786
1	2019	4	5.6571	89.1633	882.2622	0.4490	2.3245
31	2019	5	5.2714	86.9490	851.6551	0.8010	1.0051
79	2019	5	6.1286	75.1735	982.8429	0.8143	1.2990
51	2019	5	6.0857	82.2857	950.2306	0.2531	1.3337

14	2019	5	9.4857	89.3367	905.2337	0.9184	2.4153
67	2019	5	7.7000	87.6122	910.7643	0.5092	2.4388
42	2019	5	7.3571	88.8776	881.8327	0.3112	2.2469
50	2019	5	6.2000	80.7245	910.4653	0.4541	1.4673
43	2019	5	7.3571	88.8776	881.8327	0.3112	2.2469
85	2019	5	6.3143	84.3776	919.3531	0.0000	1.1704
25	2019	5	11.3143	80.3265	989.4490	0.9031	0.7959
69	2019	5	7.6429	78.0204	950.9245	0.1051	1.1327
57	2019	5	6.2429	90.0408	894.5551	0.2163	1.8653
9	2019	5	8.7143	90.7347	859.7898	0.3541	2.1357
72	2019	5	11.2571	86.6633	884.4306	0.6184	1.8357
26	2019	5	14.5000	84.7347	873.0714	2.8786	2.1806
7	2019	5	14.1571	81.8571	864.8041	3.9306	2.1796
83	2019	5	16.1286	74.5102	953.6541	1.8663	0.6082
76	2019	5	5.1286	85.4694	929.1816	0.0990	1.7663
36	2019	5	7.9286	80.9898	939.1480	0.5980	1.5265
81	2019	5	6.0857	82.2857	950.2306	0.2531	1.3337
15	2019	5	5.2571	80.8673	928.3235	0.6847	1.2867
32	2019	5	7.3571	88.8776	881.8327	0.3112	2.2469
73	2019	5	7.1429	75.9796	974.6337	0.1082	0.6745
71	2019	5	7.9286	80.9898	939.1480	0.5980	1.5265
41	2019	5	6.3429	83.1837	881.2582	0.5571	1.2051
10	2019	5	7.8000	80.1735	976.6439	0.1796	0.9673
23	2019	5	9.3286	72.9490	777.8214	6.2541	2.1173
27	2019	5	14.1571	81.8571	864.8041	3.9306	2.1796
60	2019	5	6.0857	82.2857	950.2306	0.2531	1.3337
53	2019	5	8.7143	90.7347	859.7898	0.3541	2.1357
66	2019	5	9.4857	89.3367	905.2337	0.9184	2.4153
59	2019	5	6.2429	90.0408	894.5551	0.2163	1.8653
61	2019	5	7.1429	75.9796	974.6337	0.1082	0.6745
84	2019	5	7.1429	75.9796	974.6337	0.1082	0.6745
38	2019	5	6.2429	90.0408	894.5551	0.2163	1.8653
87	2019	5	9.9286	85.0408	907.3969	0.4714	1.8755
34	2019	5	6.2429	90.0408	894.5551	0.2163	1.8653
29	2019	5	7.6429	78.0204	950.9245	0.1051	1.1327
5	2019	5	12.1714	87.8061	836.7337	3.9990	1.9837
8	2019	5	8.7143	90.7347	859.7898	0.3541	2.1357
12	2019	5	12.1714	87.8061	836.7337	3.9990	1.9837
13	2019	5	16.1286	74.5102	953.6541	1.8663	0.6082
18	2019	5	5.6286	80.4694	976.8082	0.6051	0.7388
33	2019	5	6.2000	80.7245	910.4653	0.4541	1.4673
56	2019	5	11.3143	80.3265	989.4490	0.9031	0.7959
77	2019	5	5.2571	80.8673	928.3235	0.6847	1.2867
54	2019	5	12.1714	87.8061	836.7337	3.9990	1.9837
21	2019	5	6.2000	80.7245	910.4653	0.4541	1.4673
68	2019	5	6.1286	75.1735	982.8429	0.8143	1.2990
74	2019	5	7.1429	75.9796	974.6337	0.1082	0.6745
88	2019	5	7.3571	88.8776	881.8327	0.3112	2.2469
16	2019	5	5.1286	85.4694	929.1816	0.0990	1.7663
30	2019	5	9.4857	89.3367	905.2337	0.9184	2.4153

6	2019	5	6.1286	75.1735	982.8429	0.8143	1.2990
49	2019	5	7.6429	78.0204	950.9245	0.1051	1.1327
22	2019	5	7.3571	88.8776	881.8327	0.3112	2.2469
45	2019	5	13.9714	68.4592	821.2337	5.5520	2.3602
58	2019	5	7.6429	78.0204	950.9245	0.1051	1.1327
37	2019	5	6.1286	75.1735	982.8429	0.8143	1.2990
17	2019	5	7.7000	87.6122	910.7643	0.5092	2.4388
55	2019	5	11.2571	86.6633	884.4306	0.6184	1.8357
46	2019	5	5.1286	85.4694	929.1816	0.0990	1.7663
86	2019	5	8.2143	86.7245	872.4000	0.3204	1.3112
2	2019	5	8.2143	86.7245	872.4000	0.3204	1.3112
4	2019	5	6.2000	80.7245	910.4653	0.4541	1.4673
47	2019	5	15.0714	76.7143	969.0184	0.9765	0.6592
82	2019	5	7.3571	88.8776	881.8327	0.3112	2.2469
19	2019	5	12.2714	77.3061	971.5622	0.5571	0.7857
20	2019	5	8.7143	90.7347	859.7898	0.3541	2.1357
80	2019	5	7.3571	88.8776	881.8327	0.3112	2.2469
3	2019	5	16.1286	74.5102	953.6541	1.8663	0.6082
52	2019	5	7.7000	87.6122	910.7643	0.5092	2.4388
70	2019	5	6.3143	84.3776	919.3531	0.0000	1.1704
64	2019	5	9.3286	72.9490	777.8214	6.2541	2.1173
48	2019	5	5.2571	80.8673	928.3235	0.6847	1.2867
65	2019	5	7.7000	87.6122	910.7643	0.5092	2.4388
44	2019	5	6.3143	84.3776	919.3531	0.0000	1.1704
75	2019	5	9.3286	72.9490	777.8214	6.2541	2.1173
40	2019	5	8.3714	84.2959	956.4969	0.8092	1.4020
11	2019	5	11.2571	86.6633	884.4306	0.6184	1.8357
35	2019	5	6.0857	82.2857	950.2306	0.2531	1.3337
78	2019	5	9.9286	85.0408	907.3969	0.4714	1.8755
28	2019	5	7.9286	80.9898	939.1480	0.5980	1.5265
39	2019	5	7.7000	87.6122	910.7643	0.5092	2.4388
24	2019	5	7.6429	78.0204	950.9245	0.1051	1.1327
63	2019	5	8.3714	84.2959	956.4969	0.8092	1.4020
62	2019	5	6.3429	83.1837	881.2582	0.5571	1.2051
1	2019	5	7.3571	88.8776	881.8327	0.3112	2.2469
31	2019	6	8.4286	80.7245	849.1816	2.3969	1.1724
79	2019	6	6.6571	75.4796	979.7245	1.4296	1.7245
51	2019	6	5.9429	81.6837	947.1245	1.6367	1.8163
14	2019	6	8.2857	90.8265	902.5602	1.5765	2.4816
67	2019	6	7.0571	88.1735	907.9510	1.2653	3.1398
42	2019	6	7.1857	86.1429	879.1633	1.9327	2.6724
50	2019	6	7.8286	80.3265	907.4367	1.7102	1.7184
43	2019	6	7.1857	86.1429	879.1633	1.9327	2.6724
85	2019	6	9.2714	82.8673	916.2551	1.2224	1.2245
25	2019	6	10.8857	75.1224	986.0959	1.3092	1.3439
69	2019	6	8.6143	76.5612	947.6857	0.9347	1.4765
57	2019	6	7.3429	86.2143	891.6337	1.9480	2.3867
9	2019	6	9.1571	85.7551	857.3541	2.4765	2.4163
72	2019	6	11.1143	82.0408	881.7673	2.4653	2.1398
26	2019	6	17.6000	70.7551	870.5367	6.6724	2.7561

7	2019	6	14.6143	69.5918	862.3510	7.0286	2.6000
83	2019	6	16.2000	72.7857	950.1571	4.1082	0.8296
76	2019	6	6.6000	88.1020	926.0102	1.4112	2.0480
36	2019	6	7.5429	80.1429	936.0704	2.0571	1.7684
81	2019	6	5.9429	81.6837	947.1245	1.6367	1.8163
15	2019	6	7.3857	82.7551	925.2112	1.3265	1.4847
32	2019	6	7.1857	86.1429	879.1633	1.9327	2.6724
73	2019	6	8.9714	76.4388	971.2398	1.2531	0.9276
71	2019	6	7.5429	80.1429	936.0704	2.0571	1.7684
41	2019	6	7.9286	79.5918	878.5286	1.9439	1.3020
10	2019	6	7.3143	76.9490	973.4265	1.3347	1.4592
23	2019	6	10.8000	56.4388	776.3796	8.4949	2.3837
27	2019	6	14.6143	69.5918	862.3510	7.0286	2.6000
60	2019	6	5.9429	81.6837	947.1245	1.6367	1.8163
53	2019	6	9.1571	85.7551	857.3541	2.4765	2.4163
66	2019	6	8.2857	90.8265	902.5602	1.5765	2.4816
59	2019	6	7.3429	86.2143	891.6337	1.9480	2.3867
61	2019	6	8.9714	76.4388	971.2398	1.2531	0.9276
84	2019	6	8.9714	76.4388	971.2398	1.2531	0.9276
38	2019	6	7.3429	86.2143	891.6337	1.9480	2.3867
87	2019	6	10.2571	80.4082	904.5163	2.0643	2.2745
34	2019	6	7.3429	86.2143	891.6337	1.9480	2.3867
29	2019	6	8.6143	76.5612	947.6857	0.9347	1.4765
5	2019	6	12.6857	71.6020	834.5724	7.5827	2.6010
8	2019	6	9.1571	85.7551	857.3541	2.4765	2.4163
12	2019	6	12.6857	71.6020	834.5724	7.5827	2.6010
13	2019	6	16.2000	72.7857	950.1571	4.1082	0.8296
18	2019	6	7.0429	81.0714	973.7133	1.0041	0.9857
33	2019	6	7.8286	80.3265	907.4367	1.7102	1.7184
56	2019	6	10.8857	75.1224	986.0959	1.3092	1.3439
77	2019	6	7.3857	82.7551	925.2112	1.3265	1.4847
54	2019	6	12.6857	71.6020	834.5724	7.5827	2.6010
21	2019	6	7.8286	80.3265	907.4367	1.7102	1.7184
68	2019	6	6.6571	75.4796	979.7245	1.4296	1.7245
74	2019	6	8.9714	76.4388	971.2398	1.2531	0.9276
88	2019	6	7.1857	86.1429	879.1633	1.9327	2.6724
16	2019	6	6.6000	88.1020	926.0102	1.4112	2.0480
30	2019	6	8.2857	90.8265	902.5602	1.5765	2.4816
6	2019	6	6.6571	75.4796	979.7245	1.4296	1.7245
49	2019	6	8.6143	76.5612	947.6857	0.9347	1.4765
22	2019	6	7.1857	86.1429	879.1633	1.9327	2.6724
45	2019	6	15.9286	51.4796	819.4949	8.3837	2.8367
58	2019	6	8.6143	76.5612	947.6857	0.9347	1.4765
37	2019	6	6.6571	75.4796	979.7245	1.4296	1.7245
17	2019	6	7.0571	88.1735	907.9510	1.2653	3.1398
55	2019	6	11.1143	82.0408	881.7673	2.4653	2.1398
46	2019	6	6.6000	88.1020	926.0102	1.4112	2.0480
86	2019	6	8.9571	82.3469	869.7755	1.1173	1.5133
2	2019	6	8.9571	82.3469	869.7755	1.1173	1.5133
4	2019	6	7.8286	80.3265	907.4367	1.7102	1.7184

47	2019	6	14.2714	74.5714	965.5041	2.3520	0.7878
82	2019	6	7.1857	86.1429	879.1633	1.9327	2.6724
19	2019	6	12.2571	75.9388	968.3010	1.1541	1.0041
20	2019	6	9.1571	85.7551	857.3541	2.4765	2.4163
80	2019	6	7.1857	86.1429	879.1633	1.9327	2.6724
3	2019	6	16.2000	72.7857	950.1571	4.1082	0.8296
52	2019	6	7.0571	88.1735	907.9510	1.2653	3.1398
70	2019	6	9.2714	82.8673	916.2551	1.2224	1.2245
64	2019	6	10.8000	56.4388	776.3796	8.4949	2.3837
48	2019	6	7.3857	82.7551	925.2112	1.3265	1.4847
65	2019	6	7.0571	88.1735	907.9510	1.2653	3.1398
44	2019	6	9.2714	82.8673	916.2551	1.2224	1.2245
75	2019	6	10.8000	56.4388	776.3796	8.4949	2.3837
40	2019	6	7.4000	80.8061	953.5684	1.6194	1.8337
11	2019	6	11.1143	82.0408	881.7673	2.4653	2.1398
35	2019	6	5.9429	81.6837	947.1245	1.6367	1.8163
78	2019	6	10.2571	80.4082	904.5163	2.0643	2.2745
28	2019	6	7.5429	80.1429	936.0704	2.0571	1.7684
39	2019	6	7.0571	88.1735	907.9510	1.2653	3.1398
24	2019	6	8.6143	76.5612	947.6857	0.9347	1.4765
63	2019	6	7.4000	80.8061	953.5684	1.6194	1.8337
62	2019	6	7.9286	79.5918	878.5286	1.9439	1.3020
1	2019	6	7.1857	86.1429	879.1633	1.9327	2.6724
31	2019	7	3.7286	79.2755	848.6459	3.1163	1.2776
79	2019	7	2.3286	80.4694	980.2449	1.4265	1.9582
51	2019	7	1.1571	86.0000	947.3786	1.6684	1.9531
14	2019	7	2.6857	92.6531	902.1939	1.6959	2.0490
67	2019	7	1.5571	89.7143	907.8378	1.3480	3.3510
42	2019	7	2.6000	87.1735	878.8255	2.1724	2.7888
50	2019	7	3.7571	79.9796	907.1235	1.8102	1.9276
43	2019	7	2.6000	87.1735	878.8255	2.1724	2.7888
85	2019	7	4.6857	80.9388	915.8898	1.6500	1.3031
25	2019	7	5.4714	77.4490	986.4969	1.4041	1.5449
69	2019	7	3.2000	78.9286	947.8378	1.1449	1.7143
57	2019	7	2.0286	86.8265	891.3673	2.1806	2.3337
9	2019	7	4.4714	85.3265	856.8653	3.0378	2.4622
72	2019	7	6.3714	82.2653	881.2071	3.3459	2.3612
26	2019	7	12.2571	66.3469	869.7122	7.5357	2.7061
7	2019	7	10.1714	68.7653	861.6704	7.4684	2.4959
83	2019	7	11.1143	74.5510	949.6684	3.9694	0.9959
76	2019	7	2.1857	90.0306	925.9500	1.6010	2.1765
36	2019	7	1.8714	82.8265	936.2520	1.9480	1.9806
81	2019	7	1.1571	86.0000	947.3786	1.6684	1.9531
15	2019	7	3.3714	83.2959	925.0255	1.4888	1.7990
32	2019	7	2.6000	87.1735	878.8255	2.1724	2.7888
73	2019	7	4.5571	77.2755	971.4367	1.4490	1.1541
71	2019	7	1.8714	82.8265	936.2520	1.9480	1.9806
41	2019	7	3.1429	79.7245	878.2133	2.2051	1.4224
10	2019	7	2.3000	82.2449	973.8929	1.3847	1.6296
23	2019	7	9.9429	44.8571	776.1163	9.3980	2.4867

27	2019	7	10.1714	68.7653	861.6704	7.4684	2.4959
60	2019	7	1.1571	86.0000	947.3786	1.6684	1.9531
53	2019	7	4.4714	85.3265	856.8653	3.0378	2.4622
66	2019	7	2.6857	92.6531	902.1939	1.6959	2.0490
59	2019	7	2.0286	86.8265	891.3673	2.1806	2.3337
61	2019	7	4.5571	77.2755	971.4367	1.4490	1.1541
84	2019	7	4.5571	77.2755	971.4367	1.4490	1.1541
38	2019	7	2.0286	86.8265	891.3673	2.1806	2.3337
87	2019	7	5.2571	80.1122	904.1755	2.5724	2.6653
34	2019	7	2.0286	86.8265	891.3673	2.1806	2.3337
29	2019	7	3.2000	78.9286	947.8378	1.1449	1.7143
5	2019	7	8.1143	68.8776	833.9622	7.7898	2.6347
8	2019	7	4.4714	85.3265	856.8653	3.0378	2.4622
12	2019	7	8.1143	68.8776	833.9622	7.7898	2.6347
13	2019	7	11.1143	74.5510	949.6684	3.9694	0.9959
18	2019	7	2.9429	82.3265	974.1204	1.1429	1.2265
33	2019	7	3.7571	79.9796	907.1235	1.8102	1.9276
56	2019	7	5.4714	77.4490	986.4969	1.4041	1.5449
77	2019	7	3.3714	83.2959	925.0255	1.4888	1.7990
54	2019	7	8.1143	68.8776	833.9622	7.7898	2.6347
21	2019	7	3.7571	79.9796	907.1235	1.8102	1.9276
68	2019	7	2.3286	80.4694	980.2449	1.4265	1.9582
74	2019	7	4.5571	77.2755	971.4367	1.4490	1.1541
88	2019	7	2.6000	87.1735	878.8255	2.1724	2.7888
16	2019	7	2.1857	90.0306	925.9500	1.6010	2.1765
30	2019	7	2.6857	92.6531	902.1939	1.6959	2.0490
6	2019	7	2.3286	80.4694	980.2449	1.4265	1.9582
49	2019	7	3.2000	78.9286	947.8378	1.1449	1.7143
22	2019	7	2.6000	87.1735	878.8255	2.1724	2.7888
45	2019	7	15.2857	41.9898	818.9816	9.3684	2.8459
58	2019	7	3.2000	78.9286	947.8378	1.1449	1.7143
37	2019	7	2.3286	80.4694	980.2449	1.4265	1.9582
17	2019	7	1.5571	89.7143	907.8378	1.3480	3.3510
55	2019	7	6.3714	82.2653	881.2071	3.3459	2.3612
46	2019	7	2.1857	90.0306	925.9500	1.6010	2.1765
86	2019	7	5.2000	81.5918	869.3398	1.2612	1.5990
2	2019	7	5.2000	81.5918	869.3398	1.2612	1.5990
4	2019	7	3.7571	79.9796	907.1235	1.8102	1.9276
47	2019	7	8.9857	76.2143	965.4806	2.6694	0.8163
82	2019	7	2.6000	87.1735	878.8255	2.1724	2.7888
19	2019	7	6.4143	77.5612	968.2888	1.4929	1.4265
20	2019	7	4.4714	85.3265	856.8653	3.0378	2.4622
80	2019	7	2.6000	87.1735	878.8255	2.1724	2.7888
3	2019	7	11.1143	74.5510	949.6684	3.9694	0.9959
52	2019	7	1.5571	89.7143	907.8378	1.3480	3.3510
70	2019	7	4.6857	80.9388	915.8898	1.6500	1.3031
64	2019	7	9.9429	44.8571	776.1163	9.3980	2.4867
48	2019	7	3.3714	83.2959	925.0255	1.4888	1.7990
65	2019	7	1.5571	89.7143	907.8378	1.3480	3.3510
44	2019	7	4.6857	80.9388	915.8898	1.6500	1.3031

75	2019	7	9.9429	44.8571	776.1163	9.3980	2.4867
40	2019	7	1.5286	85.8469	953.7827	1.4265	2.1541
11	2019	7	6.3714	82.2653	881.2071	3.3459	2.3612
35	2019	7	1.1571	86.0000	947.3786	1.6684	1.9531
78	2019	7	5.2571	80.1122	904.1755	2.5724	2.6653
28	2019	7	1.8714	82.8265	936.2520	1.9480	1.9806
39	2019	7	1.5571	89.7143	907.8378	1.3480	3.3510
24	2019	7	3.2000	78.9286	947.8378	1.1449	1.7143
63	2019	7	1.5286	85.8469	953.7827	1.4265	2.1541
62	2019	7	3.1429	79.7245	878.2133	2.2051	1.4224
1	2019	7	2.6000	87.1735	878.8255	2.1724	2.7888
31	2019	8	3.6714	89.5204	849.0245	1.0980	1.1367
79	2019	8	3.7714	86.5510	982.3684	0.2194	1.9327
51	2019	8	2.2571	92.9184	949.1265	0.2490	1.8010
14	2019	8	3.3571	97.2041	902.7684	0.6694	1.6939
67	2019	8	2.3143	96.8367	908.8296	0.3827	2.5306
42	2019	8	3.0286	93.9796	879.3673	0.4633	2.5500
50	2019	8	4.6714	82.3878	908.1898	0.3153	1.8612
43	2019	8	3.0286	93.9796	879.3673	0.4633	2.5500
85	2019	8	5.6143	84.2653	916.9643	0.4276	1.2173
25	2019	8	6.4857	85.6429	988.4490	0.3888	1.1122
69	2019	8	4.1857	87.1122	949.5663	0.2500	1.3418
57	2019	8	2.7571	94.1429	892.3204	0.4878	1.7163
9	2019	8	4.6000	92.0816	857.0816	1.1245	2.1510
72	2019	8	6.6571	89.4592	881.4969	1.7469	2.3286
26	2019	8	11.0571	77.2959	869.6173	5.3816	2.5347
7	2019	8	9.6000	78.8980	861.6633	4.9347	2.2684
83	2019	8	11.4429	80.8878	950.5765	1.7939	0.9041
76	2019	8	3.2857	92.9388	927.4235	0.3133	2.1878
36	2019	8	3.0571	91.1429	937.7633	0.2643	1.9837
81	2019	8	2.2571	92.9184	949.1265	0.2490	1.8010
15	2019	8	4.4000	85.5612	926.3592	0.2510	1.7224
32	2019	8	3.0286	93.9796	879.3673	0.4633	2.5500
73	2019	8	5.5429	80.7041	973.3918	0.2857	1.1265
71	2019	8	3.0571	91.1429	937.7633	0.2643	1.9837
41	2019	8	3.6857	86.5816	878.9663	0.4633	1.4000
10	2019	8	3.5000	91.1531	975.9704	0.2347	1.2765
23	2019	8	6.8714	51.4898	775.5153	8.6857	2.4684
27	2019	8	9.6000	78.8980	861.6633	4.9347	2.2684
60	2019	8	2.2571	92.9184	949.1265	0.2490	1.8010
53	2019	8	4.6000	92.0816	857.0816	1.1245	2.1510
66	2019	8	3.3571	97.2041	902.7684	0.6694	1.6939
59	2019	8	2.7571	94.1429	892.3204	0.4878	1.7163
61	2019	8	5.5429	80.7041	973.3918	0.2857	1.1265
84	2019	8	5.5429	80.7041	973.3918	0.2857	1.1265
38	2019	8	2.7571	94.1429	892.3204	0.4878	1.7163
87	2019	8	6.1143	87.0714	904.8704	0.9857	2.5051
34	2019	8	2.7571	94.1429	892.3204	0.4878	1.7163
29	2019	8	4.1857	87.1122	949.5663	0.2500	1.3418
5	2019	8	7.4857	80.9082	833.7980	4.9245	2.4755

8	2019	8	4.6000	92.0816	857.0816	1.1245	2.1510
12	2019	8	7.4857	80.9082	833.7980	4.9245	2.4755
13	2019	8	11.4429	80.8878	950.5765	1.7939	0.9041
18	2019	8	4.1714	85.8776	976.0918	0.2051	1.1918
33	2019	8	4.6714	82.3878	908.1898	0.3153	1.8612
56	2019	8	6.4857	85.6429	988.4490	0.3888	1.1122
77	2019	8	4.4000	85.5612	926.3592	0.2510	1.7224
54	2019	8	7.4857	80.9082	833.7980	4.9245	2.4755
21	2019	8	4.6714	82.3878	908.1898	0.3153	1.8612
68	2019	8	3.7714	86.5510	982.3684	0.2194	1.9327
74	2019	8	5.5429	80.7041	973.3918	0.2857	1.1265
88	2019	8	3.0286	93.9796	879.3673	0.4633	2.5500
16	2019	8	3.2857	92.9388	927.4235	0.3133	2.1878
30	2019	8	3.3571	97.2041	902.7684	0.6694	1.6939
6	2019	8	3.7714	86.5510	982.3684	0.2194	1.9327
49	2019	8	4.1857	87.1122	949.5663	0.2500	1.3418
22	2019	8	3.0286	93.9796	879.3673	0.4633	2.5500
45	2019	8	11.1286	46.0408	818.4367	8.9010	2.9571
58	2019	8	4.1857	87.1122	949.5663	0.2500	1.3418
37	2019	8	3.7714	86.5510	982.3684	0.2194	1.9327
17	2019	8	2.3143	96.8367	908.8296	0.3827	2.5306
55	2019	8	6.6571	89.4592	881.4969	1.7469	2.3286
46	2019	8	3.2857	92.9388	927.4235	0.3133	2.1878
86	2019	8	4.5857	88.2143	869.8786	0.5051	1.2276
2	2019	8	4.5857	88.2143	869.8786	0.5051	1.2276
4	2019	8	4.6714	82.3878	908.1898	0.3153	1.8612
47	2019	8	10.1143	81.5408	966.8214	0.8918	0.7582
82	2019	8	3.0286	93.9796	879.3673	0.4633	2.5500
19	2019	8	7.1857	82.2959	969.5949	0.5184	1.6000
20	2019	8	4.6000	92.0816	857.0816	1.1245	2.1510
80	2019	8	3.0286	93.9796	879.3673	0.4633	2.5500
3	2019	8	11.4429	80.8878	950.5765	1.7939	0.9041
52	2019	8	2.3143	96.8367	908.8296	0.3827	2.5306
70	2019	8	5.6143	84.2653	916.9643	0.4276	1.2173
64	2019	8	6.8714	51.4898	775.5153	8.6857	2.4684
48	2019	8	4.4000	85.5612	926.3592	0.2510	1.7224
65	2019	8	2.3143	96.8367	908.8296	0.3827	2.5306
44	2019	8	5.6143	84.2653	916.9643	0.4276	1.2173
75	2019	8	6.8714	51.4898	775.5153	8.6857	2.4684
40	2019	8	2.4571	94.4490	955.3306	0.2316	1.9418
11	2019	8	6.6571	89.4592	881.4969	1.7469	2.3286
35	2019	8	2.2571	92.9184	949.1265	0.2490	1.8010
78	2019	8	6.1143	87.0714	904.8704	0.9857	2.5051
28	2019	8	3.0571	91.1429	937.7633	0.2643	1.9837
39	2019	8	2.3143	96.8367	908.8296	0.3827	2.5306
24	2019	8	4.1857	87.1122	949.5663	0.2500	1.3418
63	2019	8	2.4571	94.4490	955.3306	0.2316	1.9418
62	2019	8	3.6857	86.5816	878.9663	0.4633	1.4000
1	2019	8	3.0286	93.9796	879.3673	0.4633	2.5500
31	2019	9	6.4714	91.7143	848.2163	0.2173	1.0388

79	2019	9	8.0286	84.0102	980.4133	0.2235	1.5847
51	2019	9	6.3286	91.0102	947.6194	0.2173	1.5061
14	2019	9	7.7857	95.0000	901.6388	0.8347	1.7867
67	2019	9	6.1429	94.5510	907.6796	0.2969	2.4561
42	2019	9	7.1429	91.7755	878.3867	0.3388	2.4949
50	2019	9	7.1571	81.5612	907.1806	0.1031	1.6571
43	2019	9	7.1429	91.7755	878.3867	0.3388	2.4949
85	2019	9	7.9000	83.6633	915.9337	0.1337	1.0878
25	2019	9	10.2143	86.3878	986.8480	0.4061	0.8582
69	2019	9	7.8000	85.5612	948.2000	0.3235	1.0418
57	2019	9	5.9429	93.1020	891.3153	0.2276	1.7082
9	2019	9	8.5429	90.8265	856.1755	0.9929	2.0408
72	2019	9	10.6286	87.0714	880.5704	1.4480	2.3367
26	2019	9	14.5857	80.2653	868.9061	4.1378	2.3949
7	2019	9	14.0286	78.9388	860.8867	3.9878	2.3959
83	2019	9	16.8714	78.7959	949.5786	2.1806	0.8929
76	2019	9	6.2571	90.9592	926.2622	0.0929	1.9010
36	2019	9	6.9286	88.4898	936.4194	0.2684	1.9184
81	2019	9	6.3286	91.0102	947.6194	0.2173	1.5061
15	2019	9	7.1571	83.6122	925.2133	0.3439	1.5031
32	2019	9	7.1429	91.7755	878.3867	0.3388	2.4949
73	2019	9	9.0143	80.5816	971.8571	0.0949	0.9184
71	2019	9	6.9286	88.4898	936.4194	0.2684	1.9184
41	2019	9	6.5143	86.0612	878.0439	0.0857	1.2510
10	2019	9	7.4000	89.1122	974.2184	0.3847	0.9745
23	2019	9	9.7714	62.1122	774.4643	7.7286	2.4735
27	2019	9	14.0286	78.9388	860.8867	3.9878	2.3959
60	2019	9	6.3286	91.0102	947.6194	0.2173	1.5061
53	2019	9	8.5429	90.8265	856.1755	0.9929	2.0408
66	2019	9	7.7857	95.0000	901.6388	0.8347	1.7867
59	2019	9	5.9429	93.1020	891.3153	0.2276	1.7082
61	2019	9	9.0143	80.5816	971.8571	0.0949	0.9184
84	2019	9	9.0143	80.5816	971.8571	0.0949	0.9184
38	2019	9	5.9429	93.1020	891.3153	0.2276	1.7082
87	2019	9	10.0429	83.9388	903.8143	1.0031	2.4929
34	2019	9	5.9429	93.1020	891.3153	0.2276	1.7082
29	2019	9	7.8000	85.5612	948.2000	0.3235	1.0418
5	2019	9	12.8714	80.4490	833.0102	3.7439	2.7551
8	2019	9	8.5429	90.8265	856.1755	0.9929	2.0408
12	2019	9	12.8714	80.4490	833.0102	3.7439	2.7551
13	2019	9	16.8714	78.7959	949.5786	2.1806	0.8929
18	2019	9	8.3429	84.5102	974.2112	0.0102	1.0092
33	2019	9	7.1571	81.5612	907.1806	0.1031	1.6571
56	2019	9	10.2143	86.3878	986.8480	0.4061	0.8582
77	2019	9	7.1571	83.6122	925.2133	0.3439	1.5031
54	2019	9	12.8714	80.4490	833.0102	3.7439	2.7551
21	2019	9	7.1571	81.5612	907.1806	0.1031	1.6571
68	2019	9	8.0286	84.0102	980.4133	0.2235	1.5847
74	2019	9	9.0143	80.5816	971.8571	0.0949	0.9184
88	2019	9	7.1429	91.7755	878.3867	0.3388	2.4949

16	2019	9	6.2571	90.9592	926.2622	0.0929	1.9010
30	2019	9	7.7857	95.0000	901.6388	0.8347	1.7867
6	2019	9	8.0286	84.0102	980.4133	0.2235	1.5847
49	2019	9	7.8000	85.5612	948.2000	0.3235	1.0418
22	2019	9	7.1429	91.7755	878.3867	0.3388	2.4949
45	2019	9	13.8857	57.3469	817.6061	7.8133	2.8255
58	2019	9	7.8000	85.5612	948.2000	0.3235	1.0418
37	2019	9	8.0286	84.0102	980.4133	0.2235	1.5847
17	2019	9	6.1429	94.5510	907.6796	0.2969	2.4561
55	2019	9	10.6286	87.0714	880.5704	1.4480	2.3367
46	2019	9	6.2571	90.9592	926.2622	0.0929	1.9010
86	2019	9	8.4143	87.8367	869.0122	0.5020	1.0520
2	2019	9	8.4143	87.8367	869.0122	0.5020	1.0520
4	2019	9	7.1571	81.5612	907.1806	0.1031	1.6571
47	2019	9	14.1857	78.9592	965.5684	0.7367	0.7071
82	2019	9	7.1429	91.7755	878.3867	0.3388	2.4949
19	2019	9	11.4286	80.5000	968.2765	0.3633	1.4500
20	2019	9	8.5429	90.8265	856.1755	0.9929	2.0408
80	2019	9	7.1429	91.7755	878.3867	0.3388	2.4949
3	2019	9	16.8714	78.7959	949.5786	2.1806	0.8929
52	2019	9	6.1429	94.5510	907.6796	0.2969	2.4561
70	2019	9	7.9000	83.6633	915.9337	0.1337	1.0878
64	2019	9	9.7714	62.1122	774.4643	7.7286	2.4735
48	2019	9	7.1571	83.6122	925.2133	0.3439	1.5031
65	2019	9	6.1429	94.5510	907.6796	0.2969	2.4561
44	2019	9	7.9000	83.6633	915.9337	0.1337	1.0878
75	2019	9	9.7714	62.1122	774.4643	7.7286	2.4735
40	2019	9	6.5714	93.0714	953.8031	0.0980	1.6398
11	2019	9	10.6286	87.0714	880.5704	1.4480	2.3367
35	2019	9	6.3286	91.0102	947.6194	0.2173	1.5061
78	2019	9	10.0429	83.9388	903.8143	1.0031	2.4929
28	2019	9	6.9286	88.4898	936.4194	0.2684	1.9184
39	2019	9	6.1429	94.5510	907.6796	0.2969	2.4561
24	2019	9	7.8000	85.5612	948.2000	0.3235	1.0418
63	2019	9	6.5714	93.0714	953.8031	0.0980	1.6398
62	2019	9	6.5143	86.0612	878.0439	0.0857	1.2510
1	2019	9	7.1429	91.7755	878.3867	0.3388	2.4949
31	2019	10	6.4000	86.5714	847.1500	0.7888	1.0520
79	2019	10	9.5571	82.6939	977.3653	0.5408	1.2092
51	2019	10	8.3000	89.3980	945.1255	0.6673	1.4276
14	2019	10	9.1714	92.2143	899.9245	1.2633	2.2878
67	2019	10	8.4714	91.9184	905.7592	0.6908	2.5316
42	2019	10	7.6143	87.3061	876.9612	0.5714	2.4643
50	2019	10	7.9714	80.9796	905.4867	0.7459	1.6959
43	2019	10	7.6143	87.3061	876.9612	0.5714	2.4643
85	2019	10	9.2429	81.2857	914.1796	0.8357	1.1082
25	2019	10	12.0000	86.2143	983.8755	0.9347	0.9643
69	2019	10	9.6857	84.1939	945.7214	0.6010	1.0704
57	2019	10	7.2143	90.0102	889.6694	0.8520	1.8582
9	2019	10	8.0143	85.8469	854.9949	1.8837	2.3235

72	2019	10	9.8714	82.4694	879.2010	2.0643	2.3041
26	2019	10	12.6857	73.9592	867.8888	4.8204	2.3918
7	2019	10	11.0286	73.8878	859.8286	4.8531	2.5378
83	2019	10	15.1857	73.7857	947.4541	3.2020	0.9031
76	2019	10	7.7714	89.5816	924.1500	0.2388	1.8735
36	2019	10	9.1000	86.2347	934.1051	0.9000	1.8214
81	2019	10	8.3000	89.3980	945.1255	0.6673	1.4276
15	2019	10	7.8714	83.2755	923.2102	1.1327	1.4663
32	2019	10	7.6143	87.3061	876.9612	0.5714	2.4643
73	2019	10	10.1571	80.4796	969.0888	0.3041	0.7990
71	2019	10	9.1000	86.2347	934.1051	0.9000	1.8214
41	2019	10	7.9143	82.0306	876.5214	0.8276	1.2612
10	2019	10	9.2714	87.3878	971.2908	0.8051	0.8949
23	2019	10	5.7000	62.9490	774.0602	6.6051	2.5224
27	2019	10	11.0286	73.8878	859.8286	4.8531	2.5378
60	2019	10	8.3000	89.3980	945.1255	0.6673	1.4276
53	2019	10	8.0143	85.8469	854.9949	1.8837	2.3235
66	2019	10	9.1714	92.2143	899.9245	1.2633	2.2878
59	2019	10	7.2143	90.0102	889.6694	0.8520	1.8582
61	2019	10	10.1571	80.4796	969.0888	0.3041	0.7990
84	2019	10	10.1571	80.4796	969.0888	0.3041	0.7990
38	2019	10	7.2143	90.0102	889.6694	0.8520	1.8582
87	2019	10	10.0286	80.2551	902.1561	1.4918	2.4939
34	2019	10	7.2143	90.0102	889.6694	0.8520	1.8582
29	2019	10	9.6857	84.1939	945.7214	0.6010	1.0704
5	2019	10	9.3000	71.6633	832.1439	4.6888	2.9071
8	2019	10	8.0143	85.8469	854.9949	1.8837	2.3235
12	2019	10	9.3000	71.6633	832.1439	4.6888	2.9071
13	2019	10	15.1857	73.7857	947.4541	3.2020	0.9031
18	2019	10	9.2714	82.5000	971.3306	0.1510	0.9327
33	2019	10	7.9714	80.9796	905.4867	0.7459	1.6959
56	2019	10	12.0000	86.2143	983.8755	0.9347	0.9643
77	2019	10	7.8714	83.2755	923.2102	1.1327	1.4663
54	2019	10	9.3000	71.6633	832.1439	4.6888	2.9071
21	2019	10	7.9714	80.9796	905.4867	0.7459	1.6959
68	2019	10	9.5571	82.6939	977.3653	0.5408	1.2092
74	2019	10	10.1571	80.4796	969.0888	0.3041	0.7990
88	2019	10	7.6143	87.3061	876.9612	0.5714	2.4643
16	2019	10	7.7714	89.5816	924.1500	0.2388	1.8735
30	2019	10	9.1714	92.2143	899.9245	1.2633	2.2878
6	2019	10	9.5571	82.6939	977.3653	0.5408	1.2092
49	2019	10	9.6857	84.1939	945.7214	0.6010	1.0704
22	2019	10	7.6143	87.3061	876.9612	0.5714	2.4643
45	2019	10	10.5571	60.0918	817.0378	6.9265	2.6255
58	2019	10	9.6857	84.1939	945.7214	0.6010	1.0704
37	2019	10	9.5571	82.6939	977.3653	0.5408	1.2092
17	2019	10	8.4714	91.9184	905.7592	0.6908	2.5316
55	2019	10	9.8714	82.4694	879.2010	2.0643	2.3041
46	2019	10	7.7714	89.5816	924.1500	0.2388	1.8735
86	2019	10	8.6857	82.4490	867.6316	0.8296	1.2245

2	2019	10	8.6857	82.4490	867.6316	0.8296	1.2245
4	2019	10	7.9714	80.9796	905.4867	0.7459	1.6959
47	2019	10	14.3143	77.0612	963.0796	1.4143	0.6316
82	2019	10	7.6143	87.3061	876.9612	0.5714	2.4643
19	2019	10	12.9143	79.7245	965.7459	0.9571	1.2071
20	2019	10	8.0143	85.8469	854.9949	1.8837	2.3235
80	2019	10	7.6143	87.3061	876.9612	0.5714	2.4643
3	2019	10	15.1857	73.7857	947.4541	3.2020	0.9031
52	2019	10	8.4714	91.9184	905.7592	0.6908	2.5316
70	2019	10	9.2429	81.2857	914.1796	0.8357	1.1082
64	2019	10	5.7000	62.9490	774.0602	6.6051	2.5224
48	2019	10	7.8714	83.2755	923.2102	1.1327	1.4663
65	2019	10	8.4714	91.9184	905.7592	0.6908	2.5316
44	2019	10	9.2429	81.2857	914.1796	0.8357	1.1082
75	2019	10	5.7000	62.9490	774.0602	6.6051	2.5224
40	2019	10	8.7714	91.7857	951.2061	0.4847	1.4367
11	2019	10	9.8714	82.4694	879.2010	2.0643	2.3041
35	2019	10	8.3000	89.3980	945.1255	0.6673	1.4276
78	2019	10	10.0286	80.2551	902.1561	1.4918	2.4939
28	2019	10	9.1000	86.2347	934.1051	0.9000	1.8214
39	2019	10	8.4714	91.9184	905.7592	0.6908	2.5316
24	2019	10	9.6857	84.1939	945.7214	0.6010	1.0704
63	2019	10	8.7714	91.7857	951.2061	0.4847	1.4367
62	2019	10	7.9143	82.0306	876.5214	0.8276	1.2612
1	2019	10	7.6143	87.3061	876.9612	0.5714	2.4643
31	2019	11	7.9429	83.8367	848.2796	0.7571	0.9786
79	2019	11	12.8857	80.7755	976.7480	1.4908	0.9061
51	2019	11	11.1571	86.9184	944.8408	1.2031	1.2643
14	2019	11	11.1571	87.4184	900.4143	1.4439	2.1663
67	2019	11	10.5143	87.8469	906.0071	1.2602	2.3816
42	2019	11	9.5857	84.8571	877.7551	0.5755	2.2806
50	2019	11	10.2286	80.3980	906.0316	0.9469	1.6857
43	2019	11	9.5857	84.8571	877.7551	0.5755	2.2806
85	2019	11	10.7714	80.4286	914.7296	0.7020	1.1041
25	2019	11	13.9143	85.4592	983.3112	1.9092	0.9735
69	2019	11	12.0143	82.5714	945.5694	1.3255	1.0684
57	2019	11	8.9714	88.8776	890.3214	0.9724	1.8418
9	2019	11	9.1429	83.1224	856.0102	1.6633	2.1990
72	2019	11	11.0714	80.5816	880.0633	1.6235	2.1551
26	2019	11	12.1857	74.9184	868.7735	3.9490	2.1714
7	2019	11	10.9571	75.6735	860.8163	3.5735	2.0980
83	2019	11	16.0571	72.8878	948.0592	2.6653	0.7531
76	2019	11	9.9714	88.0510	924.2796	0.9000	1.7367
36	2019	11	11.4286	83.9082	934.0112	1.7867	1.6204
81	2019	11	11.1571	86.9184	944.8408	1.2031	1.2643
15	2019	11	10.6714	83.8163	923.5010	1.6296	1.4122
32	2019	11	9.5857	84.8571	877.7551	0.5755	2.2806
73	2019	11	12.5000	81.0816	968.7561	1.2092	0.6990
71	2019	11	11.4286	83.9082	934.0112	1.7867	1.6204
41	2019	11	9.2143	80.2143	877.3490	1.0857	1.1235

10	2019	11	12.3714	83.1735	970.6867	1.8459	0.8653
23	2019	11	5.6429	73.6939	775.3327	3.9561	2.2143
27	2019	11	10.9571	75.6735	860.8163	3.5735	2.0980
60	2019	11	11.1571	86.9184	944.8408	1.2031	1.2643
53	2019	11	9.1429	83.1224	856.0102	1.6633	2.1990
66	2019	11	11.1571	87.4184	900.4143	1.4439	2.1663
59	2019	11	8.9714	88.8776	890.3214	0.9724	1.8418
61	2019	11	12.5000	81.0816	968.7561	1.2092	0.6990
84	2019	11	12.5000	81.0816	968.7561	1.2092	0.6990
38	2019	11	8.9714	88.8776	890.3214	0.9724	1.8418
87	2019	11	11.7714	80.1633	902.8296	1.1510	2.0245
34	2019	11	8.9714	88.8776	890.3214	0.9724	1.8418
29	2019	11	12.0143	82.5714	945.5694	1.3255	1.0684
5	2019	11	8.6429	77.2245	833.2306	3.2082	2.4500
8	2019	11	9.1429	83.1224	856.0102	1.6633	2.1990
12	2019	11	8.6429	77.2245	833.2306	3.2082	2.4500
13	2019	11	16.0571	72.8878	948.0592	2.6653	0.7531
18	2019	11	12.1571	82.8878	970.8388	1.0643	0.8684
33	2019	11	10.2286	80.3980	906.0316	0.9469	1.6857
56	2019	11	13.9143	85.4592	983.3112	1.9092	0.9735
77	2019	11	10.6714	83.8163	923.5010	1.6296	1.4122
54	2019	11	8.6429	77.2245	833.2306	3.2082	2.4500
21	2019	11	10.2286	80.3980	906.0316	0.9469	1.6857
68	2019	11	12.8857	80.7755	976.7480	1.4908	0.9061
74	2019	11	12.5000	81.0816	968.7561	1.2092	0.6990
88	2019	11	9.5857	84.8571	877.7551	0.5755	2.2806
16	2019	11	9.9714	88.0510	924.2796	0.9000	1.7367
30	2019	11	11.1571	87.4184	900.4143	1.4439	2.1663
6	2019	11	12.8857	80.7755	976.7480	1.4908	0.9061
49	2019	11	12.0143	82.5714	945.5694	1.3255	1.0684
22	2019	11	9.5857	84.8571	877.7551	0.5755	2.2806
45	2019	11	9.3571	69.1531	818.0469	4.2816	2.1643
58	2019	11	12.0143	82.5714	945.5694	1.3255	1.0684
37	2019	11	12.8857	80.7755	976.7480	1.4908	0.9061
17	2019	11	10.5143	87.8469	906.0071	1.2602	2.3816
55	2019	11	11.0714	80.5816	880.0633	1.6235	2.1551
46	2019	11	9.9714	88.0510	924.2796	0.9000	1.7367
86	2019	11	9.1571	81.7959	868.6112	0.5663	1.0673
2	2019	11	9.1571	81.7959	868.6112	0.5663	1.0673
4	2019	11	10.2286	80.3980	906.0316	0.9469	1.6857
47	2019	11	15.9429	76.5306	963.3031	1.5796	0.5643
82	2019	11	9.5857	84.8571	877.7551	0.5755	2.2806
19	2019	11	14.9286	76.3163	965.5143	1.8010	1.0776
20	2019	11	9.1429	83.1224	856.0102	1.6633	2.1990
80	2019	11	9.5857	84.8571	877.7551	0.5755	2.2806
3	2019	11	16.0571	72.8878	948.0592	2.6653	0.7531
52	2019	11	10.5143	87.8469	906.0071	1.2602	2.3816
70	2019	11	10.7714	80.4286	914.7296	0.7020	1.1041
64	2019	11	5.6429	73.6939	775.3327	3.9561	2.2143
48	2019	11	10.6714	83.8163	923.5010	1.6296	1.4122

65	2019	11	10.5143	87.8469	906.0071	1.2602	2.3816
44	2019	11	10.7714	80.4286	914.7296	0.7020	1.1041
75	2019	11	5.6429	73.6939	775.3327	3.9561	2.2143
40	2019	11	11.6714	87.8163	950.8245	1.9939	1.2755
11	2019	11	11.0714	80.5816	880.0633	1.6235	2.1551
35	2019	11	11.1571	86.9184	944.8408	1.2031	1.2643
78	2019	11	11.7714	80.1633	902.8296	1.1510	2.0245
28	2019	11	11.4286	83.9082	934.0112	1.7867	1.6204
39	2019	11	10.5143	87.8469	906.0071	1.2602	2.3816
24	2019	11	12.0143	82.5714	945.5694	1.3255	1.0684
63	2019	11	11.6714	87.8163	950.8245	1.9939	1.2755
62	2019	11	9.2143	80.2143	877.3490	1.0857	1.1235
1	2019	11	9.5857	84.8571	877.7551	0.5755	2.2806
31	2019	12	11.8286	79.2143	849.4704	1.6888	1.0582
79	2019	12	14.6857	76.1735	976.4908	2.6316	1.0735
51	2019	12	14.0714	81.1122	945.0000	2.0255	1.1816
14	2019	12	13.9286	83.3980	901.6357	2.0918	2.1480
67	2019	12	13.9286	82.6224	906.8398	1.9878	2.6388
42	2019	12	13.6000	80.2347	878.8776	2.2163	2.4929
50	2019	12	13.6143	76.7755	906.6051	1.9276	1.7520
43	2019	12	13.6000	80.2347	878.8776	2.2163	2.4929
85	2019	12	14.1000	77.7653	915.3214	0.9755	1.2102
25	2019	12	16.9714	82.1224	983.6857	2.3245	1.1194
69	2019	12	14.9429	76.7653	945.8388	2.6133	1.1684
57	2019	12	13.5429	83.3571	891.1439	2.1765	2.2163
9	2019	12	13.1286	79.7653	857.4602	2.2000	2.1112
72	2019	12	15.0857	77.3673	881.5194	1.9102	2.2133
26	2019	12	16.5143	77.6122	870.5214	2.4561	2.0194
7	2019	12	14.9286	77.5204	862.5082	2.8878	1.9745
83	2019	12	18.6714	73.5612	949.6806	2.5337	0.7184
76	2019	12	13.1571	82.1429	924.6622	2.3388	1.5347
36	2019	12	14.9857	78.0612	934.4724	2.6204	1.7653
81	2019	12	14.0714	81.1122	945.0000	2.0255	1.1816
15	2019	12	13.3143	79.0102	923.8235	1.9735	1.4969
32	2019	12	13.6000	80.2347	878.8776	2.2163	2.4929
73	2019	12	15.3286	77.9898	968.7306	2.7724	0.7827
71	2019	12	14.9857	78.0612	934.4724	2.6204	1.7653
41	2019	12	13.4143	75.6837	878.3765	1.1041	1.1663
10	2019	12	14.9571	77.2245	970.7041	2.4765	0.9898
23	2019	12	9.6143	78.7959	776.8939	3.8418	2.2459
27	2019	12	14.9286	77.5204	862.5082	2.8878	1.9745
60	2019	12	14.0714	81.1122	945.0000	2.0255	1.1816
53	2019	12	13.1286	79.7653	857.4602	2.2000	2.1112
66	2019	12	13.9286	83.3980	901.6357	2.0918	2.1480
59	2019	12	13.5429	83.3571	891.1439	2.1765	2.2163
61	2019	12	15.3286	77.9898	968.7306	2.7724	0.7827
84	2019	12	15.3286	77.9898	968.7306	2.7724	0.7827
38	2019	12	13.5429	83.3571	891.1439	2.1765	2.2163
87	2019	12	15.6286	76.1122	903.9306	2.3429	1.9449
34	2019	12	13.5429	83.3571	891.1439	2.1765	2.2163

29	2019	12	14.9429	76.7653	945.8388	2.6133	1.1684
5	2019	12	13.1857	81.8367	834.9133	2.6031	2.2755
8	2019	12	13.1286	79.7653	857.4602	2.2000	2.1112
12	2019	12	13.1857	81.8367	834.9133	2.6031	2.2755
13	2019	12	18.6714	73.5612	949.6806	2.5337	0.7184
18	2019	12	13.9429	80.4082	970.6735	2.1194	0.8694
33	2019	12	13.6143	76.7755	906.6051	1.9276	1.7520
56	2019	12	16.9714	82.1224	983.6857	2.3245	1.1194
77	2019	12	13.3143	79.0102	923.8235	1.9735	1.4969
54	2019	12	13.1857	81.8367	834.9133	2.6031	2.2755
21	2019	12	13.6143	76.7755	906.6051	1.9276	1.7520
68	2019	12	14.6857	76.1735	976.4908	2.6316	1.0735
74	2019	12	15.3286	77.9898	968.7306	2.7724	0.7827
88	2019	12	13.6000	80.2347	878.8776	2.2163	2.4929
16	2019	12	13.1571	82.1429	924.6622	2.3388	1.5347
30	2019	12	13.9286	83.3980	901.6357	2.0918	2.1480
6	2019	12	14.6857	76.1735	976.4908	2.6316	1.0735
49	2019	12	14.9429	76.7653	945.8388	2.6133	1.1684
22	2019	12	13.6000	80.2347	878.8776	2.2163	2.4929
45	2019	12	14.1857	75.5918	819.7041	3.4857	1.9990
58	2019	12	14.9429	76.7653	945.8388	2.6133	1.1684
37	2019	12	14.6857	76.1735	976.4908	2.6316	1.0735
17	2019	12	13.9286	82.6224	906.8398	1.9878	2.6388
55	2019	12	15.0857	77.3673	881.5194	1.9102	2.2133
46	2019	12	13.1571	82.1429	924.6622	2.3388	1.5347
86	2019	12	13.2429	79.3673	869.8367	1.4153	1.1184
2	2019	12	13.2429	79.3673	869.8367	1.4153	1.1184
4	2019	12	13.6143	76.7755	906.6051	1.9276	1.7520
47	2019	12	18.6000	73.3469	964.4704	1.8888	0.5592
82	2019	12	13.6000	80.2347	878.8776	2.2163	2.4929
19	2019	12	17.2714	73.7347	966.4449	2.1633	1.0847
20	2019	12	13.1286	79.7653	857.4602	2.2000	2.1112
80	2019	12	13.6000	80.2347	878.8776	2.2163	2.4929
3	2019	12	18.6714	73.5612	949.6806	2.5337	0.7184
52	2019	12	13.9286	82.6224	906.8398	1.9878	2.6388
70	2019	12	14.1000	77.7653	915.3214	0.9755	1.2102
64	2019	12	9.6143	78.7959	776.8939	3.8418	2.2459
48	2019	12	13.3143	79.0102	923.8235	1.9735	1.4969
65	2019	12	13.9286	82.6224	906.8398	1.9878	2.6388
44	2019	12	14.1000	77.7653	915.3214	0.9755	1.2102
75	2019	12	9.6143	78.7959	776.8939	3.8418	2.2459
40	2019	12	14.3286	82.8061	951.3214	3.0969	1.4806
11	2019	12	15.0857	77.3673	881.5194	1.9102	2.2133
35	2019	12	14.0714	81.1122	945.0000	2.0255	1.1816
78	2019	12	15.6286	76.1122	903.9306	2.3429	1.9449
28	2019	12	14.9857	78.0612	934.4724	2.6204	1.7653
39	2019	12	13.9286	82.6224	906.8398	1.9878	2.6388
24	2019	12	14.9429	76.7653	945.8388	2.6133	1.1684
63	2019	12	14.3286	82.8061	951.3214	3.0969	1.4806
62	2019	12	13.4143	75.6837	878.3765	1.1041	1.1663

1	2019	12	13.6000	80.2347	878.8776	2.2163	2.4929
31	2019	13	13.1000	72.2041	848.5949	3.0816	1.2827
79	2019	13	16.3429	74.6939	975.0694	2.8276	1.4255
51	2019	13	15.1714	78.3061	943.7724	3.0000	1.3878
14	2019	13	15.0286	84.0612	900.7643	2.4653	2.4735
67	2019	13	15.2286	81.4388	905.8122	2.3735	3.0235
42	2019	13	15.1857	75.6735	877.8867	3.7827	2.6439
50	2019	13	14.9571	73.5000	905.3337	3.6122	1.9245
43	2019	13	15.1857	75.6735	877.8867	3.7827	2.6439
85	2019	13	15.9143	74.0714	914.0745	1.8020	1.3153
25	2019	13	18.3286	80.3673	982.3929	2.2592	1.3357
69	2019	13	16.3714	74.3673	944.4847	3.2163	1.4000
57	2019	13	14.5571	77.6633	889.9673	3.9000	2.3827
9	2019	13	14.3000	76.4184	856.6418	2.9500	2.3735
72	2019	13	15.8286	74.5000	880.6357	2.9439	2.2816
26	2019	13	17.0429	72.7551	869.7796	3.8041	2.3673
7	2019	13	16.1714	72.8061	861.7337	4.5194	2.2714
83	2019	13	20.4143	70.3367	948.4735	3.1184	0.8878
76	2019	13	14.6000	77.3061	923.3439	3.5133	1.6837
36	2019	13	15.9857	74.9388	933.2133	3.3357	1.8592
81	2019	13	15.1714	78.3061	943.7724	3.0000	1.3878
15	2019	13	15.0714	74.8980	922.4418	2.8000	1.6133
32	2019	13	15.1857	75.6735	877.8867	3.7827	2.6439
73	2019	13	17.1857	73.1327	967.1582	3.7724	0.9755
71	2019	13	15.9857	74.9388	933.2133	3.3357	1.8592
41	2019	13	14.9000	69.3571	877.2500	2.1092	1.4010
10	2019	13	16.0286	77.3980	969.4163	2.6245	1.2153
23	2019	13	12.2857	67.9184	776.6010	6.3214	2.4622
27	2019	13	16.1714	72.8061	861.7337	4.5194	2.2714
60	2019	13	15.1714	78.3061	943.7724	3.0000	1.3878
53	2019	13	14.3000	76.4184	856.6418	2.9500	2.3735
66	2019	13	15.0286	84.0612	900.7643	2.4653	2.4735
59	2019	13	14.5571	77.6633	889.9673	3.9000	2.3827
61	2019	13	17.1857	73.1327	967.1582	3.7724	0.9755
84	2019	13	17.1857	73.1327	967.1582	3.7724	0.9755
38	2019	13	14.5571	77.6633	889.9673	3.9000	2.3827
87	2019	13	17.0714	71.3980	902.8327	3.4031	2.3153
34	2019	13	14.5571	77.6633	889.9673	3.9000	2.3827
29	2019	13	16.3714	74.3673	944.4847	3.2163	1.4000
5	2019	13	14.8286	74.6327	834.2398	4.7214	2.5990
8	2019	13	14.3000	76.4184	856.6418	2.9500	2.3735
12	2019	13	14.8286	74.6327	834.2398	4.7214	2.5990
13	2019	13	20.4143	70.3367	948.4735	3.1184	0.8878
18	2019	13	16.2857	77.1837	969.2255	2.6939	1.0265
33	2019	13	14.9571	73.5000	905.3337	3.6122	1.9245
56	2019	13	18.3286	80.3673	982.3929	2.2592	1.3357
77	2019	13	15.0714	74.8980	922.4418	2.8000	1.6133
54	2019	13	14.8286	74.6327	834.2398	4.7214	2.5990
21	2019	13	14.9571	73.5000	905.3337	3.6122	1.9245
68	2019	13	16.3429	74.6939	975.0694	2.8276	1.4255

74	2019	13	17.1857	73.1327	967.1582	3.7724	0.9755
88	2019	13	15.1857	75.6735	877.8867	3.7827	2.6439
16	2019	13	14.6000	77.3061	923.3439	3.5133	1.6837
30	2019	13	15.0286	84.0612	900.7643	2.4653	2.4735
6	2019	13	16.3429	74.6939	975.0694	2.8276	1.4255
49	2019	13	16.3714	74.3673	944.4847	3.2163	1.4000
22	2019	13	15.1857	75.6735	877.8867	3.7827	2.6439
45	2019	13	16.9143	62.5204	819.1327	6.0204	2.4296
58	2019	13	16.3714	74.3673	944.4847	3.2163	1.4000
37	2019	13	16.3429	74.6939	975.0694	2.8276	1.4255
17	2019	13	15.2286	81.4388	905.8122	2.3735	3.0235
55	2019	13	15.8286	74.5000	880.6357	2.9439	2.2816
46	2019	13	14.6000	77.3061	923.3439	3.5133	1.6837
86	2019	13	14.9000	73.4592	868.8061	2.5653	1.2449
2	2019	13	14.9000	73.4592	868.8061	2.5653	1.2449
4	2019	13	14.9571	73.5000	905.3337	3.6122	1.9245
47	2019	13	20.1714	70.3469	963.2337	2.2673	0.6704
82	2019	13	15.1857	75.6735	877.8867	3.7827	2.6439
19	2019	13	17.9000	75.4490	965.4653	2.2827	1.0898
20	2019	13	14.3000	76.4184	856.6418	2.9500	2.3735
80	2019	13	15.1857	75.6735	877.8867	3.7827	2.6439
3	2019	13	20.4143	70.3367	948.4735	3.1184	0.8878
52	2019	13	15.2286	81.4388	905.8122	2.3735	3.0235
70	2019	13	15.9143	74.0714	914.0745	1.8020	1.3153
64	2019	13	12.2857	67.9184	776.6010	6.3214	2.4622
48	2019	13	15.0714	74.8980	922.4418	2.8000	1.6133
65	2019	13	15.2286	81.4388	905.8122	2.3735	3.0235
44	2019	13	15.9143	74.0714	914.0745	1.8020	1.3153
75	2019	13	12.2857	67.9184	776.6010	6.3214	2.4622
40	2019	13	16.4143	82.2551	950.2296	3.2306	1.7582
11	2019	13	15.8286	74.5000	880.6357	2.9439	2.2816
35	2019	13	15.1714	78.3061	943.7724	3.0000	1.3878
78	2019	13	17.0714	71.3980	902.8327	3.4031	2.3153
28	2019	13	15.9857	74.9388	933.2133	3.3357	1.8592
39	2019	13	15.2286	81.4388	905.8122	2.3735	3.0235
24	2019	13	16.3714	74.3673	944.4847	3.2163	1.4000
63	2019	13	16.4143	82.2551	950.2296	3.2306	1.7582
62	2019	13	14.9000	69.3571	877.2500	2.1092	1.4010
1	2019	13	15.1857	75.6735	877.8867	3.7827	2.6439
31	2019	14	12.5286	72.4286	849.1663	3.0235	1.2582
79	2019	14	16.0429	76.5204	975.5449	2.5776	1.4541
51	2019	14	14.2143	80.0204	944.2286	2.8908	1.5010
14	2019	14	13.7714	86.6327	901.0276	2.2857	2.4031
67	2019	14	13.4286	83.9490	906.1827	2.0531	2.9020
42	2019	14	13.1143	77.3469	878.3337	3.2959	2.4500
50	2019	14	13.8857	74.2041	905.8663	3.2459	1.8551
43	2019	14	13.1143	77.3469	878.3337	3.2959	2.4500
85	2019	14	14.9571	74.2551	914.5031	2.0429	1.2378
25	2019	14	16.8571	80.7041	982.4796	2.3673	1.1908
69	2019	14	14.8143	76.6633	944.9408	2.7041	1.4286

57	2019	14	13.1714	79.3571	890.4827	3.2724	2.0296
9	2019	14	13.2000	78.3980	856.9918	2.5378	2.2643
72	2019	14	14.4857	76.3061	880.8714	2.9837	2.1867
26	2019	14	16.3143	74.8980	869.7898	4.7235	2.0806
7	2019	14	15.4429	73.7755	861.8439	4.8051	2.1286
83	2019	14	19.1714	68.8163	948.2602	3.3204	0.9510
76	2019	14	13.1857	79.8673	923.9061	2.9837	1.7735
36	2019	14	14.4429	76.9490	933.5051	3.0480	1.5827
81	2019	14	14.2143	80.0204	944.2286	2.8908	1.5010
15	2019	14	13.9429	76.6327	923.0582	3.0031	1.5388
32	2019	14	13.1143	77.3469	878.3337	3.2959	2.4500
73	2019	14	15.3714	73.1429	967.6898	3.1082	0.9429
71	2019	14	14.4429	76.9490	933.5051	3.0480	1.5827
41	2019	14	13.6143	70.5714	877.7000	2.6520	1.3939
10	2019	14	15.3143	79.1429	969.7194	2.8357	1.1765
23	2019	14	10.3429	69.4592	777.3622	5.9806	2.2786
27	2019	14	15.4429	73.7755	861.8439	4.8051	2.1286
60	2019	14	14.2143	80.0204	944.2286	2.8908	1.5010
53	2019	14	13.2000	78.3980	856.9918	2.5378	2.2643
66	2019	14	13.7714	86.6327	901.0276	2.2857	2.4031
59	2019	14	13.1714	79.3571	890.4827	3.2724	2.0296
61	2019	14	15.3714	73.1429	967.6898	3.1082	0.9429
84	2019	14	15.3714	73.1429	967.6898	3.1082	0.9429
38	2019	14	13.1714	79.3571	890.4827	3.2724	2.0296
87	2019	14	14.7714	72.8367	903.1337	3.1490	2.2398
34	2019	14	13.1714	79.3571	890.4827	3.2724	2.0296
29	2019	14	14.8143	76.6633	944.9408	2.7041	1.4286
5	2019	14	13.8429	75.8980	834.5112	4.9990	2.4327
8	2019	14	13.2000	78.3980	856.9918	2.5378	2.2643
12	2019	14	13.8429	75.8980	834.5112	4.9990	2.4327
13	2019	14	19.1714	68.8163	948.2602	3.3204	0.9510
18	2019	14	15.2571	78.3061	969.7214	2.5582	1.0327
33	2019	14	13.8857	74.2041	905.8663	3.2459	1.8551
56	2019	14	16.8571	80.7041	982.4796	2.3673	1.1908
77	2019	14	13.9429	76.6327	923.0582	3.0031	1.5388
54	2019	14	13.8429	75.8980	834.5112	4.9990	2.4327
21	2019	14	13.8857	74.2041	905.8663	3.2459	1.8551
68	2019	14	16.0429	76.5204	975.5449	2.5776	1.4541
74	2019	14	15.3714	73.1429	967.6898	3.1082	0.9429
88	2019	14	13.1143	77.3469	878.3337	3.2959	2.4500
16	2019	14	13.1857	79.8673	923.9061	2.9837	1.7735
30	2019	14	13.7714	86.6327	901.0276	2.2857	2.4031
6	2019	14	16.0429	76.5204	975.5449	2.5776	1.4541
49	2019	14	14.8143	76.6633	944.9408	2.7041	1.4286
22	2019	14	13.1143	77.3469	878.3337	3.2959	2.4500
45	2019	14	14.1714	62.5204	819.4327	6.3020	2.2592
58	2019	14	14.8143	76.6633	944.9408	2.7041	1.4286
37	2019	14	16.0429	76.5204	975.5449	2.5776	1.4541
17	2019	14	13.4286	83.9490	906.1827	2.0531	2.9020
55	2019	14	14.4857	76.3061	880.8714	2.9837	2.1867

46	2019	14	13.1857	79.8673	923.9061	2.9837	1.7735
86	2019	14	13.9143	74.8673	869.2194	2.4214	1.1878
2	2019	14	13.9143	74.8673	869.2194	2.4214	1.1878
4	2019	14	13.8857	74.2041	905.8663	3.2459	1.8551
47	2019	14	18.6857	71.2653	963.1888	2.6122	0.7398
82	2019	14	13.1143	77.3469	878.3337	3.2959	2.4500
19	2019	14	17.2143	76.8367	965.4980	2.0929	1.0214
20	2019	14	13.2000	78.3980	856.9918	2.5378	2.2643
80	2019	14	13.1143	77.3469	878.3337	3.2959	2.4500
3	2019	14	19.1714	68.8163	948.2602	3.3204	0.9510
52	2019	14	13.4286	83.9490	906.1827	2.0531	2.9020
70	2019	14	14.9571	74.2551	914.5031	2.0429	1.2378
64	2019	14	10.3429	69.4592	777.3622	5.9806	2.2786
48	2019	14	13.9429	76.6327	923.0582	3.0031	1.5388
65	2019	14	13.4286	83.9490	906.1827	2.0531	2.9020
44	2019	14	14.9571	74.2551	914.5031	2.0429	1.2378
75	2019	14	10.3429	69.4592	777.3622	5.9806	2.2786
40	2019	14	15.0571	82.5306	950.3776	3.3255	1.6643
11	2019	14	14.4857	76.3061	880.8714	2.9837	2.1867
35	2019	14	14.2143	80.0204	944.2286	2.8908	1.5010
78	2019	14	14.7714	72.8367	903.1337	3.1490	2.2398
28	2019	14	14.4429	76.9490	933.5051	3.0480	1.5827
39	2019	14	13.4286	83.9490	906.1827	2.0531	2.9020
24	2019	14	14.8143	76.6633	944.9408	2.7041	1.4286
63	2019	14	15.0571	82.5306	950.3776	3.3255	1.6643
62	2019	14	13.6143	70.5714	877.7000	2.6520	1.3939
1	2019	14	13.1143	77.3469	878.3337	3.2959	2.4500
31	2019	15	17.5143	69.2041	847.9633	4.5204	1.2459
79	2019	15	17.6286	73.4184	973.4071	3.9582	1.4745
51	2019	15	16.5571	78.9082	942.4459	3.0520	1.6847
14	2019	15	17.8857	85.3878	899.8255	4.3776	2.4122
67	2019	15	16.4857	83.5816	904.8959	3.0929	2.8480
42	2019	15	16.9143	76.7653	877.2000	4.3776	2.5592
50	2019	15	17.2714	72.1020	904.2173	4.1337	1.9510
43	2019	15	16.9143	76.7653	877.2000	4.3776	2.5592
85	2019	15	18.5143	71.2959	912.5847	3.9714	1.5153
25	2019	15	20.2429	79.5102	980.5765	3.5306	1.3296
69	2019	15	18.1571	75.5306	943.2224	3.1561	1.6296
57	2019	15	16.7000	76.9388	889.1459	4.1510	2.6286
9	2019	15	18.7000	74.2245	856.0357	5.4867	2.3276
72	2019	15	19.9000	74.0510	879.8265	4.7480	2.2122
26	2019	15	21.2143	72.9388	869.0571	5.8316	1.9327
7	2019	15	20.0286	71.8469	861.1133	5.8847	2.2837
83	2019	15	23.6857	71.5306	946.7480	4.7878	1.0255
76	2019	15	16.3000	79.8469	922.2398	3.3745	1.8949
36	2019	15	17.5143	77.3980	931.9429	3.3949	1.6745
81	2019	15	16.5571	78.9082	942.4459	3.0520	1.6847
15	2019	15	17.7571	73.5306	921.2061	4.1265	1.6602
32	2019	15	16.9143	76.7653	877.2000	4.3776	2.5592
73	2019	15	18.7286	72.4286	965.6031	3.1398	1.1051

71	2019	15	17.5143	77.3980	931.9429	3.3949	1.6745
41	2019	15	17.4143	69.2653	876.4041	4.2520	1.6439
10	2019	15	17.4000	77.7347	967.7327	3.4694	1.2092
23	2019	15	15.1286	65.4184	777.2337	6.0367	2.3388
27	2019	15	20.0286	71.8469	861.1133	5.8847	2.2837
60	2019	15	16.5571	78.9082	942.4459	3.0520	1.6847
53	2019	15	18.7000	74.2245	856.0357	5.4867	2.3276
66	2019	15	17.8857	85.3878	899.8255	4.3776	2.4122
59	2019	15	16.7000	76.9388	889.1459	4.1510	2.6286
61	2019	15	18.7286	72.4286	965.6031	3.1398	1.1051
84	2019	15	18.7286	72.4286	965.6031	3.1398	1.1051
38	2019	15	16.7000	76.9388	889.1459	4.1510	2.6286
87	2019	15	19.8429	72.5918	901.8255	5.1541	2.1041
34	2019	15	16.7000	76.9388	889.1459	4.1510	2.6286
29	2019	15	18.1571	75.5306	943.2224	3.1561	1.6296
5	2019	15	18.8000	70.8061	833.9571	5.9776	2.3602
8	2019	15	18.7000	74.2245	856.0357	5.4867	2.3276
12	2019	15	18.8000	70.8061	833.9571	5.9776	2.3602
13	2019	15	23.6857	71.5306	946.7480	4.7878	1.0255
18	2019	15	17.6571	77.0714	967.6092	3.0194	1.0867
33	2019	15	17.2714	72.1020	904.2173	4.1337	1.9510
56	2019	15	20.2429	79.5102	980.5765	3.5306	1.3296
77	2019	15	17.7571	73.5306	921.2061	4.1265	1.6602
54	2019	15	18.8000	70.8061	833.9571	5.9776	2.3602
21	2019	15	17.2714	72.1020	904.2173	4.1337	1.9510
68	2019	15	17.6286	73.4184	973.4071	3.9582	1.4745
74	2019	15	18.7286	72.4286	965.6031	3.1398	1.1051
88	2019	15	16.9143	76.7653	877.2000	4.3776	2.5592
16	2019	15	16.3000	79.8469	922.2398	3.3745	1.8949
30	2019	15	17.8857	85.3878	899.8255	4.3776	2.4122
6	2019	15	17.6286	73.4184	973.4071	3.9582	1.4745
49	2019	15	18.1571	75.5306	943.2224	3.1561	1.6296
22	2019	15	16.9143	76.7653	877.2000	4.3776	2.5592
45	2019	15	18.8714	63.5102	819.1908	6.6663	2.2173
58	2019	15	18.1571	75.5306	943.2224	3.1561	1.6296
37	2019	15	17.6286	73.4184	973.4071	3.9582	1.4745
17	2019	15	16.4857	83.5816	904.8959	3.0929	2.8480
55	2019	15	19.9000	74.0510	879.8265	4.7480	2.2122
46	2019	15	16.3000	79.8469	922.2398	3.3745	1.8949
86	2019	15	18.5000	71.7755	868.0173	3.6520	1.4673
2	2019	15	18.5000	71.7755	868.0173	3.6520	1.4673
4	2019	15	17.2714	72.1020	904.2173	4.1337	1.9510
47	2019	15	23.5857	72.2041	961.4031	4.7520	0.7571
82	2019	15	16.9143	76.7653	877.2000	4.3776	2.5592
19	2019	15	21.5000	75.8061	963.6816	3.4408	1.2439
20	2019	15	18.7000	74.2245	856.0357	5.4867	2.3276
80	2019	15	16.9143	76.7653	877.2000	4.3776	2.5592
3	2019	15	23.6857	71.5306	946.7480	4.7878	1.0255
52	2019	15	16.4857	83.5816	904.8959	3.0929	2.8480
70	2019	15	18.5143	71.2959	912.5847	3.9714	1.5153

64	2019	15	15.1286	65.4184	777.2337	6.0367	2.3388
48	2019	15	17.7571	73.5306	921.2061	4.1265	1.6602
65	2019	15	16.4857	83.5816	904.8959	3.0929	2.8480
44	2019	15	18.5143	71.2959	912.5847	3.9714	1.5153
75	2019	15	15.1286	65.4184	777.2337	6.0367	2.3388
40	2019	15	17.1429	81.0714	948.7173	4.2459	1.9143
11	2019	15	19.9000	74.0510	879.8265	4.7480	2.2122
35	2019	15	16.5571	78.9082	942.4459	3.0520	1.6847
78	2019	15	19.8429	72.5918	901.8255	5.1541	2.1041
28	2019	15	17.5143	77.3980	931.9429	3.3949	1.6745
39	2019	15	16.4857	83.5816	904.8959	3.0929	2.8480
24	2019	15	18.1571	75.5306	943.2224	3.1561	1.6296
63	2019	15	17.1429	81.0714	948.7173	4.2459	1.9143
62	2019	15	17.4143	69.2653	876.4041	4.2520	1.6439
1	2019	15	16.9143	76.7653	877.2000	4.3776	2.5592
31	2019	16	17.3429	70.1633	846.5245	5.3959	1.2857
79	2019	16	19.6286	73.0306	971.5245	3.6337	1.5265
51	2019	16	18.0571	81.1327	940.7541	2.8541	1.6520
14	2019	16	18.8429	86.4796	898.1776	4.7827	2.3684
67	2019	16	18.2143	85.7449	903.3276	3.3480	2.5500
42	2019	16	17.9571	78.3265	875.6898	4.9173	2.6133
50	2019	16	18.4857	72.9286	902.6133	4.1449	1.8694
43	2019	16	17.9571	78.3265	875.6898	4.9173	2.6133
85	2019	16	19.9167	72.5510	911.1296	4.0908	1.6133
25	2019	16	21.9286	80.0102	978.5816	3.6255	1.3245
69	2019	16	19.5714	76.2245	941.5061	3.0316	1.6969
57	2019	16	17.9000	78.4388	887.6031	4.5378	2.8602
9	2019	16	18.0571	73.3878	854.6327	6.9173	2.3398
72	2019	16	19.7571	73.8265	878.2867	5.3020	2.0714
26	2019	16	21.1143	71.3673	867.5684	6.5296	1.9755
7	2019	16	19.7429	71.7449	859.7102	6.4867	2.2765
83	2019	16	24.2714	72.2653	944.7551	5.2235	1.0429
76	2019	16	17.9000	81.2347	920.5898	3.0071	1.8704
36	2019	16	19.1429	79.5306	930.2745	3.3806	1.7500
81	2019	16	18.0571	81.1327	940.7541	2.8541	1.6520
15	2019	16	18.6714	72.9796	919.4765	3.8837	1.6408
32	2019	16	17.9571	78.3265	875.6898	4.9173	2.6133
73	2019	16	19.4857	74.7755	963.6684	2.8469	1.0714
71	2019	16	19.1429	79.5306	930.2745	3.3806	1.7500
41	2019	16	18.4714	70.3571	874.9286	5.3918	1.6449
10	2019	16	19.1714	80.5816	965.9551	3.1898	1.2051
23	2019	16	15.3143	64.2041	776.2551	7.2857	2.3816
27	2019	16	19.7429	71.7449	859.7102	6.4867	2.2765
60	2019	16	18.0571	81.1327	940.7541	2.8541	1.6520
53	2019	16	18.0571	73.3878	854.6327	6.9173	2.3398
66	2019	16	18.8429	86.4796	898.1776	4.7827	2.3684
59	2019	16	17.9000	78.4388	887.6031	4.5378	2.8602
61	2019	16	19.4857	74.7755	963.6684	2.8469	1.0714
84	2019	16	19.4857	74.7755	963.6684	2.8469	1.0714
38	2019	16	17.9000	78.4388	887.6031	4.5378	2.8602

87	2019	16	20.4000	73.0408	900.1143	5.4694	1.9510
34	2019	16	17.9000	78.4388	887.6031	4.5378	2.8602
29	2019	16	19.5714	76.2245	941.5061	3.0316	1.6969
5	2019	16	17.9000	71.1939	832.7214	6.6796	2.2633
8	2019	16	18.0571	73.3878	854.6327	6.9173	2.3398
12	2019	16	17.9000	71.1939	832.7214	6.6796	2.2633
13	2019	16	24.2714	72.2653	944.7551	5.2235	1.0429
18	2019	16	19.5000	76.5204	965.6541	3.3327	1.1286
33	2019	16	18.4857	72.9286	902.6133	4.1449	1.8694
56	2019	16	21.9286	80.0102	978.5816	3.6255	1.3245
77	2019	16	18.6714	72.9796	919.4765	3.8837	1.6408
54	2019	16	17.9000	71.1939	832.7214	6.6796	2.2633
21	2019	16	18.4857	72.9286	902.6133	4.1449	1.8694
68	2019	16	19.6286	73.0306	971.5245	3.6337	1.5265
74	2019	16	19.4857	74.7755	963.6684	2.8469	1.0714
88	2019	16	17.9571	78.3265	875.6898	4.9173	2.6133
16	2019	16	17.9000	81.2347	920.5898	3.0071	1.8704
30	2019	16	18.8429	86.4796	898.1776	4.7827	2.3684
6	2019	16	19.6286	73.0306	971.5245	3.6337	1.5265
49	2019	16	19.5714	76.2245	941.5061	3.0316	1.6969
22	2019	16	17.9571	78.3265	875.6898	4.9173	2.6133
45	2019	16	19.3286	62.5102	817.9980	7.2092	2.1878
58	2019	16	19.5714	76.2245	941.5061	3.0316	1.6969
37	2019	16	19.6286	73.0306	971.5245	3.6337	1.5265
17	2019	16	18.2143	85.7449	903.3276	3.3480	2.5500
55	2019	16	19.7571	73.8265	878.2867	5.3020	2.0714
46	2019	16	17.9000	81.2347	920.5898	3.0071	1.8704
86	2019	16	18.6571	71.0816	866.4959	4.7255	1.4949
2	2019	16	18.6571	71.0816	866.4959	4.7255	1.4949
4	2019	16	18.4857	72.9286	902.6133	4.1449	1.8694
47	2019	16	23.8143	72.4286	959.2878	5.6184	0.7724
82	2019	16	17.9571	78.3265	875.6898	4.9173	2.6133
19	2019	16	22.7857	75.0102	961.6429	4.2480	1.3510
20	2019	16	18.0571	73.3878	854.6327	6.9173	2.3398
80	2019	16	17.9571	78.3265	875.6898	4.9173	2.6133
3	2019	16	24.2714	72.2653	944.7551	5.2235	1.0429
52	2019	16	18.2143	85.7449	903.3276	3.3480	2.5500
70	2019	16	19.9167	72.5510	911.1296	4.0908	1.6133
64	2019	16	15.3143	64.2041	776.2551	7.2857	2.3816
48	2019	16	18.6714	72.9796	919.4765	3.8837	1.6408
65	2019	16	18.2143	85.7449	903.3276	3.3480	2.5500
44	2019	16	19.9167	72.5510	911.1296	4.0908	1.6133
75	2019	16	15.3143	64.2041	776.2551	7.2857	2.3816
40	2019	16	18.9000	83.4286	947.0082	3.6878	1.9061
11	2019	16	19.7571	73.8265	878.2867	5.3020	2.0714
35	2019	16	18.0571	81.1327	940.7541	2.8541	1.6520
78	2019	16	20.4000	73.0408	900.1143	5.4694	1.9510
28	2019	16	19.1429	79.5306	930.2745	3.3806	1.7500
39	2019	16	18.2143	85.7449	903.3276	3.3480	2.5500
24	2019	16	19.5714	76.2245	941.5061	3.0316	1.6969

63	2019	16	18.9000	83.4286	947.0082	3.6878	1.9061
62	2019	16	18.4714	70.3571	874.9286	5.3918	1.6449
1	2019	16	17.9571	78.3265	875.6898	4.9173	2.6133
31	2019	17	20.8571	73.7347	845.5112	5.1653	1.2153
79	2019	17	20.4143	80.1633	969.7796	1.9337	1.5878
51	2019	17	19.6000	87.4694	939.1092	2.1184	1.4449
14	2019	17	21.7143	90.6939	896.5071	3.9959	2.6041
67	2019	17	20.6714	90.9184	901.7071	2.4765	2.2316
42	2019	17	21.6714	80.2755	874.2551	4.9531	2.5653
50	2019	17	21.6429	77.1531	901.2827	3.1786	1.7633
43	2019	17	21.6714	80.2755	874.2551	4.9531	2.5653
85	2019	17	23.6286	74.6837	909.8051	3.3367	1.3612
25	2019	17	24.1429	83.2143	976.1684	2.8969	1.2031
69	2019	17	21.7429	80.4796	939.6622	2.0531	1.4969
57	2019	17	20.6857	83.5408	886.2347	3.6622	2.3163
9	2019	17	21.2286	75.8367	853.3704	6.3704	2.4000
72	2019	17	23.0000	74.2857	876.6816	4.8520	1.9816
26	2019	17	24.5143	66.0612	865.8276	7.4296	2.0031
7	2019	17	23.1714	69.1633	858.1327	7.1255	2.0714
83	2019	17	27.4286	68.7551	942.3571	6.1663	1.0755
76	2019	17	19.6286	86.6939	919.0704	1.7112	1.7459
36	2019	17	21.0286	84.0510	928.4439	2.7867	1.4204
81	2019	17	19.6000	87.4694	939.1092	2.1184	1.4449
15	2019	17	21.4143	78.1735	918.0429	2.4347	1.4571
32	2019	17	21.6714	80.2755	874.2551	4.9531	2.5653
73	2019	17	21.4000	82.2755	961.8990	1.6837	0.7439
71	2019	17	21.0286	84.0510	928.4439	2.7867	1.4204
41	2019	17	21.3714	74.2041	873.6867	5.4163	1.4051
10	2019	17	20.2000	86.0000	964.1347	1.9918	1.1602
23	2019	17	18.5571	66.1224	775.4551	8.0388	2.1908
27	2019	17	23.1714	69.1633	858.1327	7.1255	2.0714
60	2019	17	19.6000	87.4694	939.1092	2.1184	1.4449
53	2019	17	21.2286	75.8367	853.3704	6.3704	2.4000
66	2019	17	21.7143	90.6939	896.5071	3.9959	2.6041
59	2019	17	20.6857	83.5408	886.2347	3.6622	2.3163
61	2019	17	21.4000	82.2755	961.8990	1.6837	0.7439
84	2019	17	21.4000	82.2755	961.8990	1.6837	0.7439
38	2019	17	20.6857	83.5408	886.2347	3.6622	2.3163
87	2019	17	23.8286	73.8469	898.4173	5.5010	1.7663
34	2019	17	20.6857	83.5408	886.2347	3.6622	2.3163
29	2019	17	21.7429	80.4796	939.6622	2.0531	1.4969
5	2019	17	21.5571	70.3469	831.4500	7.4255	1.9704
8	2019	17	21.2286	75.8367	853.3704	6.3704	2.4000
12	2019	17	21.5571	70.3469	831.4500	7.4255	1.9704
13	2019	17	27.4286	68.7551	942.3571	6.1663	1.0755
18	2019	17	20.6714	81.2755	963.9500	2.1398	1.0786
33	2019	17	21.6429	77.1531	901.2827	3.1786	1.7633
56	2019	17	24.1429	83.2143	976.1684	2.8969	1.2031
77	2019	17	21.4143	78.1735	918.0429	2.4347	1.4571
54	2019	17	21.5571	70.3469	831.4500	7.4255	1.9704

21	2019	17	21.6429	77.1531	901.2827	3.1786	1.7633
68	2019	17	20.4143	80.1633	969.7796	1.9337	1.5878
74	2019	17	21.4000	82.2755	961.8990	1.6837	0.7439
88	2019	17	21.6714	80.2755	874.2551	4.9531	2.5653
16	2019	17	19.6286	86.6939	919.0704	1.7112	1.7459
30	2019	17	21.7143	90.6939	896.5071	3.9959	2.6041
6	2019	17	20.4143	80.1633	969.7796	1.9337	1.5878
49	2019	17	21.7429	80.4796	939.6622	2.0531	1.4969
22	2019	17	21.6714	80.2755	874.2551	4.9531	2.5653
45	2019	17	21.4143	57.4592	816.7796	8.0888	1.8929
58	2019	17	21.7429	80.4796	939.6622	2.0531	1.4969
37	2019	17	20.4143	80.1633	969.7796	1.9337	1.5878
17	2019	17	20.6714	90.9184	901.7071	2.4765	2.2316
55	2019	17	23.0000	74.2857	876.6816	4.8520	1.9816
46	2019	17	19.6286	86.6939	919.0704	1.7112	1.7459
86	2019	17	21.7429	74.2857	865.2449	5.4122	1.2776
2	2019	17	21.7429	74.2857	865.2449	5.4122	1.2776
4	2019	17	21.6429	77.1531	901.2827	3.1786	1.7633
47	2019	17	27.2429	73.5306	956.8000	5.6541	0.6765
82	2019	17	21.6714	80.2755	874.2551	4.9531	2.5653
19	2019	17	25.7429	75.7449	959.2541	3.8939	1.2122
20	2019	17	21.2286	75.8367	853.3704	6.3704	2.4000
80	2019	17	21.6714	80.2755	874.2551	4.9531	2.5653
3	2019	17	27.4286	68.7551	942.3571	6.1663	1.0755
52	2019	17	20.6714	90.9184	901.7071	2.4765	2.2316
70	2019	17	23.6286	74.6837	909.8051	3.3367	1.3612
64	2019	17	18.5571	66.1224	775.4551	8.0388	2.1908
48	2019	17	21.4143	78.1735	918.0429	2.4347	1.4571
65	2019	17	20.6714	90.9184	901.7071	2.4765	2.2316
44	2019	17	23.6286	74.6837	909.8051	3.3367	1.3612
75	2019	17	18.5571	66.1224	775.4551	8.0388	2.1908
40	2019	17	21.1714	88.1735	945.1429	2.1949	1.5235
11	2019	17	23.0000	74.2857	876.6816	4.8520	1.9816
35	2019	17	19.6000	87.4694	939.1092	2.1184	1.4449
78	2019	17	23.8286	73.8469	898.4173	5.5010	1.7663
28	2019	17	21.0286	84.0510	928.4439	2.7867	1.4204
39	2019	17	20.6714	90.9184	901.7071	2.4765	2.2316
24	2019	17	21.7429	80.4796	939.6622	2.0531	1.4969
63	2019	17	21.1714	88.1735	945.1429	2.1949	1.5235
62	2019	17	21.3714	74.2041	873.6867	5.4163	1.4051
1	2019	17	21.6714	80.2755	874.2551	4.9531	2.5653
31	2019	18	14.9714	72.9694	845.3061	4.3347	1.2520
79	2019	18	17.4857	82.8878	969.3296	2.0306	1.5745
51	2019	18	15.4286	88.4898	938.6551	2.1316	1.4500
14	2019	18	16.1143	89.4184	896.2398	3.5643	2.5969
67	2019	18	15.4857	89.7449	901.2827	2.3959	2.1939
42	2019	18	15.6143	77.6531	873.9704	4.6592	2.4929
50	2019	18	16.2429	77.8265	900.7245	3.4541	1.9133
43	2019	18	15.6143	77.6531	873.9704	4.6592	2.4929
85	2019	18	17.2143	73.1939	908.9255	3.3459	1.4398

25	2019	18	19.1429	82.8469	975.4837	2.7408	1.2969
69	2019	18	16.9000	81.2551	939.0622	1.9592	1.3888
57	2019	18	15.2571	83.1224	885.8367	3.9847	2.2224
9	2019	18	15.2571	74.5000	853.2694	5.4908	2.5122
72	2019	18	17.0143	72.3980	876.5235	4.0082	2.0122
26	2019	18	18.1429	62.6531	865.6939	6.8959	2.1816
7	2019	18	17.1000	65.4388	858.0224	6.2551	2.1367
83	2019	18	21.7429	65.5918	942.0245	5.7816	1.1980
76	2019	18	15.1714	88.3061	918.5367	1.5684	1.8388
36	2019	18	16.2857	83.0918	927.9296	2.5939	1.2949
81	2019	18	15.4286	88.4898	938.6551	2.1316	1.4500
15	2019	18	15.8286	80.8265	917.4949	2.2296	1.5173
32	2019	18	15.6143	77.6531	873.9704	4.6592	2.4929
73	2019	18	17.4286	84.3469	961.3673	1.6347	0.6449
71	2019	18	16.2857	83.0918	927.9296	2.5939	1.2949
41	2019	18	16.1857	73.3265	873.3806	4.8929	1.5102
10	2019	18	16.3857	86.4796	963.6398	2.0357	1.1694
23	2019	18	12.6000	65.1429	775.5071	6.4551	2.2082
27	2019	18	17.1000	65.4388	858.0224	6.2551	2.1367
60	2019	18	15.4286	88.4898	938.6551	2.1316	1.4500
53	2019	18	15.2571	74.5000	853.2694	5.4908	2.5122
66	2019	18	16.1143	89.4184	896.2398	3.5643	2.5969
59	2019	18	15.2571	83.1224	885.8367	3.9847	2.2224
61	2019	18	17.4286	84.3469	961.3673	1.6347	0.6449
84	2019	18	17.4286	84.3469	961.3673	1.6347	0.6449
38	2019	18	15.2571	83.1224	885.8367	3.9847	2.2224
87	2019	18	17.7857	72.3571	898.1102	5.1020	1.7061
34	2019	18	15.2571	83.1224	885.8367	3.9847	2.2224
29	2019	18	16.9000	81.2551	939.0622	1.9592	1.3888
5	2019	18	15.2429	68.1122	831.4153	6.2765	1.7133
8	2019	18	15.2571	74.5000	853.2694	5.4908	2.5122
12	2019	18	15.2429	68.1122	831.4153	6.2765	1.7133
13	2019	18	21.7429	65.5918	942.0245	5.7816	1.1980
18	2019	18	17.3429	84.4388	963.5439	1.5418	1.0061
33	2019	18	16.2429	77.8265	900.7245	3.4541	1.9133
56	2019	18	19.1429	82.8469	975.4837	2.7408	1.2969
77	2019	18	15.8286	80.8265	917.4949	2.2296	1.5173
54	2019	18	15.2429	68.1122	831.4153	6.2765	1.7133
21	2019	18	16.2429	77.8265	900.7245	3.4541	1.9133
68	2019	18	17.4857	82.8878	969.3296	2.0306	1.5745
74	2019	18	17.4286	84.3469	961.3673	1.6347	0.6449
88	2019	18	15.6143	77.6531	873.9704	4.6592	2.4929
16	2019	18	15.1714	88.3061	918.5367	1.5684	1.8388
30	2019	18	16.1143	89.4184	896.2398	3.5643	2.5969
6	2019	18	17.4857	82.8878	969.3296	2.0306	1.5745
49	2019	18	16.9000	81.2551	939.0622	1.9592	1.3888
22	2019	18	15.6143	77.6531	873.9704	4.6592	2.4929
45	2019	18	16.2000	56.6633	816.7898	6.9133	1.8051
58	2019	18	16.9000	81.2551	939.0622	1.9592	1.3888
37	2019	18	17.4857	82.8878	969.3296	2.0306	1.5745

17	2019	18	15.4857	89.7449	901.2827	2.3959	2.1939
55	2019	18	17.0143	72.3980	876.5235	4.0082	2.0122
46	2019	18	15.1714	88.3061	918.5367	1.5684	1.8388
86	2019	18	16.2429	72.9796	865.0408	4.7806	1.2745
2	2019	18	16.2429	72.9796	865.0408	4.7806	1.2745
4	2019	18	16.2429	77.8265	900.7245	3.4541	1.9133
47	2019	18	21.1571	72.8776	956.3541	4.7796	0.5929
82	2019	18	15.6143	77.6531	873.9704	4.6592	2.4929
19	2019	18	20.0000	74.3469	958.7276	3.2306	1.1122
20	2019	18	15.2571	74.5000	853.2694	5.4908	2.5122
80	2019	18	15.6143	77.6531	873.9704	4.6592	2.4929
3	2019	18	21.7429	65.5918	942.0245	5.7816	1.1980
52	2019	18	15.4857	89.7449	901.2827	2.3959	2.1939
70	2019	18	17.2143	73.1939	908.9255	3.3459	1.4398
64	2019	18	12.6000	65.1429	775.5071	6.4551	2.2082
48	2019	18	15.8286	80.8265	917.4949	2.2296	1.5173
65	2019	18	15.4857	89.7449	901.2827	2.3959	2.1939
44	2019	18	17.2143	73.1939	908.9255	3.3459	1.4398
75	2019	18	12.6000	65.1429	775.5071	6.4551	2.2082
40	2019	18	15.9286	87.3163	944.6653	2.1469	1.4561
11	2019	18	17.0143	72.3980	876.5235	4.0082	2.0122
35	2019	18	15.4286	88.4898	938.6551	2.1316	1.4500
78	2019	18	17.7857	72.3571	898.1102	5.1020	1.7061
28	2019	18	16.2857	83.0918	927.9296	2.5939	1.2949
39	2019	18	15.4857	89.7449	901.2827	2.3959	2.1939
24	2019	18	16.9000	81.2551	939.0622	1.9592	1.3888
63	2019	18	15.9286	87.3163	944.6653	2.1469	1.4561
62	2019	18	16.1857	73.3265	873.3806	4.8929	1.5102
1	2019	18	15.6143	77.6531	873.9704	4.6592	2.4929
31	2019	19	14.6429	79.8061	846.7776	1.7041	1.1806
79	2019	19	17.9000	84.5306	971.7673	1.7490	1.3714
51	2019	19	16.7286	89.5510	940.8459	1.4724	1.3439
14	2019	19	17.0833	90.7653	898.0816	1.5449	1.8714
67	2019	19	16.1429	91.1327	903.1510	1.5888	2.1020
42	2019	19	15.2143	83.0714	875.7316	2.0173	2.3143
50	2019	19	16.0286	80.4388	902.8265	1.9061	1.8245
43	2019	19	15.2143	83.0714	875.7316	2.0173	2.3143
85	2019	19	16.8143	79.1224	911.0959	1.4827	1.4520
25	2019	19	19.7714	84.6939	978.1082	1.6347	1.0704
69	2019	19	18.1429	82.3163	941.4408	1.1367	1.3173
57	2019	19	14.9286	87.0918	887.7337	2.2776	2.0235
9	2019	19	15.1286	81.5000	854.7439	2.2735	2.1235
72	2019	19	16.7286	79.2347	878.2429	1.4378	1.9673
26	2019	19	17.4000	78.7347	867.2255	2.8439	1.8020
7	2019	19	16.5429	78.4694	859.5378	2.0561	1.8857
83	2019	19	21.2143	72.5306	944.5643	2.1010	0.9367
76	2019	19	16.2000	90.3061	920.7776	0.7286	1.8092
36	2019	19	17.5286	84.0000	930.0959	1.4418	1.3000
81	2019	19	16.7286	89.5510	940.8459	1.4724	1.3439
15	2019	19	15.7571	85.8673	919.9694	1.3490	1.4102

32	2019	19	15.2143	83.0714	875.7316	2.0173	2.3143
73	2019	19	18.3857	84.0510	963.9796	1.2714	0.6643
71	2019	19	17.5286	84.0000	930.0959	1.4418	1.3000
41	2019	19	15.3000	77.9490	875.3163	2.1204	1.4643
10	2019	19	17.8429	87.1633	965.9714	1.6449	1.0082
23	2019	19	12.6000	79.1735	776.1418	2.7745	2.1214
27	2019	19	16.5429	78.4694	859.5378	2.0561	1.8857
60	2019	19	16.7286	89.5510	940.8459	1.4724	1.3439
53	2019	19	15.1286	81.5000	854.7439	2.2735	2.1235
66	2019	19	17.0833	90.7653	898.0816	1.5449	1.8714
59	2019	19	14.9286	87.0918	887.7337	2.2776	2.0235
61	2019	19	18.3857	84.0510	963.9796	1.2714	0.6643
84	2019	19	18.3857	84.0510	963.9796	1.2714	0.6643
38	2019	19	14.9286	87.0918	887.7337	2.2776	2.0235
87	2019	19	17.8429	77.2143	900.0816	1.9969	1.6388
34	2019	19	14.9286	87.0918	887.7337	2.2776	2.0235
29	2019	19	18.1429	82.3163	941.4408	1.1367	1.3173
5	2019	19	14.6714	83.6531	832.6633	1.9878	1.5939
8	2019	19	15.1286	81.5000	854.7439	2.2735	2.1235
12	2019	19	14.6714	83.6531	832.6633	1.9878	1.5939
13	2019	19	21.2143	72.5306	944.5643	2.1010	0.9367
18	2019	19	17.8429	86.0000	966.0235	1.8245	0.8653
33	2019	19	16.0286	80.4388	902.8265	1.9061	1.8245
56	2019	19	19.7714	84.6939	978.1082	1.6347	1.0704
77	2019	19	15.7571	85.8673	919.9694	1.3490	1.4102
54	2019	19	14.6714	83.6531	832.6633	1.9878	1.5939
21	2019	19	16.0286	80.4388	902.8265	1.9061	1.8245
68	2019	19	17.9000	84.5306	971.7673	1.7490	1.3714
74	2019	19	18.3857	84.0510	963.9796	1.2714	0.6643
88	2019	19	15.2143	83.0714	875.7316	2.0173	2.3143
16	2019	19	16.2000	90.3061	920.7776	0.7286	1.8092
30	2019	19	17.0833	90.7653	898.0816	1.5449	1.8714
6	2019	19	17.9000	84.5306	971.7673	1.7490	1.3714
49	2019	19	18.1429	82.3163	941.4408	1.1367	1.3173
22	2019	19	15.2143	83.0714	875.7316	2.0173	2.3143
45	2019	19	16.1714	74.2449	817.7173	3.2857	1.5888
58	2019	19	18.1429	82.3163	941.4408	1.1367	1.3173
[truncated: 201,957 more chars]
