# Supplementary material for: Spatial heterogeneity in the temperature–hand, foot, and mouth disease association among children: A multicounty time-series study in western China
Source: PLoS Negl Trop Dis. 2026 Jan 2;20(1):e0013801. doi: 10.1371/journal.pntd.0013801 (PMC12758769; doi:10.1371/journal.pntd.0013801)
Supplement: S2 Data — (PDF) [file pntd.0013801.s002.pdf]

seq	Population.density	GDP.per.capita	heatwave_max_2	colaspell_min_2
1	685.8202	76400.7601	65	80
2	158.7005	18726.3950	79	74
3	223.3507	23274.5503	80	79
4	279.3286	44530.8405	60	93
5	148.0287	33670.2303	69	79
6	314.9127	35638.6692	55	80
7	1816.5944	49007.9580	65	76
8	72.6108	24024.9692	50	80
9	110.8939	28289.6646	69	80
10	185.1399	52878.8723	69	80
11	149.2266	24438.6507	76	75
12	4285.3635	73313.2821	65	80
13	164.3032	25517.1242	76	75
14	358.2194	66531.7147	65	80
15	206.1728	22994.2598	65	76
16	115.9397	18297.9274	73	67
17	108.9025	25492.5746	71	72
18	85.7070	23846.1882	69	80
19	119.2944	26757.8544	74	74
20	235.7663	31018.1099	58	86
21	320.7196	45429.3806	81	71
22	83.6534	22173.4507	71	72
23	585.1577	63454.2679	56	79
24	165.8585	20806.7835	65	76
25	162.1252	28173.1522	58	86
26	278.7313	29621.8713	55	80
27	224.1735	65418.2809	69	80
28	128.2590	23965.8834	69	79
29	166.4110	29844.7859	55	83
30	225.0693	24315.7391	66	76
31	105.9239	58308.4506	65	80
32	119.2766	20886.5986	55	83
33	188.1461	25635.9034	50	89
34	151.1669	24848.5629	71	72
35	131.2780	22678.1917	69	79
36	139.7101	23309.5028	79	74
37	295.6372	26671.3464	66	76
38	97.7997	23987.3887	72	74
39	244.9070	30822.4043	50	89
40	203.5131	16251.2152	56	79

41	96.9937	25362.0233	77	74
42	86.9265	19489.4675	66	74
43	108.6209	29881.5753	71	72
44	180.7226	20521.0818	80	79
45	101.0814	26825.9175	72	74
46	344.8732	40744.3355	72	74
47	144.0876	24690.9686	53	73
48	177.3867	27137.6103	50	89
49	345.4904	53715.0282	76	75
50	93.4737	27323.6231	81	71
51	917.0745	77146.5615	65	80
52	131.4766	37497.8460	74	81
53	143.5083	23427.0283	85	69
54	196.1488	26918.1320	55	83
55	149.0711	21457.4707	73	67
56	114.3165	22320.0266	73	67
57	311.8967	116522.8034	74	81
58	319.3075	38589.6310	69	80
59	277.2289	21161.9326	56	78
60	174.5636	47709.6011	69	79
61	277.3412	25776.5938	56	78
62	80.6400	23279.7966	50	80
63	10574.9706	69899.1089	65	80
64	90.1579	18399.0515	66	74
65	198.3675	28845.2651	79	74
66	349.0712	54428.9049	65	80
67	252.4922	56297.9337	69	80
68	143.6471	29386.5237	80	79
69	337.6452	28813.5053	63	86
70	146.9143	29006.6615	79	74
71	259.9465	47408.8149	60	90
72	147.6090	35543.5638	81	71
73	53.2014	39324.1411	68	69
74	245.9083	54858.4856	81	71
75	205.3796	33890.5429	50	80
76	356.3245	25189.5931	55	80
77	121.5355	28909.3561	53	73
78	85.9849	24550.4575	65	74
79	531.8715	34924.4459	55	80
80	85.9740	24710.5025	79	74
81	88.2154	18992.5087	77	74

82	112.2215	21792.5962	69	80
83	202.8735	18003.1817	56	79
84	202.1520	40475.3641	69	79
85	355.7647	62309.1511	65	80
86	166.7082	28032.3691	76	75
87	177.8449	26247.3179	80	79
88	224.2517	38219.6395	74	81

seq	heatwave_max_3	colaspell_min_3	heatwave_tmean_2	colaspell_tmean_2
1	42	49	70	82
2	49	44	72	86
3	50	49	81	89
4	27	65	62	87
5	37	47	71	87
6	27	54	67	89
7	35	47	80	91
8	13	53	65	91
9	34	52	66	88
10	39	51	71	87
11	46	48	82	88
12	42	49	70	82
13	46	48	82	88
14	42	49	70	82
15	35	47	80	91
16	47	37	82	80
17	41	44	74	86
18	34	52	66	88
19	40	43	79	88
20	20	60	61	91
21	53	41	85	91
22	41	44	74	86
23	26	49	63	85
24	35	47	80	91
25	20	60	61	91
26	27	54	67	89
27	39	51	71	87
28	37	47	71	87
29	22	58	61	90
30	33	53	78	81
31	42	49	70	82
32	22	58	61	90

33	21	61	60	87
34	41	44	74	86
35	37	47	71	87
36	49	44	72	86
37	33	53	78	81
38	43	43	70	93
39	21	61	60	87
40	26	49	63	85
41	51	44	75	87
42	34	44	68	83
43	41	44	74	86
44	50	49	81	89
45	43	43	70	93
46	43	43	70	93
47	25	49	70	83
48	21	61	60	87
49	46	48	82	88
50	53	41	85	91
51	42	49	70	82
52	44	55	78	90
53	57	38	83	90
54	22	58	61	90
55	47	37	82	80
56	47	37	82	80
57	44	55	78	90
58	39	51	71	87
59	27	51	69	84
60	37	47	71	87
61	27	51	69	84
62	13	53	65	91
63	42	49	70	82
64	34	44	68	83
65	49	44	72	86
66	42	49	70	82
67	39	51	71	87
68	50	49	81	89
69	27	57	71	87
70	49	44	72	86
71	29	60	67	88
72	53	41	85	91
73	35	39	62	80

74	53	41	85	91
75	13	53	65	91
76	27	54	67	89
77	25	49	70	83
78	31	48	62	90
79	27	54	67	89
80	49	44	72	86
81	51	44	75	87
82	34	52	66	88
83	26	49	63	85
84	37	47	71	87
85	42	49	70	82
86	46	48	82	88
87	50	49	81	89
88	44	55	78	90

seq	heatwave_tmean_3	colaspell_tmean_3	$PM_{2.5}$	PM_{10}
1	38	52	32.5490	51.9658
2	40	56	34.0813	53.1188
3	50	61	38.7524	61.6802
4	29	59	29.6333	49.9891
5	36	58	30.7730	47.3517
6	33	61	31.6700	49.8760
7	47	63	38.5923	61.8772
8	31	61	33.2331	53.2754
9	36	59	31.0115	48.2937
10	39	61	35.0815	54.9689
11	57	63	36.9938	57.6786
12	38	52	36.3974	59.7096
13	57	63	38.7761	60.1214
14	38	52	34.1492	54.2860
15	47	63	37.3057	58.8867
16	55	54	37.8578	59.1963
17	43	59	35.6690	56.4792
18	36	59	31.2588	48.9861
19	44	62	36.4529	56.7472
20	26	62	31.4731	51.0852
21	56	65	37.3875	61.9763
22	43	59	32.8111	50.7334
23	32	55	34.4717	55.4061
24	47	63	37.3707	58.9192

25	26	62	31.0305	51.0121
26	33	61	33.9837	53.8904
27	39	61	36.6283	57.6045
28	36	58	31.4484	48.5460
29	29	59	33.8040	54.1723
30	47	56	34.8981	54.1852
31	38	52	31.2256	48.1099
32	29	59	32.3384	51.4876
33	26	59	33.7140	54.7312
34	43	59	34.6666	54.1925
35	36	58	32.1543	49.6746
36	40	56	36.3551	57.3137
37	47	56	34.8638	54.8594
38	36	65	31.6487	48.9267
39	26	59	33.4107	52.7929
40	32	55	29.1167	48.7292
41	43	57	34.3082	52.9619
42	38	55	32.8883	50.9334
43	43	59	34.8172	54.6687
44	50	61	39.1217	61.6004
45	36	65	32.3936	50.0463
46	36	65	33.3751	51.5855
47	36	53	30.9257	48.2837
48	26	59	31.6059	50.9665
49	57	63	38.3869	60.4167
50	56	65	35.6592	56.7196
51	38	52	33.1261	53.1687
52	53	67	44.6755	68.7934
53	55	65	37.4810	59.5674
54	29	59	34.9359	56.6742
55	55	54	37.9913	59.2339
56	55	54	38.1440	59.2714
57	53	67	41.1290	64.9055
58	39	61	38.5790	61.6491
59	37	55	32.8834	51.0252
60	36	58	32.4894	50.3204
61	37	55	33.9045	52.7160
62	31	61	33.9764	54.2852
63	38	52	36.7527	59.1664
64	38	55	33.6663	51.9040
65	40	56	35.1154	54.9925

66	38	52	33.1020	52.2453
67	39	61	34.2111	53.5014
68	50	61	36.5229	57.5405
69	40	57	35.2661	54.8545
70	40	56	36.1118	56.7527
71	34	61	29.9266	48.9342
72	56	65	37.3068	60.9215
73	25	51	31.7679	49.0782
74	56	65	37.3957	60.1213
75	31	61	34.0005	54.9818
76	33	61	32.2752	50.8558
77	36	53	30.9354	48.6183
78	34	61	34.1653	54.1686
79	33	61	31.3860	49.1046
80	40	56	34.6176	54.1545
81	43	57	33.5454	51.5752
82	36	59	32.2719	49.9142
83	32	55	31.4955	50.2884
84	36	58	30.6506	47.6860
85	38	52	33.3457	51.9049
86	57	63	41.0611	63.7121
87	50	61	37.9825	60.0917
88	53	67	37.9418	59.8691

seq	O3	Nighttime.light	NDVI	NO3
1	78.6657	53.7097	0.4912	6.3083
2	72.2601	53.0391	0.5219	6.3731
3	73.9212	61.5932	0.5166	7.5111
4	76.0606	54.1914	0.5682	4.5162
5	75.5018	49.2786	0.5461	5.2687
6	79.0640	61.4970	0.4971	6.9639
7	74.4483	50.0273	0.4845	8.5416
8	77.1861	48.8898	0.6122	4.2267
9	72.9940	64.9809	0.5383	4.2943
10	76.2326	47.4942	0.5332	7.4818
11	74.3517	58.8034	0.5207	8.7794
12	73.7548	61.9386	0.4152	7.3132
13	74.7583	55.7541	0.5572	8.9283
14	76.5297	52.5720	0.4246	8.9582
15	74.2460	42.3695	0.5163	7.3304
16	74.1699	58.3680	0.5297	7.4076

17	74.2046	47.0192	0.5636	7.3329
18	75.9794	63.2901	0.5578	4.4227
19	75.7064	53.0072	0.5953	6.6780
20	76.5553	59.7170	0.5460	5.6498
21	76.2482	50.8265	0.5607	8.2516
22	72.0923	44.4017	0.5910	5.6381
23	74.3231	50.7082	0.5054	6.0805
24	73.2139	51.5984	0.5198	7.4358
25	77.0847	53.5156	0.5586	4.7136
26	79.2595	44.2650	0.5374	6.0369
27	77.7652	61.7888	0.5133	8.6970
28	72.1862	46.4639	0.5328	5.3590
29	79.8979	50.0138	0.5566	5.8408
30	77.7928	48.7796	0.5540	7.8091
31	78.4635	47.7049	0.5424	6.1216
32	78.9651	55.1090	0.5741	4.9829
33	78.0648	56.8252	0.5371	6.0250
34	72.8721	58.5713	0.5727	6.4835
35	72.4509	46.8069	0.5476	5.4703
36	73.1152	56.8975	0.5448	6.8375
37	79.0292	56.8042	0.5196	8.2620
38	72.6768	49.6348	0.5555	5.3651
39	74.8004	56.8733	0.5512	6.1684
40	83.4315	60.0877	0.5150	5.2949
41	74.2667	47.0089	0.5863	5.8617
42	73.5437	61.8084	0.5798	5.0256
43	73.5180	57.6587	0.5515	6.5668
44	73.8167	52.0446	0.5280	7.9762
45	71.1084	55.3718	0.5799	6.1812
46	71.0134	41.9579	0.5089	6.0084
47	78.1188	52.2103	0.5478	5.2361
48	76.1625	58.9570	0.5676	5.7466
49	75.6075	52.3989	0.5149	10.1797
50	74.0755	44.6523	0.5753	7.7266
51	77.3971	47.5796	0.4483	7.5558
52	75.7419	50.8061	0.5994	10.0295
53	74.6725	61.0021	0.5584	8.3664
54	79.9580	49.0815	0.5275	5.8440
55	73.7271	48.3353	0.5166	7.3793
56	75.0361	51.0395	0.5336	7.4022
57	77.2959	56.8612	0.5137	9.8035

58	76.0999	49.7973	0.5009	8.5150
59	77.5461	50.9177	0.5447	7.4654
60	73.4636	47.6338	0.5147	5.6778
61	75.5404	46.1264	0.5627	6.6424
62	78.9442	59.2622	0.6070	4.4691
63	74.8144	47.0445	0.3700	7.1989
64	73.3826	50.4805	0.5730	4.8680
65	74.9048	60.5562	0.5259	6.6164
66	79.2921	59.8008	0.5076	7.7849
67	78.7347	48.9915	0.5105	8.7956
68	74.0625	47.1517	0.5662	7.8148
69	76.8835	57.0272	0.5614	7.6651
70	73.4470	48.6825	0.5257	6.9015
71	76.2243	47.5016	0.5692	5.5757
72	76.5222	61.0987	0.5642	8.1248
73	71.7864	52.1124	0.5715	4.4219
74	75.4962	57.5217	0.5051	8.0193
75	77.2092	52.8628	0.5475	5.3434
76	78.5870	63.2747	0.5086	6.8986
77	78.4894	51.3108	0.5422	5.5477
78	79.3732	47.0634	0.5992	4.4131
79	79.8891	58.3924	0.5168	6.4457
80	72.7254	58.3152	0.5358	6.4351
81	74.9276	49.9738	0.5878	5.3186
82	72.2717	66.5911	0.5696	4.8955
83	78.6829	49.3712	0.5715	6.5485
84	73.6153	53.7361	0.5321	4.7235
85	78.0313	59.0975	0.5282	8.2745
86	75.2296	57.7403	0.5473	9.4777
87	74.5866	46.2136	0.5186	7.6946
88	77.6571	45.3879	0.5339	8.7804

seq	BC	OM	NH4
1	2.7490	11.9229	4.4924
2	2.2682	9.7961	4.8972
3	2.2538	10.4417	5.5764
4	2.0194	8.6557	3.6140
5	2.0215	8.3520	4.4131
6	2.7189	11.6393	5.0642
7	2.9109	13.0508	6.0885
8	1.7706	7.7683	3.7035

9	1.6542	7.1011	3.6140
10	2.6870	11.3949	5.7363
11	2.8545	12.5053	6.7339
12	3.1210	13.3714	5.2800
13	2.7978	12.2474	6.9773
14	3.7424	15.9663	6.4640
15	2.3533	10.5048	5.5598
16	2.2018	9.9858	5.6937
17	2.1055	9.9515	5.4126
18	1.7876	7.5342	3.8006
19	1.9191	9.2604	4.9981
20	2.5144	10.5332	4.4596
21	2.2096	10.8623	5.8699
22	1.7796	8.0343	4.4166
23	2.7868	10.9371	4.8808
24	2.3222	10.5497	5.5646
25	2.0117	8.6470	3.8674
26	2.5468	10.7232	4.6469
27	3.1501	13.4311	6.4514
28	2.0174	8.5080	4.2773
29	2.3217	10.0243	4.6569
30	2.8849	11.9907	6.0887
31	2.4247	10.0998	4.8632
32	1.9055	8.1840	4.1193
33	2.6426	11.0240	4.7520
34	1.9796	9.1570	4.7606
35	2.0577	8.8991	4.2188
36	2.1181	9.6541	5.2402
37	2.9915	12.5937	6.2652
38	1.9388	8.4719	4.1832
39	2.6135	10.3587	5.1694
40	2.1576	8.5598	4.4100
41	1.7770	8.3106	4.4835
42	1.7623	7.7846	4.1115
43	2.0511	9.3999	4.9205
44	2.2265	10.5828	5.9927
45	2.1099	9.2658	4.9257
46	2.2049	9.5213	4.5994
47	2.0586	8.6978	4.2815
48	2.5574	10.4348	4.6250
49	3.4461	15.2982	7.2175

50	2.1300	10.1271	5.7987
51	3.2856	14.2562	5.3038
52	2.7153	12.7803	7.7656
53	2.2365	10.8196	6.1862
54	2.4246	10.5043	4.5115
55	2.3404	10.1901	5.8803
56	2.2585	10.0033	5.9600
57	3.1081	13.7747	7.2393
58	2.8717	12.6856	6.1892
59	2.9516	12.3433	5.6172
60	2.0810	8.6839	4.5771
61	2.7149	10.9101	5.3926
62	1.8308	7.9956	3.8274
63	3.1103	13.4444	5.1131
64	1.6274	7.3846	3.9621
65	2.2958	9.8216	5.1843
66	3.0566	12.9959	5.6587
67	3.4105	14.4701	6.4636
68	2.2210	10.4579	5.8474
69	2.7984	11.7196	5.9790
70	2.3056	10.1979	5.2976
71	2.4365	9.5692	4.7233
72	2.1468	10.5709	5.8272
73	1.6400	7.1703	3.7473
74	2.2172	10.6946	5.7708
75	2.2325	9.7621	4.2807
76	2.8691	12.1885	5.0750
77	2.1010	8.9850	4.3261
78	1.7917	7.7836	3.7909
79	2.4493	10.4283	4.8897
80	2.1543	9.5433	4.9920
81	1.6996	7.8486	4.1826
82	1.8313	7.9146	3.9636
83	2.5980	10.3717	5.3177
84	1.8320	7.7173	3.8571
85	3.2187	13.3493	6.4831
86	2.7938	12.4852	7.4105
87	2.2879	10.6254	5.6621
88	2.9346	12.6745	6.6216
