# Supplementary material for: Spatial heterogeneity in the temperature–hand, foot, and mouth disease association among children: A multicounty time-series study in western China
Source: PLoS Negl Trop Dis. 2026 Jan 2;20(1):e0013801. doi: 10.1371/journal.pntd.0013801 (PMC12758769; doi:10.1371/journal.pntd.0013801)
Supplement: S1 Table — (DOCX) [file pntd.0013801.s003.docx]

**S1 Table**. List of 88 counties in Guizhou province included in the study.

| **No.** | **City name** | **County name** | **No.** | **City name** | **County name** |
| --- | --- | --- | --- | --- | --- |
| 1 | Guiyan | Nanming | 45 | Tongren | Jiangkou |
| 2 | Guiyan | Yunyan | 46 | Tongren | Yuping |
| 3 | Guiyan | Huaxi | 47 | Tongren | Shiqian |
| 4 | Guiyan | Wudang | 48 | Tongren | Sinan |
| 5 | Guiyan | Baiyun | 49 | Tongren | Yinjiang |
| 6 | Guiyan | Guanshanhu | 50 | Tongren | Dejiang |
| 7 | Guiyan | Kaiyang | 51 | Tongren | Yanhe |
| 8 | Guiyan | Xifeng | 52 | Tongren | Songtao |
| 9 | Guiyan | Xiuwen | 53 | Qianxinan | Xingyi |
| 10 | Guiyan | Qingzhen | 54 | Qianxinan | Xingren |
| 11 | Liupanshui | Zhongshan | 55 | Qianxinan | Puan |
| 12 | Liupanshui | Liuzhi | 56 | Qianxinan | Qinglong |
| 13 | Liupanshui | Shuicheng | 57 | Qianxinan | Zhenfeng |
| 14 | Liupanshui | Panzhou | 58 | Qianxinan | Wangmo |
| 15 | Zunyi | Honghuagang | 59 | Qianxinan | Ceheng |
| 16 | Zunyi | Huichuan | 60 | Qianxinan | Anlong |
| 17 | Zunyi | Bozhou | 61 | Qiandongnan | Kaili |
| 18 | Zunyi | Tongzi | 62 | Qiandongnan | Huangping |
| 19 | Zunyi | Suiyang | 63 | Qiandongnan | Shibing |
| 20 | Zunyi | Zhengan | 64 | Qiandongnan | Sansui |
| 21 | Zunyi | Daozhen | 65 | Qiandongnan | Zhenyuan |
| 22 | Zunyi | Wuchuan | 66 | Qiandongnan | Cengong |
| 23 | Zunyi | Fenggang | 67 | Qiandongnan | Tianzhu |
| 24 | Zunyi | Meitan | 68 | Qiandongnan | Jinping |
| 25 | Zunyi | Yuqing | 69 | Qiandongnan | Jianhe |
| 26 | Zunyi | Xishui | 70 | Qiandongnan | Taijiang |
| 27 | Zunyi | Chishui | 71 | Qiandongnan | Liping |
| 28 | Zunyi | Renhuai | 72 | Qiandongnan | Rongjiang |
| 29 | Anshun | Xixiu | 73 | Qiandongnan | Congjiang |
| 30 | Anshun | Pingba | 74 | Qiandongnan | Leishan |
| 31 | Anshun | Puding | 75 | Qiandongnan | Majiang |
| 32 | Anshun | Zhenning | 76 | Qiandongnan | Danzhai |
| 33 | Anshun | Guanling | 77 | Qiannan | Duyun |
| 34 | Bijie | Ziyun | 78 | Qiannan | Fuquan |
| 35 | Bijie | Qixingguan | 79 | Qiannan | Libo |
| 36 | Bijie | Dafang | 80 | Qiannan | Guiding |
| 37 | Bijie | Qianxi | 81 | Qiannan | Wengan |
| 38 | Bijie | Jinsha | 82 | Qiannan | Dushan |
| 39 | Bijie | Zhijin | 83 | Qiannan | Pingtang |
| 40 | Bijie | Nayong | 84 | Qiannan | Luodian |
| 41 | Bijie | Weining | 85 | Qiannan | Changshun |
| 42 | Bijie | Hezhang | 86 | Qiannan | Longli |
| 43 | Tongren | BiJiang | 87 | Qiannan | Huishui |
| 44 | Tongren | Wanshan | 88 | Qiannan | Sandu |
