# Supplementary material for: Spatial heterogeneity in the temperature–hand, foot, and mouth disease association among children: A multicounty time-series study in western China
Source: PLoS Negl Trop Dis. 2026 Jan 2;20(1):e0013801. doi: 10.1371/journal.pntd.0013801 (PMC12758769; doi:10.1371/journal.pntd.0013801)
Supplement: S2 Table — (DOCX) [file pntd.0013801.s004.docx]

**S2 Table**. Relative risk analysis of heterogeneous environmental factors in the temperature-HFMD associations.

| **Description** | **Percentiles of temperature** | | | | | |
| --- | --- | --- | --- | --- | --- | --- |
|  | **5th (-1.4 ℃)** | | **25th (5.0 ℃)** | **50th (13.0 ℃)** | **75th (20.9 ℃)** | **95th (27.3℃)** |
| Heat waves (the 10th) | 0.92 (0.73, 1.00) | | 0.93 (0.82, 1.03) | 1.00 (0.93, 1.16) | 1.21 (1.01, 1.41) | 1.23 (1.03, 1.63) |
| Heat waves (the 90th) | 0.78 (0.61, 0.96) | | 0.86 (0.76, 0.97) | 1.00 (1.00, 1.00) | 1.46 (1.25, 1.70) | 3.17 (2.39, 4.19) |
| Cold spells (the 10th) | 0.75 (0.59, 0.95) | | 0.85 (0.76, 0.96) | 1.00 (1.00, 1.00) | 1.41 (1.21, 1.63) | 3.13 (2.40, 4.07) |
| Cold spells (the 90th) | 0.92 (0.77, 0.99) | | 0.94 (0.82, 1.02) | 1.00 (0.94, 1.16) | 1.23 (1.03, 1.46) | 1.27 (1.07, 1.63) |
| PM_2.5_ (the 10th) | 0.89 (0.72, 0.99) | | 0.91 (0.82, 1.01) | 1.00 (1.00, 1.09) | 1.21 (1.06, 1.38) | 1.39 (1.10, 1.77) |
| PM_2.5_ (the 90th) | 0.81 (0.65, 0.96) | | 0.86 (0.77, 0.97) | 1.00 (1.00, 1.02) | 1.48 (1.29, 1.70) | 3.05 (2.35, 3.95) |
| PM_10_ (the 10th) | 0.89 (0.71, 1.00) | | 0.91 (0.81, 1.01) | 1.00 (1.00, 1.10) | 1.22 (1.05, 1.40) | 1.44 (1.11, 1.88) |
| PM_10_ (the 90th) | 0.81 (0.65, 0.96) | | 0.86 (0.77, 0.98) | 1.00 (1.00, 1.03) | 1.46 (1.26, 1.68) | 2.86 (2.17, 3.78) |
| $\text{NO}_{3}^{-}$ (the 10th) | 0.94 (0.73, 1.01) | | 0.95 (0.84, 1.05) | 1.00 (0.96, 1.14) | 1.15 (1.00, 1.33) | 1.42 (1.06, 1.90) |
| $\text{NO}_{3}^{-}$ (the 90th) | 0.78 (0.63, 0.93) | | 0.84 (0.75, 0.95) | 1.00 (1.00, 1.00) | 1.51 (1.32, 1.72) | 2.73 (2.09, 3.57) |
| $\text{NH}_{4}^{+}$ (the 10th) | 0.94 (0.73, 1.01) | | 0.95 (0.85, 1.05) | 1.00 (0.96, 1.14) | 1.14 (1.00, 1.33) | 1.43 (1.07, 1.92) |
| $\text{NH}_{4}^{+}$ (the 90th) | 0.78 (0.63, 0.93) | | 0.84 (0.75, 0.94) | 1.00 (1.00, 1.00) | 1.51 (1.32, 1.72) | 2.71 (2.07, 3.54) |
| O_3_ (the 10th) | 0.80 (0.61, 0.97) | | 0.86 (0.76, 0.99) | 1.00 (1.00, 1.04) | 1.43 (1.22, 1.68) | 2.83 (2.10, 3.82) |
| O_3_ (the 90th) | 0.90 (0.72, 0.99) | | 0.91 (0.82, 1.01) | 1.00 (1.00, 1.11) | 1.24 (1.08, 1.44) | 1.47 (1.12, 1.95) |
| NDVI (the 10th) | 0.83 (0.72, 0.93) | | 0.87 (0.81, 0.95) | 1.00 (1.00, 1.00) | 1.39 (1.27, 1.52) | 2.16 (1.79, 2.59) |
| NDVI (the 90th) | 0.94 (0.76, 1.01) | 0.96 (0.87, 1.05) | | 1.00 (0.96, 1.12) | 1.15 (1.00, 1.32) | 1.59 (1.21, 2.08) |
