# Supplementary material for: Spatial heterogeneity in the temperature–hand, foot, and mouth disease association among children: A multicounty time-series study in western China
Source: PLoS Negl Trop Dis. 2026 Jan 2;20(1):e0013801. doi: 10.1371/journal.pntd.0013801 (PMC12758769; doi:10.1371/journal.pntd.0013801)
Supplement: S3 Table — (DOCX) [file pntd.0013801.s005.docx]

**S3 Table**. A list of 88 counties in Guizhou Province divided into three clustering types.

| **Cluster** | **City** | **County** | **Cluster** | **City** | **County** | **Cluster** | **City** | **County** |
| --- | --- | --- | --- | --- | --- | --- | --- | --- |
| 1 | Guiyan | Baiyun | 2 | Qianxinan | Wangmo | 3 | Qiandongnan | Kaili |
| 1 | Guiyan | Guanshanhu | 2 | Bijie | Weining | 3 | Guiyan | Kaiyang |
| 1 | Zunyi | Honghuagang | 2 | Qianxinan | Xingren | 3 | Qiandongnan | Leishan |
| 1 | Guiyan | Huaxi | 2 | Qianxinan | Xingyi | 3 | Qiannan | Libo |
| 1 | Qiannan | Longli | 2 | Guiyan | Xiuwen | 3 | Qiandongnan | Liping |
| 1 | Guiyan | Nanming | 2 | Anshun | Xixiu | 3 | Qiandongnan | Majiang |
| 1 | Guiyan | Qingzhen | 2 | Qianxinan | Zhenfeng | 3 | Zunyi | Meitan |
| 1 | Zunyi | Renhuai | 2 | Anshun | Zhenning | 3 | Qiandongnan | Rongjiang |
| 1 | Guiyan | Wudang | 2 | Bijie | Zhijin | 3 | Qiannan | Sandu |
| 1 | Guiyan | Xifeng | 2 | Liupanshui | Zhongshan | 3 | Qiandongnan | Sansui |
| 1 | Guiyan | Yunyan | 2 | Bijie | Ziyun | 3 | Qiandongnan | Shibing |
| 2 | Qianxinan | Anlong | 3 | Tongren | Bijiang | 3 | Tongren | Shiqian |
| 2 | Qianxinan | Ceheng | 3 | Zunyi | Bozhou | 3 | Tongren | Sinan |
| 2 | Qiannan | Changshun | 3 | Qiandongnan | Cengong | 3 | Tongren | Songtao |
| 2 | Bijie | Dafang | 3 | Zunyi | Chishui | 3 | Zunyi | Suiyang |
| 2 | Anshun | Guanling | 3 | Qiandongnan | Congjiang | 3 | Qiandongnan | Taijiang |
| 2 | Bijie | Hezhang | 3 | Qiandongnan | Danzhai | 3 | Qiandongnan | Tianzhu |
| 2 | Qiannan | Huishui | 3 | Zunyi | Daozhen | 3 | Zunyi | Tongzi |
| 2 | Liupanshui | Liuzhi | 3 | Tongren | Dejiang | 3 | Tongren | Wanshan |
| 2 | Qiannan | Luodian | 3 | Qiannan | Dushan | 3 | Qiannan | Wengan |
| 2 | Bijie | Nayong | 3 | Qiannan | Duyun | 3 | Zunyi | Wuchuan |
| 2 | Liupanshui | Panzhou | 3 | Zunyi | Fenggang | 3 | Zunyi | Xishui |
| 2 | Anshun | Pingba | 3 | Qiannan | Fuquan | 3 | Tongren | Yanhe |
| 2 | Qiannan | Pingtang | 3 | Qiannan | Guiding | 3 | Tongren | Yinjiang |
| 2 | Qianxinan | Puan | 3 | Qiandongnan | Huangping | 3 | Tongren | Yuping |
| 2 | Anshun | Puding | 3 | Zunyi | Huichuan | 3 | Zunyi | Yuqing |
| 2 | Bijie | Qianxi | 3 | Tongren | Jiangkou | 3 | Zunyi | Zhengan |
| 2 | Qianxinan | Qinglong | 3 | Qiandongnan | Jianhe | 3 | Qiandongnan | Zhenyuan |
| 2 | Bijie | Qixingguan | 3 | Qiandongnan | Jinping |  |  |  |
| 2 | Liupanshui | Shuicheng | 3 | Bijie | Jinsha |  |  |  |
