# Supplementary material for: Spatial heterogeneity in the temperature–hand, foot, and mouth disease association among children: A multicounty time-series study in western China
Source: PLoS Negl Trop Dis. 2026 Jan 2;20(1):e0013801. doi: 10.1371/journal.pntd.0013801 (PMC12758769; doi:10.1371/journal.pntd.0013801)
Supplement: S4 Table — (DOCX) [file pntd.0013801.s006.docx]

**S4 Table.** Statistical description of the three clusters and ANOVA.

| **Factors** | **Central Region** | **Southwestern region** | **Northeastern region** | ***P*** |
| --- | --- | --- | --- | --- |
| n | 11 | 30 | 47 | – |
| Cluster (%) | 100 | 23.86 | 17.05 | – |
| Population density (10000 population,  Mean (SD)) | 1816.81 (3146.94) | 232.14 (117.97) | 157.06 (68.50) | 0.001 |
| GDP per capita (CNY, Mean (SD)) | 69935.18 (17830.71) | 30056.30 (10497.49) | 29281.61 (9732.79) | <0.001 |
| Heat waves (Mean (SD)) | 41.27 (2.37) | 25.03 (6.01) | 43.96 (6.42) | <0.001 |
| Cold spells (Mean (SD)) | 49.55 (2.02) | 54.60 (4.62) | 45.30 (4.33) | <0.001 |
| PM_2.5_ (μg/m^3^, Mean (SD)) | 35.18 (2.98) | 32.69 (1.73) | 35.59 (2.98) | 0.028 |
| O_3_ (μg/m^3^, Mean (SD)) | 76.95 (1.85) | 77.93 (1.85) | 74.03 (1.47) | <0.001 |
| NDVI (μg/m^3^, Mean (SD)) | 0.48 (0.05) | 0.55 (0.03) | 0.55 (0.03) | <0.001 |
