# Supplementary material for: Spatial heterogeneity in the temperature–hand, foot, and mouth disease association among children: A multicounty time-series study in western China
Source: PLoS Negl Trop Dis. 2026 Jan 2;20(1):e0013801. doi: 10.1371/journal.pntd.0013801 (PMC12758769; doi:10.1371/journal.pntd.0013801)
Supplement: S5 Table — (DOCX) [file pntd.0013801.s007.docx]

**S5 Table**. Sensitivity analysis comparing different definitions of heat wave and cold spell in meta-analysis.

| **Df of the exposure** | **Df of the lag-response** | **Q-AIC（Other covariates fixed *df* =3）** | **Q-AIC（Other covariates fixed *df* =4）** | **Q-AIC（Other covariates fixed *df* =5）** |
| --- | --- | --- | --- | --- |
| 3 | 3 | 6587.50 | 6589.00 | 6590.20 |
| 3 | 4 | 6589.50 | 6591.10 | 6592.40 |
| 3 | 5 | 6592.70 | 6594.20 | 6595.50 |
| 4 | 3 | 6589.90 | 6591.40 | 6592.70 |
| 4 | 4 | 6593.30 | 6594.80 | 6596.20 |
| 4 | 5 | 6597.30 | 6598.70 | 6599.90 |
| 5 | 3 | 6592.20 | 6594.10 | 6595.20 |
| 5 | 4 | 6596.70 | 6598.60 | 6599.70 |
| 5 | 5 | 6602.20 | 6604.00 | 6605.00 |
