# Supplementary material for: Spatial heterogeneity in the temperature–hand, foot, and mouth disease association among children: A multicounty time-series study in western China
Source: PLoS Negl Trop Dis. 2026 Jan 2;20(1):e0013801. doi: 10.1371/journal.pntd.0013801 (PMC12758769; doi:10.1371/journal.pntd.0013801)
Supplement: S6 Table — (DOCX) [file pntd.0013801.s008.docx]

**S6 Table**. Sensitivity analysis of differently defined heat and cold spell in meta-analysis.

| **Meta-predictors** | **Wald test** | **Model fits** | | | **Cochran Q test** | | | | **Heterogeneity (%)** | | |  |
| --- | --- | --- | --- | --- | --- | --- | --- | --- | --- | --- | --- | --- |
|  | **（*p*）** | **LogLik** | **AIC** | **BIC** | | ***Q*** | ***df*** | ***p*** | | ***I*^2^** | **Δ*I*^2b^** |  |
| **Intercept-only model** | | | | | | | | | | | |  |
| Intercept only | － | -410.60 | 839.20 | 871,28 | | 522.86 | 261 | <0.001 | | 50.08 | － |  |
| **Single meta-predictor models** | | | | | | | | | | | |  |
| **ETEs** |  |  |  |  | |  |  |  | |  |  |  |
| Heat waves (95% maximum temperature ≥ 2 days） | 0.003 | -414.80 | 853.61 | 896.25 | | 496.99 | 258 | <0.001 | | 48.09 | 1.99 |  |
| Cold spells (5% minimum temperature ≥ 2 days) | 0.001 | -411.78 | 847.56 | 890.20 | | 485.90 | 258 | <0.001 | | 46.9 | 3.18 |  |
| Heat waves (95% maximum temperature ≥ 3 days） | 0.001 | -413.71 | 851.43 | 894.06 | | 490.15 | 258 | <0.001 | | 47.36 | 2.72 |  |
| Cold spells (5% minimum temperature ≥ 3 days) | <0.001 | -409.75 | 843.50 | 886.14 | | 479.91 | 258 | <0.001 | | 46.24 | 3.84 |  |
