# Supplementary material for: Spatial heterogeneity in the temperature–hand, foot, and mouth disease association among children: A multicounty time-series study in western China
Source: PLoS Negl Trop Dis. 2026 Jan 2;20(1):e0013801. doi: 10.1371/journal.pntd.0013801 (PMC12758769; doi:10.1371/journal.pntd.0013801)
Supplement: S7 Table — (DOCX) [file pntd.0013801.s009.docx]

**S7 Table.** Multicollinearity diagnostics based on Variance Inflation Factor (VIF).

| **Variable** | **VIF** |
| --- | --- |
| Population density (10000 population) | 1.657 |
| GDP per capita (CNY) | 1.492 |
| Nighttime light (nW/cm^2^/sr） | 1.012 |
| NDVI (μg/m^3^) | 2.138 |
