# Supplementary material for: Spatial heterogeneity in the temperature–hand, foot, and mouth disease association among children: A multicounty time-series study in western China
Source: PLoS Negl Trop Dis. 2026 Jan 2;20(1):e0013801. doi: 10.1371/journal.pntd.0013801 (PMC12758769; doi:10.1371/journal.pntd.0013801)
Supplement: S1 Text — (DOCX) [file pntd.0013801.s016.docx]

**S1 Text.** Details of the clustering analysis.

In the initial heterogeneity analysis, we identified meaningful modifiers of the temperature-HFMD relationship, including cold spells, heat waves, PM_2.5_, PM_10_, NDVI, O_3_, and two PM_2.5_ components ($\text{NO}_{3}^{-}$and$\text{NH}_{4}^{+}$), and conventional socioeconomic factors (population density, GDP per capita). Prior to clustering, we assessed multicollinearity among these variables using a correlation matrix (S3 Fig). Because PM_10_ was highly correlated with PM_2.5_ ($r =$ 0.98), it was excluded based on the correlation threshold ($\left| r \right|\geq$0.8). We also excluded the two PM_2.5_ components because PM_2.5_ itself was retained as the representative pollutant. The final variables included in the clustering analysis were cold spells, heat waves, PM_2.5_, NDVI, O_3_, population density, and GDP per capita. All variables were continuous and therefore suitable for standardization using R’s scale **()** function, which transforms variables to have a mean of 0 and a standard deviation of 1. This step ensured that variables with different units contributed equally to the clustering process, eliminating potential biases from variable magnitude differences.

Next, we employed a fully data-driven approach to determine the optimal number of clusters using model-based clustering implemented in the mclust package (R). This method fits a series of Gaussian mixture models, each assuming a different number of clusters ($k$) and one of multiple possible covariance structures (up to 14 parameterizations, depending on data dimensionality), which define the geometric shape and orientation of clusters in the feature space. Rather than pre-specifying , the algorithm evaluates all candidate models across a range of $k$ values and covariance types. The optimal model—specifying both the number of clusters and the covariance structure—was selected as the one maximizing the Bayesian Information Criterion (BIC). BIC provides a statistically principled trade-off between model fit and complexity, penalizing overparameterized models to guard against overfitting. Thus, the final clustering solution represents the most parsimonious and data-supported configuration, objectively identified through this criterion-based procedure.

This clustering framework—leveraging the 14 methods in *Mclust* and data-driven BIC selection—allowed us to objectively identify regional patterns, thereby providing a more nuanced explanation of how environmental impacts on HFMD vary across different geographic areas.
